# Muscle quality index combined with plasma metabolomics: enhancing risk stratification for post-stroke dementia

**DOI:** 10.3389/fnagi.2026.1837030

**Published:** 2026-09-29

**Authors:** Zhen Tan, Shuang Li, Tianjiao Li, Lei Liu, Xinrui Xue, Xiaoping Li, Hongqiang Ren

**Affiliations:** 1 Department of Cardiology, Suining Central Hospital, Suining, Sichuan, China; 2 Department of Ultrasound Medicine, Suining Central Hospital, Suining, Sichuan, China; 3 Department of Cardiology, Sichuan Provincial People's Hospital, University of Electronic Science and Technology of China, Chengdu, Sichuan, China

**Keywords:** dementia, muscle quality index, plasma metabolomics, post-stroke, risk stratification

## Abstract

**Objective:**

Post-stroke dementia is a severe long-term stroke complication with heavy medical burdens, and early risk identification is critical. This study explored the association of muscle quality index (MQI) and plasma metabolomics with dementia among stroke survivors.

**Methods:**

This study included 7,047 UK Biobank participants with a history of stroke who were free of dementia at the baseline assessment. MQI was defined as dominant handgrip strength (HGS) divided by corresponding arm fat-free mass. Plasma metabolomics was analyzed via high-throughput nuclear magnetic resonance (NMR). Metabolomics risk scores (MRS-ACD, MRS-AD, and MRS-VaD) were constructed using metabolite features screening with LASSO regression and XGBoost algorithms. Statistical analyses included Cox proportional hazards models, time-dependent receiver operating characteristic (TD-ROC), restricted cubic spline (RCS) regression, interaction analyses, Mendelian randomization (MR), and several sensitivity analyses.

**Results:**

Higher MQI was associated with lower risks of all-cause dementia (ACD), Alzheimer’s disease (AD) and vascular dementia (VaD) (all *p* for trend <0.05). Each 1-SD increment in MRS was associated with a higher risk of ACD (HR = 1.63, 95% CI: 1.36–1.96), AD (HR = 2.40, 95% CI: 1.74–3.31), and VaD (HR = 1.92, 95% CI: 1.46–2.54). Individuals with high MRS and lowest MQI had the highest ACD (HR = 3.29, 95% CI: 1.91–5.68), AD (HR = 5.83, 95% CI: 1.75–19.37), and VaD (HR = 4.57, 95% CI: 2.28–9.14) risk. MQI was linearly negatively associated with ACD (*p* for non-linear = 0.240) and VaD (*p* for non-linear = 0.665), and non-linearly associated with AD (*p* for non-linear = 0.013). Two-sample MR using genetically proxied grip strength and lean mass provided suggestive genetic evidence for muscle-dementia relationships: higher genetically predicted lean mass reduced AD risk (OR = 0.92, 95% CI 0.86–0.99, *p* = 0.027), while grip strength showed an independent protective effect on VaD after accounting for lean mass (OR = 0.16, 95% CI 0.04–0.64, *p* = 0.009). Extensive sensitivity analyses confirmed the robustness of the main findings.

**Conclusion:**

Joint assessment of MQI and plasma metabolomics provides suggestive evidence for stratifying post-stroke dementia risk and may assist early risk screening in stroke survivors.

## 1 Introduction

Stroke is a leading global cause of neurological disability and mortality, imposing profound strain on healthcare systems and families (Zhan et al., 2025). In addition to acute neurological dysfunction, post-stroke dementia has become one of the most severe long-term consequences among stroke survivors (Shin et al., 2024). It is characterized by progressive deterioration in cognitive functions such as memory, attention, and executive control, significantly impairing patients’ daily functioning and increasing demands on caregivers (Chi et al., 2020; Elahi et al., 2023; Vermeer et al., 2003). Given the irreversible progression of advanced cognitive decline, identifying high-risk populations at an early stage and implementing timely preventive interventions are critical to optimizing clinical prognoses (Yamasaki and Ikeda, 2024). However, conventional predictive tools, such as clinical scoring systems or isolated biomarkers, frequently fail to achieve satisfactory sensitivity and specificity, which underscores the need for more reliable and comprehensive approaches to risk prediction (Nakaoku et al., 2018).

Muscle quality index (MQI) reflects skeletal muscle reserve capacity and has recently been recognized as a potential prognostic indicator in neurological disorders (Weng et al., 2023; Wang et al., 2024). Accumulating evidence demonstrates that low MQI, commonly associated with sarcopenia, correlates with adverse post-stroke outcomes such as increased mortality risk, impaired functional independence and progressive cognitive deterioration (Tan et al., 2025; Zhang A. et al., 2025; Zhang K. et al., 2025). Chronic inflammation, oxidative stress, insulin resistance, and suppressed secretion of neurotrophic factors triggered by muscle atrophy may contribute to these correlations, exacerbating ischemic brain damage and hindering the restoration of neurological function (Guerrini et al., 2025; Meng et al., 2024; Li et al., 2020). Despite its clinical significance, MQI has inherent limitations when used as a standalone predictive biomarker. It can be influenced by age, comorbidities, and nutritional status, and it does not fully reflect the complex molecular changes involved in the transition from stroke to dementia.

Plasma metabolomics, an omics-based approach that captures overall metabolic fluctuations in biofluids, provides valuable insights into disease mechanisms and holds promise for identifying novel biomarkers in neurological disorders (Flores-Torres et al., 2025; Peña-Bautista et al., 2019). Dysfunctional changes in essential amino acid, lipid and energy metabolic pathways are closely implicated in the development of cerebral ischemia and the progression of secondary cognitive impairment (Baranovicova et al., 2023; Wang X. et al., 2025; Wang Y., et al., 2025; Lei et al., 2025). Some reports suggest elevated levels of pro-inflammatory metabolites like tryptophan derivatives and decreased concentrations of neuroprotective compounds such as docosahexaenoic acid have been associated with greater risk of post-stroke dementia (Connell et al., 2022; Hong et al., 2014; Guo et al., 2023). Nevertheless, single-metabolite biomarkers often lack robust predictive performance due to the heterogeneity of post-stroke dementia, whereas the integration of multiple metabolic features may enhance the accuracy of risk prediction (Lumsden et al., 2025).

Recent advances suggest that combining phenotypic measures with molecular profiles could improve prognostic precision across various diseases (Wade et al., 2022; Johansson et al., 2023). By integrating MQI with plasma metabolomics, it may be possible to simultaneously capture systemic physiological status and underlying biochemical disturbances related to post-stroke dementia development (Wang X. et al., 2025; Wang Y., et al., 2025; Yan et al., 2025). Such a multimodal approach could overcome the shortcomings of a single biomarker and lead to the identification of a more specific and sensitive prognostic signature (Ding et al., 2025). However, few studies have investigated the combined effects of MQI and plasma metabolomics on predicting post-stroke dementia, leaving the synergistic potential of these indicators largely unexplored.

Accordingly, this study aimed to investigate the association of combined MQI and plasma metabolomic profiling with post-stroke dementia. Furthermore, this study may facilitate the development of early risk stratification tools to ameliorate long-term prognoses in stroke survivors.

## 2 Methods

### 2.1 Study design and cohort selection

The UK Biobank (UKB) is a prospective population-based cohort with extensive phenotyping, biological sampling, and long-term follow-up. Participants (*n* = 502,128) aged 40–69 years were recruited from 22 centers across the United Kingdom between 2006 and 2010. Baseline assessments included questionnaires, physical examinations, and biological sample collection. Follow-up data were obtained through linkage to national hospital episode statistics (HES), primary care records, and death registries, with a median follow-up duration exceeding 10 years. The study protocol was approved by the North West Multi-centre Research Ethics Committee, and all participants provided written informed consent. This study was conducted under UKB research license application number 106027. Inclusion criteria: (i) a confirmed diagnosis of stroke at baseline; (ii) complete measurements of dominant handgrip strength (HGS, kg) and corresponding arm fat-free mass (kg). Exclusion criteria: (i) dementia confirmed by medical records; (ii) extreme MQI values suggestive of measurement error; (iii) plasma samples with insufficient volume or hemolysis. Finally, 7,047 participants were included in the primary analysis, and Figure 1 shows the participant screening flowchart.

**Figure 1:**
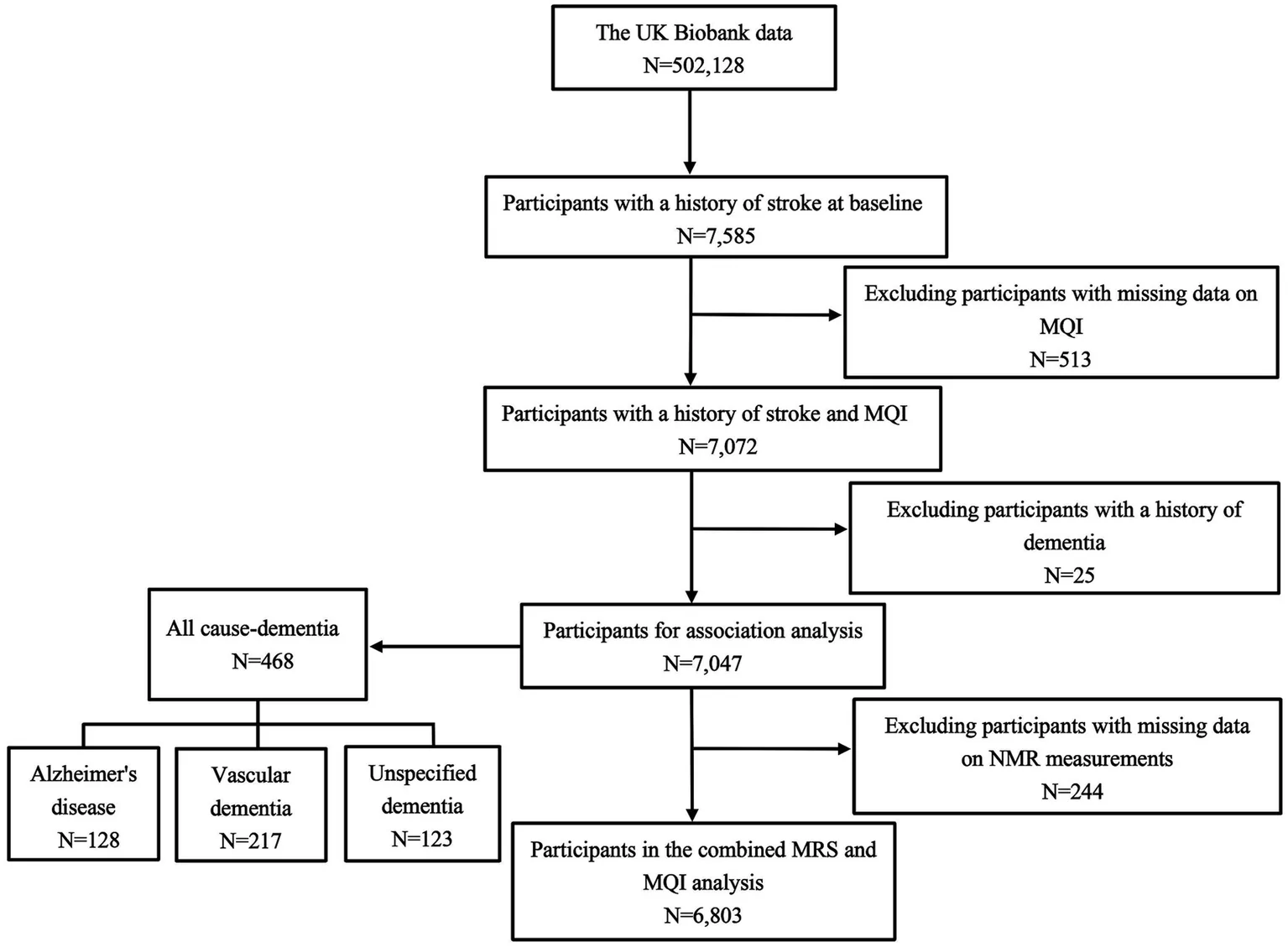
Flow chart of participant screening.

### 2.2 Post-stroke dementia outcome definition

At baseline, confirmed stroke was ascertained from HES, death registry, primary-care records (Read codes), and self-reported medical history. Stroke events were identified using International Classification of Diseases, Tenth Revision (ICD-10) codes I60–I64. For these baseline stroke survivors, incident dementia during follow-up was ascertained via the codes: all-cause dementia (ACD, F00, F01, F02, F03, and G30), Alzheimer’s disease (AD, F00, G30), and vascular dementia (VaD, F01), assigned to mutually exclusive categories according to the first recorded dementia diagnosis. Once assigned, the subtype category was not altered by subsequent dementia diagnoses. The index date for dementia was the earliest recorded date of diagnosis. Follow-up time was calculated from the baseline to the date of dementia, death, or December 31, 2023 (end of follow-up), whichever came first.

### 2.3 MQI calculation

Measurement of dominant HGS and corresponding arm fat-free mass were conducted using the same protocols as those described in previous related studies (Lopes et al., 2022). MQI was defined as the ratio of dominant HGS to corresponding arm fat-free mass, reflecting muscle strength per unit mass, a validated metric of muscle quality (Weng et al., 2023). MQI was divided into quartiles based on gender-specific cutoff points (Q1–Q4), with the Q1 quartile serving as the reference group.

### 2.4 Plasma metabolomics measurements

Plasma samples were collected using ethylenediaminetetraacetic acid (EDTA) as an anticoagulant under non-fasting conditions, following the standard procedures of the UK Biobank (Qiang et al., 2024). Baseline plasma specimens were analyzed using a high-throughput metabolic profiling platform based on nuclear magnetic resonance (NMR) technology, developed by Nightingale Health Ltd., to quantify metabolite concentrations. Comprehensive descriptions of the experimental methods and quality control protocols are available in previously published studies (Yang et al., 2025). A total of 249 metabolic biomarkers were included in the analysis, comprising 168 individual metabolite concentrations and 81 derived ratios. These biomarkers encompass a broad range of biochemical classes, including cholesterol species, fatty acids, and low-molecular weight compounds such as amino acids, ketone bodies, and metabolites associated with glycolysis.

### 2.5 Covariates

Covariates were collected at baseline via standardized questionnaires or structured interviews and were grouped into four categories: demographics, lifestyle and psychosocial factors, comorbidities and medication use, and laboratory biomarkers. Demographic characteristics: age, gender (male or female), race (White or non-White), educational level (college and above or others), fasting time, and Townsend Deprivation Index (TDI, a measure of socioeconomic status, which integrates unemployment, household overcrowding, home ownership, and car ownership into a single composite score, with higher values indicating greater deprivation). Lifestyle and psychosocial factors: body mass index (BMI), MQI, dominant HGS, corresponding arm fat-free mass, smoking status (never, former, and current), alcohol consumption status (never, former, and current), physical exercise intensity [MET-min/week, categorized as low (<600), moderate (600–3,000), high (>3,000)], sleep duration (<7 h/day, 7–9 h/day, and >9 h/day) and dietary habits (healthy or unhealthy dietary pattern, based on a dietary index score of ≥4 or <4). Comorbidities and medication use: loneliness, hypertension (defined by either hospital diagnosis, use of antihypertensive medications, and/or blood pressure ≥140/90 mmHg), diabetes (defined as self-reported physician-diagnosed diabetes, current use of hypoglycemic medications, FBG ≥ 7 mmol/L, and/or HbA1c ≥ 6.5% at baseline), chronic kidney disease (CKD), antihypertensive drugs, cholesterol-lowering medication, aspirin, and insulin. Laboratory biomarkers: C-reactive protein (CRP), hemoglobin A1c (HbA1c), glucose (GLU), albumin (ALB), serum creatinine (Scr), uric acid (Ua), blood urea nitrogen (BUN), cystatin C (Cys-C), triglycerides (TG), total cholesterol (TC), high-density lipoprotein cholesterol (HDL-C), low-density lipoprotein cholesterol (LDL-C), and estimated glomerular filtration rate (eGFR).

### 2.6 Statistical analysis

All analyses were performed using R software (version 4.3.0; Institute for Statistics and Mathematics, Vienna, Austria). A two-sided *p* < 0.05 was considered statistically significant.

Continuous variables were presented as mean ± standard deviation (SD). For normally distributed variables, differences across groups were assessed using one-way analysis of variance (ANOVA). Non-normally distributed variables were presented as median and interquartile range [M (P25, P75)], and group comparisons were conducted using the Kruskal–Wallis *H*-test. Categorical variables were expressed as frequencies and percentages, with between-group proportions compared using the chi-square test. Missing covariate data were handled using Multiple Imputation by Chained Equations (MICE), generating 20 imputed datasets. Across all covariates, the maximum missing-data rate was 14.8%, with an average missing rate of 3.0% (Supplementary Table S1).

We employed three Cox proportional hazards models to assess the associations between MQI and post-stroke dementia and prespecified the adjustment strategy using a directed acyclic graph (DAG) to minimize residual confounding and over-adjustment bias (Supplementary Figure S1). Model 1 was unadjusted. Model 2 was adjusted for age, gender, race, education level, TDI, smoking, alcohol consumption, dietary habits, physical exercise intensity, and sleep duration. Model 3 further added loneliness, BMI, and stroke duration. We calculated variance inflation factors (VIF) for all covariates in Model 3 to assess potential multicollinearity between disease states; all VIF values were below 5 (Supplementary Table S2). The proportional hazards assumption was assessed using Schoenfeld residual tests; the global test indicated no violation for any outcome (global *p* > 0.05; Supplementary Table S3).

We fitted RCS with four knots (5th, 35th, 65th, and 95th percentiles) in Cox model to examine the association of MQI and dementia. Spline significance was evaluated by a Wald test, and non-linearity by a likelihood ratio test. The exposure-response threshold was identified by a two-piecewise linear regression model screening candidates across the 10th to 90th percentiles (final value maximizing log-likelihood). HRs and 95% CIs above and below this threshold were computed using the threshold as the reference, with covariate adjustment as in Model 3.

After excluding participants with missing NMR measurements, we stratified and randomly divided the remaining 6,803 individuals into a training cohort (70%, *n* = 4,762) and a validation cohort (30%, *n* = 2,041) with the random seed set to 42 at a 7:3 ratio. Variable selection was completed using only the training cohort. Statistical performance metrics including discrimination and calibration were then assessed on the validation cohort.

A two-step feature selection workflow was applied to screen disease-related metabolites linked to ACD, AD, and VaD. Firstly, metabolite features were selected in the training cohort (70%) using LASSO-Cox regression (penalized Cox proportional hazards model, glmnet package), with metabolites log(x + 1)-transformed and Z-score standardized, *λ* selected by 10-fold event-stratified cross-validation under the lambda.min rule, and non-zero-coefficient metabolites retained as selected features. Secondly, an extreme gradient boosting (XGBoost) algorithm trained on these candidates was used to rank variable importance via SHAP (SHapley Additive exPlanations) values, from which the top 10 metabolites were selected.

Time-dependent receiver operating characteristic (TD-ROC) analysis was performed to evaluate the discriminative ability of the selected metabolites for ACD, AD, and VaD at 10 and 15 years of follow-up in the validation cohort. Discrimination was visualized using ROC curves and quantified by the area under the curve (AUC) at each time point. The 95% confidence intervals (CIs) for the time-dependent AUCs were estimated using nonparametric bootstrap resampling (1,000 replicates sampled with replacement from the validation cohort, with the selected metabolites applied and the AUC recalculated in each replicate) via the percentile method (2.5th–97.5th percentiles of the bootstrap distribution).

The outcome-specific metabolomics risk scores (MRS-ACD, MRS-AD, and MRS-VaD; hereafter, the score corresponding to the outcome under analysis is referred to as MRS) were constructed as weighted sums of the selected metabolites, with coefficients derived from the LASSO-Cox regression. Each MRS was calculated as a weighted sum of the top 10 metabolites selected for each outcome, with the weights being the LASSO-derived coefficients of these 10 metabolites:

$$\mathrm{MRS}=\sum_{i=1}^{k}\beta_{i}\times Z(\text{metabolit}e_{i})$$

Where *βi* = the non-zero coefficient of the ith metabolite among the top 10 ranked by XGBoost/SHAP, obtained from the LASSO regression; *Z(metabolite_i_)* = the standardized value of the ith metabolite in the training cohort; *k* = the number of metabolites included in the final outcome-specific MRS; here, *k* = 10, corresponding to the top 10 metabolites ranked by XGBoost/SHAP.

Then, in the validation cohort, the associations between MRS and ACD, AD, and VaD were evaluated using Cox proportional hazards regression models. The MRS was analyzed both as a continuous variable (per 1 SD increment) and as categorical tertiles (T1, T2, T3), with T1 serving as the reference group. The models were adjusted for age, gender, race, TDI, education level, smoking status, alcohol consumption status, dietary habits, sleep duration, physical exercise intensity, BMI, loneliness, and stroke duration. Fasting time was additionally included as a covariate in all subsequent MRS-related analyses.

To assess the incremental discrimination of MQI and MRS beyond conventional risk factors, we built four nested Cox models: (i) base clinical covariates (age, gender, race, TDI, education level, smoking status, alcohol consumption status, dietary habits, sleep duration, physical exercise intensity, BMI, loneliness, stroke duration, diabetes, and hypertension); (ii) base + MQI; (iii) base + MRS; and (iv) base + both. Discrimination was evaluated using Harrell’s C-index and time-dependent AUC at 10 and 15 years, with inverse probability of censoring weights (IPCW) estimated by the Kaplan–Meier method. AUCs were compared between extended and base models using DeLong’s test. Reclassification at 15 years was assessed using continuous net reclassification improvement (NRI) and integrated discrimination improvement (IDI), with censoring weights applied. Calibration was examined by comparing predicted risks with observed cumulative incidences across risk quintiles, and prediction error was quantified by Brier scores at 10 and 15 years (with IPCW). Decision curve analysis evaluated clinical utility by comparing censoring-weighted net benefit against treat-all and treat-none strategies. For all effect measures, 95% confidence intervals were derived from 1,000 bootstrap resamples using the percentile method, thereby accounting for potential optimism in the apparent estimates. Analyses were performed in R (packages survival and timeROC).

The joint association of MQI and MRS with the risk of post-stroke dementia was evaluated using Cox proportional hazards model (Model 3). The hazard ratios (HRs) and 95% confidence intervals (95% CIs) for the MRS-MQI combination were calculated, with low-risk MRS + MQI Q4 as the reference. To further explore the association of MRS and MQI with post-stroke dementia, we assessed both multiplicative and additive interactions within fully adjusted Cox proportional hazards models. Multiplicative interaction was examined by adding a product term between MRS (per 1-SD increase) and MQI (per 1-SD decrease), with statistical significance evaluated by the Wald test. Additive interaction was quantified using the relative excess risk due to interaction (RERI) and attributable proportion (AP), computed from the Cox model coefficients and their covariance matrix, with 95% CIs estimated by the delta method. An RERI > 0 with a 95% CI excluding 0 indicated significant positive additive interaction (Wang et al., 2023).

To strengthen causal inference along the muscle-dementia axis, two-sample Mendelian randomization (MR) analyses were performed using summary-level data from large-scale genome-wide association studies (GWASs) restricted to European-ancestry populations (TwoSampleMR and MR-PRESSO) (Ye et al., 2023). As no GWAS is available for the composite MQI, handgrip strength (left hand: ukb-b-7478; right hand: ukb-b-10215; low grip strength per FNIH criteria: GCST90007529), appendicular lean mass (GCST90000025), whole-body fat-free mass (ukb-b-13354), and walking pace (ukb-b-4711) were used as genetically proxied surrogate exposures reflecting muscle strength and mass. Summary statistics for AD were obtained from the IGAP consortium (ieu-b-2) and FinnGen release 12 (endpoint F5_ALZHEIMER), and those for all-cause and vascular dementia from FinnGen release 12 (endpoints F5_DEMENTIA and F5_VASCDEM, respectively).

For each exposure, single-nucleotide polymorphisms (SNPs) reaching genome-wide significance (*p* < 5 × 10^−8^) were selected as instrumental variables and clumped for linkage disequilibrium (r^2^ < 0.001, 10,000-kb window) using the 1,000 Genomes Phase 3 European reference panel; a relaxed threshold (*p* < 5 × 10^−6^) was applied and explicitly reported when fewer than three instruments survived. Palindromic SNPs were aligned using effect allele frequencies, weak instruments (*F* statistic < 10) were excluded, and effect estimates were harmonized across datasets.

The inverse-variance-weighted (IVW) estimator under a multiplicative random-effects model served as the primary analysis, complemented by MR-Egger regression, weighted-median, and simple- and weighted-mode estimators. Horizontal pleiotropy was assessed using the MR-Egger intercept and the MR-PRESSO global test (outlier-corrected estimates reported where appropriate), and heterogeneity was quantified using Cochran’s *Q* statistic. Leave-one-out analyses identified single influential variants. Reverse-direction MR (dementia traits as exposures, muscle traits as outcomes) evaluated potential reverse causation, and multivariable MR jointly modeling grip strength and lean mass estimated their mutually adjusted independent effects.

Several sensitivity analyses were presented as follows: (i) We further adjusted for antihypertensive drugs, cholesterol-lowering medication, aspirin, and insulin use on the basis of Model 3. (ii) Exclusion of dementia occurring within the first 2 years of follow-up to mitigate reverse causation. (iii) Subgroup analyses were further stratified according to age (≥65 years and <65 years), gender, education level (above and below college), smoking status (never, former, and current), alcohol consumption status (never, former, and current), dietary habits (healthy or unhealthy), physical exercise intensity (low, moderate, high) and sleep duration (<7 h, 7–9 h, >9 h). (iv) All-cause mortality was considered a competing event, and multivariate competing risk regression (Fine-Gray model) was employed to assess the associations of MQI, MRS, and MQI + MRS with post-stroke dementia.

## 3 Results

### 3.1 Study population and baseline characteristics

The baseline information of participants is presented in Table 1. A total of 7,047 participants (mean age, 60.45 ± 6.91 years) were included in the primary analysis, in which 4,068 (57.73%) were men, 6,663 (94.55%) were White, and 1,457 (20.80%) had a college degree or above. Participants with higher MQI were more likely to be younger, engage in more physical activity, be current drinkers, and sleep 7–9 h/day, and had a lower BMI. Among participants with hypertension, diabetes, CKD, and loneliness, a higher proportion was observed in those with low MQI levels.

**Table 1:** Baseline characteristics of participants stratified by MQI quartiles.

Characteristic	Overall	MQI				*p*
		Q1	Q2	Q3	Q4	
Participants, *n*	7,047	1761	1761	1761	1764	
Age, years	60.447 ± 6.911	61.224 ± 6.473	60.886 ± 6.697	60.445 ± 6.877	59.234 ± 7.404	<0.001
TDI	−0.392 ± 3.465	0.362 ± 3.684	−0.433 ± 3.395	−0.735 ± 3.337	−0.762 ± 3.316	<0.001
BMI, kg/m^2^	28.855 ± 5.162	31.524 ± 5.812	29.767 ± 4.876	28.089 ± 4.265	26.044 ± 3.802	<0.001
ALB, g/L	44.831 ± 2.761	44.237 ± 2.798	44.776 ± 2.693	44.946 ± 2.796	45.365 ± 2.639	<0.001
HbA1c, %	5.728 ± 1.021	5.977 ± 1.085	5.77 ± 0.918	5.662 ± 1.288	5.508 ± 0.622	<0.001
Gender						1.000
Male	4,068 (57.73)	1,017 (57.75)	1,017 (57.75)	1,017 (57.75)	1,017 (57.65)	
Female	2,979 (42.27)	744 (42.25)	744 (42.25)	744 (42.25)	747 (42.35)	
White race						<0.001
No	384 (5.45)	130 (7.38)	103 (5.85)	75 (4.26)	76 (4.31)	
Yes	6,663 (94.55)	1,631 (92.62)	1,658 (94.15)	1,686 (95.74)	1,688 (95.69)	
Education level						<0.001
Others	5,549 (79.20)	1,462 (83.83)	1,388 (79.18)	1,372 (78.36)	1,327 (75.48)	
College and above	1,457 (20.80)	282 (16.17)	365 (20.82)	379 (21.64)	431 (24.52)	
Smoking status						0.001
Never	2,840 (40.66)	681 (39.21)	732 (41.73)	705 (40.49)	722 (41.21)	
Former	3,009 (43.08)	790 (45.48)	766 (43.67)	759 (43.60)	694 (39.61)	
Current	1,135 (16.25)	266 (15.31)	256 (14.60)	277 (15.91)	336 (19.18)	
Alcohol consumption status						<0.001
Never	455 (6.48)	171 (9.78)	117 (6.66)	99 (5.63)	68 (3.87)	
Former	562 (8.00)	214 (12.24)	113 (6.43)	138 (7.85)	97 (5.51)	
Current	6,005 (85.52)	1,363 (77.97)	1,528 (86.92)	1,520 (86.51)	1,594 (90.62)	
Dietary habits						0.942
Unhealthy	4,175 (59.57)	1,041 (59.69)	1,035 (58.97)	1,052 (59.98)	1,047 (59.62)	
Healthy	2,834 (40.43)	703 (40.31)	720 (41.03)	702 (40.02)	709 (40.38)	
Physical exercise intensity						<0.001
Low	1,231 (19.24)	424 (27.69)	297 (18.54)	247 (15.34)	263 (15.88)	
Moderate	1,268 (19.82)	282 (18.42)	335 (20.91)	340 (21.12)	311 (18.78)	
High	3,900 (60.95)	825 (53.89)	970 (60.55)	1,023 (63.54)	1,082 (65.34)	
Sleep duration						<0.001
<7 h	2008 (28.88)	561 (32.65)	499 (28.58)	489 (28.01)	459 (26.35)	
7–9 h	3,921 (56.40)	849 (49.42)	980 (56.13)	1,006 (57.62)	1,086 (62.34)	
>9 h	1,023 (14.72)	308 (17.93)	267 (15.29)	251 (14.38)	197 (11.31)	
Loneliness						<0.001
No	5,112 (74.17)	1,159 (67.70)	1,266 (73.52)	1,318 (76.27)	1,369 (79.13)	
Yes	1780 (25.83)	553 (32.30)	456 (26.48)	410 (23.73)	361 (20.87)	
Hypertension						<0.001
No	1,463 (20.76)	279 (15.84)	317 (18.00)	380 (21.58)	487 (27.61)	
Yes	5,584 (79.24)	1,482 (84.16)	1,444 (82.00)	1,381 (78.42)	1,277 (72.39)	
Diabetes						<0.001
No	5,864 (83.21)	1,268 (72.00)	1,438 (81.66)	1,551 (88.07)	1,607 (91.10)	
Yes	1,183 (16.79)	493 (28.00)	323 (18.34)	210 (11.93)	157 (8.90)	
CKD						<0.001
No	6,218 (88.24)	1,435 (81.49)	1,548 (87.90)	1,591 (90.35)	1,644 (93.20)	
Yes	829 (11.76)	326 (18.51)	213 (12.10)	170 (9.65)	120 (6.80)	
Lipid-lowering medication						<0.001
No	2,352 (33.68)	499 (28.55)	527 (30.24)	602 (34.48)	724 (41.44)	
Yes	4,632 (66.32)	1,249 (71.45)	1,216 (69.76)	1,144 (65.52)	1,023 (58.56)	
Antihypertensive medication						<0.001
No	2,860 (40.95)	578 (33.07)	636 (36.49)	753 (43.13)	893 (51.12)	
Yes	4,124 (59.05)	1,170 (66.93)	1,107 (63.51)	993 (56.87)	854 (48.88)	
Insulin use						<0.001
No	6,746 (96.59)	1,627 (93.08)	1,683 (96.56)	1710 (97.94)	1726 (98.80)	
Yes	238 (3.41)	121 (6.92)	60 (3.44)	36 (2.06)	21 (1.20)	
Aspirin use						0.003
No	2,861 (40.97)	687 (39.30)	681 (39.07)	715 (40.95)	778 (44.53)	
Yes	4,123 (59.03)	1,061 (60.70)	1,062 (60.93)	1,031 (59.05)	969 (55.47)	
Stroke duration, years	5.777 (2.630–10.624)	5.84 (2.680–10.743)	5.793 (2.571–10.357)	5.728 (2.609–10.754)	5.744 (2.652–10.590)	0.330
Fasting time, hours	3 (2–4)	3 (2–4)	3 (2–4)	3 (3–4)	3 (3–4)	0.033
Serum creatinine, μmol/L	74.7 (64.2–86.8)	74.7 (64.6–89.1)	75.5 (64.6–88.35)	74.6 (64.65–85.95)	73.6 (63.2–84.6)	<0.001
Cys-C, mg/L	0.965 (0.861–1.089)	1.018 (0.91–1.168)	0.977 (0.876–1.094)	0.955 (0.853–1.072)	0.914 (0.824–1.024)	<0.001
eGFR, mL/min/1.73m^2^	83.319 (72.118–94.012)	79.142 (67.171–90.01)	81.992 (71.350–92.786)	84.066 (73.209–94.626)	87.671 (77.336–97.353)	<0.001
CRP, mg/L	1.7 (0.82–3.59)	2.35 (1.11–5.01)	1.83 (0.92–3.698)	1.64 (0.80–3.28)	1.24 (0.61–2.607)	<0.001
LDL-C, mmol/L	2.814 (2.325–3.449)	2.728 (2.256–3.350)	2.798 (2.306–3.353)	2.831 (2.330–3.543)	2.91 (2.409–3.530)	<0.001
GLU, mmol/L	4.994 (4.622–5.497)	5.096 (4.698–5.715)	4.998 (4.620–5.526)	4.98 (4.621–5.458)	4.912 (4.577–5.291)	<0.001
Arm fat-free mass, kg	2.9 (2.2–3.5)	3.1 (2.3–3.7)	3 (2.3–3.6)	2.9 (2.2–3.4)	2.6 (2.1–3.2)	<0.001
Handgrip strength, kg	30 (22–40)	21 (15–30)	30 (22–36)	34 (25–41)	38 (30–46)	<0.001

Data were presented as mean ± standard deviation for normally distributed continuous variables, median (interquartile range) for skewed continuous variables, and number (percentage) for categorical variables. *p*-values were calculated using ANOVA or Kruskal–Wallis test for continuous variables and chi-square test for categorical variables. Q1–Q4 represent quartiles of MQI: Q1 (MQI < 8.824), Q2 (8.824 ≤ MQI < 10.811), Q3 (10.811 ≤ MQI < 12.8), Q4 (MQI ≥ 12.8). MQI, muscle quality index; TDI, Townsend Deprivation Index; BMI, body mass index; CKD, chronic kidney disease; HbA1c, hemoglobin A1c; ALB, albumin; LDL-C, low-density lipoprotein cholesterol; CRP, C-reactive protein; eGFR, estimated glomerular filtration rate; Scr, serum creatinine; Cys-C, cystatin C; GLU, glucose.

### 3.2 The association between the MQI and dementia among stroke survivors

During a median follow-up of 15.09 years, 468 participants developed incident ACD. These comprised 128 AD, 217 VaD, and 123 unspecified dementia cases. The Cox proportional hazards models demonstrated that MQI was significantly associated with post-stroke dementia (all *p* for trend <0.05). Compared with the low MQI group, the high MQI group was associated with significantly lower risks of ACD (HR = 0.51, 95% CI: 0.39–0.67), AD (HR = 0.47, 95% CI: 0.29–0.76), and VaD (HR = 0.59, 95% CI: 0.40–0.88), respectively. For each 1-SD increase in MQI, the risk of ACD, AD, and VaD decreased by 26% (HR = 0.74, 95% CI: 0.67–0.82), 32% (HR = 0.68, 95% CI: 0.57–0.82), and 24% (HR = 0.76, 95% CI: 0.66–0.88), respectively (Table 2).

**Table 2:** Association between MQI and post-stroke dementia.

Variables	MQI	Cases/total *n* (%)	HR (95% CI)	*p*-value	*p* for trend
ACD					
Model 1	Per 1 SD	468/7047 (6.64)	0.77 (0.70–0.84)	<0.001	
	Q1	166/1761 (9.43)	Ref		<0.001
	Q2	113/1761 (6.42)	0.63 (0.50–0.80)	<0.001	
	Q3	94/1761 (5.34)	0.51 (0.40–0.66)	<0.001	
	Q4	95/1764 (5.39)	0.51 (0.40–0.66)	<0.001	
Model 2	Per 1 SD	468/7047 (6.64)	0.78 (0.71–0.85)	<0.001	
	Q1	166/1761 (9.43)	Ref		<0.001
	Q2	113/1761 (6.42)	0.68 (0.53–0.86)	0.002	
	Q3	94/1761 (5.34)	0.55 (0.42–0.71)	<0.001	
	Q4	95/1764 (5.39)	0.57 (0.44–0.74)	<0.001	
Model 3	Per 1 SD	468/7047 (6.64)	0.74 (0.67–0.82)	<0.001	
	Q1	166/1761 (9.43)	Ref		<0.001
	Q2	113/1761 (6.42)	0.66 (0.51–0.84)	0.001	
	Q3	94/1761 (5.34)	0.51 (0.40–0.67)	<0.001	
	Q4	95/1764 (5.39)	0.51 (0.39–0.67)	<0.001	
AD					
Model 1	Per 1 SD	128/7047 (1.82)	0.70 (0.59–0.83)	<0.001	
	Q1	54/1761 (3.07)	Ref		<0.001
	Q2	25/1761 (1.42)	0.43 (0.27–0.69)	0.001	
	Q3	19/1761 (1.08)	0.32 (0.19–0.54)	<0.001	
	Q4	30/1764 (1.70)	0.50 (0.32–0.78)	0.002	
Model 2	Per 1 SD	128/7047 (1.82)	0.73 (0.61–0.88)	0.001	
	Q1	54/1761 (3.07)	Ref		0.003
	Q2	25/1761 (1.42)	0.45 (0.28–0.73)	0.001	
	Q3	19/1761 (1.08)	0.34 (0.20–0.57)	<0.001	
	Q4	30/1764 (1.70)	0.56 (0.35–0.89)	0.014	
Model 3	Per 1 SD	128/7047 (1.82)	0.68 (0.57–0.82)	<0.001	
	Q1	54/1761 (3.07)	Ref		<0.001
	Q2	25/1761 (1.42)	0.43 (0.26–0.69)	0.001	
	Q3	19/1761 (1.08)	0.30 (0.17–0.52)	<0.001	
	Q4	30/1764 (1.70)	0.47 (0.29–0.76)	0.002	
VaD					
Model 1	Per 1 SD	217/7047 (3.08)	0.78 (0.69–0.89)	<0.001	
	Q1	76/1761 (4.32)	Ref		<0.001
	Q2	54/1761 (3.07)	0.67 (0.47–0.94)	0.023	
	Q3	41/1761 (2.33)	0.49 (0.34–0.72)	<0.001	
	Q4	46/1764 (2.61)	0.55 (0.38–0.79)	0.001	
Model 2	Per 1 SD	217/7047 (3.08)	0.80 (0.69–0.92)	0.001	
	Q1	76/1761 (4.32)	Ref		0.010
	Q2	54/1761 (3.07)	0.74 (0.52–1.06)	0.102	
	Q3	41/1761 (2.33)	0.56 (0.38–0.83)	0.004	
	Q4	46/1764 (2.61)	0.66 (0.46–0.97)	0.032	
Model 3	Per 1 SD	217/7047 (3.08)	0.76 (0.66–0.88)	<0.001	
	Q1	76/1761 (4.32)	Ref		0.003
	Q2	54/1761 (3.07)	0.72 (0.50–1.02)	0.067	
	Q3	41/1761 (2.33)	0.53 (0.35–0.78)	0.001	
	Q4	46/1764 (2.61)	0.59 (0.40–0.88)	0.010	

Model 1 was unadjusted. Model 2 was adjusted for age, gender, race, TDI, education level, smoking status, alcohol consumption status, dietary habits, sleep duration, and physical exercise intensity. Model 3 included all variables from Model 2, and further adjusted for BMI, loneliness, and stroke duration. HR, hazard ratio; CI, confidence interval; SD, standard deviation; Ref, reference; MQI, muscle quality index; ACD, all-cause dementia; AD, Alzheimer’s disease; VaD, vascular dementia; TDI, Townsend Deprivation Index; BMI, body mass index.

We examined the dose–response relationship between MQI and the risk of ACD, AD, and VaD, using the RCS regression (Figure 2). A significant linear association was observed in ACD (*p* for overall <0.001; *p* for non-linear = 0.24) and VaD (*p* for overall <0.001; *p* for non-linear = 0.665), indicating a progressively lower risk as MQI increased. A significant non-linear association was found in AD (*p* for overall <0.001; *p* for non-linear = 0.013). Threshold effect analysis identified an inflection point at MQI = 6.732. Below this threshold, AD risk tended to decrease with increasing MQI (HR = 0.85, 95% CI: 0.72–1.00, *p* = 0.053); above it, the inverse association persisted but was attenuated (HR = 0.90, 95% CI: 0.83–0.97, *p* = 0.006). The log-likelihood ratio test supported this non-linear relationship (*p* = 0.007; Supplementary Table S4).

**Figure 2:**
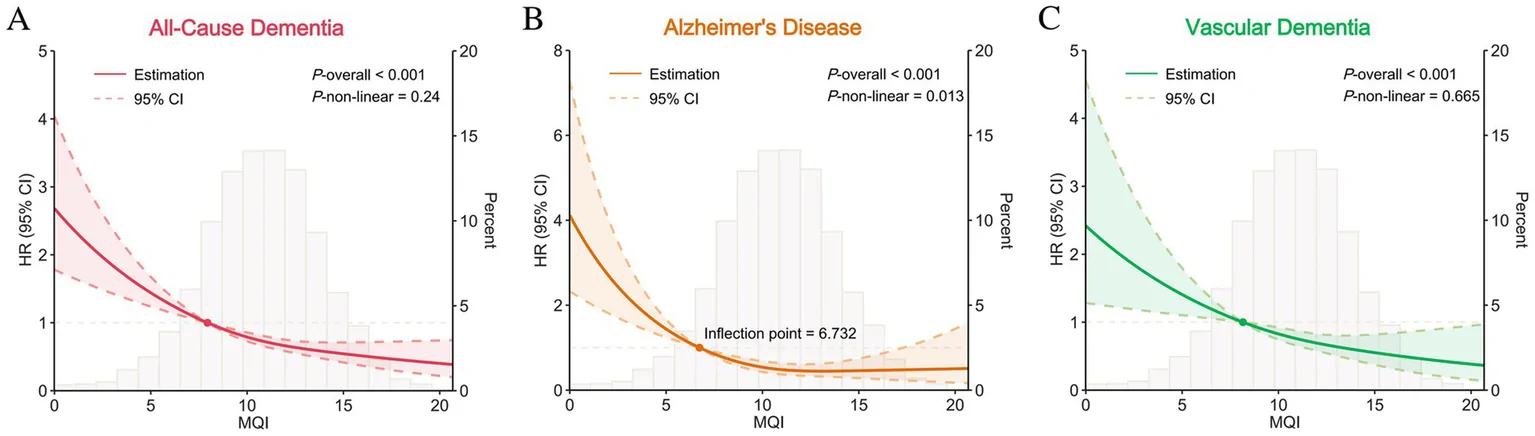
The restricted cubic spline curves of *MQI* for post-stroke dementia **(A)** All-cause dementia: Significant linear negative association (*p* for overall <0.001, *p* for non-linear = 0.240); a higher MQI was associated with a progressively lower risk of all-cause dementia. **(B)** Alzheimer’s disease: Significant non-linear association (*p* for overall <0.001, *p* for non-linear = 0.013), with an inflection point at MQI = 6.732. **(C)** Vascular dementia: Significant linear negative association (*p* for overall <0.001, *p* for non-linear = 0.665); a higher MQI was associated with a progressively lower risk of vascular dementia.

### 3.3 Identification of the metabolites related to dementia by LASSO regression and XGBoost

We performed metabolite screening following the approach described in the methods section. Within the training set, LASSO regression retained 33, 33, and 22 metabolites with non-zero coefficients for ACD, AD, and VaD, respectively, at the *λ* values determined under the lambda.min rule (Supplementary Figure S2; Supplementary Tables S5–S7). The selected features spanned glycolysis-related metabolites (glucose, glucose-lactate, citrate, pyruvate), ketone bodies (3-hydroxybutyrate, acetoacetate), amino acids (glutamine, glycine, alanine, tyrosine), and lipoprotein lipid composition across very-low-density-lipoprotein (VLDL), intermediate-density-lipoprotein (IDL), low-density-lipoprotein (LDL), and high-density-lipoprotein (HDL) subclasses. Ketone body 3-hydroxybutyrate exhibited the largest positive coefficient for ACD, whereas citrate and the percentage of docosahexaenoic acid to total fatty acids exhibited the largest positive coefficients for AD and VaD, respectively.

XGBoost modeling of the LASSO-selected features yielded SHAP-based importance rankings that broadly corroborated the penalized regression results (Figure 3; Supplementary Figure S3). Citrate and albumin ranked among the top features across all three outcomes; outcome-specific leading features included cholesteryl ester content of IDL for ACD (mean SHAP contribution 13.1%), free cholesterol percentage in chylomicrons and extremely large VLDL for AD (15.1%), and small-HDL phospholipids together with the omega-6 to omega-3 fatty acid ratio for VaD (13.7 and 13.0%; Figures 3A1–A3). SHAP dependence analyses further suggested non-linear, threshold-like effects for several leading metabolites, including citrate and albumin (Figures 3B1–B3).

**Figure 3:**
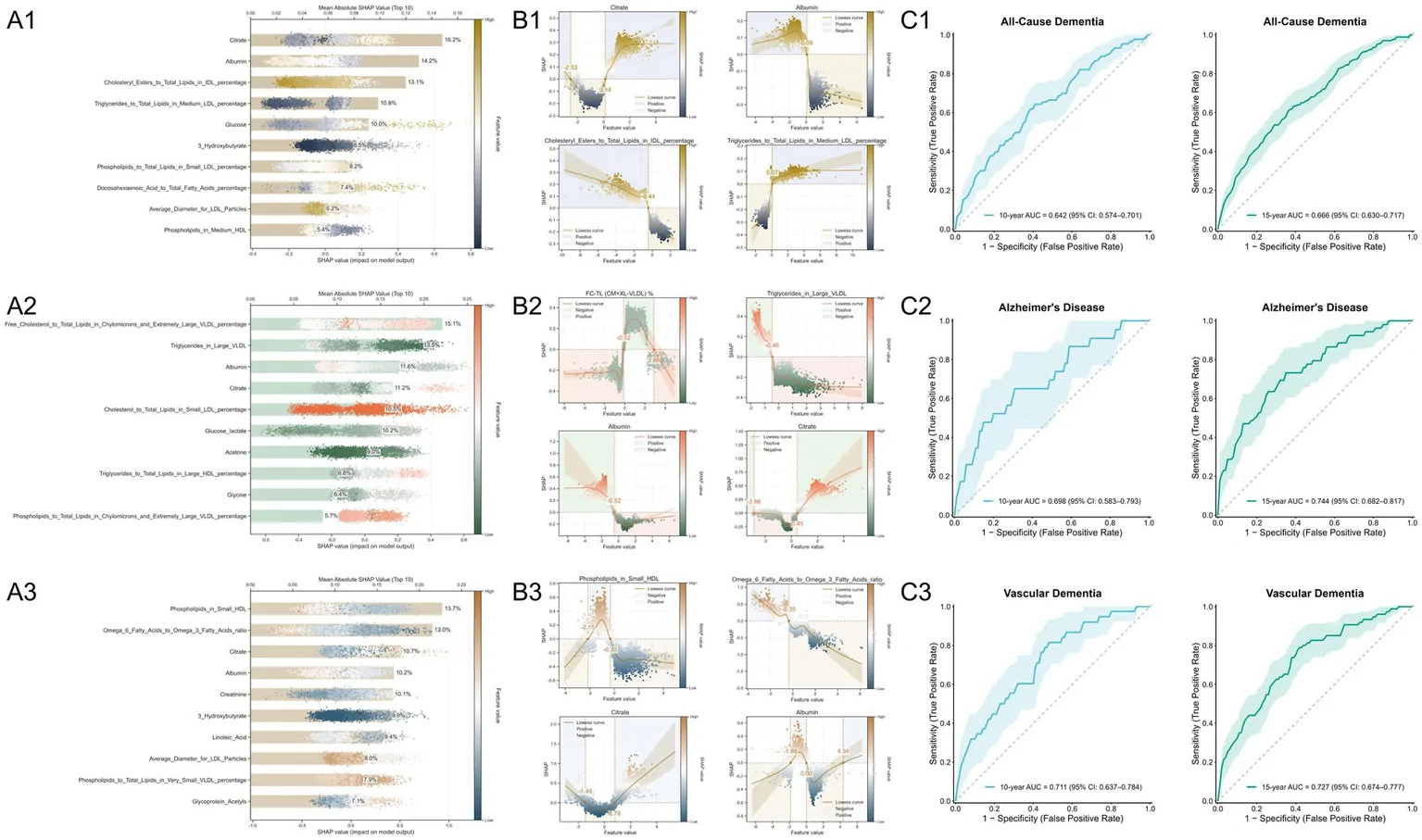
Post-LASSO machine-learning selection of top 10 metabolites and time-dependent ROC validation. **(A1–A3)** SHAP summary plots ranking the top 10 metabolites for all-cause dementia **(A1)**, Alzheimer’s disease **(A2)**, and vascular dementia **(A3)**. Bars indicate relative importance based on mean absolute SHAP values; each dot represents one participant, with color denoting the feature value from low to high. Positive SHAP values indicate higher predicted risk, whereas negative values indicate lower predicted risk. **(B1–B3)** SHAP dependence plots with LOWESS curves for representative leading variables, showing nonlinear effects and direction shifts across feature values; shaded areas indicate positive or negative SHAP regions. **(C1–C3)** Time-dependent ROC curves in the validation set for 10-year and 15-year prediction of all-cause dementia **(C1)**, Alzheimer’s disease **(C2)**, and vascular dementia **(C3)**. The shaded bands represent 95% confidence intervals, and the dashed line indicates the reference. The corresponding AUCs were as follows: all-cause dementia, 10-year AUC = 0.642 (95% CI: 0.574–0.701) and 15-year AUC = 0.666 (95% CI: 0.630–0.717); Alzheimer’s disease, 10-year AUC = 0.698 (95% CI: 0.583–0.793) and 15-year AUC = 0.744 (95% CI: 0.682–0.817); vascular dementia, 10-year AUC = 0.711 (95% CI: 0.637–0.784) and 15-year AUC = 0.727 (95% CI: 0.674–0.777).

In the validation cohort, time-dependent ROC analysis showed that the metabolite signatures discriminated incident events with 10-year AUCs of 0.642 (95% CI: 0.574–0.701) for ACD, 0.698 (0.583–0.793) for AD, and 0.711 (0.637–0.784) for VaD; corresponding 15-year AUCs were 0.666 (0.630–0.717), 0.744 (0.682–0.817), and 0.727 (0.674–0.777), respectively (Figures 3C1–C3). Collectively, these results delineate both shared and subtype-specific candidate biomarkers across distinct dementia phenotypes. Each MRS was constructed as a weighted sum of the selected metabolites.

### 3.4 The association between the MRS and post-stroke dementia

In the validation cohort, 127 ACD (6.22%), 34 AD (1.67%), and 55 VaD (2.69%) events occurred during follow-up. After full covariate adjustment, each 1-SD increment in the outcome-specific MRS was associated with a 63% higher risk of ACD (HR = 1.63, 95% CI: 1.36–1.96, *p* < 0.001), a 140% higher risk of AD (HR = 2.40, 95% CI: 1.74–3.31, *p* < 0.001), and a 92% higher risk of VaD (HR = 1.92, 95% CI: 1.46–2.54, *p* < 0.001). Tertile analyses supported a graded relationship. Compared with the lowest MRS tertile, participants in the highest tertile had significantly elevated risks of ACD (HR = 2.34, 95% CI: 1.48–3.70, *p* < 0.001), AD (HR = 3.81, 95% CI: 1.40–10.38, *p* = 0.009), and VaD (HR = 3.79, 95% CI: 1.60–8.98, *p* = 0.002). Estimates for the middle tertile did not differ significantly from the reference group for any outcome, with excess risk concentrated in the highest tertile (Supplementary Table S8).

### 3.5 Incremental discrimination of MQI and MRS

Adding MQI and MRS, individually or in combination, to the base clinical model yielded graded improvements in discrimination for all three outcomes. C-indices rose from 0.718 to 0.756 for ACD, from 0.786 to 0.850 for AD, and from 0.771 to 0.813 for VaD (Supplementary Table S9). Time-dependent AUCs followed the same pattern (Figure 4; Supplementary Table S10): at 15 years, the combined model improved the AUC by 0.038 for ACD (*p* < 0.001), 0.066 for AD (*p* = 0.001), and 0.043 for VaD (*p* = 0.008). At 10 years, the increments did not reach statistical significance for any outcome (ΔAUC = 0.021, *p* = 0.147 for ACD; 0.054, *p* = 0.130 for AD; 0.030, *p* = 0.240 for VaD), plausibly owing to fewer events accrued by mid-follow-up (Supplementary Figure S4; Supplementary Table S11). Reclassification analyses corroborated these gains: at 15 years, the combined model yielded category-free NRIs of 0.335 (95% CI: 0.210–0.583), 0.788 (0.501–1.050), and 0.648 (0.349–0.840), and IDIs of 0.023 (0.009–0.052), 0.064 (0.025–0.132), and 0.032 (0.014–0.099), for ACD, AD, and VaD, respectively (Supplementary Table S12). Calibration plots showed good agreement between predicted and observed risks, Brier scores decreased with each added predictor block at 15 years (5-year ACD: 0.0630 vs. 0.0612; AD: 0.0210 vs. 0.0193; VaD: 0.0297 vs. 0.0283), and decision curve analysis indicated the highest net benefit for the combined model across most clinically relevant thresholds at both time points (Figure 4; Supplementary Table S13). Together, MQI and MRS provided complementary prognostic information, and their combination offered modest but consistent incremental value over clinical factors alone.

**Figure 4:**
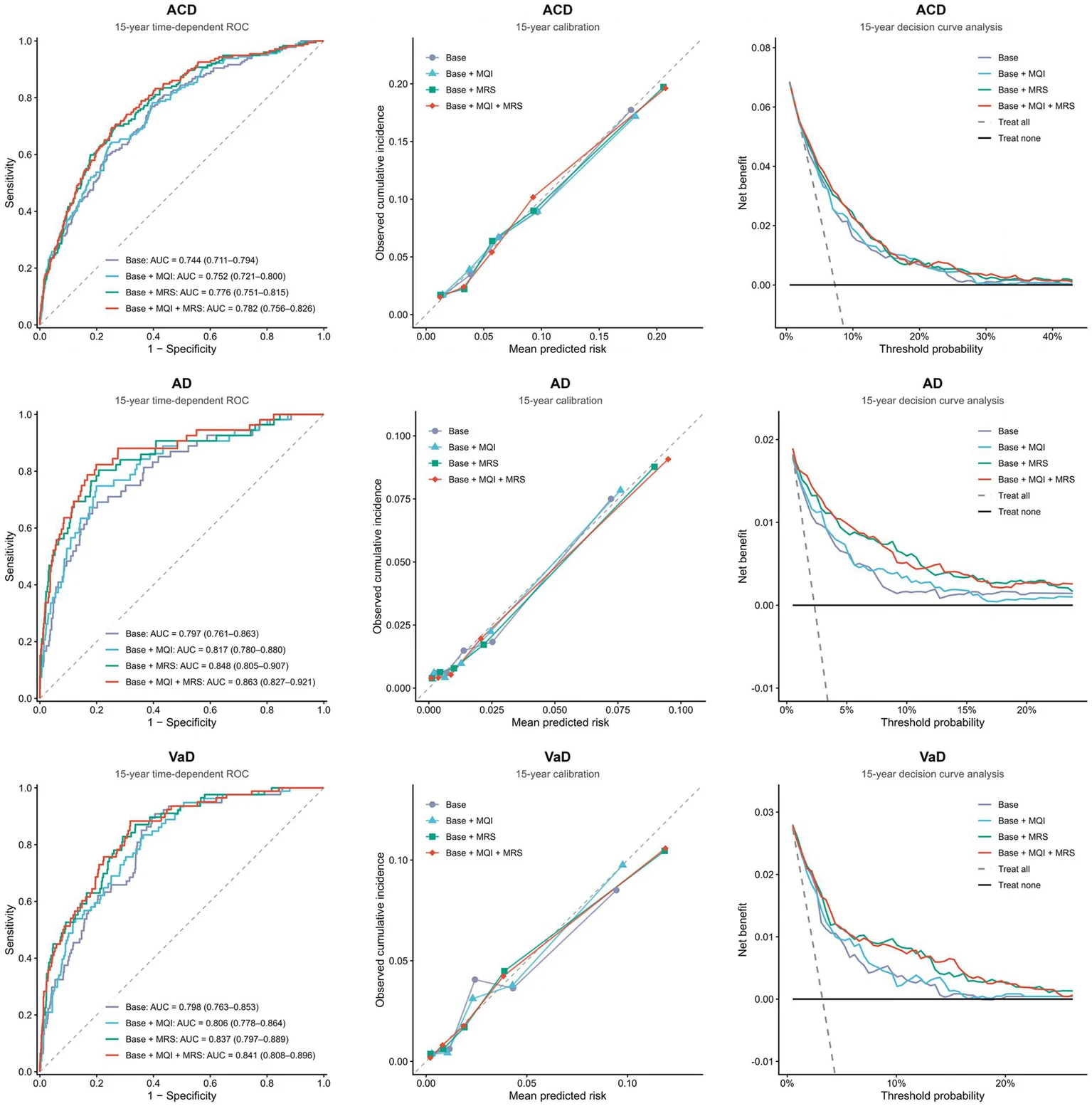
Incremental predictive performance of MQI and MRS beyond the Base model. Rows show 15-year prediction of all-cause dementia (ACD, top), Alzheimer’s disease (AD, middle), and vascular dementia (VaD, bottom). Left: Time-dependent ROC curves. For ACD, AUCs (95% CI) were 0.744 (0.711–0.794) for the Base model, 0.752 (0.721–0.800) for Base + MQI, 0.776 (0.751–0.815) for Base + MRS, and 0.782 (0.756–0.826) for Base + MQI + MRS; corresponding AUCs for AD were 0.797 (0.761–0.863), 0.817 (0.780–0.880), 0.848 (0.805–0.907), and 0.863 (0.827–0.921); and for VaD were 0.798 (0.763–0.853), 0.806 (0.778–0.864), 0.837 (0.797–0.889), and 0.841 (0.808–0.896). Middle: Calibration plots of observed cumulative incidence against mean predicted risk, with the dashed line indicating perfect agreement. Right: Decision curve analysis, showing the net benefit of each model across threshold probabilities relative to “treat all” and “treat none” strategies.

### 3.6 Joint associations of MRS and MQI with post-stroke dementia

Joint analysis revealed a consistent risk gradient across the combined MRS-MQI strata for all three outcomes (Figure 5; Supplementary Table S14). Relative to participants with low-risk MRS and the highest MQI quartile (reference), those with high-risk MRS and the lowest MQI quartile carried the greatest risk of ACD (11.28% vs. 2.37%; HR = 3.29, 95% CI: 1.91–5.68, *p* < 0.001), AD (4.57% vs. 0.51%; HR = 5.83, 95% CI: 1.75–19.37, *p* = 0.004), and VaD (7.24% vs. 1.40%; HR = 4.57, 95% CI: 2.28–9.14, *p* < 0.001). Each dimension contributed independently to the gradient: within the low-risk MRS stratum, the lowest MQI quartile was associated with elevated ACD risk (HR = 2.20, 95% CI: 1.19–4.04), whereas high-risk MRS remained hazardous even among participants with the most favorable muscle quality (Q4: HR = 2.24 for ACD, 4.55 for AD, and 2.30 for VaD; all *p* < 0.05).

**Figure 5:**
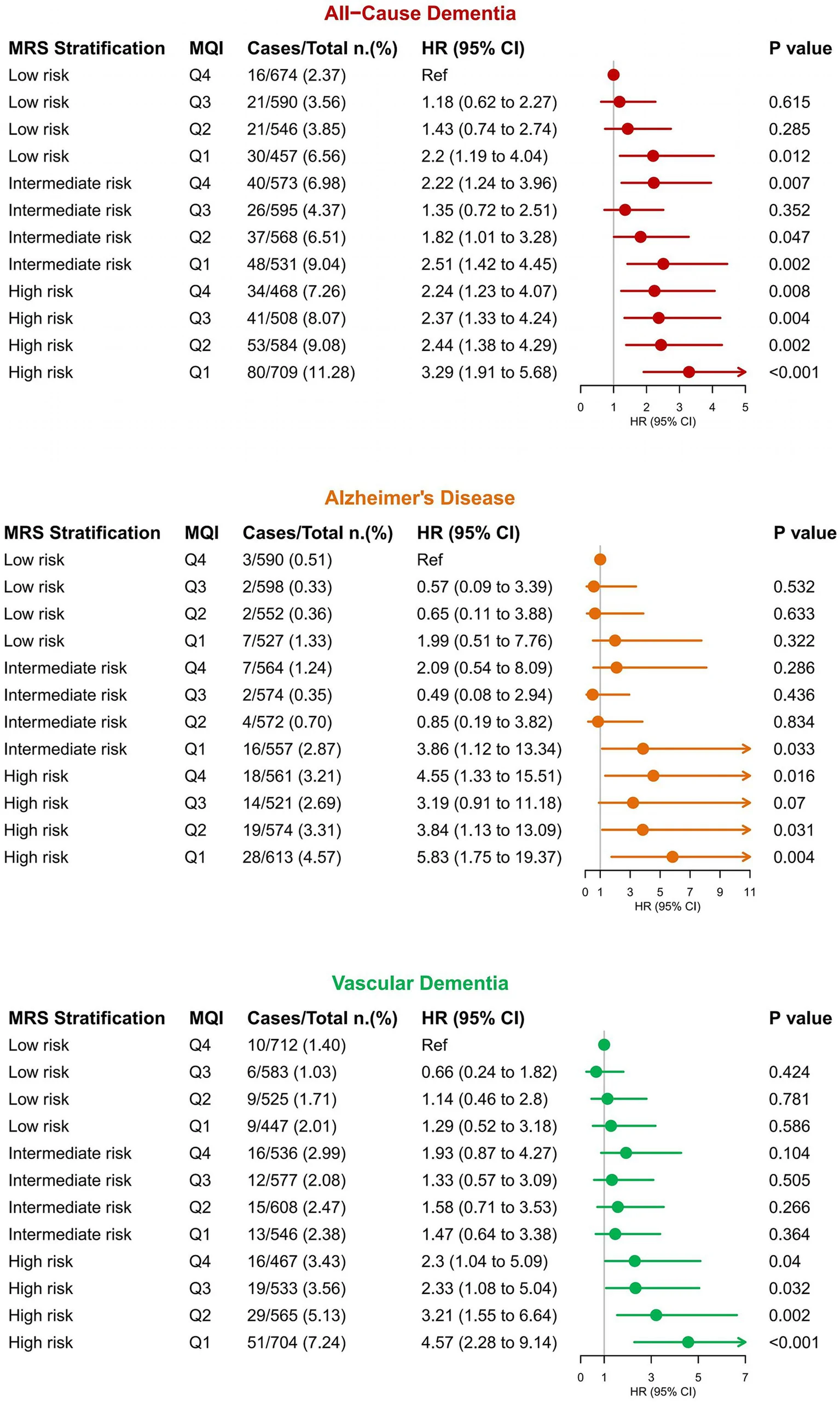
Joint association of MRS and MQI with dementia risk. HR (95% CIs) for ACD, AD and VaD across joint strata of MRS (low, intermediate, and high risk) and MQI quartiles (Q1–Q4), using low MRS risk with MQI Q4 as the reference. Case numbers and percentages are shown for each stratum. Compared with the reference group, participants with high MRS risk and the lowest MQI quartile (Q1) had the highest risk of ACD (HR = 3.29, 95% CI 1.91–5.68), AD (HR = 5.83, 95% CI 1.75–19.37), and VaD (HR = 4.57, 95% CI 2.28–9.14).

Additive interaction analyses indicated that the joint exposure exceeded the sum of the individual effects. The relative excess risk due to interaction was 0.086 (95% CI: 0.011–0.162) for ACD, 0.050 (0.007–0.094) for AD, and 0.094 (0.028–0.161) for VaD, with attributable proportions of 3.3% (0.1–6.4%), 3.9% (0.1–7.7%), and 6.8% (1.1–12.6%), respectively. No multiplicative interaction was detected for any outcome (all 95% CIs of the product term included 1; Supplementary Table S15).

### 3.7 Mendelian randomization analyses

A total of 24 exposure–outcome pairs were tested. In the primary multiplicative random-effects IVW analysis, each 1-SD increment in genetically proxied appendicular lean mass (ALM) was linked to a 10% lower risk of Alzheimer’s disease (AD; IGAP, Bellenguez et al., 2022; OR = 0.903, 95% CI 0.865–0.944, *p* = 5.1 × 10^−6^; 578 SNPs). Estimates from MR-Egger (OR = 0.903), weighted median (OR = 0.922), and outlier-corrected MR-PRESSO (OR = 0.907) pointed in the same direction, and the MR-Egger intercept gave no indication of directional pleiotropy (intercept = 0.0005, *p* = 0.30). A similar inverse relationship held for whole-body fat-free mass (IVW OR = 0.774, 95% CI 0.717–0.835, *p* = 3.3 × 10^−11^; 517 SNPs), which was corroborated by the complementary estimators (MR-Egger OR = 0.774; weighted median OR = 0.787; MR-PRESSO corrected OR = 0.790) and a null Egger intercept (*p* = 0.77). Cochran’s Q test flagged substantial between-instrument heterogeneity for both traits (Q *p* < 1 × 10^−13^); however, the agreement among pleiotropy-robust methods, together with the null intercepts, argues for balanced rather than directional bias (Supplementary Figure S5; Supplementary Tables S16, S17).

Grip strength showed a borderline inverse relationship with vascular dementia in the univariable setting (IVW OR = 0.449, 95% CI 0.198–1.020, *p* = 0.056; 142 SNPs). When grip strength and ALM were modeled jointly, this association became considerably stronger for grip strength (OR = 0.163, 95% CI 0.042–0.640, *p* = 9.3 × 10^−3^) but not for ALM (OR = 1.349, *p* = 0.097), suggesting that strength may protect against vascular cognitive impairment through pathways that do not depend on muscle mass. The pattern reversed for AD: ALM kept an independent inverse association (OR = 0.923, 95% CI 0.860–0.991, *p* = 0.027), whereas grip strength showed none (*p* = 0.40). Walking pace and the binary low-grip-strength phenotype, the latter supported by only three to four instruments, yielded no significant univariable estimates, and no association emerged for all-cause dementia or FinnGen-defined AD.

Reverse MR analyses found little support for a causal influence of AD or all-cause dementia liability on grip strength or lean mass (all IVW *p* > 0.08). The few nominal signals observed for walking pace (AD: *p* = 3.4 × 10^−4^; FinnGen AD: *p* = 1.3 × 10^−3^) rested on very small instrument sets and more plausibly reflect pleiotropy than true reverse causation. Reverse testing for vascular dementia could not be carried out, as fewer than three instruments remained even at a relaxed threshold of *p* < 5 × 10^−6^ (Figure 6; Supplementary Tables S18, S19).

**Figure 6:**
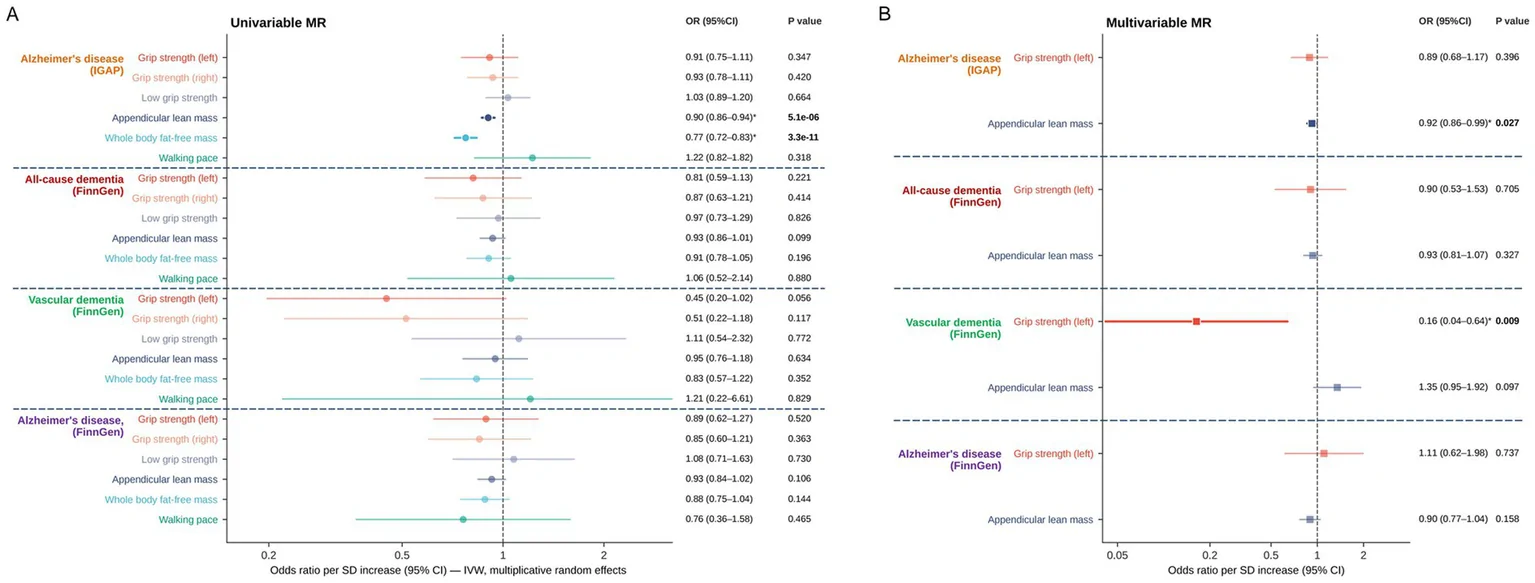
Mendelian randomization estimates of associations between muscle quality index–related traits and dementia outcomes. **(A)** Univariable Mendelian randomization analyses of left and right grip strength, low grip strength, appendicular lean mass, whole-body fat-free mass, and walking pace. **(B)** Multivariable Mendelian randomization analyses of left grip strength and appendicular lean mass. Univariable estimates were obtained using the inverse variance weighted method with multiplicative random effects. For continuous exposures, odds ratios are expressed per 1-standard deviation increase in the genetically predicted trait. Points represent odds ratios, horizontal lines represent 95% confidence intervals, and the vertical dashed line indicates the null value. Estimates were considered statistically significant at *p* < 0.05. AD outcomes were obtained from IGAP and FinnGen; ACD and VaD were obtained from FinnGen.

### 3.8 Sensitivity analysis

This study evaluated the stability of the results through four sensitivity analyses, with the specific findings outlined as follows: 1. After further adjusting for antihypertensive drugs, cholesterol-lowering medication, aspirin, and insulin use on the basis of Model 3, the association between MQI and ACD (HR = 0.56, 95% CI: 0.42–0.74), AD (HR = 0.50, 95% CI: 0.31–0.83), and VaD (HR = 0.66, 95% CI: 0.44–0.98) remained statistically significant, suggesting that the association was not confounded by those drugs (Supplementary Table S20). 2. The main findings remained essentially unchanged after excluding 11 participants who developed dementia within the first 2 years of follow-up. In Model 3, the HRs for the highest vs. lowest MQI quartile were 0.52 (95% CI: 0.39–0.69) for ACD, 0.46 (95% CI: 0.28–0.75) for AD, and 0.62 (95% CI: 0.41–0.92) for VaD (Supplementary Table S21). 3. We observed no statistically significant interaction between MQI and any of the prespecified stratification factors (all *p* for interaction > 0.05), suggesting that the association was not strongly modified by age, gender, or lifestyle characteristics. However, estimates were imprecise in some smaller strata, where CIs were wide and included the null, but they largely pointed in the same protective direction (Supplementary Table S22). 4. We further used Fine-Gray competing risk models, treating all-cause death as the competing event, to confirm the robustness of our findings. These analyses showed that the inverse association between MQI and post-stroke dementia in the overall cohort (after excluding early events), the positive MRS association in the validation cohort, and the joint MRS-MQI risk gradient all remained consistent with the primary results (Supplementary Tables S23–S25).

## 4 Discussion

This study confirms the association of combined MQI and plasma metabolomics with post-stroke dementia by integrating observational, interaction, and genetic evidence, revealing the synergistic value of both in predicting the risk of post-stroke dementia. The core finding not only verifies the association between muscle quality and metabolic status but also provides a risk stratification approach with substantial potential for clinical translation. Notably, a high MQI was inversely associated with the risk of ACD, AD, and VaD after stroke (a 24–32% reduction in risk for each 1-SD increase). These findings suggest that the observed association may not solely reflect muscle mass reserves but may also involve muscle functional efficiency, complementing previous studies focused primarily on muscle mass and indicating that rehabilitation management for stroke patients could consider not only muscle mass recovery but also muscle function training and circulating metabolite levels to help reduce the risk of cognitive decline (Cramer et al., 2023; Zhang A. et al., 2025; Zhang K. et al., 2025).

Previous studies have shown that grip strength is associated with brain health indicators in both men (HR = 1.20, 95% CI: 1.12–1.28) and women (HR = 1.12, 95% CI: 1.00–1.26), and interventions to enhance muscle strength, especially those targeting middle-aged individuals, may help maintain brain health (Duchowny et al., 2022). This precisely supports that MQI has more advantages as an indicator for assessing the risk of dementia. A previous study reported that individuals with high MQI were associated with a significantly lower risk of dementia (HR = 0.69, 95% CI: 0.64–0.76), compared to those with low MQI (Wang X. et al., 2025; Wang Y., et al., 2025). Our findings further verify that the combined MQI-MRS offers an improved solution for clinical risk screening. Individuals with both high MRS and the lowest MQI exhibited hazard ratios for ACD, AD, and VaD as high as 3.29, 5.83, and 4.57, respectively, indicating that stroke patients with both metabolic dysfunction and low muscle quality are at especially high risk of dementia and may warrant intensified follow-up.

Although metabolite screening was performed independently for each outcome, the three outcome-specific scores shared citrate and albumin as leading contributors, pointing to a common metabolic substrate of post-stroke dementia involving mitochondrial energy metabolism and hepatic synthetic function. Beyond these shared features, subtype-specific patterns were also informative: lipoprotein compositional traits dominated in a subtype-specific manner, with chylomicron and large VLDL cholesterol content most influential for AD and small HDL phospholipids with the omega-6 to omega-3 ratio for VaD, consistent with the tighter coupling of vascular cognitive impairment to atherogenic and pro-inflammatory lipid balance (Morgan and Mc Auley, 2024; Chen et al., 2025). SHAP dependence analyses further revealed threshold-like, non-linear effects for several leading metabolites, including a sign reversal of citrate around its population mean and a steep inflection of albumin at low concentrations, patterns that conventional linear models would miss. In the validation cohort, the selected metabolites alone showed moderate discrimination (15-year AUC 0.666–0.744), indicating that circulating metabolites capture one layer of a multifactorial process and that their value lies in complementing clinical and functional measures, as reflected in the incremental performance of the combined MQI-MRS model.

The combined MQI-MRS model significantly improved the AUC values for ACD, AD, and VaD at 15-year follow-up, especially for AD, with a 15-year prediction AUC of 0.863. At 10 years, the observed increments for ACD, AD, and VaD did not reach statistical significance (*p* = 0.147, 0.130, and 0.240, respectively), plausibly owing to fewer events accrued by mid-follow-up. MQI captures the functional integrity of skeletal muscle, whereas MRS reflects the systemic metabolic state; their incremental benefit is well established. Across all outcomes, MRS contributed larger increments than MQI, most notably for AD. This may reflect the long preclinical phase of AD, during which peripheral metabolic alterations accumulate and warrant further longitudinal profiling of metabolite trajectories (Xie et al., 2023). Furthermore, predicted risks aligned with observed cumulative incidences, and the combined model yielded the highest net benefit across most clinically relevant thresholds at both 10 and 15 years, providing a basis for risk stratification, for instance, identifying stroke survivors who may benefit from intensified cognitive monitoring.

Previous studies have found some patients with stroke had high glucose metabolism and low levels of omega-6 fatty acids at baseline, but these metabolic indicators gradually improved through subsequent dietary adjustments and exercise rehabilitation (Andone et al., 2025; Bian et al., 2025). Long-term bed rest after a stroke may exacerbate lipid metabolism disorders, gradually increasing the concentration of pro-inflammatory lipids, but standardized rehabilitation training may enhance the levels of neuroprotective metabolites (Haley et al., 2020; Yousaf et al., 2026). Beyond these clinical risk correlations, distinct metabolic profiles were observed across different dementia subtypes, suggesting metabolic heterogeneity among them. The damage targets of ACD involve multilevel crosstalk among neurons, blood vessels and the metabolic system, causing metabolite alterations characterized by widespread dysregulation across multiple rather than single metabolic pathways (Liu et al., 2025; Rexach et al., 2024; Hainsworth et al., 2024). Consistent with prior studies, citrate plays a critical role in dementia, regulating cerebral energy metabolism, neurotransmitter synthesis, and the primary pathological hallmarks of dementia; its dysregulation is both a consequence of dementia and a key driver of cognitive decline (Zhang et al., 2022). The metabolite profile of VaD is significantly different from that of other dementia subtypes. It is mainly associated with vascular inflammation-related metabolites (e.g., 3-hydroxybutyric acid and the omega-6/omega-3 fatty acid ratio) and atherosclerosis-related lipids (e.g., different density lipoprotein metabolites) (Ahn et al., 2025).

The linear relationships further characterized the association between MQI and post-stroke dementia. We observed inverse linear relationships between MQI and both ACD and VaD. These findings provide preliminary evidence that sustained improvements in muscle quality could potentially correlate with reduced long-term dementia risk. For AD, the existence of an inflection point (MQI = 6.732) provides a threshold reference for risk-stratified strategies. When MQI was below this threshold, the benefit of enhanced muscle strength training appeared more pronounced, although this difference did not reach statistical significance; beyond this level, further increases in MQI had an attenuated marginal preventive effect on AD. This observation may encourage further investigation of potential measures, such as cognitive training or neuroprotective therapies.

The significant additive interaction between MRS and MQI in ACD, AD, and VaD (with RERI values of 0.086, 0.050, and 0.094; A*p* values of 3.3, 3.9, and 6.8%, respectively) suggests that their joint effect may exceed simple additive expectations, pointing toward a potential synergistic interplay between metabolic-muscle-vascular pathways (Livshits and Kalinkovich, 2019; Zhang et al., 2024). This interaction accords with cohort evidence that sarcopenia and cardiometabolic risk factors jointly accelerate cognitive decline (Liu et al., 2024), and with the shared mechanistic pathways linking metabolic syndrome, sarcopenia, and dementia (Ceccarelli and Solerte, 2025). Research has shown that a decline in muscle quality may exacerbate vascular damage induced by metabolic disorders, thereby accelerating cognitive decline (St-β = 0.172, 95% CI: 0.019–0.344) (van Dinther et al., 2024), consistent with the recognized vascular impairment, muscle atrophy, and cognitive decline cascade and the contribution of endothelial dysfunction to muscle deterioration (de Lima et al., 2024; Jeon et al., 2021). Whether this additive synergy can actually be modified remains to be tested in randomized trials that combine metabolic management with resistance training among stroke survivors.

The association between MQI and dementia remained consistent across multiple sensitivity analyses. None of the pre-specified subgroup factors showed significant effect modification (all *p* for interaction >0.05). Borderline interactions were observed for alcohol consumption status and physical exercise intensity regarding AD (*p* = 0.056 and 0.097, respectively), which warrant further validation in larger cohorts. Further adjustment for medications (antihypertensive drugs, lipid-lowering medication, aspirin, and insulin) did not substantially alter the inverse associations, reducing the potential confounding by pharmacological treatment. Similarly, after excluding participants with incident dementia within the first 2 years of follow-up, the conclusions remained consistent with the primary analysis. All competing-risk models yielded consistent results, which remained robust after accounting for death as a competing event. These findings demonstrate that the association between MQI and dementia is statistically stable across diverse analytical strategies.

MR analyses add suggestive causal evidence to these observational findings, and help clarify which aspect of muscle health matters for different dementia subtypes. Genetically proxied lean mass was consistently linked to reduced AD risk across pleiotropy-robust estimators, consistent with prior MR work and the hypothesis that skeletal muscle acts as a metabolic-endocrine organ capable of mitigating brain insulin resistance via myokine signaling (Ye et al., 2023; Zhu et al., 2024; Huang et al., 2025; Song et al., 2024). By contrast, genetically predicted grip strength exhibited a contrasting association: it showed minimal relation to AD, yet appeared as a notable independent correlate of VaD after accounting for lean mass. This suggests that indicators such as muscle strength may reflect stroke functional status, hemiparesis and disability, rather than a direct intrinsic muscle-brain biological pathway (Matsushita et al., 2022; Arosio et al., 2023). In addition, grip strength may reflect broader vascular and functional traits, including endothelial integrity, cerebral perfusion, and physical fitness, that are partially independent of muscle mass per se (Biessels et al., 2026; Yoo et al., 2018). Thus, the combination of MQI (composite measure of muscle strength and mass) and metabolic features may improve insight into underlying biological mechanisms and partially explain its consistent associations across dementia outcomes.

### 4.1 Strengths and limitations

The findings from this study suggest potential translational implications. The measurement of MQI only requires a handgrip dynamometer and a body composition analyzer, and plasma metabolites can be detected through a standardized NMR platform. The combined effect of MRS and MQI exhibits promising prospects for clinical application, making it especially suitable for routine risk screening during post-stroke rehabilitation.

However, its limitations must also be acknowledged. Firstly, the UK Biobank cohort is mainly composed of White individuals, and the research results may not be directly applicable to other ethnic groups. Secondly, the detection of plasma metabolites in a non-fasting state may be affected by diet, and some short-term fluctuating metabolic indicators may not have been fully captured. Thirdly, this study did not include key variables such as the severity of stroke and rehabilitation treatment plans, which may interfere with the results by affecting the recovery of muscle function. Fourthly, several null results likely reflect limited power in MR analyses, and the general-population GWASs restrict these estimates to direction rather than post-stroke effect size. Future studies should conduct multi-center, ethnically diverse cohorts, combined with longitudinal monitoring of muscle function and metabolic dynamics, to further validate the applicability of this integrated risk-assessment approach and to clarify the potential causal pathways linking muscle quality, metabolic profiles, and dementia.

## 5 Conclusion

This study provides preliminary evidence supporting the combined use of MQI and plasma metabolomics for predicting post-stroke dementia. It reveals the potential synergistic role of muscle quality and metabolic features in dementia. These findings offer new perspectives for early risk stratification, with the potential to improve long-term prognostic outcomes among stroke survivors.

## Data Availability

The datasets presented in this study can be found in online repositories. The names of the repository/repositories and accession number(s) can be found at: www.ukbiobank.ac.uk, UK Biobank.

## Glossary

ACD: all-cause dementia
AD: Alzheimer’s disease
ALB: albumin
ALM: appendicular lean mass
ANOVA: analysis of variance
AP: attributable proportion
AUC: area under the curve
BMI: body mass index
BUN: blood urea nitrogen
CI: confidence interval
CKD: chronic kidney disease
CRP: C-reactive protein
Cys-C: cystatin C
DAG: directed acyclic graph
EDTA: ethylenediaminetetraacetic acid
eGFR: estimated glomerular filtration rate
FBG: fasting blood glucose
GLU: glucose
GWAS: genome-wide association study
HbA1c: hemoglobin A1c
HDL: high-density lipoprotein
HDL-C: high-density lipoprotein cholesterol
HES: hospital episode statistics
HGS: handgrip strength
HR: hazard ratio
ICD-10: International Classification of Diseases, Tenth Revision
IDI: integrated discrimination improvement
IDL: intermediate-density lipoprotein
IPCW: inverse probability of censoring weights
IVW: inverse-variance weighted
LASSO: least absolute shrinkage and selection operator
LDL: low-density lipoprotein
LDL-C: low-density lipoprotein cholesterol
MICE: Multiple Imputation by Chained Equations
MQI: muscle quality index
MR: Mendelian randomization
MR-PRESSO: Mendelian Randomization Pleiotropy RESidual Sum and Outlier
MRS: metabolomics risk score
NMR: nuclear magnetic resonance
NRI: net reclassification improvement
OR: odds ratio
RCS: restricted cubic spline
RERI: relative excess risk due to interaction
ROC: receiver operating characteristic
Scr: serum creatinine
SD: standard deviation
SHAP: SHapley Additive exPlanations
SNP: single-nucleotide polymorphism
TC: total cholesterol
TD-ROC: time-dependent receiver operating characteristic
TDI: Townsend Deprivation Index
TG: triglycerides
Ua: uric acid
UKB: UK Biobank
VaD: vascular dementia
VIF: variance inflation factor
VLDL: very-low-density lipoprotein
XGBoost: extreme gradient boosting
