# Supplementary material for: Muscle quality index combined with plasma metabolomics: enhancing risk stratification for post-stroke dementia
Source: Front Aging Neurosci. 2026 Sep 29;18:1837030. doi: 10.3389/fnagi.2026.1837030 (PMC13658159; doi:10.3389/fnagi.2026.1837030)
Supplement: Supplementary file 1 [file Data_Sheet_1.DOCX]

**MRSs calculation**

$$\text{MRS}\text{ = }\sum_{\text{i}\text{ }\text{=}\text{1}}^{\text{k}} \text{β}_{\text{i}}\text{ × }\text{Z}\left( \text{metabolite}_{\text{i}} \right)$$

Where *βi* = the non-zero coefficient of the ith metabolite among the top 10 ranked by XGBoost/SHAP, obtained from the LASSO regression; *Z(metabolite_i_)* = the standardized value of the ith metabolite in the training cohort; *k* = the number of metabolites included in the final outcome-specific MRS; here, *k* = 10, corresponding to the top 10 metabolites ranked by XGBoost/SHAP.

MRS-ACD = (Citrate × 0.072398) + (Albumin × −0.149894) + (Cholesteryl esters to total lipids in IDL percentage × −0.005662) + (Triglycerides to total lipids in medium LDL percentage × 0.091278) + (Glucose × 0.014653) + (3-Hydroxybutyrate × 0.127783) + (Phospholipids to total lipids in small LDL percentage × 0.100530) + (Docosahexaenoic acid to total fatty acids percentage × 0.108484) + (Average diameter for LDL particles × −0.066230) + (Phospholipids in medium HDL × −0.042127)

MRS-AD = (Free cholesterol to total lipids in chylomicrons and extremely large VLDL percentage × 0.028049) + (Triglycerides in large VLDL × −0.020590) + (Albumin × −0.165543) + (Citrate × 0.225040) + (Cholesterol to total lipids in small LDL percentage × −0.005848) + (Glucose lactate × 0.099389) + (Acetone × 0.033401) + (Triglycerides to total lipids in large HDL percentage × 0.091801) + (Glycine × 0.061401) + (Phospholipids to total lipids in chylomicrons and extremely large VLDL percentage × 0.064358)

MRS-VaD = (Phospholipids in small HDL × −0.086485) + (Omega-6 fatty acids to omega-3 fatty acids ratio × −0.026405) + (Citrate × 0.015578) + (Albumin × −0.070220) + (Creatinine × 0.108974) + (3-Hydroxybutyrate × 0.123566) + (Linoleic acid × −0.003601) + (Average diameter for LDL particles × −0.164647) + (Phospholipids to total lipids in very small VLDL percentage × 0.175760) + (Glycoprotein acetyls × 0.149535)

References

1. Li J, Man Q, Wang Y, et al. The metabolic vulnerability index as a novel tool for mortality risk stratification in a large-scale population-based cohort. Redox Biol. 2025;81:103585. doi:10.1016/j.redox.2025.103585
2. Wang Y, Xu Q, Lu S, et al. The effect of muscle quality index on risk of adverse health outcomes: a prospective cohort study of 355,209 adults. Am J Clin Nutr. 2025;122(5):1452-1460. doi:10.1016/j.ajcnut.2025.09.00
3. Tian F, Wang Y, Qian ZM, et al. Plasma metabolomic signature of healthy lifestyle, structural brain reserve and risk of dementia. Brain. 2025;148(1):143-153. doi:10.1093/brain/awae257

| **Table S1. Missing data across all baseline covariates** | | | |
| --- | --- | --- | --- |
| **Variable** | **Non-missing, n** | **Missing, n** | **Missing rate, %** |
| Age | 7047 | 0 | 0.0 |
| TDI | 7040 | 7 | 0.1 |
| BMI | 7040 | 7 | 0.1 |
| ALB | 6011 | 1036 | 14.7 |
| HbA1c | 6518 | 529 | 7.5 |
| Gender | 7047 | 0 | 0.0 |
| Race | 7047 | 0 | 0.0 |
| Education level | 7006 | 41 | 0.6 |
| Smoking status | 6984 | 63 | 0.9 |
| Alcohol consumption status | 7022 | 25 | 0.4 |
| Dietary habits | 7009 | 38 | 0.5 |
| Physical exercise intensity | 6399 | 648 | 9.2 |
| Sleep duration | 6952 | 95 | 1.3 |
| Loneliness | 6892 | 155 | 2.2 |
| Hypertension | 7047 | 0 | 0.0 |
| Diabetes | 7047 | 0 | 0.0 |
| CKD | 7047 | 0 | 0.0 |
| Cholesterol-lowering medication | 6984 | 63 | 0.9 |
| Antihypertensive drugs | 6984 | 63 | 0.9 |
| Insulin | 6984 | 63 | 0.9 |
| Aspirin | 6984 | 63 | 0.9 |
| Stroke duration | 7047 | 0 | 0.0 |
| Fasting time | 7047 | 0 | 0.0 |
| Scr | 6554 | 493 | 7.0 |
| CYS-C | 6561 | 486 | 6.9 |
| eGFR | 6547 | 500 | 7.1 |
| CRP | 6533 | 514 | 7.3 |
| LDL-C | 6547 | 500 | 7.1 |
| GLU | 6004 | 1043 | 14.8 |
| Arm fat-free mass | 7047 | 0 | 0.0 |
| Hand grip strength | 7033 | 14 | 0.2 |
| Data are presented as n or %. Missing rate was calculated as missing n divided by the total sample size (n = 7047). All 31 covariates are shown, including those with no missing data. The mean missingness across all covariates was 3.0%. | | | |

| **Table S2 Variance inflation factors for covariates in Cox proportional hazards models** | | | | | | | | | | | |
| --- | --- | --- | --- | --- | --- | --- | --- | --- | --- | --- | --- |
| **Variable** | **GVIF** | | |  | **Df** | | |  | **GVIF^(1/2Df) range** | | |
|  | **ACD AD VaD** | | |  | **ACD AD VaD** | | |  | **ACD AD VaD** | | |
| Age | 1.07 | 1.04 | 1.06 |  | 1 | 1 | 1 |  | 1.03 | 1.02 | 1.03 |
| Sex | 1.08 | 1.10 | 1.07 |  | 1 | 1 | 1 |  | 1.04 | 1.05 | 1.03 |
| Race | 1.08 | 1.12 | 1.08 |  | 1 | 1 | 1 |  | 1.04 | 1.06 | 1.04 |
| TDI | 1.15 | 1.14 | 1.15 |  | 1 | 1 | 1 |  | 1.07 | 1.07 | 1.07 |
| Education level | 1.04 | 1.04 | 1.04 |  | 1 | 1 | 1 |  | 1.02 | 1.01 | 1.02 |
| Smoking status | 1.22 | 1.20 | 1.18 |  | 2 | 2 | 2 |  | 1.05 | 1.05 | 1.04 |
| Alcohol consumption | 1.13 | 1.14 | 1.11 |  | 2 | 2 | 2 |  | 1.03 | 1.03 | 1.03 |
| Dietary habits | 1.03 | 1.03 | 1.03 |  | 1 | 1 | 1 |  | 1.02 | 1.02 | 1.01 |
| Physical exercise | 1.06 | 1.06 | 1.07 |  | 2 | 2 | 2 |  | 1.01 | 1.01 | 1.02 |
| Sleep duration | 1.06 | 1.06 | 1.06 |  | 2 | 2 | 2 |  | 1.01 | 1.02 | 1.02 |
| Loneliness | 1.09 | 1.09 | 1.09 |  | 1 | 1 | 1 |  | 1.04 | 1.04 | 1.04 |
| BMI | 1.20 | 1.22 | 1.20 |  | 1 | 1 | 1 |  | 1.10 | 1.10 | 1.09 |
| Stroke duration | 1.01 | 1.01 | 1.01 |  | 1 | 1 | 1 |  | 1.00 | 1.00 | 1.00 |
| MQI | 1.23 | 1.25 | 1.23 |  | 3 | 3 | 3 |  | 1.04 | 1.04 | 1.04 |
| All adjusted GVIF values were below the conventional threshold of 5.0, indicating no severe multicollinearity. Abbreviations: GVIF: generalized variance inflation factor; GVIF^(1/2Df): adjusted generalized variance inflation factor; ACD: All-cause dementia; AD: Alzheimer's disease; VaD: Vascular dementia; TDI: Townsend deprivation index; BMI: Body mass index; MQI: Muscle quality index. | | | | | | | | | | | |

| **Table S3 Schoenfeld residual tests for the proportional hazards assumption** | | | | |
| --- | --- | --- | --- | --- |
| **Variables** | **Variable** | **χ²** | **df** | ***P value*** |
| **ACD** |  |  |  |  |
| Schoenfeld test | Age | 1.128 | 1 | 0.288 |
|  | Gender | 0.048 | 1 | 0.827 |
|  | Race | 4.148 | 1 | **0.042** |
|  | TDI | 1.857 | 1 | 0.173 |
|  | Education level | 0.414 | 1 | 0.520 |
|  | Smoking status | 1.509 | 2 | 0.470 |
|  | Alcohol consumption status | 1.312 | 2 | 0.519 |
|  | Dietary habits | 1.176 | 1 | 0.278 |
|  | Physical exercise intensity | 1.672 | 2 | 0.433 |
|  | Sleep duration | 0.265 | 2 | 0.876 |
|  | Loneliness | 4.001 | 1 | **0.045** |
|  | BMI | 0.509 | 1 | 0.475 |
|  | Stroke duration | 2.138 | 1 | 0.144 |
|  | MQI | 3.753 | 3 | 0.289 |
|  | Global | 20.335 | 20 | 0.437 |
| **AD** |  |  |  |  |
| Schoenfeld test | Age | 0.017 | 1 | 0.897 |
|  | Gender | 0.020 | 1 | 0.888 |
|  | Race | 1.066 | 1 | 0.302 |
|  | TDI | 1.119 | 1 | 0.290 |
|  | Education | 0.346 | 1 | 0.556 |
|  | Smoking status | 1.946 | 2 | 0.378 |
|  | Alcohol consumption status | 2.227 | 2 | 0.328 |
|  | Dietary habits | 1.227 | 1 | 0.268 |
|  | Physical exercise intensity | 0.496 | 2 | 0.780 |
|  | Sleep duration | 0.330 | 2 | 0.848 |
|  | Loneliness | 2.612 | 1 | 0.106 |
|  | BMI | 0.834 | 1 | 0.361 |
|  | Stroke duration | 0.012 | 1 | 0.914 |
|  | MQI | 1.128 | 3 | 0.770 |
|  | Global | 14.491 | 20 | 0.805 |
| **VaD** |  |  |  |  |
| Schoenfeld test | Age | 0.076 | 1 | 0.783 |
|  | Gender | 0.441 | 1 | 0.507 |
|  | Race | 0.532 | 1 | 0.466 |
|  | TDI | 4.350 | 1 | **0.037** |
|  | Education | 1.773 | 1 | 0.183 |
|  | Smoking status | 2.028 | 2 | 0.363 |
|  | Alcohol consumption status | 2.226 | 2 | 0.329 |
|  | Dietary habits | 0.544 | 1 | 0.461 |
|  | Physical exercise intensity | 0.018 | 2 | 0.991 |
|  | Sleep duration | 1.274 | 2 | 0.529 |
|  | Loneliness | 0.095 | 1 | 0.759 |
|  | BMI | 0.121 | 1 | 0.728 |
|  | Stroke duration | 2.406 | 1 | 0.121 |
|  | MQI | 1.173 | 3 | 0.759 |
|  | Global | 15.284 | 20 | 0.760 |
| Schoenfeld residual tests were conducted to examine the proportional hazards assumption for Cox regression. A significant *P* value indicates violation of the proportional hazards assumption for the corresponding covariate. Bold *P* values denote statistical significance (*P* < 0.05). The global test assesses the overall proportional hazards assumption for the entire model. All global tests were non-significant, supporting the validity of the Cox model specification. Abbreviations: ACD: All-cause dementia; AD: Alzheimer's disease; VaD: Vascular dementia; TDI: Townsend deprivation index; BMI: Body mass index; MQI: Muscle quality index. | | | | |

| **Table S4 Threshold effect analysis of MQI on AD** | | |
| --- | --- | --- |
| **Variables** | **Adjusted HR (95% CI)** | ***P value*** |
| Model 1 Standard linear regression | 0.88 (0.83–0.94) | < 0.001 |
| Model 2 Two-piecewise linear regression |  |  |
| Inflection point | 6.732 |  |
| MQI < 6.732 | 0.85 (0.72–1.00) | 0.053 |
| MQI ≥ 6.732 | 0.90 (0.83–0.97) | 0.006 |
| Log-likelihood ratio test |  | 0.007 |
| Abbreviations: MQI: Muscle quality index; AD: Alzheimer's disease; HR: Hazard Ratio; CI: Confidence Interval | | |

| **Table S5. Metabolic features selected by LASSO regression for all-cause dementia (ACD), ranked by absolute coefficient.** | | | |
| --- | --- | --- | --- |
| **Rank** | **Metabolic feature** | **Coefficient (beta)** | **Direction** |
| 1 | Albumin | -0.149894 | Negative |
| 2 | 3-Hydroxybutyrate | +0.127783 | Positive |
| 3 | Glucose lactate | +0.122864 | Positive |
| 4 | Docosahexaenoic Acid to Total Fatty Acids percentage | +0.108484 | Positive |
| 5 | Phospholipids to Total Lipids in Chylomicrons and Extremely Large VLDL percentage | +0.106874 | Positive |
| 6 | Phospholipids to Total Lipids in Small LDL percentage | +0.100530 | Positive |
| 7 | Leucine | -0.096765 | Negative |
| 8 | Glutamine | +0.093910 | Positive |
| 9 | Triglycerides to Total Lipids in Medium LDL percentage | +0.091278 | Positive |
| 10 | Phospholipids to Total Lipids in IDL percentage | +0.086108 | Positive |
| 11 | Creatinine | +0.077346 | Positive |
| 12 | Citrate | +0.072398 | Positive |
| 13 | Average Diameter for LDL Particles | -0.066230 | Negative |
| 14 | Tyrosine | +0.059089 | Positive |
| 15 | Apolipoprotein B | +0.053385 | Positive |
| 16 | Free Cholesterol to Total Lipids in Large VLDL percentage | +0.044187 | Positive |
| 17 | Triglycerides to Total Lipids in Chylomicrons and Extremely Large VLDL percentage | +0.042843 | Positive |
| 18 | Phospholipids in Medium HDL | -0.042127 | Negative |
| 19 | Cholesterol to Total Lipids in Very Large HDL percentage | -0.040698 | Negative |
| 20 | Glycoprotein Acetyls | +0.031654 | Positive |
| 21 | Acetoacetate | +0.028051 | Positive |
| 22 | Alanine | +0.027761 | Positive |
| 23 | Free Cholesterol to Total Lipids in Small HDL percentage | +0.024096 | Positive |
| 24 | Acetate | +0.022988 | Positive |
| 25 | Phenylalanine | +0.021456 | Positive |
| 26 | Cholesteryl Esters to Total Lipids in Small LDL percentage | -0.016352 | Negative |
| 27 | Glucose | +0.014653 | Positive |
| 28 | Phospholipids to Total Lipids in Medium LDL percentage | -0.014223 | Negative |
| 29 | Saturated Fatty Acids to Total Fatty Acids percentage | +0.012785 | Positive |
| 30 | Glycine | +0.006738 | Positive |
| 31 | Cholesteryl Esters to Total Lipids in IDL percentage | -0.005662 | Negative |
| 32 | Linoleic Acid to Total Fatty Acids percentage | -0.004580 | Negative |
| 33 | Pyruvate | +0.002968 | Positive |
| Features are ranked in descending order of the absolute value of the LASSO regression coefficient. Positive coefficients indicate a positive association with disease risk; negative coefficients indicate an inverse association. | | | |

| **Table S6. Metabolic features selected by LASSO regression for Alzheimer's disease (AD), ranked by absolute coefficient** | | | |
| --- | --- | --- | --- |
| **Rank** | **Metabolic feature** | **Coefficient (beta)** | **Direction** |
| 1 | Cholesteryl Esters in Very Large VLDL | -0.293723 | Negative |
| 2 | Citrate | +0.225040 | Positive |
| 3 | Average Diameter for VLDL Particles | -0.221519 | Negative |
| 4 | Triglycerides to Total Lipids in Chylomicrons and Extremely Large VLDL percentage | +0.211299 | Positive |
| 5 | Albumin | -0.165543 | Negative |
| 6 | Creatinine | +0.133803 | Positive |
| 7 | Apolipoprotein B | +0.130599 | Positive |
| 8 | Triglycerides in Large HDL | +0.105316 | Positive |
| 9 | Phospholipids to Total Lipids in Very Large HDL percentage | -0.099912 | Negative |
| 10 | Glucose lactate | +0.099389 | Positive |
| 11 | Triglycerides to Total Lipids in Large HDL percentage | +0.091801 | Positive |
| 12 | Phospholipids to Total Lipids in Medium LDL percentage | -0.091076 | Negative |
| 13 | Cholesteryl Esters to Total Lipids in IDL percentage | -0.086137 | Negative |
| 14 | Tyrosine | +0.085770 | Positive |
| 15 | Concentration of Chylomicrons and Extremely Large VLDL Particles | -0.072805 | Negative |
| 16 | Leucine | -0.069217 | Negative |
| 17 | 3-Hydroxybutyrate | +0.068766 | Positive |
| 18 | Phospholipids to Total Lipids in Chylomicrons and Extremely Large VLDL percentage | +0.064358 | Positive |
| 19 | Glycine | +0.061401 | Positive |
| 20 | Cholesteryl Esters in Small HDL | -0.053307 | Negative |
| 21 | Phospholipids in Medium LDL | -0.047246 | Negative |
| 22 | Cholesteryl Esters to Total Lipids in Small LDL percentage | -0.044682 | Negative |
| 23 | Cholesteryl Esters in Small LDL | -0.035009 | Negative |
| 24 | Acetone | +0.033401 | Positive |
| 25 | Acetate | -0.032079 | Negative |
| 26 | Free Cholesterol to Total Lipids in Chylomicrons and Extremely Large VLDL percentage | +0.028049 | Positive |
| 27 | Cholesteryl Esters to Total Lipids in Large VLDL percentage | +0.024373 | Positive |
| 28 | Triglycerides in Large VLDL | -0.020590 | Negative |
| 29 | Free Cholesterol to Total Lipids in Medium VLDL percentage | +0.018592 | Positive |
| 30 | Docosahexaenoic Acid | +0.013848 | Positive |
| 31 | Docosahexaenoic Acid to Total Fatty Acids percentage | +0.012967 | Positive |
| 32 | Cholesterol to Total Lipids in Small LDL percentage | -0.005848 | Negative |
| 33 | Phospholipids to Total Lipids in IDL percentage | +0.004963 | Positive |
| Features are ranked in descending order of the absolute value of the LASSO regression coefficient. Positive coefficients indicate a positive association with disease risk; negative coefficients indicate an inverse association. | | | |

| **Table S7. Metabolic features selected by LASSO regression for vascular dementia (VaD), ranked by absolute coefficient.** | | | |
| --- | --- | --- | --- |
| **Rank** | **Metabolic feature** | **Coefficient (beta)** | **Direction** |
| 1 | Docosahexaenoic Acid to Total Fatty Acids percentage | +0.179082 | Positive |
| 2 | Phospholipids to Total Lipids in Very Small VLDL percentage | +0.175760 | Positive |
| 3 | Average Diameter for LDL Particles | -0.164647 | Negative |
| 4 | Glycoprotein Acetyls | +0.149535 | Positive |
| 5 | 3-Hydroxybutyrate | +0.123566 | Positive |
| 6 | Creatinine | +0.108974 | Positive |
| 7 | Phospholipids to Total Lipids in Large HDL percentage | -0.089873 | Negative |
| 8 | Concentration of Small HDL Particles | -0.088608 | Negative |
| 9 | Glutamine | +0.087200 | Positive |
| 10 | Phospholipids in Small HDL | -0.086485 | Negative |
| 11 | Pyruvate | +0.076955 | Positive |
| 12 | Glucose | +0.073826 | Positive |
| 13 | Albumin | -0.070220 | Negative |
| 14 | Glucose lactate | +0.046533 | Positive |
| 15 | Acetoacetate | +0.026902 | Positive |
| 16 | Omega 6 Fatty Acids to Omega 3 Fatty Acids ratio | -0.026405 | Negative |
| 17 | Citrate | +0.015578 | Positive |
| 18 | Leucine | -0.012943 | Negative |
| 19 | Free Cholesterol to Total Lipids in Large HDL percentage | +0.007937 | Positive |
| 20 | Phospholipids to Total Lipids in Small LDL percentage | +0.005622 | Positive |
| 21 | Linoleic Acid | -0.003601 | Negative |
| 22 | Alanine | +0.000825 | Positive |
| Features are ranked in descending order of the absolute value of the LASSO regression coefficient. Positive coefficients indicate a positive association with disease risk; negative coefficients indicate an inverse association. | | | |

| **Table S8. Associations of MRS with risk of post-stroke dementia in the validation cohort** | | | | | |
| --- | --- | --- | --- | --- | --- |
| **Variables** | **MRS** | **Cases/Total n (%)** | **HR (95% CI)** | ***P value*** | ***P for trend*** |
| **ACD** |  |  |  |  |  |
|  | Per 1 SD | 127/2041 (6.22) | 1.63 (1.36–1.96) | <0.001 |  |
|  | T1 | 28/680 (4.12) | Ref |  | <0.001 |
|  | T2 | 30/680 (4.41) | 0.98 (0.58–1.64) | 0.929 |  |
|  | T3 | 69/681 (10.13) | 2.34 (1.48–3.70) | <0.001 |  |
| **AD** |  |  |  |  |  |
|  | Per 1 SD | 34/2041 (1.67) | 2.40 (1.74–3.31) | <0.001 |  |
|  | T1 | 5/680 (0.74) | Ref |  | <0.001 |
|  | T2 | 10/680 (1.47) | 1.92 (0.65–5.65) | 0.239 |  |
|  | T3 | 19/681 (2.79) | 3.81 (1.40–10.38) | 0.009 |  |
| **VaD** |  |  |  |  |  |
|  | Per 1 SD | 55/2041 (2.69) | 1.92 (1.46–2.54) | <0.001 |  |
|  | T1 | 7/680 (1.03) | Ref |  | <0.001 |
|  | T2 | 13/680 (1.91) | 1.35 (0.53–3.44) | 0.533 |  |
|  | T3 | 35/681 (5.14) | 3.79 (1.60–8.98) | 0.002 |  |
| Model was adjusted for age, gender, race, TDI, education level, smoking status, alcohol consumption status, dietary habits, sleep duration, physical exercise intensity, BMI, loneliness, stroke duration, and fasting time. MRS was categorized into tertiles (T1–T3) based on the distribution in validation cohort. Abbreviations: HR: Hazard Ratio; CI: Confidence Interval; SD: Standard deviation; Ref: Reference; MRS: Metabolomics risk score; ACD: All-cause dementia; AD: Alzheimer's disease; VaD: Vascular dementia; TDI: Townsend deprivation index; BMI: Body mass index. | | | | | |

| **Table S9. C-index of different models for predicting post-stroke dementia** | | | |
| --- | --- | --- | --- |
| **Outcome** | **Model** | **C-index** | **95% CI** |
| ACD | Base | 0.718 | 0.692–0.769 |
|  | Base + MQI | 0.724 | 0.701–0.773 |
|  | Base + MRS | 0.752 | 0.730–0.791 |
|  | Base + MQI + MRS | 0.756 | 0.735–0.799 |
| AD | Base | 0.786 | 0.745–0.858 |
|  | Base + MQI | 0.803 | 0.773–0.871 |
|  | Base + MRS | 0.836 | 0.793–0.897 |
|  | Base + MQI + MRS | 0.850 | 0.818–0.910 |
| VaD | Base | 0.771 | 0.737–0.836 |
|  | Base + MQI | 0.778 | 0.756–0.846 |
|  | Base + MRS | 0.810 | 0.775–0.869 |
|  | Base + MQI + MRS | 0.813 | 0.787–0.874 |
| Model was adjusted for age, gender, race, TDI, education level, smoking status, alcohol consumption status, dietary habits, sleep duration, physical exercise intensity, BMI, loneliness, stroke duration, diabetes, and hypertension. Abbreviations: ACD: All-cause dementia; AD: Alzheimer's disease; VaD: Vascular dementia; MQI: Muscle quality index; MRS: Metabolomics risk score; CI: Confidence interval; TDI: Townsend deprivation index; BMI: Body mass index. | | | |

| **Table S10. Time-dependent AUCs of prediction models for post-stroke dementia** | | | |
| --- | --- | --- | --- |
| **Outcome** | **Time, years** | **Model** | **AUC (95% CI)** |
| ACD | 10 | Base | 0.750 (0.703–0.813) |
|  |  | Base + MQI | 0.754 (0.710–0.814) |
|  |  | Base + MRS | 0.767 (0.715–0.827) |
|  |  | Base + MQI + MRS | 0.772 (0.721–0.831) |
|  | 15 | Base | 0.744 (0.711–0.794) |
|  |  | Base + MQI | 0.752 (0.721–0.800) |
|  |  | Base + MRS | 0.776 (0.751–0.815) |
|  |  | Base + MQI + MRS | 0.782 (0.756–0.826) |
| AD | 10 | Base | 0.807 (0.711–0.910) |
|  |  | Base + MQI | 0.819 (0.754–0.909) |
|  |  | Base + MRS | 0.850 (0.755–0.940) |
|  |  | Base + MQI + MRS | 0.861 (0.784–0.949) |
|  | 15 | Base | 0.797 (0.761–0.863) |
|  |  | Base + MQI | 0.817 (0.780–0.880) |
|  |  | Base + MRS | 0.848 (0.805–0.907) |
|  |  | Base + MQI + MRS | 0.863 (0.827–0.921) |
| VaD | 10 | Base | 0.814 (0.762–0.894) |
|  |  | Base + MQI | 0.825 (0.775–0.908) |
|  |  | Base + MRS | 0.839 (0.779–0.915) |
|  |  | Base + MQI + MRS | 0.844 (0.790–0.920) |
|  | 15 | Base | 0.798 (0.763–0.853) |
|  |  | Base + MQI | 0.806 (0.778–0.864) |
|  |  | Base + MRS | 0.837 (0.797–0.889) |
|  |  | Base + MQI + MRS | 0.841 (0.808–0.896) |
| Values are AUC (95% CI). Model was adjusted for age, gender, race, TDI, education level, smoking status, alcohol consumption status, dietary habits, sleep duration, physical exercise intensity, BMI, loneliness, stroke duration, diabetes, and hypertension. Abbreviations: AUC: Area under the receiver operating characteristic curve; CI: Confidence interval; ACD: All-cause dementia; AD: Alzheimer's disease; VaD: Vascular dementia; MQI: Muscle quality index; MRS: Metabolomics risk score; TDI: Townsend deprivation index; BMI: Body mass index. | | | |

| **Table S11. Differences in time-dependent AUCs between augmented models and the base model: DeLong’s test** | | | | |
| --- | --- | --- | --- | --- |
| **Outcome** | **Time, years** | **Comparison** | **ΔAUC (95% CI)** | ***P value*** |
| ACD | 10 | Base + MQI | 0.004 (–0.006–0.023) | 0.534 |
|  |  | Base + MRS | 0.017 (–0.004–0.047) | 0.247 |
|  |  | Base + MQI + MRS | 0.021 (0–0.053) | 0.147 |
|  | 15 | Base + MQI | 0.008 (0–0.024) | 0.145 |
|  |  | Base + MRS | 0.032 (0.012–0.052) | 0.003 |
|  |  | Base + MQI + MRS | 0.038 (0.018–0.059) | <0.001 |
| AD | 10 | Base + MQI | 0.012 (–0.020–0.054) | 0.467 |
|  |  | Base + MRS | 0.043 (–0.003–0.092) | 0.196 |
|  |  | Base + MQI + MRS | 0.054 (–0.001–0.113) | 0.130 |
|  | 15 | Base + MQI | 0.020 (–0.004–0.049) | 0.125 |
|  |  | Base + MRS | 0.051 (0.019–0.081) | 0.013 |
|  |  | Base + MQI + MRS | 0.066 (0.033–0.100) | 0.001 |
| VaD | 10 | Base + MQI | 0.010 (–0.003–0.039) | 0.306 |
|  |  | Base + MRS | 0.024 (–0.007–0.064) | 0.331 |
|  |  | Base + MQI + MRS | 0.030 (–0.002–0.074) | 0.240 |
|  | 15 | Base + MQI | 0.009 (–0.002–0.028) | 0.284 |
|  |  | Base + MRS | 0.040 (0.014–0.060) | 0.015 |
|  |  | Base + MQI + MRS | 0.043 (0.018–0.069) | 0.008 |
| Values are ΔAUC (95% CI) relative to the Base model; *P* values were calculated using DeLong's test. Model was adjusted for age, gender, race, TDI, education level, smoking status, alcohol consumption status, dietary habits, sleep duration, physical exercise intensity, BMI, loneliness, stroke duration, diabetes, and hypertension. AUC: Area under the receiver operating characteristic curve; CI: Confidence interval; ACD: All-cause dementia; AD: Alzheimer's disease; VaD: Vascular dementia; MQI: Muscle quality index; MRS: Metabolomics risk score; TDI: Townsend deprivation index; BMI: Body mass index. | | | | |

| **Table S12. Reclassification and discrimination improvement of augmented models relative to the base model** | | | | |
| --- | --- | --- | --- | --- |
| **Outcome** | **Time, years** | **Comparison** | **NRI (95% CI)** | **IDI (95% CI)** |
| ACD | 10 | Base + MQI | 0.099 (–0.067–0.406) | 0.001 (–0.002–0.008) |
|  |  | Base + MRS | 0.230 (0.033–0.447) | 0.010 (–0.002–0.034) |
|  |  | Base + MQI + MRS | 0.199 (0.061–0.482) | 0.009 (–0.001–0.033) |
|  | 15 | Base + MQI | 0.209 (0.039–0.432) | 0.004 (0.001–0.015) |
|  |  | Base + MRS | 0.333 (0.129–0.501) | 0.021 (0.007–0.046) |
|  |  | Base + MQI + MRS | 0.335 (0.210–0.583) | 0.023 (0.009–0.052) |
| AD | 10 | Base + MQI | 0.251 (–0.183–0.854) | 0.003 (–0.006–0.041) |
|  |  | Base + MRS | 0.542 (0.102–1.045) | 0.010 (–0.010–0.051) |
|  |  | Base + MQI + MRS | 0.747 (0.190–1.174) | 0.015 (–0.004–0.075) |
|  | 15 | Base + MQI | 0.404 (0.102–0.746) | 0.007 (0.000–0.044) |
|  |  | Base + MRS | 0.681 (0.279–0.903) | 0.045 (0.011–0.107) |
|  |  | Base + MQI + MRS | 0.788 (0.501–1.050) | 0.064 (0.025–0.132) |
| VaD | 10 | Base + MQI | 0.484 (0.069–0.724) | 0.003 (0.000–0.022) |
|  |  | Base + MRS | 0.361 (0.058–0.793) | 0.014 (–0.003–0.059) |
|  |  | Base + MQI + MRS | 0.462 (0.195–0.913) | 0.014 (0.003–0.060) |
|  | 15 | Base + MQI | 0.398 (0.086–0.605) | 0.005 (0.001–0.029) |
|  |  | Base + MRS | 0.579 (0.305–0.778) | 0.031 (0.009–0.094) |
|  |  | Base + MQI + MRS | 0.648 (0.349–0.840) | 0.032 (0.014–0.099) |
| Values are NRI or IDI (95% CI) relative to the Base model. Positive values indicate improvement relative to the Base model. Model was adjusted for age, gender, race, TDI, education level, smoking status, alcohol consumption status, dietary habits, sleep duration, physical exercise intensity, BMI, loneliness, stroke duration, diabetes, and hypertension. CI: Confidence interval; ACD: All-cause dementia; AD: Alzheimer's disease; VaD: Vascular dementia; MQI: Muscle quality index; MRS: Metabolomics risk score; TDI: Townsend deprivation index; BMI: Body mass index. NRI: net reclassification improvement; IDI: integrated discrimination improvement | | | | |

| **Table S13. Brier scores of prediction models for post-stroke dementia** | | | |
| --- | --- | --- | --- |
| **Outcome** | **Time, years** | **Model** | **Brier score (95% CI)** |
| **ACD** | 10 | Base | 0.0331 (0.0265–0.0382) |
|  |  | Base + MQI | 0.0331 (0.0264–0.0382) |
|  |  | Base + MRS | 0.0328 (0.0264–0.0378) |
|  |  | Base + MQI + MRS | 0.0329 (0.0263–0.0378) |
|  | 15 | Base | 0.0630 (0.0539–0.0690) |
|  |  | Base + MQI | 0.0626 (0.0532–0.0688) |
|  |  | Base + MRS | 0.0615 (0.0526–0.0676) |
|  |  | Base + MQI + MRS | 0.0612 (0.0521–0.0673) |
| **AD** | 10 | Base | 0.0088 (0.0052–0.0121) |
|  |  | Base + MQI | 0.0088 (0.0052–0.0121) |
|  |  | Base + MRS | 0.0088 (0.0052–0.0122) |
|  |  | Base + MQI + MRS | 0.0088 (0.0051–0.0120) |
|  | 15 | Base | 0.0210 (0.0146–0.0262) |
|  |  | Base + MQI | 0.0209 (0.0145–0.0263) |
|  |  | Base + MRS | 0.0197 (0.0134–0.0247) |
|  |  | Base + MQI + MRS | 0.0193 (0.0130–0.0247) |
| **VaD** | 10 | Base | 0.0146 (0.0103–0.0188) |
|  |  | Base + MQI | 0.0145 (0.0101–0.0186) |
|  |  | Base + MRS | 0.0144 (0.0099–0.0185) |
|  |  | Base + MQI + MRS | 0.0144 (0.0097–0.0185) |
|  | 15 | Base | 0.0297 (0.0229–0.0347) |
|  |  | Base + MQI | 0.0294 (0.0225–0.0345) |
|  |  | Base + MRS | 0.0283 (0.0216–0.0334) |
|  |  | Base + MQI + MRS | 0.0283 (0.0216–0.0333) |
| Values are Brier score (95% CI). Lower Brier scores indicate better model calibration. Model was adjusted for age, gender, race, TDI, education level, smoking status, alcohol consumption status, dietary habits, sleep duration, physical exercise intensity, BMI, loneliness, stroke duration, diabetes, and hypertension. CI: Confidence interval; ACD: All-cause dementia; AD: Alzheimer's disease; VaD: Vascular dementia; MQI: Muscle quality index; MRS: Metabolomics risk score; TDI: Townsend deprivation index; BMI: Body mass index. | | | |

| **Table S14 Joint associations of MRS and MQI with post-stroke dementia** | | | | | |
| --- | --- | --- | --- | --- | --- |
| **Variables** | **MRS Stratification** | **MQI** | **Cases/Total, n (%)** | **HR (95% CI)** | ***P value*** |
| **ACD** |  |  |  |  |  |
|  | Low risk | Q4 | 16/674 (2.37) | Ref |  |
|  |  | Q3 | 21/590 (3.56) | 1.18 (0.62–2.27) | 0.615 |
|  |  | Q2 | 21/546 (3.85) | 1.43 (0.74–2.74) | 0.285 |
|  |  | Q1 | 30/457 (6.56) | 2.20 (1.19–4.04) | 0.012 |
|  | Intermediate risk | Q4 | 40/573 (6.98) | 2.22 (1.24–3.96) | 0.007 |
|  |  | Q3 | 26/595 (4.37) | 1.35 (0.72–2.51) | 0.352 |
|  |  | Q2 | 37/568 (6.51) | 1.82 (1.01–3.28) | 0.047 |
|  |  | Q1 | 48/531 (9.04) | 2.51 (1.42–4.45) | 0.002 |
|  | High risk | Q4 | 34/468 (7.26) | 2.24 (1.23–4.07) | 0.008 |
|  |  | Q3 | 41/508 (8.07) | 2.37 (1.33–4.24) | 0.004 |
|  |  | Q2 | 53/584 (9.08) | 2.44 (1.38–4.29) | 0.002 |
|  |  | Q1 | 80/709 (11.28) | 3.29 (1.91–5.68) | <0.001 |
| **AD** |  |  |  |  |  |
|  | Low risk | Q4 | 3/590 (0.51) | Ref |  |
|  |  | Q3 | 2/598 (0.33) | 0.57 (0.09–3.39) | 0.532 |
|  |  | Q2 | 2/552 (0.36) | 0.65 (0.11–3.88) | 0.633 |
|  |  | Q1 | 7/527 (1.33) | 1.99 (0.51–7.76) | 0.322 |
|  | Intermediate risk | Q4 | 7/564 (1.24) | 2.09 (0.54–8.09) | 0.286 |
|  |  | Q3 | 2/574 (0.35) | 0.49 (0.08–2.94) | 0.436 |
|  |  | Q2 | 4/572 (0.70) | 0.85 (0.19–3.82) | 0.834 |
|  |  | Q1 | 16/557 (2.87) | 3.86(1.12–13.34) | 0.033 |
|  | High risk | Q4 | 18/561 (3.21) | 4.55 (1.33–15.51) | 0.016 |
|  |  | Q3 | 14/521 (2.69) | 3.19 (0.91–11.18) | 0.070 |
|  |  | Q2 | 19/574 (3.31) | 3.84 (1.13–13.09) | 0.031 |
|  |  | Q1 | 28/613 (4.57) | 5.83 (1.75–19.37) | 0.004 |
| **VaD** |  |  |  |  |  |
|  | Low risk | Q4 | 10/712 (1.40) | Ref |  |
|  |  | Q3 | 6/583 (1.03) | 0.66 (0.24–1.82) | 0.424 |
|  |  | Q2 | 9/525 (1.71) | 1.14 (0.46–2.80) | 0.781 |
|  |  | Q1 | 9/447 (2.01) | 1.29 (0.52–3.18) | 0.586 |
|  | Intermediate risk | Q4 | 16/536 (2.99) | 1.93 (0.87–4.27) | 0.104 |
|  |  | Q3 | 12/577 (2.08) | 1.33 (0.57–3.09) | 0.505 |
|  |  | Q2 | 15/608 (2.47) | 1.58 (0.71–3.53) | 0.266 |
|  |  | Q1 | 13/546 (2.38) | 1.47 (0.64–3.38) | 0.364 |
|  | High risk | Q4 | 16/467 (3.43) | 2.30 (1.04–5.09) | 0.040 |
|  |  | Q3 | 19/533 (3.56) | 2.33 (1.08–5.04) | 0.032 |
|  |  | Q2 | 29/565 (5.13) | 3.21 (1.55–6.64) | 0.002 |
|  |  | Q1 | 51/704 (7.24) | 4.57 (2.28–9.14) | <0.001 |
| Model was adjusted for age, gender, race, TDI, education level, smoking status, alcohol consumption status, dietary habits, sleep duration, physical exercise intensity, BMI, loneliness, stroke duration, and fasting time. Abbreviations: HR: Hazard Ratio; CI: Confidence Interval; Ref: Reference; MRS: Metabolomics risk score; MQI: Muscle quality index; ACD: All-cause dementia; AD: Alzheimer's disease; VaD: Vascular dementia; TDI: Townsend deprivation index; BMI: Body mass index. | | | | | |

| **Table S15 Additive and multiplicative interactions between MRS and MQI on risk of post-stroke dementia** | |
| --- | --- |
| **Variables** | **Est 95% CI** |
| **ACD** |  |
| Main effects |  |
| MQI (For each decrease in SD) & MRS-ACD (For each increase in SD) |  |
| RERI | 0.086 (0.011–0.162) |
| Attributable proportion % | 3.3% (0.1%–6.4%) |
| Multiplicative interaction | 0.953 (0.853–1.065) |
| **AD** |  |
| Main effects |  |
| MQI (For each decrease in SD) & MRS-AD (For each increase in SD) |  |
| RERI | 0.050 (0.007–0.094) |
| Attributable proportion % | 3.9% (0.1%–7.7%) |
| Multiplicative interaction | 1.010 (0.906–1.125) |
| **VaD** |  |
| Main effects |  |
| MQI (For each decrease in SD) & MRS-VaD ( For each increase in SD) |  |
| RERI | 0.094 (0.028–0.161) |
| Attributable proportion % | 6.8% (1.1%–12.6%) |
| Multiplicative interaction | 1.057 (0.970–1.152) |
| Model was adjusted for age, gender, race, TDI, education level, smoking status, alcohol consumption status, dietary habits, sleep duration, physical exercise intensity, BMI, loneliness, stroke duration, and fasting time. Abbreviations: Est 95%: Estimate 95%; CI: Confidence Interval; MQI: Muscle quality index; MRS: Metabolomics risk score; ACD: All-cause dementia; AD: Alzheimer's disease; VaD: Vascular dementia; TDI: Townsend deprivation index; BMI: Body mass index; RERI: relative excess risk due to interaction; SD: Standard deviation. | |

| **Table S20 Sensitivity analysis of MQI for post-stroke dementia** | | | | | |
| --- | --- | --- | --- | --- | --- |
| **Variables** | **MQI** | **Cases/Total n (%)** | **HR (95% CI)** | ***P value*** | ***P for trend*** |
| ACD |  |  |  |  |  |
|  | Per 1 SD increase | 468/7047 (6.64) | 0.76 (0.69–0.85) | <0.001 |  |
|  | Q1 | 166/1761 (9.43) | Ref |  | <0.001 |
|  | Q2 | 113/1761 (6.42) | 0.67 (0.53–0.86) | 0.001 |  |
|  | Q3 | 94/1761 (5.34) | 0.54 (0.41–0.70) | <0.001 |  |
|  | Q4 | 95/1764 (5.39) | 0.56 (0.42–0.74) | <0.001 |  |
| AD |  |  |  |  |  |
|  | Per 1 SD increase | 128/7047 (1.82) | 0.70 (0.58–0.84) | <0.001 |  |
|  | Q1 | 54/1761 (3.07) | Ref |  | 0.001 |
|  | Q2 | 25/1761 (1.42) | 0.44 (0.27–0.71) | 0.001 |  |
|  | Q3 | 19/1761 (1.08) | 0.32 (0.18–0.54) | <0.001 |  |
|  | Q4 | 30/1764 (1.70) | 0.50 (0.31–0.83) | 0.007 |  |
| VaD |  |  |  |  |  |
|  | Per 1 SD increase | 217/7047 (3.08) | 0.78 (0.67–0.91) | 0.001 |  |
|  | Q1 | 76/1761 (4.32) | Ref |  | 0.017 |
|  | Q2 | 54/1761 (3.07) | 0.73 (0.52–1.05) | 0.090 |  |
|  | Q3 | 41/1761 (2.33) | 0.55 (0.37–0.82) | 0.003 |  |
|  | Q4 | 46/1764 (2.61) | 0.66 (0.44–0.98) | 0.041 |  |
| Model adjusted for age, gender, race, TDI, education level, smoking status, alcohol consumption status, dietary habits, sleep duration, physical exercise intensity, BMI, loneliness, stroke duration, cholesterol-lowering medication, antihypertensive medication, insulin use, and aspirin use. Abbreviations: HR: Hazard Ratio; CI: Confidence Interval; SD: Standard deviation; Ref: Reference; MQI: Muscle quality index; ACD: All-cause dementia; AD: Alzheimer's disease; VaD: Vascular dementia; TDI: Townsend deprivation index; BMI: Body mass index | | | | | |

| **Table S21. The association between MQI and post-stroke dementia: Excluding new-onset dementia within 2 years** | | | | | |
| --- | --- | --- | --- | --- | --- |
| **Outcome** | **MQI** | **Events/Total (%)** | **HR (95% CI)** | ***P value*** | ***P for trend*** |
| ACD |  |  |  |  |  |
|  | Per 1-SD increase | 457/7036 (6.50) | 0.75 (0.68–0.83) | <0.001 |  |
|  | Q1 | 164/1759 (9.32) | Ref |  | <0.001 |
|  | Q2 | 108/1756 (6.15) | 0.63 (0.50–0.81) | <0.001 |  |
|  | Q3 | 90/1757 (5.12) | 0.50 (0.38–0.65) | <0.001 |  |
|  | Q4 | 95/1764 (5.39) | 0.52 (0.39–0.69) | <0.001 |  |
| AD |  |  |  |  |  |
|  | Per 1-SD increase | 127/7036 (1.81) | 0.68(0.56–0.82) | <0.001 |  |
|  | Q1 | 54/1759 (3.07) | Ref |  | <0.001 |
|  | Q2 | 24/1756 (1.37) | 0.40 (0.25–0.66) | <0.001 |  |
|  | Q3 | 19/1757 (1.08) | 0.30 (0.17–0.51) | <0.001 |  |
|  | Q4 | 30/1764 (1.70) | 0.46 (0.28–0.75) | 0.002 |  |
| VaD |  |  |  |  |  |
|  | Per 1-SD increase | 211/7036 (3.00) | 0.77 (0.67–0.90) | 0.001 |  |
|  | Q1 | 74/1759 (4.21) | Ref |  | 0.006 |
|  | Q2 | 51/1756 (2.90) | 0.70 (0.49–1.01) | 0.055 |  |
|  | Q3 | 40/1757 (2.28) | 0.53 (0.35–0.79) | 0.002 |  |
|  | Q4 | 46/1764 (2.61) | 0.62 (0.41–0.92) | 0.019 |  |
| Event data are presented as events/total (%). P for trend was calculated across MQI quartiles. Model was adjusted for age, gender, race, Townsend deprivation index, education level, smoking status, alcohol consumption status, dietary habits, sleep duration, physical exercise intensity, BMI, loneliness, and stroke duration. Abbreviations: HR: Hazard Ratio; CI: Confidence Interval; SD: Standard deviation; Ref: Reference; MQI: Muscle quality index; ACD: All-cause dementia; AD: Alzheimer's disease; VaD: Vascular dementia; TDI: Townsend deprivation index; BMI: Body mass index | | | | | |

| **Table S22 Subgroup analyses of the association between MQI and risk of post-stroke dementia** | | | | | | |
| --- | --- | --- | --- | --- | --- | --- |
| **Subgroup** | **ACD** | | **AD** | | **VaD** | |
|  | **HR (95% CI)** | ***P* for interaction** | **HR (95% CI)** | ***P* for interaction** | **HR (95% CI)** | ***P* for interaction** |
| **Age** |  | 0.646 |  | 0.921 |  | 0.466 |
| < 65 **years** | 0.77 (0.66–0.89) |  | 0.68 (0.48–0.96) |  | 0.79 (0.62–1.00) |  |
| ≥ 65 **years** | 0.77 (0.67–0.89) |  | 0.72 (0.57–0.91) |  | 0.80 (0.65–0.97) |  |
| **Gender** |  | 0.787 |  | 0.961 |  | 0.450 |
| Male | 0.79 (0.69–0.90) |  | 0.81 (0.60–1.08) |  | 0.80 (0.66–0.97) |  |
| Female | 0.80 (0.68–0.94) |  | 0.69 (0.53–0.89) |  | 0.85 (0.65–1.10) |  |
| **Education level** |  | 0.541 |  | 0.598 |  | 0.249 |
| College or above | 0.79 (0.71–0.88) |  | 0.74 (0.60–0.91) |  | 0.78 (0.66–0.92) |  |
| Others | 0.82 (0.62–1.09) |  | 0.65 (0.38–1.12) |  | 1.15 (0.74–1.79) |  |
| **Smoking status** |  | 0.481 |  | 0.788 |  | 0.415 |
| Never | 0.77 (0.65–0.90) |  | 0.68 (0.52–0.88) |  | 0.79 (0.62–1.01) |  |
| Former | 0.79 (0.68–0.93) |  | 0.84 (0.61–1.16) |  | 0.77 (0.62–0.97) |  |
| Current | 0.85 (0.66–1.11) |  | 0.50 (0.25–0.99) |  | 0.96 (0.62–1.49) |  |
| **Alcohol consumption status** |  | 0.854 |  | 0.056 |  | 0.841 |
| Never | 0.76 (0.54–1.06) |  | 1.23 (0.65–2.31) |  | 0.75 (0.41–1.39) |  |
| Former | 0.81 (0.61–1.09) |  | 0.63 (0.36–1.10) |  | 0.84 (0.57–1.24) |  |
| Current | 0.80 (0.71–0.90) |  | 0.67 (0.54–0.84) |  | 0.82 (0.69–0.98) |  |
| **Dietary habits** |  | 0.602 |  | 0.520 |  | 0.753 |
| Unhealthy | 0.73 (0.64–0.84) |  | 0.63 (0.48–0.84) |  | 0.73 (0.60–0.90) |  |
| Healthy | 0.88 (0.75–1.03) |  | 0.85 (0.64–1.12) |  | 0.93 (0.73–1.17) |  |
| **Physical exercise intensity** |  | 0.604 |  | 0.097 |  | 0.469 |
| Low | 0.71 (0.58–0.88) |  | 0.63 (0.41–0.97) |  | 0.79 (0.59–1.05) |  |
| Moderate | 0.76 (0.60–0.96) |  | 0.54 (0.37–0.81) |  | 1.00 (0.66–1.52) |  |
| High | 0.85 (0.74–0.98) |  | 0.88 (0.68–1.14) |  | 0.78 (0.64–0.95) |  |
| **Sleep duration** |  | 0.094 |  | 0.133 |  | 0.451 |
| < 7 hours | 0.74 (0.62–0.89) |  | 0.66 (0.48–0.93) |  | 0.71 (0.55–0.93) |  |
| 7-9 hours | 0.85 (0.73–0.99) |  | 0.77 (0.58–1.03) |  | 0.80 (0.64–1.01) |  |
| > 9 hours | 0.75 (0.59–0.95) |  | 0.71 (0.45–1.10) |  | 1.04 (0.74–1.47) |  |
| HRs were estimated per 1-SD increment in MQI from multivariable-adjusted Cox models; Model was adjusted for age, gender, race, education level, TDI, smoking status, alcohol consumption status, dietary habits, physical exercise intensity, sleep duration, loneliness, BMI, and stroke duration. Abbreviations: HR: Hazard Ratio; CI: Confidence Interval; SD: Standard deviation; Ref: Reference; MQI: Muscle quality index; ACD: All-cause dementia; AD: Alzheimer's disease; VaD: Vascular dementia; TDI: Townsend deprivation index; BMI: Body mass index | | | | | | |

| **Table S23. Associations between MQI and Post-stroke dementia in Fine–Gray competing risk models.** | | | |
| --- | --- | --- | --- |
| **Outcome** | **MQI** | **sHR (95% CI)** | ***P value*** |
| ACD |  |  |  |
|  | Per 1-SD increase | 0.82 (0.74–0.91) | <0.001 |
|  | Q1 | Ref |  |
|  | Q2 | 0.72 (0.56–0.92) | 0.008 |
|  | Q3 | 0.62 (0.48–0.81) | <0.001 |
|  | Q4 | 0.68 (0.51–0.90) | 0.007 |
| AD |  |  |  |
|  | Per 1-SD increase | 0.75 (0.60–0.93) | 0.010 |
|  | Q1 | Ref |  |
|  | Q2 | 0.46 (0.29–0.74) | 0.001 |
|  | Q3 | 0.37 (0.21–0.63) | <0.001 |
|  | Q4 | 0.64 (0.38–1.07) | 0.090 |
| VaD |  |  |  |
|  | Per 1-SD increase | 0.84 (0.71–0.98) | 0.027 |
|  | Q1 | Ref |  |
|  | Q2 | 0.79 (0.56–1.13) | 0.199 |
|  | Q3 | 0.65 (0.44–0.97) | 0.036 |
|  | Q4 | 0.80 (0.53–1.20) | 0.280 |
| Values are sHR (95% CI) estimated using Fine-Gray competing risk models. Continuous MQI was analyzed as a z score (per 1-SD increase); categorical MQI was analyzed by quartiles, with Q1 as the reference. Model was adjusted for age, gender, race, education level, TDI, smoking status, alcohol consumption status, dietary habits, physical exercise intensity, sleep duration, loneliness, BMI, and stroke duration. Abbreviations: MQI: Muscle quality index; sHR: Subdistribution hazard ratio; CI: Confidence interval; SD: Standard deviation; ACD: All-cause dementia; AD: Alzheimer's disease; VaD: Vascular dementia. TDI: Townsend deprivation index; BMI: Body mass index | | | |

| **Table S24. Fine–Gray competing risk analysis of the MRS with post-stroke dementia** | | | | | | |
| --- | --- | --- | --- | --- | --- | --- |
| **MRS** | **ACD** | | **AD** | | **VaD** | |
|  | **sHR (95% CI)** | ***P value*** | **sHR (95% CI)** | ***P value*** | **sHR (95% CI)** | ***P value*** |
| Per 1-SD increase | 1.49 (1.25–1.79) | <0.001 | 2.26 (1.63–3.15) | <0.001 | 1.76 (1.33–2.33) | <0.001 |
| T1 | 1.00 (Reference) |  | 1.00 (Reference) |  | 1.00 (Reference) |  |
| T2 | 0.93 (0.56–1.56) | 0.794 | 1.90 (0.66–5.50) | 0.235 | 1.42 (0.55–3.67) | 0.471 |
| T3 | 2.09 (1.31–3.35) | 0.002 | 3.50 (1.28–9.54) | 0.014 | 3.52 (1.43–8.63) | 0.006 |
| Fine-Gray subdistribution hazard models were fitted with all-cause death treated as a competing event, adjusted for age, gender, race, TDI, education level, smoking status, alcohol consumption status, dietary habits, sleep duration, physical exercise intensity, BMI, loneliness, stroke duration, and fasting time. Outcome-specific scores (MRS-ACD, MRS-AD, MRS-VaD) were analyzed as continuous variables (per 1-SD increase) and as tertiles (T1–T3). Abbreviations: sHR: Subdistribution hazard ratio; CI: Confidence interval; MRS: Metabolomics risk score; ACD: All-cause dementia; AD: Alzheimer's disease; VaD: Vascular dementia; TDI: Townsend deprivation index; BMI: Body mass index; SD: Standard deviation. | | | | | | |

| **Table S25 Competing risk analysis of the combined effects of MRS and MQI on post-stroke dementia** | | | | | | | |
| --- | --- | --- | --- | --- | --- | --- | --- |
| **MRS stratification** | **MQI** | **ACD** | | **AD** | | **VaD** | |
|  |  | **sHR (95% CI)** | ***P value*** | **sHR (95% CI)** | ***P value*** | **sHR (95% CI)** | ***P value*** |
| Low risk | Q4 | Ref |  | Ref |  | Ref |  |
|  | Q3 | 1.48 (0.77–2.83) | 0.240 | 0.64 (0.11–3.86) | 0.630 | 0.69 (0.25–1.91) | 0.480 |
|  | Q2 | 1.60 (0.83–3.06) | 0.160 | 0.69 (0.11–4.23) | 0.690 | 1.15 (0.47–2.83) | 0.760 |
|  | Q1 | 2.71 (1.48–4.97) | 0.001 | 2.58 (0.65–10.20) | 0.180 | 1.26 (0.52–3.09) | 0.610 |
| Intermediate risk | Q4 | 2.98 (1.67–5.32) | <0.001 | 2.44 (0.63–9.41) | 0.200 | 1.92 (0.88–4.23) | 0.100 |
|  | Q3 | 1.80 (0.96–3.36) | 0.065 | 0.65 (0.11–3.95) | 0.640 | 1.34 (0.58–3.10) | 0.490 |
|  | Q2 | 2.68 (1.49–4.82) | <0.001 | 1.28 (0.29–5.74) | 0.740 | 1.55 (0.70–3.44) | 0.280 |
|  | Q1 | 3.71 (2.10–6.56) | <0.001 | 5.72 (1.62–20.19) | 0.007 | 1.43 (0.63–3.28) | 0.390 |
| High risk | Q4 | 3.01 (1.66–5.46) | <0.001 | 6.19 (1.82–21.04) | 0.004 | 2.09 (0.95–4.61) | 0.067 |
|  | Q3 | 3.33 (1.86–5.96) | <0.001 | 4.90 (1.39–17.22) | 0.013 | 2.15 (1.00–4.60) | 0.049 |
|  | Q2 | 3.80 (2.16–6.68) | <0.001 | 6.22 (1.81–21.34) | 0.004 | 3.03 (1.48–6.22) | 0.002 |
|  | Q1 | 4.66 (2.70–8.02) | <0.001 | 8.70 (2.57–29.39) | <0.001 | 4.25 (2.13–8.46) | <0.001 |
| Fine-Gray subdistribution hazard models were fitted with all-cause death treated as a competing event, adjusted for age, gender, race, TDI, education level, smoking status, alcohol consumption status, dietary habits, sleep duration, physical exercise intensity, BMI, loneliness, stroke duration, and fasting time. Q1–Q4 represent quartiles of MQI (Q4 = highest). Abbreviations: sHR: Subdistribution hazard ratio; CI: Confidence interval; MRS: Metabolomics risk score; MQI: Muscle quality index; ACD: All-cause dementia; AD: Alzheimer's disease; VaD: Vascular dementia; TDI: Townsend deprivation index; BMI: Body mass index. | | | | | | | |

| **Table S26. UK Biobank field IDs and coding algorithms for covariates** | |
| --- | --- |
| **Covariate** | **UKB Field ID** |
| Age | 21022; 21003 |
| Townsend deprivation index | 189 |
| BMI | 21001 |
| Albumin | 30600 |
| HbA1c | 30750 |
| Gender | 31 |
| Ethnic background | 21000 |
| Education level | 6138 |
| Smoking status | 20116 |
| Alcohol consumption status | 20117; 1558 |
| Dietary habits | 100117 |
| Physical exercise intensity | 22032; 864/874/884/894/904/914 |
| Sleep duration | 1160 |
| Loneliness | 2020 |
| Hypertension | 6150; 4080/4079; 20002; 6153/6177 |
| Diabetes | 2443; 30750; 6153/6177; 2976 |
| CKD | 30700; 30720 |
| Cholesterol-lowering medication | 6153; 6177 |
| Antihypertensive drugs | 6153; 6177 |
| Insulin | 6153; 6177 |
| Aspirin | 6154 |
| Stroke duration | 4056; 21022/21003 |
| Fasting time | 74 |
| Serum creatinine | 30700 |
| Cystatin C | 30720 |
| eGFR | Derived: 30700 + 21022 + 31; optional 30720 |
| CRP | 30710 |
| LDL-C | 30780 |
| Glucose | 30740 |
| Arm fat-free mass | 23121(right); 23125(left) |
| Hand grip strength | 46; 47 |
| Abbreviations: BMI: Body mass index; CKD: Chronic kidney disease; HbA1c: Hemoglobin A1c; ALB: Albumin; LDL-C: Low-density lipoprotein cholesterol; CRP: C-reactive protein; eGFR: Estimated glomerular filtration rate; Scr: Serum creatinine; Cys-C: Cystatin C; GLU: glucose | |

**Fig. S1 Directed acyclic graph (DAG) for the association between muscle quality index (MQI) and all-cause dementia (ACD) among stroke survivors.** The green node denotes the exposure (MQI), and the red node denotes the outcome (ACD). White nodes represent measured confounders, including demographic factors (age, gender, race, education level and Townsend deprivation index), lifestyle, psychosocial and anthropometric factors (smoking, alcohol, healthy diet, sleep duration, physical exercise, loneliness and body mass index), and stroke duration. Orange nodes marked “M” represent potential mediators (hypertension, diabetes, chronic kidney disease, albumin, C-reactive protein and LDL cholesterol). Dashed brown nodes marked “P” represent disease proxies (antihypertensive drugs, cholesterol-lowering drugs, aspirin and insulin use), and the dashed grey node represents unmeasured stroke severity. Grey arrows indicate assumed causal relations among covariates; the thick blue arrow indicates the primary causal pathway of interest from MQI to ACD. For estimation of the total effect of MQI on ACD, adjustment was targeted at measured confounders rather than potential mediators or disease proxies.

**Fig. S2 Selection of metabolites via the LASSO regression in the training cohort.** (A‑C) Left-hand panels display cross-validation curves for the cross-validation error (CVE) plotted against log(λ). Numbers at the top of each plot represent the count of non-zero coefficients, while the red and blue dashed lines indicate logλ.min and logλ.1se, respectively. Right-hand panels present coefficient-profile curves, in which each coloured line corresponds to one candidate metabolite predictor. As the value of log(λ) increases, the regression coefficient of each metabolite shrinks towards zero. Panels A, B and C correspond to the three dementia-specific models described.

**Fig. S3 Main and interaction effects of the top 10 metabolite features.** A1, B1 and C1 show SHAP interaction matrices for ACD, AD and VaD: values below the diagonal are mean absolute interaction strengths, and violins above the diagonal show pairwise interaction distributions colored by feature value. A2, B2 and C2 compare each feature’s main effect (mean |SHAP|) with its total interaction contribution, separating largely independent predictors from those acting through coupling with other metabolites.

**Fig. S4 Performance of the models for predicting 10-year post-stroke dementia risk.** Rows show 10-year prediction of all-cause dementia (ACD, top), Alzheimer's disease (AD, middle) and vascular dementia (VaD, bottom). Left: time-dependent ROC curves. For ACD, AUCs (95% CI) were 0.750 (0.703–0.813) for the Base model, 0.754 (0.710–0.814) for Base + MQI, 0.767 (0.715–0.827) for Base + MRS, and 0.772 (0.721–0.831) for Base + MQI + MRS; corresponding AUCs for AD were 0.807 (0.711–0.910), 0.819 (0.754–0.909), 0.850 (0.755–0.940) and 0.861 (0.784–0.949); and for VaD were 0.814 (0.762–0.894), 0.825 (0.775–0.908), 0.839 (0.779–0.915) and 0.844 (0.790–0.920). Middle: calibration plots of observed cumulative incidence against mean predicted risk, with the dashed line indicating perfect agreement. Right: decision curve analysis, showing the net benefit of each model across threshold probabilities relative to "treat all" and "treat none" strategies.

**Fig. S5 Sensitivity analyses for two‑sample MR investigating appendicular lean mass and whole-body fat-free mass in relation to Alzheimer's disease.** (A) Leave-one-out plots, showing IVW causal estimates sequentially omitting one SNP at a time. (B) Funnel plots of Wald‑ratio estimates versus precision. (C) Scatter plots comparing SNP-level effects and results from multiple pleiotropy-robust MR estimators.
