# Supplementary material for: Muscle quality index combined with plasma metabolomics: enhancing risk stratification for post-stroke dementia
Source: Front Aging Neurosci. 2026 Sep 29;18:1837030. doi: 10.3389/fnagi.2026.1837030 (PMC13658159; doi:10.3389/fnagi.2026.1837030)
Supplement: Supplementary file 2 [file Data_Sheet_2.DOCX]

**Table S16. SNP instruments for forward Mendelian randomization**

| **Exposure** | **Outcome** | **SNP** | **chr:pos** | **EA/OA** | **EAF** | **beta_x (SE)** | **P_x** | **beta_y (SE)** | **P_y** | **F** | **Wald ratio** |
| --- | --- | --- | --- | --- | --- | --- | --- | --- | --- | --- | --- |
| Hand grip strength (left) | Alzheimer's disease (Bellenguez 2022) | rs143384 | 20:34025756 | G/A | 0.404 | 0.0209 (0.0015) | 1.5E-43 | -0.0057 (0.0082) | 0.487 | 191.5 | -0.272 |
| Hand grip strength (left) | Alzheimer's disease (Bellenguez 2022) | rs12316046 | 12:15054415 | G/A | 0.378 | -0.0174 (0.0015) | 5.0E-30 | -0.0011 (0.0084) | 0.895 | 129.6 | 0.063 |
| Hand grip strength (left) | Alzheimer's disease (Bellenguez 2022) | rs10846071 | 12:15016236 | T/C | 0.394 | -0.0166 (0.0015) | 5.3E-28 | -0.0030 (0.0083) | 0.720 | 120.4 | 0.180 |
| Hand grip strength (left) | Alzheimer's disease (Bellenguez 2022) | rs1556659 | 10:130834698 | T/C | 0.382 | 0.0163 (0.0015) | 2.5E-26 | 0.0072 (0.0087) | 0.412 | 112.7 | 0.442 |
| Hand grip strength (left) | Alzheimer's disease (Bellenguez 2022) | rs72977282 | 11:74300441 | A/T | 0.414 | -0.0156 (0.0015) | 7.8E-25 | -0.0108 (0.0083) | 0.192 | 105.9 | 0.694 |
| Hand grip strength (left) | Alzheimer's disease (Bellenguez 2022) | rs13146142 | 4:17931318 | C/T | 0.159 | -0.0202 (0.0020) | 2.3E-23 | 0.0385 (0.0110) | 4.6E-04 | 99.2 | -1.906 |
| Hand grip strength (left) | Alzheimer's disease (Bellenguez 2022) | rs3118903 | 13:51099577 | A/G | 0.220 | -0.0174 (0.0018) | 2.7E-22 | -0.0029 (0.0098) | 0.768 | 94.3 | 0.166 |
| Hand grip strength (left) | Alzheimer's disease (Bellenguez 2022) | rs2871865 | 15:99194896 | G/C | 0.116 | -0.0218 (0.0023) | 5.0E-21 | 0.0288 (0.0125) | 0.022 | 88.5 | -1.321 |
| Hand grip strength (left) | Alzheimer's disease (Bellenguez 2022) | rs1044299 | 1:176811873 | T/C | 0.546 | 0.0140 (0.0015) | 6.6E-21 | -0.0075 (0.0082) | 0.360 | 88.0 | -0.535 |
| Hand grip strength (left) | Alzheimer's disease (Bellenguez 2022) | rs12790261 | 11:66988048 | A/C | 0.082 | -0.0252 (0.0027) | 1.2E-20 | 0.0232 (0.0157) | 0.138 | 86.8 | -0.921 |
| Hand grip strength (left) | Alzheimer's disease (Bellenguez 2022) | rs6977081 | 7:150542515 | T/G | 0.334 | 0.0148 (0.0016) | 1.4E-20 | 0.0041 (0.0086) | 0.634 | 86.5 | 0.278 |
| Hand grip strength (left) | Alzheimer's disease (Bellenguez 2022) | rs13107325 | 4:103188709 | T/C | 0.075 | -0.0262 (0.0028) | 1.8E-20 | 0.0455 (0.0160) | 0.004 | 86.0 | -1.740 |
| Hand grip strength (left) | Alzheimer's disease (Bellenguez 2022) | rs4713506 | 6:32113980 | A/G | 0.256 | -0.0157 (0.0017) | 2.0E-20 | -0.0104 (0.0090) | 0.253 | 85.8 | 0.661 |
| Hand grip strength (left) | Alzheimer's disease (Bellenguez 2022) | rs3819121 | 2:135622860 | C/T | 0.369 | 0.0141 (0.0015) | 2.9E-20 | 0.0382 (0.0084) | 5.5E-06 | 85.0 | 2.710 |
| Hand grip strength (left) | Alzheimer's disease (Bellenguez 2022) | rs10788958 | 1:54040670 | G/C | 0.645 | 0.0142 (0.0016) | 1.0E-19 | -0.0065 (0.0087) | 0.458 | 82.5 | -0.458 |
| Hand grip strength (left) | Alzheimer's disease (Bellenguez 2022) | rs7761063 | 6:130379973 | C/A | 0.673 | -0.0141 (0.0016) | 5.1E-19 | 0.0001 (0.0087) | 0.992 | 79.4 | -0.007 |
| Hand grip strength (left) | Alzheimer's disease (Bellenguez 2022) | rs4308051 | 18:20735461 | G/T | 0.789 | 0.0160 (0.0018) | 1.4E-18 | -0.0041 (0.0099) | 0.680 | 77.3 | -0.256 |
| Hand grip strength (left) | Alzheimer's disease (Bellenguez 2022) | rs7571789 | 2:70714793 | C/T | 0.523 | 0.0130 (0.0015) | 3.0E-18 | -0.0029 (0.0082) | 0.723 | 75.9 | -0.224 |
| Hand grip strength (left) | Alzheimer's disease (Bellenguez 2022) | rs755547 | 17:43011908 | A/G | 0.189 | 0.0165 (0.0019) | 3.2E-18 | 0.0164 (0.0103) | 0.110 | 75.7 | 0.993 |
| Hand grip strength (left) | Alzheimer's disease (Bellenguez 2022) | rs11125803 | 2:25052177 | T/C | 0.741 | 0.0143 (0.0017) | 3.8E-17 | 0.0116 (0.0093) | 0.210 | 70.9 | 0.812 |
| Hand grip strength (left) | Alzheimer's disease (Bellenguez 2022) | rs6689375 | 1:227721627 | T/A | 0.185 | -0.0160 (0.0019) | 5.3E-17 | 0.0066 (0.0106) | 0.537 | 70.2 | -0.412 |
| Hand grip strength (left) | Alzheimer's disease (Bellenguez 2022) | rs999493 | 17:46625519 | A/G | 0.622 | 0.0129 (0.0015) | 6.2E-17 | 0.0147 (0.0083) | 0.077 | 69.9 | 1.142 |
| Hand grip strength (left) | Alzheimer's disease (Bellenguez 2022) | rs635538 | 18:53273614 | A/G | 0.914 | -0.0217 (0.0027) | 3.8E-16 | 0.0097 (0.0147) | 0.509 | 66.4 | -0.448 |
| Hand grip strength (left) | Alzheimer's disease (Bellenguez 2022) | rs2871960 | 3:141121814 | C/A | 0.445 | 0.0121 (0.0015) | 5.5E-16 | -0.0129 (0.0082) | 0.118 | 65.6 | -1.067 |
| Hand grip strength (left) | Alzheimer's disease (Bellenguez 2022) | rs113434679 | 17:44126765 | A/C | 0.200 | -0.0149 (0.0019) | 1.9E-15 | -0.0484 (0.0105) | 4.3E-06 | 63.2 | 3.245 |
| Hand grip strength (left) | Alzheimer's disease (Bellenguez 2022) | rs7196917 | 16:69896527 | G/A | 0.430 | -0.0117 (0.0015) | 5.4E-15 | 0.0017 (0.0082) | 0.840 | 61.1 | -0.145 |
| Hand grip strength (left) | Alzheimer's disease (Bellenguez 2022) | rs4621706 | 7:39303296 | T/C | 0.544 | -0.0117 (0.0015) | 6.0E-15 | -0.0167 (0.0082) | 0.041 | 60.9 | 1.427 |
| Hand grip strength (left) | Alzheimer's disease (Bellenguez 2022) | rs17466480 | 15:77390870 | G/A | 0.387 | -0.0118 (0.0015) | 9.8E-15 | 0.0055 (0.0084) | 0.513 | 59.9 | -0.465 |
| Hand grip strength (left) | Alzheimer's disease (Bellenguez 2022) | rs7124681 | 11:47529947 | A/C | 0.408 | -0.0117 (0.0015) | 1.0E-14 | -0.0242 (0.0082) | 0.003 | 59.9 | 2.076 |
| Hand grip strength (left) | Alzheimer's disease (Bellenguez 2022) | rs11076004 | 16:53913930 | A/G | 0.418 | -0.0115 (0.0015) | 2.1E-14 | 0.0005 (0.0084) | 0.953 | 58.4 | -0.043 |
| Hand grip strength (left) | Alzheimer's disease (Bellenguez 2022) | rs823130 | 1:205714372 | T/C | 0.433 | -0.0113 (0.0015) | 4.4E-14 | -0.0007 (0.0082) | 0.935 | 57.0 | 0.062 |
| Hand grip strength (left) | Alzheimer's disease (Bellenguez 2022) | rs12473732 | 2:44118428 | T/C | 0.487 | 0.0110 (0.0015) | 1.4E-13 | -0.0056 (0.0083) | 0.501 | 54.7 | -0.510 |
| Hand grip strength (left) | Alzheimer's disease (Bellenguez 2022) | rs13337177 | 16:2175323 | T/G | 0.181 | -0.0143 (0.0019) | 1.6E-13 | 0.0129 (0.0108) | 0.231 | 54.4 | -0.903 |
| Hand grip strength (left) | Alzheimer's disease (Bellenguez 2022) | rs2631360 | 5:131707429 | A/G | 0.519 | -0.0109 (0.0015) | 1.9E-13 | 0.0114 (0.0081) | 0.161 | 54.1 | -1.044 |
| Hand grip strength (left) | Alzheimer's disease (Bellenguez 2022) | rs7856625 | 9:119345083 | T/C | 0.610 | -0.0111 (0.0015) | 2.5E-13 | -0.0096 (0.0083) | 0.249 | 53.5 | 0.863 |
| Hand grip strength (left) | Alzheimer's disease (Bellenguez 2022) | rs150330307 | 1:160160801 | C/T | 0.032 | -0.0308 (0.0042) | 2.9E-13 | -0.0099 (0.0245) | 0.686 | 53.3 | 0.322 |
| Hand grip strength (left) | Alzheimer's disease (Bellenguez 2022) | rs12906830 | 15:56963503 | C/T | 0.601 | 0.0108 (0.0015) | 9.3E-13 | -0.0091 (0.0083) | 0.273 | 51.0 | -0.839 |
| Hand grip strength (left) | Alzheimer's disease (Bellenguez 2022) | rs61389091 | 11:74427921 | T/C | 0.042 | 0.0262 (0.0037) | 2.4E-12 | 0.0154 (0.0203) | 0.448 | 49.1 | 0.589 |
| Hand grip strength (left) | Alzheimer's disease (Bellenguez 2022) | rs2273555 | 10:104127171 | A/G | 0.606 | 0.0106 (0.0015) | 2.7E-12 | -0.0135 (0.0083) | 0.103 | 48.9 | -1.271 |
| Hand grip strength (left) | Alzheimer's disease (Bellenguez 2022) | rs11121542 | 1:10393920 | A/G | 0.123 | -0.0158 (0.0023) | 3.0E-12 | -0.0009 (0.0123) | 0.945 | 48.7 | 0.057 |
| Hand grip strength (left) | Alzheimer's disease (Bellenguez 2022) | rs3814877 | 16:30042677 | T/G | 0.402 | 0.0105 (0.0015) | 3.4E-12 | -0.0561 (0.0084) | 2.3E-11 | 48.5 | -5.323 |
| Hand grip strength (left) | Alzheimer's disease (Bellenguez 2022) | rs7963801 | 12:79685226 | C/T | 0.572 | -0.0104 (0.0015) | 4.6E-12 | 0.0034 (0.0085) | 0.692 | 47.9 | -0.326 |
| Hand grip strength (left) | Alzheimer's disease (Bellenguez 2022) | rs12889267 | 14:21542766 | G/A | 0.167 | -0.0137 (0.0020) | 4.7E-12 | -0.0061 (0.0117) | 0.604 | 47.8 | 0.444 |
| Hand grip strength (left) | Alzheimer's disease (Bellenguez 2022) | rs10176878 | 2:59952274 | C/T | 0.191 | -0.0129 (0.0019) | 8.6E-12 | 0.0072 (0.0105) | 0.492 | 46.6 | -0.556 |
| Hand grip strength (left) | Alzheimer's disease (Bellenguez 2022) | rs7970350 | 12:66360164 | T/C | 0.494 | -0.0101 (0.0015) | 8.6E-12 | 0.0014 (0.0081) | 0.861 | 46.6 | -0.138 |
| Hand grip strength (left) | Alzheimer's disease (Bellenguez 2022) | rs28542042 | 15:74213357 | T/C | 0.309 | 0.0110 (0.0016) | 1.2E-11 | -0.0045 (0.0089) | 0.612 | 46.0 | -0.409 |
| Hand grip strength (left) | Alzheimer's disease (Bellenguez 2022) | rs1641457 | 2:40421990 | G/T | 0.223 | 0.0120 (0.0018) | 1.4E-11 | 0.0105 (0.0097) | 0.278 | 45.6 | 0.872 |
| Hand grip strength (left) | Alzheimer's disease (Bellenguez 2022) | rs10403906 | 19:37376756 | A/G | 0.476 | -0.0100 (0.0015) | 1.5E-11 | -0.0144 (0.0087) | 0.097 | 45.5 | 1.435 |
| Hand grip strength (left) | Alzheimer's disease (Bellenguez 2022) | rs4575361 | 12:124410529 | T/A | 0.312 | -0.0108 (0.0016) | 1.6E-11 | 0.0123 (0.0087) | 0.157 | 45.5 | -1.139 |
| Hand grip strength (left) | Alzheimer's disease (Bellenguez 2022) | rs12763284 | 10:104508202 | G/A | 0.466 | 0.0100 (0.0015) | 1.7E-11 | 0.0045 (0.0081) | 0.581 | 45.3 | 0.450 |
| Hand grip strength (left) | Alzheimer's disease (Bellenguez 2022) | rs10097417 | 8:110361477 | G/A | 0.171 | -0.0132 (0.0020) | 1.9E-11 | 0.0087 (0.0108) | 0.425 | 45.1 | -0.658 |
| Hand grip strength (left) | Alzheimer's disease (Bellenguez 2022) | rs4363950 | 19:52221213 | G/A | 0.708 | 0.0110 (0.0016) | 1.9E-11 | -0.0062 (0.0095) | 0.517 | 45.1 | -0.565 |
| Hand grip strength (left) | Alzheimer's disease (Bellenguez 2022) | rs34030812 | 2:144248905 | C/T | 0.367 | -0.0102 (0.0015) | 4.1E-11 | 0.0069 (0.0085) | 0.417 | 43.6 | -0.679 |
| Hand grip strength (left) | Alzheimer's disease (Bellenguez 2022) | rs2532111 | 17:62017421 | G/A | 0.640 | 0.0103 (0.0016) | 4.1E-11 | -0.0199 (0.0086) | 0.021 | 43.6 | -1.935 |
| Hand grip strength (left) | Alzheimer's disease (Bellenguez 2022) | rs11769549 | 7:23122239 | A/T | 0.062 | 0.0205 (0.0031) | 4.5E-11 | -0.0177 (0.0169) | 0.296 | 43.4 | -0.864 |
| Hand grip strength (left) | Alzheimer's disease (Bellenguez 2022) | rs62253653 | 3:53013267 | G/A | 0.295 | 0.0107 (0.0016) | 5.5E-11 | -0.0208 (0.0090) | 0.021 | 43.0 | -1.944 |
| Hand grip strength (left) | Alzheimer's disease (Bellenguez 2022) | rs6680160 | 1:32072737 | G/A | 0.628 | 0.0101 (0.0015) | 6.0E-11 | -0.0168 (0.0084) | 0.047 | 42.8 | -1.669 |
| Hand grip strength (left) | Alzheimer's disease (Bellenguez 2022) | rs1486925 | 8:78827617 | C/T | 0.315 | -0.0105 (0.0016) | 6.3E-11 | -0.0027 (0.0087) | 0.755 | 42.7 | 0.258 |
| Hand grip strength (left) | Alzheimer's disease (Bellenguez 2022) | rs11243202 | 6:7719065 | C/T | 0.486 | 0.0097 (0.0015) | 7.0E-11 | 0.0116 (0.0082) | 0.156 | 42.5 | 1.193 |
| Hand grip strength (left) | Alzheimer's disease (Bellenguez 2022) | rs12914702 | 15:96887277 | A/G | 0.300 | 0.0110 (0.0017) | 9.3E-11 | 0.0085 (0.0104) | 0.409 | 42.0 | 0.776 |
| Hand grip strength (left) | Alzheimer's disease (Bellenguez 2022) | rs4737446 | 8:57665019 | T/G | 0.695 | 0.0104 (0.0016) | 1.2E-10 | -0.0095 (0.0088) | 0.282 | 41.4 | -0.912 |
| Hand grip strength (left) | Alzheimer's disease (Bellenguez 2022) | rs16870531 | 7:120660682 | T/C | 0.238 | 0.0112 (0.0017) | 1.3E-10 | 0.0041 (0.0095) | 0.670 | 41.3 | 0.366 |
| Hand grip strength (left) | Alzheimer's disease (Bellenguez 2022) | rs2431112 | 5:103931707 | A/G | 0.441 | -0.0096 (0.0015) | 1.4E-10 | 0.0041 (0.0082) | 0.617 | 41.1 | -0.428 |
| Hand grip strength (left) | Alzheimer's disease (Bellenguez 2022) | rs56338231 | 4:30867393 | G/A | 0.258 | -0.0108 (0.0017) | 1.7E-10 | 0.0031 (0.0093) | 0.741 | 40.8 | -0.286 |
| Hand grip strength (left) | Alzheimer's disease (Bellenguez 2022) | rs11002322 | 10:79649653 | T/G | 0.340 | -0.0100 (0.0016) | 2.1E-10 | -0.0030 (0.0086) | 0.723 | 40.4 | 0.300 |
| Hand grip strength (left) | Alzheimer's disease (Bellenguez 2022) | rs217181 | 16:72114002 | T/C | 0.193 | 0.0120 (0.0019) | 2.1E-10 | 0.0313 (0.0102) | 0.002 | 40.4 | 2.618 |
| Hand grip strength (left) | Alzheimer's disease (Bellenguez 2022) | rs8108461 | 19:2186757 | C/T | 0.573 | 0.0095 (0.0015) | 2.6E-10 | 0.0087 (0.0090) | 0.330 | 40.0 | 0.913 |
| Hand grip strength (left) | Alzheimer's disease (Bellenguez 2022) | rs41271299 | 6:19839415 | T/C | 0.051 | 0.0212 (0.0034) | 3.0E-10 | -0.0015 (0.0192) | 0.936 | 39.7 | -0.071 |
| Hand grip strength (left) | Alzheimer's disease (Bellenguez 2022) | rs34587452 | 4:1009900 | C/G | 0.215 | -0.0114 (0.0018) | 3.2E-10 | 0.0276 (0.0101) | 0.006 | 39.5 | -2.429 |
| Hand grip strength (left) | Alzheimer's disease (Bellenguez 2022) | rs4121165 | 1:78276977 | A/G | 0.211 | -0.0114 (0.0018) | 3.4E-10 | -0.0049 (0.0100) | 0.621 | 39.4 | 0.429 |
| Hand grip strength (left) | Alzheimer's disease (Bellenguez 2022) | rs7575451 | 2:152352843 | G/C | 0.650 | -0.0097 (0.0016) | 3.8E-10 | 0.0026 (0.0085) | 0.760 | 39.2 | -0.267 |
| Hand grip strength (left) | Alzheimer's disease (Bellenguez 2022) | rs6802071 | 3:38574237 | T/C | 0.435 | -0.0094 (0.0015) | 3.8E-10 | 0.0099 (0.0082) | 0.231 | 39.2 | -1.053 |
| Hand grip strength (left) | Alzheimer's disease (Bellenguez 2022) | rs76895963 | 12:4384844 | G/T | 0.021 | 0.0360 (0.0057) | 3.9E-10 | -0.0069 (0.0332) | 0.836 | 39.1 | -0.192 |
| Hand grip strength (left) | Alzheimer's disease (Bellenguez 2022) | rs34845616 | 11:133792644 | A/G | 0.246 | 0.0108 (0.0017) | 4.0E-10 | 0.0234 (0.0096) | 0.015 | 39.1 | 2.159 |
| Hand grip strength (left) | Alzheimer's disease (Bellenguez 2022) | rs4498020 | 3:13810820 | A/C | 0.724 | -0.0104 (0.0017) | 4.3E-10 | 0.0151 (0.0091) | 0.096 | 39.0 | -1.449 |
| Hand grip strength (left) | Alzheimer's disease (Bellenguez 2022) | rs11642954 | 16:24824248 | A/G | 0.195 | -0.0117 (0.0019) | 4.5E-10 | 0.0075 (0.0102) | 0.465 | 38.9 | -0.641 |
| Hand grip strength (left) | Alzheimer's disease (Bellenguez 2022) | rs10821939 | 10:63751748 | A/G | 0.573 | -0.0094 (0.0015) | 4.9E-10 | -0.0077 (0.0083) | 0.349 | 38.7 | 0.823 |
| Hand grip strength (left) | Alzheimer's disease (Bellenguez 2022) | rs1434095 | 2:179254330 | C/T | 0.875 | 0.0140 (0.0023) | 5.3E-10 | -0.0166 (0.0123) | 0.178 | 38.6 | -1.183 |
| Hand grip strength (left) | Alzheimer's disease (Bellenguez 2022) | rs35609019 | 4:7847892 | C/G | 0.398 | 0.0095 (0.0015) | 7.7E-10 | 0.0056 (0.0085) | 0.510 | 37.8 | 0.591 |
| Hand grip strength (left) | Alzheimer's disease (Bellenguez 2022) | rs10831903 | 11:12758660 | T/C | 0.423 | 0.0093 (0.0015) | 8.1E-10 | 0.0085 (0.0083) | 0.307 | 37.8 | 0.915 |
| Hand grip strength (left) | Alzheimer's disease (Bellenguez 2022) | rs75497896 | 4:119636703 | C/T | 0.051 | -0.0207 (0.0034) | 9.1E-10 | 0.0525 (0.0189) | 0.005 | 37.5 | -2.540 |
| Hand grip strength (left) | Alzheimer's disease (Bellenguez 2022) | rs9371881 | 6:155638213 | A/G | 0.359 | 0.0095 (0.0015) | 9.6E-10 | 0.0016 (0.0087) | 0.850 | 37.4 | 0.169 |
| Hand grip strength (left) | Alzheimer's disease (Bellenguez 2022) | rs1981612 | 2:199235664 | A/C | 0.456 | 0.0092 (0.0015) | 1.0E-09 | 0.0125 (0.0084) | 0.137 | 37.3 | 1.355 |
| Hand grip strength (left) | Alzheimer's disease (Bellenguez 2022) | rs10205394 | 2:218150948 | C/G | 0.201 | -0.0113 (0.0019) | 1.1E-09 | -0.0093 (0.0099) | 0.348 | 37.2 | 0.821 |
| Hand grip strength (left) | Alzheimer's disease (Bellenguez 2022) | rs4677601 | 3:71368790 | G/A | 0.510 | 0.0091 (0.0015) | 1.1E-09 | 0.0143 (0.0082) | 0.081 | 37.2 | 1.579 |
| Hand grip strength (left) | Alzheimer's disease (Bellenguez 2022) | rs73307079 | 7:19020024 | C/T | 0.211 | 0.0111 (0.0018) | 1.4E-09 | 0.0230 (0.0099) | 0.020 | 36.6 | 2.072 |
| Hand grip strength (left) | Alzheimer's disease (Bellenguez 2022) | rs7197751 | 16:84940033 | T/G | 0.363 | -0.0095 (0.0016) | 1.4E-09 | 0.0016 (0.0086) | 0.855 | 36.7 | -0.169 |
| Hand grip strength (left) | Alzheimer's disease (Bellenguez 2022) | rs6433478 | 2:175241482 | C/T | 0.544 | 0.0091 (0.0015) | 1.5E-09 | 0.0235 (0.0082) | 0.004 | 36.5 | 2.595 |
| Hand grip strength (left) | Alzheimer's disease (Bellenguez 2022) | rs821100 | 8:89448877 | G/A | 0.266 | -0.0102 (0.0017) | 1.5E-09 | -0.0020 (0.0091) | 0.826 | 36.6 | 0.196 |
| Hand grip strength (left) | Alzheimer's disease (Bellenguez 2022) | rs723588 | 6:81056634 | C/T | 0.143 | 0.0128 (0.0021) | 1.6E-09 | -0.0073 (0.0116) | 0.530 | 36.4 | -0.571 |
| Hand grip strength (left) | Alzheimer's disease (Bellenguez 2022) | rs7148603 | 14:36683779 | A/G | 0.359 | 0.0096 (0.0016) | 1.6E-09 | 0.0126 (0.0086) | 0.144 | 36.4 | 1.316 |
| Hand grip strength (left) | Alzheimer's disease (Bellenguez 2022) | rs10988217 | 9:131888116 | G/A | 0.604 | -0.0092 (0.0015) | 1.7E-09 | 0.0053 (0.0083) | 0.522 | 36.2 | -0.576 |
| Hand grip strength (left) | Alzheimer's disease (Bellenguez 2022) | rs7176095 | 15:74886411 | G/A | 0.128 | -0.0134 (0.0022) | 1.7E-09 | -0.0076 (0.0123) | 0.535 | 36.3 | 0.568 |
| Hand grip strength (left) | Alzheimer's disease (Bellenguez 2022) | rs10144445 | 14:39695362 | G/C | 0.350 | -0.0094 (0.0016) | 1.9E-09 | 0.0233 (0.0084) | 0.006 | 36.1 | -2.487 |
| Hand grip strength (left) | Alzheimer's disease (Bellenguez 2022) | rs181766 | 16:14394878 | C/T | 0.322 | 0.0096 (0.0016) | 1.9E-09 | 0.0017 (0.0087) | 0.846 | 36.1 | 0.176 |
| Hand grip strength (left) | Alzheimer's disease (Bellenguez 2022) | rs2587505 | 17:77784268 | C/T | 0.420 | -0.0090 (0.0015) | 2.3E-09 | 0.0104 (0.0084) | 0.213 | 35.7 | -1.155 |
| Hand grip strength (left) | Alzheimer's disease (Bellenguez 2022) | rs12673062 | 7:4710677 | A/G | 0.216 | -0.0108 (0.0018) | 2.6E-09 | -0.0045 (0.0102) | 0.657 | 35.5 | 0.418 |
| Hand grip strength (left) | Alzheimer's disease (Bellenguez 2022) | rs6006984 | 22:45714937 | C/T | 0.278 | 0.0098 (0.0017) | 2.6E-09 | 0.0007 (0.0090) | 0.934 | 35.5 | 0.071 |
| Hand grip strength (left) | Alzheimer's disease (Bellenguez 2022) | rs6882168 | 5:39402647 | T/C | 0.337 | -0.0094 (0.0016) | 2.8E-09 | -0.0075 (0.0088) | 0.395 | 35.3 | 0.802 |
| Hand grip strength (left) | Alzheimer's disease (Bellenguez 2022) | rs12528131 | 6:105389104 | G/A | 0.488 | -0.0088 (0.0015) | 2.8E-09 | -0.0071 (0.0081) | 0.383 | 35.3 | 0.804 |
| Hand grip strength (left) | Alzheimer's disease (Bellenguez 2022) | rs9371201 | 6:150145001 | T/C | 0.335 | -0.0093 (0.0016) | 3.0E-09 | -0.0211 (0.0086) | 0.014 | 35.2 | 2.259 |
| Hand grip strength (left) | Alzheimer's disease (Bellenguez 2022) | rs6962338 | 7:69160985 | G/A | 0.044 | -0.0214 (0.0036) | 3.2E-09 | 0.0398 (0.0209) | 0.056 | 35.0 | -1.859 |
| Hand grip strength (left) | Alzheimer's disease (Bellenguez 2022) | rs77485342 | 6:30842866 | T/C | 0.018 | 0.0330 (0.0056) | 3.5E-09 | 0.0537 (0.0326) | 0.100 | 34.9 | 1.629 |
| Hand grip strength (left) | Alzheimer's disease (Bellenguez 2022) | rs4930236 | 11:68414000 | A/C | 0.836 | 0.0119 (0.0020) | 3.8E-09 | 0.0211 (0.0112) | 0.059 | 34.7 | 1.773 |
| Hand grip strength (left) | Alzheimer's disease (Bellenguez 2022) | rs13106087 | 4:145566864 | C/T | 0.830 | 0.0116 (0.0020) | 3.9E-09 | -0.0128 (0.0106) | 0.228 | 34.6 | -1.102 |
| Hand grip strength (left) | Alzheimer's disease (Bellenguez 2022) | rs17630248 | 2:201137782 | C/T | 0.348 | -0.0092 (0.0016) | 4.1E-09 | -0.0084 (0.0085) | 0.324 | 34.6 | 0.915 |
| Hand grip strength (left) | Alzheimer's disease (Bellenguez 2022) | rs4335354 | 1:88899964 | A/C | 0.316 | -0.0094 (0.0016) | 4.6E-09 | 0.0025 (0.0089) | 0.775 | 34.4 | -0.266 |
| Hand grip strength (left) | Alzheimer's disease (Bellenguez 2022) | rs9866627 | 3:135522715 | A/C | 0.084 | -0.0156 (0.0027) | 5.7E-09 | 0.0211 (0.0148) | 0.153 | 33.9 | -1.353 |
| Hand grip strength (left) | Alzheimer's disease (Bellenguez 2022) | rs35236379 | 10:5727292 | T/G | 0.142 | 0.0124 (0.0021) | 5.8E-09 | 0.0217 (0.0119) | 0.068 | 33.9 | 1.756 |
| Hand grip strength (left) | Alzheimer's disease (Bellenguez 2022) | rs1924721 | 1:190972986 | T/G | 0.117 | 0.0134 (0.0023) | 6.2E-09 | 0.0007 (0.0128) | 0.954 | 33.8 | 0.052 |
| Hand grip strength (left) | Alzheimer's disease (Bellenguez 2022) | rs997850 | 4:154838434 | C/G | 0.605 | -0.0089 (0.0015) | 6.3E-09 | -0.0189 (0.0084) | 0.024 | 33.7 | 2.130 |
| Hand grip strength (left) | Alzheimer's disease (Bellenguez 2022) | rs2038760 | 6:2680732 | T/C | 0.171 | -0.0115 (0.0020) | 6.6E-09 | 0.0056 (0.0107) | 0.603 | 33.7 | -0.486 |
| Hand grip strength (left) | Alzheimer's disease (Bellenguez 2022) | rs2359239 | 14:75326771 | T/C | 0.392 | -0.0088 (0.0015) | 6.9E-09 | 0.0060 (0.0082) | 0.464 | 33.6 | -0.681 |
| Hand grip strength (left) | Alzheimer's disease (Bellenguez 2022) | rs11204664 | 1:150531380 | C/T | 0.579 | -0.0087 (0.0015) | 8.1E-09 | 0.0091 (0.0083) | 0.269 | 33.2 | -1.052 |
| Hand grip strength (left) | Alzheimer's disease (Bellenguez 2022) | rs13227429 | 7:140560023 | C/T | 0.561 | -0.0086 (0.0015) | 9.8E-09 | 0.0196 (0.0083) | 0.018 | 32.9 | -2.282 |
| Hand grip strength (left) | Alzheimer's disease (Bellenguez 2022) | rs143002906 | 18:12992162 | T/C | 0.028 | 0.0262 (0.0046) | 9.8E-09 | -0.0060 (0.0243) | 0.805 | 32.9 | -0.229 |
| Hand grip strength (left) | Alzheimer's disease (Bellenguez 2022) | rs61286123 | 2:60205600 | C/T | 0.228 | -0.0101 (0.0018) | 1.2E-08 | 0.0004 (0.0097) | 0.969 | 32.5 | -0.040 |
| Hand grip strength (left) | Alzheimer's disease (Bellenguez 2022) | rs9944324 | 16:80929342 | G/A | 0.457 | -0.0086 (0.0015) | 1.2E-08 | 0.0061 (0.0082) | 0.455 | 32.4 | -0.713 |
| Hand grip strength (left) | Alzheimer's disease (Bellenguez 2022) | rs4739739 | 8:81304576 | G/A | 0.415 | -0.0085 (0.0015) | 1.4E-08 | 0.0024 (0.0083) | 0.776 | 32.2 | -0.281 |
| Hand grip strength (left) | Alzheimer's disease (Bellenguez 2022) | rs113918482 | 5:161289270 | G/A | 0.223 | -0.0101 (0.0018) | 1.5E-08 | 0.0174 (0.0095) | 0.068 | 32.0 | -1.723 |
| Hand grip strength (left) | Alzheimer's disease (Bellenguez 2022) | rs55681913 | 5:42687629 | C/T | 0.106 | 0.0138 (0.0024) | 1.6E-08 | -0.0202 (0.0138) | 0.144 | 32.0 | -1.467 |
| Hand grip strength (left) | Alzheimer's disease (Bellenguez 2022) | rs11003014 | 10:81231387 | G/A | 0.161 | 0.0114 (0.0020) | 1.6E-08 | -0.0127 (0.0109) | 0.246 | 32.0 | -1.111 |
| Hand grip strength (left) | Alzheimer's disease (Bellenguez 2022) | rs10860864 | 12:102822862 | T/C | 0.181 | 0.0108 (0.0019) | 1.9E-08 | 0.0001 (0.0107) | 0.992 | 31.6 | 0.009 |
| Hand grip strength (left) | Alzheimer's disease (Bellenguez 2022) | rs4398863 | 8:135695110 | C/G | 0.737 | -0.0095 (0.0017) | 2.0E-08 | 0.0032 (0.0092) | 0.732 | 31.5 | -0.339 |
| Hand grip strength (left) | Alzheimer's disease (Bellenguez 2022) | rs116922558 | 9:118802375 | G/A | 0.040 | -0.0216 (0.0039) | 2.1E-08 | 0.0084 (0.0214) | 0.696 | 31.4 | -0.389 |
| Hand grip strength (left) | Alzheimer's disease (Bellenguez 2022) | rs1884447 | 1:185021410 | A/G | 0.401 | 0.0085 (0.0015) | 2.3E-08 | 0.0032 (0.0083) | 0.703 | 31.2 | 0.378 |
| Hand grip strength (left) | Alzheimer's disease (Bellenguez 2022) | rs59116179 | 8:22603454 | T/C | 0.617 | 0.0086 (0.0015) | 2.4E-08 | -0.0003 (0.0085) | 0.968 | 31.1 | -0.035 |
| Hand grip strength (left) | Alzheimer's disease (Bellenguez 2022) | rs11168357 | 12:48412138 | A/G | 0.246 | -0.0096 (0.0017) | 2.5E-08 | -0.0001 (0.0095) | 0.994 | 31.1 | 0.010 |
| Hand grip strength (left) | Alzheimer's disease (Bellenguez 2022) | rs116409670 | 5:37327472 | T/C | 0.080 | -0.0152 (0.0027) | 2.6E-08 | -0.0154 (0.0153) | 0.312 | 31.0 | 1.010 |
| Hand grip strength (left) | Alzheimer's disease (Bellenguez 2022) | rs12533765 | 7:127699186 | G/A | 0.280 | -0.0092 (0.0017) | 2.6E-08 | -0.0005 (0.0091) | 0.959 | 31.0 | 0.054 |
| Hand grip strength (left) | Alzheimer's disease (Bellenguez 2022) | rs62081464 | 18:35142133 | T/C | 0.227 | -0.0099 (0.0018) | 2.8E-08 | 0.0195 (0.0097) | 0.045 | 30.8 | -1.973 |
| Hand grip strength (left) | Alzheimer's disease (Bellenguez 2022) | rs4811040 | 20:48968438 | G/C | 0.277 | -0.0092 (0.0017) | 3.0E-08 | 0.0075 (0.0092) | 0.419 | 30.7 | -0.811 |
| Hand grip strength (left) | Alzheimer's disease (Bellenguez 2022) | rs7516571 | 1:40733658 | G/A | 0.259 | 0.0094 (0.0017) | 3.1E-08 | -0.0017 (0.0093) | 0.857 | 30.7 | -0.181 |
| Hand grip strength (left) | Alzheimer's disease (Bellenguez 2022) | rs2800789 | 1:164578242 | C/A | 0.480 | 0.0083 (0.0015) | 3.1E-08 | -0.0067 (0.0083) | 0.420 | 30.6 | -0.811 |
| Hand grip strength (left) | Alzheimer's disease (Bellenguez 2022) | rs4962700 | 10:126479989 | G/C | 0.302 | 0.0090 (0.0016) | 3.2E-08 | -0.0233 (0.0090) | 0.010 | 30.6 | -2.577 |
| Hand grip strength (left) | Alzheimer's disease (Bellenguez 2022) | rs13091492 | 3:81891476 | G/A | 0.373 | -0.0085 (0.0015) | 3.3E-08 | 0.0076 (0.0084) | 0.370 | 30.6 | -0.896 |
| Hand grip strength (left) | Alzheimer's disease (Bellenguez 2022) | rs34722008 | 4:38659594 | A/G | 0.353 | 0.0086 (0.0016) | 3.3E-08 | -0.0095 (0.0087) | 0.277 | 30.5 | -1.106 |
| Hand grip strength (left) | Alzheimer's disease (Bellenguez 2022) | rs2850379 | 4:102917419 | A/C | 0.432 | -0.0083 (0.0015) | 3.3E-08 | 0.0049 (0.0082) | 0.552 | 30.5 | -0.591 |
| Hand grip strength (left) | Alzheimer's disease (Bellenguez 2022) | rs2974438 | 5:168250903 | A/G | 0.211 | -0.0101 (0.0018) | 3.3E-08 | 0.0009 (0.0099) | 0.926 | 30.5 | -0.089 |
| Hand grip strength (left) | Alzheimer's disease (Bellenguez 2022) | rs58670122 | 1:22492613 | G/A | 0.143 | -0.0118 (0.0021) | 3.6E-08 | 0.0211 (0.0115) | 0.066 | 30.4 | -1.786 |
| Hand grip strength (left) | Alzheimer's disease (Bellenguez 2022) | rs16910750 | 9:99084471 | C/G | 0.160 | 0.0112 (0.0020) | 3.6E-08 | 0.0005 (0.0112) | 0.965 | 30.4 | 0.044 |
| Hand grip strength (left) | Alzheimer's disease (Bellenguez 2022) | rs71298370 | 3:71164965 | A/G | 0.086 | 0.0149 (0.0027) | 3.8E-08 | -0.0078 (0.0152) | 0.609 | 30.3 | -0.524 |
| Hand grip strength (left) | Alzheimer's disease (Bellenguez 2022) | rs17282763 | 7:82520166 | C/T | 0.296 | 0.0089 (0.0016) | 4.4E-08 | -0.0072 (0.0089) | 0.420 | 30.0 | -0.805 |
| Hand grip strength (left) | Alzheimer's disease (Bellenguez 2022) | rs7026798 | 9:16427378 | C/T | 0.432 | 0.0082 (0.0015) | 4.6E-08 | -0.0004 (0.0082) | 0.960 | 29.9 | -0.049 |
| Hand grip strength (left) | Alzheimer's disease (Bellenguez 2022) | rs10934857 | 3:128199662 | A/G | 0.259 | 0.0093 (0.0017) | 4.8E-08 | -0.0011 (0.0092) | 0.907 | 29.8 | -0.118 |
| Hand grip strength (left) | Alzheimer's disease (Bellenguez 2022) | rs2789514 | 9:129833029 | A/G | 0.868 | 0.0121 (0.0022) | 4.9E-08 | 0.0167 (0.0123) | 0.175 | 29.8 | 1.385 |
| Hand grip strength (left) | All-cause dementia (FinnGen) | rs143384 | 20:34025756 | G/A | 0.404 | 0.0209 (0.0015) | 1.5E-43 | -0.0099 (0.0193) | 0.610 | 191.5 | -0.473 |
| Hand grip strength (left) | All-cause dementia (FinnGen) | rs12316046 | 12:15054415 | G/A | 0.378 | -0.0174 (0.0015) | 5.0E-30 | -0.0068 (0.0215) | 0.750 | 129.6 | 0.390 |
| Hand grip strength (left) | All-cause dementia (FinnGen) | rs10846071 | 12:15016236 | T/C | 0.394 | -0.0166 (0.0015) | 5.3E-28 | -0.0085 (0.0208) | 0.684 | 120.4 | 0.511 |
| Hand grip strength (left) | All-cause dementia (FinnGen) | rs1556659 | 10:130834698 | T/C | 0.382 | 0.0163 (0.0015) | 2.5E-26 | -0.0031 (0.0210) | 0.882 | 112.7 | -0.190 |
| Hand grip strength (left) | All-cause dementia (FinnGen) | rs13146142 | 4:17931318 | C/T | 0.159 | -0.0202 (0.0020) | 2.3E-23 | 0.0438 (0.0325) | 0.178 | 99.2 | -2.168 |
| Hand grip strength (left) | All-cause dementia (FinnGen) | rs3118903 | 13:51099577 | A/G | 0.220 | -0.0174 (0.0018) | 2.7E-22 | -0.0232 (0.0218) | 0.288 | 94.3 | 1.330 |
| Hand grip strength (left) | All-cause dementia (FinnGen) | rs2871865 | 15:99194896 | G/C | 0.116 | -0.0218 (0.0023) | 5.0E-21 | 0.0616 (0.0398) | 0.122 | 88.5 | -2.826 |
| Hand grip strength (left) | All-cause dementia (FinnGen) | rs1044299 | 1:176811873 | T/C | 0.546 | 0.0140 (0.0015) | 6.6E-21 | -0.0089 (0.0192) | 0.642 | 88.0 | -0.635 |
| Hand grip strength (left) | All-cause dementia (FinnGen) | rs12790261 | 11:66988048 | A/C | 0.082 | -0.0252 (0.0027) | 1.2E-20 | 0.0165 (0.0437) | 0.706 | 86.8 | -0.655 |
| Hand grip strength (left) | All-cause dementia (FinnGen) | rs6977081 | 7:150542515 | T/G | 0.334 | 0.0148 (0.0016) | 1.4E-20 | 0.0096 (0.0200) | 0.631 | 86.5 | 0.651 |
| Hand grip strength (left) | All-cause dementia (FinnGen) | rs13107325 | 4:103188709 | T/C | 0.075 | -0.0262 (0.0028) | 1.8E-20 | 0.1247 (0.0827) | 0.131 | 86.0 | -4.768 |
| Hand grip strength (left) | All-cause dementia (FinnGen) | rs4713506 | 6:32113980 | A/G | 0.256 | -0.0157 (0.0017) | 2.0E-20 | -0.0340 (0.0203) | 0.094 | 85.8 | 2.162 |
| Hand grip strength (left) | All-cause dementia (FinnGen) | rs3819121 | 2:135622860 | C/T | 0.369 | 0.0141 (0.0015) | 2.9E-20 | 0.0346 (0.0196) | 0.078 | 85.0 | 2.455 |
| Hand grip strength (left) | All-cause dementia (FinnGen) | rs10788958 | 1:54040670 | G/C | 0.645 | 0.0142 (0.0016) | 1.0E-19 | 0.0079 (0.0220) | 0.720 | 82.5 | 0.556 |
| Hand grip strength (left) | All-cause dementia (FinnGen) | rs7761063 | 6:130379973 | C/A | 0.673 | -0.0141 (0.0016) | 5.1E-19 | 0.0419 (0.0203) | 0.039 | 79.4 | -2.975 |
| Hand grip strength (left) | All-cause dementia (FinnGen) | rs4308051 | 18:20735461 | G/T | 0.789 | 0.0160 (0.0018) | 1.4E-18 | 0.0371 (0.0228) | 0.103 | 77.3 | 2.320 |
| Hand grip strength (left) | All-cause dementia (FinnGen) | rs7571789 | 2:70714793 | C/T | 0.523 | 0.0130 (0.0015) | 3.0E-18 | -0.0096 (0.0193) | 0.618 | 75.9 | -0.741 |
| Hand grip strength (left) | All-cause dementia (FinnGen) | rs755547 | 17:43011908 | A/G | 0.189 | 0.0165 (0.0019) | 3.2E-18 | -0.0271 (0.0279) | 0.331 | 75.7 | -1.641 |
| Hand grip strength (left) | All-cause dementia (FinnGen) | rs11125803 | 2:25052177 | T/C | 0.741 | 0.0143 (0.0017) | 3.8E-17 | -0.0200 (0.0224) | 0.372 | 70.9 | -1.400 |
| Hand grip strength (left) | All-cause dementia (FinnGen) | rs999493 | 17:46625519 | A/G | 0.622 | 0.0129 (0.0015) | 6.2E-17 | 0.0216 (0.0198) | 0.278 | 69.9 | 1.678 |
| Hand grip strength (left) | All-cause dementia (FinnGen) | rs635538 | 18:53273614 | A/G | 0.914 | -0.0217 (0.0027) | 3.8E-16 | 0.0310 (0.0315) | 0.325 | 66.4 | -1.431 |
| Hand grip strength (left) | All-cause dementia (FinnGen) | rs2871960 | 3:141121814 | C/A | 0.445 | 0.0121 (0.0015) | 5.5E-16 | 0.0486 (0.0192) | 0.011 | 65.6 | 4.021 |
| Hand grip strength (left) | All-cause dementia (FinnGen) | rs113434679 | 17:44126765 | A/C | 0.200 | -0.0149 (0.0019) | 1.9E-15 | -0.0795 (0.0363) | 0.029 | 63.2 | 5.330 |
| Hand grip strength (left) | All-cause dementia (FinnGen) | rs7196917 | 16:69896527 | G/A | 0.430 | -0.0117 (0.0015) | 5.4E-15 | 0.0238 (0.0197) | 0.225 | 61.1 | -2.029 |
| Hand grip strength (left) | All-cause dementia (FinnGen) | rs17466480 | 15:77390870 | G/A | 0.387 | -0.0118 (0.0015) | 9.8E-15 | 0.0127 (0.0198) | 0.523 | 59.9 | -1.074 |
| Hand grip strength (left) | All-cause dementia (FinnGen) | rs7124681 | 11:47529947 | A/C | 0.408 | -0.0117 (0.0015) | 1.0E-14 | -0.0158 (0.0201) | 0.430 | 59.9 | 1.356 |
| Hand grip strength (left) | All-cause dementia (FinnGen) | rs11076004 | 16:53913930 | A/G | 0.418 | -0.0115 (0.0015) | 2.1E-14 | 0.0180 (0.0191) | 0.348 | 58.4 | -1.560 |
| Hand grip strength (left) | All-cause dementia (FinnGen) | rs823130 | 1:205714372 | T/C | 0.433 | -0.0113 (0.0015) | 4.4E-14 | 0.0210 (0.0193) | 0.277 | 57.0 | -1.853 |
| Hand grip strength (left) | All-cause dementia (FinnGen) | rs12473732 | 2:44118428 | T/C | 0.487 | 0.0110 (0.0015) | 1.4E-13 | -0.0330 (0.0192) | 0.086 | 54.7 | -3.004 |
| Hand grip strength (left) | All-cause dementia (FinnGen) | rs13337177 | 16:2175323 | T/G | 0.181 | -0.0143 (0.0019) | 1.6E-13 | 0.0184 (0.0251) | 0.463 | 54.4 | -1.289 |
| Hand grip strength (left) | All-cause dementia (FinnGen) | rs2631360 | 5:131707429 | A/G | 0.519 | -0.0109 (0.0015) | 1.9E-13 | 0.0336 (0.0202) | 0.096 | 54.1 | -3.078 |
| Hand grip strength (left) | All-cause dementia (FinnGen) | rs7856625 | 9:119345083 | T/C | 0.610 | -0.0111 (0.0015) | 2.5E-13 | -0.0402 (0.0200) | 0.045 | 53.5 | 3.612 |
| Hand grip strength (left) | All-cause dementia (FinnGen) | rs150330307 | 1:160160801 | C/T | 0.032 | -0.0308 (0.0042) | 2.9E-13 | -0.0502 (0.0691) | 0.468 | 53.3 | 1.631 |
| Hand grip strength (left) | All-cause dementia (FinnGen) | rs1551042 | 3:85630551 | C/A | 0.647 | -0.0111 (0.0016) | 7.4E-13 | -0.0252 (0.0215) | 0.242 | 51.4 | 2.265 |
| Hand grip strength (left) | All-cause dementia (FinnGen) | rs12906830 | 15:56963503 | C/T | 0.601 | 0.0108 (0.0015) | 9.3E-13 | -0.0308 (0.0199) | 0.122 | 51.0 | -2.841 |
| Hand grip strength (left) | All-cause dementia (FinnGen) | rs61389091 | 11:74427921 | T/C | 0.042 | 0.0262 (0.0037) | 2.4E-12 | -0.0459 (0.0482) | 0.341 | 49.1 | -1.754 |
| Hand grip strength (left) | All-cause dementia (FinnGen) | rs2273555 | 10:104127171 | A/G | 0.606 | 0.0106 (0.0015) | 2.7E-12 | 0.0283 (0.0195) | 0.147 | 48.9 | 2.664 |
| Hand grip strength (left) | All-cause dementia (FinnGen) | rs11121542 | 1:10393920 | A/G | 0.123 | -0.0158 (0.0023) | 3.0E-12 | 0.0280 (0.0312) | 0.369 | 48.7 | -1.777 |
| Hand grip strength (left) | All-cause dementia (FinnGen) | rs3814877 | 16:30042677 | T/G | 0.402 | 0.0105 (0.0015) | 3.4E-12 | -0.0274 (0.0198) | 0.166 | 48.5 | -2.600 |
| Hand grip strength (left) | All-cause dementia (FinnGen) | rs7963801 | 12:79685226 | C/T | 0.572 | -0.0104 (0.0015) | 4.6E-12 | -0.0059 (0.0195) | 0.761 | 47.9 | 0.565 |
| Hand grip strength (left) | All-cause dementia (FinnGen) | rs12889267 | 14:21542766 | G/A | 0.167 | -0.0137 (0.0020) | 4.7E-12 | 0.0130 (0.0293) | 0.656 | 47.8 | -0.946 |
| Hand grip strength (left) | All-cause dementia (FinnGen) | rs10176878 | 2:59952274 | C/T | 0.191 | -0.0129 (0.0019) | 8.6E-12 | 0.0088 (0.0277) | 0.750 | 46.6 | -0.680 |
| Hand grip strength (left) | All-cause dementia (FinnGen) | rs7970350 | 12:66360164 | T/C | 0.494 | -0.0101 (0.0015) | 8.6E-12 | -0.0319 (0.0191) | 0.096 | 46.6 | 3.148 |
| Hand grip strength (left) | All-cause dementia (FinnGen) | rs28542042 | 15:74213357 | T/C | 0.309 | 0.0110 (0.0016) | 1.2E-11 | -0.0240 (0.0215) | 0.264 | 46.0 | -2.180 |
| Hand grip strength (left) | All-cause dementia (FinnGen) | rs1641457 | 2:40421990 | G/T | 0.223 | 0.0120 (0.0018) | 1.4E-11 | 0.0049 (0.0210) | 0.817 | 45.6 | 0.407 |
| Hand grip strength (left) | All-cause dementia (FinnGen) | rs10403906 | 19:37376756 | A/G | 0.476 | -0.0100 (0.0015) | 1.5E-11 | 0.0020 (0.0194) | 0.916 | 45.5 | -0.199 |
| Hand grip strength (left) | All-cause dementia (FinnGen) | rs4575361 | 12:124410529 | T/A | 0.312 | -0.0108 (0.0016) | 1.6E-11 | -0.0063 (0.0211) | 0.767 | 45.5 | 0.583 |
| Hand grip strength (left) | All-cause dementia (FinnGen) | rs12763284 | 10:104508202 | G/A | 0.466 | 0.0100 (0.0015) | 1.7E-11 | -0.0023 (0.0192) | 0.904 | 45.3 | -0.230 |
| Hand grip strength (left) | All-cause dementia (FinnGen) | rs10097417 | 8:110361477 | G/A | 0.171 | -0.0132 (0.0020) | 1.9E-11 | -0.0297 (0.0280) | 0.287 | 45.1 | 2.245 |
| Hand grip strength (left) | All-cause dementia (FinnGen) | rs4363950 | 19:52221213 | G/A | 0.708 | 0.0110 (0.0016) | 1.9E-11 | 0.0362 (0.0233) | 0.120 | 45.1 | 3.297 |
| Hand grip strength (left) | All-cause dementia (FinnGen) | rs34030812 | 2:144248905 | C/T | 0.367 | -0.0102 (0.0015) | 4.1E-11 | 0.0102 (0.0197) | 0.604 | 43.6 | -1.003 |
| Hand grip strength (left) | All-cause dementia (FinnGen) | rs2532111 | 17:62017421 | G/A | 0.640 | 0.0103 (0.0016) | 4.1E-11 | 0.0033 (0.0196) | 0.866 | 43.6 | 0.321 |
| Hand grip strength (left) | All-cause dementia (FinnGen) | rs11769549 | 7:23122239 | A/T | 0.062 | 0.0205 (0.0031) | 4.5E-11 | -0.0276 (0.0433) | 0.524 | 43.4 | -1.347 |
| Hand grip strength (left) | All-cause dementia (FinnGen) | rs62253653 | 3:53013267 | G/A | 0.295 | 0.0107 (0.0016) | 5.5E-11 | -0.0145 (0.0204) | 0.476 | 43.0 | -1.355 |
| Hand grip strength (left) | All-cause dementia (FinnGen) | rs6680160 | 1:32072737 | G/A | 0.628 | 0.0101 (0.0015) | 6.0E-11 | -0.0002 (0.0200) | 0.993 | 42.8 | -0.020 |
| Hand grip strength (left) | All-cause dementia (FinnGen) | rs1486925 | 8:78827617 | C/T | 0.315 | -0.0105 (0.0016) | 6.3E-11 | -0.0122 (0.0214) | 0.568 | 42.7 | 1.165 |
| Hand grip strength (left) | All-cause dementia (FinnGen) | rs11243202 | 6:7719065 | C/T | 0.486 | 0.0097 (0.0015) | 7.0E-11 | 0.0058 (0.0192) | 0.764 | 42.5 | 0.597 |
| Hand grip strength (left) | All-cause dementia (FinnGen) | rs12914702 | 15:96887277 | A/G | 0.300 | 0.0110 (0.0017) | 9.3E-11 | -0.0145 (0.0206) | 0.482 | 42.0 | -1.324 |
| Hand grip strength (left) | All-cause dementia (FinnGen) | rs4737446 | 8:57665019 | T/G | 0.695 | 0.0104 (0.0016) | 1.2E-10 | -0.0326 (0.0205) | 0.112 | 41.4 | -3.129 |
| Hand grip strength (left) | All-cause dementia (FinnGen) | rs16870531 | 7:120660682 | T/C | 0.238 | 0.0112 (0.0017) | 1.3E-10 | -0.0059 (0.0221) | 0.789 | 41.3 | -0.527 |
| Hand grip strength (left) | All-cause dementia (FinnGen) | rs2431112 | 5:103931707 | A/G | 0.441 | -0.0096 (0.0015) | 1.4E-10 | 0.0146 (0.0196) | 0.455 | 41.1 | -1.523 |
| Hand grip strength (left) | All-cause dementia (FinnGen) | rs56338231 | 4:30867393 | G/A | 0.258 | -0.0108 (0.0017) | 1.7E-10 | -0.0011 (0.0228) | 0.960 | 40.8 | 0.101 |
| Hand grip strength (left) | All-cause dementia (FinnGen) | rs11002322 | 10:79649653 | T/G | 0.340 | -0.0100 (0.0016) | 2.1E-10 | 0.0214 (0.0212) | 0.314 | 40.4 | -2.142 |
| Hand grip strength (left) | All-cause dementia (FinnGen) | rs217181 | 16:72114002 | T/C | 0.193 | 0.0120 (0.0019) | 2.1E-10 | -0.0198 (0.0235) | 0.399 | 40.4 | -1.656 |
| Hand grip strength (left) | All-cause dementia (FinnGen) | rs8108461 | 19:2186757 | C/T | 0.573 | 0.0095 (0.0015) | 2.6E-10 | -0.0036 (0.0193) | 0.854 | 40.0 | -0.378 |
| Hand grip strength (left) | All-cause dementia (FinnGen) | rs41271299 | 6:19839415 | T/C | 0.051 | 0.0212 (0.0034) | 3.0E-10 | 0.0524 (0.0757) | 0.489 | 39.7 | 2.473 |
| Hand grip strength (left) | All-cause dementia (FinnGen) | rs34587452 | 4:1009900 | C/G | 0.215 | -0.0114 (0.0018) | 3.2E-10 | 0.0663 (0.0240) | 0.006 | 39.5 | -5.834 |
| Hand grip strength (left) | All-cause dementia (FinnGen) | rs4121165 | 1:78276977 | A/G | 0.211 | -0.0114 (0.0018) | 3.4E-10 | 0.0346 (0.0254) | 0.173 | 39.4 | -3.031 |
| Hand grip strength (left) | All-cause dementia (FinnGen) | rs7575451 | 2:152352843 | G/C | 0.650 | -0.0097 (0.0016) | 3.8E-10 | -0.0005 (0.0205) | 0.979 | 39.2 | 0.051 |
| Hand grip strength (left) | All-cause dementia (FinnGen) | rs6802071 | 3:38574237 | T/C | 0.435 | -0.0094 (0.0015) | 3.8E-10 | 0.0229 (0.0193) | 0.236 | 39.2 | -2.435 |
| Hand grip strength (left) | All-cause dementia (FinnGen) | rs76895963 | 12:4384844 | G/T | 0.021 | 0.0360 (0.0057) | 3.9E-10 | -0.0735 (0.0577) | 0.203 | 39.1 | -2.044 |
| Hand grip strength (left) | All-cause dementia (FinnGen) | rs34845616 | 11:133792644 | A/G | 0.246 | 0.0108 (0.0017) | 4.0E-10 | -0.0168 (0.0204) | 0.411 | 39.1 | -1.550 |
| Hand grip strength (left) | All-cause dementia (FinnGen) | rs4498020 | 3:13810820 | A/C | 0.724 | -0.0104 (0.0017) | 4.3E-10 | -0.0247 (0.0208) | 0.234 | 39.0 | 2.370 |
| Hand grip strength (left) | All-cause dementia (FinnGen) | rs9611273 | 22:40534466 | T/C | 0.253 | 0.0108 (0.0017) | 4.5E-10 | 0.0003 (0.0218) | 0.990 | 38.9 | 0.028 |
| Hand grip strength (left) | All-cause dementia (FinnGen) | rs10821939 | 10:63751748 | A/G | 0.573 | -0.0094 (0.0015) | 4.9E-10 | 0.0019 (0.0196) | 0.922 | 38.7 | -0.203 |
| Hand grip strength (left) | All-cause dementia (FinnGen) | rs1434095 | 2:179254330 | C/T | 0.875 | 0.0140 (0.0023) | 5.3E-10 | -0.0203 (0.0324) | 0.531 | 38.6 | -1.447 |
| Hand grip strength (left) | All-cause dementia (FinnGen) | rs35609019 | 4:7847892 | C/G | 0.398 | 0.0095 (0.0015) | 7.7E-10 | 0.0059 (0.0195) | 0.760 | 37.8 | 0.623 |
| Hand grip strength (left) | All-cause dementia (FinnGen) | rs10831903 | 11:12758660 | T/C | 0.423 | 0.0093 (0.0015) | 8.1E-10 | 0.0204 (0.0204) | 0.316 | 37.8 | 2.197 |
| Hand grip strength (left) | All-cause dementia (FinnGen) | rs75497896 | 4:119636703 | C/T | 0.051 | -0.0207 (0.0034) | 9.1E-10 | 0.0276 (0.0403) | 0.492 | 37.5 | -1.335 |
| Hand grip strength (left) | All-cause dementia (FinnGen) | rs9371881 | 6:155638213 | A/G | 0.359 | 0.0095 (0.0015) | 9.6E-10 | -0.0329 (0.0218) | 0.132 | 37.4 | -3.471 |
| Hand grip strength (left) | All-cause dementia (FinnGen) | rs1981612 | 2:199235664 | A/C | 0.456 | 0.0092 (0.0015) | 1.0E-09 | -0.0215 (0.0193) | 0.265 | 37.3 | -2.330 |
| Hand grip strength (left) | All-cause dementia (FinnGen) | rs10205394 | 2:218150948 | C/G | 0.201 | -0.0113 (0.0019) | 1.1E-09 | -0.0040 (0.0216) | 0.853 | 37.2 | 0.353 |
| Hand grip strength (left) | All-cause dementia (FinnGen) | rs4677601 | 3:71368790 | G/A | 0.510 | 0.0091 (0.0015) | 1.1E-09 | 0.0303 (0.0192) | 0.113 | 37.2 | 3.345 |
| Hand grip strength (left) | All-cause dementia (FinnGen) | rs73307079 | 7:19020024 | C/T | 0.211 | 0.0111 (0.0018) | 1.4E-09 | 0.0393 (0.0219) | 0.072 | 36.6 | 3.540 |
| Hand grip strength (left) | All-cause dementia (FinnGen) | rs7197751 | 16:84940033 | T/G | 0.363 | -0.0095 (0.0016) | 1.4E-09 | 0.0119 (0.0208) | 0.568 | 36.7 | -1.258 |
| Hand grip strength (left) | All-cause dementia (FinnGen) | rs821100 | 8:89448877 | G/A | 0.266 | -0.0102 (0.0017) | 1.5E-09 | 0.0063 (0.0218) | 0.772 | 36.6 | -0.618 |
| Hand grip strength (left) | All-cause dementia (FinnGen) | rs723588 | 6:81056634 | C/T | 0.143 | 0.0128 (0.0021) | 1.6E-09 | 0.0172 (0.0258) | 0.504 | 36.4 | 1.345 |
| Hand grip strength (left) | All-cause dementia (FinnGen) | rs7148603 | 14:36683779 | A/G | 0.359 | 0.0096 (0.0016) | 1.6E-09 | 0.0044 (0.0206) | 0.830 | 36.4 | 0.460 |
| Hand grip strength (left) | All-cause dementia (FinnGen) | rs10988217 | 9:131888116 | G/A | 0.604 | -0.0092 (0.0015) | 1.7E-09 | 0.0157 (0.0195) | 0.422 | 36.2 | -1.705 |
| Hand grip strength (left) | All-cause dementia (FinnGen) | rs7176095 | 15:74886411 | G/A | 0.128 | -0.0134 (0.0022) | 1.7E-09 | 0.0034 (0.0289) | 0.905 | 36.3 | -0.254 |
| Hand grip strength (left) | All-cause dementia (FinnGen) | rs10144445 | 14:39695362 | G/C | 0.350 | -0.0094 (0.0016) | 1.9E-09 | 0.0145 (0.0200) | 0.469 | 36.1 | -1.548 |
| Hand grip strength (left) | All-cause dementia (FinnGen) | rs181766 | 16:14394878 | C/T | 0.322 | 0.0096 (0.0016) | 1.9E-09 | 0.0054 (0.0201) | 0.787 | 36.1 | 0.561 |
| Hand grip strength (left) | All-cause dementia (FinnGen) | rs2587505 | 17:77784268 | C/T | 0.420 | -0.0090 (0.0015) | 2.3E-09 | 0.0219 (0.0204) | 0.282 | 35.7 | -2.432 |
| Hand grip strength (left) | All-cause dementia (FinnGen) | rs12673062 | 7:4710677 | A/G | 0.216 | -0.0108 (0.0018) | 2.6E-09 | -0.0350 (0.0216) | 0.104 | 35.5 | 3.250 |
| Hand grip strength (left) | All-cause dementia (FinnGen) | rs6006984 | 22:45714937 | C/T | 0.278 | 0.0098 (0.0017) | 2.6E-09 | -0.0015 (0.0210) | 0.942 | 35.5 | -0.152 |
| Hand grip strength (left) | All-cause dementia (FinnGen) | rs6882168 | 5:39402647 | T/C | 0.337 | -0.0094 (0.0016) | 2.8E-09 | -0.0099 (0.0195) | 0.613 | 35.3 | 1.058 |
| Hand grip strength (left) | All-cause dementia (FinnGen) | rs12528131 | 6:105389104 | G/A | 0.488 | -0.0088 (0.0015) | 2.8E-09 | -0.0182 (0.0192) | 0.344 | 35.3 | 2.062 |
| Hand grip strength (left) | All-cause dementia (FinnGen) | rs9371201 | 6:150145001 | T/C | 0.335 | -0.0093 (0.0016) | 3.0E-09 | -0.0062 (0.0198) | 0.755 | 35.2 | 0.664 |
| Hand grip strength (left) | All-cause dementia (FinnGen) | rs6962338 | 7:69160985 | G/A | 0.044 | -0.0214 (0.0036) | 3.2E-09 | -0.0400 (0.0517) | 0.440 | 35.0 | 1.868 |
| Hand grip strength (left) | All-cause dementia (FinnGen) | rs77485342 | 6:30842866 | T/C | 0.018 | 0.0330 (0.0056) | 3.5E-09 | 0.0629 (0.0605) | 0.298 | 34.9 | 1.908 |
| Hand grip strength (left) | All-cause dementia (FinnGen) | rs4930236 | 11:68414000 | A/C | 0.836 | 0.0119 (0.0020) | 3.8E-09 | -0.0277 (0.0322) | 0.389 | 34.7 | -2.327 |
| Hand grip strength (left) | All-cause dementia (FinnGen) | rs13106087 | 4:145566864 | C/T | 0.830 | 0.0116 (0.0020) | 3.9E-09 | 0.0139 (0.0257) | 0.588 | 34.6 | 1.196 |
| Hand grip strength (left) | All-cause dementia (FinnGen) | rs17630248 | 2:201137782 | C/T | 0.348 | -0.0092 (0.0016) | 4.1E-09 | -0.0173 (0.0202) | 0.392 | 34.6 | 1.884 |
| Hand grip strength (left) | All-cause dementia (FinnGen) | rs4335354 | 1:88899964 | A/C | 0.316 | -0.0094 (0.0016) | 4.6E-09 | 0.0027 (0.0212) | 0.900 | 34.4 | -0.287 |
| Hand grip strength (left) | All-cause dementia (FinnGen) | rs9866627 | 3:135522715 | A/C | 0.084 | -0.0156 (0.0027) | 5.7E-09 | 0.0625 (0.0288) | 0.030 | 33.9 | -4.007 |
| Hand grip strength (left) | All-cause dementia (FinnGen) | rs35236379 | 10:5727292 | T/G | 0.142 | 0.0124 (0.0021) | 5.8E-09 | -0.0127 (0.0252) | 0.614 | 33.9 | -1.028 |
| Hand grip strength (left) | All-cause dementia (FinnGen) | rs1924721 | 1:190972986 | T/G | 0.117 | 0.0134 (0.0023) | 6.2E-09 | -0.0131 (0.0256) | 0.608 | 33.8 | -0.974 |
| Hand grip strength (left) | All-cause dementia (FinnGen) | rs997850 | 4:154838434 | C/G | 0.605 | -0.0089 (0.0015) | 6.3E-09 | -0.0053 (0.0192) | 0.783 | 33.7 | 0.597 |
| Hand grip strength (left) | All-cause dementia (FinnGen) | rs2038760 | 6:2680732 | T/C | 0.171 | -0.0115 (0.0020) | 6.6E-09 | -0.0216 (0.0260) | 0.405 | 33.7 | 1.874 |
| Hand grip strength (left) | All-cause dementia (FinnGen) | rs2359239 | 14:75326771 | T/C | 0.392 | -0.0088 (0.0015) | 6.9E-09 | 0.0197 (0.0192) | 0.305 | 33.6 | -2.235 |
| Hand grip strength (left) | All-cause dementia (FinnGen) | rs11204664 | 1:150531380 | C/T | 0.579 | -0.0087 (0.0015) | 8.1E-09 | -0.0019 (0.0205) | 0.926 | 33.2 | 0.220 |
| Hand grip strength (left) | All-cause dementia (FinnGen) | rs13227429 | 7:140560023 | C/T | 0.561 | -0.0086 (0.0015) | 9.8E-09 | -0.0239 (0.0192) | 0.214 | 32.9 | 2.783 |
| Hand grip strength (left) | All-cause dementia (FinnGen) | rs143002906 | 18:12992162 | T/C | 0.028 | 0.0262 (0.0046) | 9.8E-09 | -0.0531 (0.0432) | 0.218 | 32.9 | -2.025 |
| Hand grip strength (left) | All-cause dementia (FinnGen) | rs61286123 | 2:60205600 | C/T | 0.228 | -0.0101 (0.0018) | 1.2E-08 | 0.0043 (0.0225) | 0.849 | 32.5 | -0.426 |
| Hand grip strength (left) | All-cause dementia (FinnGen) | rs9944324 | 16:80929342 | G/A | 0.457 | -0.0086 (0.0015) | 1.2E-08 | 0.0197 (0.0192) | 0.306 | 32.4 | -2.303 |
| Hand grip strength (left) | All-cause dementia (FinnGen) | rs4739739 | 8:81304576 | G/A | 0.415 | -0.0085 (0.0015) | 1.4E-08 | 0.0192 (0.0198) | 0.333 | 32.2 | -2.250 |
| Hand grip strength (left) | All-cause dementia (FinnGen) | rs113918482 | 5:161289270 | G/A | 0.223 | -0.0101 (0.0018) | 1.5E-08 | -0.0036 (0.0207) | 0.861 | 32.0 | 0.356 |
| Hand grip strength (left) | All-cause dementia (FinnGen) | rs55681913 | 5:42687629 | C/T | 0.106 | 0.0138 (0.0024) | 1.6E-08 | 0.0017 (0.0293) | 0.954 | 32.0 | 0.123 |
| Hand grip strength (left) | All-cause dementia (FinnGen) | rs11003014 | 10:81231387 | G/A | 0.161 | 0.0114 (0.0020) | 1.6E-08 | -0.0225 (0.0232) | 0.333 | 32.0 | -1.968 |
| Hand grip strength (left) | All-cause dementia (FinnGen) | rs10860864 | 12:102822862 | T/C | 0.181 | 0.0108 (0.0019) | 1.9E-08 | -0.0207 (0.0240) | 0.388 | 31.6 | -1.911 |
| Hand grip strength (left) | All-cause dementia (FinnGen) | rs4398863 | 8:135695110 | C/G | 0.737 | -0.0095 (0.0017) | 2.0E-08 | -0.0098 (0.0231) | 0.670 | 31.5 | 1.037 |
| Hand grip strength (left) | All-cause dementia (FinnGen) | rs116922558 | 9:118802375 | G/A | 0.040 | -0.0216 (0.0039) | 2.1E-08 | 0.0872 (0.0459) | 0.057 | 31.4 | -4.040 |
| Hand grip strength (left) | All-cause dementia (FinnGen) | rs1884447 | 1:185021410 | A/G | 0.401 | 0.0085 (0.0015) | 2.3E-08 | 0.0068 (0.0194) | 0.727 | 31.2 | 0.804 |
| Hand grip strength (left) | All-cause dementia (FinnGen) | rs59116179 | 8:22603454 | T/C | 0.617 | 0.0086 (0.0015) | 2.4E-08 | 0.0073 (0.0199) | 0.714 | 31.1 | 0.851 |
| Hand grip strength (left) | All-cause dementia (FinnGen) | rs11168357 | 12:48412138 | A/G | 0.246 | -0.0096 (0.0017) | 2.5E-08 | 0.0176 (0.0263) | 0.502 | 31.1 | -1.828 |
| Hand grip strength (left) | All-cause dementia (FinnGen) | rs116409670 | 5:37327472 | T/C | 0.080 | -0.0152 (0.0027) | 2.6E-08 | -0.0391 (0.0520) | 0.451 | 31.0 | 2.565 |
| Hand grip strength (left) | All-cause dementia (FinnGen) | rs12533765 | 7:127699186 | G/A | 0.280 | -0.0092 (0.0017) | 2.6E-08 | 0.0113 (0.0251) | 0.652 | 31.0 | -1.228 |
| Hand grip strength (left) | All-cause dementia (FinnGen) | rs62081464 | 18:35142133 | T/C | 0.227 | -0.0099 (0.0018) | 2.8E-08 | -0.0237 (0.0235) | 0.314 | 30.8 | 2.398 |
| Hand grip strength (left) | All-cause dementia (FinnGen) | rs4811040 | 20:48968438 | G/C | 0.277 | -0.0092 (0.0017) | 3.0E-08 | -0.0008 (0.0213) | 0.971 | 30.7 | 0.086 |
| Hand grip strength (left) | All-cause dementia (FinnGen) | rs7516571 | 1:40733658 | G/A | 0.259 | 0.0094 (0.0017) | 3.1E-08 | 0.0093 (0.0209) | 0.658 | 30.7 | 0.992 |
| Hand grip strength (left) | All-cause dementia (FinnGen) | rs2800789 | 1:164578242 | C/A | 0.480 | 0.0083 (0.0015) | 3.1E-08 | -0.0061 (0.0192) | 0.751 | 30.6 | -0.738 |
| Hand grip strength (left) | All-cause dementia (FinnGen) | rs4962700 | 10:126479989 | G/C | 0.302 | 0.0090 (0.0016) | 3.2E-08 | -0.0137 (0.0196) | 0.485 | 30.6 | -1.515 |
| Hand grip strength (left) | All-cause dementia (FinnGen) | rs13091492 | 3:81891476 | G/A | 0.373 | -0.0085 (0.0015) | 3.3E-08 | -0.0005 (0.0191) | 0.979 | 30.6 | 0.059 |
| Hand grip strength (left) | All-cause dementia (FinnGen) | rs34722008 | 4:38659594 | A/G | 0.353 | 0.0086 (0.0016) | 3.3E-08 | 0.0181 (0.0197) | 0.360 | 30.5 | 2.108 |
| Hand grip strength (left) | All-cause dementia (FinnGen) | rs2850379 | 4:102917419 | A/C | 0.432 | -0.0083 (0.0015) | 3.3E-08 | 0.0187 (0.0191) | 0.327 | 30.5 | -2.256 |
| Hand grip strength (left) | All-cause dementia (FinnGen) | rs2974438 | 5:168250903 | A/G | 0.211 | -0.0101 (0.0018) | 3.3E-08 | 0.0024 (0.0243) | 0.921 | 30.5 | -0.238 |
| Hand grip strength (left) | All-cause dementia (FinnGen) | rs58670122 | 1:22492613 | G/A | 0.143 | -0.0118 (0.0021) | 3.6E-08 | 0.0514 (0.0316) | 0.103 | 30.4 | -4.350 |
| Hand grip strength (left) | All-cause dementia (FinnGen) | rs16910750 | 9:99084471 | C/G | 0.160 | 0.0112 (0.0020) | 3.6E-08 | -0.0586 (0.0283) | 0.038 | 30.4 | -5.209 |
| Hand grip strength (left) | All-cause dementia (FinnGen) | rs17282763 | 7:82520166 | C/T | 0.296 | 0.0089 (0.0016) | 4.4E-08 | -0.0056 (0.0208) | 0.788 | 30.0 | -0.626 |
| Hand grip strength (left) | All-cause dementia (FinnGen) | rs7026798 | 9:16427378 | C/T | 0.432 | 0.0082 (0.0015) | 4.6E-08 | 0.0352 (0.0192) | 0.067 | 29.9 | 4.274 |
| Hand grip strength (left) | All-cause dementia (FinnGen) | rs10934857 | 3:128199662 | A/G | 0.259 | 0.0093 (0.0017) | 4.8E-08 | 0.0138 (0.0232) | 0.551 | 29.8 | 1.486 |
| Hand grip strength (left) | All-cause dementia (FinnGen) | rs2789514 | 9:129833029 | A/G | 0.868 | 0.0121 (0.0022) | 4.9E-08 | 0.1207 (0.0428) | 0.005 | 29.8 | 10.013 |
| Hand grip strength (left) | Vascular dementia (FinnGen) | rs143384 | 20:34025756 | G/A | 0.404 | 0.0209 (0.0015) | 1.5E-43 | 0.0484 (0.0498) | 0.331 | 191.5 | 2.314 |
| Hand grip strength (left) | Vascular dementia (FinnGen) | rs12316046 | 12:15054415 | G/A | 0.378 | -0.0174 (0.0015) | 5.0E-30 | -0.0250 (0.0550) | 0.649 | 129.6 | 1.435 |
| Hand grip strength (left) | Vascular dementia (FinnGen) | rs10846071 | 12:15016236 | T/C | 0.394 | -0.0166 (0.0015) | 5.3E-28 | -0.0019 (0.0533) | 0.972 | 120.4 | 0.114 |
| Hand grip strength (left) | Vascular dementia (FinnGen) | rs1556659 | 10:130834698 | T/C | 0.382 | 0.0163 (0.0015) | 2.5E-26 | -0.0321 (0.0540) | 0.552 | 112.7 | -1.971 |
| Hand grip strength (left) | Vascular dementia (FinnGen) | rs13146142 | 4:17931318 | C/T | 0.159 | -0.0202 (0.0020) | 2.3E-23 | -0.0613 (0.0837) | 0.464 | 99.2 | 3.034 |
| Hand grip strength (left) | Vascular dementia (FinnGen) | rs3118903 | 13:51099577 | A/G | 0.220 | -0.0174 (0.0018) | 2.7E-22 | 0.0450 (0.0560) | 0.421 | 94.3 | -2.579 |
| Hand grip strength (left) | Vascular dementia (FinnGen) | rs2871865 | 15:99194896 | G/C | 0.116 | -0.0218 (0.0023) | 5.0E-21 | 0.2519 (0.1010) | 0.013 | 88.5 | -11.556 |
| Hand grip strength (left) | Vascular dementia (FinnGen) | rs1044299 | 1:176811873 | T/C | 0.546 | 0.0140 (0.0015) | 6.6E-21 | -0.0204 (0.0492) | 0.679 | 88.0 | -1.456 |
| Hand grip strength (left) | Vascular dementia (FinnGen) | rs12790261 | 11:66988048 | A/C | 0.082 | -0.0252 (0.0027) | 1.2E-20 | 0.0920 (0.1125) | 0.414 | 86.8 | -3.652 |
| Hand grip strength (left) | Vascular dementia (FinnGen) | rs6977081 | 7:150542515 | T/G | 0.334 | 0.0148 (0.0016) | 1.4E-20 | 0.0003 (0.0513) | 0.996 | 86.5 | 0.020 |
| Hand grip strength (left) | Vascular dementia (FinnGen) | rs13107325 | 4:103188709 | T/C | 0.075 | -0.0262 (0.0028) | 1.8E-20 | 0.1048 (0.2098) | 0.617 | 86.0 | -4.007 |
| Hand grip strength (left) | Vascular dementia (FinnGen) | rs4713506 | 6:32113980 | A/G | 0.256 | -0.0157 (0.0017) | 2.0E-20 | -0.0018 (0.0523) | 0.973 | 85.8 | 0.114 |
| Hand grip strength (left) | Vascular dementia (FinnGen) | rs3819121 | 2:135622860 | C/T | 0.369 | 0.0141 (0.0015) | 2.9E-20 | 0.0121 (0.0503) | 0.810 | 85.0 | 0.858 |
| Hand grip strength (left) | Vascular dementia (FinnGen) | rs10788958 | 1:54040670 | G/C | 0.645 | 0.0142 (0.0016) | 1.0E-19 | -0.0299 (0.0563) | 0.596 | 82.5 | -2.106 |
| Hand grip strength (left) | Vascular dementia (FinnGen) | rs7761063 | 6:130379973 | C/A | 0.673 | -0.0141 (0.0016) | 5.1E-19 | 0.0241 (0.0518) | 0.642 | 79.4 | -1.711 |
| Hand grip strength (left) | Vascular dementia (FinnGen) | rs4308051 | 18:20735461 | G/T | 0.789 | 0.0160 (0.0018) | 1.4E-18 | 0.0236 (0.0583) | 0.686 | 77.3 | 1.476 |
| Hand grip strength (left) | Vascular dementia (FinnGen) | rs7571789 | 2:70714793 | C/T | 0.523 | 0.0130 (0.0015) | 3.0E-18 | 0.0175 (0.0496) | 0.724 | 75.9 | 1.351 |
| Hand grip strength (left) | Vascular dementia (FinnGen) | rs755547 | 17:43011908 | A/G | 0.189 | 0.0165 (0.0019) | 3.2E-18 | -0.0907 (0.0714) | 0.204 | 75.7 | -5.492 |
| Hand grip strength (left) | Vascular dementia (FinnGen) | rs11125803 | 2:25052177 | T/C | 0.741 | 0.0143 (0.0017) | 3.8E-17 | -0.0715 (0.0576) | 0.214 | 70.9 | -5.005 |
| Hand grip strength (left) | Vascular dementia (FinnGen) | rs999493 | 17:46625519 | A/G | 0.622 | 0.0129 (0.0015) | 6.2E-17 | 0.0278 (0.0509) | 0.585 | 69.9 | 2.160 |
| Hand grip strength (left) | Vascular dementia (FinnGen) | rs635538 | 18:53273614 | A/G | 0.914 | -0.0217 (0.0027) | 3.8E-16 | -0.1039 (0.0806) | 0.197 | 66.4 | 4.798 |
| Hand grip strength (left) | Vascular dementia (FinnGen) | rs2871960 | 3:141121814 | C/A | 0.445 | 0.0121 (0.0015) | 5.5E-16 | 0.0362 (0.0496) | 0.465 | 65.6 | 2.995 |
| Hand grip strength (left) | Vascular dementia (FinnGen) | rs113434679 | 17:44126765 | A/C | 0.200 | -0.0149 (0.0019) | 1.9E-15 | -0.2409 (0.0921) | 0.009 | 63.2 | 16.151 |
| Hand grip strength (left) | Vascular dementia (FinnGen) | rs7196917 | 16:69896527 | G/A | 0.430 | -0.0117 (0.0015) | 5.4E-15 | 0.0618 (0.0507) | 0.223 | 61.1 | -5.268 |
| Hand grip strength (left) | Vascular dementia (FinnGen) | rs17466480 | 15:77390870 | G/A | 0.387 | -0.0118 (0.0015) | 9.8E-15 | -0.0148 (0.0508) | 0.771 | 59.9 | 1.252 |
| Hand grip strength (left) | Vascular dementia (FinnGen) | rs7124681 | 11:47529947 | A/C | 0.408 | -0.0117 (0.0015) | 1.0E-14 | -0.0235 (0.0515) | 0.649 | 59.9 | 2.016 |
| Hand grip strength (left) | Vascular dementia (FinnGen) | rs11076004 | 16:53913930 | A/G | 0.418 | -0.0115 (0.0015) | 2.1E-14 | 0.0800 (0.0491) | 0.103 | 58.4 | -6.935 |
| Hand grip strength (left) | Vascular dementia (FinnGen) | rs823130 | 1:205714372 | T/C | 0.433 | -0.0113 (0.0015) | 4.4E-14 | 0.0156 (0.0496) | 0.754 | 57.0 | -1.377 |
| Hand grip strength (left) | Vascular dementia (FinnGen) | rs12473732 | 2:44118428 | T/C | 0.487 | 0.0110 (0.0015) | 1.4E-13 | -0.0361 (0.0492) | 0.463 | 54.7 | -3.286 |
| Hand grip strength (left) | Vascular dementia (FinnGen) | rs13337177 | 16:2175323 | T/G | 0.181 | -0.0143 (0.0019) | 1.6E-13 | -0.0432 (0.0647) | 0.505 | 54.4 | 3.025 |
| Hand grip strength (left) | Vascular dementia (FinnGen) | rs2631360 | 5:131707429 | A/G | 0.519 | -0.0109 (0.0015) | 1.9E-13 | -0.0083 (0.0517) | 0.872 | 54.1 | 0.760 |
| Hand grip strength (left) | Vascular dementia (FinnGen) | rs7856625 | 9:119345083 | T/C | 0.610 | -0.0111 (0.0015) | 2.5E-13 | -0.0025 (0.0514) | 0.962 | 53.5 | 0.225 |
| Hand grip strength (left) | Vascular dementia (FinnGen) | rs150330307 | 1:160160801 | C/T | 0.032 | -0.0308 (0.0042) | 2.9E-13 | 0.0274 (0.1752) | 0.876 | 53.3 | -0.890 |
| Hand grip strength (left) | Vascular dementia (FinnGen) | rs1551042 | 3:85630551 | C/A | 0.647 | -0.0111 (0.0016) | 7.4E-13 | -0.0460 (0.0551) | 0.404 | 51.4 | 4.134 |
| Hand grip strength (left) | Vascular dementia (FinnGen) | rs12906830 | 15:56963503 | C/T | 0.601 | 0.0108 (0.0015) | 9.3E-13 | -0.0498 (0.0513) | 0.332 | 51.0 | -4.594 |
| Hand grip strength (left) | Vascular dementia (FinnGen) | rs61389091 | 11:74427921 | T/C | 0.042 | 0.0262 (0.0037) | 2.4E-12 | -0.0163 (0.1231) | 0.895 | 49.1 | -0.623 |
| Hand grip strength (left) | Vascular dementia (FinnGen) | rs2273555 | 10:104127171 | A/G | 0.606 | 0.0106 (0.0015) | 2.7E-12 | 0.1200 (0.0502) | 0.017 | 48.9 | 11.296 |
| Hand grip strength (left) | Vascular dementia (FinnGen) | rs11121542 | 1:10393920 | A/G | 0.123 | -0.0158 (0.0023) | 3.0E-12 | 0.0541 (0.0807) | 0.503 | 48.7 | -3.433 |
| Hand grip strength (left) | Vascular dementia (FinnGen) | rs3814877 | 16:30042677 | T/G | 0.402 | 0.0105 (0.0015) | 3.4E-12 | 0.0260 (0.0510) | 0.610 | 48.5 | 2.467 |
| Hand grip strength (left) | Vascular dementia (FinnGen) | rs7963801 | 12:79685226 | C/T | 0.572 | -0.0104 (0.0015) | 4.6E-12 | -0.0422 (0.0502) | 0.400 | 47.9 | 4.044 |
| Hand grip strength (left) | Vascular dementia (FinnGen) | rs12889267 | 14:21542766 | G/A | 0.167 | -0.0137 (0.0020) | 4.7E-12 | -0.0756 (0.0751) | 0.314 | 47.8 | 5.502 |
| Hand grip strength (left) | Vascular dementia (FinnGen) | rs10176878 | 2:59952274 | C/T | 0.191 | -0.0129 (0.0019) | 8.6E-12 | 0.1050 (0.0709) | 0.139 | 46.6 | -8.109 |
| Hand grip strength (left) | Vascular dementia (FinnGen) | rs7970350 | 12:66360164 | T/C | 0.494 | -0.0101 (0.0015) | 8.6E-12 | -0.0401 (0.0492) | 0.415 | 46.6 | 3.957 |
| Hand grip strength (left) | Vascular dementia (FinnGen) | rs28542042 | 15:74213357 | T/C | 0.309 | 0.0110 (0.0016) | 1.2E-11 | -0.0496 (0.0551) | 0.367 | 46.0 | -4.505 |
| Hand grip strength (left) | Vascular dementia (FinnGen) | rs1641457 | 2:40421990 | G/T | 0.223 | 0.0120 (0.0018) | 1.4E-11 | -0.0324 (0.0542) | 0.550 | 45.6 | -2.690 |
| Hand grip strength (left) | Vascular dementia (FinnGen) | rs10403906 | 19:37376756 | A/G | 0.476 | -0.0100 (0.0015) | 1.5E-11 | -0.0565 (0.0498) | 0.256 | 45.5 | 5.632 |
| Hand grip strength (left) | Vascular dementia (FinnGen) | rs4575361 | 12:124410529 | T/A | 0.312 | -0.0108 (0.0016) | 1.6E-11 | -0.0167 (0.0546) | 0.760 | 45.5 | 1.546 |
| Hand grip strength (left) | Vascular dementia (FinnGen) | rs12763284 | 10:104508202 | G/A | 0.466 | 0.0100 (0.0015) | 1.7E-11 | 0.0336 (0.0495) | 0.497 | 45.3 | 3.358 |
| Hand grip strength (left) | Vascular dementia (FinnGen) | rs10097417 | 8:110361477 | G/A | 0.171 | -0.0132 (0.0020) | 1.9E-11 | -0.0397 (0.0716) | 0.579 | 45.1 | 3.001 |
| Hand grip strength (left) | Vascular dementia (FinnGen) | rs4363950 | 19:52221213 | G/A | 0.708 | 0.0110 (0.0016) | 1.9E-11 | -0.0555 (0.0595) | 0.351 | 45.1 | -5.056 |
| Hand grip strength (left) | Vascular dementia (FinnGen) | rs34030812 | 2:144248905 | C/T | 0.367 | -0.0102 (0.0015) | 4.1E-11 | 0.0078 (0.0505) | 0.877 | 43.6 | -0.767 |
| Hand grip strength (left) | Vascular dementia (FinnGen) | rs2532111 | 17:62017421 | G/A | 0.640 | 0.0103 (0.0016) | 4.1E-11 | -0.0842 (0.0505) | 0.095 | 43.6 | -8.187 |
| Hand grip strength (left) | Vascular dementia (FinnGen) | rs11769549 | 7:23122239 | A/T | 0.062 | 0.0205 (0.0031) | 4.5E-11 | -0.2053 (0.1118) | 0.066 | 43.4 | -10.017 |
| Hand grip strength (left) | Vascular dementia (FinnGen) | rs62253653 | 3:53013267 | G/A | 0.295 | 0.0107 (0.0016) | 5.5E-11 | 0.0514 (0.0522) | 0.324 | 43.0 | 4.804 |
| Hand grip strength (left) | Vascular dementia (FinnGen) | rs6680160 | 1:32072737 | G/A | 0.628 | 0.0101 (0.0015) | 6.0E-11 | 0.0178 (0.0515) | 0.729 | 42.8 | 1.768 |
| Hand grip strength (left) | Vascular dementia (FinnGen) | rs1486925 | 8:78827617 | C/T | 0.315 | -0.0105 (0.0016) | 6.3E-11 | -0.0386 (0.0546) | 0.480 | 42.7 | 3.686 |
| Hand grip strength (left) | Vascular dementia (FinnGen) | rs11243202 | 6:7719065 | C/T | 0.486 | 0.0097 (0.0015) | 7.0E-11 | 0.0483 (0.0495) | 0.329 | 42.5 | 4.968 |
| Hand grip strength (left) | Vascular dementia (FinnGen) | rs12914702 | 15:96887277 | A/G | 0.300 | 0.0110 (0.0017) | 9.3E-11 | -0.0320 (0.0531) | 0.546 | 42.0 | -2.922 |
| Hand grip strength (left) | Vascular dementia (FinnGen) | rs4737446 | 8:57665019 | T/G | 0.695 | 0.0104 (0.0016) | 1.2E-10 | 0.0260 (0.0525) | 0.621 | 41.4 | 2.496 |
| Hand grip strength (left) | Vascular dementia (FinnGen) | rs16870531 | 7:120660682 | T/C | 0.238 | 0.0112 (0.0017) | 1.3E-10 | -0.0074 (0.0567) | 0.897 | 41.3 | -0.661 |
| Hand grip strength (left) | Vascular dementia (FinnGen) | rs2431112 | 5:103931707 | A/G | 0.441 | -0.0096 (0.0015) | 1.4E-10 | -0.0345 (0.0505) | 0.494 | 41.1 | 3.599 |
| Hand grip strength (left) | Vascular dementia (FinnGen) | rs56338231 | 4:30867393 | G/A | 0.258 | -0.0108 (0.0017) | 1.7E-10 | 0.1162 (0.0586) | 0.047 | 40.8 | -10.713 |
| Hand grip strength (left) | Vascular dementia (FinnGen) | rs11002322 | 10:79649653 | T/G | 0.340 | -0.0100 (0.0016) | 2.1E-10 | 0.0504 (0.0545) | 0.355 | 40.4 | -5.045 |
| Hand grip strength (left) | Vascular dementia (FinnGen) | rs217181 | 16:72114002 | T/C | 0.193 | 0.0120 (0.0019) | 2.1E-10 | -0.0132 (0.0606) | 0.828 | 40.4 | -1.104 |
| Hand grip strength (left) | Vascular dementia (FinnGen) | rs8108461 | 19:2186757 | C/T | 0.573 | 0.0095 (0.0015) | 2.6E-10 | -0.0241 (0.0496) | 0.627 | 40.0 | -2.530 |
| Hand grip strength (left) | Vascular dementia (FinnGen) | rs41271299 | 6:19839415 | T/C | 0.051 | 0.0212 (0.0034) | 3.0E-10 | 0.1350 (0.1957) | 0.490 | 39.7 | 6.371 |
| Hand grip strength (left) | Vascular dementia (FinnGen) | rs34587452 | 4:1009900 | C/G | 0.215 | -0.0114 (0.0018) | 3.2E-10 | 0.1200 (0.0620) | 0.053 | 39.5 | -10.559 |
| Hand grip strength (left) | Vascular dementia (FinnGen) | rs4121165 | 1:78276977 | A/G | 0.211 | -0.0114 (0.0018) | 3.4E-10 | 0.0146 (0.0651) | 0.823 | 39.4 | -1.279 |
| Hand grip strength (left) | Vascular dementia (FinnGen) | rs7575451 | 2:152352843 | G/C | 0.650 | -0.0097 (0.0016) | 3.8E-10 | 0.0172 (0.0527) | 0.745 | 39.2 | -1.768 |
| Hand grip strength (left) | Vascular dementia (FinnGen) | rs6802071 | 3:38574237 | T/C | 0.435 | -0.0094 (0.0015) | 3.8E-10 | -0.0373 (0.0496) | 0.452 | 39.2 | 3.966 |
| Hand grip strength (left) | Vascular dementia (FinnGen) | rs76895963 | 12:4384844 | G/T | 0.021 | 0.0360 (0.0057) | 3.9E-10 | -0.1199 (0.1500) | 0.424 | 39.1 | -3.334 |
| Hand grip strength (left) | Vascular dementia (FinnGen) | rs34845616 | 11:133792644 | A/G | 0.246 | 0.0108 (0.0017) | 4.0E-10 | -0.0496 (0.0527) | 0.347 | 39.1 | -4.576 |
| Hand grip strength (left) | Vascular dementia (FinnGen) | rs4498020 | 3:13810820 | A/C | 0.724 | -0.0104 (0.0017) | 4.3E-10 | 0.0275 (0.0532) | 0.605 | 39.0 | -2.638 |
| Hand grip strength (left) | Vascular dementia (FinnGen) | rs9611273 | 22:40534466 | T/C | 0.253 | 0.0108 (0.0017) | 4.5E-10 | 0.0353 (0.0563) | 0.530 | 38.9 | 3.272 |
| Hand grip strength (left) | Vascular dementia (FinnGen) | rs10821939 | 10:63751748 | A/G | 0.573 | -0.0094 (0.0015) | 4.9E-10 | 0.0718 (0.0503) | 0.153 | 38.7 | -7.676 |
| Hand grip strength (left) | Vascular dementia (FinnGen) | rs1434095 | 2:179254330 | C/T | 0.875 | 0.0140 (0.0023) | 5.3E-10 | 0.0787 (0.0833) | 0.345 | 38.6 | 5.609 |
| Hand grip strength (left) | Vascular dementia (FinnGen) | rs35609019 | 4:7847892 | C/G | 0.398 | 0.0095 (0.0015) | 7.7E-10 | -0.0264 (0.0502) | 0.598 | 37.8 | -2.786 |
| Hand grip strength (left) | Vascular dementia (FinnGen) | rs10831903 | 11:12758660 | T/C | 0.423 | 0.0093 (0.0015) | 8.1E-10 | -0.0692 (0.0525) | 0.188 | 37.8 | -7.452 |
| Hand grip strength (left) | Vascular dementia (FinnGen) | rs75497896 | 4:119636703 | C/T | 0.051 | -0.0207 (0.0034) | 9.1E-10 | -0.0675 (0.1044) | 0.518 | 37.5 | 3.265 |
| Hand grip strength (left) | Vascular dementia (FinnGen) | rs9371881 | 6:155638213 | A/G | 0.359 | 0.0095 (0.0015) | 9.6E-10 | -0.0396 (0.0559) | 0.479 | 37.4 | -4.177 |
| Hand grip strength (left) | Vascular dementia (FinnGen) | rs1981612 | 2:199235664 | A/C | 0.456 | 0.0092 (0.0015) | 1.0E-09 | -0.0535 (0.0496) | 0.281 | 37.3 | -5.798 |
| Hand grip strength (left) | Vascular dementia (FinnGen) | rs10205394 | 2:218150948 | C/G | 0.201 | -0.0113 (0.0019) | 1.1E-09 | 0.0201 (0.0558) | 0.718 | 37.2 | -1.775 |
| Hand grip strength (left) | Vascular dementia (FinnGen) | rs4677601 | 3:71368790 | G/A | 0.510 | 0.0091 (0.0015) | 1.1E-09 | 0.0914 (0.0492) | 0.063 | 37.2 | 10.090 |
| Hand grip strength (left) | Vascular dementia (FinnGen) | rs73307079 | 7:19020024 | C/T | 0.211 | 0.0111 (0.0018) | 1.4E-09 | 0.0369 (0.0564) | 0.513 | 36.6 | 3.324 |
| Hand grip strength (left) | Vascular dementia (FinnGen) | rs7197751 | 16:84940033 | T/G | 0.363 | -0.0095 (0.0016) | 1.4E-09 | -0.0500 (0.0538) | 0.352 | 36.7 | 5.284 |
| Hand grip strength (left) | Vascular dementia (FinnGen) | rs821100 | 8:89448877 | G/A | 0.266 | -0.0102 (0.0017) | 1.5E-09 | 0.0446 (0.0562) | 0.428 | 36.6 | -4.374 |
| Hand grip strength (left) | Vascular dementia (FinnGen) | rs723588 | 6:81056634 | C/T | 0.143 | 0.0128 (0.0021) | 1.6E-09 | -0.0431 (0.0667) | 0.518 | 36.4 | -3.370 |
| Hand grip strength (left) | Vascular dementia (FinnGen) | rs7148603 | 14:36683779 | A/G | 0.359 | 0.0096 (0.0016) | 1.6E-09 | -0.1155 (0.0531) | 0.030 | 36.4 | -12.066 |
| Hand grip strength (left) | Vascular dementia (FinnGen) | rs10988217 | 9:131888116 | G/A | 0.604 | -0.0092 (0.0015) | 1.7E-09 | 0.0838 (0.0500) | 0.094 | 36.2 | -9.102 |
| Hand grip strength (left) | Vascular dementia (FinnGen) | rs7176095 | 15:74886411 | G/A | 0.128 | -0.0134 (0.0022) | 1.7E-09 | 0.0697 (0.0743) | 0.348 | 36.3 | -5.211 |
| Hand grip strength (left) | Vascular dementia (FinnGen) | rs10144445 | 14:39695362 | G/C | 0.350 | -0.0094 (0.0016) | 1.9E-09 | -0.0284 (0.0516) | 0.582 | 36.1 | 3.031 |
| Hand grip strength (left) | Vascular dementia (FinnGen) | rs181766 | 16:14394878 | C/T | 0.322 | 0.0096 (0.0016) | 1.9E-09 | 0.0238 (0.0515) | 0.644 | 36.1 | 2.471 |
| Hand grip strength (left) | Vascular dementia (FinnGen) | rs2587505 | 17:77784268 | C/T | 0.420 | -0.0090 (0.0015) | 2.3E-09 | 0.0649 (0.0526) | 0.217 | 35.7 | -7.208 |
| Hand grip strength (left) | Vascular dementia (FinnGen) | rs12673062 | 7:4710677 | A/G | 0.216 | -0.0108 (0.0018) | 2.6E-09 | 0.0635 (0.0551) | 0.250 | 35.5 | -5.896 |
| Hand grip strength (left) | Vascular dementia (FinnGen) | rs6006984 | 22:45714937 | C/T | 0.278 | 0.0098 (0.0017) | 2.6E-09 | -0.1075 (0.0538) | 0.046 | 35.5 | -10.915 |
| Hand grip strength (left) | Vascular dementia (FinnGen) | rs6882168 | 5:39402647 | T/C | 0.337 | -0.0094 (0.0016) | 2.8E-09 | 0.0073 (0.0501) | 0.884 | 35.3 | -0.780 |
| Hand grip strength (left) | Vascular dementia (FinnGen) | rs12528131 | 6:105389104 | G/A | 0.488 | -0.0088 (0.0015) | 2.8E-09 | -0.0123 (0.0496) | 0.805 | 35.3 | 1.393 |
| Hand grip strength (left) | Vascular dementia (FinnGen) | rs9371201 | 6:150145001 | T/C | 0.335 | -0.0093 (0.0016) | 3.0E-09 | 0.0694 (0.0508) | 0.172 | 35.2 | -7.432 |
| Hand grip strength (left) | Vascular dementia (FinnGen) | rs6962338 | 7:69160985 | G/A | 0.044 | -0.0214 (0.0036) | 3.2E-09 | -0.1648 (0.1334) | 0.217 | 35.0 | 7.698 |
| Hand grip strength (left) | Vascular dementia (FinnGen) | rs77485342 | 6:30842866 | T/C | 0.018 | 0.0330 (0.0056) | 3.5E-09 | -0.4180 (0.1576) | 0.008 | 34.9 | -12.679 |
| Hand grip strength (left) | Vascular dementia (FinnGen) | rs4930236 | 11:68414000 | A/C | 0.836 | 0.0119 (0.0020) | 3.8E-09 | -0.0469 (0.0819) | 0.567 | 34.7 | -3.940 |
| Hand grip strength (left) | Vascular dementia (FinnGen) | rs13106087 | 4:145566864 | C/T | 0.830 | 0.0116 (0.0020) | 3.9E-09 | 0.0014 (0.0659) | 0.984 | 34.6 | 0.120 |
| Hand grip strength (left) | Vascular dementia (FinnGen) | rs17630248 | 2:201137782 | C/T | 0.348 | -0.0092 (0.0016) | 4.1E-09 | -0.0485 (0.0520) | 0.350 | 34.6 | 5.281 |
| Hand grip strength (left) | Vascular dementia (FinnGen) | rs4335354 | 1:88899964 | A/C | 0.316 | -0.0094 (0.0016) | 4.6E-09 | 0.0166 (0.0547) | 0.761 | 34.4 | -1.767 |
| Hand grip strength (left) | Vascular dementia (FinnGen) | rs9866627 | 3:135522715 | A/C | 0.084 | -0.0156 (0.0027) | 5.7E-09 | -0.0335 (0.0744) | 0.653 | 33.9 | 2.148 |
| Hand grip strength (left) | Vascular dementia (FinnGen) | rs35236379 | 10:5727292 | T/G | 0.142 | 0.0124 (0.0021) | 5.8E-09 | -0.0447 (0.0648) | 0.490 | 33.9 | -3.618 |
| Hand grip strength (left) | Vascular dementia (FinnGen) | rs1924721 | 1:190972986 | T/G | 0.117 | 0.0134 (0.0023) | 6.2E-09 | -0.0196 (0.0659) | 0.766 | 33.8 | -1.458 |
| Hand grip strength (left) | Vascular dementia (FinnGen) | rs997850 | 4:154838434 | C/G | 0.605 | -0.0089 (0.0015) | 6.3E-09 | 0.0535 (0.0493) | 0.279 | 33.7 | -6.029 |
| Hand grip strength (left) | Vascular dementia (FinnGen) | rs2038760 | 6:2680732 | T/C | 0.171 | -0.0115 (0.0020) | 6.6E-09 | -0.1349 (0.0667) | 0.043 | 33.7 | 11.704 |
| Hand grip strength (left) | Vascular dementia (FinnGen) | rs2359239 | 14:75326771 | T/C | 0.392 | -0.0088 (0.0015) | 6.9E-09 | -0.0372 (0.0494) | 0.451 | 33.6 | 4.220 |
| Hand grip strength (left) | Vascular dementia (FinnGen) | rs11204664 | 1:150531380 | C/T | 0.579 | -0.0087 (0.0015) | 8.1E-09 | -0.0713 (0.0526) | 0.175 | 33.2 | 8.241 |
| Hand grip strength (left) | Vascular dementia (FinnGen) | rs13227429 | 7:140560023 | C/T | 0.561 | -0.0086 (0.0015) | 9.8E-09 | 0.0270 (0.0493) | 0.584 | 32.9 | -3.144 |
| Hand grip strength (left) | Vascular dementia (FinnGen) | rs143002906 | 18:12992162 | T/C | 0.028 | 0.0262 (0.0046) | 9.8E-09 | -0.0500 (0.1102) | 0.650 | 32.9 | -1.906 |
| Hand grip strength (left) | Vascular dementia (FinnGen) | rs61286123 | 2:60205600 | C/T | 0.228 | -0.0101 (0.0018) | 1.2E-08 | 0.1522 (0.0578) | 0.008 | 32.5 | -15.063 |
| Hand grip strength (left) | Vascular dementia (FinnGen) | rs9944324 | 16:80929342 | G/A | 0.457 | -0.0086 (0.0015) | 1.2E-08 | 0.0547 (0.0496) | 0.270 | 32.4 | -6.396 |
| Hand grip strength (left) | Vascular dementia (FinnGen) | rs4739739 | 8:81304576 | G/A | 0.415 | -0.0085 (0.0015) | 1.4E-08 | 0.0520 (0.0510) | 0.308 | 32.2 | -6.093 |
| Hand grip strength (left) | Vascular dementia (FinnGen) | rs113918482 | 5:161289270 | G/A | 0.223 | -0.0101 (0.0018) | 1.5E-08 | -0.0436 (0.0533) | 0.413 | 32.0 | 4.316 |
| Hand grip strength (left) | Vascular dementia (FinnGen) | rs55681913 | 5:42687629 | C/T | 0.106 | 0.0138 (0.0024) | 1.6E-08 | 0.0725 (0.0756) | 0.338 | 32.0 | 5.264 |
| Hand grip strength (left) | Vascular dementia (FinnGen) | rs11003014 | 10:81231387 | G/A | 0.161 | 0.0114 (0.0020) | 1.6E-08 | 0.0549 (0.0596) | 0.357 | 32.0 | 4.802 |
| Hand grip strength (left) | Vascular dementia (FinnGen) | rs10860864 | 12:102822862 | T/C | 0.181 | 0.0108 (0.0019) | 1.9E-08 | -0.0537 (0.0615) | 0.383 | 31.6 | -4.958 |
| Hand grip strength (left) | Vascular dementia (FinnGen) | rs4398863 | 8:135695110 | C/G | 0.737 | -0.0095 (0.0017) | 2.0E-08 | 0.0147 (0.0596) | 0.805 | 31.5 | -1.556 |
| Hand grip strength (left) | Vascular dementia (FinnGen) | rs116922558 | 9:118802375 | G/A | 0.040 | -0.0216 (0.0039) | 2.1E-08 | 0.0940 (0.1194) | 0.431 | 31.4 | -4.355 |
| Hand grip strength (left) | Vascular dementia (FinnGen) | rs1884447 | 1:185021410 | A/G | 0.401 | 0.0085 (0.0015) | 2.3E-08 | -0.0354 (0.0500) | 0.479 | 31.2 | -4.184 |
| Hand grip strength (left) | Vascular dementia (FinnGen) | rs59116179 | 8:22603454 | T/C | 0.617 | 0.0086 (0.0015) | 2.4E-08 | -0.0012 (0.0512) | 0.981 | 31.1 | -0.140 |
| Hand grip strength (left) | Vascular dementia (FinnGen) | rs11168357 | 12:48412138 | A/G | 0.246 | -0.0096 (0.0017) | 2.5E-08 | 0.0902 (0.0675) | 0.181 | 31.1 | -9.368 |
| Hand grip strength (left) | Vascular dementia (FinnGen) | rs116409670 | 5:37327472 | T/C | 0.080 | -0.0152 (0.0027) | 2.6E-08 | 0.0159 (0.1326) | 0.904 | 31.0 | -1.043 |
| Hand grip strength (left) | Vascular dementia (FinnGen) | rs12533765 | 7:127699186 | G/A | 0.280 | -0.0092 (0.0017) | 2.6E-08 | -0.0369 (0.0646) | 0.568 | 31.0 | 4.011 |
| Hand grip strength (left) | Vascular dementia (FinnGen) | rs62081464 | 18:35142133 | T/C | 0.227 | -0.0099 (0.0018) | 2.8E-08 | -0.0501 (0.0605) | 0.407 | 30.8 | 5.069 |
| Hand grip strength (left) | Vascular dementia (FinnGen) | rs4811040 | 20:48968438 | G/C | 0.277 | -0.0092 (0.0017) | 3.0E-08 | -0.0185 (0.0548) | 0.736 | 30.7 | 2.000 |
| Hand grip strength (left) | Vascular dementia (FinnGen) | rs7516571 | 1:40733658 | G/A | 0.259 | 0.0094 (0.0017) | 3.1E-08 | 0.0088 (0.0541) | 0.870 | 30.7 | 0.938 |
| Hand grip strength (left) | Vascular dementia (FinnGen) | rs2800789 | 1:164578242 | C/A | 0.480 | 0.0083 (0.0015) | 3.1E-08 | -0.0569 (0.0493) | 0.248 | 30.6 | -6.888 |
| Hand grip strength (left) | Vascular dementia (FinnGen) | rs4962700 | 10:126479989 | G/C | 0.302 | 0.0090 (0.0016) | 3.2E-08 | -0.0501 (0.0506) | 0.322 | 30.6 | -5.540 |
| Hand grip strength (left) | Vascular dementia (FinnGen) | rs13091492 | 3:81891476 | G/A | 0.373 | -0.0085 (0.0015) | 3.3E-08 | -0.0182 (0.0491) | 0.711 | 30.6 | 2.146 |
| Hand grip strength (left) | Vascular dementia (FinnGen) | rs34722008 | 4:38659594 | A/G | 0.353 | 0.0086 (0.0016) | 3.3E-08 | 0.0171 (0.0505) | 0.735 | 30.5 | 1.992 |
| Hand grip strength (left) | Vascular dementia (FinnGen) | rs2850379 | 4:102917419 | A/C | 0.432 | -0.0083 (0.0015) | 3.3E-08 | -0.0004 (0.0491) | 0.994 | 30.5 | 0.048 |
| Hand grip strength (left) | Vascular dementia (FinnGen) | rs2974438 | 5:168250903 | A/G | 0.211 | -0.0101 (0.0018) | 3.3E-08 | -0.0642 (0.0627) | 0.306 | 30.5 | 6.373 |
| Hand grip strength (left) | Vascular dementia (FinnGen) | rs58670122 | 1:22492613 | G/A | 0.143 | -0.0118 (0.0021) | 3.6E-08 | 0.1910 (0.0814) | 0.019 | 30.4 | -16.166 |
| Hand grip strength (left) | Vascular dementia (FinnGen) | rs16910750 | 9:99084471 | C/G | 0.160 | 0.0112 (0.0020) | 3.6E-08 | -0.0796 (0.0723) | 0.271 | 30.4 | -7.076 |
| Hand grip strength (left) | Vascular dementia (FinnGen) | rs17282763 | 7:82520166 | C/T | 0.296 | 0.0089 (0.0016) | 4.4E-08 | -0.0785 (0.0534) | 0.141 | 30.0 | -8.781 |
| Hand grip strength (left) | Vascular dementia (FinnGen) | rs7026798 | 9:16427378 | C/T | 0.432 | 0.0082 (0.0015) | 4.6E-08 | 0.0733 (0.0494) | 0.138 | 29.9 | 8.900 |
| Hand grip strength (left) | Vascular dementia (FinnGen) | rs10934857 | 3:128199662 | A/G | 0.259 | 0.0093 (0.0017) | 4.8E-08 | 0.0084 (0.0595) | 0.888 | 29.8 | 0.905 |
| Hand grip strength (left) | Vascular dementia (FinnGen) | rs2789514 | 9:129833029 | A/G | 0.868 | 0.0121 (0.0022) | 4.9E-08 | -0.0367 (0.1100) | 0.739 | 29.8 | -3.044 |
| Hand grip strength (left) | Alzheimer's disease, wide (FinnGen) | rs143384 | 20:34025756 | G/A | 0.404 | 0.0209 (0.0015) | 1.5E-43 | -0.0114 (0.0214) | 0.592 | 191.5 | -0.545 |
| Hand grip strength (left) | Alzheimer's disease, wide (FinnGen) | rs12316046 | 12:15054415 | G/A | 0.378 | -0.0174 (0.0015) | 5.0E-30 | -0.0175 (0.0238) | 0.462 | 129.6 | 1.005 |
| Hand grip strength (left) | Alzheimer's disease, wide (FinnGen) | rs10846071 | 12:15016236 | T/C | 0.394 | -0.0166 (0.0015) | 5.3E-28 | -0.0202 (0.0230) | 0.380 | 120.4 | 1.214 |
| Hand grip strength (left) | Alzheimer's disease, wide (FinnGen) | rs1556659 | 10:130834698 | T/C | 0.382 | 0.0163 (0.0015) | 2.5E-26 | -0.0001 (0.0233) | 0.996 | 112.7 | -0.006 |
| Hand grip strength (left) | Alzheimer's disease, wide (FinnGen) | rs13146142 | 4:17931318 | C/T | 0.159 | -0.0202 (0.0020) | 2.3E-23 | 0.0470 (0.0361) | 0.193 | 99.2 | -2.326 |
| Hand grip strength (left) | Alzheimer's disease, wide (FinnGen) | rs3118903 | 13:51099577 | A/G | 0.220 | -0.0174 (0.0018) | 2.7E-22 | -0.0392 (0.0242) | 0.106 | 94.3 | 2.247 |
| Hand grip strength (left) | Alzheimer's disease, wide (FinnGen) | rs2871865 | 15:99194896 | G/C | 0.116 | -0.0218 (0.0023) | 5.0E-21 | 0.0749 (0.0441) | 0.089 | 88.5 | -3.436 |
| Hand grip strength (left) | Alzheimer's disease, wide (FinnGen) | rs1044299 | 1:176811873 | T/C | 0.546 | 0.0140 (0.0015) | 6.6E-21 | -0.0059 (0.0213) | 0.784 | 88.0 | -0.421 |
| Hand grip strength (left) | Alzheimer's disease, wide (FinnGen) | rs12790261 | 11:66988048 | A/C | 0.082 | -0.0252 (0.0027) | 1.2E-20 | 0.0225 (0.0484) | 0.642 | 86.8 | -0.893 |
| Hand grip strength (left) | Alzheimer's disease, wide (FinnGen) | rs6977081 | 7:150542515 | T/G | 0.334 | 0.0148 (0.0016) | 1.4E-20 | 0.0131 (0.0221) | 0.554 | 86.5 | 0.888 |
| Hand grip strength (left) | Alzheimer's disease, wide (FinnGen) | rs13107325 | 4:103188709 | T/C | 0.075 | -0.0262 (0.0028) | 1.8E-20 | 0.1306 (0.0917) | 0.154 | 86.0 | -4.993 |
| Hand grip strength (left) | Alzheimer's disease, wide (FinnGen) | rs4713506 | 6:32113980 | A/G | 0.256 | -0.0157 (0.0017) | 2.0E-20 | -0.0288 (0.0226) | 0.202 | 85.8 | 1.831 |
| Hand grip strength (left) | Alzheimer's disease, wide (FinnGen) | rs3819121 | 2:135622860 | C/T | 0.369 | 0.0141 (0.0015) | 2.9E-20 | 0.0142 (0.0217) | 0.514 | 85.0 | 1.007 |
| Hand grip strength (left) | Alzheimer's disease, wide (FinnGen) | rs10788958 | 1:54040670 | G/C | 0.645 | 0.0142 (0.0016) | 1.0E-19 | 0.0305 (0.0244) | 0.210 | 82.5 | 2.148 |
| Hand grip strength (left) | Alzheimer's disease, wide (FinnGen) | rs7761063 | 6:130379973 | C/A | 0.673 | -0.0141 (0.0016) | 5.1E-19 | 0.0405 (0.0225) | 0.072 | 79.4 | -2.876 |
| Hand grip strength (left) | Alzheimer's disease, wide (FinnGen) | rs4308051 | 18:20735461 | G/T | 0.789 | 0.0160 (0.0018) | 1.4E-18 | 0.0264 (0.0252) | 0.296 | 77.3 | 1.651 |
| Hand grip strength (left) | Alzheimer's disease, wide (FinnGen) | rs7571789 | 2:70714793 | C/T | 0.523 | 0.0130 (0.0015) | 3.0E-18 | -0.0304 (0.0214) | 0.155 | 75.9 | -2.347 |
| Hand grip strength (left) | Alzheimer's disease, wide (FinnGen) | rs755547 | 17:43011908 | A/G | 0.189 | 0.0165 (0.0019) | 3.2E-18 | -0.0017 (0.0309) | 0.956 | 75.7 | -0.103 |
| Hand grip strength (left) | Alzheimer's disease, wide (FinnGen) | rs11125803 | 2:25052177 | T/C | 0.741 | 0.0143 (0.0017) | 3.8E-17 | 0.0006 (0.0249) | 0.982 | 70.9 | 0.042 |
| Hand grip strength (left) | Alzheimer's disease, wide (FinnGen) | rs999493 | 17:46625519 | A/G | 0.622 | 0.0129 (0.0015) | 6.2E-17 | 0.0366 (0.0220) | 0.096 | 69.9 | 2.844 |
| Hand grip strength (left) | Alzheimer's disease, wide (FinnGen) | rs635538 | 18:53273614 | A/G | 0.914 | -0.0217 (0.0027) | 3.8E-16 | 0.0625 (0.0350) | 0.074 | 66.4 | -2.886 |
| Hand grip strength (left) | Alzheimer's disease, wide (FinnGen) | rs2871960 | 3:141121814 | C/A | 0.445 | 0.0121 (0.0015) | 5.5E-16 | 0.0376 (0.0213) | 0.078 | 65.6 | 3.111 |
| Hand grip strength (left) | Alzheimer's disease, wide (FinnGen) | rs113434679 | 17:44126765 | A/C | 0.200 | -0.0149 (0.0019) | 1.9E-15 | -0.0802 (0.0404) | 0.047 | 63.2 | 5.377 |
| Hand grip strength (left) | Alzheimer's disease, wide (FinnGen) | rs7196917 | 16:69896527 | G/A | 0.430 | -0.0117 (0.0015) | 5.4E-15 | 0.0238 (0.0218) | 0.275 | 61.1 | -2.029 |
| Hand grip strength (left) | Alzheimer's disease, wide (FinnGen) | rs17466480 | 15:77390870 | G/A | 0.387 | -0.0118 (0.0015) | 9.8E-15 | 0.0097 (0.0220) | 0.658 | 59.9 | -0.820 |
| Hand grip strength (left) | Alzheimer's disease, wide (FinnGen) | rs7124681 | 11:47529947 | A/C | 0.408 | -0.0117 (0.0015) | 1.0E-14 | -0.0194 (0.0223) | 0.383 | 59.9 | 1.664 |
| Hand grip strength (left) | Alzheimer's disease, wide (FinnGen) | rs11076004 | 16:53913930 | A/G | 0.418 | -0.0115 (0.0015) | 2.1E-14 | 0.0023 (0.0212) | 0.913 | 58.4 | -0.199 |
| Hand grip strength (left) | Alzheimer's disease, wide (FinnGen) | rs823130 | 1:205714372 | T/C | 0.433 | -0.0113 (0.0015) | 4.4E-14 | 0.0300 (0.0214) | 0.162 | 57.0 | -2.647 |
| Hand grip strength (left) | Alzheimer's disease, wide (FinnGen) | rs12473732 | 2:44118428 | T/C | 0.487 | 0.0110 (0.0015) | 1.4E-13 | -0.0245 (0.0213) | 0.250 | 54.7 | -2.230 |
| Hand grip strength (left) | Alzheimer's disease, wide (FinnGen) | rs13337177 | 16:2175323 | T/G | 0.181 | -0.0143 (0.0019) | 1.6E-13 | 0.0364 (0.0279) | 0.191 | 54.4 | -2.549 |
| Hand grip strength (left) | Alzheimer's disease, wide (FinnGen) | rs2631360 | 5:131707429 | A/G | 0.519 | -0.0109 (0.0015) | 1.9E-13 | 0.0152 (0.0223) | 0.497 | 54.1 | -1.392 |
| Hand grip strength (left) | Alzheimer's disease, wide (FinnGen) | rs7856625 | 9:119345083 | T/C | 0.610 | -0.0111 (0.0015) | 2.5E-13 | -0.0334 (0.0222) | 0.133 | 53.5 | 3.001 |
| Hand grip strength (left) | Alzheimer's disease, wide (FinnGen) | rs150330307 | 1:160160801 | C/T | 0.032 | -0.0308 (0.0042) | 2.9E-13 | -0.0963 (0.0767) | 0.209 | 53.3 | 3.129 |
| Hand grip strength (left) | Alzheimer's disease, wide (FinnGen) | rs1551042 | 3:85630551 | C/A | 0.647 | -0.0111 (0.0016) | 7.4E-13 | -0.0381 (0.0239) | 0.110 | 51.4 | 3.424 |
| Hand grip strength (left) | Alzheimer's disease, wide (FinnGen) | rs12906830 | 15:56963503 | C/T | 0.601 | 0.0108 (0.0015) | 9.3E-13 | -0.0294 (0.0221) | 0.183 | 51.0 | -2.712 |
| Hand grip strength (left) | Alzheimer's disease, wide (FinnGen) | rs61389091 | 11:74427921 | T/C | 0.042 | 0.0262 (0.0037) | 2.4E-12 | -0.0399 (0.0535) | 0.455 | 49.1 | -1.525 |
| Hand grip strength (left) | Alzheimer's disease, wide (FinnGen) | rs2273555 | 10:104127171 | A/G | 0.606 | 0.0106 (0.0015) | 2.7E-12 | -0.0018 (0.0217) | 0.934 | 48.9 | -0.169 |
| Hand grip strength (left) | Alzheimer's disease, wide (FinnGen) | rs11121542 | 1:10393920 | A/G | 0.123 | -0.0158 (0.0023) | 3.0E-12 | 0.0259 (0.0346) | 0.455 | 48.7 | -1.643 |
| Hand grip strength (left) | Alzheimer's disease, wide (FinnGen) | rs3814877 | 16:30042677 | T/G | 0.402 | 0.0105 (0.0015) | 3.4E-12 | -0.0320 (0.0219) | 0.145 | 48.5 | -3.036 |
| Hand grip strength (left) | Alzheimer's disease, wide (FinnGen) | rs7963801 | 12:79685226 | C/T | 0.572 | -0.0104 (0.0015) | 4.6E-12 | -0.0134 (0.0216) | 0.536 | 47.9 | 1.284 |
| Hand grip strength (left) | Alzheimer's disease, wide (FinnGen) | rs12889267 | 14:21542766 | G/A | 0.167 | -0.0137 (0.0020) | 4.7E-12 | 0.0141 (0.0325) | 0.663 | 47.8 | -1.026 |
| Hand grip strength (left) | Alzheimer's disease, wide (FinnGen) | rs10176878 | 2:59952274 | C/T | 0.191 | -0.0129 (0.0019) | 8.6E-12 | 0.0122 (0.0307) | 0.691 | 46.6 | -0.942 |
| Hand grip strength (left) | Alzheimer's disease, wide (FinnGen) | rs7970350 | 12:66360164 | T/C | 0.494 | -0.0101 (0.0015) | 8.6E-12 | -0.0278 (0.0212) | 0.190 | 46.6 | 2.743 |
| Hand grip strength (left) | Alzheimer's disease, wide (FinnGen) | rs28542042 | 15:74213357 | T/C | 0.309 | 0.0110 (0.0016) | 1.2E-11 | -0.0208 (0.0238) | 0.383 | 46.0 | -1.889 |
| Hand grip strength (left) | Alzheimer's disease, wide (FinnGen) | rs1641457 | 2:40421990 | G/T | 0.223 | 0.0120 (0.0018) | 1.4E-11 | -0.0017 (0.0233) | 0.940 | 45.6 | -0.141 |
| Hand grip strength (left) | Alzheimer's disease, wide (FinnGen) | rs10403906 | 19:37376756 | A/G | 0.476 | -0.0100 (0.0015) | 1.5E-11 | 0.0029 (0.0215) | 0.894 | 45.5 | -0.289 |
| Hand grip strength (left) | Alzheimer's disease, wide (FinnGen) | rs4575361 | 12:124410529 | T/A | 0.312 | -0.0108 (0.0016) | 1.6E-11 | -0.0098 (0.0234) | 0.676 | 45.5 | 0.907 |
| Hand grip strength (left) | Alzheimer's disease, wide (FinnGen) | rs12763284 | 10:104508202 | G/A | 0.466 | 0.0100 (0.0015) | 1.7E-11 | -0.0033 (0.0213) | 0.877 | 45.3 | -0.330 |
| Hand grip strength (left) | Alzheimer's disease, wide (FinnGen) | rs10097417 | 8:110361477 | G/A | 0.171 | -0.0132 (0.0020) | 1.9E-11 | -0.0285 (0.0310) | 0.359 | 45.1 | 2.154 |
| Hand grip strength (left) | Alzheimer's disease, wide (FinnGen) | rs4363950 | 19:52221213 | G/A | 0.708 | 0.0110 (0.0016) | 1.9E-11 | 0.0431 (0.0258) | 0.095 | 45.1 | 3.926 |
| Hand grip strength (left) | Alzheimer's disease, wide (FinnGen) | rs34030812 | 2:144248905 | C/T | 0.367 | -0.0102 (0.0015) | 4.1E-11 | 0.0091 (0.0219) | 0.678 | 43.6 | -0.895 |
| Hand grip strength (left) | Alzheimer's disease, wide (FinnGen) | rs2532111 | 17:62017421 | G/A | 0.640 | 0.0103 (0.0016) | 4.1E-11 | 0.0350 (0.0217) | 0.107 | 43.6 | 3.403 |
| Hand grip strength (left) | Alzheimer's disease, wide (FinnGen) | rs11769549 | 7:23122239 | A/T | 0.062 | 0.0205 (0.0031) | 4.5E-11 | -0.0364 (0.0479) | 0.447 | 43.4 | -1.776 |
| Hand grip strength (left) | Alzheimer's disease, wide (FinnGen) | rs62253653 | 3:53013267 | G/A | 0.295 | 0.0107 (0.0016) | 5.5E-11 | 0.0008 (0.0226) | 0.971 | 43.0 | 0.075 |
| Hand grip strength (left) | Alzheimer's disease, wide (FinnGen) | rs6680160 | 1:32072737 | G/A | 0.628 | 0.0101 (0.0015) | 6.0E-11 | 0.0134 (0.0222) | 0.547 | 42.8 | 1.331 |
| Hand grip strength (left) | Alzheimer's disease, wide (FinnGen) | rs1486925 | 8:78827617 | C/T | 0.315 | -0.0105 (0.0016) | 6.3E-11 | -0.0179 (0.0237) | 0.451 | 42.7 | 1.709 |
| Hand grip strength (left) | Alzheimer's disease, wide (FinnGen) | rs11243202 | 6:7719065 | C/T | 0.486 | 0.0097 (0.0015) | 7.0E-11 | 0.0139 (0.0213) | 0.514 | 42.5 | 1.430 |
| Hand grip strength (left) | Alzheimer's disease, wide (FinnGen) | rs12914702 | 15:96887277 | A/G | 0.300 | 0.0110 (0.0017) | 9.3E-11 | -0.0083 (0.0228) | 0.717 | 42.0 | -0.758 |
| Hand grip strength (left) | Alzheimer's disease, wide (FinnGen) | rs4737446 | 8:57665019 | T/G | 0.695 | 0.0104 (0.0016) | 1.2E-10 | -0.0304 (0.0227) | 0.181 | 41.4 | -2.918 |
| Hand grip strength (left) | Alzheimer's disease, wide (FinnGen) | rs16870531 | 7:120660682 | T/C | 0.238 | 0.0112 (0.0017) | 1.3E-10 | -0.0058 (0.0245) | 0.813 | 41.3 | -0.518 |
| Hand grip strength (left) | Alzheimer's disease, wide (FinnGen) | rs2431112 | 5:103931707 | A/G | 0.441 | -0.0096 (0.0015) | 1.4E-10 | 0.0230 (0.0217) | 0.289 | 41.1 | -2.400 |
| Hand grip strength (left) | Alzheimer's disease, wide (FinnGen) | rs56338231 | 4:30867393 | G/A | 0.258 | -0.0108 (0.0017) | 1.7E-10 | -0.0138 (0.0253) | 0.585 | 40.8 | 1.272 |
| Hand grip strength (left) | Alzheimer's disease, wide (FinnGen) | rs11002322 | 10:79649653 | T/G | 0.340 | -0.0100 (0.0016) | 2.1E-10 | 0.0203 (0.0236) | 0.389 | 40.4 | -2.032 |
| Hand grip strength (left) | Alzheimer's disease, wide (FinnGen) | rs217181 | 16:72114002 | T/C | 0.193 | 0.0120 (0.0019) | 2.1E-10 | 0.0045 (0.0261) | 0.864 | 40.4 | 0.376 |
| Hand grip strength (left) | Alzheimer's disease, wide (FinnGen) | rs8108461 | 19:2186757 | C/T | 0.573 | 0.0095 (0.0015) | 2.6E-10 | -0.0148 (0.0214) | 0.490 | 40.0 | -1.554 |
| Hand grip strength (left) | Alzheimer's disease, wide (FinnGen) | rs41271299 | 6:19839415 | T/C | 0.051 | 0.0212 (0.0034) | 3.0E-10 | 0.0636 (0.0841) | 0.450 | 39.7 | 3.002 |
| Hand grip strength (left) | Alzheimer's disease, wide (FinnGen) | rs34587452 | 4:1009900 | C/G | 0.215 | -0.0114 (0.0018) | 3.2E-10 | 0.0629 (0.0266) | 0.018 | 39.5 | -5.535 |
| Hand grip strength (left) | Alzheimer's disease, wide (FinnGen) | rs4121165 | 1:78276977 | A/G | 0.211 | -0.0114 (0.0018) | 3.4E-10 | 0.0387 (0.0281) | 0.170 | 39.4 | -3.390 |
| Hand grip strength (left) | Alzheimer's disease, wide (FinnGen) | rs7575451 | 2:152352843 | G/C | 0.650 | -0.0097 (0.0016) | 3.8E-10 | 0.0065 (0.0228) | 0.776 | 39.2 | -0.668 |
| Hand grip strength (left) | Alzheimer's disease, wide (FinnGen) | rs6802071 | 3:38574237 | T/C | 0.435 | -0.0094 (0.0015) | 3.8E-10 | 0.0135 (0.0214) | 0.530 | 39.2 | -1.436 |
| Hand grip strength (left) | Alzheimer's disease, wide (FinnGen) | rs76895963 | 12:4384844 | G/T | 0.021 | 0.0360 (0.0057) | 3.9E-10 | -0.0216 (0.0641) | 0.736 | 39.1 | -0.601 |
| Hand grip strength (left) | Alzheimer's disease, wide (FinnGen) | rs34845616 | 11:133792644 | A/G | 0.246 | 0.0108 (0.0017) | 4.0E-10 | -0.0366 (0.0227) | 0.106 | 39.1 | -3.377 |
| Hand grip strength (left) | Alzheimer's disease, wide (FinnGen) | rs4498020 | 3:13810820 | A/C | 0.724 | -0.0104 (0.0017) | 4.3E-10 | -0.0174 (0.0230) | 0.451 | 39.0 | 1.669 |
| Hand grip strength (left) | Alzheimer's disease, wide (FinnGen) | rs9611273 | 22:40534466 | T/C | 0.253 | 0.0108 (0.0017) | 4.5E-10 | 0.0008 (0.0242) | 0.973 | 38.9 | 0.074 |
| Hand grip strength (left) | Alzheimer's disease, wide (FinnGen) | rs10821939 | 10:63751748 | A/G | 0.573 | -0.0094 (0.0015) | 4.9E-10 | -0.0027 (0.0217) | 0.901 | 38.7 | 0.289 |
| Hand grip strength (left) | Alzheimer's disease, wide (FinnGen) | rs1434095 | 2:179254330 | C/T | 0.875 | 0.0140 (0.0023) | 5.3E-10 | -0.0305 (0.0359) | 0.396 | 38.6 | -2.174 |
| Hand grip strength (left) | Alzheimer's disease, wide (FinnGen) | rs35609019 | 4:7847892 | C/G | 0.398 | 0.0095 (0.0015) | 7.7E-10 | -0.0011 (0.0216) | 0.958 | 37.8 | -0.116 |
| Hand grip strength (left) | Alzheimer's disease, wide (FinnGen) | rs10831903 | 11:12758660 | T/C | 0.423 | 0.0093 (0.0015) | 8.1E-10 | 0.0320 (0.0226) | 0.158 | 37.8 | 3.446 |
| Hand grip strength (left) | Alzheimer's disease, wide (FinnGen) | rs75497896 | 4:119636703 | C/T | 0.051 | -0.0207 (0.0034) | 9.1E-10 | 0.0607 (0.0447) | 0.174 | 37.5 | -2.936 |
| Hand grip strength (left) | Alzheimer's disease, wide (FinnGen) | rs9371881 | 6:155638213 | A/G | 0.359 | 0.0095 (0.0015) | 9.6E-10 | -0.0369 (0.0242) | 0.127 | 37.4 | -3.893 |
| Hand grip strength (left) | Alzheimer's disease, wide (FinnGen) | rs1981612 | 2:199235664 | A/C | 0.456 | 0.0092 (0.0015) | 1.0E-09 | -0.0235 (0.0214) | 0.272 | 37.3 | -2.547 |
| Hand grip strength (left) | Alzheimer's disease, wide (FinnGen) | rs10205394 | 2:218150948 | C/G | 0.201 | -0.0113 (0.0019) | 1.1E-09 | -0.0054 (0.0240) | 0.822 | 37.2 | 0.477 |
| Hand grip strength (left) | Alzheimer's disease, wide (FinnGen) | rs4677601 | 3:71368790 | G/A | 0.510 | 0.0091 (0.0015) | 1.1E-09 | 0.0156 (0.0212) | 0.462 | 37.2 | 1.722 |
| Hand grip strength (left) | Alzheimer's disease, wide (FinnGen) | rs73307079 | 7:19020024 | C/T | 0.211 | 0.0111 (0.0018) | 1.4E-09 | 0.0105 (0.0243) | 0.666 | 36.6 | 0.946 |
| Hand grip strength (left) | Alzheimer's disease, wide (FinnGen) | rs7197751 | 16:84940033 | T/G | 0.363 | -0.0095 (0.0016) | 1.4E-09 | 0.0248 (0.0231) | 0.282 | 36.7 | -2.621 |
| Hand grip strength (left) | Alzheimer's disease, wide (FinnGen) | rs821100 | 8:89448877 | G/A | 0.266 | -0.0102 (0.0017) | 1.5E-09 | 0.0096 (0.0242) | 0.692 | 36.6 | -0.941 |
| Hand grip strength (left) | Alzheimer's disease, wide (FinnGen) | rs723588 | 6:81056634 | C/T | 0.143 | 0.0128 (0.0021) | 1.6E-09 | 0.0198 (0.0286) | 0.489 | 36.4 | 1.548 |
| Hand grip strength (left) | Alzheimer's disease, wide (FinnGen) | rs7148603 | 14:36683779 | A/G | 0.359 | 0.0096 (0.0016) | 1.6E-09 | 0.0233 (0.0229) | 0.307 | 36.4 | 2.434 |
| Hand grip strength (left) | Alzheimer's disease, wide (FinnGen) | rs10988217 | 9:131888116 | G/A | 0.604 | -0.0092 (0.0015) | 1.7E-09 | 0.0141 (0.0216) | 0.516 | 36.2 | -1.532 |
| Hand grip strength (left) | Alzheimer's disease, wide (FinnGen) | rs7176095 | 15:74886411 | G/A | 0.128 | -0.0134 (0.0022) | 1.7E-09 | 0.0085 (0.0320) | 0.792 | 36.3 | -0.636 |
| Hand grip strength (left) | Alzheimer's disease, wide (FinnGen) | rs10144445 | 14:39695362 | G/C | 0.350 | -0.0094 (0.0016) | 1.9E-09 | -0.0012 (0.0222) | 0.958 | 36.1 | 0.128 |
| Hand grip strength (left) | Alzheimer's disease, wide (FinnGen) | rs181766 | 16:14394878 | C/T | 0.322 | 0.0096 (0.0016) | 1.9E-09 | 0.0096 (0.0222) | 0.665 | 36.1 | 0.997 |
| Hand grip strength (left) | Alzheimer's disease, wide (FinnGen) | rs2587505 | 17:77784268 | C/T | 0.420 | -0.0090 (0.0015) | 2.3E-09 | 0.0283 (0.0226) | 0.211 | 35.7 | -3.143 |
| Hand grip strength (left) | Alzheimer's disease, wide (FinnGen) | rs12673062 | 7:4710677 | A/G | 0.216 | -0.0108 (0.0018) | 2.6E-09 | -0.0226 (0.0239) | 0.344 | 35.5 | 2.099 |
| Hand grip strength (left) | Alzheimer's disease, wide (FinnGen) | rs6006984 | 22:45714937 | C/T | 0.278 | 0.0098 (0.0017) | 2.6E-09 | 0.0027 (0.0233) | 0.908 | 35.5 | 0.274 |
| Hand grip strength (left) | Alzheimer's disease, wide (FinnGen) | rs6882168 | 5:39402647 | T/C | 0.337 | -0.0094 (0.0016) | 2.8E-09 | -0.0245 (0.0216) | 0.258 | 35.3 | 2.619 |
| Hand grip strength (left) | Alzheimer's disease, wide (FinnGen) | rs12528131 | 6:105389104 | G/A | 0.488 | -0.0088 (0.0015) | 2.8E-09 | -0.0308 (0.0213) | 0.148 | 35.3 | 3.489 |
| Hand grip strength (left) | Alzheimer's disease, wide (FinnGen) | rs9371201 | 6:150145001 | T/C | 0.335 | -0.0093 (0.0016) | 3.0E-09 | -0.0073 (0.0219) | 0.740 | 35.2 | 0.782 |
| Hand grip strength (left) | Alzheimer's disease, wide (FinnGen) | rs6962338 | 7:69160985 | G/A | 0.044 | -0.0214 (0.0036) | 3.2E-09 | -0.0162 (0.0574) | 0.778 | 35.0 | 0.757 |
| Hand grip strength (left) | Alzheimer's disease, wide (FinnGen) | rs77485342 | 6:30842866 | T/C | 0.018 | 0.0330 (0.0056) | 3.5E-09 | 0.0979 (0.0668) | 0.143 | 34.9 | 2.970 |
| Hand grip strength (left) | Alzheimer's disease, wide (FinnGen) | rs4930236 | 11:68414000 | A/C | 0.836 | 0.0119 (0.0020) | 3.8E-09 | -0.0681 (0.0357) | 0.057 | 34.7 | -5.721 |
| Hand grip strength (left) | Alzheimer's disease, wide (FinnGen) | rs13106087 | 4:145566864 | C/T | 0.830 | 0.0116 (0.0020) | 3.9E-09 | 0.0132 (0.0285) | 0.644 | 34.6 | 1.136 |
| Hand grip strength (left) | Alzheimer's disease, wide (FinnGen) | rs17630248 | 2:201137782 | C/T | 0.348 | -0.0092 (0.0016) | 4.1E-09 | -0.0003 (0.0224) | 0.989 | 34.6 | 0.033 |
| Hand grip strength (left) | Alzheimer's disease, wide (FinnGen) | rs4335354 | 1:88899964 | A/C | 0.316 | -0.0094 (0.0016) | 4.6E-09 | 0.0090 (0.0235) | 0.702 | 34.4 | -0.958 |
| Hand grip strength (left) | Alzheimer's disease, wide (FinnGen) | rs9866627 | 3:135522715 | A/C | 0.084 | -0.0156 (0.0027) | 5.7E-09 | 0.0739 (0.0320) | 0.021 | 33.9 | -4.738 |
| Hand grip strength (left) | Alzheimer's disease, wide (FinnGen) | rs35236379 | 10:5727292 | T/G | 0.142 | 0.0124 (0.0021) | 5.8E-09 | -0.0066 (0.0279) | 0.812 | 33.9 | -0.534 |
| Hand grip strength (left) | Alzheimer's disease, wide (FinnGen) | rs1924721 | 1:190972986 | T/G | 0.117 | 0.0134 (0.0023) | 6.2E-09 | -0.0255 (0.0284) | 0.370 | 33.8 | -1.897 |
| Hand grip strength (left) | Alzheimer's disease, wide (FinnGen) | rs997850 | 4:154838434 | C/G | 0.605 | -0.0089 (0.0015) | 6.3E-09 | -0.0050 (0.0212) | 0.814 | 33.7 | 0.563 |
| Hand grip strength (left) | Alzheimer's disease, wide (FinnGen) | rs2038760 | 6:2680732 | T/C | 0.171 | -0.0115 (0.0020) | 6.6E-09 | 0.0108 (0.0288) | 0.707 | 33.7 | -0.937 |
| Hand grip strength (left) | Alzheimer's disease, wide (FinnGen) | rs2359239 | 14:75326771 | T/C | 0.392 | -0.0088 (0.0015) | 6.9E-09 | 0.0377 (0.0213) | 0.077 | 33.6 | -4.277 |
| Hand grip strength (left) | Alzheimer's disease, wide (FinnGen) | rs11204664 | 1:150531380 | C/T | 0.579 | -0.0087 (0.0015) | 8.1E-09 | 0.0079 (0.0227) | 0.728 | 33.2 | -0.913 |
| Hand grip strength (left) | Alzheimer's disease, wide (FinnGen) | rs13227429 | 7:140560023 | C/T | 0.561 | -0.0086 (0.0015) | 9.8E-09 | -0.0362 (0.0213) | 0.089 | 32.9 | 4.215 |
| Hand grip strength (left) | Alzheimer's disease, wide (FinnGen) | rs143002906 | 18:12992162 | T/C | 0.028 | 0.0262 (0.0046) | 9.8E-09 | -0.0563 (0.0479) | 0.239 | 32.9 | -2.147 |
| Hand grip strength (left) | Alzheimer's disease, wide (FinnGen) | rs61286123 | 2:60205600 | C/T | 0.228 | -0.0101 (0.0018) | 1.2E-08 | -0.0097 (0.0250) | 0.698 | 32.5 | 0.960 |
| Hand grip strength (left) | Alzheimer's disease, wide (FinnGen) | rs9944324 | 16:80929342 | G/A | 0.457 | -0.0086 (0.0015) | 1.2E-08 | 0.0120 (0.0213) | 0.573 | 32.4 | -1.403 |
| Hand grip strength (left) | Alzheimer's disease, wide (FinnGen) | rs4739739 | 8:81304576 | G/A | 0.415 | -0.0085 (0.0015) | 1.4E-08 | 0.0014 (0.0220) | 0.950 | 32.2 | -0.164 |
| Hand grip strength (left) | Alzheimer's disease, wide (FinnGen) | rs113918482 | 5:161289270 | G/A | 0.223 | -0.0101 (0.0018) | 1.5E-08 | 0.0136 (0.0230) | 0.553 | 32.0 | -1.346 |
| Hand grip strength (left) | Alzheimer's disease, wide (FinnGen) | rs55681913 | 5:42687629 | C/T | 0.106 | 0.0138 (0.0024) | 1.6E-08 | -0.0121 (0.0324) | 0.709 | 32.0 | -0.878 |
| Hand grip strength (left) | Alzheimer's disease, wide (FinnGen) | rs11003014 | 10:81231387 | G/A | 0.161 | 0.0114 (0.0020) | 1.6E-08 | -0.0175 (0.0257) | 0.496 | 32.0 | -1.531 |
| Hand grip strength (left) | Alzheimer's disease, wide (FinnGen) | rs10860864 | 12:102822862 | T/C | 0.181 | 0.0108 (0.0019) | 1.9E-08 | -0.0213 (0.0266) | 0.423 | 31.6 | -1.966 |
| Hand grip strength (left) | Alzheimer's disease, wide (FinnGen) | rs4398863 | 8:135695110 | C/G | 0.737 | -0.0095 (0.0017) | 2.0E-08 | -0.0252 (0.0256) | 0.324 | 31.5 | 2.667 |
| Hand grip strength (left) | Alzheimer's disease, wide (FinnGen) | rs116922558 | 9:118802375 | G/A | 0.040 | -0.0216 (0.0039) | 2.1E-08 | 0.0586 (0.0510) | 0.250 | 31.4 | -2.715 |
| Hand grip strength (left) | Alzheimer's disease, wide (FinnGen) | rs1884447 | 1:185021410 | A/G | 0.401 | 0.0085 (0.0015) | 2.3E-08 | 0.0088 (0.0215) | 0.685 | 31.2 | 1.040 |
| Hand grip strength (left) | Alzheimer's disease, wide (FinnGen) | rs59116179 | 8:22603454 | T/C | 0.617 | 0.0086 (0.0015) | 2.4E-08 | -0.0030 (0.0221) | 0.891 | 31.1 | -0.350 |
| Hand grip strength (left) | Alzheimer's disease, wide (FinnGen) | rs11168357 | 12:48412138 | A/G | 0.246 | -0.0096 (0.0017) | 2.5E-08 | 0.0094 (0.0292) | 0.748 | 31.1 | -0.976 |
| Hand grip strength (left) | Alzheimer's disease, wide (FinnGen) | rs116409670 | 5:37327472 | T/C | 0.080 | -0.0152 (0.0027) | 2.6E-08 | -0.0148 (0.0576) | 0.798 | 31.0 | 0.971 |
| Hand grip strength (left) | Alzheimer's disease, wide (FinnGen) | rs12533765 | 7:127699186 | G/A | 0.280 | -0.0092 (0.0017) | 2.6E-08 | 0.0287 (0.0278) | 0.302 | 31.0 | -3.120 |
| Hand grip strength (left) | Alzheimer's disease, wide (FinnGen) | rs62081464 | 18:35142133 | T/C | 0.227 | -0.0099 (0.0018) | 2.8E-08 | -0.0392 (0.0261) | 0.132 | 30.8 | 3.967 |
| Hand grip strength (left) | Alzheimer's disease, wide (FinnGen) | rs4811040 | 20:48968438 | G/C | 0.277 | -0.0092 (0.0017) | 3.0E-08 | -0.0085 (0.0236) | 0.719 | 30.7 | 0.919 |
| Hand grip strength (left) | Alzheimer's disease, wide (FinnGen) | rs7516571 | 1:40733658 | G/A | 0.259 | 0.0094 (0.0017) | 3.1E-08 | 0.0105 (0.0232) | 0.652 | 30.7 | 1.120 |
| Hand grip strength (left) | Alzheimer's disease, wide (FinnGen) | rs2800789 | 1:164578242 | C/A | 0.480 | 0.0083 (0.0015) | 3.1E-08 | 0.0058 (0.0213) | 0.785 | 30.6 | 0.702 |
| Hand grip strength (left) | Alzheimer's disease, wide (FinnGen) | rs4962700 | 10:126479989 | G/C | 0.302 | 0.0090 (0.0016) | 3.2E-08 | 0.0093 (0.0217) | 0.667 | 30.6 | 1.028 |
| Hand grip strength (left) | Alzheimer's disease, wide (FinnGen) | rs13091492 | 3:81891476 | G/A | 0.373 | -0.0085 (0.0015) | 3.3E-08 | -0.0041 (0.0212) | 0.848 | 30.6 | 0.484 |
| Hand grip strength (left) | Alzheimer's disease, wide (FinnGen) | rs34722008 | 4:38659594 | A/G | 0.353 | 0.0086 (0.0016) | 3.3E-08 | 0.0206 (0.0218) | 0.345 | 30.5 | 2.399 |
| Hand grip strength (left) | Alzheimer's disease, wide (FinnGen) | rs2850379 | 4:102917419 | A/C | 0.432 | -0.0083 (0.0015) | 3.3E-08 | 0.0223 (0.0212) | 0.292 | 30.5 | -2.690 |
| Hand grip strength (left) | Alzheimer's disease, wide (FinnGen) | rs2974438 | 5:168250903 | A/G | 0.211 | -0.0101 (0.0018) | 3.3E-08 | -0.0166 (0.0269) | 0.537 | 30.5 | 1.648 |
| Hand grip strength (left) | Alzheimer's disease, wide (FinnGen) | rs58670122 | 1:22492613 | G/A | 0.143 | -0.0118 (0.0021) | 3.6E-08 | 0.0590 (0.0350) | 0.092 | 30.4 | -4.994 |
| Hand grip strength (left) | Alzheimer's disease, wide (FinnGen) | rs16910750 | 9:99084471 | C/G | 0.160 | 0.0112 (0.0020) | 3.6E-08 | -0.0597 (0.0314) | 0.057 | 30.4 | -5.307 |
| Hand grip strength (left) | Alzheimer's disease, wide (FinnGen) | rs17282763 | 7:82520166 | C/T | 0.296 | 0.0089 (0.0016) | 4.4E-08 | 0.0031 (0.0230) | 0.892 | 30.0 | 0.347 |
| Hand grip strength (left) | Alzheimer's disease, wide (FinnGen) | rs7026798 | 9:16427378 | C/T | 0.432 | 0.0082 (0.0015) | 4.6E-08 | 0.0544 (0.0213) | 0.011 | 29.9 | 6.605 |
| Hand grip strength (left) | Alzheimer's disease, wide (FinnGen) | rs10934857 | 3:128199662 | A/G | 0.259 | 0.0093 (0.0017) | 4.8E-08 | 0.0289 (0.0257) | 0.262 | 29.8 | 3.112 |
| Hand grip strength (left) | Alzheimer's disease, wide (FinnGen) | rs2789514 | 9:129833029 | A/G | 0.868 | 0.0121 (0.0022) | 4.9E-08 | 0.0945 (0.0474) | 0.046 | 29.8 | 7.839 |
| Hand grip strength (right) | Alzheimer's disease (Bellenguez 2022) | rs143384 | 20:34025756 | G/A | 0.404 | 0.0230 (0.0015) | 2.5E-52 | -0.0057 (0.0082) | 0.487 | 231.7 | -0.247 |
| Hand grip strength (right) | Alzheimer's disease (Bellenguez 2022) | rs1556659 | 10:130834698 | T/C | 0.382 | 0.0175 (0.0015) | 3.8E-30 | 0.0072 (0.0087) | 0.412 | 130.2 | 0.411 |
| Hand grip strength (right) | Alzheimer's disease (Bellenguez 2022) | rs72977282 | 11:74300441 | A/T | 0.414 | -0.0168 (0.0015) | 1.2E-28 | -0.0108 (0.0083) | 0.192 | 123.3 | 0.642 |
| Hand grip strength (right) | Alzheimer's disease (Bellenguez 2022) | rs3118914 | 13:51116901 | T/G | 0.215 | -0.0194 (0.0018) | 5.9E-27 | -0.0032 (0.0098) | 0.745 | 115.6 | 0.165 |
| Hand grip strength (right) | Alzheimer's disease (Bellenguez 2022) | rs12316046 | 12:15054415 | G/A | 0.378 | -0.0162 (0.0015) | 3.8E-26 | -0.0011 (0.0084) | 0.895 | 111.9 | 0.068 |
| Hand grip strength (right) | Alzheimer's disease (Bellenguez 2022) | rs10846071 | 12:15016236 | T/C | 0.394 | -0.0157 (0.0015) | 6.5E-25 | -0.0030 (0.0083) | 0.720 | 106.2 | 0.192 |
| Hand grip strength (right) | Alzheimer's disease (Bellenguez 2022) | rs13146142 | 4:17931318 | C/T | 0.159 | -0.0208 (0.0020) | 1.4E-24 | 0.0385 (0.0110) | 4.6E-04 | 104.8 | -1.851 |
| Hand grip strength (right) | Alzheimer's disease (Bellenguez 2022) | rs2871865 | 15:99194896 | G/C | 0.116 | -0.0237 (0.0023) | 1.8E-24 | 0.0288 (0.0125) | 0.022 | 104.2 | -1.216 |
| Hand grip strength (right) | Alzheimer's disease (Bellenguez 2022) | rs9267806 | 6:32110886 | A/G | 0.256 | -0.0167 (0.0017) | 7.7E-23 | -0.0105 (0.0091) | 0.244 | 96.8 | 0.627 |
| Hand grip strength (right) | Alzheimer's disease (Bellenguez 2022) | rs7451021 | 6:130381246 | C/T | 0.689 | -0.0158 (0.0016) | 7.7E-23 | 0.0032 (0.0088) | 0.715 | 96.8 | -0.203 |
| Hand grip strength (right) | Alzheimer's disease (Bellenguez 2022) | rs12790261 | 11:66988048 | A/C | 0.082 | -0.0264 (0.0027) | 2.0E-22 | 0.0232 (0.0157) | 0.138 | 94.9 | -0.880 |
| Hand grip strength (right) | Alzheimer's disease (Bellenguez 2022) | rs13107325 | 4:103188709 | T/C | 0.075 | -0.0275 (0.0028) | 2.1E-22 | 0.0455 (0.0160) | 0.004 | 94.8 | -1.655 |
| Hand grip strength (right) | Alzheimer's disease (Bellenguez 2022) | rs10798483 | 1:176799143 | A/G | 0.547 | 0.0145 (0.0015) | 2.8E-22 | -0.0087 (0.0082) | 0.287 | 94.2 | -0.600 |
| Hand grip strength (right) | Alzheimer's disease (Bellenguez 2022) | rs3771498 | 2:70720070 | T/C | 0.514 | 0.0142 (0.0015) | 1.9E-21 | -0.0016 (0.0082) | 0.847 | 90.5 | -0.113 |
| Hand grip strength (right) | Alzheimer's disease (Bellenguez 2022) | rs4369779 | 18:20735408 | C/T | 0.789 | 0.0172 (0.0018) | 3.4E-21 | -0.0045 (0.0099) | 0.647 | 89.3 | -0.262 |
| Hand grip strength (right) | Alzheimer's disease (Bellenguez 2022) | rs1043515 | 17:36922196 | G/A | 0.566 | 0.0138 (0.0015) | 2.8E-20 | -0.0028 (0.0082) | 0.730 | 85.2 | -0.202 |
| Hand grip strength (right) | Alzheimer's disease (Bellenguez 2022) | rs1550115 | 2:25041620 | T/C | 0.749 | 0.0153 (0.0017) | 4.5E-19 | 0.0101 (0.0094) | 0.279 | 79.6 | 0.661 |
| Hand grip strength (right) | Alzheimer's disease (Bellenguez 2022) | rs4784329 | 16:53910261 | C/A | 0.426 | -0.0133 (0.0015) | 9.0E-19 | 0.0001 (0.0083) | 0.986 | 78.3 | -0.007 |
| Hand grip strength (right) | Alzheimer's disease (Bellenguez 2022) | rs12473839 | 2:135638044 | T/C | 0.371 | 0.0133 (0.0015) | 4.0E-18 | 0.0366 (0.0084) | 1.4E-05 | 75.3 | 2.758 |
| Hand grip strength (right) | Alzheimer's disease (Bellenguez 2022) | rs4927015 | 1:54072471 | A/G | 0.583 | 0.0131 (0.0015) | 5.0E-18 | -0.0041 (0.0084) | 0.626 | 74.9 | -0.314 |
| Hand grip strength (right) | Alzheimer's disease (Bellenguez 2022) | rs635538 | 18:53273614 | A/G | 0.914 | -0.0220 (0.0027) | 1.5E-16 | 0.0097 (0.0147) | 0.509 | 68.2 | -0.441 |
| Hand grip strength (right) | Alzheimer's disease (Bellenguez 2022) | rs823130 | 1:205714372 | T/C | 0.433 | -0.0124 (0.0015) | 1.7E-16 | -0.0007 (0.0082) | 0.935 | 67.9 | 0.057 |
| Hand grip strength (right) | Alzheimer's disease (Bellenguez 2022) | rs2165241 | 15:74222202 | C/T | 0.509 | 0.0122 (0.0015) | 1.9E-16 | -0.0235 (0.0081) | 0.004 | 67.7 | -1.919 |
| Hand grip strength (right) | Alzheimer's disease (Bellenguez 2022) | rs6977081 | 7:150542515 | T/G | 0.334 | 0.0129 (0.0016) | 5.7E-16 | 0.0041 (0.0086) | 0.634 | 65.5 | 0.319 |
| Hand grip strength (right) | Alzheimer's disease (Bellenguez 2022) | rs56365901 | 17:43960323 | G/A | 0.223 | -0.0143 (0.0018) | 1.8E-15 | -0.0539 (0.0098) | 3.9E-08 | 63.3 | 3.779 |
| Hand grip strength (right) | Alzheimer's disease (Bellenguez 2022) | rs7206195 | 16:2145280 | T/C | 0.180 | -0.0153 (0.0019) | 2.8E-15 | 0.0184 (0.0107) | 0.084 | 62.4 | -1.201 |
| Hand grip strength (right) | Alzheimer's disease (Bellenguez 2022) | rs10520770 | 18:46602964 | C/T | 0.449 | 0.0117 (0.0015) | 4.5E-15 | 0.0054 (0.0082) | 0.510 | 61.5 | 0.460 |
| Hand grip strength (right) | Alzheimer's disease (Bellenguez 2022) | rs11243202 | 6:7719065 | C/T | 0.486 | 0.0116 (0.0015) | 6.5E-15 | 0.0116 (0.0082) | 0.156 | 60.8 | 0.997 |
| Hand grip strength (right) | Alzheimer's disease (Bellenguez 2022) | rs150330307 | 1:160160801 | C/T | 0.032 | -0.0326 (0.0042) | 1.2E-14 | -0.0099 (0.0245) | 0.686 | 59.5 | 0.304 |
| Hand grip strength (right) | Alzheimer's disease (Bellenguez 2022) | rs2208562 | 9:119344528 | T/C | 0.610 | -0.0116 (0.0015) | 3.1E-14 | -0.0092 (0.0083) | 0.266 | 57.7 | 0.796 |
| Hand grip strength (right) | Alzheimer's disease (Bellenguez 2022) | rs10799428 | 1:227798565 | T/C | 0.187 | -0.0144 (0.0019) | 4.6E-14 | 0.0122 (0.0106) | 0.252 | 56.9 | -0.850 |
| Hand grip strength (right) | Alzheimer's disease (Bellenguez 2022) | rs2631360 | 5:131707429 | A/G | 0.519 | -0.0112 (0.0015) | 5.4E-14 | 0.0114 (0.0081) | 0.161 | 56.6 | -1.020 |
| Hand grip strength (right) | Alzheimer's disease (Bellenguez 2022) | rs7963801 | 12:79685226 | C/T | 0.572 | -0.0113 (0.0015) | 8.4E-14 | 0.0034 (0.0085) | 0.692 | 55.7 | -0.302 |
| Hand grip strength (right) | Alzheimer's disease (Bellenguez 2022) | rs2431112 | 5:103931707 | A/G | 0.441 | -0.0112 (0.0015) | 8.6E-14 | 0.0041 (0.0082) | 0.617 | 55.7 | -0.367 |
| Hand grip strength (right) | Alzheimer's disease (Bellenguez 2022) | rs1641457 | 2:40421990 | G/T | 0.223 | 0.0131 (0.0018) | 2.4E-13 | 0.0105 (0.0097) | 0.278 | 53.6 | 0.803 |
| Hand grip strength (right) | Alzheimer's disease (Bellenguez 2022) | rs6693965 | 1:10378416 | T/G | 0.128 | -0.0163 (0.0022) | 3.2E-13 | -0.0011 (0.0120) | 0.930 | 53.1 | 0.068 |
| Hand grip strength (right) | Alzheimer's disease (Bellenguez 2022) | rs9652468 | 15:56823913 | A/G | 0.249 | -0.0125 (0.0017) | 3.3E-13 | 0.0042 (0.0093) | 0.654 | 53.0 | -0.335 |
| Hand grip strength (right) | Alzheimer's disease (Bellenguez 2022) | rs2273555 | 10:104127171 | A/G | 0.606 | 0.0110 (0.0015) | 4.2E-13 | -0.0135 (0.0083) | 0.103 | 52.6 | -1.224 |
| Hand grip strength (right) | Alzheimer's disease (Bellenguez 2022) | rs7301953 | 12:124405871 | A/G | 0.312 | -0.0115 (0.0016) | 6.4E-13 | 0.0116 (0.0087) | 0.182 | 51.7 | -1.005 |
| Hand grip strength (right) | Alzheimer's disease (Bellenguez 2022) | rs7196917 | 16:69896527 | G/A | 0.430 | -0.0106 (0.0015) | 1.9E-12 | 0.0017 (0.0082) | 0.840 | 49.6 | -0.161 |
| Hand grip strength (right) | Alzheimer's disease (Bellenguez 2022) | rs11642954 | 16:24824248 | A/G | 0.195 | -0.0132 (0.0019) | 2.2E-12 | 0.0075 (0.0102) | 0.465 | 49.3 | -0.569 |
| Hand grip strength (right) | Alzheimer's disease (Bellenguez 2022) | rs2717351 | 7:19019880 | G/A | 0.212 | 0.0128 (0.0018) | 3.4E-12 | 0.0227 (0.0099) | 0.022 | 48.4 | 1.778 |
| Hand grip strength (right) | Alzheimer's disease (Bellenguez 2022) | rs9322822 | 6:105369598 | T/C | 0.320 | 0.0111 (0.0016) | 3.5E-12 | 0.0032 (0.0087) | 0.710 | 48.4 | 0.289 |
| Hand grip strength (right) | Alzheimer's disease (Bellenguez 2022) | rs7565148 | 2:44188396 | G/T | 0.501 | -0.0104 (0.0015) | 3.6E-12 | 0.0016 (0.0082) | 0.850 | 48.3 | -0.155 |
| Hand grip strength (right) | Alzheimer's disease (Bellenguez 2022) | rs72820369 | 10:81251539 | T/A | 0.118 | 0.0161 (0.0023) | 4.4E-12 | 0.0093 (0.0124) | 0.452 | 47.9 | 0.577 |
| Hand grip strength (right) | Alzheimer's disease (Bellenguez 2022) | rs4785574 | 16:89568875 | G/A | 0.555 | -0.0104 (0.0015) | 4.5E-12 | 0.0069 (0.0083) | 0.404 | 47.9 | -0.665 |
| Hand grip strength (right) | Alzheimer's disease (Bellenguez 2022) | rs2854152 | 17:61986027 | G/A | 0.678 | 0.0110 (0.0016) | 6.0E-12 | -0.0164 (0.0088) | 0.061 | 47.3 | -1.492 |
| Hand grip strength (right) | Alzheimer's disease (Bellenguez 2022) | rs9853018 | 3:141101961 | T/C | 0.443 | 0.0102 (0.0015) | 8.8E-12 | -0.0138 (0.0083) | 0.095 | 46.6 | -1.353 |
| Hand grip strength (right) | Alzheimer's disease (Bellenguez 2022) | rs7575451 | 2:152352843 | G/C | 0.650 | -0.0106 (0.0016) | 9.3E-12 | 0.0026 (0.0085) | 0.760 | 46.5 | -0.245 |
| Hand grip strength (right) | Alzheimer's disease (Bellenguez 2022) | rs12452505 | 17:63556402 | G/C | 0.142 | -0.0144 (0.0021) | 1.4E-11 | 0.0023 (0.0119) | 0.844 | 45.7 | -0.159 |
| Hand grip strength (right) | Alzheimer's disease (Bellenguez 2022) | rs62509875 | 8:110360944 | G/A | 0.171 | -0.0132 (0.0020) | 2.4E-11 | 0.0089 (0.0108) | 0.411 | 44.6 | -0.675 |
| Hand grip strength (right) | Alzheimer's disease (Bellenguez 2022) | rs4802848 | 19:52218342 | C/G | 0.730 | 0.0111 (0.0017) | 3.8E-11 | -0.0086 (0.0098) | 0.382 | 43.7 | -0.776 |
| Hand grip strength (right) | Alzheimer's disease (Bellenguez 2022) | rs4121165 | 1:78276977 | A/G | 0.211 | -0.0120 (0.0018) | 4.8E-11 | -0.0049 (0.0100) | 0.621 | 43.2 | 0.409 |
| Hand grip strength (right) | Alzheimer's disease (Bellenguez 2022) | rs13106087 | 4:145566864 | C/T | 0.830 | 0.0129 (0.0020) | 6.2E-11 | -0.0128 (0.0106) | 0.228 | 42.8 | -0.990 |
| Hand grip strength (right) | Alzheimer's disease (Bellenguez 2022) | rs36065733 | 19:2163771 | G/T | 0.473 | 0.0097 (0.0015) | 8.2E-11 | 0.0082 (0.0088) | 0.354 | 42.2 | 0.844 |
| Hand grip strength (right) | Alzheimer's disease (Bellenguez 2022) | rs1892425 | 1:41744821 | A/G | 0.239 | 0.0113 (0.0018) | 1.1E-10 | -0.0057 (0.0096) | 0.550 | 41.6 | -0.503 |
| Hand grip strength (right) | Alzheimer's disease (Bellenguez 2022) | rs7657558 | 4:30648636 | G/T | 0.720 | 0.0107 (0.0017) | 1.1E-10 | -0.0040 (0.0090) | 0.661 | 41.6 | -0.373 |
| Hand grip strength (right) | Alzheimer's disease (Bellenguez 2022) | rs12763284 | 10:104508202 | G/A | 0.466 | 0.0096 (0.0015) | 1.2E-10 | 0.0045 (0.0081) | 0.581 | 41.5 | 0.469 |
| Hand grip strength (right) | Alzheimer's disease (Bellenguez 2022) | rs34217742 | 19:37376830 | A/T | 0.124 | 0.0146 (0.0023) | 1.4E-10 | -0.0057 (0.0130) | 0.663 | 41.2 | -0.390 |
| Hand grip strength (right) | Alzheimer's disease (Bellenguez 2022) | rs12823922 | 12:24186697 | G/A | 0.222 | -0.0114 (0.0018) | 1.6E-10 | 0.0194 (0.0100) | 0.053 | 40.9 | -1.699 |
| Hand grip strength (right) | Alzheimer's disease (Bellenguez 2022) | rs116922558 | 9:118802375 | G/A | 0.040 | -0.0246 (0.0039) | 1.8E-10 | 0.0084 (0.0214) | 0.696 | 40.7 | -0.341 |
| Hand grip strength (right) | Alzheimer's disease (Bellenguez 2022) | rs1125 | 6:149979416 | A/G | 0.337 | -0.0100 (0.0016) | 1.9E-10 | -0.0222 (0.0086) | 0.010 | 40.6 | 2.213 |
| Hand grip strength (right) | Alzheimer's disease (Bellenguez 2022) | rs12914702 | 15:96887277 | A/G | 0.300 | 0.0108 (0.0017) | 1.9E-10 | 0.0085 (0.0104) | 0.409 | 40.5 | 0.789 |
| Hand grip strength (right) | Alzheimer's disease (Bellenguez 2022) | rs2194411 | 3:185548663 | A/G | 0.128 | 0.0143 (0.0022) | 2.1E-10 | 0.0156 (0.0120) | 0.194 | 40.4 | 1.092 |
| Hand grip strength (right) | Alzheimer's disease (Bellenguez 2022) | rs4737446 | 8:57665019 | T/G | 0.695 | 0.0103 (0.0016) | 2.4E-10 | -0.0095 (0.0088) | 0.282 | 40.1 | -0.925 |
| Hand grip strength (right) | Alzheimer's disease (Bellenguez 2022) | rs77485342 | 6:30842866 | T/C | 0.018 | 0.0353 (0.0056) | 2.6E-10 | 0.0537 (0.0326) | 0.100 | 40.0 | 1.520 |
| Hand grip strength (right) | Alzheimer's disease (Bellenguez 2022) | rs7871404 | 9:99262296 | G/A | 0.189 | 0.0119 (0.0019) | 3.4E-10 | -0.0085 (0.0104) | 0.414 | 39.4 | -0.712 |
| Hand grip strength (right) | Alzheimer's disease (Bellenguez 2022) | rs246181 | 16:14392641 | T/C | 0.373 | 0.0097 (0.0015) | 3.7E-10 | 0.0049 (0.0084) | 0.564 | 39.3 | 0.505 |
| Hand grip strength (right) | Alzheimer's disease (Bellenguez 2022) | rs56144131 | 1:208977656 | C/T | 0.149 | -0.0131 (0.0021) | 3.8E-10 | -0.0039 (0.0120) | 0.745 | 39.2 | 0.298 |
| Hand grip strength (right) | Alzheimer's disease (Bellenguez 2022) | rs11039348 | 11:47728617 | A/G | 0.348 | -0.0098 (0.0016) | 3.9E-10 | -0.0387 (0.0086) | 6.5E-06 | 39.2 | 3.962 |
| Hand grip strength (right) | Alzheimer's disease (Bellenguez 2022) | rs1952256 | 1:184035116 | G/A | 0.345 | 0.0098 (0.0016) | 4.1E-10 | 0.0010 (0.0086) | 0.909 | 39.0 | 0.102 |
| Hand grip strength (right) | Alzheimer's disease (Bellenguez 2022) | rs10193039 | 2:99692373 | T/A | 0.281 | -0.0103 (0.0017) | 4.1E-10 | -0.0152 (0.0090) | 0.091 | 39.1 | 1.472 |
| Hand grip strength (right) | Alzheimer's disease (Bellenguez 2022) | rs76895963 | 12:4384844 | G/T | 0.021 | 0.0360 (0.0058) | 4.1E-10 | -0.0069 (0.0332) | 0.836 | 39.0 | -0.192 |
| Hand grip strength (right) | Alzheimer's disease (Bellenguez 2022) | rs4553566 | 15:58336319 | C/T | 0.453 | -0.0093 (0.0015) | 4.6E-10 | -0.0015 (0.0082) | 0.851 | 38.9 | 0.161 |
| Hand grip strength (right) | Alzheimer's disease (Bellenguez 2022) | rs7266065 | 20:47531817 | A/G | 0.323 | 0.0099 (0.0016) | 4.6E-10 | 0.0006 (0.0088) | 0.946 | 38.8 | 0.060 |
| Hand grip strength (right) | Alzheimer's disease (Bellenguez 2022) | rs3848369 | 16:415078 | T/C | 0.387 | -0.0095 (0.0015) | 4.7E-10 | -0.0287 (0.0085) | 7.0E-04 | 38.8 | 3.009 |
| Hand grip strength (right) | Alzheimer's disease (Bellenguez 2022) | rs6006984 | 22:45714937 | C/T | 0.278 | 0.0103 (0.0017) | 5.1E-10 | 0.0007 (0.0090) | 0.934 | 38.6 | 0.068 |
| Hand grip strength (right) | Alzheimer's disease (Bellenguez 2022) | rs7576964 | 2:48601448 | T/G | 0.342 | 0.0097 (0.0016) | 5.3E-10 | -0.0087 (0.0087) | 0.316 | 38.6 | -0.893 |
| Hand grip strength (right) | Alzheimer's disease (Bellenguez 2022) | rs12899474 | 15:77391603 | T/C | 0.108 | -0.0149 (0.0024) | 5.4E-10 | -0.0082 (0.0137) | 0.550 | 38.5 | 0.551 |
| Hand grip strength (right) | Alzheimer's disease (Bellenguez 2022) | rs9396861 | 6:18404133 | A/C | 0.599 | -0.0096 (0.0016) | 5.7E-10 | 0.0073 (0.0085) | 0.392 | 38.4 | -0.758 |
| Hand grip strength (right) | Alzheimer's disease (Bellenguez 2022) | rs1442883 | 2:59970660 | A/C | 0.253 | -0.0106 (0.0017) | 5.8E-10 | 0.0099 (0.0095) | 0.296 | 38.4 | -0.932 |
| Hand grip strength (right) | Alzheimer's disease (Bellenguez 2022) | rs12889267 | 14:21542766 | G/A | 0.167 | -0.0123 (0.0020) | 6.7E-10 | -0.0061 (0.0117) | 0.604 | 38.1 | 0.496 |
| Hand grip strength (right) | Alzheimer's disease (Bellenguez 2022) | rs58670122 | 1:22492613 | G/A | 0.143 | -0.0132 (0.0021) | 7.4E-10 | 0.0211 (0.0115) | 0.066 | 37.9 | -1.596 |
| Hand grip strength (right) | Alzheimer's disease (Bellenguez 2022) | rs4549685 | 7:39326478 | T/C | 0.330 | 0.0097 (0.0016) | 8.1E-10 | 0.0183 (0.0086) | 0.033 | 37.8 | 1.884 |
| Hand grip strength (right) | Alzheimer's disease (Bellenguez 2022) | rs2587505 | 17:77784268 | C/T | 0.420 | -0.0093 (0.0015) | 8.1E-10 | 0.0104 (0.0084) | 0.213 | 37.8 | -1.122 |
| Hand grip strength (right) | Alzheimer's disease (Bellenguez 2022) | rs9757079 | 3:53155158 | T/C | 0.318 | 0.0098 (0.0016) | 8.4E-10 | -0.0033 (0.0088) | 0.710 | 37.7 | -0.337 |
| Hand grip strength (right) | Alzheimer's disease (Bellenguez 2022) | rs62234790 | 3:13750642 | A/C | 0.246 | 0.0106 (0.0017) | 8.8E-10 | -0.0179 (0.0094) | 0.058 | 37.6 | -1.691 |
| Hand grip strength (right) | Alzheimer's disease (Bellenguez 2022) | rs71298370 | 3:71164965 | A/G | 0.086 | 0.0166 (0.0027) | 8.9E-10 | -0.0078 (0.0152) | 0.609 | 37.6 | -0.470 |
| Hand grip strength (right) | Alzheimer's disease (Bellenguez 2022) | rs34587452 | 4:1009900 | C/G | 0.215 | -0.0111 (0.0018) | 9.1E-10 | 0.0276 (0.0101) | 0.006 | 37.5 | -2.489 |
| Hand grip strength (right) | Alzheimer's disease (Bellenguez 2022) | rs4868110 | 5:171164168 | T/A | 0.323 | -0.0097 (0.0016) | 1.0E-09 | 0.0032 (0.0087) | 0.717 | 37.3 | -0.330 |
| Hand grip strength (right) | Alzheimer's disease (Bellenguez 2022) | rs11022513 | 11:12840986 | T/C | 0.569 | -0.0092 (0.0015) | 1.1E-09 | -0.0137 (0.0083) | 0.099 | 37.2 | 1.487 |
| Hand grip strength (right) | Alzheimer's disease (Bellenguez 2022) | rs1047891 | 2:211540507 | A/C | 0.316 | 0.0097 (0.0016) | 1.3E-09 | -0.0040 (0.0088) | 0.648 | 36.8 | -0.413 |
| Hand grip strength (right) | Alzheimer's disease (Bellenguez 2022) | rs12101479 | 15:74248548 | C/G | 0.237 | -0.0106 (0.0018) | 1.5E-09 | -0.0263 (0.0097) | 0.007 | 36.6 | 2.478 |
| Hand grip strength (right) | Alzheimer's disease (Bellenguez 2022) | rs2341184 | 3:196930781 | C/T | 0.272 | 0.0101 (0.0017) | 1.7E-09 | 0.0002 (0.0092) | 0.982 | 36.2 | 0.020 |
| Hand grip strength (right) | Alzheimer's disease (Bellenguez 2022) | rs6792762 | 3:38574491 | A/G | 0.420 | -0.0091 (0.0015) | 1.8E-09 | 0.0077 (0.0083) | 0.352 | 36.2 | -0.845 |
| Hand grip strength (right) | Alzheimer's disease (Bellenguez 2022) | rs935728 | 14:80957923 | T/C | 0.328 | 0.0096 (0.0016) | 1.8E-09 | 0.0056 (0.0089) | 0.528 | 36.2 | 0.586 |
| Hand grip strength (right) | Alzheimer's disease (Bellenguez 2022) | rs2194747 | 2:218126181 | G/A | 0.707 | 0.0099 (0.0016) | 1.9E-09 | -0.0044 (0.0089) | 0.620 | 36.1 | -0.447 |
| Hand grip strength (right) | Alzheimer's disease (Bellenguez 2022) | rs16870531 | 7:120660682 | T/C | 0.238 | 0.0104 (0.0017) | 2.2E-09 | 0.0041 (0.0095) | 0.670 | 35.8 | 0.393 |
| Hand grip strength (right) | Alzheimer's disease (Bellenguez 2022) | rs6592728 | 11:77247260 | G/A | 0.371 | -0.0092 (0.0015) | 2.5E-09 | 0.0064 (0.0085) | 0.454 | 35.5 | -0.697 |
| Hand grip strength (right) | Alzheimer's disease (Bellenguez 2022) | rs1486925 | 8:78827617 | C/T | 0.315 | -0.0095 (0.0016) | 2.9E-09 | -0.0027 (0.0087) | 0.755 | 35.3 | 0.283 |
| Hand grip strength (right) | Alzheimer's disease (Bellenguez 2022) | rs6870324 | 5:141787317 | G/C | 0.270 | -0.0100 (0.0017) | 3.0E-09 | -0.0082 (0.0091) | 0.368 | 35.2 | 0.821 |
| Hand grip strength (right) | Alzheimer's disease (Bellenguez 2022) | rs2226685 | 21:40069825 | C/T | 0.759 | 0.0103 (0.0017) | 3.1E-09 | 0.0036 (0.0095) | 0.704 | 35.1 | 0.348 |
| Hand grip strength (right) | Alzheimer's disease (Bellenguez 2022) | rs34030812 | 2:144248905 | C/T | 0.367 | -0.0091 (0.0015) | 3.3E-09 | 0.0069 (0.0085) | 0.417 | 35.0 | -0.756 |
| Hand grip strength (right) | Alzheimer's disease (Bellenguez 2022) | rs10483727 | 14:61072875 | C/T | 0.610 | -0.0090 (0.0015) | 3.3E-09 | 0.0120 (0.0083) | 0.147 | 35.0 | -1.331 |
| Hand grip strength (right) | Alzheimer's disease (Bellenguez 2022) | rs61389091 | 11:74427921 | T/C | 0.042 | 0.0221 (0.0037) | 3.6E-09 | 0.0154 (0.0203) | 0.448 | 34.8 | 0.698 |
| Hand grip strength (right) | Alzheimer's disease (Bellenguez 2022) | rs997850 | 4:154838434 | C/G | 0.605 | -0.0090 (0.0015) | 3.7E-09 | -0.0189 (0.0084) | 0.024 | 34.8 | 2.095 |
| Hand grip strength (right) | Alzheimer's disease (Bellenguez 2022) | rs2322754 | 6:81046299 | A/G | 0.832 | -0.0117 (0.0020) | 3.8E-09 | 0.0130 (0.0108) | 0.229 | 34.7 | -1.111 |
| Hand grip strength (right) | Alzheimer's disease (Bellenguez 2022) | rs56074046 | 17:7358930 | A/G | 0.372 | -0.0090 (0.0015) | 4.4E-09 | -0.0019 (0.0084) | 0.824 | 34.4 | 0.210 |
| Hand grip strength (right) | Alzheimer's disease (Bellenguez 2022) | rs12052508 | 2:72757843 | T/C | 0.879 | -0.0134 (0.0023) | 4.7E-09 | 0.0009 (0.0126) | 0.943 | 34.3 | -0.067 |
| Hand grip strength (right) | Alzheimer's disease (Bellenguez 2022) | rs7148603 | 14:36683779 | A/G | 0.359 | 0.0093 (0.0016) | 4.7E-09 | 0.0126 (0.0086) | 0.144 | 34.3 | 1.354 |
| Hand grip strength (right) | Alzheimer's disease (Bellenguez 2022) | rs7034200 | 9:4289050 | A/C | 0.480 | 0.0087 (0.0015) | 5.3E-09 | 0.0100 (0.0081) | 0.213 | 34.1 | 1.151 |
| Hand grip strength (right) | Alzheimer's disease (Bellenguez 2022) | rs12616285 | 2:169156907 | G/T | 0.149 | 0.0122 (0.0021) | 5.4E-09 | 0.0097 (0.0112) | 0.385 | 34.0 | 0.794 |
| Hand grip strength (right) | Alzheimer's disease (Bellenguez 2022) | rs6473015 | 8:78178485 | C/A | 0.286 | 0.0096 (0.0016) | 5.5E-09 | -0.0140 (0.0090) | 0.120 | 34.0 | -1.458 |
| Hand grip strength (right) | Alzheimer's disease (Bellenguez 2022) | rs7549184 | 1:215412906 | A/G | 0.787 | 0.0106 (0.0018) | 5.8E-09 | -0.0116 (0.0098) | 0.236 | 33.9 | -1.098 |
| Hand grip strength (right) | Alzheimer's disease (Bellenguez 2022) | rs2147461 | 1:191051627 | C/T | 0.117 | 0.0134 (0.0023) | 6.1E-09 | -0.0001 (0.0127) | 0.995 | 33.8 | -0.007 |
| Hand grip strength (right) | Alzheimer's disease (Bellenguez 2022) | rs6693567 | 1:150510660 | T/C | 0.733 | -0.0097 (0.0017) | 6.3E-09 | 0.0027 (0.0092) | 0.773 | 33.8 | -0.277 |
| Hand grip strength (right) | Alzheimer's disease (Bellenguez 2022) | rs4752689 | 10:124131176 | A/G | 0.584 | 0.0088 (0.0015) | 6.4E-09 | -0.0258 (0.0082) | 0.002 | 33.7 | -2.948 |
| Hand grip strength (right) | Alzheimer's disease (Bellenguez 2022) | rs12522139 | 5:122699812 | G/T | 0.171 | -0.0114 (0.0020) | 6.8E-09 | -0.0026 (0.0109) | 0.809 | 33.6 | 0.227 |
| Hand grip strength (right) | Alzheimer's disease (Bellenguez 2022) | rs6882168 | 5:39402647 | T/C | 0.337 | -0.0091 (0.0016) | 7.9E-09 | -0.0075 (0.0088) | 0.395 | 33.3 | 0.825 |
| Hand grip strength (right) | Alzheimer's disease (Bellenguez 2022) | rs114924396 | 4:119755621 | G/A | 0.054 | -0.0191 (0.0033) | 8.2E-09 | 0.0465 (0.0187) | 0.013 | 33.2 | -2.431 |
| Hand grip strength (right) | Alzheimer's disease (Bellenguez 2022) | rs248831 | 16:11281218 | A/G | 0.265 | 0.0099 (0.0017) | 8.4E-09 | -0.0185 (0.0093) | 0.047 | 33.2 | -1.871 |
| Hand grip strength (right) | Alzheimer's disease (Bellenguez 2022) | rs35833641 | 2:179462494 | G/A | 0.312 | 0.0092 (0.0016) | 8.5E-09 | -0.0134 (0.0089) | 0.130 | 33.1 | -1.453 |
| Hand grip strength (right) | Alzheimer's disease (Bellenguez 2022) | rs6715064 | 2:220041928 | T/C | 0.310 | -0.0092 (0.0016) | 9.6E-09 | 0.0037 (0.0088) | 0.669 | 32.9 | -0.402 |
| Hand grip strength (right) | Alzheimer's disease (Bellenguez 2022) | rs2894602 | 2:227249802 | G/A | 0.766 | 0.0101 (0.0018) | 9.9E-09 | -0.0024 (0.0099) | 0.808 | 32.9 | -0.237 |
| Hand grip strength (right) | Alzheimer's disease (Bellenguez 2022) | rs113835839 | 6:13784625 | T/C | 0.248 | -0.0099 (0.0017) | 1.1E-08 | 0.0050 (0.0095) | 0.595 | 32.7 | -0.507 |
| Hand grip strength (right) | Alzheimer's disease (Bellenguez 2022) | rs10278546 | 7:100516003 | C/A | 0.195 | 0.0108 (0.0019) | 1.1E-08 | -0.0126 (0.0103) | 0.223 | 32.6 | -1.172 |
| Hand grip strength (right) | Alzheimer's disease (Bellenguez 2022) | rs7790322 | 7:2830498 | T/C | 0.416 | -0.0086 (0.0015) | 1.2E-08 | 0.0061 (0.0082) | 0.462 | 32.5 | -0.709 |
| Hand grip strength (right) | Alzheimer's disease (Bellenguez 2022) | rs4962700 | 10:126479989 | G/C | 0.302 | 0.0093 (0.0016) | 1.2E-08 | -0.0233 (0.0090) | 0.010 | 32.5 | -2.498 |
| Hand grip strength (right) | Alzheimer's disease (Bellenguez 2022) | rs8055199 | 16:84867404 | A/G | 0.660 | -0.0090 (0.0016) | 1.2E-08 | -0.0076 (0.0086) | 0.379 | 32.5 | 0.848 |
| Hand grip strength (right) | Alzheimer's disease (Bellenguez 2022) | rs2362972 | 3:158163272 | A/C | 0.578 | -0.0086 (0.0015) | 1.3E-08 | 0.0072 (0.0082) | 0.382 | 32.3 | -0.840 |
| Hand grip strength (right) | Alzheimer's disease (Bellenguez 2022) | rs10761411 | 9:136973826 | T/C | 0.812 | -0.0115 (0.0020) | 1.3E-08 | -0.0264 (0.0107) | 0.014 | 32.4 | 2.296 |
| Hand grip strength (right) | Alzheimer's disease (Bellenguez 2022) | rs1840753 | 2:199060177 | T/C | 0.063 | 0.0177 (0.0031) | 1.4E-08 | 0.0025 (0.0168) | 0.883 | 32.1 | 0.141 |
| Hand grip strength (right) | Alzheimer's disease (Bellenguez 2022) | rs10770125 | 11:2169014 | G/A | 0.477 | 0.0084 (0.0015) | 1.4E-08 | 0.0086 (0.0082) | 0.293 | 32.2 | 1.019 |
| Hand grip strength (right) | Alzheimer's disease (Bellenguez 2022) | rs35701422 | 3:85575775 | C/T | 0.626 | -0.0087 (0.0015) | 1.5E-08 | -0.0001 (0.0084) | 0.987 | 32.1 | 0.012 |
| Hand grip strength (right) | Alzheimer's disease (Bellenguez 2022) | rs600038 | 9:136151806 | C/T | 0.207 | -0.0104 (0.0018) | 1.5E-08 | -0.0021 (0.0097) | 0.830 | 32.0 | 0.202 |
| Hand grip strength (right) | Alzheimer's disease (Bellenguez 2022) | rs721101 | 6:155632844 | C/T | 0.271 | 0.0095 (0.0017) | 1.6E-08 | 0.0059 (0.0095) | 0.534 | 32.0 | 0.623 |
| Hand grip strength (right) | Alzheimer's disease (Bellenguez 2022) | rs79723785 | 19:55818225 | C/T | 0.016 | -0.0340 (0.0060) | 1.6E-08 | -0.0504 (0.0335) | 0.132 | 31.9 | 1.480 |
| Hand grip strength (right) | Alzheimer's disease (Bellenguez 2022) | rs112330055 | 7:23109316 | A/G | 0.063 | 0.0180 (0.0032) | 1.7E-08 | -0.0044 (0.0173) | 0.797 | 31.8 | -0.245 |
| Hand grip strength (right) | Alzheimer's disease (Bellenguez 2022) | rs34845616 | 11:133792644 | A/G | 0.246 | 0.0098 (0.0017) | 1.7E-08 | 0.0234 (0.0096) | 0.015 | 31.8 | 2.389 |
| Hand grip strength (right) | Alzheimer's disease (Bellenguez 2022) | rs12562146 | 1:86247267 | A/T | 0.145 | 0.0119 (0.0021) | 2.0E-08 | -0.0129 (0.0112) | 0.252 | 31.5 | -1.087 |
| Hand grip strength (right) | Alzheimer's disease (Bellenguez 2022) | rs6962338 | 7:69160985 | G/A | 0.044 | -0.0203 (0.0036) | 2.0E-08 | 0.0398 (0.0209) | 0.056 | 31.5 | -1.956 |
| Hand grip strength (right) | Alzheimer's disease (Bellenguez 2022) | rs7214252 | 17:27486673 | A/G | 0.211 | -0.0102 (0.0018) | 2.0E-08 | -0.0033 (0.0100) | 0.742 | 31.5 | 0.322 |
| Hand grip strength (right) | Alzheimer's disease (Bellenguez 2022) | rs911642 | 20:13260252 | T/C | 0.376 | 0.0086 (0.0015) | 2.0E-08 | 0.0077 (0.0085) | 0.366 | 31.5 | 0.892 |
| Hand grip strength (right) | Alzheimer's disease (Bellenguez 2022) | rs35457492 | 3:70194564 | C/A | 0.495 | 0.0083 (0.0015) | 2.2E-08 | 0.0046 (0.0082) | 0.577 | 31.3 | 0.552 |
| Hand grip strength (right) | Alzheimer's disease (Bellenguez 2022) | rs76749769 | 16:3291408 | T/C | 0.091 | 0.0144 (0.0026) | 2.2E-08 | -0.0176 (0.0147) | 0.233 | 31.3 | -1.220 |
| Hand grip strength (right) | Alzheimer's disease (Bellenguez 2022) | rs4768725 | 12:46848478 | C/T | 0.700 | 0.0091 (0.0016) | 2.3E-08 | -0.0098 (0.0087) | 0.262 | 31.2 | -1.082 |
| Hand grip strength (right) | Alzheimer's disease (Bellenguez 2022) | rs9388051 | 6:122577108 | A/G | 0.186 | 0.0106 (0.0019) | 2.7E-08 | -0.0027 (0.0107) | 0.801 | 30.9 | -0.254 |
| Hand grip strength (right) | Alzheimer's disease (Bellenguez 2022) | rs1885690 | 6:109672998 | A/C | 0.410 | -0.0084 (0.0015) | 2.8E-08 | 0.0149 (0.0082) | 0.072 | 30.9 | -1.774 |
| Hand grip strength (right) | Alzheimer's disease (Bellenguez 2022) | rs11998884 | 9:33684436 | T/C | 0.062 | 0.0173 (0.0031) | 2.8E-08 | -0.0375 (0.0173) | 0.031 | 30.8 | -2.162 |
| Hand grip strength (right) | Alzheimer's disease (Bellenguez 2022) | rs12412806 | 10:24860913 | A/G | 0.294 | -0.0091 (0.0016) | 3.0E-08 | 0.0101 (0.0091) | 0.268 | 30.7 | -1.110 |
| Hand grip strength (right) | Alzheimer's disease (Bellenguez 2022) | rs75457267 | 5:102658770 | T/C | 0.051 | -0.0187 (0.0034) | 3.4E-08 | -0.0235 (0.0202) | 0.244 | 30.4 | 1.255 |
| Hand grip strength (right) | Alzheimer's disease (Bellenguez 2022) | rs13355365 | 5:154942606 | T/C | 0.380 | -0.0085 (0.0015) | 3.4E-08 | -0.0040 (0.0084) | 0.633 | 30.5 | 0.472 |
| Hand grip strength (right) | Alzheimer's disease (Bellenguez 2022) | rs2389763 | 7:17307847 | C/T | 0.592 | -0.0083 (0.0015) | 3.8E-08 | 0.0009 (0.0082) | 0.912 | 30.3 | -0.108 |
| Hand grip strength (right) | Alzheimer's disease (Bellenguez 2022) | rs4793658 | 17:45878733 | C/A | 0.110 | -0.0137 (0.0025) | 3.8E-08 | -0.0006 (0.0135) | 0.967 | 30.2 | 0.044 |
| Hand grip strength (right) | Alzheimer's disease (Bellenguez 2022) | rs35304341 | 1:200971049 | A/G | 0.089 | -0.0143 (0.0026) | 4.0E-08 | -0.0267 (0.0145) | 0.066 | 30.1 | 1.869 |
| Hand grip strength (right) | Alzheimer's disease (Bellenguez 2022) | rs7249 | 19:18391171 | T/C | 0.366 | 0.0085 (0.0015) | 4.0E-08 | -0.0106 (0.0090) | 0.240 | 30.2 | -1.250 |
| Hand grip strength (right) | Alzheimer's disease (Bellenguez 2022) | rs4751671 | 10:116138744 | A/G | 0.531 | 0.0082 (0.0015) | 4.1E-08 | 0.0074 (0.0083) | 0.371 | 30.1 | 0.899 |
| Hand grip strength (right) | Alzheimer's disease (Bellenguez 2022) | rs13169333 | 5:153064994 | C/T | 0.260 | 0.0093 (0.0017) | 4.3E-08 | -0.0002 (0.0093) | 0.982 | 30.0 | -0.022 |
| Hand grip strength (right) | Alzheimer's disease (Bellenguez 2022) | rs645144 | 6:141180780 | C/T | 0.330 | -0.0087 (0.0016) | 4.3E-08 | 0.0034 (0.0087) | 0.695 | 30.0 | -0.391 |
| Hand grip strength (right) | Alzheimer's disease (Bellenguez 2022) | rs113851275 | 9:98297220 | A/G | 0.108 | 0.0131 (0.0024) | 4.3E-08 | 0.0189 (0.0132) | 0.150 | 30.0 | 1.438 |
| Hand grip strength (right) | Alzheimer's disease (Bellenguez 2022) | rs2244621 | 11:64026219 | T/C | 0.144 | 0.0116 (0.0021) | 4.5E-08 | -0.0018 (0.0112) | 0.870 | 29.9 | -0.155 |
| Hand grip strength (right) | Alzheimer's disease (Bellenguez 2022) | rs2296316 | 14:65520246 | C/T | 0.464 | -0.0082 (0.0015) | 4.6E-08 | 0.0045 (0.0082) | 0.584 | 29.9 | -0.548 |
| Hand grip strength (right) | All-cause dementia (FinnGen) | rs143384 | 20:34025756 | G/A | 0.404 | 0.0230 (0.0015) | 2.5E-52 | -0.0099 (0.0193) | 0.610 | 231.7 | -0.430 |
| Hand grip strength (right) | All-cause dementia (FinnGen) | rs1556659 | 10:130834698 | T/C | 0.382 | 0.0175 (0.0015) | 3.8E-30 | -0.0031 (0.0210) | 0.882 | 130.2 | -0.177 |
| Hand grip strength (right) | All-cause dementia (FinnGen) | rs3118914 | 13:51116901 | T/G | 0.215 | -0.0194 (0.0018) | 5.9E-27 | -0.0221 (0.0220) | 0.313 | 115.6 | 1.137 |
| Hand grip strength (right) | All-cause dementia (FinnGen) | rs12316046 | 12:15054415 | G/A | 0.378 | -0.0162 (0.0015) | 3.8E-26 | -0.0068 (0.0215) | 0.750 | 111.9 | 0.420 |
| Hand grip strength (right) | All-cause dementia (FinnGen) | rs10846071 | 12:15016236 | T/C | 0.394 | -0.0157 (0.0015) | 6.5E-25 | -0.0085 (0.0208) | 0.684 | 106.2 | 0.543 |
| Hand grip strength (right) | All-cause dementia (FinnGen) | rs13146142 | 4:17931318 | C/T | 0.159 | -0.0208 (0.0020) | 1.4E-24 | 0.0438 (0.0325) | 0.178 | 104.8 | -2.106 |
| Hand grip strength (right) | All-cause dementia (FinnGen) | rs2871865 | 15:99194896 | G/C | 0.116 | -0.0237 (0.0023) | 1.8E-24 | 0.0616 (0.0398) | 0.122 | 104.2 | -2.601 |
| Hand grip strength (right) | All-cause dementia (FinnGen) | rs9267806 | 6:32110886 | A/G | 0.256 | -0.0167 (0.0017) | 7.7E-23 | -0.0325 (0.0204) | 0.110 | 96.8 | 1.941 |
| Hand grip strength (right) | All-cause dementia (FinnGen) | rs7451021 | 6:130381246 | C/T | 0.689 | -0.0158 (0.0016) | 7.7E-23 | 0.0467 (0.0205) | 0.023 | 96.8 | -2.956 |
| Hand grip strength (right) | All-cause dementia (FinnGen) | rs12790261 | 11:66988048 | A/C | 0.082 | -0.0264 (0.0027) | 2.0E-22 | 0.0165 (0.0437) | 0.706 | 94.9 | -0.626 |
| Hand grip strength (right) | All-cause dementia (FinnGen) | rs13107325 | 4:103188709 | T/C | 0.075 | -0.0275 (0.0028) | 2.1E-22 | 0.1247 (0.0827) | 0.131 | 94.8 | -4.535 |
| Hand grip strength (right) | All-cause dementia (FinnGen) | rs10798483 | 1:176799143 | A/G | 0.547 | 0.0145 (0.0015) | 2.8E-22 | -0.0093 (0.0192) | 0.627 | 94.2 | -0.641 |
| Hand grip strength (right) | All-cause dementia (FinnGen) | rs3771498 | 2:70720070 | T/C | 0.514 | 0.0142 (0.0015) | 1.9E-21 | -0.0174 (0.0191) | 0.361 | 90.5 | -1.229 |
| Hand grip strength (right) | All-cause dementia (FinnGen) | rs4369779 | 18:20735408 | C/T | 0.789 | 0.0172 (0.0018) | 3.4E-21 | 0.0371 (0.0228) | 0.104 | 89.3 | 2.157 |
| Hand grip strength (right) | All-cause dementia (FinnGen) | rs1043515 | 17:36922196 | G/A | 0.566 | 0.0138 (0.0015) | 2.8E-20 | 0.0155 (0.0192) | 0.420 | 85.2 | 1.121 |
| Hand grip strength (right) | All-cause dementia (FinnGen) | rs1550115 | 2:25041620 | T/C | 0.749 | 0.0153 (0.0017) | 4.5E-19 | -0.0204 (0.0226) | 0.366 | 79.6 | -1.335 |
| Hand grip strength (right) | All-cause dementia (FinnGen) | rs4784329 | 16:53910261 | C/A | 0.426 | -0.0133 (0.0015) | 9.0E-19 | 0.0210 (0.0191) | 0.272 | 78.3 | -1.575 |
| Hand grip strength (right) | All-cause dementia (FinnGen) | rs12473839 | 2:135638044 | T/C | 0.371 | 0.0133 (0.0015) | 4.0E-18 | 0.0346 (0.0196) | 0.077 | 75.3 | 2.607 |
| Hand grip strength (right) | All-cause dementia (FinnGen) | rs4927015 | 1:54072471 | A/G | 0.583 | 0.0131 (0.0015) | 5.0E-18 | 0.0126 (0.0204) | 0.538 | 74.9 | 0.965 |
| Hand grip strength (right) | All-cause dementia (FinnGen) | rs635538 | 18:53273614 | A/G | 0.914 | -0.0220 (0.0027) | 1.5E-16 | 0.0310 (0.0315) | 0.325 | 68.2 | -1.409 |
| Hand grip strength (right) | All-cause dementia (FinnGen) | rs823130 | 1:205714372 | T/C | 0.433 | -0.0124 (0.0015) | 1.7E-16 | 0.0210 (0.0193) | 0.277 | 67.9 | -1.695 |
| Hand grip strength (right) | All-cause dementia (FinnGen) | rs2165241 | 15:74222202 | C/T | 0.509 | 0.0122 (0.0015) | 1.9E-16 | 0.0215 (0.0190) | 0.258 | 67.7 | 1.755 |
| Hand grip strength (right) | All-cause dementia (FinnGen) | rs6977081 | 7:150542515 | T/G | 0.334 | 0.0129 (0.0016) | 5.7E-16 | 0.0096 (0.0200) | 0.631 | 65.5 | 0.747 |
| Hand grip strength (right) | All-cause dementia (FinnGen) | rs56365901 | 17:43960323 | G/A | 0.223 | -0.0143 (0.0018) | 1.8E-15 | -0.0954 (0.0353) | 0.007 | 63.3 | 6.689 |
| Hand grip strength (right) | All-cause dementia (FinnGen) | rs7206195 | 16:2145280 | T/C | 0.180 | -0.0153 (0.0019) | 2.8E-15 | 0.0285 (0.0233) | 0.220 | 62.4 | -1.860 |
| Hand grip strength (right) | All-cause dementia (FinnGen) | rs10520770 | 18:46602964 | C/T | 0.449 | 0.0117 (0.0015) | 4.5E-15 | 0.0220 (0.0205) | 0.285 | 61.5 | 1.874 |
| Hand grip strength (right) | All-cause dementia (FinnGen) | rs11243202 | 6:7719065 | C/T | 0.486 | 0.0116 (0.0015) | 6.5E-15 | 0.0058 (0.0192) | 0.764 | 60.8 | 0.498 |
| Hand grip strength (right) | All-cause dementia (FinnGen) | rs150330307 | 1:160160801 | C/T | 0.032 | -0.0326 (0.0042) | 1.2E-14 | -0.0502 (0.0691) | 0.468 | 59.5 | 1.541 |
| Hand grip strength (right) | All-cause dementia (FinnGen) | rs2208562 | 9:119344528 | T/C | 0.610 | -0.0116 (0.0015) | 3.1E-14 | -0.0395 (0.0200) | 0.049 | 57.7 | 3.416 |
| Hand grip strength (right) | All-cause dementia (FinnGen) | rs10799428 | 1:227798565 | T/C | 0.187 | -0.0144 (0.0019) | 4.6E-14 | -0.0248 (0.0241) | 0.302 | 56.9 | 1.727 |
| Hand grip strength (right) | All-cause dementia (FinnGen) | rs2631360 | 5:131707429 | A/G | 0.519 | -0.0112 (0.0015) | 5.4E-14 | 0.0336 (0.0202) | 0.096 | 56.6 | -3.005 |
| Hand grip strength (right) | All-cause dementia (FinnGen) | rs10784502 | 12:66343810 | T/C | 0.512 | -0.0111 (0.0015) | 7.1E-14 | -0.0206 (0.0191) | 0.282 | 56.0 | 1.852 |
| Hand grip strength (right) | All-cause dementia (FinnGen) | rs7963801 | 12:79685226 | C/T | 0.572 | -0.0113 (0.0015) | 8.4E-14 | -0.0059 (0.0195) | 0.761 | 55.7 | 0.523 |
| Hand grip strength (right) | All-cause dementia (FinnGen) | rs2431112 | 5:103931707 | A/G | 0.441 | -0.0112 (0.0015) | 8.6E-14 | 0.0146 (0.0196) | 0.455 | 55.7 | -1.307 |
| Hand grip strength (right) | All-cause dementia (FinnGen) | rs1641457 | 2:40421990 | G/T | 0.223 | 0.0131 (0.0018) | 2.4E-13 | 0.0049 (0.0210) | 0.817 | 53.6 | 0.375 |
| Hand grip strength (right) | All-cause dementia (FinnGen) | rs6693965 | 1:10378416 | T/G | 0.128 | -0.0163 (0.0022) | 3.2E-13 | 0.0212 (0.0307) | 0.488 | 53.1 | -1.305 |
| Hand grip strength (right) | All-cause dementia (FinnGen) | rs9652468 | 15:56823913 | A/G | 0.249 | -0.0125 (0.0017) | 3.3E-13 | 0.0263 (0.0207) | 0.203 | 53.0 | -2.099 |
| Hand grip strength (right) | All-cause dementia (FinnGen) | rs2273555 | 10:104127171 | A/G | 0.606 | 0.0110 (0.0015) | 4.2E-13 | 0.0283 (0.0195) | 0.147 | 52.6 | 2.565 |
| Hand grip strength (right) | All-cause dementia (FinnGen) | rs7301953 | 12:124405871 | A/G | 0.312 | -0.0115 (0.0016) | 6.4E-13 | -0.0085 (0.0211) | 0.687 | 51.7 | 0.737 |
| Hand grip strength (right) | All-cause dementia (FinnGen) | rs7196917 | 16:69896527 | G/A | 0.430 | -0.0106 (0.0015) | 1.9E-12 | 0.0238 (0.0197) | 0.225 | 49.6 | -2.249 |
| Hand grip strength (right) | All-cause dementia (FinnGen) | rs2717351 | 7:19019880 | G/A | 0.212 | 0.0128 (0.0018) | 3.4E-12 | 0.0388 (0.0219) | 0.076 | 48.4 | 3.038 |
| Hand grip strength (right) | All-cause dementia (FinnGen) | rs9322822 | 6:105369598 | T/C | 0.320 | 0.0111 (0.0016) | 3.5E-12 | 0.0215 (0.0206) | 0.296 | 48.4 | 1.940 |
| Hand grip strength (right) | All-cause dementia (FinnGen) | rs7565148 | 2:44188396 | G/T | 0.501 | -0.0104 (0.0015) | 3.6E-12 | 0.0462 (0.0192) | 0.016 | 48.3 | -4.463 |
| Hand grip strength (right) | All-cause dementia (FinnGen) | rs72820369 | 10:81251539 | T/A | 0.118 | 0.0161 (0.0023) | 4.4E-12 | -0.0202 (0.0272) | 0.458 | 47.9 | -1.253 |
| Hand grip strength (right) | All-cause dementia (FinnGen) | rs4785574 | 16:89568875 | G/A | 0.555 | -0.0104 (0.0015) | 4.5E-12 | -0.0314 (0.0195) | 0.108 | 47.9 | 3.026 |
| Hand grip strength (right) | All-cause dementia (FinnGen) | rs2854152 | 17:61986027 | G/A | 0.678 | 0.0110 (0.0016) | 6.0E-12 | 0.0153 (0.0198) | 0.440 | 47.3 | 1.392 |
| Hand grip strength (right) | All-cause dementia (FinnGen) | rs9853018 | 3:141101961 | T/C | 0.443 | 0.0102 (0.0015) | 8.8E-12 | 0.0504 (0.0192) | 0.009 | 46.6 | 4.942 |
| Hand grip strength (right) | All-cause dementia (FinnGen) | rs7575451 | 2:152352843 | G/C | 0.650 | -0.0106 (0.0016) | 9.3E-12 | -0.0005 (0.0205) | 0.979 | 46.5 | 0.047 |
| Hand grip strength (right) | All-cause dementia (FinnGen) | rs12452505 | 17:63556402 | G/C | 0.142 | -0.0144 (0.0021) | 1.4E-11 | 0.0056 (0.0320) | 0.861 | 45.7 | -0.388 |
| Hand grip strength (right) | All-cause dementia (FinnGen) | rs62509875 | 8:110360944 | G/A | 0.171 | -0.0132 (0.0020) | 2.4E-11 | -0.0298 (0.0280) | 0.287 | 44.6 | 2.261 |
| Hand grip strength (right) | All-cause dementia (FinnGen) | rs4802848 | 19:52218342 | C/G | 0.730 | 0.0111 (0.0017) | 3.8E-11 | 0.0412 (0.0240) | 0.085 | 43.7 | 3.720 |
| Hand grip strength (right) | All-cause dementia (FinnGen) | rs4121165 | 1:78276977 | A/G | 0.211 | -0.0120 (0.0018) | 4.8E-11 | 0.0346 (0.0254) | 0.173 | 43.2 | -2.888 |
| Hand grip strength (right) | All-cause dementia (FinnGen) | rs13106087 | 4:145566864 | C/T | 0.830 | 0.0129 (0.0020) | 6.2E-11 | 0.0139 (0.0257) | 0.588 | 42.8 | 1.075 |
| Hand grip strength (right) | All-cause dementia (FinnGen) | rs1892425 | 1:41744821 | A/G | 0.239 | 0.0113 (0.0018) | 1.1E-10 | -0.0123 (0.0221) | 0.578 | 41.6 | -1.085 |
| Hand grip strength (right) | All-cause dementia (FinnGen) | rs7657558 | 4:30648636 | G/T | 0.720 | 0.0107 (0.0017) | 1.1E-10 | -0.0075 (0.0216) | 0.728 | 41.6 | -0.699 |
| Hand grip strength (right) | All-cause dementia (FinnGen) | rs12763284 | 10:104508202 | G/A | 0.466 | 0.0096 (0.0015) | 1.2E-10 | -0.0023 (0.0192) | 0.904 | 41.5 | -0.240 |
| Hand grip strength (right) | All-cause dementia (FinnGen) | rs34217742 | 19:37376830 | A/T | 0.124 | 0.0146 (0.0023) | 1.4E-10 | 0.0370 (0.0275) | 0.179 | 41.2 | 2.530 |
| Hand grip strength (right) | All-cause dementia (FinnGen) | rs12823922 | 12:24186697 | G/A | 0.222 | -0.0114 (0.0018) | 1.6E-10 | -0.0071 (0.0225) | 0.752 | 40.9 | 0.622 |
| Hand grip strength (right) | All-cause dementia (FinnGen) | rs116922558 | 9:118802375 | G/A | 0.040 | -0.0246 (0.0039) | 1.8E-10 | 0.0872 (0.0459) | 0.057 | 40.7 | -3.541 |
| Hand grip strength (right) | All-cause dementia (FinnGen) | rs1125 | 6:149979416 | A/G | 0.337 | -0.0100 (0.0016) | 1.9E-10 | -0.0062 (0.0198) | 0.755 | 40.6 | 0.618 |
| Hand grip strength (right) | All-cause dementia (FinnGen) | rs12914702 | 15:96887277 | A/G | 0.300 | 0.0108 (0.0017) | 1.9E-10 | -0.0145 (0.0206) | 0.482 | 40.5 | -1.345 |
| Hand grip strength (right) | All-cause dementia (FinnGen) | rs2194411 | 3:185548663 | A/G | 0.128 | 0.0143 (0.0022) | 2.1E-10 | 0.0117 (0.0280) | 0.676 | 40.4 | 0.819 |
| Hand grip strength (right) | All-cause dementia (FinnGen) | rs4737446 | 8:57665019 | T/G | 0.695 | 0.0103 (0.0016) | 2.4E-10 | -0.0326 (0.0205) | 0.112 | 40.1 | -3.175 |
| Hand grip strength (right) | All-cause dementia (FinnGen) | rs77485342 | 6:30842866 | T/C | 0.018 | 0.0353 (0.0056) | 2.6E-10 | 0.0629 (0.0605) | 0.298 | 40.0 | 1.780 |
| Hand grip strength (right) | All-cause dementia (FinnGen) | rs7871404 | 9:99262296 | G/A | 0.189 | 0.0119 (0.0019) | 3.4E-10 | -0.0478 (0.0260) | 0.066 | 39.4 | -4.005 |
| Hand grip strength (right) | All-cause dementia (FinnGen) | rs246181 | 16:14392641 | T/C | 0.373 | 0.0097 (0.0015) | 3.7E-10 | -0.0035 (0.0196) | 0.858 | 39.3 | -0.361 |
| Hand grip strength (right) | All-cause dementia (FinnGen) | rs56144131 | 1:208977656 | C/T | 0.149 | -0.0131 (0.0021) | 3.8E-10 | 0.0117 (0.0264) | 0.659 | 39.2 | -0.895 |
| Hand grip strength (right) | All-cause dementia (FinnGen) | rs11039348 | 11:47728617 | A/G | 0.348 | -0.0098 (0.0016) | 3.9E-10 | -0.0288 (0.0207) | 0.164 | 39.2 | 2.948 |
| Hand grip strength (right) | All-cause dementia (FinnGen) | rs1952256 | 1:184035116 | G/A | 0.345 | 0.0098 (0.0016) | 4.1E-10 | 0.0206 (0.0195) | 0.291 | 39.0 | 2.109 |
| Hand grip strength (right) | All-cause dementia (FinnGen) | rs10193039 | 2:99692373 | T/A | 0.281 | -0.0103 (0.0017) | 4.1E-10 | -0.0036 (0.0219) | 0.871 | 39.1 | 0.349 |
| Hand grip strength (right) | All-cause dementia (FinnGen) | rs76895963 | 12:4384844 | G/T | 0.021 | 0.0360 (0.0058) | 4.1E-10 | -0.0735 (0.0577) | 0.203 | 39.0 | -2.043 |
| Hand grip strength (right) | All-cause dementia (FinnGen) | rs4553566 | 15:58336319 | C/T | 0.453 | -0.0093 (0.0015) | 4.6E-10 | -0.0021 (0.0191) | 0.915 | 38.9 | 0.226 |
| Hand grip strength (right) | All-cause dementia (FinnGen) | rs7266065 | 20:47531817 | A/G | 0.323 | 0.0099 (0.0016) | 4.6E-10 | 0.0278 (0.0214) | 0.194 | 38.8 | 2.797 |
| Hand grip strength (right) | All-cause dementia (FinnGen) | rs3848369 | 16:415078 | T/C | 0.387 | -0.0095 (0.0015) | 4.7E-10 | -0.0382 (0.0195) | 0.050 | 38.8 | 4.005 |
| Hand grip strength (right) | All-cause dementia (FinnGen) | rs6006984 | 22:45714937 | C/T | 0.278 | 0.0103 (0.0017) | 5.1E-10 | -0.0015 (0.0210) | 0.942 | 38.6 | -0.146 |
| Hand grip strength (right) | All-cause dementia (FinnGen) | rs7576964 | 2:48601448 | T/G | 0.342 | 0.0097 (0.0016) | 5.3E-10 | 0.0123 (0.0196) | 0.530 | 38.6 | 1.262 |
| Hand grip strength (right) | All-cause dementia (FinnGen) | rs12899474 | 15:77391603 | T/C | 0.108 | -0.0149 (0.0024) | 5.4E-10 | -0.0342 (0.0321) | 0.286 | 38.5 | 2.296 |
| Hand grip strength (right) | All-cause dementia (FinnGen) | rs9396861 | 6:18404133 | A/C | 0.599 | -0.0096 (0.0016) | 5.7E-10 | 0.0509 (0.0223) | 0.023 | 38.4 | -5.286 |
| Hand grip strength (right) | All-cause dementia (FinnGen) | rs1442883 | 2:59970660 | A/C | 0.253 | -0.0106 (0.0017) | 5.8E-10 | -0.0115 (0.0232) | 0.619 | 38.4 | 1.083 |
| Hand grip strength (right) | All-cause dementia (FinnGen) | rs12889267 | 14:21542766 | G/A | 0.167 | -0.0123 (0.0020) | 6.7E-10 | 0.0130 (0.0293) | 0.656 | 38.1 | -1.058 |
| Hand grip strength (right) | All-cause dementia (FinnGen) | rs58670122 | 1:22492613 | G/A | 0.143 | -0.0132 (0.0021) | 7.4E-10 | 0.0514 (0.0316) | 0.103 | 37.9 | -3.887 |
| Hand grip strength (right) | All-cause dementia (FinnGen) | rs4549685 | 7:39326478 | T/C | 0.330 | 0.0097 (0.0016) | 8.1E-10 | -0.0043 (0.0204) | 0.833 | 37.8 | -0.443 |
| Hand grip strength (right) | All-cause dementia (FinnGen) | rs2587505 | 17:77784268 | C/T | 0.420 | -0.0093 (0.0015) | 8.1E-10 | 0.0219 (0.0204) | 0.282 | 37.8 | -2.362 |
| Hand grip strength (right) | All-cause dementia (FinnGen) | rs9757079 | 3:53155158 | T/C | 0.318 | 0.0098 (0.0016) | 8.4E-10 | -0.0016 (0.0206) | 0.940 | 37.7 | -0.163 |
| Hand grip strength (right) | All-cause dementia (FinnGen) | rs62234790 | 3:13750642 | A/C | 0.246 | 0.0106 (0.0017) | 8.8E-10 | 0.0144 (0.0215) | 0.504 | 37.6 | 1.360 |
| Hand grip strength (right) | All-cause dementia (FinnGen) | rs34587452 | 4:1009900 | C/G | 0.215 | -0.0111 (0.0018) | 9.1E-10 | 0.0663 (0.0240) | 0.006 | 37.5 | -5.980 |
| Hand grip strength (right) | All-cause dementia (FinnGen) | rs4868110 | 5:171164168 | T/A | 0.323 | -0.0097 (0.0016) | 1.0E-09 | 0.0331 (0.0199) | 0.096 | 37.3 | -3.409 |
| Hand grip strength (right) | All-cause dementia (FinnGen) | rs11022513 | 11:12840986 | T/C | 0.569 | -0.0092 (0.0015) | 1.1E-09 | -0.0169 (0.0195) | 0.387 | 37.2 | 1.835 |
| Hand grip strength (right) | All-cause dementia (FinnGen) | rs1047891 | 2:211540507 | A/C | 0.316 | 0.0097 (0.0016) | 1.3E-09 | -0.0402 (0.0206) | 0.051 | 36.8 | -4.150 |
| Hand grip strength (right) | All-cause dementia (FinnGen) | rs12101479 | 15:74248548 | C/G | 0.237 | -0.0106 (0.0018) | 1.5E-09 | -0.0047 (0.0208) | 0.822 | 36.6 | 0.443 |
| Hand grip strength (right) | All-cause dementia (FinnGen) | rs2341184 | 3:196930781 | C/T | 0.272 | 0.0101 (0.0017) | 1.7E-09 | -0.0194 (0.0206) | 0.347 | 36.2 | -1.928 |
| Hand grip strength (right) | All-cause dementia (FinnGen) | rs6792762 | 3:38574491 | A/G | 0.420 | -0.0091 (0.0015) | 1.8E-09 | 0.0258 (0.0193) | 0.182 | 36.2 | -2.830 |
| Hand grip strength (right) | All-cause dementia (FinnGen) | rs935728 | 14:80957923 | T/C | 0.328 | 0.0096 (0.0016) | 1.8E-09 | 0.0126 (0.0201) | 0.530 | 36.2 | 1.319 |
| Hand grip strength (right) | All-cause dementia (FinnGen) | rs2194747 | 2:218126181 | G/A | 0.707 | 0.0099 (0.0016) | 1.9E-09 | 0.0037 (0.0199) | 0.851 | 36.1 | 0.376 |
| Hand grip strength (right) | All-cause dementia (FinnGen) | rs62037412 | 16:28917746 | A/G | 0.357 | 0.0093 (0.0016) | 1.9E-09 | 0.0499 (0.0193) | 0.010 | 36.0 | 5.349 |
| Hand grip strength (right) | All-cause dementia (FinnGen) | rs16870531 | 7:120660682 | T/C | 0.238 | 0.0104 (0.0017) | 2.2E-09 | -0.0059 (0.0221) | 0.789 | 35.8 | -0.565 |
| Hand grip strength (right) | All-cause dementia (FinnGen) | rs6592728 | 11:77247260 | G/A | 0.371 | -0.0092 (0.0015) | 2.5E-09 | -0.0310 (0.0194) | 0.110 | 35.5 | 3.376 |
| Hand grip strength (right) | All-cause dementia (FinnGen) | rs1486925 | 8:78827617 | C/T | 0.315 | -0.0095 (0.0016) | 2.9E-09 | -0.0122 (0.0214) | 0.568 | 35.3 | 1.280 |
| Hand grip strength (right) | All-cause dementia (FinnGen) | rs6870324 | 5:141787317 | G/C | 0.270 | -0.0100 (0.0017) | 3.0E-09 | -0.0282 (0.0228) | 0.215 | 35.2 | 2.824 |
| Hand grip strength (right) | All-cause dementia (FinnGen) | rs2226685 | 21:40069825 | C/T | 0.759 | 0.0103 (0.0017) | 3.1E-09 | 0.0004 (0.0210) | 0.985 | 35.1 | 0.039 |
| Hand grip strength (right) | All-cause dementia (FinnGen) | rs34030812 | 2:144248905 | C/T | 0.367 | -0.0091 (0.0015) | 3.3E-09 | 0.0102 (0.0197) | 0.604 | 35.0 | -1.117 |
| Hand grip strength (right) | All-cause dementia (FinnGen) | rs10483727 | 14:61072875 | C/T | 0.610 | -0.0090 (0.0015) | 3.3E-09 | 0.0392 (0.0211) | 0.063 | 35.0 | -4.347 |
| Hand grip strength (right) | All-cause dementia (FinnGen) | rs61389091 | 11:74427921 | T/C | 0.042 | 0.0221 (0.0037) | 3.6E-09 | -0.0459 (0.0482) | 0.341 | 34.8 | -2.081 |
| Hand grip strength (right) | All-cause dementia (FinnGen) | rs997850 | 4:154838434 | C/G | 0.605 | -0.0090 (0.0015) | 3.7E-09 | -0.0053 (0.0192) | 0.783 | 34.8 | 0.587 |
| Hand grip strength (right) | All-cause dementia (FinnGen) | rs2322754 | 6:81046299 | A/G | 0.832 | -0.0117 (0.0020) | 3.8E-09 | -0.0023 (0.0243) | 0.925 | 34.7 | 0.197 |
| Hand grip strength (right) | All-cause dementia (FinnGen) | rs56074046 | 17:7358930 | A/G | 0.372 | -0.0090 (0.0015) | 4.4E-09 | -0.0182 (0.0212) | 0.391 | 34.4 | 2.014 |
| Hand grip strength (right) | All-cause dementia (FinnGen) | rs12052508 | 2:72757843 | T/C | 0.879 | -0.0134 (0.0023) | 4.7E-09 | -0.0053 (0.0323) | 0.870 | 34.3 | 0.397 |
| Hand grip strength (right) | All-cause dementia (FinnGen) | rs7148603 | 14:36683779 | A/G | 0.359 | 0.0093 (0.0016) | 4.7E-09 | 0.0044 (0.0206) | 0.830 | 34.3 | 0.473 |
| Hand grip strength (right) | All-cause dementia (FinnGen) | rs7034200 | 9:4289050 | A/C | 0.480 | 0.0087 (0.0015) | 5.3E-09 | 0.0100 (0.0191) | 0.600 | 34.1 | 1.151 |
| Hand grip strength (right) | All-cause dementia (FinnGen) | rs12616285 | 2:169156907 | G/T | 0.149 | 0.0122 (0.0021) | 5.4E-09 | 0.0123 (0.0247) | 0.619 | 34.0 | 1.007 |
| Hand grip strength (right) | All-cause dementia (FinnGen) | rs6473015 | 8:78178485 | C/A | 0.286 | 0.0096 (0.0016) | 5.5E-09 | -0.0042 (0.0233) | 0.856 | 34.0 | -0.438 |
| Hand grip strength (right) | All-cause dementia (FinnGen) | rs7549184 | 1:215412906 | A/G | 0.787 | 0.0106 (0.0018) | 5.8E-09 | 0.0014 (0.0236) | 0.954 | 33.9 | 0.133 |
| Hand grip strength (right) | All-cause dementia (FinnGen) | rs2147461 | 1:191051627 | C/T | 0.117 | 0.0134 (0.0023) | 6.1E-09 | -0.0165 (0.0257) | 0.522 | 33.8 | -1.229 |
| Hand grip strength (right) | All-cause dementia (FinnGen) | rs6693567 | 1:150510660 | T/C | 0.733 | -0.0097 (0.0017) | 6.3E-09 | -0.0027 (0.0233) | 0.909 | 33.8 | 0.277 |
| Hand grip strength (right) | All-cause dementia (FinnGen) | rs4752689 | 10:124131176 | A/G | 0.584 | 0.0088 (0.0015) | 6.4E-09 | -0.0445 (0.0210) | 0.034 | 33.7 | -5.084 |
| Hand grip strength (right) | All-cause dementia (FinnGen) | rs12522139 | 5:122699812 | G/T | 0.171 | -0.0114 (0.0020) | 6.8E-09 | -0.0061 (0.0250) | 0.807 | 33.6 | 0.533 |
| Hand grip strength (right) | All-cause dementia (FinnGen) | rs6882168 | 5:39402647 | T/C | 0.337 | -0.0091 (0.0016) | 7.9E-09 | -0.0099 (0.0195) | 0.613 | 33.3 | 1.089 |
| Hand grip strength (right) | All-cause dementia (FinnGen) | rs114924396 | 4:119755621 | G/A | 0.054 | -0.0191 (0.0033) | 8.2E-09 | 0.0035 (0.0397) | 0.930 | 33.2 | -0.183 |
| Hand grip strength (right) | All-cause dementia (FinnGen) | rs248831 | 16:11281218 | A/G | 0.265 | 0.0099 (0.0017) | 8.4E-09 | -0.0047 (0.0228) | 0.838 | 33.2 | -0.475 |
| Hand grip strength (right) | All-cause dementia (FinnGen) | rs35833641 | 2:179462494 | G/A | 0.312 | 0.0092 (0.0016) | 8.5E-09 | -0.0206 (0.0198) | 0.299 | 33.1 | -2.234 |
| Hand grip strength (right) | All-cause dementia (FinnGen) | rs6715064 | 2:220041928 | T/C | 0.310 | -0.0092 (0.0016) | 9.6E-09 | 0.0263 (0.0207) | 0.202 | 32.9 | -2.855 |
| Hand grip strength (right) | All-cause dementia (FinnGen) | rs2894602 | 2:227249802 | G/A | 0.766 | 0.0101 (0.0018) | 9.9E-09 | -0.0234 (0.0231) | 0.310 | 32.9 | -2.309 |
| Hand grip strength (right) | All-cause dementia (FinnGen) | rs113835839 | 6:13784625 | T/C | 0.248 | -0.0099 (0.0017) | 1.1E-08 | 0.0056 (0.0267) | 0.833 | 32.7 | -0.568 |
| Hand grip strength (right) | All-cause dementia (FinnGen) | rs10278546 | 7:100516003 | C/A | 0.195 | 0.0108 (0.0019) | 1.1E-08 | -0.0534 (0.0232) | 0.022 | 32.6 | -4.966 |
| Hand grip strength (right) | All-cause dementia (FinnGen) | rs7790322 | 7:2830498 | T/C | 0.416 | -0.0086 (0.0015) | 1.2E-08 | 0.0147 (0.0191) | 0.441 | 32.5 | -1.709 |
| Hand grip strength (right) | All-cause dementia (FinnGen) | rs4962700 | 10:126479989 | G/C | 0.302 | 0.0093 (0.0016) | 1.2E-08 | -0.0137 (0.0196) | 0.485 | 32.5 | -1.469 |
| Hand grip strength (right) | All-cause dementia (FinnGen) | rs8055199 | 16:84867404 | A/G | 0.660 | -0.0090 (0.0016) | 1.2E-08 | -0.0245 (0.0220) | 0.266 | 32.5 | 2.734 |
| Hand grip strength (right) | All-cause dementia (FinnGen) | rs2362972 | 3:158163272 | A/C | 0.578 | -0.0086 (0.0015) | 1.3E-08 | 0.0067 (0.0192) | 0.727 | 32.3 | -0.782 |
| Hand grip strength (right) | All-cause dementia (FinnGen) | rs10761411 | 9:136973826 | T/C | 0.812 | -0.0115 (0.0020) | 1.3E-08 | -0.0146 (0.0231) | 0.529 | 32.4 | 1.270 |
| Hand grip strength (right) | All-cause dementia (FinnGen) | rs1840753 | 2:199060177 | T/C | 0.063 | 0.0177 (0.0031) | 1.4E-08 | -0.1170 (0.0537) | 0.029 | 32.1 | -6.612 |
| Hand grip strength (right) | All-cause dementia (FinnGen) | rs10770125 | 11:2169014 | G/A | 0.477 | 0.0084 (0.0015) | 1.4E-08 | -0.0122 (0.0192) | 0.525 | 32.2 | -1.445 |
| Hand grip strength (right) | All-cause dementia (FinnGen) | rs35701422 | 3:85575775 | C/T | 0.626 | -0.0087 (0.0015) | 1.5E-08 | -0.0151 (0.0212) | 0.476 | 32.1 | 1.737 |
| Hand grip strength (right) | All-cause dementia (FinnGen) | rs852520 | 7:5695267 | A/C | 0.662 | -0.0089 (0.0016) | 1.5E-08 | 0.0038 (0.0200) | 0.849 | 32.0 | -0.427 |
| Hand grip strength (right) | All-cause dementia (FinnGen) | rs600038 | 9:136151806 | C/T | 0.207 | -0.0104 (0.0018) | 1.5E-08 | -0.0120 (0.0230) | 0.603 | 32.0 | 1.157 |
| Hand grip strength (right) | All-cause dementia (FinnGen) | rs721101 | 6:155632844 | C/T | 0.271 | 0.0095 (0.0017) | 1.6E-08 | -0.0094 (0.0242) | 0.699 | 32.0 | -0.993 |
| Hand grip strength (right) | All-cause dementia (FinnGen) | rs79723785 | 19:55818225 | C/T | 0.016 | -0.0340 (0.0060) | 1.6E-08 | 0.0801 (0.0745) | 0.283 | 31.9 | -2.352 |
| Hand grip strength (right) | All-cause dementia (FinnGen) | rs112330055 | 7:23109316 | A/G | 0.063 | 0.0180 (0.0032) | 1.7E-08 | -0.0287 (0.0430) | 0.504 | 31.8 | -1.596 |
| Hand grip strength (right) | All-cause dementia (FinnGen) | rs34845616 | 11:133792644 | A/G | 0.246 | 0.0098 (0.0017) | 1.7E-08 | -0.0168 (0.0204) | 0.411 | 31.8 | -1.715 |
| Hand grip strength (right) | All-cause dementia (FinnGen) | rs12562146 | 1:86247267 | A/T | 0.145 | 0.0119 (0.0021) | 2.0E-08 | 0.0124 (0.0292) | 0.671 | 31.5 | 1.045 |
| Hand grip strength (right) | All-cause dementia (FinnGen) | rs6962338 | 7:69160985 | G/A | 0.044 | -0.0203 (0.0036) | 2.0E-08 | -0.0400 (0.0517) | 0.440 | 31.5 | 1.966 |
| Hand grip strength (right) | All-cause dementia (FinnGen) | rs7214252 | 17:27486673 | A/G | 0.211 | -0.0102 (0.0018) | 2.0E-08 | -0.0190 (0.0222) | 0.394 | 31.5 | 1.855 |
| Hand grip strength (right) | All-cause dementia (FinnGen) | rs911642 | 20:13260252 | T/C | 0.376 | 0.0086 (0.0015) | 2.0E-08 | 0.0002 (0.0196) | 0.993 | 31.5 | 0.023 |
| Hand grip strength (right) | All-cause dementia (FinnGen) | rs35457492 | 3:70194564 | C/A | 0.495 | 0.0083 (0.0015) | 2.2E-08 | -0.0158 (0.0196) | 0.420 | 31.3 | -1.895 |
| Hand grip strength (right) | All-cause dementia (FinnGen) | rs76749769 | 16:3291408 | T/C | 0.091 | 0.0144 (0.0026) | 2.2E-08 | 0.0181 (0.0382) | 0.635 | 31.3 | 1.254 |
| Hand grip strength (right) | All-cause dementia (FinnGen) | rs4768725 | 12:46848478 | C/T | 0.700 | 0.0091 (0.0016) | 2.3E-08 | -0.0415 (0.0201) | 0.039 | 31.2 | -4.581 |
| Hand grip strength (right) | All-cause dementia (FinnGen) | rs9388051 | 6:122577108 | A/G | 0.186 | 0.0106 (0.0019) | 2.7E-08 | -0.0238 (0.0286) | 0.406 | 30.9 | -2.240 |
| Hand grip strength (right) | All-cause dementia (FinnGen) | rs1885690 | 6:109672998 | A/C | 0.410 | -0.0084 (0.0015) | 2.8E-08 | 0.0116 (0.0192) | 0.546 | 30.9 | -1.381 |
| Hand grip strength (right) | All-cause dementia (FinnGen) | rs11998884 | 9:33684436 | T/C | 0.062 | 0.0173 (0.0031) | 2.8E-08 | -0.0170 (0.0353) | 0.630 | 30.8 | -0.980 |
| Hand grip strength (right) | All-cause dementia (FinnGen) | rs12412806 | 10:24860913 | A/G | 0.294 | -0.0091 (0.0016) | 3.0E-08 | 0.0114 (0.0196) | 0.560 | 30.7 | -1.253 |
| Hand grip strength (right) | All-cause dementia (FinnGen) | rs75457267 | 5:102658770 | T/C | 0.051 | -0.0187 (0.0034) | 3.4E-08 | 0.0732 (0.0455) | 0.108 | 30.4 | -3.908 |
| Hand grip strength (right) | All-cause dementia (FinnGen) | rs13355365 | 5:154942606 | T/C | 0.380 | -0.0085 (0.0015) | 3.4E-08 | 0.0178 (0.0202) | 0.377 | 30.5 | -2.103 |
| Hand grip strength (right) | All-cause dementia (FinnGen) | rs2389763 | 7:17307847 | C/T | 0.592 | -0.0083 (0.0015) | 3.8E-08 | 0.0184 (0.0197) | 0.350 | 30.3 | -2.210 |
| Hand grip strength (right) | All-cause dementia (FinnGen) | rs4793658 | 17:45878733 | C/A | 0.110 | -0.0137 (0.0025) | 3.8E-08 | 0.0534 (0.0337) | 0.113 | 30.2 | -3.906 |
| Hand grip strength (right) | All-cause dementia (FinnGen) | rs35304341 | 1:200971049 | A/G | 0.089 | -0.0143 (0.0026) | 4.0E-08 | 0.0285 (0.0325) | 0.381 | 30.1 | -1.995 |
| Hand grip strength (right) | All-cause dementia (FinnGen) | rs7249 | 19:18391171 | T/C | 0.366 | 0.0085 (0.0015) | 4.0E-08 | 0.0073 (0.0202) | 0.716 | 30.2 | 0.861 |
| Hand grip strength (right) | All-cause dementia (FinnGen) | rs4751671 | 10:116138744 | A/G | 0.531 | 0.0082 (0.0015) | 4.1E-08 | 0.0118 (0.0201) | 0.557 | 30.1 | 1.433 |
| Hand grip strength (right) | All-cause dementia (FinnGen) | rs13169333 | 5:153064994 | C/T | 0.260 | 0.0093 (0.0017) | 4.3E-08 | 0.0328 (0.0213) | 0.123 | 30.0 | 3.530 |
| Hand grip strength (right) | All-cause dementia (FinnGen) | rs645144 | 6:141180780 | C/T | 0.330 | -0.0087 (0.0016) | 4.3E-08 | -0.0273 (0.0213) | 0.200 | 30.0 | 3.142 |
| Hand grip strength (right) | All-cause dementia (FinnGen) | rs113851275 | 9:98297220 | A/G | 0.108 | 0.0131 (0.0024) | 4.3E-08 | -0.0264 (0.0299) | 0.376 | 30.0 | -2.009 |
| Hand grip strength (right) | All-cause dementia (FinnGen) | rs2244621 | 11:64026219 | T/C | 0.144 | 0.0116 (0.0021) | 4.5E-08 | -0.0093 (0.0262) | 0.721 | 29.9 | -0.802 |
| Hand grip strength (right) | All-cause dementia (FinnGen) | rs2296316 | 14:65520246 | C/T | 0.464 | -0.0082 (0.0015) | 4.6E-08 | -0.0114 (0.0191) | 0.552 | 29.9 | 1.388 |
| Hand grip strength (right) | Vascular dementia (FinnGen) | rs143384 | 20:34025756 | G/A | 0.404 | 0.0230 (0.0015) | 2.5E-52 | 0.0484 (0.0498) | 0.331 | 231.7 | 2.100 |
| Hand grip strength (right) | Vascular dementia (FinnGen) | rs1556659 | 10:130834698 | T/C | 0.382 | 0.0175 (0.0015) | 3.8E-30 | -0.0321 (0.0540) | 0.552 | 130.2 | -1.831 |
| Hand grip strength (right) | Vascular dementia (FinnGen) | rs3118914 | 13:51116901 | T/G | 0.215 | -0.0194 (0.0018) | 5.9E-27 | 0.0379 (0.0562) | 0.500 | 115.6 | -1.950 |
| Hand grip strength (right) | Vascular dementia (FinnGen) | rs12316046 | 12:15054415 | G/A | 0.378 | -0.0162 (0.0015) | 3.8E-26 | -0.0250 (0.0550) | 0.649 | 111.9 | 1.543 |
| Hand grip strength (right) | Vascular dementia (FinnGen) | rs10846071 | 12:15016236 | T/C | 0.394 | -0.0157 (0.0015) | 6.5E-25 | -0.0019 (0.0533) | 0.972 | 106.2 | 0.121 |
| Hand grip strength (right) | Vascular dementia (FinnGen) | rs13146142 | 4:17931318 | C/T | 0.159 | -0.0208 (0.0020) | 1.4E-24 | -0.0613 (0.0837) | 0.464 | 104.8 | 2.948 |
| Hand grip strength (right) | Vascular dementia (FinnGen) | rs2871865 | 15:99194896 | G/C | 0.116 | -0.0237 (0.0023) | 1.8E-24 | 0.2519 (0.1010) | 0.013 | 104.2 | -10.635 |
| Hand grip strength (right) | Vascular dementia (FinnGen) | rs9267806 | 6:32110886 | A/G | 0.256 | -0.0167 (0.0017) | 7.7E-23 | 0.0008 (0.0523) | 0.988 | 96.8 | -0.048 |
| Hand grip strength (right) | Vascular dementia (FinnGen) | rs7451021 | 6:130381246 | C/T | 0.689 | -0.0158 (0.0016) | 7.7E-23 | 0.0378 (0.0524) | 0.471 | 96.8 | -2.393 |
| Hand grip strength (right) | Vascular dementia (FinnGen) | rs12790261 | 11:66988048 | A/C | 0.082 | -0.0264 (0.0027) | 2.0E-22 | 0.0920 (0.1125) | 0.414 | 94.9 | -3.489 |
| Hand grip strength (right) | Vascular dementia (FinnGen) | rs13107325 | 4:103188709 | T/C | 0.075 | -0.0275 (0.0028) | 2.1E-22 | 0.1048 (0.2098) | 0.617 | 94.8 | -3.811 |
| Hand grip strength (right) | Vascular dementia (FinnGen) | rs10798483 | 1:176799143 | A/G | 0.547 | 0.0145 (0.0015) | 2.8E-22 | -0.0216 (0.0492) | 0.661 | 94.2 | -1.489 |
| Hand grip strength (right) | Vascular dementia (FinnGen) | rs3771498 | 2:70720070 | T/C | 0.514 | 0.0142 (0.0015) | 1.9E-21 | -0.0066 (0.0489) | 0.893 | 90.5 | -0.466 |
| Hand grip strength (right) | Vascular dementia (FinnGen) | rs4369779 | 18:20735408 | C/T | 0.789 | 0.0172 (0.0018) | 3.4E-21 | 0.0232 (0.0583) | 0.691 | 89.3 | 1.349 |
| Hand grip strength (right) | Vascular dementia (FinnGen) | rs1043515 | 17:36922196 | G/A | 0.566 | 0.0138 (0.0015) | 2.8E-20 | 0.0028 (0.0493) | 0.954 | 85.2 | 0.202 |
| Hand grip strength (right) | Vascular dementia (FinnGen) | rs1550115 | 2:25041620 | T/C | 0.749 | 0.0153 (0.0017) | 4.5E-19 | -0.0666 (0.0580) | 0.251 | 79.6 | -4.358 |
| Hand grip strength (right) | Vascular dementia (FinnGen) | rs4784329 | 16:53910261 | C/A | 0.426 | -0.0133 (0.0015) | 9.0E-19 | 0.0773 (0.0489) | 0.114 | 78.3 | -5.796 |
| Hand grip strength (right) | Vascular dementia (FinnGen) | rs12473839 | 2:135638044 | T/C | 0.371 | 0.0133 (0.0015) | 4.0E-18 | 0.0159 (0.0503) | 0.752 | 75.3 | 1.198 |
| Hand grip strength (right) | Vascular dementia (FinnGen) | rs4927015 | 1:54072471 | A/G | 0.583 | 0.0131 (0.0015) | 5.0E-18 | 0.0203 (0.0525) | 0.700 | 74.9 | 1.554 |
| Hand grip strength (right) | Vascular dementia (FinnGen) | rs635538 | 18:53273614 | A/G | 0.914 | -0.0220 (0.0027) | 1.5E-16 | -0.1039 (0.0806) | 0.197 | 68.2 | 4.724 |
| Hand grip strength (right) | Vascular dementia (FinnGen) | rs823130 | 1:205714372 | T/C | 0.433 | -0.0124 (0.0015) | 1.7E-16 | 0.0156 (0.0496) | 0.754 | 67.9 | -1.259 |
| Hand grip strength (right) | Vascular dementia (FinnGen) | rs2165241 | 15:74222202 | C/T | 0.509 | 0.0122 (0.0015) | 1.9E-16 | 0.0469 (0.0488) | 0.336 | 67.7 | 3.829 |
| Hand grip strength (right) | Vascular dementia (FinnGen) | rs6977081 | 7:150542515 | T/G | 0.334 | 0.0129 (0.0016) | 5.7E-16 | 0.0003 (0.0513) | 0.996 | 65.5 | 0.023 |
| Hand grip strength (right) | Vascular dementia (FinnGen) | rs56365901 | 17:43960323 | G/A | 0.223 | -0.0143 (0.0018) | 1.8E-15 | -0.2169 (0.0895) | 0.015 | 63.3 | 15.208 |
| Hand grip strength (right) | Vascular dementia (FinnGen) | rs7206195 | 16:2145280 | T/C | 0.180 | -0.0153 (0.0019) | 2.8E-15 | 0.0129 (0.0601) | 0.831 | 62.4 | -0.842 |
| Hand grip strength (right) | Vascular dementia (FinnGen) | rs10520770 | 18:46602964 | C/T | 0.449 | 0.0117 (0.0015) | 4.5E-15 | -0.0667 (0.0528) | 0.207 | 61.5 | -5.680 |
| Hand grip strength (right) | Vascular dementia (FinnGen) | rs11243202 | 6:7719065 | C/T | 0.486 | 0.0116 (0.0015) | 6.5E-15 | 0.0483 (0.0495) | 0.329 | 60.8 | 4.150 |
| Hand grip strength (right) | Vascular dementia (FinnGen) | rs150330307 | 1:160160801 | C/T | 0.032 | -0.0326 (0.0042) | 1.2E-14 | 0.0274 (0.1752) | 0.876 | 59.5 | -0.841 |
| Hand grip strength (right) | Vascular dementia (FinnGen) | rs2208562 | 9:119344528 | T/C | 0.610 | -0.0116 (0.0015) | 3.1E-14 | -0.0063 (0.0514) | 0.902 | 57.7 | 0.545 |
| Hand grip strength (right) | Vascular dementia (FinnGen) | rs10799428 | 1:227798565 | T/C | 0.187 | -0.0144 (0.0019) | 4.6E-14 | -0.0364 (0.0617) | 0.555 | 56.9 | 2.535 |
| Hand grip strength (right) | Vascular dementia (FinnGen) | rs2631360 | 5:131707429 | A/G | 0.519 | -0.0112 (0.0015) | 5.4E-14 | -0.0083 (0.0517) | 0.872 | 56.6 | 0.742 |
| Hand grip strength (right) | Vascular dementia (FinnGen) | rs10784502 | 12:66343810 | T/C | 0.512 | -0.0111 (0.0015) | 7.1E-14 | -0.0316 (0.0492) | 0.521 | 56.0 | 2.841 |
| Hand grip strength (right) | Vascular dementia (FinnGen) | rs7963801 | 12:79685226 | C/T | 0.572 | -0.0113 (0.0015) | 8.4E-14 | -0.0422 (0.0502) | 0.400 | 55.7 | 3.743 |
| Hand grip strength (right) | Vascular dementia (FinnGen) | rs2431112 | 5:103931707 | A/G | 0.441 | -0.0112 (0.0015) | 8.6E-14 | -0.0345 (0.0505) | 0.494 | 55.7 | 3.088 |
| Hand grip strength (right) | Vascular dementia (FinnGen) | rs1641457 | 2:40421990 | G/T | 0.223 | 0.0131 (0.0018) | 2.4E-13 | -0.0324 (0.0542) | 0.550 | 53.6 | -2.478 |
| Hand grip strength (right) | Vascular dementia (FinnGen) | rs6693965 | 1:10378416 | T/G | 0.128 | -0.0163 (0.0022) | 3.2E-13 | 0.0403 (0.0793) | 0.611 | 53.1 | -2.480 |
| Hand grip strength (right) | Vascular dementia (FinnGen) | rs9652468 | 15:56823913 | A/G | 0.249 | -0.0125 (0.0017) | 3.3E-13 | 0.0481 (0.0532) | 0.366 | 53.0 | -3.839 |
| Hand grip strength (right) | Vascular dementia (FinnGen) | rs2273555 | 10:104127171 | A/G | 0.606 | 0.0110 (0.0015) | 4.2E-13 | 0.1200 (0.0502) | 0.017 | 52.6 | 10.878 |
| Hand grip strength (right) | Vascular dementia (FinnGen) | rs7301953 | 12:124405871 | A/G | 0.312 | -0.0115 (0.0016) | 6.4E-13 | -0.0227 (0.0546) | 0.677 | 51.7 | 1.967 |
| Hand grip strength (right) | Vascular dementia (FinnGen) | rs7196917 | 16:69896527 | G/A | 0.430 | -0.0106 (0.0015) | 1.9E-12 | 0.0618 (0.0507) | 0.223 | 49.6 | -5.840 |
| Hand grip strength (right) | Vascular dementia (FinnGen) | rs2717351 | 7:19019880 | G/A | 0.212 | 0.0128 (0.0018) | 3.4E-12 | 0.0367 (0.0564) | 0.515 | 48.4 | 2.874 |
| Hand grip strength (right) | Vascular dementia (FinnGen) | rs9322822 | 6:105369598 | T/C | 0.320 | 0.0111 (0.0016) | 3.5E-12 | 0.0447 (0.0531) | 0.400 | 48.4 | 4.034 |
| Hand grip strength (right) | Vascular dementia (FinnGen) | rs7565148 | 2:44188396 | G/T | 0.501 | -0.0104 (0.0015) | 3.6E-12 | 0.0432 (0.0491) | 0.379 | 48.3 | -4.173 |
| Hand grip strength (right) | Vascular dementia (FinnGen) | rs72820369 | 10:81251539 | T/A | 0.118 | 0.0161 (0.0023) | 4.4E-12 | 0.0741 (0.0700) | 0.290 | 47.9 | 4.595 |
| Hand grip strength (right) | Vascular dementia (FinnGen) | rs4785574 | 16:89568875 | G/A | 0.555 | -0.0104 (0.0015) | 4.5E-12 | -0.0854 (0.0504) | 0.090 | 47.9 | 8.230 |
| Hand grip strength (right) | Vascular dementia (FinnGen) | rs2854152 | 17:61986027 | G/A | 0.678 | 0.0110 (0.0016) | 6.0E-12 | -0.0682 (0.0511) | 0.182 | 47.3 | -6.205 |
| Hand grip strength (right) | Vascular dementia (FinnGen) | rs9853018 | 3:141101961 | T/C | 0.443 | 0.0102 (0.0015) | 8.8E-12 | 0.0354 (0.0496) | 0.476 | 46.6 | 3.471 |
| Hand grip strength (right) | Vascular dementia (FinnGen) | rs7575451 | 2:152352843 | G/C | 0.650 | -0.0106 (0.0016) | 9.3E-12 | 0.0172 (0.0527) | 0.745 | 46.5 | -1.622 |
| Hand grip strength (right) | Vascular dementia (FinnGen) | rs12452505 | 17:63556402 | G/C | 0.142 | -0.0144 (0.0021) | 1.4E-11 | -0.1108 (0.0826) | 0.180 | 45.7 | 7.678 |
| Hand grip strength (right) | Vascular dementia (FinnGen) | rs62509875 | 8:110360944 | G/A | 0.171 | -0.0132 (0.0020) | 2.4E-11 | -0.0397 (0.0716) | 0.579 | 44.6 | 3.012 |
| Hand grip strength (right) | Vascular dementia (FinnGen) | rs4802848 | 19:52218342 | C/G | 0.730 | 0.0111 (0.0017) | 3.8E-11 | -0.0622 (0.0613) | 0.310 | 43.7 | -5.615 |
| Hand grip strength (right) | Vascular dementia (FinnGen) | rs4121165 | 1:78276977 | A/G | 0.211 | -0.0120 (0.0018) | 4.8E-11 | 0.0146 (0.0651) | 0.823 | 43.2 | -1.219 |
| Hand grip strength (right) | Vascular dementia (FinnGen) | rs13106087 | 4:145566864 | C/T | 0.830 | 0.0129 (0.0020) | 6.2E-11 | 0.0014 (0.0659) | 0.984 | 42.8 | 0.108 |
| Hand grip strength (right) | Vascular dementia (FinnGen) | rs1892425 | 1:41744821 | A/G | 0.239 | 0.0113 (0.0018) | 1.1E-10 | -0.0744 (0.0568) | 0.190 | 41.6 | -6.563 |
| Hand grip strength (right) | Vascular dementia (FinnGen) | rs7657558 | 4:30648636 | G/T | 0.720 | 0.0107 (0.0017) | 1.1E-10 | -0.1460 (0.0553) | 0.008 | 41.6 | -13.612 |
| Hand grip strength (right) | Vascular dementia (FinnGen) | rs12763284 | 10:104508202 | G/A | 0.466 | 0.0096 (0.0015) | 1.2E-10 | 0.0336 (0.0495) | 0.497 | 41.5 | 3.501 |
| Hand grip strength (right) | Vascular dementia (FinnGen) | rs34217742 | 19:37376830 | A/T | 0.124 | 0.0146 (0.0023) | 1.4E-10 | -0.0029 (0.0707) | 0.967 | 41.2 | -0.198 |
| Hand grip strength (right) | Vascular dementia (FinnGen) | rs12823922 | 12:24186697 | G/A | 0.222 | -0.0114 (0.0018) | 1.6E-10 | -0.0083 (0.0577) | 0.885 | 40.9 | 0.727 |
| Hand grip strength (right) | Vascular dementia (FinnGen) | rs116922558 | 9:118802375 | G/A | 0.040 | -0.0246 (0.0039) | 1.8E-10 | 0.0940 (0.1194) | 0.431 | 40.7 | -3.817 |
| Hand grip strength (right) | Vascular dementia (FinnGen) | rs1125 | 6:149979416 | A/G | 0.337 | -0.0100 (0.0016) | 1.9E-10 | 0.0676 (0.0508) | 0.183 | 40.6 | -6.740 |
| Hand grip strength (right) | Vascular dementia (FinnGen) | rs12914702 | 15:96887277 | A/G | 0.300 | 0.0108 (0.0017) | 1.9E-10 | -0.0320 (0.0531) | 0.546 | 40.5 | -2.969 |
| Hand grip strength (right) | Vascular dementia (FinnGen) | rs2194411 | 3:185548663 | A/G | 0.128 | 0.0143 (0.0022) | 2.1E-10 | -0.0900 (0.0723) | 0.213 | 40.4 | -6.301 |
| Hand grip strength (right) | Vascular dementia (FinnGen) | rs4737446 | 8:57665019 | T/G | 0.695 | 0.0103 (0.0016) | 2.4E-10 | 0.0260 (0.0525) | 0.621 | 40.1 | 2.532 |
| Hand grip strength (right) | Vascular dementia (FinnGen) | rs77485342 | 6:30842866 | T/C | 0.018 | 0.0353 (0.0056) | 2.6E-10 | -0.4180 (0.1576) | 0.008 | 40.0 | -11.829 |
| Hand grip strength (right) | Vascular dementia (FinnGen) | rs7871404 | 9:99262296 | G/A | 0.189 | 0.0119 (0.0019) | 3.4E-10 | -0.0381 (0.0668) | 0.569 | 39.4 | -3.192 |
| Hand grip strength (right) | Vascular dementia (FinnGen) | rs246181 | 16:14392641 | T/C | 0.373 | 0.0097 (0.0015) | 3.7E-10 | 0.0216 (0.0503) | 0.668 | 39.3 | 2.226 |
| Hand grip strength (right) | Vascular dementia (FinnGen) | rs56144131 | 1:208977656 | C/T | 0.149 | -0.0131 (0.0021) | 3.8E-10 | 0.0051 (0.0680) | 0.941 | 39.2 | -0.390 |
| Hand grip strength (right) | Vascular dementia (FinnGen) | rs11039348 | 11:47728617 | A/G | 0.348 | -0.0098 (0.0016) | 3.9E-10 | -0.0233 (0.0532) | 0.661 | 39.2 | 2.385 |
| Hand grip strength (right) | Vascular dementia (FinnGen) | rs1952256 | 1:184035116 | G/A | 0.345 | 0.0098 (0.0016) | 4.1E-10 | 0.0090 (0.0502) | 0.858 | 39.0 | 0.921 |
| Hand grip strength (right) | Vascular dementia (FinnGen) | rs10193039 | 2:99692373 | T/A | 0.281 | -0.0103 (0.0017) | 4.1E-10 | 0.0194 (0.0562) | 0.731 | 39.1 | -1.878 |
| Hand grip strength (right) | Vascular dementia (FinnGen) | rs76895963 | 12:4384844 | G/T | 0.021 | 0.0360 (0.0058) | 4.1E-10 | -0.1199 (0.1500) | 0.424 | 39.0 | -3.333 |
| Hand grip strength (right) | Vascular dementia (FinnGen) | rs4553566 | 15:58336319 | C/T | 0.453 | -0.0093 (0.0015) | 4.6E-10 | -0.0762 (0.0493) | 0.122 | 38.9 | 8.192 |
| Hand grip strength (right) | Vascular dementia (FinnGen) | rs7266065 | 20:47531817 | A/G | 0.323 | 0.0099 (0.0016) | 4.6E-10 | 0.0609 (0.0550) | 0.268 | 38.8 | 6.127 |
| Hand grip strength (right) | Vascular dementia (FinnGen) | rs3848369 | 16:415078 | T/C | 0.387 | -0.0095 (0.0015) | 4.7E-10 | -0.0701 (0.0502) | 0.163 | 38.8 | 7.350 |
| Hand grip strength (right) | Vascular dementia (FinnGen) | rs6006984 | 22:45714937 | C/T | 0.278 | 0.0103 (0.0017) | 5.1E-10 | -0.1075 (0.0538) | 0.046 | 38.6 | -10.438 |
| Hand grip strength (right) | Vascular dementia (FinnGen) | rs7576964 | 2:48601448 | T/G | 0.342 | 0.0097 (0.0016) | 5.3E-10 | 0.0414 (0.0504) | 0.411 | 38.6 | 4.248 |
| Hand grip strength (right) | Vascular dementia (FinnGen) | rs12899474 | 15:77391603 | T/C | 0.108 | -0.0149 (0.0024) | 5.4E-10 | 0.0016 (0.0824) | 0.985 | 38.5 | -0.107 |
| Hand grip strength (right) | Vascular dementia (FinnGen) | rs9396861 | 6:18404133 | A/C | 0.599 | -0.0096 (0.0016) | 5.7E-10 | 0.1042 (0.0570) | 0.068 | 38.4 | -10.821 |
| Hand grip strength (right) | Vascular dementia (FinnGen) | rs1442883 | 2:59970660 | A/C | 0.253 | -0.0106 (0.0017) | 5.8E-10 | 0.0765 (0.0594) | 0.198 | 38.4 | -7.204 |
| Hand grip strength (right) | Vascular dementia (FinnGen) | rs12889267 | 14:21542766 | G/A | 0.167 | -0.0123 (0.0020) | 6.7E-10 | -0.0756 (0.0751) | 0.314 | 38.1 | 6.153 |
| Hand grip strength (right) | Vascular dementia (FinnGen) | rs58670122 | 1:22492613 | G/A | 0.143 | -0.0132 (0.0021) | 7.4E-10 | 0.1910 (0.0814) | 0.019 | 37.9 | -14.444 |
| Hand grip strength (right) | Vascular dementia (FinnGen) | rs4549685 | 7:39326478 | T/C | 0.330 | 0.0097 (0.0016) | 8.1E-10 | 0.0291 (0.0525) | 0.579 | 37.8 | 2.995 |
| Hand grip strength (right) | Vascular dementia (FinnGen) | rs2587505 | 17:77784268 | C/T | 0.420 | -0.0093 (0.0015) | 8.1E-10 | 0.0649 (0.0526) | 0.217 | 37.8 | -7.000 |
| Hand grip strength (right) | Vascular dementia (FinnGen) | rs9757079 | 3:53155158 | T/C | 0.318 | 0.0098 (0.0016) | 8.4E-10 | 0.0717 (0.0529) | 0.175 | 37.7 | 7.315 |
| Hand grip strength (right) | Vascular dementia (FinnGen) | rs62234790 | 3:13750642 | A/C | 0.246 | 0.0106 (0.0017) | 8.8E-10 | -0.0285 (0.0552) | 0.606 | 37.6 | -2.693 |
| Hand grip strength (right) | Vascular dementia (FinnGen) | rs34587452 | 4:1009900 | C/G | 0.215 | -0.0111 (0.0018) | 9.1E-10 | 0.1200 (0.0620) | 0.053 | 37.5 | -10.824 |
| Hand grip strength (right) | Vascular dementia (FinnGen) | rs4868110 | 5:171164168 | T/A | 0.323 | -0.0097 (0.0016) | 1.0E-09 | 0.0139 (0.0513) | 0.787 | 37.3 | -1.431 |
| Hand grip strength (right) | Vascular dementia (FinnGen) | rs11022513 | 11:12840986 | T/C | 0.569 | -0.0092 (0.0015) | 1.1E-09 | 0.0973 (0.0503) | 0.053 | 37.2 | -10.564 |
| Hand grip strength (right) | Vascular dementia (FinnGen) | rs1047891 | 2:211540507 | A/C | 0.316 | 0.0097 (0.0016) | 1.3E-09 | -0.1229 (0.0530) | 0.020 | 36.8 | -12.688 |
| Hand grip strength (right) | Vascular dementia (FinnGen) | rs12101479 | 15:74248548 | C/G | 0.237 | -0.0106 (0.0018) | 1.5E-09 | 0.0086 (0.0536) | 0.872 | 36.6 | -0.810 |
| Hand grip strength (right) | Vascular dementia (FinnGen) | rs2341184 | 3:196930781 | C/T | 0.272 | 0.0101 (0.0017) | 1.7E-09 | -0.0835 (0.0527) | 0.113 | 36.2 | -8.300 |
| Hand grip strength (right) | Vascular dementia (FinnGen) | rs6792762 | 3:38574491 | A/G | 0.420 | -0.0091 (0.0015) | 1.8E-09 | -0.0369 (0.0497) | 0.458 | 36.2 | 4.047 |
| Hand grip strength (right) | Vascular dementia (FinnGen) | rs935728 | 14:80957923 | T/C | 0.328 | 0.0096 (0.0016) | 1.8E-09 | 0.1136 (0.0512) | 0.027 | 36.2 | 11.891 |
| Hand grip strength (right) | Vascular dementia (FinnGen) | rs2194747 | 2:218126181 | G/A | 0.707 | 0.0099 (0.0016) | 1.9E-09 | 0.0278 (0.0514) | 0.588 | 36.1 | 2.822 |
| Hand grip strength (right) | Vascular dementia (FinnGen) | rs62037412 | 16:28917746 | A/G | 0.357 | 0.0093 (0.0016) | 1.9E-09 | 0.0567 (0.0497) | 0.255 | 36.0 | 6.077 |
| Hand grip strength (right) | Vascular dementia (FinnGen) | rs16870531 | 7:120660682 | T/C | 0.238 | 0.0104 (0.0017) | 2.2E-09 | -0.0074 (0.0567) | 0.897 | 35.8 | -0.709 |
| Hand grip strength (right) | Vascular dementia (FinnGen) | rs6592728 | 11:77247260 | G/A | 0.371 | -0.0092 (0.0015) | 2.5E-09 | -0.0584 (0.0497) | 0.240 | 35.5 | 6.360 |
| Hand grip strength (right) | Vascular dementia (FinnGen) | rs1486925 | 8:78827617 | C/T | 0.315 | -0.0095 (0.0016) | 2.9E-09 | -0.0386 (0.0546) | 0.480 | 35.3 | 4.051 |
| Hand grip strength (right) | Vascular dementia (FinnGen) | rs6870324 | 5:141787317 | G/C | 0.270 | -0.0100 (0.0017) | 3.0E-09 | -0.0270 (0.0585) | 0.645 | 35.2 | 2.704 |
| Hand grip strength (right) | Vascular dementia (FinnGen) | rs2226685 | 21:40069825 | C/T | 0.759 | 0.0103 (0.0017) | 3.1E-09 | 0.0658 (0.0541) | 0.224 | 35.1 | 6.368 |
| Hand grip strength (right) | Vascular dementia (FinnGen) | rs34030812 | 2:144248905 | C/T | 0.367 | -0.0091 (0.0015) | 3.3E-09 | 0.0078 (0.0505) | 0.877 | 35.0 | -0.854 |
| Hand grip strength (right) | Vascular dementia (FinnGen) | rs10483727 | 14:61072875 | C/T | 0.610 | -0.0090 (0.0015) | 3.3E-09 | 0.0495 (0.0541) | 0.360 | 35.0 | -5.489 |
| Hand grip strength (right) | Vascular dementia (FinnGen) | rs61389091 | 11:74427921 | T/C | 0.042 | 0.0221 (0.0037) | 3.6E-09 | -0.0163 (0.1231) | 0.895 | 34.8 | -0.739 |
| Hand grip strength (right) | Vascular dementia (FinnGen) | rs997850 | 4:154838434 | C/G | 0.605 | -0.0090 (0.0015) | 3.7E-09 | 0.0535 (0.0493) | 0.279 | 34.8 | -5.930 |
| Hand grip strength (right) | Vascular dementia (FinnGen) | rs2322754 | 6:81046299 | A/G | 0.832 | -0.0117 (0.0020) | 3.8E-09 | 0.0368 (0.0625) | 0.556 | 34.7 | -3.145 |
| Hand grip strength (right) | Vascular dementia (FinnGen) | rs56074046 | 17:7358930 | A/G | 0.372 | -0.0090 (0.0015) | 4.4E-09 | -0.0848 (0.0545) | 0.120 | 34.4 | 9.386 |
| Hand grip strength (right) | Vascular dementia (FinnGen) | rs12052508 | 2:72757843 | T/C | 0.879 | -0.0134 (0.0023) | 4.7E-09 | -0.0273 (0.0828) | 0.741 | 34.3 | 2.045 |
| Hand grip strength (right) | Vascular dementia (FinnGen) | rs7148603 | 14:36683779 | A/G | 0.359 | 0.0093 (0.0016) | 4.7E-09 | -0.1155 (0.0531) | 0.030 | 34.3 | -12.411 |
| Hand grip strength (right) | Vascular dementia (FinnGen) | rs7034200 | 9:4289050 | A/C | 0.480 | 0.0087 (0.0015) | 5.3E-09 | -0.1256 (0.0492) | 0.011 | 34.1 | -14.455 |
| Hand grip strength (right) | Vascular dementia (FinnGen) | rs12616285 | 2:169156907 | G/T | 0.149 | 0.0122 (0.0021) | 5.4E-09 | 0.0174 (0.0636) | 0.785 | 34.0 | 1.424 |
| Hand grip strength (right) | Vascular dementia (FinnGen) | rs6473015 | 8:78178485 | C/A | 0.286 | 0.0096 (0.0016) | 5.5E-09 | 0.0267 (0.0598) | 0.655 | 34.0 | 2.781 |
| Hand grip strength (right) | Vascular dementia (FinnGen) | rs7549184 | 1:215412906 | A/G | 0.787 | 0.0106 (0.0018) | 5.8E-09 | -0.0192 (0.0608) | 0.753 | 33.9 | -1.818 |
| Hand grip strength (right) | Vascular dementia (FinnGen) | rs2147461 | 1:191051627 | C/T | 0.117 | 0.0134 (0.0023) | 6.1E-09 | -0.0115 (0.0661) | 0.861 | 33.8 | -0.856 |
| Hand grip strength (right) | Vascular dementia (FinnGen) | rs6693567 | 1:150510660 | T/C | 0.733 | -0.0097 (0.0017) | 6.3E-09 | -0.0551 (0.0596) | 0.355 | 33.8 | 5.653 |
| Hand grip strength (right) | Vascular dementia (FinnGen) | rs4752689 | 10:124131176 | A/G | 0.584 | 0.0088 (0.0015) | 6.4E-09 | 0.0247 (0.0541) | 0.648 | 33.7 | 2.822 |
| Hand grip strength (right) | Vascular dementia (FinnGen) | rs12522139 | 5:122699812 | G/T | 0.171 | -0.0114 (0.0020) | 6.8E-09 | 0.0594 (0.0640) | 0.353 | 33.6 | -5.193 |
| Hand grip strength (right) | Vascular dementia (FinnGen) | rs6882168 | 5:39402647 | T/C | 0.337 | -0.0091 (0.0016) | 7.9E-09 | 0.0073 (0.0501) | 0.884 | 33.3 | -0.803 |
| Hand grip strength (right) | Vascular dementia (FinnGen) | rs114924396 | 4:119755621 | G/A | 0.054 | -0.0191 (0.0033) | 8.2E-09 | -0.0338 (0.1025) | 0.741 | 33.2 | 1.767 |
| Hand grip strength (right) | Vascular dementia (FinnGen) | rs248831 | 16:11281218 | A/G | 0.265 | 0.0099 (0.0017) | 8.4E-09 | -0.0315 (0.0587) | 0.591 | 33.2 | -3.185 |
| Hand grip strength (right) | Vascular dementia (FinnGen) | rs35833641 | 2:179462494 | G/A | 0.312 | 0.0092 (0.0016) | 8.5E-09 | 0.0854 (0.0509) | 0.093 | 33.1 | 9.259 |
| Hand grip strength (right) | Vascular dementia (FinnGen) | rs6715064 | 2:220041928 | T/C | 0.310 | -0.0092 (0.0016) | 9.6E-09 | 0.0483 (0.0527) | 0.360 | 32.9 | -5.244 |
| Hand grip strength (right) | Vascular dementia (FinnGen) | rs2894602 | 2:227249802 | G/A | 0.766 | 0.0101 (0.0018) | 9.9E-09 | -0.0944 (0.0591) | 0.111 | 32.9 | -9.316 |
| Hand grip strength (right) | Vascular dementia (FinnGen) | rs113835839 | 6:13784625 | T/C | 0.248 | -0.0099 (0.0017) | 1.1E-08 | -0.0031 (0.0685) | 0.964 | 32.7 | 0.314 |
| Hand grip strength (right) | Vascular dementia (FinnGen) | rs10278546 | 7:100516003 | C/A | 0.195 | 0.0108 (0.0019) | 1.1E-08 | -0.1218 (0.0596) | 0.041 | 32.6 | -11.326 |
| Hand grip strength (right) | Vascular dementia (FinnGen) | rs7790322 | 7:2830498 | T/C | 0.416 | -0.0086 (0.0015) | 1.2E-08 | -0.0526 (0.0491) | 0.285 | 32.5 | 6.114 |
| Hand grip strength (right) | Vascular dementia (FinnGen) | rs4962700 | 10:126479989 | G/C | 0.302 | 0.0093 (0.0016) | 1.2E-08 | -0.0501 (0.0506) | 0.322 | 32.5 | -5.371 |
| Hand grip strength (right) | Vascular dementia (FinnGen) | rs8055199 | 16:84867404 | A/G | 0.660 | -0.0090 (0.0016) | 1.2E-08 | 0.0008 (0.0565) | 0.989 | 32.5 | -0.089 |
| Hand grip strength (right) | Vascular dementia (FinnGen) | rs2362972 | 3:158163272 | A/C | 0.578 | -0.0086 (0.0015) | 1.3E-08 | 0.0799 (0.0492) | 0.104 | 32.3 | -9.325 |
| Hand grip strength (right) | Vascular dementia (FinnGen) | rs10761411 | 9:136973826 | T/C | 0.812 | -0.0115 (0.0020) | 1.3E-08 | -0.0202 (0.0596) | 0.735 | 32.4 | 1.757 |
| Hand grip strength (right) | Vascular dementia (FinnGen) | rs1840753 | 2:199060177 | T/C | 0.063 | 0.0177 (0.0031) | 1.4E-08 | -0.1212 (0.1331) | 0.363 | 32.1 | -6.850 |
| Hand grip strength (right) | Vascular dementia (FinnGen) | rs10770125 | 11:2169014 | G/A | 0.477 | 0.0084 (0.0015) | 1.4E-08 | -0.0488 (0.0495) | 0.324 | 32.2 | -5.781 |
| Hand grip strength (right) | Vascular dementia (FinnGen) | rs35701422 | 3:85575775 | C/T | 0.626 | -0.0087 (0.0015) | 1.5E-08 | -0.0365 (0.0542) | 0.501 | 32.1 | 4.200 |
| Hand grip strength (right) | Vascular dementia (FinnGen) | rs852520 | 7:5695267 | A/C | 0.662 | -0.0089 (0.0016) | 1.5E-08 | -0.0554 (0.0511) | 0.278 | 32.0 | 6.223 |
| Hand grip strength (right) | Vascular dementia (FinnGen) | rs600038 | 9:136151806 | C/T | 0.207 | -0.0104 (0.0018) | 1.5E-08 | -0.0352 (0.0589) | 0.550 | 32.0 | 3.392 |
| Hand grip strength (right) | Vascular dementia (FinnGen) | rs721101 | 6:155632844 | C/T | 0.271 | 0.0095 (0.0017) | 1.6E-08 | -0.0365 (0.0624) | 0.558 | 32.0 | -3.856 |
| Hand grip strength (right) | Vascular dementia (FinnGen) | rs79723785 | 19:55818225 | C/T | 0.016 | -0.0340 (0.0060) | 1.6E-08 | 0.2174 (0.1907) | 0.254 | 31.9 | -6.385 |
| Hand grip strength (right) | Vascular dementia (FinnGen) | rs112330055 | 7:23109316 | A/G | 0.063 | 0.0180 (0.0032) | 1.7E-08 | -0.2241 (0.1107) | 0.043 | 31.8 | -12.462 |
| Hand grip strength (right) | Vascular dementia (FinnGen) | rs34845616 | 11:133792644 | A/G | 0.246 | 0.0098 (0.0017) | 1.7E-08 | -0.0496 (0.0527) | 0.347 | 31.8 | -5.064 |
| Hand grip strength (right) | Vascular dementia (FinnGen) | rs12562146 | 1:86247267 | A/T | 0.145 | 0.0119 (0.0021) | 2.0E-08 | 0.0822 (0.0750) | 0.273 | 31.5 | 6.925 |
| Hand grip strength (right) | Vascular dementia (FinnGen) | rs6962338 | 7:69160985 | G/A | 0.044 | -0.0203 (0.0036) | 2.0E-08 | -0.1648 (0.1334) | 0.217 | 31.5 | 8.099 |
| Hand grip strength (right) | Vascular dementia (FinnGen) | rs7214252 | 17:27486673 | A/G | 0.211 | -0.0102 (0.0018) | 2.0E-08 | -0.0689 (0.0571) | 0.227 | 31.5 | 6.726 |
| Hand grip strength (right) | Vascular dementia (FinnGen) | rs911642 | 20:13260252 | T/C | 0.376 | 0.0086 (0.0015) | 2.0E-08 | -0.0375 (0.0503) | 0.455 | 31.5 | -4.345 |
| Hand grip strength (right) | Vascular dementia (FinnGen) | rs35457492 | 3:70194564 | C/A | 0.495 | 0.0083 (0.0015) | 2.2E-08 | -0.0443 (0.0503) | 0.379 | 31.3 | -5.312 |
| Hand grip strength (right) | Vascular dementia (FinnGen) | rs76749769 | 16:3291408 | T/C | 0.091 | 0.0144 (0.0026) | 2.2E-08 | 0.0258 (0.0978) | 0.791 | 31.3 | 1.788 |
| Hand grip strength (right) | Vascular dementia (FinnGen) | rs4768725 | 12:46848478 | C/T | 0.700 | 0.0091 (0.0016) | 2.3E-08 | -0.0989 (0.0519) | 0.057 | 31.2 | -10.917 |
| Hand grip strength (right) | Vascular dementia (FinnGen) | rs9388051 | 6:122577108 | A/G | 0.186 | 0.0106 (0.0019) | 2.7E-08 | 0.0411 (0.0737) | 0.577 | 30.9 | 3.868 |
| Hand grip strength (right) | Vascular dementia (FinnGen) | rs1885690 | 6:109672998 | A/C | 0.410 | -0.0084 (0.0015) | 2.8E-08 | 0.1262 (0.0494) | 0.011 | 30.9 | -15.024 |
| Hand grip strength (right) | Vascular dementia (FinnGen) | rs11998884 | 9:33684436 | T/C | 0.062 | 0.0173 (0.0031) | 2.8E-08 | -0.1106 (0.0907) | 0.223 | 30.8 | -6.377 |
| Hand grip strength (right) | Vascular dementia (FinnGen) | rs12412806 | 10:24860913 | A/G | 0.294 | -0.0091 (0.0016) | 3.0E-08 | 0.0740 (0.0504) | 0.142 | 30.7 | -8.136 |
| Hand grip strength (right) | Vascular dementia (FinnGen) | rs75457267 | 5:102658770 | T/C | 0.051 | -0.0187 (0.0034) | 3.4E-08 | 0.1616 (0.1181) | 0.171 | 30.4 | -8.628 |
| Hand grip strength (right) | Vascular dementia (FinnGen) | rs13355365 | 5:154942606 | T/C | 0.380 | -0.0085 (0.0015) | 3.4E-08 | -0.0146 (0.0520) | 0.780 | 30.5 | 1.725 |
| Hand grip strength (right) | Vascular dementia (FinnGen) | rs2389763 | 7:17307847 | C/T | 0.592 | -0.0083 (0.0015) | 3.8E-08 | -0.0306 (0.0506) | 0.545 | 30.3 | 3.675 |
| Hand grip strength (right) | Vascular dementia (FinnGen) | rs4793658 | 17:45878733 | C/A | 0.110 | -0.0137 (0.0025) | 3.8E-08 | 0.0826 (0.0868) | 0.341 | 30.2 | -6.042 |
| Hand grip strength (right) | Vascular dementia (FinnGen) | rs35304341 | 1:200971049 | A/G | 0.089 | -0.0143 (0.0026) | 4.0E-08 | -0.0422 (0.0833) | 0.613 | 30.1 | 2.954 |
| Hand grip strength (right) | Vascular dementia (FinnGen) | rs7249 | 19:18391171 | T/C | 0.366 | 0.0085 (0.0015) | 4.0E-08 | 0.0126 (0.0520) | 0.809 | 30.2 | 1.486 |
| Hand grip strength (right) | Vascular dementia (FinnGen) | rs4751671 | 10:116138744 | A/G | 0.531 | 0.0082 (0.0015) | 4.1E-08 | -0.0492 (0.0517) | 0.341 | 30.1 | -5.976 |
| Hand grip strength (right) | Vascular dementia (FinnGen) | rs13169333 | 5:153064994 | C/T | 0.260 | 0.0093 (0.0017) | 4.3E-08 | 0.0310 (0.0546) | 0.570 | 30.0 | 3.337 |
| Hand grip strength (right) | Vascular dementia (FinnGen) | rs645144 | 6:141180780 | C/T | 0.330 | -0.0087 (0.0016) | 4.3E-08 | 0.0404 (0.0547) | 0.460 | 30.0 | -4.650 |
| Hand grip strength (right) | Vascular dementia (FinnGen) | rs113851275 | 9:98297220 | A/G | 0.108 | 0.0131 (0.0024) | 4.3E-08 | 0.0803 (0.0768) | 0.296 | 30.0 | 6.110 |
| Hand grip strength (right) | Vascular dementia (FinnGen) | rs2244621 | 11:64026219 | T/C | 0.144 | 0.0116 (0.0021) | 4.5E-08 | 0.1202 (0.0675) | 0.075 | 29.9 | 10.366 |
| Hand grip strength (right) | Vascular dementia (FinnGen) | rs2296316 | 14:65520246 | C/T | 0.464 | -0.0082 (0.0015) | 4.6E-08 | -0.0443 (0.0492) | 0.368 | 29.9 | 5.394 |
| Hand grip strength (right) | Alzheimer's disease, wide (FinnGen) | rs143384 | 20:34025756 | G/A | 0.404 | 0.0230 (0.0015) | 2.5E-52 | -0.0114 (0.0214) | 0.592 | 231.7 | -0.495 |
| Hand grip strength (right) | Alzheimer's disease, wide (FinnGen) | rs1556659 | 10:130834698 | T/C | 0.382 | 0.0175 (0.0015) | 3.8E-30 | -0.0001 (0.0233) | 0.996 | 130.2 | -0.006 |
| Hand grip strength (right) | Alzheimer's disease, wide (FinnGen) | rs3118914 | 13:51116901 | T/G | 0.215 | -0.0194 (0.0018) | 5.9E-27 | -0.0380 (0.0243) | 0.118 | 115.6 | 1.955 |
| Hand grip strength (right) | Alzheimer's disease, wide (FinnGen) | rs12316046 | 12:15054415 | G/A | 0.378 | -0.0162 (0.0015) | 3.8E-26 | -0.0175 (0.0238) | 0.462 | 111.9 | 1.080 |
| Hand grip strength (right) | Alzheimer's disease, wide (FinnGen) | rs10846071 | 12:15016236 | T/C | 0.394 | -0.0157 (0.0015) | 6.5E-25 | -0.0202 (0.0230) | 0.380 | 106.2 | 1.290 |
| Hand grip strength (right) | Alzheimer's disease, wide (FinnGen) | rs13146142 | 4:17931318 | C/T | 0.159 | -0.0208 (0.0020) | 1.4E-24 | 0.0470 (0.0361) | 0.193 | 104.8 | -2.260 |
| Hand grip strength (right) | Alzheimer's disease, wide (FinnGen) | rs2871865 | 15:99194896 | G/C | 0.116 | -0.0237 (0.0023) | 1.8E-24 | 0.0749 (0.0441) | 0.089 | 104.2 | -3.162 |
| Hand grip strength (right) | Alzheimer's disease, wide (FinnGen) | rs9267806 | 6:32110886 | A/G | 0.256 | -0.0167 (0.0017) | 7.7E-23 | -0.0274 (0.0226) | 0.225 | 96.8 | 1.636 |
| Hand grip strength (right) | Alzheimer's disease, wide (FinnGen) | rs7451021 | 6:130381246 | C/T | 0.689 | -0.0158 (0.0016) | 7.7E-23 | 0.0372 (0.0227) | 0.102 | 96.8 | -2.355 |
| Hand grip strength (right) | Alzheimer's disease, wide (FinnGen) | rs12790261 | 11:66988048 | A/C | 0.082 | -0.0264 (0.0027) | 2.0E-22 | 0.0225 (0.0484) | 0.642 | 94.9 | -0.853 |
| Hand grip strength (right) | Alzheimer's disease, wide (FinnGen) | rs13107325 | 4:103188709 | T/C | 0.075 | -0.0275 (0.0028) | 2.1E-22 | 0.1306 (0.0917) | 0.154 | 94.8 | -4.749 |
| Hand grip strength (right) | Alzheimer's disease, wide (FinnGen) | rs10798483 | 1:176799143 | A/G | 0.547 | 0.0145 (0.0015) | 2.8E-22 | -0.0059 (0.0213) | 0.782 | 94.2 | -0.407 |
| Hand grip strength (right) | Alzheimer's disease, wide (FinnGen) | rs3771498 | 2:70720070 | T/C | 0.514 | 0.0142 (0.0015) | 1.9E-21 | -0.0370 (0.0212) | 0.081 | 90.5 | -2.613 |
| Hand grip strength (right) | Alzheimer's disease, wide (FinnGen) | rs4369779 | 18:20735408 | C/T | 0.789 | 0.0172 (0.0018) | 3.4E-21 | 0.0263 (0.0252) | 0.297 | 89.3 | 1.529 |
| Hand grip strength (right) | Alzheimer's disease, wide (FinnGen) | rs1043515 | 17:36922196 | G/A | 0.566 | 0.0138 (0.0015) | 2.8E-20 | 0.0115 (0.0212) | 0.589 | 85.2 | 0.832 |
| Hand grip strength (right) | Alzheimer's disease, wide (FinnGen) | rs1550115 | 2:25041620 | T/C | 0.749 | 0.0153 (0.0017) | 4.5E-19 | -0.0041 (0.0251) | 0.869 | 79.6 | -0.268 |
| Hand grip strength (right) | Alzheimer's disease, wide (FinnGen) | rs4784329 | 16:53910261 | C/A | 0.426 | -0.0133 (0.0015) | 9.0E-19 | 0.0080 (0.0212) | 0.707 | 78.3 | -0.600 |
| Hand grip strength (right) | Alzheimer's disease, wide (FinnGen) | rs12473839 | 2:135638044 | T/C | 0.371 | 0.0133 (0.0015) | 4.0E-18 | 0.0137 (0.0217) | 0.528 | 75.3 | 1.032 |
| Hand grip strength (right) | Alzheimer's disease, wide (FinnGen) | rs4927015 | 1:54072471 | A/G | 0.583 | 0.0131 (0.0015) | 5.0E-18 | 0.0216 (0.0227) | 0.342 | 74.9 | 1.654 |
| Hand grip strength (right) | Alzheimer's disease, wide (FinnGen) | rs635538 | 18:53273614 | A/G | 0.914 | -0.0220 (0.0027) | 1.5E-16 | 0.0625 (0.0350) | 0.074 | 68.2 | -2.842 |
| Hand grip strength (right) | Alzheimer's disease, wide (FinnGen) | rs823130 | 1:205714372 | T/C | 0.433 | -0.0124 (0.0015) | 1.7E-16 | 0.0300 (0.0214) | 0.162 | 67.9 | -2.422 |
| Hand grip strength (right) | Alzheimer's disease, wide (FinnGen) | rs2165241 | 15:74222202 | C/T | 0.509 | 0.0122 (0.0015) | 1.9E-16 | 0.0051 (0.0211) | 0.809 | 67.7 | 0.416 |
| Hand grip strength (right) | Alzheimer's disease, wide (FinnGen) | rs6977081 | 7:150542515 | T/G | 0.334 | 0.0129 (0.0016) | 5.7E-16 | 0.0131 (0.0221) | 0.554 | 65.5 | 1.019 |
| Hand grip strength (right) | Alzheimer's disease, wide (FinnGen) | rs56365901 | 17:43960323 | G/A | 0.223 | -0.0143 (0.0018) | 1.8E-15 | -0.0987 (0.0393) | 0.012 | 63.3 | 6.921 |
| Hand grip strength (right) | Alzheimer's disease, wide (FinnGen) | rs7206195 | 16:2145280 | T/C | 0.180 | -0.0153 (0.0019) | 2.8E-15 | 0.0441 (0.0258) | 0.087 | 62.4 | -2.878 |
| Hand grip strength (right) | Alzheimer's disease, wide (FinnGen) | rs10520770 | 18:46602964 | C/T | 0.449 | 0.0117 (0.0015) | 4.5E-15 | 0.0276 (0.0228) | 0.226 | 61.5 | 2.351 |
| Hand grip strength (right) | Alzheimer's disease, wide (FinnGen) | rs11243202 | 6:7719065 | C/T | 0.486 | 0.0116 (0.0015) | 6.5E-15 | 0.0139 (0.0213) | 0.514 | 60.8 | 1.194 |
| Hand grip strength (right) | Alzheimer's disease, wide (FinnGen) | rs150330307 | 1:160160801 | C/T | 0.032 | -0.0326 (0.0042) | 1.2E-14 | -0.0963 (0.0767) | 0.209 | 59.5 | 2.956 |
| Hand grip strength (right) | Alzheimer's disease, wide (FinnGen) | rs2208562 | 9:119344528 | T/C | 0.610 | -0.0116 (0.0015) | 3.1E-14 | -0.0323 (0.0222) | 0.146 | 57.7 | 2.794 |
| Hand grip strength (right) | Alzheimer's disease, wide (FinnGen) | rs10799428 | 1:227798565 | T/C | 0.187 | -0.0144 (0.0019) | 4.6E-14 | 0.0011 (0.0267) | 0.966 | 56.9 | -0.077 |
| Hand grip strength (right) | Alzheimer's disease, wide (FinnGen) | rs2631360 | 5:131707429 | A/G | 0.519 | -0.0112 (0.0015) | 5.4E-14 | 0.0152 (0.0223) | 0.497 | 56.6 | -1.360 |
| Hand grip strength (right) | Alzheimer's disease, wide (FinnGen) | rs10784502 | 12:66343810 | T/C | 0.512 | -0.0111 (0.0015) | 7.1E-14 | -0.0121 (0.0212) | 0.567 | 56.0 | 1.088 |
| Hand grip strength (right) | Alzheimer's disease, wide (FinnGen) | rs7963801 | 12:79685226 | C/T | 0.572 | -0.0113 (0.0015) | 8.4E-14 | -0.0134 (0.0216) | 0.536 | 55.7 | 1.189 |
| Hand grip strength (right) | Alzheimer's disease, wide (FinnGen) | rs2431112 | 5:103931707 | A/G | 0.441 | -0.0112 (0.0015) | 8.6E-14 | 0.0230 (0.0217) | 0.289 | 55.7 | -2.059 |
| Hand grip strength (right) | Alzheimer's disease, wide (FinnGen) | rs1641457 | 2:40421990 | G/T | 0.223 | 0.0131 (0.0018) | 2.4E-13 | -0.0017 (0.0233) | 0.940 | 53.6 | -0.130 |
| Hand grip strength (right) | Alzheimer's disease, wide (FinnGen) | rs6693965 | 1:10378416 | T/G | 0.128 | -0.0163 (0.0022) | 3.2E-13 | 0.0233 (0.0340) | 0.492 | 53.1 | -1.434 |
| Hand grip strength (right) | Alzheimer's disease, wide (FinnGen) | rs9652468 | 15:56823913 | A/G | 0.249 | -0.0125 (0.0017) | 3.3E-13 | 0.0165 (0.0230) | 0.472 | 53.0 | -1.317 |
| Hand grip strength (right) | Alzheimer's disease, wide (FinnGen) | rs2273555 | 10:104127171 | A/G | 0.606 | 0.0110 (0.0015) | 4.2E-13 | -0.0018 (0.0217) | 0.934 | 52.6 | -0.163 |
| Hand grip strength (right) | Alzheimer's disease, wide (FinnGen) | rs7301953 | 12:124405871 | A/G | 0.312 | -0.0115 (0.0016) | 6.4E-13 | -0.0106 (0.0234) | 0.651 | 51.7 | 0.919 |
| Hand grip strength (right) | Alzheimer's disease, wide (FinnGen) | rs7196917 | 16:69896527 | G/A | 0.430 | -0.0106 (0.0015) | 1.9E-12 | 0.0238 (0.0218) | 0.275 | 49.6 | -2.249 |
| Hand grip strength (right) | Alzheimer's disease, wide (FinnGen) | rs2717351 | 7:19019880 | G/A | 0.212 | 0.0128 (0.0018) | 3.4E-12 | 0.0099 (0.0243) | 0.683 | 48.4 | 0.775 |
| Hand grip strength (right) | Alzheimer's disease, wide (FinnGen) | rs9322822 | 6:105369598 | T/C | 0.320 | 0.0111 (0.0016) | 3.5E-12 | 0.0374 (0.0228) | 0.101 | 48.4 | 3.375 |
| Hand grip strength (right) | Alzheimer's disease, wide (FinnGen) | rs7565148 | 2:44188396 | G/T | 0.501 | -0.0104 (0.0015) | 3.6E-12 | 0.0418 (0.0212) | 0.049 | 48.3 | -4.038 |
| Hand grip strength (right) | Alzheimer's disease, wide (FinnGen) | rs72820369 | 10:81251539 | T/A | 0.118 | 0.0161 (0.0023) | 4.4E-12 | -0.0187 (0.0301) | 0.534 | 47.9 | -1.160 |
| Hand grip strength (right) | Alzheimer's disease, wide (FinnGen) | rs4785574 | 16:89568875 | G/A | 0.555 | -0.0104 (0.0015) | 4.5E-12 | -0.0239 (0.0216) | 0.270 | 47.9 | 2.303 |
| Hand grip strength (right) | Alzheimer's disease, wide (FinnGen) | rs2854152 | 17:61986027 | G/A | 0.678 | 0.0110 (0.0016) | 6.0E-12 | 0.0310 (0.0220) | 0.158 | 47.3 | 2.820 |
| Hand grip strength (right) | Alzheimer's disease, wide (FinnGen) | rs9853018 | 3:141101961 | T/C | 0.443 | 0.0102 (0.0015) | 8.8E-12 | 0.0409 (0.0213) | 0.055 | 46.6 | 4.011 |
| Hand grip strength (right) | Alzheimer's disease, wide (FinnGen) | rs7575451 | 2:152352843 | G/C | 0.650 | -0.0106 (0.0016) | 9.3E-12 | 0.0065 (0.0228) | 0.776 | 46.5 | -0.613 |
| Hand grip strength (right) | Alzheimer's disease, wide (FinnGen) | rs12452505 | 17:63556402 | G/C | 0.142 | -0.0144 (0.0021) | 1.4E-11 | 0.0281 (0.0355) | 0.428 | 45.7 | -1.947 |
| Hand grip strength (right) | Alzheimer's disease, wide (FinnGen) | rs62509875 | 8:110360944 | G/A | 0.171 | -0.0132 (0.0020) | 2.4E-11 | -0.0285 (0.0310) | 0.358 | 44.6 | 2.162 |
| Hand grip strength (right) | Alzheimer's disease, wide (FinnGen) | rs4802848 | 19:52218342 | C/G | 0.730 | 0.0111 (0.0017) | 3.8E-11 | 0.0435 (0.0266) | 0.102 | 43.7 | 3.927 |
| Hand grip strength (right) | Alzheimer's disease, wide (FinnGen) | rs4121165 | 1:78276977 | A/G | 0.211 | -0.0120 (0.0018) | 4.8E-11 | 0.0387 (0.0281) | 0.170 | 43.2 | -3.231 |
| Hand grip strength (right) | Alzheimer's disease, wide (FinnGen) | rs13106087 | 4:145566864 | C/T | 0.830 | 0.0129 (0.0020) | 6.2E-11 | 0.0132 (0.0285) | 0.644 | 42.8 | 1.021 |
| Hand grip strength (right) | Alzheimer's disease, wide (FinnGen) | rs1892425 | 1:41744821 | A/G | 0.239 | 0.0113 (0.0018) | 1.1E-10 | -0.0138 (0.0246) | 0.575 | 41.6 | -1.217 |
| Hand grip strength (right) | Alzheimer's disease, wide (FinnGen) | rs7657558 | 4:30648636 | G/T | 0.720 | 0.0107 (0.0017) | 1.1E-10 | 0.0127 (0.0240) | 0.595 | 41.6 | 1.184 |
| Hand grip strength (right) | Alzheimer's disease, wide (FinnGen) | rs12763284 | 10:104508202 | G/A | 0.466 | 0.0096 (0.0015) | 1.2E-10 | -0.0033 (0.0213) | 0.877 | 41.5 | -0.344 |
| Hand grip strength (right) | Alzheimer's disease, wide (FinnGen) | rs34217742 | 19:37376830 | A/T | 0.124 | 0.0146 (0.0023) | 1.4E-10 | 0.0177 (0.0305) | 0.562 | 41.2 | 1.210 |
| Hand grip strength (right) | Alzheimer's disease, wide (FinnGen) | rs12823922 | 12:24186697 | G/A | 0.222 | -0.0114 (0.0018) | 1.6E-10 | -0.0124 (0.0250) | 0.620 | 40.9 | 1.086 |
| Hand grip strength (right) | Alzheimer's disease, wide (FinnGen) | rs116922558 | 9:118802375 | G/A | 0.040 | -0.0246 (0.0039) | 1.8E-10 | 0.0586 (0.0510) | 0.250 | 40.7 | -2.379 |
| Hand grip strength (right) | Alzheimer's disease, wide (FinnGen) | rs1125 | 6:149979416 | A/G | 0.337 | -0.0100 (0.0016) | 1.9E-10 | -0.0090 (0.0220) | 0.684 | 40.6 | 0.897 |
| Hand grip strength (right) | Alzheimer's disease, wide (FinnGen) | rs12914702 | 15:96887277 | A/G | 0.300 | 0.0108 (0.0017) | 1.9E-10 | -0.0083 (0.0228) | 0.717 | 40.5 | -0.770 |
| Hand grip strength (right) | Alzheimer's disease, wide (FinnGen) | rs2194411 | 3:185548663 | A/G | 0.128 | 0.0143 (0.0022) | 2.1E-10 | 0.0294 (0.0311) | 0.345 | 40.4 | 2.058 |
| Hand grip strength (right) | Alzheimer's disease, wide (FinnGen) | rs4737446 | 8:57665019 | T/G | 0.695 | 0.0103 (0.0016) | 2.4E-10 | -0.0304 (0.0227) | 0.181 | 40.1 | -2.961 |
| Hand grip strength (right) | Alzheimer's disease, wide (FinnGen) | rs77485342 | 6:30842866 | T/C | 0.018 | 0.0353 (0.0056) | 2.6E-10 | 0.0979 (0.0668) | 0.143 | 40.0 | 2.770 |
| Hand grip strength (right) | Alzheimer's disease, wide (FinnGen) | rs7871404 | 9:99262296 | G/A | 0.189 | 0.0119 (0.0019) | 3.4E-10 | -0.0627 (0.0288) | 0.030 | 39.4 | -5.254 |
| Hand grip strength (right) | Alzheimer's disease, wide (FinnGen) | rs246181 | 16:14392641 | T/C | 0.373 | 0.0097 (0.0015) | 3.7E-10 | -0.0033 (0.0218) | 0.881 | 39.3 | -0.340 |
| Hand grip strength (right) | Alzheimer's disease, wide (FinnGen) | rs56144131 | 1:208977656 | C/T | 0.149 | -0.0131 (0.0021) | 3.8E-10 | 0.0143 (0.0293) | 0.625 | 39.2 | -1.094 |
| Hand grip strength (right) | Alzheimer's disease, wide (FinnGen) | rs11039348 | 11:47728617 | A/G | 0.348 | -0.0098 (0.0016) | 3.9E-10 | -0.0415 (0.0230) | 0.071 | 39.2 | 4.248 |
| Hand grip strength (right) | Alzheimer's disease, wide (FinnGen) | rs1952256 | 1:184035116 | G/A | 0.345 | 0.0098 (0.0016) | 4.1E-10 | 0.0288 (0.0216) | 0.183 | 39.0 | 2.948 |
| Hand grip strength (right) | Alzheimer's disease, wide (FinnGen) | rs10193039 | 2:99692373 | T/A | 0.281 | -0.0103 (0.0017) | 4.1E-10 | 0.0184 (0.0243) | 0.448 | 39.1 | -1.782 |
| Hand grip strength (right) | Alzheimer's disease, wide (FinnGen) | rs76895963 | 12:4384844 | G/T | 0.021 | 0.0360 (0.0058) | 4.1E-10 | -0.0216 (0.0641) | 0.736 | 39.0 | -0.600 |
| Hand grip strength (right) | Alzheimer's disease, wide (FinnGen) | rs4553566 | 15:58336319 | C/T | 0.453 | -0.0093 (0.0015) | 4.6E-10 | 0.0026 (0.0212) | 0.904 | 38.9 | -0.280 |
| Hand grip strength (right) | Alzheimer's disease, wide (FinnGen) | rs7266065 | 20:47531817 | A/G | 0.323 | 0.0099 (0.0016) | 4.6E-10 | 0.0260 (0.0237) | 0.272 | 38.8 | 2.616 |
| Hand grip strength (right) | Alzheimer's disease, wide (FinnGen) | rs3848369 | 16:415078 | T/C | 0.387 | -0.0095 (0.0015) | 4.7E-10 | -0.0365 (0.0216) | 0.091 | 38.8 | 3.827 |
| Hand grip strength (right) | Alzheimer's disease, wide (FinnGen) | rs6006984 | 22:45714937 | C/T | 0.278 | 0.0103 (0.0017) | 5.1E-10 | 0.0027 (0.0233) | 0.908 | 38.6 | 0.262 |
| Hand grip strength (right) | Alzheimer's disease, wide (FinnGen) | rs7576964 | 2:48601448 | T/G | 0.342 | 0.0097 (0.0016) | 5.3E-10 | 0.0224 (0.0217) | 0.303 | 38.6 | 2.299 |
| Hand grip strength (right) | Alzheimer's disease, wide (FinnGen) | rs12899474 | 15:77391603 | T/C | 0.108 | -0.0149 (0.0024) | 5.4E-10 | -0.0459 (0.0356) | 0.197 | 38.5 | 3.082 |
| Hand grip strength (right) | Alzheimer's disease, wide (FinnGen) | rs9396861 | 6:18404133 | A/C | 0.599 | -0.0096 (0.0016) | 5.7E-10 | 0.0413 (0.0248) | 0.095 | 38.4 | -4.289 |
| Hand grip strength (right) | Alzheimer's disease, wide (FinnGen) | rs1442883 | 2:59970660 | A/C | 0.253 | -0.0106 (0.0017) | 5.8E-10 | -0.0147 (0.0257) | 0.568 | 38.4 | 1.384 |
| Hand grip strength (right) | Alzheimer's disease, wide (FinnGen) | rs12889267 | 14:21542766 | G/A | 0.167 | -0.0123 (0.0020) | 6.7E-10 | 0.0141 (0.0325) | 0.663 | 38.1 | -1.148 |
| Hand grip strength (right) | Alzheimer's disease, wide (FinnGen) | rs58670122 | 1:22492613 | G/A | 0.143 | -0.0132 (0.0021) | 7.4E-10 | 0.0590 (0.0350) | 0.092 | 37.9 | -4.462 |
| Hand grip strength (right) | Alzheimer's disease, wide (FinnGen) | rs4549685 | 7:39326478 | T/C | 0.330 | 0.0097 (0.0016) | 8.1E-10 | -0.0065 (0.0226) | 0.775 | 37.8 | -0.669 |
| Hand grip strength (right) | Alzheimer's disease, wide (FinnGen) | rs2587505 | 17:77784268 | C/T | 0.420 | -0.0093 (0.0015) | 8.1E-10 | 0.0283 (0.0226) | 0.211 | 37.8 | -3.052 |
| Hand grip strength (right) | Alzheimer's disease, wide (FinnGen) | rs9757079 | 3:53155158 | T/C | 0.318 | 0.0098 (0.0016) | 8.4E-10 | 0.0102 (0.0229) | 0.654 | 37.7 | 1.041 |
| Hand grip strength (right) | Alzheimer's disease, wide (FinnGen) | rs62234790 | 3:13750642 | A/C | 0.246 | 0.0106 (0.0017) | 8.8E-10 | 0.0074 (0.0239) | 0.756 | 37.6 | 0.699 |
| Hand grip strength (right) | Alzheimer's disease, wide (FinnGen) | rs34587452 | 4:1009900 | C/G | 0.215 | -0.0111 (0.0018) | 9.1E-10 | 0.0629 (0.0266) | 0.018 | 37.5 | -5.673 |
| Hand grip strength (right) | Alzheimer's disease, wide (FinnGen) | rs4868110 | 5:171164168 | T/A | 0.323 | -0.0097 (0.0016) | 1.0E-09 | 0.0155 (0.0221) | 0.482 | 37.3 | -1.596 |
| Hand grip strength (right) | Alzheimer's disease, wide (FinnGen) | rs11022513 | 11:12840986 | T/C | 0.569 | -0.0092 (0.0015) | 1.1E-09 | -0.0255 (0.0216) | 0.239 | 37.2 | 2.769 |
| Hand grip strength (right) | Alzheimer's disease, wide (FinnGen) | rs1047891 | 2:211540507 | A/C | 0.316 | 0.0097 (0.0016) | 1.3E-09 | -0.0433 (0.0228) | 0.057 | 36.8 | -4.470 |
| Hand grip strength (right) | Alzheimer's disease, wide (FinnGen) | rs12101479 | 15:74248548 | C/G | 0.237 | -0.0106 (0.0018) | 1.5E-09 | -0.0166 (0.0231) | 0.471 | 36.6 | 1.564 |
| Hand grip strength (right) | Alzheimer's disease, wide (FinnGen) | rs2341184 | 3:196930781 | C/T | 0.272 | 0.0101 (0.0017) | 1.7E-09 | -0.0100 (0.0228) | 0.662 | 36.2 | -0.994 |
| Hand grip strength (right) | Alzheimer's disease, wide (FinnGen) | rs6792762 | 3:38574491 | A/G | 0.420 | -0.0091 (0.0015) | 1.8E-09 | 0.0164 (0.0215) | 0.446 | 36.2 | -1.799 |
| Hand grip strength (right) | Alzheimer's disease, wide (FinnGen) | rs935728 | 14:80957923 | T/C | 0.328 | 0.0096 (0.0016) | 1.8E-09 | -0.0087 (0.0222) | 0.696 | 36.2 | -0.911 |
| Hand grip strength (right) | Alzheimer's disease, wide (FinnGen) | rs2194747 | 2:218126181 | G/A | 0.707 | 0.0099 (0.0016) | 1.9E-09 | 0.0086 (0.0221) | 0.697 | 36.1 | 0.873 |
| Hand grip strength (right) | Alzheimer's disease, wide (FinnGen) | rs62037412 | 16:28917746 | A/G | 0.357 | 0.0093 (0.0016) | 1.9E-09 | 0.0421 (0.0214) | 0.050 | 36.0 | 4.512 |
| Hand grip strength (right) | Alzheimer's disease, wide (FinnGen) | rs16870531 | 7:120660682 | T/C | 0.238 | 0.0104 (0.0017) | 2.2E-09 | -0.0058 (0.0245) | 0.813 | 35.8 | -0.555 |
| Hand grip strength (right) | Alzheimer's disease, wide (FinnGen) | rs6592728 | 11:77247260 | G/A | 0.371 | -0.0092 (0.0015) | 2.5E-09 | -0.0225 (0.0215) | 0.296 | 35.5 | 2.450 |
| Hand grip strength (right) | Alzheimer's disease, wide (FinnGen) | rs1486925 | 8:78827617 | C/T | 0.315 | -0.0095 (0.0016) | 2.9E-09 | -0.0179 (0.0237) | 0.451 | 35.3 | 1.879 |
| Hand grip strength (right) | Alzheimer's disease, wide (FinnGen) | rs6870324 | 5:141787317 | G/C | 0.270 | -0.0100 (0.0017) | 3.0E-09 | -0.0188 (0.0253) | 0.458 | 35.2 | 1.883 |
| Hand grip strength (right) | Alzheimer's disease, wide (FinnGen) | rs2226685 | 21:40069825 | C/T | 0.759 | 0.0103 (0.0017) | 3.1E-09 | 0.0045 (0.0233) | 0.847 | 35.1 | 0.435 |
| Hand grip strength (right) | Alzheimer's disease, wide (FinnGen) | rs34030812 | 2:144248905 | C/T | 0.367 | -0.0091 (0.0015) | 3.3E-09 | 0.0091 (0.0219) | 0.678 | 35.0 | -0.997 |
| Hand grip strength (right) | Alzheimer's disease, wide (FinnGen) | rs10483727 | 14:61072875 | C/T | 0.610 | -0.0090 (0.0015) | 3.3E-09 | 0.0368 (0.0234) | 0.116 | 35.0 | -4.081 |
| Hand grip strength (right) | Alzheimer's disease, wide (FinnGen) | rs61389091 | 11:74427921 | T/C | 0.042 | 0.0221 (0.0037) | 3.6E-09 | -0.0399 (0.0535) | 0.455 | 34.8 | -1.809 |
| Hand grip strength (right) | Alzheimer's disease, wide (FinnGen) | rs997850 | 4:154838434 | C/G | 0.605 | -0.0090 (0.0015) | 3.7E-09 | -0.0050 (0.0212) | 0.814 | 34.8 | 0.554 |
| Hand grip strength (right) | Alzheimer's disease, wide (FinnGen) | rs2322754 | 6:81046299 | A/G | 0.832 | -0.0117 (0.0020) | 3.8E-09 | 0.0008 (0.0269) | 0.977 | 34.7 | -0.068 |
| Hand grip strength (right) | Alzheimer's disease, wide (FinnGen) | rs56074046 | 17:7358930 | A/G | 0.372 | -0.0090 (0.0015) | 4.4E-09 | -0.0014 (0.0235) | 0.951 | 34.4 | 0.155 |
| Hand grip strength (right) | Alzheimer's disease, wide (FinnGen) | rs12052508 | 2:72757843 | T/C | 0.879 | -0.0134 (0.0023) | 4.7E-09 | 0.0155 (0.0358) | 0.664 | 34.3 | -1.161 |
| Hand grip strength (right) | Alzheimer's disease, wide (FinnGen) | rs7148603 | 14:36683779 | A/G | 0.359 | 0.0093 (0.0016) | 4.7E-09 | 0.0233 (0.0229) | 0.307 | 34.3 | 2.504 |
| Hand grip strength (right) | Alzheimer's disease, wide (FinnGen) | rs7034200 | 9:4289050 | A/C | 0.480 | 0.0087 (0.0015) | 5.3E-09 | 0.0005 (0.0212) | 0.982 | 34.1 | 0.058 |
| Hand grip strength (right) | Alzheimer's disease, wide (FinnGen) | rs12616285 | 2:169156907 | G/T | 0.149 | 0.0122 (0.0021) | 5.4E-09 | -0.0011 (0.0274) | 0.968 | 34.0 | -0.090 |
| Hand grip strength (right) | Alzheimer's disease, wide (FinnGen) | rs6473015 | 8:78178485 | C/A | 0.286 | 0.0096 (0.0016) | 5.5E-09 | -0.0111 (0.0258) | 0.667 | 34.0 | -1.156 |
| Hand grip strength (right) | Alzheimer's disease, wide (FinnGen) | rs7549184 | 1:215412906 | A/G | 0.787 | 0.0106 (0.0018) | 5.8E-09 | 0.0168 (0.0262) | 0.522 | 33.9 | 1.591 |
| Hand grip strength (right) | Alzheimer's disease, wide (FinnGen) | rs2147461 | 1:191051627 | C/T | 0.117 | 0.0134 (0.0023) | 6.1E-09 | -0.0310 (0.0285) | 0.278 | 33.8 | -2.308 |
| Hand grip strength (right) | Alzheimer's disease, wide (FinnGen) | rs6693567 | 1:150510660 | T/C | 0.733 | -0.0097 (0.0017) | 6.3E-09 | 0.0062 (0.0259) | 0.810 | 33.8 | -0.636 |
| Hand grip strength (right) | Alzheimer's disease, wide (FinnGen) | rs4752689 | 10:124131176 | A/G | 0.584 | 0.0088 (0.0015) | 6.4E-09 | -0.0730 (0.0233) | 0.002 | 33.7 | -8.340 |
| Hand grip strength (right) | Alzheimer's disease, wide (FinnGen) | rs12522139 | 5:122699812 | G/T | 0.171 | -0.0114 (0.0020) | 6.8E-09 | -0.0294 (0.0277) | 0.290 | 33.6 | 2.570 |
| Hand grip strength (right) | Alzheimer's disease, wide (FinnGen) | rs6882168 | 5:39402647 | T/C | 0.337 | -0.0091 (0.0016) | 7.9E-09 | -0.0245 (0.0216) | 0.258 | 33.3 | 2.694 |
| Hand grip strength (right) | Alzheimer's disease, wide (FinnGen) | rs114924396 | 4:119755621 | G/A | 0.054 | -0.0191 (0.0033) | 8.2E-09 | 0.0348 (0.0440) | 0.429 | 33.2 | -1.820 |
| Hand grip strength (right) | Alzheimer's disease, wide (FinnGen) | rs248831 | 16:11281218 | A/G | 0.265 | 0.0099 (0.0017) | 8.4E-09 | -0.0008 (0.0253) | 0.975 | 33.2 | -0.081 |
| Hand grip strength (right) | Alzheimer's disease, wide (FinnGen) | rs35833641 | 2:179462494 | G/A | 0.312 | 0.0092 (0.0016) | 8.5E-09 | -0.0263 (0.0220) | 0.231 | 33.1 | -2.852 |
| Hand grip strength (right) | Alzheimer's disease, wide (FinnGen) | rs6715064 | 2:220041928 | T/C | 0.310 | -0.0092 (0.0016) | 9.6E-09 | 0.0156 (0.0229) | 0.496 | 32.9 | -1.694 |
| Hand grip strength (right) | Alzheimer's disease, wide (FinnGen) | rs2894602 | 2:227249802 | G/A | 0.766 | 0.0101 (0.0018) | 9.9E-09 | -0.0136 (0.0256) | 0.596 | 32.9 | -1.342 |
| Hand grip strength (right) | Alzheimer's disease, wide (FinnGen) | rs113835839 | 6:13784625 | T/C | 0.248 | -0.0099 (0.0017) | 1.1E-08 | 0.0164 (0.0297) | 0.581 | 32.7 | -1.663 |
| Hand grip strength (right) | Alzheimer's disease, wide (FinnGen) | rs10278546 | 7:100516003 | C/A | 0.195 | 0.0108 (0.0019) | 1.1E-08 | -0.0515 (0.0258) | 0.045 | 32.6 | -4.789 |
| Hand grip strength (right) | Alzheimer's disease, wide (FinnGen) | rs7790322 | 7:2830498 | T/C | 0.416 | -0.0086 (0.0015) | 1.2E-08 | 0.0227 (0.0212) | 0.284 | 32.5 | -2.639 |
| Hand grip strength (right) | Alzheimer's disease, wide (FinnGen) | rs4962700 | 10:126479989 | G/C | 0.302 | 0.0093 (0.0016) | 1.2E-08 | 0.0093 (0.0217) | 0.667 | 32.5 | 0.997 |
| Hand grip strength (right) | Alzheimer's disease, wide (FinnGen) | rs8055199 | 16:84867404 | A/G | 0.660 | -0.0090 (0.0016) | 1.2E-08 | -0.0063 (0.0244) | 0.796 | 32.5 | 0.703 |
| Hand grip strength (right) | Alzheimer's disease, wide (FinnGen) | rs2362972 | 3:158163272 | A/C | 0.578 | -0.0086 (0.0015) | 1.3E-08 | -0.0216 (0.0213) | 0.310 | 32.3 | 2.521 |
| Hand grip strength (right) | Alzheimer's disease, wide (FinnGen) | rs10761411 | 9:136973826 | T/C | 0.812 | -0.0115 (0.0020) | 1.3E-08 | -0.0034 (0.0257) | 0.894 | 32.4 | 0.296 |
| Hand grip strength (right) | Alzheimer's disease, wide (FinnGen) | rs1840753 | 2:199060177 | T/C | 0.063 | 0.0177 (0.0031) | 1.4E-08 | -0.1656 (0.0598) | 0.006 | 32.1 | -9.359 |
| Hand grip strength (right) | Alzheimer's disease, wide (FinnGen) | rs10770125 | 11:2169014 | G/A | 0.477 | 0.0084 (0.0015) | 1.4E-08 | -0.0035 (0.0213) | 0.871 | 32.2 | -0.415 |
| Hand grip strength (right) | Alzheimer's disease, wide (FinnGen) | rs35701422 | 3:85575775 | C/T | 0.626 | -0.0087 (0.0015) | 1.5E-08 | -0.0271 (0.0235) | 0.249 | 32.1 | 3.118 |
| Hand grip strength (right) | Alzheimer's disease, wide (FinnGen) | rs852520 | 7:5695267 | A/C | 0.662 | -0.0089 (0.0016) | 1.5E-08 | 0.0109 (0.0222) | 0.624 | 32.0 | -1.224 |
| Hand grip strength (right) | Alzheimer's disease, wide (FinnGen) | rs600038 | 9:136151806 | C/T | 0.207 | -0.0104 (0.0018) | 1.5E-08 | -0.0169 (0.0255) | 0.509 | 32.0 | 1.629 |
| Hand grip strength (right) | Alzheimer's disease, wide (FinnGen) | rs721101 | 6:155632844 | C/T | 0.271 | 0.0095 (0.0017) | 1.6E-08 | -0.0162 (0.0269) | 0.547 | 32.0 | -1.711 |
| Hand grip strength (right) | Alzheimer's disease, wide (FinnGen) | rs79723785 | 19:55818225 | C/T | 0.016 | -0.0340 (0.0060) | 1.6E-08 | 0.0677 (0.0828) | 0.414 | 31.9 | -1.988 |
| Hand grip strength (right) | Alzheimer's disease, wide (FinnGen) | rs112330055 | 7:23109316 | A/G | 0.063 | 0.0180 (0.0032) | 1.7E-08 | -0.0217 (0.0476) | 0.649 | 31.8 | -1.207 |
| Hand grip strength (right) | Alzheimer's disease, wide (FinnGen) | rs34845616 | 11:133792644 | A/G | 0.246 | 0.0098 (0.0017) | 1.7E-08 | -0.0366 (0.0227) | 0.106 | 31.8 | -3.737 |
| Hand grip strength (right) | Alzheimer's disease, wide (FinnGen) | rs12562146 | 1:86247267 | A/T | 0.145 | 0.0119 (0.0021) | 2.0E-08 | 0.0152 (0.0324) | 0.640 | 31.5 | 1.280 |
| Hand grip strength (right) | Alzheimer's disease, wide (FinnGen) | rs6962338 | 7:69160985 | G/A | 0.044 | -0.0203 (0.0036) | 2.0E-08 | -0.0162 (0.0574) | 0.778 | 31.5 | 0.796 |
| Hand grip strength (right) | Alzheimer's disease, wide (FinnGen) | rs7214252 | 17:27486673 | A/G | 0.211 | -0.0102 (0.0018) | 2.0E-08 | -0.0204 (0.0247) | 0.408 | 31.5 | 1.991 |
| Hand grip strength (right) | Alzheimer's disease, wide (FinnGen) | rs911642 | 20:13260252 | T/C | 0.376 | 0.0086 (0.0015) | 2.0E-08 | 0.0160 (0.0217) | 0.462 | 31.5 | 1.854 |
| Hand grip strength (right) | Alzheimer's disease, wide (FinnGen) | rs35457492 | 3:70194564 | C/A | 0.495 | 0.0083 (0.0015) | 2.2E-08 | -0.0186 (0.0217) | 0.391 | 31.3 | -2.231 |
| Hand grip strength (right) | Alzheimer's disease, wide (FinnGen) | rs76749769 | 16:3291408 | T/C | 0.091 | 0.0144 (0.0026) | 2.2E-08 | -0.0218 (0.0425) | 0.608 | 31.3 | -1.511 |
| Hand grip strength (right) | Alzheimer's disease, wide (FinnGen) | rs4768725 | 12:46848478 | C/T | 0.700 | 0.0091 (0.0016) | 2.3E-08 | -0.0533 (0.0223) | 0.017 | 31.2 | -5.884 |
| Hand grip strength (right) | Alzheimer's disease, wide (FinnGen) | rs9388051 | 6:122577108 | A/G | 0.186 | 0.0106 (0.0019) | 2.7E-08 | -0.0103 (0.0317) | 0.746 | 30.9 | -0.969 |
| Hand grip strength (right) | Alzheimer's disease, wide (FinnGen) | rs1885690 | 6:109672998 | A/C | 0.410 | -0.0084 (0.0015) | 2.8E-08 | 0.0090 (0.0214) | 0.674 | 30.9 | -1.071 |
| Hand grip strength (right) | Alzheimer's disease, wide (FinnGen) | rs11998884 | 9:33684436 | T/C | 0.062 | 0.0173 (0.0031) | 2.8E-08 | -0.0086 (0.0392) | 0.826 | 30.8 | -0.496 |
| Hand grip strength (right) | Alzheimer's disease, wide (FinnGen) | rs12412806 | 10:24860913 | A/G | 0.294 | -0.0091 (0.0016) | 3.0E-08 | -0.0109 (0.0217) | 0.615 | 30.7 | 1.198 |
| Hand grip strength (right) | Alzheimer's disease, wide (FinnGen) | rs75457267 | 5:102658770 | T/C | 0.051 | -0.0187 (0.0034) | 3.4E-08 | 0.0925 (0.0504) | 0.067 | 30.4 | -4.939 |
| Hand grip strength (right) | Alzheimer's disease, wide (FinnGen) | rs13355365 | 5:154942606 | T/C | 0.380 | -0.0085 (0.0015) | 3.4E-08 | 0.0058 (0.0224) | 0.795 | 30.5 | -0.685 |
| Hand grip strength (right) | Alzheimer's disease, wide (FinnGen) | rs2389763 | 7:17307847 | C/T | 0.592 | -0.0083 (0.0015) | 3.8E-08 | 0.0386 (0.0219) | 0.078 | 30.3 | -4.636 |
| Hand grip strength (right) | Alzheimer's disease, wide (FinnGen) | rs4793658 | 17:45878733 | C/A | 0.110 | -0.0137 (0.0025) | 3.8E-08 | 0.0346 (0.0374) | 0.355 | 30.2 | -2.531 |
| Hand grip strength (right) | Alzheimer's disease, wide (FinnGen) | rs35304341 | 1:200971049 | A/G | 0.089 | -0.0143 (0.0026) | 4.0E-08 | 0.0266 (0.0361) | 0.461 | 30.1 | -1.862 |
| Hand grip strength (right) | Alzheimer's disease, wide (FinnGen) | rs7249 | 19:18391171 | T/C | 0.366 | 0.0085 (0.0015) | 4.0E-08 | -0.0049 (0.0224) | 0.828 | 30.2 | -0.578 |
| Hand grip strength (right) | Alzheimer's disease, wide (FinnGen) | rs4751671 | 10:116138744 | A/G | 0.531 | 0.0082 (0.0015) | 4.1E-08 | 0.0060 (0.0223) | 0.788 | 30.1 | 0.729 |
| Hand grip strength (right) | Alzheimer's disease, wide (FinnGen) | rs13169333 | 5:153064994 | C/T | 0.260 | 0.0093 (0.0017) | 4.3E-08 | 0.0272 (0.0236) | 0.250 | 30.0 | 2.928 |
| Hand grip strength (right) | Alzheimer's disease, wide (FinnGen) | rs645144 | 6:141180780 | C/T | 0.330 | -0.0087 (0.0016) | 4.3E-08 | -0.0338 (0.0236) | 0.152 | 30.0 | 3.890 |
| Hand grip strength (right) | Alzheimer's disease, wide (FinnGen) | rs113851275 | 9:98297220 | A/G | 0.108 | 0.0131 (0.0024) | 4.3E-08 | -0.0664 (0.0332) | 0.046 | 30.0 | -5.052 |
| Hand grip strength (right) | Alzheimer's disease, wide (FinnGen) | rs2244621 | 11:64026219 | T/C | 0.144 | 0.0116 (0.0021) | 4.5E-08 | -0.0098 (0.0290) | 0.735 | 29.9 | -0.845 |
| Hand grip strength (right) | Alzheimer's disease, wide (FinnGen) | rs2296316 | 14:65520246 | C/T | 0.464 | -0.0082 (0.0015) | 4.6E-08 | -0.0191 (0.0212) | 0.367 | 29.9 | 2.325 |
| Low hand grip strength (FNIH, 60+) | Alzheimer's disease (Bellenguez 2022) | rs3771501 | 2:70717653 | G/A | 0.526 | -0.0697 (0.0106) | 5.0E-11 | -0.0029 (0.0082) | 0.720 | 43.2 | 0.042 |
| Low hand grip strength (FNIH, 60+) | Alzheimer's disease (Bellenguez 2022) | rs552086 | 18:46806432 | C/G | 0.663 | -0.0626 (0.0111) | 1.8E-08 | 0.0055 (0.0086) | 0.524 | 31.8 | -0.088 |
| Low hand grip strength (FNIH, 60+) | Alzheimer's disease (Bellenguez 2022) | rs191252760 | 4:61454905 | G/A | 0.023 | 0.1863 (0.0337) | 3.2E-08 | 0.0307 (0.0280) | 0.274 | 30.6 | 0.165 |
| Low hand grip strength (FNIH, 60+) | All-cause dementia (FinnGen) | rs7760841 | 6:32574868 | T/C | 0.164 | 0.1229 (0.0151) | 3.3E-16 | -0.0336 (0.0271) | 0.215 | 66.2 | -0.273 |
| Low hand grip strength (FNIH, 60+) | All-cause dementia (FinnGen) | rs3771501 | 2:70717653 | G/A | 0.526 | -0.0697 (0.0106) | 5.0E-11 | -0.0105 (0.0193) | 0.585 | 43.2 | 0.151 |
| Low hand grip strength (FNIH, 60+) | All-cause dementia (FinnGen) | rs552086 | 18:46806432 | C/G | 0.663 | -0.0626 (0.0111) | 1.8E-08 | -0.0091 (0.0197) | 0.647 | 31.8 | 0.145 |
| Low hand grip strength (FNIH, 60+) | All-cause dementia (FinnGen) | rs191252760 | 4:61454905 | G/A | 0.023 | 0.1863 (0.0337) | 3.2E-08 | 0.0328 (0.0953) | 0.731 | 30.6 | 0.176 |
| Low hand grip strength (FNIH, 60+) | Vascular dementia (FinnGen) | rs7760841 | 6:32574868 | T/C | 0.164 | 0.1229 (0.0151) | 3.3E-16 | 0.0074 (0.0697) | 0.915 | 66.2 | 0.060 |
| Low hand grip strength (FNIH, 60+) | Vascular dementia (FinnGen) | rs3771501 | 2:70717653 | G/A | 0.526 | -0.0697 (0.0106) | 5.0E-11 | 0.0171 (0.0496) | 0.730 | 43.2 | -0.245 |
| Low hand grip strength (FNIH, 60+) | Vascular dementia (FinnGen) | rs552086 | 18:46806432 | C/G | 0.663 | -0.0626 (0.0111) | 1.8E-08 | -0.0730 (0.0510) | 0.152 | 31.8 | 1.166 |
| Low hand grip strength (FNIH, 60+) | Vascular dementia (FinnGen) | rs191252760 | 4:61454905 | G/A | 0.023 | 0.1863 (0.0337) | 3.2E-08 | -0.2299 (0.2501) | 0.358 | 30.6 | -1.234 |
| Low hand grip strength (FNIH, 60+) | Alzheimer's disease, wide (FinnGen) | rs7760841 | 6:32574868 | T/C | 0.164 | 0.1229 (0.0151) | 3.3E-16 | -0.0380 (0.0300) | 0.206 | 66.2 | -0.309 |
| Low hand grip strength (FNIH, 60+) | Alzheimer's disease, wide (FinnGen) | rs3771501 | 2:70717653 | G/A | 0.526 | -0.0697 (0.0106) | 5.0E-11 | -0.0311 (0.0214) | 0.146 | 43.2 | 0.446 |
| Low hand grip strength (FNIH, 60+) | Alzheimer's disease, wide (FinnGen) | rs552086 | 18:46806432 | C/G | 0.663 | -0.0626 (0.0111) | 1.8E-08 | -0.0082 (0.0219) | 0.709 | 31.8 | 0.131 |
| Low hand grip strength (FNIH, 60+) | Alzheimer's disease, wide (FinnGen) | rs191252760 | 4:61454905 | G/A | 0.023 | 0.1863 (0.0337) | 3.2E-08 | 0.1318 (0.1051) | 0.210 | 30.6 | 0.707 |
| Appendicular lean mass | Alzheimer's disease (Bellenguez 2022) | rs143384 | 20:34025756 | G/A | 0.404 | 0.0725 (0.0019) | 1.1E-308 | -0.0057 (0.0082) | 0.487 | 1456.0 | -0.079 |
| Appendicular lean mass | Alzheimer's disease (Bellenguez 2022) | rs6860245 | 5:127367998 | C/G | 0.248 | 0.0589 (0.0022) | 9.7E-160 | 0.0105 (0.0095) | 0.271 | 716.8 | 0.178 |
| Appendicular lean mass | Alzheimer's disease (Bellenguez 2022) | rs9853018 | 3:141101961 | T/C | 0.444 | 0.0469 (0.0019) | 2.4E-135 | -0.0138 (0.0083) | 0.095 | 609.3 | -0.294 |
| Appendicular lean mass | Alzheimer's disease (Bellenguez 2022) | rs1472852 | 4:17910236 | A/C | 0.158 | -0.0638 (0.0026) | 8.2E-135 | 0.0401 (0.0110) | 2.8E-04 | 602.1 | -0.629 |
| Appendicular lean mass | Alzheimer's disease (Bellenguez 2022) | rs9388490 | 6:126704795 | T/C | 0.439 | 0.0462 (0.0019) | 1.3E-130 | 0.0045 (0.0082) | 0.579 | 591.3 | 0.097 |
| Appendicular lean mass | Alzheimer's disease (Bellenguez 2022) | rs2854152 | 17:61986027 | G/A | 0.678 | 0.0482 (0.0020) | 1.0E-124 | -0.0164 (0.0088) | 0.061 | 580.8 | -0.340 |
| Appendicular lean mass | Alzheimer's disease (Bellenguez 2022) | rs71519447 | 8:57157543 | A/G | 0.110 | -0.0716 (0.0030) | 3.9E-124 | -0.0071 (0.0124) | 0.565 | 569.6 | 0.099 |
| Appendicular lean mass | Alzheimer's disease (Bellenguez 2022) | rs6977416 | 7:150542711 | A/G | 0.334 | 0.0457 (0.0020) | 1.4E-113 | 0.0045 (0.0086) | 0.600 | 522.1 | 0.098 |
| Appendicular lean mass | Alzheimer's disease (Bellenguez 2022) | rs76895963 | 12:4384844 | G/T | 0.021 | 0.1639 (0.0073) | 8.2E-112 | -0.0069 (0.0332) | 0.836 | 504.1 | -0.042 |
| Appendicular lean mass | Alzheimer's disease (Bellenguez 2022) | rs42039 | 7:92244422 | T/C | 0.244 | 0.0481 (0.0022) | 3.5E-106 | -0.0099 (0.0093) | 0.285 | 478.0 | -0.206 |
| Appendicular lean mass | Alzheimer's disease (Bellenguez 2022) | rs6567160 | 18:57829135 | C/T | 0.233 | 0.0488 (0.0022) | 1.2E-104 | -0.0127 (0.0096) | 0.187 | 492.0 | -0.260 |
| Appendicular lean mass | Alzheimer's disease (Bellenguez 2022) | rs6505216 | 17:29206421 | T/G | 0.233 | -0.0498 (0.0023) | 1.8E-101 | -0.0008 (0.0097) | 0.936 | 468.8 | 0.016 |
| Appendicular lean mass | Alzheimer's disease (Bellenguez 2022) | rs9634212 | 12:93993266 | A/C | 0.221 | 0.0471 (0.0023) | 8.6E-95 | 0.0072 (0.0099) | 0.466 | 419.4 | 0.153 |
| Appendicular lean mass | Alzheimer's disease (Bellenguez 2022) | rs11187838 | 10:96038686 | A/G | 0.435 | 0.0394 (0.0019) | 1.2E-94 | 0.0094 (0.0082) | 0.251 | 430.0 | 0.239 |
| Appendicular lean mass | Alzheimer's disease (Bellenguez 2022) | rs2524139 | 6:31264133 | C/G | 0.700 | -0.0423 (0.0020) | 1.5E-94 | 0.0017 (0.0095) | 0.859 | 447.3 | -0.040 |
| Appendicular lean mass | Alzheimer's disease (Bellenguez 2022) | rs28701981 | 9:98217581 | C/T | 0.349 | 0.0395 (0.0020) | 1.5E-87 | 0.0066 (0.0086) | 0.446 | 390.1 | 0.167 |
| Appendicular lean mass | Alzheimer's disease (Bellenguez 2022) | rs10483727 | 14:61072875 | C/T | 0.611 | -0.0368 (0.0019) | 6.7E-80 | 0.0120 (0.0083) | 0.147 | 375.1 | -0.326 |
| Appendicular lean mass | Alzheimer's disease (Bellenguez 2022) | rs1355603 | 4:145566477 | C/T | 0.831 | 0.0466 (0.0025) | 3.0E-76 | -0.0138 (0.0106) | 0.195 | 347.4 | -0.296 |
| Appendicular lean mass | Alzheimer's disease (Bellenguez 2022) | rs10807137 | 6:34183026 | T/C | 0.825 | -0.0455 (0.0025) | 5.7E-75 | -0.0029 (0.0107) | 0.787 | 331.2 | 0.064 |
| Appendicular lean mass | Alzheimer's disease (Bellenguez 2022) | rs55877758 | 2:172409030 | A/T | 0.241 | -0.0394 (0.0022) | 2.5E-72 | 0.0004 (0.0096) | 0.968 | 320.7 | -0.010 |
| Appendicular lean mass | Alzheimer's disease (Bellenguez 2022) | rs12713004 | 2:23896049 | G/A | 0.725 | 0.0367 (0.0021) | 2.4E-68 | 0.0034 (0.0091) | 0.707 | 305.4 | 0.093 |
| Appendicular lean mass | Alzheimer's disease (Bellenguez 2022) | rs798528 | 7:2772431 | C/A | 0.307 | -0.0357 (0.0020) | 5.6E-68 | 0.0230 (0.0088) | 0.009 | 318.6 | -0.644 |
| Appendicular lean mass | Alzheimer's disease (Bellenguez 2022) | rs113232639 | 18:20715656 | A/G | 0.493 | 0.0327 (0.0019) | 4.8E-64 | -0.0106 (0.0084) | 0.209 | 296.2 | -0.324 |
| Appendicular lean mass | Alzheimer's disease (Bellenguez 2022) | rs1260326 | 2:27730940 | C/T | 0.605 | 0.0323 (0.0019) | 6.2E-64 | -0.0183 (0.0082) | 0.026 | 289.0 | -0.567 |
| Appendicular lean mass | Alzheimer's disease (Bellenguez 2022) | rs113898003 | 6:130341235 | C/T | 0.264 | -0.0360 (0.0021) | 1.2E-63 | 0.0107 (0.0094) | 0.255 | 293.9 | -0.297 |
| Appendicular lean mass | Alzheimer's disease (Bellenguez 2022) | rs9905385 | 17:59498250 | G/A | 0.671 | -0.0339 (0.0020) | 1.9E-63 | 0.0035 (0.0087) | 0.682 | 287.3 | -0.103 |
| Appendicular lean mass | Alzheimer's disease (Bellenguez 2022) | rs3213141 | 20:32274380 | A/G | 0.266 | -0.0363 (0.0022) | 2.0E-63 | 0.0082 (0.0096) | 0.396 | 272.2 | -0.226 |
| Appendicular lean mass | Alzheimer's disease (Bellenguez 2022) | rs485554 | 3:172137343 | C/G | 0.315 | 0.0342 (0.0020) | 3.0E-63 | -0.0003 (0.0089) | 0.973 | 292.4 | -0.009 |
| Appendicular lean mass | Alzheimer's disease (Bellenguez 2022) | rs2871865 | 15:99194896 | G/C | 0.116 | -0.0493 (0.0030) | 3.4E-62 | 0.0288 (0.0125) | 0.022 | 270.1 | -0.584 |
| Appendicular lean mass | Alzheimer's disease (Bellenguez 2022) | rs2885697 | 1:41544279 | T/G | 0.665 | -0.0323 (0.0020) | 9.2E-60 | 0.0052 (0.0086) | 0.545 | 260.8 | -0.161 |
| Appendicular lean mass | Alzheimer's disease (Bellenguez 2022) | rs12099669 | 12:46783653 | A/G | 0.696 | 0.0331 (0.0020) | 1.4E-58 | -0.0063 (0.0088) | 0.470 | 273.9 | -0.190 |
| Appendicular lean mass | Alzheimer's disease (Bellenguez 2022) | rs11243202 | 6:7719065 | C/T | 0.486 | 0.0302 (0.0019) | 2.8E-57 | 0.0116 (0.0082) | 0.156 | 252.6 | 0.384 |
| Appendicular lean mass | Alzheimer's disease (Bellenguez 2022) | rs4735761 | 8:78097161 | C/A | 0.286 | 0.0331 (0.0021) | 3.7E-56 | -0.0132 (0.0091) | 0.145 | 248.4 | -0.399 |
| Appendicular lean mass | Alzheimer's disease (Bellenguez 2022) | rs78378222 | 17:7571752 | G/T | 0.012 | 0.1380 (0.0087) | 4.5E-56 | -0.0643 (0.0383) | 0.093 | 251.6 | -0.466 |
| Appendicular lean mass | Alzheimer's disease (Bellenguez 2022) | rs2194411 | 3:185548663 | A/G | 0.128 | 0.0443 (0.0029) | 2.4E-54 | 0.0156 (0.0120) | 0.194 | 233.3 | 0.352 |
| Appendicular lean mass | Alzheimer's disease (Bellenguez 2022) | rs994533 | 2:218284278 | C/G | 0.316 | -0.0313 (0.0020) | 5.5E-54 | -0.0129 (0.0087) | 0.138 | 244.9 | 0.412 |
| Appendicular lean mass | Alzheimer's disease (Bellenguez 2022) | rs11042717 | 11:10303939 | C/T | 0.490 | -0.0290 (0.0019) | 4.0E-53 | 0.0033 (0.0081) | 0.689 | 233.0 | -0.114 |
| Appendicular lean mass | Alzheimer's disease (Bellenguez 2022) | rs7129320 | 11:68388220 | A/G | 0.166 | -0.0389 (0.0025) | 7.3E-53 | -0.0109 (0.0111) | 0.327 | 242.1 | 0.280 |
| Appendicular lean mass | Alzheimer's disease (Bellenguez 2022) | rs146851424 | 13:50377910 | C/A | 0.022 | 0.1003 (0.0066) | 1.6E-52 | -0.0028 (0.0276) | 0.920 | 230.9 | -0.028 |
| Appendicular lean mass | Alzheimer's disease (Bellenguez 2022) | rs1325596 | 1:176794066 | A/G | 0.548 | 0.0287 (0.0019) | 2.8E-52 | -0.0090 (0.0082) | 0.274 | 228.2 | -0.314 |
| Appendicular lean mass | Alzheimer's disease (Bellenguez 2022) | rs72801818 | 16:53476042 | T/C | 0.300 | 0.0313 (0.0021) | 8.9E-52 | -0.0136 (0.0088) | 0.122 | 222.2 | -0.434 |
| Appendicular lean mass | Alzheimer's disease (Bellenguez 2022) | rs13103161 | 4:106216459 | A/T | 0.389 | -0.0284 (0.0019) | 2.4E-48 | 0.0051 (0.0084) | 0.540 | 223.4 | -0.180 |
| Appendicular lean mass | Alzheimer's disease (Bellenguez 2022) | rs34517439 | 1:78450517 | A/C | 0.122 | 0.0421 (0.0029) | 5.8E-48 | 0.0009 (0.0132) | 0.943 | 210.8 | 0.021 |
| Appendicular lean mass | Alzheimer's disease (Bellenguez 2022) | rs10491967 | 12:3368093 | A/G | 0.092 | 0.0471 (0.0032) | 1.3E-47 | -0.0025 (0.0136) | 0.853 | 216.6 | -0.053 |
| Appendicular lean mass | Alzheimer's disease (Bellenguez 2022) | rs723149 | 7:46577056 | G/A | 0.563 | -0.0276 (0.0019) | 1.4E-47 | -0.0070 (0.0083) | 0.398 | 211.0 | 0.254 |
| Appendicular lean mass | Alzheimer's disease (Bellenguez 2022) | rs4909912 | 8:135572297 | T/C | 0.427 | -0.0277 (0.0019) | 1.7E-47 | 0.0062 (0.0082) | 0.450 | 212.6 | -0.224 |
| Appendicular lean mass | Alzheimer's disease (Bellenguez 2022) | rs34776209 | 7:23513093 | T/C | 0.247 | -0.0317 (0.0022) | 1.8E-47 | 0.0222 (0.0094) | 0.019 | 207.6 | -0.700 |
| Appendicular lean mass | Alzheimer's disease (Bellenguez 2022) | rs41271299 | 6:19839415 | T/C | 0.051 | 0.0616 (0.0043) | 3.2E-47 | -0.0015 (0.0192) | 0.936 | 205.2 | -0.024 |
| Appendicular lean mass | Alzheimer's disease (Bellenguez 2022) | rs12325539 | 16:30033633 | C/T | 0.402 | 0.0275 (0.0019) | 6.9E-46 | -0.0567 (0.0084) | 1.2E-11 | 209.5 | -2.062 |
| Appendicular lean mass | Alzheimer's disease (Bellenguez 2022) | rs1319012 | 6:41852616 | A/T | 0.926 | -0.0520 (0.0037) | 3.3E-45 | 0.0134 (0.0159) | 0.401 | 197.5 | -0.258 |
| Appendicular lean mass | Alzheimer's disease (Bellenguez 2022) | rs7095472 | 10:70399109 | G/A | 0.533 | 0.0267 (0.0019) | 7.7E-45 | -0.0055 (0.0082) | 0.498 | 197.5 | -0.206 |
| Appendicular lean mass | Alzheimer's disease (Bellenguez 2022) | rs59985551 | 2:56106928 | T/C | 0.226 | -0.0313 (0.0022) | 2.4E-44 | -0.0112 (0.0096) | 0.241 | 202.4 | 0.358 |
| Appendicular lean mass | Alzheimer's disease (Bellenguez 2022) | rs10283100 | 8:120596023 | G/A | 0.945 | 0.0575 (0.0041) | 4.1E-44 | -0.0223 (0.0175) | 0.202 | 196.7 | -0.388 |
| Appendicular lean mass | Alzheimer's disease (Bellenguez 2022) | rs6931421 | 6:80880138 | G/T | 0.322 | -0.0279 (0.0020) | 2.3E-43 | 0.0168 (0.0086) | 0.051 | 194.6 | -0.602 |
| Appendicular lean mass | Alzheimer's disease (Bellenguez 2022) | rs17205463 | 15:62381413 | T/C | 0.448 | -0.0263 (0.0019) | 4.2E-43 | 0.0048 (0.0082) | 0.555 | 191.6 | -0.183 |
| Appendicular lean mass | Alzheimer's disease (Bellenguez 2022) | rs2270894 | 3:9975386 | G/C | 0.203 | -0.0332 (0.0024) | 1.3E-42 | -0.0147 (0.0105) | 0.163 | 191.4 | 0.443 |
| Appendicular lean mass | Alzheimer's disease (Bellenguez 2022) | rs4282339 | 5:168256240 | A/G | 0.207 | -0.0311 (0.0023) | 6.2E-41 | 0.0008 (0.0099) | 0.938 | 182.8 | -0.026 |
| Appendicular lean mass | Alzheimer's disease (Bellenguez 2022) | rs10883555 | 10:102657908 | T/C | 0.550 | 0.0254 (0.0019) | 9.1E-41 | -0.0211 (0.0082) | 0.010 | 178.7 | -0.831 |
| Appendicular lean mass | Alzheimer's disease (Bellenguez 2022) | rs12051245 | 16:783865 | C/T | 0.232 | 0.0299 (0.0022) | 2.6E-40 | -0.0150 (0.0099) | 0.130 | 184.7 | -0.502 |
| Appendicular lean mass | Alzheimer's disease (Bellenguez 2022) | rs11014285 | 10:25178864 | A/G | 0.165 | 0.0342 (0.0026) | 2.9E-40 | -0.0126 (0.0113) | 0.263 | 173.0 | -0.368 |
| Appendicular lean mass | Alzheimer's disease (Bellenguez 2022) | rs2229840 | 12:124826462 | T/C | 0.160 | 0.0341 (0.0026) | 3.0E-40 | -0.0200 (0.0110) | 0.069 | 172.0 | -0.587 |
| Appendicular lean mass | Alzheimer's disease (Bellenguez 2022) | rs35309034 | 8:126498265 | A/C | 0.238 | -0.0297 (0.0022) | 1.1E-39 | 0.0334 (0.0098) | 6.5E-04 | 182.2 | -1.125 |
| Appendicular lean mass | Alzheimer's disease (Bellenguez 2022) | rs7952436 | 11:67024534 | T/C | 0.082 | -0.0453 (0.0034) | 1.6E-39 | 0.0271 (0.0157) | 0.084 | 177.5 | -0.598 |
| Appendicular lean mass | Alzheimer's disease (Bellenguez 2022) | rs13041213 | 20:47800829 | A/T | 0.236 | 0.0295 (0.0022) | 3.2E-39 | -0.0021 (0.0098) | 0.833 | 179.8 | -0.071 |
| Appendicular lean mass | Alzheimer's disease (Bellenguez 2022) | rs8054549 | 16:86417234 | A/C | 0.449 | -0.0251 (0.0019) | 3.4E-39 | 0.0008 (0.0082) | 0.926 | 174.5 | -0.032 |
| Appendicular lean mass | Alzheimer's disease (Bellenguez 2022) | rs3764002 | 12:108618630 | T/C | 0.262 | 0.0280 (0.0021) | 4.5E-39 | -0.0037 (0.0094) | 0.695 | 177.8 | -0.132 |
| Appendicular lean mass | Alzheimer's disease (Bellenguez 2022) | rs12714414 | 2:651407 | C/T | 0.152 | -0.0353 (0.0027) | 8.2E-39 | 0.0178 (0.0110) | 0.105 | 170.9 | -0.504 |
| Appendicular lean mass | Alzheimer's disease (Bellenguez 2022) | rs5742915 | 15:74336633 | C/T | 0.461 | 0.0248 (0.0019) | 9.3E-39 | 0.0180 (0.0081) | 0.027 | 170.4 | 0.726 |
| Appendicular lean mass | Alzheimer's disease (Bellenguez 2022) | rs33973388 | 18:46611842 | T/G | 0.435 | 0.0249 (0.0019) | 1.4E-38 | 0.0063 (0.0082) | 0.440 | 171.8 | 0.253 |
| Appendicular lean mass | Alzheimer's disease (Bellenguez 2022) | rs8019890 | 14:21538067 | A/C | 0.531 | 0.0250 (0.0019) | 2.0E-38 | -0.0024 (0.0085) | 0.778 | 173.1 | -0.096 |
| Appendicular lean mass | Alzheimer's disease (Bellenguez 2022) | rs8042545 | 15:66992821 | A/G | 0.242 | 0.0287 (0.0022) | 2.9E-38 | 0.0059 (0.0094) | 0.530 | 170.2 | 0.206 |
| Appendicular lean mass | Alzheimer's disease (Bellenguez 2022) | rs2900208 | 12:11878464 | A/C | 0.354 | 0.0255 (0.0020) | 3.8E-38 | 0.0020 (0.0086) | 0.814 | 162.6 | 0.078 |
| Appendicular lean mass | Alzheimer's disease (Bellenguez 2022) | rs10748128 | 12:69827658 | T/G | 0.345 | 0.0255 (0.0020) | 6.8E-38 | 0.0077 (0.0085) | 0.368 | 162.6 | 0.302 |
| Appendicular lean mass | Alzheimer's disease (Bellenguez 2022) | rs28592876 | 12:123866429 | A/G | 0.205 | 0.0300 (0.0023) | 9.1E-38 | -0.0123 (0.0102) | 0.227 | 170.1 | -0.410 |
| Appendicular lean mass | Alzheimer's disease (Bellenguez 2022) | rs4615815 | 1:214604236 | G/A | 0.397 | -0.0246 (0.0019) | 1.1E-37 | -0.0085 (0.0083) | 0.309 | 167.6 | 0.346 |
| Appendicular lean mass | Alzheimer's disease (Bellenguez 2022) | rs244711 | 5:176509193 | T/C | 0.686 | 0.0279 (0.0022) | 1.5E-37 | -0.0318 (0.0091) | 4.9E-04 | 160.8 | -1.140 |
| Appendicular lean mass | Alzheimer's disease (Bellenguez 2022) | rs17036160 | 3:12329783 | T/C | 0.118 | -0.0375 (0.0029) | 1.7E-37 | -0.0360 (0.0128) | 0.005 | 167.2 | 0.960 |
| Appendicular lean mass | Alzheimer's disease (Bellenguez 2022) | rs36226649 | 14:24835500 | C/T | 0.067 | 0.0485 (0.0038) | 3.1E-37 | -0.0010 (0.0167) | 0.953 | 162.9 | -0.021 |
| Appendicular lean mass | Alzheimer's disease (Bellenguez 2022) | rs61944841 | 13:27049616 | A/G | 0.414 | 0.0253 (0.0020) | 3.5E-37 | 0.0225 (0.0089) | 0.012 | 160.0 | 0.889 |
| Appendicular lean mass | Alzheimer's disease (Bellenguez 2022) | rs3853252 | 6:152170247 | A/G | 0.455 | 0.0241 (0.0019) | 8.8E-37 | 0.0016 (0.0082) | 0.845 | 160.9 | 0.066 |
| Appendicular lean mass | Alzheimer's disease (Bellenguez 2022) | rs7301341 | 12:94083105 | C/T | 0.327 | -0.0255 (0.0020) | 9.3E-37 | 0.0079 (0.0089) | 0.374 | 162.6 | -0.310 |
| Appendicular lean mass | Alzheimer's disease (Bellenguez 2022) | rs12622189 | 2:218132858 | G/A | 0.258 | -0.0272 (0.0021) | 9.3E-37 | -0.0038 (0.0091) | 0.674 | 167.8 | 0.140 |
| Appendicular lean mass | Alzheimer's disease (Bellenguez 2022) | rs2035901 | 4:145521867 | G/A | 0.468 | 0.0240 (0.0019) | 9.4E-37 | -0.0047 (0.0081) | 0.561 | 159.6 | -0.196 |
| Appendicular lean mass | Alzheimer's disease (Bellenguez 2022) | rs778384 | 1:56637166 | A/G | 0.223 | 0.0283 (0.0022) | 2.8E-36 | -0.0044 (0.0096) | 0.644 | 165.5 | -0.155 |
| Appendicular lean mass | Alzheimer's disease (Bellenguez 2022) | rs3843750 | 19:10748073 | G/C | 0.664 | -0.0252 (0.0020) | 5.1E-36 | 0.0081 (0.0092) | 0.382 | 158.8 | -0.321 |
| Appendicular lean mass | Alzheimer's disease (Bellenguez 2022) | rs4752829 | 11:47396654 | A/G | 0.286 | 0.0262 (0.0021) | 5.9E-36 | -0.0272 (0.0091) | 0.003 | 155.7 | -1.038 |
| Appendicular lean mass | Alzheimer's disease (Bellenguez 2022) | rs59950280 | 4:3452345 | A/G | 0.332 | -0.0254 (0.0020) | 7.3E-36 | 0.0032 (0.0088) | 0.714 | 161.3 | -0.126 |
| Appendicular lean mass | Alzheimer's disease (Bellenguez 2022) | rs11689546 | 2:25130451 | G/A | 0.440 | -0.0238 (0.0019) | 8.1E-36 | -0.0136 (0.0084) | 0.104 | 156.9 | 0.571 |
| Appendicular lean mass | Alzheimer's disease (Bellenguez 2022) | rs2098695 | 5:54868944 | G/A | 0.691 | -0.0255 (0.0020) | 1.1E-35 | -0.0068 (0.0088) | 0.435 | 162.6 | 0.267 |
| Appendicular lean mass | Alzheimer's disease (Bellenguez 2022) | rs7582516 | 2:227255370 | T/C | 0.304 | 0.0254 (0.0020) | 1.3E-35 | -0.0146 (0.0088) | 0.096 | 161.3 | -0.575 |
| Appendicular lean mass | Alzheimer's disease (Bellenguez 2022) | rs3818416 | 13:78474468 | C/A | 0.764 | 0.0279 (0.0022) | 2.0E-35 | -0.0023 (0.0095) | 0.811 | 160.8 | -0.082 |
| Appendicular lean mass | Alzheimer's disease (Bellenguez 2022) | rs2549677 | 16:2162361 | G/A | 0.099 | -0.0392 (0.0032) | 1.3E-34 | 0.0031 (0.0140) | 0.823 | 150.1 | -0.079 |
| Appendicular lean mass | Alzheimer's disease (Bellenguez 2022) | rs1177765 | 5:32829929 | C/T | 0.468 | -0.0232 (0.0019) | 1.3E-34 | 0.0052 (0.0081) | 0.524 | 149.1 | -0.224 |
| Appendicular lean mass | Alzheimer's disease (Bellenguez 2022) | rs1324538 | 6:45080144 | A/T | 0.384 | 0.0237 (0.0019) | 1.7E-34 | -0.0011 (0.0083) | 0.893 | 155.6 | -0.046 |
| Appendicular lean mass | Alzheimer's disease (Bellenguez 2022) | rs61838776 | 1:219618564 | A/C | 0.162 | 0.0311 (0.0025) | 2.4E-34 | 0.0224 (0.0113) | 0.047 | 154.8 | 0.720 |
| Appendicular lean mass | Alzheimer's disease (Bellenguez 2022) | rs115912456 | 5:82815158 | G/A | 0.041 | 0.0577 (0.0047) | 3.7E-34 | 0.0009 (0.0199) | 0.966 | 150.7 | 0.016 |
| Appendicular lean mass | Alzheimer's disease (Bellenguez 2022) | rs4852257 | 2:71678520 | G/T | 0.576 | -0.0231 (0.0019) | 6.2E-34 | -0.0321 (0.0083) | 1.0E-04 | 147.8 | 1.390 |
| Appendicular lean mass | Alzheimer's disease (Bellenguez 2022) | rs12761076 | 10:72423050 | A/G | 0.266 | -0.0260 (0.0021) | 1.0E-33 | -0.0083 (0.0093) | 0.370 | 153.3 | 0.319 |
| Appendicular lean mass | Alzheimer's disease (Bellenguez 2022) | rs111365325 | 5:170865229 | T/C | 0.231 | -0.0271 (0.0022) | 1.1E-33 | 0.0268 (0.0097) | 0.006 | 151.7 | -0.989 |
| Appendicular lean mass | Alzheimer's disease (Bellenguez 2022) | rs45474992 | 19:47724564 | T/C | 0.036 | -0.0617 (0.0051) | 2.2E-33 | 0.0412 (0.0253) | 0.103 | 146.4 | -0.668 |
| Appendicular lean mass | Alzheimer's disease (Bellenguez 2022) | rs7428883 | 3:128983853 | G/A | 0.781 | 0.0277 (0.0023) | 2.3E-33 | -0.0151 (0.0101) | 0.133 | 145.1 | -0.545 |
| Appendicular lean mass | Alzheimer's disease (Bellenguez 2022) | rs76364830 | 8:13372120 | A/G | 0.063 | -0.0471 (0.0039) | 2.7E-33 | 0.0418 (0.0173) | 0.016 | 145.8 | -0.887 |
| Appendicular lean mass | Alzheimer's disease (Bellenguez 2022) | rs10202701 | 2:232328681 | T/C | 0.542 | 0.0227 (0.0019) | 3.1E-33 | -0.0126 (0.0082) | 0.124 | 142.7 | -0.555 |
| Appendicular lean mass | Alzheimer's disease (Bellenguez 2022) | rs4976262 | 5:134379531 | C/T | 0.316 | -0.0245 (0.0020) | 4.4E-33 | 0.0037 (0.0088) | 0.675 | 150.1 | -0.151 |
| Appendicular lean mass | Alzheimer's disease (Bellenguez 2022) | rs6082354 | 20:21217976 | C/A | 0.667 | -0.0240 (0.0020) | 1.2E-32 | 0.0047 (0.0088) | 0.597 | 144.0 | -0.196 |
| Appendicular lean mass | Alzheimer's disease (Bellenguez 2022) | rs2209098 | 1:172167226 | C/T | 0.311 | 0.0240 (0.0020) | 1.7E-32 | 0.0042 (0.0087) | 0.631 | 144.0 | 0.175 |
| Appendicular lean mass | Alzheimer's disease (Bellenguez 2022) | rs1444628 | 12:20563643 | T/C | 0.690 | 0.0240 (0.0020) | 6.8E-32 | -0.0142 (0.0088) | 0.107 | 144.0 | -0.592 |
| Appendicular lean mass | Alzheimer's disease (Bellenguez 2022) | rs147110934 | 19:55993436 | T/G | 0.024 | -0.0722 (0.0062) | 9.4E-32 | -0.0363 (0.0290) | 0.211 | 135.6 | 0.503 |
| Appendicular lean mass | Alzheimer's disease (Bellenguez 2022) | rs148138388 | 1:51371608 | C/G | 0.083 | 0.0406 (0.0035) | 1.1E-31 | -0.0461 (0.0147) | 0.002 | 134.6 | -1.135 |
| Appendicular lean mass | Alzheimer's disease (Bellenguez 2022) | rs58738817 | 3:184012228 | A/G | 0.248 | -0.0257 (0.0022) | 1.2E-31 | 0.0094 (0.0096) | 0.329 | 136.5 | -0.366 |
| Appendicular lean mass | Alzheimer's disease (Bellenguez 2022) | rs6570509 | 6:142716286 | T/G | 0.287 | -0.0244 (0.0021) | 1.3E-31 | 0.0065 (0.0088) | 0.462 | 135.0 | -0.266 |
| Appendicular lean mass | Alzheimer's disease (Bellenguez 2022) | rs4252548 | 19:55879672 | T/C | 0.022 | -0.0753 (0.0065) | 3.0E-31 | -0.0121 (0.0287) | 0.673 | 134.2 | 0.161 |
| Appendicular lean mass | Alzheimer's disease (Bellenguez 2022) | rs35464459 | 10:4964640 | C/T | 0.116 | -0.0342 (0.0029) | 5.0E-31 | -0.0066 (0.0125) | 0.595 | 139.1 | 0.193 |
| Appendicular lean mass | Alzheimer's disease (Bellenguez 2022) | rs1047891 | 2:211540507 | A/C | 0.316 | 0.0233 (0.0020) | 5.7E-31 | -0.0040 (0.0088) | 0.648 | 135.7 | -0.172 |
| Appendicular lean mass | Alzheimer's disease (Bellenguez 2022) | rs35756741 | 12:12868701 | T/C | 0.092 | -0.0378 (0.0033) | 5.8E-31 | -0.0122 (0.0142) | 0.390 | 131.2 | 0.323 |
| Appendicular lean mass | Alzheimer's disease (Bellenguez 2022) | rs55872725 | 16:53809123 | T/C | 0.404 | 0.0222 (0.0019) | 1.5E-30 | -0.0153 (0.0082) | 0.063 | 136.5 | -0.689 |
| Appendicular lean mass | Alzheimer's disease (Bellenguez 2022) | rs6501381 | 17:68148350 | C/T | 0.113 | -0.0341 (0.0030) | 5.2E-30 | 0.0024 (0.0129) | 0.853 | 129.2 | -0.070 |
| Appendicular lean mass | Alzheimer's disease (Bellenguez 2022) | rs12509014 | 4:123825919 | T/C | 0.790 | -0.0263 (0.0023) | 5.4E-30 | 0.0034 (0.0098) | 0.731 | 130.8 | -0.129 |
| Appendicular lean mass | Alzheimer's disease (Bellenguez 2022) | rs8031704 | 15:84651185 | A/C | 0.327 | -0.0228 (0.0020) | 2.1E-29 | -0.0125 (0.0086) | 0.147 | 130.0 | 0.548 |
| Appendicular lean mass | Alzheimer's disease (Bellenguez 2022) | rs377599 | 1:2164699 | T/C | 0.383 | 0.0217 (0.0019) | 3.5E-29 | -0.0059 (0.0084) | 0.484 | 130.4 | -0.272 |
| Appendicular lean mass | Alzheimer's disease (Bellenguez 2022) | rs212526 | 1:21584941 | C/T | 0.601 | 0.0214 (0.0019) | 3.8E-29 | -0.0191 (0.0083) | 0.021 | 126.9 | -0.893 |
| Appendicular lean mass | Alzheimer's disease (Bellenguez 2022) | rs9647379 | 3:171785168 | C/G | 0.411 | 0.0215 (0.0019) | 5.5E-29 | 0.0032 (0.0085) | 0.704 | 128.1 | 0.149 |
| Appendicular lean mass | Alzheimer's disease (Bellenguez 2022) | rs4244809 | 11:2164333 | A/G | 0.208 | -0.0262 (0.0023) | 5.6E-29 | -0.0095 (0.0099) | 0.339 | 129.8 | 0.363 |
| Appendicular lean mass | Alzheimer's disease (Bellenguez 2022) | rs4076427 | 17:79087537 | C/G | 0.391 | -0.0218 (0.0019) | 6.0E-29 | 0.0001 (0.0084) | 0.993 | 131.7 | -0.005 |
| Appendicular lean mass | Alzheimer's disease (Bellenguez 2022) | rs7229520 | 18:46516468 | A/G | 0.662 | -0.0224 (0.0020) | 9.2E-29 | 0.0068 (0.0086) | 0.428 | 125.4 | -0.304 |
| Appendicular lean mass | Alzheimer's disease (Bellenguez 2022) | rs6762851 | 3:56686329 | C/T | 0.357 | -0.0218 (0.0020) | 1.5E-28 | 0.0169 (0.0084) | 0.045 | 118.8 | -0.775 |
| Appendicular lean mass | Alzheimer's disease (Bellenguez 2022) | rs1211575 | 1:11118390 | A/G | 0.743 | -0.0237 (0.0021) | 1.9E-28 | -0.0032 (0.0094) | 0.732 | 127.4 | 0.135 |
| Appendicular lean mass | Alzheimer's disease (Bellenguez 2022) | rs4274112 | 1:26746199 | G/A | 0.373 | -0.0217 (0.0020) | 2.5E-28 | -0.0024 (0.0085) | 0.782 | 117.7 | 0.111 |
| Appendicular lean mass | Alzheimer's disease (Bellenguez 2022) | rs2071518 | 8:120435812 | T/C | 0.264 | -0.0236 (0.0021) | 3.4E-28 | 0.0037 (0.0091) | 0.686 | 126.3 | -0.157 |
| Appendicular lean mass | Alzheimer's disease (Bellenguez 2022) | rs13302480 | 9:118465313 | C/G | 0.114 | -0.0328 (0.0030) | 4.4E-28 | -0.0079 (0.0125) | 0.529 | 119.5 | 0.241 |
| Appendicular lean mass | Alzheimer's disease (Bellenguez 2022) | rs36048468 | 5:122879901 | T/C | 0.209 | 0.0254 (0.0023) | 9.3E-28 | 0.0174 (0.0099) | 0.077 | 122.0 | 0.685 |
| Appendicular lean mass | Alzheimer's disease (Bellenguez 2022) | rs62370472 | 5:52767109 | C/T | 0.209 | -0.0253 (0.0023) | 1.4E-27 | -0.0014 (0.0100) | 0.886 | 121.0 | 0.055 |
| Appendicular lean mass | Alzheimer's disease (Bellenguez 2022) | rs77165542 | 2:430975 | T/C | 0.035 | -0.0557 (0.0051) | 1.8E-27 | 0.0232 (0.0223) | 0.298 | 119.3 | -0.417 |
| Appendicular lean mass | Alzheimer's disease (Bellenguez 2022) | rs62621812 | 7:127015083 | A/G | 0.020 | 0.0743 (0.0069) | 3.2E-27 | 0.0537 (0.0278) | 0.053 | 116.0 | 0.723 |
| Appendicular lean mass | Alzheimer's disease (Bellenguez 2022) | rs181766 | 16:14394878 | C/T | 0.322 | 0.0221 (0.0020) | 4.3E-27 | 0.0017 (0.0087) | 0.846 | 122.1 | 0.077 |
| Appendicular lean mass | Alzheimer's disease (Bellenguez 2022) | rs62048221 | 16:88846552 | T/C | 0.231 | -0.0242 (0.0022) | 4.9E-27 | -0.0008 (0.0096) | 0.933 | 121.0 | 0.033 |
| Appendicular lean mass | Alzheimer's disease (Bellenguez 2022) | rs7781964 | 7:139749346 | A/G | 0.185 | 0.0261 (0.0024) | 5.7E-27 | 0.0265 (0.0101) | 0.009 | 118.3 | 1.015 |
| Appendicular lean mass | Alzheimer's disease (Bellenguez 2022) | rs2070598 | 14:75360906 | A/G | 0.456 | 0.0204 (0.0019) | 6.4E-27 | 0.0014 (0.0081) | 0.863 | 115.3 | 0.069 |
| Appendicular lean mass | Alzheimer's disease (Bellenguez 2022) | rs13209685 | 6:7779729 | T/G | 0.159 | 0.0277 (0.0026) | 7.5E-27 | -0.0006 (0.0113) | 0.957 | 113.5 | -0.022 |
| Appendicular lean mass | Alzheimer's disease (Bellenguez 2022) | rs951366 | 1:205685352 | C/T | 0.393 | 0.0205 (0.0019) | 9.2E-27 | 0.0086 (0.0084) | 0.306 | 116.4 | 0.420 |
| Appendicular lean mass | Alzheimer's disease (Bellenguez 2022) | rs291970 | 10:121123633 | T/C | 0.229 | 0.0240 (0.0022) | 1.3E-26 | 0.0038 (0.0096) | 0.693 | 119.0 | 0.158 |
| Appendicular lean mass | Alzheimer's disease (Bellenguez 2022) | rs4752689 | 10:124131176 | A/G | 0.584 | 0.0205 (0.0019) | 1.4E-26 | -0.0258 (0.0082) | 0.002 | 116.4 | -1.259 |
| Appendicular lean mass | Alzheimer's disease (Bellenguez 2022) | rs7768382 | 6:166341870 | C/T | 0.477 | -0.0201 (0.0019) | 1.6E-26 | 0.0083 (0.0082) | 0.311 | 111.9 | -0.413 |
| Appendicular lean mass | Alzheimer's disease (Bellenguez 2022) | rs149094387 | 2:68356315 | G/C | 0.081 | -0.0366 (0.0034) | 2.2E-26 | -0.0303 (0.0148) | 0.041 | 115.9 | 0.828 |
| Appendicular lean mass | Alzheimer's disease (Bellenguez 2022) | rs72841270 | 10:104642237 | G/T | 0.135 | 0.0294 (0.0028) | 2.3E-26 | -0.0022 (0.0116) | 0.849 | 110.2 | -0.075 |
| Appendicular lean mass | Alzheimer's disease (Bellenguez 2022) | rs6821305 | 4:122713863 | C/A | 0.399 | 0.0204 (0.0019) | 3.1E-26 | -0.0030 (0.0083) | 0.717 | 115.3 | -0.147 |
| Appendicular lean mass | Alzheimer's disease (Bellenguez 2022) | rs7014590 | 8:89335647 | C/T | 0.261 | -0.0228 (0.0022) | 4.5E-26 | 0.0007 (0.0092) | 0.939 | 107.4 | -0.031 |
| Appendicular lean mass | Alzheimer's disease (Bellenguez 2022) | rs9659061 | 1:150527292 | A/G | 0.476 | -0.0198 (0.0019) | 6.8E-26 | 0.0064 (0.0082) | 0.433 | 108.6 | -0.323 |
| Appendicular lean mass | Alzheimer's disease (Bellenguez 2022) | rs7497684 | 15:89342720 | A/C | 0.480 | 0.0200 (0.0019) | 8.3E-26 | -0.0063 (0.0084) | 0.455 | 110.8 | -0.315 |
| Appendicular lean mass | Alzheimer's disease (Bellenguez 2022) | rs1662842 | 4:82155362 | G/A | 0.307 | 0.0214 (0.0020) | 1.7E-25 | 0.0015 (0.0088) | 0.864 | 114.5 | 0.070 |
| Appendicular lean mass | Alzheimer's disease (Bellenguez 2022) | rs7072873 | 10:77210191 | T/C | 0.539 | -0.0198 (0.0019) | 3.2E-25 | 0.0100 (0.0082) | 0.225 | 108.6 | -0.505 |
| Appendicular lean mass | Alzheimer's disease (Bellenguez 2022) | rs4640244 | 17:21284223 | G/A | 0.399 | -0.0200 (0.0019) | 3.8E-25 | 0.0038 (0.0083) | 0.648 | 110.8 | -0.190 |
| Appendicular lean mass | Alzheimer's disease (Bellenguez 2022) | rs7858712 | 9:16738312 | G/A | 0.914 | 0.0347 (0.0034) | 1.0E-24 | -0.0091 (0.0144) | 0.526 | 104.2 | -0.262 |
| Appendicular lean mass | Alzheimer's disease (Bellenguez 2022) | rs927376 | 20:38569825 | G/C | 0.260 | -0.0223 (0.0022) | 1.2E-24 | 0.0011 (0.0094) | 0.905 | 102.8 | -0.049 |
| Appendicular lean mass | Alzheimer's disease (Bellenguez 2022) | rs78457529 | 16:24950880 | T/C | 0.012 | -0.0904 (0.0088) | 1.2E-24 | -0.0146 (0.0369) | 0.693 | 105.5 | 0.162 |
| Appendicular lean mass | Alzheimer's disease (Bellenguez 2022) | rs2748501 | 6:146312258 | A/G | 0.440 | -0.0195 (0.0019) | 1.3E-24 | 0.0062 (0.0082) | 0.449 | 105.3 | -0.318 |
| Appendicular lean mass | Alzheimer's disease (Bellenguez 2022) | rs12882130 | 14:103878774 | G/C | 0.384 | -0.0202 (0.0020) | 1.9E-24 | -0.0075 (0.0086) | 0.383 | 102.0 | 0.371 |
| Appendicular lean mass | Alzheimer's disease (Bellenguez 2022) | rs45528934 | 14:23793305 | T/C | 0.162 | 0.0262 (0.0026) | 2.0E-24 | 0.0020 (0.0111) | 0.856 | 101.5 | 0.076 |
| Appendicular lean mass | Alzheimer's disease (Bellenguez 2022) | rs41311445 | 22:42070374 | C/A | 0.096 | -0.0328 (0.0032) | 4.7E-24 | 0.0081 (0.0137) | 0.552 | 105.1 | -0.247 |
| Appendicular lean mass | Alzheimer's disease (Bellenguez 2022) | rs60804050 | 1:118870373 | A/G | 0.256 | -0.0217 (0.0021) | 5.0E-24 | 0.0152 (0.0094) | 0.107 | 106.8 | -0.700 |
| Appendicular lean mass | Alzheimer's disease (Bellenguez 2022) | rs7816345 | 8:36846109 | T/C | 0.168 | 0.0255 (0.0025) | 6.2E-24 | 0.0068 (0.0106) | 0.518 | 104.0 | 0.267 |
| Appendicular lean mass | Alzheimer's disease (Bellenguez 2022) | rs2627702 | 4:88577419 | C/T | 0.519 | -0.0191 (0.0019) | 6.4E-24 | 0.0164 (0.0081) | 0.044 | 101.1 | -0.859 |
| Appendicular lean mass | Alzheimer's disease (Bellenguez 2022) | rs2296316 | 14:65520246 | C/T | 0.464 | -0.0192 (0.0019) | 1.6E-23 | 0.0045 (0.0082) | 0.584 | 102.1 | -0.234 |
| Appendicular lean mass | Alzheimer's disease (Bellenguez 2022) | rs2019203 | 17:36908672 | A/C | 0.491 | 0.0189 (0.0019) | 1.8E-23 | 0.0009 (0.0081) | 0.914 | 99.0 | 0.048 |
| Appendicular lean mass | Alzheimer's disease (Bellenguez 2022) | rs17035465 | 2:46890294 | G/A | 0.221 | 0.0226 (0.0023) | 2.1E-23 | 0.0092 (0.0098) | 0.347 | 96.5 | 0.407 |
| Appendicular lean mass | Alzheimer's disease (Bellenguez 2022) | rs8077636 | 17:79421254 | T/G | 0.418 | -0.0192 (0.0019) | 2.5E-23 | 0.0239 (0.0087) | 0.006 | 102.1 | -1.245 |
| Appendicular lean mass | Alzheimer's disease (Bellenguez 2022) | rs6919321 | 6:33724004 | A/G | 0.608 | 0.0192 (0.0019) | 3.6E-23 | -0.0010 (0.0083) | 0.903 | 102.1 | -0.052 |
| Appendicular lean mass | Alzheimer's disease (Bellenguez 2022) | rs2140046 | 2:169706079 | C/T | 0.363 | -0.0192 (0.0019) | 5.5E-23 | 0.0064 (0.0085) | 0.451 | 102.1 | -0.333 |
| Appendicular lean mass | Alzheimer's disease (Bellenguez 2022) | rs10917335 | 1:23294136 | G/A | 0.330 | -0.0198 (0.0020) | 5.7E-23 | 0.0044 (0.0089) | 0.619 | 98.0 | -0.222 |
| Appendicular lean mass | Alzheimer's disease (Bellenguez 2022) | rs1786263 | 18:13116432 | T/G | 0.606 | -0.0190 (0.0019) | 1.0E-22 | 0.0049 (0.0083) | 0.556 | 100.0 | -0.258 |
| Appendicular lean mass | Alzheimer's disease (Bellenguez 2022) | rs9807032 | 17:62757146 | C/T | 0.818 | -0.0240 (0.0024) | 1.3E-22 | 0.0193 (0.0110) | 0.080 | 100.0 | -0.804 |
| Appendicular lean mass | Alzheimer's disease (Bellenguez 2022) | rs140440099 | 3:50632595 | A/G | 0.023 | 0.0613 (0.0063) | 1.4E-22 | 0.0409 (0.0273) | 0.133 | 94.7 | 0.667 |
| Appendicular lean mass | Alzheimer's disease (Bellenguez 2022) | rs113852999 | 7:148649606 | A/G | 0.169 | 0.0246 (0.0025) | 1.8E-22 | 0.0009 (0.0110) | 0.934 | 96.8 | 0.037 |
| Appendicular lean mass | Alzheimer's disease (Bellenguez 2022) | rs35732917 | 7:73013269 | C/T | 0.284 | 0.0204 (0.0021) | 2.1E-22 | 0.0022 (0.0091) | 0.809 | 94.4 | 0.108 |
| Appendicular lean mass | Alzheimer's disease (Bellenguez 2022) | rs9344126 | 6:81907559 | C/T | 0.514 | -0.0185 (0.0019) | 2.2E-22 | 0.0048 (0.0082) | 0.555 | 94.8 | -0.259 |
| Appendicular lean mass | Alzheimer's disease (Bellenguez 2022) | rs3184504 | 12:111884608 | C/T | 0.517 | 0.0183 (0.0019) | 2.7E-22 | 0.0158 (0.0081) | 0.052 | 92.8 | 0.863 |
| Appendicular lean mass | Alzheimer's disease (Bellenguez 2022) | rs332116 | 10:28926099 | T/C | 0.280 | -0.0206 (0.0021) | 2.9E-22 | 0.0089 (0.0092) | 0.338 | 96.2 | -0.432 |
| Appendicular lean mass | Alzheimer's disease (Bellenguez 2022) | rs10202845 | 2:42575820 | G/A | 0.113 | -0.0288 (0.0030) | 5.3E-22 | 0.0165 (0.0128) | 0.197 | 92.2 | -0.573 |
| Appendicular lean mass | Alzheimer's disease (Bellenguez 2022) | rs5763821 | 22:30549071 | C/A | 0.391 | 0.0191 (0.0020) | 1.1E-21 | 0.0177 (0.0084) | 0.035 | 91.2 | 0.927 |
| Appendicular lean mass | Alzheimer's disease (Bellenguez 2022) | rs2305141 | 2:233684402 | G/A | 0.596 | 0.0183 (0.0019) | 1.1E-21 | -0.0190 (0.0084) | 0.024 | 92.8 | -1.038 |
| Appendicular lean mass | Alzheimer's disease (Bellenguez 2022) | rs1405212 | 6:117490664 | C/T | 0.628 | 0.0187 (0.0019) | 1.1E-21 | 0.0103 (0.0085) | 0.224 | 96.9 | 0.551 |
| Appendicular lean mass | Alzheimer's disease (Bellenguez 2022) | rs9838614 | 3:38537671 | G/T | 0.388 | -0.0185 (0.0019) | 1.2E-21 | 0.0077 (0.0083) | 0.356 | 94.8 | -0.416 |
| Appendicular lean mass | Alzheimer's disease (Bellenguez 2022) | rs12679359 | 8:130724556 | T/G | 0.137 | -0.0263 (0.0028) | 1.4E-21 | 0.0092 (0.0118) | 0.437 | 88.2 | -0.350 |
| Appendicular lean mass | Alzheimer's disease (Bellenguez 2022) | rs6054390 | 20:6592094 | A/G | 0.628 | -0.0188 (0.0020) | 1.4E-21 | -0.0008 (0.0085) | 0.923 | 88.4 | 0.043 |
| Appendicular lean mass | Alzheimer's disease (Bellenguez 2022) | rs12700901 | 7:28783171 | A/C | 0.406 | -0.0184 (0.0019) | 1.7E-21 | 0.0024 (0.0083) | 0.776 | 93.8 | -0.130 |
| Appendicular lean mass | Alzheimer's disease (Bellenguez 2022) | rs10945540 | 6:158738975 | A/G | 0.665 | -0.0190 (0.0020) | 1.8E-21 | -0.0002 (0.0086) | 0.978 | 90.2 | 0.011 |
| Appendicular lean mass | Alzheimer's disease (Bellenguez 2022) | rs670318 | 1:63727542 | C/T | 0.952 | 0.0413 (0.0044) | 2.5E-21 | -0.0096 (0.0202) | 0.637 | 88.1 | -0.232 |
| Appendicular lean mass | Alzheimer's disease (Bellenguez 2022) | rs112537273 | 8:38248306 | C/T | 0.230 | -0.0212 (0.0022) | 3.3E-21 | -0.0128 (0.0098) | 0.194 | 92.9 | 0.604 |
| Appendicular lean mass | Alzheimer's disease (Bellenguez 2022) | rs1040977 | 9:95554873 | T/C | 0.166 | -0.0240 (0.0025) | 3.9E-21 | -0.0027 (0.0110) | 0.807 | 92.2 | 0.113 |
| Appendicular lean mass | Alzheimer's disease (Bellenguez 2022) | rs7701233 | 5:171218388 | C/T | 0.428 | -0.0179 (0.0019) | 5.1E-21 | 0.0050 (0.0083) | 0.543 | 88.8 | -0.279 |
| Appendicular lean mass | Alzheimer's disease (Bellenguez 2022) | rs2212926 | 21:38066883 | A/C | 0.211 | -0.0220 (0.0023) | 7.7E-21 | 0.0028 (0.0103) | 0.786 | 91.5 | -0.127 |
| Appendicular lean mass | Alzheimer's disease (Bellenguez 2022) | rs10832963 | 11:18664241 | G/T | 0.745 | -0.0203 (0.0022) | 9.2E-21 | 0.0158 (0.0093) | 0.089 | 85.1 | -0.778 |
| Appendicular lean mass | Alzheimer's disease (Bellenguez 2022) | rs7636265 | 3:134374223 | A/G | 0.638 | -0.0183 (0.0020) | 9.4E-21 | 0.0088 (0.0085) | 0.298 | 83.7 | -0.481 |
| Appendicular lean mass | Alzheimer's disease (Bellenguez 2022) | rs9384679 | 6:108864419 | T/C | 0.370 | -0.0182 (0.0019) | 9.9E-21 | -0.0238 (0.0084) | 0.004 | 91.8 | 1.308 |
| Appendicular lean mass | Alzheimer's disease (Bellenguez 2022) | rs17478946 | 12:24093062 | G/A | 0.300 | -0.0192 (0.0021) | 1.0E-20 | 0.0135 (0.0090) | 0.134 | 83.6 | -0.703 |
| Appendicular lean mass | Alzheimer's disease (Bellenguez 2022) | rs9957318 | 18:33039106 | G/A | 0.348 | 0.0187 (0.0020) | 1.0E-20 | 0.0049 (0.0085) | 0.567 | 87.4 | 0.262 |
| Appendicular lean mass | Alzheimer's disease (Bellenguez 2022) | rs1473441 | 8:49588463 | G/A | 0.724 | -0.0198 (0.0021) | 1.2E-20 | 0.0009 (0.0090) | 0.923 | 88.9 | -0.045 |
| Appendicular lean mass | Alzheimer's disease (Bellenguez 2022) | rs34287 | 5:67585143 | A/G | 0.341 | 0.0187 (0.0020) | 1.2E-20 | 0.0118 (0.0086) | 0.172 | 87.4 | 0.631 |
| Appendicular lean mass | Alzheimer's disease (Bellenguez 2022) | rs35816944 | 16:1828030 | A/G | 0.007 | -0.1088 (0.0117) | 1.3E-20 | 0.0009 (0.0567) | 0.987 | 86.5 | -0.008 |
| Appendicular lean mass | Alzheimer's disease (Bellenguez 2022) | rs7633464 | 3:98715823 | A/G | 0.479 | 0.0175 (0.0019) | 1.3E-20 | 0.0011 (0.0082) | 0.888 | 84.8 | 0.063 |
| Appendicular lean mass | Alzheimer's disease (Bellenguez 2022) | rs2993531 | 1:212190116 | C/A | 0.589 | 0.0176 (0.0019) | 1.7E-20 | 0.0038 (0.0082) | 0.642 | 85.8 | 0.216 |
| Appendicular lean mass | Alzheimer's disease (Bellenguez 2022) | rs9696116 | 9:139100805 | C/T | 0.318 | -0.0188 (0.0020) | 2.4E-20 | 0.0104 (0.0090) | 0.250 | 88.4 | -0.553 |
| Appendicular lean mass | Alzheimer's disease (Bellenguez 2022) | rs3103268 | 2:232984969 | T/C | 0.902 | 0.0291 (0.0031) | 2.7E-20 | -0.0044 (0.0138) | 0.752 | 88.1 | -0.151 |
| Appendicular lean mass | Alzheimer's disease (Bellenguez 2022) | rs4985445 | 16:69867835 | G/A | 0.457 | -0.0175 (0.0019) | 3.3E-20 | 0.0031 (0.0082) | 0.706 | 84.8 | -0.177 |
| Appendicular lean mass | Alzheimer's disease (Bellenguez 2022) | rs11590254 | 1:92316573 | T/A | 0.311 | 0.0186 (0.0020) | 4.3E-20 | 0.0103 (0.0088) | 0.240 | 86.5 | 0.554 |
| Appendicular lean mass | Alzheimer's disease (Bellenguez 2022) | rs73125634 | 20:20069826 | T/G | 0.278 | -0.0195 (0.0021) | 5.1E-20 | 0.0099 (0.0091) | 0.278 | 86.2 | -0.508 |
| Appendicular lean mass | Alzheimer's disease (Bellenguez 2022) | rs6874142 | 5:172753555 | G/T | 0.114 | 0.0288 (0.0031) | 5.1E-20 | 0.0053 (0.0140) | 0.707 | 86.3 | 0.184 |
| Appendicular lean mass | Alzheimer's disease (Bellenguez 2022) | rs140657345 | 15:41615909 | TCCTC/T | 0.252 | -0.0200 (0.0022) | 5.5E-20 | -0.0238 (0.0096) | 0.014 | 82.6 | 1.190 |
| Appendicular lean mass | Alzheimer's disease (Bellenguez 2022) | rs62466110 | 7:92623541 | C/T | 0.067 | -0.0371 (0.0041) | 5.7E-20 | -0.0146 (0.0186) | 0.435 | 81.9 | 0.394 |
| Appendicular lean mass | Alzheimer's disease (Bellenguez 2022) | rs12702693 | 7:8101039 | T/C | 0.454 | 0.0173 (0.0019) | 6.6E-20 | -0.0090 (0.0082) | 0.274 | 82.9 | -0.520 |
| Appendicular lean mass | Alzheimer's disease (Bellenguez 2022) | rs34345560 | 11:69081998 | A/G | 0.195 | 0.0219 (0.0024) | 7.1E-20 | 0.0015 (0.0106) | 0.884 | 83.3 | 0.068 |
| Appendicular lean mass | Alzheimer's disease (Bellenguez 2022) | rs12055045 | 5:137608056 | T/C | 0.206 | 0.0213 (0.0023) | 7.1E-20 | -0.0092 (0.0098) | 0.350 | 85.8 | -0.432 |
| Appendicular lean mass | Alzheimer's disease (Bellenguez 2022) | rs655113 | 6:52269151 | C/T | 0.301 | 0.0188 (0.0021) | 7.4E-20 | -0.0082 (0.0089) | 0.356 | 80.2 | -0.436 |
| Appendicular lean mass | Alzheimer's disease (Bellenguez 2022) | rs9832919 | 3:132184526 | G/A | 0.357 | -0.0179 (0.0020) | 7.9E-20 | -0.0050 (0.0085) | 0.555 | 80.1 | 0.279 |
| Appendicular lean mass | Alzheimer's disease (Bellenguez 2022) | rs8107967 | 19:7972615 | G/A | 0.566 | -0.0174 (0.0019) | 9.5E-20 | 0.0047 (0.0089) | 0.595 | 83.9 | -0.270 |
| Appendicular lean mass | Alzheimer's disease (Bellenguez 2022) | rs36012032 | 3:52814709 | A/C | 0.091 | 0.0298 (0.0033) | 9.9E-20 | 0.0101 (0.0138) | 0.464 | 81.5 | 0.339 |
| Appendicular lean mass | Alzheimer's disease (Bellenguez 2022) | rs76693355 | 11:120292477 | C/T | 0.116 | -0.0268 (0.0030) | 1.2E-19 | 0.0029 (0.0130) | 0.823 | 79.8 | -0.108 |
| Appendicular lean mass | Alzheimer's disease (Bellenguez 2022) | rs78180894 | 17:7483662 | C/G | 0.074 | 0.0338 (0.0037) | 1.3E-19 | 0.0309 (0.0166) | 0.062 | 83.5 | 0.914 |
| Appendicular lean mass | Alzheimer's disease (Bellenguez 2022) | rs12533452 | 7:19016871 | T/C | 0.157 | 0.0237 (0.0026) | 1.3E-19 | 0.0226 (0.0111) | 0.043 | 83.1 | 0.954 |
| Appendicular lean mass | Alzheimer's disease (Bellenguez 2022) | rs77542162 | 17:67081278 | G/A | 0.023 | 0.0576 (0.0064) | 1.5E-19 | 0.0144 (0.0318) | 0.651 | 81.0 | 0.250 |
| Appendicular lean mass | Alzheimer's disease (Bellenguez 2022) | rs12997625 | 2:202970250 | T/C | 0.527 | -0.0170 (0.0019) | 1.5E-19 | 0.0241 (0.0082) | 0.003 | 80.1 | -1.418 |
| Appendicular lean mass | Alzheimer's disease (Bellenguez 2022) | rs4900578 | 14:103926010 | T/A | 0.369 | -0.0177 (0.0020) | 1.8E-19 | -0.0015 (0.0087) | 0.863 | 78.3 | 0.085 |
| Appendicular lean mass | Alzheimer's disease (Bellenguez 2022) | rs591668 | 3:27535931 | A/G | 0.396 | -0.0174 (0.0019) | 2.0E-19 | -0.0004 (0.0083) | 0.964 | 83.9 | 0.023 |
| Appendicular lean mass | Alzheimer's disease (Bellenguez 2022) | rs11959466 | 5:42803824 | T/C | 0.056 | 0.0380 (0.0042) | 2.2E-19 | -0.0108 (0.0188) | 0.566 | 81.9 | -0.284 |
| Appendicular lean mass | Alzheimer's disease (Bellenguez 2022) | rs261999 | 5:95899427 | T/C | 0.370 | 0.0175 (0.0019) | 2.5E-19 | 0.0022 (0.0084) | 0.793 | 84.8 | 0.126 |
| Appendicular lean mass | Alzheimer's disease (Bellenguez 2022) | rs2142644 | 6:19053843 | A/C | 0.672 | -0.0181 (0.0020) | 3.4E-19 | 0.0333 (0.0089) | 1.7E-04 | 81.9 | -1.840 |
| Appendicular lean mass | Alzheimer's disease (Bellenguez 2022) | rs11672848 | 19:37570704 | T/C | 0.525 | -0.0171 (0.0019) | 7.7E-19 | -0.0042 (0.0088) | 0.635 | 81.0 | 0.246 |
| Appendicular lean mass | Alzheimer's disease (Bellenguez 2022) | rs894736 | 12:54418166 | G/A | 0.363 | -0.0174 (0.0020) | 1.1E-18 | 0.0157 (0.0086) | 0.067 | 75.7 | -0.902 |
| Appendicular lean mass | Alzheimer's disease (Bellenguez 2022) | rs2112617 | 17:46977125 | A/G | 0.529 | -0.0167 (0.0019) | 1.1E-18 | -0.0148 (0.0081) | 0.068 | 77.2 | 0.886 |
| Appendicular lean mass | Alzheimer's disease (Bellenguez 2022) | rs11049704 | 12:28691701 | G/C | 0.293 | -0.0183 (0.0021) | 1.1E-18 | 0.0115 (0.0092) | 0.212 | 75.9 | -0.628 |
| Appendicular lean mass | Alzheimer's disease (Bellenguez 2022) | rs700677 | 2:198702424 | A/C | 0.350 | 0.0173 (0.0020) | 1.1E-18 | -0.0017 (0.0085) | 0.843 | 74.8 | -0.098 |
| Appendicular lean mass | Alzheimer's disease (Bellenguez 2022) | rs6739278 | 2:44401055 | C/T | 0.808 | -0.0210 (0.0024) | 1.3E-18 | 0.0033 (0.0102) | 0.743 | 76.6 | -0.157 |
| Appendicular lean mass | Alzheimer's disease (Bellenguez 2022) | rs1805165 | 2:88874891 | A/C | 0.720 | -0.0184 (0.0021) | 1.3E-18 | 0.0084 (0.0090) | 0.349 | 76.8 | -0.457 |
| Appendicular lean mass | Alzheimer's disease (Bellenguez 2022) | rs6569643 | 6:130312646 | A/G | 0.526 | -0.0167 (0.0019) | 1.4E-18 | 0.0115 (0.0083) | 0.164 | 77.2 | -0.689 |
| Appendicular lean mass | Alzheimer's disease (Bellenguez 2022) | rs2052478 | 5:77437291 | T/C | 0.235 | -0.0195 (0.0022) | 1.8E-18 | 0.0041 (0.0098) | 0.673 | 78.6 | -0.210 |
| Appendicular lean mass | Alzheimer's disease (Bellenguez 2022) | rs9513510 | 13:99571922 | C/G | 0.699 | -0.0181 (0.0021) | 2.5E-18 | 0.0108 (0.0088) | 0.221 | 74.3 | -0.597 |
| Appendicular lean mass | Alzheimer's disease (Bellenguez 2022) | rs7731023 | 5:36181627 | G/A | 0.575 | 0.0166 (0.0019) | 3.5E-18 | 0.0006 (0.0083) | 0.943 | 76.3 | 0.036 |
| Appendicular lean mass | Alzheimer's disease (Bellenguez 2022) | rs2531991 | 16:4023553 | A/G | 0.748 | 0.0189 (0.0022) | 5.2E-18 | -0.0197 (0.0094) | 0.037 | 73.8 | -1.042 |
| Appendicular lean mass | Alzheimer's disease (Bellenguez 2022) | rs12909863 | 15:75825822 | C/G | 0.251 | 0.0189 (0.0022) | 6.0E-18 | -0.0090 (0.0093) | 0.334 | 73.8 | -0.476 |
| Appendicular lean mass | Alzheimer's disease (Bellenguez 2022) | rs12351226 | 9:98405230 | T/C | 0.171 | 0.0218 (0.0025) | 9.1E-18 | -0.0018 (0.0108) | 0.866 | 76.0 | -0.083 |
| Appendicular lean mass | Alzheimer's disease (Bellenguez 2022) | rs7512641 | 1:10822171 | A/T | 0.741 | -0.0185 (0.0022) | 9.9E-18 | -0.0078 (0.0094) | 0.407 | 70.7 | 0.422 |
| Appendicular lean mass | Alzheimer's disease (Bellenguez 2022) | rs7666804 | 4:57750148 | C/T | 0.389 | -0.0167 (0.0019) | 9.9E-18 | -0.0069 (0.0085) | 0.419 | 77.2 | 0.413 |
| Appendicular lean mass | Alzheimer's disease (Bellenguez 2022) | rs395980 | 4:177430072 | G/T | 0.264 | -0.0184 (0.0021) | 1.0E-17 | 0.0028 (0.0092) | 0.762 | 76.8 | -0.152 |
| Appendicular lean mass | Alzheimer's disease (Bellenguez 2022) | rs12037677 | 1:109918766 | C/T | 0.718 | -0.0178 (0.0021) | 1.2E-17 | -0.0035 (0.0092) | 0.705 | 71.8 | 0.197 |
| Appendicular lean mass | Alzheimer's disease (Bellenguez 2022) | rs73384223 | 9:3869315 | C/T | 0.197 | -0.0205 (0.0024) | 1.3E-17 | -0.0069 (0.0104) | 0.505 | 73.0 | 0.337 |
| Appendicular lean mass | Alzheimer's disease (Bellenguez 2022) | rs7598430 | 2:219193963 | T/C | 0.505 | -0.0160 (0.0019) | 1.4E-17 | -0.0118 (0.0082) | 0.148 | 70.9 | 0.738 |
| Appendicular lean mass | Alzheimer's disease (Bellenguez 2022) | rs2721940 | 8:116636281 | C/A | 0.602 | -0.0165 (0.0019) | 1.5E-17 | 0.0169 (0.0083) | 0.042 | 75.4 | -1.024 |
| Appendicular lean mass | Alzheimer's disease (Bellenguez 2022) | rs9817452 | 3:156795414 | T/G | 0.388 | 0.0165 (0.0019) | 1.9E-17 | 0.0095 (0.0084) | 0.260 | 75.4 | 0.576 |
| Appendicular lean mass | Alzheimer's disease (Bellenguez 2022) | rs10922475 | 1:89142142 | A/C | 0.539 | 0.0159 (0.0019) | 2.2E-17 | 0.0064 (0.0082) | 0.435 | 70.0 | 0.403 |
| Appendicular lean mass | Alzheimer's disease (Bellenguez 2022) | rs12299065 | 12:117442304 | A/G | 0.127 | -0.0240 (0.0028) | 2.2E-17 | 0.0095 (0.0118) | 0.420 | 73.5 | -0.396 |
| Appendicular lean mass | Alzheimer's disease (Bellenguez 2022) | rs56208656 | 19:3575343 | A/G | 0.110 | -0.0284 (0.0034) | 3.0E-17 | 0.0037 (0.0160) | 0.819 | 69.8 | -0.130 |
| Appendicular lean mass | Alzheimer's disease (Bellenguez 2022) | rs10040039 | 5:88354597 | G/A | 0.387 | -0.0163 (0.0019) | 3.3E-17 | 0.0042 (0.0084) | 0.612 | 73.6 | -0.258 |
| Appendicular lean mass | Alzheimer's disease (Bellenguez 2022) | rs59951000 | 18:74975828 | T/C | 0.042 | -0.0400 (0.0047) | 3.3E-17 | 0.0111 (0.0201) | 0.579 | 72.4 | -0.278 |
| Appendicular lean mass | Alzheimer's disease (Bellenguez 2022) | rs2347603 | 2:47297426 | A/T | 0.742 | -0.0181 (0.0022) | 5.6E-17 | 0.0061 (0.0093) | 0.513 | 67.7 | -0.337 |
| Appendicular lean mass | Alzheimer's disease (Bellenguez 2022) | rs1341215 | 9:111662350 | A/G | 0.137 | 0.0229 (0.0027) | 6.3E-17 | 0.0050 (0.0116) | 0.666 | 71.9 | 0.218 |
| Appendicular lean mass | Alzheimer's disease (Bellenguez 2022) | rs10005035 | 4:12865684 | G/C | 0.282 | -0.0175 (0.0021) | 6.5E-17 | 0.0028 (0.0091) | 0.756 | 69.4 | -0.160 |
| Appendicular lean mass | Alzheimer's disease (Bellenguez 2022) | rs1556659 | 10:130834698 | T/C | 0.382 | 0.0163 (0.0020) | 7.2E-17 | 0.0072 (0.0087) | 0.412 | 66.4 | 0.442 |
| Appendicular lean mass | Alzheimer's disease (Bellenguez 2022) | rs10776560 | 10:50542358 | T/C | 0.499 | -0.0157 (0.0019) | 7.9E-17 | -0.0013 (0.0081) | 0.871 | 68.3 | 0.083 |
| Appendicular lean mass | Alzheimer's disease (Bellenguez 2022) | rs10514518 | 16:81579827 | T/C | 0.685 | 0.0171 (0.0020) | 8.3E-17 | -0.0066 (0.0087) | 0.450 | 73.1 | -0.386 |
| Appendicular lean mass | Alzheimer's disease (Bellenguez 2022) | rs112898929 | 16:88298795 | A/T | 0.080 | -0.0296 (0.0036) | 9.6E-17 | 0.0030 (0.0160) | 0.853 | 67.6 | -0.101 |
| Appendicular lean mass | Alzheimer's disease (Bellenguez 2022) | rs59725651 | 18:77184667 | G/A | 0.339 | -0.0167 (0.0020) | 1.1E-16 | -0.0144 (0.0088) | 0.102 | 69.7 | 0.862 |
| Appendicular lean mass | Alzheimer's disease (Bellenguez 2022) | rs1063582 | 8:23167353 | G/T | 0.765 | -0.0185 (0.0022) | 1.1E-16 | -0.0206 (0.0096) | 0.032 | 70.7 | 1.114 |
| Appendicular lean mass | Alzheimer's disease (Bellenguez 2022) | rs10823149 | 10:69935396 | T/G | 0.548 | 0.0157 (0.0019) | 1.2E-16 | -0.0121 (0.0082) | 0.137 | 68.3 | -0.771 |
| Appendicular lean mass | Alzheimer's disease (Bellenguez 2022) | rs4815952 | 20:6934897 | C/T | 0.522 | -0.0161 (0.0019) | 1.2E-16 | 0.0019 (0.0085) | 0.823 | 71.8 | -0.118 |
| Appendicular lean mass | Alzheimer's disease (Bellenguez 2022) | rs9809116 | 3:72397279 | G/A | 0.408 | -0.0160 (0.0019) | 1.3E-16 | -0.0004 (0.0083) | 0.961 | 70.9 | 0.025 |
| Appendicular lean mass | Alzheimer's disease (Bellenguez 2022) | rs12512942 | 4:177766307 | A/G | 0.638 | -0.0162 (0.0020) | 1.6E-16 | 0.0002 (0.0085) | 0.981 | 65.6 | -0.012 |
| Appendicular lean mass | Alzheimer's disease (Bellenguez 2022) | rs10843391 | 12:29508642 | G/A | 0.285 | 0.0172 (0.0021) | 2.1E-16 | 0.0029 (0.0089) | 0.741 | 67.1 | 0.169 |
| Appendicular lean mass | Alzheimer's disease (Bellenguez 2022) | rs9391254 | 6:105377347 | T/C | 0.321 | 0.0166 (0.0020) | 2.1E-16 | 0.0031 (0.0087) | 0.726 | 68.9 | 0.187 |
| Appendicular lean mass | Alzheimer's disease (Bellenguez 2022) | rs56363908 | 7:96611052 | G/A | 0.042 | -0.0382 (0.0047) | 3.9E-16 | -0.0086 (0.0204) | 0.675 | 66.1 | 0.225 |
| Appendicular lean mass | Alzheimer's disease (Bellenguez 2022) | rs9479012 | 6:151657994 | G/A | 0.094 | 0.0263 (0.0032) | 4.1E-16 | 0.0019 (0.0135) | 0.890 | 67.5 | 0.072 |
| Appendicular lean mass | Alzheimer's disease (Bellenguez 2022) | rs6543146 | 2:103096695 | G/T | 0.558 | 0.0154 (0.0019) | 4.2E-16 | 0.0045 (0.0082) | 0.583 | 65.7 | 0.292 |
| Appendicular lean mass | Alzheimer's disease (Bellenguez 2022) | rs17138358 | 7:17920253 | C/G | 0.398 | 0.0157 (0.0019) | 4.4E-16 | -0.0193 (0.0083) | 0.019 | 68.3 | -1.229 |
| Appendicular lean mass | Alzheimer's disease (Bellenguez 2022) | rs7367519 | 1:204479176 | C/T | 0.685 | 0.0164 (0.0020) | 4.7E-16 | 0.0062 (0.0087) | 0.477 | 67.2 | 0.378 |
| Appendicular lean mass | Alzheimer's disease (Bellenguez 2022) | rs61729527 | 8:77761919 | T/C | 0.052 | -0.0346 (0.0043) | 4.9E-16 | -0.0007 (0.0190) | 0.969 | 64.8 | 0.020 |
| Appendicular lean mass | Alzheimer's disease (Bellenguez 2022) | rs10208668 | 2:20556151 | C/G | 0.035 | 0.0415 (0.0051) | 5.0E-16 | -0.0455 (0.0220) | 0.038 | 66.2 | -1.096 |
| Appendicular lean mass | Alzheimer's disease (Bellenguez 2022) | rs2287821 | 19:33935102 | T/C | 0.509 | -0.0153 (0.0019) | 7.0E-16 | 0.0028 (0.0087) | 0.747 | 64.8 | -0.183 |
| Appendicular lean mass | Alzheimer's disease (Bellenguez 2022) | rs35963161 | 20:49210635 | A/G | 0.476 | -0.0157 (0.0019) | 7.5E-16 | 0.0111 (0.0085) | 0.189 | 68.3 | -0.707 |
| Appendicular lean mass | Alzheimer's disease (Bellenguez 2022) | rs1056747 | 9:35690102 | G/A | 0.412 | -0.0155 (0.0019) | 8.0E-16 | 0.0027 (0.0082) | 0.742 | 66.5 | -0.174 |
| Appendicular lean mass | Alzheimer's disease (Bellenguez 2022) | rs11777835 | 8:144986103 | C/T | 0.473 | -0.0152 (0.0019) | 9.6E-16 | 0.0112 (0.0082) | 0.173 | 64.0 | -0.737 |
| Appendicular lean mass | Alzheimer's disease (Bellenguez 2022) | rs2165772 | 11:27091907 | A/G | 0.354 | 0.0160 (0.0020) | 1.0E-15 | -0.0093 (0.0086) | 0.280 | 64.0 | -0.581 |
| Appendicular lean mass | Alzheimer's disease (Bellenguez 2022) | rs2596144 | 12:122297738 | C/T | 0.870 | -0.0223 (0.0028) | 1.5E-15 | 0.0193 (0.0119) | 0.105 | 63.4 | -0.865 |
| Appendicular lean mass | Alzheimer's disease (Bellenguez 2022) | rs8904 | 14:35871217 | A/G | 0.363 | -0.0157 (0.0020) | 1.5E-15 | 0.0099 (0.0083) | 0.234 | 61.6 | -0.631 |
| Appendicular lean mass | Alzheimer's disease (Bellenguez 2022) | rs4504126 | 3:33600582 | C/A | 0.028 | 0.0460 (0.0058) | 1.6E-15 | -0.0013 (0.0259) | 0.960 | 62.9 | -0.028 |
| Appendicular lean mass | Alzheimer's disease (Bellenguez 2022) | rs2007022 | 20:3237674 | A/C | 0.232 | 0.0179 (0.0022) | 1.6E-15 | -0.0159 (0.0098) | 0.103 | 66.2 | -0.888 |
| Appendicular lean mass | Alzheimer's disease (Bellenguez 2022) | rs2324154 | 4:24027226 | A/C | 0.510 | 0.0150 (0.0019) | 1.9E-15 | 0.0065 (0.0081) | 0.425 | 62.3 | 0.433 |
| Appendicular lean mass | Alzheimer's disease (Bellenguez 2022) | rs704660 | 11:30447998 | T/C | 0.409 | 0.0153 (0.0019) | 2.3E-15 | -0.0130 (0.0082) | 0.115 | 64.8 | -0.850 |
| Appendicular lean mass | Alzheimer's disease (Bellenguez 2022) | rs4076108 | 3:13736088 | T/A | 0.245 | 0.0174 (0.0022) | 2.3E-15 | -0.0173 (0.0095) | 0.068 | 62.5 | -0.994 |
| Appendicular lean mass | Alzheimer's disease (Bellenguez 2022) | rs7646501 | 3:24079291 | G/A | 0.264 | -0.0170 (0.0021) | 2.3E-15 | 0.0162 (0.0092) | 0.078 | 65.5 | -0.953 |
| Appendicular lean mass | Alzheimer's disease (Bellenguez 2022) | rs16844417 | 1:172377745 | A/G | 0.124 | 0.0226 (0.0029) | 2.6E-15 | -0.0346 (0.0129) | 0.008 | 60.7 | -1.531 |
| Appendicular lean mass | Alzheimer's disease (Bellenguez 2022) | rs12371664 | 12:91130760 | G/A | 0.272 | -0.0168 (0.0021) | 2.7E-15 | 0.0059 (0.0091) | 0.516 | 64.0 | -0.351 |
| Appendicular lean mass | Alzheimer's disease (Bellenguez 2022) | rs3121580 | 1:218492121 | C/T | 0.827 | 0.0197 (0.0025) | 2.7E-15 | -0.0121 (0.0106) | 0.255 | 62.1 | -0.614 |
| Appendicular lean mass | Alzheimer's disease (Bellenguez 2022) | rs7516643 | 1:33873010 | C/T | 0.672 | 0.0157 (0.0020) | 3.1E-15 | -0.0075 (0.0088) | 0.390 | 61.6 | -0.478 |
| Appendicular lean mass | Alzheimer's disease (Bellenguez 2022) | rs7228151 | 18:57181694 | C/T | 0.206 | -0.0185 (0.0023) | 3.2E-15 | 0.0018 (0.0100) | 0.860 | 64.7 | -0.097 |
| Appendicular lean mass | Alzheimer's disease (Bellenguez 2022) | rs35436119 | 9:119303347 | A/G | 0.075 | 0.0282 (0.0036) | 3.3E-15 | -0.0039 (0.0154) | 0.802 | 61.4 | -0.138 |
| Appendicular lean mass | Alzheimer's disease (Bellenguez 2022) | rs4903321 | 14:75911083 | A/G | 0.444 | -0.0151 (0.0019) | 3.4E-15 | 0.0158 (0.0082) | 0.055 | 63.2 | -1.046 |
| Appendicular lean mass | Alzheimer's disease (Bellenguez 2022) | rs190801170 | 12:57177813 | G/A | 0.074 | -0.0286 (0.0036) | 3.7E-15 | 0.0334 (0.0159) | 0.035 | 63.1 | -1.168 |
| Appendicular lean mass | Alzheimer's disease (Bellenguez 2022) | rs72829852 | 17:46633974 | T/C | 0.062 | 0.0309 (0.0039) | 3.7E-15 | 0.0371 (0.0171) | 0.030 | 62.8 | 1.201 |
| Appendicular lean mass | Alzheimer's disease (Bellenguez 2022) | rs13170063 | 5:157895013 | A/G | 0.592 | -0.0152 (0.0019) | 4.1E-15 | 0.0109 (0.0085) | 0.201 | 64.0 | -0.717 |
| Appendicular lean mass | Alzheimer's disease (Bellenguez 2022) | rs10822117 | 10:52786701 | G/A | 0.237 | -0.0176 (0.0022) | 4.2E-15 | 0.0015 (0.0096) | 0.879 | 64.0 | -0.085 |
| Appendicular lean mass | Alzheimer's disease (Bellenguez 2022) | rs7761910 | 6:155553832 | T/C | 0.309 | -0.0160 (0.0020) | 5.1E-15 | -0.0008 (0.0088) | 0.931 | 64.0 | 0.050 |
| Appendicular lean mass | Alzheimer's disease (Bellenguez 2022) | rs10796828 | 11:69490346 | G/T | 0.635 | 0.0154 (0.0020) | 5.8E-15 | 0.0038 (0.0086) | 0.658 | 59.3 | 0.247 |
| Appendicular lean mass | Alzheimer's disease (Bellenguez 2022) | rs28817902 | 5:138916010 | G/A | 0.118 | 0.0230 (0.0029) | 6.2E-15 | -0.0135 (0.0129) | 0.294 | 62.9 | -0.587 |
| Appendicular lean mass | Alzheimer's disease (Bellenguez 2022) | rs11021305 | 11:95498269 | C/T | 0.384 | 0.0152 (0.0019) | 6.3E-15 | 0.0063 (0.0084) | 0.452 | 64.0 | 0.414 |
| Appendicular lean mass | Alzheimer's disease (Bellenguez 2022) | rs34786000 | 19:9942123 | T/G | 0.554 | -0.0150 (0.0019) | 6.6E-15 | 0.0068 (0.0089) | 0.442 | 62.3 | -0.453 |
| Appendicular lean mass | Alzheimer's disease (Bellenguez 2022) | rs62501195 | 8:24041988 | C/A | 0.171 | -0.0198 (0.0025) | 8.1E-15 | 0.0118 (0.0109) | 0.276 | 62.7 | -0.596 |
| Appendicular lean mass | Alzheimer's disease (Bellenguez 2022) | rs4776624 | 15:73359881 | T/C | 0.397 | -0.0150 (0.0019) | 1.2E-14 | -0.0017 (0.0083) | 0.842 | 62.3 | 0.113 |
| Appendicular lean mass | Alzheimer's disease (Bellenguez 2022) | rs7320878 | 13:91994132 | A/G | 0.603 | -0.0150 (0.0019) | 1.3E-14 | -0.0160 (0.0085) | 0.059 | 62.3 | 1.067 |
| Appendicular lean mass | Alzheimer's disease (Bellenguez 2022) | rs28529055 | 14:92428216 | T/G | 0.437 | -0.0147 (0.0019) | 1.9E-14 | 0.0020 (0.0084) | 0.807 | 59.9 | -0.136 |
| Appendicular lean mass | Alzheimer's disease (Bellenguez 2022) | rs1035583 | 2:207326937 | A/G | 0.618 | 0.0148 (0.0019) | 2.0E-14 | 0.0098 (0.0083) | 0.237 | 60.7 | 0.662 |
| Appendicular lean mass | Alzheimer's disease (Bellenguez 2022) | rs9394578 | 6:39165859 | A/C | 0.258 | -0.0164 (0.0022) | 2.6E-14 | 0.0027 (0.0094) | 0.774 | 55.6 | -0.165 |
| Appendicular lean mass | Alzheimer's disease (Bellenguez 2022) | rs73382475 | 21:47549715 | T/C | 0.089 | 0.0254 (0.0033) | 3.0E-14 | -0.0157 (0.0144) | 0.275 | 59.2 | -0.618 |
| Appendicular lean mass | Alzheimer's disease (Bellenguez 2022) | rs6721191 | 2:10190115 | G/A | 0.578 | -0.0144 (0.0019) | 3.2E-14 | -0.0065 (0.0083) | 0.435 | 57.4 | 0.451 |
| Appendicular lean mass | Alzheimer's disease (Bellenguez 2022) | rs2609334 | 6:168847175 | C/T | 0.226 | -0.0171 (0.0022) | 3.4E-14 | 0.0053 (0.0097) | 0.586 | 60.4 | -0.310 |
| Appendicular lean mass | Alzheimer's disease (Bellenguez 2022) | rs73197345 | 21:36770120 | A/T | 0.137 | 0.0211 (0.0028) | 3.5E-14 | 0.0124 (0.0119) | 0.296 | 56.8 | 0.588 |
| Appendicular lean mass | Alzheimer's disease (Bellenguez 2022) | rs9525326 | 13:115075715 | G/A | 0.188 | -0.0184 (0.0024) | 3.6E-14 | 0.0080 (0.0105) | 0.444 | 58.8 | -0.435 |
| Appendicular lean mass | Alzheimer's disease (Bellenguez 2022) | rs11673381 | 19:4506296 | G/A | 0.530 | -0.0144 (0.0019) | 3.7E-14 | -0.0056 (0.0088) | 0.524 | 57.4 | 0.389 |
| Appendicular lean mass | Alzheimer's disease (Bellenguez 2022) | rs74379684 | 15:94050205 | T/C | 0.075 | -0.0272 (0.0036) | 4.4E-14 | 0.0122 (0.0155) | 0.432 | 57.1 | -0.449 |
| Appendicular lean mass | Alzheimer's disease (Bellenguez 2022) | rs12662115 | 6:169360124 | G/A | 0.327 | -0.0152 (0.0020) | 4.5E-14 | -0.0038 (0.0087) | 0.663 | 57.8 | 0.250 |
| Appendicular lean mass | Alzheimer's disease (Bellenguez 2022) | rs165849 | 22:19958669 | A/G | 0.698 | 0.0157 (0.0021) | 4.6E-14 | 0.0016 (0.0090) | 0.859 | 55.9 | 0.102 |
| Appendicular lean mass | Alzheimer's disease (Bellenguez 2022) | rs11198591 | 10:120515892 | A/G | 0.368 | 0.0148 (0.0020) | 4.9E-14 | 0.0005 (0.0085) | 0.951 | 54.8 | 0.034 |
| Appendicular lean mass | Alzheimer's disease (Bellenguez 2022) | rs7286917 | 22:39860868 | G/A | 0.746 | 0.0171 (0.0023) | 5.2E-14 | -0.0063 (0.0095) | 0.511 | 55.3 | -0.368 |
| Appendicular lean mass | Alzheimer's disease (Bellenguez 2022) | rs1557341 | 18:35127427 | C/A | 0.667 | 0.0153 (0.0020) | 5.8E-14 | -0.0075 (0.0087) | 0.391 | 58.5 | -0.490 |
| Appendicular lean mass | Alzheimer's disease (Bellenguez 2022) | rs599004 | 6:140439740 | T/C | 0.281 | -0.0157 (0.0021) | 6.5E-14 | 0.0037 (0.0090) | 0.679 | 55.9 | -0.236 |
| Appendicular lean mass | Alzheimer's disease (Bellenguez 2022) | rs1290786 | 3:169097381 | T/C | 0.431 | -0.0143 (0.0019) | 7.1E-14 | -0.0060 (0.0082) | 0.462 | 56.6 | 0.420 |
| Appendicular lean mass | Alzheimer's disease (Bellenguez 2022) | rs4622329 | 12:102321935 | A/G | 0.350 | 0.0149 (0.0020) | 8.6E-14 | -0.0025 (0.0084) | 0.764 | 55.5 | -0.168 |
| Appendicular lean mass | Alzheimer's disease (Bellenguez 2022) | rs73040028 | 3:23171805 | T/C | 0.229 | 0.0168 (0.0022) | 9.0E-14 | -0.0059 (0.0099) | 0.550 | 58.3 | -0.351 |
| Appendicular lean mass | Alzheimer's disease (Bellenguez 2022) | rs10810474 | 9:15911359 | T/C | 0.418 | 0.0143 (0.0019) | 9.6E-14 | 0.0029 (0.0083) | 0.726 | 56.6 | 0.203 |
| Appendicular lean mass | Alzheimer's disease (Bellenguez 2022) | rs2232460 | 1:6659505 | A/G | 0.334 | 0.0148 (0.0020) | 1.0E-13 | 0.0298 (0.0086) | 5.6E-04 | 54.8 | 2.014 |
| Appendicular lean mass | Alzheimer's disease (Bellenguez 2022) | rs3760456 | 17:27948844 | T/C | 0.440 | -0.0142 (0.0019) | 1.1E-13 | -0.0195 (0.0082) | 0.018 | 55.9 | 1.373 |
| Appendicular lean mass | Alzheimer's disease (Bellenguez 2022) | rs2807339 | 1:22578063 | C/T | 0.759 | 0.0162 (0.0022) | 1.2E-13 | -0.0035 (0.0093) | 0.708 | 54.2 | -0.216 |
| Appendicular lean mass | Alzheimer's disease (Bellenguez 2022) | rs3798429 | 6:76604573 | G/T | 0.498 | 0.0140 (0.0019) | 1.3E-13 | -0.0047 (0.0081) | 0.561 | 54.3 | -0.336 |
| Appendicular lean mass | Alzheimer's disease (Bellenguez 2022) | rs78051210 | 6:131379491 | C/T | 0.077 | 0.0263 (0.0036) | 1.6E-13 | 0.0018 (0.0156) | 0.906 | 53.4 | 0.068 |
| Appendicular lean mass | Alzheimer's disease (Bellenguez 2022) | rs11927331 | 3:32937008 | A/C | 0.331 | -0.0147 (0.0020) | 1.9E-13 | -0.0080 (0.0086) | 0.353 | 54.0 | 0.544 |
| Appendicular lean mass | Alzheimer's disease (Bellenguez 2022) | rs17400325 | 2:178565913 | C/T | 0.042 | 0.0345 (0.0047) | 2.1E-13 | 0.0195 (0.0196) | 0.318 | 53.9 | 0.565 |
| Appendicular lean mass | Alzheimer's disease (Bellenguez 2022) | rs12344515 | 9:113801231 | T/C | 0.240 | -0.0163 (0.0022) | 2.3E-13 | 0.0040 (0.0094) | 0.675 | 54.9 | -0.245 |
| Appendicular lean mass | Alzheimer's disease (Bellenguez 2022) | rs7225068 | 17:60666425 | G/A | 0.591 | 0.0141 (0.0019) | 2.5E-13 | 0.0098 (0.0083) | 0.239 | 55.1 | 0.695 |
| Appendicular lean mass | Alzheimer's disease (Bellenguez 2022) | rs35631698 | 21:16549469 | GC/G | 0.375 | 0.0145 (0.0020) | 2.6E-13 | -0.0025 (0.0086) | 0.767 | 52.6 | -0.172 |
| Appendicular lean mass | Alzheimer's disease (Bellenguez 2022) | rs2138374 | 2:190014317 | C/T | 0.696 | -0.0149 (0.0020) | 2.8E-13 | 0.0035 (0.0088) | 0.694 | 55.5 | -0.235 |
| Appendicular lean mass | Alzheimer's disease (Bellenguez 2022) | rs35268848 | 16:67927240 | A/C | 0.012 | 0.0737 (0.0101) | 2.8E-13 | -0.0243 (0.0386) | 0.529 | 53.2 | -0.330 |
| Appendicular lean mass | Alzheimer's disease (Bellenguez 2022) | rs112521375 | 1:228099148 | T/A | 0.134 | -0.0202 (0.0028) | 3.1E-13 | -0.0048 (0.0122) | 0.697 | 52.0 | 0.238 |
| Appendicular lean mass | Alzheimer's disease (Bellenguez 2022) | rs147233090 | 15:44028047 | T/C | 0.025 | -0.0446 (0.0061) | 4.0E-13 | -0.0036 (0.0244) | 0.882 | 53.5 | 0.081 |
| Appendicular lean mass | Alzheimer's disease (Bellenguez 2022) | rs4733775 | 8:131213570 | A/G | 0.364 | 0.0143 (0.0020) | 4.1E-13 | -0.0118 (0.0084) | 0.161 | 51.1 | -0.825 |
| Appendicular lean mass | Alzheimer's disease (Bellenguez 2022) | rs610694 | 12:121304826 | C/T | 0.490 | 0.0136 (0.0019) | 4.3E-13 | 0.0065 (0.0081) | 0.424 | 51.2 | 0.478 |
| Appendicular lean mass | Alzheimer's disease (Bellenguez 2022) | rs5753518 | 22:31631314 | A/G | 0.090 | 0.0242 (0.0033) | 5.0E-13 | 0.0033 (0.0142) | 0.813 | 53.8 | 0.136 |
| Appendicular lean mass | Alzheimer's disease (Bellenguez 2022) | rs72976986 | 19:4050424 | A/G | 0.190 | 0.0176 (0.0024) | 5.3E-13 | -0.0029 (0.0111) | 0.793 | 53.8 | -0.165 |
| Appendicular lean mass | Alzheimer's disease (Bellenguez 2022) | rs56207248 | 2:25065986 | T/C | 0.066 | -0.0273 (0.0038) | 6.0E-13 | 0.0253 (0.0161) | 0.117 | 51.6 | -0.927 |
| Appendicular lean mass | Alzheimer's disease (Bellenguez 2022) | rs1487441 | 6:98553894 | A/G | 0.484 | 0.0136 (0.0019) | 7.5E-13 | -0.0012 (0.0081) | 0.881 | 51.2 | -0.088 |
| Appendicular lean mass | Alzheimer's disease (Bellenguez 2022) | rs1216743 | 11:100573120 | A/G | 0.719 | -0.0152 (0.0021) | 8.0E-13 | 0.0017 (0.0091) | 0.852 | 52.4 | -0.112 |
| Appendicular lean mass | Alzheimer's disease (Bellenguez 2022) | rs6975015 | 7:50617215 | A/G | 0.117 | 0.0211 (0.0029) | 8.0E-13 | -0.0148 (0.0124) | 0.234 | 52.9 | -0.701 |
| Appendicular lean mass | Alzheimer's disease (Bellenguez 2022) | rs10453441 | 22:46363739 | G/A | 0.402 | -0.0139 (0.0020) | 9.1E-13 | 0.0099 (0.0086) | 0.254 | 48.3 | -0.712 |
| Appendicular lean mass | Alzheimer's disease (Bellenguez 2022) | rs1330826 | 9:85129970 | C/G | 0.227 | 0.0162 (0.0023) | 1.0E-12 | -0.0133 (0.0098) | 0.175 | 49.6 | -0.821 |
| Appendicular lean mass | Alzheimer's disease (Bellenguez 2022) | rs55758152 | 5:171317318 | A/G | 0.326 | 0.0145 (0.0020) | 1.1E-12 | -0.0178 (0.0091) | 0.050 | 52.6 | -1.228 |
| Appendicular lean mass | Alzheimer's disease (Bellenguez 2022) | rs143076454 | 16:921179 | A/G | 0.019 | -0.0499 (0.0070) | 1.1E-12 | 0.0469 (0.0302) | 0.120 | 50.8 | -0.940 |
| Appendicular lean mass | Alzheimer's disease (Bellenguez 2022) | rs73158215 | 7:148390300 | G/A | 0.226 | -0.0163 (0.0023) | 1.1E-12 | 0.0026 (0.0099) | 0.793 | 50.2 | -0.160 |
| Appendicular lean mass | Alzheimer's disease (Bellenguez 2022) | rs60408354 | 7:70158495 | A/G | 0.073 | 0.0259 (0.0036) | 1.2E-12 | 0.0029 (0.0160) | 0.856 | 51.8 | 0.112 |
| Appendicular lean mass | Alzheimer's disease (Bellenguez 2022) | rs853168 | 5:142538764 | C/G | 0.247 | 0.0156 (0.0022) | 1.3E-12 | -0.0019 (0.0095) | 0.838 | 50.3 | -0.122 |
| Appendicular lean mass | Alzheimer's disease (Bellenguez 2022) | rs72771070 | 16:19993750 | T/C | 0.283 | 0.0150 (0.0021) | 1.3E-12 | 0.0059 (0.0091) | 0.516 | 51.0 | 0.393 |
| Appendicular lean mass | Alzheimer's disease (Bellenguez 2022) | rs12672217 | 7:156310948 | A/G | 0.361 | 0.0139 (0.0020) | 1.3E-12 | -0.0066 (0.0086) | 0.439 | 48.3 | -0.475 |
| Appendicular lean mass | Alzheimer's disease (Bellenguez 2022) | rs2578565 | 5:5460569 | T/C | 0.657 | -0.0141 (0.0020) | 1.4E-12 | -0.0164 (0.0086) | 0.058 | 49.7 | 1.163 |
| Appendicular lean mass | Alzheimer's disease (Bellenguez 2022) | rs572474 | 10:89791654 | G/A | 0.166 | 0.0180 (0.0025) | 1.5E-12 | -0.0073 (0.0109) | 0.507 | 51.8 | -0.406 |
| Appendicular lean mass | Alzheimer's disease (Bellenguez 2022) | rs17197114 | 14:21894526 | C/T | 0.177 | 0.0177 (0.0025) | 1.5E-12 | -0.0121 (0.0109) | 0.264 | 50.1 | -0.684 |
| Appendicular lean mass | Alzheimer's disease (Bellenguez 2022) | rs2289976 | 2:85818797 | A/G | 0.305 | 0.0144 (0.0020) | 1.6E-12 | 0.0155 (0.0089) | 0.081 | 51.8 | 1.076 |
| Appendicular lean mass | Alzheimer's disease (Bellenguez 2022) | rs4847378 | 1:93324634 | T/G | 0.612 | 0.0136 (0.0019) | 1.6E-12 | -0.0180 (0.0083) | 0.030 | 51.2 | -1.324 |
| Appendicular lean mass | Alzheimer's disease (Bellenguez 2022) | rs73114154 | 20:31449386 | T/C | 0.388 | 0.0138 (0.0020) | 1.7E-12 | 0.0217 (0.0083) | 0.009 | 47.6 | 1.572 |
| Appendicular lean mass | Alzheimer's disease (Bellenguez 2022) | rs7107356 | 11:47676170 | G/A | 0.507 | 0.0133 (0.0019) | 1.9E-12 | 0.0354 (0.0081) | 1.3E-05 | 49.0 | 2.662 |
| Appendicular lean mass | Alzheimer's disease (Bellenguez 2022) | rs6129229 | 20:37882317 | G/A | 0.075 | -0.0257 (0.0036) | 1.9E-12 | 0.0022 (0.0156) | 0.886 | 51.0 | -0.086 |
| Appendicular lean mass | Alzheimer's disease (Bellenguez 2022) | rs177592 | 7:28559785 | C/G | 0.101 | -0.0220 (0.0031) | 2.0E-12 | 0.0052 (0.0133) | 0.695 | 50.4 | -0.236 |
| Appendicular lean mass | Alzheimer's disease (Bellenguez 2022) | rs2607234 | 19:35563834 | G/A | 0.948 | -0.0302 (0.0043) | 2.0E-12 | 0.0737 (0.0197) | 1.8E-04 | 49.3 | -2.440 |
| Appendicular lean mass | Alzheimer's disease (Bellenguez 2022) | rs1977337 | 1:67419517 | C/T | 0.851 | 0.0186 (0.0026) | 2.1E-12 | -0.0034 (0.0116) | 0.767 | 51.2 | -0.183 |
| Appendicular lean mass | Alzheimer's disease (Bellenguez 2022) | rs6693481 | 1:203766395 | C/T | 0.695 | -0.0143 (0.0020) | 2.2E-12 | 0.0032 (0.0090) | 0.723 | 51.1 | -0.224 |
| Appendicular lean mass | Alzheimer's disease (Bellenguez 2022) | rs7574162 | 2:172133086 | T/C | 0.757 | 0.0153 (0.0022) | 2.4E-12 | -0.0084 (0.0095) | 0.377 | 48.4 | -0.549 |
| Appendicular lean mass | Alzheimer's disease (Bellenguez 2022) | rs310796 | 12:77453226 | T/G | 0.681 | 0.0142 (0.0020) | 2.5E-12 | -0.0123 (0.0087) | 0.160 | 50.4 | -0.866 |
| Appendicular lean mass | Alzheimer's disease (Bellenguez 2022) | rs11720869 | 3:185619716 | A/G | 0.669 | 0.0141 (0.0020) | 2.5E-12 | -0.0069 (0.0087) | 0.422 | 49.7 | -0.489 |
| Appendicular lean mass | Alzheimer's disease (Bellenguez 2022) | rs1717720 | 7:38112615 | G/A | 0.654 | 0.0139 (0.0020) | 2.7E-12 | 0.0017 (0.0085) | 0.842 | 48.3 | 0.122 |
| Appendicular lean mass | Alzheimer's disease (Bellenguez 2022) | rs75022676 | 2:60293216 | A/G | 0.208 | -0.0163 (0.0023) | 2.8E-12 | -0.0086 (0.0103) | 0.404 | 50.2 | 0.528 |
| Appendicular lean mass | Alzheimer's disease (Bellenguez 2022) | rs76488803 | 4:157770271 | A/G | 0.083 | -0.0240 (0.0034) | 3.0E-12 | 0.0048 (0.0143) | 0.735 | 49.8 | -0.200 |
| Appendicular lean mass | Alzheimer's disease (Bellenguez 2022) | rs7692387 | 4:156635309 | A/G | 0.185 | 0.0169 (0.0024) | 3.5E-12 | -0.0024 (0.0102) | 0.818 | 49.6 | -0.142 |
| Appendicular lean mass | Alzheimer's disease (Bellenguez 2022) | rs2362487 | 10:126208402 | G/C | 0.247 | 0.0154 (0.0022) | 3.6E-12 | -0.0040 (0.0094) | 0.668 | 49.0 | -0.260 |
| Appendicular lean mass | Alzheimer's disease (Bellenguez 2022) | rs112873218 | 11:1960119 | T/C | 0.105 | 0.0216 (0.0031) | 4.0E-12 | -0.0180 (0.0136) | 0.188 | 48.5 | -0.833 |
| Appendicular lean mass | Alzheimer's disease (Bellenguez 2022) | rs7259285 | 19:17153149 | A/G | 0.444 | -0.0132 (0.0019) | 5.1E-12 | 0.0087 (0.0088) | 0.323 | 48.3 | -0.659 |
| Appendicular lean mass | Alzheimer's disease (Bellenguez 2022) | rs36052389 | 4:39357183 | AT/A | 0.455 | 0.0132 (0.0019) | 5.3E-12 | -0.0016 (0.0084) | 0.853 | 48.3 | -0.121 |
| Appendicular lean mass | Alzheimer's disease (Bellenguez 2022) | rs9610447 | 22:20768891 | T/C | 0.747 | 0.0152 (0.0022) | 5.3E-12 | 0.0078 (0.0096) | 0.414 | 47.7 | 0.513 |
| Appendicular lean mass | Alzheimer's disease (Bellenguez 2022) | rs2925155 | 8:75886297 | T/C | 0.261 | -0.0150 (0.0022) | 5.5E-12 | -0.0064 (0.0093) | 0.492 | 46.5 | 0.427 |
| Appendicular lean mass | Alzheimer's disease (Bellenguez 2022) | rs17681189 | 2:65976175 | A/C | 0.423 | -0.0131 (0.0019) | 5.8E-12 | -0.0074 (0.0083) | 0.367 | 47.5 | 0.565 |
| Appendicular lean mass | Alzheimer's disease (Bellenguez 2022) | rs7679276 | 4:146860186 | G/A | 0.954 | -0.0330 (0.0048) | 5.9E-12 | -0.0016 (0.0212) | 0.938 | 47.3 | 0.048 |
| Appendicular lean mass | Alzheimer's disease (Bellenguez 2022) | rs75478182 | 20:50807839 | C/G | 0.088 | 0.0230 (0.0033) | 6.6E-12 | -0.0148 (0.0146) | 0.312 | 48.6 | -0.643 |
| Appendicular lean mass | Alzheimer's disease (Bellenguez 2022) | rs2343034 | 20:35860288 | A/T | 0.809 | 0.0166 (0.0024) | 6.7E-12 | -0.0126 (0.0105) | 0.228 | 47.8 | -0.759 |
| Appendicular lean mass | Alzheimer's disease (Bellenguez 2022) | rs10427685 | 22:46440359 | G/C | 0.064 | 0.0269 (0.0039) | 7.0E-12 | 0.0136 (0.0167) | 0.413 | 47.6 | 0.506 |
| Appendicular lean mass | Alzheimer's disease (Bellenguez 2022) | rs12463908 | 2:121470971 | C/A | 0.188 | -0.0165 (0.0024) | 7.3E-12 | -0.0105 (0.0103) | 0.305 | 47.3 | 0.636 |
| Appendicular lean mass | Alzheimer's disease (Bellenguez 2022) | rs10008637 | 4:77414144 | C/T | 0.462 | -0.0130 (0.0019) | 7.5E-12 | 0.0139 (0.0082) | 0.090 | 46.8 | -1.069 |
| Appendicular lean mass | Alzheimer's disease (Bellenguez 2022) | rs2125125 | 3:142600087 | C/T | 0.783 | 0.0158 (0.0023) | 7.8E-12 | -0.0043 (0.0102) | 0.672 | 47.2 | -0.272 |
| Appendicular lean mass | Alzheimer's disease (Bellenguez 2022) | rs6684045 | 1:184183215 | A/G | 0.382 | 0.0132 (0.0019) | 7.9E-12 | -0.0083 (0.0084) | 0.320 | 48.3 | -0.629 |
| Appendicular lean mass | Alzheimer's disease (Bellenguez 2022) | rs78444298 | 1:184672098 | A/G | 0.020 | -0.0465 (0.0068) | 8.3E-12 | -0.0468 (0.0324) | 0.149 | 46.8 | 1.006 |
| Appendicular lean mass | Alzheimer's disease (Bellenguez 2022) | rs116493405 | 3:114733556 | A/G | 0.054 | 0.0287 (0.0042) | 9.5E-12 | -0.0018 (0.0173) | 0.917 | 46.7 | -0.063 |
| Appendicular lean mass | Alzheimer's disease (Bellenguez 2022) | rs61919240 | 12:8831954 | A/T | 0.324 | 0.0137 (0.0020) | 9.8E-12 | 0.0099 (0.0089) | 0.268 | 46.9 | 0.723 |
| Appendicular lean mass | Alzheimer's disease (Bellenguez 2022) | rs963317 | 4:45129970 | G/A | 0.663 | -0.0136 (0.0020) | 1.0E-11 | -0.0086 (0.0086) | 0.319 | 46.2 | 0.632 |
| Appendicular lean mass | Alzheimer's disease (Bellenguez 2022) | rs945508 | 1:156907081 | C/T | 0.536 | -0.0128 (0.0019) | 1.1E-11 | 0.0007 (0.0082) | 0.935 | 45.4 | -0.055 |
| Appendicular lean mass | Alzheimer's disease (Bellenguez 2022) | rs9890062 | 17:17434352 | A/G | 0.061 | 0.0267 (0.0039) | 1.2E-11 | 0.0230 (0.0171) | 0.177 | 46.9 | 0.861 |
| Appendicular lean mass | Alzheimer's disease (Bellenguez 2022) | rs67625472 | 19:4968620 | C/T | 0.276 | -0.0144 (0.0021) | 1.3E-11 | 0.0076 (0.0097) | 0.429 | 47.0 | -0.528 |
| Appendicular lean mass | Alzheimer's disease (Bellenguez 2022) | rs2663126 | 15:99563857 | A/G | 0.691 | -0.0139 (0.0021) | 1.4E-11 | -0.0051 (0.0089) | 0.564 | 43.8 | 0.367 |
| Appendicular lean mass | Alzheimer's disease (Bellenguez 2022) | rs115233595 | 4:119744214 | C/T | 0.062 | -0.0264 (0.0039) | 1.4E-11 | 0.0330 (0.0172) | 0.055 | 45.8 | -1.250 |
| Appendicular lean mass | Alzheimer's disease (Bellenguez 2022) | rs12340775 | 9:13226945 | A/G | 0.055 | -0.0287 (0.0043) | 1.7E-11 | 0.0009 (0.0186) | 0.960 | 44.5 | -0.031 |
| Appendicular lean mass | Alzheimer's disease (Bellenguez 2022) | rs2252031 | 12:103248463 | C/T | 0.846 | -0.0176 (0.0026) | 1.8E-11 | 0.0220 (0.0112) | 0.049 | 45.8 | -1.250 |
| Appendicular lean mass | Alzheimer's disease (Bellenguez 2022) | rs6923230 | 6:88213949 | C/T | 0.534 | -0.0127 (0.0019) | 1.8E-11 | -0.0037 (0.0081) | 0.651 | 44.7 | 0.291 |
| Appendicular lean mass | Alzheimer's disease (Bellenguez 2022) | rs10461725 | 5:39437129 | C/G | 0.656 | 0.0134 (0.0020) | 2.0E-11 | 0.0034 (0.0087) | 0.697 | 44.9 | 0.254 |
| Appendicular lean mass | Alzheimer's disease (Bellenguez 2022) | rs501250 | 5:112155793 | C/G | 0.619 | -0.0130 (0.0019) | 2.1E-11 | 0.0073 (0.0084) | 0.381 | 46.8 | -0.562 |
| Appendicular lean mass | Alzheimer's disease (Bellenguez 2022) | rs10840399 | 11:10399121 | T/C | 0.101 | -0.0209 (0.0031) | 2.8E-11 | -0.0021 (0.0134) | 0.878 | 45.5 | 0.100 |
| Appendicular lean mass | Alzheimer's disease (Bellenguez 2022) | rs7543202 | 1:73872885 | G/A | 0.623 | 0.0129 (0.0019) | 2.9E-11 | -0.0004 (0.0083) | 0.965 | 46.1 | -0.031 |
| Appendicular lean mass | Alzheimer's disease (Bellenguez 2022) | rs2678898 | 2:58171964 | C/T | 0.380 | -0.0129 (0.0019) | 3.1E-11 | 0.0030 (0.0085) | 0.724 | 46.1 | -0.233 |
| Appendicular lean mass | Alzheimer's disease (Bellenguez 2022) | rs2994329 | 1:243647921 | T/C | 0.202 | 0.0155 (0.0023) | 3.1E-11 | 0.0062 (0.0102) | 0.545 | 45.4 | 0.400 |
| Appendicular lean mass | Alzheimer's disease (Bellenguez 2022) | rs75702986 | 19:35566151 | A/G | 0.186 | -0.0163 (0.0025) | 3.1E-11 | 0.0242 (0.0117) | 0.038 | 42.5 | -1.485 |
| Appendicular lean mass | Alzheimer's disease (Bellenguez 2022) | rs2268718 | 6:52415023 | T/C | 0.270 | 0.0141 (0.0021) | 3.2E-11 | -0.0009 (0.0092) | 0.924 | 45.1 | -0.064 |
| Appendicular lean mass | Alzheimer's disease (Bellenguez 2022) | rs8084413 | 18:22869123 | A/G | 0.469 | -0.0127 (0.0019) | 3.2E-11 | 0.0072 (0.0082) | 0.379 | 44.7 | -0.567 |
| Appendicular lean mass | Alzheimer's disease (Bellenguez 2022) | rs34522021 | 9:23350420 | T/C | 0.455 | 0.0126 (0.0019) | 3.4E-11 | -0.0043 (0.0082) | 0.605 | 44.0 | -0.341 |
| Appendicular lean mass | Alzheimer's disease (Bellenguez 2022) | rs137998108 | 12:124322727 | CA/C | 0.133 | -0.0188 (0.0028) | 3.6E-11 | 0.0035 (0.0125) | 0.780 | 45.1 | -0.186 |
| Appendicular lean mass | Alzheimer's disease (Bellenguez 2022) | rs10827415 | 10:35042616 | G/T | 0.261 | -0.0142 (0.0021) | 3.9E-11 | 0.0165 (0.0091) | 0.070 | 45.7 | -1.162 |
| Appendicular lean mass | Alzheimer's disease (Bellenguez 2022) | rs116008080 | 16:67254841 | A/G | 0.024 | -0.0415 (0.0063) | 4.1E-11 | 0.0493 (0.0265) | 0.063 | 43.4 | -1.188 |
| Appendicular lean mass | Alzheimer's disease (Bellenguez 2022) | rs6738207 | 2:105989716 | A/G | 0.401 | 0.0127 (0.0019) | 4.1E-11 | -0.0008 (0.0083) | 0.922 | 44.7 | -0.063 |
| Appendicular lean mass | Alzheimer's disease (Bellenguez 2022) | rs113671109 | 3:12620885 | C/T | 0.220 | -0.0150 (0.0023) | 4.2E-11 | -0.0091 (0.0099) | 0.358 | 42.5 | 0.607 |
| Appendicular lean mass | Alzheimer's disease (Bellenguez 2022) | rs2965074 | 7:45983129 | T/G | 0.452 | 0.0125 (0.0019) | 4.3E-11 | -0.0071 (0.0082) | 0.386 | 43.3 | -0.568 |
| Appendicular lean mass | Alzheimer's disease (Bellenguez 2022) | rs1800504 | 7:50742180 | T/C | 0.552 | -0.0125 (0.0019) | 4.4E-11 | 0.0026 (0.0082) | 0.746 | 43.3 | -0.208 |
| Appendicular lean mass | Alzheimer's disease (Bellenguez 2022) | rs61397287 | 2:144223279 | T/A | 0.075 | 0.0235 (0.0036) | 4.5E-11 | 0.0047 (0.0162) | 0.772 | 42.6 | 0.200 |
| Appendicular lean mass | Alzheimer's disease (Bellenguez 2022) | rs34203781 | 6:41666292 | C/A | 0.137 | 0.0181 (0.0028) | 5.6E-11 | -0.0070 (0.0119) | 0.559 | 41.8 | -0.387 |
| Appendicular lean mass | Alzheimer's disease (Bellenguez 2022) | rs2347808 | 18:2750856 | A/G | 0.514 | -0.0125 (0.0019) | 5.8E-11 | 0.0039 (0.0082) | 0.632 | 43.3 | -0.312 |
| Appendicular lean mass | Alzheimer's disease (Bellenguez 2022) | rs6000886 | 22:38176670 | C/T | 0.648 | 0.0131 (0.0020) | 5.9E-11 | -0.0111 (0.0086) | 0.197 | 42.9 | -0.847 |
| Appendicular lean mass | Alzheimer's disease (Bellenguez 2022) | rs3830008 | 11:117093764 | C/A | 0.204 | -0.0154 (0.0023) | 6.1E-11 | 0.0019 (0.0101) | 0.848 | 44.8 | -0.123 |
| Appendicular lean mass | Alzheimer's disease (Bellenguez 2022) | rs2490302 | 10:37702435 | A/T | 0.914 | 0.0221 (0.0034) | 6.2E-11 | 0.0082 (0.0146) | 0.577 | 42.2 | 0.371 |
| Appendicular lean mass | Alzheimer's disease (Bellenguez 2022) | rs757042 | 7:25662829 | C/T | 0.724 | -0.0140 (0.0021) | 6.3E-11 | -0.0012 (0.0091) | 0.893 | 44.4 | 0.086 |
| Appendicular lean mass | Alzheimer's disease (Bellenguez 2022) | rs8180765 | 7:4680614 | G/A | 0.217 | -0.0151 (0.0023) | 6.5E-11 | -0.0011 (0.0103) | 0.919 | 43.1 | 0.073 |
| Appendicular lean mass | Alzheimer's disease (Bellenguez 2022) | rs11605297 | 11:58296806 | A/G | 0.233 | 0.0146 (0.0022) | 8.0E-11 | 0.0096 (0.0095) | 0.311 | 44.0 | 0.658 |
| Appendicular lean mass | Alzheimer's disease (Bellenguez 2022) | rs496783 | 10:116137961 | G/A | 0.465 | -0.0124 (0.0019) | 8.1E-11 | -0.0083 (0.0082) | 0.311 | 42.6 | 0.669 |
| Appendicular lean mass | Alzheimer's disease (Bellenguez 2022) | rs603486 | 11:118587347 | C/T | 0.617 | 0.0127 (0.0020) | 8.8E-11 | -0.0073 (0.0084) | 0.385 | 40.3 | -0.575 |
| Appendicular lean mass | Alzheimer's disease (Bellenguez 2022) | rs1899040 | 2:223901896 | T/C | 0.796 | 0.0152 (0.0023) | 9.0E-11 | -0.0098 (0.0100) | 0.329 | 43.7 | -0.645 |
| Appendicular lean mass | Alzheimer's disease (Bellenguez 2022) | rs35874463 | 15:67457698 | G/A | 0.058 | 0.0263 (0.0041) | 9.1E-11 | 0.0133 (0.0188) | 0.478 | 41.1 | 0.506 |
| Appendicular lean mass | Alzheimer's disease (Bellenguez 2022) | rs13109280 | 4:54380513 | G/A | 0.662 | 0.0131 (0.0020) | 9.1E-11 | 0.0073 (0.0087) | 0.400 | 42.9 | 0.557 |
| Appendicular lean mass | Alzheimer's disease (Bellenguez 2022) | rs13127468 | 4:8599658 | A/C | 0.463 | -0.0123 (0.0019) | 9.9E-11 | -0.0090 (0.0084) | 0.282 | 41.9 | 0.732 |
| Appendicular lean mass | Alzheimer's disease (Bellenguez 2022) | rs8064946 | 17:7589311 | C/G | 0.110 | -0.0195 (0.0030) | 1.0E-10 | 0.0009 (0.0127) | 0.943 | 42.2 | -0.046 |
| Appendicular lean mass | Alzheimer's disease (Bellenguez 2022) | rs10815304 | 9:5908217 | C/T | 0.206 | 0.0151 (0.0023) | 1.1E-10 | 0.0142 (0.0099) | 0.150 | 43.1 | 0.940 |
| Appendicular lean mass | Alzheimer's disease (Bellenguez 2022) | rs56112295 | 14:105877057 | T/C | 0.226 | 0.0154 (0.0024) | 1.1E-10 | -0.0104 (0.0107) | 0.332 | 41.2 | -0.675 |
| Appendicular lean mass | Alzheimer's disease (Bellenguez 2022) | rs11221657 | 11:129181358 | G/T | 0.135 | 0.0179 (0.0028) | 1.2E-10 | 0.0012 (0.0121) | 0.923 | 40.9 | 0.067 |
| Appendicular lean mass | Alzheimer's disease (Bellenguez 2022) | rs10019221 | 4:21785364 | T/G | 0.597 | -0.0124 (0.0019) | 1.2E-10 | 0.0024 (0.0084) | 0.777 | 42.6 | -0.194 |
| Appendicular lean mass | Alzheimer's disease (Bellenguez 2022) | rs55717234 | 1:150999863 | G/A | 0.573 | 0.0122 (0.0019) | 1.3E-10 | -0.0099 (0.0084) | 0.242 | 41.2 | -0.811 |
| Appendicular lean mass | Alzheimer's disease (Bellenguez 2022) | rs1405227 | 1:98873390 | A/G | 0.317 | 0.0129 (0.0020) | 1.6E-10 | 0.0053 (0.0088) | 0.544 | 41.6 | 0.411 |
| Appendicular lean mass | Alzheimer's disease (Bellenguez 2022) | rs10116740 | 9:133446357 | A/G | 0.477 | -0.0121 (0.0019) | 1.8E-10 | 0.0108 (0.0081) | 0.184 | 40.6 | -0.893 |
| Appendicular lean mass | Alzheimer's disease (Bellenguez 2022) | rs111622870 | 4:2613109 | C/T | 0.048 | -0.0282 (0.0044) | 1.9E-10 | -0.0136 (0.0198) | 0.492 | 41.1 | 0.482 |
| Appendicular lean mass | Alzheimer's disease (Bellenguez 2022) | rs650508 | 2:45880122 | C/G | 0.301 | -0.0130 (0.0020) | 1.9E-10 | -0.0178 (0.0090) | 0.047 | 42.2 | 1.369 |
| Appendicular lean mass | Alzheimer's disease (Bellenguez 2022) | rs6054491 | 20:6709535 | G/C | 0.240 | -0.0142 (0.0022) | 2.2E-10 | 0.0033 (0.0096) | 0.733 | 41.7 | -0.232 |
| Appendicular lean mass | Alzheimer's disease (Bellenguez 2022) | rs79680939 | 17:80523375 | A/G | 0.045 | 0.0290 (0.0046) | 2.5E-10 | -0.0393 (0.0215) | 0.067 | 39.7 | -1.355 |
| Appendicular lean mass | Alzheimer's disease (Bellenguez 2022) | rs56207600 | 11:126196537 | A/G | 0.111 | 0.0192 (0.0030) | 2.5E-10 | -0.0104 (0.0133) | 0.436 | 41.0 | -0.542 |
| Appendicular lean mass | Alzheimer's disease (Bellenguez 2022) | rs9828525 | 3:61552810 | T/C | 0.411 | 0.0121 (0.0019) | 2.5E-10 | -0.0186 (0.0083) | 0.025 | 40.6 | -1.537 |
| Appendicular lean mass | Alzheimer's disease (Bellenguez 2022) | rs4682483 | 3:112993982 | A/G | 0.153 | -0.0165 (0.0026) | 2.7E-10 | -0.0073 (0.0113) | 0.518 | 40.3 | 0.442 |
| Appendicular lean mass | Alzheimer's disease (Bellenguez 2022) | rs113146332 | 17:42565977 | C/G | 0.038 | 0.0311 (0.0049) | 3.0E-10 | 0.0426 (0.0224) | 0.057 | 40.3 | 1.370 |
| Appendicular lean mass | Alzheimer's disease (Bellenguez 2022) | rs7030071 | 9:136951556 | G/A | 0.311 | -0.0130 (0.0021) | 3.1E-10 | -0.0094 (0.0092) | 0.308 | 38.3 | 0.723 |
| Appendicular lean mass | Alzheimer's disease (Bellenguez 2022) | rs11410717 | 7:116252956 | TG/T | 0.117 | 0.0185 (0.0029) | 3.1E-10 | 0.0008 (0.0131) | 0.954 | 40.7 | 0.043 |
| Appendicular lean mass | Alzheimer's disease (Bellenguez 2022) | rs77299262 | 3:187450067 | C/T | 0.067 | 0.0237 (0.0038) | 3.4E-10 | 0.0102 (0.0163) | 0.531 | 38.9 | 0.430 |
| Appendicular lean mass | Alzheimer's disease (Bellenguez 2022) | rs1407204 | 6:119289835 | A/C | 0.810 | -0.0151 (0.0024) | 3.5E-10 | -0.0154 (0.0104) | 0.140 | 39.6 | 1.020 |
| Appendicular lean mass | Alzheimer's disease (Bellenguez 2022) | rs11191208 | 10:103838497 | A/G | 0.206 | 0.0147 (0.0024) | 3.7E-10 | -0.0086 (0.0102) | 0.397 | 37.5 | -0.585 |
| Appendicular lean mass | Alzheimer's disease (Bellenguez 2022) | rs11684531 | 2:219835489 | G/A | 0.133 | -0.0172 (0.0028) | 4.2E-10 | 0.0093 (0.0134) | 0.487 | 37.7 | -0.541 |
| Appendicular lean mass | Alzheimer's disease (Bellenguez 2022) | rs2823990 | 21:18081151 | A/G | 0.677 | -0.0127 (0.0020) | 4.2E-10 | 0.0019 (0.0087) | 0.832 | 40.3 | -0.150 |
| Appendicular lean mass | Alzheimer's disease (Bellenguez 2022) | rs117203652 | 19:49857801 | A/G | 0.030 | -0.0346 (0.0055) | 4.3E-10 | -0.0033 (0.0271) | 0.903 | 39.6 | 0.095 |
| Appendicular lean mass | Alzheimer's disease (Bellenguez 2022) | rs3792819 | 5:172576296 | G/A | 0.085 | 0.0210 (0.0034) | 4.4E-10 | 0.0151 (0.0137) | 0.271 | 38.1 | 0.719 |
| Appendicular lean mass | Alzheimer's disease (Bellenguez 2022) | rs10762866 | 10:81228430 | A/G | 0.138 | 0.0171 (0.0028) | 5.4E-10 | -0.0109 (0.0116) | 0.348 | 37.3 | -0.637 |
| Appendicular lean mass | Alzheimer's disease (Bellenguez 2022) | rs7359097 | 14:54757497 | C/T | 0.396 | -0.0121 (0.0019) | 5.5E-10 | -0.0005 (0.0084) | 0.948 | 40.6 | 0.041 |
| Appendicular lean mass | Alzheimer's disease (Bellenguez 2022) | rs10112506 | 8:13164746 | G/A | 0.390 | -0.0120 (0.0019) | 5.8E-10 | -0.0131 (0.0083) | 0.115 | 39.9 | 1.092 |
| Appendicular lean mass | Alzheimer's disease (Bellenguez 2022) | rs2974337 | 8:42390873 | T/C | 0.500 | 0.0117 (0.0019) | 5.9E-10 | 0.0222 (0.0081) | 0.007 | 37.9 | 1.897 |
| Appendicular lean mass | Alzheimer's disease (Bellenguez 2022) | rs11421589 | 10:115193949 | CT/C | 0.556 | 0.0119 (0.0019) | 6.0E-10 | 0.0114 (0.0084) | 0.177 | 39.2 | 0.958 |
| Appendicular lean mass | Alzheimer's disease (Bellenguez 2022) | rs7941305 | 11:28652116 | C/T | 0.313 | -0.0129 (0.0021) | 6.0E-10 | 0.0014 (0.0099) | 0.887 | 37.7 | -0.109 |
| Appendicular lean mass | Alzheimer's disease (Bellenguez 2022) | rs7144307 | 14:69533837 | C/T | 0.385 | -0.0122 (0.0020) | 6.1E-10 | -0.0236 (0.0085) | 0.005 | 37.2 | 1.934 |
| Appendicular lean mass | Alzheimer's disease (Bellenguez 2022) | rs4754296 | 11:108006182 | G/T | 0.146 | -0.0167 (0.0027) | 6.1E-10 | 0.0345 (0.0115) | 0.003 | 38.3 | -2.066 |
| Appendicular lean mass | Alzheimer's disease (Bellenguez 2022) | rs139921635 | 4:73181637 | T/G | 0.024 | 0.0385 (0.0062) | 6.2E-10 | 0.0234 (0.0305) | 0.444 | 38.6 | 0.608 |
| Appendicular lean mass | Alzheimer's disease (Bellenguez 2022) | rs10241451 | 7:55248926 | C/T | 0.209 | 0.0145 (0.0023) | 6.3E-10 | 0.0090 (0.0100) | 0.369 | 39.7 | 0.621 |
| Appendicular lean mass | Alzheimer's disease (Bellenguez 2022) | rs2875409 | 13:41112849 | A/G | 0.050 | -0.0270 (0.0044) | 7.1E-10 | -0.0240 (0.0177) | 0.176 | 37.6 | 0.889 |
| Appendicular lean mass | Alzheimer's disease (Bellenguez 2022) | rs839255 | 3:57974580 | G/T | 0.685 | -0.0126 (0.0021) | 8.4E-10 | 0.0060 (0.0089) | 0.501 | 36.0 | -0.476 |
| Appendicular lean mass | Alzheimer's disease (Bellenguez 2022) | rs10849576 | 12:993652 | G/T | 0.410 | -0.0117 (0.0019) | 9.2E-10 | -0.0137 (0.0083) | 0.097 | 37.9 | 1.171 |
| Appendicular lean mass | Alzheimer's disease (Bellenguez 2022) | rs11688284 | 2:174738951 | T/C | 0.388 | -0.0119 (0.0019) | 9.8E-10 | 0.0198 (0.0084) | 0.018 | 39.2 | -1.664 |
| Appendicular lean mass | Alzheimer's disease (Bellenguez 2022) | rs116339650 | 4:26200972 | G/A | 0.127 | -0.0175 (0.0029) | 1.1E-09 | -0.0003 (0.0121) | 0.981 | 36.4 | 0.017 |
| Appendicular lean mass | Alzheimer's disease (Bellenguez 2022) | rs670129 | 20:10460858 | T/C | 0.413 | -0.0118 (0.0019) | 1.1E-09 | -0.0004 (0.0084) | 0.964 | 38.6 | 0.034 |
| Appendicular lean mass | Alzheimer's disease (Bellenguez 2022) | rs7328187 | 13:74189974 | G/T | 0.498 | 0.0116 (0.0019) | 1.2E-09 | -0.0052 (0.0082) | 0.525 | 37.3 | -0.448 |
| Appendicular lean mass | Alzheimer's disease (Bellenguez 2022) | rs62143873 | 2:72035050 | A/G | 0.503 | -0.0115 (0.0019) | 1.2E-09 | -0.0082 (0.0083) | 0.326 | 36.6 | 0.713 |
| Appendicular lean mass | Alzheimer's disease (Bellenguez 2022) | rs6469845 | 8:120792195 | T/C | 0.239 | 0.0135 (0.0022) | 1.2E-09 | 0.0206 (0.0095) | 0.030 | 37.6 | 1.526 |
| Appendicular lean mass | Alzheimer's disease (Bellenguez 2022) | rs4940874 | 18:57105638 | G/A | 0.812 | 0.0148 (0.0024) | 1.2E-09 | -0.0027 (0.0104) | 0.797 | 38.0 | -0.182 |
| Appendicular lean mass | Alzheimer's disease (Bellenguez 2022) | rs75172776 | 16:9001814 | G/A | 0.066 | 0.0232 (0.0038) | 1.3E-09 | 0.0232 (0.0166) | 0.162 | 37.3 | 1.000 |
| Appendicular lean mass | Alzheimer's disease (Bellenguez 2022) | rs61925210 | 12:43974800 | T/A | 0.105 | 0.0187 (0.0031) | 1.3E-09 | -0.0021 (0.0130) | 0.870 | 36.4 | -0.112 |
| Appendicular lean mass | Alzheimer's disease (Bellenguez 2022) | rs4932439 | 15:89401109 | G/A | 0.825 | -0.0151 (0.0025) | 1.4E-09 | 0.0019 (0.0107) | 0.861 | 36.5 | -0.126 |
| Appendicular lean mass | Alzheimer's disease (Bellenguez 2022) | rs249681 | 5:141538017 | C/G | 0.618 | -0.0117 (0.0019) | 1.5E-09 | -0.0185 (0.0084) | 0.028 | 37.9 | 1.581 |
| Appendicular lean mass | Alzheimer's disease (Bellenguez 2022) | rs616488 | 1:10566215 | G/A | 0.345 | 0.0120 (0.0020) | 1.5E-09 | 0.0078 (0.0086) | 0.364 | 36.0 | 0.650 |
| Appendicular lean mass | Alzheimer's disease (Bellenguez 2022) | rs77013652 | 13:51142279 | G/T | 0.015 | 0.0490 (0.0081) | 1.5E-09 | -0.0009 (0.0347) | 0.979 | 36.6 | -0.018 |
| Appendicular lean mass | Alzheimer's disease (Bellenguez 2022) | rs17773965 | 2:217631338 | T/C | 0.141 | -0.0163 (0.0027) | 1.5E-09 | 0.0122 (0.0121) | 0.313 | 36.5 | -0.748 |
| Appendicular lean mass | Alzheimer's disease (Bellenguez 2022) | rs4554207 | 5:114388634 | C/T | 0.515 | -0.0114 (0.0019) | 1.6E-09 | 0.0008 (0.0081) | 0.924 | 36.0 | -0.070 |
| Appendicular lean mass | Alzheimer's disease (Bellenguez 2022) | rs1190540 | 14:102897009 | G/A | 0.698 | 0.0125 (0.0021) | 1.6E-09 | -0.0130 (0.0089) | 0.144 | 35.4 | -1.040 |
| Appendicular lean mass | Alzheimer's disease (Bellenguez 2022) | rs1202186 | 7:87213258 | T/C | 0.655 | -0.0120 (0.0020) | 1.7E-09 | -0.0086 (0.0085) | 0.313 | 36.0 | 0.717 |
| Appendicular lean mass | Alzheimer's disease (Bellenguez 2022) | rs62033029 | 16:50107273 | A/G | 0.206 | -0.0141 (0.0023) | 1.7E-09 | -0.0078 (0.0100) | 0.434 | 37.6 | 0.553 |
| Appendicular lean mass | Alzheimer's disease (Bellenguez 2022) | rs13112742 | 4:124888059 | A/G | 0.170 | -0.0151 (0.0025) | 2.0E-09 | 0.0080 (0.0109) | 0.463 | 36.5 | -0.530 |
| Appendicular lean mass | Alzheimer's disease (Bellenguez 2022) | rs680882 | 7:18325278 | G/T | 0.761 | 0.0133 (0.0022) | 2.0E-09 | -0.0050 (0.0094) | 0.597 | 36.5 | -0.376 |
| Appendicular lean mass | Alzheimer's disease (Bellenguez 2022) | rs631312 | 3:39508968 | A/G | 0.723 | -0.0126 (0.0021) | 2.1E-09 | -0.0010 (0.0090) | 0.908 | 36.0 | 0.079 |
| Appendicular lean mass | Alzheimer's disease (Bellenguez 2022) | rs2569888 | 6:1625803 | A/G | 0.245 | 0.0133 (0.0022) | 2.3E-09 | -0.0066 (0.0095) | 0.488 | 36.5 | -0.496 |
| Appendicular lean mass | Alzheimer's disease (Bellenguez 2022) | rs78030362 | 19:18575193 | G/A | 0.075 | 0.0216 (0.0036) | 2.5E-09 | -0.0206 (0.0169) | 0.224 | 36.0 | -0.954 |
| Appendicular lean mass | Alzheimer's disease (Bellenguez 2022) | rs10779958 | 2:74755778 | C/A | 0.149 | 0.0157 (0.0026) | 2.6E-09 | -0.0183 (0.0113) | 0.105 | 36.5 | -1.166 |
| Appendicular lean mass | Alzheimer's disease (Bellenguez 2022) | rs66613683 | 12:104409025 | C/T | 0.170 | 0.0150 (0.0025) | 2.6E-09 | -0.0019 (0.0110) | 0.863 | 36.0 | -0.127 |
| Appendicular lean mass | Alzheimer's disease (Bellenguez 2022) | rs114018835 | 1:244274455 | T/C | 0.036 | 0.0303 (0.0051) | 2.7E-09 | -0.0220 (0.0236) | 0.350 | 35.3 | -0.726 |
| Appendicular lean mass | Alzheimer's disease (Bellenguez 2022) | rs150474185 | 1:65689032 | G/C | 0.021 | 0.0440 (0.0074) | 2.7E-09 | -0.0596 (0.0309) | 0.054 | 35.4 | -1.355 |
| Appendicular lean mass | Alzheimer's disease (Bellenguez 2022) | rs114192718 | 2:128785663 | T/C | 0.075 | -0.0215 (0.0036) | 2.9E-09 | 0.0087 (0.0155) | 0.575 | 35.7 | -0.405 |
| Appendicular lean mass | Alzheimer's disease (Bellenguez 2022) | rs17713523 | 5:149923968 | A/G | 0.407 | 0.0114 (0.0019) | 3.1E-09 | -0.0163 (0.0083) | 0.050 | 36.0 | -1.430 |
| Appendicular lean mass | Alzheimer's disease (Bellenguez 2022) | rs11175919 | 12:66180277 | A/G | 0.026 | 0.0349 (0.0059) | 3.2E-09 | -0.0218 (0.0256) | 0.394 | 35.0 | -0.625 |
| Appendicular lean mass | Alzheimer's disease (Bellenguez 2022) | rs3118124 | 1:150166675 | G/A | 0.289 | -0.0122 (0.0021) | 3.2E-09 | 0.0137 (0.0091) | 0.131 | 33.8 | -1.123 |
| Appendicular lean mass | Alzheimer's disease (Bellenguez 2022) | rs78838233 | 17:18257589 | T/C | 0.237 | -0.0132 (0.0022) | 3.3E-09 | 0.0096 (0.0095) | 0.310 | 36.0 | -0.727 |
| Appendicular lean mass | Alzheimer's disease (Bellenguez 2022) | rs3769885 | 2:165592390 | A/G | 0.490 | -0.0111 (0.0019) | 3.5E-09 | -0.0007 (0.0081) | 0.933 | 34.1 | 0.063 |
| Appendicular lean mass | Alzheimer's disease (Bellenguez 2022) | rs3003137 | 20:62830802 | T/G | 0.428 | -0.0114 (0.0019) | 3.6E-09 | -0.0004 (0.0084) | 0.963 | 36.0 | 0.035 |
| Appendicular lean mass | Alzheimer's disease (Bellenguez 2022) | rs13316 | 14:93407301 | A/C | 0.430 | 0.0115 (0.0019) | 3.7E-09 | 0.0013 (0.0084) | 0.879 | 36.6 | 0.113 |
| Appendicular lean mass | Alzheimer's disease (Bellenguez 2022) | rs2788213 | 6:703249 | A/G | 0.710 | 0.0123 (0.0021) | 3.8E-09 | 0.0132 (0.0088) | 0.135 | 34.3 | 1.073 |
| Appendicular lean mass | Alzheimer's disease (Bellenguez 2022) | rs7095087 | 10:31120513 | A/G | 0.353 | 0.0117 (0.0020) | 3.8E-09 | -0.0180 (0.0086) | 0.036 | 34.2 | -1.538 |
| Appendicular lean mass | Alzheimer's disease (Bellenguez 2022) | rs603542 | 1:44079411 | C/T | 0.675 | 0.0118 (0.0020) | 3.8E-09 | -0.0028 (0.0086) | 0.744 | 34.8 | -0.237 |
| Appendicular lean mass | Alzheimer's disease (Bellenguez 2022) | rs290793 | 7:130416726 | C/G | 0.188 | 0.0142 (0.0024) | 4.0E-09 | 0.0074 (0.0105) | 0.477 | 35.0 | 0.521 |
| Appendicular lean mass | Alzheimer's disease (Bellenguez 2022) | rs41298373 | 10:7622009 | A/G | 0.101 | 0.0185 (0.0031) | 4.2E-09 | -0.0205 (0.0144) | 0.156 | 35.6 | -1.108 |
| Appendicular lean mass | Alzheimer's disease (Bellenguez 2022) | rs4836544 | 9:129236355 | T/C | 0.885 | 0.0175 (0.0030) | 4.3E-09 | -0.0312 (0.0128) | 0.015 | 34.0 | -1.783 |
| Appendicular lean mass | Alzheimer's disease (Bellenguez 2022) | rs75508358 | 9:96926382 | T/C | 0.046 | 0.0266 (0.0045) | 4.3E-09 | 0.0245 (0.0199) | 0.217 | 34.9 | 0.921 |
| Appendicular lean mass | Alzheimer's disease (Bellenguez 2022) | rs75100513 | 8:135670615 | C/T | 0.086 | 0.0199 (0.0034) | 4.4E-09 | 0.0064 (0.0146) | 0.659 | 34.3 | 0.322 |
| Appendicular lean mass | Alzheimer's disease (Bellenguez 2022) | rs4121583 | 18:125075 | T/C | 0.619 | 0.0118 (0.0020) | 4.6E-09 | 0.0043 (0.0090) | 0.635 | 34.8 | 0.364 |
| Appendicular lean mass | Alzheimer's disease (Bellenguez 2022) | rs113827862 | 14:89849527 | C/T | 0.061 | -0.0235 (0.0040) | 4.7E-09 | 0.0127 (0.0173) | 0.463 | 34.5 | -0.540 |
| Appendicular lean mass | Alzheimer's disease (Bellenguez 2022) | rs2150692 | 13:30109494 | A/C | 0.855 | -0.0158 (0.0027) | 4.8E-09 | 0.0127 (0.0117) | 0.277 | 34.2 | -0.804 |
| Appendicular lean mass | Alzheimer's disease (Bellenguez 2022) | rs9636364 | 2:111992435 | A/G | 0.543 | 0.0110 (0.0019) | 5.1E-09 | 0.0120 (0.0082) | 0.142 | 33.5 | 1.091 |
| Appendicular lean mass | Alzheimer's disease (Bellenguez 2022) | rs577289 | 15:40208911 | T/A | 0.281 | -0.0125 (0.0021) | 5.4E-09 | 0.0021 (0.0092) | 0.823 | 35.4 | -0.168 |
| Appendicular lean mass | Alzheimer's disease (Bellenguez 2022) | rs117972846 | 17:26947476 | G/T | 0.029 | 0.0335 (0.0057) | 5.5E-09 | 0.0603 (0.0262) | 0.021 | 34.5 | 1.800 |
| Appendicular lean mass | Alzheimer's disease (Bellenguez 2022) | rs4652902 | 1:36612955 | A/C | 0.768 | 0.0129 (0.0022) | 5.7E-09 | 0.0060 (0.0096) | 0.530 | 34.4 | 0.465 |
| Appendicular lean mass | Alzheimer's disease (Bellenguez 2022) | rs10060855 | 5:33216769 | G/A | 0.677 | 0.0118 (0.0020) | 6.2E-09 | -0.0015 (0.0087) | 0.862 | 34.8 | -0.127 |
| Appendicular lean mass | Alzheimer's disease (Bellenguez 2022) | rs1527149 | 7:114004088 | G/C | 0.407 | 0.0113 (0.0019) | 6.9E-09 | 0.0140 (0.0083) | 0.091 | 35.4 | 1.239 |
| Appendicular lean mass | Alzheimer's disease (Bellenguez 2022) | rs11132166 | 4:184049867 | C/T | 0.036 | -0.0292 (0.0050) | 7.6E-09 | -0.0150 (0.0219) | 0.494 | 34.1 | 0.514 |
| Appendicular lean mass | Alzheimer's disease (Bellenguez 2022) | rs34338597 | 12:106301580 | G/A | 0.383 | -0.0112 (0.0019) | 7.9E-09 | 0.0003 (0.0084) | 0.968 | 34.8 | -0.027 |
| Appendicular lean mass | Alzheimer's disease (Bellenguez 2022) | rs4849904 | 2:121336556 | G/C | 0.380 | 0.0111 (0.0019) | 8.1E-09 | -0.0130 (0.0085) | 0.126 | 34.1 | -1.171 |
| Appendicular lean mass | Alzheimer's disease (Bellenguez 2022) | rs612577 | 1:230324364 | C/T | 0.149 | -0.0152 (0.0026) | 8.2E-09 | -0.0167 (0.0115) | 0.147 | 34.2 | 1.099 |
| Appendicular lean mass | Alzheimer's disease (Bellenguez 2022) | rs111925803 | 22:50719976 | T/C | 0.285 | 0.0122 (0.0021) | 8.6E-09 | -0.0080 (0.0092) | 0.385 | 33.8 | -0.656 |
| Appendicular lean mass | Alzheimer's disease (Bellenguez 2022) | rs921142 | 8:41169276 | C/T | 0.398 | -0.0111 (0.0019) | 8.7E-09 | 0.0077 (0.0084) | 0.364 | 34.1 | -0.694 |
| Appendicular lean mass | Alzheimer's disease (Bellenguez 2022) | rs10099846 | 8:22534178 | T/C | 0.668 | 0.0116 (0.0020) | 8.8E-09 | -0.0007 (0.0086) | 0.931 | 33.6 | -0.060 |
| Appendicular lean mass | Alzheimer's disease (Bellenguez 2022) | rs7744700 | 6:53349401 | A/T | 0.291 | -0.0122 (0.0021) | 9.2E-09 | 0.0132 (0.0094) | 0.161 | 33.8 | -1.082 |
| Appendicular lean mass | Alzheimer's disease (Bellenguez 2022) | rs12483401 | 21:35443829 | C/T | 0.022 | -0.0387 (0.0067) | 9.2E-09 | 0.0078 (0.0292) | 0.789 | 33.4 | -0.202 |
| Appendicular lean mass | Alzheimer's disease (Bellenguez 2022) | rs68185109 | 10:93648306 | A/G | 0.102 | 0.0180 (0.0031) | 9.6E-09 | 0.0241 (0.0134) | 0.072 | 33.7 | 1.339 |
| Appendicular lean mass | Alzheimer's disease (Bellenguez 2022) | rs28736838 | 1:120148713 | T/C | 0.302 | -0.0117 (0.0020) | 1.1E-08 | -0.0081 (0.0089) | 0.363 | 34.2 | 0.692 |
| Appendicular lean mass | Alzheimer's disease (Bellenguez 2022) | rs2208404 | 20:55843736 | G/A | 0.416 | -0.0111 (0.0019) | 1.1E-08 | 0.0140 (0.0082) | 0.090 | 34.1 | -1.261 |
| Appendicular lean mass | Alzheimer's disease (Bellenguez 2022) | rs8018486 | 14:39818616 | G/A | 0.191 | -0.0138 (0.0024) | 1.2E-08 | -0.0051 (0.0101) | 0.616 | 33.1 | 0.370 |
| Appendicular lean mass | Alzheimer's disease (Bellenguez 2022) | rs10749157 | 10:115780129 | C/T | 0.358 | 0.0113 (0.0020) | 1.2E-08 | 0.0137 (0.0086) | 0.110 | 31.9 | 1.212 |
| Appendicular lean mass | Alzheimer's disease (Bellenguez 2022) | rs11070842 | 15:51624185 | C/T | 0.163 | -0.0146 (0.0026) | 1.3E-08 | 0.0073 (0.0108) | 0.501 | 31.5 | -0.500 |
| Appendicular lean mass | Alzheimer's disease (Bellenguez 2022) | rs62103240 | 18:77650637 | A/G | 0.071 | 0.0212 (0.0037) | 1.4E-08 | -0.0015 (0.0161) | 0.928 | 32.8 | -0.071 |
| Appendicular lean mass | Alzheimer's disease (Bellenguez 2022) | rs10222594 | 3:71554856 | A/G | 0.323 | 0.0115 (0.0020) | 1.4E-08 | -0.0123 (0.0087) | 0.159 | 33.1 | -1.070 |
| Appendicular lean mass | Alzheimer's disease (Bellenguez 2022) | rs56309431 | 17:63645289 | T/G | 0.116 | 0.0167 (0.0029) | 1.5E-08 | 0.0048 (0.0132) | 0.716 | 33.2 | 0.287 |
| Appendicular lean mass | Alzheimer's disease (Bellenguez 2022) | rs10764692 | 10:27576251 | C/A | 0.637 | -0.0111 (0.0020) | 1.6E-08 | 0.0018 (0.0085) | 0.836 | 30.8 | -0.162 |
| Appendicular lean mass | Alzheimer's disease (Bellenguez 2022) | rs10940169 | 5:67823682 | C/G | 0.382 | -0.0110 (0.0019) | 1.6E-08 | 0.0156 (0.0085) | 0.066 | 33.5 | -1.418 |
| Appendicular lean mass | Alzheimer's disease (Bellenguez 2022) | rs10221831 | 2:202107829 | T/C | 0.032 | 0.0300 (0.0053) | 1.8E-08 | -0.0217 (0.0221) | 0.325 | 32.0 | -0.723 |
| Appendicular lean mass | Alzheimer's disease (Bellenguez 2022) | rs4965298 | 15:100802766 | T/C | 0.713 | -0.0119 (0.0021) | 1.8E-08 | 0.0043 (0.0091) | 0.634 | 32.1 | -0.361 |
| Appendicular lean mass | Alzheimer's disease (Bellenguez 2022) | rs72726050 | 15:42270059 | C/T | 0.096 | -0.0192 (0.0034) | 1.8E-08 | 0.0070 (0.0144) | 0.628 | 31.9 | -0.365 |
| Appendicular lean mass | Alzheimer's disease (Bellenguez 2022) | rs28678024 | 14:25937161 | G/A | 0.285 | -0.0119 (0.0021) | 1.9E-08 | 0.0016 (0.0090) | 0.858 | 32.1 | -0.134 |
| Appendicular lean mass | Alzheimer's disease (Bellenguez 2022) | rs2188805 | 7:93078400 | C/A | 0.336 | 0.0114 (0.0020) | 2.0E-08 | 0.0027 (0.0088) | 0.762 | 32.5 | 0.237 |
| Appendicular lean mass | Alzheimer's disease (Bellenguez 2022) | rs9590328 | 13:96448383 | G/A | 0.142 | 0.0153 (0.0027) | 2.0E-08 | -0.0050 (0.0114) | 0.663 | 32.1 | -0.327 |
| Appendicular lean mass | Alzheimer's disease (Bellenguez 2022) | rs31196 | 5:158300798 | A/C | 0.572 | -0.0107 (0.0019) | 2.1E-08 | -0.0002 (0.0082) | 0.983 | 31.7 | 0.019 |
| Appendicular lean mass | Alzheimer's disease (Bellenguez 2022) | rs876122 | 6:6886297 | G/A | 0.879 | 0.0162 (0.0029) | 2.2E-08 | 0.0011 (0.0124) | 0.926 | 31.2 | 0.068 |
| Appendicular lean mass | Alzheimer's disease (Bellenguez 2022) | rs9288695 | 2:158641300 | C/G | 0.779 | 0.0127 (0.0023) | 2.2E-08 | 0.0038 (0.0099) | 0.698 | 30.5 | 0.299 |
| Appendicular lean mass | Alzheimer's disease (Bellenguez 2022) | rs7082659 | 10:12017584 | C/T | 0.866 | 0.0156 (0.0028) | 2.3E-08 | -0.0221 (0.0125) | 0.076 | 31.0 | -1.417 |
| Appendicular lean mass | Alzheimer's disease (Bellenguez 2022) | rs80280630 | 9:117030861 | T/C | 0.112 | -0.0168 (0.0030) | 2.3E-08 | -0.0004 (0.0130) | 0.973 | 31.4 | 0.024 |
| Appendicular lean mass | Alzheimer's disease (Bellenguez 2022) | rs74048171 | 11:2093603 | A/C | 0.258 | -0.0121 (0.0022) | 2.4E-08 | -0.0106 (0.0094) | 0.260 | 30.2 | 0.876 |
| Appendicular lean mass | Alzheimer's disease (Bellenguez 2022) | rs141277904 | 8:56654581 | T/C | 0.023 | 0.0384 (0.0069) | 2.4E-08 | 0.0204 (0.0307) | 0.507 | 31.0 | 0.531 |
| Appendicular lean mass | Alzheimer's disease (Bellenguez 2022) | rs4916786 | 5:89476946 | T/C | 0.196 | 0.0133 (0.0024) | 2.4E-08 | 0.0027 (0.0102) | 0.794 | 30.7 | 0.203 |
| Appendicular lean mass | Alzheimer's disease (Bellenguez 2022) | rs2303423 | 4:38120029 | C/T | 0.110 | 0.0168 (0.0030) | 2.6E-08 | 0.0262 (0.0127) | 0.040 | 31.4 | 1.560 |
| Appendicular lean mass | Alzheimer's disease (Bellenguez 2022) | rs149697773 | 17:60932424 | G/A | 0.045 | -0.0262 (0.0047) | 2.8E-08 | -0.0215 (0.0213) | 0.314 | 31.1 | 0.821 |
| Appendicular lean mass | Alzheimer's disease (Bellenguez 2022) | rs2978362 | 18:32959397 | T/C | 0.533 | 0.0106 (0.0019) | 2.8E-08 | 0.0037 (0.0083) | 0.650 | 31.1 | 0.349 |
| Appendicular lean mass | Alzheimer's disease (Bellenguez 2022) | rs4383083 | 15:63080442 | A/G | 0.647 | 0.0111 (0.0020) | 2.9E-08 | 0.0030 (0.0087) | 0.730 | 30.8 | 0.270 |
| Appendicular lean mass | Alzheimer's disease (Bellenguez 2022) | rs10940364 | 5:53487971 | C/T | 0.139 | 0.0151 (0.0027) | 3.1E-08 | -0.0117 (0.0115) | 0.310 | 31.3 | -0.775 |
| Appendicular lean mass | Alzheimer's disease (Bellenguez 2022) | rs10736029 | 12:46513442 | T/G | 0.910 | -0.0193 (0.0035) | 3.1E-08 | 0.0162 (0.0150) | 0.278 | 30.4 | -0.839 |
| Appendicular lean mass | Alzheimer's disease (Bellenguez 2022) | rs77293269 | 3:155595709 | C/T | 0.113 | -0.0165 (0.0030) | 3.1E-08 | -0.0007 (0.0132) | 0.959 | 30.2 | 0.042 |
| Appendicular lean mass | Alzheimer's disease (Bellenguez 2022) | rs62177315 | 2:161236992 | T/C | 0.096 | 0.0177 (0.0032) | 3.1E-08 | 0.0312 (0.0131) | 0.018 | 30.6 | 1.763 |
| Appendicular lean mass | Alzheimer's disease (Bellenguez 2022) | rs72809820 | 2:97360079 | T/C | 0.324 | -0.0111 (0.0020) | 3.1E-08 | 0.0048 (0.0088) | 0.587 | 30.8 | -0.432 |
| Appendicular lean mass | Alzheimer's disease (Bellenguez 2022) | rs9568031 | 13:48897520 | T/C | 0.706 | -0.0115 (0.0021) | 3.3E-08 | -0.0028 (0.0090) | 0.757 | 30.0 | 0.243 |
| Appendicular lean mass | Alzheimer's disease (Bellenguez 2022) | rs10821713 | 10:62055781 | T/C | 0.418 | 0.0106 (0.0019) | 3.4E-08 | -0.0020 (0.0083) | 0.809 | 31.1 | -0.189 |
| Appendicular lean mass | Alzheimer's disease (Bellenguez 2022) | rs12185775 | 20:20293769 | C/G | 0.117 | -0.0167 (0.0030) | 3.7E-08 | 0.0074 (0.0137) | 0.589 | 31.0 | -0.443 |
| Appendicular lean mass | Alzheimer's disease (Bellenguez 2022) | rs6461948 | 7:26540727 | T/C | 0.673 | 0.0112 (0.0020) | 4.0E-08 | -0.0125 (0.0087) | 0.150 | 31.4 | -1.116 |
| Appendicular lean mass | Alzheimer's disease (Bellenguez 2022) | rs11721522 | 4:156976051 | G/A | 0.412 | 0.0106 (0.0019) | 4.0E-08 | 0.0208 (0.0083) | 0.012 | 31.1 | 1.962 |
| Appendicular lean mass | Alzheimer's disease (Bellenguez 2022) | rs3002284 | 1:213181916 | C/T | 0.320 | -0.0110 (0.0020) | 4.1E-08 | 0.0170 (0.0088) | 0.052 | 30.2 | -1.545 |
| Appendicular lean mass | Alzheimer's disease (Bellenguez 2022) | rs190912824 | 8:67329931 | A/T | 0.022 | 0.0353 (0.0064) | 4.1E-08 | 0.0151 (0.0275) | 0.583 | 30.4 | 0.428 |
| Appendicular lean mass | Alzheimer's disease (Bellenguez 2022) | rs34856058 | 3:101365150 | G/A | 0.726 | 0.0116 (0.0021) | 4.4E-08 | 0.0084 (0.0091) | 0.355 | 30.5 | 0.724 |
| Appendicular lean mass | Alzheimer's disease (Bellenguez 2022) | rs1884066 | 14:67452834 | A/G | 0.150 | -0.0145 (0.0027) | 5.0E-08 | -0.0053 (0.0111) | 0.634 | 28.8 | 0.366 |
| Appendicular lean mass | All-cause dementia (FinnGen) | rs143384 | 20:34025756 | G/A | 0.404 | 0.0725 (0.0019) | 1.1E-308 | -0.0099 (0.0193) | 0.610 | 1456.0 | -0.137 |
| Appendicular lean mass | All-cause dementia (FinnGen) | rs6860245 | 5:127367998 | C/G | 0.248 | 0.0589 (0.0022) | 9.7E-160 | -0.0182 (0.0264) | 0.489 | 716.8 | -0.309 |
| Appendicular lean mass | All-cause dementia (FinnGen) | rs71190381 | 13:51120097 | GAGTGA/G | 0.214 | -0.0614 (0.0023) | 9.9E-155 | -0.0214 (0.0220) | 0.331 | 712.7 | 0.349 |
| Appendicular lean mass | All-cause dementia (FinnGen) | rs9853018 | 3:141101961 | T/C | 0.444 | 0.0469 (0.0019) | 2.4E-135 | 0.0504 (0.0192) | 0.009 | 609.3 | 1.075 |
| Appendicular lean mass | All-cause dementia (FinnGen) | rs1472852 | 4:17910236 | A/C | 0.158 | -0.0638 (0.0026) | 8.2E-135 | 0.0435 (0.0326) | 0.183 | 602.1 | -0.682 |
| Appendicular lean mass | All-cause dementia (FinnGen) | rs9388490 | 6:126704795 | T/C | 0.439 | 0.0462 (0.0019) | 1.3E-130 | 0.0322 (0.0192) | 0.093 | 591.3 | 0.697 |
| Appendicular lean mass | All-cause dementia (FinnGen) | rs2854152 | 17:61986027 | G/A | 0.678 | 0.0482 (0.0020) | 1.0E-124 | 0.0153 (0.0198) | 0.440 | 580.8 | 0.317 |
| Appendicular lean mass | All-cause dementia (FinnGen) | rs71519447 | 8:57157543 | A/G | 0.110 | -0.0716 (0.0030) | 3.9E-124 | 0.0699 (0.0304) | 0.021 | 569.6 | -0.976 |
| Appendicular lean mass | All-cause dementia (FinnGen) | rs6977416 | 7:150542711 | A/G | 0.334 | 0.0457 (0.0020) | 1.4E-113 | 0.0094 (0.0200) | 0.637 | 522.1 | 0.206 |
| Appendicular lean mass | All-cause dementia (FinnGen) | rs76895963 | 12:4384844 | G/T | 0.021 | 0.1639 (0.0073) | 8.2E-112 | -0.0735 (0.0577) | 0.203 | 504.1 | -0.448 |
| Appendicular lean mass | All-cause dementia (FinnGen) | rs42039 | 7:92244422 | T/C | 0.244 | 0.0481 (0.0022) | 3.5E-106 | 0.0088 (0.0226) | 0.698 | 478.0 | 0.183 |
| Appendicular lean mass | All-cause dementia (FinnGen) | rs6567160 | 18:57829135 | C/T | 0.233 | 0.0488 (0.0022) | 1.2E-104 | 0.0373 (0.0246) | 0.130 | 492.0 | 0.764 |
| Appendicular lean mass | All-cause dementia (FinnGen) | rs9634212 | 12:93993266 | A/C | 0.221 | 0.0471 (0.0023) | 8.6E-95 | 0.0014 (0.0221) | 0.950 | 419.4 | 0.030 |
| Appendicular lean mass | All-cause dementia (FinnGen) | rs11187838 | 10:96038686 | A/G | 0.435 | 0.0394 (0.0019) | 1.2E-94 | -0.0119 (0.0202) | 0.554 | 430.0 | -0.302 |
| Appendicular lean mass | All-cause dementia (FinnGen) | rs2524139 | 6:31264133 | C/G | 0.700 | -0.0423 (0.0020) | 1.5E-94 | -0.0159 (0.0242) | 0.512 | 447.3 | 0.376 |
| Appendicular lean mass | All-cause dementia (FinnGen) | rs28701981 | 9:98217581 | C/T | 0.349 | 0.0395 (0.0020) | 1.5E-87 | 0.0098 (0.0199) | 0.622 | 390.1 | 0.248 |
| Appendicular lean mass | All-cause dementia (FinnGen) | rs113459995 | 9:119122551 | GTCTGGGC/G | 0.201 | -0.0460 (0.0024) | 3.8E-84 | -0.0592 (0.0227) | 0.009 | 367.4 | 1.287 |
| Appendicular lean mass | All-cause dementia (FinnGen) | rs10483727 | 14:61072875 | C/T | 0.611 | -0.0368 (0.0019) | 6.7E-80 | 0.0392 (0.0211) | 0.063 | 375.1 | -1.065 |
| Appendicular lean mass | All-cause dementia (FinnGen) | rs1355603 | 4:145566477 | C/T | 0.831 | 0.0466 (0.0025) | 3.0E-76 | 0.0141 (0.0257) | 0.583 | 347.4 | 0.303 |
| Appendicular lean mass | All-cause dementia (FinnGen) | rs10807137 | 6:34183026 | T/C | 0.825 | -0.0455 (0.0025) | 5.7E-75 | 0.0378 (0.0276) | 0.171 | 331.2 | -0.831 |
| Appendicular lean mass | All-cause dementia (FinnGen) | rs55877758 | 2:172409030 | A/T | 0.241 | -0.0394 (0.0022) | 2.5E-72 | -0.0210 (0.0246) | 0.394 | 320.7 | 0.533 |
| Appendicular lean mass | All-cause dementia (FinnGen) | rs12713004 | 2:23896049 | G/A | 0.725 | 0.0367 (0.0021) | 2.4E-68 | -0.0115 (0.0250) | 0.647 | 305.4 | -0.313 |
| Appendicular lean mass | All-cause dementia (FinnGen) | rs798528 | 7:2772431 | C/A | 0.307 | -0.0357 (0.0020) | 5.6E-68 | 0.0118 (0.0198) | 0.551 | 318.6 | -0.331 |
| Appendicular lean mass | All-cause dementia (FinnGen) | rs1260326 | 2:27730940 | C/T | 0.605 | 0.0323 (0.0019) | 6.2E-64 | -0.0128 (0.0201) | 0.526 | 289.0 | -0.396 |
| Appendicular lean mass | All-cause dementia (FinnGen) | rs9905385 | 17:59498250 | G/A | 0.671 | -0.0339 (0.0020) | 1.9E-63 | -0.0011 (0.0215) | 0.960 | 287.3 | 0.032 |
| Appendicular lean mass | All-cause dementia (FinnGen) | rs3213141 | 20:32274380 | A/G | 0.266 | -0.0363 (0.0022) | 2.0E-63 | -0.0119 (0.0220) | 0.589 | 272.2 | 0.328 |
| Appendicular lean mass | All-cause dementia (FinnGen) | rs485554 | 3:172137343 | C/G | 0.315 | 0.0342 (0.0020) | 3.0E-63 | -0.0029 (0.0204) | 0.887 | 292.4 | -0.085 |
| Appendicular lean mass | All-cause dementia (FinnGen) | rs2871865 | 15:99194896 | G/C | 0.116 | -0.0493 (0.0030) | 3.4E-62 | 0.0616 (0.0398) | 0.122 | 270.1 | -1.249 |
| Appendicular lean mass | All-cause dementia (FinnGen) | rs117068593 | 14:93118229 | T/C | 0.190 | 0.0403 (0.0024) | 8.8E-62 | 0.0216 (0.0275) | 0.433 | 282.0 | 0.536 |
| Appendicular lean mass | All-cause dementia (FinnGen) | rs2885697 | 1:41544279 | T/G | 0.665 | -0.0323 (0.0020) | 9.2E-60 | 0.0256 (0.0202) | 0.206 | 260.8 | -0.793 |
| Appendicular lean mass | All-cause dementia (FinnGen) | rs12099669 | 12:46783653 | A/G | 0.696 | 0.0331 (0.0020) | 1.4E-58 | -0.0424 (0.0202) | 0.035 | 273.9 | -1.281 |
| Appendicular lean mass | All-cause dementia (FinnGen) | rs11243202 | 6:7719065 | C/T | 0.486 | 0.0302 (0.0019) | 2.8E-57 | 0.0058 (0.0192) | 0.764 | 252.6 | 0.192 |
| Appendicular lean mass | All-cause dementia (FinnGen) | rs4735761 | 8:78097161 | C/A | 0.286 | 0.0331 (0.0021) | 3.7E-56 | -0.0068 (0.0231) | 0.768 | 248.4 | -0.205 |
| Appendicular lean mass | All-cause dementia (FinnGen) | rs78378222 | 17:7571752 | G/T | 0.012 | 0.1380 (0.0087) | 4.5E-56 | -0.1299 (0.0749) | 0.083 | 251.6 | -0.941 |
| Appendicular lean mass | All-cause dementia (FinnGen) | rs2194411 | 3:185548663 | A/G | 0.128 | 0.0443 (0.0029) | 2.4E-54 | 0.0117 (0.0280) | 0.676 | 233.3 | 0.264 |
| Appendicular lean mass | All-cause dementia (FinnGen) | rs994533 | 2:218284278 | C/G | 0.316 | -0.0313 (0.0020) | 5.5E-54 | 0.0179 (0.0194) | 0.354 | 244.9 | -0.572 |
| Appendicular lean mass | All-cause dementia (FinnGen) | rs11042717 | 11:10303939 | C/T | 0.490 | -0.0290 (0.0019) | 4.0E-53 | -0.0129 (0.0191) | 0.501 | 233.0 | 0.445 |
| Appendicular lean mass | All-cause dementia (FinnGen) | rs7129320 | 11:68388220 | A/G | 0.166 | -0.0389 (0.0025) | 7.3E-53 | 0.0292 (0.0339) | 0.389 | 242.1 | -0.751 |
| Appendicular lean mass | All-cause dementia (FinnGen) | rs9894577 | 17:43223292 | A/G | 0.318 | -0.0310 (0.0020) | 1.4E-52 | 0.0013 (0.0203) | 0.948 | 240.2 | -0.042 |
| Appendicular lean mass | All-cause dementia (FinnGen) | rs146851424 | 13:50377910 | C/A | 0.022 | 0.1003 (0.0066) | 1.6E-52 | -0.0915 (0.0729) | 0.209 | 230.9 | -0.912 |
| Appendicular lean mass | All-cause dementia (FinnGen) | rs1325596 | 1:176794066 | A/G | 0.548 | 0.0287 (0.0019) | 2.8E-52 | -0.0095 (0.0192) | 0.622 | 228.2 | -0.331 |
| Appendicular lean mass | All-cause dementia (FinnGen) | rs72801818 | 16:53476042 | T/C | 0.300 | 0.0313 (0.0021) | 8.9E-52 | -0.0310 (0.0219) | 0.156 | 222.2 | -0.990 |
| Appendicular lean mass | All-cause dementia (FinnGen) | rs13103161 | 4:106216459 | A/T | 0.389 | -0.0284 (0.0019) | 2.4E-48 | -0.0080 (0.0202) | 0.693 | 223.4 | 0.282 |
| Appendicular lean mass | All-cause dementia (FinnGen) | rs34517439 | 1:78450517 | A/C | 0.122 | 0.0421 (0.0029) | 5.8E-48 | -0.0318 (0.0290) | 0.272 | 210.8 | -0.755 |
| Appendicular lean mass | All-cause dementia (FinnGen) | rs10491967 | 12:3368093 | A/G | 0.092 | 0.0471 (0.0032) | 1.3E-47 | 0.0039 (0.0357) | 0.914 | 216.6 | 0.083 |
| Appendicular lean mass | All-cause dementia (FinnGen) | rs723149 | 7:46577056 | G/A | 0.563 | -0.0276 (0.0019) | 1.4E-47 | -0.0060 (0.0193) | 0.756 | 211.0 | 0.217 |
| Appendicular lean mass | All-cause dementia (FinnGen) | rs4909912 | 8:135572297 | T/C | 0.427 | -0.0277 (0.0019) | 1.7E-47 | -0.0125 (0.0191) | 0.513 | 212.6 | 0.451 |
| Appendicular lean mass | All-cause dementia (FinnGen) | rs34776209 | 7:23513093 | T/C | 0.247 | -0.0317 (0.0022) | 1.8E-47 | -0.0124 (0.0236) | 0.599 | 207.6 | 0.391 |
| Appendicular lean mass | All-cause dementia (FinnGen) | rs41271299 | 6:19839415 | T/C | 0.051 | 0.0616 (0.0043) | 3.2E-47 | 0.0524 (0.0757) | 0.489 | 205.2 | 0.851 |
| Appendicular lean mass | All-cause dementia (FinnGen) | rs1319012 | 6:41852616 | A/T | 0.926 | -0.0520 (0.0037) | 3.3E-45 | -0.0361 (0.0322) | 0.263 | 197.5 | 0.694 |
| Appendicular lean mass | All-cause dementia (FinnGen) | rs7095472 | 10:70399109 | G/A | 0.533 | 0.0267 (0.0019) | 7.7E-45 | -0.0131 (0.0192) | 0.496 | 197.5 | -0.491 |
| Appendicular lean mass | All-cause dementia (FinnGen) | rs59985551 | 2:56106928 | T/C | 0.226 | -0.0313 (0.0022) | 2.4E-44 | 0.0390 (0.0229) | 0.088 | 202.4 | -1.246 |
| Appendicular lean mass | All-cause dementia (FinnGen) | rs10283100 | 8:120596023 | G/A | 0.945 | 0.0575 (0.0041) | 4.1E-44 | -0.0149 (0.0370) | 0.688 | 196.7 | -0.259 |
| Appendicular lean mass | All-cause dementia (FinnGen) | rs6931421 | 6:80880138 | G/T | 0.322 | -0.0279 (0.0020) | 2.3E-43 | -0.0274 (0.0210) | 0.192 | 194.6 | 0.982 |
| Appendicular lean mass | All-cause dementia (FinnGen) | rs17205463 | 15:62381413 | T/C | 0.448 | -0.0263 (0.0019) | 4.2E-43 | 0.0076 (0.0203) | 0.706 | 191.6 | -0.289 |
| Appendicular lean mass | All-cause dementia (FinnGen) | rs2270894 | 3:9975386 | G/C | 0.203 | -0.0332 (0.0024) | 1.3E-42 | -0.0117 (0.0218) | 0.591 | 191.4 | 0.352 |
| Appendicular lean mass | All-cause dementia (FinnGen) | rs4282339 | 5:168256240 | A/G | 0.207 | -0.0311 (0.0023) | 6.2E-41 | -0.0054 (0.0245) | 0.826 | 182.8 | 0.174 |
| Appendicular lean mass | All-cause dementia (FinnGen) | rs10883555 | 10:102657908 | T/C | 0.550 | 0.0254 (0.0019) | 9.1E-41 | 0.0099 (0.0193) | 0.606 | 178.7 | 0.390 |
| Appendicular lean mass | All-cause dementia (FinnGen) | rs12051245 | 16:783865 | C/T | 0.232 | 0.0299 (0.0022) | 2.6E-40 | 0.0103 (0.0202) | 0.612 | 184.7 | 0.344 |
| Appendicular lean mass | All-cause dementia (FinnGen) | rs11014285 | 10:25178864 | A/G | 0.165 | 0.0342 (0.0026) | 2.9E-40 | 0.0128 (0.0319) | 0.688 | 173.0 | 0.374 |
| Appendicular lean mass | All-cause dementia (FinnGen) | rs2229840 | 12:124826462 | T/C | 0.160 | 0.0341 (0.0026) | 3.0E-40 | -0.0160 (0.0270) | 0.552 | 172.0 | -0.469 |
| Appendicular lean mass | All-cause dementia (FinnGen) | rs35230100 | 14:70348141 | CATAT/C | 0.433 | -0.0262 (0.0020) | 4.4E-40 | -0.0143 (0.0195) | 0.463 | 171.6 | 0.546 |
| Appendicular lean mass | All-cause dementia (FinnGen) | rs35309034 | 8:126498265 | A/C | 0.238 | -0.0297 (0.0022) | 1.1E-39 | 0.0214 (0.0209) | 0.307 | 182.2 | -0.721 |
| Appendicular lean mass | All-cause dementia (FinnGen) | rs7952436 | 11:67024534 | T/C | 0.082 | -0.0453 (0.0034) | 1.6E-39 | 0.0165 (0.0437) | 0.705 | 177.5 | -0.364 |
| Appendicular lean mass | All-cause dementia (FinnGen) | rs8054549 | 16:86417234 | A/C | 0.449 | -0.0251 (0.0019) | 3.4E-39 | -0.0073 (0.0196) | 0.711 | 174.5 | 0.291 |
| Appendicular lean mass | All-cause dementia (FinnGen) | rs3764002 | 12:108618630 | T/C | 0.262 | 0.0280 (0.0021) | 4.5E-39 | -0.0040 (0.0201) | 0.843 | 177.8 | -0.143 |
| Appendicular lean mass | All-cause dementia (FinnGen) | rs12714414 | 2:651407 | C/T | 0.152 | -0.0353 (0.0027) | 8.2E-39 | 0.0099 (0.0258) | 0.702 | 170.9 | -0.280 |
| Appendicular lean mass | All-cause dementia (FinnGen) | rs5742915 | 15:74336633 | C/T | 0.461 | 0.0248 (0.0019) | 9.3E-39 | -0.0331 (0.0195) | 0.089 | 170.4 | -1.335 |
| Appendicular lean mass | All-cause dementia (FinnGen) | rs33973388 | 18:46611842 | T/G | 0.435 | 0.0249 (0.0019) | 1.4E-38 | 0.0101 (0.0208) | 0.626 | 171.8 | 0.406 |
| Appendicular lean mass | All-cause dementia (FinnGen) | rs8019890 | 14:21538067 | A/C | 0.531 | 0.0250 (0.0019) | 2.0E-38 | 0.0261 (0.0193) | 0.177 | 173.1 | 1.044 |
| Appendicular lean mass | All-cause dementia (FinnGen) | rs8042545 | 15:66992821 | A/G | 0.242 | 0.0287 (0.0022) | 2.9E-38 | -0.0150 (0.0229) | 0.514 | 170.2 | -0.523 |
| Appendicular lean mass | All-cause dementia (FinnGen) | rs2900208 | 12:11878464 | A/C | 0.354 | 0.0255 (0.0020) | 3.8E-38 | -0.0149 (0.0197) | 0.449 | 162.6 | -0.584 |
| Appendicular lean mass | All-cause dementia (FinnGen) | rs10748128 | 12:69827658 | T/G | 0.345 | 0.0255 (0.0020) | 6.8E-38 | 0.0287 (0.0200) | 0.152 | 162.6 | 1.125 |
| Appendicular lean mass | All-cause dementia (FinnGen) | rs28592876 | 12:123866429 | A/G | 0.205 | 0.0300 (0.0023) | 9.1E-38 | -0.0139 (0.0235) | 0.555 | 170.1 | -0.463 |
| Appendicular lean mass | All-cause dementia (FinnGen) | rs4615815 | 1:214604236 | G/A | 0.397 | -0.0246 (0.0019) | 1.1E-37 | -0.0105 (0.0201) | 0.603 | 167.6 | 0.427 |
| Appendicular lean mass | All-cause dementia (FinnGen) | rs244711 | 5:176509193 | T/C | 0.686 | 0.0279 (0.0022) | 1.5E-37 | -0.0576 (0.0208) | 0.006 | 160.8 | -2.065 |
| Appendicular lean mass | All-cause dementia (FinnGen) | rs17036160 | 3:12329783 | T/C | 0.118 | -0.0375 (0.0029) | 1.7E-37 | 0.0609 (0.0254) | 0.017 | 167.2 | -1.624 |
| Appendicular lean mass | All-cause dementia (FinnGen) | rs36226649 | 14:24835500 | C/T | 0.067 | 0.0485 (0.0038) | 3.1E-37 | 0.0367 (0.0444) | 0.409 | 162.9 | 0.757 |
| Appendicular lean mass | All-cause dementia (FinnGen) | rs61944841 | 13:27049616 | A/G | 0.414 | 0.0253 (0.0020) | 3.5E-37 | 0.0058 (0.0197) | 0.768 | 160.0 | 0.229 |
| Appendicular lean mass | All-cause dementia (FinnGen) | rs3853252 | 6:152170247 | A/G | 0.455 | 0.0241 (0.0019) | 8.8E-37 | -0.0068 (0.0194) | 0.728 | 160.9 | -0.282 |
| Appendicular lean mass | All-cause dementia (FinnGen) | rs7301341 | 12:94083105 | C/T | 0.327 | -0.0255 (0.0020) | 9.3E-37 | -0.0340 (0.0201) | 0.091 | 162.6 | 1.333 |
| Appendicular lean mass | All-cause dementia (FinnGen) | rs12622189 | 2:218132858 | G/A | 0.258 | -0.0272 (0.0021) | 9.3E-37 | 0.0129 (0.0199) | 0.518 | 167.8 | -0.474 |
| Appendicular lean mass | All-cause dementia (FinnGen) | rs2035901 | 4:145521867 | G/A | 0.468 | 0.0240 (0.0019) | 9.4E-37 | 0.0096 (0.0194) | 0.622 | 159.6 | 0.400 |
| Appendicular lean mass | All-cause dementia (FinnGen) | rs778384 | 1:56637166 | A/G | 0.223 | 0.0283 (0.0022) | 2.8E-36 | -0.0146 (0.0234) | 0.533 | 165.5 | -0.516 |
| Appendicular lean mass | All-cause dementia (FinnGen) | rs3843750 | 19:10748073 | G/C | 0.664 | -0.0252 (0.0020) | 5.1E-36 | 0.0379 (0.0213) | 0.076 | 158.8 | -1.504 |
| Appendicular lean mass | All-cause dementia (FinnGen) | rs4752829 | 11:47396654 | A/G | 0.286 | 0.0262 (0.0021) | 5.9E-36 | 0.0053 (0.0202) | 0.791 | 155.7 | 0.202 |
| Appendicular lean mass | All-cause dementia (FinnGen) | rs10691068 | 6:109723017 | TCC/T | 0.407 | -0.0242 (0.0019) | 6.5E-36 | 0.0126 (0.0192) | 0.511 | 162.2 | -0.521 |
| Appendicular lean mass | All-cause dementia (FinnGen) | rs59950280 | 4:3452345 | A/G | 0.332 | -0.0254 (0.0020) | 7.3E-36 | 0.0011 (0.0224) | 0.960 | 161.3 | -0.043 |
| Appendicular lean mass | All-cause dementia (FinnGen) | rs11689546 | 2:25130451 | G/A | 0.440 | -0.0238 (0.0019) | 8.1E-36 | 0.0051 (0.0196) | 0.793 | 156.9 | -0.214 |
| Appendicular lean mass | All-cause dementia (FinnGen) | rs2098695 | 5:54868944 | G/A | 0.691 | -0.0255 (0.0020) | 1.1E-35 | 0.0117 (0.0208) | 0.575 | 162.6 | -0.459 |
| Appendicular lean mass | All-cause dementia (FinnGen) | rs7582516 | 2:227255370 | T/C | 0.304 | 0.0254 (0.0020) | 1.3E-35 | -0.0261 (0.0215) | 0.224 | 161.3 | -1.028 |
| Appendicular lean mass | All-cause dementia (FinnGen) | rs3818416 | 13:78474468 | C/A | 0.764 | 0.0279 (0.0022) | 2.0E-35 | -0.0004 (0.0230) | 0.987 | 160.8 | -0.014 |
| Appendicular lean mass | All-cause dementia (FinnGen) | rs2549677 | 16:2162361 | G/A | 0.099 | -0.0392 (0.0032) | 1.3E-34 | 0.0067 (0.0381) | 0.861 | 150.1 | -0.171 |
| Appendicular lean mass | All-cause dementia (FinnGen) | rs1177765 | 5:32829929 | C/T | 0.468 | -0.0232 (0.0019) | 1.3E-34 | -0.0100 (0.0191) | 0.599 | 149.1 | 0.431 |
| Appendicular lean mass | All-cause dementia (FinnGen) | rs61838776 | 1:219618564 | A/C | 0.162 | 0.0311 (0.0025) | 2.4E-34 | 0.0567 (0.0293) | 0.053 | 154.8 | 1.823 |
| Appendicular lean mass | All-cause dementia (FinnGen) | rs115912456 | 5:82815158 | G/A | 0.041 | 0.0577 (0.0047) | 3.7E-34 | 0.0013 (0.0703) | 0.986 | 150.7 | 0.023 |
| Appendicular lean mass | All-cause dementia (FinnGen) | rs4852257 | 2:71678520 | G/T | 0.576 | -0.0231 (0.0019) | 6.2E-34 | -0.0006 (0.0191) | 0.975 | 147.8 | 0.026 |
| Appendicular lean mass | All-cause dementia (FinnGen) | rs12761076 | 10:72423050 | A/G | 0.266 | -0.0260 (0.0021) | 1.0E-33 | 0.0079 (0.0216) | 0.715 | 153.3 | -0.304 |
| Appendicular lean mass | All-cause dementia (FinnGen) | rs111365325 | 5:170865229 | T/C | 0.231 | -0.0271 (0.0022) | 1.1E-33 | 0.0238 (0.0238) | 0.319 | 151.7 | -0.878 |
| Appendicular lean mass | All-cause dementia (FinnGen) | rs45474992 | 19:47724564 | T/C | 0.036 | -0.0617 (0.0051) | 2.2E-33 | -0.0126 (0.0536) | 0.813 | 146.4 | 0.204 |
| Appendicular lean mass | All-cause dementia (FinnGen) | rs7428883 | 3:128983853 | G/A | 0.781 | 0.0277 (0.0023) | 2.3E-33 | 0.0273 (0.0257) | 0.287 | 145.1 | 0.986 |
| Appendicular lean mass | All-cause dementia (FinnGen) | rs76364830 | 8:13372120 | A/G | 0.063 | -0.0471 (0.0039) | 2.7E-33 | 0.0004 (0.0524) | 0.995 | 145.8 | -0.008 |
| Appendicular lean mass | All-cause dementia (FinnGen) | rs10202701 | 2:232328681 | T/C | 0.542 | 0.0227 (0.0019) | 3.1E-33 | -0.0150 (0.0192) | 0.433 | 142.7 | -0.661 |
| Appendicular lean mass | All-cause dementia (FinnGen) | rs4976262 | 5:134379531 | C/T | 0.316 | -0.0245 (0.0020) | 4.4E-33 | -0.0248 (0.0200) | 0.214 | 150.1 | 1.012 |
| Appendicular lean mass | All-cause dementia (FinnGen) | rs6082354 | 20:21217976 | C/A | 0.667 | -0.0240 (0.0020) | 1.2E-32 | 0.0139 (0.0211) | 0.508 | 144.0 | -0.579 |
| Appendicular lean mass | All-cause dementia (FinnGen) | rs2209098 | 1:172167226 | C/T | 0.311 | 0.0240 (0.0020) | 1.7E-32 | 0.0144 (0.0209) | 0.491 | 144.0 | 0.600 |
| Appendicular lean mass | All-cause dementia (FinnGen) | rs1444628 | 12:20563643 | T/C | 0.690 | 0.0240 (0.0020) | 6.8E-32 | -0.0040 (0.0234) | 0.863 | 144.0 | -0.167 |
| Appendicular lean mass | All-cause dementia (FinnGen) | rs147110934 | 19:55993436 | T/G | 0.024 | -0.0722 (0.0062) | 9.4E-32 | -0.0892 (0.1267) | 0.481 | 135.6 | 1.235 |
| Appendicular lean mass | All-cause dementia (FinnGen) | rs148138388 | 1:51371608 | C/G | 0.083 | 0.0406 (0.0035) | 1.1E-31 | 0.0142 (0.0411) | 0.729 | 134.6 | 0.350 |
| Appendicular lean mass | All-cause dementia (FinnGen) | rs58738817 | 3:184012228 | A/G | 0.248 | -0.0257 (0.0022) | 1.2E-31 | 0.0238 (0.0209) | 0.254 | 136.5 | -0.926 |
| Appendicular lean mass | All-cause dementia (FinnGen) | rs6570509 | 6:142716286 | T/G | 0.287 | -0.0244 (0.0021) | 1.3E-31 | -0.0057 (0.0219) | 0.794 | 135.0 | 0.234 |
| Appendicular lean mass | All-cause dementia (FinnGen) | rs4252548 | 19:55879672 | T/C | 0.022 | -0.0753 (0.0065) | 3.0E-31 | -0.0149 (0.0612) | 0.807 | 134.2 | 0.198 |
| Appendicular lean mass | All-cause dementia (FinnGen) | rs35464459 | 10:4964640 | C/T | 0.116 | -0.0342 (0.0029) | 5.0E-31 | -0.0339 (0.0286) | 0.236 | 139.1 | 0.991 |
| Appendicular lean mass | All-cause dementia (FinnGen) | rs1047891 | 2:211540507 | A/C | 0.316 | 0.0233 (0.0020) | 5.7E-31 | -0.0402 (0.0206) | 0.051 | 135.7 | -1.725 |
| Appendicular lean mass | All-cause dementia (FinnGen) | rs35756741 | 12:12868701 | T/C | 0.092 | -0.0378 (0.0033) | 5.8E-31 | -0.0136 (0.0315) | 0.666 | 131.2 | 0.360 |
| Appendicular lean mass | All-cause dementia (FinnGen) | rs55872725 | 16:53809123 | T/C | 0.404 | 0.0222 (0.0019) | 1.5E-30 | 0.0096 (0.0194) | 0.619 | 136.5 | 0.432 |
| Appendicular lean mass | All-cause dementia (FinnGen) | rs6501381 | 17:68148350 | C/T | 0.113 | -0.0341 (0.0030) | 5.2E-30 | -0.0107 (0.0336) | 0.749 | 129.2 | 0.314 |
| Appendicular lean mass | All-cause dementia (FinnGen) | rs12509014 | 4:123825919 | T/C | 0.790 | -0.0263 (0.0023) | 5.4E-30 | 0.0176 (0.0248) | 0.479 | 130.8 | -0.669 |
| Appendicular lean mass | All-cause dementia (FinnGen) | rs8031704 | 15:84651185 | A/C | 0.327 | -0.0228 (0.0020) | 2.1E-29 | -0.0104 (0.0203) | 0.610 | 130.0 | 0.456 |
| Appendicular lean mass | All-cause dementia (FinnGen) | rs212526 | 1:21584941 | C/T | 0.601 | 0.0214 (0.0019) | 3.8E-29 | -0.0191 (0.0198) | 0.334 | 126.9 | -0.893 |
| Appendicular lean mass | All-cause dementia (FinnGen) | rs9647379 | 3:171785168 | C/G | 0.411 | 0.0215 (0.0019) | 5.5E-29 | 0.0123 (0.0196) | 0.528 | 128.1 | 0.572 |
| Appendicular lean mass | All-cause dementia (FinnGen) | rs4244809 | 11:2164333 | A/G | 0.208 | -0.0262 (0.0023) | 5.6E-29 | 0.0133 (0.0249) | 0.594 | 129.8 | -0.508 |
| Appendicular lean mass | All-cause dementia (FinnGen) | rs4076427 | 17:79087537 | C/G | 0.391 | -0.0218 (0.0019) | 6.0E-29 | 0.0041 (0.0204) | 0.840 | 131.7 | -0.188 |
| Appendicular lean mass | All-cause dementia (FinnGen) | rs7229520 | 18:46516468 | A/G | 0.662 | -0.0224 (0.0020) | 9.2E-29 | -0.0087 (0.0201) | 0.664 | 125.4 | 0.388 |
| Appendicular lean mass | All-cause dementia (FinnGen) | rs6762851 | 3:56686329 | C/T | 0.357 | -0.0218 (0.0020) | 1.5E-28 | 0.0291 (0.0193) | 0.132 | 118.8 | -1.335 |
| Appendicular lean mass | All-cause dementia (FinnGen) | rs1211575 | 1:11118390 | A/G | 0.743 | -0.0237 (0.0021) | 1.9E-28 | 0.0050 (0.0214) | 0.815 | 127.4 | -0.211 |
| Appendicular lean mass | All-cause dementia (FinnGen) | rs4274112 | 1:26746199 | G/A | 0.373 | -0.0217 (0.0020) | 2.5E-28 | 0.0148 (0.0202) | 0.463 | 117.7 | -0.682 |
| Appendicular lean mass | All-cause dementia (FinnGen) | rs2071518 | 8:120435812 | T/C | 0.264 | -0.0236 (0.0021) | 3.4E-28 | 0.0255 (0.0239) | 0.286 | 126.3 | -1.081 |
| Appendicular lean mass | All-cause dementia (FinnGen) | rs13302480 | 9:118465313 | C/G | 0.114 | -0.0328 (0.0030) | 4.4E-28 | 0.0627 (0.0384) | 0.102 | 119.5 | -1.912 |
| Appendicular lean mass | All-cause dementia (FinnGen) | rs36048468 | 5:122879901 | T/C | 0.209 | 0.0254 (0.0023) | 9.3E-28 | 0.0114 (0.0260) | 0.662 | 122.0 | 0.449 |
| Appendicular lean mass | All-cause dementia (FinnGen) | rs62370472 | 5:52767109 | C/T | 0.209 | -0.0253 (0.0023) | 1.4E-27 | 0.0152 (0.0226) | 0.501 | 121.0 | -0.601 |
| Appendicular lean mass | All-cause dementia (FinnGen) | rs77165542 | 2:430975 | T/C | 0.035 | -0.0557 (0.0051) | 1.8E-27 | -0.1662 (0.0731) | 0.023 | 119.3 | 2.984 |
| Appendicular lean mass | All-cause dementia (FinnGen) | rs62621812 | 7:127015083 | A/G | 0.020 | 0.0743 (0.0069) | 3.2E-27 | -0.0268 (0.0478) | 0.574 | 116.0 | -0.361 |
| Appendicular lean mass | All-cause dementia (FinnGen) | rs181766 | 16:14394878 | C/T | 0.322 | 0.0221 (0.0020) | 4.3E-27 | 0.0054 (0.0201) | 0.787 | 122.1 | 0.244 |
| Appendicular lean mass | All-cause dementia (FinnGen) | rs62048221 | 16:88846552 | T/C | 0.231 | -0.0242 (0.0022) | 4.9E-27 | 0.0282 (0.0240) | 0.239 | 121.0 | -1.165 |
| Appendicular lean mass | All-cause dementia (FinnGen) | rs7781964 | 7:139749346 | A/G | 0.185 | 0.0261 (0.0024) | 5.7E-27 | -0.0234 (0.0251) | 0.351 | 118.3 | -0.897 |
| Appendicular lean mass | All-cause dementia (FinnGen) | rs2070598 | 14:75360906 | A/G | 0.456 | 0.0204 (0.0019) | 6.4E-27 | 0.0251 (0.0193) | 0.195 | 115.3 | 1.230 |
| Appendicular lean mass | All-cause dementia (FinnGen) | rs13209685 | 6:7779729 | T/G | 0.159 | 0.0277 (0.0026) | 7.5E-27 | 0.0033 (0.0237) | 0.888 | 113.5 | 0.119 |
| Appendicular lean mass | All-cause dementia (FinnGen) | rs951366 | 1:205685352 | C/T | 0.393 | 0.0205 (0.0019) | 9.2E-27 | -0.0279 (0.0199) | 0.160 | 116.4 | -1.361 |
| Appendicular lean mass | All-cause dementia (FinnGen) | rs291970 | 10:121123633 | T/C | 0.229 | 0.0240 (0.0022) | 1.3E-26 | -0.0016 (0.0259) | 0.950 | 119.0 | -0.067 |
| Appendicular lean mass | All-cause dementia (FinnGen) | rs4752689 | 10:124131176 | A/G | 0.584 | 0.0205 (0.0019) | 1.4E-26 | -0.0445 (0.0210) | 0.034 | 116.4 | -2.171 |
| Appendicular lean mass | All-cause dementia (FinnGen) | rs7768382 | 6:166341870 | C/T | 0.477 | -0.0201 (0.0019) | 1.6E-26 | 0.0005 (0.0191) | 0.980 | 111.9 | -0.025 |
| Appendicular lean mass | All-cause dementia (FinnGen) | rs72841270 | 10:104642237 | G/T | 0.135 | 0.0294 (0.0028) | 2.3E-26 | 0.0305 (0.0277) | 0.270 | 110.2 | 1.037 |
| Appendicular lean mass | All-cause dementia (FinnGen) | rs7014590 | 8:89335647 | C/T | 0.261 | -0.0228 (0.0022) | 4.5E-26 | 0.0034 (0.0221) | 0.876 | 107.4 | -0.149 |
| Appendicular lean mass | All-cause dementia (FinnGen) | rs9659061 | 1:150527292 | A/G | 0.476 | -0.0198 (0.0019) | 6.8E-26 | -0.0085 (0.0194) | 0.661 | 108.6 | 0.429 |
| Appendicular lean mass | All-cause dementia (FinnGen) | rs7497684 | 15:89342720 | A/C | 0.480 | 0.0200 (0.0019) | 8.3E-26 | -0.0044 (0.0194) | 0.820 | 110.8 | -0.220 |
| Appendicular lean mass | All-cause dementia (FinnGen) | rs1662842 | 4:82155362 | G/A | 0.307 | 0.0214 (0.0020) | 1.7E-25 | 0.0022 (0.0198) | 0.913 | 114.5 | 0.103 |
| Appendicular lean mass | All-cause dementia (FinnGen) | rs7072873 | 10:77210191 | T/C | 0.539 | -0.0198 (0.0019) | 3.2E-25 | 0.0018 (0.0191) | 0.925 | 108.6 | -0.091 |
| Appendicular lean mass | All-cause dementia (FinnGen) | rs4640244 | 17:21284223 | G/A | 0.399 | -0.0200 (0.0019) | 3.8E-25 | 0.0207 (0.0195) | 0.289 | 110.8 | -1.035 |
| Appendicular lean mass | All-cause dementia (FinnGen) | rs7858712 | 9:16738312 | G/A | 0.914 | 0.0347 (0.0034) | 1.0E-24 | -0.0224 (0.0352) | 0.524 | 104.2 | -0.646 |
| Appendicular lean mass | All-cause dementia (FinnGen) | rs927376 | 20:38569825 | G/C | 0.260 | -0.0223 (0.0022) | 1.2E-24 | -0.0028 (0.0246) | 0.910 | 102.8 | 0.126 |
| Appendicular lean mass | All-cause dementia (FinnGen) | rs78457529 | 16:24950880 | T/C | 0.012 | -0.0904 (0.0088) | 1.2E-24 | -0.0466 (0.0724) | 0.520 | 105.5 | 0.515 |
| Appendicular lean mass | All-cause dementia (FinnGen) | rs2748501 | 6:146312258 | A/G | 0.440 | -0.0195 (0.0019) | 1.3E-24 | 0.0012 (0.0192) | 0.952 | 105.3 | -0.062 |
| Appendicular lean mass | All-cause dementia (FinnGen) | rs12882130 | 14:103878774 | G/C | 0.384 | -0.0202 (0.0020) | 1.9E-24 | 0.0064 (0.0198) | 0.748 | 102.0 | -0.317 |
| Appendicular lean mass | All-cause dementia (FinnGen) | rs45528934 | 14:23793305 | T/C | 0.162 | 0.0262 (0.0026) | 2.0E-24 | 0.0428 (0.0283) | 0.131 | 101.5 | 1.634 |
| Appendicular lean mass | All-cause dementia (FinnGen) | rs41311445 | 22:42070374 | C/A | 0.096 | -0.0328 (0.0032) | 4.7E-24 | -0.0070 (0.0280) | 0.802 | 105.1 | 0.213 |
| Appendicular lean mass | All-cause dementia (FinnGen) | rs60804050 | 1:118870373 | A/G | 0.256 | -0.0217 (0.0021) | 5.0E-24 | 0.0111 (0.0224) | 0.619 | 106.8 | -0.512 |
| Appendicular lean mass | All-cause dementia (FinnGen) | rs7816345 | 8:36846109 | T/C | 0.168 | 0.0255 (0.0025) | 6.2E-24 | 0.0359 (0.0277) | 0.194 | 104.0 | 1.408 |
| Appendicular lean mass | All-cause dementia (FinnGen) | rs2627702 | 4:88577419 | C/T | 0.519 | -0.0191 (0.0019) | 6.4E-24 | 0.0298 (0.0196) | 0.129 | 101.1 | -1.560 |
| Appendicular lean mass | All-cause dementia (FinnGen) | rs7485647 | 12:131631133 | A/G | 0.844 | -0.0261 (0.0026) | 1.0E-23 | -0.0266 (0.0219) | 0.223 | 100.8 | 1.019 |
| Appendicular lean mass | All-cause dementia (FinnGen) | rs2296316 | 14:65520246 | C/T | 0.464 | -0.0192 (0.0019) | 1.6E-23 | -0.0114 (0.0191) | 0.552 | 102.1 | 0.594 |
| Appendicular lean mass | All-cause dementia (FinnGen) | rs2019203 | 17:36908672 | A/C | 0.491 | 0.0189 (0.0019) | 1.8E-23 | -0.0082 (0.0191) | 0.668 | 99.0 | -0.434 |
| Appendicular lean mass | All-cause dementia (FinnGen) | rs17035465 | 2:46890294 | G/A | 0.221 | 0.0226 (0.0023) | 2.1E-23 | 0.0179 (0.0270) | 0.506 | 96.5 | 0.792 |
| Appendicular lean mass | All-cause dementia (FinnGen) | rs8077636 | 17:79421254 | T/G | 0.418 | -0.0192 (0.0019) | 2.5E-23 | 0.0304 (0.0200) | 0.128 | 102.1 | -1.583 |
| Appendicular lean mass | All-cause dementia (FinnGen) | rs6919321 | 6:33724004 | A/G | 0.608 | 0.0192 (0.0019) | 3.6E-23 | 0.0036 (0.0214) | 0.866 | 102.1 | 0.188 |
| Appendicular lean mass | All-cause dementia (FinnGen) | rs2140046 | 2:169706079 | C/T | 0.363 | -0.0192 (0.0019) | 5.5E-23 | -0.0037 (0.0194) | 0.850 | 102.1 | 0.193 |
| Appendicular lean mass | All-cause dementia (FinnGen) | rs1786263 | 18:13116432 | T/G | 0.606 | -0.0190 (0.0019) | 1.0E-22 | -0.0085 (0.0195) | 0.661 | 100.0 | 0.447 |
| Appendicular lean mass | All-cause dementia (FinnGen) | rs9807032 | 17:62757146 | C/T | 0.818 | -0.0240 (0.0024) | 1.3E-22 | 0.0332 (0.0259) | 0.201 | 100.0 | -1.383 |
| Appendicular lean mass | All-cause dementia (FinnGen) | rs140440099 | 3:50632595 | A/G | 0.023 | 0.0613 (0.0063) | 1.4E-22 | 0.0232 (0.0763) | 0.761 | 94.7 | 0.378 |
| Appendicular lean mass | All-cause dementia (FinnGen) | rs113852999 | 7:148649606 | A/G | 0.169 | 0.0246 (0.0025) | 1.8E-22 | -0.0306 (0.0252) | 0.226 | 96.8 | -1.244 |
| Appendicular lean mass | All-cause dementia (FinnGen) | rs35732917 | 7:73013269 | C/T | 0.284 | 0.0204 (0.0021) | 2.1E-22 | 0.0023 (0.0227) | 0.921 | 94.4 | 0.113 |
| Appendicular lean mass | All-cause dementia (FinnGen) | rs9344126 | 6:81907559 | C/T | 0.514 | -0.0185 (0.0019) | 2.2E-22 | -0.0314 (0.0195) | 0.108 | 94.8 | 1.697 |
| Appendicular lean mass | All-cause dementia (FinnGen) | rs3184504 | 12:111884608 | C/T | 0.517 | 0.0183 (0.0019) | 2.7E-22 | -0.0064 (0.0194) | 0.740 | 92.8 | -0.350 |
| Appendicular lean mass | All-cause dementia (FinnGen) | rs332116 | 10:28926099 | T/C | 0.280 | -0.0206 (0.0021) | 2.9E-22 | -0.0108 (0.0196) | 0.580 | 96.2 | 0.524 |
| Appendicular lean mass | All-cause dementia (FinnGen) | rs10202845 | 2:42575820 | G/A | 0.113 | -0.0288 (0.0030) | 5.3E-22 | -0.0191 (0.0314) | 0.543 | 92.2 | 0.663 |
| Appendicular lean mass | All-cause dementia (FinnGen) | rs2305141 | 2:233684402 | G/A | 0.596 | 0.0183 (0.0019) | 1.1E-21 | -0.0292 (0.0216) | 0.176 | 92.8 | -1.596 |
| Appendicular lean mass | All-cause dementia (FinnGen) | rs1405212 | 6:117490664 | C/T | 0.628 | 0.0187 (0.0019) | 1.1E-21 | -0.0207 (0.0202) | 0.306 | 96.9 | -1.107 |
| Appendicular lean mass | All-cause dementia (FinnGen) | rs9838614 | 3:38537671 | G/T | 0.388 | -0.0185 (0.0019) | 1.2E-21 | 0.0229 (0.0194) | 0.238 | 94.8 | -1.238 |
| Appendicular lean mass | All-cause dementia (FinnGen) | rs12679359 | 8:130724556 | T/G | 0.137 | -0.0263 (0.0028) | 1.4E-21 | 0.0152 (0.0276) | 0.582 | 88.2 | -0.578 |
| Appendicular lean mass | All-cause dementia (FinnGen) | rs6054390 | 20:6592094 | A/G | 0.628 | -0.0188 (0.0020) | 1.4E-21 | 0.0128 (0.0209) | 0.542 | 88.4 | -0.681 |
| Appendicular lean mass | All-cause dementia (FinnGen) | rs10945540 | 6:158738975 | A/G | 0.665 | -0.0190 (0.0020) | 1.8E-21 | 0.0072 (0.0197) | 0.716 | 90.2 | -0.379 |
| Appendicular lean mass | All-cause dementia (FinnGen) | rs670318 | 1:63727542 | C/T | 0.952 | 0.0413 (0.0044) | 2.5E-21 | 0.0267 (0.0410) | 0.515 | 88.1 | 0.646 |
| Appendicular lean mass | All-cause dementia (FinnGen) | rs112537273 | 8:38248306 | C/T | 0.230 | -0.0212 (0.0022) | 3.3E-21 | -0.0060 (0.0249) | 0.810 | 92.9 | 0.283 |
| Appendicular lean mass | All-cause dementia (FinnGen) | rs1040977 | 9:95554873 | T/C | 0.166 | -0.0240 (0.0025) | 3.9E-21 | 0.0268 (0.0303) | 0.376 | 92.2 | -1.117 |
| Appendicular lean mass | All-cause dementia (FinnGen) | rs7701233 | 5:171218388 | C/T | 0.428 | -0.0179 (0.0019) | 5.1E-21 | 0.0074 (0.0191) | 0.699 | 88.8 | -0.413 |
| Appendicular lean mass | All-cause dementia (FinnGen) | rs2212926 | 21:38066883 | A/C | 0.211 | -0.0220 (0.0023) | 7.7E-21 | -0.0038 (0.0273) | 0.890 | 91.5 | 0.173 |
| Appendicular lean mass | All-cause dementia (FinnGen) | rs10832963 | 11:18664241 | G/T | 0.745 | -0.0203 (0.0022) | 9.2E-21 | -0.0238 (0.0197) | 0.226 | 85.1 | 1.172 |
| Appendicular lean mass | All-cause dementia (FinnGen) | rs7636265 | 3:134374223 | A/G | 0.638 | -0.0183 (0.0020) | 9.4E-21 | 0.0118 (0.0207) | 0.570 | 83.7 | -0.645 |
| Appendicular lean mass | All-cause dementia (FinnGen) | rs9384679 | 6:108864419 | T/C | 0.370 | -0.0182 (0.0019) | 9.9E-21 | -0.0022 (0.0194) | 0.910 | 91.8 | 0.121 |
| Appendicular lean mass | All-cause dementia (FinnGen) | rs17478946 | 12:24093062 | G/A | 0.300 | -0.0192 (0.0021) | 1.0E-20 | -0.0155 (0.0218) | 0.477 | 83.6 | 0.807 |
| Appendicular lean mass | All-cause dementia (FinnGen) | rs9957318 | 18:33039106 | G/A | 0.348 | 0.0187 (0.0020) | 1.0E-20 | 0.0077 (0.0205) | 0.709 | 87.4 | 0.412 |
| Appendicular lean mass | All-cause dementia (FinnGen) | rs28787734 | 4:87560357 | T/C | 0.058 | 0.0385 (0.0041) | 1.1E-20 | 0.0921 (0.0595) | 0.122 | 88.2 | 2.392 |
| Appendicular lean mass | All-cause dementia (FinnGen) | rs1473441 | 8:49588463 | G/A | 0.724 | -0.0198 (0.0021) | 1.2E-20 | -0.0263 (0.0218) | 0.228 | 88.9 | 1.328 |
| Appendicular lean mass | All-cause dementia (FinnGen) | rs34287 | 5:67585143 | A/G | 0.341 | 0.0187 (0.0020) | 1.2E-20 | -0.0102 (0.0194) | 0.598 | 87.4 | -0.545 |
| Appendicular lean mass | All-cause dementia (FinnGen) | rs35816944 | 16:1828030 | A/G | 0.007 | -0.1088 (0.0117) | 1.3E-20 | 0.0676 (0.1186) | 0.569 | 86.5 | -0.621 |
| Appendicular lean mass | All-cause dementia (FinnGen) | rs7633464 | 3:98715823 | A/G | 0.479 | 0.0175 (0.0019) | 1.3E-20 | 0.0104 (0.0191) | 0.586 | 84.8 | 0.594 |
| Appendicular lean mass | All-cause dementia (FinnGen) | rs2993531 | 1:212190116 | C/A | 0.589 | 0.0176 (0.0019) | 1.7E-20 | -0.0283 (0.0198) | 0.154 | 85.8 | -1.608 |
| Appendicular lean mass | All-cause dementia (FinnGen) | rs9696116 | 9:139100805 | C/T | 0.318 | -0.0188 (0.0020) | 2.4E-20 | 0.0272 (0.0204) | 0.183 | 88.4 | -1.447 |
| Appendicular lean mass | All-cause dementia (FinnGen) | rs3103268 | 2:232984969 | T/C | 0.902 | 0.0291 (0.0031) | 2.7E-20 | 0.0113 (0.0444) | 0.799 | 88.1 | 0.388 |
| Appendicular lean mass | All-cause dementia (FinnGen) | rs4985445 | 16:69867835 | G/A | 0.457 | -0.0175 (0.0019) | 3.3E-20 | 0.0104 (0.0192) | 0.590 | 84.8 | -0.594 |
| Appendicular lean mass | All-cause dementia (FinnGen) | rs11590254 | 1:92316573 | T/A | 0.311 | 0.0186 (0.0020) | 4.3E-20 | 0.0151 (0.0199) | 0.447 | 86.5 | 0.812 |
| Appendicular lean mass | All-cause dementia (FinnGen) | rs73125634 | 20:20069826 | T/G | 0.278 | -0.0195 (0.0021) | 5.1E-20 | -0.0049 (0.0200) | 0.806 | 86.2 | 0.251 |
| Appendicular lean mass | All-cause dementia (FinnGen) | rs6874142 | 5:172753555 | G/T | 0.114 | 0.0288 (0.0031) | 5.1E-20 | -0.0659 (0.0332) | 0.047 | 86.3 | -2.288 |
| Appendicular lean mass | All-cause dementia (FinnGen) | rs140657345 | 15:41615909 | TCCTC/T | 0.252 | -0.0200 (0.0022) | 5.5E-20 | -0.0004 (0.0221) | 0.987 | 82.6 | 0.020 |
| Appendicular lean mass | All-cause dementia (FinnGen) | rs62466110 | 7:92623541 | C/T | 0.067 | -0.0371 (0.0041) | 5.7E-20 | -0.0152 (0.0287) | 0.597 | 81.9 | 0.410 |
| Appendicular lean mass | All-cause dementia (FinnGen) | rs12702693 | 7:8101039 | T/C | 0.454 | 0.0173 (0.0019) | 6.6E-20 | -0.0151 (0.0199) | 0.446 | 82.9 | -0.873 |
| Appendicular lean mass | All-cause dementia (FinnGen) | rs34345560 | 11:69081998 | A/G | 0.195 | 0.0219 (0.0024) | 7.1E-20 | -0.0093 (0.0220) | 0.674 | 83.3 | -0.425 |
| Appendicular lean mass | All-cause dementia (FinnGen) | rs12055045 | 5:137608056 | T/C | 0.206 | 0.0213 (0.0023) | 7.1E-20 | -0.0065 (0.0223) | 0.770 | 85.8 | -0.305 |
| Appendicular lean mass | All-cause dementia (FinnGen) | rs655113 | 6:52269151 | C/T | 0.301 | 0.0188 (0.0021) | 7.4E-20 | 0.0091 (0.0202) | 0.654 | 80.2 | 0.484 |
| Appendicular lean mass | All-cause dementia (FinnGen) | rs9832919 | 3:132184526 | G/A | 0.357 | -0.0179 (0.0020) | 7.9E-20 | 0.0055 (0.0200) | 0.784 | 80.1 | -0.307 |
| Appendicular lean mass | All-cause dementia (FinnGen) | rs8107967 | 19:7972615 | G/A | 0.566 | -0.0174 (0.0019) | 9.5E-20 | -0.0252 (0.0192) | 0.191 | 83.9 | 1.448 |
| Appendicular lean mass | All-cause dementia (FinnGen) | rs36012032 | 3:52814709 | A/C | 0.091 | 0.0298 (0.0033) | 9.9E-20 | 0.0034 (0.0343) | 0.921 | 81.5 | 0.114 |
| Appendicular lean mass | All-cause dementia (FinnGen) | rs59753424 | 20:54884760 | A/G | 0.267 | 0.0200 (0.0022) | 1.1E-19 | -0.0069 (0.0240) | 0.774 | 82.6 | -0.345 |
| Appendicular lean mass | All-cause dementia (FinnGen) | rs76693355 | 11:120292477 | C/T | 0.116 | -0.0268 (0.0030) | 1.2E-19 | 0.0148 (0.0289) | 0.607 | 79.8 | -0.552 |
| Appendicular lean mass | All-cause dementia (FinnGen) | rs78180894 | 17:7483662 | C/G | 0.074 | 0.0338 (0.0037) | 1.3E-19 | -0.0741 (0.0707) | 0.294 | 83.5 | -2.192 |
| Appendicular lean mass | All-cause dementia (FinnGen) | rs12533452 | 7:19016871 | T/C | 0.157 | 0.0237 (0.0026) | 1.3E-19 | 0.0040 (0.0274) | 0.884 | 83.1 | 0.169 |
| Appendicular lean mass | All-cause dementia (FinnGen) | rs77542162 | 17:67081278 | G/A | 0.023 | 0.0576 (0.0064) | 1.5E-19 | 0.1063 (0.1182) | 0.368 | 81.0 | 1.845 |
| Appendicular lean mass | All-cause dementia (FinnGen) | rs12997625 | 2:202970250 | T/C | 0.527 | -0.0170 (0.0019) | 1.5E-19 | 0.0138 (0.0193) | 0.473 | 80.1 | -0.812 |
| Appendicular lean mass | All-cause dementia (FinnGen) | rs591668 | 3:27535931 | A/G | 0.396 | -0.0174 (0.0019) | 2.0E-19 | -0.0180 (0.0195) | 0.354 | 83.9 | 1.034 |
| Appendicular lean mass | All-cause dementia (FinnGen) | rs11959466 | 5:42803824 | T/C | 0.056 | 0.0380 (0.0042) | 2.2E-19 | -0.0064 (0.0367) | 0.861 | 81.9 | -0.168 |
| Appendicular lean mass | All-cause dementia (FinnGen) | rs261999 | 5:95899427 | T/C | 0.370 | 0.0175 (0.0019) | 2.5E-19 | -0.0084 (0.0194) | 0.666 | 84.8 | -0.480 |
| Appendicular lean mass | All-cause dementia (FinnGen) | rs3063063 | 2:183231361 | TGAA/T | 0.686 | -0.0182 (0.0020) | 2.6E-19 | -0.0006 (0.0218) | 0.979 | 82.8 | 0.033 |
| Appendicular lean mass | All-cause dementia (FinnGen) | rs11672848 | 19:37570704 | T/C | 0.525 | -0.0171 (0.0019) | 7.7E-19 | -0.0144 (0.0191) | 0.449 | 81.0 | 0.842 |
| Appendicular lean mass | All-cause dementia (FinnGen) | rs894736 | 12:54418166 | G/A | 0.363 | -0.0174 (0.0020) | 1.1E-18 | 0.0138 (0.0191) | 0.471 | 75.7 | -0.793 |
| Appendicular lean mass | All-cause dementia (FinnGen) | rs2112617 | 17:46977125 | A/G | 0.529 | -0.0167 (0.0019) | 1.1E-18 | -0.0054 (0.0191) | 0.778 | 77.2 | 0.323 |
| Appendicular lean mass | All-cause dementia (FinnGen) | rs11049704 | 12:28691701 | G/C | 0.293 | -0.0183 (0.0021) | 1.1E-18 | 0.0443 (0.0218) | 0.042 | 75.9 | -2.421 |
| Appendicular lean mass | All-cause dementia (FinnGen) | rs700677 | 2:198702424 | A/C | 0.350 | 0.0173 (0.0020) | 1.1E-18 | 0.0109 (0.0213) | 0.609 | 74.8 | 0.630 |
| Appendicular lean mass | All-cause dementia (FinnGen) | rs6739278 | 2:44401055 | C/T | 0.808 | -0.0210 (0.0024) | 1.3E-18 | 0.0187 (0.0222) | 0.401 | 76.6 | -0.890 |
| Appendicular lean mass | All-cause dementia (FinnGen) | rs1805165 | 2:88874891 | A/C | 0.720 | -0.0184 (0.0021) | 1.3E-18 | 0.0241 (0.0203) | 0.234 | 76.8 | -1.310 |
| Appendicular lean mass | All-cause dementia (FinnGen) | rs6569643 | 6:130312646 | A/G | 0.526 | -0.0167 (0.0019) | 1.4E-18 | 0.0246 (0.0197) | 0.211 | 77.2 | -1.473 |
| Appendicular lean mass | All-cause dementia (FinnGen) | rs2052478 | 5:77437291 | T/C | 0.235 | -0.0195 (0.0022) | 1.8E-18 | 0.0063 (0.0249) | 0.799 | 78.6 | -0.323 |
| Appendicular lean mass | All-cause dementia (FinnGen) | rs9513510 | 13:99571922 | C/G | 0.699 | -0.0181 (0.0021) | 2.5E-18 | -0.0119 (0.0204) | 0.561 | 74.3 | 0.657 |
| Appendicular lean mass | All-cause dementia (FinnGen) | rs7731023 | 5:36181627 | G/A | 0.575 | 0.0166 (0.0019) | 3.5E-18 | 0.0133 (0.0201) | 0.507 | 76.3 | 0.801 |
| Appendicular lean mass | All-cause dementia (FinnGen) | rs2531991 | 16:4023553 | A/G | 0.748 | 0.0189 (0.0022) | 5.2E-18 | -0.0117 (0.0217) | 0.589 | 73.8 | -0.619 |
| Appendicular lean mass | All-cause dementia (FinnGen) | rs12909863 | 15:75825822 | C/G | 0.251 | 0.0189 (0.0022) | 6.0E-18 | -0.0275 (0.0229) | 0.229 | 73.8 | -1.455 |
| Appendicular lean mass | All-cause dementia (FinnGen) | rs12351226 | 9:98405230 | T/C | 0.171 | 0.0218 (0.0025) | 9.1E-18 | 0.0332 (0.0309) | 0.282 | 76.0 | 1.523 |
| Appendicular lean mass | All-cause dementia (FinnGen) | rs7512641 | 1:10822171 | A/T | 0.741 | -0.0185 (0.0022) | 9.9E-18 | 0.0270 (0.0208) | 0.195 | 70.7 | -1.459 |
| Appendicular lean mass | All-cause dementia (FinnGen) | rs7666804 | 4:57750148 | C/T | 0.389 | -0.0167 (0.0019) | 9.9E-18 | -0.0281 (0.0200) | 0.160 | 77.2 | 1.683 |
| Appendicular lean mass | All-cause dementia (FinnGen) | rs395980 | 4:177430072 | G/T | 0.264 | -0.0184 (0.0021) | 1.0E-17 | 0.0056 (0.0215) | 0.794 | 76.8 | -0.304 |
| Appendicular lean mass | All-cause dementia (FinnGen) | rs12037677 | 1:109918766 | C/T | 0.718 | -0.0178 (0.0021) | 1.2E-17 | 0.0410 (0.0212) | 0.053 | 71.8 | -2.303 |
| Appendicular lean mass | All-cause dementia (FinnGen) | rs73384223 | 9:3869315 | C/T | 0.197 | -0.0205 (0.0024) | 1.3E-17 | 0.0110 (0.0244) | 0.651 | 73.0 | -0.537 |
| Appendicular lean mass | All-cause dementia (FinnGen) | rs7598430 | 2:219193963 | T/C | 0.505 | -0.0160 (0.0019) | 1.4E-17 | 0.0008 (0.0193) | 0.967 | 70.9 | -0.050 |
| Appendicular lean mass | All-cause dementia (FinnGen) | rs2721940 | 8:116636281 | C/A | 0.602 | -0.0165 (0.0019) | 1.5E-17 | 0.0136 (0.0211) | 0.520 | 75.4 | -0.824 |
| Appendicular lean mass | All-cause dementia (FinnGen) | rs9817452 | 3:156795414 | T/G | 0.388 | 0.0165 (0.0019) | 1.9E-17 | -0.0076 (0.0207) | 0.714 | 75.4 | -0.461 |
| Appendicular lean mass | All-cause dementia (FinnGen) | rs10922475 | 1:89142142 | A/C | 0.539 | 0.0159 (0.0019) | 2.2E-17 | 0.0455 (0.0197) | 0.021 | 70.0 | 2.862 |
| Appendicular lean mass | All-cause dementia (FinnGen) | rs12299065 | 12:117442304 | A/G | 0.127 | -0.0240 (0.0028) | 2.2E-17 | 0.0196 (0.0273) | 0.474 | 73.5 | -0.817 |
| Appendicular lean mass | All-cause dementia (FinnGen) | rs56208656 | 19:3575343 | A/G | 0.110 | -0.0284 (0.0034) | 3.0E-17 | 0.0395 (0.0299) | 0.187 | 69.8 | -1.391 |
| Appendicular lean mass | All-cause dementia (FinnGen) | rs10040039 | 5:88354597 | G/A | 0.387 | -0.0163 (0.0019) | 3.3E-17 | -0.0198 (0.0209) | 0.343 | 73.6 | 1.215 |
| Appendicular lean mass | All-cause dementia (FinnGen) | rs59951000 | 18:74975828 | T/C | 0.042 | -0.0400 (0.0047) | 3.3E-17 | 0.0489 (0.0356) | 0.170 | 72.4 | -1.222 |
| Appendicular lean mass | All-cause dementia (FinnGen) | rs201570119 | 2:112256320 | C/T | 0.210 | 0.0194 (0.0023) | 5.5E-17 | 0.0248 (0.0257) | 0.335 | 71.2 | 1.278 |
| Appendicular lean mass | All-cause dementia (FinnGen) | rs2347603 | 2:47297426 | A/T | 0.742 | -0.0181 (0.0022) | 5.6E-17 | -0.0093 (0.0217) | 0.668 | 67.7 | 0.514 |
| Appendicular lean mass | All-cause dementia (FinnGen) | rs1341215 | 9:111662350 | A/G | 0.137 | 0.0229 (0.0027) | 6.3E-17 | -0.0214 (0.0248) | 0.388 | 71.9 | -0.934 |
| Appendicular lean mass | All-cause dementia (FinnGen) | rs10005035 | 4:12865684 | G/C | 0.282 | -0.0175 (0.0021) | 6.5E-17 | 0.0169 (0.0222) | 0.445 | 69.4 | -0.966 |
| Appendicular lean mass | All-cause dementia (FinnGen) | rs1556659 | 10:130834698 | T/C | 0.382 | 0.0163 (0.0020) | 7.2E-17 | -0.0031 (0.0210) | 0.882 | 66.4 | -0.190 |
| Appendicular lean mass | All-cause dementia (FinnGen) | rs10776560 | 10:50542358 | T/C | 0.499 | -0.0157 (0.0019) | 7.9E-17 | 0.0096 (0.0198) | 0.627 | 68.3 | -0.611 |
| Appendicular lean mass | All-cause dementia (FinnGen) | rs10514518 | 16:81579827 | T/C | 0.685 | 0.0171 (0.0020) | 8.3E-17 | 0.0130 (0.0211) | 0.540 | 73.1 | 0.760 |
| Appendicular lean mass | All-cause dementia (FinnGen) | rs112898929 | 16:88298795 | A/T | 0.080 | -0.0296 (0.0036) | 9.6E-17 | 0.0055 (0.0317) | 0.863 | 67.6 | -0.186 |
| Appendicular lean mass | All-cause dementia (FinnGen) | rs9784904 | 7:55835078 | C/T | 0.155 | -0.0217 (0.0026) | 9.7E-17 | 0.0186 (0.0226) | 0.411 | 69.7 | -0.857 |
| Appendicular lean mass | All-cause dementia (FinnGen) | rs1063582 | 8:23167353 | G/T | 0.765 | -0.0185 (0.0022) | 1.1E-16 | 0.0037 (0.0233) | 0.875 | 70.7 | -0.200 |
| Appendicular lean mass | All-cause dementia (FinnGen) | rs10823149 | 10:69935396 | T/G | 0.548 | 0.0157 (0.0019) | 1.2E-16 | -0.0089 (0.0194) | 0.645 | 68.3 | -0.567 |
| Appendicular lean mass | All-cause dementia (FinnGen) | rs4815952 | 20:6934897 | C/T | 0.522 | -0.0161 (0.0019) | 1.2E-16 | 0.0203 (0.0193) | 0.293 | 71.8 | -1.261 |
| Appendicular lean mass | All-cause dementia (FinnGen) | rs9809116 | 3:72397279 | G/A | 0.408 | -0.0160 (0.0019) | 1.3E-16 | -0.0028 (0.0204) | 0.892 | 70.9 | 0.175 |
| Appendicular lean mass | All-cause dementia (FinnGen) | rs35166681 | 6:85369727 | TA/T | 0.159 | 0.0214 (0.0026) | 1.5E-16 | 0.0276 (0.0273) | 0.311 | 67.8 | 1.290 |
| Appendicular lean mass | All-cause dementia (FinnGen) | rs12512942 | 4:177766307 | A/G | 0.638 | -0.0162 (0.0020) | 1.6E-16 | -0.0385 (0.0194) | 0.047 | 65.6 | 2.377 |
| Appendicular lean mass | All-cause dementia (FinnGen) | rs10843391 | 12:29508642 | G/A | 0.285 | 0.0172 (0.0021) | 2.1E-16 | 0.0055 (0.0210) | 0.794 | 67.1 | 0.320 |
| Appendicular lean mass | All-cause dementia (FinnGen) | rs9391254 | 6:105377347 | T/C | 0.321 | 0.0166 (0.0020) | 2.1E-16 | 0.0215 (0.0206) | 0.295 | 68.9 | 1.295 |
| Appendicular lean mass | All-cause dementia (FinnGen) | rs71635721 | 3:171960170 | G/C | 0.065 | 0.0316 (0.0039) | 3.5E-16 | -0.0422 (0.0460) | 0.359 | 65.7 | -1.335 |
| Appendicular lean mass | All-cause dementia (FinnGen) | rs56363908 | 7:96611052 | G/A | 0.042 | -0.0382 (0.0047) | 3.9E-16 | -0.0578 (0.0525) | 0.271 | 66.1 | 1.513 |
| Appendicular lean mass | All-cause dementia (FinnGen) | rs9479012 | 6:151657994 | G/A | 0.094 | 0.0263 (0.0032) | 4.1E-16 | -0.0118 (0.0334) | 0.723 | 67.5 | -0.449 |
| Appendicular lean mass | All-cause dementia (FinnGen) | rs6543146 | 2:103096695 | G/T | 0.558 | 0.0154 (0.0019) | 4.2E-16 | -0.0060 (0.0214) | 0.781 | 65.7 | -0.390 |
| Appendicular lean mass | All-cause dementia (FinnGen) | rs17138358 | 7:17920253 | C/G | 0.398 | 0.0157 (0.0019) | 4.4E-16 | -0.0400 (0.0208) | 0.055 | 68.3 | -2.548 |
| Appendicular lean mass | All-cause dementia (FinnGen) | rs7367519 | 1:204479176 | C/T | 0.685 | 0.0164 (0.0020) | 4.7E-16 | -0.0408 (0.0233) | 0.079 | 67.2 | -2.488 |
| Appendicular lean mass | All-cause dementia (FinnGen) | rs61729527 | 8:77761919 | T/C | 0.052 | -0.0346 (0.0043) | 4.9E-16 | -0.0123 (0.0387) | 0.751 | 64.8 | 0.355 |
| Appendicular lean mass | All-cause dementia (FinnGen) | rs10208668 | 2:20556151 | C/G | 0.035 | 0.0415 (0.0051) | 5.0E-16 | 0.0145 (0.0679) | 0.830 | 66.2 | 0.349 |
| Appendicular lean mass | All-cause dementia (FinnGen) | rs2287821 | 19:33935102 | T/C | 0.509 | -0.0153 (0.0019) | 7.0E-16 | -0.0302 (0.0192) | 0.115 | 64.8 | 1.974 |
| Appendicular lean mass | All-cause dementia (FinnGen) | rs35963161 | 20:49210635 | A/G | 0.476 | -0.0157 (0.0019) | 7.5E-16 | 0.0204 (0.0193) | 0.292 | 68.3 | -1.299 |
| Appendicular lean mass | All-cause dementia (FinnGen) | rs1056747 | 9:35690102 | G/A | 0.412 | -0.0155 (0.0019) | 8.0E-16 | 0.0048 (0.0194) | 0.802 | 66.5 | -0.310 |
| Appendicular lean mass | All-cause dementia (FinnGen) | rs11777835 | 8:144986103 | C/T | 0.473 | -0.0152 (0.0019) | 9.6E-16 | 0.0488 (0.0192) | 0.011 | 64.0 | -3.211 |
| Appendicular lean mass | All-cause dementia (FinnGen) | rs2165772 | 11:27091907 | A/G | 0.354 | 0.0160 (0.0020) | 1.0E-15 | -0.0055 (0.0198) | 0.781 | 64.0 | -0.344 |
| Appendicular lean mass | All-cause dementia (FinnGen) | rs2596144 | 12:122297738 | C/T | 0.870 | -0.0223 (0.0028) | 1.5E-15 | 0.0000 (0.0231) | 0.999 | 63.4 | 0.000 |
| Appendicular lean mass | All-cause dementia (FinnGen) | rs8904 | 14:35871217 | A/G | 0.363 | -0.0157 (0.0020) | 1.5E-15 | 0.0167 (0.0197) | 0.396 | 61.6 | -1.064 |
| Appendicular lean mass | All-cause dementia (FinnGen) | rs4504126 | 3:33600582 | C/A | 0.028 | 0.0460 (0.0058) | 1.6E-15 | 0.0363 (0.0768) | 0.637 | 62.9 | 0.789 |
| Appendicular lean mass | All-cause dementia (FinnGen) | rs2007022 | 20:3237674 | A/C | 0.232 | 0.0179 (0.0022) | 1.6E-15 | 0.0104 (0.0265) | 0.696 | 66.2 | 0.581 |
| Appendicular lean mass | All-cause dementia (FinnGen) | rs2324154 | 4:24027226 | A/C | 0.510 | 0.0150 (0.0019) | 1.9E-15 | 0.0053 (0.0192) | 0.784 | 62.3 | 0.353 |
| Appendicular lean mass | All-cause dementia (FinnGen) | rs704660 | 11:30447998 | T/C | 0.409 | 0.0153 (0.0019) | 2.3E-15 | -0.0105 (0.0195) | 0.590 | 64.8 | -0.686 |
| Appendicular lean mass | All-cause dementia (FinnGen) | rs4076108 | 3:13736088 | T/A | 0.245 | 0.0174 (0.0022) | 2.3E-15 | 0.0130 (0.0216) | 0.547 | 62.5 | 0.747 |
| Appendicular lean mass | All-cause dementia (FinnGen) | rs7646501 | 3:24079291 | G/A | 0.264 | -0.0170 (0.0021) | 2.3E-15 | 0.0023 (0.0210) | 0.914 | 65.5 | -0.135 |
| Appendicular lean mass | All-cause dementia (FinnGen) | rs16844417 | 1:172377745 | A/G | 0.124 | 0.0226 (0.0029) | 2.6E-15 | 0.0145 (0.0283) | 0.609 | 60.7 | 0.642 |
| Appendicular lean mass | All-cause dementia (FinnGen) | rs12371664 | 12:91130760 | G/A | 0.272 | -0.0168 (0.0021) | 2.7E-15 | 0.0394 (0.0225) | 0.080 | 64.0 | -2.345 |
| Appendicular lean mass | All-cause dementia (FinnGen) | rs3121580 | 1:218492121 | C/T | 0.827 | 0.0197 (0.0025) | 2.7E-15 | -0.0242 (0.0242) | 0.317 | 62.1 | -1.228 |
| Appendicular lean mass | All-cause dementia (FinnGen) | rs7516643 | 1:33873010 | C/T | 0.672 | 0.0157 (0.0020) | 3.1E-15 | -0.0026 (0.0203) | 0.898 | 61.6 | -0.166 |
| Appendicular lean mass | All-cause dementia (FinnGen) | rs7228151 | 18:57181694 | C/T | 0.206 | -0.0185 (0.0023) | 3.2E-15 | 0.0189 (0.0217) | 0.383 | 64.7 | -1.022 |
| Appendicular lean mass | All-cause dementia (FinnGen) | rs35436119 | 9:119303347 | A/G | 0.075 | 0.0282 (0.0036) | 3.3E-15 | -0.0176 (0.0556) | 0.751 | 61.4 | -0.624 |
| Appendicular lean mass | All-cause dementia (FinnGen) | rs4903321 | 14:75911083 | A/G | 0.444 | -0.0151 (0.0019) | 3.4E-15 | 0.0200 (0.0192) | 0.297 | 63.2 | -1.325 |
| Appendicular lean mass | All-cause dementia (FinnGen) | rs11991823 | 8:92188440 | G/A | 0.679 | 0.0161 (0.0020) | 3.5E-15 | 0.0186 (0.0199) | 0.348 | 64.8 | 1.155 |
| Appendicular lean mass | All-cause dementia (FinnGen) | rs72829852 | 17:46633974 | T/C | 0.062 | 0.0309 (0.0039) | 3.7E-15 | -0.0006 (0.0382) | 0.988 | 62.8 | -0.019 |
| Appendicular lean mass | All-cause dementia (FinnGen) | rs13170063 | 5:157895013 | A/G | 0.592 | -0.0152 (0.0019) | 4.1E-15 | 0.0198 (0.0202) | 0.327 | 64.0 | -1.303 |
| Appendicular lean mass | All-cause dementia (FinnGen) | rs10822117 | 10:52786701 | G/A | 0.237 | -0.0176 (0.0022) | 4.2E-15 | 0.0334 (0.0220) | 0.129 | 64.0 | -1.898 |
| Appendicular lean mass | All-cause dementia (FinnGen) | rs7761910 | 6:155553832 | T/C | 0.309 | -0.0160 (0.0020) | 5.1E-15 | -0.0128 (0.0205) | 0.531 | 64.0 | 0.800 |
| Appendicular lean mass | All-cause dementia (FinnGen) | rs10796828 | 11:69490346 | G/T | 0.635 | 0.0154 (0.0020) | 5.8E-15 | 0.0493 (0.0220) | 0.025 | 59.3 | 3.201 |
| Appendicular lean mass | All-cause dementia (FinnGen) | rs28817902 | 5:138916010 | G/A | 0.118 | 0.0230 (0.0029) | 6.2E-15 | 0.0165 (0.0386) | 0.669 | 62.9 | 0.717 |
| Appendicular lean mass | All-cause dementia (FinnGen) | rs11021305 | 11:95498269 | C/T | 0.384 | 0.0152 (0.0019) | 6.3E-15 | -0.0088 (0.0220) | 0.691 | 64.0 | -0.579 |
| Appendicular lean mass | All-cause dementia (FinnGen) | rs34786000 | 19:9942123 | T/G | 0.554 | -0.0150 (0.0019) | 6.6E-15 | -0.0206 (0.0193) | 0.286 | 62.3 | 1.373 |
| Appendicular lean mass | All-cause dementia (FinnGen) | rs62501195 | 8:24041988 | C/A | 0.171 | -0.0198 (0.0025) | 8.1E-15 | 0.0118 (0.0227) | 0.603 | 62.7 | -0.596 |
| Appendicular lean mass | All-cause dementia (FinnGen) | rs4776624 | 15:73359881 | T/C | 0.397 | -0.0150 (0.0019) | 1.2E-14 | -0.0052 (0.0205) | 0.799 | 62.3 | 0.347 |
| Appendicular lean mass | All-cause dementia (FinnGen) | rs7320878 | 13:91994132 | A/G | 0.603 | -0.0150 (0.0019) | 1.3E-14 | -0.0073 (0.0191) | 0.703 | 62.3 | 0.487 |
| Appendicular lean mass | All-cause dementia (FinnGen) | rs9910161 | 17:59606244 | G/A | 0.285 | 0.0162 (0.0021) | 1.6E-14 | -0.0335 (0.0218) | 0.124 | 59.5 | -2.068 |
| Appendicular lean mass | All-cause dementia (FinnGen) | rs1035583 | 2:207326937 | A/G | 0.618 | 0.0148 (0.0019) | 2.0E-14 | -0.0268 (0.0192) | 0.162 | 60.7 | -1.811 |
| Appendicular lean mass | All-cause dementia (FinnGen) | rs9394578 | 6:39165859 | A/C | 0.258 | -0.0164 (0.0022) | 2.6E-14 | 0.0199 (0.0244) | 0.415 | 55.6 | -1.213 |
| Appendicular lean mass | All-cause dementia (FinnGen) | rs73382475 | 21:47549715 | T/C | 0.089 | 0.0254 (0.0033) | 3.0E-14 | -0.0358 (0.0344) | 0.298 | 59.2 | -1.409 |
| Appendicular lean mass | All-cause dementia (FinnGen) | rs6721191 | 2:10190115 | G/A | 0.578 | -0.0144 (0.0019) | 3.2E-14 | 0.0095 (0.0216) | 0.660 | 57.4 | -0.660 |
| Appendicular lean mass | All-cause dementia (FinnGen) | rs2609334 | 6:168847175 | C/T | 0.226 | -0.0171 (0.0022) | 3.4E-14 | -0.0154 (0.0205) | 0.452 | 60.4 | 0.901 |
| Appendicular lean mass | All-cause dementia (FinnGen) | rs9525326 | 13:115075715 | G/A | 0.188 | -0.0184 (0.0024) | 3.6E-14 | -0.0142 (0.0244) | 0.559 | 58.8 | 0.772 |
| Appendicular lean mass | All-cause dementia (FinnGen) | rs11673381 | 19:4506296 | G/A | 0.530 | -0.0144 (0.0019) | 3.7E-14 | -0.0136 (0.0192) | 0.478 | 57.4 | 0.944 |
| Appendicular lean mass | All-cause dementia (FinnGen) | rs74379684 | 15:94050205 | T/C | 0.075 | -0.0272 (0.0036) | 4.4E-14 | -0.0189 (0.0338) | 0.577 | 57.1 | 0.695 |
| Appendicular lean mass | All-cause dementia (FinnGen) | rs12662115 | 6:169360124 | G/A | 0.327 | -0.0152 (0.0020) | 4.5E-14 | -0.0093 (0.0194) | 0.634 | 57.8 | 0.612 |
| Appendicular lean mass | All-cause dementia (FinnGen) | rs165849 | 22:19958669 | A/G | 0.698 | 0.0157 (0.0021) | 4.6E-14 | 0.0239 (0.0204) | 0.241 | 55.9 | 1.522 |
| Appendicular lean mass | All-cause dementia (FinnGen) | rs11198591 | 10:120515892 | A/G | 0.368 | 0.0148 (0.0020) | 4.9E-14 | -0.0052 (0.0199) | 0.796 | 54.8 | -0.351 |
| Appendicular lean mass | All-cause dementia (FinnGen) | rs7286917 | 22:39860868 | G/A | 0.746 | 0.0171 (0.0023) | 5.2E-14 | 0.0025 (0.0219) | 0.910 | 55.3 | 0.146 |
| Appendicular lean mass | All-cause dementia (FinnGen) | rs1557341 | 18:35127427 | C/A | 0.667 | 0.0153 (0.0020) | 5.8E-14 | 0.0103 (0.0214) | 0.630 | 58.5 | 0.673 |
| Appendicular lean mass | All-cause dementia (FinnGen) | rs599004 | 6:140439740 | T/C | 0.281 | -0.0157 (0.0021) | 6.5E-14 | -0.0188 (0.0234) | 0.421 | 55.9 | 1.197 |
| Appendicular lean mass | All-cause dementia (FinnGen) | rs1290786 | 3:169097381 | T/C | 0.431 | -0.0143 (0.0019) | 7.1E-14 | -0.0108 (0.0195) | 0.580 | 56.6 | 0.755 |
| Appendicular lean mass | All-cause dementia (FinnGen) | rs4622329 | 12:102321935 | A/G | 0.350 | 0.0149 (0.0020) | 8.6E-14 | 0.0132 (0.0194) | 0.495 | 55.5 | 0.886 |
| Appendicular lean mass | All-cause dementia (FinnGen) | rs73040028 | 3:23171805 | T/C | 0.229 | 0.0168 (0.0022) | 9.0E-14 | -0.0018 (0.0218) | 0.936 | 58.3 | -0.107 |
| Appendicular lean mass | All-cause dementia (FinnGen) | rs10810474 | 9:15911359 | T/C | 0.418 | 0.0143 (0.0019) | 9.6E-14 | 0.0024 (0.0192) | 0.900 | 56.6 | 0.168 |
| Appendicular lean mass | All-cause dementia (FinnGen) | rs2232460 | 1:6659505 | A/G | 0.334 | 0.0148 (0.0020) | 1.0E-13 | 0.0074 (0.0213) | 0.729 | 54.8 | 0.500 |
| Appendicular lean mass | All-cause dementia (FinnGen) | rs3760456 | 17:27948844 | T/C | 0.440 | -0.0142 (0.0019) | 1.1E-13 | 0.0011 (0.0191) | 0.954 | 55.9 | -0.077 |
| Appendicular lean mass | All-cause dementia (FinnGen) | rs2807339 | 1:22578063 | C/T | 0.759 | 0.0162 (0.0022) | 1.2E-13 | -0.0091 (0.0229) | 0.692 | 54.2 | -0.562 |
| Appendicular lean mass | All-cause dementia (FinnGen) | rs3798429 | 6:76604573 | G/T | 0.498 | 0.0140 (0.0019) | 1.3E-13 | 0.0011 (0.0192) | 0.955 | 54.3 | 0.079 |
| Appendicular lean mass | All-cause dementia (FinnGen) | rs78051210 | 6:131379491 | C/T | 0.077 | 0.0263 (0.0036) | 1.6E-13 | 0.0047 (0.0467) | 0.920 | 53.4 | 0.179 |
| Appendicular lean mass | All-cause dementia (FinnGen) | rs11927331 | 3:32937008 | A/C | 0.331 | -0.0147 (0.0020) | 1.9E-13 | -0.0044 (0.0200) | 0.826 | 54.0 | 0.299 |
| Appendicular lean mass | All-cause dementia (FinnGen) | rs17400325 | 2:178565913 | C/T | 0.042 | 0.0345 (0.0047) | 2.1E-13 | -0.0709 (0.0672) | 0.291 | 53.9 | -2.055 |
| Appendicular lean mass | All-cause dementia (FinnGen) | rs12344515 | 9:113801231 | T/C | 0.240 | -0.0163 (0.0022) | 2.3E-13 | 0.0003 (0.0235) | 0.990 | 54.9 | -0.018 |
| Appendicular lean mass | All-cause dementia (FinnGen) | rs7225068 | 17:60666425 | G/A | 0.591 | 0.0141 (0.0019) | 2.5E-13 | 0.0087 (0.0194) | 0.653 | 55.1 | 0.617 |
| Appendicular lean mass | All-cause dementia (FinnGen) | rs35631698 | 21:16549469 | GC/G | 0.375 | 0.0145 (0.0020) | 2.6E-13 | -0.0159 (0.0193) | 0.410 | 52.6 | -1.097 |
| Appendicular lean mass | All-cause dementia (FinnGen) | rs2138374 | 2:190014317 | C/T | 0.696 | -0.0149 (0.0020) | 2.8E-13 | 0.0340 (0.0202) | 0.093 | 55.5 | -2.282 |
| Appendicular lean mass | All-cause dementia (FinnGen) | rs35268848 | 16:67927240 | A/C | 0.012 | 0.0737 (0.0101) | 2.8E-13 | -0.0391 (0.0662) | 0.555 | 53.2 | -0.531 |
| Appendicular lean mass | All-cause dementia (FinnGen) | rs147233090 | 15:44028047 | T/C | 0.025 | -0.0446 (0.0061) | 4.0E-13 | 0.0595 (0.1365) | 0.663 | 53.5 | -1.334 |
| Appendicular lean mass | All-cause dementia (FinnGen) | rs4733775 | 8:131213570 | A/G | 0.364 | 0.0143 (0.0020) | 4.1E-13 | -0.0156 (0.0202) | 0.439 | 51.1 | -1.091 |
| Appendicular lean mass | All-cause dementia (FinnGen) | rs610694 | 12:121304826 | C/T | 0.490 | 0.0136 (0.0019) | 4.3E-13 | -0.0239 (0.0192) | 0.213 | 51.2 | -1.757 |
| Appendicular lean mass | All-cause dementia (FinnGen) | rs5753518 | 22:31631314 | A/G | 0.090 | 0.0242 (0.0033) | 5.0E-13 | -0.0051 (0.0278) | 0.853 | 53.8 | -0.211 |
| Appendicular lean mass | All-cause dementia (FinnGen) | rs72976986 | 19:4050424 | A/G | 0.190 | 0.0176 (0.0024) | 5.3E-13 | 0.0300 (0.0262) | 0.251 | 53.8 | 1.705 |
| Appendicular lean mass | All-cause dementia (FinnGen) | rs56207248 | 2:25065986 | T/C | 0.066 | -0.0273 (0.0038) | 6.0E-13 | -0.0228 (0.0403) | 0.571 | 51.6 | 0.835 |
| Appendicular lean mass | All-cause dementia (FinnGen) | rs1487441 | 6:98553894 | A/G | 0.484 | 0.0136 (0.0019) | 7.5E-13 | 0.0117 (0.0191) | 0.542 | 51.2 | 0.860 |
| Appendicular lean mass | All-cause dementia (FinnGen) | rs1216743 | 11:100573120 | A/G | 0.719 | -0.0152 (0.0021) | 8.0E-13 | 0.0021 (0.0214) | 0.922 | 52.4 | -0.138 |
| Appendicular lean mass | All-cause dementia (FinnGen) | rs6975015 | 7:50617215 | A/G | 0.117 | 0.0211 (0.0029) | 8.0E-13 | -0.0326 (0.0330) | 0.323 | 52.9 | -1.545 |
| Appendicular lean mass | All-cause dementia (FinnGen) | rs10453441 | 22:46363739 | G/A | 0.402 | -0.0139 (0.0020) | 9.1E-13 | 0.0226 (0.0205) | 0.269 | 48.3 | -1.626 |
| Appendicular lean mass | All-cause dementia (FinnGen) | rs1330826 | 9:85129970 | C/G | 0.227 | 0.0162 (0.0023) | 1.0E-12 | 0.0380 (0.0221) | 0.086 | 49.6 | 2.346 |
| Appendicular lean mass | All-cause dementia (FinnGen) | rs55758152 | 5:171317318 | A/G | 0.326 | 0.0145 (0.0020) | 1.1E-12 | -0.0137 (0.0203) | 0.501 | 52.6 | -0.945 |
| Appendicular lean mass | All-cause dementia (FinnGen) | rs143076454 | 16:921179 | A/G | 0.019 | -0.0499 (0.0070) | 1.1E-12 | -0.0350 (0.0823) | 0.671 | 50.8 | 0.701 |
| Appendicular lean mass | All-cause dementia (FinnGen) | rs60408354 | 7:70158495 | A/G | 0.073 | 0.0259 (0.0036) | 1.2E-12 | -0.0553 (0.0355) | 0.120 | 51.8 | -2.135 |
| Appendicular lean mass | All-cause dementia (FinnGen) | rs853168 | 5:142538764 | C/G | 0.247 | 0.0156 (0.0022) | 1.3E-12 | 0.0002 (0.0238) | 0.995 | 50.3 | 0.013 |
| Appendicular lean mass | All-cause dementia (FinnGen) | rs72771070 | 16:19993750 | T/C | 0.283 | 0.0150 (0.0021) | 1.3E-12 | 0.0062 (0.0203) | 0.761 | 51.0 | 0.413 |
| Appendicular lean mass | All-cause dementia (FinnGen) | rs12672217 | 7:156310948 | A/G | 0.361 | 0.0139 (0.0020) | 1.3E-12 | 0.0101 (0.0193) | 0.599 | 48.3 | 0.727 |
| Appendicular lean mass | All-cause dementia (FinnGen) | rs2578565 | 5:5460569 | T/C | 0.657 | -0.0141 (0.0020) | 1.4E-12 | 0.0003 (0.0196) | 0.988 | 49.7 | -0.021 |
| Appendicular lean mass | All-cause dementia (FinnGen) | rs572474 | 10:89791654 | G/A | 0.166 | 0.0180 (0.0025) | 1.5E-12 | 0.0071 (0.0257) | 0.781 | 51.8 | 0.394 |
| Appendicular lean mass | All-cause dementia (FinnGen) | rs17197114 | 14:21894526 | C/T | 0.177 | 0.0177 (0.0025) | 1.5E-12 | -0.0835 (0.0268) | 0.002 | 50.1 | -4.718 |
| Appendicular lean mass | All-cause dementia (FinnGen) | rs2289976 | 2:85818797 | A/G | 0.305 | 0.0144 (0.0020) | 1.6E-12 | -0.0418 (0.0226) | 0.065 | 51.8 | -2.903 |
| Appendicular lean mass | All-cause dementia (FinnGen) | rs4847378 | 1:93324634 | T/G | 0.612 | 0.0136 (0.0019) | 1.6E-12 | -0.0251 (0.0196) | 0.200 | 51.2 | -1.846 |
| Appendicular lean mass | All-cause dementia (FinnGen) | rs73114154 | 20:31449386 | T/C | 0.388 | 0.0138 (0.0020) | 1.7E-12 | 0.0328 (0.0193) | 0.089 | 47.6 | 2.377 |
| Appendicular lean mass | All-cause dementia (FinnGen) | rs7107356 | 11:47676170 | G/A | 0.507 | 0.0133 (0.0019) | 1.9E-12 | 0.0053 (0.0196) | 0.788 | 49.0 | 0.399 |
| Appendicular lean mass | All-cause dementia (FinnGen) | rs6129229 | 20:37882317 | G/A | 0.075 | -0.0257 (0.0036) | 1.9E-12 | 0.0309 (0.0357) | 0.387 | 51.0 | -1.202 |
| Appendicular lean mass | All-cause dementia (FinnGen) | rs177592 | 7:28559785 | C/G | 0.101 | -0.0220 (0.0031) | 2.0E-12 | -0.0221 (0.0352) | 0.529 | 50.4 | 1.005 |
| Appendicular lean mass | All-cause dementia (FinnGen) | rs2607234 | 19:35563834 | G/A | 0.948 | -0.0302 (0.0043) | 2.0E-12 | 0.0125 (0.0414) | 0.762 | 49.3 | -0.414 |
| Appendicular lean mass | All-cause dementia (FinnGen) | rs1977337 | 1:67419517 | C/T | 0.851 | 0.0186 (0.0026) | 2.1E-12 | -0.0174 (0.0230) | 0.448 | 51.2 | -0.935 |
| Appendicular lean mass | All-cause dementia (FinnGen) | rs6693481 | 1:203766395 | C/T | 0.695 | -0.0143 (0.0020) | 2.2E-12 | -0.0306 (0.0200) | 0.126 | 51.1 | 2.140 |
| Appendicular lean mass | All-cause dementia (FinnGen) | rs7574162 | 2:172133086 | T/C | 0.757 | 0.0153 (0.0022) | 2.4E-12 | -0.0180 (0.0231) | 0.435 | 48.4 | -1.176 |
| Appendicular lean mass | All-cause dementia (FinnGen) | rs310796 | 12:77453226 | T/G | 0.681 | 0.0142 (0.0020) | 2.5E-12 | 0.0016 (0.0217) | 0.942 | 50.4 | 0.113 |
| Appendicular lean mass | All-cause dementia (FinnGen) | rs11720869 | 3:185619716 | A/G | 0.669 | 0.0141 (0.0020) | 2.5E-12 | -0.0306 (0.0194) | 0.115 | 49.7 | -2.170 |
| Appendicular lean mass | All-cause dementia (FinnGen) | rs1717720 | 7:38112615 | G/A | 0.654 | 0.0139 (0.0020) | 2.7E-12 | -0.0304 (0.0217) | 0.162 | 48.3 | -2.187 |
| Appendicular lean mass | All-cause dementia (FinnGen) | rs75022676 | 2:60293216 | A/G | 0.208 | -0.0163 (0.0023) | 2.8E-12 | -0.0108 (0.0218) | 0.620 | 50.2 | 0.663 |
| Appendicular lean mass | All-cause dementia (FinnGen) | rs76488803 | 4:157770271 | A/G | 0.083 | -0.0240 (0.0034) | 3.0E-12 | -0.0531 (0.0427) | 0.214 | 49.8 | 2.212 |
| Appendicular lean mass | All-cause dementia (FinnGen) | rs7692387 | 4:156635309 | A/G | 0.185 | 0.0169 (0.0024) | 3.5E-12 | 0.0277 (0.0224) | 0.217 | 49.6 | 1.639 |
| Appendicular lean mass | All-cause dementia (FinnGen) | rs112873218 | 11:1960119 | T/C | 0.105 | 0.0216 (0.0031) | 4.0E-12 | -0.0185 (0.0299) | 0.536 | 48.5 | -0.856 |
| Appendicular lean mass | All-cause dementia (FinnGen) | rs7259285 | 19:17153149 | A/G | 0.444 | -0.0132 (0.0019) | 5.1E-12 | -0.0318 (0.0191) | 0.097 | 48.3 | 2.409 |
| Appendicular lean mass | All-cause dementia (FinnGen) | rs36052389 | 4:39357183 | AT/A | 0.455 | 0.0132 (0.0019) | 5.3E-12 | 0.0171 (0.0196) | 0.381 | 48.3 | 1.295 |
| Appendicular lean mass | All-cause dementia (FinnGen) | rs9610447 | 22:20768891 | T/C | 0.747 | 0.0152 (0.0022) | 5.3E-12 | -0.0060 (0.0230) | 0.796 | 47.7 | -0.395 |
| Appendicular lean mass | All-cause dementia (FinnGen) | rs2925155 | 8:75886297 | T/C | 0.261 | -0.0150 (0.0022) | 5.5E-12 | -0.0141 (0.0215) | 0.512 | 46.5 | 0.940 |
| Appendicular lean mass | All-cause dementia (FinnGen) | rs17681189 | 2:65976175 | A/C | 0.423 | -0.0131 (0.0019) | 5.8E-12 | 0.0261 (0.0193) | 0.174 | 47.5 | -1.992 |
| Appendicular lean mass | All-cause dementia (FinnGen) | rs7679276 | 4:146860186 | G/A | 0.954 | -0.0330 (0.0048) | 5.9E-12 | -0.0665 (0.0834) | 0.425 | 47.3 | 2.015 |
| Appendicular lean mass | All-cause dementia (FinnGen) | rs75478182 | 20:50807839 | C/G | 0.088 | 0.0230 (0.0033) | 6.6E-12 | -0.0567 (0.0382) | 0.138 | 48.6 | -2.465 |
| Appendicular lean mass | All-cause dementia (FinnGen) | rs2343034 | 20:35860288 | A/T | 0.809 | 0.0166 (0.0024) | 6.7E-12 | 0.0252 (0.0209) | 0.226 | 47.8 | 1.518 |
| Appendicular lean mass | All-cause dementia (FinnGen) | rs10427685 | 22:46440359 | G/C | 0.064 | 0.0269 (0.0039) | 7.0E-12 | -0.0180 (0.0410) | 0.660 | 47.6 | -0.669 |
| Appendicular lean mass | All-cause dementia (FinnGen) | rs12463908 | 2:121470971 | C/A | 0.188 | -0.0165 (0.0024) | 7.3E-12 | -0.0446 (0.0238) | 0.061 | 47.3 | 2.703 |
| Appendicular lean mass | All-cause dementia (FinnGen) | rs10008637 | 4:77414144 | C/T | 0.462 | -0.0130 (0.0019) | 7.5E-12 | -0.0038 (0.0192) | 0.844 | 46.8 | 0.292 |
| Appendicular lean mass | All-cause dementia (FinnGen) | rs2125125 | 3:142600087 | C/T | 0.783 | 0.0158 (0.0023) | 7.8E-12 | 0.0253 (0.0265) | 0.339 | 47.2 | 1.601 |
| Appendicular lean mass | All-cause dementia (FinnGen) | rs61827272 | 1:203810763 | C/T | 0.275 | 0.0144 (0.0021) | 7.9E-12 | 0.0196 (0.0209) | 0.347 | 47.0 | 1.361 |
| Appendicular lean mass | All-cause dementia (FinnGen) | rs78444298 | 1:184672098 | A/G | 0.020 | -0.0465 (0.0068) | 8.3E-12 | -0.0897 (0.0743) | 0.228 | 46.8 | 1.929 |
| Appendicular lean mass | All-cause dementia (FinnGen) | rs116493405 | 3:114733556 | A/G | 0.054 | 0.0287 (0.0042) | 9.5E-12 | 0.1300 (0.0568) | 0.022 | 46.7 | 4.530 |
| Appendicular lean mass | All-cause dementia (FinnGen) | rs61919240 | 12:8831954 | A/T | 0.324 | 0.0137 (0.0020) | 9.8E-12 | 0.0407 (0.0210) | 0.053 | 46.9 | 2.971 |
| Appendicular lean mass | All-cause dementia (FinnGen) | rs963317 | 4:45129970 | G/A | 0.663 | -0.0136 (0.0020) | 1.0E-11 | -0.0020 (0.0205) | 0.922 | 46.2 | 0.147 |
| Appendicular lean mass | All-cause dementia (FinnGen) | rs945508 | 1:156907081 | C/T | 0.536 | -0.0128 (0.0019) | 1.1E-11 | -0.0062 (0.0192) | 0.748 | 45.4 | 0.484 |
| Appendicular lean mass | All-cause dementia (FinnGen) | rs67625472 | 19:4968620 | C/T | 0.276 | -0.0144 (0.0021) | 1.3E-11 | -0.0126 (0.0223) | 0.570 | 47.0 | 0.875 |
| Appendicular lean mass | All-cause dementia (FinnGen) | rs2663126 | 15:99563857 | A/G | 0.691 | -0.0139 (0.0021) | 1.4E-11 | -0.0091 (0.0226) | 0.688 | 43.8 | 0.655 |
| Appendicular lean mass | All-cause dementia (FinnGen) | rs115233595 | 4:119744214 | C/T | 0.062 | -0.0264 (0.0039) | 1.4E-11 | 0.0243 (0.0326) | 0.456 | 45.8 | -0.920 |
| Appendicular lean mass | All-cause dementia (FinnGen) | rs12340775 | 9:13226945 | A/G | 0.055 | -0.0287 (0.0043) | 1.7E-11 | -0.0355 (0.0357) | 0.320 | 44.5 | 1.237 |
| Appendicular lean mass | All-cause dementia (FinnGen) | rs2252031 | 12:103248463 | C/T | 0.846 | -0.0176 (0.0026) | 1.8E-11 | 0.0123 (0.0327) | 0.706 | 45.8 | -0.699 |
| Appendicular lean mass | All-cause dementia (FinnGen) | rs6923230 | 6:88213949 | C/T | 0.534 | -0.0127 (0.0019) | 1.8E-11 | 0.0146 (0.0192) | 0.446 | 44.7 | -1.150 |
| Appendicular lean mass | All-cause dementia (FinnGen) | rs10461725 | 5:39437129 | C/G | 0.656 | 0.0134 (0.0020) | 2.0E-11 | 0.0140 (0.0195) | 0.474 | 44.9 | 1.045 |
| Appendicular lean mass | All-cause dementia (FinnGen) | rs501250 | 5:112155793 | C/G | 0.619 | -0.0130 (0.0019) | 2.1E-11 | 0.0339 (0.0195) | 0.081 | 46.8 | -2.608 |
| Appendicular lean mass | All-cause dementia (FinnGen) | rs10840399 | 11:10399121 | T/C | 0.101 | -0.0209 (0.0031) | 2.8E-11 | 0.0045 (0.0284) | 0.874 | 45.5 | -0.215 |
| Appendicular lean mass | All-cause dementia (FinnGen) | rs7543202 | 1:73872885 | G/A | 0.623 | 0.0129 (0.0019) | 2.9E-11 | 0.0021 (0.0198) | 0.914 | 46.1 | 0.163 |
| Appendicular lean mass | All-cause dementia (FinnGen) | rs2678898 | 2:58171964 | C/T | 0.380 | -0.0129 (0.0019) | 3.1E-11 | 0.0089 (0.0198) | 0.655 | 46.1 | -0.690 |
| Appendicular lean mass | All-cause dementia (FinnGen) | rs2994329 | 1:243647921 | T/C | 0.202 | 0.0155 (0.0023) | 3.1E-11 | 0.0282 (0.0229) | 0.218 | 45.4 | 1.819 |
| Appendicular lean mass | All-cause dementia (FinnGen) | rs75702986 | 19:35566151 | A/G | 0.186 | -0.0163 (0.0025) | 3.1E-11 | 0.0149 (0.0237) | 0.528 | 42.5 | -0.914 |
| Appendicular lean mass | All-cause dementia (FinnGen) | rs2268718 | 6:52415023 | T/C | 0.270 | 0.0141 (0.0021) | 3.2E-11 | 0.0069 (0.0201) | 0.731 | 45.1 | 0.489 |
| Appendicular lean mass | All-cause dementia (FinnGen) | rs8084413 | 18:22869123 | A/G | 0.469 | -0.0127 (0.0019) | 3.2E-11 | -0.0177 (0.0193) | 0.358 | 44.7 | 1.394 |
| Appendicular lean mass | All-cause dementia (FinnGen) | rs34522021 | 9:23350420 | T/C | 0.455 | 0.0126 (0.0019) | 3.4E-11 | 0.0268 (0.0192) | 0.163 | 44.0 | 2.127 |
| Appendicular lean mass | All-cause dementia (FinnGen) | rs137998108 | 12:124322727 | CA/C | 0.133 | -0.0188 (0.0028) | 3.6E-11 | -0.0199 (0.0323) | 0.538 | 45.1 | 1.059 |
| Appendicular lean mass | All-cause dementia (FinnGen) | rs2126942 | 5:98155979 | C/T | 0.580 | -0.0127 (0.0019) | 3.8E-11 | 0.0068 (0.0199) | 0.734 | 44.7 | -0.535 |
| Appendicular lean mass | All-cause dementia (FinnGen) | rs10827415 | 10:35042616 | G/T | 0.261 | -0.0142 (0.0021) | 3.9E-11 | 0.0025 (0.0244) | 0.918 | 45.7 | -0.176 |
| Appendicular lean mass | All-cause dementia (FinnGen) | rs116008080 | 16:67254841 | A/G | 0.024 | -0.0415 (0.0063) | 4.1E-11 | -0.0575 (0.0580) | 0.322 | 43.4 | 1.386 |
| Appendicular lean mass | All-cause dementia (FinnGen) | rs6738207 | 2:105989716 | A/G | 0.401 | 0.0127 (0.0019) | 4.1E-11 | -0.0151 (0.0204) | 0.457 | 44.7 | -1.189 |
| Appendicular lean mass | All-cause dementia (FinnGen) | rs113671109 | 3:12620885 | C/T | 0.220 | -0.0150 (0.0023) | 4.2E-11 | 0.0179 (0.0221) | 0.417 | 42.5 | -1.193 |
| Appendicular lean mass | All-cause dementia (FinnGen) | rs2965074 | 7:45983129 | T/G | 0.452 | 0.0125 (0.0019) | 4.3E-11 | 0.0031 (0.0197) | 0.875 | 43.3 | 0.248 |
| Appendicular lean mass | All-cause dementia (FinnGen) | rs1800504 | 7:50742180 | T/C | 0.552 | -0.0125 (0.0019) | 4.4E-11 | -0.0077 (0.0193) | 0.691 | 43.3 | 0.616 |
| Appendicular lean mass | All-cause dementia (FinnGen) | rs34203781 | 6:41666292 | C/A | 0.137 | 0.0181 (0.0028) | 5.6E-11 | -0.0310 (0.0261) | 0.235 | 41.8 | -1.713 |
| Appendicular lean mass | All-cause dementia (FinnGen) | rs2347808 | 18:2750856 | A/G | 0.514 | -0.0125 (0.0019) | 5.8E-11 | 0.0231 (0.0191) | 0.227 | 43.3 | -1.848 |
| Appendicular lean mass | All-cause dementia (FinnGen) | rs3830008 | 11:117093764 | C/A | 0.204 | -0.0154 (0.0023) | 6.1E-11 | -0.0163 (0.0219) | 0.459 | 44.8 | 1.058 |
| Appendicular lean mass | All-cause dementia (FinnGen) | rs2490302 | 10:37702435 | A/T | 0.914 | 0.0221 (0.0034) | 6.2E-11 | -0.0304 (0.0403) | 0.451 | 42.2 | -1.376 |
| Appendicular lean mass | All-cause dementia (FinnGen) | rs757042 | 7:25662829 | C/T | 0.724 | -0.0140 (0.0021) | 6.3E-11 | 0.0135 (0.0202) | 0.504 | 44.4 | -0.964 |
| Appendicular lean mass | All-cause dementia (FinnGen) | rs8180765 | 7:4680614 | G/A | 0.217 | -0.0151 (0.0023) | 6.5E-11 | -0.0402 (0.0217) | 0.064 | 43.1 | 2.662 |
| Appendicular lean mass | All-cause dementia (FinnGen) | rs11605297 | 11:58296806 | A/G | 0.233 | 0.0146 (0.0022) | 8.0E-11 | -0.0178 (0.0220) | 0.419 | 44.0 | -1.219 |
| Appendicular lean mass | All-cause dementia (FinnGen) | rs496783 | 10:116137961 | G/A | 0.465 | -0.0124 (0.0019) | 8.1E-11 | -0.0104 (0.0201) | 0.603 | 42.6 | 0.839 |
| Appendicular lean mass | All-cause dementia (FinnGen) | rs603486 | 11:118587347 | C/T | 0.617 | 0.0127 (0.0020) | 8.8E-11 | -0.0008 (0.0200) | 0.967 | 40.3 | -0.063 |
| Appendicular lean mass | All-cause dementia (FinnGen) | rs1899040 | 2:223901896 | T/C | 0.796 | 0.0152 (0.0023) | 9.0E-11 | 0.0149 (0.0236) | 0.528 | 43.7 | 0.980 |
| Appendicular lean mass | All-cause dementia (FinnGen) | rs35874463 | 15:67457698 | G/A | 0.058 | 0.0263 (0.0041) | 9.1E-11 | 0.0005 (0.0497) | 0.993 | 41.1 | 0.019 |
| Appendicular lean mass | All-cause dementia (FinnGen) | rs13109280 | 4:54380513 | G/A | 0.662 | 0.0131 (0.0020) | 9.1E-11 | 0.0066 (0.0201) | 0.744 | 42.9 | 0.504 |
| Appendicular lean mass | All-cause dementia (FinnGen) | rs13127468 | 4:8599658 | A/C | 0.463 | -0.0123 (0.0019) | 9.9E-11 | -0.0119 (0.0195) | 0.541 | 41.9 | 0.967 |
| Appendicular lean mass | All-cause dementia (FinnGen) | rs8064946 | 17:7589311 | C/G | 0.110 | -0.0195 (0.0030) | 1.0E-10 | 0.0075 (0.0378) | 0.843 | 42.2 | -0.385 |
| Appendicular lean mass | All-cause dementia (FinnGen) | rs10815304 | 9:5908217 | C/T | 0.206 | 0.0151 (0.0023) | 1.1E-10 | -0.0043 (0.0250) | 0.864 | 43.1 | -0.285 |
| Appendicular lean mass | All-cause dementia (FinnGen) | rs56112295 | 14:105877057 | T/C | 0.226 | 0.0154 (0.0024) | 1.1E-10 | 0.0046 (0.0242) | 0.848 | 41.2 | 0.299 |
| Appendicular lean mass | All-cause dementia (FinnGen) | rs11221657 | 11:129181358 | G/T | 0.135 | 0.0179 (0.0028) | 1.2E-10 | 0.0108 (0.0263) | 0.681 | 40.9 | 0.603 |
| Appendicular lean mass | All-cause dementia (FinnGen) | rs10019221 | 4:21785364 | T/G | 0.597 | -0.0124 (0.0019) | 1.2E-10 | -0.0196 (0.0199) | 0.324 | 42.6 | 1.581 |
| Appendicular lean mass | All-cause dementia (FinnGen) | rs55717234 | 1:150999863 | G/A | 0.573 | 0.0122 (0.0019) | 1.3E-10 | -0.0060 (0.0197) | 0.762 | 41.2 | -0.492 |
| Appendicular lean mass | All-cause dementia (FinnGen) | rs1405227 | 1:98873390 | A/G | 0.317 | 0.0129 (0.0020) | 1.6E-10 | -0.0194 (0.0198) | 0.326 | 41.6 | -1.504 |
| Appendicular lean mass | All-cause dementia (FinnGen) | rs200302461 | 2:9770589 | GCGGGGC/G | 0.259 | 0.0145 (0.0023) | 1.6E-10 | -0.0078 (0.0224) | 0.728 | 39.7 | -0.538 |
| Appendicular lean mass | All-cause dementia (FinnGen) | rs10116740 | 9:133446357 | A/G | 0.477 | -0.0121 (0.0019) | 1.8E-10 | -0.0213 (0.0192) | 0.267 | 40.6 | 1.760 |
| Appendicular lean mass | All-cause dementia (FinnGen) | rs111622870 | 4:2613109 | C/T | 0.048 | -0.0282 (0.0044) | 1.9E-10 | 0.0392 (0.0619) | 0.527 | 41.1 | -1.390 |
| Appendicular lean mass | All-cause dementia (FinnGen) | rs650508 | 2:45880122 | C/G | 0.301 | -0.0130 (0.0020) | 1.9E-10 | -0.0102 (0.0232) | 0.661 | 42.2 | 0.785 |
| Appendicular lean mass | All-cause dementia (FinnGen) | rs6054491 | 20:6709535 | G/C | 0.240 | -0.0142 (0.0022) | 2.2E-10 | 0.0310 (0.0217) | 0.153 | 41.7 | -2.183 |
| Appendicular lean mass | All-cause dementia (FinnGen) | rs79680939 | 17:80523375 | A/G | 0.045 | 0.0290 (0.0046) | 2.5E-10 | 0.0417 (0.0627) | 0.506 | 39.7 | 1.438 |
| Appendicular lean mass | All-cause dementia (FinnGen) | rs56207600 | 11:126196537 | A/G | 0.111 | 0.0192 (0.0030) | 2.5E-10 | -0.0057 (0.0281) | 0.839 | 41.0 | -0.297 |
| Appendicular lean mass | All-cause dementia (FinnGen) | rs9828525 | 3:61552810 | T/C | 0.411 | 0.0121 (0.0019) | 2.5E-10 | -0.0122 (0.0195) | 0.532 | 40.6 | -1.008 |
| Appendicular lean mass | All-cause dementia (FinnGen) | rs4682483 | 3:112993982 | A/G | 0.153 | -0.0165 (0.0026) | 2.7E-10 | 0.0086 (0.0217) | 0.692 | 40.3 | -0.521 |
| Appendicular lean mass | All-cause dementia (FinnGen) | rs113146332 | 17:42565977 | C/G | 0.038 | 0.0311 (0.0049) | 3.0E-10 | -0.0305 (0.0873) | 0.727 | 40.3 | -0.981 |
| Appendicular lean mass | All-cause dementia (FinnGen) | rs7030071 | 9:136951556 | G/A | 0.311 | -0.0130 (0.0021) | 3.1E-10 | -0.0302 (0.0200) | 0.132 | 38.3 | 2.323 |
| Appendicular lean mass | All-cause dementia (FinnGen) | rs11410717 | 7:116252956 | TG/T | 0.117 | 0.0185 (0.0029) | 3.1E-10 | -0.0511 (0.0269) | 0.058 | 40.7 | -2.762 |
| Appendicular lean mass | All-cause dementia (FinnGen) | rs34995241 | 6:83631850 | AT/A | 0.708 | -0.0131 (0.0021) | 3.2E-10 | 0.0154 (0.0217) | 0.477 | 38.9 | -1.176 |
| Appendicular lean mass | All-cause dementia (FinnGen) | rs77299262 | 3:187450067 | C/T | 0.067 | 0.0237 (0.0038) | 3.4E-10 | 0.0728 (0.0348) | 0.037 | 38.9 | 3.072 |
| Appendicular lean mass | All-cause dementia (FinnGen) | rs1407204 | 6:119289835 | A/C | 0.810 | -0.0151 (0.0024) | 3.5E-10 | -0.0258 (0.0285) | 0.364 | 39.6 | 1.709 |
| Appendicular lean mass | All-cause dementia (FinnGen) | rs11191208 | 10:103838497 | A/G | 0.206 | 0.0147 (0.0024) | 3.7E-10 | 0.0002 (0.0237) | 0.995 | 37.5 | 0.014 |
| Appendicular lean mass | All-cause dementia (FinnGen) | rs11684531 | 2:219835489 | G/A | 0.133 | -0.0172 (0.0028) | 4.2E-10 | -0.0582 (0.0379) | 0.125 | 37.7 | 3.384 |
| Appendicular lean mass | All-cause dementia (FinnGen) | rs2823990 | 21:18081151 | A/G | 0.677 | -0.0127 (0.0020) | 4.2E-10 | 0.0161 (0.0198) | 0.417 | 40.3 | -1.268 |
| Appendicular lean mass | All-cause dementia (FinnGen) | rs3792819 | 5:172576296 | G/A | 0.085 | 0.0210 (0.0034) | 4.4E-10 | 0.0872 (0.0530) | 0.100 | 38.1 | 4.152 |
| Appendicular lean mass | All-cause dementia (FinnGen) | rs10762866 | 10:81228430 | A/G | 0.138 | 0.0171 (0.0028) | 5.4E-10 | -0.0102 (0.0247) | 0.680 | 37.3 | -0.596 |
| Appendicular lean mass | All-cause dementia (FinnGen) | rs7359097 | 14:54757497 | C/T | 0.396 | -0.0121 (0.0019) | 5.5E-10 | -0.0148 (0.0195) | 0.446 | 40.6 | 1.223 |
| Appendicular lean mass | All-cause dementia (FinnGen) | rs10112506 | 8:13164746 | G/A | 0.390 | -0.0120 (0.0019) | 5.8E-10 | -0.0036 (0.0207) | 0.862 | 39.9 | 0.300 |
| Appendicular lean mass | All-cause dementia (FinnGen) | rs2974337 | 8:42390873 | T/C | 0.500 | 0.0117 (0.0019) | 5.9E-10 | 0.0188 (0.0192) | 0.328 | 37.9 | 1.607 |
| Appendicular lean mass | All-cause dementia (FinnGen) | rs11421589 | 10:115193949 | CT/C | 0.556 | 0.0119 (0.0019) | 6.0E-10 | -0.0372 (0.0193) | 0.054 | 39.2 | -3.126 |
| Appendicular lean mass | All-cause dementia (FinnGen) | rs7144307 | 14:69533837 | C/T | 0.385 | -0.0122 (0.0020) | 6.1E-10 | 0.0212 (0.0195) | 0.277 | 37.2 | -1.738 |
| Appendicular lean mass | All-cause dementia (FinnGen) | rs4754296 | 11:108006182 | G/T | 0.146 | -0.0167 (0.0027) | 6.1E-10 | -0.0039 (0.0229) | 0.866 | 38.3 | 0.234 |
| Appendicular lean mass | All-cause dementia (FinnGen) | rs139921635 | 4:73181637 | T/G | 0.024 | 0.0385 (0.0062) | 6.2E-10 | 0.0651 (0.0554) | 0.240 | 38.6 | 1.691 |
| Appendicular lean mass | All-cause dementia (FinnGen) | rs10241451 | 7:55248926 | C/T | 0.209 | 0.0145 (0.0023) | 6.3E-10 | -0.0019 (0.0243) | 0.936 | 39.7 | -0.131 |
| Appendicular lean mass | All-cause dementia (FinnGen) | rs447352 | 5:678750 | T/C | 0.141 | -0.0181 (0.0029) | 6.6E-10 | -0.0153 (0.0320) | 0.632 | 39.0 | 0.845 |
| Appendicular lean mass | All-cause dementia (FinnGen) | rs718603 | 6:2644245 | T/C | 0.277 | 0.0131 (0.0021) | 6.7E-10 | -0.0369 (0.0214) | 0.085 | 38.9 | -2.817 |
| Appendicular lean mass | All-cause dementia (FinnGen) | rs2875409 | 13:41112849 | A/G | 0.050 | -0.0270 (0.0044) | 7.1E-10 | 0.0441 (0.0503) | 0.381 | 37.6 | -1.633 |
| Appendicular lean mass | All-cause dementia (FinnGen) | rs839255 | 3:57974580 | G/T | 0.685 | -0.0126 (0.0021) | 8.4E-10 | -0.0192 (0.0207) | 0.355 | 36.0 | 1.524 |
| Appendicular lean mass | All-cause dementia (FinnGen) | rs10849576 | 12:993652 | G/T | 0.410 | -0.0117 (0.0019) | 9.2E-10 | -0.0082 (0.0206) | 0.688 | 37.9 | 0.701 |
| Appendicular lean mass | All-cause dementia (FinnGen) | rs11688284 | 2:174738951 | T/C | 0.388 | -0.0119 (0.0019) | 9.8E-10 | 0.0098 (0.0200) | 0.623 | 39.2 | -0.824 |
| Appendicular lean mass | All-cause dementia (FinnGen) | rs116339650 | 4:26200972 | G/A | 0.127 | -0.0175 (0.0029) | 1.1E-09 | 0.0069 (0.0259) | 0.792 | 36.4 | -0.394 |
| Appendicular lean mass | All-cause dementia (FinnGen) | rs670129 | 20:10460858 | T/C | 0.413 | -0.0118 (0.0019) | 1.1E-09 | 0.0318 (0.0202) | 0.117 | 38.6 | -2.695 |
| Appendicular lean mass | All-cause dementia (FinnGen) | rs7328187 | 13:74189974 | G/T | 0.498 | 0.0116 (0.0019) | 1.2E-09 | -0.0095 (0.0195) | 0.625 | 37.3 | -0.819 |
| Appendicular lean mass | All-cause dementia (FinnGen) | rs62143873 | 2:72035050 | A/G | 0.503 | -0.0115 (0.0019) | 1.2E-09 | -0.0299 (0.0194) | 0.124 | 36.6 | 2.600 |
| Appendicular lean mass | All-cause dementia (FinnGen) | rs6469845 | 8:120792195 | T/C | 0.239 | 0.0135 (0.0022) | 1.2E-09 | -0.0144 (0.0210) | 0.494 | 37.6 | -1.067 |
| Appendicular lean mass | All-cause dementia (FinnGen) | rs4940874 | 18:57105638 | G/A | 0.812 | 0.0148 (0.0024) | 1.2E-09 | 0.0543 (0.0228) | 0.017 | 38.0 | 3.669 |
| Appendicular lean mass | All-cause dementia (FinnGen) | rs75172776 | 16:9001814 | G/A | 0.066 | 0.0232 (0.0038) | 1.3E-09 | -0.0642 (0.0467) | 0.170 | 37.3 | -2.767 |
| Appendicular lean mass | All-cause dementia (FinnGen) | rs61925210 | 12:43974800 | T/A | 0.105 | 0.0187 (0.0031) | 1.3E-09 | -0.0529 (0.0300) | 0.078 | 36.4 | -2.829 |
| Appendicular lean mass | All-cause dementia (FinnGen) | rs4932439 | 15:89401109 | G/A | 0.825 | -0.0151 (0.0025) | 1.4E-09 | 0.0098 (0.0213) | 0.644 | 36.5 | -0.649 |
| Appendicular lean mass | All-cause dementia (FinnGen) | rs249681 | 5:141538017 | C/G | 0.618 | -0.0117 (0.0019) | 1.5E-09 | -0.0127 (0.0201) | 0.526 | 37.9 | 1.085 |
| Appendicular lean mass | All-cause dementia (FinnGen) | rs616488 | 1:10566215 | G/A | 0.345 | 0.0120 (0.0020) | 1.5E-09 | -0.0315 (0.0202) | 0.120 | 36.0 | -2.625 |
| Appendicular lean mass | All-cause dementia (FinnGen) | rs17773965 | 2:217631338 | T/C | 0.141 | -0.0163 (0.0027) | 1.5E-09 | 0.0265 (0.0223) | 0.236 | 36.5 | -1.626 |
| Appendicular lean mass | All-cause dementia (FinnGen) | rs4554207 | 5:114388634 | C/T | 0.515 | -0.0114 (0.0019) | 1.6E-09 | -0.0264 (0.0191) | 0.167 | 36.0 | 2.316 |
| Appendicular lean mass | All-cause dementia (FinnGen) | rs1190540 | 14:102897009 | G/A | 0.698 | 0.0125 (0.0021) | 1.6E-09 | -0.0097 (0.0203) | 0.634 | 35.4 | -0.776 |
| Appendicular lean mass | All-cause dementia (FinnGen) | rs1202186 | 7:87213258 | T/C | 0.655 | -0.0120 (0.0020) | 1.7E-09 | -0.0070 (0.0207) | 0.736 | 36.0 | 0.583 |
| Appendicular lean mass | All-cause dementia (FinnGen) | rs62033029 | 16:50107273 | A/G | 0.206 | -0.0141 (0.0023) | 1.7E-09 | 0.0313 (0.0247) | 0.205 | 37.6 | -2.220 |
| Appendicular lean mass | All-cause dementia (FinnGen) | rs13112742 | 4:124888059 | A/G | 0.170 | -0.0151 (0.0025) | 2.0E-09 | -0.0231 (0.0279) | 0.407 | 36.5 | 1.530 |
| Appendicular lean mass | All-cause dementia (FinnGen) | rs680882 | 7:18325278 | G/T | 0.761 | 0.0133 (0.0022) | 2.0E-09 | -0.0224 (0.0221) | 0.310 | 36.5 | -1.684 |
| Appendicular lean mass | All-cause dementia (FinnGen) | rs8017006 | 14:42745052 | G/A | 0.328 | 0.0122 (0.0020) | 2.2E-09 | -0.0228 (0.0217) | 0.292 | 37.2 | -1.869 |
| Appendicular lean mass | All-cause dementia (FinnGen) | rs2569888 | 6:1625803 | A/G | 0.245 | 0.0133 (0.0022) | 2.3E-09 | 0.0221 (0.0222) | 0.320 | 36.5 | 1.662 |
| Appendicular lean mass | All-cause dementia (FinnGen) | rs10779958 | 2:74755778 | C/A | 0.149 | 0.0157 (0.0026) | 2.6E-09 | -0.0431 (0.0275) | 0.117 | 36.5 | -2.745 |
| Appendicular lean mass | All-cause dementia (FinnGen) | rs66613683 | 12:104409025 | C/T | 0.170 | 0.0150 (0.0025) | 2.6E-09 | -0.0207 (0.0273) | 0.448 | 36.0 | -1.380 |
| Appendicular lean mass | All-cause dementia (FinnGen) | rs114018835 | 1:244274455 | T/C | 0.036 | 0.0303 (0.0051) | 2.7E-09 | 0.1141 (0.0810) | 0.159 | 35.3 | 3.766 |
| Appendicular lean mass | All-cause dementia (FinnGen) | rs150474185 | 1:65689032 | G/C | 0.021 | 0.0440 (0.0074) | 2.7E-09 | 0.3003 (0.1154) | 0.009 | 35.4 | 6.825 |
| Appendicular lean mass | All-cause dementia (FinnGen) | rs114192718 | 2:128785663 | T/C | 0.075 | -0.0215 (0.0036) | 2.9E-09 | -0.0364 (0.0453) | 0.422 | 35.7 | 1.693 |
| Appendicular lean mass | All-cause dementia (FinnGen) | rs17713523 | 5:149923968 | A/G | 0.407 | 0.0114 (0.0019) | 3.1E-09 | -0.0429 (0.0192) | 0.025 | 36.0 | -3.763 |
| Appendicular lean mass | All-cause dementia (FinnGen) | rs11175919 | 12:66180277 | A/G | 0.026 | 0.0349 (0.0059) | 3.2E-09 | 0.0084 (0.0586) | 0.886 | 35.0 | 0.241 |
| Appendicular lean mass | All-cause dementia (FinnGen) | rs3118124 | 1:150166675 | G/A | 0.289 | -0.0122 (0.0021) | 3.2E-09 | -0.0174 (0.0220) | 0.428 | 33.8 | 1.426 |
| Appendicular lean mass | All-cause dementia (FinnGen) | rs78838233 | 17:18257589 | T/C | 0.237 | -0.0132 (0.0022) | 3.3E-09 | -0.0159 (0.0218) | 0.465 | 36.0 | 1.205 |
| Appendicular lean mass | All-cause dementia (FinnGen) | rs3769885 | 2:165592390 | A/G | 0.490 | -0.0111 (0.0019) | 3.5E-09 | 0.0091 (0.0193) | 0.637 | 34.1 | -0.820 |
| Appendicular lean mass | All-cause dementia (FinnGen) | rs3003137 | 20:62830802 | T/G | 0.428 | -0.0114 (0.0019) | 3.6E-09 | -0.0211 (0.0200) | 0.292 | 36.0 | 1.851 |
| Appendicular lean mass | All-cause dementia (FinnGen) | rs13316 | 14:93407301 | A/C | 0.430 | 0.0115 (0.0019) | 3.7E-09 | 0.0057 (0.0193) | 0.769 | 36.6 | 0.496 |
| Appendicular lean mass | All-cause dementia (FinnGen) | rs2788213 | 6:703249 | A/G | 0.710 | 0.0123 (0.0021) | 3.8E-09 | 0.0101 (0.0213) | 0.636 | 34.3 | 0.821 |
| Appendicular lean mass | All-cause dementia (FinnGen) | rs7095087 | 10:31120513 | A/G | 0.353 | 0.0117 (0.0020) | 3.8E-09 | 0.0008 (0.0214) | 0.972 | 34.2 | 0.068 |
| Appendicular lean mass | All-cause dementia (FinnGen) | rs603542 | 1:44079411 | C/T | 0.675 | 0.0118 (0.0020) | 3.8E-09 | 0.0216 (0.0203) | 0.287 | 34.8 | 1.831 |
| Appendicular lean mass | All-cause dementia (FinnGen) | rs290793 | 7:130416726 | C/G | 0.188 | 0.0142 (0.0024) | 4.0E-09 | -0.0248 (0.0233) | 0.286 | 35.0 | -1.746 |
| Appendicular lean mass | All-cause dementia (FinnGen) | rs41298373 | 10:7622009 | A/G | 0.101 | 0.0185 (0.0031) | 4.2E-09 | 0.0153 (0.0283) | 0.588 | 35.6 | 0.827 |
| Appendicular lean mass | All-cause dementia (FinnGen) | rs4836544 | 9:129236355 | T/C | 0.885 | 0.0175 (0.0030) | 4.3E-09 | 0.0027 (0.0367) | 0.942 | 34.0 | 0.154 |
| Appendicular lean mass | All-cause dementia (FinnGen) | rs4121583 | 18:125075 | T/C | 0.619 | 0.0118 (0.0020) | 4.6E-09 | -0.0144 (0.0200) | 0.473 | 34.8 | -1.220 |
| Appendicular lean mass | All-cause dementia (FinnGen) | rs113827862 | 14:89849527 | C/T | 0.061 | -0.0235 (0.0040) | 4.7E-09 | 0.0224 (0.0362) | 0.536 | 34.5 | -0.953 |
| Appendicular lean mass | All-cause dementia (FinnGen) | rs2150692 | 13:30109494 | A/C | 0.855 | -0.0158 (0.0027) | 4.8E-09 | -0.0177 (0.0229) | 0.441 | 34.2 | 1.120 |
| Appendicular lean mass | All-cause dementia (FinnGen) | rs9636364 | 2:111992435 | A/G | 0.543 | 0.0110 (0.0019) | 5.1E-09 | 0.0379 (0.0193) | 0.050 | 33.5 | 3.445 |
| Appendicular lean mass | All-cause dementia (FinnGen) | rs577289 | 15:40208911 | T/A | 0.281 | -0.0125 (0.0021) | 5.4E-09 | -0.0159 (0.0209) | 0.445 | 35.4 | 1.272 |
| Appendicular lean mass | All-cause dementia (FinnGen) | rs117972846 | 17:26947476 | G/T | 0.029 | 0.0335 (0.0057) | 5.5E-09 | -0.0355 (0.0587) | 0.545 | 34.5 | -1.060 |
| Appendicular lean mass | All-cause dementia (FinnGen) | rs4652902 | 1:36612955 | A/C | 0.768 | 0.0129 (0.0022) | 5.7E-09 | -0.0436 (0.0212) | 0.040 | 34.4 | -3.380 |
| Appendicular lean mass | All-cause dementia (FinnGen) | rs3103223 | 4:42402721 | C/T | 0.740 | 0.0126 (0.0022) | 6.0E-09 | 0.0104 (0.0225) | 0.644 | 32.8 | 0.825 |
| Appendicular lean mass | All-cause dementia (FinnGen) | rs10060855 | 5:33216769 | G/A | 0.677 | 0.0118 (0.0020) | 6.2E-09 | 0.0265 (0.0198) | 0.181 | 34.8 | 2.246 |
| Appendicular lean mass | All-cause dementia (FinnGen) | rs1527149 | 7:114004088 | G/C | 0.407 | 0.0113 (0.0019) | 6.9E-09 | 0.0030 (0.0197) | 0.879 | 35.4 | 0.265 |
| Appendicular lean mass | All-cause dementia (FinnGen) | rs35701316 | 20:39805449 | GTGTT/G | 0.174 | -0.0145 (0.0025) | 7.0E-09 | -0.0133 (0.0218) | 0.541 | 33.6 | 0.917 |
| Appendicular lean mass | All-cause dementia (FinnGen) | rs11132166 | 4:184049867 | C/T | 0.036 | -0.0292 (0.0050) | 7.6E-09 | -0.0297 (0.0681) | 0.662 | 34.1 | 1.017 |
| Appendicular lean mass | All-cause dementia (FinnGen) | rs34338597 | 12:106301580 | G/A | 0.383 | -0.0112 (0.0019) | 7.9E-09 | -0.0078 (0.0194) | 0.689 | 34.8 | 0.696 |
| Appendicular lean mass | All-cause dementia (FinnGen) | rs4849904 | 2:121336556 | G/C | 0.380 | 0.0111 (0.0019) | 8.1E-09 | 0.0366 (0.0215) | 0.088 | 34.1 | 3.297 |
| Appendicular lean mass | All-cause dementia (FinnGen) | rs612577 | 1:230324364 | C/T | 0.149 | -0.0152 (0.0026) | 8.2E-09 | 0.0192 (0.0246) | 0.434 | 34.2 | -1.263 |
| Appendicular lean mass | All-cause dementia (FinnGen) | rs111925803 | 22:50719976 | T/C | 0.285 | 0.0122 (0.0021) | 8.6E-09 | 0.0308 (0.0218) | 0.157 | 33.8 | 2.525 |
| Appendicular lean mass | All-cause dementia (FinnGen) | rs921142 | 8:41169276 | C/T | 0.398 | -0.0111 (0.0019) | 8.7E-09 | -0.0428 (0.0192) | 0.026 | 34.1 | 3.856 |
| Appendicular lean mass | All-cause dementia (FinnGen) | rs10099846 | 8:22534178 | T/C | 0.668 | 0.0116 (0.0020) | 8.8E-09 | -0.0170 (0.0213) | 0.424 | 33.6 | -1.466 |
| Appendicular lean mass | All-cause dementia (FinnGen) | rs7744700 | 6:53349401 | A/T | 0.291 | -0.0122 (0.0021) | 9.2E-09 | -0.0183 (0.0210) | 0.382 | 33.8 | 1.500 |
| Appendicular lean mass | All-cause dementia (FinnGen) | rs12483401 | 21:35443829 | C/T | 0.022 | -0.0387 (0.0067) | 9.2E-09 | -0.0815 (0.0756) | 0.281 | 33.4 | 2.106 |
| Appendicular lean mass | All-cause dementia (FinnGen) | rs68185109 | 10:93648306 | A/G | 0.102 | 0.0180 (0.0031) | 9.6E-09 | 0.0489 (0.0326) | 0.134 | 33.7 | 2.717 |
| Appendicular lean mass | All-cause dementia (FinnGen) | rs28736838 | 1:120148713 | T/C | 0.302 | -0.0117 (0.0020) | 1.1E-08 | 0.0184 (0.0206) | 0.370 | 34.2 | -1.573 |
| Appendicular lean mass | All-cause dementia (FinnGen) | rs2208404 | 20:55843736 | G/A | 0.416 | -0.0111 (0.0019) | 1.1E-08 | 0.0192 (0.0196) | 0.326 | 34.1 | -1.730 |
| Appendicular lean mass | All-cause dementia (FinnGen) | rs8018486 | 14:39818616 | G/A | 0.191 | -0.0138 (0.0024) | 1.2E-08 | 0.0237 (0.0231) | 0.303 | 33.1 | -1.717 |
| Appendicular lean mass | All-cause dementia (FinnGen) | rs10749157 | 10:115780129 | C/T | 0.358 | 0.0113 (0.0020) | 1.2E-08 | -0.0086 (0.0192) | 0.655 | 31.9 | -0.761 |
| Appendicular lean mass | All-cause dementia (FinnGen) | rs11070842 | 15:51624185 | C/T | 0.163 | -0.0146 (0.0026) | 1.3E-08 | 0.0022 (0.0325) | 0.945 | 31.5 | -0.151 |
| Appendicular lean mass | All-cause dementia (FinnGen) | rs62103240 | 18:77650637 | A/G | 0.071 | 0.0212 (0.0037) | 1.4E-08 | 0.0861 (0.0446) | 0.053 | 32.8 | 4.061 |
| Appendicular lean mass | All-cause dementia (FinnGen) | rs10222594 | 3:71554856 | A/G | 0.323 | 0.0115 (0.0020) | 1.4E-08 | 0.0254 (0.0211) | 0.228 | 33.1 | 2.209 |
| Appendicular lean mass | All-cause dementia (FinnGen) | rs56309431 | 17:63645289 | T/G | 0.116 | 0.0167 (0.0029) | 1.5E-08 | -0.0255 (0.0309) | 0.409 | 33.2 | -1.527 |
| Appendicular lean mass | All-cause dementia (FinnGen) | rs10764692 | 10:27576251 | C/A | 0.637 | -0.0111 (0.0020) | 1.6E-08 | 0.0064 (0.0207) | 0.758 | 30.8 | -0.577 |
| Appendicular lean mass | All-cause dementia (FinnGen) | rs10940169 | 5:67823682 | C/G | 0.382 | -0.0110 (0.0019) | 1.6E-08 | 0.0258 (0.0202) | 0.202 | 33.5 | -2.345 |
| Appendicular lean mass | All-cause dementia (FinnGen) | rs10221831 | 2:202107829 | T/C | 0.032 | 0.0300 (0.0053) | 1.8E-08 | 0.0565 (0.0550) | 0.305 | 32.0 | 1.883 |
| Appendicular lean mass | All-cause dementia (FinnGen) | rs4965298 | 15:100802766 | T/C | 0.713 | -0.0119 (0.0021) | 1.8E-08 | 0.0094 (0.0229) | 0.682 | 32.1 | -0.790 |
| Appendicular lean mass | All-cause dementia (FinnGen) | rs28678024 | 14:25937161 | G/A | 0.285 | -0.0119 (0.0021) | 1.9E-08 | -0.0288 (0.0204) | 0.158 | 32.1 | 2.420 |
| Appendicular lean mass | All-cause dementia (FinnGen) | rs2188805 | 7:93078400 | C/A | 0.336 | 0.0114 (0.0020) | 2.0E-08 | -0.0220 (0.0221) | 0.320 | 32.5 | -1.930 |
| Appendicular lean mass | All-cause dementia (FinnGen) | rs9590328 | 13:96448383 | G/A | 0.142 | 0.0153 (0.0027) | 2.0E-08 | -0.0606 (0.0280) | 0.030 | 32.1 | -3.961 |
| Appendicular lean mass | All-cause dementia (FinnGen) | rs31196 | 5:158300798 | A/C | 0.572 | -0.0107 (0.0019) | 2.1E-08 | -0.0200 (0.0192) | 0.296 | 31.7 | 1.869 |
| Appendicular lean mass | All-cause dementia (FinnGen) | rs876122 | 6:6886297 | G/A | 0.879 | 0.0162 (0.0029) | 2.2E-08 | -0.0164 (0.0262) | 0.530 | 31.2 | -1.012 |
| Appendicular lean mass | All-cause dementia (FinnGen) | rs9288695 | 2:158641300 | C/G | 0.779 | 0.0127 (0.0023) | 2.2E-08 | 0.0038 (0.0231) | 0.869 | 30.5 | 0.299 |
| Appendicular lean mass | All-cause dementia (FinnGen) | rs7082659 | 10:12017584 | C/T | 0.866 | 0.0156 (0.0028) | 2.3E-08 | -0.0501 (0.0298) | 0.092 | 31.0 | -3.212 |
| Appendicular lean mass | All-cause dementia (FinnGen) | rs80280630 | 9:117030861 | T/C | 0.112 | -0.0168 (0.0030) | 2.3E-08 | 0.0266 (0.0355) | 0.455 | 31.4 | -1.583 |
| Appendicular lean mass | All-cause dementia (FinnGen) | rs74048171 | 11:2093603 | A/C | 0.258 | -0.0121 (0.0022) | 2.4E-08 | 0.0136 (0.0230) | 0.552 | 30.2 | -1.124 |
| Appendicular lean mass | All-cause dementia (FinnGen) | rs141277904 | 8:56654581 | T/C | 0.023 | 0.0384 (0.0069) | 2.4E-08 | 0.0913 (0.0772) | 0.237 | 31.0 | 2.378 |
| Appendicular lean mass | All-cause dementia (FinnGen) | rs4916786 | 5:89476946 | T/C | 0.196 | 0.0133 (0.0024) | 2.4E-08 | -0.0387 (0.0232) | 0.096 | 30.7 | -2.910 |
| Appendicular lean mass | All-cause dementia (FinnGen) | rs60142646 | 2:46532856 | TC/T | 0.948 | -0.0237 (0.0042) | 2.5E-08 | -0.0132 (0.0481) | 0.784 | 31.8 | 0.557 |
| Appendicular lean mass | All-cause dementia (FinnGen) | rs2303423 | 4:38120029 | C/T | 0.110 | 0.0168 (0.0030) | 2.6E-08 | 0.0387 (0.0366) | 0.289 | 31.4 | 2.304 |
| Appendicular lean mass | All-cause dementia (FinnGen) | rs9265546 | 6:31298202 | A/G | 0.223 | 0.0147 (0.0026) | 2.8E-08 | -0.0013 (0.0196) | 0.947 | 32.0 | -0.088 |
| Appendicular lean mass | All-cause dementia (FinnGen) | rs149697773 | 17:60932424 | G/A | 0.045 | -0.0262 (0.0047) | 2.8E-08 | 0.0247 (0.0450) | 0.584 | 31.1 | -0.943 |
| Appendicular lean mass | All-cause dementia (FinnGen) | rs2978362 | 18:32959397 | T/C | 0.533 | 0.0106 (0.0019) | 2.8E-08 | -0.0306 (0.0195) | 0.116 | 31.1 | -2.887 |
| Appendicular lean mass | All-cause dementia (FinnGen) | rs4383083 | 15:63080442 | A/G | 0.647 | 0.0111 (0.0020) | 2.9E-08 | -0.0048 (0.0218) | 0.825 | 30.8 | -0.432 |
| Appendicular lean mass | All-cause dementia (FinnGen) | rs10940364 | 5:53487971 | C/T | 0.139 | 0.0151 (0.0027) | 3.1E-08 | 0.0086 (0.0300) | 0.773 | 31.3 | 0.570 |
| Appendicular lean mass | All-cause dementia (FinnGen) | rs77293269 | 3:155595709 | C/T | 0.113 | -0.0165 (0.0030) | 3.1E-08 | -0.0153 (0.0307) | 0.619 | 30.2 | 0.927 |
| Appendicular lean mass | All-cause dementia (FinnGen) | rs62177315 | 2:161236992 | T/C | 0.096 | 0.0177 (0.0032) | 3.1E-08 | 0.0230 (0.0389) | 0.554 | 30.6 | 1.299 |
| Appendicular lean mass | All-cause dementia (FinnGen) | rs72809820 | 2:97360079 | T/C | 0.324 | -0.0111 (0.0020) | 3.1E-08 | -0.0077 (0.0192) | 0.687 | 30.8 | 0.694 |
| Appendicular lean mass | All-cause dementia (FinnGen) | rs9568031 | 13:48897520 | T/C | 0.706 | -0.0115 (0.0021) | 3.3E-08 | 0.0267 (0.0205) | 0.193 | 30.0 | -2.322 |
| Appendicular lean mass | All-cause dementia (FinnGen) | rs10821713 | 10:62055781 | T/C | 0.418 | 0.0106 (0.0019) | 3.4E-08 | 0.0090 (0.0197) | 0.650 | 31.1 | 0.849 |
| Appendicular lean mass | All-cause dementia (FinnGen) | rs12185775 | 20:20293769 | C/G | 0.117 | -0.0167 (0.0030) | 3.7E-08 | 0.0515 (0.0280) | 0.066 | 31.0 | -3.084 |
| Appendicular lean mass | All-cause dementia (FinnGen) | rs6461948 | 7:26540727 | T/C | 0.673 | 0.0112 (0.0020) | 4.0E-08 | -0.0068 (0.0218) | 0.754 | 31.4 | -0.607 |
| Appendicular lean mass | All-cause dementia (FinnGen) | rs11721522 | 4:156976051 | G/A | 0.412 | 0.0106 (0.0019) | 4.0E-08 | -0.0151 (0.0204) | 0.459 | 31.1 | -1.425 |
| Appendicular lean mass | All-cause dementia (FinnGen) | rs3002284 | 1:213181916 | C/T | 0.320 | -0.0110 (0.0020) | 4.1E-08 | 0.0351 (0.0203) | 0.085 | 30.2 | -3.191 |
| Appendicular lean mass | All-cause dementia (FinnGen) | rs35223841 | 2:144053000 | GAA/G | 0.389 | 0.0106 (0.0019) | 4.2E-08 | 0.0199 (0.0202) | 0.326 | 31.1 | 1.877 |
| Appendicular lean mass | All-cause dementia (FinnGen) | rs34856058 | 3:101365150 | G/A | 0.726 | 0.0116 (0.0021) | 4.4E-08 | 0.0111 (0.0222) | 0.615 | 30.5 | 0.957 |
| Appendicular lean mass | All-cause dementia (FinnGen) | rs1884066 | 14:67452834 | A/G | 0.150 | -0.0145 (0.0027) | 5.0E-08 | 0.0157 (0.0254) | 0.535 | 28.8 | -1.083 |
| Appendicular lean mass | Vascular dementia (FinnGen) | rs143384 | 20:34025756 | G/A | 0.404 | 0.0725 (0.0019) | 1.1E-308 | 0.0484 (0.0498) | 0.331 | 1456.0 | 0.668 |
| Appendicular lean mass | Vascular dementia (FinnGen) | rs6860245 | 5:127367998 | C/G | 0.248 | 0.0589 (0.0022) | 9.7E-160 | -0.0670 (0.0674) | 0.320 | 716.8 | -1.138 |
| Appendicular lean mass | Vascular dementia (FinnGen) | rs71190381 | 13:51120097 | GAGTGA/G | 0.214 | -0.0614 (0.0023) | 9.9E-155 | 0.0429 (0.0563) | 0.446 | 712.7 | -0.699 |
| Appendicular lean mass | Vascular dementia (FinnGen) | rs9853018 | 3:141101961 | T/C | 0.444 | 0.0469 (0.0019) | 2.4E-135 | 0.0354 (0.0496) | 0.476 | 609.3 | 0.755 |
| Appendicular lean mass | Vascular dementia (FinnGen) | rs1472852 | 4:17910236 | A/C | 0.158 | -0.0638 (0.0026) | 8.2E-135 | -0.0677 (0.0840) | 0.420 | 602.1 | 1.061 |
| Appendicular lean mass | Vascular dementia (FinnGen) | rs9388490 | 6:126704795 | T/C | 0.439 | 0.0462 (0.0019) | 1.3E-130 | 0.0820 (0.0494) | 0.097 | 591.3 | 1.775 |
| Appendicular lean mass | Vascular dementia (FinnGen) | rs2854152 | 17:61986027 | G/A | 0.678 | 0.0482 (0.0020) | 1.0E-124 | -0.0682 (0.0511) | 0.182 | 580.8 | -1.415 |
| Appendicular lean mass | Vascular dementia (FinnGen) | rs71519447 | 8:57157543 | A/G | 0.110 | -0.0716 (0.0030) | 3.9E-124 | 0.0856 (0.0785) | 0.276 | 569.6 | -1.196 |
| Appendicular lean mass | Vascular dementia (FinnGen) | rs6977416 | 7:150542711 | A/G | 0.334 | 0.0457 (0.0020) | 1.4E-113 | 0.0005 (0.0513) | 0.992 | 522.1 | 0.011 |
| Appendicular lean mass | Vascular dementia (FinnGen) | rs76895963 | 12:4384844 | G/T | 0.021 | 0.1639 (0.0073) | 8.2E-112 | -0.1199 (0.1500) | 0.424 | 504.1 | -0.732 |
| Appendicular lean mass | Vascular dementia (FinnGen) | rs42039 | 7:92244422 | T/C | 0.244 | 0.0481 (0.0022) | 3.5E-106 | -0.0349 (0.0584) | 0.550 | 478.0 | -0.726 |
| Appendicular lean mass | Vascular dementia (FinnGen) | rs6567160 | 18:57829135 | C/T | 0.233 | 0.0488 (0.0022) | 1.2E-104 | 0.0133 (0.0632) | 0.834 | 492.0 | 0.273 |
| Appendicular lean mass | Vascular dementia (FinnGen) | rs9634212 | 12:93993266 | A/C | 0.221 | 0.0471 (0.0023) | 8.6E-95 | 0.0647 (0.0569) | 0.256 | 419.4 | 1.374 |
| Appendicular lean mass | Vascular dementia (FinnGen) | rs11187838 | 10:96038686 | A/G | 0.435 | 0.0394 (0.0019) | 1.2E-94 | -0.0405 (0.0519) | 0.435 | 430.0 | -1.028 |
| Appendicular lean mass | Vascular dementia (FinnGen) | rs2524139 | 6:31264133 | C/G | 0.700 | -0.0423 (0.0020) | 1.5E-94 | 0.0410 (0.0625) | 0.512 | 447.3 | -0.969 |
| Appendicular lean mass | Vascular dementia (FinnGen) | rs28701981 | 9:98217581 | C/T | 0.349 | 0.0395 (0.0020) | 1.5E-87 | 0.0165 (0.0511) | 0.746 | 390.1 | 0.418 |
| Appendicular lean mass | Vascular dementia (FinnGen) | rs113459995 | 9:119122551 | GTCTGGGC/G | 0.201 | -0.0460 (0.0024) | 3.8E-84 | -0.0822 (0.0580) | 0.157 | 367.4 | 1.787 |
| Appendicular lean mass | Vascular dementia (FinnGen) | rs10483727 | 14:61072875 | C/T | 0.611 | -0.0368 (0.0019) | 6.7E-80 | 0.0495 (0.0541) | 0.360 | 375.1 | -1.345 |
| Appendicular lean mass | Vascular dementia (FinnGen) | rs1355603 | 4:145566477 | C/T | 0.831 | 0.0466 (0.0025) | 3.0E-76 | 0.0012 (0.0659) | 0.986 | 347.4 | 0.026 |
| Appendicular lean mass | Vascular dementia (FinnGen) | rs10807137 | 6:34183026 | T/C | 0.825 | -0.0455 (0.0025) | 5.7E-75 | 0.0803 (0.0707) | 0.256 | 331.2 | -1.765 |
| Appendicular lean mass | Vascular dementia (FinnGen) | rs55877758 | 2:172409030 | A/T | 0.241 | -0.0394 (0.0022) | 2.5E-72 | 0.0272 (0.0629) | 0.665 | 320.7 | -0.690 |
| Appendicular lean mass | Vascular dementia (FinnGen) | rs12713004 | 2:23896049 | G/A | 0.725 | 0.0367 (0.0021) | 2.4E-68 | -0.0824 (0.0642) | 0.199 | 305.4 | -2.245 |
| Appendicular lean mass | Vascular dementia (FinnGen) | rs798528 | 7:2772431 | C/A | 0.307 | -0.0357 (0.0020) | 5.6E-68 | -0.0265 (0.0509) | 0.602 | 318.6 | 0.742 |
| Appendicular lean mass | Vascular dementia (FinnGen) | rs1260326 | 2:27730940 | C/T | 0.605 | 0.0323 (0.0019) | 6.2E-64 | -0.0084 (0.0516) | 0.870 | 289.0 | -0.260 |
| Appendicular lean mass | Vascular dementia (FinnGen) | rs9905385 | 17:59498250 | G/A | 0.671 | -0.0339 (0.0020) | 1.9E-63 | 0.0564 (0.0553) | 0.307 | 287.3 | -1.664 |
| Appendicular lean mass | Vascular dementia (FinnGen) | rs3213141 | 20:32274380 | A/G | 0.266 | -0.0363 (0.0022) | 2.0E-63 | -0.0664 (0.0568) | 0.242 | 272.2 | 1.829 |
| Appendicular lean mass | Vascular dementia (FinnGen) | rs485554 | 3:172137343 | C/G | 0.315 | 0.0342 (0.0020) | 3.0E-63 | 0.0725 (0.0525) | 0.167 | 292.4 | 2.120 |
| Appendicular lean mass | Vascular dementia (FinnGen) | rs2871865 | 15:99194896 | G/C | 0.116 | -0.0493 (0.0030) | 3.4E-62 | 0.2519 (0.1010) | 0.013 | 270.1 | -5.110 |
| Appendicular lean mass | Vascular dementia (FinnGen) | rs117068593 | 14:93118229 | T/C | 0.190 | 0.0403 (0.0024) | 8.8E-62 | -0.0108 (0.0710) | 0.879 | 282.0 | -0.268 |
| Appendicular lean mass | Vascular dementia (FinnGen) | rs2885697 | 1:41544279 | T/G | 0.665 | -0.0323 (0.0020) | 9.2E-60 | -0.0071 (0.0519) | 0.891 | 260.8 | 0.220 |
| Appendicular lean mass | Vascular dementia (FinnGen) | rs12099669 | 12:46783653 | A/G | 0.696 | 0.0331 (0.0020) | 1.4E-58 | -0.1027 (0.0519) | 0.048 | 273.9 | -3.103 |
| Appendicular lean mass | Vascular dementia (FinnGen) | rs11243202 | 6:7719065 | C/T | 0.486 | 0.0302 (0.0019) | 2.8E-57 | 0.0483 (0.0495) | 0.329 | 252.6 | 1.599 |
| Appendicular lean mass | Vascular dementia (FinnGen) | rs4735761 | 8:78097161 | C/A | 0.286 | 0.0331 (0.0021) | 3.7E-56 | 0.0414 (0.0593) | 0.485 | 248.4 | 1.251 |
| Appendicular lean mass | Vascular dementia (FinnGen) | rs78378222 | 17:7571752 | G/T | 0.012 | 0.1380 (0.0087) | 4.5E-56 | -0.1583 (0.1917) | 0.409 | 251.6 | -1.147 |
| Appendicular lean mass | Vascular dementia (FinnGen) | rs2194411 | 3:185548663 | A/G | 0.128 | 0.0443 (0.0029) | 2.4E-54 | -0.0900 (0.0723) | 0.213 | 233.3 | -2.032 |
| Appendicular lean mass | Vascular dementia (FinnGen) | rs994533 | 2:218284278 | C/G | 0.316 | -0.0313 (0.0020) | 5.5E-54 | 0.0093 (0.0496) | 0.851 | 244.9 | -0.297 |
| Appendicular lean mass | Vascular dementia (FinnGen) | rs11042717 | 11:10303939 | C/T | 0.490 | -0.0290 (0.0019) | 4.0E-53 | 0.0564 (0.0491) | 0.251 | 233.0 | -1.945 |
| Appendicular lean mass | Vascular dementia (FinnGen) | rs7129320 | 11:68388220 | A/G | 0.166 | -0.0389 (0.0025) | 7.3E-53 | 0.1099 (0.0864) | 0.203 | 242.1 | -2.825 |
| Appendicular lean mass | Vascular dementia (FinnGen) | rs9894577 | 17:43223292 | A/G | 0.318 | -0.0310 (0.0020) | 1.4E-52 | -0.0225 (0.0522) | 0.667 | 240.2 | 0.726 |
| Appendicular lean mass | Vascular dementia (FinnGen) | rs146851424 | 13:50377910 | C/A | 0.022 | 0.1003 (0.0066) | 1.6E-52 | 0.1165 (0.1841) | 0.527 | 230.9 | 1.162 |
| Appendicular lean mass | Vascular dementia (FinnGen) | rs1325596 | 1:176794066 | A/G | 0.548 | 0.0287 (0.0019) | 2.8E-52 | -0.0218 (0.0492) | 0.657 | 228.2 | -0.760 |
| Appendicular lean mass | Vascular dementia (FinnGen) | rs72801818 | 16:53476042 | T/C | 0.300 | 0.0313 (0.0021) | 8.9E-52 | 0.0011 (0.0560) | 0.984 | 222.2 | 0.035 |
| Appendicular lean mass | Vascular dementia (FinnGen) | rs13103161 | 4:106216459 | A/T | 0.389 | -0.0284 (0.0019) | 2.4E-48 | -0.0384 (0.0516) | 0.456 | 223.4 | 1.352 |
| Appendicular lean mass | Vascular dementia (FinnGen) | rs34517439 | 1:78450517 | A/C | 0.122 | 0.0421 (0.0029) | 5.8E-48 | 0.0595 (0.0740) | 0.422 | 210.8 | 1.413 |
| Appendicular lean mass | Vascular dementia (FinnGen) | rs10491967 | 12:3368093 | A/G | 0.092 | 0.0471 (0.0032) | 1.3E-47 | 0.0341 (0.0924) | 0.712 | 216.6 | 0.724 |
| Appendicular lean mass | Vascular dementia (FinnGen) | rs723149 | 7:46577056 | G/A | 0.563 | -0.0276 (0.0019) | 1.4E-47 | 0.0204 (0.0495) | 0.680 | 211.0 | -0.739 |
| Appendicular lean mass | Vascular dementia (FinnGen) | rs4909912 | 8:135572297 | T/C | 0.427 | -0.0277 (0.0019) | 1.7E-47 | 0.0067 (0.0493) | 0.892 | 212.6 | -0.242 |
| Appendicular lean mass | Vascular dementia (FinnGen) | rs34776209 | 7:23513093 | T/C | 0.247 | -0.0317 (0.0022) | 1.8E-47 | 0.0852 (0.0606) | 0.160 | 207.6 | -2.688 |
| Appendicular lean mass | Vascular dementia (FinnGen) | rs41271299 | 6:19839415 | T/C | 0.051 | 0.0616 (0.0043) | 3.2E-47 | 0.1350 (0.1957) | 0.490 | 205.2 | 2.192 |
| Appendicular lean mass | Vascular dementia (FinnGen) | rs1319012 | 6:41852616 | A/T | 0.926 | -0.0520 (0.0037) | 3.3E-45 | 0.0235 (0.0838) | 0.779 | 197.5 | -0.452 |
| Appendicular lean mass | Vascular dementia (FinnGen) | rs7095472 | 10:70399109 | G/A | 0.533 | 0.0267 (0.0019) | 7.7E-45 | 0.0223 (0.0493) | 0.651 | 197.5 | 0.835 |
| Appendicular lean mass | Vascular dementia (FinnGen) | rs59985551 | 2:56106928 | T/C | 0.226 | -0.0313 (0.0022) | 2.4E-44 | 0.0247 (0.0592) | 0.676 | 202.4 | -0.789 |
| Appendicular lean mass | Vascular dementia (FinnGen) | rs10283100 | 8:120596023 | G/A | 0.945 | 0.0575 (0.0041) | 4.1E-44 | 0.0807 (0.0961) | 0.401 | 196.7 | 1.403 |
| Appendicular lean mass | Vascular dementia (FinnGen) | rs6931421 | 6:80880138 | G/T | 0.322 | -0.0279 (0.0020) | 2.3E-43 | -0.0288 (0.0540) | 0.594 | 194.6 | 1.032 |
| Appendicular lean mass | Vascular dementia (FinnGen) | rs17205463 | 15:62381413 | T/C | 0.448 | -0.0263 (0.0019) | 4.2E-43 | 0.0342 (0.0520) | 0.510 | 191.6 | -1.300 |
| Appendicular lean mass | Vascular dementia (FinnGen) | rs2270894 | 3:9975386 | G/C | 0.203 | -0.0332 (0.0024) | 1.3E-42 | -0.0381 (0.0560) | 0.497 | 191.4 | 1.148 |
| Appendicular lean mass | Vascular dementia (FinnGen) | rs4282339 | 5:168256240 | A/G | 0.207 | -0.0311 (0.0023) | 6.2E-41 | -0.0753 (0.0632) | 0.234 | 182.8 | 2.421 |
| Appendicular lean mass | Vascular dementia (FinnGen) | rs10883555 | 10:102657908 | T/C | 0.550 | 0.0254 (0.0019) | 9.1E-41 | -0.0272 (0.0495) | 0.583 | 178.7 | -1.071 |
| Appendicular lean mass | Vascular dementia (FinnGen) | rs12051245 | 16:783865 | C/T | 0.232 | 0.0299 (0.0022) | 2.6E-40 | 0.0447 (0.0522) | 0.392 | 184.7 | 1.495 |
| Appendicular lean mass | Vascular dementia (FinnGen) | rs11014285 | 10:25178864 | A/G | 0.165 | 0.0342 (0.0026) | 2.9E-40 | -0.0681 (0.0814) | 0.403 | 173.0 | -1.991 |
| Appendicular lean mass | Vascular dementia (FinnGen) | rs2229840 | 12:124826462 | T/C | 0.160 | 0.0341 (0.0026) | 3.0E-40 | -0.0195 (0.0689) | 0.777 | 172.0 | -0.572 |
| Appendicular lean mass | Vascular dementia (FinnGen) | rs35230100 | 14:70348141 | CATAT/C | 0.433 | -0.0262 (0.0020) | 4.4E-40 | -0.0018 (0.0500) | 0.972 | 171.6 | 0.069 |
| Appendicular lean mass | Vascular dementia (FinnGen) | rs35309034 | 8:126498265 | A/C | 0.238 | -0.0297 (0.0022) | 1.1E-39 | 0.0014 (0.0538) | 0.980 | 182.2 | -0.047 |
| Appendicular lean mass | Vascular dementia (FinnGen) | rs7952436 | 11:67024534 | T/C | 0.082 | -0.0453 (0.0034) | 1.6E-39 | 0.0919 (0.1125) | 0.414 | 177.5 | -2.029 |
| Appendicular lean mass | Vascular dementia (FinnGen) | rs8054549 | 16:86417234 | A/C | 0.449 | -0.0251 (0.0019) | 3.4E-39 | 0.0053 (0.0504) | 0.916 | 174.5 | -0.211 |
| Appendicular lean mass | Vascular dementia (FinnGen) | rs3764002 | 12:108618630 | T/C | 0.262 | 0.0280 (0.0021) | 4.5E-39 | 0.0341 (0.0516) | 0.509 | 177.8 | 1.218 |
| Appendicular lean mass | Vascular dementia (FinnGen) | rs12714414 | 2:651407 | C/T | 0.152 | -0.0353 (0.0027) | 8.2E-39 | -0.0372 (0.0663) | 0.575 | 170.9 | 1.054 |
| Appendicular lean mass | Vascular dementia (FinnGen) | rs5742915 | 15:74336633 | C/T | 0.461 | 0.0248 (0.0019) | 9.3E-39 | -0.0265 (0.0502) | 0.598 | 170.4 | -1.069 |
| Appendicular lean mass | Vascular dementia (FinnGen) | rs33973388 | 18:46611842 | T/G | 0.435 | 0.0249 (0.0019) | 1.4E-38 | -0.0808 (0.0535) | 0.131 | 171.8 | -3.245 |
| Appendicular lean mass | Vascular dementia (FinnGen) | rs8019890 | 14:21538067 | A/C | 0.531 | 0.0250 (0.0019) | 2.0E-38 | 0.1005 (0.0497) | 0.043 | 173.1 | 4.020 |
| Appendicular lean mass | Vascular dementia (FinnGen) | rs8042545 | 15:66992821 | A/G | 0.242 | 0.0287 (0.0022) | 2.9E-38 | 0.0315 (0.0586) | 0.592 | 170.2 | 1.098 |
| Appendicular lean mass | Vascular dementia (FinnGen) | rs2900208 | 12:11878464 | A/C | 0.354 | 0.0255 (0.0020) | 3.8E-38 | -0.0436 (0.0504) | 0.387 | 162.6 | -1.710 |
| Appendicular lean mass | Vascular dementia (FinnGen) | rs10748128 | 12:69827658 | T/G | 0.345 | 0.0255 (0.0020) | 6.8E-38 | 0.0271 (0.0515) | 0.599 | 162.6 | 1.063 |
| Appendicular lean mass | Vascular dementia (FinnGen) | rs28592876 | 12:123866429 | A/G | 0.205 | 0.0300 (0.0023) | 9.1E-38 | -0.0813 (0.0602) | 0.176 | 170.1 | -2.710 |
| Appendicular lean mass | Vascular dementia (FinnGen) | rs4615815 | 1:214604236 | G/A | 0.397 | -0.0246 (0.0019) | 1.1E-37 | -0.0074 (0.0516) | 0.885 | 167.6 | 0.301 |
| Appendicular lean mass | Vascular dementia (FinnGen) | rs244711 | 5:176509193 | T/C | 0.686 | 0.0279 (0.0022) | 1.5E-37 | -0.1214 (0.0537) | 0.024 | 160.8 | -4.351 |
| Appendicular lean mass | Vascular dementia (FinnGen) | rs17036160 | 3:12329783 | T/C | 0.118 | -0.0375 (0.0029) | 1.7E-37 | -0.0748 (0.0661) | 0.258 | 167.2 | 1.995 |
| Appendicular lean mass | Vascular dementia (FinnGen) | rs36226649 | 14:24835500 | C/T | 0.067 | 0.0485 (0.0038) | 3.1E-37 | 0.1508 (0.1139) | 0.186 | 162.9 | 3.109 |
| Appendicular lean mass | Vascular dementia (FinnGen) | rs61944841 | 13:27049616 | A/G | 0.414 | 0.0253 (0.0020) | 3.5E-37 | -0.0439 (0.0508) | 0.387 | 160.0 | -1.735 |
| Appendicular lean mass | Vascular dementia (FinnGen) | rs3853252 | 6:152170247 | A/G | 0.455 | 0.0241 (0.0019) | 8.8E-37 | 0.0659 (0.0502) | 0.189 | 160.9 | 2.734 |
| Appendicular lean mass | Vascular dementia (FinnGen) | rs7301341 | 12:94083105 | C/T | 0.327 | -0.0255 (0.0020) | 9.3E-37 | -0.0596 (0.0518) | 0.249 | 162.6 | 2.337 |
| Appendicular lean mass | Vascular dementia (FinnGen) | rs12622189 | 2:218132858 | G/A | 0.258 | -0.0272 (0.0021) | 9.3E-37 | 0.0410 (0.0514) | 0.425 | 167.8 | -1.507 |
| Appendicular lean mass | Vascular dementia (FinnGen) | rs2035901 | 4:145521867 | G/A | 0.468 | 0.0240 (0.0019) | 9.4E-37 | -0.0220 (0.0501) | 0.660 | 159.6 | -0.917 |
| Appendicular lean mass | Vascular dementia (FinnGen) | rs778384 | 1:56637166 | A/G | 0.223 | 0.0283 (0.0022) | 2.8E-36 | 0.0097 (0.0602) | 0.873 | 165.5 | 0.343 |
| Appendicular lean mass | Vascular dementia (FinnGen) | rs3843750 | 19:10748073 | G/C | 0.664 | -0.0252 (0.0020) | 5.1E-36 | 0.0140 (0.0548) | 0.799 | 158.8 | -0.556 |
| Appendicular lean mass | Vascular dementia (FinnGen) | rs4752829 | 11:47396654 | A/G | 0.286 | 0.0262 (0.0021) | 5.9E-36 | 0.0189 (0.0519) | 0.716 | 155.7 | 0.721 |
| Appendicular lean mass | Vascular dementia (FinnGen) | rs10691068 | 6:109723017 | TCC/T | 0.407 | -0.0242 (0.0019) | 6.5E-36 | 0.1217 (0.0493) | 0.014 | 162.2 | -5.029 |
| Appendicular lean mass | Vascular dementia (FinnGen) | rs59950280 | 4:3452345 | A/G | 0.332 | -0.0254 (0.0020) | 7.3E-36 | -0.0338 (0.0573) | 0.556 | 161.3 | 1.331 |
| Appendicular lean mass | Vascular dementia (FinnGen) | rs11689546 | 2:25130451 | G/A | 0.440 | -0.0238 (0.0019) | 8.1E-36 | 0.0136 (0.0504) | 0.788 | 156.9 | -0.571 |
| Appendicular lean mass | Vascular dementia (FinnGen) | rs2098695 | 5:54868944 | G/A | 0.691 | -0.0255 (0.0020) | 1.1E-35 | 0.0613 (0.0537) | 0.254 | 162.6 | -2.404 |
| Appendicular lean mass | Vascular dementia (FinnGen) | rs7582516 | 2:227255370 | T/C | 0.304 | 0.0254 (0.0020) | 1.3E-35 | -0.0466 (0.0549) | 0.396 | 161.3 | -1.835 |
| Appendicular lean mass | Vascular dementia (FinnGen) | rs3818416 | 13:78474468 | C/A | 0.764 | 0.0279 (0.0022) | 2.0E-35 | -0.0764 (0.0591) | 0.196 | 160.8 | -2.738 |
| Appendicular lean mass | Vascular dementia (FinnGen) | rs2549677 | 16:2162361 | G/A | 0.099 | -0.0392 (0.0032) | 1.3E-34 | -0.0021 (0.0984) | 0.983 | 150.1 | 0.054 |
| Appendicular lean mass | Vascular dementia (FinnGen) | rs1177765 | 5:32829929 | C/T | 0.468 | -0.0232 (0.0019) | 1.3E-34 | -0.0261 (0.0489) | 0.594 | 149.1 | 1.125 |
| Appendicular lean mass | Vascular dementia (FinnGen) | rs61838776 | 1:219618564 | A/C | 0.162 | 0.0311 (0.0025) | 2.4E-34 | 0.0796 (0.0761) | 0.295 | 154.8 | 2.559 |
| Appendicular lean mass | Vascular dementia (FinnGen) | rs115912456 | 5:82815158 | G/A | 0.041 | 0.0577 (0.0047) | 3.7E-34 | 0.1082 (0.1768) | 0.540 | 150.7 | 1.875 |
| Appendicular lean mass | Vascular dementia (FinnGen) | rs4852257 | 2:71678520 | G/T | 0.576 | -0.0231 (0.0019) | 6.2E-34 | -0.0655 (0.0492) | 0.183 | 147.8 | 2.836 |
| Appendicular lean mass | Vascular dementia (FinnGen) | rs12761076 | 10:72423050 | A/G | 0.266 | -0.0260 (0.0021) | 1.0E-33 | -0.0256 (0.0555) | 0.644 | 153.3 | 0.985 |
| Appendicular lean mass | Vascular dementia (FinnGen) | rs111365325 | 5:170865229 | T/C | 0.231 | -0.0271 (0.0022) | 1.1E-33 | -0.0038 (0.0612) | 0.951 | 151.7 | 0.140 |
| Appendicular lean mass | Vascular dementia (FinnGen) | rs45474992 | 19:47724564 | T/C | 0.036 | -0.0617 (0.0051) | 2.2E-33 | 0.0103 (0.1405) | 0.942 | 146.4 | -0.167 |
| Appendicular lean mass | Vascular dementia (FinnGen) | rs7428883 | 3:128983853 | G/A | 0.781 | 0.0277 (0.0023) | 2.3E-33 | 0.0339 (0.0656) | 0.605 | 145.1 | 1.224 |
| Appendicular lean mass | Vascular dementia (FinnGen) | rs76364830 | 8:13372120 | A/G | 0.063 | -0.0471 (0.0039) | 2.7E-33 | 0.0855 (0.1345) | 0.525 | 145.8 | -1.815 |
| Appendicular lean mass | Vascular dementia (FinnGen) | rs10202701 | 2:232328681 | T/C | 0.542 | 0.0227 (0.0019) | 3.1E-33 | 0.0110 (0.0491) | 0.822 | 142.7 | 0.485 |
| Appendicular lean mass | Vascular dementia (FinnGen) | rs4976262 | 5:134379531 | C/T | 0.316 | -0.0245 (0.0020) | 4.4E-33 | -0.0639 (0.0513) | 0.213 | 150.1 | 2.608 |
| Appendicular lean mass | Vascular dementia (FinnGen) | rs6082354 | 20:21217976 | C/A | 0.667 | -0.0240 (0.0020) | 1.2E-32 | 0.0565 (0.0540) | 0.296 | 144.0 | -2.354 |
| Appendicular lean mass | Vascular dementia (FinnGen) | rs2209098 | 1:172167226 | C/T | 0.311 | 0.0240 (0.0020) | 1.7E-32 | 0.0336 (0.0535) | 0.530 | 144.0 | 1.400 |
| Appendicular lean mass | Vascular dementia (FinnGen) | rs1444628 | 12:20563643 | T/C | 0.690 | 0.0240 (0.0020) | 6.8E-32 | 0.1047 (0.0603) | 0.082 | 144.0 | 4.362 |
| Appendicular lean mass | Vascular dementia (FinnGen) | rs147110934 | 19:55993436 | T/G | 0.024 | -0.0722 (0.0062) | 9.4E-32 | -0.5121 (0.3209) | 0.111 | 135.6 | 7.093 |
| Appendicular lean mass | Vascular dementia (FinnGen) | rs148138388 | 1:51371608 | C/G | 0.083 | 0.0406 (0.0035) | 1.1E-31 | -0.0765 (0.1050) | 0.466 | 134.6 | -1.884 |
| Appendicular lean mass | Vascular dementia (FinnGen) | rs58738817 | 3:184012228 | A/G | 0.248 | -0.0257 (0.0022) | 1.2E-31 | 0.0152 (0.0538) | 0.777 | 136.5 | -0.591 |
| Appendicular lean mass | Vascular dementia (FinnGen) | rs6570509 | 6:142716286 | T/G | 0.287 | -0.0244 (0.0021) | 1.3E-31 | 0.1316 (0.0561) | 0.019 | 135.0 | -5.393 |
| Appendicular lean mass | Vascular dementia (FinnGen) | rs4252548 | 19:55879672 | T/C | 0.022 | -0.0753 (0.0065) | 3.0E-31 | 0.0433 (0.1567) | 0.782 | 134.2 | -0.575 |
| Appendicular lean mass | Vascular dementia (FinnGen) | rs35464459 | 10:4964640 | C/T | 0.116 | -0.0342 (0.0029) | 5.0E-31 | -0.0454 (0.0733) | 0.536 | 139.1 | 1.327 |
| Appendicular lean mass | Vascular dementia (FinnGen) | rs1047891 | 2:211540507 | A/C | 0.316 | 0.0233 (0.0020) | 5.7E-31 | -0.1229 (0.0530) | 0.020 | 135.7 | -5.275 |
| Appendicular lean mass | Vascular dementia (FinnGen) | rs35756741 | 12:12868701 | T/C | 0.092 | -0.0378 (0.0033) | 5.8E-31 | -0.1247 (0.0811) | 0.124 | 131.2 | 3.299 |
| Appendicular lean mass | Vascular dementia (FinnGen) | rs55872725 | 16:53809123 | T/C | 0.404 | 0.0222 (0.0019) | 1.5E-30 | 0.0166 (0.0498) | 0.739 | 136.5 | 0.748 |
| Appendicular lean mass | Vascular dementia (FinnGen) | rs6501381 | 17:68148350 | C/T | 0.113 | -0.0341 (0.0030) | 5.2E-30 | -0.0696 (0.0863) | 0.420 | 129.2 | 2.041 |
| Appendicular lean mass | Vascular dementia (FinnGen) | rs12509014 | 4:123825919 | T/C | 0.790 | -0.0263 (0.0023) | 5.4E-30 | 0.0038 (0.0637) | 0.952 | 130.8 | -0.144 |
| Appendicular lean mass | Vascular dementia (FinnGen) | rs8031704 | 15:84651185 | A/C | 0.327 | -0.0228 (0.0020) | 2.1E-29 | -0.0080 (0.0523) | 0.878 | 130.0 | 0.351 |
| Appendicular lean mass | Vascular dementia (FinnGen) | rs212526 | 1:21584941 | C/T | 0.601 | 0.0214 (0.0019) | 3.8E-29 | 0.0057 (0.0508) | 0.911 | 126.9 | 0.266 |
| Appendicular lean mass | Vascular dementia (FinnGen) | rs9647379 | 3:171785168 | C/G | 0.411 | 0.0215 (0.0019) | 5.5E-29 | 0.0827 (0.0504) | 0.101 | 128.1 | 3.847 |
| Appendicular lean mass | Vascular dementia (FinnGen) | rs4244809 | 11:2164333 | A/G | 0.208 | -0.0262 (0.0023) | 5.6E-29 | 0.1339 (0.0643) | 0.037 | 129.8 | -5.111 |
| Appendicular lean mass | Vascular dementia (FinnGen) | rs4076427 | 17:79087537 | C/G | 0.391 | -0.0218 (0.0019) | 6.0E-29 | 0.0005 (0.0524) | 0.993 | 131.7 | -0.023 |
| Appendicular lean mass | Vascular dementia (FinnGen) | rs7229520 | 18:46516468 | A/G | 0.662 | -0.0224 (0.0020) | 9.2E-29 | -0.0576 (0.0517) | 0.265 | 125.4 | 2.571 |
| Appendicular lean mass | Vascular dementia (FinnGen) | rs6762851 | 3:56686329 | C/T | 0.357 | -0.0218 (0.0020) | 1.5E-28 | 0.0143 (0.0498) | 0.773 | 118.8 | -0.656 |
| Appendicular lean mass | Vascular dementia (FinnGen) | rs1211575 | 1:11118390 | A/G | 0.743 | -0.0237 (0.0021) | 1.9E-28 | 0.0591 (0.0551) | 0.284 | 127.4 | -2.494 |
| Appendicular lean mass | Vascular dementia (FinnGen) | rs4274112 | 1:26746199 | G/A | 0.373 | -0.0217 (0.0020) | 2.5E-28 | -0.0829 (0.0518) | 0.110 | 117.7 | 3.820 |
| Appendicular lean mass | Vascular dementia (FinnGen) | rs2071518 | 8:120435812 | T/C | 0.264 | -0.0236 (0.0021) | 3.4E-28 | -0.0426 (0.0617) | 0.490 | 126.3 | 1.805 |
| Appendicular lean mass | Vascular dementia (FinnGen) | rs13302480 | 9:118465313 | C/G | 0.114 | -0.0328 (0.0030) | 4.4E-28 | 0.0222 (0.0994) | 0.823 | 119.5 | -0.677 |
| Appendicular lean mass | Vascular dementia (FinnGen) | rs36048468 | 5:122879901 | T/C | 0.209 | 0.0254 (0.0023) | 9.3E-28 | -0.0537 (0.0669) | 0.422 | 122.0 | -2.114 |
| Appendicular lean mass | Vascular dementia (FinnGen) | rs62370472 | 5:52767109 | C/T | 0.209 | -0.0253 (0.0023) | 1.4E-27 | 0.1001 (0.0580) | 0.085 | 121.0 | -3.957 |
| Appendicular lean mass | Vascular dementia (FinnGen) | rs77165542 | 2:430975 | T/C | 0.035 | -0.0557 (0.0051) | 1.8E-27 | -0.4020 (0.1837) | 0.029 | 119.3 | 7.217 |
| Appendicular lean mass | Vascular dementia (FinnGen) | rs62621812 | 7:127015083 | A/G | 0.020 | 0.0743 (0.0069) | 3.2E-27 | 0.0922 (0.1237) | 0.456 | 116.0 | 1.241 |
| Appendicular lean mass | Vascular dementia (FinnGen) | rs181766 | 16:14394878 | C/T | 0.322 | 0.0221 (0.0020) | 4.3E-27 | 0.0238 (0.0515) | 0.644 | 122.1 | 1.077 |
| Appendicular lean mass | Vascular dementia (FinnGen) | rs62048221 | 16:88846552 | T/C | 0.231 | -0.0242 (0.0022) | 4.9E-27 | 0.0117 (0.0617) | 0.850 | 121.0 | -0.483 |
| Appendicular lean mass | Vascular dementia (FinnGen) | rs7781964 | 7:139749346 | A/G | 0.185 | 0.0261 (0.0024) | 5.7E-27 | -0.0354 (0.0643) | 0.582 | 118.3 | -1.356 |
| Appendicular lean mass | Vascular dementia (FinnGen) | rs2070598 | 14:75360906 | A/G | 0.456 | 0.0204 (0.0019) | 6.4E-27 | -0.0444 (0.0496) | 0.370 | 115.3 | -2.176 |
| Appendicular lean mass | Vascular dementia (FinnGen) | rs13209685 | 6:7779729 | T/G | 0.159 | 0.0277 (0.0026) | 7.5E-27 | -0.0433 (0.0610) | 0.478 | 113.5 | -1.563 |
| Appendicular lean mass | Vascular dementia (FinnGen) | rs951366 | 1:205685352 | C/T | 0.393 | 0.0205 (0.0019) | 9.2E-27 | -0.0661 (0.0513) | 0.197 | 116.4 | -3.224 |
| Appendicular lean mass | Vascular dementia (FinnGen) | rs291970 | 10:121123633 | T/C | 0.229 | 0.0240 (0.0022) | 1.3E-26 | -0.0248 (0.0664) | 0.709 | 119.0 | -1.033 |
| Appendicular lean mass | Vascular dementia (FinnGen) | rs4752689 | 10:124131176 | A/G | 0.584 | 0.0205 (0.0019) | 1.4E-26 | 0.0247 (0.0541) | 0.648 | 116.4 | 1.205 |
| Appendicular lean mass | Vascular dementia (FinnGen) | rs7768382 | 6:166341870 | C/T | 0.477 | -0.0201 (0.0019) | 1.6E-26 | 0.0251 (0.0494) | 0.612 | 111.9 | -1.249 |
| Appendicular lean mass | Vascular dementia (FinnGen) | rs72841270 | 10:104642237 | G/T | 0.135 | 0.0294 (0.0028) | 2.3E-26 | 0.1181 (0.0710) | 0.096 | 110.2 | 4.017 |
| Appendicular lean mass | Vascular dementia (FinnGen) | rs7014590 | 8:89335647 | C/T | 0.261 | -0.0228 (0.0022) | 4.5E-26 | 0.0212 (0.0570) | 0.709 | 107.4 | -0.930 |
| Appendicular lean mass | Vascular dementia (FinnGen) | rs9659061 | 1:150527292 | A/G | 0.476 | -0.0198 (0.0019) | 6.8E-26 | -0.0483 (0.0498) | 0.332 | 108.6 | 2.439 |
| Appendicular lean mass | Vascular dementia (FinnGen) | rs7497684 | 15:89342720 | A/C | 0.480 | 0.0200 (0.0019) | 8.3E-26 | 0.0071 (0.0499) | 0.887 | 110.8 | 0.355 |
| Appendicular lean mass | Vascular dementia (FinnGen) | rs1662842 | 4:82155362 | G/A | 0.307 | 0.0214 (0.0020) | 1.7E-25 | -0.0034 (0.0510) | 0.946 | 114.5 | -0.159 |
| Appendicular lean mass | Vascular dementia (FinnGen) | rs7072873 | 10:77210191 | T/C | 0.539 | -0.0198 (0.0019) | 3.2E-25 | 0.0407 (0.0491) | 0.407 | 108.6 | -2.056 |
| Appendicular lean mass | Vascular dementia (FinnGen) | rs4640244 | 17:21284223 | G/A | 0.399 | -0.0200 (0.0019) | 3.8E-25 | 0.0354 (0.0502) | 0.481 | 110.8 | -1.770 |
| Appendicular lean mass | Vascular dementia (FinnGen) | rs7858712 | 9:16738312 | G/A | 0.914 | 0.0347 (0.0034) | 1.0E-24 | 0.1718 (0.0916) | 0.061 | 104.2 | 4.951 |
| Appendicular lean mass | Vascular dementia (FinnGen) | rs927376 | 20:38569825 | G/C | 0.260 | -0.0223 (0.0022) | 1.2E-24 | 0.0426 (0.0627) | 0.497 | 102.8 | -1.910 |
| Appendicular lean mass | Vascular dementia (FinnGen) | rs78457529 | 16:24950880 | T/C | 0.012 | -0.0904 (0.0088) | 1.2E-24 | -0.2364 (0.1868) | 0.206 | 105.5 | 2.615 |
| Appendicular lean mass | Vascular dementia (FinnGen) | rs2748501 | 6:146312258 | A/G | 0.440 | -0.0195 (0.0019) | 1.3E-24 | -0.0726 (0.0494) | 0.141 | 105.3 | 3.723 |
| Appendicular lean mass | Vascular dementia (FinnGen) | rs12882130 | 14:103878774 | G/C | 0.384 | -0.0202 (0.0020) | 1.9E-24 | 0.0134 (0.0509) | 0.793 | 102.0 | -0.663 |
| Appendicular lean mass | Vascular dementia (FinnGen) | rs45528934 | 14:23793305 | T/C | 0.162 | 0.0262 (0.0026) | 2.0E-24 | 0.0163 (0.0740) | 0.825 | 101.5 | 0.622 |
| Appendicular lean mass | Vascular dementia (FinnGen) | rs41311445 | 22:42070374 | C/A | 0.096 | -0.0328 (0.0032) | 4.7E-24 | 0.0008 (0.0725) | 0.991 | 105.1 | -0.024 |
| Appendicular lean mass | Vascular dementia (FinnGen) | rs60804050 | 1:118870373 | A/G | 0.256 | -0.0217 (0.0021) | 5.0E-24 | 0.0874 (0.0574) | 0.128 | 106.8 | -4.028 |
| Appendicular lean mass | Vascular dementia (FinnGen) | rs7816345 | 8:36846109 | T/C | 0.168 | 0.0255 (0.0025) | 6.2E-24 | 0.1034 (0.0710) | 0.145 | 104.0 | 4.055 |
| Appendicular lean mass | Vascular dementia (FinnGen) | rs2627702 | 4:88577419 | C/T | 0.519 | -0.0191 (0.0019) | 6.4E-24 | -0.0470 (0.0505) | 0.352 | 101.1 | 2.461 |
| Appendicular lean mass | Vascular dementia (FinnGen) | rs7485647 | 12:131631133 | A/G | 0.844 | -0.0261 (0.0026) | 1.0E-23 | -0.1016 (0.0563) | 0.071 | 100.8 | 3.893 |
| Appendicular lean mass | Vascular dementia (FinnGen) | rs2296316 | 14:65520246 | C/T | 0.464 | -0.0192 (0.0019) | 1.6E-23 | -0.0443 (0.0492) | 0.368 | 102.1 | 2.307 |
| Appendicular lean mass | Vascular dementia (FinnGen) | rs2019203 | 17:36908672 | A/C | 0.491 | 0.0189 (0.0019) | 1.8E-23 | 0.0289 (0.0491) | 0.557 | 99.0 | 1.529 |
| Appendicular lean mass | Vascular dementia (FinnGen) | rs17035465 | 2:46890294 | G/A | 0.221 | 0.0226 (0.0023) | 2.1E-23 | -0.0101 (0.0698) | 0.885 | 96.5 | -0.447 |
| Appendicular lean mass | Vascular dementia (FinnGen) | rs8077636 | 17:79421254 | T/G | 0.418 | -0.0192 (0.0019) | 2.5E-23 | 0.0551 (0.0514) | 0.283 | 102.1 | -2.870 |
| Appendicular lean mass | Vascular dementia (FinnGen) | rs6919321 | 6:33724004 | A/G | 0.608 | 0.0192 (0.0019) | 3.6E-23 | -0.0542 (0.0550) | 0.324 | 102.1 | -2.823 |
| Appendicular lean mass | Vascular dementia (FinnGen) | rs2140046 | 2:169706079 | C/T | 0.363 | -0.0192 (0.0019) | 5.5E-23 | 0.0347 (0.0498) | 0.486 | 102.1 | -1.807 |
| Appendicular lean mass | Vascular dementia (FinnGen) | rs1786263 | 18:13116432 | T/G | 0.606 | -0.0190 (0.0019) | 1.0E-22 | 0.0572 (0.0500) | 0.253 | 100.0 | -3.011 |
| Appendicular lean mass | Vascular dementia (FinnGen) | rs9807032 | 17:62757146 | C/T | 0.818 | -0.0240 (0.0024) | 1.3E-22 | -0.0090 (0.0667) | 0.893 | 100.0 | 0.375 |
| Appendicular lean mass | Vascular dementia (FinnGen) | rs140440099 | 3:50632595 | A/G | 0.023 | 0.0613 (0.0063) | 1.4E-22 | 0.0290 (0.1965) | 0.882 | 94.7 | 0.473 |
| Appendicular lean mass | Vascular dementia (FinnGen) | rs113852999 | 7:148649606 | A/G | 0.169 | 0.0246 (0.0025) | 1.8E-22 | 0.1157 (0.0647) | 0.074 | 96.8 | 4.703 |
| Appendicular lean mass | Vascular dementia (FinnGen) | rs35732917 | 7:73013269 | C/T | 0.284 | 0.0204 (0.0021) | 2.1E-22 | 0.0036 (0.0581) | 0.951 | 94.4 | 0.176 |
| Appendicular lean mass | Vascular dementia (FinnGen) | rs9344126 | 6:81907559 | C/T | 0.514 | -0.0185 (0.0019) | 2.2E-22 | -0.0495 (0.0502) | 0.324 | 94.8 | 2.676 |
| Appendicular lean mass | Vascular dementia (FinnGen) | rs3184504 | 12:111884608 | C/T | 0.517 | 0.0183 (0.0019) | 2.7E-22 | -0.0199 (0.0500) | 0.690 | 92.8 | -1.087 |
| Appendicular lean mass | Vascular dementia (FinnGen) | rs332116 | 10:28926099 | T/C | 0.280 | -0.0206 (0.0021) | 2.9E-22 | -0.0412 (0.0505) | 0.415 | 96.2 | 2.000 |
| Appendicular lean mass | Vascular dementia (FinnGen) | rs10202845 | 2:42575820 | G/A | 0.113 | -0.0288 (0.0030) | 5.3E-22 | -0.1171 (0.0805) | 0.146 | 92.2 | 4.066 |
| Appendicular lean mass | Vascular dementia (FinnGen) | rs2305141 | 2:233684402 | G/A | 0.596 | 0.0183 (0.0019) | 1.1E-21 | -0.0381 (0.0553) | 0.491 | 92.8 | -2.082 |
| Appendicular lean mass | Vascular dementia (FinnGen) | rs1405212 | 6:117490664 | C/T | 0.628 | 0.0187 (0.0019) | 1.1E-21 | -0.0722 (0.0519) | 0.164 | 96.9 | -3.861 |
| Appendicular lean mass | Vascular dementia (FinnGen) | rs9838614 | 3:38537671 | G/T | 0.388 | -0.0185 (0.0019) | 1.2E-21 | -0.0227 (0.0500) | 0.650 | 94.8 | 1.227 |
| Appendicular lean mass | Vascular dementia (FinnGen) | rs12679359 | 8:130724556 | T/G | 0.137 | -0.0263 (0.0028) | 1.4E-21 | 0.0391 (0.0704) | 0.579 | 88.2 | -1.487 |
| Appendicular lean mass | Vascular dementia (FinnGen) | rs6054390 | 20:6592094 | A/G | 0.628 | -0.0188 (0.0020) | 1.4E-21 | 0.1167 (0.0538) | 0.030 | 88.4 | -6.207 |
| Appendicular lean mass | Vascular dementia (FinnGen) | rs10945540 | 6:158738975 | A/G | 0.665 | -0.0190 (0.0020) | 1.8E-21 | 0.0193 (0.0505) | 0.702 | 90.2 | -1.016 |
| Appendicular lean mass | Vascular dementia (FinnGen) | rs670318 | 1:63727542 | C/T | 0.952 | 0.0413 (0.0044) | 2.5E-21 | 0.1977 (0.1056) | 0.061 | 88.1 | 4.787 |
| Appendicular lean mass | Vascular dementia (FinnGen) | rs112537273 | 8:38248306 | C/T | 0.230 | -0.0212 (0.0022) | 3.3E-21 | -0.0028 (0.0640) | 0.966 | 92.9 | 0.132 |
| Appendicular lean mass | Vascular dementia (FinnGen) | rs1040977 | 9:95554873 | T/C | 0.166 | -0.0240 (0.0025) | 3.9E-21 | -0.0396 (0.0773) | 0.608 | 92.2 | 1.650 |
| Appendicular lean mass | Vascular dementia (FinnGen) | rs7701233 | 5:171218388 | C/T | 0.428 | -0.0179 (0.0019) | 5.1E-21 | 0.0056 (0.0491) | 0.909 | 88.8 | -0.313 |
| Appendicular lean mass | Vascular dementia (FinnGen) | rs2212926 | 21:38066883 | A/C | 0.211 | -0.0220 (0.0023) | 7.7E-21 | 0.0673 (0.0702) | 0.338 | 91.5 | -3.059 |
| Appendicular lean mass | Vascular dementia (FinnGen) | rs10832963 | 11:18664241 | G/T | 0.745 | -0.0203 (0.0022) | 9.2E-21 | 0.0129 (0.0506) | 0.798 | 85.1 | -0.635 |
| Appendicular lean mass | Vascular dementia (FinnGen) | rs7636265 | 3:134374223 | A/G | 0.638 | -0.0183 (0.0020) | 9.4E-21 | 0.0018 (0.0534) | 0.973 | 83.7 | -0.098 |
| Appendicular lean mass | Vascular dementia (FinnGen) | rs9384679 | 6:108864419 | T/C | 0.370 | -0.0182 (0.0019) | 9.9E-21 | -0.0441 (0.0500) | 0.378 | 91.8 | 2.423 |
| Appendicular lean mass | Vascular dementia (FinnGen) | rs17478946 | 12:24093062 | G/A | 0.300 | -0.0192 (0.0021) | 1.0E-20 | -0.0729 (0.0561) | 0.194 | 83.6 | 3.797 |
| Appendicular lean mass | Vascular dementia (FinnGen) | rs9957318 | 18:33039106 | G/A | 0.348 | 0.0187 (0.0020) | 1.0E-20 | 0.0632 (0.0528) | 0.232 | 87.4 | 3.380 |
| Appendicular lean mass | Vascular dementia (FinnGen) | rs28787734 | 4:87560357 | T/C | 0.058 | 0.0385 (0.0041) | 1.1E-20 | 0.0901 (0.1523) | 0.554 | 88.2 | 2.340 |
| Appendicular lean mass | Vascular dementia (FinnGen) | rs1473441 | 8:49588463 | G/A | 0.724 | -0.0198 (0.0021) | 1.2E-20 | -0.0945 (0.0560) | 0.092 | 88.9 | 4.773 |
| Appendicular lean mass | Vascular dementia (FinnGen) | rs34287 | 5:67585143 | A/G | 0.341 | 0.0187 (0.0020) | 1.2E-20 | 0.0307 (0.0499) | 0.538 | 87.4 | 1.642 |
| Appendicular lean mass | Vascular dementia (FinnGen) | rs35816944 | 16:1828030 | A/G | 0.007 | -0.1088 (0.0117) | 1.3E-20 | 0.8606 (0.3176) | 0.007 | 86.5 | -7.910 |
| Appendicular lean mass | Vascular dementia (FinnGen) | rs7633464 | 3:98715823 | A/G | 0.479 | 0.0175 (0.0019) | 1.3E-20 | 0.0272 (0.0492) | 0.580 | 84.8 | 1.554 |
| Appendicular lean mass | Vascular dementia (FinnGen) | rs2993531 | 1:212190116 | C/A | 0.589 | 0.0176 (0.0019) | 1.7E-20 | -0.1014 (0.0510) | 0.046 | 85.8 | -5.761 |
| Appendicular lean mass | Vascular dementia (FinnGen) | rs9696116 | 9:139100805 | C/T | 0.318 | -0.0188 (0.0020) | 2.4E-20 | 0.0681 (0.0525) | 0.194 | 88.4 | -3.622 |
| Appendicular lean mass | Vascular dementia (FinnGen) | rs3103268 | 2:232984969 | T/C | 0.902 | 0.0291 (0.0031) | 2.7E-20 | 0.0146 (0.1121) | 0.896 | 88.1 | 0.502 |
| Appendicular lean mass | Vascular dementia (FinnGen) | rs4985445 | 16:69867835 | G/A | 0.457 | -0.0175 (0.0019) | 3.3E-20 | 0.0683 (0.0494) | 0.167 | 84.8 | -3.903 |
| Appendicular lean mass | Vascular dementia (FinnGen) | rs11590254 | 1:92316573 | T/A | 0.311 | 0.0186 (0.0020) | 4.3E-20 | 0.0102 (0.0513) | 0.842 | 86.5 | 0.548 |
| Appendicular lean mass | Vascular dementia (FinnGen) | rs73125634 | 20:20069826 | T/G | 0.278 | -0.0195 (0.0021) | 5.1E-20 | -0.0224 (0.0514) | 0.662 | 86.2 | 1.149 |
| Appendicular lean mass | Vascular dementia (FinnGen) | rs6874142 | 5:172753555 | G/T | 0.114 | 0.0288 (0.0031) | 5.1E-20 | -0.0483 (0.0858) | 0.573 | 86.3 | -1.677 |
| Appendicular lean mass | Vascular dementia (FinnGen) | rs140657345 | 15:41615909 | TCCTC/T | 0.252 | -0.0200 (0.0022) | 5.5E-20 | 0.0094 (0.0568) | 0.868 | 82.6 | -0.470 |
| Appendicular lean mass | Vascular dementia (FinnGen) | rs62466110 | 7:92623541 | C/T | 0.067 | -0.0371 (0.0041) | 5.7E-20 | -0.0730 (0.0734) | 0.320 | 81.9 | 1.968 |
| Appendicular lean mass | Vascular dementia (FinnGen) | rs12702693 | 7:8101039 | T/C | 0.454 | 0.0173 (0.0019) | 6.6E-20 | 0.0028 (0.0510) | 0.957 | 82.9 | 0.162 |
| Appendicular lean mass | Vascular dementia (FinnGen) | rs34345560 | 11:69081998 | A/G | 0.195 | 0.0219 (0.0024) | 7.1E-20 | -0.0666 (0.0568) | 0.241 | 83.3 | -3.041 |
| Appendicular lean mass | Vascular dementia (FinnGen) | rs12055045 | 5:137608056 | T/C | 0.206 | 0.0213 (0.0023) | 7.1E-20 | -0.0081 (0.0571) | 0.887 | 85.8 | -0.380 |
| Appendicular lean mass | Vascular dementia (FinnGen) | rs655113 | 6:52269151 | C/T | 0.301 | 0.0188 (0.0021) | 7.4E-20 | -0.0123 (0.0519) | 0.812 | 80.2 | -0.654 |
| Appendicular lean mass | Vascular dementia (FinnGen) | rs9832919 | 3:132184526 | G/A | 0.357 | -0.0179 (0.0020) | 7.9E-20 | 0.0050 (0.0514) | 0.922 | 80.1 | -0.279 |
| Appendicular lean mass | Vascular dementia (FinnGen) | rs8107967 | 19:7972615 | G/A | 0.566 | -0.0174 (0.0019) | 9.5E-20 | -0.0131 (0.0496) | 0.792 | 83.9 | 0.753 |
| Appendicular lean mass | Vascular dementia (FinnGen) | rs36012032 | 3:52814709 | A/C | 0.091 | 0.0298 (0.0033) | 9.9E-20 | 0.0854 (0.0878) | 0.331 | 81.5 | 2.866 |
| Appendicular lean mass | Vascular dementia (FinnGen) | rs59753424 | 20:54884760 | A/G | 0.267 | 0.0200 (0.0022) | 1.1E-19 | 0.1026 (0.0621) | 0.098 | 82.6 | 5.130 |
| Appendicular lean mass | Vascular dementia (FinnGen) | rs76693355 | 11:120292477 | C/T | 0.116 | -0.0268 (0.0030) | 1.2E-19 | 0.0675 (0.0742) | 0.363 | 79.8 | -2.519 |
| Appendicular lean mass | Vascular dementia (FinnGen) | rs78180894 | 17:7483662 | C/G | 0.074 | 0.0338 (0.0037) | 1.3E-19 | -0.1656 (0.1810) | 0.360 | 83.5 | -4.899 |
| Appendicular lean mass | Vascular dementia (FinnGen) | rs12533452 | 7:19016871 | T/C | 0.157 | 0.0237 (0.0026) | 1.3E-19 | -0.0431 (0.0704) | 0.540 | 83.1 | -1.819 |
| Appendicular lean mass | Vascular dementia (FinnGen) | rs77542162 | 17:67081278 | G/A | 0.023 | 0.0576 (0.0064) | 1.5E-19 | 0.2660 (0.3071) | 0.386 | 81.0 | 4.618 |
| Appendicular lean mass | Vascular dementia (FinnGen) | rs12997625 | 2:202970250 | T/C | 0.527 | -0.0170 (0.0019) | 1.5E-19 | 0.0019 (0.0496) | 0.969 | 80.1 | -0.112 |
| Appendicular lean mass | Vascular dementia (FinnGen) | rs591668 | 3:27535931 | A/G | 0.396 | -0.0174 (0.0019) | 2.0E-19 | 0.0166 (0.0500) | 0.740 | 83.9 | -0.954 |
| Appendicular lean mass | Vascular dementia (FinnGen) | rs11959466 | 5:42803824 | T/C | 0.056 | 0.0380 (0.0042) | 2.2E-19 | 0.0958 (0.0942) | 0.309 | 81.9 | 2.521 |
| Appendicular lean mass | Vascular dementia (FinnGen) | rs261999 | 5:95899427 | T/C | 0.370 | 0.0175 (0.0019) | 2.5E-19 | 0.0363 (0.0499) | 0.467 | 84.8 | 2.074 |
| Appendicular lean mass | Vascular dementia (FinnGen) | rs3063063 | 2:183231361 | TGAA/T | 0.686 | -0.0182 (0.0020) | 2.6E-19 | -0.0214 (0.0560) | 0.702 | 82.8 | 1.176 |
| Appendicular lean mass | Vascular dementia (FinnGen) | rs11672848 | 19:37570704 | T/C | 0.525 | -0.0171 (0.0019) | 7.7E-19 | -0.0636 (0.0489) | 0.194 | 81.0 | 3.719 |
| Appendicular lean mass | Vascular dementia (FinnGen) | rs894736 | 12:54418166 | G/A | 0.363 | -0.0174 (0.0020) | 1.1E-18 | -0.0636 (0.0491) | 0.195 | 75.7 | 3.655 |
| Appendicular lean mass | Vascular dementia (FinnGen) | rs2112617 | 17:46977125 | A/G | 0.529 | -0.0167 (0.0019) | 1.1E-18 | -0.0250 (0.0489) | 0.610 | 77.2 | 1.497 |
| Appendicular lean mass | Vascular dementia (FinnGen) | rs11049704 | 12:28691701 | G/C | 0.293 | -0.0183 (0.0021) | 1.1E-18 | 0.1315 (0.0558) | 0.019 | 75.9 | -7.186 |
| Appendicular lean mass | Vascular dementia (FinnGen) | rs700677 | 2:198702424 | A/C | 0.350 | 0.0173 (0.0020) | 1.1E-18 | 0.0138 (0.0547) | 0.801 | 74.8 | 0.798 |
| Appendicular lean mass | Vascular dementia (FinnGen) | rs6739278 | 2:44401055 | C/T | 0.808 | -0.0210 (0.0024) | 1.3E-18 | -0.0063 (0.0575) | 0.912 | 76.6 | 0.300 |
| Appendicular lean mass | Vascular dementia (FinnGen) | rs1805165 | 2:88874891 | A/C | 0.720 | -0.0184 (0.0021) | 1.3E-18 | 0.0724 (0.0519) | 0.163 | 76.8 | -3.935 |
| Appendicular lean mass | Vascular dementia (FinnGen) | rs6569643 | 6:130312646 | A/G | 0.526 | -0.0167 (0.0019) | 1.4E-18 | 0.0365 (0.0505) | 0.470 | 77.2 | -2.186 |
| Appendicular lean mass | Vascular dementia (FinnGen) | rs2052478 | 5:77437291 | T/C | 0.235 | -0.0195 (0.0022) | 1.8E-18 | 0.0378 (0.0639) | 0.554 | 78.6 | -1.938 |
| Appendicular lean mass | Vascular dementia (FinnGen) | rs9513510 | 13:99571922 | C/G | 0.699 | -0.0181 (0.0021) | 2.5E-18 | -0.0721 (0.0525) | 0.170 | 74.3 | 3.983 |
| Appendicular lean mass | Vascular dementia (FinnGen) | rs7731023 | 5:36181627 | G/A | 0.575 | 0.0166 (0.0019) | 3.5E-18 | 0.0880 (0.0513) | 0.087 | 76.3 | 5.301 |
| Appendicular lean mass | Vascular dementia (FinnGen) | rs2531991 | 16:4023553 | A/G | 0.748 | 0.0189 (0.0022) | 5.2E-18 | -0.0386 (0.0562) | 0.492 | 73.8 | -2.042 |
| Appendicular lean mass | Vascular dementia (FinnGen) | rs12909863 | 15:75825822 | C/G | 0.251 | 0.0189 (0.0022) | 6.0E-18 | -0.1433 (0.0588) | 0.015 | 73.8 | -7.582 |
| Appendicular lean mass | Vascular dementia (FinnGen) | rs12351226 | 9:98405230 | T/C | 0.171 | 0.0218 (0.0025) | 9.1E-18 | 0.0819 (0.0798) | 0.305 | 76.0 | 3.757 |
| Appendicular lean mass | Vascular dementia (FinnGen) | rs7512641 | 1:10822171 | A/T | 0.741 | -0.0185 (0.0022) | 9.9E-18 | 0.1213 (0.0536) | 0.024 | 70.7 | -6.557 |
| Appendicular lean mass | Vascular dementia (FinnGen) | rs7666804 | 4:57750148 | C/T | 0.389 | -0.0167 (0.0019) | 9.9E-18 | -0.1270 (0.0516) | 0.014 | 77.2 | 7.605 |
| Appendicular lean mass | Vascular dementia (FinnGen) | rs395980 | 4:177430072 | G/T | 0.264 | -0.0184 (0.0021) | 1.0E-17 | 0.0439 (0.0551) | 0.425 | 76.8 | -2.386 |
| Appendicular lean mass | Vascular dementia (FinnGen) | rs12037677 | 1:109918766 | C/T | 0.718 | -0.0178 (0.0021) | 1.2E-17 | 0.0722 (0.0541) | 0.182 | 71.8 | -4.056 |
| Appendicular lean mass | Vascular dementia (FinnGen) | rs73384223 | 9:3869315 | C/T | 0.197 | -0.0205 (0.0024) | 1.3E-17 | -0.1172 (0.0627) | 0.062 | 73.0 | 5.717 |
| Appendicular lean mass | Vascular dementia (FinnGen) | rs7598430 | 2:219193963 | T/C | 0.505 | -0.0160 (0.0019) | 1.4E-17 | 0.0866 (0.0496) | 0.081 | 70.9 | -5.412 |
| Appendicular lean mass | Vascular dementia (FinnGen) | rs2721940 | 8:116636281 | C/A | 0.602 | -0.0165 (0.0019) | 1.5E-17 | 0.0748 (0.0542) | 0.167 | 75.4 | -4.533 |
| Appendicular lean mass | Vascular dementia (FinnGen) | rs9817452 | 3:156795414 | T/G | 0.388 | 0.0165 (0.0019) | 1.9E-17 | 0.0390 (0.0532) | 0.464 | 75.4 | 2.364 |
| Appendicular lean mass | Vascular dementia (FinnGen) | rs10922475 | 1:89142142 | A/C | 0.539 | 0.0159 (0.0019) | 2.2E-17 | 0.0911 (0.0506) | 0.072 | 70.0 | 5.730 |
| Appendicular lean mass | Vascular dementia (FinnGen) | rs12299065 | 12:117442304 | A/G | 0.127 | -0.0240 (0.0028) | 2.2E-17 | 0.1376 (0.0705) | 0.051 | 73.5 | -5.733 |
| Appendicular lean mass | Vascular dementia (FinnGen) | rs56208656 | 19:3575343 | A/G | 0.110 | -0.0284 (0.0034) | 3.0E-17 | -0.0900 (0.0773) | 0.244 | 69.8 | 3.169 |
| Appendicular lean mass | Vascular dementia (FinnGen) | rs10040039 | 5:88354597 | G/A | 0.387 | -0.0163 (0.0019) | 3.3E-17 | -0.0179 (0.0537) | 0.739 | 73.6 | 1.098 |
| Appendicular lean mass | Vascular dementia (FinnGen) | rs59951000 | 18:74975828 | T/C | 0.042 | -0.0400 (0.0047) | 3.3E-17 | -0.0134 (0.0925) | 0.885 | 72.4 | 0.335 |
| Appendicular lean mass | Vascular dementia (FinnGen) | rs201570119 | 2:112256320 | C/T | 0.210 | 0.0194 (0.0023) | 5.5E-17 | 0.1129 (0.0663) | 0.089 | 71.2 | 5.820 |
| Appendicular lean mass | Vascular dementia (FinnGen) | rs2347603 | 2:47297426 | A/T | 0.742 | -0.0181 (0.0022) | 5.6E-17 | -0.0024 (0.0557) | 0.966 | 67.7 | 0.133 |
| Appendicular lean mass | Vascular dementia (FinnGen) | rs1341215 | 9:111662350 | A/G | 0.137 | 0.0229 (0.0027) | 6.3E-17 | 0.0180 (0.0637) | 0.777 | 71.9 | 0.786 |
| Appendicular lean mass | Vascular dementia (FinnGen) | rs10005035 | 4:12865684 | G/C | 0.282 | -0.0175 (0.0021) | 6.5E-17 | 0.0492 (0.0569) | 0.387 | 69.4 | -2.811 |
| Appendicular lean mass | Vascular dementia (FinnGen) | rs1556659 | 10:130834698 | T/C | 0.382 | 0.0163 (0.0020) | 7.2E-17 | -0.0321 (0.0540) | 0.552 | 66.4 | -1.969 |
| Appendicular lean mass | Vascular dementia (FinnGen) | rs10776560 | 10:50542358 | T/C | 0.499 | -0.0157 (0.0019) | 7.9E-17 | 0.0423 (0.0512) | 0.409 | 68.3 | -2.694 |
| Appendicular lean mass | Vascular dementia (FinnGen) | rs10514518 | 16:81579827 | T/C | 0.685 | 0.0171 (0.0020) | 8.3E-17 | 0.0909 (0.0544) | 0.095 | 73.1 | 5.316 |
| Appendicular lean mass | Vascular dementia (FinnGen) | rs112898929 | 16:88298795 | A/T | 0.080 | -0.0296 (0.0036) | 9.6E-17 | -0.0550 (0.0819) | 0.502 | 67.6 | 1.858 |
| Appendicular lean mass | Vascular dementia (FinnGen) | rs9784904 | 7:55835078 | C/T | 0.155 | -0.0217 (0.0026) | 9.7E-17 | 0.0926 (0.0584) | 0.113 | 69.7 | -4.267 |
| Appendicular lean mass | Vascular dementia (FinnGen) | rs1063582 | 8:23167353 | G/T | 0.765 | -0.0185 (0.0022) | 1.1E-16 | 0.0122 (0.0597) | 0.838 | 70.7 | -0.659 |
| Appendicular lean mass | Vascular dementia (FinnGen) | rs10823149 | 10:69935396 | T/G | 0.548 | 0.0157 (0.0019) | 1.2E-16 | 0.0002 (0.0497) | 0.997 | 68.3 | 0.013 |
| Appendicular lean mass | Vascular dementia (FinnGen) | rs4815952 | 20:6934897 | C/T | 0.522 | -0.0161 (0.0019) | 1.2E-16 | 0.0264 (0.0497) | 0.595 | 71.8 | -1.640 |
| Appendicular lean mass | Vascular dementia (FinnGen) | rs9809116 | 3:72397279 | G/A | 0.408 | -0.0160 (0.0019) | 1.3E-16 | 0.1152 (0.0523) | 0.028 | 70.9 | -7.200 |
| Appendicular lean mass | Vascular dementia (FinnGen) | rs35166681 | 6:85369727 | TA/T | 0.159 | 0.0214 (0.0026) | 1.5E-16 | 0.0598 (0.0698) | 0.392 | 67.8 | 2.794 |
| Appendicular lean mass | Vascular dementia (FinnGen) | rs12512942 | 4:177766307 | A/G | 0.638 | -0.0162 (0.0020) | 1.6E-16 | -0.0296 (0.0497) | 0.552 | 65.6 | 1.827 |
| Appendicular lean mass | Vascular dementia (FinnGen) | rs10843391 | 12:29508642 | G/A | 0.285 | 0.0172 (0.0021) | 2.1E-16 | -0.0340 (0.0539) | 0.528 | 67.1 | -1.977 |
| Appendicular lean mass | Vascular dementia (FinnGen) | rs9391254 | 6:105377347 | T/C | 0.321 | 0.0166 (0.0020) | 2.1E-16 | 0.0458 (0.0531) | 0.388 | 68.9 | 2.759 |
| Appendicular lean mass | Vascular dementia (FinnGen) | rs71635721 | 3:171960170 | G/C | 0.065 | 0.0316 (0.0039) | 3.5E-16 | -0.1804 (0.1185) | 0.128 | 65.7 | -5.709 |
| Appendicular lean mass | Vascular dementia (FinnGen) | rs56363908 | 7:96611052 | G/A | 0.042 | -0.0382 (0.0047) | 3.9E-16 | -0.0246 (0.1328) | 0.853 | 66.1 | 0.644 |
| Appendicular lean mass | Vascular dementia (FinnGen) | rs9479012 | 6:151657994 | G/A | 0.094 | 0.0263 (0.0032) | 4.1E-16 | -0.0887 (0.0858) | 0.302 | 67.5 | -3.373 |
| Appendicular lean mass | Vascular dementia (FinnGen) | rs6543146 | 2:103096695 | G/T | 0.558 | 0.0154 (0.0019) | 4.2E-16 | -0.0129 (0.0553) | 0.816 | 65.7 | -0.838 |
| Appendicular lean mass | Vascular dementia (FinnGen) | rs17138358 | 7:17920253 | C/G | 0.398 | 0.0157 (0.0019) | 4.4E-16 | -0.0793 (0.0535) | 0.138 | 68.3 | -5.051 |
| Appendicular lean mass | Vascular dementia (FinnGen) | rs7367519 | 1:204479176 | C/T | 0.685 | 0.0164 (0.0020) | 4.7E-16 | -0.1714 (0.0596) | 0.004 | 67.2 | -10.451 |
| Appendicular lean mass | Vascular dementia (FinnGen) | rs61729527 | 8:77761919 | T/C | 0.052 | -0.0346 (0.0043) | 4.9E-16 | -0.0926 (0.1010) | 0.359 | 64.8 | 2.676 |
| Appendicular lean mass | Vascular dementia (FinnGen) | rs10208668 | 2:20556151 | C/G | 0.035 | 0.0415 (0.0051) | 5.0E-16 | 0.1600 (0.1680) | 0.341 | 66.2 | 3.855 |
| Appendicular lean mass | Vascular dementia (FinnGen) | rs2287821 | 19:33935102 | T/C | 0.509 | -0.0153 (0.0019) | 7.0E-16 | 0.0700 (0.0493) | 0.156 | 64.8 | -4.575 |
| Appendicular lean mass | Vascular dementia (FinnGen) | rs35963161 | 20:49210635 | A/G | 0.476 | -0.0157 (0.0019) | 7.5E-16 | -0.0553 (0.0496) | 0.265 | 68.3 | 3.522 |
| Appendicular lean mass | Vascular dementia (FinnGen) | rs1056747 | 9:35690102 | G/A | 0.412 | -0.0155 (0.0019) | 8.0E-16 | 0.0475 (0.0500) | 0.343 | 66.5 | -3.065 |
| Appendicular lean mass | Vascular dementia (FinnGen) | rs11777835 | 8:144986103 | C/T | 0.473 | -0.0152 (0.0019) | 9.6E-16 | -0.0303 (0.0492) | 0.538 | 64.0 | 1.993 |
| Appendicular lean mass | Vascular dementia (FinnGen) | rs2165772 | 11:27091907 | A/G | 0.354 | 0.0160 (0.0020) | 1.0E-15 | 0.0003 (0.0509) | 0.995 | 64.0 | 0.019 |
| Appendicular lean mass | Vascular dementia (FinnGen) | rs2596144 | 12:122297738 | C/T | 0.870 | -0.0223 (0.0028) | 1.5E-15 | 0.1222 (0.0595) | 0.040 | 63.4 | -5.480 |
| Appendicular lean mass | Vascular dementia (FinnGen) | rs8904 | 14:35871217 | A/G | 0.363 | -0.0157 (0.0020) | 1.5E-15 | -0.0525 (0.0506) | 0.299 | 61.6 | 3.344 |
| Appendicular lean mass | Vascular dementia (FinnGen) | rs4504126 | 3:33600582 | C/A | 0.028 | 0.0460 (0.0058) | 1.6E-15 | -0.0636 (0.1930) | 0.742 | 62.9 | -1.383 |
| Appendicular lean mass | Vascular dementia (FinnGen) | rs2007022 | 20:3237674 | A/C | 0.232 | 0.0179 (0.0022) | 1.6E-15 | 0.0101 (0.0679) | 0.882 | 66.2 | 0.564 |
| Appendicular lean mass | Vascular dementia (FinnGen) | rs2324154 | 4:24027226 | A/C | 0.510 | 0.0150 (0.0019) | 1.9E-15 | 0.0348 (0.0495) | 0.482 | 62.3 | 2.320 |
| Appendicular lean mass | Vascular dementia (FinnGen) | rs704660 | 11:30447998 | T/C | 0.409 | 0.0153 (0.0019) | 2.3E-15 | -0.0709 (0.0500) | 0.156 | 64.8 | -4.634 |
| Appendicular lean mass | Vascular dementia (FinnGen) | rs4076108 | 3:13736088 | T/A | 0.245 | 0.0174 (0.0022) | 2.3E-15 | -0.0292 (0.0553) | 0.598 | 62.5 | -1.678 |
| Appendicular lean mass | Vascular dementia (FinnGen) | rs7646501 | 3:24079291 | G/A | 0.264 | -0.0170 (0.0021) | 2.3E-15 | 0.1553 (0.0542) | 0.004 | 65.5 | -9.135 |
| Appendicular lean mass | Vascular dementia (FinnGen) | rs16844417 | 1:172377745 | A/G | 0.124 | 0.0226 (0.0029) | 2.6E-15 | 0.0280 (0.0731) | 0.702 | 60.7 | 1.239 |
| Appendicular lean mass | Vascular dementia (FinnGen) | rs12371664 | 12:91130760 | G/A | 0.272 | -0.0168 (0.0021) | 2.7E-15 | 0.0764 (0.0577) | 0.185 | 64.0 | -4.548 |
| Appendicular lean mass | Vascular dementia (FinnGen) | rs3121580 | 1:218492121 | C/T | 0.827 | 0.0197 (0.0025) | 2.7E-15 | -0.0601 (0.0628) | 0.338 | 62.1 | -3.051 |
| Appendicular lean mass | Vascular dementia (FinnGen) | rs7516643 | 1:33873010 | C/T | 0.672 | 0.0157 (0.0020) | 3.1E-15 | 0.0232 (0.0517) | 0.654 | 61.6 | 1.478 |
| Appendicular lean mass | Vascular dementia (FinnGen) | rs7228151 | 18:57181694 | C/T | 0.206 | -0.0185 (0.0023) | 3.2E-15 | 0.0218 (0.0555) | 0.694 | 64.7 | -1.178 |
| Appendicular lean mass | Vascular dementia (FinnGen) | rs35436119 | 9:119303347 | A/G | 0.075 | 0.0282 (0.0036) | 3.3E-15 | -0.2158 (0.1410) | 0.126 | 61.4 | -7.652 |
| Appendicular lean mass | Vascular dementia (FinnGen) | rs4903321 | 14:75911083 | A/G | 0.444 | -0.0151 (0.0019) | 3.4E-15 | -0.0261 (0.0493) | 0.596 | 63.2 | 1.728 |
| Appendicular lean mass | Vascular dementia (FinnGen) | rs11991823 | 8:92188440 | G/A | 0.679 | 0.0161 (0.0020) | 3.5E-15 | 0.0085 (0.0513) | 0.868 | 64.8 | 0.528 |
| Appendicular lean mass | Vascular dementia (FinnGen) | rs72829852 | 17:46633974 | T/C | 0.062 | 0.0309 (0.0039) | 3.7E-15 | -0.0014 (0.0974) | 0.989 | 62.8 | -0.045 |
| Appendicular lean mass | Vascular dementia (FinnGen) | rs13170063 | 5:157895013 | A/G | 0.592 | -0.0152 (0.0019) | 4.1E-15 | -0.0200 (0.0519) | 0.700 | 64.0 | 1.316 |
| Appendicular lean mass | Vascular dementia (FinnGen) | rs10822117 | 10:52786701 | G/A | 0.237 | -0.0176 (0.0022) | 4.2E-15 | 0.0639 (0.0563) | 0.256 | 64.0 | -3.631 |
| Appendicular lean mass | Vascular dementia (FinnGen) | rs7761910 | 6:155553832 | T/C | 0.309 | -0.0160 (0.0020) | 5.1E-15 | 0.0002 (0.0527) | 0.997 | 64.0 | -0.013 |
| Appendicular lean mass | Vascular dementia (FinnGen) | rs10796828 | 11:69490346 | G/T | 0.635 | 0.0154 (0.0020) | 5.8E-15 | 0.0224 (0.0563) | 0.691 | 59.3 | 1.455 |
| Appendicular lean mass | Vascular dementia (FinnGen) | rs28817902 | 5:138916010 | G/A | 0.118 | 0.0230 (0.0029) | 6.2E-15 | -0.0295 (0.0986) | 0.765 | 62.9 | -1.283 |
| Appendicular lean mass | Vascular dementia (FinnGen) | rs11021305 | 11:95498269 | C/T | 0.384 | 0.0152 (0.0019) | 6.3E-15 | -0.0903 (0.0569) | 0.112 | 64.0 | -5.941 |
| Appendicular lean mass | Vascular dementia (FinnGen) | rs34786000 | 19:9942123 | T/G | 0.554 | -0.0150 (0.0019) | 6.6E-15 | -0.0055 (0.0496) | 0.911 | 62.3 | 0.367 |
| Appendicular lean mass | Vascular dementia (FinnGen) | rs62501195 | 8:24041988 | C/A | 0.171 | -0.0198 (0.0025) | 8.1E-15 | -0.0185 (0.0585) | 0.752 | 62.7 | 0.934 |
| Appendicular lean mass | Vascular dementia (FinnGen) | rs4776624 | 15:73359881 | T/C | 0.397 | -0.0150 (0.0019) | 1.2E-14 | 0.0594 (0.0525) | 0.257 | 62.3 | -3.960 |
| Appendicular lean mass | Vascular dementia (FinnGen) | rs7320878 | 13:91994132 | A/G | 0.603 | -0.0150 (0.0019) | 1.3E-14 | -0.0206 (0.0493) | 0.675 | 62.3 | 1.373 |
| Appendicular lean mass | Vascular dementia (FinnGen) | rs9910161 | 17:59606244 | G/A | 0.285 | 0.0162 (0.0021) | 1.6E-14 | 0.0079 (0.0558) | 0.887 | 59.5 | 0.488 |
| Appendicular lean mass | Vascular dementia (FinnGen) | rs1035583 | 2:207326937 | A/G | 0.618 | 0.0148 (0.0019) | 2.0E-14 | 0.0693 (0.0493) | 0.160 | 60.7 | 4.682 |
| Appendicular lean mass | Vascular dementia (FinnGen) | rs9394578 | 6:39165859 | A/C | 0.258 | -0.0164 (0.0022) | 2.6E-14 | -0.0029 (0.0628) | 0.963 | 55.6 | 0.177 |
| Appendicular lean mass | Vascular dementia (FinnGen) | rs73382475 | 21:47549715 | T/C | 0.089 | 0.0254 (0.0033) | 3.0E-14 | -0.1659 (0.0872) | 0.057 | 59.2 | -6.532 |
| Appendicular lean mass | Vascular dementia (FinnGen) | rs6721191 | 2:10190115 | G/A | 0.578 | -0.0144 (0.0019) | 3.2E-14 | -0.0639 (0.0553) | 0.248 | 57.4 | 4.438 |
| Appendicular lean mass | Vascular dementia (FinnGen) | rs2609334 | 6:168847175 | C/T | 0.226 | -0.0171 (0.0022) | 3.4E-14 | -0.0367 (0.0527) | 0.486 | 60.4 | 2.146 |
| Appendicular lean mass | Vascular dementia (FinnGen) | rs9525326 | 13:115075715 | G/A | 0.188 | -0.0184 (0.0024) | 3.6E-14 | -0.0007 (0.0628) | 0.991 | 58.8 | 0.038 |
| Appendicular lean mass | Vascular dementia (FinnGen) | rs11673381 | 19:4506296 | G/A | 0.530 | -0.0144 (0.0019) | 3.7E-14 | -0.0874 (0.0495) | 0.078 | 57.4 | 6.069 |
| Appendicular lean mass | Vascular dementia (FinnGen) | rs74379684 | 15:94050205 | T/C | 0.075 | -0.0272 (0.0036) | 4.4E-14 | -0.1364 (0.0872) | 0.118 | 57.1 | 5.015 |
| Appendicular lean mass | Vascular dementia (FinnGen) | rs12662115 | 6:169360124 | G/A | 0.327 | -0.0152 (0.0020) | 4.5E-14 | -0.0693 (0.0498) | 0.164 | 57.8 | 4.559 |
| Appendicular lean mass | Vascular dementia (FinnGen) | rs165849 | 22:19958669 | A/G | 0.698 | 0.0157 (0.0021) | 4.6E-14 | 0.0899 (0.0527) | 0.088 | 55.9 | 5.726 |
| Appendicular lean mass | Vascular dementia (FinnGen) | rs11198591 | 10:120515892 | A/G | 0.368 | 0.0148 (0.0020) | 4.9E-14 | -0.0430 (0.0513) | 0.403 | 54.8 | -2.905 |
| Appendicular lean mass | Vascular dementia (FinnGen) | rs7286917 | 22:39860868 | G/A | 0.746 | 0.0171 (0.0023) | 5.2E-14 | 0.0399 (0.0561) | 0.477 | 55.3 | 2.333 |
| Appendicular lean mass | Vascular dementia (FinnGen) | rs1557341 | 18:35127427 | C/A | 0.667 | 0.0153 (0.0020) | 5.8E-14 | -0.0243 (0.0550) | 0.658 | 58.5 | -1.588 |
| Appendicular lean mass | Vascular dementia (FinnGen) | rs599004 | 6:140439740 | T/C | 0.281 | -0.0157 (0.0021) | 6.5E-14 | -0.0091 (0.0599) | 0.880 | 55.9 | 0.580 |
| Appendicular lean mass | Vascular dementia (FinnGen) | rs1290786 | 3:169097381 | T/C | 0.431 | -0.0143 (0.0019) | 7.1E-14 | -0.0294 (0.0502) | 0.558 | 56.6 | 2.056 |
| Appendicular lean mass | Vascular dementia (FinnGen) | rs4622329 | 12:102321935 | A/G | 0.350 | 0.0149 (0.0020) | 8.6E-14 | 0.0559 (0.0497) | 0.260 | 55.5 | 3.752 |
| Appendicular lean mass | Vascular dementia (FinnGen) | rs73040028 | 3:23171805 | T/C | 0.229 | 0.0168 (0.0022) | 9.0E-14 | 0.0823 (0.0562) | 0.143 | 58.3 | 4.899 |
| Appendicular lean mass | Vascular dementia (FinnGen) | rs10810474 | 9:15911359 | T/C | 0.418 | 0.0143 (0.0019) | 9.6E-14 | -0.0263 (0.0492) | 0.593 | 56.6 | -1.839 |
| Appendicular lean mass | Vascular dementia (FinnGen) | rs2232460 | 1:6659505 | A/G | 0.334 | 0.0148 (0.0020) | 1.0E-13 | -0.0086 (0.0549) | 0.876 | 54.8 | -0.581 |
| Appendicular lean mass | Vascular dementia (FinnGen) | rs3760456 | 17:27948844 | T/C | 0.440 | -0.0142 (0.0019) | 1.1E-13 | 0.0207 (0.0491) | 0.673 | 55.9 | -1.458 |
| Appendicular lean mass | Vascular dementia (FinnGen) | rs2807339 | 1:22578063 | C/T | 0.759 | 0.0162 (0.0022) | 1.2E-13 | -0.0440 (0.0584) | 0.452 | 54.2 | -2.716 |
| Appendicular lean mass | Vascular dementia (FinnGen) | rs3798429 | 6:76604573 | G/T | 0.498 | 0.0140 (0.0019) | 1.3E-13 | -0.0532 (0.0494) | 0.281 | 54.3 | -3.800 |
| Appendicular lean mass | Vascular dementia (FinnGen) | rs78051210 | 6:131379491 | C/T | 0.077 | 0.0263 (0.0036) | 1.6E-13 | -0.1719 (0.1217) | 0.158 | 53.4 | -6.536 |
| Appendicular lean mass | Vascular dementia (FinnGen) | rs11927331 | 3:32937008 | A/C | 0.331 | -0.0147 (0.0020) | 1.9E-13 | 0.0124 (0.0513) | 0.809 | 54.0 | -0.844 |
| Appendicular lean mass | Vascular dementia (FinnGen) | rs17400325 | 2:178565913 | C/T | 0.042 | 0.0345 (0.0047) | 2.1E-13 | -0.0648 (0.1708) | 0.705 | 53.9 | -1.878 |
| Appendicular lean mass | Vascular dementia (FinnGen) | rs12344515 | 9:113801231 | T/C | 0.240 | -0.0163 (0.0022) | 2.3E-13 | 0.0657 (0.0605) | 0.278 | 54.9 | -4.031 |
| Appendicular lean mass | Vascular dementia (FinnGen) | rs7225068 | 17:60666425 | G/A | 0.591 | 0.0141 (0.0019) | 2.5E-13 | -0.0302 (0.0500) | 0.546 | 55.1 | -2.142 |
| Appendicular lean mass | Vascular dementia (FinnGen) | rs35631698 | 21:16549469 | GC/G | 0.375 | 0.0145 (0.0020) | 2.6E-13 | -0.0309 (0.0497) | 0.533 | 52.6 | -2.131 |
| Appendicular lean mass | Vascular dementia (FinnGen) | rs2138374 | 2:190014317 | C/T | 0.696 | -0.0149 (0.0020) | 2.8E-13 | 0.0816 (0.0517) | 0.114 | 55.5 | -5.477 |
| Appendicular lean mass | Vascular dementia (FinnGen) | rs35268848 | 16:67927240 | A/C | 0.012 | 0.0737 (0.0101) | 2.8E-13 | 0.0524 (0.1720) | 0.761 | 53.2 | 0.711 |
| Appendicular lean mass | Vascular dementia (FinnGen) | rs147233090 | 15:44028047 | T/C | 0.025 | -0.0446 (0.0061) | 4.0E-13 | -0.0346 (0.3222) | 0.914 | 53.5 | 0.776 |
| Appendicular lean mass | Vascular dementia (FinnGen) | rs4733775 | 8:131213570 | A/G | 0.364 | 0.0143 (0.0020) | 4.1E-13 | -0.0825 (0.0519) | 0.112 | 51.1 | -5.769 |
| Appendicular lean mass | Vascular dementia (FinnGen) | rs610694 | 12:121304826 | C/T | 0.490 | 0.0136 (0.0019) | 4.3E-13 | -0.0287 (0.0493) | 0.561 | 51.2 | -2.110 |
| Appendicular lean mass | Vascular dementia (FinnGen) | rs5753518 | 22:31631314 | A/G | 0.090 | 0.0242 (0.0033) | 5.0E-13 | 0.0132 (0.0714) | 0.853 | 53.8 | 0.545 |
| Appendicular lean mass | Vascular dementia (FinnGen) | rs72976986 | 19:4050424 | A/G | 0.190 | 0.0176 (0.0024) | 5.3E-13 | -0.0067 (0.0678) | 0.921 | 53.8 | -0.381 |
| Appendicular lean mass | Vascular dementia (FinnGen) | rs56207248 | 2:25065986 | T/C | 0.066 | -0.0273 (0.0038) | 6.0E-13 | 0.1045 (0.1043) | 0.317 | 51.6 | -3.828 |
| Appendicular lean mass | Vascular dementia (FinnGen) | rs1487441 | 6:98553894 | A/G | 0.484 | 0.0136 (0.0019) | 7.5E-13 | -0.0136 (0.0492) | 0.782 | 51.2 | -1.000 |
| Appendicular lean mass | Vascular dementia (FinnGen) | rs1216743 | 11:100573120 | A/G | 0.719 | -0.0152 (0.0021) | 8.0E-13 | -0.0044 (0.0551) | 0.937 | 52.4 | 0.289 |
| Appendicular lean mass | Vascular dementia (FinnGen) | rs6975015 | 7:50617215 | A/G | 0.117 | 0.0211 (0.0029) | 8.0E-13 | -0.0912 (0.0846) | 0.281 | 52.9 | -4.322 |
| Appendicular lean mass | Vascular dementia (FinnGen) | rs10453441 | 22:46363739 | G/A | 0.402 | -0.0139 (0.0020) | 9.1E-13 | -0.0267 (0.0530) | 0.615 | 48.3 | 1.921 |
| Appendicular lean mass | Vascular dementia (FinnGen) | rs1330826 | 9:85129970 | C/G | 0.227 | 0.0162 (0.0023) | 1.0E-12 | 0.0289 (0.0569) | 0.611 | 49.6 | 1.784 |
| Appendicular lean mass | Vascular dementia (FinnGen) | rs55758152 | 5:171317318 | A/G | 0.326 | 0.0145 (0.0020) | 1.1E-12 | 0.0777 (0.0523) | 0.138 | 52.6 | 5.359 |
| Appendicular lean mass | Vascular dementia (FinnGen) | rs143076454 | 16:921179 | A/G | 0.019 | -0.0499 (0.0070) | 1.1E-12 | 0.2133 (0.2115) | 0.313 | 50.8 | -4.275 |
| Appendicular lean mass | Vascular dementia (FinnGen) | rs60408354 | 7:70158495 | A/G | 0.073 | 0.0259 (0.0036) | 1.2E-12 | -0.0554 (0.0910) | 0.542 | 51.8 | -2.139 |
| Appendicular lean mass | Vascular dementia (FinnGen) | rs853168 | 5:142538764 | C/G | 0.247 | 0.0156 (0.0022) | 1.3E-12 | -0.1438 (0.0611) | 0.019 | 50.3 | -9.218 |
| Appendicular lean mass | Vascular dementia (FinnGen) | rs72771070 | 16:19993750 | T/C | 0.283 | 0.0150 (0.0021) | 1.3E-12 | -0.0129 (0.0525) | 0.805 | 51.0 | -0.860 |
| Appendicular lean mass | Vascular dementia (FinnGen) | rs12672217 | 7:156310948 | A/G | 0.361 | 0.0139 (0.0020) | 1.3E-12 | -0.0903 (0.0496) | 0.069 | 48.3 | -6.496 |
| Appendicular lean mass | Vascular dementia (FinnGen) | rs2578565 | 5:5460569 | T/C | 0.657 | -0.0141 (0.0020) | 1.4E-12 | 0.0780 (0.0505) | 0.122 | 49.7 | -5.532 |
| Appendicular lean mass | Vascular dementia (FinnGen) | rs572474 | 10:89791654 | G/A | 0.166 | 0.0180 (0.0025) | 1.5E-12 | 0.0577 (0.0656) | 0.380 | 51.8 | 3.206 |
| Appendicular lean mass | Vascular dementia (FinnGen) | rs17197114 | 14:21894526 | C/T | 0.177 | 0.0177 (0.0025) | 1.5E-12 | -0.1581 (0.0688) | 0.022 | 50.1 | -8.932 |
| Appendicular lean mass | Vascular dementia (FinnGen) | rs2289976 | 2:85818797 | A/G | 0.305 | 0.0144 (0.0020) | 1.6E-12 | -0.0316 (0.0580) | 0.587 | 51.8 | -2.194 |
| Appendicular lean mass | Vascular dementia (FinnGen) | rs4847378 | 1:93324634 | T/G | 0.612 | 0.0136 (0.0019) | 1.6E-12 | -0.0151 (0.0505) | 0.766 | 51.2 | -1.110 |
| Appendicular lean mass | Vascular dementia (FinnGen) | rs73114154 | 20:31449386 | T/C | 0.388 | 0.0138 (0.0020) | 1.7E-12 | 0.0543 (0.0497) | 0.275 | 47.6 | 3.935 |
| Appendicular lean mass | Vascular dementia (FinnGen) | rs7107356 | 11:47676170 | G/A | 0.507 | 0.0133 (0.0019) | 1.9E-12 | 0.0080 (0.0502) | 0.874 | 49.0 | 0.602 |
| Appendicular lean mass | Vascular dementia (FinnGen) | rs6129229 | 20:37882317 | G/A | 0.075 | -0.0257 (0.0036) | 1.9E-12 | 0.2131 (0.0921) | 0.021 | 51.0 | -8.292 |
| Appendicular lean mass | Vascular dementia (FinnGen) | rs177592 | 7:28559785 | C/G | 0.101 | -0.0220 (0.0031) | 2.0E-12 | -0.0609 (0.0901) | 0.499 | 50.4 | 2.768 |
| Appendicular lean mass | Vascular dementia (FinnGen) | rs2607234 | 19:35563834 | G/A | 0.948 | -0.0302 (0.0043) | 2.0E-12 | -0.0612 (0.1065) | 0.566 | 49.3 | 2.026 |
| Appendicular lean mass | Vascular dementia (FinnGen) | rs1977337 | 1:67419517 | C/T | 0.851 | 0.0186 (0.0026) | 2.1E-12 | -0.0691 (0.0592) | 0.243 | 51.2 | -3.715 |
| Appendicular lean mass | Vascular dementia (FinnGen) | rs6693481 | 1:203766395 | C/T | 0.695 | -0.0143 (0.0020) | 2.2E-12 | -0.0235 (0.0517) | 0.650 | 51.1 | 1.643 |
| Appendicular lean mass | Vascular dementia (FinnGen) | rs7574162 | 2:172133086 | T/C | 0.757 | 0.0153 (0.0022) | 2.4E-12 | 0.0522 (0.0592) | 0.378 | 48.4 | 3.412 |
| Appendicular lean mass | Vascular dementia (FinnGen) | rs310796 | 12:77453226 | T/G | 0.681 | 0.0142 (0.0020) | 2.5E-12 | 0.0290 (0.0555) | 0.601 | 50.4 | 2.042 |
| Appendicular lean mass | Vascular dementia (FinnGen) | rs11720869 | 3:185619716 | A/G | 0.669 | 0.0141 (0.0020) | 2.5E-12 | -0.0463 (0.0501) | 0.355 | 49.7 | -3.284 |
| Appendicular lean mass | Vascular dementia (FinnGen) | rs1717720 | 7:38112615 | G/A | 0.654 | 0.0139 (0.0020) | 2.7E-12 | -0.0392 (0.0559) | 0.483 | 48.3 | -2.820 |
| Appendicular lean mass | Vascular dementia (FinnGen) | rs75022676 | 2:60293216 | A/G | 0.208 | -0.0163 (0.0023) | 2.8E-12 | 0.0214 (0.0561) | 0.704 | 50.2 | -1.313 |
| Appendicular lean mass | Vascular dementia (FinnGen) | rs76488803 | 4:157770271 | A/G | 0.083 | -0.0240 (0.0034) | 3.0E-12 | -0.1600 (0.1095) | 0.144 | 49.8 | 6.667 |
| Appendicular lean mass | Vascular dementia (FinnGen) | rs7692387 | 4:156635309 | A/G | 0.185 | 0.0169 (0.0024) | 3.5E-12 | -0.0561 (0.0581) | 0.334 | 49.6 | -3.320 |
| Appendicular lean mass | Vascular dementia (FinnGen) | rs112873218 | 11:1960119 | T/C | 0.105 | 0.0216 (0.0031) | 4.0E-12 | -0.0464 (0.0773) | 0.549 | 48.5 | -2.148 |
| Appendicular lean mass | Vascular dementia (FinnGen) | rs7259285 | 19:17153149 | A/G | 0.444 | -0.0132 (0.0019) | 5.1E-12 | -0.0197 (0.0492) | 0.690 | 48.3 | 1.492 |
| Appendicular lean mass | Vascular dementia (FinnGen) | rs36052389 | 4:39357183 | AT/A | 0.455 | 0.0132 (0.0019) | 5.3E-12 | -0.0352 (0.0504) | 0.485 | 48.3 | -2.667 |
| Appendicular lean mass | Vascular dementia (FinnGen) | rs9610447 | 22:20768891 | T/C | 0.747 | 0.0152 (0.0022) | 5.3E-12 | -0.0060 (0.0586) | 0.918 | 47.7 | -0.395 |
| Appendicular lean mass | Vascular dementia (FinnGen) | rs2925155 | 8:75886297 | T/C | 0.261 | -0.0150 (0.0022) | 5.5E-12 | 0.0615 (0.0554) | 0.267 | 46.5 | -4.100 |
| Appendicular lean mass | Vascular dementia (FinnGen) | rs17681189 | 2:65976175 | A/C | 0.423 | -0.0131 (0.0019) | 5.8E-12 | 0.0271 (0.0495) | 0.584 | 47.5 | -2.069 |
| Appendicular lean mass | Vascular dementia (FinnGen) | rs7679276 | 4:146860186 | G/A | 0.954 | -0.0330 (0.0048) | 5.9E-12 | -0.2623 (0.2120) | 0.216 | 47.3 | 7.948 |
| Appendicular lean mass | Vascular dementia (FinnGen) | rs75478182 | 20:50807839 | C/G | 0.088 | 0.0230 (0.0033) | 6.6E-12 | -0.0678 (0.0973) | 0.486 | 48.6 | -2.948 |
| Appendicular lean mass | Vascular dementia (FinnGen) | rs2343034 | 20:35860288 | A/T | 0.809 | 0.0166 (0.0024) | 6.7E-12 | 0.0129 (0.0537) | 0.809 | 47.8 | 0.777 |
| Appendicular lean mass | Vascular dementia (FinnGen) | rs10427685 | 22:46440359 | G/C | 0.064 | 0.0269 (0.0039) | 7.0E-12 | 0.0775 (0.1059) | 0.464 | 47.6 | 2.881 |
| Appendicular lean mass | Vascular dementia (FinnGen) | rs12463908 | 2:121470971 | C/A | 0.188 | -0.0165 (0.0024) | 7.3E-12 | -0.0434 (0.0610) | 0.476 | 47.3 | 2.630 |
| Appendicular lean mass | Vascular dementia (FinnGen) | rs10008637 | 4:77414144 | C/T | 0.462 | -0.0130 (0.0019) | 7.5E-12 | -0.0462 (0.0494) | 0.350 | 46.8 | 3.554 |
| Appendicular lean mass | Vascular dementia (FinnGen) | rs2125125 | 3:142600087 | C/T | 0.783 | 0.0158 (0.0023) | 7.8E-12 | 0.0466 (0.0679) | 0.492 | 47.2 | 2.949 |
| Appendicular lean mass | Vascular dementia (FinnGen) | rs61827272 | 1:203810763 | C/T | 0.275 | 0.0144 (0.0021) | 7.9E-12 | 0.0284 (0.0539) | 0.599 | 47.0 | 1.972 |
| Appendicular lean mass | Vascular dementia (FinnGen) | rs78444298 | 1:184672098 | A/G | 0.020 | -0.0465 (0.0068) | 8.3E-12 | 0.1594 (0.1842) | 0.387 | 46.8 | -3.428 |
| Appendicular lean mass | Vascular dementia (FinnGen) | rs116493405 | 3:114733556 | A/G | 0.054 | 0.0287 (0.0042) | 9.5E-12 | 0.0691 (0.1449) | 0.633 | 46.7 | 2.408 |
| Appendicular lean mass | Vascular dementia (FinnGen) | rs61919240 | 12:8831954 | A/T | 0.324 | 0.0137 (0.0020) | 9.8E-12 | -0.0288 (0.0541) | 0.595 | 46.9 | -2.102 |
| Appendicular lean mass | Vascular dementia (FinnGen) | rs963317 | 4:45129970 | G/A | 0.663 | -0.0136 (0.0020) | 1.0E-11 | -0.0001 (0.0526) | 0.998 | 46.2 | 0.007 |
| Appendicular lean mass | Vascular dementia (FinnGen) | rs945508 | 1:156907081 | C/T | 0.536 | -0.0128 (0.0019) | 1.1E-11 | -0.0343 (0.0494) | 0.487 | 45.4 | 2.680 |
| Appendicular lean mass | Vascular dementia (FinnGen) | rs67625472 | 19:4968620 | C/T | 0.276 | -0.0144 (0.0021) | 1.3E-11 | -0.0035 (0.0571) | 0.951 | 47.0 | 0.243 |
| Appendicular lean mass | Vascular dementia (FinnGen) | rs2663126 | 15:99563857 | A/G | 0.691 | -0.0139 (0.0021) | 1.4E-11 | -0.0091 (0.0582) | 0.876 | 43.8 | 0.655 |
| Appendicular lean mass | Vascular dementia (FinnGen) | rs115233595 | 4:119744214 | C/T | 0.062 | -0.0264 (0.0039) | 1.4E-11 | 0.0500 (0.0841) | 0.553 | 45.8 | -1.894 |
| Appendicular lean mass | Vascular dementia (FinnGen) | rs12340775 | 9:13226945 | A/G | 0.055 | -0.0287 (0.0043) | 1.7E-11 | -0.0242 (0.0907) | 0.790 | 44.5 | 0.843 |
| Appendicular lean mass | Vascular dementia (FinnGen) | rs2252031 | 12:103248463 | C/T | 0.846 | -0.0176 (0.0026) | 1.8E-11 | -0.0100 (0.0841) | 0.905 | 45.8 | 0.568 |
| Appendicular lean mass | Vascular dementia (FinnGen) | rs6923230 | 6:88213949 | C/T | 0.534 | -0.0127 (0.0019) | 1.8E-11 | -0.0012 (0.0494) | 0.980 | 44.7 | 0.094 |
| Appendicular lean mass | Vascular dementia (FinnGen) | rs10461725 | 5:39437129 | C/G | 0.656 | 0.0134 (0.0020) | 2.0E-11 | -0.0053 (0.0501) | 0.917 | 44.9 | -0.396 |
| Appendicular lean mass | Vascular dementia (FinnGen) | rs501250 | 5:112155793 | C/G | 0.619 | -0.0130 (0.0019) | 2.1E-11 | 0.0034 (0.0501) | 0.946 | 46.8 | -0.262 |
| Appendicular lean mass | Vascular dementia (FinnGen) | rs10840399 | 11:10399121 | T/C | 0.101 | -0.0209 (0.0031) | 2.8E-11 | -0.0357 (0.0730) | 0.625 | 45.5 | 1.708 |
| Appendicular lean mass | Vascular dementia (FinnGen) | rs7543202 | 1:73872885 | G/A | 0.623 | 0.0129 (0.0019) | 2.9E-11 | -0.0089 (0.0508) | 0.861 | 46.1 | -0.690 |
| Appendicular lean mass | Vascular dementia (FinnGen) | rs2678898 | 2:58171964 | C/T | 0.380 | -0.0129 (0.0019) | 3.1E-11 | -0.0140 (0.0509) | 0.783 | 46.1 | 1.085 |
| Appendicular lean mass | Vascular dementia (FinnGen) | rs2994329 | 1:243647921 | T/C | 0.202 | 0.0155 (0.0023) | 3.1E-11 | -0.0030 (0.0587) | 0.959 | 45.4 | -0.194 |
| Appendicular lean mass | Vascular dementia (FinnGen) | rs75702986 | 19:35566151 | A/G | 0.186 | -0.0163 (0.0025) | 3.1E-11 | 0.0117 (0.0612) | 0.849 | 42.5 | -0.718 |
| Appendicular lean mass | Vascular dementia (FinnGen) | rs2268718 | 6:52415023 | T/C | 0.270 | 0.0141 (0.0021) | 3.2E-11 | 0.0030 (0.0516) | 0.954 | 45.1 | 0.213 |
| Appendicular lean mass | Vascular dementia (FinnGen) | rs8084413 | 18:22869123 | A/G | 0.469 | -0.0127 (0.0019) | 3.2E-11 | 0.0498 (0.0497) | 0.316 | 44.7 | -3.921 |
| Appendicular lean mass | Vascular dementia (FinnGen) | rs34522021 | 9:23350420 | T/C | 0.455 | 0.0126 (0.0019) | 3.4E-11 | -0.0398 (0.0493) | 0.420 | 44.0 | -3.159 |
| Appendicular lean mass | Vascular dementia (FinnGen) | rs137998108 | 12:124322727 | CA/C | 0.133 | -0.0188 (0.0028) | 3.6E-11 | -0.0937 (0.0827) | 0.257 | 45.1 | 4.984 |
| Appendicular lean mass | Vascular dementia (FinnGen) | rs2126942 | 5:98155979 | C/T | 0.580 | -0.0127 (0.0019) | 3.8E-11 | 0.0745 (0.0510) | 0.144 | 44.7 | -5.866 |
| Appendicular lean mass | Vascular dementia (FinnGen) | rs10827415 | 10:35042616 | G/T | 0.261 | -0.0142 (0.0021) | 3.9E-11 | -0.0580 (0.0626) | 0.354 | 45.7 | 4.085 |
| Appendicular lean mass | Vascular dementia (FinnGen) | rs116008080 | 16:67254841 | A/G | 0.024 | -0.0415 (0.0063) | 4.1E-11 | -0.2039 (0.1493) | 0.172 | 43.4 | 4.913 |
| Appendicular lean mass | Vascular dementia (FinnGen) | rs6738207 | 2:105989716 | A/G | 0.401 | 0.0127 (0.0019) | 4.1E-11 | 0.0819 (0.0522) | 0.117 | 44.7 | 6.449 |
| Appendicular lean mass | Vascular dementia (FinnGen) | rs113671109 | 3:12620885 | C/T | 0.220 | -0.0150 (0.0023) | 4.2E-11 | 0.0553 (0.0569) | 0.331 | 42.5 | -3.687 |
| Appendicular lean mass | Vascular dementia (FinnGen) | rs2965074 | 7:45983129 | T/G | 0.452 | 0.0125 (0.0019) | 4.3E-11 | 0.0056 (0.0505) | 0.911 | 43.3 | 0.448 |
| Appendicular lean mass | Vascular dementia (FinnGen) | rs1800504 | 7:50742180 | T/C | 0.552 | -0.0125 (0.0019) | 4.4E-11 | -0.0004 (0.0496) | 0.994 | 43.3 | 0.032 |
| Appendicular lean mass | Vascular dementia (FinnGen) | rs34203781 | 6:41666292 | C/A | 0.137 | 0.0181 (0.0028) | 5.6E-11 | 0.0762 (0.0668) | 0.254 | 41.8 | 4.210 |
| Appendicular lean mass | Vascular dementia (FinnGen) | rs2347808 | 18:2750856 | A/G | 0.514 | -0.0125 (0.0019) | 5.8E-11 | -0.0020 (0.0491) | 0.967 | 43.3 | 0.160 |
| Appendicular lean mass | Vascular dementia (FinnGen) | rs3830008 | 11:117093764 | C/A | 0.204 | -0.0154 (0.0023) | 6.1E-11 | -0.0406 (0.0561) | 0.469 | 44.8 | 2.636 |
| Appendicular lean mass | Vascular dementia (FinnGen) | rs2490302 | 10:37702435 | A/T | 0.914 | 0.0221 (0.0034) | 6.2E-11 | -0.1106 (0.1010) | 0.274 | 42.2 | -5.005 |
| Appendicular lean mass | Vascular dementia (FinnGen) | rs757042 | 7:25662829 | C/T | 0.724 | -0.0140 (0.0021) | 6.3E-11 | 0.0495 (0.0520) | 0.341 | 44.4 | -3.536 |
| Appendicular lean mass | Vascular dementia (FinnGen) | rs8180765 | 7:4680614 | G/A | 0.217 | -0.0151 (0.0023) | 6.5E-11 | 0.0729 (0.0555) | 0.189 | 43.1 | -4.828 |
| Appendicular lean mass | Vascular dementia (FinnGen) | rs11605297 | 11:58296806 | A/G | 0.233 | 0.0146 (0.0022) | 8.0E-11 | 0.0422 (0.0564) | 0.454 | 44.0 | 2.890 |
| Appendicular lean mass | Vascular dementia (FinnGen) | rs496783 | 10:116137961 | G/A | 0.465 | -0.0124 (0.0019) | 8.1E-11 | 0.0475 (0.0516) | 0.357 | 42.6 | -3.831 |
| Appendicular lean mass | Vascular dementia (FinnGen) | rs603486 | 11:118587347 | C/T | 0.617 | 0.0127 (0.0020) | 8.8E-11 | 0.0677 (0.0514) | 0.187 | 40.3 | 5.331 |
| Appendicular lean mass | Vascular dementia (FinnGen) | rs1899040 | 2:223901896 | T/C | 0.796 | 0.0152 (0.0023) | 9.0E-11 | 0.0934 (0.0605) | 0.122 | 43.7 | 6.145 |
| Appendicular lean mass | Vascular dementia (FinnGen) | rs35874463 | 15:67457698 | G/A | 0.058 | 0.0263 (0.0041) | 9.1E-11 | 0.2087 (0.1291) | 0.106 | 41.1 | 7.935 |
| Appendicular lean mass | Vascular dementia (FinnGen) | rs13109280 | 4:54380513 | G/A | 0.662 | 0.0131 (0.0020) | 9.1E-11 | 0.0972 (0.0518) | 0.061 | 42.9 | 7.420 |
| Appendicular lean mass | Vascular dementia (FinnGen) | rs13127468 | 4:8599658 | A/C | 0.463 | -0.0123 (0.0019) | 9.9E-11 | -0.0489 (0.0501) | 0.329 | 41.9 | 3.976 |
| Appendicular lean mass | Vascular dementia (FinnGen) | rs8064946 | 17:7589311 | C/G | 0.110 | -0.0195 (0.0030) | 1.0E-10 | 0.0308 (0.0970) | 0.751 | 42.2 | -1.579 |
| Appendicular lean mass | Vascular dementia (FinnGen) | rs10815304 | 9:5908217 | C/T | 0.206 | 0.0151 (0.0023) | 1.1E-10 | -0.1136 (0.0644) | 0.078 | 43.1 | -7.523 |
| Appendicular lean mass | Vascular dementia (FinnGen) | rs56112295 | 14:105877057 | T/C | 0.226 | 0.0154 (0.0024) | 1.1E-10 | 0.0523 (0.0625) | 0.404 | 41.2 | 3.396 |
| Appendicular lean mass | Vascular dementia (FinnGen) | rs11221657 | 11:129181358 | G/T | 0.135 | 0.0179 (0.0028) | 1.2E-10 | 0.0793 (0.0679) | 0.243 | 40.9 | 4.430 |
| Appendicular lean mass | Vascular dementia (FinnGen) | rs10019221 | 4:21785364 | T/G | 0.597 | -0.0124 (0.0019) | 1.2E-10 | 0.0163 (0.0510) | 0.749 | 42.6 | -1.315 |
| Appendicular lean mass | Vascular dementia (FinnGen) | rs55717234 | 1:150999863 | G/A | 0.573 | 0.0122 (0.0019) | 1.3E-10 | 0.0039 (0.0506) | 0.939 | 41.2 | 0.320 |
| Appendicular lean mass | Vascular dementia (FinnGen) | rs1405227 | 1:98873390 | A/G | 0.317 | 0.0129 (0.0020) | 1.6E-10 | -0.0874 (0.0508) | 0.085 | 41.6 | -6.775 |
| Appendicular lean mass | Vascular dementia (FinnGen) | rs200302461 | 2:9770589 | GCGGGGC/G | 0.259 | 0.0145 (0.0023) | 1.6E-10 | 0.0304 (0.0575) | 0.597 | 39.7 | 2.097 |
| Appendicular lean mass | Vascular dementia (FinnGen) | rs10116740 | 9:133446357 | A/G | 0.477 | -0.0121 (0.0019) | 1.8E-10 | -0.0085 (0.0495) | 0.864 | 40.6 | 0.702 |
| Appendicular lean mass | Vascular dementia (FinnGen) | rs111622870 | 4:2613109 | C/T | 0.048 | -0.0282 (0.0044) | 1.9E-10 | 0.1571 (0.1632) | 0.336 | 41.1 | -5.571 |
| Appendicular lean mass | Vascular dementia (FinnGen) | rs650508 | 2:45880122 | C/G | 0.301 | -0.0130 (0.0020) | 1.9E-10 | 0.0014 (0.0593) | 0.981 | 42.2 | -0.108 |
| Appendicular lean mass | Vascular dementia (FinnGen) | rs6054491 | 20:6709535 | G/C | 0.240 | -0.0142 (0.0022) | 2.2E-10 | 0.0181 (0.0559) | 0.747 | 41.7 | -1.275 |
| Appendicular lean mass | Vascular dementia (FinnGen) | rs79680939 | 17:80523375 | A/G | 0.045 | 0.0290 (0.0046) | 2.5E-10 | 0.0359 (0.1613) | 0.824 | 39.7 | 1.238 |
| Appendicular lean mass | Vascular dementia (FinnGen) | rs56207600 | 11:126196537 | A/G | 0.111 | 0.0192 (0.0030) | 2.5E-10 | -0.0119 (0.0724) | 0.870 | 41.0 | -0.620 |
| Appendicular lean mass | Vascular dementia (FinnGen) | rs9828525 | 3:61552810 | T/C | 0.411 | 0.0121 (0.0019) | 2.5E-10 | -0.0362 (0.0502) | 0.470 | 40.6 | -2.992 |
| Appendicular lean mass | Vascular dementia (FinnGen) | rs4682483 | 3:112993982 | A/G | 0.153 | -0.0165 (0.0026) | 2.7E-10 | 0.0544 (0.0558) | 0.330 | 40.3 | -3.297 |
| Appendicular lean mass | Vascular dementia (FinnGen) | rs113146332 | 17:42565977 | C/G | 0.038 | 0.0311 (0.0049) | 3.0E-10 | -0.0609 (0.2216) | 0.783 | 40.3 | -1.958 |
| Appendicular lean mass | Vascular dementia (FinnGen) | rs7030071 | 9:136951556 | G/A | 0.311 | -0.0130 (0.0021) | 3.1E-10 | -0.0836 (0.0517) | 0.105 | 38.3 | 6.431 |
| Appendicular lean mass | Vascular dementia (FinnGen) | rs11410717 | 7:116252956 | TG/T | 0.117 | 0.0185 (0.0029) | 3.1E-10 | -0.0756 (0.0694) | 0.276 | 40.7 | -4.086 |
| Appendicular lean mass | Vascular dementia (FinnGen) | rs34995241 | 6:83631850 | AT/A | 0.708 | -0.0131 (0.0021) | 3.2E-10 | -0.0050 (0.0557) | 0.929 | 38.9 | 0.382 |
| Appendicular lean mass | Vascular dementia (FinnGen) | rs77299262 | 3:187450067 | C/T | 0.067 | 0.0237 (0.0038) | 3.4E-10 | -0.0152 (0.0895) | 0.865 | 38.9 | -0.641 |
| Appendicular lean mass | Vascular dementia (FinnGen) | rs1407204 | 6:119289835 | A/C | 0.810 | -0.0151 (0.0024) | 3.5E-10 | -0.0644 (0.0732) | 0.379 | 39.6 | 4.265 |
| Appendicular lean mass | Vascular dementia (FinnGen) | rs11191208 | 10:103838497 | A/G | 0.206 | 0.0147 (0.0024) | 3.7E-10 | -0.0007 (0.0610) | 0.990 | 37.5 | -0.048 |
| Appendicular lean mass | Vascular dementia (FinnGen) | rs11684531 | 2:219835489 | G/A | 0.133 | -0.0172 (0.0028) | 4.2E-10 | 0.0016 (0.0969) | 0.987 | 37.7 | -0.093 |
| Appendicular lean mass | Vascular dementia (FinnGen) | rs2823990 | 21:18081151 | A/G | 0.677 | -0.0127 (0.0020) | 4.2E-10 | 0.0881 (0.0508) | 0.083 | 40.3 | -6.937 |
| Appendicular lean mass | Vascular dementia (FinnGen) | rs3792819 | 5:172576296 | G/A | 0.085 | 0.0210 (0.0034) | 4.4E-10 | 0.0676 (0.1362) | 0.620 | 38.1 | 3.219 |
| Appendicular lean mass | Vascular dementia (FinnGen) | rs10762866 | 10:81228430 | A/G | 0.138 | 0.0171 (0.0028) | 5.4E-10 | 0.0735 (0.0636) | 0.248 | 37.3 | 4.298 |
| Appendicular lean mass | Vascular dementia (FinnGen) | rs7359097 | 14:54757497 | C/T | 0.396 | -0.0121 (0.0019) | 5.5E-10 | -0.0229 (0.0498) | 0.646 | 40.6 | 1.893 |
| Appendicular lean mass | Vascular dementia (FinnGen) | rs10112506 | 8:13164746 | G/A | 0.390 | -0.0120 (0.0019) | 5.8E-10 | 0.0210 (0.0532) | 0.694 | 39.9 | -1.750 |
| Appendicular lean mass | Vascular dementia (FinnGen) | rs2974337 | 8:42390873 | T/C | 0.500 | 0.0117 (0.0019) | 5.9E-10 | 0.0784 (0.0495) | 0.113 | 37.9 | 6.701 |
| Appendicular lean mass | Vascular dementia (FinnGen) | rs11421589 | 10:115193949 | CT/C | 0.556 | 0.0119 (0.0019) | 6.0E-10 | 0.0495 (0.0494) | 0.317 | 39.2 | 4.160 |
| Appendicular lean mass | Vascular dementia (FinnGen) | rs7144307 | 14:69533837 | C/T | 0.385 | -0.0122 (0.0020) | 6.1E-10 | 0.0405 (0.0500) | 0.418 | 37.2 | -3.320 |
| Appendicular lean mass | Vascular dementia (FinnGen) | rs4754296 | 11:108006182 | G/T | 0.146 | -0.0167 (0.0027) | 6.1E-10 | 0.0190 (0.0592) | 0.748 | 38.3 | -1.138 |
| Appendicular lean mass | Vascular dementia (FinnGen) | rs139921635 | 4:73181637 | T/G | 0.024 | 0.0385 (0.0062) | 6.2E-10 | -0.0020 (0.1451) | 0.989 | 38.6 | -0.052 |
| Appendicular lean mass | Vascular dementia (FinnGen) | rs10241451 | 7:55248926 | C/T | 0.209 | 0.0145 (0.0023) | 6.3E-10 | -0.0163 (0.0623) | 0.794 | 39.7 | -1.124 |
| Appendicular lean mass | Vascular dementia (FinnGen) | rs447352 | 5:678750 | T/C | 0.141 | -0.0181 (0.0029) | 6.6E-10 | 0.0170 (0.0815) | 0.834 | 39.0 | -0.939 |
| Appendicular lean mass | Vascular dementia (FinnGen) | rs718603 | 6:2644245 | T/C | 0.277 | 0.0131 (0.0021) | 6.7E-10 | -0.0686 (0.0549) | 0.211 | 38.9 | -5.237 |
| Appendicular lean mass | Vascular dementia (FinnGen) | rs2875409 | 13:41112849 | A/G | 0.050 | -0.0270 (0.0044) | 7.1E-10 | 0.1403 (0.1290) | 0.277 | 37.6 | -5.196 |
| Appendicular lean mass | Vascular dementia (FinnGen) | rs839255 | 3:57974580 | G/T | 0.685 | -0.0126 (0.0021) | 8.4E-10 | 0.0415 (0.0530) | 0.434 | 36.0 | -3.294 |
| Appendicular lean mass | Vascular dementia (FinnGen) | rs10849576 | 12:993652 | G/T | 0.410 | -0.0117 (0.0019) | 9.2E-10 | -0.0434 (0.0528) | 0.411 | 37.9 | 3.709 |
| Appendicular lean mass | Vascular dementia (FinnGen) | rs11688284 | 2:174738951 | T/C | 0.388 | -0.0119 (0.0019) | 9.8E-10 | 0.0316 (0.0515) | 0.539 | 39.2 | -2.655 |
| Appendicular lean mass | Vascular dementia (FinnGen) | rs116339650 | 4:26200972 | G/A | 0.127 | -0.0175 (0.0029) | 1.1E-09 | -0.0463 (0.0666) | 0.487 | 36.4 | 2.646 |
| Appendicular lean mass | Vascular dementia (FinnGen) | rs670129 | 20:10460858 | T/C | 0.413 | -0.0118 (0.0019) | 1.1E-09 | -0.0066 (0.0521) | 0.899 | 38.6 | 0.559 |
| Appendicular lean mass | Vascular dementia (FinnGen) | rs7328187 | 13:74189974 | G/T | 0.498 | 0.0116 (0.0019) | 1.2E-09 | -0.0253 (0.0502) | 0.614 | 37.3 | -2.181 |
| Appendicular lean mass | Vascular dementia (FinnGen) | rs62143873 | 2:72035050 | A/G | 0.503 | -0.0115 (0.0019) | 1.2E-09 | -0.0843 (0.0500) | 0.092 | 36.6 | 7.330 |
| Appendicular lean mass | Vascular dementia (FinnGen) | rs6469845 | 8:120792195 | T/C | 0.239 | 0.0135 (0.0022) | 1.2E-09 | -0.0047 (0.0542) | 0.931 | 37.6 | -0.348 |
| Appendicular lean mass | Vascular dementia (FinnGen) | rs4940874 | 18:57105638 | G/A | 0.812 | 0.0148 (0.0024) | 1.2E-09 | 0.0116 (0.0586) | 0.843 | 38.0 | 0.784 |
| Appendicular lean mass | Vascular dementia (FinnGen) | rs75172776 | 16:9001814 | G/A | 0.066 | 0.0232 (0.0038) | 1.3E-09 | 0.0409 (0.1200) | 0.733 | 37.3 | 1.763 |
| Appendicular lean mass | Vascular dementia (FinnGen) | rs61925210 | 12:43974800 | T/A | 0.105 | 0.0187 (0.0031) | 1.3E-09 | -0.0871 (0.0764) | 0.254 | 36.4 | -4.658 |
| Appendicular lean mass | Vascular dementia (FinnGen) | rs4932439 | 15:89401109 | G/A | 0.825 | -0.0151 (0.0025) | 1.4E-09 | 0.0124 (0.0544) | 0.820 | 36.5 | -0.821 |
| Appendicular lean mass | Vascular dementia (FinnGen) | rs249681 | 5:141538017 | C/G | 0.618 | -0.0117 (0.0019) | 1.5E-09 | 0.0479 (0.0514) | 0.351 | 37.9 | -4.094 |
| Appendicular lean mass | Vascular dementia (FinnGen) | rs616488 | 1:10566215 | G/A | 0.345 | 0.0120 (0.0020) | 1.5E-09 | 0.0143 (0.0520) | 0.783 | 36.0 | 1.192 |
| Appendicular lean mass | Vascular dementia (FinnGen) | rs17773965 | 2:217631338 | T/C | 0.141 | -0.0163 (0.0027) | 1.5E-09 | 0.0670 (0.0577) | 0.246 | 36.5 | -4.110 |
| Appendicular lean mass | Vascular dementia (FinnGen) | rs4554207 | 5:114388634 | C/T | 0.515 | -0.0114 (0.0019) | 1.6E-09 | 0.0861 (0.0490) | 0.079 | 36.0 | -7.553 |
| Appendicular lean mass | Vascular dementia (FinnGen) | rs1190540 | 14:102897009 | G/A | 0.698 | 0.0125 (0.0021) | 1.6E-09 | 0.0004 (0.0523) | 0.994 | 35.4 | 0.032 |
| Appendicular lean mass | Vascular dementia (FinnGen) | rs1202186 | 7:87213258 | T/C | 0.655 | -0.0120 (0.0020) | 1.7E-09 | -0.0890 (0.0533) | 0.095 | 36.0 | 7.417 |
| Appendicular lean mass | Vascular dementia (FinnGen) | rs62033029 | 16:50107273 | A/G | 0.206 | -0.0141 (0.0023) | 1.7E-09 | 0.0155 (0.0637) | 0.807 | 37.6 | -1.099 |
| Appendicular lean mass | Vascular dementia (FinnGen) | rs13112742 | 4:124888059 | A/G | 0.170 | -0.0151 (0.0025) | 2.0E-09 | -0.1222 (0.0718) | 0.089 | 36.5 | 8.093 |
| Appendicular lean mass | Vascular dementia (FinnGen) | rs680882 | 7:18325278 | G/T | 0.761 | 0.0133 (0.0022) | 2.0E-09 | 0.0164 (0.0565) | 0.771 | 36.5 | 1.233 |
| Appendicular lean mass | Vascular dementia (FinnGen) | rs8017006 | 14:42745052 | G/A | 0.328 | 0.0122 (0.0020) | 2.2E-09 | -0.0623 (0.0555) | 0.261 | 37.2 | -5.107 |
| Appendicular lean mass | Vascular dementia (FinnGen) | rs2569888 | 6:1625803 | A/G | 0.245 | 0.0133 (0.0022) | 2.3E-09 | 0.0149 (0.0570) | 0.793 | 36.5 | 1.120 |
| Appendicular lean mass | Vascular dementia (FinnGen) | rs10779958 | 2:74755778 | C/A | 0.149 | 0.0157 (0.0026) | 2.6E-09 | -0.0122 (0.0707) | 0.863 | 36.5 | -0.777 |
| Appendicular lean mass | Vascular dementia (FinnGen) | rs66613683 | 12:104409025 | C/T | 0.170 | 0.0150 (0.0025) | 2.6E-09 | -0.0102 (0.0702) | 0.884 | 36.0 | -0.680 |
| Appendicular lean mass | Vascular dementia (FinnGen) | rs114018835 | 1:244274455 | T/C | 0.036 | 0.0303 (0.0051) | 2.7E-09 | -0.0481 (0.2087) | 0.818 | 35.3 | -1.587 |
| Appendicular lean mass | Vascular dementia (FinnGen) | rs150474185 | 1:65689032 | G/C | 0.021 | 0.0440 (0.0074) | 2.7E-09 | -0.0789 (0.3030) | 0.794 | 35.4 | -1.793 |
| Appendicular lean mass | Vascular dementia (FinnGen) | rs114192718 | 2:128785663 | T/C | 0.075 | -0.0215 (0.0036) | 2.9E-09 | 0.0790 (0.1150) | 0.492 | 35.7 | -3.674 |
| Appendicular lean mass | Vascular dementia (FinnGen) | rs17713523 | 5:149923968 | A/G | 0.407 | 0.0114 (0.0019) | 3.1E-09 | -0.0250 (0.0492) | 0.611 | 36.0 | -2.193 |
| Appendicular lean mass | Vascular dementia (FinnGen) | rs11175919 | 12:66180277 | A/G | 0.026 | 0.0349 (0.0059) | 3.2E-09 | 0.1877 (0.1523) | 0.218 | 35.0 | 5.378 |
| Appendicular lean mass | Vascular dementia (FinnGen) | rs3118124 | 1:150166675 | G/A | 0.289 | -0.0122 (0.0021) | 3.2E-09 | -0.1140 (0.0565) | 0.044 | 33.8 | 9.344 |
| Appendicular lean mass | Vascular dementia (FinnGen) | rs78838233 | 17:18257589 | T/C | 0.237 | -0.0132 (0.0022) | 3.3E-09 | -0.0759 (0.0559) | 0.175 | 36.0 | 5.750 |
| Appendicular lean mass | Vascular dementia (FinnGen) | rs3769885 | 2:165592390 | A/G | 0.490 | -0.0111 (0.0019) | 3.5E-09 | 0.0048 (0.0495) | 0.923 | 34.1 | -0.432 |
| Appendicular lean mass | Vascular dementia (FinnGen) | rs3003137 | 20:62830802 | T/G | 0.428 | -0.0114 (0.0019) | 3.6E-09 | -0.0607 (0.0515) | 0.238 | 36.0 | 5.325 |
| Appendicular lean mass | Vascular dementia (FinnGen) | rs13316 | 14:93407301 | A/C | 0.430 | 0.0115 (0.0019) | 3.7E-09 | 0.0801 (0.0497) | 0.107 | 36.6 | 6.965 |
| Appendicular lean mass | Vascular dementia (FinnGen) | rs2788213 | 6:703249 | A/G | 0.710 | 0.0123 (0.0021) | 3.8E-09 | -0.0033 (0.0549) | 0.953 | 34.3 | -0.268 |
| Appendicular lean mass | Vascular dementia (FinnGen) | rs7095087 | 10:31120513 | A/G | 0.353 | 0.0117 (0.0020) | 3.8E-09 | 0.0450 (0.0552) | 0.415 | 34.2 | 3.846 |
| Appendicular lean mass | Vascular dementia (FinnGen) | rs603542 | 1:44079411 | C/T | 0.675 | 0.0118 (0.0020) | 3.8E-09 | -0.0375 (0.0522) | 0.473 | 34.8 | -3.178 |
| Appendicular lean mass | Vascular dementia (FinnGen) | rs290793 | 7:130416726 | C/G | 0.188 | 0.0142 (0.0024) | 4.0E-09 | 0.0196 (0.0596) | 0.743 | 35.0 | 1.380 |
| Appendicular lean mass | Vascular dementia (FinnGen) | rs41298373 | 10:7622009 | A/G | 0.101 | 0.0185 (0.0031) | 4.2E-09 | -0.0111 (0.0728) | 0.879 | 35.6 | -0.600 |
| Appendicular lean mass | Vascular dementia (FinnGen) | rs4836544 | 9:129236355 | T/C | 0.885 | 0.0175 (0.0030) | 4.3E-09 | 0.1066 (0.0937) | 0.255 | 34.0 | 6.091 |
| Appendicular lean mass | Vascular dementia (FinnGen) | rs4121583 | 18:125075 | T/C | 0.619 | 0.0118 (0.0020) | 4.6E-09 | 0.0295 (0.0516) | 0.567 | 34.8 | 2.500 |
| Appendicular lean mass | Vascular dementia (FinnGen) | rs113827862 | 14:89849527 | C/T | 0.061 | -0.0235 (0.0040) | 4.7E-09 | 0.0748 (0.0926) | 0.419 | 34.5 | -3.183 |
| Appendicular lean mass | Vascular dementia (FinnGen) | rs2150692 | 13:30109494 | A/C | 0.855 | -0.0158 (0.0027) | 4.8E-09 | 0.0038 (0.0590) | 0.948 | 34.2 | -0.241 |
| Appendicular lean mass | Vascular dementia (FinnGen) | rs9636364 | 2:111992435 | A/G | 0.543 | 0.0110 (0.0019) | 5.1E-09 | 0.1447 (0.0494) | 0.003 | 33.5 | 13.155 |
| Appendicular lean mass | Vascular dementia (FinnGen) | rs577289 | 15:40208911 | T/A | 0.281 | -0.0125 (0.0021) | 5.4E-09 | 0.0323 (0.0535) | 0.545 | 35.4 | -2.584 |
| Appendicular lean mass | Vascular dementia (FinnGen) | rs117972846 | 17:26947476 | G/T | 0.029 | 0.0335 (0.0057) | 5.5E-09 | -0.2537 (0.1511) | 0.093 | 34.5 | -7.573 |
| Appendicular lean mass | Vascular dementia (FinnGen) | rs4652902 | 1:36612955 | A/C | 0.768 | 0.0129 (0.0022) | 5.7E-09 | -0.0638 (0.0548) | 0.244 | 34.4 | -4.946 |
| Appendicular lean mass | Vascular dementia (FinnGen) | rs3103223 | 4:42402721 | C/T | 0.740 | 0.0126 (0.0022) | 6.0E-09 | 0.0884 (0.0576) | 0.125 | 32.8 | 7.016 |
| Appendicular lean mass | Vascular dementia (FinnGen) | rs10060855 | 5:33216769 | G/A | 0.677 | 0.0118 (0.0020) | 6.2E-09 | 0.0751 (0.0511) | 0.142 | 34.8 | 6.364 |
| Appendicular lean mass | Vascular dementia (FinnGen) | rs1527149 | 7:114004088 | G/C | 0.407 | 0.0113 (0.0019) | 6.9E-09 | 0.1201 (0.0507) | 0.018 | 35.4 | 10.628 |
| Appendicular lean mass | Vascular dementia (FinnGen) | rs35701316 | 20:39805449 | GTGTT/G | 0.174 | -0.0145 (0.0025) | 7.0E-09 | -0.0013 (0.0564) | 0.982 | 33.6 | 0.090 |
| Appendicular lean mass | Vascular dementia (FinnGen) | rs11132166 | 4:184049867 | C/T | 0.036 | -0.0292 (0.0050) | 7.6E-09 | -0.1492 (0.1714) | 0.384 | 34.1 | 5.110 |
| Appendicular lean mass | Vascular dementia (FinnGen) | rs34338597 | 12:106301580 | G/A | 0.383 | -0.0112 (0.0019) | 7.9E-09 | 0.0400 (0.0498) | 0.422 | 34.8 | -3.571 |
| Appendicular lean mass | Vascular dementia (FinnGen) | rs4849904 | 2:121336556 | G/C | 0.380 | 0.0111 (0.0019) | 8.1E-09 | 0.0744 (0.0552) | 0.178 | 34.1 | 6.703 |
| Appendicular lean mass | Vascular dementia (FinnGen) | rs612577 | 1:230324364 | C/T | 0.149 | -0.0152 (0.0026) | 8.2E-09 | -0.0133 (0.0633) | 0.833 | 34.2 | 0.875 |
| Appendicular lean mass | Vascular dementia (FinnGen) | rs111925803 | 22:50719976 | T/C | 0.285 | 0.0122 (0.0021) | 8.6E-09 | -0.0253 (0.0563) | 0.653 | 33.8 | -2.074 |
| Appendicular lean mass | Vascular dementia (FinnGen) | rs921142 | 8:41169276 | C/T | 0.398 | -0.0111 (0.0019) | 8.7E-09 | -0.0380 (0.0494) | 0.442 | 34.1 | 3.423 |
| Appendicular lean mass | Vascular dementia (FinnGen) | rs10099846 | 8:22534178 | T/C | 0.668 | 0.0116 (0.0020) | 8.8E-09 | -0.0062 (0.0546) | 0.910 | 33.6 | -0.534 |
| Appendicular lean mass | Vascular dementia (FinnGen) | rs7744700 | 6:53349401 | A/T | 0.291 | -0.0122 (0.0021) | 9.2E-09 | 0.0310 (0.0537) | 0.564 | 33.8 | -2.541 |
| Appendicular lean mass | Vascular dementia (FinnGen) | rs12483401 | 21:35443829 | C/T | 0.022 | -0.0387 (0.0067) | 9.2E-09 | -0.2272 (0.1916) | 0.236 | 33.4 | 5.871 |
| Appendicular lean mass | Vascular dementia (FinnGen) | rs68185109 | 10:93648306 | A/G | 0.102 | 0.0180 (0.0031) | 9.6E-09 | 0.1357 (0.0847) | 0.109 | 33.7 | 7.539 |
| Appendicular lean mass | Vascular dementia (FinnGen) | rs28736838 | 1:120148713 | T/C | 0.302 | -0.0117 (0.0020) | 1.1E-08 | -0.0233 (0.0527) | 0.658 | 34.2 | 1.991 |
| Appendicular lean mass | Vascular dementia (FinnGen) | rs2208404 | 20:55843736 | G/A | 0.416 | -0.0111 (0.0019) | 1.1E-08 | -0.0075 (0.0504) | 0.882 | 34.1 | 0.676 |
| Appendicular lean mass | Vascular dementia (FinnGen) | rs8018486 | 14:39818616 | G/A | 0.191 | -0.0138 (0.0024) | 1.2E-08 | 0.0256 (0.0595) | 0.667 | 33.1 | -1.855 |
| Appendicular lean mass | Vascular dementia (FinnGen) | rs10749157 | 10:115780129 | C/T | 0.358 | 0.0113 (0.0020) | 1.2E-08 | -0.0325 (0.0494) | 0.510 | 31.9 | -2.876 |
| Appendicular lean mass | Vascular dementia (FinnGen) | rs11070842 | 15:51624185 | C/T | 0.163 | -0.0146 (0.0026) | 1.3E-08 | 0.0518 (0.0831) | 0.533 | 31.5 | -3.548 |
| Appendicular lean mass | Vascular dementia (FinnGen) | rs62103240 | 18:77650637 | A/G | 0.071 | 0.0212 (0.0037) | 1.4E-08 | -0.0799 (0.1144) | 0.485 | 32.8 | -3.769 |
| Appendicular lean mass | Vascular dementia (FinnGen) | rs10222594 | 3:71554856 | A/G | 0.323 | 0.0115 (0.0020) | 1.4E-08 | 0.1011 (0.0544) | 0.063 | 33.1 | 8.791 |
| Appendicular lean mass | Vascular dementia (FinnGen) | rs56309431 | 17:63645289 | T/G | 0.116 | 0.0167 (0.0029) | 1.5E-08 | 0.0541 (0.0791) | 0.494 | 33.2 | 3.240 |
| Appendicular lean mass | Vascular dementia (FinnGen) | rs10764692 | 10:27576251 | C/A | 0.637 | -0.0111 (0.0020) | 1.6E-08 | -0.0213 (0.0534) | 0.690 | 30.8 | 1.919 |
| Appendicular lean mass | Vascular dementia (FinnGen) | rs10940169 | 5:67823682 | C/G | 0.382 | -0.0110 (0.0019) | 1.6E-08 | -0.0115 (0.0524) | 0.826 | 33.5 | 1.045 |
| Appendicular lean mass | Vascular dementia (FinnGen) | rs10221831 | 2:202107829 | T/C | 0.032 | 0.0300 (0.0053) | 1.8E-08 | 0.0942 (0.1425) | 0.509 | 32.0 | 3.140 |
| Appendicular lean mass | Vascular dementia (FinnGen) | rs4965298 | 15:100802766 | T/C | 0.713 | -0.0119 (0.0021) | 1.8E-08 | 0.0370 (0.0589) | 0.530 | 32.1 | -3.109 |
| Appendicular lean mass | Vascular dementia (FinnGen) | rs28678024 | 14:25937161 | G/A | 0.285 | -0.0119 (0.0021) | 1.9E-08 | 0.0369 (0.0523) | 0.480 | 32.1 | -3.101 |
| Appendicular lean mass | Vascular dementia (FinnGen) | rs2188805 | 7:93078400 | C/A | 0.336 | 0.0114 (0.0020) | 2.0E-08 | -0.0170 (0.0567) | 0.764 | 32.5 | -1.491 |
| Appendicular lean mass | Vascular dementia (FinnGen) | rs9590328 | 13:96448383 | G/A | 0.142 | 0.0153 (0.0027) | 2.0E-08 | -0.1743 (0.0722) | 0.016 | 32.1 | -11.392 |
| Appendicular lean mass | Vascular dementia (FinnGen) | rs31196 | 5:158300798 | A/C | 0.572 | -0.0107 (0.0019) | 2.1E-08 | -0.0236 (0.0494) | 0.633 | 31.7 | 2.206 |
| Appendicular lean mass | Vascular dementia (FinnGen) | rs876122 | 6:6886297 | G/A | 0.879 | 0.0162 (0.0029) | 2.2E-08 | 0.0380 (0.0674) | 0.572 | 31.2 | 2.346 |
| Appendicular lean mass | Vascular dementia (FinnGen) | rs9288695 | 2:158641300 | C/G | 0.779 | 0.0127 (0.0023) | 2.2E-08 | -0.0184 (0.0596) | 0.757 | 30.5 | -1.449 |
| Appendicular lean mass | Vascular dementia (FinnGen) | rs7082659 | 10:12017584 | C/T | 0.866 | 0.0156 (0.0028) | 2.3E-08 | -0.0705 (0.0766) | 0.357 | 31.0 | -4.519 |
| Appendicular lean mass | Vascular dementia (FinnGen) | rs80280630 | 9:117030861 | T/C | 0.112 | -0.0168 (0.0030) | 2.3E-08 | 0.0486 (0.0918) | 0.596 | 31.4 | -2.893 |
| Appendicular lean mass | Vascular dementia (FinnGen) | rs74048171 | 11:2093603 | A/C | 0.258 | -0.0121 (0.0022) | 2.4E-08 | -0.0607 (0.0593) | 0.306 | 30.2 | 5.017 |
| Appendicular lean mass | Vascular dementia (FinnGen) | rs141277904 | 8:56654581 | T/C | 0.023 | 0.0384 (0.0069) | 2.4E-08 | 0.2405 (0.1994) | 0.228 | 31.0 | 6.263 |
| Appendicular lean mass | Vascular dementia (FinnGen) | rs4916786 | 5:89476946 | T/C | 0.196 | 0.0133 (0.0024) | 2.4E-08 | -0.0532 (0.0594) | 0.370 | 30.7 | -4.000 |
| Appendicular lean mass | Vascular dementia (FinnGen) | rs60142646 | 2:46532856 | TC/T | 0.948 | -0.0237 (0.0042) | 2.5E-08 | 0.0873 (0.1238) | 0.481 | 31.8 | -3.684 |
| Appendicular lean mass | Vascular dementia (FinnGen) | rs2303423 | 4:38120029 | C/T | 0.110 | 0.0168 (0.0030) | 2.6E-08 | 0.1010 (0.0938) | 0.282 | 31.4 | 6.012 |
| Appendicular lean mass | Vascular dementia (FinnGen) | rs9265546 | 6:31298202 | A/G | 0.223 | 0.0147 (0.0026) | 2.8E-08 | 0.0194 (0.0505) | 0.701 | 32.0 | 1.320 |
| Appendicular lean mass | Vascular dementia (FinnGen) | rs149697773 | 17:60932424 | G/A | 0.045 | -0.0262 (0.0047) | 2.8E-08 | 0.0525 (0.1157) | 0.650 | 31.1 | -2.004 |
| Appendicular lean mass | Vascular dementia (FinnGen) | rs2978362 | 18:32959397 | T/C | 0.533 | 0.0106 (0.0019) | 2.8E-08 | -0.0369 (0.0501) | 0.462 | 31.1 | -3.481 |
| Appendicular lean mass | Vascular dementia (FinnGen) | rs4383083 | 15:63080442 | A/G | 0.647 | 0.0111 (0.0020) | 2.9E-08 | -0.0456 (0.0562) | 0.417 | 30.8 | -4.108 |
| Appendicular lean mass | Vascular dementia (FinnGen) | rs10940364 | 5:53487971 | C/T | 0.139 | 0.0151 (0.0027) | 3.1E-08 | 0.0534 (0.0775) | 0.490 | 31.3 | 3.536 |
| Appendicular lean mass | Vascular dementia (FinnGen) | rs77293269 | 3:155595709 | C/T | 0.113 | -0.0165 (0.0030) | 3.1E-08 | 0.0400 (0.0782) | 0.609 | 30.2 | -2.424 |
| Appendicular lean mass | Vascular dementia (FinnGen) | rs62177315 | 2:161236992 | T/C | 0.096 | 0.0177 (0.0032) | 3.1E-08 | 0.0742 (0.1009) | 0.463 | 30.6 | 4.192 |
| Appendicular lean mass | Vascular dementia (FinnGen) | rs72809820 | 2:97360079 | T/C | 0.324 | -0.0111 (0.0020) | 3.1E-08 | -0.0714 (0.0495) | 0.149 | 30.8 | 6.432 |
| Appendicular lean mass | Vascular dementia (FinnGen) | rs9568031 | 13:48897520 | T/C | 0.706 | -0.0115 (0.0021) | 3.3E-08 | 0.0806 (0.0527) | 0.126 | 30.0 | -7.009 |
| Appendicular lean mass | Vascular dementia (FinnGen) | rs10821713 | 10:62055781 | T/C | 0.418 | 0.0106 (0.0019) | 3.4E-08 | 0.0304 (0.0508) | 0.550 | 31.1 | 2.868 |
| Appendicular lean mass | Vascular dementia (FinnGen) | rs12185775 | 20:20293769 | C/G | 0.117 | -0.0167 (0.0030) | 3.7E-08 | 0.0238 (0.0725) | 0.742 | 31.0 | -1.425 |
| Appendicular lean mass | Vascular dementia (FinnGen) | rs6461948 | 7:26540727 | T/C | 0.673 | 0.0112 (0.0020) | 4.0E-08 | 0.0095 (0.0561) | 0.866 | 31.4 | 0.848 |
| Appendicular lean mass | Vascular dementia (FinnGen) | rs11721522 | 4:156976051 | G/A | 0.412 | 0.0106 (0.0019) | 4.0E-08 | 0.0969 (0.0522) | 0.063 | 31.1 | 9.142 |
| Appendicular lean mass | Vascular dementia (FinnGen) | rs3002284 | 1:213181916 | C/T | 0.320 | -0.0110 (0.0020) | 4.1E-08 | 0.0461 (0.0521) | 0.376 | 30.2 | -4.191 |
| Appendicular lean mass | Vascular dementia (FinnGen) | rs35223841 | 2:144053000 | GAA/G | 0.389 | 0.0106 (0.0019) | 4.2E-08 | 0.1241 (0.0523) | 0.018 | 31.1 | 11.708 |
| Appendicular lean mass | Vascular dementia (FinnGen) | rs34856058 | 3:101365150 | G/A | 0.726 | 0.0116 (0.0021) | 4.4E-08 | -0.0385 (0.0568) | 0.498 | 30.5 | -3.319 |
| Appendicular lean mass | Vascular dementia (FinnGen) | rs1884066 | 14:67452834 | A/G | 0.150 | -0.0145 (0.0027) | 5.0E-08 | -0.0971 (0.0652) | 0.136 | 28.8 | 6.697 |
| Appendicular lean mass | Alzheimer's disease, wide (FinnGen) | rs143384 | 20:34025756 | G/A | 0.404 | 0.0725 (0.0019) | 1.1E-308 | -0.0114 (0.0214) | 0.592 | 1456.0 | -0.157 |
| Appendicular lean mass | Alzheimer's disease, wide (FinnGen) | rs6860245 | 5:127367998 | C/G | 0.248 | 0.0589 (0.0022) | 9.7E-160 | -0.0071 (0.0293) | 0.807 | 716.8 | -0.121 |
| Appendicular lean mass | Alzheimer's disease, wide (FinnGen) | rs71190381 | 13:51120097 | GAGTGA/G | 0.214 | -0.0614 (0.0023) | 9.9E-155 | -0.0363 (0.0244) | 0.136 | 712.7 | 0.591 |
| Appendicular lean mass | Alzheimer's disease, wide (FinnGen) | rs9853018 | 3:141101961 | T/C | 0.444 | 0.0469 (0.0019) | 2.4E-135 | 0.0409 (0.0213) | 0.055 | 609.3 | 0.872 |
| Appendicular lean mass | Alzheimer's disease, wide (FinnGen) | rs1472852 | 4:17910236 | A/C | 0.158 | -0.0638 (0.0026) | 8.2E-135 | 0.0460 (0.0362) | 0.204 | 602.1 | -0.721 |
| Appendicular lean mass | Alzheimer's disease, wide (FinnGen) | rs9388490 | 6:126704795 | T/C | 0.439 | 0.0462 (0.0019) | 1.3E-130 | 0.0160 (0.0213) | 0.452 | 591.3 | 0.346 |
| Appendicular lean mass | Alzheimer's disease, wide (FinnGen) | rs2854152 | 17:61986027 | G/A | 0.678 | 0.0482 (0.0020) | 1.0E-124 | 0.0310 (0.0220) | 0.158 | 580.8 | 0.643 |
| Appendicular lean mass | Alzheimer's disease, wide (FinnGen) | rs71519447 | 8:57157543 | A/G | 0.110 | -0.0716 (0.0030) | 3.9E-124 | 0.1007 (0.0337) | 0.003 | 569.6 | -1.406 |
| Appendicular lean mass | Alzheimer's disease, wide (FinnGen) | rs6977416 | 7:150542711 | A/G | 0.334 | 0.0457 (0.0020) | 1.4E-113 | 0.0129 (0.0221) | 0.562 | 522.1 | 0.282 |
| Appendicular lean mass | Alzheimer's disease, wide (FinnGen) | rs76895963 | 12:4384844 | G/T | 0.021 | 0.1639 (0.0073) | 8.2E-112 | -0.0216 (0.0641) | 0.736 | 504.1 | -0.132 |
| Appendicular lean mass | Alzheimer's disease, wide (FinnGen) | rs42039 | 7:92244422 | T/C | 0.244 | 0.0481 (0.0022) | 3.5E-106 | 0.0196 (0.0251) | 0.434 | 478.0 | 0.407 |
| Appendicular lean mass | Alzheimer's disease, wide (FinnGen) | rs6567160 | 18:57829135 | C/T | 0.233 | 0.0488 (0.0022) | 1.2E-104 | 0.0336 (0.0273) | 0.219 | 492.0 | 0.689 |
| Appendicular lean mass | Alzheimer's disease, wide (FinnGen) | rs9634212 | 12:93993266 | A/C | 0.221 | 0.0471 (0.0023) | 8.6E-95 | 0.0099 (0.0245) | 0.688 | 419.4 | 0.210 |
| Appendicular lean mass | Alzheimer's disease, wide (FinnGen) | rs11187838 | 10:96038686 | A/G | 0.435 | 0.0394 (0.0019) | 1.2E-94 | -0.0023 (0.0224) | 0.919 | 430.0 | -0.058 |
| Appendicular lean mass | Alzheimer's disease, wide (FinnGen) | rs2524139 | 6:31264133 | C/G | 0.700 | -0.0423 (0.0020) | 1.5E-94 | -0.0205 (0.0269) | 0.445 | 447.3 | 0.485 |
| Appendicular lean mass | Alzheimer's disease, wide (FinnGen) | rs28701981 | 9:98217581 | C/T | 0.349 | 0.0395 (0.0020) | 1.5E-87 | 0.0115 (0.0220) | 0.602 | 390.1 | 0.291 |
| Appendicular lean mass | Alzheimer's disease, wide (FinnGen) | rs113459995 | 9:119122551 | GTCTGGGC/G | 0.201 | -0.0460 (0.0024) | 3.8E-84 | -0.0465 (0.0252) | 0.064 | 367.4 | 1.011 |
| Appendicular lean mass | Alzheimer's disease, wide (FinnGen) | rs10483727 | 14:61072875 | C/T | 0.611 | -0.0368 (0.0019) | 6.7E-80 | 0.0368 (0.0234) | 0.116 | 375.1 | -1.000 |
| Appendicular lean mass | Alzheimer's disease, wide (FinnGen) | rs1355603 | 4:145566477 | C/T | 0.831 | 0.0466 (0.0025) | 3.0E-76 | 0.0133 (0.0285) | 0.642 | 347.4 | 0.285 |
| Appendicular lean mass | Alzheimer's disease, wide (FinnGen) | rs10807137 | 6:34183026 | T/C | 0.825 | -0.0455 (0.0025) | 5.7E-75 | 0.0206 (0.0306) | 0.501 | 331.2 | -0.453 |
| Appendicular lean mass | Alzheimer's disease, wide (FinnGen) | rs55877758 | 2:172409030 | A/T | 0.241 | -0.0394 (0.0022) | 2.5E-72 | -0.0197 (0.0273) | 0.470 | 320.7 | 0.500 |
| Appendicular lean mass | Alzheimer's disease, wide (FinnGen) | rs12713004 | 2:23896049 | G/A | 0.725 | 0.0367 (0.0021) | 2.4E-68 | 0.0076 (0.0278) | 0.785 | 305.4 | 0.207 |
| Appendicular lean mass | Alzheimer's disease, wide (FinnGen) | rs798528 | 7:2772431 | C/A | 0.307 | -0.0357 (0.0020) | 5.6E-68 | 0.0231 (0.0219) | 0.292 | 318.6 | -0.647 |
| Appendicular lean mass | Alzheimer's disease, wide (FinnGen) | rs1260326 | 2:27730940 | C/T | 0.605 | 0.0323 (0.0019) | 6.2E-64 | -0.0039 (0.0223) | 0.861 | 289.0 | -0.121 |
| Appendicular lean mass | Alzheimer's disease, wide (FinnGen) | rs9905385 | 17:59498250 | G/A | 0.671 | -0.0339 (0.0020) | 1.9E-63 | -0.0002 (0.0238) | 0.992 | 287.3 | 0.006 |
| Appendicular lean mass | Alzheimer's disease, wide (FinnGen) | rs3213141 | 20:32274380 | A/G | 0.266 | -0.0363 (0.0022) | 2.0E-63 | -0.0130 (0.0244) | 0.595 | 272.2 | 0.358 |
| Appendicular lean mass | Alzheimer's disease, wide (FinnGen) | rs485554 | 3:172137343 | C/G | 0.315 | 0.0342 (0.0020) | 3.0E-63 | -0.0222 (0.0226) | 0.328 | 292.4 | -0.649 |
| Appendicular lean mass | Alzheimer's disease, wide (FinnGen) | rs2871865 | 15:99194896 | G/C | 0.116 | -0.0493 (0.0030) | 3.4E-62 | 0.0749 (0.0441) | 0.089 | 270.1 | -1.519 |
| Appendicular lean mass | Alzheimer's disease, wide (FinnGen) | rs117068593 | 14:93118229 | T/C | 0.190 | 0.0403 (0.0024) | 8.8E-62 | 0.0449 (0.0305) | 0.141 | 282.0 | 1.114 |
| Appendicular lean mass | Alzheimer's disease, wide (FinnGen) | rs2885697 | 1:41544279 | T/G | 0.665 | -0.0323 (0.0020) | 9.2E-60 | 0.0308 (0.0224) | 0.170 | 260.8 | -0.954 |
| Appendicular lean mass | Alzheimer's disease, wide (FinnGen) | rs12099669 | 12:46783653 | A/G | 0.696 | 0.0331 (0.0020) | 1.4E-58 | -0.0533 (0.0224) | 0.017 | 273.9 | -1.610 |
| Appendicular lean mass | Alzheimer's disease, wide (FinnGen) | rs11243202 | 6:7719065 | C/T | 0.486 | 0.0302 (0.0019) | 2.8E-57 | 0.0139 (0.0213) | 0.514 | 252.6 | 0.460 |
| Appendicular lean mass | Alzheimer's disease, wide (FinnGen) | rs4735761 | 8:78097161 | C/A | 0.286 | 0.0331 (0.0021) | 3.7E-56 | -0.0182 (0.0256) | 0.476 | 248.4 | -0.550 |
| Appendicular lean mass | Alzheimer's disease, wide (FinnGen) | rs78378222 | 17:7571752 | G/T | 0.012 | 0.1380 (0.0087) | 4.5E-56 | -0.0500 (0.0833) | 0.548 | 251.6 | -0.362 |
| Appendicular lean mass | Alzheimer's disease, wide (FinnGen) | rs2194411 | 3:185548663 | A/G | 0.128 | 0.0443 (0.0029) | 2.4E-54 | 0.0294 (0.0311) | 0.345 | 233.3 | 0.664 |
| Appendicular lean mass | Alzheimer's disease, wide (FinnGen) | rs994533 | 2:218284278 | C/G | 0.316 | -0.0313 (0.0020) | 5.5E-54 | 0.0249 (0.0215) | 0.247 | 244.9 | -0.796 |
| Appendicular lean mass | Alzheimer's disease, wide (FinnGen) | rs11042717 | 11:10303939 | C/T | 0.490 | -0.0290 (0.0019) | 4.0E-53 | -0.0139 (0.0212) | 0.513 | 233.0 | 0.479 |
| Appendicular lean mass | Alzheimer's disease, wide (FinnGen) | rs7129320 | 11:68388220 | A/G | 0.166 | -0.0389 (0.0025) | 7.3E-53 | 0.0730 (0.0376) | 0.052 | 242.1 | -1.877 |
| Appendicular lean mass | Alzheimer's disease, wide (FinnGen) | rs9894577 | 17:43223292 | A/G | 0.318 | -0.0310 (0.0020) | 1.4E-52 | -0.0128 (0.0225) | 0.569 | 240.2 | 0.413 |
| Appendicular lean mass | Alzheimer's disease, wide (FinnGen) | rs146851424 | 13:50377910 | C/A | 0.022 | 0.1003 (0.0066) | 1.6E-52 | -0.1613 (0.0809) | 0.046 | 230.9 | -1.608 |
| Appendicular lean mass | Alzheimer's disease, wide (FinnGen) | rs1325596 | 1:176794066 | A/G | 0.548 | 0.0287 (0.0019) | 2.8E-52 | -0.0058 (0.0213) | 0.786 | 228.2 | -0.202 |
| Appendicular lean mass | Alzheimer's disease, wide (FinnGen) | rs72801818 | 16:53476042 | T/C | 0.300 | 0.0313 (0.0021) | 8.9E-52 | -0.0260 (0.0243) | 0.284 | 222.2 | -0.831 |
| Appendicular lean mass | Alzheimer's disease, wide (FinnGen) | rs13103161 | 4:106216459 | A/T | 0.389 | -0.0284 (0.0019) | 2.4E-48 | -0.0020 (0.0224) | 0.928 | 223.4 | 0.070 |
| Appendicular lean mass | Alzheimer's disease, wide (FinnGen) | rs34517439 | 1:78450517 | A/C | 0.122 | 0.0421 (0.0029) | 5.8E-48 | -0.0389 (0.0321) | 0.226 | 210.8 | -0.924 |
| Appendicular lean mass | Alzheimer's disease, wide (FinnGen) | rs10491967 | 12:3368093 | A/G | 0.092 | 0.0471 (0.0032) | 1.3E-47 | 0.0035 (0.0396) | 0.929 | 216.6 | 0.074 |
| Appendicular lean mass | Alzheimer's disease, wide (FinnGen) | rs723149 | 7:46577056 | G/A | 0.563 | -0.0276 (0.0019) | 1.4E-47 | 0.0030 (0.0214) | 0.890 | 211.0 | -0.109 |
| Appendicular lean mass | Alzheimer's disease, wide (FinnGen) | rs4909912 | 8:135572297 | T/C | 0.427 | -0.0277 (0.0019) | 1.7E-47 | -0.0201 (0.0212) | 0.344 | 212.6 | 0.726 |
| Appendicular lean mass | Alzheimer's disease, wide (FinnGen) | rs34776209 | 7:23513093 | T/C | 0.247 | -0.0317 (0.0022) | 1.8E-47 | -0.0152 (0.0262) | 0.561 | 207.6 | 0.479 |
| Appendicular lean mass | Alzheimer's disease, wide (FinnGen) | rs41271299 | 6:19839415 | T/C | 0.051 | 0.0616 (0.0043) | 3.2E-47 | 0.0636 (0.0841) | 0.450 | 205.2 | 1.032 |
| Appendicular lean mass | Alzheimer's disease, wide (FinnGen) | rs1319012 | 6:41852616 | A/T | 0.926 | -0.0520 (0.0037) | 3.3E-45 | -0.0185 (0.0357) | 0.604 | 197.5 | 0.356 |
| Appendicular lean mass | Alzheimer's disease, wide (FinnGen) | rs7095472 | 10:70399109 | G/A | 0.533 | 0.0267 (0.0019) | 7.7E-45 | -0.0213 (0.0213) | 0.318 | 197.5 | -0.798 |
| Appendicular lean mass | Alzheimer's disease, wide (FinnGen) | rs59985551 | 2:56106928 | T/C | 0.226 | -0.0313 (0.0022) | 2.4E-44 | 0.0204 (0.0254) | 0.421 | 202.4 | -0.652 |
| Appendicular lean mass | Alzheimer's disease, wide (FinnGen) | rs10283100 | 8:120596023 | G/A | 0.945 | 0.0575 (0.0041) | 4.1E-44 | -0.0258 (0.0411) | 0.530 | 196.7 | -0.449 |
| Appendicular lean mass | Alzheimer's disease, wide (FinnGen) | rs6931421 | 6:80880138 | G/T | 0.322 | -0.0279 (0.0020) | 2.3E-43 | -0.0321 (0.0233) | 0.168 | 194.6 | 1.151 |
| Appendicular lean mass | Alzheimer's disease, wide (FinnGen) | rs17205463 | 15:62381413 | T/C | 0.448 | -0.0263 (0.0019) | 4.2E-43 | 0.0120 (0.0225) | 0.592 | 191.6 | -0.456 |
| Appendicular lean mass | Alzheimer's disease, wide (FinnGen) | rs2270894 | 3:9975386 | G/C | 0.203 | -0.0332 (0.0024) | 1.3E-42 | -0.0224 (0.0242) | 0.354 | 191.4 | 0.675 |
| Appendicular lean mass | Alzheimer's disease, wide (FinnGen) | rs4282339 | 5:168256240 | A/G | 0.207 | -0.0311 (0.0023) | 6.2E-41 | -0.0233 (0.0271) | 0.391 | 182.8 | 0.749 |
| Appendicular lean mass | Alzheimer's disease, wide (FinnGen) | rs10883555 | 10:102657908 | T/C | 0.550 | 0.0254 (0.0019) | 9.1E-41 | 0.0177 (0.0214) | 0.406 | 178.7 | 0.697 |
| Appendicular lean mass | Alzheimer's disease, wide (FinnGen) | rs12051245 | 16:783865 | C/T | 0.232 | 0.0299 (0.0022) | 2.6E-40 | 0.0010 (0.0224) | 0.963 | 184.7 | 0.033 |
| Appendicular lean mass | Alzheimer's disease, wide (FinnGen) | rs11014285 | 10:25178864 | A/G | 0.165 | 0.0342 (0.0026) | 2.9E-40 | -0.0153 (0.0354) | 0.666 | 173.0 | -0.447 |
| Appendicular lean mass | Alzheimer's disease, wide (FinnGen) | rs2229840 | 12:124826462 | T/C | 0.160 | 0.0341 (0.0026) | 3.0E-40 | -0.0065 (0.0299) | 0.828 | 172.0 | -0.191 |
| Appendicular lean mass | Alzheimer's disease, wide (FinnGen) | rs35230100 | 14:70348141 | CATAT/C | 0.433 | -0.0262 (0.0020) | 4.4E-40 | 0.0018 (0.0216) | 0.935 | 171.6 | -0.069 |
| Appendicular lean mass | Alzheimer's disease, wide (FinnGen) | rs35309034 | 8:126498265 | A/C | 0.238 | -0.0297 (0.0022) | 1.1E-39 | 0.0177 (0.0232) | 0.445 | 182.2 | -0.596 |
| Appendicular lean mass | Alzheimer's disease, wide (FinnGen) | rs7952436 | 11:67024534 | T/C | 0.082 | -0.0453 (0.0034) | 1.6E-39 | 0.0225 (0.0484) | 0.642 | 177.5 | -0.497 |
| Appendicular lean mass | Alzheimer's disease, wide (FinnGen) | rs8054549 | 16:86417234 | A/C | 0.449 | -0.0251 (0.0019) | 3.4E-39 | -0.0004 (0.0217) | 0.987 | 174.5 | 0.016 |
| Appendicular lean mass | Alzheimer's disease, wide (FinnGen) | rs3764002 | 12:108618630 | T/C | 0.262 | 0.0280 (0.0021) | 4.5E-39 | -0.0167 (0.0223) | 0.453 | 177.8 | -0.596 |
| Appendicular lean mass | Alzheimer's disease, wide (FinnGen) | rs12714414 | 2:651407 | C/T | 0.152 | -0.0353 (0.0027) | 8.2E-39 | 0.0114 (0.0286) | 0.689 | 170.9 | -0.323 |
| Appendicular lean mass | Alzheimer's disease, wide (FinnGen) | rs5742915 | 15:74336633 | C/T | 0.461 | 0.0248 (0.0019) | 9.3E-39 | -0.0296 (0.0216) | 0.171 | 170.4 | -1.194 |
| Appendicular lean mass | Alzheimer's disease, wide (FinnGen) | rs33973388 | 18:46611842 | T/G | 0.435 | 0.0249 (0.0019) | 1.4E-38 | 0.0127 (0.0231) | 0.583 | 171.8 | 0.510 |
| Appendicular lean mass | Alzheimer's disease, wide (FinnGen) | rs8019890 | 14:21538067 | A/C | 0.531 | 0.0250 (0.0019) | 2.0E-38 | 0.0042 (0.0215) | 0.845 | 173.1 | 0.168 |
| Appendicular lean mass | Alzheimer's disease, wide (FinnGen) | rs8042545 | 15:66992821 | A/G | 0.242 | 0.0287 (0.0022) | 2.9E-38 | -0.0136 (0.0254) | 0.592 | 170.2 | -0.474 |
| Appendicular lean mass | Alzheimer's disease, wide (FinnGen) | rs2900208 | 12:11878464 | A/C | 0.354 | 0.0255 (0.0020) | 3.8E-38 | -0.0214 (0.0218) | 0.327 | 162.6 | -0.839 |
| Appendicular lean mass | Alzheimer's disease, wide (FinnGen) | rs10748128 | 12:69827658 | T/G | 0.345 | 0.0255 (0.0020) | 6.8E-38 | 0.0072 (0.0222) | 0.746 | 162.6 | 0.282 |
| Appendicular lean mass | Alzheimer's disease, wide (FinnGen) | rs28592876 | 12:123866429 | A/G | 0.205 | 0.0300 (0.0023) | 9.1E-38 | -0.0157 (0.0261) | 0.548 | 170.1 | -0.523 |
| Appendicular lean mass | Alzheimer's disease, wide (FinnGen) | rs4615815 | 1:214604236 | G/A | 0.397 | -0.0246 (0.0019) | 1.1E-37 | -0.0117 (0.0223) | 0.599 | 167.6 | 0.476 |
| Appendicular lean mass | Alzheimer's disease, wide (FinnGen) | rs244711 | 5:176509193 | T/C | 0.686 | 0.0279 (0.0022) | 1.5E-37 | -0.0428 (0.0230) | 0.064 | 160.8 | -1.534 |
| Appendicular lean mass | Alzheimer's disease, wide (FinnGen) | rs17036160 | 3:12329783 | T/C | 0.118 | -0.0375 (0.0029) | 1.7E-37 | 0.0356 (0.0282) | 0.207 | 167.2 | -0.949 |
| Appendicular lean mass | Alzheimer's disease, wide (FinnGen) | rs36226649 | 14:24835500 | C/T | 0.067 | 0.0485 (0.0038) | 3.1E-37 | -0.0134 (0.0494) | 0.786 | 162.9 | -0.276 |
| Appendicular lean mass | Alzheimer's disease, wide (FinnGen) | rs61944841 | 13:27049616 | A/G | 0.414 | 0.0253 (0.0020) | 3.5E-37 | 0.0002 (0.0219) | 0.994 | 160.0 | 0.008 |
| Appendicular lean mass | Alzheimer's disease, wide (FinnGen) | rs3853252 | 6:152170247 | A/G | 0.455 | 0.0241 (0.0019) | 8.8E-37 | -0.0158 (0.0216) | 0.463 | 160.9 | -0.656 |
| Appendicular lean mass | Alzheimer's disease, wide (FinnGen) | rs7301341 | 12:94083105 | C/T | 0.327 | -0.0255 (0.0020) | 9.3E-37 | -0.0242 (0.0223) | 0.279 | 162.6 | 0.949 |
| Appendicular lean mass | Alzheimer's disease, wide (FinnGen) | rs12622189 | 2:218132858 | G/A | 0.258 | -0.0272 (0.0021) | 9.3E-37 | 0.0038 (0.0221) | 0.864 | 167.8 | -0.140 |
| Appendicular lean mass | Alzheimer's disease, wide (FinnGen) | rs2035901 | 4:145521867 | G/A | 0.468 | 0.0240 (0.0019) | 9.4E-37 | 0.0143 (0.0216) | 0.507 | 159.6 | 0.596 |
| Appendicular lean mass | Alzheimer's disease, wide (FinnGen) | rs778384 | 1:56637166 | A/G | 0.223 | 0.0283 (0.0022) | 2.8E-36 | -0.0266 (0.0260) | 0.306 | 165.5 | -0.940 |
| Appendicular lean mass | Alzheimer's disease, wide (FinnGen) | rs3843750 | 19:10748073 | G/C | 0.664 | -0.0252 (0.0020) | 5.1E-36 | 0.0190 (0.0236) | 0.421 | 158.8 | -0.754 |
| Appendicular lean mass | Alzheimer's disease, wide (FinnGen) | rs4752829 | 11:47396654 | A/G | 0.286 | 0.0262 (0.0021) | 5.9E-36 | 0.0044 (0.0224) | 0.844 | 155.7 | 0.168 |
| Appendicular lean mass | Alzheimer's disease, wide (FinnGen) | rs10691068 | 6:109723017 | TCC/T | 0.407 | -0.0242 (0.0019) | 6.5E-36 | 0.0106 (0.0213) | 0.620 | 162.2 | -0.438 |
| Appendicular lean mass | Alzheimer's disease, wide (FinnGen) | rs59950280 | 4:3452345 | A/G | 0.332 | -0.0254 (0.0020) | 7.3E-36 | 0.0030 (0.0248) | 0.905 | 161.3 | -0.118 |
| Appendicular lean mass | Alzheimer's disease, wide (FinnGen) | rs11689546 | 2:25130451 | G/A | 0.440 | -0.0238 (0.0019) | 8.1E-36 | -0.0107 (0.0217) | 0.623 | 156.9 | 0.450 |
| Appendicular lean mass | Alzheimer's disease, wide (FinnGen) | rs2098695 | 5:54868944 | G/A | 0.691 | -0.0255 (0.0020) | 1.1E-35 | 0.0174 (0.0231) | 0.453 | 162.6 | -0.682 |
| Appendicular lean mass | Alzheimer's disease, wide (FinnGen) | rs7582516 | 2:227255370 | T/C | 0.304 | 0.0254 (0.0020) | 1.3E-35 | -0.0326 (0.0238) | 0.170 | 161.3 | -1.283 |
| Appendicular lean mass | Alzheimer's disease, wide (FinnGen) | rs3818416 | 13:78474468 | C/A | 0.764 | 0.0279 (0.0022) | 2.0E-35 | -0.0142 (0.0255) | 0.577 | 160.8 | -0.509 |
| Appendicular lean mass | Alzheimer's disease, wide (FinnGen) | rs2549677 | 16:2162361 | G/A | 0.099 | -0.0392 (0.0032) | 1.3E-34 | 0.0199 (0.0422) | 0.637 | 150.1 | -0.508 |
| Appendicular lean mass | Alzheimer's disease, wide (FinnGen) | rs1177765 | 5:32829929 | C/T | 0.468 | -0.0232 (0.0019) | 1.3E-34 | 0.0022 (0.0211) | 0.917 | 149.1 | -0.095 |
| Appendicular lean mass | Alzheimer's disease, wide (FinnGen) | rs61838776 | 1:219618564 | A/C | 0.162 | 0.0311 (0.0025) | 2.4E-34 | 0.0641 (0.0325) | 0.049 | 154.8 | 2.061 |
| Appendicular lean mass | Alzheimer's disease, wide (FinnGen) | rs115912456 | 5:82815158 | G/A | 0.041 | 0.0577 (0.0047) | 3.7E-34 | -0.0249 (0.0778) | 0.749 | 150.7 | -0.432 |
| Appendicular lean mass | Alzheimer's disease, wide (FinnGen) | rs4852257 | 2:71678520 | G/T | 0.576 | -0.0231 (0.0019) | 6.2E-34 | 0.0037 (0.0212) | 0.861 | 147.8 | -0.160 |
| Appendicular lean mass | Alzheimer's disease, wide (FinnGen) | rs12761076 | 10:72423050 | A/G | 0.266 | -0.0260 (0.0021) | 1.0E-33 | 0.0020 (0.0239) | 0.935 | 153.3 | -0.077 |
| Appendicular lean mass | Alzheimer's disease, wide (FinnGen) | rs111365325 | 5:170865229 | T/C | 0.231 | -0.0271 (0.0022) | 1.1E-33 | 0.0233 (0.0264) | 0.377 | 151.7 | -0.860 |
| Appendicular lean mass | Alzheimer's disease, wide (FinnGen) | rs45474992 | 19:47724564 | T/C | 0.036 | -0.0617 (0.0051) | 2.2E-33 | 0.0014 (0.0595) | 0.981 | 146.4 | -0.023 |
| Appendicular lean mass | Alzheimer's disease, wide (FinnGen) | rs7428883 | 3:128983853 | G/A | 0.781 | 0.0277 (0.0023) | 2.3E-33 | 0.0100 (0.0285) | 0.724 | 145.1 | 0.361 |
| Appendicular lean mass | Alzheimer's disease, wide (FinnGen) | rs76364830 | 8:13372120 | A/G | 0.063 | -0.0471 (0.0039) | 2.7E-33 | 0.0182 (0.0582) | 0.754 | 145.8 | -0.386 |
| Appendicular lean mass | Alzheimer's disease, wide (FinnGen) | rs10202701 | 2:232328681 | T/C | 0.542 | 0.0227 (0.0019) | 3.1E-33 | -0.0205 (0.0213) | 0.335 | 142.7 | -0.903 |
| Appendicular lean mass | Alzheimer's disease, wide (FinnGen) | rs4976262 | 5:134379531 | C/T | 0.316 | -0.0245 (0.0020) | 4.4E-33 | -0.0090 (0.0222) | 0.683 | 150.1 | 0.367 |
| Appendicular lean mass | Alzheimer's disease, wide (FinnGen) | rs6082354 | 20:21217976 | C/A | 0.667 | -0.0240 (0.0020) | 1.2E-32 | 0.0281 (0.0234) | 0.229 | 144.0 | -1.171 |
| Appendicular lean mass | Alzheimer's disease, wide (FinnGen) | rs2209098 | 1:172167226 | C/T | 0.311 | 0.0240 (0.0020) | 1.7E-32 | 0.0078 (0.0231) | 0.736 | 144.0 | 0.325 |
| Appendicular lean mass | Alzheimer's disease, wide (FinnGen) | rs1444628 | 12:20563643 | T/C | 0.690 | 0.0240 (0.0020) | 6.8E-32 | -0.0161 (0.0259) | 0.534 | 144.0 | -0.671 |
| Appendicular lean mass | Alzheimer's disease, wide (FinnGen) | rs147110934 | 19:55993436 | T/G | 0.024 | -0.0722 (0.0062) | 9.4E-32 | -0.0181 (0.1386) | 0.896 | 135.6 | 0.251 |
| Appendicular lean mass | Alzheimer's disease, wide (FinnGen) | rs148138388 | 1:51371608 | C/G | 0.083 | 0.0406 (0.0035) | 1.1E-31 | -0.0061 (0.0456) | 0.894 | 134.6 | -0.150 |
| Appendicular lean mass | Alzheimer's disease, wide (FinnGen) | rs58738817 | 3:184012228 | A/G | 0.248 | -0.0257 (0.0022) | 1.2E-31 | 0.0198 (0.0232) | 0.392 | 136.5 | -0.770 |
| Appendicular lean mass | Alzheimer's disease, wide (FinnGen) | rs6570509 | 6:142716286 | T/G | 0.287 | -0.0244 (0.0021) | 1.3E-31 | -0.0084 (0.0243) | 0.730 | 135.0 | 0.344 |
| Appendicular lean mass | Alzheimer's disease, wide (FinnGen) | rs4252548 | 19:55879672 | T/C | 0.022 | -0.0753 (0.0065) | 3.0E-31 | -0.0147 (0.0680) | 0.829 | 134.2 | 0.195 |
| Appendicular lean mass | Alzheimer's disease, wide (FinnGen) | rs35464459 | 10:4964640 | C/T | 0.116 | -0.0342 (0.0029) | 5.0E-31 | -0.0093 (0.0317) | 0.770 | 139.1 | 0.272 |
| Appendicular lean mass | Alzheimer's disease, wide (FinnGen) | rs1047891 | 2:211540507 | A/C | 0.316 | 0.0233 (0.0020) | 5.7E-31 | -0.0433 (0.0228) | 0.057 | 135.7 | -1.858 |
| Appendicular lean mass | Alzheimer's disease, wide (FinnGen) | rs35756741 | 12:12868701 | T/C | 0.092 | -0.0378 (0.0033) | 5.8E-31 | 0.0106 (0.0349) | 0.762 | 131.2 | -0.280 |
| Appendicular lean mass | Alzheimer's disease, wide (FinnGen) | rs55872725 | 16:53809123 | T/C | 0.404 | 0.0222 (0.0019) | 1.5E-30 | -0.0028 (0.0215) | 0.896 | 136.5 | -0.126 |
| Appendicular lean mass | Alzheimer's disease, wide (FinnGen) | rs6501381 | 17:68148350 | C/T | 0.113 | -0.0341 (0.0030) | 5.2E-30 | -0.0010 (0.0373) | 0.980 | 129.2 | 0.029 |
| Appendicular lean mass | Alzheimer's disease, wide (FinnGen) | rs12509014 | 4:123825919 | T/C | 0.790 | -0.0263 (0.0023) | 5.4E-30 | 0.0084 (0.0275) | 0.761 | 130.8 | -0.319 |
| Appendicular lean mass | Alzheimer's disease, wide (FinnGen) | rs8031704 | 15:84651185 | A/C | 0.327 | -0.0228 (0.0020) | 2.1E-29 | 0.0151 (0.0225) | 0.502 | 130.0 | -0.662 |
| Appendicular lean mass | Alzheimer's disease, wide (FinnGen) | rs212526 | 1:21584941 | C/T | 0.601 | 0.0214 (0.0019) | 3.8E-29 | -0.0142 (0.0219) | 0.518 | 126.9 | -0.664 |
| Appendicular lean mass | Alzheimer's disease, wide (FinnGen) | rs9647379 | 3:171785168 | C/G | 0.411 | 0.0215 (0.0019) | 5.5E-29 | 0.0032 (0.0217) | 0.882 | 128.1 | 0.149 |
| Appendicular lean mass | Alzheimer's disease, wide (FinnGen) | rs4244809 | 11:2164333 | A/G | 0.208 | -0.0262 (0.0023) | 5.6E-29 | -0.0194 (0.0276) | 0.483 | 129.8 | 0.740 |
| Appendicular lean mass | Alzheimer's disease, wide (FinnGen) | rs4076427 | 17:79087537 | C/G | 0.391 | -0.0218 (0.0019) | 6.0E-29 | 0.0043 (0.0227) | 0.850 | 131.7 | -0.197 |
| Appendicular lean mass | Alzheimer's disease, wide (FinnGen) | rs7229520 | 18:46516468 | A/G | 0.662 | -0.0224 (0.0020) | 9.2E-29 | -0.0047 (0.0222) | 0.832 | 125.4 | 0.210 |
| Appendicular lean mass | Alzheimer's disease, wide (FinnGen) | rs6762851 | 3:56686329 | C/T | 0.357 | -0.0218 (0.0020) | 1.5E-28 | 0.0318 (0.0215) | 0.138 | 118.8 | -1.459 |
| Appendicular lean mass | Alzheimer's disease, wide (FinnGen) | rs1211575 | 1:11118390 | A/G | 0.743 | -0.0237 (0.0021) | 1.9E-28 | 0.0201 (0.0237) | 0.397 | 127.4 | -0.848 |
| Appendicular lean mass | Alzheimer's disease, wide (FinnGen) | rs4274112 | 1:26746199 | G/A | 0.373 | -0.0217 (0.0020) | 2.5E-28 | 0.0162 (0.0223) | 0.469 | 117.7 | -0.747 |
| Appendicular lean mass | Alzheimer's disease, wide (FinnGen) | rs2071518 | 8:120435812 | T/C | 0.264 | -0.0236 (0.0021) | 3.4E-28 | 0.0372 (0.0265) | 0.160 | 126.3 | -1.576 |
| Appendicular lean mass | Alzheimer's disease, wide (FinnGen) | rs13302480 | 9:118465313 | C/G | 0.114 | -0.0328 (0.0030) | 4.4E-28 | 0.0891 (0.0426) | 0.037 | 119.5 | -2.716 |
| Appendicular lean mass | Alzheimer's disease, wide (FinnGen) | rs36048468 | 5:122879901 | T/C | 0.209 | 0.0254 (0.0023) | 9.3E-28 | 0.0411 (0.0289) | 0.154 | 122.0 | 1.618 |
| Appendicular lean mass | Alzheimer's disease, wide (FinnGen) | rs62370472 | 5:52767109 | C/T | 0.209 | -0.0253 (0.0023) | 1.4E-27 | 0.0175 (0.0250) | 0.486 | 121.0 | -0.692 |
| Appendicular lean mass | Alzheimer's disease, wide (FinnGen) | rs77165542 | 2:430975 | T/C | 0.035 | -0.0557 (0.0051) | 1.8E-27 | -0.1192 (0.0812) | 0.142 | 119.3 | 2.140 |
| Appendicular lean mass | Alzheimer's disease, wide (FinnGen) | rs62621812 | 7:127015083 | A/G | 0.020 | 0.0743 (0.0069) | 3.2E-27 | -0.0435 (0.0529) | 0.411 | 116.0 | -0.585 |
| Appendicular lean mass | Alzheimer's disease, wide (FinnGen) | rs181766 | 16:14394878 | C/T | 0.322 | 0.0221 (0.0020) | 4.3E-27 | 0.0096 (0.0222) | 0.665 | 122.1 | 0.434 |
| Appendicular lean mass | Alzheimer's disease, wide (FinnGen) | rs62048221 | 16:88846552 | T/C | 0.231 | -0.0242 (0.0022) | 4.9E-27 | 0.0315 (0.0265) | 0.235 | 121.0 | -1.302 |
| Appendicular lean mass | Alzheimer's disease, wide (FinnGen) | rs7781964 | 7:139749346 | A/G | 0.185 | 0.0261 (0.0024) | 5.7E-27 | -0.0090 (0.0278) | 0.746 | 118.3 | -0.345 |
| Appendicular lean mass | Alzheimer's disease, wide (FinnGen) | rs2070598 | 14:75360906 | A/G | 0.456 | 0.0204 (0.0019) | 6.4E-27 | 0.0243 (0.0214) | 0.257 | 115.3 | 1.191 |
| Appendicular lean mass | Alzheimer's disease, wide (FinnGen) | rs13209685 | 6:7779729 | T/G | 0.159 | 0.0277 (0.0026) | 7.5E-27 | 0.0086 (0.0263) | 0.743 | 113.5 | 0.310 |
| Appendicular lean mass | Alzheimer's disease, wide (FinnGen) | rs951366 | 1:205685352 | C/T | 0.393 | 0.0205 (0.0019) | 9.2E-27 | -0.0347 (0.0221) | 0.115 | 116.4 | -1.693 |
| Appendicular lean mass | Alzheimer's disease, wide (FinnGen) | rs291970 | 10:121123633 | T/C | 0.229 | 0.0240 (0.0022) | 1.3E-26 | -0.0115 (0.0288) | 0.688 | 119.0 | -0.479 |
| Appendicular lean mass | Alzheimer's disease, wide (FinnGen) | rs4752689 | 10:124131176 | A/G | 0.584 | 0.0205 (0.0019) | 1.4E-26 | -0.0730 (0.0233) | 0.002 | 116.4 | -3.561 |
| Appendicular lean mass | Alzheimer's disease, wide (FinnGen) | rs7768382 | 6:166341870 | C/T | 0.477 | -0.0201 (0.0019) | 1.6E-26 | 0.0222 (0.0212) | 0.294 | 111.9 | -1.104 |
| Appendicular lean mass | Alzheimer's disease, wide (FinnGen) | rs72841270 | 10:104642237 | G/T | 0.135 | 0.0294 (0.0028) | 2.3E-26 | 0.0125 (0.0307) | 0.684 | 110.2 | 0.425 |
| Appendicular lean mass | Alzheimer's disease, wide (FinnGen) | rs7014590 | 8:89335647 | C/T | 0.261 | -0.0228 (0.0022) | 4.5E-26 | 0.0089 (0.0245) | 0.718 | 107.4 | -0.390 |
| Appendicular lean mass | Alzheimer's disease, wide (FinnGen) | rs9659061 | 1:150527292 | A/G | 0.476 | -0.0198 (0.0019) | 6.8E-26 | -0.0070 (0.0215) | 0.743 | 108.6 | 0.354 |
| Appendicular lean mass | Alzheimer's disease, wide (FinnGen) | rs7497684 | 15:89342720 | A/C | 0.480 | 0.0200 (0.0019) | 8.3E-26 | -0.0123 (0.0215) | 0.569 | 110.8 | -0.615 |
| Appendicular lean mass | Alzheimer's disease, wide (FinnGen) | rs1662842 | 4:82155362 | G/A | 0.307 | 0.0214 (0.0020) | 1.7E-25 | 0.0092 (0.0219) | 0.674 | 114.5 | 0.430 |
| Appendicular lean mass | Alzheimer's disease, wide (FinnGen) | rs7072873 | 10:77210191 | T/C | 0.539 | -0.0198 (0.0019) | 3.2E-25 | 0.0034 (0.0212) | 0.874 | 108.6 | -0.172 |
| Appendicular lean mass | Alzheimer's disease, wide (FinnGen) | rs4640244 | 17:21284223 | G/A | 0.399 | -0.0200 (0.0019) | 3.8E-25 | 0.0089 (0.0216) | 0.683 | 110.8 | -0.445 |
| Appendicular lean mass | Alzheimer's disease, wide (FinnGen) | rs7858712 | 9:16738312 | G/A | 0.914 | 0.0347 (0.0034) | 1.0E-24 | -0.0600 (0.0390) | 0.124 | 104.2 | -1.729 |
| Appendicular lean mass | Alzheimer's disease, wide (FinnGen) | rs927376 | 20:38569825 | G/C | 0.260 | -0.0223 (0.0022) | 1.2E-24 | -0.0185 (0.0273) | 0.499 | 102.8 | 0.830 |
| Appendicular lean mass | Alzheimer's disease, wide (FinnGen) | rs78457529 | 16:24950880 | T/C | 0.012 | -0.0904 (0.0088) | 1.2E-24 | -0.0472 (0.0804) | 0.557 | 105.5 | 0.522 |
| Appendicular lean mass | Alzheimer's disease, wide (FinnGen) | rs2748501 | 6:146312258 | A/G | 0.440 | -0.0195 (0.0019) | 1.3E-24 | 0.0160 (0.0213) | 0.451 | 105.3 | -0.821 |
| Appendicular lean mass | Alzheimer's disease, wide (FinnGen) | rs12882130 | 14:103878774 | G/C | 0.384 | -0.0202 (0.0020) | 1.9E-24 | 0.0061 (0.0219) | 0.780 | 102.0 | -0.302 |
| Appendicular lean mass | Alzheimer's disease, wide (FinnGen) | rs45528934 | 14:23793305 | T/C | 0.162 | 0.0262 (0.0026) | 2.0E-24 | 0.0688 (0.0314) | 0.028 | 101.5 | 2.626 |
| Appendicular lean mass | Alzheimer's disease, wide (FinnGen) | rs41311445 | 22:42070374 | C/A | 0.096 | -0.0328 (0.0032) | 4.7E-24 | 0.0073 (0.0311) | 0.813 | 105.1 | -0.223 |
| Appendicular lean mass | Alzheimer's disease, wide (FinnGen) | rs60804050 | 1:118870373 | A/G | 0.256 | -0.0217 (0.0021) | 5.0E-24 | 0.0092 (0.0248) | 0.711 | 106.8 | -0.424 |
| Appendicular lean mass | Alzheimer's disease, wide (FinnGen) | rs7816345 | 8:36846109 | T/C | 0.168 | 0.0255 (0.0025) | 6.2E-24 | 0.0151 (0.0307) | 0.623 | 104.0 | 0.592 |
| Appendicular lean mass | Alzheimer's disease, wide (FinnGen) | rs2627702 | 4:88577419 | C/T | 0.519 | -0.0191 (0.0019) | 6.4E-24 | 0.0327 (0.0218) | 0.133 | 101.1 | -1.712 |
| Appendicular lean mass | Alzheimer's disease, wide (FinnGen) | rs7485647 | 12:131631133 | A/G | 0.844 | -0.0261 (0.0026) | 1.0E-23 | -0.0093 (0.0242) | 0.702 | 100.8 | 0.356 |
| Appendicular lean mass | Alzheimer's disease, wide (FinnGen) | rs2296316 | 14:65520246 | C/T | 0.464 | -0.0192 (0.0019) | 1.6E-23 | -0.0191 (0.0212) | 0.367 | 102.1 | 0.995 |
| Appendicular lean mass | Alzheimer's disease, wide (FinnGen) | rs2019203 | 17:36908672 | A/C | 0.491 | 0.0189 (0.0019) | 1.8E-23 | -0.0077 (0.0212) | 0.716 | 99.0 | -0.407 |
| Appendicular lean mass | Alzheimer's disease, wide (FinnGen) | rs17035465 | 2:46890294 | G/A | 0.221 | 0.0226 (0.0023) | 2.1E-23 | 0.0297 (0.0299) | 0.321 | 96.5 | 1.314 |
| Appendicular lean mass | Alzheimer's disease, wide (FinnGen) | rs8077636 | 17:79421254 | T/G | 0.418 | -0.0192 (0.0019) | 2.5E-23 | 0.0220 (0.0222) | 0.322 | 102.1 | -1.146 |
| Appendicular lean mass | Alzheimer's disease, wide (FinnGen) | rs6919321 | 6:33724004 | A/G | 0.608 | 0.0192 (0.0019) | 3.6E-23 | 0.0022 (0.0238) | 0.925 | 102.1 | 0.115 |
| Appendicular lean mass | Alzheimer's disease, wide (FinnGen) | rs2140046 | 2:169706079 | C/T | 0.363 | -0.0192 (0.0019) | 5.5E-23 | -0.0034 (0.0215) | 0.875 | 102.1 | 0.177 |
| Appendicular lean mass | Alzheimer's disease, wide (FinnGen) | rs1786263 | 18:13116432 | T/G | 0.606 | -0.0190 (0.0019) | 1.0E-22 | -0.0077 (0.0216) | 0.720 | 100.0 | 0.405 |
| Appendicular lean mass | Alzheimer's disease, wide (FinnGen) | rs9807032 | 17:62757146 | C/T | 0.818 | -0.0240 (0.0024) | 1.3E-22 | 0.0168 (0.0287) | 0.559 | 100.0 | -0.700 |
| Appendicular lean mass | Alzheimer's disease, wide (FinnGen) | rs140440099 | 3:50632595 | A/G | 0.023 | 0.0613 (0.0063) | 1.4E-22 | 0.0874 (0.0846) | 0.301 | 94.7 | 1.426 |
| Appendicular lean mass | Alzheimer's disease, wide (FinnGen) | rs113852999 | 7:148649606 | A/G | 0.169 | 0.0246 (0.0025) | 1.8E-22 | -0.0591 (0.0280) | 0.035 | 96.8 | -2.402 |
| Appendicular lean mass | Alzheimer's disease, wide (FinnGen) | rs35732917 | 7:73013269 | C/T | 0.284 | 0.0204 (0.0021) | 2.1E-22 | 0.0003 (0.0252) | 0.992 | 94.4 | 0.015 |
| Appendicular lean mass | Alzheimer's disease, wide (FinnGen) | rs9344126 | 6:81907559 | C/T | 0.514 | -0.0185 (0.0019) | 2.2E-22 | -0.0125 (0.0216) | 0.562 | 94.8 | 0.676 |
| Appendicular lean mass | Alzheimer's disease, wide (FinnGen) | rs3184504 | 12:111884608 | C/T | 0.517 | 0.0183 (0.0019) | 2.7E-22 | -0.0226 (0.0215) | 0.292 | 92.8 | -1.235 |
| Appendicular lean mass | Alzheimer's disease, wide (FinnGen) | rs332116 | 10:28926099 | T/C | 0.280 | -0.0206 (0.0021) | 2.9E-22 | -0.0018 (0.0217) | 0.935 | 96.2 | 0.087 |
| Appendicular lean mass | Alzheimer's disease, wide (FinnGen) | rs10202845 | 2:42575820 | G/A | 0.113 | -0.0288 (0.0030) | 5.3E-22 | -0.0247 (0.0349) | 0.478 | 92.2 | 0.858 |
| Appendicular lean mass | Alzheimer's disease, wide (FinnGen) | rs2305141 | 2:233684402 | G/A | 0.596 | 0.0183 (0.0019) | 1.1E-21 | -0.0094 (0.0239) | 0.694 | 92.8 | -0.514 |
| Appendicular lean mass | Alzheimer's disease, wide (FinnGen) | rs1405212 | 6:117490664 | C/T | 0.628 | 0.0187 (0.0019) | 1.1E-21 | -0.0156 (0.0224) | 0.487 | 96.9 | -0.834 |
| Appendicular lean mass | Alzheimer's disease, wide (FinnGen) | rs9838614 | 3:38537671 | G/T | 0.388 | -0.0185 (0.0019) | 1.2E-21 | 0.0080 (0.0215) | 0.710 | 94.8 | -0.432 |
| Appendicular lean mass | Alzheimer's disease, wide (FinnGen) | rs12679359 | 8:130724556 | T/G | 0.137 | -0.0263 (0.0028) | 1.4E-21 | -0.0131 (0.0307) | 0.669 | 88.2 | 0.498 |
| Appendicular lean mass | Alzheimer's disease, wide (FinnGen) | rs6054390 | 20:6592094 | A/G | 0.628 | -0.0188 (0.0020) | 1.4E-21 | 0.0028 (0.0232) | 0.903 | 88.4 | -0.149 |
| Appendicular lean mass | Alzheimer's disease, wide (FinnGen) | rs10945540 | 6:158738975 | A/G | 0.665 | -0.0190 (0.0020) | 1.8E-21 | 0.0166 (0.0218) | 0.449 | 90.2 | -0.874 |
| Appendicular lean mass | Alzheimer's disease, wide (FinnGen) | rs670318 | 1:63727542 | C/T | 0.952 | 0.0413 (0.0044) | 2.5E-21 | -0.0177 (0.0455) | 0.696 | 88.1 | -0.429 |
| Appendicular lean mass | Alzheimer's disease, wide (FinnGen) | rs112537273 | 8:38248306 | C/T | 0.230 | -0.0212 (0.0022) | 3.3E-21 | 0.0100 (0.0277) | 0.717 | 92.9 | -0.472 |
| Appendicular lean mass | Alzheimer's disease, wide (FinnGen) | rs1040977 | 9:95554873 | T/C | 0.166 | -0.0240 (0.0025) | 3.9E-21 | 0.0211 (0.0335) | 0.529 | 92.2 | -0.879 |
| Appendicular lean mass | Alzheimer's disease, wide (FinnGen) | rs7701233 | 5:171218388 | C/T | 0.428 | -0.0179 (0.0019) | 5.1E-21 | 0.0143 (0.0212) | 0.499 | 88.8 | -0.799 |
| Appendicular lean mass | Alzheimer's disease, wide (FinnGen) | rs2212926 | 21:38066883 | A/C | 0.211 | -0.0220 (0.0023) | 7.7E-21 | 0.0440 (0.0303) | 0.146 | 91.5 | -2.000 |
| Appendicular lean mass | Alzheimer's disease, wide (FinnGen) | rs10832963 | 11:18664241 | G/T | 0.745 | -0.0203 (0.0022) | 9.2E-21 | -0.0282 (0.0218) | 0.196 | 85.1 | 1.389 |
| Appendicular lean mass | Alzheimer's disease, wide (FinnGen) | rs7636265 | 3:134374223 | A/G | 0.638 | -0.0183 (0.0020) | 9.4E-21 | 0.0049 (0.0230) | 0.831 | 83.7 | -0.268 |
| Appendicular lean mass | Alzheimer's disease, wide (FinnGen) | rs9384679 | 6:108864419 | T/C | 0.370 | -0.0182 (0.0019) | 9.9E-21 | -0.0102 (0.0215) | 0.633 | 91.8 | 0.560 |
| Appendicular lean mass | Alzheimer's disease, wide (FinnGen) | rs17478946 | 12:24093062 | G/A | 0.300 | -0.0192 (0.0021) | 1.0E-20 | -0.0096 (0.0242) | 0.692 | 83.6 | 0.500 |
| Appendicular lean mass | Alzheimer's disease, wide (FinnGen) | rs9957318 | 18:33039106 | G/A | 0.348 | 0.0187 (0.0020) | 1.0E-20 | 0.0032 (0.0228) | 0.888 | 87.4 | 0.171 |
| Appendicular lean mass | Alzheimer's disease, wide (FinnGen) | rs28787734 | 4:87560357 | T/C | 0.058 | 0.0385 (0.0041) | 1.1E-20 | 0.0601 (0.0659) | 0.362 | 88.2 | 1.561 |
| Appendicular lean mass | Alzheimer's disease, wide (FinnGen) | rs1473441 | 8:49588463 | G/A | 0.724 | -0.0198 (0.0021) | 1.2E-20 | -0.0491 (0.0242) | 0.043 | 88.9 | 2.480 |
| Appendicular lean mass | Alzheimer's disease, wide (FinnGen) | rs34287 | 5:67585143 | A/G | 0.341 | 0.0187 (0.0020) | 1.2E-20 | -0.0022 (0.0215) | 0.917 | 87.4 | -0.118 |
| Appendicular lean mass | Alzheimer's disease, wide (FinnGen) | rs35816944 | 16:1828030 | A/G | 0.007 | -0.1088 (0.0117) | 1.3E-20 | 0.0958 (0.1316) | 0.467 | 86.5 | -0.881 |
| Appendicular lean mass | Alzheimer's disease, wide (FinnGen) | rs7633464 | 3:98715823 | A/G | 0.479 | 0.0175 (0.0019) | 1.3E-20 | 0.0098 (0.0212) | 0.643 | 84.8 | 0.560 |
| Appendicular lean mass | Alzheimer's disease, wide (FinnGen) | rs2993531 | 1:212190116 | C/A | 0.589 | 0.0176 (0.0019) | 1.7E-20 | -0.0200 (0.0220) | 0.363 | 85.8 | -1.136 |
| Appendicular lean mass | Alzheimer's disease, wide (FinnGen) | rs9696116 | 9:139100805 | C/T | 0.318 | -0.0188 (0.0020) | 2.4E-20 | 0.0226 (0.0226) | 0.318 | 88.4 | -1.202 |
| Appendicular lean mass | Alzheimer's disease, wide (FinnGen) | rs3103268 | 2:232984969 | T/C | 0.902 | 0.0291 (0.0031) | 2.7E-20 | 0.0127 (0.0492) | 0.797 | 88.1 | 0.436 |
| Appendicular lean mass | Alzheimer's disease, wide (FinnGen) | rs4985445 | 16:69867835 | G/A | 0.457 | -0.0175 (0.0019) | 3.3E-20 | 0.0150 (0.0213) | 0.480 | 84.8 | -0.857 |
| Appendicular lean mass | Alzheimer's disease, wide (FinnGen) | rs11590254 | 1:92316573 | T/A | 0.311 | 0.0186 (0.0020) | 4.3E-20 | 0.0265 (0.0220) | 0.230 | 86.5 | 1.425 |
| Appendicular lean mass | Alzheimer's disease, wide (FinnGen) | rs73125634 | 20:20069826 | T/G | 0.278 | -0.0195 (0.0021) | 5.1E-20 | 0.0045 (0.0222) | 0.840 | 86.2 | -0.231 |
| Appendicular lean mass | Alzheimer's disease, wide (FinnGen) | rs6874142 | 5:172753555 | G/T | 0.114 | 0.0288 (0.0031) | 5.1E-20 | -0.0689 (0.0368) | 0.061 | 86.3 | -2.392 |
| Appendicular lean mass | Alzheimer's disease, wide (FinnGen) | rs140657345 | 15:41615909 | TCCTC/T | 0.252 | -0.0200 (0.0022) | 5.5E-20 | -0.0039 (0.0245) | 0.874 | 82.6 | 0.195 |
| Appendicular lean mass | Alzheimer's disease, wide (FinnGen) | rs62466110 | 7:92623541 | C/T | 0.067 | -0.0371 (0.0041) | 5.7E-20 | -0.0198 (0.0318) | 0.534 | 81.9 | 0.534 |
| Appendicular lean mass | Alzheimer's disease, wide (FinnGen) | rs12702693 | 7:8101039 | T/C | 0.454 | 0.0173 (0.0019) | 6.6E-20 | -0.0270 (0.0220) | 0.221 | 82.9 | -1.561 |
| Appendicular lean mass | Alzheimer's disease, wide (FinnGen) | rs34345560 | 11:69081998 | A/G | 0.195 | 0.0219 (0.0024) | 7.1E-20 | 0.0139 (0.0245) | 0.569 | 83.3 | 0.635 |
| Appendicular lean mass | Alzheimer's disease, wide (FinnGen) | rs12055045 | 5:137608056 | T/C | 0.206 | 0.0213 (0.0023) | 7.1E-20 | 0.0044 (0.0247) | 0.857 | 85.8 | 0.207 |
| Appendicular lean mass | Alzheimer's disease, wide (FinnGen) | rs655113 | 6:52269151 | C/T | 0.301 | 0.0188 (0.0021) | 7.4E-20 | 0.0018 (0.0224) | 0.935 | 80.2 | 0.096 |
| Appendicular lean mass | Alzheimer's disease, wide (FinnGen) | rs9832919 | 3:132184526 | G/A | 0.357 | -0.0179 (0.0020) | 7.9E-20 | -0.0090 (0.0222) | 0.685 | 80.1 | 0.503 |
| Appendicular lean mass | Alzheimer's disease, wide (FinnGen) | rs8107967 | 19:7972615 | G/A | 0.566 | -0.0174 (0.0019) | 9.5E-20 | -0.0075 (0.0213) | 0.725 | 83.9 | 0.431 |
| Appendicular lean mass | Alzheimer's disease, wide (FinnGen) | rs36012032 | 3:52814709 | A/C | 0.091 | 0.0298 (0.0033) | 9.9E-20 | 0.0086 (0.0381) | 0.821 | 81.5 | 0.289 |
| Appendicular lean mass | Alzheimer's disease, wide (FinnGen) | rs59753424 | 20:54884760 | A/G | 0.267 | 0.0200 (0.0022) | 1.1E-19 | 0.0024 (0.0267) | 0.929 | 82.6 | 0.120 |
| Appendicular lean mass | Alzheimer's disease, wide (FinnGen) | rs76693355 | 11:120292477 | C/T | 0.116 | -0.0268 (0.0030) | 1.2E-19 | 0.0335 (0.0320) | 0.295 | 79.8 | -1.250 |
| Appendicular lean mass | Alzheimer's disease, wide (FinnGen) | rs78180894 | 17:7483662 | C/G | 0.074 | 0.0338 (0.0037) | 1.3E-19 | -0.0935 (0.0784) | 0.233 | 83.5 | -2.766 |
| Appendicular lean mass | Alzheimer's disease, wide (FinnGen) | rs12533452 | 7:19016871 | T/C | 0.157 | 0.0237 (0.0026) | 1.3E-19 | -0.0119 (0.0305) | 0.697 | 83.1 | -0.502 |
| Appendicular lean mass | Alzheimer's disease, wide (FinnGen) | rs77542162 | 17:67081278 | G/A | 0.023 | 0.0576 (0.0064) | 1.5E-19 | 0.1281 (0.1312) | 0.329 | 81.0 | 2.224 |
| Appendicular lean mass | Alzheimer's disease, wide (FinnGen) | rs12997625 | 2:202970250 | T/C | 0.527 | -0.0170 (0.0019) | 1.5E-19 | 0.0094 (0.0214) | 0.660 | 80.1 | -0.553 |
| Appendicular lean mass | Alzheimer's disease, wide (FinnGen) | rs591668 | 3:27535931 | A/G | 0.396 | -0.0174 (0.0019) | 2.0E-19 | -0.0191 (0.0216) | 0.377 | 83.9 | 1.098 |
| Appendicular lean mass | Alzheimer's disease, wide (FinnGen) | rs11959466 | 5:42803824 | T/C | 0.056 | 0.0380 (0.0042) | 2.2E-19 | -0.0131 (0.0407) | 0.748 | 81.9 | -0.345 |
| Appendicular lean mass | Alzheimer's disease, wide (FinnGen) | rs261999 | 5:95899427 | T/C | 0.370 | 0.0175 (0.0019) | 2.5E-19 | -0.0157 (0.0216) | 0.468 | 84.8 | -0.897 |
| Appendicular lean mass | Alzheimer's disease, wide (FinnGen) | rs3063063 | 2:183231361 | TGAA/T | 0.686 | -0.0182 (0.0020) | 2.6E-19 | -0.0187 (0.0242) | 0.439 | 82.8 | 1.027 |
| Appendicular lean mass | Alzheimer's disease, wide (FinnGen) | rs11672848 | 19:37570704 | T/C | 0.525 | -0.0171 (0.0019) | 7.7E-19 | -0.0140 (0.0212) | 0.507 | 81.0 | 0.819 |
| Appendicular lean mass | Alzheimer's disease, wide (FinnGen) | rs894736 | 12:54418166 | G/A | 0.363 | -0.0174 (0.0020) | 1.1E-18 | 0.0266 (0.0212) | 0.211 | 75.7 | -1.529 |
| Appendicular lean mass | Alzheimer's disease, wide (FinnGen) | rs2112617 | 17:46977125 | A/G | 0.529 | -0.0167 (0.0019) | 1.1E-18 | 0.0143 (0.0211) | 0.498 | 77.2 | -0.856 |
| Appendicular lean mass | Alzheimer's disease, wide (FinnGen) | rs11049704 | 12:28691701 | G/C | 0.293 | -0.0183 (0.0021) | 1.1E-18 | 0.0604 (0.0242) | 0.013 | 75.9 | -3.301 |
| Appendicular lean mass | Alzheimer's disease, wide (FinnGen) | rs700677 | 2:198702424 | A/C | 0.350 | 0.0173 (0.0020) | 1.1E-18 | 0.0124 (0.0237) | 0.601 | 74.8 | 0.717 |
| Appendicular lean mass | Alzheimer's disease, wide (FinnGen) | rs6739278 | 2:44401055 | C/T | 0.808 | -0.0210 (0.0024) | 1.3E-18 | 0.0019 (0.0247) | 0.939 | 76.6 | -0.090 |
| Appendicular lean mass | Alzheimer's disease, wide (FinnGen) | rs1805165 | 2:88874891 | A/C | 0.720 | -0.0184 (0.0021) | 1.3E-18 | 0.0114 (0.0225) | 0.613 | 76.8 | -0.620 |
| Appendicular lean mass | Alzheimer's disease, wide (FinnGen) | rs6569643 | 6:130312646 | A/G | 0.526 | -0.0167 (0.0019) | 1.4E-18 | 0.0216 (0.0218) | 0.323 | 77.2 | -1.293 |
| Appendicular lean mass | Alzheimer's disease, wide (FinnGen) | rs2052478 | 5:77437291 | T/C | 0.235 | -0.0195 (0.0022) | 1.8E-18 | 0.0125 (0.0276) | 0.651 | 78.6 | -0.641 |
| Appendicular lean mass | Alzheimer's disease, wide (FinnGen) | rs9513510 | 13:99571922 | C/G | 0.699 | -0.0181 (0.0021) | 2.5E-18 | 0.0048 (0.0227) | 0.832 | 74.3 | -0.265 |
| Appendicular lean mass | Alzheimer's disease, wide (FinnGen) | rs7731023 | 5:36181627 | G/A | 0.575 | 0.0166 (0.0019) | 3.5E-18 | -0.0142 (0.0222) | 0.522 | 76.3 | -0.855 |
| Appendicular lean mass | Alzheimer's disease, wide (FinnGen) | rs2531991 | 16:4023553 | A/G | 0.748 | 0.0189 (0.0022) | 5.2E-18 | -0.0210 (0.0240) | 0.383 | 73.8 | -1.111 |
| Appendicular lean mass | Alzheimer's disease, wide (FinnGen) | rs12909863 | 15:75825822 | C/G | 0.251 | 0.0189 (0.0022) | 6.0E-18 | -0.0199 (0.0253) | 0.432 | 73.8 | -1.053 |
| Appendicular lean mass | Alzheimer's disease, wide (FinnGen) | rs12351226 | 9:98405230 | T/C | 0.171 | 0.0218 (0.0025) | 9.1E-18 | 0.0377 (0.0343) | 0.272 | 76.0 | 1.729 |
| Appendicular lean mass | Alzheimer's disease, wide (FinnGen) | rs7512641 | 1:10822171 | A/T | 0.741 | -0.0185 (0.0022) | 9.9E-18 | 0.0415 (0.0231) | 0.072 | 70.7 | -2.243 |
| Appendicular lean mass | Alzheimer's disease, wide (FinnGen) | rs7666804 | 4:57750148 | C/T | 0.389 | -0.0167 (0.0019) | 9.9E-18 | -0.0278 (0.0222) | 0.210 | 77.2 | 1.665 |
| Appendicular lean mass | Alzheimer's disease, wide (FinnGen) | rs395980 | 4:177430072 | G/T | 0.264 | -0.0184 (0.0021) | 1.0E-17 | -0.0160 (0.0239) | 0.501 | 76.8 | 0.870 |
| Appendicular lean mass | Alzheimer's disease, wide (FinnGen) | rs12037677 | 1:109918766 | C/T | 0.718 | -0.0178 (0.0021) | 1.2E-17 | 0.0417 (0.0234) | 0.075 | 71.8 | -2.343 |
| Appendicular lean mass | Alzheimer's disease, wide (FinnGen) | rs73384223 | 9:3869315 | C/T | 0.197 | -0.0205 (0.0024) | 1.3E-17 | 0.0142 (0.0270) | 0.600 | 73.0 | -0.693 |
| Appendicular lean mass | Alzheimer's disease, wide (FinnGen) | rs7598430 | 2:219193963 | T/C | 0.505 | -0.0160 (0.0019) | 1.4E-17 | 0.0202 (0.0214) | 0.345 | 70.9 | -1.262 |
| Appendicular lean mass | Alzheimer's disease, wide (FinnGen) | rs2721940 | 8:116636281 | C/A | 0.602 | -0.0165 (0.0019) | 1.5E-17 | 0.0134 (0.0234) | 0.566 | 75.4 | -0.812 |
| Appendicular lean mass | Alzheimer's disease, wide (FinnGen) | rs9817452 | 3:156795414 | T/G | 0.388 | 0.0165 (0.0019) | 1.9E-17 | 0.0066 (0.0229) | 0.773 | 75.4 | 0.400 |
| Appendicular lean mass | Alzheimer's disease, wide (FinnGen) | rs10922475 | 1:89142142 | A/C | 0.539 | 0.0159 (0.0019) | 2.2E-17 | 0.0393 (0.0218) | 0.072 | 70.0 | 2.472 |
| Appendicular lean mass | Alzheimer's disease, wide (FinnGen) | rs12299065 | 12:117442304 | A/G | 0.127 | -0.0240 (0.0028) | 2.2E-17 | 0.0199 (0.0303) | 0.512 | 73.5 | -0.829 |
| Appendicular lean mass | Alzheimer's disease, wide (FinnGen) | rs56208656 | 19:3575343 | A/G | 0.110 | -0.0284 (0.0034) | 3.0E-17 | 0.0515 (0.0332) | 0.120 | 69.8 | -1.813 |
| Appendicular lean mass | Alzheimer's disease, wide (FinnGen) | rs10040039 | 5:88354597 | G/A | 0.387 | -0.0163 (0.0019) | 3.3E-17 | -0.0059 (0.0231) | 0.799 | 73.6 | 0.362 |
| Appendicular lean mass | Alzheimer's disease, wide (FinnGen) | rs59951000 | 18:74975828 | T/C | 0.042 | -0.0400 (0.0047) | 3.3E-17 | 0.0718 (0.0395) | 0.069 | 72.4 | -1.795 |
| Appendicular lean mass | Alzheimer's disease, wide (FinnGen) | rs201570119 | 2:112256320 | C/T | 0.210 | 0.0194 (0.0023) | 5.5E-17 | 0.0206 (0.0285) | 0.470 | 71.2 | 1.062 |
| Appendicular lean mass | Alzheimer's disease, wide (FinnGen) | rs2347603 | 2:47297426 | A/T | 0.742 | -0.0181 (0.0022) | 5.6E-17 | -0.0064 (0.0240) | 0.791 | 67.7 | 0.354 |
| Appendicular lean mass | Alzheimer's disease, wide (FinnGen) | rs1341215 | 9:111662350 | A/G | 0.137 | 0.0229 (0.0027) | 6.3E-17 | -0.0455 (0.0275) | 0.099 | 71.9 | -1.987 |
| Appendicular lean mass | Alzheimer's disease, wide (FinnGen) | rs10005035 | 4:12865684 | G/C | 0.282 | -0.0175 (0.0021) | 6.5E-17 | -0.0095 (0.0246) | 0.698 | 69.4 | 0.543 |
| Appendicular lean mass | Alzheimer's disease, wide (FinnGen) | rs1556659 | 10:130834698 | T/C | 0.382 | 0.0163 (0.0020) | 7.2E-17 | -0.0001 (0.0233) | 0.996 | 66.4 | -0.006 |
| Appendicular lean mass | Alzheimer's disease, wide (FinnGen) | rs10776560 | 10:50542358 | T/C | 0.499 | -0.0157 (0.0019) | 7.9E-17 | 0.0121 (0.0220) | 0.583 | 68.3 | -0.771 |
| Appendicular lean mass | Alzheimer's disease, wide (FinnGen) | rs10514518 | 16:81579827 | T/C | 0.685 | 0.0171 (0.0020) | 8.3E-17 | 0.0011 (0.0234) | 0.963 | 73.1 | 0.064 |
| Appendicular lean mass | Alzheimer's disease, wide (FinnGen) | rs112898929 | 16:88298795 | A/T | 0.080 | -0.0296 (0.0036) | 9.6E-17 | 0.0198 (0.0351) | 0.572 | 67.6 | -0.669 |
| Appendicular lean mass | Alzheimer's disease, wide (FinnGen) | rs9784904 | 7:55835078 | C/T | 0.155 | -0.0217 (0.0026) | 9.7E-17 | 0.0303 (0.0251) | 0.227 | 69.7 | -1.396 |
| Appendicular lean mass | Alzheimer's disease, wide (FinnGen) | rs1063582 | 8:23167353 | G/T | 0.765 | -0.0185 (0.0022) | 1.1E-16 | -0.0016 (0.0258) | 0.949 | 70.7 | 0.086 |
| Appendicular lean mass | Alzheimer's disease, wide (FinnGen) | rs10823149 | 10:69935396 | T/G | 0.548 | 0.0157 (0.0019) | 1.2E-16 | -0.0249 (0.0215) | 0.246 | 68.3 | -1.586 |
| Appendicular lean mass | Alzheimer's disease, wide (FinnGen) | rs4815952 | 20:6934897 | C/T | 0.522 | -0.0161 (0.0019) | 1.2E-16 | 0.0154 (0.0214) | 0.474 | 71.8 | -0.957 |
| Appendicular lean mass | Alzheimer's disease, wide (FinnGen) | rs9809116 | 3:72397279 | G/A | 0.408 | -0.0160 (0.0019) | 1.3E-16 | -0.0043 (0.0226) | 0.848 | 70.9 | 0.269 |
| Appendicular lean mass | Alzheimer's disease, wide (FinnGen) | rs35166681 | 6:85369727 | TA/T | 0.159 | 0.0214 (0.0026) | 1.5E-16 | 0.0265 (0.0303) | 0.381 | 67.8 | 1.238 |
| Appendicular lean mass | Alzheimer's disease, wide (FinnGen) | rs12512942 | 4:177766307 | A/G | 0.638 | -0.0162 (0.0020) | 1.6E-16 | -0.0437 (0.0215) | 0.042 | 65.6 | 2.698 |
| Appendicular lean mass | Alzheimer's disease, wide (FinnGen) | rs10843391 | 12:29508642 | G/A | 0.285 | 0.0172 (0.0021) | 2.1E-16 | 0.0252 (0.0233) | 0.278 | 67.1 | 1.465 |
| Appendicular lean mass | Alzheimer's disease, wide (FinnGen) | rs9391254 | 6:105377347 | T/C | 0.321 | 0.0166 (0.0020) | 2.1E-16 | 0.0375 (0.0228) | 0.101 | 68.9 | 2.259 |
| Appendicular lean mass | Alzheimer's disease, wide (FinnGen) | rs71635721 | 3:171960170 | G/C | 0.065 | 0.0316 (0.0039) | 3.5E-16 | -0.0482 (0.0511) | 0.346 | 65.7 | -1.525 |
| Appendicular lean mass | Alzheimer's disease, wide (FinnGen) | rs56363908 | 7:96611052 | G/A | 0.042 | -0.0382 (0.0047) | 3.9E-16 | -0.0798 (0.0584) | 0.171 | 66.1 | 2.089 |
| Appendicular lean mass | Alzheimer's disease, wide (FinnGen) | rs9479012 | 6:151657994 | G/A | 0.094 | 0.0263 (0.0032) | 4.1E-16 | -0.0020 (0.0370) | 0.958 | 67.5 | -0.076 |
| Appendicular lean mass | Alzheimer's disease, wide (FinnGen) | rs6543146 | 2:103096695 | G/T | 0.558 | 0.0154 (0.0019) | 4.2E-16 | -0.0111 (0.0238) | 0.642 | 65.7 | -0.721 |
| Appendicular lean mass | Alzheimer's disease, wide (FinnGen) | rs17138358 | 7:17920253 | C/G | 0.398 | 0.0157 (0.0019) | 4.4E-16 | -0.0420 (0.0231) | 0.069 | 68.3 | -2.675 |
| Appendicular lean mass | Alzheimer's disease, wide (FinnGen) | rs7367519 | 1:204479176 | C/T | 0.685 | 0.0164 (0.0020) | 4.7E-16 | -0.0471 (0.0258) | 0.068 | 67.2 | -2.872 |
| Appendicular lean mass | Alzheimer's disease, wide (FinnGen) | rs61729527 | 8:77761919 | T/C | 0.052 | -0.0346 (0.0043) | 4.9E-16 | -0.0182 (0.0430) | 0.672 | 64.8 | 0.526 |
| Appendicular lean mass | Alzheimer's disease, wide (FinnGen) | rs10208668 | 2:20556151 | C/G | 0.035 | 0.0415 (0.0051) | 5.0E-16 | -0.0130 (0.0752) | 0.862 | 66.2 | -0.313 |
| Appendicular lean mass | Alzheimer's disease, wide (FinnGen) | rs2287821 | 19:33935102 | T/C | 0.509 | -0.0153 (0.0019) | 7.0E-16 | -0.0359 (0.0212) | 0.091 | 64.8 | 2.346 |
| Appendicular lean mass | Alzheimer's disease, wide (FinnGen) | rs35963161 | 20:49210635 | A/G | 0.476 | -0.0157 (0.0019) | 7.5E-16 | 0.0460 (0.0214) | 0.032 | 68.3 | -2.930 |
| Appendicular lean mass | Alzheimer's disease, wide (FinnGen) | rs1056747 | 9:35690102 | G/A | 0.412 | -0.0155 (0.0019) | 8.0E-16 | -0.0100 (0.0215) | 0.643 | 66.5 | 0.645 |
| Appendicular lean mass | Alzheimer's disease, wide (FinnGen) | rs11777835 | 8:144986103 | C/T | 0.473 | -0.0152 (0.0019) | 9.6E-16 | 0.0508 (0.0213) | 0.017 | 64.0 | -3.342 |
| Appendicular lean mass | Alzheimer's disease, wide (FinnGen) | rs2165772 | 11:27091907 | A/G | 0.354 | 0.0160 (0.0020) | 1.0E-15 | -0.0151 (0.0220) | 0.491 | 64.0 | -0.944 |
| Appendicular lean mass | Alzheimer's disease, wide (FinnGen) | rs2596144 | 12:122297738 | C/T | 0.870 | -0.0223 (0.0028) | 1.5E-15 | -0.0087 (0.0256) | 0.734 | 63.4 | 0.390 |
| Appendicular lean mass | Alzheimer's disease, wide (FinnGen) | rs8904 | 14:35871217 | A/G | 0.363 | -0.0157 (0.0020) | 1.5E-15 | 0.0303 (0.0218) | 0.165 | 61.6 | -1.930 |
| Appendicular lean mass | Alzheimer's disease, wide (FinnGen) | rs4504126 | 3:33600582 | C/A | 0.028 | 0.0460 (0.0058) | 1.6E-15 | 0.0133 (0.0852) | 0.876 | 62.9 | 0.289 |
| Appendicular lean mass | Alzheimer's disease, wide (FinnGen) | rs2007022 | 20:3237674 | A/C | 0.232 | 0.0179 (0.0022) | 1.6E-15 | 0.0067 (0.0294) | 0.820 | 66.2 | 0.374 |
| Appendicular lean mass | Alzheimer's disease, wide (FinnGen) | rs2324154 | 4:24027226 | A/C | 0.510 | 0.0150 (0.0019) | 1.9E-15 | 0.0096 (0.0213) | 0.654 | 62.3 | 0.640 |
| Appendicular lean mass | Alzheimer's disease, wide (FinnGen) | rs704660 | 11:30447998 | T/C | 0.409 | 0.0153 (0.0019) | 2.3E-15 | 0.0110 (0.0216) | 0.611 | 64.8 | 0.719 |
| Appendicular lean mass | Alzheimer's disease, wide (FinnGen) | rs4076108 | 3:13736088 | T/A | 0.245 | 0.0174 (0.0022) | 2.3E-15 | 0.0105 (0.0239) | 0.660 | 62.5 | 0.603 |
| Appendicular lean mass | Alzheimer's disease, wide (FinnGen) | rs7646501 | 3:24079291 | G/A | 0.264 | -0.0170 (0.0021) | 2.3E-15 | -0.0281 (0.0233) | 0.227 | 65.5 | 1.653 |
| Appendicular lean mass | Alzheimer's disease, wide (FinnGen) | rs16844417 | 1:172377745 | A/G | 0.124 | 0.0226 (0.0029) | 2.6E-15 | 0.0074 (0.0313) | 0.813 | 60.7 | 0.327 |
| Appendicular lean mass | Alzheimer's disease, wide (FinnGen) | rs12371664 | 12:91130760 | G/A | 0.272 | -0.0168 (0.0021) | 2.7E-15 | 0.0406 (0.0249) | 0.103 | 64.0 | -2.417 |
| Appendicular lean mass | Alzheimer's disease, wide (FinnGen) | rs3121580 | 1:218492121 | C/T | 0.827 | 0.0197 (0.0025) | 2.7E-15 | -0.0339 (0.0269) | 0.207 | 62.1 | -1.721 |
| Appendicular lean mass | Alzheimer's disease, wide (FinnGen) | rs7516643 | 1:33873010 | C/T | 0.672 | 0.0157 (0.0020) | 3.1E-15 | 0.0162 (0.0225) | 0.471 | 61.6 | 1.032 |
| Appendicular lean mass | Alzheimer's disease, wide (FinnGen) | rs7228151 | 18:57181694 | C/T | 0.206 | -0.0185 (0.0023) | 3.2E-15 | 0.0196 (0.0240) | 0.415 | 64.7 | -1.059 |
| Appendicular lean mass | Alzheimer's disease, wide (FinnGen) | rs35436119 | 9:119303347 | A/G | 0.075 | 0.0282 (0.0036) | 3.3E-15 | 0.0277 (0.0617) | 0.654 | 61.4 | 0.982 |
| Appendicular lean mass | Alzheimer's disease, wide (FinnGen) | rs4903321 | 14:75911083 | A/G | 0.444 | -0.0151 (0.0019) | 3.4E-15 | 0.0248 (0.0213) | 0.243 | 63.2 | -1.642 |
| Appendicular lean mass | Alzheimer's disease, wide (FinnGen) | rs11991823 | 8:92188440 | G/A | 0.679 | 0.0161 (0.0020) | 3.5E-15 | 0.0121 (0.0220) | 0.584 | 64.8 | 0.752 |
| Appendicular lean mass | Alzheimer's disease, wide (FinnGen) | rs72829852 | 17:46633974 | T/C | 0.062 | 0.0309 (0.0039) | 3.7E-15 | 0.0402 (0.0424) | 0.343 | 62.8 | 1.301 |
| Appendicular lean mass | Alzheimer's disease, wide (FinnGen) | rs13170063 | 5:157895013 | A/G | 0.592 | -0.0152 (0.0019) | 4.1E-15 | 0.0241 (0.0224) | 0.282 | 64.0 | -1.586 |
| Appendicular lean mass | Alzheimer's disease, wide (FinnGen) | rs10822117 | 10:52786701 | G/A | 0.237 | -0.0176 (0.0022) | 4.2E-15 | -0.0045 (0.0244) | 0.853 | 64.0 | 0.256 |
| Appendicular lean mass | Alzheimer's disease, wide (FinnGen) | rs7761910 | 6:155553832 | T/C | 0.309 | -0.0160 (0.0020) | 5.1E-15 | 0.0055 (0.0227) | 0.808 | 64.0 | -0.344 |
| Appendicular lean mass | Alzheimer's disease, wide (FinnGen) | rs10796828 | 11:69490346 | G/T | 0.635 | 0.0154 (0.0020) | 5.8E-15 | 0.0371 (0.0244) | 0.128 | 59.3 | 2.409 |
| Appendicular lean mass | Alzheimer's disease, wide (FinnGen) | rs28817902 | 5:138916010 | G/A | 0.118 | 0.0230 (0.0029) | 6.2E-15 | 0.0289 (0.0428) | 0.500 | 62.9 | 1.257 |
| Appendicular lean mass | Alzheimer's disease, wide (FinnGen) | rs11021305 | 11:95498269 | C/T | 0.384 | 0.0152 (0.0019) | 6.3E-15 | -0.0079 (0.0244) | 0.745 | 64.0 | -0.520 |
| Appendicular lean mass | Alzheimer's disease, wide (FinnGen) | rs34786000 | 19:9942123 | T/G | 0.554 | -0.0150 (0.0019) | 6.6E-15 | -0.0237 (0.0214) | 0.268 | 62.3 | 1.580 |
| Appendicular lean mass | Alzheimer's disease, wide (FinnGen) | rs62501195 | 8:24041988 | C/A | 0.171 | -0.0198 (0.0025) | 8.1E-15 | 0.0033 (0.0252) | 0.897 | 62.7 | -0.167 |
| Appendicular lean mass | Alzheimer's disease, wide (FinnGen) | rs4776624 | 15:73359881 | T/C | 0.397 | -0.0150 (0.0019) | 1.2E-14 | -0.0328 (0.0227) | 0.149 | 62.3 | 2.187 |
| Appendicular lean mass | Alzheimer's disease, wide (FinnGen) | rs7320878 | 13:91994132 | A/G | 0.603 | -0.0150 (0.0019) | 1.3E-14 | -0.0022 (0.0212) | 0.918 | 62.3 | 0.147 |
| Appendicular lean mass | Alzheimer's disease, wide (FinnGen) | rs9910161 | 17:59606244 | G/A | 0.285 | 0.0162 (0.0021) | 1.6E-14 | -0.0483 (0.0241) | 0.045 | 59.5 | -2.981 |
| Appendicular lean mass | Alzheimer's disease, wide (FinnGen) | rs1035583 | 2:207326937 | A/G | 0.618 | 0.0148 (0.0019) | 2.0E-14 | -0.0226 (0.0213) | 0.288 | 60.7 | -1.527 |
| Appendicular lean mass | Alzheimer's disease, wide (FinnGen) | rs9394578 | 6:39165859 | A/C | 0.258 | -0.0164 (0.0022) | 2.6E-14 | 0.0181 (0.0270) | 0.502 | 55.6 | -1.104 |
| Appendicular lean mass | Alzheimer's disease, wide (FinnGen) | rs73382475 | 21:47549715 | T/C | 0.089 | 0.0254 (0.0033) | 3.0E-14 | -0.0399 (0.0380) | 0.295 | 59.2 | -1.571 |
| Appendicular lean mass | Alzheimer's disease, wide (FinnGen) | rs6721191 | 2:10190115 | G/A | 0.578 | -0.0144 (0.0019) | 3.2E-14 | 0.0019 (0.0239) | 0.936 | 57.4 | -0.132 |
| Appendicular lean mass | Alzheimer's disease, wide (FinnGen) | rs2609334 | 6:168847175 | C/T | 0.226 | -0.0171 (0.0022) | 3.4E-14 | -0.0243 (0.0228) | 0.286 | 60.4 | 1.421 |
| Appendicular lean mass | Alzheimer's disease, wide (FinnGen) | rs9525326 | 13:115075715 | G/A | 0.188 | -0.0184 (0.0024) | 3.6E-14 | -0.0048 (0.0270) | 0.859 | 58.8 | 0.261 |
| Appendicular lean mass | Alzheimer's disease, wide (FinnGen) | rs11673381 | 19:4506296 | G/A | 0.530 | -0.0144 (0.0019) | 3.7E-14 | -0.0161 (0.0213) | 0.450 | 57.4 | 1.118 |
| Appendicular lean mass | Alzheimer's disease, wide (FinnGen) | rs74379684 | 15:94050205 | T/C | 0.075 | -0.0272 (0.0036) | 4.4E-14 | -0.0269 (0.0375) | 0.473 | 57.1 | 0.989 |
| Appendicular lean mass | Alzheimer's disease, wide (FinnGen) | rs12662115 | 6:169360124 | G/A | 0.327 | -0.0152 (0.0020) | 4.5E-14 | -0.0119 (0.0216) | 0.582 | 57.8 | 0.783 |
| Appendicular lean mass | Alzheimer's disease, wide (FinnGen) | rs165849 | 22:19958669 | A/G | 0.698 | 0.0157 (0.0021) | 4.6E-14 | 0.0192 (0.0226) | 0.396 | 55.9 | 1.223 |
| Appendicular lean mass | Alzheimer's disease, wide (FinnGen) | rs11198591 | 10:120515892 | A/G | 0.368 | 0.0148 (0.0020) | 4.9E-14 | -0.0168 (0.0221) | 0.448 | 54.8 | -1.135 |
| Appendicular lean mass | Alzheimer's disease, wide (FinnGen) | rs7286917 | 22:39860868 | G/A | 0.746 | 0.0171 (0.0023) | 5.2E-14 | 0.0190 (0.0242) | 0.434 | 55.3 | 1.111 |
| Appendicular lean mass | Alzheimer's disease, wide (FinnGen) | rs1557341 | 18:35127427 | C/A | 0.667 | 0.0153 (0.0020) | 5.8E-14 | 0.0275 (0.0238) | 0.246 | 58.5 | 1.797 |
| Appendicular lean mass | Alzheimer's disease, wide (FinnGen) | rs599004 | 6:140439740 | T/C | 0.281 | -0.0157 (0.0021) | 6.5E-14 | 0.0013 (0.0259) | 0.960 | 55.9 | -0.083 |
| Appendicular lean mass | Alzheimer's disease, wide (FinnGen) | rs1290786 | 3:169097381 | T/C | 0.431 | -0.0143 (0.0019) | 7.1E-14 | -0.0132 (0.0216) | 0.542 | 56.6 | 0.923 |
| Appendicular lean mass | Alzheimer's disease, wide (FinnGen) | rs4622329 | 12:102321935 | A/G | 0.350 | 0.0149 (0.0020) | 8.6E-14 | 0.0210 (0.0215) | 0.328 | 55.5 | 1.409 |
| Appendicular lean mass | Alzheimer's disease, wide (FinnGen) | rs73040028 | 3:23171805 | T/C | 0.229 | 0.0168 (0.0022) | 9.0E-14 | 0.0013 (0.0242) | 0.958 | 58.3 | 0.077 |
| Appendicular lean mass | Alzheimer's disease, wide (FinnGen) | rs10810474 | 9:15911359 | T/C | 0.418 | 0.0143 (0.0019) | 9.6E-14 | 0.0048 (0.0213) | 0.821 | 56.6 | 0.336 |
| Appendicular lean mass | Alzheimer's disease, wide (FinnGen) | rs2232460 | 1:6659505 | A/G | 0.334 | 0.0148 (0.0020) | 1.0E-13 | 0.0075 (0.0237) | 0.751 | 54.8 | 0.507 |
| Appendicular lean mass | Alzheimer's disease, wide (FinnGen) | rs3760456 | 17:27948844 | T/C | 0.440 | -0.0142 (0.0019) | 1.1E-13 | -0.0074 (0.0212) | 0.726 | 55.9 | 0.521 |
| Appendicular lean mass | Alzheimer's disease, wide (FinnGen) | rs2807339 | 1:22578063 | C/T | 0.759 | 0.0162 (0.0022) | 1.2E-13 | -0.0101 (0.0254) | 0.690 | 54.2 | -0.623 |
| Appendicular lean mass | Alzheimer's disease, wide (FinnGen) | rs3798429 | 6:76604573 | G/T | 0.498 | 0.0140 (0.0019) | 1.3E-13 | 0.0095 (0.0213) | 0.655 | 54.3 | 0.679 |
| Appendicular lean mass | Alzheimer's disease, wide (FinnGen) | rs78051210 | 6:131379491 | C/T | 0.077 | 0.0263 (0.0036) | 1.6E-13 | 0.0338 (0.0518) | 0.514 | 53.4 | 1.285 |
| Appendicular lean mass | Alzheimer's disease, wide (FinnGen) | rs11927331 | 3:32937008 | A/C | 0.331 | -0.0147 (0.0020) | 1.9E-13 | -0.0113 (0.0222) | 0.611 | 54.0 | 0.769 |
| Appendicular lean mass | Alzheimer's disease, wide (FinnGen) | rs17400325 | 2:178565913 | C/T | 0.042 | 0.0345 (0.0047) | 2.1E-13 | -0.0909 (0.0746) | 0.223 | 53.9 | -2.635 |
| Appendicular lean mass | Alzheimer's disease, wide (FinnGen) | rs12344515 | 9:113801231 | T/C | 0.240 | -0.0163 (0.0022) | 2.3E-13 | -0.0113 (0.0261) | 0.665 | 54.9 | 0.693 |
| Appendicular lean mass | Alzheimer's disease, wide (FinnGen) | rs7225068 | 17:60666425 | G/A | 0.591 | 0.0141 (0.0019) | 2.5E-13 | 0.0090 (0.0215) | 0.674 | 55.1 | 0.638 |
| Appendicular lean mass | Alzheimer's disease, wide (FinnGen) | rs35631698 | 21:16549469 | GC/G | 0.375 | 0.0145 (0.0020) | 2.6E-13 | -0.0092 (0.0214) | 0.668 | 52.6 | -0.634 |
| Appendicular lean mass | Alzheimer's disease, wide (FinnGen) | rs2138374 | 2:190014317 | C/T | 0.696 | -0.0149 (0.0020) | 2.8E-13 | 0.0239 (0.0224) | 0.286 | 55.5 | -1.604 |
| Appendicular lean mass | Alzheimer's disease, wide (FinnGen) | rs35268848 | 16:67927240 | A/C | 0.012 | 0.0737 (0.0101) | 2.8E-13 | -0.0062 (0.0732) | 0.932 | 53.2 | -0.084 |
| Appendicular lean mass | Alzheimer's disease, wide (FinnGen) | rs147233090 | 15:44028047 | T/C | 0.025 | -0.0446 (0.0061) | 4.0E-13 | -0.0330 (0.1512) | 0.827 | 53.5 | 0.740 |
| Appendicular lean mass | Alzheimer's disease, wide (FinnGen) | rs4733775 | 8:131213570 | A/G | 0.364 | 0.0143 (0.0020) | 4.1E-13 | -0.0114 (0.0224) | 0.610 | 51.1 | -0.797 |
| Appendicular lean mass | Alzheimer's disease, wide (FinnGen) | rs610694 | 12:121304826 | C/T | 0.490 | 0.0136 (0.0019) | 4.3E-13 | -0.0012 (0.0213) | 0.955 | 51.2 | -0.088 |
| Appendicular lean mass | Alzheimer's disease, wide (FinnGen) | rs5753518 | 22:31631314 | A/G | 0.090 | 0.0242 (0.0033) | 5.0E-13 | -0.0083 (0.0308) | 0.788 | 53.8 | -0.343 |
| Appendicular lean mass | Alzheimer's disease, wide (FinnGen) | rs72976986 | 19:4050424 | A/G | 0.190 | 0.0176 (0.0024) | 5.3E-13 | 0.0307 (0.0290) | 0.290 | 53.8 | 1.744 |
| Appendicular lean mass | Alzheimer's disease, wide (FinnGen) | rs56207248 | 2:25065986 | T/C | 0.066 | -0.0273 (0.0038) | 6.0E-13 | -0.0419 (0.0448) | 0.349 | 51.6 | 1.535 |
| Appendicular lean mass | Alzheimer's disease, wide (FinnGen) | rs1487441 | 6:98553894 | A/G | 0.484 | 0.0136 (0.0019) | 7.5E-13 | 0.0146 (0.0212) | 0.491 | 51.2 | 1.074 |
| Appendicular lean mass | Alzheimer's disease, wide (FinnGen) | rs1216743 | 11:100573120 | A/G | 0.719 | -0.0152 (0.0021) | 8.0E-13 | 0.0306 (0.0237) | 0.198 | 52.4 | -2.013 |
| Appendicular lean mass | Alzheimer's disease, wide (FinnGen) | rs6975015 | 7:50617215 | A/G | 0.117 | 0.0211 (0.0029) | 8.0E-13 | -0.0209 (0.0365) | 0.567 | 52.9 | -0.991 |
| Appendicular lean mass | Alzheimer's disease, wide (FinnGen) | rs10453441 | 22:46363739 | G/A | 0.402 | -0.0139 (0.0020) | 9.1E-13 | 0.0474 (0.0227) | 0.037 | 48.3 | -3.410 |
| Appendicular lean mass | Alzheimer's disease, wide (FinnGen) | rs1330826 | 9:85129970 | C/G | 0.227 | 0.0162 (0.0023) | 1.0E-12 | 0.0312 (0.0245) | 0.203 | 49.6 | 1.926 |
| Appendicular lean mass | Alzheimer's disease, wide (FinnGen) | rs55758152 | 5:171317318 | A/G | 0.326 | 0.0145 (0.0020) | 1.1E-12 | -0.0030 (0.0225) | 0.893 | 52.6 | -0.207 |
| Appendicular lean mass | Alzheimer's disease, wide (FinnGen) | rs143076454 | 16:921179 | A/G | 0.019 | -0.0499 (0.0070) | 1.1E-12 | -0.0564 (0.0914) | 0.537 | 50.8 | 1.130 |
| Appendicular lean mass | Alzheimer's disease, wide (FinnGen) | rs60408354 | 7:70158495 | A/G | 0.073 | 0.0259 (0.0036) | 1.2E-12 | -0.0422 (0.0394) | 0.283 | 51.8 | -1.629 |
| Appendicular lean mass | Alzheimer's disease, wide (FinnGen) | rs853168 | 5:142538764 | C/G | 0.247 | 0.0156 (0.0022) | 1.3E-12 | -0.0209 (0.0265) | 0.430 | 50.3 | -1.340 |
| Appendicular lean mass | Alzheimer's disease, wide (FinnGen) | rs72771070 | 16:19993750 | T/C | 0.283 | 0.0150 (0.0021) | 1.3E-12 | 0.0051 (0.0225) | 0.820 | 51.0 | 0.340 |
| Appendicular lean mass | Alzheimer's disease, wide (FinnGen) | rs12672217 | 7:156310948 | A/G | 0.361 | 0.0139 (0.0020) | 1.3E-12 | 0.0187 (0.0214) | 0.383 | 48.3 | 1.345 |
| Appendicular lean mass | Alzheimer's disease, wide (FinnGen) | rs2578565 | 5:5460569 | T/C | 0.657 | -0.0141 (0.0020) | 1.4E-12 | -0.0153 (0.0217) | 0.481 | 49.7 | 1.085 |
| Appendicular lean mass | Alzheimer's disease, wide (FinnGen) | rs572474 | 10:89791654 | G/A | 0.166 | 0.0180 (0.0025) | 1.5E-12 | 0.0107 (0.0284) | 0.708 | 51.8 | 0.594 |
| Appendicular lean mass | Alzheimer's disease, wide (FinnGen) | rs17197114 | 14:21894526 | C/T | 0.177 | 0.0177 (0.0025) | 1.5E-12 | -0.0853 (0.0297) | 0.004 | 50.1 | -4.819 |
| Appendicular lean mass | Alzheimer's disease, wide (FinnGen) | rs2289976 | 2:85818797 | A/G | 0.305 | 0.0144 (0.0020) | 1.6E-12 | -0.0509 (0.0251) | 0.042 | 51.8 | -3.535 |
| Appendicular lean mass | Alzheimer's disease, wide (FinnGen) | rs4847378 | 1:93324634 | T/G | 0.612 | 0.0136 (0.0019) | 1.6E-12 | -0.0218 (0.0217) | 0.317 | 51.2 | -1.603 |
| Appendicular lean mass | Alzheimer's disease, wide (FinnGen) | rs73114154 | 20:31449386 | T/C | 0.388 | 0.0138 (0.0020) | 1.7E-12 | 0.0280 (0.0214) | 0.191 | 47.6 | 2.029 |
| Appendicular lean mass | Alzheimer's disease, wide (FinnGen) | rs7107356 | 11:47676170 | G/A | 0.507 | 0.0133 (0.0019) | 1.9E-12 | 0.0115 (0.0218) | 0.597 | 49.0 | 0.865 |
| Appendicular lean mass | Alzheimer's disease, wide (FinnGen) | rs6129229 | 20:37882317 | G/A | 0.075 | -0.0257 (0.0036) | 1.9E-12 | 0.0392 (0.0396) | 0.323 | 51.0 | -1.525 |
| Appendicular lean mass | Alzheimer's disease, wide (FinnGen) | rs177592 | 7:28559785 | C/G | 0.101 | -0.0220 (0.0031) | 2.0E-12 | -0.0167 (0.0391) | 0.669 | 50.4 | 0.759 |
| Appendicular lean mass | Alzheimer's disease, wide (FinnGen) | rs2607234 | 19:35563834 | G/A | 0.948 | -0.0302 (0.0043) | 2.0E-12 | -0.0032 (0.0459) | 0.944 | 49.3 | 0.106 |
| Appendicular lean mass | Alzheimer's disease, wide (FinnGen) | rs1977337 | 1:67419517 | C/T | 0.851 | 0.0186 (0.0026) | 2.1E-12 | -0.0110 (0.0255) | 0.666 | 51.2 | -0.591 |
| Appendicular lean mass | Alzheimer's disease, wide (FinnGen) | rs6693481 | 1:203766395 | C/T | 0.695 | -0.0143 (0.0020) | 2.2E-12 | -0.0272 (0.0222) | 0.220 | 51.1 | 1.902 |
| Appendicular lean mass | Alzheimer's disease, wide (FinnGen) | rs7574162 | 2:172133086 | T/C | 0.757 | 0.0153 (0.0022) | 2.4E-12 | -0.0302 (0.0256) | 0.238 | 48.4 | -1.974 |
| Appendicular lean mass | Alzheimer's disease, wide (FinnGen) | rs310796 | 12:77453226 | T/G | 0.681 | 0.0142 (0.0020) | 2.5E-12 | 0.0200 (0.0241) | 0.406 | 50.4 | 1.408 |
| Appendicular lean mass | Alzheimer's disease, wide (FinnGen) | rs11720869 | 3:185619716 | A/G | 0.669 | 0.0141 (0.0020) | 2.5E-12 | -0.0098 (0.0215) | 0.648 | 49.7 | -0.695 |
| Appendicular lean mass | Alzheimer's disease, wide (FinnGen) | rs1717720 | 7:38112615 | G/A | 0.654 | 0.0139 (0.0020) | 2.7E-12 | -0.0173 (0.0241) | 0.472 | 48.3 | -1.245 |
| Appendicular lean mass | Alzheimer's disease, wide (FinnGen) | rs75022676 | 2:60293216 | A/G | 0.208 | -0.0163 (0.0023) | 2.8E-12 | -0.0266 (0.0242) | 0.272 | 50.2 | 1.632 |
| Appendicular lean mass | Alzheimer's disease, wide (FinnGen) | rs76488803 | 4:157770271 | A/G | 0.083 | -0.0240 (0.0034) | 3.0E-12 | -0.0129 (0.0474) | 0.785 | 49.8 | 0.537 |
| Appendicular lean mass | Alzheimer's disease, wide (FinnGen) | rs7692387 | 4:156635309 | A/G | 0.185 | 0.0169 (0.0024) | 3.5E-12 | 0.0136 (0.0248) | 0.583 | 49.6 | 0.805 |
| Appendicular lean mass | Alzheimer's disease, wide (FinnGen) | rs112873218 | 11:1960119 | T/C | 0.105 | 0.0216 (0.0031) | 4.0E-12 | -0.0065 (0.0332) | 0.845 | 48.5 | -0.301 |
| Appendicular lean mass | Alzheimer's disease, wide (FinnGen) | rs7259285 | 19:17153149 | A/G | 0.444 | -0.0132 (0.0019) | 5.1E-12 | -0.0461 (0.0212) | 0.030 | 48.3 | 3.492 |
| Appendicular lean mass | Alzheimer's disease, wide (FinnGen) | rs36052389 | 4:39357183 | AT/A | 0.455 | 0.0132 (0.0019) | 5.3E-12 | 0.0307 (0.0217) | 0.158 | 48.3 | 2.326 |
| Appendicular lean mass | Alzheimer's disease, wide (FinnGen) | rs9610447 | 22:20768891 | T/C | 0.747 | 0.0152 (0.0022) | 5.3E-12 | 0.0287 (0.0255) | 0.261 | 47.7 | 1.888 |
| Appendicular lean mass | Alzheimer's disease, wide (FinnGen) | rs2925155 | 8:75886297 | T/C | 0.261 | -0.0150 (0.0022) | 5.5E-12 | -0.0210 (0.0239) | 0.378 | 46.5 | 1.400 |
| Appendicular lean mass | Alzheimer's disease, wide (FinnGen) | rs17681189 | 2:65976175 | A/C | 0.423 | -0.0131 (0.0019) | 5.8E-12 | 0.0149 (0.0214) | 0.485 | 47.5 | -1.137 |
| Appendicular lean mass | Alzheimer's disease, wide (FinnGen) | rs7679276 | 4:146860186 | G/A | 0.954 | -0.0330 (0.0048) | 5.9E-12 | -0.1167 (0.0925) | 0.207 | 47.3 | 3.536 |
| Appendicular lean mass | Alzheimer's disease, wide (FinnGen) | rs75478182 | 20:50807839 | C/G | 0.088 | 0.0230 (0.0033) | 6.6E-12 | -0.0818 (0.0424) | 0.054 | 48.6 | -3.557 |
| Appendicular lean mass | Alzheimer's disease, wide (FinnGen) | rs2343034 | 20:35860288 | A/T | 0.809 | 0.0166 (0.0024) | 6.7E-12 | 0.0429 (0.0231) | 0.064 | 47.8 | 2.584 |
| Appendicular lean mass | Alzheimer's disease, wide (FinnGen) | rs10427685 | 22:46440359 | G/C | 0.064 | 0.0269 (0.0039) | 7.0E-12 | -0.0288 (0.0455) | 0.526 | 47.6 | -1.071 |
| Appendicular lean mass | Alzheimer's disease, wide (FinnGen) | rs12463908 | 2:121470971 | C/A | 0.188 | -0.0165 (0.0024) | 7.3E-12 | -0.0508 (0.0264) | 0.055 | 47.3 | 3.079 |
| Appendicular lean mass | Alzheimer's disease, wide (FinnGen) | rs10008637 | 4:77414144 | C/T | 0.462 | -0.0130 (0.0019) | 7.5E-12 | 0.0041 (0.0213) | 0.848 | 46.8 | -0.315 |
| Appendicular lean mass | Alzheimer's disease, wide (FinnGen) | rs2125125 | 3:142600087 | C/T | 0.783 | 0.0158 (0.0023) | 7.8E-12 | 0.0246 (0.0294) | 0.402 | 47.2 | 1.557 |
| Appendicular lean mass | Alzheimer's disease, wide (FinnGen) | rs61827272 | 1:203810763 | C/T | 0.275 | 0.0144 (0.0021) | 7.9E-12 | 0.0070 (0.0231) | 0.762 | 47.0 | 0.486 |
| Appendicular lean mass | Alzheimer's disease, wide (FinnGen) | rs78444298 | 1:184672098 | A/G | 0.020 | -0.0465 (0.0068) | 8.3E-12 | -0.0995 (0.0823) | 0.227 | 46.8 | 2.140 |
| Appendicular lean mass | Alzheimer's disease, wide (FinnGen) | rs116493405 | 3:114733556 | A/G | 0.054 | 0.0287 (0.0042) | 9.5E-12 | 0.1192 (0.0631) | 0.059 | 46.7 | 4.153 |
| Appendicular lean mass | Alzheimer's disease, wide (FinnGen) | rs61919240 | 12:8831954 | A/T | 0.324 | 0.0137 (0.0020) | 9.8E-12 | 0.0625 (0.0233) | 0.007 | 46.9 | 4.562 |
| Appendicular lean mass | Alzheimer's disease, wide (FinnGen) | rs963317 | 4:45129970 | G/A | 0.663 | -0.0136 (0.0020) | 1.0E-11 | -0.0005 (0.0228) | 0.984 | 46.2 | 0.037 |
| Appendicular lean mass | Alzheimer's disease, wide (FinnGen) | rs945508 | 1:156907081 | C/T | 0.536 | -0.0128 (0.0019) | 1.1E-11 | 0.0025 (0.0213) | 0.906 | 45.4 | -0.195 |
| Appendicular lean mass | Alzheimer's disease, wide (FinnGen) | rs67625472 | 19:4968620 | C/T | 0.276 | -0.0144 (0.0021) | 1.3E-11 | -0.0134 (0.0247) | 0.587 | 47.0 | 0.931 |
| Appendicular lean mass | Alzheimer's disease, wide (FinnGen) | rs2663126 | 15:99563857 | A/G | 0.691 | -0.0139 (0.0021) | 1.4E-11 | -0.0119 (0.0251) | 0.635 | 43.8 | 0.856 |
| Appendicular lean mass | Alzheimer's disease, wide (FinnGen) | rs115233595 | 4:119744214 | C/T | 0.062 | -0.0264 (0.0039) | 1.4E-11 | 0.0274 (0.0362) | 0.449 | 45.8 | -1.038 |
| Appendicular lean mass | Alzheimer's disease, wide (FinnGen) | rs12340775 | 9:13226945 | A/G | 0.055 | -0.0287 (0.0043) | 1.7E-11 | -0.0057 (0.0396) | 0.885 | 44.5 | 0.199 |
| Appendicular lean mass | Alzheimer's disease, wide (FinnGen) | rs2252031 | 12:103248463 | C/T | 0.846 | -0.0176 (0.0026) | 1.8E-11 | 0.0072 (0.0362) | 0.842 | 45.8 | -0.409 |
| Appendicular lean mass | Alzheimer's disease, wide (FinnGen) | rs6923230 | 6:88213949 | C/T | 0.534 | -0.0127 (0.0019) | 1.8E-11 | -0.0035 (0.0213) | 0.871 | 44.7 | 0.276 |
| Appendicular lean mass | Alzheimer's disease, wide (FinnGen) | rs10461725 | 5:39437129 | C/G | 0.656 | 0.0134 (0.0020) | 2.0E-11 | 0.0278 (0.0217) | 0.200 | 44.9 | 2.075 |
| Appendicular lean mass | Alzheimer's disease, wide (FinnGen) | rs501250 | 5:112155793 | C/G | 0.619 | -0.0130 (0.0019) | 2.1E-11 | 0.0271 (0.0216) | 0.209 | 46.8 | -2.085 |
| Appendicular lean mass | Alzheimer's disease, wide (FinnGen) | rs10840399 | 11:10399121 | T/C | 0.101 | -0.0209 (0.0031) | 2.8E-11 | 0.0126 (0.0315) | 0.689 | 45.5 | -0.603 |
| Appendicular lean mass | Alzheimer's disease, wide (FinnGen) | rs7543202 | 1:73872885 | G/A | 0.623 | 0.0129 (0.0019) | 2.9E-11 | 0.0137 (0.0219) | 0.531 | 46.1 | 1.062 |
| Appendicular lean mass | Alzheimer's disease, wide (FinnGen) | rs2678898 | 2:58171964 | C/T | 0.380 | -0.0129 (0.0019) | 3.1E-11 | 0.0156 (0.0219) | 0.476 | 46.1 | -1.209 |
| Appendicular lean mass | Alzheimer's disease, wide (FinnGen) | rs2994329 | 1:243647921 | T/C | 0.202 | 0.0155 (0.0023) | 3.1E-11 | 0.0327 (0.0254) | 0.197 | 45.4 | 2.110 |
| Appendicular lean mass | Alzheimer's disease, wide (FinnGen) | rs75702986 | 19:35566151 | A/G | 0.186 | -0.0163 (0.0025) | 3.1E-11 | 0.0243 (0.0262) | 0.355 | 42.5 | -1.491 |
| Appendicular lean mass | Alzheimer's disease, wide (FinnGen) | rs2268718 | 6:52415023 | T/C | 0.270 | 0.0141 (0.0021) | 3.2E-11 | -0.0009 (0.0223) | 0.967 | 45.1 | -0.064 |
| Appendicular lean mass | Alzheimer's disease, wide (FinnGen) | rs8084413 | 18:22869123 | A/G | 0.469 | -0.0127 (0.0019) | 3.2E-11 | -0.0320 (0.0214) | 0.135 | 44.7 | 2.520 |
| Appendicular lean mass | Alzheimer's disease, wide (FinnGen) | rs34522021 | 9:23350420 | T/C | 0.455 | 0.0126 (0.0019) | 3.4E-11 | 0.0370 (0.0213) | 0.083 | 44.0 | 2.937 |
| Appendicular lean mass | Alzheimer's disease, wide (FinnGen) | rs137998108 | 12:124322727 | CA/C | 0.133 | -0.0188 (0.0028) | 3.6E-11 | -0.0253 (0.0358) | 0.480 | 45.1 | 1.346 |
| Appendicular lean mass | Alzheimer's disease, wide (FinnGen) | rs2126942 | 5:98155979 | C/T | 0.580 | -0.0127 (0.0019) | 3.8E-11 | -0.0097 (0.0220) | 0.660 | 44.7 | 0.764 |
| Appendicular lean mass | Alzheimer's disease, wide (FinnGen) | rs10827415 | 10:35042616 | G/T | 0.261 | -0.0142 (0.0021) | 3.9E-11 | 0.0185 (0.0271) | 0.494 | 45.7 | -1.303 |
| Appendicular lean mass | Alzheimer's disease, wide (FinnGen) | rs116008080 | 16:67254841 | A/G | 0.024 | -0.0415 (0.0063) | 4.1E-11 | -0.0511 (0.0644) | 0.428 | 43.4 | 1.231 |
| Appendicular lean mass | Alzheimer's disease, wide (FinnGen) | rs6738207 | 2:105989716 | A/G | 0.401 | 0.0127 (0.0019) | 4.1E-11 | -0.0117 (0.0226) | 0.605 | 44.7 | -0.921 |
| Appendicular lean mass | Alzheimer's disease, wide (FinnGen) | rs113671109 | 3:12620885 | C/T | 0.220 | -0.0150 (0.0023) | 4.2E-11 | 0.0159 (0.0245) | 0.517 | 42.5 | -1.060 |
| Appendicular lean mass | Alzheimer's disease, wide (FinnGen) | rs2965074 | 7:45983129 | T/G | 0.452 | 0.0125 (0.0019) | 4.3E-11 | 0.0059 (0.0218) | 0.788 | 43.3 | 0.472 |
| Appendicular lean mass | Alzheimer's disease, wide (FinnGen) | rs1800504 | 7:50742180 | T/C | 0.552 | -0.0125 (0.0019) | 4.4E-11 | -0.0252 (0.0214) | 0.238 | 43.3 | 2.016 |
| Appendicular lean mass | Alzheimer's disease, wide (FinnGen) | rs34203781 | 6:41666292 | C/A | 0.137 | 0.0181 (0.0028) | 5.6E-11 | -0.0225 (0.0289) | 0.435 | 41.8 | -1.243 |
| Appendicular lean mass | Alzheimer's disease, wide (FinnGen) | rs2347808 | 18:2750856 | A/G | 0.514 | -0.0125 (0.0019) | 5.8E-11 | 0.0186 (0.0212) | 0.379 | 43.3 | -1.488 |
| Appendicular lean mass | Alzheimer's disease, wide (FinnGen) | rs3830008 | 11:117093764 | C/A | 0.204 | -0.0154 (0.0023) | 6.1E-11 | -0.0081 (0.0243) | 0.738 | 44.8 | 0.526 |
| Appendicular lean mass | Alzheimer's disease, wide (FinnGen) | rs2490302 | 10:37702435 | A/T | 0.914 | 0.0221 (0.0034) | 6.2E-11 | -0.0504 (0.0448) | 0.261 | 42.2 | -2.281 |
| Appendicular lean mass | Alzheimer's disease, wide (FinnGen) | rs757042 | 7:25662829 | C/T | 0.724 | -0.0140 (0.0021) | 6.3E-11 | 0.0029 (0.0224) | 0.897 | 44.4 | -0.207 |
| Appendicular lean mass | Alzheimer's disease, wide (FinnGen) | rs8180765 | 7:4680614 | G/A | 0.217 | -0.0151 (0.0023) | 6.5E-11 | -0.0282 (0.0241) | 0.242 | 43.1 | 1.868 |
| Appendicular lean mass | Alzheimer's disease, wide (FinnGen) | rs11605297 | 11:58296806 | A/G | 0.233 | 0.0146 (0.0022) | 8.0E-11 | -0.0290 (0.0244) | 0.235 | 44.0 | -1.986 |
| Appendicular lean mass | Alzheimer's disease, wide (FinnGen) | rs496783 | 10:116137961 | G/A | 0.465 | -0.0124 (0.0019) | 8.1E-11 | -0.0047 (0.0223) | 0.833 | 42.6 | 0.379 |
| Appendicular lean mass | Alzheimer's disease, wide (FinnGen) | rs603486 | 11:118587347 | C/T | 0.617 | 0.0127 (0.0020) | 8.8E-11 | 0.0014 (0.0222) | 0.950 | 40.3 | 0.110 |
| Appendicular lean mass | Alzheimer's disease, wide (FinnGen) | rs1899040 | 2:223901896 | T/C | 0.796 | 0.0152 (0.0023) | 9.0E-11 | -0.0156 (0.0262) | 0.552 | 43.7 | -1.026 |
| Appendicular lean mass | Alzheimer's disease, wide (FinnGen) | rs35874463 | 15:67457698 | G/A | 0.058 | 0.0263 (0.0041) | 9.1E-11 | 0.0248 (0.0552) | 0.653 | 41.1 | 0.943 |
| Appendicular lean mass | Alzheimer's disease, wide (FinnGen) | rs13109280 | 4:54380513 | G/A | 0.662 | 0.0131 (0.0020) | 9.1E-11 | 0.0027 (0.0223) | 0.905 | 42.9 | 0.206 |
| Appendicular lean mass | Alzheimer's disease, wide (FinnGen) | rs13127468 | 4:8599658 | A/C | 0.463 | -0.0123 (0.0019) | 9.9E-11 | 0.0059 (0.0216) | 0.785 | 41.9 | -0.480 |
| Appendicular lean mass | Alzheimer's disease, wide (FinnGen) | rs8064946 | 17:7589311 | C/G | 0.110 | -0.0195 (0.0030) | 1.0E-10 | 0.0001 (0.0419) | 0.998 | 42.2 | -0.005 |
| Appendicular lean mass | Alzheimer's disease, wide (FinnGen) | rs10815304 | 9:5908217 | C/T | 0.206 | 0.0151 (0.0023) | 1.1E-10 | 0.0169 (0.0277) | 0.541 | 43.1 | 1.119 |
| Appendicular lean mass | Alzheimer's disease, wide (FinnGen) | rs56112295 | 14:105877057 | T/C | 0.226 | 0.0154 (0.0024) | 1.1E-10 | -0.0108 (0.0269) | 0.688 | 41.2 | -0.701 |
| Appendicular lean mass | Alzheimer's disease, wide (FinnGen) | rs11221657 | 11:129181358 | G/T | 0.135 | 0.0179 (0.0028) | 1.2E-10 | 0.0068 (0.0291) | 0.817 | 40.9 | 0.380 |
| Appendicular lean mass | Alzheimer's disease, wide (FinnGen) | rs10019221 | 4:21785364 | T/G | 0.597 | -0.0124 (0.0019) | 1.2E-10 | -0.0215 (0.0220) | 0.330 | 42.6 | 1.734 |
| Appendicular lean mass | Alzheimer's disease, wide (FinnGen) | rs55717234 | 1:150999863 | G/A | 0.573 | 0.0122 (0.0019) | 1.3E-10 | -0.0142 (0.0218) | 0.515 | 41.2 | -1.164 |
| Appendicular lean mass | Alzheimer's disease, wide (FinnGen) | rs1405227 | 1:98873390 | A/G | 0.317 | 0.0129 (0.0020) | 1.6E-10 | -0.0238 (0.0219) | 0.278 | 41.6 | -1.845 |
| Appendicular lean mass | Alzheimer's disease, wide (FinnGen) | rs200302461 | 2:9770589 | GCGGGGC/G | 0.259 | 0.0145 (0.0023) | 1.6E-10 | -0.0408 (0.0248) | 0.100 | 39.7 | -2.814 |
| Appendicular lean mass | Alzheimer's disease, wide (FinnGen) | rs10116740 | 9:133446357 | A/G | 0.477 | -0.0121 (0.0019) | 1.8E-10 | -0.0137 (0.0213) | 0.518 | 40.6 | 1.132 |
| Appendicular lean mass | Alzheimer's disease, wide (FinnGen) | rs111622870 | 4:2613109 | C/T | 0.048 | -0.0282 (0.0044) | 1.9E-10 | 0.0339 (0.0688) | 0.622 | 41.1 | -1.202 |
| Appendicular lean mass | Alzheimer's disease, wide (FinnGen) | rs650508 | 2:45880122 | C/G | 0.301 | -0.0130 (0.0020) | 1.9E-10 | -0.0111 (0.0257) | 0.666 | 42.2 | 0.854 |
| Appendicular lean mass | Alzheimer's disease, wide (FinnGen) | rs6054491 | 20:6709535 | G/C | 0.240 | -0.0142 (0.0022) | 2.2E-10 | 0.0091 (0.0241) | 0.704 | 41.7 | -0.641 |
| Appendicular lean mass | Alzheimer's disease, wide (FinnGen) | rs79680939 | 17:80523375 | A/G | 0.045 | 0.0290 (0.0046) | 2.5E-10 | 0.0891 (0.0695) | 0.199 | 39.7 | 3.072 |
| Appendicular lean mass | Alzheimer's disease, wide (FinnGen) | rs56207600 | 11:126196537 | A/G | 0.111 | 0.0192 (0.0030) | 2.5E-10 | -0.0253 (0.0312) | 0.417 | 41.0 | -1.318 |
| Appendicular lean mass | Alzheimer's disease, wide (FinnGen) | rs9828525 | 3:61552810 | T/C | 0.411 | 0.0121 (0.0019) | 2.5E-10 | -0.0138 (0.0216) | 0.524 | 40.6 | -1.141 |
| Appendicular lean mass | Alzheimer's disease, wide (FinnGen) | rs4682483 | 3:112993982 | A/G | 0.153 | -0.0165 (0.0026) | 2.7E-10 | -0.0061 (0.0240) | 0.800 | 40.3 | 0.370 |
| Appendicular lean mass | Alzheimer's disease, wide (FinnGen) | rs113146332 | 17:42565977 | C/G | 0.038 | 0.0311 (0.0049) | 3.0E-10 | 0.0584 (0.0967) | 0.546 | 40.3 | 1.878 |
| Appendicular lean mass | Alzheimer's disease, wide (FinnGen) | rs7030071 | 9:136951556 | G/A | 0.311 | -0.0130 (0.0021) | 3.1E-10 | -0.0133 (0.0222) | 0.550 | 38.3 | 1.023 |
| Appendicular lean mass | Alzheimer's disease, wide (FinnGen) | rs11410717 | 7:116252956 | TG/T | 0.117 | 0.0185 (0.0029) | 3.1E-10 | -0.0298 (0.0299) | 0.319 | 40.7 | -1.611 |
| Appendicular lean mass | Alzheimer's disease, wide (FinnGen) | rs34995241 | 6:83631850 | AT/A | 0.708 | -0.0131 (0.0021) | 3.2E-10 | 0.0092 (0.0240) | 0.702 | 38.9 | -0.702 |
| Appendicular lean mass | Alzheimer's disease, wide (FinnGen) | rs77299262 | 3:187450067 | C/T | 0.067 | 0.0237 (0.0038) | 3.4E-10 | 0.0888 (0.0386) | 0.022 | 38.9 | 3.747 |
| Appendicular lean mass | Alzheimer's disease, wide (FinnGen) | rs1407204 | 6:119289835 | A/C | 0.810 | -0.0151 (0.0024) | 3.5E-10 | -0.0222 (0.0316) | 0.483 | 39.6 | 1.470 |
| Appendicular lean mass | Alzheimer's disease, wide (FinnGen) | rs11191208 | 10:103838497 | A/G | 0.206 | 0.0147 (0.0024) | 3.7E-10 | -0.0134 (0.0263) | 0.610 | 37.5 | -0.912 |
| Appendicular lean mass | Alzheimer's disease, wide (FinnGen) | rs11684531 | 2:219835489 | G/A | 0.133 | -0.0172 (0.0028) | 4.2E-10 | -0.0428 (0.0421) | 0.309 | 37.7 | 2.488 |
| Appendicular lean mass | Alzheimer's disease, wide (FinnGen) | rs2823990 | 21:18081151 | A/G | 0.677 | -0.0127 (0.0020) | 4.2E-10 | 0.0132 (0.0220) | 0.548 | 40.3 | -1.039 |
| Appendicular lean mass | Alzheimer's disease, wide (FinnGen) | rs3792819 | 5:172576296 | G/A | 0.085 | 0.0210 (0.0034) | 4.4E-10 | 0.1154 (0.0588) | 0.050 | 38.1 | 5.495 |
| Appendicular lean mass | Alzheimer's disease, wide (FinnGen) | rs10762866 | 10:81228430 | A/G | 0.138 | 0.0171 (0.0028) | 5.4E-10 | 0.0033 (0.0274) | 0.903 | 37.3 | 0.193 |
| Appendicular lean mass | Alzheimer's disease, wide (FinnGen) | rs7359097 | 14:54757497 | C/T | 0.396 | -0.0121 (0.0019) | 5.5E-10 | -0.0170 (0.0216) | 0.431 | 40.6 | 1.405 |
| Appendicular lean mass | Alzheimer's disease, wide (FinnGen) | rs10112506 | 8:13164746 | G/A | 0.390 | -0.0120 (0.0019) | 5.8E-10 | -0.0085 (0.0230) | 0.711 | 39.9 | 0.708 |
| Appendicular lean mass | Alzheimer's disease, wide (FinnGen) | rs2974337 | 8:42390873 | T/C | 0.500 | 0.0117 (0.0019) | 5.9E-10 | -0.0023 (0.0213) | 0.913 | 37.9 | -0.197 |
| Appendicular lean mass | Alzheimer's disease, wide (FinnGen) | rs11421589 | 10:115193949 | CT/C | 0.556 | 0.0119 (0.0019) | 6.0E-10 | -0.0423 (0.0214) | 0.048 | 39.2 | -3.555 |
| Appendicular lean mass | Alzheimer's disease, wide (FinnGen) | rs7144307 | 14:69533837 | C/T | 0.385 | -0.0122 (0.0020) | 6.1E-10 | 0.0146 (0.0216) | 0.499 | 37.2 | -1.197 |
| Appendicular lean mass | Alzheimer's disease, wide (FinnGen) | rs4754296 | 11:108006182 | G/T | 0.146 | -0.0167 (0.0027) | 6.1E-10 | 0.0089 (0.0254) | 0.727 | 38.3 | -0.533 |
| Appendicular lean mass | Alzheimer's disease, wide (FinnGen) | rs139921635 | 4:73181637 | T/G | 0.024 | 0.0385 (0.0062) | 6.2E-10 | 0.0997 (0.0614) | 0.104 | 38.6 | 2.590 |
| Appendicular lean mass | Alzheimer's disease, wide (FinnGen) | rs10241451 | 7:55248926 | C/T | 0.209 | 0.0145 (0.0023) | 6.3E-10 | 0.0127 (0.0269) | 0.638 | 39.7 | 0.876 |
| Appendicular lean mass | Alzheimer's disease, wide (FinnGen) | rs447352 | 5:678750 | T/C | 0.141 | -0.0181 (0.0029) | 6.6E-10 | -0.0269 (0.0355) | 0.449 | 39.0 | 1.486 |
| Appendicular lean mass | Alzheimer's disease, wide (FinnGen) | rs718603 | 6:2644245 | T/C | 0.277 | 0.0131 (0.0021) | 6.7E-10 | -0.0572 (0.0238) | 0.016 | 38.9 | -4.366 |
| Appendicular lean mass | Alzheimer's disease, wide (FinnGen) | rs2875409 | 13:41112849 | A/G | 0.050 | -0.0270 (0.0044) | 7.1E-10 | 0.0347 (0.0558) | 0.534 | 37.6 | -1.285 |
| Appendicular lean mass | Alzheimer's disease, wide (FinnGen) | rs839255 | 3:57974580 | G/T | 0.685 | -0.0126 (0.0021) | 8.4E-10 | -0.0319 (0.0230) | 0.165 | 36.0 | 2.532 |
| Appendicular lean mass | Alzheimer's disease, wide (FinnGen) | rs10849576 | 12:993652 | G/T | 0.410 | -0.0117 (0.0019) | 9.2E-10 | 0.0071 (0.0228) | 0.757 | 37.9 | -0.607 |
| Appendicular lean mass | Alzheimer's disease, wide (FinnGen) | rs11688284 | 2:174738951 | T/C | 0.388 | -0.0119 (0.0019) | 9.8E-10 | 0.0172 (0.0222) | 0.439 | 39.2 | -1.445 |
| Appendicular lean mass | Alzheimer's disease, wide (FinnGen) | rs116339650 | 4:26200972 | G/A | 0.127 | -0.0175 (0.0029) | 1.1E-09 | -0.0123 (0.0288) | 0.669 | 36.4 | 0.703 |
| Appendicular lean mass | Alzheimer's disease, wide (FinnGen) | rs670129 | 20:10460858 | T/C | 0.413 | -0.0118 (0.0019) | 1.1E-09 | 0.0480 (0.0224) | 0.032 | 38.6 | -4.068 |
| Appendicular lean mass | Alzheimer's disease, wide (FinnGen) | rs7328187 | 13:74189974 | G/T | 0.498 | 0.0116 (0.0019) | 1.2E-09 | -0.0202 (0.0217) | 0.352 | 37.3 | -1.741 |
| Appendicular lean mass | Alzheimer's disease, wide (FinnGen) | rs62143873 | 2:72035050 | A/G | 0.503 | -0.0115 (0.0019) | 1.2E-09 | -0.0274 (0.0215) | 0.203 | 36.6 | 2.383 |
| Appendicular lean mass | Alzheimer's disease, wide (FinnGen) | rs6469845 | 8:120792195 | T/C | 0.239 | 0.0135 (0.0022) | 1.2E-09 | -0.0284 (0.0233) | 0.224 | 37.6 | -2.104 |
| Appendicular lean mass | Alzheimer's disease, wide (FinnGen) | rs4940874 | 18:57105638 | G/A | 0.812 | 0.0148 (0.0024) | 1.2E-09 | 0.0516 (0.0252) | 0.041 | 38.0 | 3.486 |
| Appendicular lean mass | Alzheimer's disease, wide (FinnGen) | rs75172776 | 16:9001814 | G/A | 0.066 | 0.0232 (0.0038) | 1.3E-09 | -0.0022 (0.0518) | 0.967 | 37.3 | -0.095 |
| Appendicular lean mass | Alzheimer's disease, wide (FinnGen) | rs61925210 | 12:43974800 | T/A | 0.105 | 0.0187 (0.0031) | 1.3E-09 | -0.0472 (0.0332) | 0.155 | 36.4 | -2.524 |
| Appendicular lean mass | Alzheimer's disease, wide (FinnGen) | rs4932439 | 15:89401109 | G/A | 0.825 | -0.0151 (0.0025) | 1.4E-09 | -0.0035 (0.0235) | 0.882 | 36.5 | 0.232 |
| Appendicular lean mass | Alzheimer's disease, wide (FinnGen) | rs249681 | 5:141538017 | C/G | 0.618 | -0.0117 (0.0019) | 1.5E-09 | -0.0230 (0.0223) | 0.302 | 37.9 | 1.966 |
| Appendicular lean mass | Alzheimer's disease, wide (FinnGen) | rs616488 | 1:10566215 | G/A | 0.345 | 0.0120 (0.0020) | 1.5E-09 | -0.0320 (0.0224) | 0.154 | 36.0 | -2.667 |
| Appendicular lean mass | Alzheimer's disease, wide (FinnGen) | rs17773965 | 2:217631338 | T/C | 0.141 | -0.0163 (0.0027) | 1.5E-09 | 0.0064 (0.0248) | 0.798 | 36.5 | -0.393 |
| Appendicular lean mass | Alzheimer's disease, wide (FinnGen) | rs4554207 | 5:114388634 | C/T | 0.515 | -0.0114 (0.0019) | 1.6E-09 | -0.0684 (0.0212) | 0.001 | 36.0 | 6.000 |
| Appendicular lean mass | Alzheimer's disease, wide (FinnGen) | rs1190540 | 14:102897009 | G/A | 0.698 | 0.0125 (0.0021) | 1.6E-09 | -0.0032 (0.0225) | 0.885 | 35.4 | -0.256 |
| Appendicular lean mass | Alzheimer's disease, wide (FinnGen) | rs1202186 | 7:87213258 | T/C | 0.655 | -0.0120 (0.0020) | 1.7E-09 | 0.0074 (0.0230) | 0.749 | 36.0 | -0.617 |
| Appendicular lean mass | Alzheimer's disease, wide (FinnGen) | rs62033029 | 16:50107273 | A/G | 0.206 | -0.0141 (0.0023) | 1.7E-09 | 0.0384 (0.0274) | 0.161 | 37.6 | -2.723 |
| Appendicular lean mass | Alzheimer's disease, wide (FinnGen) | rs13112742 | 4:124888059 | A/G | 0.170 | -0.0151 (0.0025) | 2.0E-09 | -0.0029 (0.0310) | 0.926 | 36.5 | 0.192 |
| Appendicular lean mass | Alzheimer's disease, wide (FinnGen) | rs680882 | 7:18325278 | G/T | 0.761 | 0.0133 (0.0022) | 2.0E-09 | -0.0094 (0.0244) | 0.700 | 36.5 | -0.707 |
| Appendicular lean mass | Alzheimer's disease, wide (FinnGen) | rs8017006 | 14:42745052 | G/A | 0.328 | 0.0122 (0.0020) | 2.2E-09 | 0.0020 (0.0241) | 0.934 | 37.2 | 0.164 |
| Appendicular lean mass | Alzheimer's disease, wide (FinnGen) | rs2569888 | 6:1625803 | A/G | 0.245 | 0.0133 (0.0022) | 2.3E-09 | 0.0409 (0.0246) | 0.097 | 36.5 | 3.075 |
| Appendicular lean mass | Alzheimer's disease, wide (FinnGen) | rs10779958 | 2:74755778 | C/A | 0.149 | 0.0157 (0.0026) | 2.6E-09 | -0.0327 (0.0306) | 0.284 | 36.5 | -2.083 |
| Appendicular lean mass | Alzheimer's disease, wide (FinnGen) | rs66613683 | 12:104409025 | C/T | 0.170 | 0.0150 (0.0025) | 2.6E-09 | -0.0375 (0.0303) | 0.216 | 36.0 | -2.500 |
| Appendicular lean mass | Alzheimer's disease, wide (FinnGen) | rs114018835 | 1:244274455 | T/C | 0.036 | 0.0303 (0.0051) | 2.7E-09 | 0.1244 (0.0895) | 0.165 | 35.3 | 4.106 |
| Appendicular lean mass | Alzheimer's disease, wide (FinnGen) | rs150474185 | 1:65689032 | G/C | 0.021 | 0.0440 (0.0074) | 2.7E-09 | 0.3689 (0.1280) | 0.004 | 35.4 | 8.384 |
| Appendicular lean mass | Alzheimer's disease, wide (FinnGen) | rs114192718 | 2:128785663 | T/C | 0.075 | -0.0215 (0.0036) | 2.9E-09 | -0.0483 (0.0503) | 0.337 | 35.7 | 2.247 |
| Appendicular lean mass | Alzheimer's disease, wide (FinnGen) | rs17713523 | 5:149923968 | A/G | 0.407 | 0.0114 (0.0019) | 3.1E-09 | -0.0419 (0.0212) | 0.049 | 36.0 | -3.675 |
| Appendicular lean mass | Alzheimer's disease, wide (FinnGen) | rs11175919 | 12:66180277 | A/G | 0.026 | 0.0349 (0.0059) | 3.2E-09 | 0.0447 (0.0651) | 0.492 | 35.0 | 1.281 |
| Appendicular lean mass | Alzheimer's disease, wide (FinnGen) | rs3118124 | 1:150166675 | G/A | 0.289 | -0.0122 (0.0021) | 3.2E-09 | 0.0038 (0.0244) | 0.876 | 33.8 | -0.311 |
| Appendicular lean mass | Alzheimer's disease, wide (FinnGen) | rs78838233 | 17:18257589 | T/C | 0.237 | -0.0132 (0.0022) | 3.3E-09 | -0.0092 (0.0242) | 0.703 | 36.0 | 0.697 |
| Appendicular lean mass | Alzheimer's disease, wide (FinnGen) | rs3769885 | 2:165592390 | A/G | 0.490 | -0.0111 (0.0019) | 3.5E-09 | 0.0173 (0.0214) | 0.419 | 34.1 | -1.559 |
| Appendicular lean mass | Alzheimer's disease, wide (FinnGen) | rs3003137 | 20:62830802 | T/G | 0.428 | -0.0114 (0.0019) | 3.6E-09 | -0.0339 (0.0222) | 0.128 | 36.0 | 2.974 |
| Appendicular lean mass | Alzheimer's disease, wide (FinnGen) | rs13316 | 14:93407301 | A/C | 0.430 | 0.0115 (0.0019) | 3.7E-09 | 0.0180 (0.0214) | 0.400 | 36.6 | 1.565 |
| Appendicular lean mass | Alzheimer's disease, wide (FinnGen) | rs2788213 | 6:703249 | A/G | 0.710 | 0.0123 (0.0021) | 3.8E-09 | -0.0026 (0.0236) | 0.912 | 34.3 | -0.211 |
| Appendicular lean mass | Alzheimer's disease, wide (FinnGen) | rs7095087 | 10:31120513 | A/G | 0.353 | 0.0117 (0.0020) | 3.8E-09 | 0.0041 (0.0238) | 0.864 | 34.2 | 0.350 |
| Appendicular lean mass | Alzheimer's disease, wide (FinnGen) | rs603542 | 1:44079411 | C/T | 0.675 | 0.0118 (0.0020) | 3.8E-09 | 0.0372 (0.0225) | 0.099 | 34.8 | 3.153 |
| Appendicular lean mass | Alzheimer's disease, wide (FinnGen) | rs290793 | 7:130416726 | C/G | 0.188 | 0.0142 (0.0024) | 4.0E-09 | -0.0295 (0.0258) | 0.253 | 35.0 | -2.077 |
| Appendicular lean mass | Alzheimer's disease, wide (FinnGen) | rs41298373 | 10:7622009 | A/G | 0.101 | 0.0185 (0.0031) | 4.2E-09 | 0.0048 (0.0313) | 0.879 | 35.6 | 0.259 |
| Appendicular lean mass | Alzheimer's disease, wide (FinnGen) | rs4836544 | 9:129236355 | T/C | 0.885 | 0.0175 (0.0030) | 4.3E-09 | -0.0379 (0.0407) | 0.351 | 34.0 | -2.166 |
| Appendicular lean mass | Alzheimer's disease, wide (FinnGen) | rs4121583 | 18:125075 | T/C | 0.619 | 0.0118 (0.0020) | 4.6E-09 | -0.0084 (0.0222) | 0.705 | 34.8 | -0.712 |
| Appendicular lean mass | Alzheimer's disease, wide (FinnGen) | rs113827862 | 14:89849527 | C/T | 0.061 | -0.0235 (0.0040) | 4.7E-09 | 0.0012 (0.0402) | 0.976 | 34.5 | -0.051 |
| Appendicular lean mass | Alzheimer's disease, wide (FinnGen) | rs2150692 | 13:30109494 | A/C | 0.855 | -0.0158 (0.0027) | 4.8E-09 | -0.0027 (0.0254) | 0.917 | 34.2 | 0.171 |
| Appendicular lean mass | Alzheimer's disease, wide (FinnGen) | rs9636364 | 2:111992435 | A/G | 0.543 | 0.0110 (0.0019) | 5.1E-09 | 0.0417 (0.0214) | 0.051 | 33.5 | 3.791 |
| Appendicular lean mass | Alzheimer's disease, wide (FinnGen) | rs577289 | 15:40208911 | T/A | 0.281 | -0.0125 (0.0021) | 5.4E-09 | -0.0231 (0.0231) | 0.317 | 35.4 | 1.848 |
| Appendicular lean mass | Alzheimer's disease, wide (FinnGen) | rs117972846 | 17:26947476 | G/T | 0.029 | 0.0335 (0.0057) | 5.5E-09 | -0.0212 (0.0653) | 0.745 | 34.5 | -0.633 |
| Appendicular lean mass | Alzheimer's disease, wide (FinnGen) | rs4652902 | 1:36612955 | A/C | 0.768 | 0.0129 (0.0022) | 5.7E-09 | -0.0273 (0.0236) | 0.246 | 34.4 | -2.116 |
| Appendicular lean mass | Alzheimer's disease, wide (FinnGen) | rs3103223 | 4:42402721 | C/T | 0.740 | 0.0126 (0.0022) | 6.0E-09 | 0.0299 (0.0250) | 0.232 | 32.8 | 2.373 |
| Appendicular lean mass | Alzheimer's disease, wide (FinnGen) | rs10060855 | 5:33216769 | G/A | 0.677 | 0.0118 (0.0020) | 6.2E-09 | -0.0026 (0.0220) | 0.906 | 34.8 | -0.220 |
| Appendicular lean mass | Alzheimer's disease, wide (FinnGen) | rs1527149 | 7:114004088 | G/C | 0.407 | 0.0113 (0.0019) | 6.9E-09 | -0.0005 (0.0219) | 0.982 | 35.4 | -0.044 |
| Appendicular lean mass | Alzheimer's disease, wide (FinnGen) | rs35701316 | 20:39805449 | GTGTT/G | 0.174 | -0.0145 (0.0025) | 7.0E-09 | -0.0037 (0.0242) | 0.879 | 33.6 | 0.255 |
| Appendicular lean mass | Alzheimer's disease, wide (FinnGen) | rs11132166 | 4:184049867 | C/T | 0.036 | -0.0292 (0.0050) | 7.6E-09 | 0.0105 (0.0757) | 0.890 | 34.1 | -0.360 |
| Appendicular lean mass | Alzheimer's disease, wide (FinnGen) | rs34338597 | 12:106301580 | G/A | 0.383 | -0.0112 (0.0019) | 7.9E-09 | -0.0007 (0.0216) | 0.975 | 34.8 | 0.062 |
| Appendicular lean mass | Alzheimer's disease, wide (FinnGen) | rs4849904 | 2:121336556 | G/C | 0.380 | 0.0111 (0.0019) | 8.1E-09 | 0.0341 (0.0238) | 0.153 | 34.1 | 3.072 |
| Appendicular lean mass | Alzheimer's disease, wide (FinnGen) | rs612577 | 1:230324364 | C/T | 0.149 | -0.0152 (0.0026) | 8.2E-09 | -0.0044 (0.0273) | 0.871 | 34.2 | 0.289 |
| Appendicular lean mass | Alzheimer's disease, wide (FinnGen) | rs111925803 | 22:50719976 | T/C | 0.285 | 0.0122 (0.0021) | 8.6E-09 | 0.0026 (0.0241) | 0.914 | 33.8 | 0.213 |
| Appendicular lean mass | Alzheimer's disease, wide (FinnGen) | rs921142 | 8:41169276 | C/T | 0.398 | -0.0111 (0.0019) | 8.7E-09 | -0.0311 (0.0213) | 0.145 | 34.1 | 2.802 |
| Appendicular lean mass | Alzheimer's disease, wide (FinnGen) | rs10099846 | 8:22534178 | T/C | 0.668 | 0.0116 (0.0020) | 8.8E-09 | -0.0108 (0.0236) | 0.648 | 33.6 | -0.931 |
| Appendicular lean mass | Alzheimer's disease, wide (FinnGen) | rs7744700 | 6:53349401 | A/T | 0.291 | -0.0122 (0.0021) | 9.2E-09 | -0.0311 (0.0233) | 0.182 | 33.8 | 2.549 |
| Appendicular lean mass | Alzheimer's disease, wide (FinnGen) | rs12483401 | 21:35443829 | C/T | 0.022 | -0.0387 (0.0067) | 9.2E-09 | -0.0017 (0.0842) | 0.983 | 33.4 | 0.044 |
| Appendicular lean mass | Alzheimer's disease, wide (FinnGen) | rs68185109 | 10:93648306 | A/G | 0.102 | 0.0180 (0.0031) | 9.6E-09 | 0.0328 (0.0361) | 0.365 | 33.7 | 1.822 |
| Appendicular lean mass | Alzheimer's disease, wide (FinnGen) | rs28736838 | 1:120148713 | T/C | 0.302 | -0.0117 (0.0020) | 1.1E-08 | 0.0155 (0.0228) | 0.495 | 34.2 | -1.325 |
| Appendicular lean mass | Alzheimer's disease, wide (FinnGen) | rs2208404 | 20:55843736 | G/A | 0.416 | -0.0111 (0.0019) | 1.1E-08 | 0.0014 (0.0217) | 0.947 | 34.1 | -0.126 |
| Appendicular lean mass | Alzheimer's disease, wide (FinnGen) | rs8018486 | 14:39818616 | G/A | 0.191 | -0.0138 (0.0024) | 1.2E-08 | 0.0050 (0.0256) | 0.845 | 33.1 | -0.362 |
| Appendicular lean mass | Alzheimer's disease, wide (FinnGen) | rs10749157 | 10:115780129 | C/T | 0.358 | 0.0113 (0.0020) | 1.2E-08 | -0.0205 (0.0213) | 0.336 | 31.9 | -1.814 |
| Appendicular lean mass | Alzheimer's disease, wide (FinnGen) | rs11070842 | 15:51624185 | C/T | 0.163 | -0.0146 (0.0026) | 1.3E-08 | -0.0149 (0.0361) | 0.679 | 31.5 | 1.021 |
| Appendicular lean mass | Alzheimer's disease, wide (FinnGen) | rs62103240 | 18:77650637 | A/G | 0.071 | 0.0212 (0.0037) | 1.4E-08 | 0.0837 (0.0496) | 0.091 | 32.8 | 3.948 |
| Appendicular lean mass | Alzheimer's disease, wide (FinnGen) | rs10222594 | 3:71554856 | A/G | 0.323 | 0.0115 (0.0020) | 1.4E-08 | 0.0135 (0.0234) | 0.565 | 33.1 | 1.174 |
| Appendicular lean mass | Alzheimer's disease, wide (FinnGen) | rs56309431 | 17:63645289 | T/G | 0.116 | 0.0167 (0.0029) | 1.5E-08 | -0.0292 (0.0343) | 0.394 | 33.2 | -1.748 |
| Appendicular lean mass | Alzheimer's disease, wide (FinnGen) | rs10764692 | 10:27576251 | C/A | 0.637 | -0.0111 (0.0020) | 1.6E-08 | -0.0103 (0.0229) | 0.652 | 30.8 | 0.928 |
| Appendicular lean mass | Alzheimer's disease, wide (FinnGen) | rs10940169 | 5:67823682 | C/G | 0.382 | -0.0110 (0.0019) | 1.6E-08 | 0.0177 (0.0224) | 0.429 | 33.5 | -1.609 |
| Appendicular lean mass | Alzheimer's disease, wide (FinnGen) | rs10221831 | 2:202107829 | T/C | 0.032 | 0.0300 (0.0053) | 1.8E-08 | 0.0847 (0.0610) | 0.165 | 32.0 | 2.823 |
| Appendicular lean mass | Alzheimer's disease, wide (FinnGen) | rs4965298 | 15:100802766 | T/C | 0.713 | -0.0119 (0.0021) | 1.8E-08 | 0.0183 (0.0254) | 0.472 | 32.1 | -1.538 |
| Appendicular lean mass | Alzheimer's disease, wide (FinnGen) | rs28678024 | 14:25937161 | G/A | 0.285 | -0.0119 (0.0021) | 1.9E-08 | -0.0439 (0.0227) | 0.053 | 32.1 | 3.689 |
| Appendicular lean mass | Alzheimer's disease, wide (FinnGen) | rs2188805 | 7:93078400 | C/A | 0.336 | 0.0114 (0.0020) | 2.0E-08 | -0.0241 (0.0245) | 0.326 | 32.5 | -2.114 |
| Appendicular lean mass | Alzheimer's disease, wide (FinnGen) | rs9590328 | 13:96448383 | G/A | 0.142 | 0.0153 (0.0027) | 2.0E-08 | -0.0503 (0.0310) | 0.105 | 32.1 | -3.288 |
| Appendicular lean mass | Alzheimer's disease, wide (FinnGen) | rs31196 | 5:158300798 | A/C | 0.572 | -0.0107 (0.0019) | 2.1E-08 | -0.0024 (0.0213) | 0.909 | 31.7 | 0.224 |
| Appendicular lean mass | Alzheimer's disease, wide (FinnGen) | rs876122 | 6:6886297 | G/A | 0.879 | 0.0162 (0.0029) | 2.2E-08 | 0.0218 (0.0290) | 0.453 | 31.2 | 1.346 |
| Appendicular lean mass | Alzheimer's disease, wide (FinnGen) | rs9288695 | 2:158641300 | C/G | 0.779 | 0.0127 (0.0023) | 2.2E-08 | 0.0139 (0.0257) | 0.589 | 30.5 | 1.094 |
| Appendicular lean mass | Alzheimer's disease, wide (FinnGen) | rs7082659 | 10:12017584 | C/T | 0.866 | 0.0156 (0.0028) | 2.3E-08 | -0.0347 (0.0330) | 0.294 | 31.0 | -2.224 |
| Appendicular lean mass | Alzheimer's disease, wide (FinnGen) | rs80280630 | 9:117030861 | T/C | 0.112 | -0.0168 (0.0030) | 2.3E-08 | 0.0270 (0.0394) | 0.492 | 31.4 | -1.607 |
| Appendicular lean mass | Alzheimer's disease, wide (FinnGen) | rs74048171 | 11:2093603 | A/C | 0.258 | -0.0121 (0.0022) | 2.4E-08 | 0.0155 (0.0255) | 0.544 | 30.2 | -1.281 |
| Appendicular lean mass | Alzheimer's disease, wide (FinnGen) | rs141277904 | 8:56654581 | T/C | 0.023 | 0.0384 (0.0069) | 2.4E-08 | 0.1231 (0.0855) | 0.150 | 31.0 | 3.206 |
| Appendicular lean mass | Alzheimer's disease, wide (FinnGen) | rs4916786 | 5:89476946 | T/C | 0.196 | 0.0133 (0.0024) | 2.4E-08 | -0.0446 (0.0258) | 0.083 | 30.7 | -3.353 |
| Appendicular lean mass | Alzheimer's disease, wide (FinnGen) | rs60142646 | 2:46532856 | TC/T | 0.948 | -0.0237 (0.0042) | 2.5E-08 | -0.0226 (0.0534) | 0.672 | 31.8 | 0.954 |
| Appendicular lean mass | Alzheimer's disease, wide (FinnGen) | rs2303423 | 4:38120029 | C/T | 0.110 | 0.0168 (0.0030) | 2.6E-08 | 0.0054 (0.0406) | 0.893 | 31.4 | 0.321 |
| Appendicular lean mass | Alzheimer's disease, wide (FinnGen) | rs9265546 | 6:31298202 | A/G | 0.223 | 0.0147 (0.0026) | 2.8E-08 | -0.0045 (0.0218) | 0.836 | 32.0 | -0.306 |
| Appendicular lean mass | Alzheimer's disease, wide (FinnGen) | rs149697773 | 17:60932424 | G/A | 0.045 | -0.0262 (0.0047) | 2.8E-08 | 0.0231 (0.0499) | 0.643 | 31.1 | -0.882 |
| Appendicular lean mass | Alzheimer's disease, wide (FinnGen) | rs2978362 | 18:32959397 | T/C | 0.533 | 0.0106 (0.0019) | 2.8E-08 | -0.0368 (0.0216) | 0.088 | 31.1 | -3.472 |
| Appendicular lean mass | Alzheimer's disease, wide (FinnGen) | rs4383083 | 15:63080442 | A/G | 0.647 | 0.0111 (0.0020) | 2.9E-08 | 0.0070 (0.0242) | 0.773 | 30.8 | 0.631 |
| Appendicular lean mass | Alzheimer's disease, wide (FinnGen) | rs10940364 | 5:53487971 | C/T | 0.139 | 0.0151 (0.0027) | 3.1E-08 | 0.0169 (0.0333) | 0.613 | 31.3 | 1.119 |
| Appendicular lean mass | Alzheimer's disease, wide (FinnGen) | rs77293269 | 3:155595709 | C/T | 0.113 | -0.0165 (0.0030) | 3.1E-08 | -0.0322 (0.0340) | 0.344 | 30.2 | 1.952 |
| Appendicular lean mass | Alzheimer's disease, wide (FinnGen) | rs62177315 | 2:161236992 | T/C | 0.096 | 0.0177 (0.0032) | 3.1E-08 | 0.0535 (0.0431) | 0.215 | 30.6 | 3.023 |
| Appendicular lean mass | Alzheimer's disease, wide (FinnGen) | rs72809820 | 2:97360079 | T/C | 0.324 | -0.0111 (0.0020) | 3.1E-08 | -0.0110 (0.0213) | 0.607 | 30.8 | 0.991 |
| Appendicular lean mass | Alzheimer's disease, wide (FinnGen) | rs9568031 | 13:48897520 | T/C | 0.706 | -0.0115 (0.0021) | 3.3E-08 | 0.0051 (0.0228) | 0.823 | 30.0 | -0.443 |
| Appendicular lean mass | Alzheimer's disease, wide (FinnGen) | rs10821713 | 10:62055781 | T/C | 0.418 | 0.0106 (0.0019) | 3.4E-08 | -0.0153 (0.0219) | 0.484 | 31.1 | -1.443 |
| Appendicular lean mass | Alzheimer's disease, wide (FinnGen) | rs12185775 | 20:20293769 | C/G | 0.117 | -0.0167 (0.0030) | 3.7E-08 | 0.0531 (0.0310) | 0.087 | 31.0 | -3.180 |
| Appendicular lean mass | Alzheimer's disease, wide (FinnGen) | rs6461948 | 7:26540727 | T/C | 0.673 | 0.0112 (0.0020) | 4.0E-08 | -0.0141 (0.0242) | 0.561 | 31.4 | -1.259 |
| Appendicular lean mass | Alzheimer's disease, wide (FinnGen) | rs11721522 | 4:156976051 | G/A | 0.412 | 0.0106 (0.0019) | 4.0E-08 | -0.0168 (0.0226) | 0.458 | 31.1 | -1.585 |
| Appendicular lean mass | Alzheimer's disease, wide (FinnGen) | rs3002284 | 1:213181916 | C/T | 0.320 | -0.0110 (0.0020) | 4.1E-08 | 0.0151 (0.0226) | 0.503 | 30.2 | -1.373 |
| Appendicular lean mass | Alzheimer's disease, wide (FinnGen) | rs35223841 | 2:144053000 | GAA/G | 0.389 | 0.0106 (0.0019) | 4.2E-08 | 0.0245 (0.0224) | 0.275 | 31.1 | 2.311 |
| Appendicular lean mass | Alzheimer's disease, wide (FinnGen) | rs34856058 | 3:101365150 | G/A | 0.726 | 0.0116 (0.0021) | 4.4E-08 | 0.0137 (0.0246) | 0.578 | 30.5 | 1.181 |
| Appendicular lean mass | Alzheimer's disease, wide (FinnGen) | rs1884066 | 14:67452834 | A/G | 0.150 | -0.0145 (0.0027) | 5.0E-08 | -0.0053 (0.0281) | 0.850 | 28.8 | 0.366 |
| Whole body fat-free mass | Alzheimer's disease (Bellenguez 2022) | rs12967135 | 18:57849023 | A/G | 0.233 | 0.0433 (0.0015) | 2.3E-190 | -0.0141 (0.0096) | 0.143 | 866.1 | -0.326 |
| Whole body fat-free mass | Alzheimer's disease (Bellenguez 2022) | rs56094641 | 16:53806453 | G/A | 0.405 | 0.0361 (0.0013) | 1.0E-178 | -0.0159 (0.0083) | 0.055 | 812.5 | -0.441 |
| Whole body fat-free mass | Alzheimer's disease (Bellenguez 2022) | rs143384 | 20:34025756 | G/A | 0.404 | 0.0354 (0.0013) | 1.5E-171 | -0.0057 (0.0082) | 0.487 | 779.6 | -0.161 |
| Whole body fat-free mass | Alzheimer's disease (Bellenguez 2022) | rs9853018 | 3:141101961 | T/C | 0.443 | 0.0300 (0.0012) | 6.3E-128 | -0.0138 (0.0083) | 0.095 | 579.0 | -0.460 |
| Whole body fat-free mass | Alzheimer's disease (Bellenguez 2022) | rs2744956 | 6:34618937 | C/T | 0.139 | 0.0378 (0.0018) | 2.9E-99 | -0.0479 (0.0117) | 4.5E-05 | 447.2 | -1.268 |
| Whole body fat-free mass | Alzheimer's disease (Bellenguez 2022) | rs1351394 | 12:66351826 | C/T | 0.512 | -0.0258 (0.0012) | 5.3E-96 | -0.0012 (0.0081) | 0.885 | 432.2 | 0.047 |
| Whole body fat-free mass | Alzheimer's disease (Bellenguez 2022) | rs7740107 | 6:130374461 | A/T | 0.736 | -0.0291 (0.0014) | 1.1E-95 | 0.0090 (0.0093) | 0.331 | 430.7 | -0.309 |
| Whole body fat-free mass | Alzheimer's disease (Bellenguez 2022) | rs12995666 | 2:629959 | C/T | 0.828 | 0.0337 (0.0016) | 1.1E-93 | -0.0200 (0.0106) | 0.060 | 421.6 | -0.594 |
| Whole body fat-free mass | Alzheimer's disease (Bellenguez 2022) | rs72656010 | 8:57122215 | C/T | 0.132 | -0.0366 (0.0018) | 2.8E-88 | -0.0039 (0.0115) | 0.738 | 396.7 | 0.106 |
| Whole body fat-free mass | Alzheimer's disease (Bellenguez 2022) | rs76895963 | 12:4384844 | G/T | 0.021 | 0.0952 (0.0048) | 2.8E-87 | -0.0069 (0.0332) | 0.836 | 392.2 | -0.072 |
| Whole body fat-free mass | Alzheimer's disease (Bellenguez 2022) | rs10269774 | 7:92253972 | A/G | 0.326 | 0.0262 (0.0013) | 4.7E-87 | 0.0079 (0.0085) | 0.355 | 391.1 | 0.302 |
| Whole body fat-free mass | Alzheimer's disease (Bellenguez 2022) | rs2225226 | 13:51127270 | T/C | 0.216 | -0.0298 (0.0015) | 3.1E-86 | -0.0021 (0.0098) | 0.828 | 387.4 | 0.070 |
| Whole body fat-free mass | Alzheimer's disease (Bellenguez 2022) | rs34517439 | 1:78450517 | A/C | 0.122 | 0.0372 (0.0019) | 3.3E-84 | 0.0009 (0.0132) | 0.943 | 378.1 | 0.024 |
| Whole body fat-free mass | Alzheimer's disease (Bellenguez 2022) | rs7671110 | 4:17874089 | T/C | 0.158 | -0.0318 (0.0017) | 2.8E-78 | 0.0401 (0.0110) | 2.8E-04 | 350.8 | -1.262 |
| Whole body fat-free mass | Alzheimer's disease (Bellenguez 2022) | rs7223535 | 17:29211667 | A/G | 0.270 | -0.0251 (0.0014) | 1.0E-71 | -0.0004 (0.0091) | 0.964 | 320.7 | 0.016 |
| Whole body fat-free mass | Alzheimer's disease (Bellenguez 2022) | rs77165542 | 2:430975 | T/C | 0.035 | -0.0585 (0.0034) | 6.6E-67 | 0.0232 (0.0223) | 0.298 | 298.6 | -0.397 |
| Whole body fat-free mass | Alzheimer's disease (Bellenguez 2022) | rs2005172 | 17:61996255 | C/A | 0.640 | 0.0226 (0.0013) | 1.0E-66 | -0.0188 (0.0086) | 0.029 | 297.7 | -0.832 |
| Whole body fat-free mass | Alzheimer's disease (Bellenguez 2022) | rs4788218 | 16:30055750 | C/T | 0.401 | 0.0218 (0.0013) | 3.6E-66 | -0.0558 (0.0084) | 3.6E-11 | 295.2 | -2.561 |
| Whole body fat-free mass | Alzheimer's disease (Bellenguez 2022) | rs4369779 | 18:20735408 | C/T | 0.789 | 0.0260 (0.0015) | 2.6E-65 | -0.0045 (0.0099) | 0.647 | 291.3 | -0.173 |
| Whole body fat-free mass | Alzheimer's disease (Bellenguez 2022) | rs4240326 | 4:145839264 | G/A | 0.550 | -0.0212 (0.0012) | 3.7E-65 | 0.0011 (0.0081) | 0.897 | 290.6 | -0.052 |
| Whole body fat-free mass | Alzheimer's disease (Bellenguez 2022) | rs4899012 | 14:61003889 | C/G | 0.607 | -0.0215 (0.0013) | 1.1E-63 | 0.0121 (0.0083) | 0.145 | 283.8 | -0.564 |
| Whole body fat-free mass | Alzheimer's disease (Bellenguez 2022) | rs527248 | 1:177875514 | G/A | 0.209 | 0.0255 (0.0015) | 2.5E-63 | 0.0034 (0.0103) | 0.742 | 282.2 | 0.133 |
| Whole body fat-free mass | Alzheimer's disease (Bellenguez 2022) | rs76798800 | 1:154994978 | T/G | 0.266 | 0.0228 (0.0014) | 2.3E-59 | -0.0087 (0.0093) | 0.348 | 264.0 | -0.382 |
| Whole body fat-free mass | Alzheimer's disease (Bellenguez 2022) | rs9634212 | 12:93993266 | A/C | 0.221 | 0.0237 (0.0015) | 4.0E-56 | 0.0072 (0.0099) | 0.466 | 249.2 | 0.304 |
| Whole body fat-free mass | Alzheimer's disease (Bellenguez 2022) | rs9398171 | 6:108983527 | T/C | 0.711 | 0.0208 (0.0014) | 1.8E-52 | 0.0219 (0.0088) | 0.013 | 232.4 | 1.053 |
| Whole body fat-free mass | Alzheimer's disease (Bellenguez 2022) | rs34879158 | 20:32300634 | C/A | 0.263 | -0.0215 (0.0014) | 2.2E-52 | 0.0100 (0.0094) | 0.291 | 232.0 | -0.464 |
| Whole body fat-free mass | Alzheimer's disease (Bellenguez 2022) | rs2524137 | 6:31264582 | T/C | 0.696 | -0.0206 (0.0014) | 2.3E-52 | 0.0013 (0.0091) | 0.884 | 231.9 | -0.063 |
| Whole body fat-free mass | Alzheimer's disease (Bellenguez 2022) | rs2533879 | 7:2859847 | A/G | 0.300 | -0.0206 (0.0014) | 3.4E-52 | 0.0185 (0.0088) | 0.036 | 231.1 | -0.899 |
| Whole body fat-free mass | Alzheimer's disease (Bellenguez 2022) | rs7033487 | 9:119129257 | C/T | 0.198 | -0.0235 (0.0016) | 4.5E-51 | -0.0204 (0.0105) | 0.053 | 226.0 | 0.869 |
| Whole body fat-free mass | Alzheimer's disease (Bellenguez 2022) | rs1582931 | 5:122657199 | A/G | 0.473 | -0.0188 (0.0013) | 8.7E-51 | 0.0106 (0.0082) | 0.198 | 224.7 | -0.565 |
| Whole body fat-free mass | Alzheimer's disease (Bellenguez 2022) | rs1260326 | 2:27730940 | C/T | 0.604 | 0.0189 (0.0013) | 1.1E-50 | -0.0183 (0.0082) | 0.026 | 224.2 | -0.967 |
| Whole body fat-free mass | Alzheimer's disease (Bellenguez 2022) | rs41271299 | 6:19839415 | T/C | 0.051 | 0.0419 (0.0028) | 2.3E-50 | -0.0015 (0.0192) | 0.936 | 222.7 | -0.036 |
| Whole body fat-free mass | Alzheimer's disease (Bellenguez 2022) | rs7952436 | 11:67024534 | T/C | 0.082 | -0.0336 (0.0023) | 6.7E-50 | 0.0271 (0.0157) | 0.084 | 220.6 | -0.806 |
| Whole body fat-free mass | Alzheimer's disease (Bellenguez 2022) | rs894360 | 8:135601194 | C/T | 0.366 | -0.0190 (0.0013) | 5.7E-49 | 0.0016 (0.0084) | 0.850 | 216.3 | -0.084 |
| Whole body fat-free mass | Alzheimer's disease (Bellenguez 2022) | rs11243202 | 6:7719065 | C/T | 0.486 | 0.0183 (0.0012) | 6.3E-49 | 0.0116 (0.0082) | 0.156 | 216.2 | 0.634 |
| Whole body fat-free mass | Alzheimer's disease (Bellenguez 2022) | rs6946415 | 7:150684548 | G/A | 0.627 | 0.0189 (0.0013) | 6.3E-49 | 0.0100 (0.0084) | 0.232 | 216.1 | 0.529 |
| Whole body fat-free mass | Alzheimer's disease (Bellenguez 2022) | rs2885697 | 1:41544279 | T/G | 0.665 | -0.0189 (0.0013) | 5.1E-47 | 0.0052 (0.0086) | 0.545 | 207.4 | -0.276 |
| Whole body fat-free mass | Alzheimer's disease (Bellenguez 2022) | rs72885917 | 2:172416376 | C/A | 0.247 | -0.0205 (0.0014) | 2.2E-46 | 0.0012 (0.0096) | 0.896 | 204.4 | -0.059 |
| Whole body fat-free mass | Alzheimer's disease (Bellenguez 2022) | rs78378222 | 17:7571752 | G/T | 0.012 | 0.0820 (0.0058) | 7.4E-46 | -0.0643 (0.0383) | 0.093 | 202.1 | -0.784 |
| Whole body fat-free mass | Alzheimer's disease (Bellenguez 2022) | rs73175572 | 3:185490184 | G/A | 0.112 | 0.0280 (0.0020) | 6.1E-45 | 0.0172 (0.0126) | 0.172 | 197.9 | 0.615 |
| Whole body fat-free mass | Alzheimer's disease (Bellenguez 2022) | rs28620532 | 9:98216876 | G/A | 0.349 | 0.0182 (0.0013) | 6.4E-44 | 0.0064 (0.0086) | 0.456 | 193.2 | 0.352 |
| Whole body fat-free mass | Alzheimer's disease (Bellenguez 2022) | rs7842996 | 8:78107140 | A/T | 0.284 | 0.0192 (0.0014) | 7.7E-44 | -0.0128 (0.0091) | 0.158 | 192.8 | -0.668 |
| Whole body fat-free mass | Alzheimer's disease (Bellenguez 2022) | rs7129320 | 11:68388220 | A/G | 0.166 | -0.0231 (0.0017) | 1.1E-43 | -0.0109 (0.0111) | 0.327 | 192.1 | 0.471 |
| Whole body fat-free mass | Alzheimer's disease (Bellenguez 2022) | rs71385734 | 16:2160503 | G/T | 0.170 | -0.0231 (0.0017) | 1.2E-43 | 0.0196 (0.0111) | 0.077 | 191.9 | -0.850 |
| Whole body fat-free mass | Alzheimer's disease (Bellenguez 2022) | rs146851424 | 13:50377910 | C/A | 0.022 | 0.0597 (0.0043) | 1.6E-43 | -0.0028 (0.0276) | 0.920 | 191.3 | -0.047 |
| Whole body fat-free mass | Alzheimer's disease (Bellenguez 2022) | rs62372052 | 5:42724294 | G/A | 0.110 | 0.0266 (0.0020) | 6.8E-41 | -0.0233 (0.0137) | 0.089 | 179.3 | -0.875 |
| Whole body fat-free mass | Alzheimer's disease (Bellenguez 2022) | rs1478575 | 2:218278555 | A/T | 0.684 | 0.0175 (0.0013) | 1.7E-39 | 0.0128 (0.0087) | 0.140 | 172.9 | 0.730 |
| Whole body fat-free mass | Alzheimer's disease (Bellenguez 2022) | rs2071286 | 6:32179896 | T/C | 0.182 | -0.0209 (0.0016) | 4.2E-39 | -0.0033 (0.0099) | 0.743 | 171.1 | 0.158 |
| Whole body fat-free mass | Alzheimer's disease (Bellenguez 2022) | rs2101975 | 4:106216667 | G/A | 0.432 | -0.0164 (0.0013) | 7.4E-39 | 0.0033 (0.0082) | 0.689 | 170.0 | -0.202 |
| Whole body fat-free mass | Alzheimer's disease (Bellenguez 2022) | rs62515437 | 8:57160328 | T/G | 0.225 | 0.0193 (0.0015) | 1.4E-38 | -0.0025 (0.0097) | 0.794 | 168.7 | -0.129 |
| Whole body fat-free mass | Alzheimer's disease (Bellenguez 2022) | rs2307111 | 5:75003678 | C/T | 0.395 | -0.0164 (0.0013) | 2.1E-38 | 0.0075 (0.0083) | 0.363 | 167.9 | -0.456 |
| Whole body fat-free mass | Alzheimer's disease (Bellenguez 2022) | rs1573891 | 15:99186488 | C/G | 0.158 | -0.0220 (0.0017) | 7.5E-38 | 0.0185 (0.0112) | 0.099 | 165.4 | -0.840 |
| Whole body fat-free mass | Alzheimer's disease (Bellenguez 2022) | rs36000545 | 17:79093822 | G/A | 0.396 | -0.0166 (0.0013) | 8.2E-38 | -0.0024 (0.0085) | 0.777 | 165.2 | 0.145 |
| Whole body fat-free mass | Alzheimer's disease (Bellenguez 2022) | rs485554 | 3:172137343 | C/G | 0.315 | 0.0171 (0.0013) | 1.8E-37 | -0.0003 (0.0089) | 0.973 | 163.7 | -0.018 |
| Whole body fat-free mass | Alzheimer's disease (Bellenguez 2022) | rs3810291 | 19:47569003 | A/G | 0.675 | 0.0170 (0.0013) | 2.4E-37 | -0.0088 (0.0094) | 0.344 | 163.1 | -0.519 |
| Whole body fat-free mass | Alzheimer's disease (Bellenguez 2022) | rs1047891 | 2:211540507 | A/C | 0.316 | 0.0169 (0.0013) | 6.0E-37 | -0.0040 (0.0088) | 0.648 | 161.3 | -0.237 |
| Whole body fat-free mass | Alzheimer's disease (Bellenguez 2022) | rs72892910 | 6:50816887 | T/G | 0.172 | 0.0207 (0.0016) | 2.5E-36 | -0.0001 (0.0108) | 0.990 | 158.4 | -0.005 |
| Whole body fat-free mass | Alzheimer's disease (Bellenguez 2022) | rs11030112 | 11:27705188 | A/G | 0.319 | 0.0167 (0.0013) | 3.6E-36 | 0.0017 (0.0088) | 0.843 | 157.7 | 0.102 |
| Whole body fat-free mass | Alzheimer's disease (Bellenguez 2022) | rs59985551 | 2:56106928 | T/C | 0.226 | -0.0185 (0.0015) | 8.6E-36 | -0.0112 (0.0096) | 0.241 | 156.0 | 0.607 |
| Whole body fat-free mass | Alzheimer's disease (Bellenguez 2022) | rs4282339 | 5:168256240 | A/G | 0.208 | -0.0189 (0.0015) | 2.4E-35 | 0.0008 (0.0099) | 0.938 | 153.9 | -0.042 |
| Whole body fat-free mass | Alzheimer's disease (Bellenguez 2022) | rs7132908 | 12:50263148 | A/G | 0.384 | 0.0158 (0.0013) | 2.9E-35 | 0.0139 (0.0083) | 0.095 | 153.6 | 0.879 |
| Whole body fat-free mass | Alzheimer's disease (Bellenguez 2022) | rs12713004 | 2:23896049 | G/A | 0.725 | 0.0172 (0.0014) | 3.9E-35 | 0.0034 (0.0091) | 0.707 | 153.0 | 0.198 |
| Whole body fat-free mass | Alzheimer's disease (Bellenguez 2022) | rs10773172 | 12:122939655 | A/G | 0.742 | 0.0175 (0.0014) | 6.6E-35 | 0.0282 (0.0093) | 0.002 | 151.9 | 1.612 |
| Whole body fat-free mass | Alzheimer's disease (Bellenguez 2022) | rs35506085 | 11:2165576 | A/G | 0.185 | -0.0198 (0.0016) | 8.1E-35 | -0.0115 (0.0103) | 0.265 | 151.5 | 0.580 |
| Whole body fat-free mass | Alzheimer's disease (Bellenguez 2022) | rs25849 | 16:2265071 | G/C | 0.289 | 0.0169 (0.0014) | 1.3E-34 | 0.0061 (0.0094) | 0.514 | 150.5 | 0.361 |
| Whole body fat-free mass | Alzheimer's disease (Bellenguez 2022) | rs2897968 | 12:46763686 | A/G | 0.607 | 0.0156 (0.0013) | 1.4E-34 | -0.0023 (0.0083) | 0.781 | 150.4 | -0.147 |
| Whole body fat-free mass | Alzheimer's disease (Bellenguez 2022) | rs3853252 | 6:152170247 | A/G | 0.455 | 0.0153 (0.0012) | 1.7E-34 | 0.0016 (0.0082) | 0.845 | 150.0 | 0.105 |
| Whole body fat-free mass | Alzheimer's disease (Bellenguez 2022) | rs9372837 | 6:126638124 | A/G | 0.471 | 0.0152 (0.0013) | 6.8E-34 | 0.0053 (0.0081) | 0.516 | 147.3 | 0.349 |
| Whole body fat-free mass | Alzheimer's disease (Bellenguez 2022) | rs12314162 | 12:124826676 | T/C | 0.161 | 0.0204 (0.0017) | 1.5E-33 | -0.0206 (0.0110) | 0.061 | 145.7 | -1.011 |
| Whole body fat-free mass | Alzheimer's disease (Bellenguez 2022) | rs72801843 | 16:53508802 | A/T | 0.301 | 0.0162 (0.0014) | 3.9E-33 | -0.0139 (0.0088) | 0.112 | 143.8 | -0.856 |
| Whole body fat-free mass | Alzheimer's disease (Bellenguez 2022) | rs9892365 | 17:59491384 | G/A | 0.670 | -0.0158 (0.0013) | 8.9E-33 | 0.0038 (0.0086) | 0.658 | 142.2 | -0.241 |
| Whole body fat-free mass | Alzheimer's disease (Bellenguez 2022) | rs34776209 | 7:23513093 | T/C | 0.247 | -0.0171 (0.0014) | 1.5E-32 | 0.0222 (0.0094) | 0.019 | 141.1 | -1.297 |
| Whole body fat-free mass | Alzheimer's disease (Bellenguez 2022) | rs244711 | 5:176509193 | T/C | 0.686 | 0.0168 (0.0014) | 1.2E-31 | -0.0318 (0.0091) | 4.9E-04 | 136.9 | -1.896 |
| Whole body fat-free mass | Alzheimer's disease (Bellenguez 2022) | rs13043303 | 20:51091385 | A/G | 0.183 | -0.0186 (0.0016) | 6.2E-31 | -0.0078 (0.0105) | 0.458 | 133.7 | 0.419 |
| Whole body fat-free mass | Alzheimer's disease (Bellenguez 2022) | rs41311445 | 22:42070374 | C/A | 0.096 | -0.0244 (0.0021) | 1.6E-30 | 0.0081 (0.0137) | 0.552 | 131.9 | -0.333 |
| Whole body fat-free mass | Alzheimer's disease (Bellenguez 2022) | rs2531991 | 16:4023553 | A/G | 0.747 | 0.0164 (0.0014) | 2.3E-30 | -0.0197 (0.0094) | 0.037 | 131.1 | -1.200 |
| Whole body fat-free mass | Alzheimer's disease (Bellenguez 2022) | rs6235 | 5:95728898 | G/C | 0.268 | 0.0159 (0.0014) | 8.3E-30 | -0.0009 (0.0092) | 0.923 | 128.6 | -0.057 |
| Whole body fat-free mass | Alzheimer's disease (Bellenguez 2022) | rs11014285 | 10:25178864 | A/G | 0.165 | 0.0192 (0.0017) | 1.1E-29 | -0.0126 (0.0113) | 0.263 | 128.1 | -0.658 |
| Whole body fat-free mass | Alzheimer's disease (Bellenguez 2022) | rs1022523 | 1:176788394 | A/G | 0.727 | 0.0156 (0.0014) | 4.4E-29 | -0.0249 (0.0092) | 0.007 | 125.3 | -1.600 |
| Whole body fat-free mass | Alzheimer's disease (Bellenguez 2022) | rs12072845 | 1:214630757 | A/G | 0.395 | -0.0140 (0.0013) | 2.8E-28 | -0.0073 (0.0083) | 0.382 | 121.7 | 0.523 |
| Whole body fat-free mass | Alzheimer's disease (Bellenguez 2022) | rs11880992 | 19:2176403 | A/G | 0.408 | 0.0139 (0.0013) | 4.6E-28 | 0.0013 (0.0087) | 0.883 | 120.6 | 0.093 |
| Whole body fat-free mass | Alzheimer's disease (Bellenguez 2022) | rs2270894 | 3:9975386 | G/C | 0.203 | -0.0174 (0.0016) | 1.0E-27 | -0.0147 (0.0105) | 0.163 | 119.0 | 0.843 |
| Whole body fat-free mass | Alzheimer's disease (Bellenguez 2022) | rs111640872 | 19:30290357 | C/G | 0.331 | 0.0145 (0.0013) | 1.1E-27 | -0.0039 (0.0093) | 0.676 | 118.9 | -0.269 |
| Whole body fat-free mass | Alzheimer's disease (Bellenguez 2022) | rs6800021 | 3:50190346 | A/G | 0.425 | 0.0136 (0.0013) | 1.6E-27 | -0.0081 (0.0082) | 0.325 | 118.1 | -0.594 |
| Whole body fat-free mass | Alzheimer's disease (Bellenguez 2022) | rs11065979 | 12:112059557 | T/C | 0.438 | -0.0135 (0.0013) | 4.3E-27 | -0.0083 (0.0082) | 0.309 | 116.2 | 0.615 |
| Whole body fat-free mass | Alzheimer's disease (Bellenguez 2022) | rs17277008 | 1:172105162 | C/T | 0.313 | 0.0144 (0.0013) | 4.6E-27 | 0.0009 (0.0088) | 0.919 | 116.1 | 0.063 |
| Whole body fat-free mass | Alzheimer's disease (Bellenguez 2022) | rs2900208 | 12:11878464 | A/C | 0.354 | 0.0140 (0.0013) | 4.9E-27 | 0.0020 (0.0086) | 0.814 | 115.9 | 0.143 |
| Whole body fat-free mass | Alzheimer's disease (Bellenguez 2022) | rs12657771 | 5:36787962 | A/G | 0.439 | -0.0134 (0.0013) | 9.4E-27 | -0.0030 (0.0082) | 0.714 | 114.7 | 0.223 |
| Whole body fat-free mass | Alzheimer's disease (Bellenguez 2022) | rs12879423 | 14:25927832 | G/A | 0.679 | 0.0142 (0.0013) | 3.4E-26 | -0.0059 (0.0087) | 0.497 | 112.1 | -0.416 |
| Whole body fat-free mass | Alzheimer's disease (Bellenguez 2022) | rs6762578 | 3:128992047 | A/G | 0.778 | 0.0157 (0.0015) | 8.9E-26 | -0.0150 (0.0100) | 0.133 | 110.2 | -0.954 |
| Whole body fat-free mass | Alzheimer's disease (Bellenguez 2022) | rs7141420 | 14:79899454 | T/C | 0.516 | 0.0131 (0.0013) | 1.2E-25 | -0.0043 (0.0081) | 0.594 | 109.5 | -0.328 |
| Whole body fat-free mass | Alzheimer's disease (Bellenguez 2022) | rs3822742 | 5:139059017 | A/C | 0.371 | 0.0134 (0.0013) | 1.6E-25 | -0.0082 (0.0085) | 0.333 | 109.0 | -0.610 |
| Whole body fat-free mass | Alzheimer's disease (Bellenguez 2022) | rs8067974 | 17:43232566 | G/A | 0.329 | -0.0138 (0.0013) | 1.9E-25 | -0.0094 (0.0086) | 0.273 | 108.7 | 0.680 |
| Whole body fat-free mass | Alzheimer's disease (Bellenguez 2022) | rs12041740 | 1:11236524 | A/G | 0.740 | -0.0147 (0.0014) | 2.1E-25 | -0.0006 (0.0093) | 0.947 | 108.5 | 0.041 |
| Whole body fat-free mass | Alzheimer's disease (Bellenguez 2022) | rs12095997 | 1:51391845 | T/C | 0.089 | 0.0225 (0.0022) | 2.7E-25 | -0.0377 (0.0140) | 0.007 | 108.0 | -1.674 |
| Whole body fat-free mass | Alzheimer's disease (Bellenguez 2022) | rs6821305 | 4:122713863 | C/A | 0.399 | 0.0131 (0.0013) | 3.3E-25 | -0.0030 (0.0083) | 0.717 | 107.6 | -0.229 |
| Whole body fat-free mass | Alzheimer's disease (Bellenguez 2022) | rs6570509 | 6:142716286 | T/G | 0.287 | -0.0142 (0.0014) | 3.9E-25 | 0.0065 (0.0088) | 0.462 | 107.3 | -0.459 |
| Whole body fat-free mass | Alzheimer's disease (Bellenguez 2022) | rs7080472 | 10:96012950 | T/G | 0.425 | 0.0130 (0.0013) | 5.2E-25 | 0.0103 (0.0082) | 0.208 | 106.7 | 0.792 |
| Whole body fat-free mass | Alzheimer's disease (Bellenguez 2022) | rs1924936 | 13:78443297 | A/T | 0.774 | 0.0153 (0.0015) | 7.8E-25 | -0.0032 (0.0097) | 0.741 | 105.9 | -0.209 |
| Whole body fat-free mass | Alzheimer's disease (Bellenguez 2022) | rs10283100 | 8:120596023 | G/A | 0.945 | 0.0279 (0.0027) | 8.7E-25 | -0.0223 (0.0175) | 0.202 | 105.7 | -0.799 |
| Whole body fat-free mass | Alzheimer's disease (Bellenguez 2022) | rs11240565 | 1:205722958 | T/C | 0.398 | 0.0130 (0.0013) | 8.8E-25 | 0.0085 (0.0083) | 0.307 | 105.6 | 0.655 |
| Whole body fat-free mass | Alzheimer's disease (Bellenguez 2022) | rs365352 | 5:77401152 | A/G | 0.244 | -0.0148 (0.0014) | 9.3E-25 | 0.0078 (0.0096) | 0.416 | 105.5 | -0.527 |
| Whole body fat-free mass | Alzheimer's disease (Bellenguez 2022) | rs9938120 | 16:19977733 | T/C | 0.144 | -0.0184 (0.0018) | 9.4E-25 | -0.0396 (0.0119) | 8.4E-04 | 105.5 | 2.149 |
| Whole body fat-free mass | Alzheimer's disease (Bellenguez 2022) | rs9350850 | 6:81050236 | C/T | 0.079 | 0.0234 (0.0023) | 1.5E-24 | -0.0124 (0.0157) | 0.430 | 104.6 | -0.530 |
| Whole body fat-free mass | Alzheimer's disease (Bellenguez 2022) | rs946197 | 1:56602308 | C/G | 0.232 | 0.0150 (0.0015) | 1.6E-24 | -0.0076 (0.0095) | 0.422 | 104.5 | -0.506 |
| Whole body fat-free mass | Alzheimer's disease (Bellenguez 2022) | rs11613704 | 12:980925 | A/C | 0.200 | 0.0158 (0.0015) | 2.1E-24 | 0.0207 (0.0105) | 0.048 | 103.9 | 1.310 |
| Whole body fat-free mass | Alzheimer's disease (Bellenguez 2022) | rs12193797 | 6:41748352 | G/A | 0.133 | -0.0185 (0.0018) | 2.9E-24 | -0.0198 (0.0123) | 0.106 | 103.3 | 1.068 |
| Whole body fat-free mass | Alzheimer's disease (Bellenguez 2022) | rs2252720 | 20:21223663 | T/C | 0.674 | -0.0135 (0.0013) | 4.6E-24 | 0.0024 (0.0087) | 0.786 | 102.4 | -0.177 |
| Whole body fat-free mass | Alzheimer's disease (Bellenguez 2022) | rs10119967 | 9:99268421 | C/A | 0.204 | 0.0156 (0.0015) | 5.9E-24 | -0.0059 (0.0102) | 0.560 | 101.9 | -0.378 |
| Whole body fat-free mass | Alzheimer's disease (Bellenguez 2022) | rs2163832 | 19:10745764 | C/T | 0.660 | -0.0133 (0.0013) | 6.4E-24 | 0.0088 (0.0092) | 0.338 | 101.7 | -0.663 |
| Whole body fat-free mass | Alzheimer's disease (Bellenguez 2022) | rs676105 | 11:30443688 | C/T | 0.304 | 0.0136 (0.0014) | 7.8E-24 | -0.0219 (0.0089) | 0.014 | 101.3 | -1.607 |
| Whole body fat-free mass | Alzheimer's disease (Bellenguez 2022) | rs9480933 | 6:109671391 | G/C | 0.350 | -0.0130 (0.0013) | 9.3E-24 | 0.0127 (0.0085) | 0.134 | 101.0 | -0.973 |
| Whole body fat-free mass | Alzheimer's disease (Bellenguez 2022) | rs11648796 | 16:792190 | G/A | 0.232 | 0.0154 (0.0015) | 1.4E-23 | -0.0106 (0.0100) | 0.290 | 100.1 | -0.686 |
| Whole body fat-free mass | Alzheimer's disease (Bellenguez 2022) | rs2265309 | 10:104487871 | C/T | 0.517 | -0.0124 (0.0012) | 1.5E-23 | -0.0032 (0.0081) | 0.691 | 100.0 | 0.257 |
| Whole body fat-free mass | Alzheimer's disease (Bellenguez 2022) | rs13271368 | 8:126506140 | T/C | 0.237 | -0.0148 (0.0015) | 2.0E-23 | 0.0330 (0.0098) | 7.7E-04 | 99.4 | -2.236 |
| Whole body fat-free mass | Alzheimer's disease (Bellenguez 2022) | rs7815955 | 8:130719567 | T/A | 0.203 | -0.0152 (0.0015) | 7.8E-23 | 0.0191 (0.0100) | 0.057 | 96.8 | -1.257 |
| Whole body fat-free mass | Alzheimer's disease (Bellenguez 2022) | rs6779752 | 3:85663849 | A/G | 0.638 | -0.0128 (0.0013) | 8.5E-23 | 0.0033 (0.0084) | 0.692 | 96.6 | -0.259 |
| Whole body fat-free mass | Alzheimer's disease (Bellenguez 2022) | rs111365325 | 5:170865229 | T/C | 0.231 | -0.0145 (0.0015) | 9.6E-23 | 0.0268 (0.0097) | 0.006 | 96.3 | -1.854 |
| Whole body fat-free mass | Alzheimer's disease (Bellenguez 2022) | rs33973388 | 18:46611842 | T/G | 0.435 | 0.0123 (0.0013) | 9.7E-23 | 0.0063 (0.0082) | 0.440 | 96.3 | 0.511 |
| Whole body fat-free mass | Alzheimer's disease (Bellenguez 2022) | rs11578046 | 1:23425139 | A/G | 0.327 | -0.0129 (0.0013) | 1.1E-22 | 0.0065 (0.0086) | 0.454 | 96.1 | -0.503 |
| Whole body fat-free mass | Alzheimer's disease (Bellenguez 2022) | rs67551338 | 12:3393100 | T/C | 0.061 | 0.0255 (0.0026) | 1.4E-22 | -0.0297 (0.0171) | 0.083 | 95.6 | -1.163 |
| Whole body fat-free mass | Alzheimer's disease (Bellenguez 2022) | rs3809570 | 15:67000117 | A/C | 0.243 | 0.0142 (0.0015) | 2.1E-22 | 0.0060 (0.0093) | 0.519 | 94.8 | 0.424 |
| Whole body fat-free mass | Alzheimer's disease (Bellenguez 2022) | rs16942324 | 15:89383854 | A/C | 0.027 | -0.0373 (0.0038) | 2.2E-22 | -0.0160 (0.0256) | 0.532 | 94.7 | 0.429 |
| Whole body fat-free mass | Alzheimer's disease (Bellenguez 2022) | rs34848742 | 4:123828042 | G/T | 0.788 | -0.0148 (0.0015) | 2.3E-22 | 0.0030 (0.0098) | 0.759 | 94.7 | -0.203 |
| Whole body fat-free mass | Alzheimer's disease (Bellenguez 2022) | rs13240065 | 7:73015369 | A/G | 0.128 | 0.0180 (0.0019) | 2.9E-22 | 0.0076 (0.0126) | 0.547 | 94.2 | 0.423 |
| Whole body fat-free mass | Alzheimer's disease (Bellenguez 2022) | rs12907384 | 15:86276000 | C/T | 0.532 | -0.0120 (0.0012) | 4.7E-22 | -0.0001 (0.0082) | 0.991 | 93.2 | 0.008 |
| Whole body fat-free mass | Alzheimer's disease (Bellenguez 2022) | rs73052033 | 3:185828465 | C/T | 0.185 | -0.0154 (0.0016) | 5.2E-22 | -0.0143 (0.0107) | 0.180 | 93.0 | 0.928 |
| Whole body fat-free mass | Alzheimer's disease (Bellenguez 2022) | rs7780752 | 7:93241640 | C/T | 0.360 | 0.0124 (0.0013) | 5.4E-22 | -0.0059 (0.0085) | 0.485 | 92.9 | -0.474 |
| Whole body fat-free mass | Alzheimer's disease (Bellenguez 2022) | rs17066856 | 18:58049656 | C/T | 0.092 | -0.0208 (0.0022) | 5.6E-22 | -0.0134 (0.0139) | 0.333 | 92.9 | 0.643 |
| Whole body fat-free mass | Alzheimer's disease (Bellenguez 2022) | rs9291926 | 5:67599656 | G/T | 0.532 | -0.0120 (0.0012) | 6.1E-22 | -0.0112 (0.0082) | 0.171 | 92.7 | 0.936 |
| Whole body fat-free mass | Alzheimer's disease (Bellenguez 2022) | rs2721938 | 8:116635611 | T/C | 0.600 | -0.0121 (0.0013) | 1.3E-21 | 0.0160 (0.0083) | 0.055 | 91.2 | -1.319 |
| Whole body fat-free mass | Alzheimer's disease (Bellenguez 2022) | rs13430869 | 2:218146818 | T/G | 0.742 | 0.0135 (0.0014) | 1.4E-21 | -0.0003 (0.0091) | 0.976 | 91.1 | -0.022 |
| Whole body fat-free mass | Alzheimer's disease (Bellenguez 2022) | rs62621197 | 19:8670147 | T/C | 0.037 | -0.0326 (0.0034) | 1.4E-21 | 0.0303 (0.0236) | 0.200 | 91.0 | -0.930 |
| Whole body fat-free mass | Alzheimer's disease (Bellenguez 2022) | rs10938397 | 4:45182527 | G/A | 0.434 | 0.0119 (0.0013) | 1.7E-21 | -0.0052 (0.0082) | 0.521 | 90.6 | -0.436 |
| Whole body fat-free mass | Alzheimer's disease (Bellenguez 2022) | rs10775348 | 16:88806348 | G/A | 0.704 | 0.0130 (0.0014) | 2.0E-21 | -0.0007 (0.0091) | 0.938 | 90.4 | -0.054 |
| Whole body fat-free mass | Alzheimer's disease (Bellenguez 2022) | rs11042725 | 11:10325325 | A/C | 0.488 | -0.0117 (0.0012) | 3.1E-21 | 0.0041 (0.0081) | 0.614 | 89.5 | -0.349 |
| Whole body fat-free mass | Alzheimer's disease (Bellenguez 2022) | rs10748128 | 12:69827658 | T/G | 0.345 | 0.0123 (0.0013) | 3.2E-21 | 0.0077 (0.0085) | 0.368 | 89.5 | 0.623 |
| Whole body fat-free mass | Alzheimer's disease (Bellenguez 2022) | rs7619139 | 3:25110415 | A/T | 0.589 | 0.0119 (0.0013) | 3.4E-21 | -0.0022 (0.0083) | 0.795 | 89.3 | -0.184 |
| Whole body fat-free mass | Alzheimer's disease (Bellenguez 2022) | rs247008 | 5:131447104 | G/A | 0.670 | 0.0125 (0.0013) | 3.6E-21 | -0.0135 (0.0086) | 0.117 | 89.2 | -1.077 |
| Whole body fat-free mass | Alzheimer's disease (Bellenguez 2022) | rs62621812 | 7:127015083 | A/G | 0.020 | 0.0425 (0.0045) | 5.9E-21 | 0.0537 (0.0278) | 0.053 | 88.2 | 1.263 |
| Whole body fat-free mass | Alzheimer's disease (Bellenguez 2022) | rs67408364 | 1:39618556 | G/A | 0.214 | 0.0142 (0.0015) | 6.0E-21 | 0.0095 (0.0099) | 0.338 | 88.2 | 0.670 |
| Whole body fat-free mass | Alzheimer's disease (Bellenguez 2022) | rs4968799 | 17:68158831 | T/A | 0.112 | -0.0185 (0.0020) | 7.3E-21 | 0.0046 (0.0130) | 0.725 | 87.8 | -0.249 |
| Whole body fat-free mass | Alzheimer's disease (Bellenguez 2022) | rs2726036 | 16:28347140 | C/A | 0.398 | 0.0119 (0.0013) | 8.0E-21 | -0.0109 (0.0084) | 0.197 | 87.6 | -0.916 |
| Whole body fat-free mass | Alzheimer's disease (Bellenguez 2022) | rs2952615 | 5:112138888 | C/G | 0.618 | -0.0119 (0.0013) | 8.9E-21 | 0.0076 (0.0084) | 0.366 | 87.4 | -0.637 |
| Whole body fat-free mass | Alzheimer's disease (Bellenguez 2022) | rs17246129 | 2:227259964 | A/G | 0.305 | 0.0125 (0.0013) | 1.4E-20 | -0.0138 (0.0088) | 0.116 | 86.5 | -1.102 |
| Whole body fat-free mass | Alzheimer's disease (Bellenguez 2022) | rs9461246 | 6:26381660 | A/G | 0.118 | -0.0178 (0.0019) | 1.5E-20 | 0.0202 (0.0123) | 0.102 | 86.3 | -1.136 |
| Whole body fat-free mass | Alzheimer's disease (Bellenguez 2022) | rs1064213 | 2:198950240 | A/G | 0.478 | 0.0115 (0.0012) | 1.8E-20 | -0.0072 (0.0081) | 0.371 | 86.0 | -0.627 |
| Whole body fat-free mass | Alzheimer's disease (Bellenguez 2022) | rs2015561 | 17:38593294 | G/A | 0.424 | -0.0117 (0.0013) | 1.9E-20 | 0.0162 (0.0082) | 0.050 | 85.9 | -1.390 |
| Whole body fat-free mass | Alzheimer's disease (Bellenguez 2022) | rs1542224 | 2:223963874 | C/T | 0.719 | 0.0128 (0.0014) | 2.0E-20 | -0.0003 (0.0090) | 0.973 | 85.8 | -0.024 |
| Whole body fat-free mass | Alzheimer's disease (Bellenguez 2022) | rs2678204 | 1:201800511 | G/T | 0.340 | 0.0121 (0.0013) | 2.4E-20 | -0.0137 (0.0087) | 0.116 | 85.4 | -1.135 |
| Whole body fat-free mass | Alzheimer's disease (Bellenguez 2022) | rs3212260 | 14:24804088 | T/A | 0.258 | 0.0131 (0.0014) | 3.6E-20 | 0.0040 (0.0094) | 0.674 | 84.6 | 0.305 |
| Whole body fat-free mass | Alzheimer's disease (Bellenguez 2022) | rs663344 | 9:98316331 | C/G | 0.181 | -0.0151 (0.0016) | 3.7E-20 | -0.0058 (0.0108) | 0.593 | 84.6 | 0.385 |
| Whole body fat-free mass | Alzheimer's disease (Bellenguez 2022) | rs6585827 | 10:124165615 | A/G | 0.471 | 0.0114 (0.0012) | 3.8E-20 | -0.0379 (0.0081) | 2.9E-06 | 84.5 | -3.316 |
| Whole body fat-free mass | Alzheimer's disease (Bellenguez 2022) | rs1910252 | 8:49407362 | T/C | 0.168 | 0.0153 (0.0017) | 3.9E-20 | 0.0075 (0.0107) | 0.483 | 84.5 | 0.490 |
| Whole body fat-free mass | Alzheimer's disease (Bellenguez 2022) | rs703593 | 12:103064247 | G/A | 0.482 | -0.0114 (0.0012) | 4.1E-20 | -0.0045 (0.0081) | 0.585 | 84.4 | 0.395 |
| Whole body fat-free mass | Alzheimer's disease (Bellenguez 2022) | rs55831773 | 17:7559037 | T/C | 0.199 | -0.0146 (0.0016) | 4.1E-20 | 0.0029 (0.0104) | 0.776 | 84.4 | -0.199 |
| Whole body fat-free mass | Alzheimer's disease (Bellenguez 2022) | rs115179432 | 2:33348679 | G/A | 0.072 | -0.0221 (0.0024) | 4.5E-20 | 0.0176 (0.0156) | 0.259 | 84.2 | -0.797 |
| Whole body fat-free mass | Alzheimer's disease (Bellenguez 2022) | rs55727637 | 16:15145252 | T/C | 0.305 | -0.0123 (0.0013) | 6.1E-20 | 0.0065 (0.0088) | 0.461 | 83.6 | -0.527 |
| Whole body fat-free mass | Alzheimer's disease (Bellenguez 2022) | rs31211 | 5:134363145 | A/G | 0.251 | -0.0130 (0.0014) | 7.2E-20 | 0.0039 (0.0094) | 0.678 | 83.2 | -0.299 |
| Whole body fat-free mass | Alzheimer's disease (Bellenguez 2022) | rs2140046 | 2:169706079 | C/T | 0.364 | -0.0117 (0.0013) | 8.2E-20 | 0.0064 (0.0085) | 0.451 | 83.0 | -0.546 |
| Whole body fat-free mass | Alzheimer's disease (Bellenguez 2022) | rs3740591 | 10:70287303 | T/C | 0.445 | 0.0114 (0.0013) | 1.7E-19 | -0.0067 (0.0083) | 0.424 | 81.5 | -0.588 |
| Whole body fat-free mass | Alzheimer's disease (Bellenguez 2022) | rs4980067 | 10:81136129 | A/C | 0.505 | -0.0112 (0.0012) | 1.8E-19 | -0.0149 (0.0082) | 0.070 | 81.4 | 1.330 |
| Whole body fat-free mass | Alzheimer's disease (Bellenguez 2022) | rs1841738 | 4:88557753 | G/A | 0.518 | -0.0112 (0.0012) | 3.2E-19 | 0.0145 (0.0083) | 0.080 | 80.3 | -1.297 |
| Whole body fat-free mass | Alzheimer's disease (Bellenguez 2022) | rs10202845 | 2:42575820 | G/A | 0.113 | -0.0176 (0.0020) | 4.3E-19 | 0.0165 (0.0128) | 0.197 | 79.7 | -0.937 |
| Whole body fat-free mass | Alzheimer's disease (Bellenguez 2022) | rs11001399 | 10:77221744 | C/A | 0.559 | -0.0112 (0.0013) | 5.2E-19 | 0.0039 (0.0082) | 0.636 | 79.4 | -0.349 |
| Whole body fat-free mass | Alzheimer's disease (Bellenguez 2022) | rs843374 | 3:183997261 | T/A | 0.587 | -0.0112 (0.0013) | 5.4E-19 | 0.0111 (0.0083) | 0.181 | 79.3 | -0.990 |
| Whole body fat-free mass | Alzheimer's disease (Bellenguez 2022) | rs7706886 | 5:32722319 | A/G | 0.271 | 0.0126 (0.0014) | 6.2E-19 | 0.0077 (0.0095) | 0.421 | 79.0 | 0.611 |
| Whole body fat-free mass | Alzheimer's disease (Bellenguez 2022) | rs10803955 | 2:183228114 | G/A | 0.508 | -0.0110 (0.0012) | 6.4E-19 | -0.0149 (0.0081) | 0.066 | 79.0 | 1.357 |
| Whole body fat-free mass | Alzheimer's disease (Bellenguez 2022) | rs111391498 | 4:1341553 | G/A | 0.047 | -0.0258 (0.0029) | 6.7E-19 | 0.0337 (0.0207) | 0.103 | 78.8 | -1.304 |
| Whole body fat-free mass | Alzheimer's disease (Bellenguez 2022) | rs4865956 | 5:54882505 | A/T | 0.697 | -0.0120 (0.0014) | 7.3E-19 | -0.0055 (0.0088) | 0.534 | 78.7 | 0.458 |
| Whole body fat-free mass | Alzheimer's disease (Bellenguez 2022) | rs28547519 | 7:121964618 | C/T | 0.366 | 0.0114 (0.0013) | 8.5E-19 | -0.0021 (0.0085) | 0.801 | 78.4 | -0.184 |
| Whole body fat-free mass | Alzheimer's disease (Bellenguez 2022) | rs7647657 | 3:38573746 | G/A | 0.386 | -0.0113 (0.0013) | 9.1E-19 | 0.0070 (0.0084) | 0.400 | 78.2 | -0.620 |
| Whole body fat-free mass | Alzheimer's disease (Bellenguez 2022) | rs2602713 | 19:4910021 | C/A | 0.440 | 0.0111 (0.0013) | 1.2E-18 | -0.0020 (0.0088) | 0.825 | 77.6 | -0.179 |
| Whole body fat-free mass | Alzheimer's disease (Bellenguez 2022) | rs6874142 | 5:172753555 | G/T | 0.114 | 0.0182 (0.0021) | 1.4E-18 | 0.0053 (0.0140) | 0.707 | 77.5 | 0.291 |
| Whole body fat-free mass | Alzheimer's disease (Bellenguez 2022) | rs75786059 | 1:26449327 | G/A | 0.175 | 0.0143 (0.0016) | 1.7E-18 | 0.0041 (0.0106) | 0.699 | 77.0 | 0.287 |
| Whole body fat-free mass | Alzheimer's disease (Bellenguez 2022) | rs76364830 | 8:13372120 | A/G | 0.063 | -0.0225 (0.0026) | 2.4E-18 | 0.0418 (0.0173) | 0.016 | 76.4 | -1.860 |
| Whole body fat-free mass | Alzheimer's disease (Bellenguez 2022) | rs17556750 | 4:82155568 | A/C | 0.288 | 0.0119 (0.0014) | 3.7E-18 | 0.0009 (0.0090) | 0.921 | 75.5 | 0.076 |
| Whole body fat-free mass | Alzheimer's disease (Bellenguez 2022) | rs11712872 | 3:52835514 | A/G | 0.115 | 0.0168 (0.0019) | 4.3E-18 | -0.0021 (0.0125) | 0.865 | 75.2 | -0.125 |
| Whole body fat-free mass | Alzheimer's disease (Bellenguez 2022) | rs7728690 | 5:88411214 | T/C | 0.386 | -0.0110 (0.0013) | 5.0E-18 | 0.0033 (0.0084) | 0.692 | 74.9 | -0.300 |
| Whole body fat-free mass | Alzheimer's disease (Bellenguez 2022) | rs76513770 | 16:72505534 | C/T | 0.128 | -0.0161 (0.0019) | 5.1E-18 | 0.0156 (0.0125) | 0.213 | 74.8 | -0.970 |
| Whole body fat-free mass | Alzheimer's disease (Bellenguez 2022) | rs7994814 | 13:27054201 | T/C | 0.417 | 0.0110 (0.0013) | 5.7E-18 | 0.0210 (0.0087) | 0.016 | 74.6 | 1.903 |
| Whole body fat-free mass | Alzheimer's disease (Bellenguez 2022) | rs3116201 | 2:233074205 | A/G | 0.098 | -0.0180 (0.0021) | 7.1E-18 | 0.0045 (0.0139) | 0.744 | 74.2 | -0.251 |
| Whole body fat-free mass | Alzheimer's disease (Bellenguez 2022) | rs508347 | 7:28212824 | C/T | 0.703 | -0.0117 (0.0014) | 7.8E-18 | -0.0125 (0.0089) | 0.159 | 74.0 | 1.070 |
| Whole body fat-free mass | Alzheimer's disease (Bellenguez 2022) | rs10777860 | 12:97792792 | A/G | 0.528 | -0.0107 (0.0012) | 7.8E-18 | -0.0036 (0.0082) | 0.660 | 74.0 | 0.337 |
| Whole body fat-free mass | Alzheimer's disease (Bellenguez 2022) | rs744205 | 11:69929677 | A/G | 0.544 | 0.0107 (0.0012) | 1.0E-17 | -0.0039 (0.0082) | 0.635 | 73.5 | -0.364 |
| Whole body fat-free mass | Alzheimer's disease (Bellenguez 2022) | rs7134283 | 12:24071748 | A/G | 0.283 | -0.0118 (0.0014) | 1.2E-17 | 0.0203 (0.0091) | 0.026 | 73.2 | -1.719 |
| Whole body fat-free mass | Alzheimer's disease (Bellenguez 2022) | rs61729527 | 8:77761919 | T/C | 0.052 | -0.0238 (0.0028) | 1.9E-17 | -0.0007 (0.0190) | 0.969 | 72.3 | 0.029 |
| Whole body fat-free mass | Alzheimer's disease (Bellenguez 2022) | rs968821 | 9:94187634 | C/G | 0.340 | -0.0111 (0.0013) | 2.1E-17 | -0.0107 (0.0087) | 0.219 | 72.1 | 0.961 |
| Whole body fat-free mass | Alzheimer's disease (Bellenguez 2022) | rs9317002 | 13:59175727 | A/C | 0.515 | 0.0106 (0.0013) | 2.3E-17 | 0.0064 (0.0082) | 0.430 | 71.8 | 0.602 |
| Whole body fat-free mass | Alzheimer's disease (Bellenguez 2022) | rs9951619 | 18:56882326 | G/T | 0.767 | 0.0125 (0.0015) | 4.0E-17 | -0.0143 (0.0096) | 0.135 | 70.8 | -1.147 |
| Whole body fat-free mass | Alzheimer's disease (Bellenguez 2022) | rs3782232 | 12:57116249 | A/G | 0.071 | -0.0203 (0.0024) | 4.4E-17 | 0.0406 (0.0162) | 0.012 | 70.6 | -2.001 |
| Whole body fat-free mass | Alzheimer's disease (Bellenguez 2022) | rs12188627 | 5:60720682 | G/A | 0.488 | -0.0104 (0.0012) | 6.3E-17 | 0.0146 (0.0082) | 0.074 | 69.9 | -1.408 |
| Whole body fat-free mass | Alzheimer's disease (Bellenguez 2022) | rs11689727 | 2:25458100 | A/C | 0.331 | -0.0110 (0.0013) | 7.0E-17 | -0.0122 (0.0087) | 0.160 | 69.7 | 1.110 |
| Whole body fat-free mass | Alzheimer's disease (Bellenguez 2022) | rs7312646 | 12:102388920 | C/A | 0.521 | -0.0105 (0.0013) | 9.2E-17 | -0.0023 (0.0084) | 0.782 | 69.1 | 0.219 |
| Whole body fat-free mass | Alzheimer's disease (Bellenguez 2022) | rs6902789 | 6:105358192 | A/G | 0.368 | 0.0107 (0.0013) | 1.1E-16 | 0.0054 (0.0084) | 0.525 | 68.8 | 0.505 |
| Whole body fat-free mass | Alzheimer's disease (Bellenguez 2022) | rs4858940 | 3:88254820 | C/T | 0.886 | 0.0161 (0.0019) | 1.2E-16 | 0.0099 (0.0135) | 0.463 | 68.6 | 0.614 |
| Whole body fat-free mass | Alzheimer's disease (Bellenguez 2022) | rs2296316 | 14:65520246 | C/T | 0.465 | -0.0104 (0.0013) | 1.2E-16 | 0.0045 (0.0082) | 0.584 | 68.6 | -0.432 |
| Whole body fat-free mass | Alzheimer's disease (Bellenguez 2022) | rs112560164 | 14:93112924 | A/G | 0.191 | 0.0131 (0.0016) | 1.3E-16 | 0.0267 (0.0104) | 0.011 | 68.5 | 2.033 |
| Whole body fat-free mass | Alzheimer's disease (Bellenguez 2022) | rs9380508 | 6:35398689 | T/C | 0.775 | 0.0123 (0.0015) | 1.4E-16 | 0.0189 (0.0097) | 0.052 | 68.3 | 1.541 |
| Whole body fat-free mass | Alzheimer's disease (Bellenguez 2022) | rs1228024 | 11:47951353 | A/C | 0.661 | -0.0108 (0.0013) | 2.1E-16 | 0.0056 (0.0086) | 0.517 | 67.5 | -0.519 |
| Whole body fat-free mass | Alzheimer's disease (Bellenguez 2022) | rs310796 | 12:77453226 | T/G | 0.681 | 0.0110 (0.0013) | 2.1E-16 | -0.0123 (0.0087) | 0.160 | 67.5 | -1.122 |
| Whole body fat-free mass | Alzheimer's disease (Bellenguez 2022) | rs11794152 | 9:23345347 | G/A | 0.415 | 0.0104 (0.0013) | 2.4E-16 | -0.0066 (0.0083) | 0.427 | 67.3 | -0.637 |
| Whole body fat-free mass | Alzheimer's disease (Bellenguez 2022) | rs1412234 | 9:28410683 | C/T | 0.327 | 0.0109 (0.0013) | 2.6E-16 | 0.0095 (0.0088) | 0.281 | 67.0 | 0.874 |
| Whole body fat-free mass | Alzheimer's disease (Bellenguez 2022) | rs55674305 | 8:89435868 | A/G | 0.306 | -0.0110 (0.0014) | 3.1E-16 | 0.0038 (0.0087) | 0.667 | 66.8 | -0.344 |
| Whole body fat-free mass | Alzheimer's disease (Bellenguez 2022) | rs578366 | 6:81591034 | G/A | 0.420 | -0.0102 (0.0013) | 3.5E-16 | -0.0056 (0.0082) | 0.491 | 66.5 | 0.548 |
| Whole body fat-free mass | Alzheimer's disease (Bellenguez 2022) | rs2197563 | 2:233687080 | A/G | 0.595 | 0.0103 (0.0013) | 3.8E-16 | -0.0189 (0.0084) | 0.025 | 66.3 | -1.839 |
| Whole body fat-free mass | Alzheimer's disease (Bellenguez 2022) | rs12820906 | 12:123493123 | G/A | 0.247 | 0.0117 (0.0014) | 3.9E-16 | -0.0080 (0.0095) | 0.395 | 66.3 | -0.682 |
| Whole body fat-free mass | Alzheimer's disease (Bellenguez 2022) | rs10899736 | 7:50546925 | A/G | 0.440 | -0.0102 (0.0013) | 4.4E-16 | 0.0132 (0.0081) | 0.106 | 66.1 | -1.298 |
| Whole body fat-free mass | Alzheimer's disease (Bellenguez 2022) | rs1049193 | 12:53873844 | C/G | 0.171 | -0.0134 (0.0016) | 4.6E-16 | 0.0089 (0.0112) | 0.426 | 66.0 | -0.664 |
| Whole body fat-free mass | Alzheimer's disease (Bellenguez 2022) | rs141729694 | 5:87999371 | T/C | 0.074 | 0.0193 (0.0024) | 4.7E-16 | -0.0086 (0.0152) | 0.571 | 65.9 | -0.446 |
| Whole body fat-free mass | Alzheimer's disease (Bellenguez 2022) | rs11855017 | 15:42096146 | A/C | 0.182 | 0.0131 (0.0016) | 4.8E-16 | 0.0128 (0.0106) | 0.228 | 65.9 | 0.976 |
| Whole body fat-free mass | Alzheimer's disease (Bellenguez 2022) | rs13299559 | 9:128041828 | T/C | 0.450 | -0.0101 (0.0013) | 5.2E-16 | 0.0130 (0.0082) | 0.113 | 65.7 | -1.282 |
| Whole body fat-free mass | Alzheimer's disease (Bellenguez 2022) | rs667515 | 11:69449076 | C/G | 0.386 | -0.0104 (0.0013) | 5.9E-16 | 0.0038 (0.0086) | 0.660 | 65.5 | -0.367 |
| Whole body fat-free mass | Alzheimer's disease (Bellenguez 2022) | rs357486 | 3:153885503 | C/T | 0.543 | 0.0100 (0.0012) | 6.7E-16 | 0.0038 (0.0081) | 0.639 | 65.2 | 0.379 |
| Whole body fat-free mass | Alzheimer's disease (Bellenguez 2022) | rs11245450 | 10:126658075 | A/G | 0.422 | -0.0103 (0.0013) | 7.0E-16 | 0.0223 (0.0083) | 0.007 | 65.2 | -2.175 |
| Whole body fat-free mass | Alzheimer's disease (Bellenguez 2022) | rs6984820 | 8:144993324 | T/C | 0.422 | -0.0101 (0.0013) | 8.5E-16 | 0.0169 (0.0082) | 0.040 | 64.7 | -1.666 |
| Whole body fat-free mass | Alzheimer's disease (Bellenguez 2022) | rs9513510 | 13:99571922 | C/G | 0.699 | -0.0109 (0.0014) | 1.0E-15 | 0.0108 (0.0088) | 0.221 | 64.4 | -0.992 |
| Whole body fat-free mass | Alzheimer's disease (Bellenguez 2022) | rs117543413 | 10:79543740 | T/C | 0.018 | -0.0382 (0.0048) | 1.2E-15 | -0.0092 (0.0325) | 0.776 | 64.0 | 0.241 |
| Whole body fat-free mass | Alzheimer's disease (Bellenguez 2022) | rs10172196 | 2:36780549 | A/G | 0.306 | 0.0108 (0.0013) | 1.3E-15 | -0.0023 (0.0088) | 0.795 | 63.9 | -0.214 |
| Whole body fat-free mass | Alzheimer's disease (Bellenguez 2022) | rs603321 | 6:140256669 | G/A | 0.245 | -0.0115 (0.0014) | 1.6E-15 | 0.0021 (0.0094) | 0.822 | 63.5 | -0.182 |
| Whole body fat-free mass | Alzheimer's disease (Bellenguez 2022) | rs7229520 | 18:46516468 | A/G | 0.662 | -0.0105 (0.0013) | 2.0E-15 | 0.0068 (0.0086) | 0.428 | 63.0 | -0.650 |
| Whole body fat-free mass | Alzheimer's disease (Bellenguez 2022) | rs1780673 | 20:35758359 | A/G | 0.811 | 0.0126 (0.0016) | 2.2E-15 | -0.0143 (0.0105) | 0.174 | 62.9 | -1.134 |
| Whole body fat-free mass | Alzheimer's disease (Bellenguez 2022) | rs10434434 | 4:54271611 | C/A | 0.148 | -0.0139 (0.0018) | 2.4E-15 | -0.0091 (0.0115) | 0.430 | 62.7 | 0.656 |
| Whole body fat-free mass | Alzheimer's disease (Bellenguez 2022) | rs1971955 | 12:122420437 | G/A | 0.854 | -0.0139 (0.0018) | 2.9E-15 | 0.0207 (0.0114) | 0.070 | 62.3 | -1.489 |
| Whole body fat-free mass | Alzheimer's disease (Bellenguez 2022) | rs5752989 | 22:30365780 | A/G | 0.571 | -0.0099 (0.0013) | 2.9E-15 | -0.0108 (0.0082) | 0.187 | 62.3 | 1.086 |
| Whole body fat-free mass | Alzheimer's disease (Bellenguez 2022) | rs34693680 | 3:98665549 | T/C | 0.132 | 0.0144 (0.0018) | 3.3E-15 | 0.0217 (0.0124) | 0.081 | 62.1 | 1.505 |
| Whole body fat-free mass | Alzheimer's disease (Bellenguez 2022) | rs12140153 | 1:62579891 | T/G | 0.094 | -0.0171 (0.0022) | 3.8E-15 | -0.0095 (0.0147) | 0.516 | 61.8 | 0.557 |
| Whole body fat-free mass | Alzheimer's disease (Bellenguez 2022) | rs35251247 | 11:43878459 | A/G | 0.292 | 0.0108 (0.0014) | 3.8E-15 | 0.0081 (0.0090) | 0.366 | 61.8 | 0.749 |
| Whole body fat-free mass | Alzheimer's disease (Bellenguez 2022) | rs6081869 | 20:20060745 | G/T | 0.386 | -0.0102 (0.0013) | 4.4E-15 | 0.0148 (0.0085) | 0.082 | 61.5 | -1.457 |
| Whole body fat-free mass | Alzheimer's disease (Bellenguez 2022) | rs74494415 | 18:74972138 | T/C | 0.040 | -0.0252 (0.0032) | 4.6E-15 | 0.0103 (0.0208) | 0.619 | 61.4 | -0.409 |
| Whole body fat-free mass | Alzheimer's disease (Bellenguez 2022) | rs11049684 | 12:28665359 | T/C | 0.299 | -0.0106 (0.0014) | 4.8E-15 | 0.0138 (0.0090) | 0.124 | 61.4 | -1.301 |
| Whole body fat-free mass | Alzheimer's disease (Bellenguez 2022) | rs10979612 | 9:111687379 | C/T | 0.073 | 0.0187 (0.0024) | 4.9E-15 | 0.0007 (0.0155) | 0.965 | 61.3 | 0.037 |
| Whole body fat-free mass | Alzheimer's disease (Bellenguez 2022) | rs116211567 | 2:68425404 | C/T | 0.081 | -0.0178 (0.0023) | 5.2E-15 | -0.0320 (0.0149) | 0.032 | 61.2 | 1.799 |
| Whole body fat-free mass | Alzheimer's disease (Bellenguez 2022) | rs147110934 | 19:55993436 | T/G | 0.024 | -0.0316 (0.0040) | 5.6E-15 | -0.0363 (0.0290) | 0.211 | 61.0 | 1.150 |
| Whole body fat-free mass | Alzheimer's disease (Bellenguez 2022) | rs10780906 | 9:90841143 | A/G | 0.588 | -0.0099 (0.0013) | 8.4E-15 | 0.0201 (0.0084) | 0.017 | 60.2 | -2.035 |
| Whole body fat-free mass | Alzheimer's disease (Bellenguez 2022) | rs2287163 | 12:107349294 | T/C | 0.374 | -0.0099 (0.0013) | 8.4E-15 | 0.0220 (0.0083) | 0.008 | 60.2 | -2.215 |
| Whole body fat-free mass | Alzheimer's disease (Bellenguez 2022) | rs7301341 | 12:94083105 | C/T | 0.327 | -0.0103 (0.0013) | 9.3E-15 | 0.0079 (0.0089) | 0.374 | 60.0 | -0.770 |
| Whole body fat-free mass | Alzheimer's disease (Bellenguez 2022) | rs2104449 | 1:97082532 | T/G | 0.283 | 0.0107 (0.0014) | 9.4E-15 | -0.0073 (0.0091) | 0.423 | 60.0 | -0.684 |
| Whole body fat-free mass | Alzheimer's disease (Bellenguez 2022) | rs4974223 | 3:56602088 | T/C | 0.904 | 0.0163 (0.0021) | 1.0E-14 | -0.0206 (0.0133) | 0.120 | 59.9 | -1.267 |
| Whole body fat-free mass | Alzheimer's disease (Bellenguez 2022) | rs6142059 | 20:32544327 | C/T | 0.493 | 0.0096 (0.0012) | 1.1E-14 | -0.0065 (0.0082) | 0.424 | 59.8 | -0.674 |
| Whole body fat-free mass | Alzheimer's disease (Bellenguez 2022) | rs2615075 | 1:225934295 | G/A | 0.376 | 0.0098 (0.0013) | 2.0E-14 | -0.0028 (0.0085) | 0.742 | 58.5 | -0.286 |
| Whole body fat-free mass | Alzheimer's disease (Bellenguez 2022) | rs9379084 | 6:7231843 | A/G | 0.116 | -0.0152 (0.0020) | 2.7E-14 | 0.0049 (0.0126) | 0.696 | 57.9 | -0.323 |
| Whole body fat-free mass | Alzheimer's disease (Bellenguez 2022) | rs11785562 | 8:23391493 | A/G | 0.201 | 0.0119 (0.0016) | 2.7E-14 | 0.0133 (0.0102) | 0.191 | 58.0 | 1.115 |
| Whole body fat-free mass | Alzheimer's disease (Bellenguez 2022) | rs5742915 | 15:74336633 | C/T | 0.460 | 0.0095 (0.0012) | 3.2E-14 | 0.0180 (0.0081) | 0.027 | 57.6 | 1.899 |
| Whole body fat-free mass | Alzheimer's disease (Bellenguez 2022) | rs1374370 | 2:85818273 | A/G | 0.305 | 0.0102 (0.0013) | 3.5E-14 | 0.0164 (0.0089) | 0.065 | 57.5 | 1.610 |
| Whole body fat-free mass | Alzheimer's disease (Bellenguez 2022) | rs11611651 | 12:133380790 | A/G | 0.088 | 0.0166 (0.0022) | 4.3E-14 | -0.0032 (0.0146) | 0.824 | 57.0 | -0.193 |
| Whole body fat-free mass | Alzheimer's disease (Bellenguez 2022) | rs981002 | 4:12881731 | A/T | 0.267 | -0.0106 (0.0014) | 5.3E-14 | 0.0058 (0.0093) | 0.535 | 56.6 | -0.550 |
| Whole body fat-free mass | Alzheimer's disease (Bellenguez 2022) | rs2568958 | 1:72765116 | A/G | 0.604 | 0.0095 (0.0013) | 5.5E-14 | -0.0063 (0.0083) | 0.452 | 56.5 | -0.664 |
| Whole body fat-free mass | Alzheimer's disease (Bellenguez 2022) | rs12375196 | 7:103416541 | A/C | 0.424 | 0.0095 (0.0013) | 6.5E-14 | 0.0076 (0.0083) | 0.362 | 56.2 | 0.803 |
| Whole body fat-free mass | Alzheimer's disease (Bellenguez 2022) | rs6026578 | 20:57463472 | G/C | 0.625 | -0.0097 (0.0013) | 6.8E-14 | -0.0128 (0.0086) | 0.136 | 56.1 | 1.323 |
| Whole body fat-free mass | Alzheimer's disease (Bellenguez 2022) | rs58857770 | 19:7208110 | G/C | 0.408 | -0.0095 (0.0013) | 7.6E-14 | -0.0217 (0.0089) | 0.014 | 55.9 | 2.278 |
| Whole body fat-free mass | Alzheimer's disease (Bellenguez 2022) | rs9960619 | 18:33040095 | T/C | 0.344 | 0.0098 (0.0013) | 8.7E-14 | 0.0065 (0.0085) | 0.441 | 55.6 | 0.665 |
| Whole body fat-free mass | Alzheimer's disease (Bellenguez 2022) | rs13272451 | 8:8886183 | T/G | 0.439 | 0.0093 (0.0013) | 8.8E-14 | -0.0097 (0.0082) | 0.238 | 55.6 | -1.038 |
| Whole body fat-free mass | Alzheimer's disease (Bellenguez 2022) | rs11217863 | 11:120293138 | A/G | 0.116 | -0.0145 (0.0019) | 9.0E-14 | 0.0031 (0.0130) | 0.812 | 55.6 | -0.214 |
| Whole body fat-free mass | Alzheimer's disease (Bellenguez 2022) | rs62621400 | 15:101718239 | G/C | 0.058 | -0.0199 (0.0027) | 9.8E-14 | -0.0171 (0.0173) | 0.324 | 55.4 | 0.860 |
| Whole body fat-free mass | Alzheimer's disease (Bellenguez 2022) | rs140493137 | 4:87666916 | A/G | 0.060 | 0.0195 (0.0026) | 1.1E-13 | 0.0027 (0.0176) | 0.879 | 55.2 | 0.139 |
| Whole body fat-free mass | Alzheimer's disease (Bellenguez 2022) | rs3925 | 8:38281658 | A/G | 0.245 | -0.0107 (0.0014) | 1.2E-13 | -0.0175 (0.0096) | 0.069 | 55.0 | 1.631 |
| Whole body fat-free mass | Alzheimer's disease (Bellenguez 2022) | rs12070699 | 1:190230408 | T/C | 0.563 | -0.0092 (0.0012) | 1.5E-13 | 0.0016 (0.0082) | 0.847 | 54.5 | -0.174 |
| Whole body fat-free mass | Alzheimer's disease (Bellenguez 2022) | rs35756741 | 12:12868701 | T/C | 0.092 | -0.0159 (0.0022) | 1.6E-13 | -0.0122 (0.0142) | 0.390 | 54.4 | 0.768 |
| Whole body fat-free mass | Alzheimer's disease (Bellenguez 2022) | rs35492502 | 1:217806224 | A/G | 0.296 | 0.0100 (0.0014) | 2.1E-13 | -0.0168 (0.0091) | 0.066 | 53.9 | -1.687 |
| Whole body fat-free mass | Alzheimer's disease (Bellenguez 2022) | rs284662 | 19:41932275 | C/T | 0.620 | -0.0094 (0.0013) | 2.2E-13 | 0.0102 (0.0089) | 0.251 | 53.8 | -1.085 |
| Whole body fat-free mass | Alzheimer's disease (Bellenguez 2022) | rs36695 | 5:127549187 | A/G | 0.504 | -0.0091 (0.0012) | 2.3E-13 | 0.0061 (0.0081) | 0.451 | 53.7 | -0.671 |
| Whole body fat-free mass | Alzheimer's disease (Bellenguez 2022) | rs4926542 | 1:50263773 | T/C | 0.682 | -0.0098 (0.0013) | 2.4E-13 | 0.0016 (0.0088) | 0.855 | 53.7 | -0.163 |
| Whole body fat-free mass | Alzheimer's disease (Bellenguez 2022) | rs1805165 | 2:88874891 | A/C | 0.721 | -0.0101 (0.0014) | 2.5E-13 | 0.0084 (0.0090) | 0.349 | 53.6 | -0.834 |
| Whole body fat-free mass | Alzheimer's disease (Bellenguez 2022) | rs12209223 | 6:76164589 | A/C | 0.101 | 0.0151 (0.0021) | 2.6E-13 | -0.0414 (0.0136) | 0.002 | 53.5 | -2.746 |
| Whole body fat-free mass | Alzheimer's disease (Bellenguez 2022) | rs9925273 | 16:375900 | G/A | 0.181 | -0.0118 (0.0016) | 2.7E-13 | 0.0138 (0.0106) | 0.194 | 53.4 | -1.168 |
| Whole body fat-free mass | Alzheimer's disease (Bellenguez 2022) | rs55758152 | 5:171317318 | A/G | 0.326 | 0.0098 (0.0013) | 2.8E-13 | -0.0178 (0.0091) | 0.050 | 53.3 | -1.822 |
| Whole body fat-free mass | Alzheimer's disease (Bellenguez 2022) | rs2578557 | 5:5473063 | T/C | 0.642 | -0.0095 (0.0013) | 2.9E-13 | -0.0131 (0.0085) | 0.125 | 53.3 | 1.386 |
| Whole body fat-free mass | Alzheimer's disease (Bellenguez 2022) | rs2970592 | 1:212220847 | A/G | 0.588 | 0.0092 (0.0013) | 3.0E-13 | 0.0037 (0.0082) | 0.656 | 53.2 | 0.404 |
| Whole body fat-free mass | Alzheimer's disease (Bellenguez 2022) | rs74048171 | 11:2093603 | A/C | 0.258 | -0.0104 (0.0014) | 3.2E-13 | -0.0106 (0.0094) | 0.260 | 53.1 | 1.024 |
| Whole body fat-free mass | Alzheimer's disease (Bellenguez 2022) | rs57126421 | 18:2656989 | G/A | 0.238 | -0.0107 (0.0015) | 3.8E-13 | 0.0011 (0.0098) | 0.911 | 52.8 | -0.103 |
| Whole body fat-free mass | Alzheimer's disease (Bellenguez 2022) | rs1521624 | 15:79412399 | A/C | 0.471 | -0.0091 (0.0013) | 4.0E-13 | -0.0202 (0.0083) | 0.015 | 52.7 | 2.222 |
| Whole body fat-free mass | Alzheimer's disease (Bellenguez 2022) | rs11065015 | 12:120520863 | T/C | 0.027 | -0.0282 (0.0039) | 4.1E-13 | -0.0020 (0.0272) | 0.942 | 52.6 | 0.071 |
| Whole body fat-free mass | Alzheimer's disease (Bellenguez 2022) | rs11545482 | 2:70315987 | T/C | 0.020 | -0.0316 (0.0044) | 4.4E-13 | 0.0273 (0.0310) | 0.379 | 52.5 | -0.863 |
| Whole body fat-free mass | Alzheimer's disease (Bellenguez 2022) | rs73601548 | 10:18549889 | T/C | 0.115 | 0.0142 (0.0020) | 4.8E-13 | 0.0261 (0.0128) | 0.041 | 52.3 | 1.843 |
| Whole body fat-free mass | Alzheimer's disease (Bellenguez 2022) | rs55800172 | 1:32414767 | A/G | 0.064 | 0.0182 (0.0025) | 5.7E-13 | -0.0051 (0.0164) | 0.756 | 52.0 | -0.280 |
| Whole body fat-free mass | Alzheimer's disease (Bellenguez 2022) | rs11071182 | 15:55644676 | G/A | 0.870 | 0.0134 (0.0019) | 6.3E-13 | 0.0012 (0.0124) | 0.925 | 51.7 | 0.090 |
| Whole body fat-free mass | Alzheimer's disease (Bellenguez 2022) | rs11160601 | 14:101186641 | T/C | 0.092 | 0.0156 (0.0022) | 6.5E-13 | 0.0146 (0.0142) | 0.304 | 51.7 | 0.938 |
| Whole body fat-free mass | Alzheimer's disease (Bellenguez 2022) | rs3783256 | 13:30107388 | C/T | 0.648 | -0.0094 (0.0013) | 6.7E-13 | 0.0033 (0.0086) | 0.701 | 51.6 | -0.351 |
| Whole body fat-free mass | Alzheimer's disease (Bellenguez 2022) | rs17828687 | 8:73454767 | A/C | 0.573 | -0.0091 (0.0013) | 6.8E-13 | 0.0078 (0.0082) | 0.344 | 51.6 | -0.862 |
| Whole body fat-free mass | Alzheimer's disease (Bellenguez 2022) | rs71393968 | 16:67847341 | A/G | 0.046 | 0.0214 (0.0030) | 6.9E-13 | -0.0181 (0.0202) | 0.370 | 51.6 | -0.847 |
| Whole body fat-free mass | Alzheimer's disease (Bellenguez 2022) | rs11709402 | 3:131551027 | G/A | 0.279 | 0.0100 (0.0014) | 7.1E-13 | -0.0119 (0.0092) | 0.196 | 51.5 | -1.195 |
| Whole body fat-free mass | Alzheimer's disease (Bellenguez 2022) | rs4795318 | 17:36949053 | T/C | 0.520 | 0.0089 (0.0012) | 7.1E-13 | -0.0004 (0.0082) | 0.964 | 51.5 | -0.045 |
| Whole body fat-free mass | Alzheimer's disease (Bellenguez 2022) | rs73013411 | 6:164126233 | A/C | 0.131 | 0.0131 (0.0018) | 8.9E-13 | -0.0088 (0.0121) | 0.469 | 51.1 | -0.670 |
| Whole body fat-free mass | Alzheimer's disease (Bellenguez 2022) | rs16916881 | 8:95566270 | A/C | 0.235 | -0.0104 (0.0015) | 9.2E-13 | -0.0035 (0.0093) | 0.708 | 51.0 | 0.335 |
| Whole body fat-free mass | Alzheimer's disease (Bellenguez 2022) | rs10870597 | 13:114999636 | G/A | 0.235 | -0.0105 (0.0015) | 1.0E-12 | 0.0062 (0.0095) | 0.514 | 50.8 | -0.593 |
| Whole body fat-free mass | Alzheimer's disease (Bellenguez 2022) | rs6719296 | 2:103101108 | A/G | 0.495 | 0.0089 (0.0012) | 1.1E-12 | 0.0022 (0.0082) | 0.786 | 50.6 | 0.249 |
| Whole body fat-free mass | Alzheimer's disease (Bellenguez 2022) | rs10945541 | 6:158740218 | G/A | 0.665 | -0.0093 (0.0013) | 1.1E-12 | -0.0003 (0.0086) | 0.968 | 50.7 | 0.032 |
| Whole body fat-free mass | Alzheimer's disease (Bellenguez 2022) | rs60804050 | 1:118870373 | A/G | 0.256 | -0.0100 (0.0014) | 1.5E-12 | 0.0152 (0.0094) | 0.107 | 50.1 | -1.517 |
| Whole body fat-free mass | Alzheimer's disease (Bellenguez 2022) | rs9540493 | 13:66205704 | G/A | 0.545 | -0.0089 (0.0013) | 1.5E-12 | 0.0014 (0.0082) | 0.864 | 50.1 | -0.157 |
| Whole body fat-free mass | Alzheimer's disease (Bellenguez 2022) | rs7245985 | 19:30710410 | G/T | 0.208 | -0.0109 (0.0015) | 1.5E-12 | 0.0039 (0.0106) | 0.716 | 50.1 | -0.359 |
| Whole body fat-free mass | Alzheimer's disease (Bellenguez 2022) | rs73619441 | 20:61564901 | G/T | 0.144 | -0.0126 (0.0018) | 1.5E-12 | 0.0229 (0.0113) | 0.043 | 50.1 | -1.824 |
| Whole body fat-free mass | Alzheimer's disease (Bellenguez 2022) | rs2789366 | 1:235508270 | A/G | 0.346 | -0.0092 (0.0013) | 1.6E-12 | -0.0122 (0.0087) | 0.159 | 50.0 | 1.328 |
| Whole body fat-free mass | Alzheimer's disease (Bellenguez 2022) | rs16844418 | 4:3471754 | C/T | 0.143 | -0.0125 (0.0018) | 1.9E-12 | 0.0165 (0.0114) | 0.146 | 49.6 | -1.322 |
| Whole body fat-free mass | Alzheimer's disease (Bellenguez 2022) | rs10222924 | 4:49016761 | G/A | 0.699 | -0.0095 (0.0014) | 2.0E-12 | 0.0226 (0.0091) | 0.013 | 49.5 | -2.367 |
| Whole body fat-free mass | Alzheimer's disease (Bellenguez 2022) | rs7192870 | 16:14397436 | C/T | 0.469 | -0.0089 (0.0013) | 2.0E-12 | 0.0087 (0.0083) | 0.294 | 49.4 | -0.983 |
| Whole body fat-free mass | Alzheimer's disease (Bellenguez 2022) | rs11052457 | 12:33268956 | T/A | 0.038 | 0.0228 (0.0032) | 2.1E-12 | -0.0276 (0.0206) | 0.180 | 49.4 | -1.211 |
| Whole body fat-free mass | Alzheimer's disease (Bellenguez 2022) | rs892511 | 1:110078255 | G/C | 0.028 | 0.0263 (0.0038) | 3.7E-12 | 0.0008 (0.0249) | 0.975 | 48.3 | 0.030 |
| Whole body fat-free mass | Alzheimer's disease (Bellenguez 2022) | rs17400325 | 2:178565913 | C/T | 0.042 | 0.0215 (0.0031) | 3.8E-12 | 0.0195 (0.0196) | 0.318 | 48.2 | 0.906 |
| Whole body fat-free mass | Alzheimer's disease (Bellenguez 2022) | rs9394166 | 6:33751122 | C/G | 0.380 | 0.0089 (0.0013) | 3.8E-12 | 0.0101 (0.0084) | 0.227 | 48.2 | 1.139 |
| Whole body fat-free mass | Alzheimer's disease (Bellenguez 2022) | rs338361 | 15:68171116 | C/T | 0.706 | 0.0095 (0.0014) | 3.9E-12 | -0.0024 (0.0089) | 0.789 | 48.1 | -0.253 |
| Whole body fat-free mass | Alzheimer's disease (Bellenguez 2022) | rs115461836 | 6:19078931 | C/T | 0.046 | 0.0208 (0.0030) | 4.2E-12 | -0.0702 (0.0214) | 0.001 | 48.0 | -3.368 |
| Whole body fat-free mass | Alzheimer's disease (Bellenguez 2022) | rs1296328 | 4:137083193 | C/A | 0.559 | -0.0087 (0.0013) | 4.5E-12 | 0.0023 (0.0083) | 0.782 | 47.9 | -0.265 |
| Whole body fat-free mass | Alzheimer's disease (Bellenguez 2022) | rs4132132 | 4:31001301 | C/T | 0.444 | 0.0087 (0.0013) | 4.7E-12 | -0.0107 (0.0082) | 0.193 | 47.8 | -1.228 |
| Whole body fat-free mass | Alzheimer's disease (Bellenguez 2022) | rs9596810 | 13:54056553 | T/C | 0.435 | -0.0089 (0.0013) | 4.7E-12 | 0.0145 (0.0084) | 0.086 | 47.8 | -1.621 |
| Whole body fat-free mass | Alzheimer's disease (Bellenguez 2022) | rs1927635 | 9:16461905 | C/T | 0.354 | 0.0091 (0.0013) | 5.0E-12 | -0.0028 (0.0086) | 0.745 | 47.7 | -0.309 |
| Whole body fat-free mass | Alzheimer's disease (Bellenguez 2022) | rs1218826 | 13:27995760 | G/A | 0.666 | 0.0091 (0.0013) | 5.0E-12 | -0.0128 (0.0087) | 0.138 | 47.7 | -1.400 |
| Whole body fat-free mass | Alzheimer's disease (Bellenguez 2022) | rs17363646 | 1:86823503 | G/A | 0.136 | 0.0124 (0.0018) | 5.5E-12 | 0.0060 (0.0115) | 0.604 | 47.5 | 0.483 |
| Whole body fat-free mass | Alzheimer's disease (Bellenguez 2022) | rs17443541 | 2:200402624 | C/T | 0.189 | -0.0110 (0.0016) | 5.8E-12 | 0.0087 (0.0106) | 0.415 | 47.4 | -0.792 |
| Whole body fat-free mass | Alzheimer's disease (Bellenguez 2022) | rs12889702 | 14:42894143 | C/A | 0.313 | 0.0092 (0.0013) | 6.4E-12 | 0.0008 (0.0087) | 0.928 | 47.2 | 0.087 |
| Whole body fat-free mass | Alzheimer's disease (Bellenguez 2022) | rs254963 | 5:171224403 | G/A | 0.447 | -0.0086 (0.0013) | 6.6E-12 | 0.0050 (0.0083) | 0.550 | 47.1 | -0.580 |
| Whole body fat-free mass | Alzheimer's disease (Bellenguez 2022) | rs1813212 | 11:89301382 | G/A | 0.446 | -0.0086 (0.0013) | 6.7E-12 | -0.0038 (0.0083) | 0.646 | 47.1 | 0.442 |
| Whole body fat-free mass | Alzheimer's disease (Bellenguez 2022) | rs774214 | 12:56918566 | C/T | 0.663 | -0.0090 (0.0013) | 7.2E-12 | 0.0133 (0.0087) | 0.124 | 47.0 | -1.479 |
| Whole body fat-free mass | Alzheimer's disease (Bellenguez 2022) | rs6972291 | 7:55802063 | C/T | 0.176 | -0.0112 (0.0016) | 7.7E-12 | 0.0036 (0.0105) | 0.730 | 46.8 | -0.322 |
| Whole body fat-free mass | Alzheimer's disease (Bellenguez 2022) | rs7731023 | 5:36181627 | G/A | 0.574 | 0.0086 (0.0013) | 7.8E-12 | 0.0006 (0.0083) | 0.943 | 46.8 | 0.070 |
| Whole body fat-free mass | Alzheimer's disease (Bellenguez 2022) | rs12981554 | 19:3416668 | G/A | 0.542 | -0.0086 (0.0013) | 8.9E-12 | 0.0030 (0.0090) | 0.738 | 46.6 | -0.349 |
| Whole body fat-free mass | Alzheimer's disease (Bellenguez 2022) | rs35874463 | 15:67457698 | G/A | 0.058 | 0.0182 (0.0027) | 9.7E-12 | 0.0133 (0.0188) | 0.478 | 46.4 | 0.732 |
| Whole body fat-free mass | Alzheimer's disease (Bellenguez 2022) | rs10995366 | 10:52772113 | A/G | 0.251 | -0.0098 (0.0014) | 1.1E-11 | 0.0046 (0.0095) | 0.625 | 46.1 | -0.471 |
| Whole body fat-free mass | Alzheimer's disease (Bellenguez 2022) | rs77344209 | 3:55515277 | T/C | 0.252 | 0.0098 (0.0014) | 1.2E-11 | 0.0081 (0.0097) | 0.401 | 46.0 | 0.826 |
| Whole body fat-free mass | Alzheimer's disease (Bellenguez 2022) | rs11593630 | 10:120491506 | T/C | 0.353 | -0.0088 (0.0013) | 1.2E-11 | 0.0083 (0.0085) | 0.332 | 46.0 | -0.943 |
| Whole body fat-free mass | Alzheimer's disease (Bellenguez 2022) | rs1418433 | 6:44752568 | A/G | 0.588 | -0.0085 (0.0013) | 1.3E-11 | 0.0006 (0.0083) | 0.938 | 45.8 | -0.070 |
| Whole body fat-free mass | Alzheimer's disease (Bellenguez 2022) | rs7912286 | 10:114693230 | G/A | 0.602 | -0.0087 (0.0013) | 1.3E-11 | -0.0038 (0.0085) | 0.654 | 45.8 | 0.438 |
| Whole body fat-free mass | Alzheimer's disease (Bellenguez 2022) | rs7321045 | 13:97034410 | A/G | 0.449 | 0.0085 (0.0013) | 1.3E-11 | 0.0036 (0.0082) | 0.660 | 45.8 | 0.422 |
| Whole body fat-free mass | Alzheimer's disease (Bellenguez 2022) | rs73004967 | 19:19717056 | G/A | 0.069 | -0.0167 (0.0025) | 1.3E-11 | 0.0336 (0.0176) | 0.056 | 45.8 | -2.007 |
| Whole body fat-free mass | Alzheimer's disease (Bellenguez 2022) | rs71423263 | 2:144024781 | G/T | 0.141 | 0.0120 (0.0018) | 1.5E-11 | -0.0024 (0.0116) | 0.836 | 45.5 | -0.200 |
| Whole body fat-free mass | Alzheimer's disease (Bellenguez 2022) | rs73213484 | 4:28489339 | T/A | 0.141 | -0.0120 (0.0018) | 1.5E-11 | 0.0056 (0.0113) | 0.620 | 45.5 | -0.466 |
| Whole body fat-free mass | Alzheimer's disease (Bellenguez 2022) | rs6085658 | 20:6685377 | T/C | 0.403 | -0.0086 (0.0013) | 1.6E-11 | 0.0089 (0.0083) | 0.284 | 45.4 | -1.035 |
| Whole body fat-free mass | Alzheimer's disease (Bellenguez 2022) | rs836511 | 7:6458319 | G/A | 0.199 | 0.0104 (0.0016) | 2.0E-11 | -0.0185 (0.0101) | 0.066 | 45.0 | -1.776 |
| Whole body fat-free mass | Alzheimer's disease (Bellenguez 2022) | rs2122823 | 7:25939161 | T/C | 0.211 | 0.0103 (0.0015) | 2.0E-11 | 0.0056 (0.0101) | 0.578 | 45.0 | 0.543 |
| Whole body fat-free mass | Alzheimer's disease (Bellenguez 2022) | rs12427047 | 12:90213070 | T/C | 0.243 | -0.0097 (0.0014) | 2.1E-11 | 0.0076 (0.0095) | 0.424 | 44.9 | -0.784 |
| Whole body fat-free mass | Alzheimer's disease (Bellenguez 2022) | rs112957890 | 14:36220876 | G/A | 0.265 | 0.0095 (0.0014) | 2.1E-11 | -0.0188 (0.0095) | 0.048 | 44.8 | -1.972 |
| Whole body fat-free mass | Alzheimer's disease (Bellenguez 2022) | rs12729817 | 1:218971896 | G/A | 0.474 | -0.0083 (0.0012) | 2.2E-11 | -0.0084 (0.0081) | 0.300 | 44.8 | 1.015 |
| Whole body fat-free mass | Alzheimer's disease (Bellenguez 2022) | rs4268495 | 11:28645341 | C/G | 0.374 | -0.0086 (0.0013) | 2.2E-11 | 0.0081 (0.0084) | 0.333 | 44.8 | -0.944 |
| Whole body fat-free mass | Alzheimer's disease (Bellenguez 2022) | rs114949263 | 7:150498245 | C/T | 0.112 | -0.0132 (0.0020) | 2.5E-11 | 0.0079 (0.0133) | 0.551 | 44.5 | -0.599 |
| Whole body fat-free mass | Alzheimer's disease (Bellenguez 2022) | rs505575 | 5:111256812 | C/T | 0.673 | -0.0088 (0.0013) | 2.6E-11 | -0.0002 (0.0087) | 0.981 | 44.4 | 0.023 |
| Whole body fat-free mass | Alzheimer's disease (Bellenguez 2022) | rs61878760 | 11:12807189 | A/G | 0.083 | 0.0151 (0.0023) | 2.8E-11 | 0.0058 (0.0148) | 0.694 | 44.3 | 0.385 |
| Whole body fat-free mass | Alzheimer's disease (Bellenguez 2022) | rs3845344 | 1:75001480 | T/C | 0.391 | 0.0084 (0.0013) | 3.1E-11 | -0.0023 (0.0083) | 0.781 | 44.1 | -0.273 |
| Whole body fat-free mass | Alzheimer's disease (Bellenguez 2022) | rs9327336 | 5:123990270 | C/T | 0.343 | 0.0087 (0.0013) | 3.2E-11 | -0.0138 (0.0086) | 0.107 | 44.1 | -1.593 |
| Whole body fat-free mass | Alzheimer's disease (Bellenguez 2022) | rs2663125 | 15:99563891 | C/T | 0.691 | -0.0089 (0.0013) | 3.2E-11 | -0.0051 (0.0089) | 0.567 | 44.0 | 0.571 |
| Whole body fat-free mass | Alzheimer's disease (Bellenguez 2022) | rs67560975 | 17:47142930 | C/T | 0.116 | 0.0129 (0.0019) | 3.3E-11 | -0.0152 (0.0129) | 0.238 | 44.0 | -1.178 |
| Whole body fat-free mass | Alzheimer's disease (Bellenguez 2022) | rs1056747 | 9:35690102 | G/A | 0.412 | -0.0084 (0.0013) | 3.4E-11 | 0.0027 (0.0082) | 0.742 | 43.9 | -0.323 |
| Whole body fat-free mass | Alzheimer's disease (Bellenguez 2022) | rs17197114 | 14:21894526 | C/T | 0.177 | 0.0109 (0.0016) | 3.4E-11 | -0.0121 (0.0109) | 0.264 | 43.9 | -1.113 |
| Whole body fat-free mass | Alzheimer's disease (Bellenguez 2022) | rs4912905 | 5:142730376 | C/G | 0.249 | -0.0095 (0.0014) | 3.8E-11 | -0.0094 (0.0096) | 0.326 | 43.7 | 0.987 |
| Whole body fat-free mass | Alzheimer's disease (Bellenguez 2022) | rs77848106 | 1:107971673 | A/C | 0.296 | -0.0090 (0.0014) | 3.9E-11 | -0.0010 (0.0090) | 0.907 | 43.7 | 0.111 |
| Whole body fat-free mass | Alzheimer's disease (Bellenguez 2022) | rs4504126 | 3:33600582 | C/A | 0.027 | 0.0250 (0.0038) | 4.3E-11 | -0.0013 (0.0259) | 0.960 | 43.5 | -0.052 |
| Whole body fat-free mass | Alzheimer's disease (Bellenguez 2022) | rs4439140 | 8:23113908 | A/G | 0.418 | -0.0085 (0.0013) | 4.7E-11 | -0.0082 (0.0085) | 0.336 | 43.3 | 0.965 |
| Whole body fat-free mass | Alzheimer's disease (Bellenguez 2022) | rs10775406 | 17:46197755 | G/A | 0.760 | -0.0096 (0.0015) | 4.7E-11 | -0.0056 (0.0093) | 0.545 | 43.3 | 0.585 |
| Whole body fat-free mass | Alzheimer's disease (Bellenguez 2022) | rs6665399 | 1:21553933 | C/T | 0.445 | 0.0082 (0.0012) | 4.9E-11 | -0.0086 (0.0082) | 0.294 | 43.2 | -1.054 |
| Whole body fat-free mass | Alzheimer's disease (Bellenguez 2022) | rs3110496 | 17:27917771 | G/A | 0.686 | 0.0088 (0.0013) | 5.0E-11 | -0.0043 (0.0086) | 0.615 | 43.2 | -0.489 |
| Whole body fat-free mass | Alzheimer's disease (Bellenguez 2022) | rs10950207 | 7:70105168 | T/C | 0.384 | 0.0084 (0.0013) | 5.8E-11 | -0.0115 (0.0085) | 0.174 | 42.9 | -1.374 |
| Whole body fat-free mass | Alzheimer's disease (Bellenguez 2022) | rs9788443 | 14:23878279 | C/T | 0.048 | 0.0191 (0.0029) | 5.8E-11 | 0.0046 (0.0190) | 0.811 | 42.9 | 0.241 |
| Whole body fat-free mass | Alzheimer's disease (Bellenguez 2022) | rs4525525 | 17:1866892 | T/G | 0.264 | -0.0094 (0.0014) | 5.9E-11 | -0.0114 (0.0095) | 0.231 | 42.9 | 1.218 |
| Whole body fat-free mass | Alzheimer's disease (Bellenguez 2022) | rs1179905 | 6:90322922 | G/A | 0.195 | 0.0103 (0.0016) | 6.3E-11 | -0.0059 (0.0101) | 0.559 | 42.7 | -0.575 |
| Whole body fat-free mass | Alzheimer's disease (Bellenguez 2022) | rs6416914 | 17:39262850 | G/A | 0.695 | -0.0088 (0.0014) | 6.5E-11 | 0.0114 (0.0088) | 0.197 | 42.7 | -1.291 |
| Whole body fat-free mass | Alzheimer's disease (Bellenguez 2022) | rs8074074 | 17:17407816 | T/C | 0.755 | 0.0095 (0.0014) | 6.8E-11 | 0.0051 (0.0092) | 0.577 | 42.6 | 0.539 |
| Whole body fat-free mass | Alzheimer's disease (Bellenguez 2022) | rs34017457 | 16:67166731 | A/G | 0.007 | 0.0488 (0.0075) | 6.9E-11 | -0.1414 (0.0454) | 0.002 | 42.5 | -2.899 |
| Whole body fat-free mass | Alzheimer's disease (Bellenguez 2022) | rs10843397 | 12:29529523 | T/C | 0.242 | 0.0094 (0.0014) | 7.6E-11 | 0.0041 (0.0093) | 0.663 | 42.4 | 0.435 |
| Whole body fat-free mass | Alzheimer's disease (Bellenguez 2022) | rs10511111 | 3:80599757 | C/T | 0.309 | 0.0087 (0.0013) | 7.9E-11 | -0.0073 (0.0086) | 0.395 | 42.3 | -0.837 |
| Whole body fat-free mass | Alzheimer's disease (Bellenguez 2022) | rs113619763 | 22:46399175 | T/A | 0.062 | 0.0168 (0.0026) | 8.0E-11 | -0.0011 (0.0170) | 0.950 | 42.3 | -0.066 |
| Whole body fat-free mass | Alzheimer's disease (Bellenguez 2022) | rs1268947 | 10:121132631 | C/G | 0.126 | 0.0122 (0.0019) | 8.9E-11 | -0.0186 (0.0126) | 0.139 | 42.1 | -1.528 |
| Whole body fat-free mass | Alzheimer's disease (Bellenguez 2022) | rs60385590 | 3:53630199 | A/C | 0.258 | 0.0093 (0.0014) | 1.0E-10 | 0.0054 (0.0094) | 0.561 | 41.8 | 0.583 |
| Whole body fat-free mass | Alzheimer's disease (Bellenguez 2022) | rs7755185 | 6:152339615 | G/A | 0.311 | 0.0087 (0.0013) | 1.0E-10 | -0.0231 (0.0088) | 0.009 | 41.8 | -2.668 |
| Whole body fat-free mass | Alzheimer's disease (Bellenguez 2022) | rs56141370 | 9:127056213 | T/C | 0.115 | 0.0126 (0.0020) | 1.0E-10 | -0.0193 (0.0137) | 0.157 | 41.7 | -1.531 |
| Whole body fat-free mass | Alzheimer's disease (Bellenguez 2022) | rs4635681 | 3:152310614 | G/A | 0.156 | 0.0110 (0.0017) | 1.1E-10 | 0.0037 (0.0111) | 0.736 | 41.6 | 0.336 |
| Whole body fat-free mass | Alzheimer's disease (Bellenguez 2022) | rs1266876 | 6:51781580 | T/C | 0.350 | 0.0084 (0.0013) | 1.1E-10 | -0.0169 (0.0085) | 0.048 | 41.6 | -2.018 |
| Whole body fat-free mass | Alzheimer's disease (Bellenguez 2022) | rs240113 | 6:101058886 | A/G | 0.531 | -0.0080 (0.0012) | 1.1E-10 | 0.0225 (0.0081) | 0.006 | 41.7 | -2.804 |
| Whole body fat-free mass | Alzheimer's disease (Bellenguez 2022) | rs6900690 | 6:126223032 | A/G | 0.562 | 0.0080 (0.0012) | 1.1E-10 | 0.0109 (0.0082) | 0.186 | 41.6 | 1.357 |
| Whole body fat-free mass | Alzheimer's disease (Bellenguez 2022) | rs12156265 | 8:144848756 | A/G | 0.590 | 0.0082 (0.0013) | 1.1E-10 | -0.0004 (0.0083) | 0.960 | 41.6 | -0.049 |
| Whole body fat-free mass | Alzheimer's disease (Bellenguez 2022) | rs1008158 | 9:113828811 | G/A | 0.342 | 0.0085 (0.0013) | 1.1E-10 | -0.0061 (0.0087) | 0.487 | 41.6 | -0.718 |
| Whole body fat-free mass | Alzheimer's disease (Bellenguez 2022) | rs1407031 | 20:6542634 | T/C | 0.397 | -0.0082 (0.0013) | 1.2E-10 | 0.0041 (0.0084) | 0.628 | 41.4 | -0.499 |
| Whole body fat-free mass | Alzheimer's disease (Bellenguez 2022) | rs17115481 | 5:153358226 | A/G | 0.270 | -0.0090 (0.0014) | 1.3E-10 | 0.0082 (0.0090) | 0.363 | 41.4 | -0.911 |
| Whole body fat-free mass | Alzheimer's disease (Bellenguez 2022) | rs62466118 | 7:92716556 | A/G | 0.028 | -0.0255 (0.0040) | 1.4E-10 | 0.0083 (0.0295) | 0.778 | 41.1 | -0.326 |
| Whole body fat-free mass | Alzheimer's disease (Bellenguez 2022) | rs36100359 | 14:21578007 | A/G | 0.139 | -0.0115 (0.0018) | 1.4E-10 | -0.0046 (0.0126) | 0.712 | 41.2 | 0.399 |
| Whole body fat-free mass | Alzheimer's disease (Bellenguez 2022) | rs10498672 | 6:7797840 | G/C | 0.176 | 0.0104 (0.0016) | 1.6E-10 | -0.0032 (0.0108) | 0.770 | 40.9 | -0.308 |
| Whole body fat-free mass | Alzheimer's disease (Bellenguez 2022) | rs1657222 | 10:34837139 | A/G | 0.396 | 0.0081 (0.0013) | 1.7E-10 | -0.0093 (0.0083) | 0.263 | 40.8 | -1.143 |
| Whole body fat-free mass | Alzheimer's disease (Bellenguez 2022) | rs67141907 | 13:42805360 | T/C | 0.149 | 0.0112 (0.0018) | 1.7E-10 | -0.0221 (0.0112) | 0.049 | 40.8 | -1.974 |
| Whole body fat-free mass | Alzheimer's disease (Bellenguez 2022) | rs281385 | 19:49217261 | G/A | 0.879 | -0.0122 (0.0019) | 1.7E-10 | 0.0072 (0.0129) | 0.576 | 40.8 | -0.590 |
| Whole body fat-free mass | Alzheimer's disease (Bellenguez 2022) | rs62439025 | 6:166592164 | C/G | 0.152 | 0.0110 (0.0017) | 1.8E-10 | 0.0091 (0.0117) | 0.435 | 40.6 | 0.825 |
| Whole body fat-free mass | Alzheimer's disease (Bellenguez 2022) | rs6591 | 11:840363 | T/C | 0.544 | 0.0080 (0.0013) | 1.9E-10 | -0.0028 (0.0082) | 0.732 | 40.6 | -0.351 |
| Whole body fat-free mass | Alzheimer's disease (Bellenguez 2022) | rs7843128 | 8:22473465 | C/T | 0.357 | -0.0083 (0.0013) | 2.0E-10 | -0.0007 (0.0085) | 0.938 | 40.5 | 0.085 |
| Whole body fat-free mass | Alzheimer's disease (Bellenguez 2022) | rs112758380 | 11:14296329 | A/G | 0.057 | 0.0171 (0.0027) | 2.1E-10 | -0.0175 (0.0183) | 0.338 | 40.4 | -1.022 |
| Whole body fat-free mass | Alzheimer's disease (Bellenguez 2022) | rs62246311 | 3:9498143 | A/G | 0.102 | 0.0130 (0.0020) | 2.2E-10 | -0.0190 (0.0135) | 0.160 | 40.3 | -1.464 |
| Whole body fat-free mass | Alzheimer's disease (Bellenguez 2022) | rs12146350 | 10:22843111 | G/C | 0.602 | -0.0081 (0.0013) | 2.3E-10 | 0.0087 (0.0084) | 0.297 | 40.2 | -1.079 |
| Whole body fat-free mass | Alzheimer's disease (Bellenguez 2022) | rs4906203 | 14:102928991 | T/C | 0.234 | -0.0093 (0.0015) | 2.6E-10 | 0.0153 (0.0095) | 0.109 | 40.0 | -1.647 |
| Whole body fat-free mass | Alzheimer's disease (Bellenguez 2022) | rs10188231 | 2:142297493 | G/C | 0.186 | -0.0100 (0.0016) | 2.7E-10 | 0.0049 (0.0105) | 0.640 | 39.9 | -0.488 |
| Whole body fat-free mass | Alzheimer's disease (Bellenguez 2022) | rs10770705 | 12:20857467 | C/A | 0.663 | -0.0083 (0.0013) | 2.8E-10 | 0.0248 (0.0087) | 0.005 | 39.8 | -2.998 |
| Whole body fat-free mass | Alzheimer's disease (Bellenguez 2022) | rs6772164 | 3:196078149 | A/C | 0.358 | 0.0081 (0.0013) | 3.1E-10 | -0.0024 (0.0085) | 0.778 | 39.6 | -0.295 |
| Whole body fat-free mass | Alzheimer's disease (Bellenguez 2022) | rs76307059 | 6:169000888 | G/C | 0.046 | -0.0186 (0.0030) | 3.1E-10 | 0.0081 (0.0182) | 0.656 | 39.6 | -0.436 |
| Whole body fat-free mass | Alzheimer's disease (Bellenguez 2022) | rs3730071 | 12:49168798 | A/C | 0.030 | -0.0228 (0.0036) | 3.2E-10 | 0.0356 (0.0247) | 0.150 | 39.6 | -1.562 |
| Whole body fat-free mass | Alzheimer's disease (Bellenguez 2022) | rs11142700 | 9:73757155 | C/T | 0.408 | -0.0080 (0.0013) | 3.3E-10 | 0.0111 (0.0083) | 0.180 | 39.5 | -1.394 |
| Whole body fat-free mass | Alzheimer's disease (Bellenguez 2022) | rs10881583 | 9:137267439 | C/T | 0.241 | -0.0091 (0.0015) | 3.4E-10 | -0.0028 (0.0096) | 0.771 | 39.4 | 0.306 |
| Whole body fat-free mass | Alzheimer's disease (Bellenguez 2022) | rs8007644 | 14:35218831 | A/G | 0.388 | 0.0081 (0.0013) | 3.4E-10 | 0.0022 (0.0084) | 0.797 | 39.4 | 0.272 |
| Whole body fat-free mass | Alzheimer's disease (Bellenguez 2022) | rs10282707 | 7:17911038 | T/C | 0.397 | 0.0079 (0.0013) | 3.6E-10 | -0.0195 (0.0083) | 0.018 | 39.3 | -2.453 |
| Whole body fat-free mass | Alzheimer's disease (Bellenguez 2022) | rs6575340 | 14:94023972 | A/G | 0.636 | 0.0081 (0.0013) | 3.6E-10 | -0.0037 (0.0085) | 0.665 | 39.3 | -0.456 |
| Whole body fat-free mass | Alzheimer's disease (Bellenguez 2022) | rs11205354 | 1:150249101 | A/C | 0.444 | -0.0078 (0.0013) | 3.7E-10 | 0.0195 (0.0083) | 0.018 | 39.3 | -2.486 |
| Whole body fat-free mass | Alzheimer's disease (Bellenguez 2022) | rs13085472 | 3:171129859 | C/T | 0.666 | -0.0083 (0.0013) | 4.1E-10 | -0.0030 (0.0087) | 0.727 | 39.1 | 0.364 |
| Whole body fat-free mass | Alzheimer's disease (Bellenguez 2022) | rs7994783 | 13:41661922 | G/T | 0.302 | -0.0085 (0.0014) | 4.3E-10 | -0.0017 (0.0089) | 0.852 | 39.0 | 0.201 |
| Whole body fat-free mass | Alzheimer's disease (Bellenguez 2022) | rs12533548 | 7:148660579 | G/A | 0.651 | 0.0081 (0.0013) | 4.6E-10 | -0.0053 (0.0086) | 0.538 | 38.8 | -0.651 |
| Whole body fat-free mass | Alzheimer's disease (Bellenguez 2022) | rs112544217 | 2:20222686 | T/C | 0.022 | -0.0268 (0.0043) | 4.8E-10 | 0.0249 (0.0298) | 0.404 | 38.8 | -0.928 |
| Whole body fat-free mass | Alzheimer's disease (Bellenguez 2022) | rs284315 | 1:10734800 | G/A | 0.498 | -0.0077 (0.0012) | 4.9E-10 | 0.0083 (0.0083) | 0.318 | 38.7 | -1.080 |
| Whole body fat-free mass | Alzheimer's disease (Bellenguez 2022) | rs117451679 | 12:17245591 | G/A | 0.106 | 0.0127 (0.0020) | 5.0E-10 | 0.0124 (0.0138) | 0.369 | 38.7 | 0.975 |
| Whole body fat-free mass | Alzheimer's disease (Bellenguez 2022) | rs12047986 | 1:155043812 | G/A | 0.522 | -0.0077 (0.0012) | 5.1E-10 | -0.0028 (0.0082) | 0.732 | 38.6 | 0.364 |
| Whole body fat-free mass | Alzheimer's disease (Bellenguez 2022) | rs11937249 | 4:110755321 | T/G | 0.636 | -0.0080 (0.0013) | 5.1E-10 | -0.0017 (0.0084) | 0.838 | 38.6 | 0.212 |
| Whole body fat-free mass | Alzheimer's disease (Bellenguez 2022) | rs2422054 | 5:161382474 | A/T | 0.631 | 0.0080 (0.0013) | 5.1E-10 | -0.0034 (0.0084) | 0.688 | 38.6 | -0.425 |
| Whole body fat-free mass | Alzheimer's disease (Bellenguez 2022) | rs4819021 | 21:46466927 | C/T | 0.477 | -0.0078 (0.0013) | 6.4E-10 | -0.0027 (0.0083) | 0.743 | 38.2 | 0.347 |
| Whole body fat-free mass | Alzheimer's disease (Bellenguez 2022) | rs11684531 | 2:219835489 | G/A | 0.133 | -0.0113 (0.0018) | 6.6E-10 | 0.0093 (0.0134) | 0.487 | 38.1 | -0.826 |
| Whole body fat-free mass | Alzheimer's disease (Bellenguez 2022) | rs11833839 | 12:132560957 | T/C | 0.057 | 0.0166 (0.0027) | 6.8E-10 | 0.0136 (0.0166) | 0.413 | 38.1 | 0.821 |
| Whole body fat-free mass | Alzheimer's disease (Bellenguez 2022) | rs77093479 | 17:79429575 | G/C | 0.165 | -0.0104 (0.0017) | 6.9E-10 | 0.0017 (0.0112) | 0.882 | 38.0 | -0.163 |
| Whole body fat-free mass | Alzheimer's disease (Bellenguez 2022) | rs34825238 | 12:103328352 | T/G | 0.255 | 0.0089 (0.0014) | 7.1E-10 | -0.0101 (0.0095) | 0.284 | 38.0 | -1.136 |
| Whole body fat-free mass | Alzheimer's disease (Bellenguez 2022) | rs6946419 | 7:28768853 | C/T | 0.377 | -0.0079 (0.0013) | 7.2E-10 | 0.0080 (0.0084) | 0.341 | 38.0 | -1.011 |
| Whole body fat-free mass | Alzheimer's disease (Bellenguez 2022) | rs752070 | 2:74824970 | G/A | 0.126 | 0.0114 (0.0019) | 8.2E-10 | -0.0245 (0.0121) | 0.044 | 37.7 | -2.140 |
| Whole body fat-free mass | Alzheimer's disease (Bellenguez 2022) | rs2274116 | 9:139094805 | T/C | 0.341 | -0.0081 (0.0013) | 8.3E-10 | 0.0119 (0.0089) | 0.182 | 37.7 | -1.471 |
| Whole body fat-free mass | Alzheimer's disease (Bellenguez 2022) | rs6693481 | 1:203766395 | C/T | 0.695 | -0.0083 (0.0013) | 8.6E-10 | 0.0032 (0.0090) | 0.723 | 37.6 | -0.387 |
| Whole body fat-free mass | Alzheimer's disease (Bellenguez 2022) | rs2807742 | 10:28781367 | A/G | 0.771 | -0.0091 (0.0015) | 8.6E-10 | 0.0287 (0.0098) | 0.003 | 37.6 | -3.153 |
| Whole body fat-free mass | Alzheimer's disease (Bellenguez 2022) | rs161799 | 1:8015560 | G/A | 0.344 | -0.0080 (0.0013) | 9.0E-10 | 0.0070 (0.0086) | 0.412 | 37.5 | -0.872 |
| Whole body fat-free mass | Alzheimer's disease (Bellenguez 2022) | rs72754922 | 5:42547714 | A/G | 0.061 | -0.0160 (0.0026) | 9.1E-10 | -0.0022 (0.0175) | 0.898 | 37.5 | 0.138 |
| Whole body fat-free mass | Alzheimer's disease (Bellenguez 2022) | rs35665085 | 22:17625915 | A/G | 0.056 | -0.0166 (0.0027) | 9.2E-10 | 0.0204 (0.0179) | 0.256 | 37.5 | -1.229 |
| Whole body fat-free mass | Alzheimer's disease (Bellenguez 2022) | rs34227797 | 4:159982551 | C/G | 0.310 | 0.0082 (0.0013) | 9.3E-10 | -0.0138 (0.0088) | 0.120 | 37.5 | -1.681 |
| Whole body fat-free mass | Alzheimer's disease (Bellenguez 2022) | rs2611732 | 5:95855576 | G/A | 0.602 | 0.0077 (0.0013) | 9.3E-10 | -0.0034 (0.0083) | 0.684 | 37.5 | -0.439 |
| Whole body fat-free mass | Alzheimer's disease (Bellenguez 2022) | rs4980826 | 12:578349 | A/C | 0.396 | 0.0078 (0.0013) | 9.3E-10 | 0.0049 (0.0083) | 0.552 | 37.5 | 0.632 |
| Whole body fat-free mass | Alzheimer's disease (Bellenguez 2022) | rs1910466 | 3:147086268 | C/T | 0.498 | -0.0076 (0.0012) | 9.4E-10 | 0.0123 (0.0081) | 0.131 | 37.5 | -1.612 |
| Whole body fat-free mass | Alzheimer's disease (Bellenguez 2022) | rs7503332 | 17:63563513 | G/T | 0.482 | 0.0077 (0.0013) | 9.4E-10 | 0.0098 (0.0084) | 0.243 | 37.5 | 1.273 |
| Whole body fat-free mass | Alzheimer's disease (Bellenguez 2022) | rs56207600 | 11:126196537 | A/G | 0.111 | 0.0122 (0.0020) | 9.6E-10 | -0.0104 (0.0133) | 0.436 | 37.4 | -0.855 |
| Whole body fat-free mass | Alzheimer's disease (Bellenguez 2022) | rs2332175 | 14:70345411 | A/G | 0.547 | -0.0077 (0.0013) | 9.6E-10 | 0.0009 (0.0082) | 0.913 | 37.4 | -0.117 |
| Whole body fat-free mass | Alzheimer's disease (Bellenguez 2022) | rs11925245 | 3:114183579 | G/A | 0.183 | -0.0099 (0.0016) | 1.0E-09 | 0.0085 (0.0105) | 0.421 | 37.3 | -0.859 |
| Whole body fat-free mass | Alzheimer's disease (Bellenguez 2022) | rs1928850 | 9:16765520 | T/A | 0.922 | 0.0142 (0.0023) | 1.0E-09 | -0.0119 (0.0150) | 0.425 | 37.3 | -0.836 |
| Whole body fat-free mass | Alzheimer's disease (Bellenguez 2022) | rs1941697 | 18:31251276 | A/G | 0.449 | 0.0076 (0.0013) | 1.0E-09 | -0.0089 (0.0082) | 0.280 | 37.3 | -1.165 |
| Whole body fat-free mass | Alzheimer's disease (Bellenguez 2022) | rs4887925 | 16:73784416 | C/G | 0.413 | -0.0077 (0.0013) | 1.2E-09 | -0.0065 (0.0083) | 0.438 | 36.9 | 0.844 |
| Whole body fat-free mass | Alzheimer's disease (Bellenguez 2022) | rs6781248 | 3:13726684 | A/G | 0.546 | 0.0076 (0.0012) | 1.3E-09 | 0.0068 (0.0082) | 0.402 | 36.8 | 0.900 |
| Whole body fat-free mass | Alzheimer's disease (Bellenguez 2022) | rs3853474 | 5:141816555 | T/C | 0.377 | -0.0078 (0.0013) | 1.3E-09 | 0.0093 (0.0084) | 0.267 | 36.8 | -1.199 |
| Whole body fat-free mass | Alzheimer's disease (Bellenguez 2022) | rs9654451 | 5:299543 | T/C | 0.129 | 0.0112 (0.0019) | 1.4E-09 | 0.0099 (0.0122) | 0.420 | 36.7 | 0.882 |
| Whole body fat-free mass | Alzheimer's disease (Bellenguez 2022) | rs8002779 | 13:92015977 | A/G | 0.603 | -0.0077 (0.0013) | 1.5E-09 | -0.0166 (0.0082) | 0.044 | 36.5 | 2.168 |
| Whole body fat-free mass | Alzheimer's disease (Bellenguez 2022) | rs2521349 | 17:67503501 | A/G | 0.385 | 0.0077 (0.0013) | 1.6E-09 | -0.0111 (0.0084) | 0.184 | 36.4 | -1.435 |
| Whole body fat-free mass | Alzheimer's disease (Bellenguez 2022) | rs695922 | 5:53385529 | G/A | 0.837 | -0.0102 (0.0017) | 1.7E-09 | -0.0096 (0.0110) | 0.381 | 36.4 | 0.939 |
| Whole body fat-free mass | Alzheimer's disease (Bellenguez 2022) | rs6563808 | 13:40766662 | C/T | 0.734 | -0.0085 (0.0014) | 1.7E-09 | -0.0046 (0.0091) | 0.615 | 36.3 | 0.539 |
| Whole body fat-free mass | Alzheimer's disease (Bellenguez 2022) | rs5771118 | 22:50714289 | C/T | 0.742 | 0.0086 (0.0014) | 1.8E-09 | -0.0089 (0.0095) | 0.347 | 36.2 | -1.032 |
| Whole body fat-free mass | Alzheimer's disease (Bellenguez 2022) | rs41458449 | 2:46929951 | C/G | 0.144 | -0.0106 (0.0018) | 1.9E-09 | -0.0131 (0.0115) | 0.254 | 36.1 | 1.234 |
| Whole body fat-free mass | Alzheimer's disease (Bellenguez 2022) | rs6721191 | 2:10190115 | G/A | 0.578 | -0.0075 (0.0013) | 2.0E-09 | -0.0065 (0.0083) | 0.435 | 36.0 | 0.865 |
| Whole body fat-free mass | Alzheimer's disease (Bellenguez 2022) | rs72703409 | 4:184225295 | C/G | 0.066 | -0.0150 (0.0025) | 2.0E-09 | 0.0037 (0.0165) | 0.823 | 35.9 | -0.246 |
| Whole body fat-free mass | Alzheimer's disease (Bellenguez 2022) | rs572474 | 10:89791654 | G/A | 0.166 | 0.0100 (0.0017) | 2.0E-09 | -0.0073 (0.0109) | 0.507 | 36.0 | -0.728 |
| Whole body fat-free mass | Alzheimer's disease (Bellenguez 2022) | rs9898189 | 17:80480516 | G/C | 0.644 | -0.0081 (0.0013) | 2.1E-09 | -0.0029 (0.0087) | 0.739 | 35.9 | 0.359 |
| Whole body fat-free mass | Alzheimer's disease (Bellenguez 2022) | rs12731187 | 1:202013757 | T/C | 0.358 | -0.0078 (0.0013) | 2.5E-09 | -0.0047 (0.0086) | 0.586 | 35.5 | 0.605 |
| Whole body fat-free mass | Alzheimer's disease (Bellenguez 2022) | rs9365939 | 6:166336825 | G/A | 0.548 | -0.0074 (0.0012) | 2.5E-09 | 0.0007 (0.0082) | 0.934 | 35.5 | -0.095 |
| Whole body fat-free mass | Alzheimer's disease (Bellenguez 2022) | rs56130943 | 14:105906522 | C/A | 0.233 | 0.0090 (0.0015) | 2.9E-09 | -0.0106 (0.0105) | 0.315 | 35.3 | -1.181 |
| Whole body fat-free mass | Alzheimer's disease (Bellenguez 2022) | rs10939792 | 4:18483405 | G/C | 0.320 | 0.0079 (0.0013) | 3.2E-09 | -0.0012 (0.0087) | 0.891 | 35.1 | -0.152 |
| Whole body fat-free mass | Alzheimer's disease (Bellenguez 2022) | rs876672 | 16:81571600 | G/A | 0.085 | -0.0132 (0.0022) | 3.2E-09 | 0.0159 (0.0150) | 0.288 | 35.0 | -1.201 |
| Whole body fat-free mass | Alzheimer's disease (Bellenguez 2022) | rs7535501 | 1:176462695 | T/C | 0.944 | -0.0159 (0.0027) | 3.3E-09 | -0.0023 (0.0172) | 0.893 | 35.0 | 0.144 |
| Whole body fat-free mass | Alzheimer's disease (Bellenguez 2022) | rs116337081 | 2:183954625 | T/C | 0.070 | 0.0143 (0.0024) | 3.6E-09 | 0.0220 (0.0153) | 0.151 | 34.8 | 1.534 |
| Whole body fat-free mass | Alzheimer's disease (Bellenguez 2022) | rs2920665 | 8:6316097 | G/T | 0.174 | 0.0097 (0.0016) | 3.6E-09 | 0.0060 (0.0106) | 0.573 | 34.8 | 0.618 |
| Whole body fat-free mass | Alzheimer's disease (Bellenguez 2022) | rs1176314 | 13:81221014 | G/T | 0.550 | -0.0074 (0.0013) | 3.6E-09 | -0.0004 (0.0082) | 0.963 | 34.9 | 0.054 |
| Whole body fat-free mass | Alzheimer's disease (Bellenguez 2022) | rs13392079 | 2:169119178 | C/T | 0.756 | -0.0085 (0.0014) | 3.8E-09 | -0.0039 (0.0092) | 0.669 | 34.7 | 0.459 |
| Whole body fat-free mass | Alzheimer's disease (Bellenguez 2022) | rs224048 | 10:64491549 | A/G | 0.550 | -0.0073 (0.0012) | 3.8E-09 | 0.0145 (0.0081) | 0.075 | 34.7 | -1.975 |
| Whole body fat-free mass | Alzheimer's disease (Bellenguez 2022) | rs7280982 | 21:37468223 | A/G | 0.221 | -0.0088 (0.0015) | 4.2E-09 | 0.0045 (0.0097) | 0.641 | 34.5 | -0.509 |
| Whole body fat-free mass | Alzheimer's disease (Bellenguez 2022) | rs116165844 | 20:62610556 | T/G | 0.137 | -0.0106 (0.0018) | 4.3E-09 | 0.0119 (0.0121) | 0.322 | 34.5 | -1.121 |
| Whole body fat-free mass | Alzheimer's disease (Bellenguez 2022) | rs29946 | 19:34299865 | C/T | 0.557 | 0.0075 (0.0013) | 4.5E-09 | 0.0077 (0.0089) | 0.389 | 34.4 | 1.031 |
| Whole body fat-free mass | Alzheimer's disease (Bellenguez 2022) | rs182224 | 5:67316442 | T/C | 0.090 | 0.0127 (0.0022) | 4.6E-09 | -0.0081 (0.0144) | 0.575 | 34.4 | -0.636 |
| Whole body fat-free mass | Alzheimer's disease (Bellenguez 2022) | rs6747657 | 2:112003163 | A/G | 0.282 | 0.0081 (0.0014) | 4.7E-09 | -0.0003 (0.0091) | 0.974 | 34.3 | -0.037 |
| Whole body fat-free mass | Alzheimer's disease (Bellenguez 2022) | rs11263853 | 1:36579648 | A/G | 0.810 | 0.0092 (0.0016) | 4.8E-09 | -0.0021 (0.0103) | 0.841 | 34.2 | -0.227 |
| Whole body fat-free mass | Alzheimer's disease (Bellenguez 2022) | rs9985795 | 4:135213286 | C/T | 0.483 | -0.0073 (0.0012) | 5.1E-09 | 0.0049 (0.0081) | 0.543 | 34.2 | -0.674 |
| Whole body fat-free mass | Alzheimer's disease (Bellenguez 2022) | rs34055910 | 17:64460104 | G/A | 0.379 | -0.0075 (0.0013) | 5.1E-09 | 0.0031 (0.0083) | 0.705 | 34.2 | -0.413 |
| Whole body fat-free mass | Alzheimer's disease (Bellenguez 2022) | rs13185520 | 5:137807260 | A/G | 0.574 | -0.0073 (0.0013) | 5.4E-09 | 0.0119 (0.0082) | 0.147 | 34.1 | -1.629 |
| Whole body fat-free mass | Alzheimer's disease (Bellenguez 2022) | rs116817990 | 1:178989260 | G/A | 0.034 | -0.0201 (0.0034) | 5.7E-09 | 0.0329 (0.0238) | 0.168 | 33.9 | -1.637 |
| Whole body fat-free mass | Alzheimer's disease (Bellenguez 2022) | rs3772051 | 2:241672904 | A/G | 0.237 | -0.0086 (0.0015) | 5.9E-09 | 0.0115 (0.0098) | 0.241 | 33.9 | -1.336 |
| Whole body fat-free mass | Alzheimer's disease (Bellenguez 2022) | rs236587 | 17:68203915 | C/T | 0.737 | -0.0083 (0.0014) | 6.2E-09 | -0.0023 (0.0092) | 0.801 | 33.8 | 0.277 |
| Whole body fat-free mass | Alzheimer's disease (Bellenguez 2022) | rs118173451 | 22:28356600 | C/T | 0.016 | -0.0292 (0.0050) | 6.3E-09 | 0.0152 (0.0364) | 0.677 | 33.7 | -0.521 |
| Whole body fat-free mass | Alzheimer's disease (Bellenguez 2022) | rs6500249 | 16:49778147 | G/A | 0.735 | -0.0082 (0.0014) | 6.4E-09 | -0.0014 (0.0094) | 0.880 | 33.7 | 0.170 |
| Whole body fat-free mass | Alzheimer's disease (Bellenguez 2022) | rs5753630 | 22:31861950 | G/A | 0.439 | 0.0073 (0.0013) | 6.7E-09 | -0.0152 (0.0083) | 0.065 | 33.6 | -2.080 |
| Whole body fat-free mass | Alzheimer's disease (Bellenguez 2022) | rs237738 | 20:47904920 | C/T | 0.823 | 0.0094 (0.0016) | 7.3E-09 | -0.0190 (0.0105) | 0.071 | 33.5 | -2.016 |
| Whole body fat-free mass | Alzheimer's disease (Bellenguez 2022) | rs9800418 | 5:77641008 | C/T | 0.245 | 0.0084 (0.0014) | 7.9E-09 | 0.0125 (0.0095) | 0.186 | 33.3 | 1.495 |
| Whole body fat-free mass | Alzheimer's disease (Bellenguez 2022) | rs1421035 | 10:131136401 | T/C | 0.348 | 0.0076 (0.0013) | 8.0E-09 | 0.0031 (0.0087) | 0.722 | 33.3 | 0.409 |
| Whole body fat-free mass | Alzheimer's disease (Bellenguez 2022) | rs10020631 | 4:69353863 | A/G | 0.248 | -0.0083 (0.0014) | 8.2E-09 | -0.0027 (0.0099) | 0.783 | 33.2 | 0.325 |
| Whole body fat-free mass | Alzheimer's disease (Bellenguez 2022) | rs4676442 | 2:241740907 | C/T | 0.356 | 0.0074 (0.0013) | 9.1E-09 | -0.0102 (0.0085) | 0.231 | 33.0 | -1.373 |
| Whole body fat-free mass | Alzheimer's disease (Bellenguez 2022) | rs9491201 | 6:124934212 | T/C | 0.164 | 0.0096 (0.0017) | 9.5E-09 | -0.0041 (0.0108) | 0.705 | 32.9 | -0.427 |
| Whole body fat-free mass | Alzheimer's disease (Bellenguez 2022) | rs1846221 | 10:4667302 | C/G | 0.175 | -0.0095 (0.0017) | 9.7E-09 | -0.0116 (0.0106) | 0.274 | 32.9 | 1.220 |
| Whole body fat-free mass | Alzheimer's disease (Bellenguez 2022) | rs35651070 | 2:121613739 | A/G | 0.167 | -0.0095 (0.0017) | 9.9E-09 | 0.0104 (0.0113) | 0.356 | 32.9 | -1.094 |
| Whole body fat-free mass | Alzheimer's disease (Bellenguez 2022) | rs2920974 | 2:55537126 | C/A | 0.560 | 0.0072 (0.0013) | 1.1E-08 | -0.0126 (0.0082) | 0.127 | 32.7 | -1.761 |
| Whole body fat-free mass | Alzheimer's disease (Bellenguez 2022) | rs35612982 | 6:20682622 | C/T | 0.178 | -0.0092 (0.0016) | 1.1E-08 | -0.0107 (0.0104) | 0.305 | 32.7 | 1.157 |
| Whole body fat-free mass | Alzheimer's disease (Bellenguez 2022) | rs8091374 | 18:71904385 | A/G | 0.161 | -0.0099 (0.0017) | 1.1E-08 | 0.0300 (0.0118) | 0.011 | 32.7 | -3.039 |
| Whole body fat-free mass | Alzheimer's disease (Bellenguez 2022) | rs34345560 | 11:69081998 | A/G | 0.195 | 0.0090 (0.0016) | 1.2E-08 | 0.0015 (0.0106) | 0.884 | 32.4 | 0.167 |
| Whole body fat-free mass | Alzheimer's disease (Bellenguez 2022) | rs6743107 | 2:100844781 | T/C | 0.275 | 0.0079 (0.0014) | 1.3E-08 | -0.0069 (0.0091) | 0.449 | 32.3 | -0.877 |
| Whole body fat-free mass | Alzheimer's disease (Bellenguez 2022) | rs9783665 | 14:103271158 | A/T | 0.653 | -0.0074 (0.0013) | 1.3E-08 | 0.0313 (0.0085) | 2.4E-04 | 32.3 | -4.220 |
| Whole body fat-free mass | Alzheimer's disease (Bellenguez 2022) | rs8088739 | 18:60767088 | C/T | 0.244 | 0.0082 (0.0014) | 1.3E-08 | -0.0008 (0.0095) | 0.932 | 32.3 | -0.097 |
| Whole body fat-free mass | Alzheimer's disease (Bellenguez 2022) | rs34831515 | 19:17275777 | T/C | 0.232 | -0.0084 (0.0015) | 1.3E-08 | -0.0063 (0.0104) | 0.543 | 32.3 | 0.750 |
| Whole body fat-free mass | Alzheimer's disease (Bellenguez 2022) | rs35050648 | 19:46991243 | T/G | 0.236 | 0.0083 (0.0015) | 1.6E-08 | 0.0181 (0.0102) | 0.075 | 32.0 | 2.184 |
| Whole body fat-free mass | Alzheimer's disease (Bellenguez 2022) | rs62460525 | 7:44902676 | A/C | 0.070 | 0.0137 (0.0024) | 1.7E-08 | -0.0274 (0.0162) | 0.091 | 31.8 | -1.998 |
| Whole body fat-free mass | Alzheimer's disease (Bellenguez 2022) | rs2740761 | 7:55255986 | T/C | 0.208 | 0.0087 (0.0015) | 1.7E-08 | 0.0082 (0.0100) | 0.408 | 31.8 | 0.940 |
| Whole body fat-free mass | Alzheimer's disease (Bellenguez 2022) | rs113978196 | 15:75916965 | A/G | 0.251 | -0.0081 (0.0014) | 1.8E-08 | 0.0028 (0.0095) | 0.766 | 31.8 | -0.347 |
| Whole body fat-free mass | Alzheimer's disease (Bellenguez 2022) | rs10848288 | 12:131600054 | T/C | 0.304 | -0.0076 (0.0014) | 1.9E-08 | -0.0012 (0.0090) | 0.895 | 31.6 | 0.158 |
| Whole body fat-free mass | Alzheimer's disease (Bellenguez 2022) | rs3751866 | 16:84771304 | C/T | 0.247 | -0.0081 (0.0014) | 1.9E-08 | -0.0109 (0.0095) | 0.253 | 31.6 | 1.342 |
| Whole body fat-free mass | Alzheimer's disease (Bellenguez 2022) | rs111598585 | 4:171635471 | T/C | 0.209 | -0.0086 (0.0015) | 2.0E-08 | -0.0106 (0.0100) | 0.290 | 31.5 | 1.235 |
| Whole body fat-free mass | Alzheimer's disease (Bellenguez 2022) | rs2884345 | 14:53323211 | A/G | 0.710 | -0.0077 (0.0014) | 2.1E-08 | -0.0418 (0.0090) | 3.3E-06 | 31.4 | 5.436 |
| Whole body fat-free mass | Alzheimer's disease (Bellenguez 2022) | rs2252909 | 17:2278609 | T/C | 0.657 | -0.0075 (0.0013) | 2.1E-08 | 0.0040 (0.0085) | 0.636 | 31.4 | -0.536 |
| Whole body fat-free mass | Alzheimer's disease (Bellenguez 2022) | rs12553221 | 9:37234008 | A/G | 0.362 | 0.0073 (0.0013) | 2.2E-08 | 0.0120 (0.0085) | 0.161 | 31.3 | 1.654 |
| Whole body fat-free mass | Alzheimer's disease (Bellenguez 2022) | rs498685 | 18:13088673 | C/T | 0.594 | -0.0071 (0.0013) | 2.2E-08 | 0.0034 (0.0083) | 0.685 | 31.3 | -0.480 |
| Whole body fat-free mass | Alzheimer's disease (Bellenguez 2022) | rs17011108 | 4:86800806 | C/T | 0.307 | 0.0076 (0.0014) | 2.3E-08 | -0.0034 (0.0089) | 0.703 | 31.2 | -0.449 |
| Whole body fat-free mass | Alzheimer's disease (Bellenguez 2022) | rs12795042 | 11:133658168 | C/A | 0.622 | 0.0073 (0.0013) | 2.3E-08 | 0.0038 (0.0085) | 0.657 | 31.3 | 0.524 |
| Whole body fat-free mass | Alzheimer's disease (Bellenguez 2022) | rs4800670 | 18:23415263 | C/G | 0.386 | -0.0072 (0.0013) | 2.3E-08 | -0.0069 (0.0084) | 0.412 | 31.3 | 0.962 |
| Whole body fat-free mass | Alzheimer's disease (Bellenguez 2022) | rs73189390 | 21:17383170 | A/G | 0.184 | -0.0090 (0.0016) | 2.3E-08 | -0.0152 (0.0104) | 0.144 | 31.3 | 1.681 |
| Whole body fat-free mass | Alzheimer's disease (Bellenguez 2022) | rs55854145 | 18:45928049 | C/A | 0.055 | -0.0153 (0.0027) | 2.4E-08 | 0.0136 (0.0180) | 0.449 | 31.1 | -0.888 |
| Whole body fat-free mass | Alzheimer's disease (Bellenguez 2022) | rs7513326 | 1:159895536 | A/G | 0.508 | -0.0070 (0.0013) | 2.5E-08 | 0.0053 (0.0084) | 0.532 | 31.1 | -0.756 |
| Whole body fat-free mass | Alzheimer's disease (Bellenguez 2022) | rs73158212 | 7:148383377 | G/A | 0.224 | -0.0084 (0.0015) | 2.5E-08 | 0.0009 (0.0099) | 0.925 | 31.1 | -0.107 |
| Whole body fat-free mass | Alzheimer's disease (Bellenguez 2022) | rs6514066 | 20:44500794 | G/T | 0.624 | 0.0072 (0.0013) | 2.5E-08 | -0.0076 (0.0083) | 0.362 | 31.1 | -1.060 |
| Whole body fat-free mass | Alzheimer's disease (Bellenguez 2022) | rs2427320 | 20:60976723 | G/A | 0.232 | -0.0082 (0.0015) | 2.5E-08 | 0.0214 (0.0097) | 0.028 | 31.1 | -2.601 |
| Whole body fat-free mass | Alzheimer's disease (Bellenguez 2022) | rs4821940 | 22:40659573 | C/T | 0.550 | -0.0070 (0.0013) | 2.5E-08 | 0.0151 (0.0082) | 0.066 | 31.1 | -2.157 |
| Whole body fat-free mass | Alzheimer's disease (Bellenguez 2022) | rs1168768 | 12:66509650 | T/C | 0.975 | 0.0221 (0.0040) | 2.6E-08 | -0.0102 (0.0289) | 0.724 | 31.0 | -0.461 |
| Whole body fat-free mass | Alzheimer's disease (Bellenguez 2022) | rs7095768 | 10:53256505 | T/G | 0.263 | 0.0078 (0.0014) | 2.7E-08 | -0.0006 (0.0091) | 0.944 | 30.9 | -0.077 |
| Whole body fat-free mass | Alzheimer's disease (Bellenguez 2022) | rs2293177 | 7:140244560 | T/C | 0.313 | 0.0074 (0.0013) | 2.9E-08 | -0.0101 (0.0087) | 0.248 | 30.8 | -1.361 |
| Whole body fat-free mass | Alzheimer's disease (Bellenguez 2022) | rs13125082 | 4:57787000 | G/T | 0.278 | -0.0076 (0.0014) | 3.4E-08 | -0.0064 (0.0090) | 0.478 | 30.5 | 0.838 |
| Whole body fat-free mass | Alzheimer's disease (Bellenguez 2022) | rs7683836 | 4:180167906 | A/G | 0.557 | -0.0069 (0.0013) | 3.4E-08 | 0.0037 (0.0083) | 0.654 | 30.5 | -0.536 |
| Whole body fat-free mass | Alzheimer's disease (Bellenguez 2022) | rs7150606 | 14:68413076 | T/G | 0.142 | -0.0098 (0.0018) | 3.4E-08 | 0.0021 (0.0113) | 0.856 | 30.5 | -0.213 |
| Whole body fat-free mass | Alzheimer's disease (Bellenguez 2022) | rs926436 | 1:22563429 | A/G | 0.857 | 0.0098 (0.0018) | 3.5E-08 | -0.0110 (0.0113) | 0.327 | 30.4 | -1.125 |
| Whole body fat-free mass | Alzheimer's disease (Bellenguez 2022) | rs34345690 | 3:192445575 | G/A | 0.227 | 0.0082 (0.0015) | 3.5E-08 | -0.0027 (0.0097) | 0.785 | 30.4 | -0.331 |
| Whole body fat-free mass | Alzheimer's disease (Bellenguez 2022) | rs11021307 | 11:95501407 | T/C | 0.418 | 0.0069 (0.0013) | 3.6E-08 | 0.0112 (0.0082) | 0.173 | 30.4 | 1.616 |
| Whole body fat-free mass | Alzheimer's disease (Bellenguez 2022) | rs294382 | 11:30607515 | C/A | 0.720 | -0.0076 (0.0014) | 4.0E-08 | -0.0106 (0.0090) | 0.240 | 30.1 | 1.395 |
| Whole body fat-free mass | Alzheimer's disease (Bellenguez 2022) | rs434072 | 3:12916946 | G/T | 0.709 | -0.0075 (0.0014) | 4.1E-08 | 0.0003 (0.0090) | 0.971 | 30.1 | -0.040 |
| Whole body fat-free mass | Alzheimer's disease (Bellenguez 2022) | rs1028197 | 4:124977659 | T/C | 0.225 | 0.0082 (0.0015) | 4.1E-08 | 0.0135 (0.0097) | 0.165 | 30.1 | 1.648 |
| Whole body fat-free mass | Alzheimer's disease (Bellenguez 2022) | rs12213409 | 6:142610314 | G/T | 0.485 | -0.0068 (0.0012) | 4.7E-08 | -0.0134 (0.0082) | 0.102 | 29.8 | 1.966 |
| Whole body fat-free mass | All-cause dementia (FinnGen) | rs12967135 | 18:57849023 | A/G | 0.233 | 0.0433 (0.0015) | 2.3E-190 | 0.0397 (0.0246) | 0.106 | 866.1 | 0.917 |
| Whole body fat-free mass | All-cause dementia (FinnGen) | rs56094641 | 16:53806453 | G/A | 0.405 | 0.0361 (0.0013) | 1.0E-178 | 0.0089 (0.0194) | 0.645 | 812.5 | 0.247 |
| Whole body fat-free mass | All-cause dementia (FinnGen) | rs143384 | 20:34025756 | G/A | 0.404 | 0.0354 (0.0013) | 1.5E-171 | -0.0099 (0.0193) | 0.610 | 779.6 | -0.280 |
| Whole body fat-free mass | All-cause dementia (FinnGen) | rs9853018 | 3:141101961 | T/C | 0.443 | 0.0300 (0.0012) | 6.3E-128 | 0.0504 (0.0192) | 0.009 | 579.0 | 1.681 |
| Whole body fat-free mass | All-cause dementia (FinnGen) | rs2744956 | 6:34618937 | C/T | 0.139 | 0.0378 (0.0018) | 2.9E-99 | -0.0221 (0.0242) | 0.361 | 447.2 | -0.585 |
| Whole body fat-free mass | All-cause dementia (FinnGen) | rs1351394 | 12:66351826 | C/T | 0.512 | -0.0258 (0.0012) | 5.3E-96 | -0.0264 (0.0191) | 0.167 | 432.2 | 1.024 |
| Whole body fat-free mass | All-cause dementia (FinnGen) | rs7740107 | 6:130374461 | A/T | 0.736 | -0.0291 (0.0014) | 1.1E-95 | 0.0376 (0.0215) | 0.081 | 430.7 | -1.290 |
| Whole body fat-free mass | All-cause dementia (FinnGen) | rs12995666 | 2:629959 | C/T | 0.828 | 0.0337 (0.0016) | 1.1E-93 | -0.0090 (0.0258) | 0.729 | 421.6 | -0.267 |
| Whole body fat-free mass | All-cause dementia (FinnGen) | rs72656010 | 8:57122215 | C/T | 0.132 | -0.0366 (0.0018) | 2.8E-88 | 0.0598 (0.0287) | 0.037 | 396.7 | -1.632 |
| Whole body fat-free mass | All-cause dementia (FinnGen) | rs76895963 | 12:4384844 | G/T | 0.021 | 0.0952 (0.0048) | 2.8E-87 | -0.0735 (0.0577) | 0.203 | 392.2 | -0.772 |
| Whole body fat-free mass | All-cause dementia (FinnGen) | rs10269774 | 7:92253972 | A/G | 0.326 | 0.0262 (0.0013) | 4.7E-87 | 0.0047 (0.0207) | 0.822 | 391.1 | 0.180 |
| Whole body fat-free mass | All-cause dementia (FinnGen) | rs2225226 | 13:51127270 | T/C | 0.216 | -0.0298 (0.0015) | 3.1E-86 | -0.0181 (0.0220) | 0.411 | 387.4 | 0.607 |
| Whole body fat-free mass | All-cause dementia (FinnGen) | rs34517439 | 1:78450517 | A/C | 0.122 | 0.0372 (0.0019) | 3.3E-84 | -0.0318 (0.0290) | 0.272 | 378.1 | -0.854 |
| Whole body fat-free mass | All-cause dementia (FinnGen) | rs7671110 | 4:17874089 | T/C | 0.158 | -0.0318 (0.0017) | 2.8E-78 | 0.0432 (0.0326) | 0.186 | 350.8 | -1.359 |
| Whole body fat-free mass | All-cause dementia (FinnGen) | rs7223535 | 17:29211667 | A/G | 0.270 | -0.0251 (0.0014) | 1.0E-71 | 0.0033 (0.0212) | 0.878 | 320.7 | -0.132 |
| Whole body fat-free mass | All-cause dementia (FinnGen) | rs77165542 | 2:430975 | T/C | 0.035 | -0.0585 (0.0034) | 6.6E-67 | -0.1662 (0.0731) | 0.023 | 298.6 | 2.842 |
| Whole body fat-free mass | All-cause dementia (FinnGen) | rs2005172 | 17:61996255 | C/A | 0.640 | 0.0226 (0.0013) | 1.0E-66 | 0.0001 (0.0195) | 0.995 | 297.7 | 0.004 |
| Whole body fat-free mass | All-cause dementia (FinnGen) | rs4788218 | 16:30055750 | C/T | 0.401 | 0.0218 (0.0013) | 3.6E-66 | -0.0239 (0.0199) | 0.229 | 295.2 | -1.097 |
| Whole body fat-free mass | All-cause dementia (FinnGen) | rs4369779 | 18:20735408 | C/T | 0.789 | 0.0260 (0.0015) | 2.6E-65 | 0.0371 (0.0228) | 0.104 | 291.3 | 1.427 |
| Whole body fat-free mass | All-cause dementia (FinnGen) | rs4240326 | 4:145839264 | G/A | 0.550 | -0.0212 (0.0012) | 3.7E-65 | -0.0373 (0.0192) | 0.051 | 290.6 | 1.757 |
| Whole body fat-free mass | All-cause dementia (FinnGen) | rs4899012 | 14:61003889 | C/G | 0.607 | -0.0215 (0.0013) | 1.1E-63 | 0.0317 (0.0210) | 0.131 | 283.8 | -1.477 |
| Whole body fat-free mass | All-cause dementia (FinnGen) | rs527248 | 1:177875514 | G/A | 0.209 | 0.0255 (0.0015) | 2.5E-63 | -0.0199 (0.0248) | 0.421 | 282.2 | -0.779 |
| Whole body fat-free mass | All-cause dementia (FinnGen) | rs76798800 | 1:154994978 | T/G | 0.266 | 0.0228 (0.0014) | 2.3E-59 | -0.0513 (0.0227) | 0.024 | 264.0 | -2.252 |
| Whole body fat-free mass | All-cause dementia (FinnGen) | rs9634212 | 12:93993266 | A/C | 0.221 | 0.0237 (0.0015) | 4.0E-56 | 0.0014 (0.0221) | 0.950 | 249.2 | 0.059 |
| Whole body fat-free mass | All-cause dementia (FinnGen) | rs9398171 | 6:108983527 | T/C | 0.711 | 0.0208 (0.0014) | 1.8E-52 | -0.0066 (0.0199) | 0.742 | 232.4 | -0.317 |
| Whole body fat-free mass | All-cause dementia (FinnGen) | rs34879158 | 20:32300634 | C/A | 0.263 | -0.0215 (0.0014) | 2.2E-52 | -0.0216 (0.0226) | 0.339 | 232.0 | 1.003 |
| Whole body fat-free mass | All-cause dementia (FinnGen) | rs2524137 | 6:31264582 | T/C | 0.696 | -0.0206 (0.0014) | 2.3E-52 | 0.0110 (0.0223) | 0.621 | 231.9 | -0.535 |
| Whole body fat-free mass | All-cause dementia (FinnGen) | rs2533879 | 7:2859847 | A/G | 0.300 | -0.0206 (0.0014) | 3.4E-52 | 0.0152 (0.0198) | 0.444 | 231.1 | -0.738 |
| Whole body fat-free mass | All-cause dementia (FinnGen) | rs7033487 | 9:119129257 | C/T | 0.198 | -0.0235 (0.0016) | 4.5E-51 | -0.0545 (0.0228) | 0.017 | 226.0 | 2.322 |
| Whole body fat-free mass | All-cause dementia (FinnGen) | rs1582931 | 5:122657199 | A/G | 0.473 | -0.0188 (0.0013) | 8.7E-51 | 0.0238 (0.0191) | 0.213 | 224.7 | -1.268 |
| Whole body fat-free mass | All-cause dementia (FinnGen) | rs1260326 | 2:27730940 | C/T | 0.604 | 0.0189 (0.0013) | 1.1E-50 | -0.0128 (0.0201) | 0.526 | 224.2 | -0.677 |
| Whole body fat-free mass | All-cause dementia (FinnGen) | rs41271299 | 6:19839415 | T/C | 0.051 | 0.0419 (0.0028) | 2.3E-50 | 0.0524 (0.0757) | 0.489 | 222.7 | 1.252 |
| Whole body fat-free mass | All-cause dementia (FinnGen) | rs7952436 | 11:67024534 | T/C | 0.082 | -0.0336 (0.0023) | 6.7E-50 | 0.0165 (0.0437) | 0.705 | 220.6 | -0.491 |
| Whole body fat-free mass | All-cause dementia (FinnGen) | rs894360 | 8:135601194 | C/T | 0.366 | -0.0190 (0.0013) | 5.7E-49 | -0.0126 (0.0192) | 0.511 | 216.3 | 0.662 |
| Whole body fat-free mass | All-cause dementia (FinnGen) | rs11243202 | 6:7719065 | C/T | 0.486 | 0.0183 (0.0012) | 6.3E-49 | 0.0058 (0.0192) | 0.764 | 216.2 | 0.317 |
| Whole body fat-free mass | All-cause dementia (FinnGen) | rs6946415 | 7:150684548 | G/A | 0.627 | 0.0189 (0.0013) | 6.3E-49 | -0.0152 (0.0202) | 0.452 | 216.1 | -0.804 |
| Whole body fat-free mass | All-cause dementia (FinnGen) | rs2885697 | 1:41544279 | T/G | 0.665 | -0.0189 (0.0013) | 5.1E-47 | 0.0256 (0.0202) | 0.206 | 207.4 | -1.358 |
| Whole body fat-free mass | All-cause dementia (FinnGen) | rs72885917 | 2:172416376 | C/A | 0.247 | -0.0205 (0.0014) | 2.2E-46 | -0.0147 (0.0244) | 0.545 | 204.4 | 0.718 |
| Whole body fat-free mass | All-cause dementia (FinnGen) | rs78378222 | 17:7571752 | G/T | 0.012 | 0.0820 (0.0058) | 7.4E-46 | -0.1299 (0.0749) | 0.083 | 202.1 | -1.585 |
| Whole body fat-free mass | All-cause dementia (FinnGen) | rs73175572 | 3:185490184 | G/A | 0.112 | 0.0280 (0.0020) | 6.1E-45 | 0.0090 (0.0299) | 0.763 | 197.9 | 0.322 |
| Whole body fat-free mass | All-cause dementia (FinnGen) | rs28620532 | 9:98216876 | G/A | 0.349 | 0.0182 (0.0013) | 6.4E-44 | 0.0101 (0.0199) | 0.610 | 193.2 | 0.555 |
| Whole body fat-free mass | All-cause dementia (FinnGen) | rs7842996 | 8:78107140 | A/T | 0.284 | 0.0192 (0.0014) | 7.7E-44 | -0.0066 (0.0231) | 0.777 | 192.8 | -0.344 |
| Whole body fat-free mass | All-cause dementia (FinnGen) | rs7129320 | 11:68388220 | A/G | 0.166 | -0.0231 (0.0017) | 1.1E-43 | 0.0292 (0.0339) | 0.389 | 192.1 | -1.262 |
| Whole body fat-free mass | All-cause dementia (FinnGen) | rs71385734 | 16:2160503 | G/T | 0.170 | -0.0231 (0.0017) | 1.2E-43 | 0.0232 (0.0251) | 0.354 | 191.9 | -1.006 |
| Whole body fat-free mass | All-cause dementia (FinnGen) | rs146851424 | 13:50377910 | C/A | 0.022 | 0.0597 (0.0043) | 1.6E-43 | -0.0915 (0.0729) | 0.209 | 191.3 | -1.533 |
| Whole body fat-free mass | All-cause dementia (FinnGen) | rs62372052 | 5:42724294 | G/A | 0.110 | 0.0266 (0.0020) | 6.8E-41 | -0.0021 (0.0293) | 0.942 | 179.3 | -0.079 |
| Whole body fat-free mass | All-cause dementia (FinnGen) | rs1478575 | 2:218278555 | A/T | 0.684 | 0.0175 (0.0013) | 1.7E-39 | -0.0178 (0.0194) | 0.357 | 172.9 | -1.015 |
| Whole body fat-free mass | All-cause dementia (FinnGen) | rs2071286 | 6:32179896 | T/C | 0.182 | -0.0209 (0.0016) | 4.2E-39 | -0.0090 (0.0224) | 0.687 | 171.1 | 0.431 |
| Whole body fat-free mass | All-cause dementia (FinnGen) | rs2101975 | 4:106216667 | G/A | 0.432 | -0.0164 (0.0013) | 7.4E-39 | -0.0208 (0.0192) | 0.278 | 170.0 | 1.271 |
| Whole body fat-free mass | All-cause dementia (FinnGen) | rs62515437 | 8:57160328 | T/G | 0.225 | 0.0193 (0.0015) | 1.4E-38 | -0.0151 (0.0210) | 0.471 | 168.7 | -0.782 |
| Whole body fat-free mass | All-cause dementia (FinnGen) | rs2307111 | 5:75003678 | C/T | 0.395 | -0.0164 (0.0013) | 2.1E-38 | 0.0100 (0.0194) | 0.607 | 167.9 | -0.608 |
| Whole body fat-free mass | All-cause dementia (FinnGen) | rs1573891 | 15:99186488 | C/G | 0.158 | -0.0220 (0.0017) | 7.5E-38 | 0.0797 (0.0367) | 0.030 | 165.4 | -3.617 |
| Whole body fat-free mass | All-cause dementia (FinnGen) | rs36000545 | 17:79093822 | G/A | 0.396 | -0.0166 (0.0013) | 8.2E-38 | 0.0095 (0.0203) | 0.639 | 165.2 | -0.574 |
| Whole body fat-free mass | All-cause dementia (FinnGen) | rs485554 | 3:172137343 | C/G | 0.315 | 0.0171 (0.0013) | 1.8E-37 | -0.0029 (0.0204) | 0.887 | 163.7 | -0.169 |
| Whole body fat-free mass | All-cause dementia (FinnGen) | rs3810291 | 19:47569003 | A/G | 0.675 | 0.0170 (0.0013) | 2.4E-37 | -0.0178 (0.0198) | 0.368 | 163.1 | -1.050 |
| Whole body fat-free mass | All-cause dementia (FinnGen) | rs1047891 | 2:211540507 | A/C | 0.316 | 0.0169 (0.0013) | 6.0E-37 | -0.0402 (0.0206) | 0.051 | 161.3 | -2.381 |
| Whole body fat-free mass | All-cause dementia (FinnGen) | rs72892910 | 6:50816887 | T/G | 0.172 | 0.0207 (0.0016) | 2.5E-36 | 0.0049 (0.0239) | 0.838 | 158.4 | 0.237 |
| Whole body fat-free mass | All-cause dementia (FinnGen) | rs11030112 | 11:27705188 | A/G | 0.319 | 0.0167 (0.0013) | 3.6E-36 | -0.0195 (0.0203) | 0.337 | 157.7 | -1.166 |
| Whole body fat-free mass | All-cause dementia (FinnGen) | rs59985551 | 2:56106928 | T/C | 0.226 | -0.0185 (0.0015) | 8.6E-36 | 0.0390 (0.0229) | 0.088 | 156.0 | -2.114 |
| Whole body fat-free mass | All-cause dementia (FinnGen) | rs4282339 | 5:168256240 | A/G | 0.208 | -0.0189 (0.0015) | 2.4E-35 | -0.0054 (0.0245) | 0.826 | 153.9 | 0.285 |
| Whole body fat-free mass | All-cause dementia (FinnGen) | rs7132908 | 12:50263148 | A/G | 0.384 | 0.0158 (0.0013) | 2.9E-35 | -0.0010 (0.0197) | 0.961 | 153.6 | -0.063 |
| Whole body fat-free mass | All-cause dementia (FinnGen) | rs12713004 | 2:23896049 | G/A | 0.725 | 0.0172 (0.0014) | 3.9E-35 | -0.0115 (0.0250) | 0.647 | 153.0 | -0.670 |
| Whole body fat-free mass | All-cause dementia (FinnGen) | rs10773172 | 12:122939655 | A/G | 0.742 | 0.0175 (0.0014) | 6.6E-35 | 0.0250 (0.0243) | 0.302 | 151.9 | 1.429 |
| Whole body fat-free mass | All-cause dementia (FinnGen) | rs35506085 | 11:2165576 | A/G | 0.185 | -0.0198 (0.0016) | 8.1E-35 | 0.0052 (0.0254) | 0.837 | 151.5 | -0.262 |
| Whole body fat-free mass | All-cause dementia (FinnGen) | rs25849 | 16:2265071 | G/C | 0.289 | 0.0169 (0.0014) | 1.3E-34 | -0.0106 (0.0200) | 0.595 | 150.5 | -0.628 |
| Whole body fat-free mass | All-cause dementia (FinnGen) | rs2897968 | 12:46763686 | A/G | 0.607 | 0.0156 (0.0013) | 1.4E-34 | -0.0185 (0.0195) | 0.342 | 150.4 | -1.185 |
| Whole body fat-free mass | All-cause dementia (FinnGen) | rs3853252 | 6:152170247 | A/G | 0.455 | 0.0153 (0.0012) | 1.7E-34 | -0.0068 (0.0194) | 0.728 | 150.0 | -0.445 |
| Whole body fat-free mass | All-cause dementia (FinnGen) | rs9372837 | 6:126638124 | A/G | 0.471 | 0.0152 (0.0013) | 6.8E-34 | 0.0099 (0.0192) | 0.606 | 147.3 | 0.652 |
| Whole body fat-free mass | All-cause dementia (FinnGen) | rs12314162 | 12:124826676 | T/C | 0.161 | 0.0204 (0.0017) | 1.5E-33 | -0.0163 (0.0270) | 0.546 | 145.7 | -0.800 |
| Whole body fat-free mass | All-cause dementia (FinnGen) | rs9892365 | 17:59491384 | G/A | 0.670 | -0.0158 (0.0013) | 8.9E-33 | 0.0019 (0.0215) | 0.928 | 142.2 | -0.120 |
| Whole body fat-free mass | All-cause dementia (FinnGen) | rs34776209 | 7:23513093 | T/C | 0.247 | -0.0171 (0.0014) | 1.5E-32 | -0.0124 (0.0236) | 0.599 | 141.1 | 0.725 |
| Whole body fat-free mass | All-cause dementia (FinnGen) | rs244711 | 5:176509193 | T/C | 0.686 | 0.0168 (0.0014) | 1.2E-31 | -0.0576 (0.0208) | 0.006 | 136.9 | -3.434 |
| Whole body fat-free mass | All-cause dementia (FinnGen) | rs13043303 | 20:51091385 | A/G | 0.183 | -0.0186 (0.0016) | 6.2E-31 | 0.0125 (0.0263) | 0.635 | 133.7 | -0.671 |
| Whole body fat-free mass | All-cause dementia (FinnGen) | rs41311445 | 22:42070374 | C/A | 0.096 | -0.0244 (0.0021) | 1.6E-30 | -0.0070 (0.0280) | 0.802 | 131.9 | 0.287 |
| Whole body fat-free mass | All-cause dementia (FinnGen) | rs2531991 | 16:4023553 | A/G | 0.747 | 0.0164 (0.0014) | 2.3E-30 | -0.0117 (0.0217) | 0.589 | 131.1 | -0.713 |
| Whole body fat-free mass | All-cause dementia (FinnGen) | rs6235 | 5:95728898 | G/C | 0.268 | 0.0159 (0.0014) | 8.3E-30 | -0.0215 (0.0212) | 0.311 | 128.6 | -1.355 |
| Whole body fat-free mass | All-cause dementia (FinnGen) | rs11014285 | 10:25178864 | A/G | 0.165 | 0.0192 (0.0017) | 1.1E-29 | 0.0128 (0.0319) | 0.688 | 128.1 | 0.668 |
| Whole body fat-free mass | All-cause dementia (FinnGen) | rs1022523 | 1:176788394 | A/G | 0.727 | 0.0156 (0.0014) | 4.4E-29 | 0.0032 (0.0210) | 0.880 | 125.3 | 0.206 |
| Whole body fat-free mass | All-cause dementia (FinnGen) | rs12072845 | 1:214630757 | A/G | 0.395 | -0.0140 (0.0013) | 2.8E-28 | -0.0110 (0.0201) | 0.586 | 121.7 | 0.788 |
| Whole body fat-free mass | All-cause dementia (FinnGen) | rs11880992 | 19:2176403 | A/G | 0.408 | 0.0139 (0.0013) | 4.6E-28 | -0.0098 (0.0199) | 0.623 | 120.6 | -0.704 |
| Whole body fat-free mass | All-cause dementia (FinnGen) | rs2270894 | 3:9975386 | G/C | 0.203 | -0.0174 (0.0016) | 1.0E-27 | -0.0117 (0.0218) | 0.591 | 119.0 | 0.671 |
| Whole body fat-free mass | All-cause dementia (FinnGen) | rs111640872 | 19:30290357 | C/G | 0.331 | 0.0145 (0.0013) | 1.1E-27 | 0.0078 (0.0201) | 0.699 | 118.9 | 0.539 |
| Whole body fat-free mass | All-cause dementia (FinnGen) | rs6800021 | 3:50190346 | A/G | 0.425 | 0.0136 (0.0013) | 1.6E-27 | 0.0610 (0.0205) | 0.003 | 118.1 | 4.473 |
| Whole body fat-free mass | All-cause dementia (FinnGen) | rs11065979 | 12:112059557 | T/C | 0.438 | -0.0135 (0.0013) | 4.3E-27 | 0.0104 (0.0196) | 0.594 | 116.2 | -0.771 |
| Whole body fat-free mass | All-cause dementia (FinnGen) | rs17277008 | 1:172105162 | C/T | 0.313 | 0.0144 (0.0013) | 4.6E-27 | 0.0213 (0.0218) | 0.329 | 116.1 | 1.483 |
| Whole body fat-free mass | All-cause dementia (FinnGen) | rs2900208 | 12:11878464 | A/C | 0.354 | 0.0140 (0.0013) | 4.9E-27 | -0.0149 (0.0197) | 0.449 | 115.9 | -1.065 |
| Whole body fat-free mass | All-cause dementia (FinnGen) | rs12657771 | 5:36787962 | A/G | 0.439 | -0.0134 (0.0013) | 9.4E-27 | -0.0019 (0.0194) | 0.924 | 114.7 | 0.141 |
| Whole body fat-free mass | All-cause dementia (FinnGen) | rs12879423 | 14:25927832 | G/A | 0.679 | 0.0142 (0.0013) | 3.4E-26 | 0.0229 (0.0199) | 0.248 | 112.1 | 1.614 |
| Whole body fat-free mass | All-cause dementia (FinnGen) | rs6762578 | 3:128992047 | A/G | 0.778 | 0.0157 (0.0015) | 8.9E-26 | 0.0246 (0.0255) | 0.336 | 110.2 | 1.564 |
| Whole body fat-free mass | All-cause dementia (FinnGen) | rs7141420 | 14:79899454 | T/C | 0.516 | 0.0131 (0.0013) | 1.2E-25 | -0.0120 (0.0194) | 0.537 | 109.5 | -0.915 |
| Whole body fat-free mass | All-cause dementia (FinnGen) | rs3822742 | 5:139059017 | A/C | 0.371 | 0.0134 (0.0013) | 1.6E-25 | -0.0281 (0.0205) | 0.169 | 109.0 | -2.092 |
| Whole body fat-free mass | All-cause dementia (FinnGen) | rs12041740 | 1:11236524 | A/G | 0.740 | -0.0147 (0.0014) | 2.1E-25 | 0.0006 (0.0213) | 0.978 | 108.5 | -0.041 |
| Whole body fat-free mass | All-cause dementia (FinnGen) | rs12095997 | 1:51391845 | T/C | 0.089 | 0.0225 (0.0022) | 2.7E-25 | 0.0168 (0.0415) | 0.687 | 108.0 | 0.746 |
| Whole body fat-free mass | All-cause dementia (FinnGen) | rs6570509 | 6:142716286 | T/G | 0.287 | -0.0142 (0.0014) | 3.9E-25 | -0.0057 (0.0219) | 0.794 | 107.3 | 0.402 |
| Whole body fat-free mass | All-cause dementia (FinnGen) | rs7080472 | 10:96012950 | T/G | 0.425 | 0.0130 (0.0013) | 5.2E-25 | -0.0142 (0.0204) | 0.488 | 106.7 | -1.092 |
| Whole body fat-free mass | All-cause dementia (FinnGen) | rs1924936 | 13:78443297 | A/T | 0.774 | 0.0153 (0.0015) | 7.8E-25 | 0.0165 (0.0248) | 0.506 | 105.9 | 1.077 |
| Whole body fat-free mass | All-cause dementia (FinnGen) | rs10283100 | 8:120596023 | G/A | 0.945 | 0.0279 (0.0027) | 8.7E-25 | -0.0149 (0.0370) | 0.688 | 105.7 | -0.534 |
| Whole body fat-free mass | All-cause dementia (FinnGen) | rs11240565 | 1:205722958 | T/C | 0.398 | 0.0130 (0.0013) | 8.8E-25 | -0.0289 (0.0199) | 0.146 | 105.6 | -2.228 |
| Whole body fat-free mass | All-cause dementia (FinnGen) | rs365352 | 5:77401152 | A/G | 0.244 | -0.0148 (0.0014) | 9.3E-25 | 0.0125 (0.0246) | 0.612 | 105.5 | -0.845 |
| Whole body fat-free mass | All-cause dementia (FinnGen) | rs9938120 | 16:19977733 | T/C | 0.144 | -0.0184 (0.0018) | 9.4E-25 | -0.0040 (0.0291) | 0.890 | 105.5 | 0.217 |
| Whole body fat-free mass | All-cause dementia (FinnGen) | rs9350850 | 6:81050236 | C/T | 0.079 | 0.0234 (0.0023) | 1.5E-24 | 0.0257 (0.0303) | 0.396 | 104.6 | 1.099 |
| Whole body fat-free mass | All-cause dementia (FinnGen) | rs946197 | 1:56602308 | C/G | 0.232 | 0.0150 (0.0015) | 1.6E-24 | -0.0062 (0.0226) | 0.786 | 104.5 | -0.413 |
| Whole body fat-free mass | All-cause dementia (FinnGen) | rs11613704 | 12:980925 | A/C | 0.200 | 0.0158 (0.0015) | 2.1E-24 | 0.0181 (0.0239) | 0.448 | 103.9 | 1.145 |
| Whole body fat-free mass | All-cause dementia (FinnGen) | rs12193797 | 6:41748352 | G/A | 0.133 | -0.0185 (0.0018) | 2.9E-24 | -0.0065 (0.0357) | 0.856 | 103.3 | 0.351 |
| Whole body fat-free mass | All-cause dementia (FinnGen) | rs2252720 | 20:21223663 | T/C | 0.674 | -0.0135 (0.0013) | 4.6E-24 | 0.0157 (0.0213) | 0.459 | 102.4 | -1.160 |
| Whole body fat-free mass | All-cause dementia (FinnGen) | rs10119967 | 9:99268421 | C/A | 0.204 | 0.0156 (0.0015) | 5.9E-24 | -0.0540 (0.0252) | 0.032 | 101.9 | -3.464 |
| Whole body fat-free mass | All-cause dementia (FinnGen) | rs2163832 | 19:10745764 | C/T | 0.660 | -0.0133 (0.0013) | 6.4E-24 | 0.0360 (0.0212) | 0.090 | 101.7 | -2.713 |
| Whole body fat-free mass | All-cause dementia (FinnGen) | rs676105 | 11:30443688 | C/T | 0.304 | 0.0136 (0.0014) | 7.8E-24 | -0.0057 (0.0209) | 0.784 | 101.3 | -0.418 |
| Whole body fat-free mass | All-cause dementia (FinnGen) | rs9480933 | 6:109671391 | G/C | 0.350 | -0.0130 (0.0013) | 9.3E-24 | 0.0339 (0.0197) | 0.086 | 101.0 | -2.598 |
| Whole body fat-free mass | All-cause dementia (FinnGen) | rs11648796 | 16:792190 | G/A | 0.232 | 0.0154 (0.0015) | 1.4E-23 | 0.0098 (0.0206) | 0.634 | 100.1 | 0.634 |
| Whole body fat-free mass | All-cause dementia (FinnGen) | rs2265309 | 10:104487871 | C/T | 0.517 | -0.0124 (0.0012) | 1.5E-23 | 0.0006 (0.0192) | 0.976 | 100.0 | -0.048 |
| Whole body fat-free mass | All-cause dementia (FinnGen) | rs13271368 | 8:126506140 | T/C | 0.237 | -0.0148 (0.0015) | 2.0E-23 | 0.0196 (0.0209) | 0.350 | 99.4 | -1.328 |
| Whole body fat-free mass | All-cause dementia (FinnGen) | rs7815955 | 8:130719567 | T/A | 0.203 | -0.0152 (0.0015) | 7.8E-23 | 0.0248 (0.0251) | 0.323 | 96.8 | -1.633 |
| Whole body fat-free mass | All-cause dementia (FinnGen) | rs6779752 | 3:85663849 | A/G | 0.638 | -0.0128 (0.0013) | 8.5E-23 | -0.0150 (0.0213) | 0.480 | 96.6 | 1.175 |
| Whole body fat-free mass | All-cause dementia (FinnGen) | rs111365325 | 5:170865229 | T/C | 0.231 | -0.0145 (0.0015) | 9.6E-23 | 0.0238 (0.0238) | 0.319 | 96.3 | -1.646 |
| Whole body fat-free mass | All-cause dementia (FinnGen) | rs33973388 | 18:46611842 | T/G | 0.435 | 0.0123 (0.0013) | 9.7E-23 | 0.0101 (0.0208) | 0.626 | 96.3 | 0.820 |
| Whole body fat-free mass | All-cause dementia (FinnGen) | rs11578046 | 1:23425139 | A/G | 0.327 | -0.0129 (0.0013) | 1.1E-22 | -0.0107 (0.0204) | 0.598 | 96.1 | 0.827 |
| Whole body fat-free mass | All-cause dementia (FinnGen) | rs67551338 | 12:3393100 | T/C | 0.061 | 0.0255 (0.0026) | 1.4E-22 | -0.0383 (0.0493) | 0.438 | 95.6 | -1.500 |
| Whole body fat-free mass | All-cause dementia (FinnGen) | rs3809570 | 15:67000117 | A/C | 0.243 | 0.0142 (0.0015) | 2.1E-22 | -0.0141 (0.0230) | 0.539 | 94.8 | -0.996 |
| Whole body fat-free mass | All-cause dementia (FinnGen) | rs16942324 | 15:89383854 | A/C | 0.027 | -0.0373 (0.0038) | 2.2E-22 | 0.1100 (0.0899) | 0.221 | 94.7 | -2.951 |
| Whole body fat-free mass | All-cause dementia (FinnGen) | rs34848742 | 4:123828042 | G/T | 0.788 | -0.0148 (0.0015) | 2.3E-22 | 0.0177 (0.0248) | 0.476 | 94.7 | -1.200 |
| Whole body fat-free mass | All-cause dementia (FinnGen) | rs13240065 | 7:73015369 | A/G | 0.128 | 0.0180 (0.0019) | 2.9E-22 | 0.0135 (0.0285) | 0.636 | 94.2 | 0.751 |
| Whole body fat-free mass | All-cause dementia (FinnGen) | rs73052033 | 3:185828465 | C/T | 0.185 | -0.0154 (0.0016) | 5.2E-22 | 0.0319 (0.0260) | 0.219 | 93.0 | -2.069 |
| Whole body fat-free mass | All-cause dementia (FinnGen) | rs7780752 | 7:93241640 | C/T | 0.360 | 0.0124 (0.0013) | 5.4E-22 | -0.0155 (0.0217) | 0.476 | 92.9 | -1.246 |
| Whole body fat-free mass | All-cause dementia (FinnGen) | rs9291926 | 5:67599656 | G/T | 0.532 | -0.0120 (0.0012) | 6.1E-22 | 0.0104 (0.0192) | 0.588 | 92.7 | -0.869 |
| Whole body fat-free mass | All-cause dementia (FinnGen) | rs2721938 | 8:116635611 | T/C | 0.600 | -0.0121 (0.0013) | 1.3E-21 | 0.0135 (0.0211) | 0.521 | 91.2 | -1.113 |
| Whole body fat-free mass | All-cause dementia (FinnGen) | rs13430869 | 2:218146818 | T/G | 0.742 | 0.0135 (0.0014) | 1.4E-21 | -0.0128 (0.0200) | 0.522 | 91.1 | -0.948 |
| Whole body fat-free mass | All-cause dementia (FinnGen) | rs62621197 | 19:8670147 | T/C | 0.037 | -0.0326 (0.0034) | 1.4E-21 | 0.1577 (0.0688) | 0.022 | 91.0 | -4.840 |
| Whole body fat-free mass | All-cause dementia (FinnGen) | rs10938397 | 4:45182527 | G/A | 0.434 | 0.0119 (0.0013) | 1.7E-21 | -0.0117 (0.0192) | 0.542 | 90.6 | -0.981 |
| Whole body fat-free mass | All-cause dementia (FinnGen) | rs10775348 | 16:88806348 | G/A | 0.704 | 0.0130 (0.0014) | 2.0E-21 | -0.0109 (0.0209) | 0.601 | 90.4 | -0.840 |
| Whole body fat-free mass | All-cause dementia (FinnGen) | rs11042725 | 11:10325325 | A/C | 0.488 | -0.0117 (0.0012) | 3.1E-21 | -0.0196 (0.0191) | 0.306 | 89.5 | 1.670 |
| Whole body fat-free mass | All-cause dementia (FinnGen) | rs10748128 | 12:69827658 | T/G | 0.345 | 0.0123 (0.0013) | 3.2E-21 | 0.0287 (0.0200) | 0.152 | 89.5 | 2.324 |
| Whole body fat-free mass | All-cause dementia (FinnGen) | rs7619139 | 3:25110415 | A/T | 0.589 | 0.0119 (0.0013) | 3.4E-21 | -0.0284 (0.0194) | 0.145 | 89.3 | -2.380 |
| Whole body fat-free mass | All-cause dementia (FinnGen) | rs247008 | 5:131447104 | G/A | 0.670 | 0.0125 (0.0013) | 3.6E-21 | -0.0402 (0.0196) | 0.041 | 89.2 | -3.208 |
| Whole body fat-free mass | All-cause dementia (FinnGen) | rs62621812 | 7:127015083 | A/G | 0.020 | 0.0425 (0.0045) | 5.9E-21 | -0.0268 (0.0478) | 0.574 | 88.2 | -0.630 |
| Whole body fat-free mass | All-cause dementia (FinnGen) | rs4968799 | 17:68158831 | T/A | 0.112 | -0.0185 (0.0020) | 7.3E-21 | -0.0052 (0.0325) | 0.874 | 87.8 | 0.282 |
| Whole body fat-free mass | All-cause dementia (FinnGen) | rs2726036 | 16:28347140 | C/A | 0.398 | 0.0119 (0.0013) | 8.0E-21 | -0.0207 (0.0193) | 0.283 | 87.6 | -1.739 |
| Whole body fat-free mass | All-cause dementia (FinnGen) | rs2952615 | 5:112138888 | C/G | 0.618 | -0.0119 (0.0013) | 8.9E-21 | 0.0337 (0.0195) | 0.083 | 87.4 | -2.824 |
| Whole body fat-free mass | All-cause dementia (FinnGen) | rs17246129 | 2:227259964 | A/G | 0.305 | 0.0125 (0.0013) | 1.4E-20 | -0.0261 (0.0215) | 0.224 | 86.5 | -2.085 |
| Whole body fat-free mass | All-cause dementia (FinnGen) | rs9461246 | 6:26381660 | A/G | 0.118 | -0.0178 (0.0019) | 1.5E-20 | -0.0175 (0.0270) | 0.516 | 86.3 | 0.984 |
| Whole body fat-free mass | All-cause dementia (FinnGen) | rs1064213 | 2:198950240 | A/G | 0.478 | 0.0115 (0.0012) | 1.8E-20 | 0.0172 (0.0196) | 0.380 | 86.0 | 1.498 |
| Whole body fat-free mass | All-cause dementia (FinnGen) | rs2015561 | 17:38593294 | G/A | 0.424 | -0.0117 (0.0013) | 1.9E-20 | 0.0078 (0.0193) | 0.686 | 85.9 | -0.669 |
| Whole body fat-free mass | All-cause dementia (FinnGen) | rs1542224 | 2:223963874 | C/T | 0.719 | 0.0128 (0.0014) | 2.0E-20 | 0.0175 (0.0218) | 0.420 | 85.8 | 1.371 |
| Whole body fat-free mass | All-cause dementia (FinnGen) | rs2678204 | 1:201800511 | G/T | 0.340 | 0.0121 (0.0013) | 2.4E-20 | -0.0015 (0.0210) | 0.942 | 85.4 | -0.124 |
| Whole body fat-free mass | All-cause dementia (FinnGen) | rs3212260 | 14:24804088 | T/A | 0.258 | 0.0131 (0.0014) | 3.6E-20 | 0.0039 (0.0230) | 0.865 | 84.6 | 0.298 |
| Whole body fat-free mass | All-cause dementia (FinnGen) | rs663344 | 9:98316331 | C/G | 0.181 | -0.0151 (0.0016) | 3.7E-20 | -0.0013 (0.0244) | 0.956 | 84.6 | 0.086 |
| Whole body fat-free mass | All-cause dementia (FinnGen) | rs6585827 | 10:124165615 | A/G | 0.471 | 0.0114 (0.0012) | 3.8E-20 | -0.0649 (0.0198) | 0.001 | 84.5 | -5.678 |
| Whole body fat-free mass | All-cause dementia (FinnGen) | rs1910252 | 8:49407362 | T/C | 0.168 | 0.0153 (0.0017) | 3.9E-20 | -0.0009 (0.0277) | 0.976 | 84.5 | -0.059 |
| Whole body fat-free mass | All-cause dementia (FinnGen) | rs703593 | 12:103064247 | G/A | 0.482 | -0.0114 (0.0012) | 4.1E-20 | 0.0393 (0.0193) | 0.042 | 84.4 | -3.448 |
| Whole body fat-free mass | All-cause dementia (FinnGen) | rs55831773 | 17:7559037 | T/C | 0.199 | -0.0146 (0.0016) | 4.1E-20 | -0.0496 (0.0274) | 0.070 | 84.4 | 3.403 |
| Whole body fat-free mass | All-cause dementia (FinnGen) | rs115179432 | 2:33348679 | G/A | 0.072 | -0.0221 (0.0024) | 4.5E-20 | -0.0383 (0.0409) | 0.349 | 84.2 | 1.734 |
| Whole body fat-free mass | All-cause dementia (FinnGen) | rs55727637 | 16:15145252 | T/C | 0.305 | -0.0123 (0.0013) | 6.1E-20 | 0.0031 (0.0208) | 0.882 | 83.6 | -0.251 |
| Whole body fat-free mass | All-cause dementia (FinnGen) | rs31211 | 5:134363145 | A/G | 0.251 | -0.0130 (0.0014) | 7.2E-20 | -0.0325 (0.0218) | 0.137 | 83.2 | 2.491 |
| Whole body fat-free mass | All-cause dementia (FinnGen) | rs2140046 | 2:169706079 | C/T | 0.364 | -0.0117 (0.0013) | 8.2E-20 | -0.0037 (0.0194) | 0.850 | 83.0 | 0.316 |
| Whole body fat-free mass | All-cause dementia (FinnGen) | rs11778491 | 8:120451362 | C/G | 0.252 | -0.0130 (0.0014) | 9.2E-20 | 0.0325 (0.0245) | 0.185 | 82.8 | -2.498 |
| Whole body fat-free mass | All-cause dementia (FinnGen) | rs3740591 | 10:70287303 | T/C | 0.445 | 0.0114 (0.0013) | 1.7E-19 | -0.0180 (0.0203) | 0.376 | 81.5 | -1.579 |
| Whole body fat-free mass | All-cause dementia (FinnGen) | rs4980067 | 10:81136129 | A/C | 0.505 | -0.0112 (0.0012) | 1.8E-19 | -0.0040 (0.0193) | 0.837 | 81.4 | 0.357 |
| Whole body fat-free mass | All-cause dementia (FinnGen) | rs1841738 | 4:88557753 | G/A | 0.518 | -0.0112 (0.0012) | 3.2E-19 | 0.0286 (0.0197) | 0.146 | 80.3 | -2.557 |
| Whole body fat-free mass | All-cause dementia (FinnGen) | rs10202845 | 2:42575820 | G/A | 0.113 | -0.0176 (0.0020) | 4.3E-19 | -0.0191 (0.0314) | 0.543 | 79.7 | 1.084 |
| Whole body fat-free mass | All-cause dementia (FinnGen) | rs11001399 | 10:77221744 | C/A | 0.559 | -0.0112 (0.0013) | 5.2E-19 | -0.0124 (0.0193) | 0.520 | 79.4 | 1.109 |
| Whole body fat-free mass | All-cause dementia (FinnGen) | rs843374 | 3:183997261 | T/A | 0.587 | -0.0112 (0.0013) | 5.4E-19 | 0.0070 (0.0195) | 0.721 | 79.3 | -0.624 |
| Whole body fat-free mass | All-cause dementia (FinnGen) | rs7706886 | 5:32722319 | A/G | 0.271 | 0.0126 (0.0014) | 6.2E-19 | 0.0134 (0.0226) | 0.552 | 79.0 | 1.062 |
| Whole body fat-free mass | All-cause dementia (FinnGen) | rs10803955 | 2:183228114 | G/A | 0.508 | -0.0110 (0.0012) | 6.4E-19 | -0.0079 (0.0192) | 0.680 | 79.0 | 0.720 |
| Whole body fat-free mass | All-cause dementia (FinnGen) | rs111391498 | 4:1341553 | G/A | 0.047 | -0.0258 (0.0029) | 6.7E-19 | 0.0010 (0.0564) | 0.986 | 78.8 | -0.039 |
| Whole body fat-free mass | All-cause dementia (FinnGen) | rs4865956 | 5:54882505 | A/T | 0.697 | -0.0120 (0.0014) | 7.3E-19 | 0.0151 (0.0213) | 0.477 | 78.7 | -1.258 |
| Whole body fat-free mass | All-cause dementia (FinnGen) | rs28547519 | 7:121964618 | C/T | 0.366 | 0.0114 (0.0013) | 8.5E-19 | 0.0259 (0.0196) | 0.187 | 78.4 | 2.273 |
| Whole body fat-free mass | All-cause dementia (FinnGen) | rs7647657 | 3:38573746 | G/A | 0.386 | -0.0113 (0.0013) | 9.1E-19 | 0.0222 (0.0194) | 0.255 | 78.2 | -1.966 |
| Whole body fat-free mass | All-cause dementia (FinnGen) | rs2602713 | 19:4910021 | C/A | 0.440 | 0.0111 (0.0013) | 1.2E-18 | -0.0142 (0.0199) | 0.475 | 77.6 | -1.274 |
| Whole body fat-free mass | All-cause dementia (FinnGen) | rs6874142 | 5:172753555 | G/T | 0.114 | 0.0182 (0.0021) | 1.4E-18 | -0.0659 (0.0332) | 0.047 | 77.5 | -3.622 |
| Whole body fat-free mass | All-cause dementia (FinnGen) | rs76364830 | 8:13372120 | A/G | 0.063 | -0.0225 (0.0026) | 2.4E-18 | 0.0004 (0.0524) | 0.995 | 76.4 | -0.018 |
| Whole body fat-free mass | All-cause dementia (FinnGen) | rs17556750 | 4:82155568 | A/C | 0.288 | 0.0119 (0.0014) | 3.7E-18 | 0.0074 (0.0199) | 0.711 | 75.5 | 0.622 |
| Whole body fat-free mass | All-cause dementia (FinnGen) | rs11712872 | 3:52835514 | A/G | 0.115 | 0.0168 (0.0019) | 4.3E-18 | -0.0095 (0.0335) | 0.777 | 75.2 | -0.565 |
| Whole body fat-free mass | All-cause dementia (FinnGen) | rs7728690 | 5:88411214 | T/C | 0.386 | -0.0110 (0.0013) | 5.0E-18 | -0.0170 (0.0205) | 0.407 | 74.9 | 1.543 |
| Whole body fat-free mass | All-cause dementia (FinnGen) | rs76513770 | 16:72505534 | C/T | 0.128 | -0.0161 (0.0019) | 5.1E-18 | 0.0094 (0.0257) | 0.713 | 74.8 | -0.584 |
| Whole body fat-free mass | All-cause dementia (FinnGen) | rs2281175 | 1:1665702 | C/T | 0.405 | 0.0114 (0.0013) | 6.3E-18 | 0.0073 (0.0195) | 0.708 | 74.4 | 0.638 |
| Whole body fat-free mass | All-cause dementia (FinnGen) | rs3116201 | 2:233074205 | A/G | 0.098 | -0.0180 (0.0021) | 7.1E-18 | -0.0143 (0.0444) | 0.747 | 74.2 | 0.796 |
| Whole body fat-free mass | All-cause dementia (FinnGen) | rs508347 | 7:28212824 | C/T | 0.703 | -0.0117 (0.0014) | 7.8E-18 | -0.0214 (0.0200) | 0.285 | 74.0 | 1.831 |
| Whole body fat-free mass | All-cause dementia (FinnGen) | rs10777860 | 12:97792792 | A/G | 0.528 | -0.0107 (0.0012) | 7.8E-18 | 0.0013 (0.0193) | 0.948 | 74.0 | -0.122 |
| Whole body fat-free mass | All-cause dementia (FinnGen) | rs744205 | 11:69929677 | A/G | 0.544 | 0.0107 (0.0012) | 1.0E-17 | 0.0052 (0.0192) | 0.788 | 73.5 | 0.485 |
| Whole body fat-free mass | All-cause dementia (FinnGen) | rs7134283 | 12:24071748 | A/G | 0.283 | -0.0118 (0.0014) | 1.2E-17 | -0.0024 (0.0214) | 0.912 | 73.2 | 0.203 |
| Whole body fat-free mass | All-cause dementia (FinnGen) | rs822549 | 7:148649180 | C/T | 0.267 | 0.0120 (0.0014) | 1.4E-17 | -0.0261 (0.0211) | 0.217 | 72.9 | -2.179 |
| Whole body fat-free mass | All-cause dementia (FinnGen) | rs61729527 | 8:77761919 | T/C | 0.052 | -0.0238 (0.0028) | 1.9E-17 | -0.0123 (0.0387) | 0.751 | 72.3 | 0.516 |
| Whole body fat-free mass | All-cause dementia (FinnGen) | rs968821 | 9:94187634 | C/G | 0.340 | -0.0111 (0.0013) | 2.1E-17 | 0.0151 (0.0217) | 0.485 | 72.1 | -1.357 |
| Whole body fat-free mass | All-cause dementia (FinnGen) | rs9317002 | 13:59175727 | A/C | 0.515 | 0.0106 (0.0013) | 2.3E-17 | -0.0151 (0.0192) | 0.430 | 71.8 | -1.421 |
| Whole body fat-free mass | All-cause dementia (FinnGen) | rs9951619 | 18:56882326 | G/T | 0.767 | 0.0125 (0.0015) | 4.0E-17 | 0.0054 (0.0205) | 0.791 | 70.8 | 0.433 |
| Whole body fat-free mass | All-cause dementia (FinnGen) | rs3782232 | 12:57116249 | A/G | 0.071 | -0.0203 (0.0024) | 4.4E-17 | 0.0674 (0.0350) | 0.054 | 70.6 | -3.322 |
| Whole body fat-free mass | All-cause dementia (FinnGen) | rs12188627 | 5:60720682 | G/A | 0.488 | -0.0104 (0.0012) | 6.3E-17 | -0.0053 (0.0192) | 0.781 | 69.9 | 0.511 |
| Whole body fat-free mass | All-cause dementia (FinnGen) | rs11689727 | 2:25458100 | A/C | 0.331 | -0.0110 (0.0013) | 7.0E-17 | 0.0014 (0.0205) | 0.945 | 69.7 | -0.127 |
| Whole body fat-free mass | All-cause dementia (FinnGen) | rs7312646 | 12:102388920 | C/A | 0.521 | -0.0105 (0.0013) | 9.2E-17 | 0.0001 (0.0192) | 0.996 | 69.1 | -0.010 |
| Whole body fat-free mass | All-cause dementia (FinnGen) | rs6902789 | 6:105358192 | A/G | 0.368 | 0.0107 (0.0013) | 1.1E-16 | 0.0076 (0.0199) | 0.704 | 68.8 | 0.711 |
| Whole body fat-free mass | All-cause dementia (FinnGen) | rs4858940 | 3:88254820 | C/T | 0.886 | 0.0161 (0.0019) | 1.2E-16 | 0.0142 (0.0318) | 0.656 | 68.6 | 0.881 |
| Whole body fat-free mass | All-cause dementia (FinnGen) | rs2296316 | 14:65520246 | C/T | 0.465 | -0.0104 (0.0013) | 1.2E-16 | -0.0114 (0.0191) | 0.552 | 68.6 | 1.096 |
| Whole body fat-free mass | All-cause dementia (FinnGen) | rs112560164 | 14:93112924 | A/G | 0.191 | 0.0131 (0.0016) | 1.3E-16 | 0.0252 (0.0276) | 0.361 | 68.5 | 1.919 |
| Whole body fat-free mass | All-cause dementia (FinnGen) | rs9380508 | 6:35398689 | T/C | 0.775 | 0.0123 (0.0015) | 1.4E-16 | 0.0108 (0.0295) | 0.716 | 68.3 | 0.880 |
| Whole body fat-free mass | All-cause dementia (FinnGen) | rs61992671 | 14:101531854 | G/A | 0.492 | -0.0107 (0.0013) | 1.4E-16 | -0.0101 (0.0197) | 0.609 | 68.2 | 0.940 |
| Whole body fat-free mass | All-cause dementia (FinnGen) | rs1228024 | 11:47951353 | A/C | 0.661 | -0.0108 (0.0013) | 2.1E-16 | -0.0156 (0.0207) | 0.451 | 67.5 | 1.446 |
| Whole body fat-free mass | All-cause dementia (FinnGen) | rs310796 | 12:77453226 | T/G | 0.681 | 0.0110 (0.0013) | 2.1E-16 | 0.0016 (0.0217) | 0.942 | 67.5 | 0.146 |
| Whole body fat-free mass | All-cause dementia (FinnGen) | rs11794152 | 9:23345347 | G/A | 0.415 | 0.0104 (0.0013) | 2.4E-16 | 0.0286 (0.0193) | 0.139 | 67.3 | 2.761 |
| Whole body fat-free mass | All-cause dementia (FinnGen) | rs1412234 | 9:28410683 | C/T | 0.327 | 0.0109 (0.0013) | 2.6E-16 | 0.0259 (0.0198) | 0.190 | 67.0 | 2.384 |
| Whole body fat-free mass | All-cause dementia (FinnGen) | rs55674305 | 8:89435868 | A/G | 0.306 | -0.0110 (0.0014) | 3.1E-16 | -0.0124 (0.0208) | 0.550 | 66.8 | 1.123 |
| Whole body fat-free mass | All-cause dementia (FinnGen) | rs578366 | 6:81591034 | G/A | 0.420 | -0.0102 (0.0013) | 3.5E-16 | -0.0093 (0.0192) | 0.627 | 66.5 | 0.910 |
| Whole body fat-free mass | All-cause dementia (FinnGen) | rs2197563 | 2:233687080 | A/G | 0.595 | 0.0103 (0.0013) | 3.8E-16 | -0.0287 (0.0216) | 0.184 | 66.3 | -2.793 |
| Whole body fat-free mass | All-cause dementia (FinnGen) | rs12820906 | 12:123493123 | G/A | 0.247 | 0.0117 (0.0014) | 3.9E-16 | -0.0174 (0.0237) | 0.464 | 66.3 | -1.483 |
| Whole body fat-free mass | All-cause dementia (FinnGen) | rs10899736 | 7:50546925 | A/G | 0.440 | -0.0102 (0.0013) | 4.4E-16 | -0.0116 (0.0196) | 0.553 | 66.1 | 1.141 |
| Whole body fat-free mass | All-cause dementia (FinnGen) | rs1049193 | 12:53873844 | C/G | 0.171 | -0.0134 (0.0016) | 4.6E-16 | -0.0603 (0.0305) | 0.048 | 66.0 | 4.501 |
| Whole body fat-free mass | All-cause dementia (FinnGen) | rs141729694 | 5:87999371 | T/C | 0.074 | 0.0193 (0.0024) | 4.7E-16 | -0.0006 (0.0485) | 0.989 | 65.9 | -0.031 |
| Whole body fat-free mass | All-cause dementia (FinnGen) | rs11855017 | 15:42096146 | A/C | 0.182 | 0.0131 (0.0016) | 4.8E-16 | 0.0384 (0.0286) | 0.179 | 65.9 | 2.929 |
| Whole body fat-free mass | All-cause dementia (FinnGen) | rs667515 | 11:69449076 | C/G | 0.386 | -0.0104 (0.0013) | 5.9E-16 | -0.0277 (0.0202) | 0.171 | 65.5 | 2.674 |
| Whole body fat-free mass | All-cause dementia (FinnGen) | rs357486 | 3:153885503 | C/T | 0.543 | 0.0100 (0.0012) | 6.7E-16 | 0.0288 (0.0193) | 0.137 | 65.2 | 2.869 |
| Whole body fat-free mass | All-cause dementia (FinnGen) | rs11245450 | 10:126658075 | A/G | 0.422 | -0.0103 (0.0013) | 7.0E-16 | 0.0013 (0.0193) | 0.948 | 65.2 | -0.127 |
| Whole body fat-free mass | All-cause dementia (FinnGen) | rs6984820 | 8:144993324 | T/C | 0.422 | -0.0101 (0.0013) | 8.5E-16 | 0.0526 (0.0195) | 0.007 | 64.7 | -5.186 |
| Whole body fat-free mass | All-cause dementia (FinnGen) | rs9513510 | 13:99571922 | C/G | 0.699 | -0.0109 (0.0014) | 1.0E-15 | -0.0119 (0.0204) | 0.561 | 64.4 | 1.093 |
| Whole body fat-free mass | All-cause dementia (FinnGen) | rs117543413 | 10:79543740 | T/C | 0.018 | -0.0382 (0.0048) | 1.2E-15 | 0.0298 (0.0792) | 0.706 | 64.0 | -0.779 |
| Whole body fat-free mass | All-cause dementia (FinnGen) | rs10172196 | 2:36780549 | A/G | 0.306 | 0.0108 (0.0013) | 1.3E-15 | -0.0585 (0.0203) | 0.004 | 63.9 | -5.436 |
| Whole body fat-free mass | All-cause dementia (FinnGen) | rs603321 | 6:140256669 | G/A | 0.245 | -0.0115 (0.0014) | 1.6E-15 | -0.0204 (0.0250) | 0.414 | 63.5 | 1.768 |
| Whole body fat-free mass | All-cause dementia (FinnGen) | rs12764498 | 10:5042294 | C/T | 0.118 | -0.0155 (0.0019) | 1.6E-15 | -0.0309 (0.0286) | 0.279 | 63.6 | 1.998 |
| Whole body fat-free mass | All-cause dementia (FinnGen) | rs7229520 | 18:46516468 | A/G | 0.662 | -0.0105 (0.0013) | 2.0E-15 | -0.0087 (0.0201) | 0.664 | 63.0 | 0.832 |
| Whole body fat-free mass | All-cause dementia (FinnGen) | rs1780673 | 20:35758359 | A/G | 0.811 | 0.0126 (0.0016) | 2.2E-15 | 0.0230 (0.0209) | 0.270 | 62.9 | 1.824 |
| Whole body fat-free mass | All-cause dementia (FinnGen) | rs10434434 | 4:54271611 | C/A | 0.148 | -0.0139 (0.0018) | 2.4E-15 | -0.0023 (0.0300) | 0.939 | 62.7 | 0.166 |
| Whole body fat-free mass | All-cause dementia (FinnGen) | rs1971955 | 12:122420437 | G/A | 0.854 | -0.0139 (0.0018) | 2.9E-15 | -0.0306 (0.0246) | 0.215 | 62.3 | 2.202 |
| Whole body fat-free mass | All-cause dementia (FinnGen) | rs5752989 | 22:30365780 | A/G | 0.571 | -0.0099 (0.0013) | 2.9E-15 | 0.0089 (0.0191) | 0.641 | 62.3 | -0.895 |
| Whole body fat-free mass | All-cause dementia (FinnGen) | rs34693680 | 3:98665549 | T/C | 0.132 | 0.0144 (0.0018) | 3.3E-15 | 0.0018 (0.0292) | 0.952 | 62.1 | 0.125 |
| Whole body fat-free mass | All-cause dementia (FinnGen) | rs12140153 | 1:62579891 | T/G | 0.094 | -0.0171 (0.0022) | 3.8E-15 | -0.0426 (0.0357) | 0.233 | 61.8 | 2.498 |
| Whole body fat-free mass | All-cause dementia (FinnGen) | rs35251247 | 11:43878459 | A/G | 0.292 | 0.0108 (0.0014) | 3.8E-15 | 0.0083 (0.0211) | 0.695 | 61.8 | 0.768 |
| Whole body fat-free mass | All-cause dementia (FinnGen) | rs6081869 | 20:20060745 | G/T | 0.386 | -0.0102 (0.0013) | 4.4E-15 | 0.0048 (0.0193) | 0.803 | 61.5 | -0.473 |
| Whole body fat-free mass | All-cause dementia (FinnGen) | rs74494415 | 18:74972138 | T/C | 0.040 | -0.0252 (0.0032) | 4.6E-15 | 0.0516 (0.0364) | 0.156 | 61.4 | -2.051 |
| Whole body fat-free mass | All-cause dementia (FinnGen) | rs11049684 | 12:28665359 | T/C | 0.299 | -0.0106 (0.0014) | 4.8E-15 | 0.0484 (0.0218) | 0.027 | 61.4 | -4.561 |
| Whole body fat-free mass | All-cause dementia (FinnGen) | rs10979612 | 9:111687379 | C/T | 0.073 | 0.0187 (0.0024) | 4.9E-15 | -0.0026 (0.0300) | 0.931 | 61.3 | -0.139 |
| Whole body fat-free mass | All-cause dementia (FinnGen) | rs147110934 | 19:55993436 | T/G | 0.024 | -0.0316 (0.0040) | 5.6E-15 | -0.0892 (0.1267) | 0.481 | 61.0 | 2.826 |
| Whole body fat-free mass | All-cause dementia (FinnGen) | rs798759 | 4:1730299 | G/A | 0.464 | -0.0097 (0.0012) | 6.7E-15 | -0.0076 (0.0200) | 0.703 | 60.7 | 0.787 |
| Whole body fat-free mass | All-cause dementia (FinnGen) | rs10780906 | 9:90841143 | A/G | 0.588 | -0.0099 (0.0013) | 8.4E-15 | -0.0194 (0.0192) | 0.314 | 60.2 | 1.964 |
| Whole body fat-free mass | All-cause dementia (FinnGen) | rs2287163 | 12:107349294 | T/C | 0.374 | -0.0099 (0.0013) | 8.4E-15 | 0.0189 (0.0202) | 0.348 | 60.2 | -1.903 |
| Whole body fat-free mass | All-cause dementia (FinnGen) | rs7301341 | 12:94083105 | C/T | 0.327 | -0.0103 (0.0013) | 9.3E-15 | -0.0340 (0.0201) | 0.091 | 60.0 | 3.315 |
| Whole body fat-free mass | All-cause dementia (FinnGen) | rs2104449 | 1:97082532 | T/G | 0.283 | 0.0107 (0.0014) | 9.4E-15 | 0.0125 (0.0249) | 0.615 | 60.0 | 1.172 |
| Whole body fat-free mass | All-cause dementia (FinnGen) | rs4974223 | 3:56602088 | T/C | 0.904 | 0.0163 (0.0021) | 1.0E-14 | -0.0021 (0.0299) | 0.943 | 59.9 | -0.129 |
| Whole body fat-free mass | All-cause dementia (FinnGen) | rs6142059 | 20:32544327 | C/T | 0.493 | 0.0096 (0.0012) | 1.1E-14 | -0.0245 (0.0191) | 0.201 | 59.8 | -2.542 |
| Whole body fat-free mass | All-cause dementia (FinnGen) | rs2615075 | 1:225934295 | G/A | 0.376 | 0.0098 (0.0013) | 2.0E-14 | 0.0056 (0.0197) | 0.775 | 58.5 | 0.571 |
| Whole body fat-free mass | All-cause dementia (FinnGen) | rs9379084 | 6:7231843 | A/G | 0.116 | -0.0152 (0.0020) | 2.7E-14 | -0.0347 (0.0307) | 0.259 | 57.9 | 2.285 |
| Whole body fat-free mass | All-cause dementia (FinnGen) | rs11785562 | 8:23391493 | A/G | 0.201 | 0.0119 (0.0016) | 2.7E-14 | -0.0324 (0.0220) | 0.140 | 58.0 | -2.716 |
| Whole body fat-free mass | All-cause dementia (FinnGen) | rs5742915 | 15:74336633 | C/T | 0.460 | 0.0095 (0.0012) | 3.2E-14 | -0.0331 (0.0195) | 0.089 | 57.6 | -3.493 |
| Whole body fat-free mass | All-cause dementia (FinnGen) | rs1374370 | 2:85818273 | A/G | 0.305 | 0.0102 (0.0013) | 3.5E-14 | -0.0416 (0.0226) | 0.066 | 57.5 | -4.083 |
| Whole body fat-free mass | All-cause dementia (FinnGen) | rs11611651 | 12:133380790 | A/G | 0.088 | 0.0166 (0.0022) | 4.3E-14 | 0.0215 (0.0453) | 0.636 | 57.0 | 1.299 |
| Whole body fat-free mass | All-cause dementia (FinnGen) | rs981002 | 4:12881731 | A/T | 0.267 | -0.0106 (0.0014) | 5.3E-14 | 0.0126 (0.0223) | 0.574 | 56.6 | -1.194 |
| Whole body fat-free mass | All-cause dementia (FinnGen) | rs2568958 | 1:72765116 | A/G | 0.604 | 0.0095 (0.0013) | 5.5E-14 | -0.0013 (0.0200) | 0.946 | 56.5 | -0.137 |
| Whole body fat-free mass | All-cause dementia (FinnGen) | rs12375196 | 7:103416541 | A/C | 0.424 | 0.0095 (0.0013) | 6.5E-14 | 0.0139 (0.0192) | 0.470 | 56.2 | 1.468 |
| Whole body fat-free mass | All-cause dementia (FinnGen) | rs6026578 | 20:57463472 | G/C | 0.625 | -0.0097 (0.0013) | 6.8E-14 | -0.0050 (0.0200) | 0.802 | 56.1 | 0.517 |
| Whole body fat-free mass | All-cause dementia (FinnGen) | rs58857770 | 19:7208110 | G/C | 0.408 | -0.0095 (0.0013) | 7.6E-14 | 0.0363 (0.0191) | 0.057 | 55.9 | -3.811 |
| Whole body fat-free mass | All-cause dementia (FinnGen) | rs9960619 | 18:33040095 | T/C | 0.344 | 0.0098 (0.0013) | 8.7E-14 | 0.0048 (0.0204) | 0.815 | 55.6 | 0.491 |
| Whole body fat-free mass | All-cause dementia (FinnGen) | rs13272451 | 8:8886183 | T/G | 0.439 | 0.0093 (0.0013) | 8.8E-14 | 0.0064 (0.0324) | 0.844 | 55.6 | 0.685 |
| Whole body fat-free mass | All-cause dementia (FinnGen) | rs11217863 | 11:120293138 | A/G | 0.116 | -0.0145 (0.0019) | 9.0E-14 | 0.0146 (0.0289) | 0.614 | 55.6 | -1.009 |
| Whole body fat-free mass | All-cause dementia (FinnGen) | rs62621400 | 15:101718239 | G/C | 0.058 | -0.0199 (0.0027) | 9.8E-14 | -0.0196 (0.0402) | 0.627 | 55.4 | 0.985 |
| Whole body fat-free mass | All-cause dementia (FinnGen) | rs3925 | 8:38281658 | A/G | 0.245 | -0.0107 (0.0014) | 1.2E-13 | -0.0056 (0.0248) | 0.821 | 55.0 | 0.522 |
| Whole body fat-free mass | All-cause dementia (FinnGen) | rs12070699 | 1:190230408 | T/C | 0.563 | -0.0092 (0.0012) | 1.5E-13 | -0.0031 (0.0192) | 0.872 | 54.5 | 0.337 |
| Whole body fat-free mass | All-cause dementia (FinnGen) | rs35756741 | 12:12868701 | T/C | 0.092 | -0.0159 (0.0022) | 1.6E-13 | -0.0136 (0.0315) | 0.666 | 54.4 | 0.856 |
| Whole body fat-free mass | All-cause dementia (FinnGen) | rs35492502 | 1:217806224 | A/G | 0.296 | 0.0100 (0.0014) | 2.1E-13 | -0.0056 (0.0204) | 0.783 | 53.9 | -0.562 |
| Whole body fat-free mass | All-cause dementia (FinnGen) | rs284662 | 19:41932275 | C/T | 0.620 | -0.0094 (0.0013) | 2.2E-13 | 0.0073 (0.0200) | 0.715 | 53.8 | -0.777 |
| Whole body fat-free mass | All-cause dementia (FinnGen) | rs36695 | 5:127549187 | A/G | 0.504 | -0.0091 (0.0012) | 2.3E-13 | -0.0037 (0.0195) | 0.850 | 53.7 | 0.407 |
| Whole body fat-free mass | All-cause dementia (FinnGen) | rs4926542 | 1:50263773 | T/C | 0.682 | -0.0098 (0.0013) | 2.4E-13 | -0.0230 (0.0198) | 0.245 | 53.7 | 2.348 |
| Whole body fat-free mass | All-cause dementia (FinnGen) | rs1805165 | 2:88874891 | A/C | 0.721 | -0.0101 (0.0014) | 2.5E-13 | 0.0241 (0.0203) | 0.234 | 53.6 | -2.392 |
| Whole body fat-free mass | All-cause dementia (FinnGen) | rs12209223 | 6:76164589 | A/C | 0.101 | 0.0151 (0.0021) | 2.6E-13 | 0.0051 (0.0280) | 0.855 | 53.5 | 0.338 |
| Whole body fat-free mass | All-cause dementia (FinnGen) | rs9925273 | 16:375900 | G/A | 0.181 | -0.0118 (0.0016) | 2.7E-13 | 0.0118 (0.0260) | 0.650 | 53.4 | -0.999 |
| Whole body fat-free mass | All-cause dementia (FinnGen) | rs55758152 | 5:171317318 | A/G | 0.326 | 0.0098 (0.0013) | 2.8E-13 | -0.0137 (0.0203) | 0.501 | 53.3 | -1.402 |
| Whole body fat-free mass | All-cause dementia (FinnGen) | rs2578557 | 5:5473063 | T/C | 0.642 | -0.0095 (0.0013) | 2.9E-13 | -0.0081 (0.0193) | 0.675 | 53.3 | 0.857 |
| Whole body fat-free mass | All-cause dementia (FinnGen) | rs2970592 | 1:212220847 | A/G | 0.588 | 0.0092 (0.0013) | 3.0E-13 | -0.0285 (0.0198) | 0.151 | 53.2 | -3.114 |
| Whole body fat-free mass | All-cause dementia (FinnGen) | rs74048171 | 11:2093603 | A/C | 0.258 | -0.0104 (0.0014) | 3.2E-13 | 0.0136 (0.0230) | 0.552 | 53.1 | -1.313 |
| Whole body fat-free mass | All-cause dementia (FinnGen) | rs57126421 | 18:2656989 | G/A | 0.238 | -0.0107 (0.0015) | 3.8E-13 | 0.0404 (0.0228) | 0.077 | 52.8 | -3.780 |
| Whole body fat-free mass | All-cause dementia (FinnGen) | rs1521624 | 15:79412399 | A/C | 0.471 | -0.0091 (0.0013) | 4.0E-13 | -0.0256 (0.0191) | 0.181 | 52.7 | 2.816 |
| Whole body fat-free mass | All-cause dementia (FinnGen) | rs11065015 | 12:120520863 | T/C | 0.027 | -0.0282 (0.0039) | 4.1E-13 | 0.0167 (0.0432) | 0.698 | 52.6 | -0.593 |
| Whole body fat-free mass | All-cause dementia (FinnGen) | rs11545482 | 2:70315987 | T/C | 0.020 | -0.0316 (0.0044) | 4.4E-13 | -0.0646 (0.1349) | 0.632 | 52.5 | 2.043 |
| Whole body fat-free mass | All-cause dementia (FinnGen) | rs73601548 | 10:18549889 | T/C | 0.115 | 0.0142 (0.0020) | 4.8E-13 | -0.0574 (0.0388) | 0.139 | 52.3 | -4.054 |
| Whole body fat-free mass | All-cause dementia (FinnGen) | rs55800172 | 1:32414767 | A/G | 0.064 | 0.0182 (0.0025) | 5.7E-13 | 0.0280 (0.0502) | 0.578 | 52.0 | 1.537 |
| Whole body fat-free mass | All-cause dementia (FinnGen) | rs11071182 | 15:55644676 | G/A | 0.870 | 0.0134 (0.0019) | 6.3E-13 | -0.0053 (0.0305) | 0.862 | 51.7 | -0.396 |
| Whole body fat-free mass | All-cause dementia (FinnGen) | rs11160601 | 14:101186641 | T/C | 0.092 | 0.0156 (0.0022) | 6.5E-13 | -0.0111 (0.0265) | 0.674 | 51.7 | -0.713 |
| Whole body fat-free mass | All-cause dementia (FinnGen) | rs3783256 | 13:30107388 | C/T | 0.648 | -0.0094 (0.0013) | 6.7E-13 | -0.0144 (0.0193) | 0.454 | 51.6 | 1.532 |
| Whole body fat-free mass | All-cause dementia (FinnGen) | rs17828687 | 8:73454767 | A/C | 0.573 | -0.0091 (0.0013) | 6.8E-13 | -0.0065 (0.0195) | 0.741 | 51.6 | 0.718 |
| Whole body fat-free mass | All-cause dementia (FinnGen) | rs71393968 | 16:67847341 | A/G | 0.046 | 0.0214 (0.0030) | 6.9E-13 | -0.0241 (0.0474) | 0.611 | 51.6 | -1.128 |
| Whole body fat-free mass | All-cause dementia (FinnGen) | rs11709402 | 3:131551027 | G/A | 0.279 | 0.0100 (0.0014) | 7.1E-13 | 0.0129 (0.0217) | 0.551 | 51.5 | 1.296 |
| Whole body fat-free mass | All-cause dementia (FinnGen) | rs4795318 | 17:36949053 | T/C | 0.520 | 0.0089 (0.0012) | 7.1E-13 | 0.0130 (0.0193) | 0.500 | 51.5 | 1.455 |
| Whole body fat-free mass | All-cause dementia (FinnGen) | rs73013411 | 6:164126233 | A/C | 0.131 | 0.0131 (0.0018) | 8.9E-13 | 0.0093 (0.0353) | 0.791 | 51.1 | 0.708 |
| Whole body fat-free mass | All-cause dementia (FinnGen) | rs16916881 | 8:95566270 | A/C | 0.235 | -0.0104 (0.0015) | 9.2E-13 | -0.0028 (0.0198) | 0.888 | 51.0 | 0.268 |
| Whole body fat-free mass | All-cause dementia (FinnGen) | rs10870597 | 13:114999636 | G/A | 0.235 | -0.0105 (0.0015) | 1.0E-12 | -0.0095 (0.0225) | 0.674 | 50.8 | 0.909 |
| Whole body fat-free mass | All-cause dementia (FinnGen) | rs6719296 | 2:103101108 | A/G | 0.495 | 0.0089 (0.0012) | 1.1E-12 | -0.0118 (0.0198) | 0.549 | 50.6 | -1.333 |
| Whole body fat-free mass | All-cause dementia (FinnGen) | rs10945541 | 6:158740218 | G/A | 0.665 | -0.0093 (0.0013) | 1.1E-12 | 0.0072 (0.0197) | 0.715 | 50.7 | -0.770 |
| Whole body fat-free mass | All-cause dementia (FinnGen) | rs60804050 | 1:118870373 | A/G | 0.256 | -0.0100 (0.0014) | 1.5E-12 | 0.0111 (0.0224) | 0.619 | 50.1 | -1.108 |
| Whole body fat-free mass | All-cause dementia (FinnGen) | rs9540493 | 13:66205704 | G/A | 0.545 | -0.0089 (0.0013) | 1.5E-12 | -0.0126 (0.0193) | 0.513 | 50.1 | 1.417 |
| Whole body fat-free mass | All-cause dementia (FinnGen) | rs7245985 | 19:30710410 | G/T | 0.208 | -0.0109 (0.0015) | 1.5E-12 | 0.0147 (0.0243) | 0.546 | 50.1 | -1.351 |
| Whole body fat-free mass | All-cause dementia (FinnGen) | rs73619441 | 20:61564901 | G/T | 0.144 | -0.0126 (0.0018) | 1.5E-12 | 0.0209 (0.0303) | 0.490 | 50.1 | -1.664 |
| Whole body fat-free mass | All-cause dementia (FinnGen) | rs2789366 | 1:235508270 | A/G | 0.346 | -0.0092 (0.0013) | 1.6E-12 | -0.0199 (0.0198) | 0.315 | 50.0 | 2.166 |
| Whole body fat-free mass | All-cause dementia (FinnGen) | rs16844418 | 4:3471754 | C/T | 0.143 | -0.0125 (0.0018) | 1.9E-12 | -0.0446 (0.0291) | 0.125 | 49.6 | 3.572 |
| Whole body fat-free mass | All-cause dementia (FinnGen) | rs10222924 | 4:49016761 | G/A | 0.699 | -0.0095 (0.0014) | 2.0E-12 | 0.0098 (0.0219) | 0.654 | 49.5 | -1.027 |
| Whole body fat-free mass | All-cause dementia (FinnGen) | rs7192870 | 16:14397436 | C/T | 0.469 | -0.0089 (0.0013) | 2.0E-12 | -0.0043 (0.0196) | 0.826 | 49.4 | 0.486 |
| Whole body fat-free mass | All-cause dementia (FinnGen) | rs11052457 | 12:33268956 | T/A | 0.038 | 0.0228 (0.0032) | 2.1E-12 | 0.0109 (0.0506) | 0.829 | 49.4 | 0.478 |
| Whole body fat-free mass | All-cause dementia (FinnGen) | rs892511 | 1:110078255 | G/C | 0.028 | 0.0263 (0.0038) | 3.7E-12 | 0.0563 (0.0443) | 0.204 | 48.3 | 2.138 |
| Whole body fat-free mass | All-cause dementia (FinnGen) | rs17400325 | 2:178565913 | C/T | 0.042 | 0.0215 (0.0031) | 3.8E-12 | -0.0709 (0.0672) | 0.291 | 48.2 | -3.296 |
| Whole body fat-free mass | All-cause dementia (FinnGen) | rs9394166 | 6:33751122 | C/G | 0.380 | 0.0089 (0.0013) | 3.8E-12 | 0.0021 (0.0195) | 0.915 | 48.2 | 0.237 |
| Whole body fat-free mass | All-cause dementia (FinnGen) | rs338361 | 15:68171116 | C/T | 0.706 | 0.0095 (0.0014) | 3.9E-12 | 0.0328 (0.0227) | 0.148 | 48.1 | 3.455 |
| Whole body fat-free mass | All-cause dementia (FinnGen) | rs115461836 | 6:19078931 | C/T | 0.046 | 0.0208 (0.0030) | 4.2E-12 | 0.0371 (0.0578) | 0.521 | 48.0 | 1.780 |
| Whole body fat-free mass | All-cause dementia (FinnGen) | rs1296328 | 4:137083193 | C/A | 0.559 | -0.0087 (0.0013) | 4.5E-12 | -0.0206 (0.0192) | 0.284 | 47.9 | 2.373 |
| Whole body fat-free mass | All-cause dementia (FinnGen) | rs4132132 | 4:31001301 | C/T | 0.444 | 0.0087 (0.0013) | 4.7E-12 | -0.0213 (0.0196) | 0.276 | 47.8 | -2.445 |
| Whole body fat-free mass | All-cause dementia (FinnGen) | rs9596810 | 13:54056553 | T/C | 0.435 | -0.0089 (0.0013) | 4.7E-12 | 0.0017 (0.0196) | 0.930 | 47.8 | -0.190 |
| Whole body fat-free mass | All-cause dementia (FinnGen) | rs1927635 | 9:16461905 | C/T | 0.354 | 0.0091 (0.0013) | 5.0E-12 | 0.0233 (0.0192) | 0.225 | 47.7 | 2.568 |
| Whole body fat-free mass | All-cause dementia (FinnGen) | rs1218826 | 13:27995760 | G/A | 0.666 | 0.0091 (0.0013) | 5.0E-12 | 0.0121 (0.0204) | 0.554 | 47.7 | 1.324 |
| Whole body fat-free mass | All-cause dementia (FinnGen) | rs17363646 | 1:86823503 | G/A | 0.136 | 0.0124 (0.0018) | 5.5E-12 | -0.0169 (0.0220) | 0.442 | 47.5 | -1.360 |
| Whole body fat-free mass | All-cause dementia (FinnGen) | rs17443541 | 2:200402624 | C/T | 0.189 | -0.0110 (0.0016) | 5.8E-12 | -0.0163 (0.0256) | 0.524 | 47.4 | 1.484 |
| Whole body fat-free mass | All-cause dementia (FinnGen) | rs12889702 | 14:42894143 | C/A | 0.313 | 0.0092 (0.0013) | 6.4E-12 | -0.0168 (0.0210) | 0.424 | 47.2 | -1.819 |
| Whole body fat-free mass | All-cause dementia (FinnGen) | rs254963 | 5:171224403 | G/A | 0.447 | -0.0086 (0.0013) | 6.6E-12 | 0.0177 (0.0192) | 0.355 | 47.1 | -2.052 |
| Whole body fat-free mass | All-cause dementia (FinnGen) | rs1813212 | 11:89301382 | G/A | 0.446 | -0.0086 (0.0013) | 6.7E-12 | 0.0052 (0.0193) | 0.787 | 47.1 | -0.605 |
| Whole body fat-free mass | All-cause dementia (FinnGen) | rs7188009 | 16:81660642 | A/G | 0.404 | 0.0087 (0.0013) | 7.0E-12 | 0.0338 (0.0218) | 0.120 | 47.0 | 3.887 |
| Whole body fat-free mass | All-cause dementia (FinnGen) | rs774214 | 12:56918566 | C/T | 0.663 | -0.0090 (0.0013) | 7.2E-12 | -0.0076 (0.0200) | 0.705 | 47.0 | 0.845 |
| Whole body fat-free mass | All-cause dementia (FinnGen) | rs6972291 | 7:55802063 | C/T | 0.176 | -0.0112 (0.0016) | 7.7E-12 | 0.0093 (0.0220) | 0.672 | 46.8 | -0.832 |
| Whole body fat-free mass | All-cause dementia (FinnGen) | rs7731023 | 5:36181627 | G/A | 0.574 | 0.0086 (0.0013) | 7.8E-12 | 0.0133 (0.0201) | 0.507 | 46.8 | 1.552 |
| Whole body fat-free mass | All-cause dementia (FinnGen) | rs12981554 | 19:3416668 | G/A | 0.542 | -0.0086 (0.0013) | 8.9E-12 | -0.0118 (0.0194) | 0.544 | 46.6 | 1.374 |
| Whole body fat-free mass | All-cause dementia (FinnGen) | rs4648626 | 1:2200390 | A/C | 0.457 | 0.0085 (0.0012) | 9.1E-12 | -0.0027 (0.0193) | 0.890 | 46.5 | -0.318 |
| Whole body fat-free mass | All-cause dementia (FinnGen) | rs35874463 | 15:67457698 | G/A | 0.058 | 0.0182 (0.0027) | 9.7E-12 | 0.0005 (0.0497) | 0.993 | 46.4 | 0.028 |
| Whole body fat-free mass | All-cause dementia (FinnGen) | rs10995366 | 10:52772113 | A/G | 0.251 | -0.0098 (0.0014) | 1.1E-11 | 0.0348 (0.0218) | 0.110 | 46.1 | -3.561 |
| Whole body fat-free mass | All-cause dementia (FinnGen) | rs77344209 | 3:55515277 | T/C | 0.252 | 0.0098 (0.0014) | 1.2E-11 | -0.0046 (0.0245) | 0.852 | 46.0 | -0.469 |
| Whole body fat-free mass | All-cause dementia (FinnGen) | rs11593630 | 10:120491506 | T/C | 0.353 | -0.0088 (0.0013) | 1.2E-11 | -0.0055 (0.0199) | 0.781 | 46.0 | 0.625 |
| Whole body fat-free mass | All-cause dementia (FinnGen) | rs1418433 | 6:44752568 | A/G | 0.588 | -0.0085 (0.0013) | 1.3E-11 | 0.0095 (0.0194) | 0.623 | 45.8 | -1.116 |
| Whole body fat-free mass | All-cause dementia (FinnGen) | rs7912286 | 10:114693230 | G/A | 0.602 | -0.0087 (0.0013) | 1.3E-11 | 0.0448 (0.0196) | 0.022 | 45.8 | -5.168 |
| Whole body fat-free mass | All-cause dementia (FinnGen) | rs7321045 | 13:97034410 | A/G | 0.449 | 0.0085 (0.0013) | 1.3E-11 | -0.0567 (0.0192) | 0.003 | 45.8 | -6.648 |
| Whole body fat-free mass | All-cause dementia (FinnGen) | rs73004967 | 19:19717056 | G/A | 0.069 | -0.0167 (0.0025) | 1.3E-11 | -0.0076 (0.0402) | 0.849 | 45.8 | 0.454 |
| Whole body fat-free mass | All-cause dementia (FinnGen) | rs71423263 | 2:144024781 | G/T | 0.141 | 0.0120 (0.0018) | 1.5E-11 | 0.0293 (0.0307) | 0.340 | 45.5 | 2.443 |
| Whole body fat-free mass | All-cause dementia (FinnGen) | rs73213484 | 4:28489339 | T/A | 0.141 | -0.0120 (0.0018) | 1.5E-11 | 0.0071 (0.0271) | 0.794 | 45.5 | -0.591 |
| Whole body fat-free mass | All-cause dementia (FinnGen) | rs6085658 | 20:6685377 | T/C | 0.403 | -0.0086 (0.0013) | 1.6E-11 | 0.0004 (0.0193) | 0.983 | 45.4 | -0.047 |
| Whole body fat-free mass | All-cause dementia (FinnGen) | rs2122823 | 7:25939161 | T/C | 0.211 | 0.0103 (0.0015) | 2.0E-11 | 0.0017 (0.0223) | 0.940 | 45.0 | 0.165 |
| Whole body fat-free mass | All-cause dementia (FinnGen) | rs12427047 | 12:90213070 | T/C | 0.243 | -0.0097 (0.0014) | 2.1E-11 | 0.0027 (0.0201) | 0.894 | 44.9 | -0.279 |
| Whole body fat-free mass | All-cause dementia (FinnGen) | rs112957890 | 14:36220876 | G/A | 0.265 | 0.0095 (0.0014) | 2.1E-11 | -0.0419 (0.0229) | 0.067 | 44.8 | -4.394 |
| Whole body fat-free mass | All-cause dementia (FinnGen) | rs12729817 | 1:218971896 | G/A | 0.474 | -0.0083 (0.0012) | 2.2E-11 | -0.0253 (0.0191) | 0.184 | 44.8 | 3.057 |
| Whole body fat-free mass | All-cause dementia (FinnGen) | rs4268495 | 11:28645341 | C/G | 0.374 | -0.0086 (0.0013) | 2.2E-11 | -0.0123 (0.0198) | 0.535 | 44.8 | 1.433 |
| Whole body fat-free mass | All-cause dementia (FinnGen) | rs114949263 | 7:150498245 | C/T | 0.112 | -0.0132 (0.0020) | 2.5E-11 | -0.0176 (0.0358) | 0.623 | 44.5 | 1.335 |
| Whole body fat-free mass | All-cause dementia (FinnGen) | rs505575 | 5:111256812 | C/T | 0.673 | -0.0088 (0.0013) | 2.6E-11 | 0.0160 (0.0197) | 0.415 | 44.4 | -1.813 |
| Whole body fat-free mass | All-cause dementia (FinnGen) | rs114177791 | 17:78555512 | T/G | 0.214 | 0.0101 (0.0015) | 2.7E-11 | -0.0092 (0.0242) | 0.705 | 44.4 | -0.910 |
| Whole body fat-free mass | All-cause dementia (FinnGen) | rs61878760 | 11:12807189 | A/G | 0.083 | 0.0151 (0.0023) | 2.8E-11 | 0.0722 (0.0535) | 0.177 | 44.3 | 4.792 |
| Whole body fat-free mass | All-cause dementia (FinnGen) | rs3845344 | 1:75001480 | T/C | 0.391 | 0.0084 (0.0013) | 3.1E-11 | 0.0400 (0.0193) | 0.038 | 44.1 | 4.753 |
| Whole body fat-free mass | All-cause dementia (FinnGen) | rs9327336 | 5:123990270 | C/T | 0.343 | 0.0087 (0.0013) | 3.2E-11 | -0.0118 (0.0193) | 0.542 | 44.1 | -1.362 |
| Whole body fat-free mass | All-cause dementia (FinnGen) | rs2663125 | 15:99563891 | C/T | 0.691 | -0.0089 (0.0013) | 3.2E-11 | -0.0080 (0.0226) | 0.723 | 44.0 | 0.896 |
| Whole body fat-free mass | All-cause dementia (FinnGen) | rs67560975 | 17:47142930 | C/T | 0.116 | 0.0129 (0.0019) | 3.3E-11 | -0.0428 (0.0286) | 0.135 | 44.0 | -3.316 |
| Whole body fat-free mass | All-cause dementia (FinnGen) | rs1056747 | 9:35690102 | G/A | 0.412 | -0.0084 (0.0013) | 3.4E-11 | 0.0048 (0.0194) | 0.802 | 43.9 | -0.574 |
| Whole body fat-free mass | All-cause dementia (FinnGen) | rs17197114 | 14:21894526 | C/T | 0.177 | 0.0109 (0.0016) | 3.4E-11 | -0.0835 (0.0268) | 0.002 | 43.9 | -7.678 |
| Whole body fat-free mass | All-cause dementia (FinnGen) | rs12694042 | 2:207029825 | T/C | 0.502 | -0.0082 (0.0012) | 3.8E-11 | 0.0104 (0.0196) | 0.596 | 43.7 | -1.266 |
| Whole body fat-free mass | All-cause dementia (FinnGen) | rs4912905 | 5:142730376 | C/G | 0.249 | -0.0095 (0.0014) | 3.8E-11 | 0.0053 (0.0220) | 0.810 | 43.7 | -0.557 |
| Whole body fat-free mass | All-cause dementia (FinnGen) | rs77848106 | 1:107971673 | A/C | 0.296 | -0.0090 (0.0014) | 3.9E-11 | 0.0110 (0.0193) | 0.568 | 43.7 | -1.219 |
| Whole body fat-free mass | All-cause dementia (FinnGen) | rs4504126 | 3:33600582 | C/A | 0.027 | 0.0250 (0.0038) | 4.3E-11 | 0.0363 (0.0768) | 0.637 | 43.5 | 1.450 |
| Whole body fat-free mass | All-cause dementia (FinnGen) | rs4439140 | 8:23113908 | A/G | 0.418 | -0.0085 (0.0013) | 4.7E-11 | 0.0124 (0.0196) | 0.526 | 43.3 | -1.460 |
| Whole body fat-free mass | All-cause dementia (FinnGen) | rs10775406 | 17:46197755 | G/A | 0.760 | -0.0096 (0.0015) | 4.7E-11 | -0.0119 (0.0217) | 0.586 | 43.3 | 1.244 |
| Whole body fat-free mass | All-cause dementia (FinnGen) | rs6665399 | 1:21553933 | C/T | 0.445 | 0.0082 (0.0012) | 4.9E-11 | 0.0116 (0.0192) | 0.546 | 43.2 | 1.422 |
| Whole body fat-free mass | All-cause dementia (FinnGen) | rs3110496 | 17:27917771 | G/A | 0.686 | 0.0088 (0.0013) | 5.0E-11 | -0.0019 (0.0196) | 0.922 | 43.2 | -0.216 |
| Whole body fat-free mass | All-cause dementia (FinnGen) | rs10950207 | 7:70105168 | T/C | 0.384 | 0.0084 (0.0013) | 5.8E-11 | 0.0083 (0.0201) | 0.678 | 42.9 | 0.992 |
| Whole body fat-free mass | All-cause dementia (FinnGen) | rs9788443 | 14:23878279 | C/T | 0.048 | 0.0191 (0.0029) | 5.8E-11 | 0.0468 (0.0811) | 0.564 | 42.9 | 2.450 |
| Whole body fat-free mass | All-cause dementia (FinnGen) | rs4525525 | 17:1866892 | T/G | 0.264 | -0.0094 (0.0014) | 5.9E-11 | -0.0066 (0.0220) | 0.766 | 42.9 | 0.705 |
| Whole body fat-free mass | All-cause dementia (FinnGen) | rs1179905 | 6:90322922 | G/A | 0.195 | 0.0103 (0.0016) | 6.3E-11 | 0.0408 (0.0254) | 0.108 | 42.7 | 3.974 |
| Whole body fat-free mass | All-cause dementia (FinnGen) | rs6416914 | 17:39262850 | G/A | 0.695 | -0.0088 (0.0014) | 6.5E-11 | 0.0067 (0.0221) | 0.763 | 42.7 | -0.759 |
| Whole body fat-free mass | All-cause dementia (FinnGen) | rs8074074 | 17:17407816 | T/C | 0.755 | 0.0095 (0.0014) | 6.8E-11 | 0.0566 (0.0211) | 0.007 | 42.6 | 5.984 |
| Whole body fat-free mass | All-cause dementia (FinnGen) | rs34017457 | 16:67166731 | A/G | 0.007 | 0.0488 (0.0075) | 6.9E-11 | -0.0295 (0.1269) | 0.816 | 42.5 | -0.605 |
| Whole body fat-free mass | All-cause dementia (FinnGen) | rs10843397 | 12:29529523 | T/C | 0.242 | 0.0094 (0.0014) | 7.6E-11 | 0.0197 (0.0223) | 0.377 | 42.4 | 2.093 |
| Whole body fat-free mass | All-cause dementia (FinnGen) | rs10511111 | 3:80599757 | C/T | 0.309 | 0.0087 (0.0013) | 7.9E-11 | 0.0096 (0.0207) | 0.641 | 42.3 | 1.100 |
| Whole body fat-free mass | All-cause dementia (FinnGen) | rs113619763 | 22:46399175 | T/A | 0.062 | 0.0168 (0.0026) | 8.0E-11 | 0.0460 (0.0415) | 0.268 | 42.3 | 2.744 |
| Whole body fat-free mass | All-cause dementia (FinnGen) | rs1268947 | 10:121132631 | C/G | 0.126 | 0.0122 (0.0019) | 8.9E-11 | -0.0087 (0.0300) | 0.772 | 42.1 | -0.715 |
| Whole body fat-free mass | All-cause dementia (FinnGen) | rs60385590 | 3:53630199 | A/C | 0.258 | 0.0093 (0.0014) | 1.0E-10 | 0.0247 (0.0225) | 0.272 | 41.8 | 2.666 |
| Whole body fat-free mass | All-cause dementia (FinnGen) | rs7755185 | 6:152339615 | G/A | 0.311 | 0.0087 (0.0013) | 1.0E-10 | -0.0111 (0.0197) | 0.575 | 41.8 | -1.282 |
| Whole body fat-free mass | All-cause dementia (FinnGen) | rs56141370 | 9:127056213 | T/C | 0.115 | 0.0126 (0.0020) | 1.0E-10 | -0.0079 (0.0305) | 0.795 | 41.7 | -0.627 |
| Whole body fat-free mass | All-cause dementia (FinnGen) | rs4635681 | 3:152310614 | G/A | 0.156 | 0.0110 (0.0017) | 1.1E-10 | -0.0146 (0.0241) | 0.543 | 41.6 | -1.328 |
| Whole body fat-free mass | All-cause dementia (FinnGen) | rs1266876 | 6:51781580 | T/C | 0.350 | 0.0084 (0.0013) | 1.1E-10 | 0.0390 (0.0192) | 0.042 | 41.6 | 4.656 |
| Whole body fat-free mass | All-cause dementia (FinnGen) | rs240113 | 6:101058886 | A/G | 0.531 | -0.0080 (0.0012) | 1.1E-10 | -0.0115 (0.0191) | 0.548 | 41.7 | 1.433 |
| Whole body fat-free mass | All-cause dementia (FinnGen) | rs6900690 | 6:126223032 | A/G | 0.562 | 0.0080 (0.0012) | 1.1E-10 | 0.0281 (0.0194) | 0.148 | 41.6 | 3.499 |
| Whole body fat-free mass | All-cause dementia (FinnGen) | rs12156265 | 8:144848756 | A/G | 0.590 | 0.0082 (0.0013) | 1.1E-10 | -0.0281 (0.0193) | 0.146 | 41.6 | -3.442 |
| Whole body fat-free mass | All-cause dementia (FinnGen) | rs1008158 | 9:113828811 | G/A | 0.342 | 0.0085 (0.0013) | 1.1E-10 | -0.0026 (0.0197) | 0.896 | 41.6 | -0.306 |
| Whole body fat-free mass | All-cause dementia (FinnGen) | rs1407031 | 20:6542634 | T/C | 0.397 | -0.0082 (0.0013) | 1.2E-10 | -0.0053 (0.0194) | 0.785 | 41.4 | 0.645 |
| Whole body fat-free mass | All-cause dementia (FinnGen) | rs17115481 | 5:153358226 | A/G | 0.270 | -0.0090 (0.0014) | 1.3E-10 | -0.0021 (0.0208) | 0.920 | 41.4 | 0.233 |
| Whole body fat-free mass | All-cause dementia (FinnGen) | rs62466118 | 7:92716556 | A/G | 0.028 | -0.0255 (0.0040) | 1.4E-10 | -0.0336 (0.1084) | 0.757 | 41.1 | 1.320 |
| Whole body fat-free mass | All-cause dementia (FinnGen) | rs36100359 | 14:21578007 | A/G | 0.139 | -0.0115 (0.0018) | 1.4E-10 | 0.0117 (0.0271) | 0.664 | 41.2 | -1.016 |
| Whole body fat-free mass | All-cause dementia (FinnGen) | rs10498672 | 6:7797840 | G/C | 0.176 | 0.0104 (0.0016) | 1.6E-10 | 0.0108 (0.0232) | 0.641 | 40.9 | 1.040 |
| Whole body fat-free mass | All-cause dementia (FinnGen) | rs1657222 | 10:34837139 | A/G | 0.396 | 0.0081 (0.0013) | 1.7E-10 | 0.0253 (0.0191) | 0.186 | 40.8 | 3.110 |
| Whole body fat-free mass | All-cause dementia (FinnGen) | rs67141907 | 13:42805360 | T/C | 0.149 | 0.0112 (0.0018) | 1.7E-10 | 0.0103 (0.0308) | 0.737 | 40.8 | 0.920 |
| Whole body fat-free mass | All-cause dementia (FinnGen) | rs281385 | 19:49217261 | G/A | 0.879 | -0.0122 (0.0019) | 1.7E-10 | -0.0200 (0.0253) | 0.429 | 40.8 | 1.639 |
| Whole body fat-free mass | All-cause dementia (FinnGen) | rs62439025 | 6:166592164 | C/G | 0.152 | 0.0110 (0.0017) | 1.8E-10 | 0.0370 (0.0305) | 0.226 | 40.6 | 3.355 |
| Whole body fat-free mass | All-cause dementia (FinnGen) | rs6591 | 11:840363 | T/C | 0.544 | 0.0080 (0.0013) | 1.9E-10 | 0.0092 (0.0193) | 0.632 | 40.6 | 1.153 |
| Whole body fat-free mass | All-cause dementia (FinnGen) | rs7843128 | 8:22473465 | C/T | 0.357 | -0.0083 (0.0013) | 2.0E-10 | -0.0109 (0.0204) | 0.594 | 40.5 | 1.319 |
| Whole body fat-free mass | All-cause dementia (FinnGen) | rs62246311 | 3:9498143 | A/G | 0.102 | 0.0130 (0.0020) | 2.2E-10 | -0.0531 (0.0391) | 0.174 | 40.3 | -4.091 |
| Whole body fat-free mass | All-cause dementia (FinnGen) | rs12146350 | 10:22843111 | G/C | 0.602 | -0.0081 (0.0013) | 2.3E-10 | -0.0048 (0.0198) | 0.807 | 40.2 | 0.595 |
| Whole body fat-free mass | All-cause dementia (FinnGen) | rs4906203 | 14:102928991 | T/C | 0.234 | -0.0093 (0.0015) | 2.6E-10 | 0.0091 (0.0215) | 0.674 | 40.0 | -0.980 |
| Whole body fat-free mass | All-cause dementia (FinnGen) | rs10188231 | 2:142297493 | G/C | 0.186 | -0.0100 (0.0016) | 2.7E-10 | -0.0046 (0.0234) | 0.844 | 39.9 | 0.458 |
| Whole body fat-free mass | All-cause dementia (FinnGen) | rs10770705 | 12:20857467 | C/A | 0.663 | -0.0083 (0.0013) | 2.8E-10 | 0.0271 (0.0208) | 0.191 | 39.8 | -3.276 |
| Whole body fat-free mass | All-cause dementia (FinnGen) | rs6772164 | 3:196078149 | A/C | 0.358 | 0.0081 (0.0013) | 3.1E-10 | 0.0024 (0.0209) | 0.908 | 39.6 | 0.295 |
| Whole body fat-free mass | All-cause dementia (FinnGen) | rs76307059 | 6:169000888 | G/C | 0.046 | -0.0186 (0.0030) | 3.1E-10 | 0.0598 (0.0413) | 0.147 | 39.6 | -3.216 |
| Whole body fat-free mass | All-cause dementia (FinnGen) | rs141866277 | 15:43950699 | T/A | 0.024 | -0.0257 (0.0041) | 3.1E-10 | 0.0111 (0.1395) | 0.937 | 39.6 | -0.433 |
| Whole body fat-free mass | All-cause dementia (FinnGen) | rs3730071 | 12:49168798 | A/C | 0.030 | -0.0228 (0.0036) | 3.2E-10 | 0.1875 (0.0944) | 0.047 | 39.6 | -8.229 |
| Whole body fat-free mass | All-cause dementia (FinnGen) | rs11142700 | 9:73757155 | C/T | 0.408 | -0.0080 (0.0013) | 3.3E-10 | -0.0114 (0.0203) | 0.573 | 39.5 | 1.432 |
| Whole body fat-free mass | All-cause dementia (FinnGen) | rs10881583 | 9:137267439 | C/T | 0.241 | -0.0091 (0.0015) | 3.4E-10 | -0.0278 (0.0223) | 0.212 | 39.4 | 3.040 |
| Whole body fat-free mass | All-cause dementia (FinnGen) | rs8007644 | 14:35218831 | A/G | 0.388 | 0.0081 (0.0013) | 3.4E-10 | 0.0227 (0.0192) | 0.238 | 39.4 | 2.810 |
| Whole body fat-free mass | All-cause dementia (FinnGen) | rs10282707 | 7:17911038 | T/C | 0.397 | 0.0079 (0.0013) | 3.6E-10 | -0.0275 (0.0203) | 0.176 | 39.3 | -3.460 |
| Whole body fat-free mass | All-cause dementia (FinnGen) | rs6575340 | 14:94023972 | A/G | 0.636 | 0.0081 (0.0013) | 3.6E-10 | 0.0092 (0.0197) | 0.641 | 39.3 | 1.133 |
| Whole body fat-free mass | All-cause dementia (FinnGen) | rs11205354 | 1:150249101 | A/C | 0.444 | -0.0078 (0.0013) | 3.7E-10 | 0.0007 (0.0193) | 0.973 | 39.3 | -0.089 |
| Whole body fat-free mass | All-cause dementia (FinnGen) | rs13085472 | 3:171129859 | C/T | 0.666 | -0.0083 (0.0013) | 4.1E-10 | -0.0218 (0.0201) | 0.279 | 39.1 | 2.642 |
| Whole body fat-free mass | All-cause dementia (FinnGen) | rs12533548 | 7:148660579 | G/A | 0.651 | 0.0081 (0.0013) | 4.6E-10 | 0.0238 (0.0205) | 0.244 | 38.8 | 2.922 |
| Whole body fat-free mass | All-cause dementia (FinnGen) | rs112544217 | 2:20222686 | T/C | 0.022 | -0.0268 (0.0043) | 4.8E-10 | -0.0514 (0.0805) | 0.523 | 38.8 | 1.917 |
| Whole body fat-free mass | All-cause dementia (FinnGen) | rs284315 | 1:10734800 | G/A | 0.498 | -0.0077 (0.0012) | 4.9E-10 | -0.0034 (0.0198) | 0.863 | 38.7 | 0.443 |
| Whole body fat-free mass | All-cause dementia (FinnGen) | rs117451679 | 12:17245591 | G/A | 0.106 | 0.0127 (0.0020) | 5.0E-10 | 0.0047 (0.0290) | 0.871 | 38.7 | 0.370 |
| Whole body fat-free mass | All-cause dementia (FinnGen) | rs12047986 | 1:155043812 | G/A | 0.522 | -0.0077 (0.0012) | 5.1E-10 | -0.0313 (0.0199) | 0.116 | 38.6 | 4.066 |
| Whole body fat-free mass | All-cause dementia (FinnGen) | rs11937249 | 4:110755321 | T/G | 0.636 | -0.0080 (0.0013) | 5.1E-10 | -0.0030 (0.0194) | 0.879 | 38.6 | 0.374 |
| Whole body fat-free mass | All-cause dementia (FinnGen) | rs2422054 | 5:161382474 | A/T | 0.631 | 0.0080 (0.0013) | 5.1E-10 | -0.0106 (0.0192) | 0.581 | 38.6 | -1.326 |
| Whole body fat-free mass | All-cause dementia (FinnGen) | rs4819021 | 21:46466927 | C/T | 0.477 | -0.0078 (0.0013) | 6.4E-10 | 0.0404 (0.0192) | 0.035 | 38.2 | -5.187 |
| Whole body fat-free mass | All-cause dementia (FinnGen) | rs11684531 | 2:219835489 | G/A | 0.133 | -0.0113 (0.0018) | 6.6E-10 | -0.0582 (0.0379) | 0.125 | 38.1 | 5.171 |
| Whole body fat-free mass | All-cause dementia (FinnGen) | rs11833839 | 12:132560957 | T/C | 0.057 | 0.0166 (0.0027) | 6.8E-10 | 0.0031 (0.0271) | 0.910 | 38.1 | 0.187 |
| Whole body fat-free mass | All-cause dementia (FinnGen) | rs77093479 | 17:79429575 | G/C | 0.165 | -0.0104 (0.0017) | 6.9E-10 | 0.0060 (0.0254) | 0.814 | 38.0 | -0.576 |
| Whole body fat-free mass | All-cause dementia (FinnGen) | rs34825238 | 12:103328352 | T/G | 0.255 | 0.0089 (0.0014) | 7.1E-10 | -0.0114 (0.0244) | 0.640 | 38.0 | -1.282 |
| Whole body fat-free mass | All-cause dementia (FinnGen) | rs6946419 | 7:28768853 | C/T | 0.377 | -0.0079 (0.0013) | 7.2E-10 | 0.0232 (0.0196) | 0.237 | 38.0 | -2.931 |
| Whole body fat-free mass | All-cause dementia (FinnGen) | rs752070 | 2:74824970 | G/A | 0.126 | 0.0114 (0.0019) | 8.2E-10 | -0.0159 (0.0276) | 0.566 | 37.7 | -1.389 |
| Whole body fat-free mass | All-cause dementia (FinnGen) | rs2274116 | 9:139094805 | T/C | 0.341 | -0.0081 (0.0013) | 8.3E-10 | 0.0340 (0.0204) | 0.095 | 37.7 | -4.203 |
| Whole body fat-free mass | All-cause dementia (FinnGen) | rs6693481 | 1:203766395 | C/T | 0.695 | -0.0083 (0.0013) | 8.6E-10 | -0.0306 (0.0200) | 0.126 | 37.6 | 3.704 |
| Whole body fat-free mass | All-cause dementia (FinnGen) | rs2807742 | 10:28781367 | A/G | 0.771 | -0.0091 (0.0015) | 8.6E-10 | -0.0155 (0.0241) | 0.520 | 37.6 | 1.703 |
| Whole body fat-free mass | All-cause dementia (FinnGen) | rs161799 | 1:8015560 | G/A | 0.344 | -0.0080 (0.0013) | 9.0E-10 | -0.0197 (0.0209) | 0.346 | 37.5 | 2.453 |
| Whole body fat-free mass | All-cause dementia (FinnGen) | rs72754922 | 5:42547714 | A/G | 0.061 | -0.0160 (0.0026) | 9.1E-10 | 0.0017 (0.0444) | 0.969 | 37.5 | -0.107 |
| Whole body fat-free mass | All-cause dementia (FinnGen) | rs35665085 | 22:17625915 | A/G | 0.056 | -0.0166 (0.0027) | 9.2E-10 | 0.0091 (0.0427) | 0.832 | 37.5 | -0.548 |
| Whole body fat-free mass | All-cause dementia (FinnGen) | rs34227797 | 4:159982551 | C/G | 0.310 | 0.0082 (0.0013) | 9.3E-10 | 0.0059 (0.0208) | 0.776 | 37.5 | 0.719 |
| Whole body fat-free mass | All-cause dementia (FinnGen) | rs2611732 | 5:95855576 | G/A | 0.602 | 0.0077 (0.0013) | 9.3E-10 | -0.0034 (0.0208) | 0.871 | 37.5 | -0.439 |
| Whole body fat-free mass | All-cause dementia (FinnGen) | rs4980826 | 12:578349 | A/C | 0.396 | 0.0078 (0.0013) | 9.3E-10 | 0.0261 (0.0197) | 0.185 | 37.5 | 3.366 |
| Whole body fat-free mass | All-cause dementia (FinnGen) | rs1910466 | 3:147086268 | C/T | 0.498 | -0.0076 (0.0012) | 9.4E-10 | -0.0249 (0.0192) | 0.195 | 37.5 | 3.263 |
| Whole body fat-free mass | All-cause dementia (FinnGen) | rs56207600 | 11:126196537 | A/G | 0.111 | 0.0122 (0.0020) | 9.6E-10 | -0.0057 (0.0281) | 0.839 | 37.4 | -0.469 |
| Whole body fat-free mass | All-cause dementia (FinnGen) | rs11925245 | 3:114183579 | G/A | 0.183 | -0.0099 (0.0016) | 1.0E-09 | -0.0085 (0.0244) | 0.729 | 37.3 | 0.859 |
| Whole body fat-free mass | All-cause dementia (FinnGen) | rs1928850 | 9:16765520 | T/A | 0.922 | 0.0142 (0.0023) | 1.0E-09 | -0.0228 (0.0341) | 0.505 | 37.3 | -1.602 |
| Whole body fat-free mass | All-cause dementia (FinnGen) | rs1941697 | 18:31251276 | A/G | 0.449 | 0.0076 (0.0013) | 1.0E-09 | 0.0340 (0.0192) | 0.076 | 37.3 | 4.452 |
| Whole body fat-free mass | All-cause dementia (FinnGen) | rs4887925 | 16:73784416 | C/G | 0.413 | -0.0077 (0.0013) | 1.2E-09 | 0.0020 (0.0192) | 0.919 | 36.9 | -0.260 |
| Whole body fat-free mass | All-cause dementia (FinnGen) | rs6781248 | 3:13726684 | A/G | 0.546 | 0.0076 (0.0012) | 1.3E-09 | 0.0149 (0.0196) | 0.449 | 36.8 | 1.972 |
| Whole body fat-free mass | All-cause dementia (FinnGen) | rs3853474 | 5:141816555 | T/C | 0.377 | -0.0078 (0.0013) | 1.3E-09 | -0.0300 (0.0198) | 0.130 | 36.8 | 3.868 |
| Whole body fat-free mass | All-cause dementia (FinnGen) | rs9654451 | 5:299543 | T/C | 0.129 | 0.0112 (0.0019) | 1.4E-09 | 0.0004 (0.0334) | 0.991 | 36.7 | 0.036 |
| Whole body fat-free mass | All-cause dementia (FinnGen) | rs8002779 | 13:92015977 | A/G | 0.603 | -0.0077 (0.0013) | 1.5E-09 | -0.0069 (0.0191) | 0.719 | 36.5 | 0.901 |
| Whole body fat-free mass | All-cause dementia (FinnGen) | rs2521349 | 17:67503501 | A/G | 0.385 | 0.0077 (0.0013) | 1.6E-09 | 0.0105 (0.0195) | 0.590 | 36.4 | 1.358 |
| Whole body fat-free mass | All-cause dementia (FinnGen) | rs695922 | 5:53385529 | G/A | 0.837 | -0.0102 (0.0017) | 1.7E-09 | 0.0002 (0.0258) | 0.993 | 36.4 | -0.020 |
| Whole body fat-free mass | All-cause dementia (FinnGen) | rs6563808 | 13:40766662 | C/T | 0.734 | -0.0085 (0.0014) | 1.7E-09 | -0.0014 (0.0204) | 0.947 | 36.3 | 0.164 |
| Whole body fat-free mass | All-cause dementia (FinnGen) | rs5771118 | 22:50714289 | C/T | 0.742 | 0.0086 (0.0014) | 1.8E-09 | 0.0008 (0.0237) | 0.971 | 36.2 | 0.093 |
| Whole body fat-free mass | All-cause dementia (FinnGen) | rs41458449 | 2:46929951 | C/G | 0.144 | -0.0106 (0.0018) | 1.9E-09 | 0.0240 (0.0245) | 0.327 | 36.1 | -2.260 |
| Whole body fat-free mass | All-cause dementia (FinnGen) | rs6721191 | 2:10190115 | G/A | 0.578 | -0.0075 (0.0013) | 2.0E-09 | 0.0095 (0.0216) | 0.660 | 36.0 | -1.264 |
| Whole body fat-free mass | All-cause dementia (FinnGen) | rs72703409 | 4:184225295 | C/G | 0.066 | -0.0150 (0.0025) | 2.0E-09 | -0.0471 (0.0506) | 0.353 | 35.9 | 3.133 |
| Whole body fat-free mass | All-cause dementia (FinnGen) | rs572474 | 10:89791654 | G/A | 0.166 | 0.0100 (0.0017) | 2.0E-09 | 0.0071 (0.0257) | 0.781 | 36.0 | 0.708 |
| Whole body fat-free mass | All-cause dementia (FinnGen) | rs9898189 | 17:80480516 | G/C | 0.644 | -0.0081 (0.0013) | 2.1E-09 | 0.0192 (0.0195) | 0.325 | 35.9 | -2.375 |
| Whole body fat-free mass | All-cause dementia (FinnGen) | rs12731187 | 1:202013757 | T/C | 0.358 | -0.0078 (0.0013) | 2.5E-09 | 0.0089 (0.0197) | 0.652 | 35.5 | -1.146 |
| Whole body fat-free mass | All-cause dementia (FinnGen) | rs9365939 | 6:166336825 | G/A | 0.548 | -0.0074 (0.0012) | 2.5E-09 | 0.0036 (0.0199) | 0.858 | 35.5 | -0.486 |
| Whole body fat-free mass | All-cause dementia (FinnGen) | rs56130943 | 14:105906522 | C/A | 0.233 | 0.0090 (0.0015) | 2.9E-09 | -0.0025 (0.0242) | 0.916 | 35.3 | -0.279 |
| Whole body fat-free mass | All-cause dementia (FinnGen) | rs148662000 | 1:228721530 | T/G | 0.064 | -0.0151 (0.0025) | 3.0E-09 | 0.0078 (0.0461) | 0.865 | 35.2 | -0.517 |
| Whole body fat-free mass | All-cause dementia (FinnGen) | rs10939792 | 4:18483405 | G/C | 0.320 | 0.0079 (0.0013) | 3.2E-09 | -0.0035 (0.0219) | 0.875 | 35.1 | -0.445 |
| Whole body fat-free mass | All-cause dementia (FinnGen) | rs876672 | 16:81571600 | G/A | 0.085 | -0.0132 (0.0022) | 3.2E-09 | -0.0050 (0.0331) | 0.880 | 35.0 | 0.378 |
| Whole body fat-free mass | All-cause dementia (FinnGen) | rs7535501 | 1:176462695 | T/C | 0.944 | -0.0159 (0.0027) | 3.3E-09 | 0.0414 (0.0485) | 0.394 | 35.0 | -2.599 |
| Whole body fat-free mass | All-cause dementia (FinnGen) | rs116337081 | 2:183954625 | T/C | 0.070 | 0.0143 (0.0024) | 3.6E-09 | 0.0819 (0.0452) | 0.070 | 34.8 | 5.712 |
| Whole body fat-free mass | All-cause dementia (FinnGen) | rs2920665 | 8:6316097 | G/T | 0.174 | 0.0097 (0.0016) | 3.6E-09 | 0.0147 (0.0292) | 0.615 | 34.8 | 1.515 |
| Whole body fat-free mass | All-cause dementia (FinnGen) | rs1176314 | 13:81221014 | G/T | 0.550 | -0.0074 (0.0013) | 3.6E-09 | -0.0146 (0.0197) | 0.459 | 34.9 | 1.967 |
| Whole body fat-free mass | All-cause dementia (FinnGen) | rs13392079 | 2:169119178 | C/T | 0.756 | -0.0085 (0.0014) | 3.8E-09 | 0.0080 (0.0206) | 0.696 | 34.7 | -0.942 |
| Whole body fat-free mass | All-cause dementia (FinnGen) | rs224048 | 10:64491549 | A/G | 0.550 | -0.0073 (0.0012) | 3.8E-09 | 0.0322 (0.0191) | 0.092 | 34.7 | -4.385 |
| Whole body fat-free mass | All-cause dementia (FinnGen) | rs7280982 | 21:37468223 | A/G | 0.221 | -0.0088 (0.0015) | 4.2E-09 | -0.0096 (0.0226) | 0.670 | 34.5 | 1.085 |
| Whole body fat-free mass | All-cause dementia (FinnGen) | rs116165844 | 20:62610556 | T/G | 0.137 | -0.0106 (0.0018) | 4.3E-09 | -0.0278 (0.0303) | 0.358 | 34.5 | 2.620 |
| Whole body fat-free mass | All-cause dementia (FinnGen) | rs29946 | 19:34299865 | C/T | 0.557 | 0.0075 (0.0013) | 4.5E-09 | -0.0171 (0.0193) | 0.376 | 34.4 | -2.289 |
| Whole body fat-free mass | All-cause dementia (FinnGen) | rs182224 | 5:67316442 | T/C | 0.090 | 0.0127 (0.0022) | 4.6E-09 | 0.0274 (0.0294) | 0.352 | 34.4 | 2.153 |
| Whole body fat-free mass | All-cause dementia (FinnGen) | rs6747657 | 2:112003163 | A/G | 0.282 | 0.0081 (0.0014) | 4.7E-09 | 0.0408 (0.0214) | 0.057 | 34.3 | 5.068 |
| Whole body fat-free mass | All-cause dementia (FinnGen) | rs11263853 | 1:36579648 | A/G | 0.810 | 0.0092 (0.0016) | 4.8E-09 | -0.0360 (0.0230) | 0.118 | 34.2 | -3.896 |
| Whole body fat-free mass | All-cause dementia (FinnGen) | rs9985795 | 4:135213286 | C/T | 0.483 | -0.0073 (0.0012) | 5.1E-09 | -0.0155 (0.0193) | 0.420 | 34.2 | 2.132 |
| Whole body fat-free mass | All-cause dementia (FinnGen) | rs34055910 | 17:64460104 | G/A | 0.379 | -0.0075 (0.0013) | 5.1E-09 | 0.0224 (0.0192) | 0.242 | 34.2 | -2.985 |
| Whole body fat-free mass | All-cause dementia (FinnGen) | rs13185520 | 5:137807260 | A/G | 0.574 | -0.0073 (0.0013) | 5.4E-09 | 0.0079 (0.0199) | 0.691 | 34.1 | -1.081 |
| Whole body fat-free mass | All-cause dementia (FinnGen) | rs116817990 | 1:178989260 | G/A | 0.034 | -0.0201 (0.0034) | 5.7E-09 | -0.0641 (0.0531) | 0.228 | 33.9 | 3.190 |
| Whole body fat-free mass | All-cause dementia (FinnGen) | rs3772051 | 2:241672904 | A/G | 0.237 | -0.0086 (0.0015) | 5.9E-09 | -0.0242 (0.0300) | 0.419 | 33.9 | 2.811 |
| Whole body fat-free mass | All-cause dementia (FinnGen) | rs236587 | 17:68203915 | C/T | 0.737 | -0.0083 (0.0014) | 6.2E-09 | -0.0125 (0.0209) | 0.552 | 33.8 | 1.507 |
| Whole body fat-free mass | All-cause dementia (FinnGen) | rs118173451 | 22:28356600 | C/T | 0.016 | -0.0292 (0.0050) | 6.3E-09 | 0.0825 (0.0803) | 0.304 | 33.7 | -2.830 |
| Whole body fat-free mass | All-cause dementia (FinnGen) | rs6500249 | 16:49778147 | G/A | 0.735 | -0.0082 (0.0014) | 6.4E-09 | 0.0293 (0.0241) | 0.224 | 33.7 | -3.566 |
| Whole body fat-free mass | All-cause dementia (FinnGen) | rs5753630 | 22:31861950 | G/A | 0.439 | 0.0073 (0.0013) | 6.7E-09 | -0.0113 (0.0192) | 0.554 | 33.6 | -1.547 |
| Whole body fat-free mass | All-cause dementia (FinnGen) | rs237738 | 20:47904920 | C/T | 0.823 | 0.0094 (0.0016) | 7.3E-09 | -0.0092 (0.0253) | 0.716 | 33.5 | -0.976 |
| Whole body fat-free mass | All-cause dementia (FinnGen) | rs9800418 | 5:77641008 | C/T | 0.245 | 0.0084 (0.0014) | 7.9E-09 | 0.0071 (0.0219) | 0.744 | 33.3 | 0.849 |
| Whole body fat-free mass | All-cause dementia (FinnGen) | rs1421035 | 10:131136401 | T/C | 0.348 | 0.0076 (0.0013) | 8.0E-09 | -0.0200 (0.0199) | 0.317 | 33.3 | -2.637 |
| Whole body fat-free mass | All-cause dementia (FinnGen) | rs10020631 | 4:69353863 | A/G | 0.248 | -0.0083 (0.0014) | 8.2E-09 | -0.0019 (0.0224) | 0.932 | 33.2 | 0.229 |
| Whole body fat-free mass | All-cause dementia (FinnGen) | rs4676442 | 2:241740907 | C/T | 0.356 | 0.0074 (0.0013) | 9.1E-09 | 0.0087 (0.0195) | 0.654 | 33.0 | 1.171 |
| Whole body fat-free mass | All-cause dementia (FinnGen) | rs9491201 | 6:124934212 | T/C | 0.164 | 0.0096 (0.0017) | 9.5E-09 | 0.0449 (0.0374) | 0.230 | 32.9 | 4.677 |
| Whole body fat-free mass | All-cause dementia (FinnGen) | rs1846221 | 10:4667302 | C/G | 0.175 | -0.0095 (0.0017) | 9.7E-09 | 0.0159 (0.0253) | 0.529 | 32.9 | -1.673 |
| Whole body fat-free mass | All-cause dementia (FinnGen) | rs35651070 | 2:121613739 | A/G | 0.167 | -0.0095 (0.0017) | 9.9E-09 | -0.0062 (0.0267) | 0.817 | 32.9 | 0.652 |
| Whole body fat-free mass | All-cause dementia (FinnGen) | rs2920974 | 2:55537126 | C/A | 0.560 | 0.0072 (0.0013) | 1.1E-08 | -0.0347 (0.0206) | 0.092 | 32.7 | -4.850 |
| Whole body fat-free mass | All-cause dementia (FinnGen) | rs35612982 | 6:20682622 | C/T | 0.178 | -0.0092 (0.0016) | 1.1E-08 | -0.0029 (0.0249) | 0.907 | 32.7 | 0.314 |
| Whole body fat-free mass | All-cause dementia (FinnGen) | rs8091374 | 18:71904385 | A/G | 0.161 | -0.0099 (0.0017) | 1.1E-08 | -0.0349 (0.0244) | 0.153 | 32.7 | 3.535 |
| Whole body fat-free mass | All-cause dementia (FinnGen) | rs34345560 | 11:69081998 | A/G | 0.195 | 0.0090 (0.0016) | 1.2E-08 | -0.0093 (0.0220) | 0.674 | 32.4 | -1.034 |
| Whole body fat-free mass | All-cause dementia (FinnGen) | rs6743107 | 2:100844781 | T/C | 0.275 | 0.0079 (0.0014) | 1.3E-08 | 0.0340 (0.0208) | 0.101 | 32.3 | 4.321 |
| Whole body fat-free mass | All-cause dementia (FinnGen) | rs9783665 | 14:103271158 | A/T | 0.653 | -0.0074 (0.0013) | 1.3E-08 | 0.0354 (0.0201) | 0.078 | 32.3 | -4.772 |
| Whole body fat-free mass | All-cause dementia (FinnGen) | rs8088739 | 18:60767088 | C/T | 0.244 | 0.0082 (0.0014) | 1.3E-08 | 0.0336 (0.0226) | 0.138 | 32.3 | 4.084 |
| Whole body fat-free mass | All-cause dementia (FinnGen) | rs34831515 | 19:17275777 | T/C | 0.232 | -0.0084 (0.0015) | 1.3E-08 | 0.0265 (0.0206) | 0.198 | 32.3 | -3.154 |
| Whole body fat-free mass | All-cause dementia (FinnGen) | rs35050648 | 19:46991243 | T/G | 0.236 | 0.0083 (0.0015) | 1.6E-08 | 0.0339 (0.0220) | 0.124 | 32.0 | 4.091 |
| Whole body fat-free mass | All-cause dementia (FinnGen) | rs62460525 | 7:44902676 | A/C | 0.070 | 0.0137 (0.0024) | 1.7E-08 | 0.0135 (0.0558) | 0.809 | 31.8 | 0.985 |
| Whole body fat-free mass | All-cause dementia (FinnGen) | rs2740761 | 7:55255986 | T/C | 0.208 | 0.0087 (0.0015) | 1.7E-08 | -0.0032 (0.0243) | 0.894 | 31.8 | -0.367 |
| Whole body fat-free mass | All-cause dementia (FinnGen) | rs113978196 | 15:75916965 | A/G | 0.251 | -0.0081 (0.0014) | 1.8E-08 | 0.0456 (0.0215) | 0.034 | 31.8 | -5.645 |
| Whole body fat-free mass | All-cause dementia (FinnGen) | rs1043801 | 21:47552527 | A/G | 0.058 | 0.0151 (0.0027) | 1.8E-08 | -0.0699 (0.0403) | 0.083 | 31.7 | -4.636 |
| Whole body fat-free mass | All-cause dementia (FinnGen) | rs10848288 | 12:131600054 | T/C | 0.304 | -0.0076 (0.0014) | 1.9E-08 | -0.0260 (0.0216) | 0.228 | 31.6 | 3.418 |
| Whole body fat-free mass | All-cause dementia (FinnGen) | rs3751866 | 16:84771304 | C/T | 0.247 | -0.0081 (0.0014) | 1.9E-08 | -0.0187 (0.0224) | 0.404 | 31.6 | 2.303 |
| Whole body fat-free mass | All-cause dementia (FinnGen) | rs111598585 | 4:171635471 | T/C | 0.209 | -0.0086 (0.0015) | 2.0E-08 | -0.0759 (0.0316) | 0.016 | 31.5 | 8.840 |
| Whole body fat-free mass | All-cause dementia (FinnGen) | rs2884345 | 14:53323211 | A/G | 0.710 | -0.0077 (0.0014) | 2.1E-08 | -0.0267 (0.0211) | 0.206 | 31.4 | 3.472 |
| Whole body fat-free mass | All-cause dementia (FinnGen) | rs2252909 | 17:2278609 | T/C | 0.657 | -0.0075 (0.0013) | 2.1E-08 | -0.0325 (0.0194) | 0.093 | 31.4 | 4.354 |
| Whole body fat-free mass | All-cause dementia (FinnGen) | rs12553221 | 9:37234008 | A/G | 0.362 | 0.0073 (0.0013) | 2.2E-08 | -0.0103 (0.0199) | 0.606 | 31.3 | -1.420 |
| Whole body fat-free mass | All-cause dementia (FinnGen) | rs498685 | 18:13088673 | C/T | 0.594 | -0.0071 (0.0013) | 2.2E-08 | -0.0110 (0.0193) | 0.569 | 31.3 | 1.553 |
| Whole body fat-free mass | All-cause dementia (FinnGen) | rs17011108 | 4:86800806 | C/T | 0.307 | 0.0076 (0.0014) | 2.3E-08 | 0.0236 (0.0214) | 0.271 | 31.2 | 3.117 |
| Whole body fat-free mass | All-cause dementia (FinnGen) | rs12795042 | 11:133658168 | C/A | 0.622 | 0.0073 (0.0013) | 2.3E-08 | 0.0094 (0.0210) | 0.652 | 31.3 | 1.296 |
| Whole body fat-free mass | All-cause dementia (FinnGen) | rs4800670 | 18:23415263 | C/G | 0.386 | -0.0072 (0.0013) | 2.3E-08 | -0.0431 (0.0196) | 0.028 | 31.3 | 6.011 |
| Whole body fat-free mass | All-cause dementia (FinnGen) | rs73189390 | 21:17383170 | A/G | 0.184 | -0.0090 (0.0016) | 2.3E-08 | -0.0255 (0.0241) | 0.290 | 31.3 | 2.819 |
| Whole body fat-free mass | All-cause dementia (FinnGen) | rs55854145 | 18:45928049 | C/A | 0.055 | -0.0153 (0.0027) | 2.4E-08 | -0.0191 (0.0490) | 0.697 | 31.1 | 1.247 |
| Whole body fat-free mass | All-cause dementia (FinnGen) | rs7513326 | 1:159895536 | A/G | 0.508 | -0.0070 (0.0013) | 2.5E-08 | 0.0265 (0.0193) | 0.169 | 31.1 | -3.780 |
| Whole body fat-free mass | All-cause dementia (FinnGen) | rs73158212 | 7:148383377 | G/A | 0.224 | -0.0084 (0.0015) | 2.5E-08 | -0.0638 (0.0257) | 0.013 | 31.1 | 7.598 |
| Whole body fat-free mass | All-cause dementia (FinnGen) | rs6514066 | 20:44500794 | G/T | 0.624 | 0.0072 (0.0013) | 2.5E-08 | -0.0514 (0.0198) | 0.009 | 31.1 | -7.170 |
| Whole body fat-free mass | All-cause dementia (FinnGen) | rs2427320 | 20:60976723 | G/A | 0.232 | -0.0082 (0.0015) | 2.5E-08 | -0.0016 (0.0257) | 0.951 | 31.1 | 0.194 |
| Whole body fat-free mass | All-cause dementia (FinnGen) | rs4821940 | 22:40659573 | C/T | 0.550 | -0.0070 (0.0013) | 2.5E-08 | -0.0160 (0.0192) | 0.403 | 31.1 | 2.286 |
| Whole body fat-free mass | All-cause dementia (FinnGen) | rs1168768 | 12:66509650 | T/C | 0.975 | 0.0221 (0.0040) | 2.6E-08 | 0.0855 (0.0643) | 0.184 | 31.0 | 3.864 |
| Whole body fat-free mass | All-cause dementia (FinnGen) | rs7095768 | 10:53256505 | T/G | 0.263 | 0.0078 (0.0014) | 2.7E-08 | -0.0138 (0.0222) | 0.536 | 30.9 | -1.763 |
| Whole body fat-free mass | All-cause dementia (FinnGen) | rs2293177 | 7:140244560 | T/C | 0.313 | 0.0074 (0.0013) | 2.9E-08 | 0.0361 (0.0203) | 0.075 | 30.8 | 4.864 |
| Whole body fat-free mass | All-cause dementia (FinnGen) | rs13125082 | 4:57787000 | G/T | 0.278 | -0.0076 (0.0014) | 3.4E-08 | -0.0129 (0.0232) | 0.580 | 30.5 | 1.690 |
| Whole body fat-free mass | All-cause dementia (FinnGen) | rs7683836 | 4:180167906 | A/G | 0.557 | -0.0069 (0.0013) | 3.4E-08 | 0.0145 (0.0191) | 0.449 | 30.5 | -2.099 |
| Whole body fat-free mass | All-cause dementia (FinnGen) | rs926436 | 1:22563429 | A/G | 0.857 | 0.0098 (0.0018) | 3.5E-08 | -0.0186 (0.0269) | 0.489 | 30.4 | -1.902 |
| Whole body fat-free mass | All-cause dementia (FinnGen) | rs34345690 | 3:192445575 | G/A | 0.227 | 0.0082 (0.0015) | 3.5E-08 | 0.0211 (0.0240) | 0.379 | 30.4 | 2.587 |
| Whole body fat-free mass | All-cause dementia (FinnGen) | rs11021307 | 11:95501407 | T/C | 0.418 | 0.0069 (0.0013) | 3.6E-08 | 0.0204 (0.0201) | 0.309 | 30.4 | 2.943 |
| Whole body fat-free mass | All-cause dementia (FinnGen) | rs294382 | 11:30607515 | C/A | 0.720 | -0.0076 (0.0014) | 4.0E-08 | 0.0109 (0.0230) | 0.635 | 30.1 | -1.434 |
| Whole body fat-free mass | All-cause dementia (FinnGen) | rs434072 | 3:12916946 | G/T | 0.709 | -0.0075 (0.0014) | 4.1E-08 | -0.0231 (0.0203) | 0.254 | 30.1 | 3.069 |
| Whole body fat-free mass | All-cause dementia (FinnGen) | rs1028197 | 4:124977659 | T/C | 0.225 | 0.0082 (0.0015) | 4.1E-08 | 0.0009 (0.0256) | 0.971 | 30.1 | 0.110 |
| Whole body fat-free mass | All-cause dementia (FinnGen) | rs12213409 | 6:142610314 | G/T | 0.485 | -0.0068 (0.0012) | 4.7E-08 | -0.0194 (0.0191) | 0.310 | 29.8 | 2.847 |
| Whole body fat-free mass | Vascular dementia (FinnGen) | rs12967135 | 18:57849023 | A/G | 0.233 | 0.0433 (0.0015) | 2.3E-190 | 0.0187 (0.0632) | 0.767 | 866.1 | 0.432 |
| Whole body fat-free mass | Vascular dementia (FinnGen) | rs56094641 | 16:53806453 | G/A | 0.405 | 0.0361 (0.0013) | 1.0E-178 | 0.0131 (0.0498) | 0.793 | 812.5 | 0.363 |
| Whole body fat-free mass | Vascular dementia (FinnGen) | rs143384 | 20:34025756 | G/A | 0.404 | 0.0354 (0.0013) | 1.5E-171 | 0.0484 (0.0498) | 0.331 | 779.6 | 1.369 |
| Whole body fat-free mass | Vascular dementia (FinnGen) | rs9853018 | 3:141101961 | T/C | 0.443 | 0.0300 (0.0012) | 6.3E-128 | 0.0354 (0.0496) | 0.476 | 579.0 | 1.181 |
| Whole body fat-free mass | Vascular dementia (FinnGen) | rs2744956 | 6:34618937 | C/T | 0.139 | 0.0378 (0.0018) | 2.9E-99 | 0.0352 (0.0627) | 0.574 | 447.2 | 0.932 |
| Whole body fat-free mass | Vascular dementia (FinnGen) | rs1351394 | 12:66351826 | C/T | 0.512 | -0.0258 (0.0012) | 5.3E-96 | -0.0450 (0.0492) | 0.360 | 432.2 | 1.745 |
| Whole body fat-free mass | Vascular dementia (FinnGen) | rs7740107 | 6:130374461 | A/T | 0.736 | -0.0291 (0.0014) | 1.1E-95 | 0.0423 (0.0552) | 0.443 | 430.7 | -1.452 |
| Whole body fat-free mass | Vascular dementia (FinnGen) | rs12995666 | 2:629959 | C/T | 0.828 | 0.0337 (0.0016) | 1.1E-93 | 0.0358 (0.0664) | 0.590 | 421.6 | 1.063 |
| Whole body fat-free mass | Vascular dementia (FinnGen) | rs72656010 | 8:57122215 | C/T | 0.132 | -0.0366 (0.0018) | 2.8E-88 | 0.0155 (0.0743) | 0.835 | 396.7 | -0.423 |
| Whole body fat-free mass | Vascular dementia (FinnGen) | rs76895963 | 12:4384844 | G/T | 0.021 | 0.0952 (0.0048) | 2.8E-87 | -0.1199 (0.1500) | 0.424 | 392.2 | -1.259 |
| Whole body fat-free mass | Vascular dementia (FinnGen) | rs10269774 | 7:92253972 | A/G | 0.326 | 0.0262 (0.0013) | 4.7E-87 | -0.0407 (0.0536) | 0.447 | 391.1 | -1.555 |
| Whole body fat-free mass | Vascular dementia (FinnGen) | rs2225226 | 13:51127270 | T/C | 0.216 | -0.0298 (0.0015) | 3.1E-86 | 0.0462 (0.0563) | 0.412 | 387.4 | -1.549 |
| Whole body fat-free mass | Vascular dementia (FinnGen) | rs34517439 | 1:78450517 | A/C | 0.122 | 0.0372 (0.0019) | 3.3E-84 | 0.0595 (0.0740) | 0.422 | 378.1 | 1.598 |
| Whole body fat-free mass | Vascular dementia (FinnGen) | rs7671110 | 4:17874089 | T/C | 0.158 | -0.0318 (0.0017) | 2.8E-78 | -0.0671 (0.0840) | 0.424 | 350.8 | 2.111 |
| Whole body fat-free mass | Vascular dementia (FinnGen) | rs7223535 | 17:29211667 | A/G | 0.270 | -0.0251 (0.0014) | 1.0E-71 | -0.0446 (0.0544) | 0.412 | 320.7 | 1.780 |
| Whole body fat-free mass | Vascular dementia (FinnGen) | rs77165542 | 2:430975 | T/C | 0.035 | -0.0585 (0.0034) | 6.6E-67 | -0.4020 (0.1837) | 0.029 | 298.6 | 6.875 |
| Whole body fat-free mass | Vascular dementia (FinnGen) | rs2005172 | 17:61996255 | C/A | 0.640 | 0.0226 (0.0013) | 1.0E-66 | -0.1046 (0.0503) | 0.038 | 297.7 | -4.627 |
| Whole body fat-free mass | Vascular dementia (FinnGen) | rs4788218 | 16:30055750 | C/T | 0.401 | 0.0218 (0.0013) | 3.6E-66 | 0.0151 (0.0512) | 0.768 | 295.2 | 0.693 |
| Whole body fat-free mass | Vascular dementia (FinnGen) | rs4369779 | 18:20735408 | C/T | 0.789 | 0.0260 (0.0015) | 2.6E-65 | 0.0232 (0.0583) | 0.691 | 291.3 | 0.892 |
| Whole body fat-free mass | Vascular dementia (FinnGen) | rs4240326 | 4:145839264 | G/A | 0.550 | -0.0212 (0.0012) | 3.7E-65 | -0.0971 (0.0492) | 0.048 | 290.6 | 4.573 |
| Whole body fat-free mass | Vascular dementia (FinnGen) | rs4899012 | 14:61003889 | C/G | 0.607 | -0.0215 (0.0013) | 1.1E-63 | 0.0441 (0.0538) | 0.413 | 283.8 | -2.055 |
| Whole body fat-free mass | Vascular dementia (FinnGen) | rs527248 | 1:177875514 | G/A | 0.209 | 0.0255 (0.0015) | 2.5E-63 | -0.0558 (0.0635) | 0.379 | 282.2 | -2.185 |
| Whole body fat-free mass | Vascular dementia (FinnGen) | rs76798800 | 1:154994978 | T/G | 0.266 | 0.0228 (0.0014) | 2.3E-59 | 0.0333 (0.0582) | 0.567 | 264.0 | 1.462 |
| Whole body fat-free mass | Vascular dementia (FinnGen) | rs9634212 | 12:93993266 | A/C | 0.221 | 0.0237 (0.0015) | 4.0E-56 | 0.0647 (0.0569) | 0.256 | 249.2 | 2.730 |
| Whole body fat-free mass | Vascular dementia (FinnGen) | rs9398171 | 6:108983527 | T/C | 0.711 | 0.0208 (0.0014) | 1.8E-52 | 0.0008 (0.0515) | 0.988 | 232.4 | 0.038 |
| Whole body fat-free mass | Vascular dementia (FinnGen) | rs34879158 | 20:32300634 | C/A | 0.263 | -0.0215 (0.0014) | 2.2E-52 | -0.0920 (0.0585) | 0.116 | 232.0 | 4.270 |
| Whole body fat-free mass | Vascular dementia (FinnGen) | rs2524137 | 6:31264582 | T/C | 0.696 | -0.0206 (0.0014) | 2.3E-52 | 0.0869 (0.0574) | 0.130 | 231.9 | -4.226 |
| Whole body fat-free mass | Vascular dementia (FinnGen) | rs2533879 | 7:2859847 | A/G | 0.300 | -0.0206 (0.0014) | 3.4E-52 | -0.0187 (0.0511) | 0.715 | 231.1 | 0.908 |
| Whole body fat-free mass | Vascular dementia (FinnGen) | rs7033487 | 9:119129257 | C/T | 0.198 | -0.0235 (0.0016) | 4.5E-51 | -0.0646 (0.0585) | 0.269 | 226.0 | 2.753 |
| Whole body fat-free mass | Vascular dementia (FinnGen) | rs1582931 | 5:122657199 | A/G | 0.473 | -0.0188 (0.0013) | 8.7E-51 | 0.0606 (0.0490) | 0.216 | 224.7 | -3.228 |
| Whole body fat-free mass | Vascular dementia (FinnGen) | rs1260326 | 2:27730940 | C/T | 0.604 | 0.0189 (0.0013) | 1.1E-50 | -0.0084 (0.0516) | 0.870 | 224.2 | -0.444 |
| Whole body fat-free mass | Vascular dementia (FinnGen) | rs41271299 | 6:19839415 | T/C | 0.051 | 0.0419 (0.0028) | 2.3E-50 | 0.1350 (0.1957) | 0.490 | 222.7 | 3.224 |
| Whole body fat-free mass | Vascular dementia (FinnGen) | rs7952436 | 11:67024534 | T/C | 0.082 | -0.0336 (0.0023) | 6.7E-50 | 0.0919 (0.1125) | 0.414 | 220.6 | -2.733 |
| Whole body fat-free mass | Vascular dementia (FinnGen) | rs894360 | 8:135601194 | C/T | 0.366 | -0.0190 (0.0013) | 5.7E-49 | 0.0179 (0.0495) | 0.718 | 216.3 | -0.941 |
| Whole body fat-free mass | Vascular dementia (FinnGen) | rs11243202 | 6:7719065 | C/T | 0.486 | 0.0183 (0.0012) | 6.3E-49 | 0.0483 (0.0495) | 0.329 | 216.2 | 2.642 |
| Whole body fat-free mass | Vascular dementia (FinnGen) | rs6946415 | 7:150684548 | G/A | 0.627 | 0.0189 (0.0013) | 6.3E-49 | 0.0254 (0.0519) | 0.624 | 216.1 | 1.344 |
| Whole body fat-free mass | Vascular dementia (FinnGen) | rs2885697 | 1:41544279 | T/G | 0.665 | -0.0189 (0.0013) | 5.1E-47 | -0.0071 (0.0519) | 0.891 | 207.4 | 0.377 |
| Whole body fat-free mass | Vascular dementia (FinnGen) | rs72885917 | 2:172416376 | C/A | 0.247 | -0.0205 (0.0014) | 2.2E-46 | 0.0455 (0.0623) | 0.466 | 204.4 | -2.222 |
| Whole body fat-free mass | Vascular dementia (FinnGen) | rs78378222 | 17:7571752 | G/T | 0.012 | 0.0820 (0.0058) | 7.4E-46 | -0.1583 (0.1917) | 0.409 | 202.1 | -1.931 |
| Whole body fat-free mass | Vascular dementia (FinnGen) | rs73175572 | 3:185490184 | G/A | 0.112 | 0.0280 (0.0020) | 6.1E-45 | -0.0529 (0.0772) | 0.494 | 197.9 | -1.892 |
| Whole body fat-free mass | Vascular dementia (FinnGen) | rs28620532 | 9:98216876 | G/A | 0.349 | 0.0182 (0.0013) | 6.4E-44 | 0.0171 (0.0511) | 0.737 | 193.2 | 0.940 |
| Whole body fat-free mass | Vascular dementia (FinnGen) | rs7842996 | 8:78107140 | A/T | 0.284 | 0.0192 (0.0014) | 7.7E-44 | 0.0414 (0.0593) | 0.485 | 192.8 | 2.160 |
| Whole body fat-free mass | Vascular dementia (FinnGen) | rs7129320 | 11:68388220 | A/G | 0.166 | -0.0231 (0.0017) | 1.1E-43 | 0.1099 (0.0864) | 0.203 | 192.1 | -4.751 |
| Whole body fat-free mass | Vascular dementia (FinnGen) | rs71385734 | 16:2160503 | G/T | 0.170 | -0.0231 (0.0017) | 1.2E-43 | -0.0392 (0.0647) | 0.544 | 191.9 | 1.699 |
| Whole body fat-free mass | Vascular dementia (FinnGen) | rs146851424 | 13:50377910 | C/A | 0.022 | 0.0597 (0.0043) | 1.6E-43 | 0.1165 (0.1841) | 0.527 | 191.3 | 1.952 |
| Whole body fat-free mass | Vascular dementia (FinnGen) | rs62372052 | 5:42724294 | G/A | 0.110 | 0.0266 (0.0020) | 6.8E-41 | 0.0678 (0.0757) | 0.371 | 179.3 | 2.547 |
| Whole body fat-free mass | Vascular dementia (FinnGen) | rs1478575 | 2:218278555 | A/T | 0.684 | 0.0175 (0.0013) | 1.7E-39 | -0.0093 (0.0496) | 0.852 | 172.9 | -0.531 |
| Whole body fat-free mass | Vascular dementia (FinnGen) | rs2071286 | 6:32179896 | T/C | 0.182 | -0.0209 (0.0016) | 4.2E-39 | 0.0181 (0.0575) | 0.753 | 171.1 | -0.866 |
| Whole body fat-free mass | Vascular dementia (FinnGen) | rs2101975 | 4:106216667 | G/A | 0.432 | -0.0164 (0.0013) | 7.4E-39 | -0.0396 (0.0491) | 0.420 | 170.0 | 2.419 |
| Whole body fat-free mass | Vascular dementia (FinnGen) | rs62515437 | 8:57160328 | T/G | 0.225 | 0.0193 (0.0015) | 1.4E-38 | -0.0658 (0.0542) | 0.225 | 168.7 | -3.406 |
| Whole body fat-free mass | Vascular dementia (FinnGen) | rs2307111 | 5:75003678 | C/T | 0.395 | -0.0164 (0.0013) | 2.1E-38 | 0.0360 (0.0498) | 0.470 | 167.9 | -2.189 |
| Whole body fat-free mass | Vascular dementia (FinnGen) | rs1573891 | 15:99186488 | C/G | 0.158 | -0.0220 (0.0017) | 7.5E-38 | 0.1875 (0.0939) | 0.046 | 165.4 | -8.508 |
| Whole body fat-free mass | Vascular dementia (FinnGen) | rs36000545 | 17:79093822 | G/A | 0.396 | -0.0166 (0.0013) | 8.2E-38 | 0.0294 (0.0521) | 0.572 | 165.2 | -1.776 |
| Whole body fat-free mass | Vascular dementia (FinnGen) | rs485554 | 3:172137343 | C/G | 0.315 | 0.0171 (0.0013) | 1.8E-37 | 0.0725 (0.0525) | 0.167 | 163.7 | 4.232 |
| Whole body fat-free mass | Vascular dementia (FinnGen) | rs3810291 | 19:47569003 | A/G | 0.675 | 0.0170 (0.0013) | 2.4E-37 | -0.0418 (0.0512) | 0.414 | 163.1 | -2.465 |
| Whole body fat-free mass | Vascular dementia (FinnGen) | rs1047891 | 2:211540507 | A/C | 0.316 | 0.0169 (0.0013) | 6.0E-37 | -0.1229 (0.0530) | 0.020 | 161.3 | -7.278 |
| Whole body fat-free mass | Vascular dementia (FinnGen) | rs72892910 | 6:50816887 | T/G | 0.172 | 0.0207 (0.0016) | 2.5E-36 | -0.0123 (0.0612) | 0.841 | 158.4 | -0.595 |
| Whole body fat-free mass | Vascular dementia (FinnGen) | rs11030112 | 11:27705188 | A/G | 0.319 | 0.0167 (0.0013) | 3.6E-36 | -0.0985 (0.0522) | 0.059 | 157.7 | -5.889 |
| Whole body fat-free mass | Vascular dementia (FinnGen) | rs59985551 | 2:56106928 | T/C | 0.226 | -0.0185 (0.0015) | 8.6E-36 | 0.0247 (0.0592) | 0.676 | 156.0 | -1.339 |
| Whole body fat-free mass | Vascular dementia (FinnGen) | rs4282339 | 5:168256240 | A/G | 0.208 | -0.0189 (0.0015) | 2.4E-35 | -0.0753 (0.0632) | 0.234 | 153.9 | 3.980 |
| Whole body fat-free mass | Vascular dementia (FinnGen) | rs7132908 | 12:50263148 | A/G | 0.384 | 0.0158 (0.0013) | 2.9E-35 | 0.0277 (0.0507) | 0.584 | 153.6 | 1.752 |
| Whole body fat-free mass | Vascular dementia (FinnGen) | rs12713004 | 2:23896049 | G/A | 0.725 | 0.0172 (0.0014) | 3.9E-35 | -0.0824 (0.0642) | 0.199 | 153.0 | -4.804 |
| Whole body fat-free mass | Vascular dementia (FinnGen) | rs10773172 | 12:122939655 | A/G | 0.742 | 0.0175 (0.0014) | 6.6E-35 | 0.0383 (0.0622) | 0.538 | 151.9 | 2.190 |
| Whole body fat-free mass | Vascular dementia (FinnGen) | rs35506085 | 11:2165576 | A/G | 0.185 | -0.0198 (0.0016) | 8.1E-35 | 0.1220 (0.0657) | 0.063 | 151.5 | -6.148 |
| Whole body fat-free mass | Vascular dementia (FinnGen) | rs25849 | 16:2265071 | G/C | 0.289 | 0.0169 (0.0014) | 1.3E-34 | -0.0128 (0.0515) | 0.804 | 150.5 | -0.758 |
| Whole body fat-free mass | Vascular dementia (FinnGen) | rs2897968 | 12:46763686 | A/G | 0.607 | 0.0156 (0.0013) | 1.4E-34 | -0.0992 (0.0503) | 0.049 | 150.4 | -6.356 |
| Whole body fat-free mass | Vascular dementia (FinnGen) | rs3853252 | 6:152170247 | A/G | 0.455 | 0.0153 (0.0012) | 1.7E-34 | 0.0659 (0.0502) | 0.189 | 150.0 | 4.309 |
| Whole body fat-free mass | Vascular dementia (FinnGen) | rs9372837 | 6:126638124 | A/G | 0.471 | 0.0152 (0.0013) | 6.8E-34 | -0.0241 (0.0491) | 0.623 | 147.3 | -1.587 |
| Whole body fat-free mass | Vascular dementia (FinnGen) | rs12314162 | 12:124826676 | T/C | 0.161 | 0.0204 (0.0017) | 1.5E-33 | -0.0198 (0.0689) | 0.774 | 145.7 | -0.971 |
| Whole body fat-free mass | Vascular dementia (FinnGen) | rs9892365 | 17:59491384 | G/A | 0.670 | -0.0158 (0.0013) | 8.9E-33 | 0.0560 (0.0553) | 0.311 | 142.2 | -3.549 |
| Whole body fat-free mass | Vascular dementia (FinnGen) | rs34776209 | 7:23513093 | T/C | 0.247 | -0.0171 (0.0014) | 1.5E-32 | 0.0852 (0.0606) | 0.160 | 141.1 | -4.978 |
| Whole body fat-free mass | Vascular dementia (FinnGen) | rs244711 | 5:176509193 | T/C | 0.686 | 0.0168 (0.0014) | 1.2E-31 | -0.1214 (0.0537) | 0.024 | 136.9 | -7.238 |
| Whole body fat-free mass | Vascular dementia (FinnGen) | rs13043303 | 20:51091385 | A/G | 0.183 | -0.0186 (0.0016) | 6.2E-31 | 0.0516 (0.0675) | 0.445 | 133.7 | -2.772 |
| Whole body fat-free mass | Vascular dementia (FinnGen) | rs41311445 | 22:42070374 | C/A | 0.096 | -0.0244 (0.0021) | 1.6E-30 | 0.0008 (0.0725) | 0.991 | 131.9 | -0.033 |
| Whole body fat-free mass | Vascular dementia (FinnGen) | rs2531991 | 16:4023553 | A/G | 0.747 | 0.0164 (0.0014) | 2.3E-30 | -0.0386 (0.0562) | 0.492 | 131.1 | -2.352 |
| Whole body fat-free mass | Vascular dementia (FinnGen) | rs6235 | 5:95728898 | G/C | 0.268 | 0.0159 (0.0014) | 8.3E-30 | -0.0366 (0.0545) | 0.502 | 128.6 | -2.307 |
| Whole body fat-free mass | Vascular dementia (FinnGen) | rs11014285 | 10:25178864 | A/G | 0.165 | 0.0192 (0.0017) | 1.1E-29 | -0.0681 (0.0814) | 0.403 | 128.1 | -3.554 |
| Whole body fat-free mass | Vascular dementia (FinnGen) | rs1022523 | 1:176788394 | A/G | 0.727 | 0.0156 (0.0014) | 4.4E-29 | -0.0911 (0.0538) | 0.090 | 125.3 | -5.853 |
| Whole body fat-free mass | Vascular dementia (FinnGen) | rs12072845 | 1:214630757 | A/G | 0.395 | -0.0140 (0.0013) | 2.8E-28 | -0.0092 (0.0516) | 0.859 | 121.7 | 0.659 |
| Whole body fat-free mass | Vascular dementia (FinnGen) | rs11880992 | 19:2176403 | A/G | 0.408 | 0.0139 (0.0013) | 4.6E-28 | -0.0097 (0.0509) | 0.849 | 120.6 | -0.697 |
| Whole body fat-free mass | Vascular dementia (FinnGen) | rs2270894 | 3:9975386 | G/C | 0.203 | -0.0174 (0.0016) | 1.0E-27 | -0.0381 (0.0560) | 0.497 | 119.0 | 2.184 |
| Whole body fat-free mass | Vascular dementia (FinnGen) | rs111640872 | 19:30290357 | C/G | 0.331 | 0.0145 (0.0013) | 1.1E-27 | 0.0865 (0.0516) | 0.094 | 118.9 | 5.977 |
| Whole body fat-free mass | Vascular dementia (FinnGen) | rs6800021 | 3:50190346 | A/G | 0.425 | 0.0136 (0.0013) | 1.6E-27 | 0.0370 (0.0527) | 0.483 | 118.1 | 2.713 |
| Whole body fat-free mass | Vascular dementia (FinnGen) | rs11065979 | 12:112059557 | T/C | 0.438 | -0.0135 (0.0013) | 4.3E-27 | 0.0004 (0.0505) | 0.994 | 116.2 | -0.030 |
| Whole body fat-free mass | Vascular dementia (FinnGen) | rs17277008 | 1:172105162 | C/T | 0.313 | 0.0144 (0.0013) | 4.6E-27 | 0.0593 (0.0558) | 0.289 | 116.1 | 4.130 |
| Whole body fat-free mass | Vascular dementia (FinnGen) | rs2900208 | 12:11878464 | A/C | 0.354 | 0.0140 (0.0013) | 4.9E-27 | -0.0436 (0.0504) | 0.387 | 115.9 | -3.118 |
| Whole body fat-free mass | Vascular dementia (FinnGen) | rs12657771 | 5:36787962 | A/G | 0.439 | -0.0134 (0.0013) | 9.4E-27 | 0.0014 (0.0498) | 0.978 | 114.7 | -0.104 |
| Whole body fat-free mass | Vascular dementia (FinnGen) | rs12879423 | 14:25927832 | G/A | 0.679 | 0.0142 (0.0013) | 3.4E-26 | -0.0296 (0.0509) | 0.560 | 112.1 | -2.087 |
| Whole body fat-free mass | Vascular dementia (FinnGen) | rs6762578 | 3:128992047 | A/G | 0.778 | 0.0157 (0.0015) | 8.9E-26 | 0.0357 (0.0653) | 0.585 | 110.2 | 2.270 |
| Whole body fat-free mass | Vascular dementia (FinnGen) | rs7141420 | 14:79899454 | T/C | 0.516 | 0.0131 (0.0013) | 1.2E-25 | -0.0385 (0.0498) | 0.440 | 109.5 | -2.935 |
| Whole body fat-free mass | Vascular dementia (FinnGen) | rs3822742 | 5:139059017 | A/C | 0.371 | 0.0134 (0.0013) | 1.6E-25 | -0.0107 (0.0524) | 0.838 | 109.0 | -0.796 |
| Whole body fat-free mass | Vascular dementia (FinnGen) | rs12041740 | 1:11236524 | A/G | 0.740 | -0.0147 (0.0014) | 2.1E-25 | 0.0514 (0.0550) | 0.350 | 108.5 | -3.504 |
| Whole body fat-free mass | Vascular dementia (FinnGen) | rs12095997 | 1:51391845 | T/C | 0.089 | 0.0225 (0.0022) | 2.7E-25 | -0.0476 (0.1061) | 0.654 | 108.0 | -2.114 |
| Whole body fat-free mass | Vascular dementia (FinnGen) | rs6570509 | 6:142716286 | T/G | 0.287 | -0.0142 (0.0014) | 3.9E-25 | 0.1316 (0.0561) | 0.019 | 107.3 | -9.289 |
| Whole body fat-free mass | Vascular dementia (FinnGen) | rs7080472 | 10:96012950 | T/G | 0.425 | 0.0130 (0.0013) | 5.2E-25 | -0.0465 (0.0525) | 0.375 | 106.7 | -3.575 |
| Whole body fat-free mass | Vascular dementia (FinnGen) | rs1924936 | 13:78443297 | A/T | 0.774 | 0.0153 (0.0015) | 7.8E-25 | -0.0415 (0.0636) | 0.513 | 105.9 | -2.708 |
| Whole body fat-free mass | Vascular dementia (FinnGen) | rs10283100 | 8:120596023 | G/A | 0.945 | 0.0279 (0.0027) | 8.7E-25 | 0.0807 (0.0961) | 0.401 | 105.7 | 2.892 |
| Whole body fat-free mass | Vascular dementia (FinnGen) | rs11240565 | 1:205722958 | T/C | 0.398 | 0.0130 (0.0013) | 8.8E-25 | -0.0639 (0.0512) | 0.212 | 105.6 | -4.926 |
| Whole body fat-free mass | Vascular dementia (FinnGen) | rs365352 | 5:77401152 | A/G | 0.244 | -0.0148 (0.0014) | 9.3E-25 | 0.0412 (0.0631) | 0.514 | 105.5 | -2.785 |
| Whole body fat-free mass | Vascular dementia (FinnGen) | rs9938120 | 16:19977733 | T/C | 0.144 | -0.0184 (0.0018) | 9.4E-25 | 0.0925 (0.0742) | 0.212 | 105.5 | -5.021 |
| Whole body fat-free mass | Vascular dementia (FinnGen) | rs9350850 | 6:81050236 | C/T | 0.079 | 0.0234 (0.0023) | 1.5E-24 | 0.0597 (0.0787) | 0.448 | 104.6 | 2.552 |
| Whole body fat-free mass | Vascular dementia (FinnGen) | rs946197 | 1:56602308 | C/G | 0.232 | 0.0150 (0.0015) | 1.6E-24 | 0.0431 (0.0582) | 0.460 | 104.5 | 2.872 |
| Whole body fat-free mass | Vascular dementia (FinnGen) | rs11613704 | 12:980925 | A/C | 0.200 | 0.0158 (0.0015) | 2.1E-24 | 0.1141 (0.0613) | 0.063 | 103.9 | 7.219 |
| Whole body fat-free mass | Vascular dementia (FinnGen) | rs12193797 | 6:41748352 | G/A | 0.133 | -0.0185 (0.0018) | 2.9E-24 | -0.0136 (0.0919) | 0.882 | 103.3 | 0.734 |
| Whole body fat-free mass | Vascular dementia (FinnGen) | rs2252720 | 20:21223663 | T/C | 0.674 | -0.0135 (0.0013) | 4.6E-24 | 0.0704 (0.0545) | 0.197 | 102.4 | -5.202 |
| Whole body fat-free mass | Vascular dementia (FinnGen) | rs10119967 | 9:99268421 | C/A | 0.204 | 0.0156 (0.0015) | 5.9E-24 | -0.0664 (0.0648) | 0.306 | 101.9 | -4.259 |
| Whole body fat-free mass | Vascular dementia (FinnGen) | rs2163832 | 19:10745764 | C/T | 0.660 | -0.0133 (0.0013) | 6.4E-24 | 0.0220 (0.0544) | 0.687 | 101.7 | -1.658 |
| Whole body fat-free mass | Vascular dementia (FinnGen) | rs676105 | 11:30443688 | C/T | 0.304 | 0.0136 (0.0014) | 7.8E-24 | -0.0818 (0.0536) | 0.127 | 101.3 | -6.002 |
| Whole body fat-free mass | Vascular dementia (FinnGen) | rs9480933 | 6:109671391 | G/C | 0.350 | -0.0130 (0.0013) | 9.3E-24 | 0.1560 (0.0505) | 0.002 | 101.0 | -11.956 |
| Whole body fat-free mass | Vascular dementia (FinnGen) | rs11648796 | 16:792190 | G/A | 0.232 | 0.0154 (0.0015) | 1.4E-23 | 0.0555 (0.0532) | 0.296 | 100.1 | 3.592 |
| Whole body fat-free mass | Vascular dementia (FinnGen) | rs2265309 | 10:104487871 | C/T | 0.517 | -0.0124 (0.0012) | 1.5E-23 | -0.0386 (0.0495) | 0.436 | 100.0 | 3.104 |
| Whole body fat-free mass | Vascular dementia (FinnGen) | rs13271368 | 8:126506140 | T/C | 0.237 | -0.0148 (0.0015) | 2.0E-23 | 0.0006 (0.0538) | 0.991 | 99.4 | -0.041 |
| Whole body fat-free mass | Vascular dementia (FinnGen) | rs7815955 | 8:130719567 | T/A | 0.203 | -0.0152 (0.0015) | 7.8E-23 | 0.0280 (0.0644) | 0.663 | 96.8 | -1.843 |
| Whole body fat-free mass | Vascular dementia (FinnGen) | rs6779752 | 3:85663849 | A/G | 0.638 | -0.0128 (0.0013) | 8.5E-23 | -0.0219 (0.0546) | 0.688 | 96.6 | 1.716 |
| Whole body fat-free mass | Vascular dementia (FinnGen) | rs111365325 | 5:170865229 | T/C | 0.231 | -0.0145 (0.0015) | 9.6E-23 | -0.0038 (0.0612) | 0.951 | 96.3 | 0.263 |
| Whole body fat-free mass | Vascular dementia (FinnGen) | rs33973388 | 18:46611842 | T/G | 0.435 | 0.0123 (0.0013) | 9.7E-23 | -0.0808 (0.0535) | 0.131 | 96.3 | -6.556 |
| Whole body fat-free mass | Vascular dementia (FinnGen) | rs11578046 | 1:23425139 | A/G | 0.327 | -0.0129 (0.0013) | 1.1E-22 | 0.0501 (0.0525) | 0.340 | 96.1 | -3.874 |
| Whole body fat-free mass | Vascular dementia (FinnGen) | rs67551338 | 12:3393100 | T/C | 0.061 | 0.0255 (0.0026) | 1.4E-22 | 0.1192 (0.1266) | 0.347 | 95.6 | 4.668 |
| Whole body fat-free mass | Vascular dementia (FinnGen) | rs3809570 | 15:67000117 | A/C | 0.243 | 0.0142 (0.0015) | 2.1E-22 | 0.0343 (0.0587) | 0.559 | 94.8 | 2.424 |
| Whole body fat-free mass | Vascular dementia (FinnGen) | rs16942324 | 15:89383854 | A/C | 0.027 | -0.0373 (0.0038) | 2.2E-22 | 0.4790 (0.2269) | 0.035 | 94.7 | -12.851 |
| Whole body fat-free mass | Vascular dementia (FinnGen) | rs34848742 | 4:123828042 | G/T | 0.788 | -0.0148 (0.0015) | 2.3E-22 | 0.0037 (0.0637) | 0.954 | 94.7 | -0.251 |
| Whole body fat-free mass | Vascular dementia (FinnGen) | rs13240065 | 7:73015369 | A/G | 0.128 | 0.0180 (0.0019) | 2.9E-22 | 0.0407 (0.0729) | 0.577 | 94.2 | 2.264 |
| Whole body fat-free mass | Vascular dementia (FinnGen) | rs73052033 | 3:185828465 | C/T | 0.185 | -0.0154 (0.0016) | 5.2E-22 | -0.0178 (0.0670) | 0.790 | 93.0 | 1.155 |
| Whole body fat-free mass | Vascular dementia (FinnGen) | rs7780752 | 7:93241640 | C/T | 0.360 | 0.0124 (0.0013) | 5.4E-22 | -0.0407 (0.0556) | 0.464 | 92.9 | -3.271 |
| Whole body fat-free mass | Vascular dementia (FinnGen) | rs9291926 | 5:67599656 | G/T | 0.532 | -0.0120 (0.0012) | 6.1E-22 | -0.0377 (0.0492) | 0.444 | 92.7 | 3.149 |
| Whole body fat-free mass | Vascular dementia (FinnGen) | rs2721938 | 8:116635611 | T/C | 0.600 | -0.0121 (0.0013) | 1.3E-21 | 0.0747 (0.0542) | 0.168 | 91.2 | -6.157 |
| Whole body fat-free mass | Vascular dementia (FinnGen) | rs13430869 | 2:218146818 | T/G | 0.742 | 0.0135 (0.0014) | 1.4E-21 | -0.0296 (0.0515) | 0.566 | 91.1 | -2.192 |
| Whole body fat-free mass | Vascular dementia (FinnGen) | rs62621197 | 19:8670147 | T/C | 0.037 | -0.0326 (0.0034) | 1.4E-21 | -0.1428 (0.1782) | 0.423 | 91.0 | 4.383 |
| Whole body fat-free mass | Vascular dementia (FinnGen) | rs10938397 | 4:45182527 | G/A | 0.434 | 0.0119 (0.0013) | 1.7E-21 | -0.0090 (0.0492) | 0.855 | 90.6 | -0.755 |
| Whole body fat-free mass | Vascular dementia (FinnGen) | rs10775348 | 16:88806348 | G/A | 0.704 | 0.0130 (0.0014) | 2.0E-21 | -0.0433 (0.0540) | 0.422 | 90.4 | -3.337 |
| Whole body fat-free mass | Vascular dementia (FinnGen) | rs11042725 | 11:10325325 | A/C | 0.488 | -0.0117 (0.0012) | 3.1E-21 | 0.0540 (0.0491) | 0.272 | 89.5 | -4.600 |
| Whole body fat-free mass | Vascular dementia (FinnGen) | rs10748128 | 12:69827658 | T/G | 0.345 | 0.0123 (0.0013) | 3.2E-21 | 0.0271 (0.0515) | 0.599 | 89.5 | 2.194 |
| Whole body fat-free mass | Vascular dementia (FinnGen) | rs7619139 | 3:25110415 | A/T | 0.589 | 0.0119 (0.0013) | 3.4E-21 | 0.0170 (0.0503) | 0.735 | 89.3 | 1.425 |
| Whole body fat-free mass | Vascular dementia (FinnGen) | rs247008 | 5:131447104 | G/A | 0.670 | 0.0125 (0.0013) | 3.6E-21 | -0.0829 (0.0503) | 0.100 | 89.2 | -6.615 |
| Whole body fat-free mass | Vascular dementia (FinnGen) | rs62621812 | 7:127015083 | A/G | 0.020 | 0.0425 (0.0045) | 5.9E-21 | 0.0922 (0.1237) | 0.456 | 88.2 | 2.168 |
| Whole body fat-free mass | Vascular dementia (FinnGen) | rs4968799 | 17:68158831 | T/A | 0.112 | -0.0185 (0.0020) | 7.3E-21 | -0.0591 (0.0838) | 0.481 | 87.8 | 3.203 |
| Whole body fat-free mass | Vascular dementia (FinnGen) | rs2726036 | 16:28347140 | C/A | 0.398 | 0.0119 (0.0013) | 8.0E-21 | -0.0486 (0.0495) | 0.327 | 87.6 | -4.082 |
| Whole body fat-free mass | Vascular dementia (FinnGen) | rs2952615 | 5:112138888 | C/G | 0.618 | -0.0119 (0.0013) | 8.9E-21 | -0.0005 (0.0501) | 0.993 | 87.4 | 0.042 |
| Whole body fat-free mass | Vascular dementia (FinnGen) | rs17246129 | 2:227259964 | A/G | 0.305 | 0.0125 (0.0013) | 1.4E-20 | -0.0466 (0.0549) | 0.396 | 86.5 | -3.722 |
| Whole body fat-free mass | Vascular dementia (FinnGen) | rs9461246 | 6:26381660 | A/G | 0.118 | -0.0178 (0.0019) | 1.5E-20 | 0.1081 (0.0692) | 0.118 | 86.3 | -6.078 |
| Whole body fat-free mass | Vascular dementia (FinnGen) | rs1064213 | 2:198950240 | A/G | 0.478 | 0.0115 (0.0012) | 1.8E-20 | 0.0366 (0.0505) | 0.469 | 86.0 | 3.188 |
| Whole body fat-free mass | Vascular dementia (FinnGen) | rs2015561 | 17:38593294 | G/A | 0.424 | -0.0117 (0.0013) | 1.9E-20 | 0.0039 (0.0497) | 0.938 | 85.9 | -0.335 |
| Whole body fat-free mass | Vascular dementia (FinnGen) | rs1542224 | 2:223963874 | C/T | 0.719 | 0.0128 (0.0014) | 2.0E-20 | -0.0057 (0.0560) | 0.919 | 85.8 | -0.447 |
| Whole body fat-free mass | Vascular dementia (FinnGen) | rs2678204 | 1:201800511 | G/T | 0.340 | 0.0121 (0.0013) | 2.4E-20 | -0.0774 (0.0538) | 0.150 | 85.4 | -6.412 |
| Whole body fat-free mass | Vascular dementia (FinnGen) | rs3212260 | 14:24804088 | T/A | 0.258 | 0.0131 (0.0014) | 3.6E-20 | 0.0071 (0.0589) | 0.904 | 84.6 | 0.542 |
| Whole body fat-free mass | Vascular dementia (FinnGen) | rs663344 | 9:98316331 | C/G | 0.181 | -0.0151 (0.0016) | 3.7E-20 | 0.0379 (0.0625) | 0.545 | 84.6 | -2.513 |
| Whole body fat-free mass | Vascular dementia (FinnGen) | rs6585827 | 10:124165615 | A/G | 0.471 | 0.0114 (0.0012) | 3.8E-20 | -0.0012 (0.0510) | 0.981 | 84.5 | -0.105 |
| Whole body fat-free mass | Vascular dementia (FinnGen) | rs1910252 | 8:49407362 | T/C | 0.168 | 0.0153 (0.0017) | 3.9E-20 | 0.0760 (0.0710) | 0.284 | 84.5 | 4.964 |
| Whole body fat-free mass | Vascular dementia (FinnGen) | rs703593 | 12:103064247 | G/A | 0.482 | -0.0114 (0.0012) | 4.1E-20 | 0.1425 (0.0497) | 0.004 | 84.4 | -12.503 |
| Whole body fat-free mass | Vascular dementia (FinnGen) | rs55831773 | 17:7559037 | T/C | 0.199 | -0.0146 (0.0016) | 4.1E-20 | -0.0574 (0.0696) | 0.409 | 84.4 | 3.938 |
| Whole body fat-free mass | Vascular dementia (FinnGen) | rs115179432 | 2:33348679 | G/A | 0.072 | -0.0221 (0.0024) | 4.5E-20 | 0.0451 (0.1044) | 0.666 | 84.2 | -2.042 |
| Whole body fat-free mass | Vascular dementia (FinnGen) | rs55727637 | 16:15145252 | T/C | 0.305 | -0.0123 (0.0013) | 6.1E-20 | 0.0009 (0.0534) | 0.987 | 83.6 | -0.073 |
| Whole body fat-free mass | Vascular dementia (FinnGen) | rs31211 | 5:134363145 | A/G | 0.251 | -0.0130 (0.0014) | 7.2E-20 | -0.0183 (0.0559) | 0.743 | 83.2 | 1.402 |
| Whole body fat-free mass | Vascular dementia (FinnGen) | rs2140046 | 2:169706079 | C/T | 0.364 | -0.0117 (0.0013) | 8.2E-20 | 0.0347 (0.0498) | 0.486 | 83.0 | -2.960 |
| Whole body fat-free mass | Vascular dementia (FinnGen) | rs11778491 | 8:120451362 | C/G | 0.252 | -0.0130 (0.0014) | 9.2E-20 | -0.0383 (0.0633) | 0.545 | 82.8 | 2.944 |
| Whole body fat-free mass | Vascular dementia (FinnGen) | rs3740591 | 10:70287303 | T/C | 0.445 | 0.0114 (0.0013) | 1.7E-19 | 0.0220 (0.0520) | 0.672 | 81.5 | 1.930 |
| Whole body fat-free mass | Vascular dementia (FinnGen) | rs4980067 | 10:81136129 | A/C | 0.505 | -0.0112 (0.0012) | 1.8E-19 | -0.0369 (0.0495) | 0.457 | 81.4 | 3.293 |
| Whole body fat-free mass | Vascular dementia (FinnGen) | rs1841738 | 4:88557753 | G/A | 0.518 | -0.0112 (0.0012) | 3.2E-19 | -0.0479 (0.0507) | 0.345 | 80.3 | 4.283 |
| Whole body fat-free mass | Vascular dementia (FinnGen) | rs10202845 | 2:42575820 | G/A | 0.113 | -0.0176 (0.0020) | 4.3E-19 | -0.1171 (0.0805) | 0.146 | 79.7 | 6.648 |
| Whole body fat-free mass | Vascular dementia (FinnGen) | rs11001399 | 10:77221744 | C/A | 0.559 | -0.0112 (0.0013) | 5.2E-19 | 0.0313 (0.0496) | 0.529 | 79.4 | -2.799 |
| Whole body fat-free mass | Vascular dementia (FinnGen) | rs843374 | 3:183997261 | T/A | 0.587 | -0.0112 (0.0013) | 5.4E-19 | -0.0842 (0.0502) | 0.093 | 79.3 | 7.508 |
| Whole body fat-free mass | Vascular dementia (FinnGen) | rs7706886 | 5:32722319 | A/G | 0.271 | 0.0126 (0.0014) | 6.2E-19 | 0.0975 (0.0576) | 0.091 | 79.0 | 7.730 |
| Whole body fat-free mass | Vascular dementia (FinnGen) | rs10803955 | 2:183228114 | G/A | 0.508 | -0.0110 (0.0012) | 6.4E-19 | -0.0790 (0.0491) | 0.107 | 79.0 | 7.197 |
| Whole body fat-free mass | Vascular dementia (FinnGen) | rs111391498 | 4:1341553 | G/A | 0.047 | -0.0258 (0.0029) | 6.7E-19 | -0.1224 (0.1436) | 0.394 | 78.8 | 4.737 |
| Whole body fat-free mass | Vascular dementia (FinnGen) | rs4865956 | 5:54882505 | A/T | 0.697 | -0.0120 (0.0014) | 7.3E-19 | 0.0382 (0.0548) | 0.486 | 78.7 | -3.183 |
| Whole body fat-free mass | Vascular dementia (FinnGen) | rs28547519 | 7:121964618 | C/T | 0.366 | 0.0114 (0.0013) | 8.5E-19 | -0.0012 (0.0505) | 0.981 | 78.4 | -0.105 |
| Whole body fat-free mass | Vascular dementia (FinnGen) | rs7647657 | 3:38573746 | G/A | 0.386 | -0.0113 (0.0013) | 9.1E-19 | -0.0189 (0.0501) | 0.706 | 78.2 | 1.673 |
| Whole body fat-free mass | Vascular dementia (FinnGen) | rs2602713 | 19:4910021 | C/A | 0.440 | 0.0111 (0.0013) | 1.2E-18 | -0.0010 (0.0512) | 0.984 | 77.6 | -0.090 |
| Whole body fat-free mass | Vascular dementia (FinnGen) | rs6874142 | 5:172753555 | G/T | 0.114 | 0.0182 (0.0021) | 1.4E-18 | -0.0483 (0.0858) | 0.573 | 77.5 | -2.655 |
| Whole body fat-free mass | Vascular dementia (FinnGen) | rs76364830 | 8:13372120 | A/G | 0.063 | -0.0225 (0.0026) | 2.4E-18 | 0.0855 (0.1345) | 0.525 | 76.4 | -3.805 |
| Whole body fat-free mass | Vascular dementia (FinnGen) | rs17556750 | 4:82155568 | A/C | 0.288 | 0.0119 (0.0014) | 3.7E-18 | 0.0057 (0.0512) | 0.912 | 75.5 | 0.479 |
| Whole body fat-free mass | Vascular dementia (FinnGen) | rs11712872 | 3:52835514 | A/G | 0.115 | 0.0168 (0.0019) | 4.3E-18 | 0.0630 (0.0855) | 0.461 | 75.2 | 3.749 |
| Whole body fat-free mass | Vascular dementia (FinnGen) | rs7728690 | 5:88411214 | T/C | 0.386 | -0.0110 (0.0013) | 5.0E-18 | -0.0154 (0.0528) | 0.770 | 74.9 | 1.398 |
| Whole body fat-free mass | Vascular dementia (FinnGen) | rs76513770 | 16:72505534 | C/T | 0.128 | -0.0161 (0.0019) | 5.1E-18 | 0.0605 (0.0659) | 0.358 | 74.8 | -3.760 |
| Whole body fat-free mass | Vascular dementia (FinnGen) | rs2281175 | 1:1665702 | C/T | 0.405 | 0.0114 (0.0013) | 6.3E-18 | 0.0176 (0.0502) | 0.726 | 74.4 | 1.539 |
| Whole body fat-free mass | Vascular dementia (FinnGen) | rs3116201 | 2:233074205 | A/G | 0.098 | -0.0180 (0.0021) | 7.1E-18 | -0.0217 (0.1121) | 0.846 | 74.2 | 1.208 |
| Whole body fat-free mass | Vascular dementia (FinnGen) | rs508347 | 7:28212824 | C/T | 0.703 | -0.0117 (0.0014) | 7.8E-18 | -0.0829 (0.0515) | 0.108 | 74.0 | 7.094 |
| Whole body fat-free mass | Vascular dementia (FinnGen) | rs10777860 | 12:97792792 | A/G | 0.528 | -0.0107 (0.0012) | 7.8E-18 | -0.0146 (0.0497) | 0.769 | 74.0 | 1.366 |
| Whole body fat-free mass | Vascular dementia (FinnGen) | rs744205 | 11:69929677 | A/G | 0.544 | 0.0107 (0.0012) | 1.0E-17 | 0.0236 (0.0494) | 0.632 | 73.5 | 2.203 |
| Whole body fat-free mass | Vascular dementia (FinnGen) | rs7134283 | 12:24071748 | A/G | 0.283 | -0.0118 (0.0014) | 1.2E-17 | -0.0387 (0.0548) | 0.480 | 73.2 | 3.276 |
| Whole body fat-free mass | Vascular dementia (FinnGen) | rs822549 | 7:148649180 | C/T | 0.267 | 0.0120 (0.0014) | 1.4E-17 | 0.0823 (0.0542) | 0.129 | 72.9 | 6.871 |
| Whole body fat-free mass | Vascular dementia (FinnGen) | rs61729527 | 8:77761919 | T/C | 0.052 | -0.0238 (0.0028) | 1.9E-17 | -0.0926 (0.1010) | 0.359 | 72.3 | 3.887 |
| Whole body fat-free mass | Vascular dementia (FinnGen) | rs968821 | 9:94187634 | C/G | 0.340 | -0.0111 (0.0013) | 2.1E-17 | 0.0181 (0.0558) | 0.746 | 72.1 | -1.626 |
| Whole body fat-free mass | Vascular dementia (FinnGen) | rs9317002 | 13:59175727 | A/C | 0.515 | 0.0106 (0.0013) | 2.3E-17 | 0.0248 (0.0492) | 0.614 | 71.8 | 2.334 |
| Whole body fat-free mass | Vascular dementia (FinnGen) | rs9951619 | 18:56882326 | G/T | 0.767 | 0.0125 (0.0015) | 4.0E-17 | 0.0576 (0.0527) | 0.274 | 70.8 | 4.619 |
| Whole body fat-free mass | Vascular dementia (FinnGen) | rs3782232 | 12:57116249 | A/G | 0.071 | -0.0203 (0.0024) | 4.4E-17 | 0.0690 (0.0910) | 0.448 | 70.6 | -3.400 |
| Whole body fat-free mass | Vascular dementia (FinnGen) | rs12188627 | 5:60720682 | G/A | 0.488 | -0.0104 (0.0012) | 6.3E-17 | -0.0107 (0.0492) | 0.828 | 69.9 | 1.032 |
| Whole body fat-free mass | Vascular dementia (FinnGen) | rs11689727 | 2:25458100 | A/C | 0.331 | -0.0110 (0.0013) | 7.0E-17 | -0.0422 (0.0528) | 0.425 | 69.7 | 3.840 |
| Whole body fat-free mass | Vascular dementia (FinnGen) | rs7312646 | 12:102388920 | C/A | 0.521 | -0.0105 (0.0013) | 9.2E-17 | -0.0493 (0.0494) | 0.319 | 69.1 | 4.687 |
| Whole body fat-free mass | Vascular dementia (FinnGen) | rs6902789 | 6:105358192 | A/G | 0.368 | 0.0107 (0.0013) | 1.1E-16 | -0.0046 (0.0513) | 0.928 | 68.8 | -0.430 |
| Whole body fat-free mass | Vascular dementia (FinnGen) | rs4858940 | 3:88254820 | C/T | 0.886 | 0.0161 (0.0019) | 1.2E-16 | 0.0579 (0.0819) | 0.480 | 68.6 | 3.591 |
| Whole body fat-free mass | Vascular dementia (FinnGen) | rs2296316 | 14:65520246 | C/T | 0.465 | -0.0104 (0.0013) | 1.2E-16 | -0.0443 (0.0492) | 0.368 | 68.6 | 4.258 |
| Whole body fat-free mass | Vascular dementia (FinnGen) | rs112560164 | 14:93112924 | A/G | 0.191 | 0.0131 (0.0016) | 1.3E-16 | 0.0054 (0.0712) | 0.939 | 68.5 | 0.411 |
| Whole body fat-free mass | Vascular dementia (FinnGen) | rs9380508 | 6:35398689 | T/C | 0.775 | 0.0123 (0.0015) | 1.4E-16 | 0.0366 (0.0759) | 0.630 | 68.3 | 2.984 |
| Whole body fat-free mass | Vascular dementia (FinnGen) | rs61992671 | 14:101531854 | G/A | 0.492 | -0.0107 (0.0013) | 1.4E-16 | -0.0203 (0.0506) | 0.688 | 68.2 | 1.889 |
| Whole body fat-free mass | Vascular dementia (FinnGen) | rs1228024 | 11:47951353 | A/C | 0.661 | -0.0108 (0.0013) | 2.1E-16 | -0.0360 (0.0531) | 0.498 | 67.5 | 3.337 |
| Whole body fat-free mass | Vascular dementia (FinnGen) | rs310796 | 12:77453226 | T/G | 0.681 | 0.0110 (0.0013) | 2.1E-16 | 0.0290 (0.0555) | 0.601 | 67.5 | 2.644 |
| Whole body fat-free mass | Vascular dementia (FinnGen) | rs11794152 | 9:23345347 | G/A | 0.415 | 0.0104 (0.0013) | 2.4E-16 | -0.0331 (0.0496) | 0.504 | 67.3 | -3.196 |
| Whole body fat-free mass | Vascular dementia (FinnGen) | rs1412234 | 9:28410683 | C/T | 0.327 | 0.0109 (0.0013) | 2.6E-16 | 0.0253 (0.0509) | 0.619 | 67.0 | 2.328 |
| Whole body fat-free mass | Vascular dementia (FinnGen) | rs55674305 | 8:89435868 | A/G | 0.306 | -0.0110 (0.0014) | 3.1E-16 | 0.0116 (0.0537) | 0.828 | 66.8 | -1.051 |
| Whole body fat-free mass | Vascular dementia (FinnGen) | rs578366 | 6:81591034 | G/A | 0.420 | -0.0102 (0.0013) | 3.5E-16 | -0.0349 (0.0496) | 0.481 | 66.5 | 3.416 |
| Whole body fat-free mass | Vascular dementia (FinnGen) | rs2197563 | 2:233687080 | A/G | 0.595 | 0.0103 (0.0013) | 3.8E-16 | -0.0358 (0.0553) | 0.517 | 66.3 | -3.484 |
| Whole body fat-free mass | Vascular dementia (FinnGen) | rs12820906 | 12:123493123 | G/A | 0.247 | 0.0117 (0.0014) | 3.9E-16 | -0.0458 (0.0608) | 0.452 | 66.3 | -3.904 |
| Whole body fat-free mass | Vascular dementia (FinnGen) | rs10899736 | 7:50546925 | A/G | 0.440 | -0.0102 (0.0013) | 4.4E-16 | 0.0165 (0.0504) | 0.743 | 66.1 | -1.623 |
| Whole body fat-free mass | Vascular dementia (FinnGen) | rs1049193 | 12:53873844 | C/G | 0.171 | -0.0134 (0.0016) | 4.6E-16 | 0.0739 (0.0779) | 0.343 | 66.0 | -5.516 |
| Whole body fat-free mass | Vascular dementia (FinnGen) | rs141729694 | 5:87999371 | T/C | 0.074 | 0.0193 (0.0024) | 4.7E-16 | -0.0079 (0.1247) | 0.949 | 65.9 | -0.409 |
| Whole body fat-free mass | Vascular dementia (FinnGen) | rs11855017 | 15:42096146 | A/C | 0.182 | 0.0131 (0.0016) | 4.8E-16 | 0.0326 (0.0732) | 0.655 | 65.9 | 2.487 |
| Whole body fat-free mass | Vascular dementia (FinnGen) | rs667515 | 11:69449076 | C/G | 0.386 | -0.0104 (0.0013) | 5.9E-16 | -0.0019 (0.0519) | 0.971 | 65.5 | 0.183 |
| Whole body fat-free mass | Vascular dementia (FinnGen) | rs357486 | 3:153885503 | C/T | 0.543 | 0.0100 (0.0012) | 6.7E-16 | -0.0064 (0.0497) | 0.898 | 65.2 | -0.638 |
| Whole body fat-free mass | Vascular dementia (FinnGen) | rs11245450 | 10:126658075 | A/G | 0.422 | -0.0103 (0.0013) | 7.0E-16 | 0.0035 (0.0496) | 0.944 | 65.2 | -0.341 |
| Whole body fat-free mass | Vascular dementia (FinnGen) | rs6984820 | 8:144993324 | T/C | 0.422 | -0.0101 (0.0013) | 8.5E-16 | -0.0443 (0.0501) | 0.377 | 64.7 | 4.368 |
| Whole body fat-free mass | Vascular dementia (FinnGen) | rs9513510 | 13:99571922 | C/G | 0.699 | -0.0109 (0.0014) | 1.0E-15 | -0.0721 (0.0525) | 0.170 | 64.4 | 6.625 |
| Whole body fat-free mass | Vascular dementia (FinnGen) | rs117543413 | 10:79543740 | T/C | 0.018 | -0.0382 (0.0048) | 1.2E-15 | 0.0312 (0.2039) | 0.878 | 64.0 | -0.816 |
| Whole body fat-free mass | Vascular dementia (FinnGen) | rs10172196 | 2:36780549 | A/G | 0.306 | 0.0108 (0.0013) | 1.3E-15 | -0.1374 (0.0522) | 0.008 | 63.9 | -12.768 |
| Whole body fat-free mass | Vascular dementia (FinnGen) | rs603321 | 6:140256669 | G/A | 0.245 | -0.0115 (0.0014) | 1.6E-15 | 0.0095 (0.0639) | 0.881 | 63.5 | -0.823 |
| Whole body fat-free mass | Vascular dementia (FinnGen) | rs12764498 | 10:5042294 | C/T | 0.118 | -0.0155 (0.0019) | 1.6E-15 | -0.0477 (0.0733) | 0.515 | 63.6 | 3.084 |
| Whole body fat-free mass | Vascular dementia (FinnGen) | rs7229520 | 18:46516468 | A/G | 0.662 | -0.0105 (0.0013) | 2.0E-15 | -0.0576 (0.0517) | 0.265 | 63.0 | 5.507 |
| Whole body fat-free mass | Vascular dementia (FinnGen) | rs1780673 | 20:35758359 | A/G | 0.811 | 0.0126 (0.0016) | 2.2E-15 | 0.0219 (0.0537) | 0.683 | 62.9 | 1.736 |
| Whole body fat-free mass | Vascular dementia (FinnGen) | rs10434434 | 4:54271611 | C/A | 0.148 | -0.0139 (0.0018) | 2.4E-15 | -0.0827 (0.0771) | 0.284 | 62.7 | 5.960 |
| Whole body fat-free mass | Vascular dementia (FinnGen) | rs1971955 | 12:122420437 | G/A | 0.854 | -0.0139 (0.0018) | 2.9E-15 | 0.1302 (0.0636) | 0.041 | 62.3 | -9.368 |
| Whole body fat-free mass | Vascular dementia (FinnGen) | rs5752989 | 22:30365780 | A/G | 0.571 | -0.0099 (0.0013) | 2.9E-15 | 0.0343 (0.0493) | 0.487 | 62.3 | -3.449 |
| Whole body fat-free mass | Vascular dementia (FinnGen) | rs34693680 | 3:98665549 | T/C | 0.132 | 0.0144 (0.0018) | 3.3E-15 | 0.0265 (0.0751) | 0.724 | 62.1 | 1.838 |
| Whole body fat-free mass | Vascular dementia (FinnGen) | rs12140153 | 1:62579891 | T/G | 0.094 | -0.0171 (0.0022) | 3.8E-15 | 0.0991 (0.0919) | 0.281 | 61.8 | -5.810 |
| Whole body fat-free mass | Vascular dementia (FinnGen) | rs35251247 | 11:43878459 | A/G | 0.292 | 0.0108 (0.0014) | 3.8E-15 | -0.0082 (0.0542) | 0.879 | 61.8 | -0.759 |
| Whole body fat-free mass | Vascular dementia (FinnGen) | rs6081869 | 20:20060745 | G/T | 0.386 | -0.0102 (0.0013) | 4.4E-15 | 0.0048 (0.0494) | 0.923 | 61.5 | -0.473 |
| Whole body fat-free mass | Vascular dementia (FinnGen) | rs74494415 | 18:74972138 | T/C | 0.040 | -0.0252 (0.0032) | 4.6E-15 | -0.0074 (0.0946) | 0.938 | 61.4 | 0.294 |
| Whole body fat-free mass | Vascular dementia (FinnGen) | rs11049684 | 12:28665359 | T/C | 0.299 | -0.0106 (0.0014) | 4.8E-15 | 0.1482 (0.0559) | 0.008 | 61.4 | -13.966 |
| Whole body fat-free mass | Vascular dementia (FinnGen) | rs10979612 | 9:111687379 | C/T | 0.073 | 0.0187 (0.0024) | 4.9E-15 | 0.0076 (0.0769) | 0.921 | 61.3 | 0.407 |
| Whole body fat-free mass | Vascular dementia (FinnGen) | rs147110934 | 19:55993436 | T/G | 0.024 | -0.0316 (0.0040) | 5.6E-15 | -0.5121 (0.3209) | 0.111 | 61.0 | 16.226 |
| Whole body fat-free mass | Vascular dementia (FinnGen) | rs798759 | 4:1730299 | G/A | 0.464 | -0.0097 (0.0012) | 6.7E-15 | -0.0235 (0.0514) | 0.648 | 60.7 | 2.433 |
| Whole body fat-free mass | Vascular dementia (FinnGen) | rs10780906 | 9:90841143 | A/G | 0.588 | -0.0099 (0.0013) | 8.4E-15 | -0.0067 (0.0496) | 0.892 | 60.2 | 0.678 |
| Whole body fat-free mass | Vascular dementia (FinnGen) | rs2287163 | 12:107349294 | T/C | 0.374 | -0.0099 (0.0013) | 8.4E-15 | 0.0192 (0.0520) | 0.711 | 60.2 | -1.933 |
| Whole body fat-free mass | Vascular dementia (FinnGen) | rs7301341 | 12:94083105 | C/T | 0.327 | -0.0103 (0.0013) | 9.3E-15 | -0.0596 (0.0518) | 0.249 | 60.0 | 5.811 |
| Whole body fat-free mass | Vascular dementia (FinnGen) | rs2104449 | 1:97082532 | T/G | 0.283 | 0.0107 (0.0014) | 9.4E-15 | 0.0173 (0.0638) | 0.786 | 60.0 | 1.622 |
| Whole body fat-free mass | Vascular dementia (FinnGen) | rs4974223 | 3:56602088 | T/C | 0.904 | 0.0163 (0.0021) | 1.0E-14 | -0.0228 (0.0769) | 0.767 | 59.9 | -1.403 |
| Whole body fat-free mass | Vascular dementia (FinnGen) | rs6142059 | 20:32544327 | C/T | 0.493 | 0.0096 (0.0012) | 1.1E-14 | -0.0511 (0.0492) | 0.299 | 59.8 | -5.302 |
| Whole body fat-free mass | Vascular dementia (FinnGen) | rs2615075 | 1:225934295 | G/A | 0.376 | 0.0098 (0.0013) | 2.0E-14 | -0.0975 (0.0509) | 0.055 | 58.5 | -9.942 |
| Whole body fat-free mass | Vascular dementia (FinnGen) | rs9379084 | 6:7231843 | A/G | 0.116 | -0.0152 (0.0020) | 2.7E-14 | -0.0044 (0.0791) | 0.956 | 57.9 | 0.290 |
| Whole body fat-free mass | Vascular dementia (FinnGen) | rs11785562 | 8:23391493 | A/G | 0.201 | 0.0119 (0.0016) | 2.7E-14 | 0.0015 (0.0566) | 0.979 | 58.0 | 0.126 |
| Whole body fat-free mass | Vascular dementia (FinnGen) | rs5742915 | 15:74336633 | C/T | 0.460 | 0.0095 (0.0012) | 3.2E-14 | -0.0265 (0.0502) | 0.598 | 57.6 | -2.796 |
| Whole body fat-free mass | Vascular dementia (FinnGen) | rs1374370 | 2:85818273 | A/G | 0.305 | 0.0102 (0.0013) | 3.5E-14 | -0.0307 (0.0581) | 0.596 | 57.5 | -3.014 |
| Whole body fat-free mass | Vascular dementia (FinnGen) | rs11611651 | 12:133380790 | A/G | 0.088 | 0.0166 (0.0022) | 4.3E-14 | -0.1076 (0.1158) | 0.353 | 57.0 | -6.500 |
| Whole body fat-free mass | Vascular dementia (FinnGen) | rs981002 | 4:12881731 | A/T | 0.267 | -0.0106 (0.0014) | 5.3E-14 | 0.0537 (0.0573) | 0.348 | 56.6 | -5.089 |
| Whole body fat-free mass | Vascular dementia (FinnGen) | rs2568958 | 1:72765116 | A/G | 0.604 | 0.0095 (0.0013) | 5.5E-14 | 0.1061 (0.0513) | 0.039 | 56.5 | 11.176 |
| Whole body fat-free mass | Vascular dementia (FinnGen) | rs12375196 | 7:103416541 | A/C | 0.424 | 0.0095 (0.0013) | 6.5E-14 | 0.1043 (0.0492) | 0.034 | 56.2 | 11.014 |
| Whole body fat-free mass | Vascular dementia (FinnGen) | rs6026578 | 20:57463472 | G/C | 0.625 | -0.0097 (0.0013) | 6.8E-14 | 0.0344 (0.0514) | 0.503 | 56.1 | -3.555 |
| Whole body fat-free mass | Vascular dementia (FinnGen) | rs58857770 | 19:7208110 | G/C | 0.408 | -0.0095 (0.0013) | 7.6E-14 | 0.0495 (0.0489) | 0.312 | 55.9 | -5.197 |
| Whole body fat-free mass | Vascular dementia (FinnGen) | rs9960619 | 18:33040095 | T/C | 0.344 | 0.0098 (0.0013) | 8.7E-14 | 0.0631 (0.0525) | 0.229 | 55.6 | 6.455 |
| Whole body fat-free mass | Vascular dementia (FinnGen) | rs13272451 | 8:8886183 | T/G | 0.439 | 0.0093 (0.0013) | 8.8E-14 | 0.1250 (0.0829) | 0.132 | 55.6 | 13.374 |
| Whole body fat-free mass | Vascular dementia (FinnGen) | rs11217863 | 11:120293138 | A/G | 0.116 | -0.0145 (0.0019) | 9.0E-14 | 0.0675 (0.0742) | 0.363 | 55.6 | -4.665 |
| Whole body fat-free mass | Vascular dementia (FinnGen) | rs62621400 | 15:101718239 | G/C | 0.058 | -0.0199 (0.0027) | 9.8E-14 | -0.0033 (0.1040) | 0.975 | 55.4 | 0.166 |
| Whole body fat-free mass | Vascular dementia (FinnGen) | rs3925 | 8:38281658 | A/G | 0.245 | -0.0107 (0.0014) | 1.2E-13 | -0.0217 (0.0636) | 0.733 | 55.0 | 2.023 |
| Whole body fat-free mass | Vascular dementia (FinnGen) | rs12070699 | 1:190230408 | T/C | 0.563 | -0.0092 (0.0012) | 1.5E-13 | -0.0121 (0.0492) | 0.806 | 54.5 | 1.315 |
| Whole body fat-free mass | Vascular dementia (FinnGen) | rs35756741 | 12:12868701 | T/C | 0.092 | -0.0159 (0.0022) | 1.6E-13 | -0.1247 (0.0811) | 0.124 | 54.4 | 7.846 |
| Whole body fat-free mass | Vascular dementia (FinnGen) | rs35492502 | 1:217806224 | A/G | 0.296 | 0.0100 (0.0014) | 2.1E-13 | 0.0391 (0.0524) | 0.455 | 53.9 | 3.925 |
| Whole body fat-free mass | Vascular dementia (FinnGen) | rs284662 | 19:41932275 | C/T | 0.620 | -0.0094 (0.0013) | 2.2E-13 | -0.0288 (0.0514) | 0.575 | 53.8 | 3.064 |
| Whole body fat-free mass | Vascular dementia (FinnGen) | rs36695 | 5:127549187 | A/G | 0.504 | -0.0091 (0.0012) | 2.3E-13 | -0.0035 (0.0498) | 0.944 | 53.7 | 0.385 |
| Whole body fat-free mass | Vascular dementia (FinnGen) | rs4926542 | 1:50263773 | T/C | 0.682 | -0.0098 (0.0013) | 2.4E-13 | -0.0273 (0.0512) | 0.594 | 53.7 | 2.787 |
| Whole body fat-free mass | Vascular dementia (FinnGen) | rs1805165 | 2:88874891 | A/C | 0.721 | -0.0101 (0.0014) | 2.5E-13 | 0.0724 (0.0519) | 0.163 | 53.6 | -7.186 |
| Whole body fat-free mass | Vascular dementia (FinnGen) | rs12209223 | 6:76164589 | A/C | 0.101 | 0.0151 (0.0021) | 2.6E-13 | -0.0042 (0.0722) | 0.953 | 53.5 | -0.279 |
| Whole body fat-free mass | Vascular dementia (FinnGen) | rs9925273 | 16:375900 | G/A | 0.181 | -0.0118 (0.0016) | 2.7E-13 | 0.0153 (0.0672) | 0.820 | 53.4 | -1.295 |
| Whole body fat-free mass | Vascular dementia (FinnGen) | rs55758152 | 5:171317318 | A/G | 0.326 | 0.0098 (0.0013) | 2.8E-13 | 0.0777 (0.0523) | 0.138 | 53.3 | 7.952 |
| Whole body fat-free mass | Vascular dementia (FinnGen) | rs2578557 | 5:5473063 | T/C | 0.642 | -0.0095 (0.0013) | 2.9E-13 | 0.0645 (0.0499) | 0.197 | 53.3 | -6.823 |
| Whole body fat-free mass | Vascular dementia (FinnGen) | rs2970592 | 1:212220847 | A/G | 0.588 | 0.0092 (0.0013) | 3.0E-13 | -0.1016 (0.0510) | 0.046 | 53.2 | -11.101 |
| Whole body fat-free mass | Vascular dementia (FinnGen) | rs74048171 | 11:2093603 | A/C | 0.258 | -0.0104 (0.0014) | 3.2E-13 | -0.0607 (0.0593) | 0.306 | 53.1 | 5.862 |
| Whole body fat-free mass | Vascular dementia (FinnGen) | rs57126421 | 18:2656989 | G/A | 0.238 | -0.0107 (0.0015) | 3.8E-13 | 0.0374 (0.0584) | 0.522 | 52.8 | -3.499 |
| Whole body fat-free mass | Vascular dementia (FinnGen) | rs1521624 | 15:79412399 | A/C | 0.471 | -0.0091 (0.0013) | 4.0E-13 | -0.0079 (0.0493) | 0.873 | 52.7 | 0.869 |
| Whole body fat-free mass | Vascular dementia (FinnGen) | rs11065015 | 12:120520863 | T/C | 0.027 | -0.0282 (0.0039) | 4.1E-13 | -0.0161 (0.1128) | 0.886 | 52.6 | 0.571 |
| Whole body fat-free mass | Vascular dementia (FinnGen) | rs11545482 | 2:70315987 | T/C | 0.020 | -0.0316 (0.0044) | 4.4E-13 | 0.6646 (0.3435) | 0.053 | 52.5 | -21.018 |
| Whole body fat-free mass | Vascular dementia (FinnGen) | rs73601548 | 10:18549889 | T/C | 0.115 | 0.0142 (0.0020) | 4.8E-13 | -0.0657 (0.0985) | 0.505 | 52.3 | -4.640 |
| Whole body fat-free mass | Vascular dementia (FinnGen) | rs55800172 | 1:32414767 | A/G | 0.064 | 0.0182 (0.0025) | 5.7E-13 | -0.0588 (0.1288) | 0.648 | 52.0 | -3.227 |
| Whole body fat-free mass | Vascular dementia (FinnGen) | rs11071182 | 15:55644676 | G/A | 0.870 | 0.0134 (0.0019) | 6.3E-13 | -0.0416 (0.0777) | 0.592 | 51.7 | -3.109 |
| Whole body fat-free mass | Vascular dementia (FinnGen) | rs11160601 | 14:101186641 | T/C | 0.092 | 0.0156 (0.0022) | 6.5E-13 | -0.0354 (0.0685) | 0.605 | 51.7 | -2.274 |
| Whole body fat-free mass | Vascular dementia (FinnGen) | rs3783256 | 13:30107388 | C/T | 0.648 | -0.0094 (0.0013) | 6.7E-13 | 0.0200 (0.0496) | 0.686 | 51.6 | -2.128 |
| Whole body fat-free mass | Vascular dementia (FinnGen) | rs17828687 | 8:73454767 | A/C | 0.573 | -0.0091 (0.0013) | 6.8E-13 | 0.0006 (0.0500) | 0.990 | 51.6 | -0.066 |
| Whole body fat-free mass | Vascular dementia (FinnGen) | rs71393968 | 16:67847341 | A/G | 0.046 | 0.0214 (0.0030) | 6.9E-13 | -0.0418 (0.1226) | 0.734 | 51.6 | -1.956 |
| Whole body fat-free mass | Vascular dementia (FinnGen) | rs11709402 | 3:131551027 | G/A | 0.279 | 0.0100 (0.0014) | 7.1E-13 | 0.0232 (0.0558) | 0.677 | 51.5 | 2.330 |
| Whole body fat-free mass | Vascular dementia (FinnGen) | rs4795318 | 17:36949053 | T/C | 0.520 | 0.0089 (0.0012) | 7.1E-13 | -0.0166 (0.0494) | 0.738 | 51.5 | -1.858 |
| Whole body fat-free mass | Vascular dementia (FinnGen) | rs73013411 | 6:164126233 | A/C | 0.131 | 0.0131 (0.0018) | 8.9E-13 | -0.0393 (0.0898) | 0.661 | 51.1 | -2.993 |
| Whole body fat-free mass | Vascular dementia (FinnGen) | rs16916881 | 8:95566270 | A/C | 0.235 | -0.0104 (0.0015) | 9.2E-13 | -0.0772 (0.0512) | 0.132 | 51.0 | 7.394 |
| Whole body fat-free mass | Vascular dementia (FinnGen) | rs10870597 | 13:114999636 | G/A | 0.235 | -0.0105 (0.0015) | 1.0E-12 | 0.0152 (0.0577) | 0.792 | 50.8 | -1.454 |
| Whole body fat-free mass | Vascular dementia (FinnGen) | rs6719296 | 2:103101108 | A/G | 0.495 | 0.0089 (0.0012) | 1.1E-12 | -0.0248 (0.0511) | 0.627 | 50.6 | -2.802 |
| Whole body fat-free mass | Vascular dementia (FinnGen) | rs10945541 | 6:158740218 | G/A | 0.665 | -0.0093 (0.0013) | 1.1E-12 | 0.0193 (0.0505) | 0.702 | 50.7 | -2.065 |
| Whole body fat-free mass | Vascular dementia (FinnGen) | rs60804050 | 1:118870373 | A/G | 0.256 | -0.0100 (0.0014) | 1.5E-12 | 0.0874 (0.0574) | 0.128 | 50.1 | -8.723 |
| Whole body fat-free mass | Vascular dementia (FinnGen) | rs9540493 | 13:66205704 | G/A | 0.545 | -0.0089 (0.0013) | 1.5E-12 | -0.0072 (0.0495) | 0.884 | 50.1 | 0.810 |
| Whole body fat-free mass | Vascular dementia (FinnGen) | rs7245985 | 19:30710410 | G/T | 0.208 | -0.0109 (0.0015) | 1.5E-12 | 0.1336 (0.0625) | 0.032 | 50.1 | -12.282 |
| Whole body fat-free mass | Vascular dementia (FinnGen) | rs73619441 | 20:61564901 | G/T | 0.144 | -0.0126 (0.0018) | 1.5E-12 | -0.1825 (0.0783) | 0.020 | 50.1 | 14.534 |
| Whole body fat-free mass | Vascular dementia (FinnGen) | rs2789366 | 1:235508270 | A/G | 0.346 | -0.0092 (0.0013) | 1.6E-12 | -0.0393 (0.0509) | 0.440 | 50.0 | 4.277 |
| Whole body fat-free mass | Vascular dementia (FinnGen) | rs16844418 | 4:3471754 | C/T | 0.143 | -0.0125 (0.0018) | 1.9E-12 | -0.0208 (0.0749) | 0.781 | 49.6 | 1.666 |
| Whole body fat-free mass | Vascular dementia (FinnGen) | rs10222924 | 4:49016761 | G/A | 0.699 | -0.0095 (0.0014) | 2.0E-12 | -0.0147 (0.0566) | 0.795 | 49.5 | 1.540 |
| Whole body fat-free mass | Vascular dementia (FinnGen) | rs7192870 | 16:14397436 | C/T | 0.469 | -0.0089 (0.0013) | 2.0E-12 | -0.0096 (0.0505) | 0.849 | 49.4 | 1.084 |
| Whole body fat-free mass | Vascular dementia (FinnGen) | rs11052457 | 12:33268956 | T/A | 0.038 | 0.0228 (0.0032) | 2.1E-12 | 0.0531 (0.1307) | 0.685 | 49.4 | 2.330 |
| Whole body fat-free mass | Vascular dementia (FinnGen) | rs892511 | 1:110078255 | G/C | 0.028 | 0.0263 (0.0038) | 3.7E-12 | 0.0620 (0.1134) | 0.585 | 48.3 | 2.354 |
| Whole body fat-free mass | Vascular dementia (FinnGen) | rs17400325 | 2:178565913 | C/T | 0.042 | 0.0215 (0.0031) | 3.8E-12 | -0.0648 (0.1708) | 0.705 | 48.2 | -3.012 |
| Whole body fat-free mass | Vascular dementia (FinnGen) | rs9394166 | 6:33751122 | C/G | 0.380 | 0.0089 (0.0013) | 3.8E-12 | -0.0656 (0.0502) | 0.192 | 48.2 | -7.395 |
| Whole body fat-free mass | Vascular dementia (FinnGen) | rs338361 | 15:68171116 | C/T | 0.706 | 0.0095 (0.0014) | 3.9E-12 | -0.0338 (0.0583) | 0.562 | 48.1 | -3.560 |
| Whole body fat-free mass | Vascular dementia (FinnGen) | rs115461836 | 6:19078931 | C/T | 0.046 | 0.0208 (0.0030) | 4.2E-12 | 0.1840 (0.1470) | 0.211 | 48.0 | 8.829 |
| Whole body fat-free mass | Vascular dementia (FinnGen) | rs1296328 | 4:137083193 | C/A | 0.559 | -0.0087 (0.0013) | 4.5E-12 | 0.0685 (0.0492) | 0.164 | 47.9 | -7.891 |
| Whole body fat-free mass | Vascular dementia (FinnGen) | rs4132132 | 4:31001301 | C/T | 0.444 | 0.0087 (0.0013) | 4.7E-12 | -0.0688 (0.0502) | 0.171 | 47.8 | -7.899 |
| Whole body fat-free mass | Vascular dementia (FinnGen) | rs9596810 | 13:54056553 | T/C | 0.435 | -0.0089 (0.0013) | 4.7E-12 | -0.0292 (0.0503) | 0.562 | 47.8 | 3.265 |
| Whole body fat-free mass | Vascular dementia (FinnGen) | rs1927635 | 9:16461905 | C/T | 0.354 | 0.0091 (0.0013) | 5.0E-12 | 0.0425 (0.0496) | 0.391 | 47.7 | 4.684 |
| Whole body fat-free mass | Vascular dementia (FinnGen) | rs1218826 | 13:27995760 | G/A | 0.666 | 0.0091 (0.0013) | 5.0E-12 | -0.0353 (0.0526) | 0.502 | 47.7 | -3.862 |
| Whole body fat-free mass | Vascular dementia (FinnGen) | rs17363646 | 1:86823503 | G/A | 0.136 | 0.0124 (0.0018) | 5.5E-12 | -0.0697 (0.0567) | 0.219 | 47.5 | -5.607 |
| Whole body fat-free mass | Vascular dementia (FinnGen) | rs17443541 | 2:200402624 | C/T | 0.189 | -0.0110 (0.0016) | 5.8E-12 | -0.1192 (0.0659) | 0.070 | 47.4 | 10.855 |
| Whole body fat-free mass | Vascular dementia (FinnGen) | rs12889702 | 14:42894143 | C/A | 0.313 | 0.0092 (0.0013) | 6.4E-12 | -0.0465 (0.0538) | 0.388 | 47.2 | -5.035 |
| Whole body fat-free mass | Vascular dementia (FinnGen) | rs254963 | 5:171224403 | G/A | 0.447 | -0.0086 (0.0013) | 6.6E-12 | 0.0185 (0.0493) | 0.708 | 47.1 | -2.145 |
| Whole body fat-free mass | Vascular dementia (FinnGen) | rs1813212 | 11:89301382 | G/A | 0.446 | -0.0086 (0.0013) | 6.7E-12 | -0.0434 (0.0496) | 0.381 | 47.1 | 5.051 |
| Whole body fat-free mass | Vascular dementia (FinnGen) | rs7188009 | 16:81660642 | A/G | 0.404 | 0.0087 (0.0013) | 7.0E-12 | -0.0002 (0.0559) | 0.997 | 47.0 | -0.023 |
| Whole body fat-free mass | Vascular dementia (FinnGen) | rs774214 | 12:56918566 | C/T | 0.663 | -0.0090 (0.0013) | 7.2E-12 | -0.0464 (0.0515) | 0.367 | 47.0 | 5.160 |
| Whole body fat-free mass | Vascular dementia (FinnGen) | rs6972291 | 7:55802063 | C/T | 0.176 | -0.0112 (0.0016) | 7.7E-12 | 0.0795 (0.0565) | 0.160 | 46.8 | -7.115 |
| Whole body fat-free mass | Vascular dementia (FinnGen) | rs7731023 | 5:36181627 | G/A | 0.574 | 0.0086 (0.0013) | 7.8E-12 | 0.0880 (0.0513) | 0.087 | 46.8 | 10.266 |
| Whole body fat-free mass | Vascular dementia (FinnGen) | rs12981554 | 19:3416668 | G/A | 0.542 | -0.0086 (0.0013) | 8.9E-12 | 0.0499 (0.0498) | 0.316 | 46.6 | -5.811 |
| Whole body fat-free mass | Vascular dementia (FinnGen) | rs4648626 | 1:2200390 | A/C | 0.457 | 0.0085 (0.0012) | 9.1E-12 | -0.0511 (0.0497) | 0.305 | 46.5 | -6.023 |
| Whole body fat-free mass | Vascular dementia (FinnGen) | rs35874463 | 15:67457698 | G/A | 0.058 | 0.0182 (0.0027) | 9.7E-12 | 0.2087 (0.1291) | 0.106 | 46.4 | 11.494 |
| Whole body fat-free mass | Vascular dementia (FinnGen) | rs10995366 | 10:52772113 | A/G | 0.251 | -0.0098 (0.0014) | 1.1E-11 | 0.0710 (0.0557) | 0.202 | 46.1 | -7.265 |
| Whole body fat-free mass | Vascular dementia (FinnGen) | rs77344209 | 3:55515277 | T/C | 0.252 | 0.0098 (0.0014) | 1.2E-11 | -0.0271 (0.0628) | 0.666 | 46.0 | -2.765 |
| Whole body fat-free mass | Vascular dementia (FinnGen) | rs11593630 | 10:120491506 | T/C | 0.353 | -0.0088 (0.0013) | 1.2E-11 | 0.0025 (0.0512) | 0.961 | 46.0 | -0.284 |
| Whole body fat-free mass | Vascular dementia (FinnGen) | rs1418433 | 6:44752568 | A/G | 0.588 | -0.0085 (0.0013) | 1.3E-11 | -0.0033 (0.0498) | 0.947 | 45.8 | 0.388 |
| Whole body fat-free mass | Vascular dementia (FinnGen) | rs7912286 | 10:114693230 | G/A | 0.602 | -0.0087 (0.0013) | 1.3E-11 | 0.0779 (0.0503) | 0.122 | 45.8 | -8.986 |
| Whole body fat-free mass | Vascular dementia (FinnGen) | rs7321045 | 13:97034410 | A/G | 0.449 | 0.0085 (0.0013) | 1.3E-11 | -0.0618 (0.0495) | 0.212 | 45.8 | -7.246 |
| Whole body fat-free mass | Vascular dementia (FinnGen) | rs73004967 | 19:19717056 | G/A | 0.069 | -0.0167 (0.0025) | 1.3E-11 | -0.0218 (0.1042) | 0.834 | 45.8 | 1.302 |
| Whole body fat-free mass | Vascular dementia (FinnGen) | rs71423263 | 2:144024781 | G/T | 0.141 | 0.0120 (0.0018) | 1.5E-11 | 0.0522 (0.0791) | 0.509 | 45.5 | 4.353 |
| Whole body fat-free mass | Vascular dementia (FinnGen) | rs73213484 | 4:28489339 | T/A | 0.141 | -0.0120 (0.0018) | 1.5E-11 | 0.0795 (0.0698) | 0.255 | 45.5 | -6.618 |
| Whole body fat-free mass | Vascular dementia (FinnGen) | rs6085658 | 20:6685377 | T/C | 0.403 | -0.0086 (0.0013) | 1.6E-11 | 0.0300 (0.0496) | 0.545 | 45.4 | -3.488 |
| Whole body fat-free mass | Vascular dementia (FinnGen) | rs2122823 | 7:25939161 | T/C | 0.211 | 0.0103 (0.0015) | 2.0E-11 | 0.0008 (0.0574) | 0.989 | 45.0 | 0.077 |
| Whole body fat-free mass | Vascular dementia (FinnGen) | rs12427047 | 12:90213070 | T/C | 0.243 | -0.0097 (0.0014) | 2.1E-11 | -0.0385 (0.0517) | 0.457 | 44.9 | 3.973 |
| Whole body fat-free mass | Vascular dementia (FinnGen) | rs112957890 | 14:36220876 | G/A | 0.265 | 0.0095 (0.0014) | 2.1E-11 | -0.0330 (0.0588) | 0.575 | 44.8 | -3.461 |
| Whole body fat-free mass | Vascular dementia (FinnGen) | rs12729817 | 1:218971896 | G/A | 0.474 | -0.0083 (0.0012) | 2.2E-11 | -0.0175 (0.0490) | 0.721 | 44.8 | 2.114 |
| Whole body fat-free mass | Vascular dementia (FinnGen) | rs4268495 | 11:28645341 | C/G | 0.374 | -0.0086 (0.0013) | 2.2E-11 | 0.0531 (0.0509) | 0.296 | 44.8 | -6.189 |
| Whole body fat-free mass | Vascular dementia (FinnGen) | rs114949263 | 7:150498245 | C/T | 0.112 | -0.0132 (0.0020) | 2.5E-11 | -0.0357 (0.0930) | 0.701 | 44.5 | 2.707 |
| Whole body fat-free mass | Vascular dementia (FinnGen) | rs505575 | 5:111256812 | C/T | 0.673 | -0.0088 (0.0013) | 2.6E-11 | -0.0250 (0.0506) | 0.622 | 44.4 | 2.832 |
| Whole body fat-free mass | Vascular dementia (FinnGen) | rs114177791 | 17:78555512 | T/G | 0.214 | 0.0101 (0.0015) | 2.7E-11 | 0.1960 (0.0619) | 0.002 | 44.4 | 19.385 |
| Whole body fat-free mass | Vascular dementia (FinnGen) | rs61878760 | 11:12807189 | A/G | 0.083 | 0.0151 (0.0023) | 2.8E-11 | -0.0259 (0.1379) | 0.851 | 44.3 | -1.719 |
| Whole body fat-free mass | Vascular dementia (FinnGen) | rs3845344 | 1:75001480 | T/C | 0.391 | 0.0084 (0.0013) | 3.1E-11 | 0.1113 (0.0496) | 0.025 | 44.1 | 13.225 |
| Whole body fat-free mass | Vascular dementia (FinnGen) | rs9327336 | 5:123990270 | C/T | 0.343 | 0.0087 (0.0013) | 3.2E-11 | 0.0172 (0.0496) | 0.728 | 44.1 | 1.985 |
| Whole body fat-free mass | Vascular dementia (FinnGen) | rs2663125 | 15:99563891 | C/T | 0.691 | -0.0089 (0.0013) | 3.2E-11 | -0.0040 (0.0582) | 0.945 | 44.0 | 0.448 |
| Whole body fat-free mass | Vascular dementia (FinnGen) | rs67560975 | 17:47142930 | C/T | 0.116 | 0.0129 (0.0019) | 3.3E-11 | -0.0343 (0.0739) | 0.642 | 44.0 | -2.658 |
| Whole body fat-free mass | Vascular dementia (FinnGen) | rs1056747 | 9:35690102 | G/A | 0.412 | -0.0084 (0.0013) | 3.4E-11 | 0.0475 (0.0500) | 0.343 | 43.9 | -5.681 |
| Whole body fat-free mass | Vascular dementia (FinnGen) | rs17197114 | 14:21894526 | C/T | 0.177 | 0.0109 (0.0016) | 3.4E-11 | -0.1581 (0.0688) | 0.022 | 43.9 | -14.537 |
| Whole body fat-free mass | Vascular dementia (FinnGen) | rs12694042 | 2:207029825 | T/C | 0.502 | -0.0082 (0.0012) | 3.8E-11 | 0.0452 (0.0503) | 0.369 | 43.7 | -5.502 |
| Whole body fat-free mass | Vascular dementia (FinnGen) | rs4912905 | 5:142730376 | C/G | 0.249 | -0.0095 (0.0014) | 3.8E-11 | 0.1017 (0.0568) | 0.073 | 43.7 | -10.681 |
| Whole body fat-free mass | Vascular dementia (FinnGen) | rs77848106 | 1:107971673 | A/C | 0.296 | -0.0090 (0.0014) | 3.9E-11 | -0.0534 (0.0496) | 0.282 | 43.7 | 5.917 |
| Whole body fat-free mass | Vascular dementia (FinnGen) | rs4504126 | 3:33600582 | C/A | 0.027 | 0.0250 (0.0038) | 4.3E-11 | -0.0636 (0.1930) | 0.742 | 43.5 | -2.540 |
| Whole body fat-free mass | Vascular dementia (FinnGen) | rs4439140 | 8:23113908 | A/G | 0.418 | -0.0085 (0.0013) | 4.7E-11 | 0.0473 (0.0505) | 0.349 | 43.3 | -5.568 |
| Whole body fat-free mass | Vascular dementia (FinnGen) | rs10775406 | 17:46197755 | G/A | 0.760 | -0.0096 (0.0015) | 4.7E-11 | 0.0632 (0.0562) | 0.261 | 43.3 | -6.605 |
| Whole body fat-free mass | Vascular dementia (FinnGen) | rs6665399 | 1:21553933 | C/T | 0.445 | 0.0082 (0.0012) | 4.9E-11 | -0.0147 (0.0493) | 0.766 | 43.2 | -1.801 |
| Whole body fat-free mass | Vascular dementia (FinnGen) | rs3110496 | 17:27917771 | G/A | 0.686 | 0.0088 (0.0013) | 5.0E-11 | -0.0343 (0.0507) | 0.498 | 43.2 | -3.899 |
| Whole body fat-free mass | Vascular dementia (FinnGen) | rs10950207 | 7:70105168 | T/C | 0.384 | 0.0084 (0.0013) | 5.8E-11 | -0.1007 (0.0518) | 0.052 | 42.9 | -12.032 |
| Whole body fat-free mass | Vascular dementia (FinnGen) | rs9788443 | 14:23878279 | C/T | 0.048 | 0.0191 (0.0029) | 5.8E-11 | -0.3745 (0.2086) | 0.073 | 42.9 | -19.607 |
| Whole body fat-free mass | Vascular dementia (FinnGen) | rs4525525 | 17:1866892 | T/G | 0.264 | -0.0094 (0.0014) | 5.9E-11 | -0.0687 (0.0565) | 0.224 | 42.9 | 7.341 |
| Whole body fat-free mass | Vascular dementia (FinnGen) | rs1179905 | 6:90322922 | G/A | 0.195 | 0.0103 (0.0016) | 6.3E-11 | 0.0735 (0.0652) | 0.259 | 42.7 | 7.159 |
| Whole body fat-free mass | Vascular dementia (FinnGen) | rs6416914 | 17:39262850 | G/A | 0.695 | -0.0088 (0.0014) | 6.5E-11 | 0.0140 (0.0568) | 0.805 | 42.7 | -1.586 |
| Whole body fat-free mass | Vascular dementia (FinnGen) | rs8074074 | 17:17407816 | T/C | 0.755 | 0.0095 (0.0014) | 6.8E-11 | 0.0431 (0.0541) | 0.425 | 42.6 | 4.557 |
| Whole body fat-free mass | Vascular dementia (FinnGen) | rs34017457 | 16:67166731 | A/G | 0.007 | 0.0488 (0.0075) | 6.9E-11 | -0.1207 (0.3262) | 0.711 | 42.5 | -2.475 |
| Whole body fat-free mass | Vascular dementia (FinnGen) | rs10843397 | 12:29529523 | T/C | 0.242 | 0.0094 (0.0014) | 7.6E-11 | -0.0507 (0.0574) | 0.377 | 42.4 | -5.385 |
| Whole body fat-free mass | Vascular dementia (FinnGen) | rs10511111 | 3:80599757 | C/T | 0.309 | 0.0087 (0.0013) | 7.9E-11 | -0.0320 (0.0531) | 0.547 | 42.3 | -3.668 |
| Whole body fat-free mass | Vascular dementia (FinnGen) | rs113619763 | 22:46399175 | T/A | 0.062 | 0.0168 (0.0026) | 8.0E-11 | -0.0768 (0.1092) | 0.482 | 42.3 | -4.581 |
| Whole body fat-free mass | Vascular dementia (FinnGen) | rs1268947 | 10:121132631 | C/G | 0.126 | 0.0122 (0.0019) | 8.9E-11 | 0.0048 (0.0775) | 0.950 | 42.1 | 0.394 |
| Whole body fat-free mass | Vascular dementia (FinnGen) | rs60385590 | 3:53630199 | A/C | 0.258 | 0.0093 (0.0014) | 1.0E-10 | 0.0184 (0.0578) | 0.750 | 41.8 | 1.986 |
| Whole body fat-free mass | Vascular dementia (FinnGen) | rs7755185 | 6:152339615 | G/A | 0.311 | 0.0087 (0.0013) | 1.0E-10 | 0.0444 (0.0505) | 0.379 | 41.8 | 5.129 |
| Whole body fat-free mass | Vascular dementia (FinnGen) | rs56141370 | 9:127056213 | T/C | 0.115 | 0.0126 (0.0020) | 1.0E-10 | -0.0838 (0.0787) | 0.287 | 41.7 | -6.648 |
| Whole body fat-free mass | Vascular dementia (FinnGen) | rs4635681 | 3:152310614 | G/A | 0.156 | 0.0110 (0.0017) | 1.1E-10 | -0.1034 (0.0616) | 0.093 | 41.6 | -9.402 |
| Whole body fat-free mass | Vascular dementia (FinnGen) | rs1266876 | 6:51781580 | T/C | 0.350 | 0.0084 (0.0013) | 1.1E-10 | 0.0361 (0.0493) | 0.464 | 41.6 | 4.310 |
| Whole body fat-free mass | Vascular dementia (FinnGen) | rs240113 | 6:101058886 | A/G | 0.531 | -0.0080 (0.0012) | 1.1E-10 | -0.0368 (0.0492) | 0.454 | 41.7 | 4.586 |
| Whole body fat-free mass | Vascular dementia (FinnGen) | rs6900690 | 6:126223032 | A/G | 0.562 | 0.0080 (0.0012) | 1.1E-10 | -0.0905 (0.0502) | 0.071 | 41.6 | -11.269 |
| Whole body fat-free mass | Vascular dementia (FinnGen) | rs12156265 | 8:144848756 | A/G | 0.590 | 0.0082 (0.0013) | 1.1E-10 | -0.0075 (0.0497) | 0.880 | 41.6 | -0.919 |
| Whole body fat-free mass | Vascular dementia (FinnGen) | rs1008158 | 9:113828811 | G/A | 0.342 | 0.0085 (0.0013) | 1.1E-10 | -0.1263 (0.0506) | 0.013 | 41.6 | -14.868 |
| Whole body fat-free mass | Vascular dementia (FinnGen) | rs1407031 | 20:6542634 | T/C | 0.397 | -0.0082 (0.0013) | 1.2E-10 | 0.0812 (0.0497) | 0.103 | 41.4 | -9.889 |
| Whole body fat-free mass | Vascular dementia (FinnGen) | rs17115481 | 5:153358226 | A/G | 0.270 | -0.0090 (0.0014) | 1.3E-10 | -0.0337 (0.0534) | 0.528 | 41.4 | 3.743 |
| Whole body fat-free mass | Vascular dementia (FinnGen) | rs62466118 | 7:92716556 | A/G | 0.028 | -0.0255 (0.0040) | 1.4E-10 | 0.0778 (0.2838) | 0.784 | 41.1 | -3.056 |
| Whole body fat-free mass | Vascular dementia (FinnGen) | rs36100359 | 14:21578007 | A/G | 0.139 | -0.0115 (0.0018) | 1.4E-10 | -0.0629 (0.0696) | 0.366 | 41.2 | 5.462 |
| Whole body fat-free mass | Vascular dementia (FinnGen) | rs10498672 | 6:7797840 | G/C | 0.176 | 0.0104 (0.0016) | 1.6E-10 | -0.0446 (0.0598) | 0.455 | 40.9 | -4.293 |
| Whole body fat-free mass | Vascular dementia (FinnGen) | rs1657222 | 10:34837139 | A/G | 0.396 | 0.0081 (0.0013) | 1.7E-10 | 0.0668 (0.0491) | 0.174 | 40.8 | 8.212 |
| Whole body fat-free mass | Vascular dementia (FinnGen) | rs67141907 | 13:42805360 | T/C | 0.149 | 0.0112 (0.0018) | 1.7E-10 | -0.1455 (0.0787) | 0.065 | 40.8 | -12.995 |
| Whole body fat-free mass | Vascular dementia (FinnGen) | rs281385 | 19:49217261 | G/A | 0.879 | -0.0122 (0.0019) | 1.7E-10 | -0.0556 (0.0653) | 0.394 | 40.8 | 4.555 |
| Whole body fat-free mass | Vascular dementia (FinnGen) | rs62439025 | 6:166592164 | C/G | 0.152 | 0.0110 (0.0017) | 1.8E-10 | 0.0635 (0.0790) | 0.422 | 40.6 | 5.758 |
| Whole body fat-free mass | Vascular dementia (FinnGen) | rs6591 | 11:840363 | T/C | 0.544 | 0.0080 (0.0013) | 1.9E-10 | -0.0380 (0.0495) | 0.442 | 40.6 | -4.761 |
| Whole body fat-free mass | Vascular dementia (FinnGen) | rs7843128 | 8:22473465 | C/T | 0.357 | -0.0083 (0.0013) | 2.0E-10 | -0.0440 (0.0522) | 0.400 | 40.5 | 5.325 |
| Whole body fat-free mass | Vascular dementia (FinnGen) | rs62246311 | 3:9498143 | A/G | 0.102 | 0.0130 (0.0020) | 2.2E-10 | 0.0643 (0.0994) | 0.518 | 40.3 | 4.954 |
| Whole body fat-free mass | Vascular dementia (FinnGen) | rs12146350 | 10:22843111 | G/C | 0.602 | -0.0081 (0.0013) | 2.3E-10 | 0.0935 (0.0510) | 0.067 | 40.2 | -11.595 |
| Whole body fat-free mass | Vascular dementia (FinnGen) | rs4906203 | 14:102928991 | T/C | 0.234 | -0.0093 (0.0015) | 2.6E-10 | -0.0121 (0.0552) | 0.827 | 40.0 | 1.303 |
| Whole body fat-free mass | Vascular dementia (FinnGen) | rs10188231 | 2:142297493 | G/C | 0.186 | -0.0100 (0.0016) | 2.7E-10 | -0.0095 (0.0600) | 0.874 | 39.9 | 0.946 |
| Whole body fat-free mass | Vascular dementia (FinnGen) | rs10770705 | 12:20857467 | C/A | 0.663 | -0.0083 (0.0013) | 2.8E-10 | -0.0194 (0.0535) | 0.716 | 39.8 | 2.345 |
| Whole body fat-free mass | Vascular dementia (FinnGen) | rs6772164 | 3:196078149 | A/C | 0.358 | 0.0081 (0.0013) | 3.1E-10 | -0.0395 (0.0539) | 0.464 | 39.6 | -4.850 |
| Whole body fat-free mass | Vascular dementia (FinnGen) | rs76307059 | 6:169000888 | G/C | 0.046 | -0.0186 (0.0030) | 3.1E-10 | 0.1238 (0.1070) | 0.247 | 39.6 | -6.657 |
| Whole body fat-free mass | Vascular dementia (FinnGen) | rs141866277 | 15:43950699 | T/A | 0.024 | -0.0257 (0.0041) | 3.1E-10 | 0.0081 (0.3293) | 0.980 | 39.6 | -0.316 |
| Whole body fat-free mass | Vascular dementia (FinnGen) | rs3730071 | 12:49168798 | A/C | 0.030 | -0.0228 (0.0036) | 3.2E-10 | 0.0359 (0.2473) | 0.884 | 39.6 | -1.576 |
| Whole body fat-free mass | Vascular dementia (FinnGen) | rs11142700 | 9:73757155 | C/T | 0.408 | -0.0080 (0.0013) | 3.3E-10 | -0.0934 (0.0520) | 0.072 | 39.5 | 11.734 |
| Whole body fat-free mass | Vascular dementia (FinnGen) | rs10881583 | 9:137267439 | C/T | 0.241 | -0.0091 (0.0015) | 3.4E-10 | -0.0228 (0.0571) | 0.691 | 39.4 | 2.493 |
| Whole body fat-free mass | Vascular dementia (FinnGen) | rs8007644 | 14:35218831 | A/G | 0.388 | 0.0081 (0.0013) | 3.4E-10 | 0.0616 (0.0494) | 0.212 | 39.4 | 7.625 |
| Whole body fat-free mass | Vascular dementia (FinnGen) | rs10282707 | 7:17911038 | T/C | 0.397 | 0.0079 (0.0013) | 3.6E-10 | -0.0721 (0.0522) | 0.167 | 39.3 | -9.071 |
| Whole body fat-free mass | Vascular dementia (FinnGen) | rs6575340 | 14:94023972 | A/G | 0.636 | 0.0081 (0.0013) | 3.6E-10 | 0.0878 (0.0506) | 0.083 | 39.3 | 10.810 |
| Whole body fat-free mass | Vascular dementia (FinnGen) | rs11205354 | 1:150249101 | A/C | 0.444 | -0.0078 (0.0013) | 3.7E-10 | -0.0899 (0.0497) | 0.070 | 39.3 | 11.459 |
| Whole body fat-free mass | Vascular dementia (FinnGen) | rs13085472 | 3:171129859 | C/T | 0.666 | -0.0083 (0.0013) | 4.1E-10 | -0.0162 (0.0518) | 0.755 | 39.1 | 1.963 |
| Whole body fat-free mass | Vascular dementia (FinnGen) | rs12533548 | 7:148660579 | G/A | 0.651 | 0.0081 (0.0013) | 4.6E-10 | 0.0365 (0.0525) | 0.487 | 38.8 | 4.481 |
| Whole body fat-free mass | Vascular dementia (FinnGen) | rs112544217 | 2:20222686 | T/C | 0.022 | -0.0268 (0.0043) | 4.8E-10 | -0.1635 (0.2088) | 0.434 | 38.8 | 6.096 |
| Whole body fat-free mass | Vascular dementia (FinnGen) | rs284315 | 1:10734800 | G/A | 0.498 | -0.0077 (0.0012) | 4.9E-10 | 0.0119 (0.0511) | 0.815 | 38.7 | -1.549 |
| Whole body fat-free mass | Vascular dementia (FinnGen) | rs117451679 | 12:17245591 | G/A | 0.106 | 0.0127 (0.0020) | 5.0E-10 | 0.0311 (0.0752) | 0.679 | 38.7 | 2.445 |
| Whole body fat-free mass | Vascular dementia (FinnGen) | rs12047986 | 1:155043812 | G/A | 0.522 | -0.0077 (0.0012) | 5.1E-10 | -0.0938 (0.0511) | 0.067 | 38.6 | 12.185 |
| Whole body fat-free mass | Vascular dementia (FinnGen) | rs11937249 | 4:110755321 | T/G | 0.636 | -0.0080 (0.0013) | 5.1E-10 | 0.0213 (0.0499) | 0.669 | 38.6 | -2.658 |
| Whole body fat-free mass | Vascular dementia (FinnGen) | rs2422054 | 5:161382474 | A/T | 0.631 | 0.0080 (0.0013) | 5.1E-10 | 0.0260 (0.0494) | 0.599 | 38.6 | 3.253 |
| Whole body fat-free mass | Vascular dementia (FinnGen) | rs4819021 | 21:46466927 | C/T | 0.477 | -0.0078 (0.0013) | 6.4E-10 | 0.0354 (0.0492) | 0.473 | 38.2 | -4.545 |
| Whole body fat-free mass | Vascular dementia (FinnGen) | rs11684531 | 2:219835489 | G/A | 0.133 | -0.0113 (0.0018) | 6.6E-10 | 0.0016 (0.0969) | 0.987 | 38.1 | -0.142 |
| Whole body fat-free mass | Vascular dementia (FinnGen) | rs11833839 | 12:132560957 | T/C | 0.057 | 0.0166 (0.0027) | 6.8E-10 | 0.1007 (0.0702) | 0.151 | 38.1 | 6.083 |
| Whole body fat-free mass | Vascular dementia (FinnGen) | rs77093479 | 17:79429575 | G/C | 0.165 | -0.0104 (0.0017) | 6.9E-10 | -0.0005 (0.0654) | 0.993 | 38.0 | 0.048 |
| Whole body fat-free mass | Vascular dementia (FinnGen) | rs34825238 | 12:103328352 | T/G | 0.255 | 0.0089 (0.0014) | 7.1E-10 | -0.0379 (0.0629) | 0.546 | 38.0 | -4.263 |
| Whole body fat-free mass | Vascular dementia (FinnGen) | rs6946419 | 7:28768853 | C/T | 0.377 | -0.0079 (0.0013) | 7.2E-10 | -0.0410 (0.0505) | 0.416 | 38.0 | 5.180 |
| Whole body fat-free mass | Vascular dementia (FinnGen) | rs752070 | 2:74824970 | G/A | 0.126 | 0.0114 (0.0019) | 8.2E-10 | 0.0018 (0.0715) | 0.979 | 37.7 | 0.157 |
| Whole body fat-free mass | Vascular dementia (FinnGen) | rs2274116 | 9:139094805 | T/C | 0.341 | -0.0081 (0.0013) | 8.3E-10 | 0.0821 (0.0525) | 0.118 | 37.7 | -10.149 |
| Whole body fat-free mass | Vascular dementia (FinnGen) | rs6693481 | 1:203766395 | C/T | 0.695 | -0.0083 (0.0013) | 8.6E-10 | -0.0235 (0.0517) | 0.650 | 37.6 | 2.844 |
| Whole body fat-free mass | Vascular dementia (FinnGen) | rs2807742 | 10:28781367 | A/G | 0.771 | -0.0091 (0.0015) | 8.6E-10 | -0.1079 (0.0614) | 0.079 | 37.6 | 11.856 |
| Whole body fat-free mass | Vascular dementia (FinnGen) | rs161799 | 1:8015560 | G/A | 0.344 | -0.0080 (0.0013) | 9.0E-10 | -0.0360 (0.0537) | 0.503 | 37.5 | 4.483 |
| Whole body fat-free mass | Vascular dementia (FinnGen) | rs72754922 | 5:42547714 | A/G | 0.061 | -0.0160 (0.0026) | 9.1E-10 | 0.1431 (0.1144) | 0.211 | 37.5 | -8.967 |
| Whole body fat-free mass | Vascular dementia (FinnGen) | rs35665085 | 22:17625915 | A/G | 0.056 | -0.0166 (0.0027) | 9.2E-10 | 0.1852 (0.1092) | 0.090 | 37.5 | -11.162 |
| Whole body fat-free mass | Vascular dementia (FinnGen) | rs34227797 | 4:159982551 | C/G | 0.310 | 0.0082 (0.0013) | 9.3E-10 | 0.0554 (0.0534) | 0.300 | 37.5 | 6.750 |
| Whole body fat-free mass | Vascular dementia (FinnGen) | rs2611732 | 5:95855576 | G/A | 0.602 | 0.0077 (0.0013) | 9.3E-10 | -0.0324 (0.0534) | 0.544 | 37.5 | -4.182 |
| Whole body fat-free mass | Vascular dementia (FinnGen) | rs4980826 | 12:578349 | A/C | 0.396 | 0.0078 (0.0013) | 9.3E-10 | -0.0706 (0.0507) | 0.164 | 37.5 | -9.106 |
| Whole body fat-free mass | Vascular dementia (FinnGen) | rs1910466 | 3:147086268 | C/T | 0.498 | -0.0076 (0.0012) | 9.4E-10 | 0.0129 (0.0494) | 0.794 | 37.5 | -1.690 |
| Whole body fat-free mass | Vascular dementia (FinnGen) | rs56207600 | 11:126196537 | A/G | 0.111 | 0.0122 (0.0020) | 9.6E-10 | -0.0119 (0.0724) | 0.870 | 37.4 | -0.978 |
| Whole body fat-free mass | Vascular dementia (FinnGen) | rs11925245 | 3:114183579 | G/A | 0.183 | -0.0099 (0.0016) | 1.0E-09 | -0.0966 (0.0630) | 0.125 | 37.3 | 9.759 |
| Whole body fat-free mass | Vascular dementia (FinnGen) | rs1928850 | 9:16765520 | T/A | 0.922 | 0.0142 (0.0023) | 1.0E-09 | 0.1345 (0.0887) | 0.130 | 37.3 | 9.449 |
| Whole body fat-free mass | Vascular dementia (FinnGen) | rs1941697 | 18:31251276 | A/G | 0.449 | 0.0076 (0.0013) | 1.0E-09 | 0.0368 (0.0493) | 0.456 | 37.3 | 4.819 |
| Whole body fat-free mass | Vascular dementia (FinnGen) | rs4887925 | 16:73784416 | C/G | 0.413 | -0.0077 (0.0013) | 1.2E-09 | -0.0115 (0.0492) | 0.815 | 36.9 | 1.494 |
| Whole body fat-free mass | Vascular dementia (FinnGen) | rs6781248 | 3:13726684 | A/G | 0.546 | 0.0076 (0.0012) | 1.3E-09 | -0.0949 (0.0503) | 0.059 | 36.8 | -12.558 |
| Whole body fat-free mass | Vascular dementia (FinnGen) | rs3853474 | 5:141816555 | T/C | 0.377 | -0.0078 (0.0013) | 1.3E-09 | -0.0268 (0.0509) | 0.599 | 36.8 | 3.456 |
| Whole body fat-free mass | Vascular dementia (FinnGen) | rs9654451 | 5:299543 | T/C | 0.129 | 0.0112 (0.0019) | 1.4E-09 | 0.0473 (0.0858) | 0.581 | 36.7 | 4.215 |
| Whole body fat-free mass | Vascular dementia (FinnGen) | rs8002779 | 13:92015977 | A/G | 0.603 | -0.0077 (0.0013) | 1.5E-09 | -0.0197 (0.0493) | 0.689 | 36.5 | 2.573 |
| Whole body fat-free mass | Vascular dementia (FinnGen) | rs2521349 | 17:67503501 | A/G | 0.385 | 0.0077 (0.0013) | 1.6E-09 | 0.0210 (0.0502) | 0.675 | 36.4 | 2.715 |
| Whole body fat-free mass | Vascular dementia (FinnGen) | rs695922 | 5:53385529 | G/A | 0.837 | -0.0102 (0.0017) | 1.7E-09 | 0.0190 (0.0664) | 0.775 | 36.4 | -1.858 |
| Whole body fat-free mass | Vascular dementia (FinnGen) | rs6563808 | 13:40766662 | C/T | 0.734 | -0.0085 (0.0014) | 1.7E-09 | 0.0107 (0.0527) | 0.840 | 36.3 | -1.255 |
| Whole body fat-free mass | Vascular dementia (FinnGen) | rs5771118 | 22:50714289 | C/T | 0.742 | 0.0086 (0.0014) | 1.8E-09 | -0.0181 (0.0607) | 0.766 | 36.2 | -2.100 |
| Whole body fat-free mass | Vascular dementia (FinnGen) | rs41458449 | 2:46929951 | C/G | 0.144 | -0.0106 (0.0018) | 1.9E-09 | 0.0128 (0.0630) | 0.839 | 36.1 | -1.205 |
| Whole body fat-free mass | Vascular dementia (FinnGen) | rs6721191 | 2:10190115 | G/A | 0.578 | -0.0075 (0.0013) | 2.0E-09 | -0.0639 (0.0553) | 0.248 | 36.0 | 8.501 |
| Whole body fat-free mass | Vascular dementia (FinnGen) | rs72703409 | 4:184225295 | C/G | 0.066 | -0.0150 (0.0025) | 2.0E-09 | -0.0684 (0.1279) | 0.593 | 35.9 | 4.550 |
| Whole body fat-free mass | Vascular dementia (FinnGen) | rs572474 | 10:89791654 | G/A | 0.166 | 0.0100 (0.0017) | 2.0E-09 | 0.0577 (0.0656) | 0.380 | 36.0 | 5.754 |
| Whole body fat-free mass | Vascular dementia (FinnGen) | rs9898189 | 17:80480516 | G/C | 0.644 | -0.0081 (0.0013) | 2.1E-09 | -0.0138 (0.0500) | 0.783 | 35.9 | 1.707 |
| Whole body fat-free mass | Vascular dementia (FinnGen) | rs12731187 | 1:202013757 | T/C | 0.358 | -0.0078 (0.0013) | 2.5E-09 | 0.0318 (0.0508) | 0.531 | 35.5 | -4.095 |
| Whole body fat-free mass | Vascular dementia (FinnGen) | rs9365939 | 6:166336825 | G/A | 0.548 | -0.0074 (0.0012) | 2.5E-09 | -0.0201 (0.0513) | 0.695 | 35.5 | 2.716 |
| Whole body fat-free mass | Vascular dementia (FinnGen) | rs56130943 | 14:105906522 | C/A | 0.233 | 0.0090 (0.0015) | 2.9E-09 | 0.0417 (0.0625) | 0.504 | 35.3 | 4.646 |
| Whole body fat-free mass | Vascular dementia (FinnGen) | rs148662000 | 1:228721530 | T/G | 0.064 | -0.0151 (0.0025) | 3.0E-09 | 0.0872 (0.1175) | 0.458 | 35.2 | -5.777 |
| Whole body fat-free mass | Vascular dementia (FinnGen) | rs10939792 | 4:18483405 | G/C | 0.320 | 0.0079 (0.0013) | 3.2E-09 | -0.0221 (0.0559) | 0.693 | 35.1 | -2.808 |
| Whole body fat-free mass | Vascular dementia (FinnGen) | rs876672 | 16:81571600 | G/A | 0.085 | -0.0132 (0.0022) | 3.2E-09 | -0.0664 (0.0859) | 0.440 | 35.0 | 5.016 |
| Whole body fat-free mass | Vascular dementia (FinnGen) | rs7535501 | 1:176462695 | T/C | 0.944 | -0.0159 (0.0027) | 3.3E-09 | 0.0471 (0.1249) | 0.706 | 35.0 | -2.957 |
| Whole body fat-free mass | Vascular dementia (FinnGen) | rs116337081 | 2:183954625 | T/C | 0.070 | 0.0143 (0.0024) | 3.6E-09 | 0.0111 (0.1186) | 0.925 | 34.8 | 0.774 |
| Whole body fat-free mass | Vascular dementia (FinnGen) | rs2920665 | 8:6316097 | G/T | 0.174 | 0.0097 (0.0016) | 3.6E-09 | -0.0710 (0.0748) | 0.343 | 34.8 | -7.317 |
| Whole body fat-free mass | Vascular dementia (FinnGen) | rs1176314 | 13:81221014 | G/T | 0.550 | -0.0074 (0.0013) | 3.6E-09 | 0.0356 (0.0508) | 0.483 | 34.9 | -4.796 |
| Whole body fat-free mass | Vascular dementia (FinnGen) | rs13392079 | 2:169119178 | C/T | 0.756 | -0.0085 (0.0014) | 3.8E-09 | -0.0131 (0.0527) | 0.804 | 34.7 | 1.542 |
| Whole body fat-free mass | Vascular dementia (FinnGen) | rs224048 | 10:64491549 | A/G | 0.550 | -0.0073 (0.0012) | 3.8E-09 | 0.1344 (0.0490) | 0.006 | 34.7 | -18.304 |
| Whole body fat-free mass | Vascular dementia (FinnGen) | rs7280982 | 21:37468223 | A/G | 0.221 | -0.0088 (0.0015) | 4.2E-09 | -0.0229 (0.0582) | 0.695 | 34.5 | 2.588 |
| Whole body fat-free mass | Vascular dementia (FinnGen) | rs116165844 | 20:62610556 | T/G | 0.137 | -0.0106 (0.0018) | 4.3E-09 | 0.0622 (0.0775) | 0.422 | 34.5 | -5.862 |
| Whole body fat-free mass | Vascular dementia (FinnGen) | rs29946 | 19:34299865 | C/T | 0.557 | 0.0075 (0.0013) | 4.5E-09 | -0.0443 (0.0494) | 0.370 | 34.4 | -5.931 |
| Whole body fat-free mass | Vascular dementia (FinnGen) | rs182224 | 5:67316442 | T/C | 0.090 | 0.0127 (0.0022) | 4.6E-09 | 0.0048 (0.0755) | 0.949 | 34.4 | 0.377 |
| Whole body fat-free mass | Vascular dementia (FinnGen) | rs6747657 | 2:112003163 | A/G | 0.282 | 0.0081 (0.0014) | 4.7E-09 | 0.0896 (0.0552) | 0.104 | 34.3 | 11.129 |
| Whole body fat-free mass | Vascular dementia (FinnGen) | rs11263853 | 1:36579648 | A/G | 0.810 | 0.0092 (0.0016) | 4.8E-09 | -0.0592 (0.0595) | 0.320 | 34.2 | -6.407 |
| Whole body fat-free mass | Vascular dementia (FinnGen) | rs9985795 | 4:135213286 | C/T | 0.483 | -0.0073 (0.0012) | 5.1E-09 | -0.0497 (0.0497) | 0.317 | 34.2 | 6.835 |
| Whole body fat-free mass | Vascular dementia (FinnGen) | rs34055910 | 17:64460104 | G/A | 0.379 | -0.0075 (0.0013) | 5.1E-09 | -0.0004 (0.0493) | 0.994 | 34.2 | 0.053 |
| Whole body fat-free mass | Vascular dementia (FinnGen) | rs13185520 | 5:137807260 | A/G | 0.574 | -0.0073 (0.0013) | 5.4E-09 | 0.0272 (0.0512) | 0.596 | 34.1 | -3.723 |
| Whole body fat-free mass | Vascular dementia (FinnGen) | rs116817990 | 1:178989260 | G/A | 0.034 | -0.0201 (0.0034) | 5.7E-09 | -0.0961 (0.1358) | 0.479 | 33.9 | 4.783 |
| Whole body fat-free mass | Vascular dementia (FinnGen) | rs3772051 | 2:241672904 | A/G | 0.237 | -0.0086 (0.0015) | 5.9E-09 | -0.1443 (0.0772) | 0.062 | 33.9 | 16.763 |
| Whole body fat-free mass | Vascular dementia (FinnGen) | rs236587 | 17:68203915 | C/T | 0.737 | -0.0083 (0.0014) | 6.2E-09 | -0.0377 (0.0539) | 0.484 | 33.8 | 4.546 |
| Whole body fat-free mass | Vascular dementia (FinnGen) | rs118173451 | 22:28356600 | C/T | 0.016 | -0.0292 (0.0050) | 6.3E-09 | 0.1037 (0.2048) | 0.613 | 33.7 | -3.557 |
| Whole body fat-free mass | Vascular dementia (FinnGen) | rs6500249 | 16:49778147 | G/A | 0.735 | -0.0082 (0.0014) | 6.4E-09 | 0.0366 (0.0618) | 0.554 | 33.7 | -4.454 |
| Whole body fat-free mass | Vascular dementia (FinnGen) | rs5753630 | 22:31861950 | G/A | 0.439 | 0.0073 (0.0013) | 6.7E-09 | 0.0196 (0.0492) | 0.690 | 33.6 | 2.683 |
| Whole body fat-free mass | Vascular dementia (FinnGen) | rs237738 | 20:47904920 | C/T | 0.823 | 0.0094 (0.0016) | 7.3E-09 | -0.0464 (0.0653) | 0.478 | 33.5 | -4.924 |
| Whole body fat-free mass | Vascular dementia (FinnGen) | rs9800418 | 5:77641008 | C/T | 0.245 | 0.0084 (0.0014) | 7.9E-09 | -0.0139 (0.0561) | 0.804 | 33.3 | -1.663 |
| Whole body fat-free mass | Vascular dementia (FinnGen) | rs1421035 | 10:131136401 | T/C | 0.348 | 0.0076 (0.0013) | 8.0E-09 | -0.1045 (0.0512) | 0.041 | 33.3 | -13.778 |
| Whole body fat-free mass | Vascular dementia (FinnGen) | rs10020631 | 4:69353863 | A/G | 0.248 | -0.0083 (0.0014) | 8.2E-09 | 0.0259 (0.0575) | 0.653 | 33.2 | -3.115 |
| Whole body fat-free mass | Vascular dementia (FinnGen) | rs4676442 | 2:241740907 | C/T | 0.356 | 0.0074 (0.0013) | 9.1E-09 | 0.0939 (0.0501) | 0.061 | 33.0 | 12.644 |
| Whole body fat-free mass | Vascular dementia (FinnGen) | rs9491201 | 6:124934212 | T/C | 0.164 | 0.0096 (0.0017) | 9.5E-09 | 0.0403 (0.0953) | 0.672 | 32.9 | 4.198 |
| Whole body fat-free mass | Vascular dementia (FinnGen) | rs1846221 | 10:4667302 | C/G | 0.175 | -0.0095 (0.0017) | 9.7E-09 | -0.0326 (0.0650) | 0.616 | 32.9 | 3.430 |
| Whole body fat-free mass | Vascular dementia (FinnGen) | rs35651070 | 2:121613739 | A/G | 0.167 | -0.0095 (0.0017) | 9.9E-09 | -0.0699 (0.0688) | 0.310 | 32.9 | 7.355 |
| Whole body fat-free mass | Vascular dementia (FinnGen) | rs2920974 | 2:55537126 | C/A | 0.560 | 0.0072 (0.0013) | 1.1E-08 | -0.1041 (0.0528) | 0.049 | 32.7 | -14.551 |
| Whole body fat-free mass | Vascular dementia (FinnGen) | rs35612982 | 6:20682622 | C/T | 0.178 | -0.0092 (0.0016) | 1.1E-08 | 0.0360 (0.0641) | 0.575 | 32.7 | -3.894 |
| Whole body fat-free mass | Vascular dementia (FinnGen) | rs8091374 | 18:71904385 | A/G | 0.161 | -0.0099 (0.0017) | 1.1E-08 | -0.0541 (0.0625) | 0.386 | 32.7 | 5.480 |
| Whole body fat-free mass | Vascular dementia (FinnGen) | rs34345560 | 11:69081998 | A/G | 0.195 | 0.0090 (0.0016) | 1.2E-08 | -0.0666 (0.0568) | 0.241 | 32.4 | -7.403 |
| Whole body fat-free mass | Vascular dementia (FinnGen) | rs6743107 | 2:100844781 | T/C | 0.275 | 0.0079 (0.0014) | 1.3E-08 | 0.1076 (0.0533) | 0.044 | 32.3 | 13.676 |
| Whole body fat-free mass | Vascular dementia (FinnGen) | rs9783665 | 14:103271158 | A/T | 0.653 | -0.0074 (0.0013) | 1.3E-08 | 0.0398 (0.0517) | 0.442 | 32.3 | -5.365 |
| Whole body fat-free mass | Vascular dementia (FinnGen) | rs8088739 | 18:60767088 | C/T | 0.244 | 0.0082 (0.0014) | 1.3E-08 | 0.0650 (0.0584) | 0.266 | 32.3 | 7.900 |
| Whole body fat-free mass | Vascular dementia (FinnGen) | rs34831515 | 19:17275777 | T/C | 0.232 | -0.0084 (0.0015) | 1.3E-08 | 0.0953 (0.0531) | 0.073 | 32.3 | -11.341 |
| Whole body fat-free mass | Vascular dementia (FinnGen) | rs35050648 | 19:46991243 | T/G | 0.236 | 0.0083 (0.0015) | 1.6E-08 | 0.0443 (0.0567) | 0.435 | 32.0 | 5.346 |
| Whole body fat-free mass | Vascular dementia (FinnGen) | rs62460525 | 7:44902676 | A/C | 0.070 | 0.0137 (0.0024) | 1.7E-08 | 0.1091 (0.1437) | 0.448 | 31.8 | 7.956 |
| Whole body fat-free mass | Vascular dementia (FinnGen) | rs2740761 | 7:55255986 | T/C | 0.208 | 0.0087 (0.0015) | 1.7E-08 | -0.0136 (0.0623) | 0.827 | 31.8 | -1.559 |
| Whole body fat-free mass | Vascular dementia (FinnGen) | rs113978196 | 15:75916965 | A/G | 0.251 | -0.0081 (0.0014) | 1.8E-08 | -0.0449 (0.0553) | 0.417 | 31.8 | 5.558 |
| Whole body fat-free mass | Vascular dementia (FinnGen) | rs1043801 | 21:47552527 | A/G | 0.058 | 0.0151 (0.0027) | 1.8E-08 | -0.1083 (0.1020) | 0.288 | 31.7 | -7.183 |
| Whole body fat-free mass | Vascular dementia (FinnGen) | rs10848288 | 12:131600054 | T/C | 0.304 | -0.0076 (0.0014) | 1.9E-08 | -0.0296 (0.0553) | 0.593 | 31.6 | 3.892 |
| Whole body fat-free mass | Vascular dementia (FinnGen) | rs3751866 | 16:84771304 | C/T | 0.247 | -0.0081 (0.0014) | 1.9E-08 | 0.0081 (0.0579) | 0.889 | 31.6 | -0.998 |
| Whole body fat-free mass | Vascular dementia (FinnGen) | rs111598585 | 4:171635471 | T/C | 0.209 | -0.0086 (0.0015) | 2.0E-08 | -0.1565 (0.0807) | 0.052 | 31.5 | 18.228 |
| Whole body fat-free mass | Vascular dementia (FinnGen) | rs2884345 | 14:53323211 | A/G | 0.710 | -0.0077 (0.0014) | 2.1E-08 | -0.0003 (0.0542) | 0.995 | 31.4 | 0.039 |
| Whole body fat-free mass | Vascular dementia (FinnGen) | rs2252909 | 17:2278609 | T/C | 0.657 | -0.0075 (0.0013) | 2.1E-08 | -0.0218 (0.0497) | 0.661 | 31.4 | 2.921 |
| Whole body fat-free mass | Vascular dementia (FinnGen) | rs12553221 | 9:37234008 | A/G | 0.362 | 0.0073 (0.0013) | 2.2E-08 | 0.0352 (0.0511) | 0.491 | 31.3 | 4.851 |
| Whole body fat-free mass | Vascular dementia (FinnGen) | rs498685 | 18:13088673 | C/T | 0.594 | -0.0071 (0.0013) | 2.2E-08 | 0.0532 (0.0495) | 0.283 | 31.3 | -7.510 |
| Whole body fat-free mass | Vascular dementia (FinnGen) | rs17011108 | 4:86800806 | C/T | 0.307 | 0.0076 (0.0014) | 2.3E-08 | 0.0304 (0.0553) | 0.583 | 31.2 | 4.016 |
| Whole body fat-free mass | Vascular dementia (FinnGen) | rs12795042 | 11:133658168 | C/A | 0.622 | 0.0073 (0.0013) | 2.3E-08 | -0.0184 (0.0538) | 0.733 | 31.3 | -2.536 |
| Whole body fat-free mass | Vascular dementia (FinnGen) | rs4800670 | 18:23415263 | C/G | 0.386 | -0.0072 (0.0013) | 2.3E-08 | -0.0545 (0.0504) | 0.279 | 31.3 | 7.601 |
| Whole body fat-free mass | Vascular dementia (FinnGen) | rs73189390 | 21:17383170 | A/G | 0.184 | -0.0090 (0.0016) | 2.3E-08 | 0.0980 (0.0619) | 0.113 | 31.3 | -10.835 |
| Whole body fat-free mass | Vascular dementia (FinnGen) | rs55854145 | 18:45928049 | C/A | 0.055 | -0.0153 (0.0027) | 2.4E-08 | 0.0966 (0.1253) | 0.441 | 31.1 | -6.308 |
| Whole body fat-free mass | Vascular dementia (FinnGen) | rs7513326 | 1:159895536 | A/G | 0.508 | -0.0070 (0.0013) | 2.5E-08 | 0.0608 (0.0498) | 0.222 | 31.1 | -8.672 |
| Whole body fat-free mass | Vascular dementia (FinnGen) | rs73158212 | 7:148383377 | G/A | 0.224 | -0.0084 (0.0015) | 2.5E-08 | -0.0603 (0.0657) | 0.358 | 31.1 | 7.181 |
| Whole body fat-free mass | Vascular dementia (FinnGen) | rs6514066 | 20:44500794 | G/T | 0.624 | 0.0072 (0.0013) | 2.5E-08 | -0.0204 (0.0509) | 0.689 | 31.1 | -2.845 |
| Whole body fat-free mass | Vascular dementia (FinnGen) | rs2427320 | 20:60976723 | G/A | 0.232 | -0.0082 (0.0015) | 2.5E-08 | -0.0764 (0.0660) | 0.248 | 31.1 | 9.284 |
| Whole body fat-free mass | Vascular dementia (FinnGen) | rs4821940 | 22:40659573 | C/T | 0.550 | -0.0070 (0.0013) | 2.5E-08 | -0.1521 (0.0494) | 0.002 | 31.1 | 21.730 |
| Whole body fat-free mass | Vascular dementia (FinnGen) | rs1168768 | 12:66509650 | T/C | 0.975 | 0.0221 (0.0040) | 2.6E-08 | -0.1357 (0.1641) | 0.408 | 31.0 | -6.133 |
| Whole body fat-free mass | Vascular dementia (FinnGen) | rs7095768 | 10:53256505 | T/G | 0.263 | 0.0078 (0.0014) | 2.7E-08 | -0.1407 (0.0571) | 0.014 | 30.9 | -17.973 |
| Whole body fat-free mass | Vascular dementia (FinnGen) | rs2293177 | 7:140244560 | T/C | 0.313 | 0.0074 (0.0013) | 2.9E-08 | 0.0553 (0.0522) | 0.289 | 30.8 | 7.451 |
| Whole body fat-free mass | Vascular dementia (FinnGen) | rs13125082 | 4:57787000 | G/T | 0.278 | -0.0076 (0.0014) | 3.4E-08 | -0.1775 (0.0601) | 0.003 | 30.5 | 23.252 |
| Whole body fat-free mass | Vascular dementia (FinnGen) | rs7683836 | 4:180167906 | A/G | 0.557 | -0.0069 (0.0013) | 3.4E-08 | -0.0400 (0.0492) | 0.416 | 30.5 | 5.790 |
| Whole body fat-free mass | Vascular dementia (FinnGen) | rs926436 | 1:22563429 | A/G | 0.857 | 0.0098 (0.0018) | 3.5E-08 | -0.0074 (0.0689) | 0.914 | 30.4 | -0.757 |
| Whole body fat-free mass | Vascular dementia (FinnGen) | rs34345690 | 3:192445575 | G/A | 0.227 | 0.0082 (0.0015) | 3.5E-08 | 0.0175 (0.0614) | 0.776 | 30.4 | 2.146 |
| Whole body fat-free mass | Vascular dementia (FinnGen) | rs11021307 | 11:95501407 | T/C | 0.418 | 0.0069 (0.0013) | 3.6E-08 | -0.0598 (0.0520) | 0.250 | 30.4 | -8.627 |
| Whole body fat-free mass | Vascular dementia (FinnGen) | rs294382 | 11:30607515 | C/A | 0.720 | -0.0076 (0.0014) | 4.0E-08 | 0.0160 (0.0590) | 0.786 | 30.1 | -2.105 |
| Whole body fat-free mass | Vascular dementia (FinnGen) | rs434072 | 3:12916946 | G/T | 0.709 | -0.0075 (0.0014) | 4.1E-08 | 0.0082 (0.0522) | 0.875 | 30.1 | -1.089 |
| Whole body fat-free mass | Vascular dementia (FinnGen) | rs1028197 | 4:124977659 | T/C | 0.225 | 0.0082 (0.0015) | 4.1E-08 | 0.0606 (0.0658) | 0.357 | 30.1 | 7.399 |
| Whole body fat-free mass | Vascular dementia (FinnGen) | rs12213409 | 6:142610314 | G/T | 0.485 | -0.0068 (0.0012) | 4.7E-08 | 0.0295 (0.0491) | 0.548 | 29.8 | -4.329 |
| Whole body fat-free mass | Alzheimer's disease, wide (FinnGen) | rs12967135 | 18:57849023 | A/G | 0.233 | 0.0433 (0.0015) | 2.3E-190 | 0.0363 (0.0273) | 0.183 | 866.1 | 0.838 |
| Whole body fat-free mass | Alzheimer's disease, wide (FinnGen) | rs56094641 | 16:53806453 | G/A | 0.405 | 0.0361 (0.0013) | 1.0E-178 | -0.0036 (0.0215) | 0.867 | 812.5 | -0.100 |
| Whole body fat-free mass | Alzheimer's disease, wide (FinnGen) | rs143384 | 20:34025756 | G/A | 0.404 | 0.0354 (0.0013) | 1.5E-171 | -0.0114 (0.0214) | 0.592 | 779.6 | -0.322 |
| Whole body fat-free mass | Alzheimer's disease, wide (FinnGen) | rs9853018 | 3:141101961 | T/C | 0.443 | 0.0300 (0.0012) | 6.3E-128 | 0.0409 (0.0213) | 0.055 | 579.0 | 1.364 |
| Whole body fat-free mass | Alzheimer's disease, wide (FinnGen) | rs2744956 | 6:34618937 | C/T | 0.139 | 0.0378 (0.0018) | 2.9E-99 | -0.0353 (0.0269) | 0.189 | 447.2 | -0.935 |
| Whole body fat-free mass | Alzheimer's disease, wide (FinnGen) | rs1351394 | 12:66351826 | C/T | 0.512 | -0.0258 (0.0012) | 5.3E-96 | -0.0219 (0.0212) | 0.302 | 432.2 | 0.849 |
| Whole body fat-free mass | Alzheimer's disease, wide (FinnGen) | rs7740107 | 6:130374461 | A/T | 0.736 | -0.0291 (0.0014) | 1.1E-95 | 0.0336 (0.0239) | 0.160 | 430.7 | -1.153 |
| Whole body fat-free mass | Alzheimer's disease, wide (FinnGen) | rs12995666 | 2:629959 | C/T | 0.828 | 0.0337 (0.0016) | 1.1E-93 | -0.0099 (0.0286) | 0.730 | 421.6 | -0.294 |
| Whole body fat-free mass | Alzheimer's disease, wide (FinnGen) | rs72656010 | 8:57122215 | C/T | 0.132 | -0.0366 (0.0018) | 2.8E-88 | 0.0757 (0.0319) | 0.018 | 396.7 | -2.066 |
| Whole body fat-free mass | Alzheimer's disease, wide (FinnGen) | rs76895963 | 12:4384844 | G/T | 0.021 | 0.0952 (0.0048) | 2.8E-87 | -0.0216 (0.0641) | 0.736 | 392.2 | -0.227 |
| Whole body fat-free mass | Alzheimer's disease, wide (FinnGen) | rs10269774 | 7:92253972 | A/G | 0.326 | 0.0262 (0.0013) | 4.7E-87 | 0.0193 (0.0230) | 0.401 | 391.1 | 0.737 |
| Whole body fat-free mass | Alzheimer's disease, wide (FinnGen) | rs2225226 | 13:51127270 | T/C | 0.216 | -0.0298 (0.0015) | 3.1E-86 | -0.0327 (0.0244) | 0.179 | 387.4 | 1.096 |
| Whole body fat-free mass | Alzheimer's disease, wide (FinnGen) | rs34517439 | 1:78450517 | A/C | 0.122 | 0.0372 (0.0019) | 3.3E-84 | -0.0389 (0.0321) | 0.226 | 378.1 | -1.045 |
| Whole body fat-free mass | Alzheimer's disease, wide (FinnGen) | rs7671110 | 4:17874089 | T/C | 0.158 | -0.0318 (0.0017) | 2.8E-78 | 0.0454 (0.0362) | 0.210 | 350.8 | -1.429 |
| Whole body fat-free mass | Alzheimer's disease, wide (FinnGen) | rs7223535 | 17:29211667 | A/G | 0.270 | -0.0251 (0.0014) | 1.0E-71 | 0.0163 (0.0235) | 0.488 | 320.7 | -0.650 |
| Whole body fat-free mass | Alzheimer's disease, wide (FinnGen) | rs77165542 | 2:430975 | T/C | 0.035 | -0.0585 (0.0034) | 6.6E-67 | -0.1192 (0.0812) | 0.142 | 298.6 | 2.038 |
| Whole body fat-free mass | Alzheimer's disease, wide (FinnGen) | rs2005172 | 17:61996255 | C/A | 0.640 | 0.0226 (0.0013) | 1.0E-66 | 0.0332 (0.0216) | 0.125 | 297.7 | 1.468 |
| Whole body fat-free mass | Alzheimer's disease, wide (FinnGen) | rs4788218 | 16:30055750 | C/T | 0.401 | 0.0218 (0.0013) | 3.6E-66 | -0.0280 (0.0220) | 0.203 | 295.2 | -1.285 |
| Whole body fat-free mass | Alzheimer's disease, wide (FinnGen) | rs4369779 | 18:20735408 | C/T | 0.789 | 0.0260 (0.0015) | 2.6E-65 | 0.0263 (0.0252) | 0.297 | 291.3 | 1.011 |
| Whole body fat-free mass | Alzheimer's disease, wide (FinnGen) | rs4240326 | 4:145839264 | G/A | 0.550 | -0.0212 (0.0012) | 3.7E-65 | -0.0317 (0.0213) | 0.136 | 290.6 | 1.493 |
| Whole body fat-free mass | Alzheimer's disease, wide (FinnGen) | rs4899012 | 14:61003889 | C/G | 0.607 | -0.0215 (0.0013) | 1.1E-63 | 0.0307 (0.0233) | 0.188 | 283.8 | -1.431 |
| Whole body fat-free mass | Alzheimer's disease, wide (FinnGen) | rs527248 | 1:177875514 | G/A | 0.209 | 0.0255 (0.0015) | 2.5E-63 | -0.0182 (0.0275) | 0.509 | 282.2 | -0.713 |
| Whole body fat-free mass | Alzheimer's disease, wide (FinnGen) | rs76798800 | 1:154994978 | T/G | 0.266 | 0.0228 (0.0014) | 2.3E-59 | -0.0273 (0.0252) | 0.278 | 264.0 | -1.198 |
| Whole body fat-free mass | Alzheimer's disease, wide (FinnGen) | rs9634212 | 12:93993266 | A/C | 0.221 | 0.0237 (0.0015) | 4.0E-56 | 0.0099 (0.0245) | 0.688 | 249.2 | 0.418 |
| Whole body fat-free mass | Alzheimer's disease, wide (FinnGen) | rs9398171 | 6:108983527 | T/C | 0.711 | 0.0208 (0.0014) | 1.8E-52 | -0.0010 (0.0221) | 0.966 | 232.4 | -0.048 |
| Whole body fat-free mass | Alzheimer's disease, wide (FinnGen) | rs34879158 | 20:32300634 | C/A | 0.263 | -0.0215 (0.0014) | 2.2E-52 | -0.0178 (0.0251) | 0.477 | 232.0 | 0.826 |
| Whole body fat-free mass | Alzheimer's disease, wide (FinnGen) | rs2524137 | 6:31264582 | T/C | 0.696 | -0.0206 (0.0014) | 2.3E-52 | 0.0025 (0.0248) | 0.920 | 231.9 | -0.122 |
| Whole body fat-free mass | Alzheimer's disease, wide (FinnGen) | rs2533879 | 7:2859847 | A/G | 0.300 | -0.0206 (0.0014) | 3.4E-52 | 0.0275 (0.0220) | 0.211 | 231.1 | -1.336 |
| Whole body fat-free mass | Alzheimer's disease, wide (FinnGen) | rs7033487 | 9:119129257 | C/T | 0.198 | -0.0235 (0.0016) | 4.5E-51 | -0.0459 (0.0253) | 0.070 | 226.0 | 1.956 |
| Whole body fat-free mass | Alzheimer's disease, wide (FinnGen) | rs1582931 | 5:122657199 | A/G | 0.473 | -0.0188 (0.0013) | 8.7E-51 | 0.0051 (0.0212) | 0.811 | 224.7 | -0.272 |
| Whole body fat-free mass | Alzheimer's disease, wide (FinnGen) | rs1260326 | 2:27730940 | C/T | 0.604 | 0.0189 (0.0013) | 1.1E-50 | -0.0039 (0.0223) | 0.861 | 224.2 | -0.206 |
| Whole body fat-free mass | Alzheimer's disease, wide (FinnGen) | rs41271299 | 6:19839415 | T/C | 0.051 | 0.0419 (0.0028) | 2.3E-50 | 0.0636 (0.0841) | 0.450 | 222.7 | 1.519 |
| Whole body fat-free mass | Alzheimer's disease, wide (FinnGen) | rs7952436 | 11:67024534 | T/C | 0.082 | -0.0336 (0.0023) | 6.7E-50 | 0.0225 (0.0484) | 0.642 | 220.6 | -0.669 |
| Whole body fat-free mass | Alzheimer's disease, wide (FinnGen) | rs894360 | 8:135601194 | C/T | 0.366 | -0.0190 (0.0013) | 5.7E-49 | -0.0255 (0.0213) | 0.232 | 216.3 | 1.340 |
| Whole body fat-free mass | Alzheimer's disease, wide (FinnGen) | rs11243202 | 6:7719065 | C/T | 0.486 | 0.0183 (0.0012) | 6.3E-49 | 0.0139 (0.0213) | 0.514 | 216.2 | 0.760 |
| Whole body fat-free mass | Alzheimer's disease, wide (FinnGen) | rs6946415 | 7:150684548 | G/A | 0.627 | 0.0189 (0.0013) | 6.3E-49 | -0.0212 (0.0224) | 0.343 | 216.1 | -1.122 |
| Whole body fat-free mass | Alzheimer's disease, wide (FinnGen) | rs2885697 | 1:41544279 | T/G | 0.665 | -0.0189 (0.0013) | 5.1E-47 | 0.0308 (0.0224) | 0.170 | 207.4 | -1.634 |
| Whole body fat-free mass | Alzheimer's disease, wide (FinnGen) | rs72885917 | 2:172416376 | C/A | 0.247 | -0.0205 (0.0014) | 2.2E-46 | -0.0156 (0.0270) | 0.563 | 204.4 | 0.762 |
| Whole body fat-free mass | Alzheimer's disease, wide (FinnGen) | rs78378222 | 17:7571752 | G/T | 0.012 | 0.0820 (0.0058) | 7.4E-46 | -0.0500 (0.0833) | 0.548 | 202.1 | -0.610 |
| Whole body fat-free mass | Alzheimer's disease, wide (FinnGen) | rs73175572 | 3:185490184 | G/A | 0.112 | 0.0280 (0.0020) | 6.1E-45 | 0.0228 (0.0332) | 0.492 | 197.9 | 0.815 |
| Whole body fat-free mass | Alzheimer's disease, wide (FinnGen) | rs28620532 | 9:98216876 | G/A | 0.349 | 0.0182 (0.0013) | 6.4E-44 | 0.0118 (0.0220) | 0.591 | 193.2 | 0.649 |
| Whole body fat-free mass | Alzheimer's disease, wide (FinnGen) | rs7842996 | 8:78107140 | A/T | 0.284 | 0.0192 (0.0014) | 7.7E-44 | -0.0180 (0.0256) | 0.481 | 192.8 | -0.939 |
| Whole body fat-free mass | Alzheimer's disease, wide (FinnGen) | rs7129320 | 11:68388220 | A/G | 0.166 | -0.0231 (0.0017) | 1.1E-43 | 0.0730 (0.0376) | 0.052 | 192.1 | -3.156 |
| Whole body fat-free mass | Alzheimer's disease, wide (FinnGen) | rs71385734 | 16:2160503 | G/T | 0.170 | -0.0231 (0.0017) | 1.2E-43 | 0.0424 (0.0278) | 0.127 | 191.9 | -1.838 |
| Whole body fat-free mass | Alzheimer's disease, wide (FinnGen) | rs146851424 | 13:50377910 | C/A | 0.022 | 0.0597 (0.0043) | 1.6E-43 | -0.1613 (0.0809) | 0.046 | 191.3 | -2.703 |
| Whole body fat-free mass | Alzheimer's disease, wide (FinnGen) | rs62372052 | 5:42724294 | G/A | 0.110 | 0.0266 (0.0020) | 6.8E-41 | -0.0124 (0.0325) | 0.702 | 179.3 | -0.466 |
| Whole body fat-free mass | Alzheimer's disease, wide (FinnGen) | rs1478575 | 2:218278555 | A/T | 0.684 | 0.0175 (0.0013) | 1.7E-39 | -0.0252 (0.0215) | 0.240 | 172.9 | -1.438 |
| Whole body fat-free mass | Alzheimer's disease, wide (FinnGen) | rs2071286 | 6:32179896 | T/C | 0.182 | -0.0209 (0.0016) | 4.2E-39 | -0.0175 (0.0248) | 0.481 | 171.1 | 0.837 |
| Whole body fat-free mass | Alzheimer's disease, wide (FinnGen) | rs2101975 | 4:106216667 | G/A | 0.432 | -0.0164 (0.0013) | 7.4E-39 | -0.0156 (0.0213) | 0.463 | 170.0 | 0.953 |
| Whole body fat-free mass | Alzheimer's disease, wide (FinnGen) | rs62515437 | 8:57160328 | T/G | 0.225 | 0.0193 (0.0015) | 1.4E-38 | 0.0061 (0.0233) | 0.795 | 168.7 | 0.316 |
| Whole body fat-free mass | Alzheimer's disease, wide (FinnGen) | rs2307111 | 5:75003678 | C/T | 0.395 | -0.0164 (0.0013) | 2.1E-38 | 0.0057 (0.0215) | 0.792 | 167.9 | -0.347 |
| Whole body fat-free mass | Alzheimer's disease, wide (FinnGen) | rs1573891 | 15:99186488 | C/G | 0.158 | -0.0220 (0.0017) | 7.5E-38 | 0.0835 (0.0408) | 0.041 | 165.4 | -3.789 |
| Whole body fat-free mass | Alzheimer's disease, wide (FinnGen) | rs36000545 | 17:79093822 | G/A | 0.396 | -0.0166 (0.0013) | 8.2E-38 | 0.0089 (0.0225) | 0.694 | 165.2 | -0.538 |
| Whole body fat-free mass | Alzheimer's disease, wide (FinnGen) | rs485554 | 3:172137343 | C/G | 0.315 | 0.0171 (0.0013) | 1.8E-37 | -0.0222 (0.0226) | 0.328 | 163.7 | -1.296 |
| Whole body fat-free mass | Alzheimer's disease, wide (FinnGen) | rs3810291 | 19:47569003 | A/G | 0.675 | 0.0170 (0.0013) | 2.4E-37 | -0.0079 (0.0220) | 0.721 | 163.1 | -0.466 |
| Whole body fat-free mass | Alzheimer's disease, wide (FinnGen) | rs1047891 | 2:211540507 | A/C | 0.316 | 0.0169 (0.0013) | 6.0E-37 | -0.0433 (0.0228) | 0.057 | 161.3 | -2.564 |
| Whole body fat-free mass | Alzheimer's disease, wide (FinnGen) | rs72892910 | 6:50816887 | T/G | 0.172 | 0.0207 (0.0016) | 2.5E-36 | -0.0078 (0.0264) | 0.767 | 158.4 | -0.377 |
| Whole body fat-free mass | Alzheimer's disease, wide (FinnGen) | rs11030112 | 11:27705188 | A/G | 0.319 | 0.0167 (0.0013) | 3.6E-36 | 0.0013 (0.0225) | 0.955 | 157.7 | 0.078 |
| Whole body fat-free mass | Alzheimer's disease, wide (FinnGen) | rs59985551 | 2:56106928 | T/C | 0.226 | -0.0185 (0.0015) | 8.6E-36 | 0.0204 (0.0254) | 0.421 | 156.0 | -1.106 |
| Whole body fat-free mass | Alzheimer's disease, wide (FinnGen) | rs4282339 | 5:168256240 | A/G | 0.208 | -0.0189 (0.0015) | 2.4E-35 | -0.0233 (0.0271) | 0.391 | 153.9 | 1.231 |
| Whole body fat-free mass | Alzheimer's disease, wide (FinnGen) | rs7132908 | 12:50263148 | A/G | 0.384 | 0.0158 (0.0013) | 2.9E-35 | -0.0159 (0.0219) | 0.467 | 153.6 | -1.006 |
| Whole body fat-free mass | Alzheimer's disease, wide (FinnGen) | rs12713004 | 2:23896049 | G/A | 0.725 | 0.0172 (0.0014) | 3.9E-35 | 0.0076 (0.0278) | 0.785 | 153.0 | 0.443 |
| Whole body fat-free mass | Alzheimer's disease, wide (FinnGen) | rs10773172 | 12:122939655 | A/G | 0.742 | 0.0175 (0.0014) | 6.6E-35 | 0.0402 (0.0269) | 0.135 | 151.9 | 2.299 |
| Whole body fat-free mass | Alzheimer's disease, wide (FinnGen) | rs35506085 | 11:2165576 | A/G | 0.185 | -0.0198 (0.0016) | 8.1E-35 | -0.0308 (0.0282) | 0.275 | 151.5 | 1.552 |
| Whole body fat-free mass | Alzheimer's disease, wide (FinnGen) | rs25849 | 16:2265071 | G/C | 0.289 | 0.0169 (0.0014) | 1.3E-34 | -0.0132 (0.0221) | 0.551 | 150.5 | -0.782 |
| Whole body fat-free mass | Alzheimer's disease, wide (FinnGen) | rs2897968 | 12:46763686 | A/G | 0.607 | 0.0156 (0.0013) | 1.4E-34 | -0.0215 (0.0216) | 0.319 | 150.4 | -1.378 |
| Whole body fat-free mass | Alzheimer's disease, wide (FinnGen) | rs3853252 | 6:152170247 | A/G | 0.455 | 0.0153 (0.0012) | 1.7E-34 | -0.0158 (0.0216) | 0.463 | 150.0 | -1.033 |
| Whole body fat-free mass | Alzheimer's disease, wide (FinnGen) | rs9372837 | 6:126638124 | A/G | 0.471 | 0.0152 (0.0013) | 6.8E-34 | 0.0067 (0.0213) | 0.751 | 147.3 | 0.441 |
| Whole body fat-free mass | Alzheimer's disease, wide (FinnGen) | rs12314162 | 12:124826676 | T/C | 0.161 | 0.0204 (0.0017) | 1.5E-33 | -0.0068 (0.0299) | 0.820 | 145.7 | -0.334 |
| Whole body fat-free mass | Alzheimer's disease, wide (FinnGen) | rs9892365 | 17:59491384 | G/A | 0.670 | -0.0158 (0.0013) | 8.9E-33 | 0.0025 (0.0239) | 0.917 | 142.2 | -0.158 |
| Whole body fat-free mass | Alzheimer's disease, wide (FinnGen) | rs34776209 | 7:23513093 | T/C | 0.247 | -0.0171 (0.0014) | 1.5E-32 | -0.0152 (0.0262) | 0.561 | 141.1 | 0.888 |
| Whole body fat-free mass | Alzheimer's disease, wide (FinnGen) | rs244711 | 5:176509193 | T/C | 0.686 | 0.0168 (0.0014) | 1.2E-31 | -0.0428 (0.0230) | 0.064 | 136.9 | -2.552 |
| Whole body fat-free mass | Alzheimer's disease, wide (FinnGen) | rs13043303 | 20:51091385 | A/G | 0.183 | -0.0186 (0.0016) | 6.2E-31 | 0.0009 (0.0292) | 0.977 | 133.7 | -0.048 |
| Whole body fat-free mass | Alzheimer's disease, wide (FinnGen) | rs41311445 | 22:42070374 | C/A | 0.096 | -0.0244 (0.0021) | 1.6E-30 | 0.0073 (0.0311) | 0.813 | 131.9 | -0.300 |
| Whole body fat-free mass | Alzheimer's disease, wide (FinnGen) | rs2531991 | 16:4023553 | A/G | 0.747 | 0.0164 (0.0014) | 2.3E-30 | -0.0210 (0.0240) | 0.383 | 131.1 | -1.279 |
| Whole body fat-free mass | Alzheimer's disease, wide (FinnGen) | rs6235 | 5:95728898 | G/C | 0.268 | 0.0159 (0.0014) | 8.3E-30 | -0.0218 (0.0235) | 0.355 | 128.6 | -1.374 |
| Whole body fat-free mass | Alzheimer's disease, wide (FinnGen) | rs11014285 | 10:25178864 | A/G | 0.165 | 0.0192 (0.0017) | 1.1E-29 | -0.0153 (0.0354) | 0.666 | 128.1 | -0.798 |
| Whole body fat-free mass | Alzheimer's disease, wide (FinnGen) | rs1022523 | 1:176788394 | A/G | 0.727 | 0.0156 (0.0014) | 4.4E-29 | 0.0174 (0.0232) | 0.454 | 125.3 | 1.118 |
| Whole body fat-free mass | Alzheimer's disease, wide (FinnGen) | rs12072845 | 1:214630757 | A/G | 0.395 | -0.0140 (0.0013) | 2.8E-28 | -0.0108 (0.0223) | 0.628 | 121.7 | 0.773 |
| Whole body fat-free mass | Alzheimer's disease, wide (FinnGen) | rs11880992 | 19:2176403 | A/G | 0.408 | 0.0139 (0.0013) | 4.6E-28 | -0.0087 (0.0221) | 0.693 | 120.6 | -0.625 |
| Whole body fat-free mass | Alzheimer's disease, wide (FinnGen) | rs2270894 | 3:9975386 | G/C | 0.203 | -0.0174 (0.0016) | 1.0E-27 | -0.0224 (0.0242) | 0.354 | 119.0 | 1.284 |
| Whole body fat-free mass | Alzheimer's disease, wide (FinnGen) | rs111640872 | 19:30290357 | C/G | 0.331 | 0.0145 (0.0013) | 1.1E-27 | 0.0014 (0.0223) | 0.949 | 118.9 | 0.097 |
| Whole body fat-free mass | Alzheimer's disease, wide (FinnGen) | rs6800021 | 3:50190346 | A/G | 0.425 | 0.0136 (0.0013) | 1.6E-27 | 0.0815 (0.0228) | 3.4E-04 | 118.1 | 5.976 |
| Whole body fat-free mass | Alzheimer's disease, wide (FinnGen) | rs11065979 | 12:112059557 | T/C | 0.438 | -0.0135 (0.0013) | 4.3E-27 | 0.0279 (0.0217) | 0.200 | 116.2 | -2.069 |
| Whole body fat-free mass | Alzheimer's disease, wide (FinnGen) | rs17277008 | 1:172105162 | C/T | 0.313 | 0.0144 (0.0013) | 4.6E-27 | 0.0171 (0.0242) | 0.481 | 116.1 | 1.191 |
| Whole body fat-free mass | Alzheimer's disease, wide (FinnGen) | rs2900208 | 12:11878464 | A/C | 0.354 | 0.0140 (0.0013) | 4.9E-27 | -0.0214 (0.0218) | 0.327 | 115.9 | -1.530 |
| Whole body fat-free mass | Alzheimer's disease, wide (FinnGen) | rs12657771 | 5:36787962 | A/G | 0.439 | -0.0134 (0.0013) | 9.4E-27 | 0.0034 (0.0215) | 0.874 | 114.7 | -0.253 |
| Whole body fat-free mass | Alzheimer's disease, wide (FinnGen) | rs12879423 | 14:25927832 | G/A | 0.679 | 0.0142 (0.0013) | 3.4E-26 | 0.0337 (0.0220) | 0.126 | 112.1 | 2.376 |
| Whole body fat-free mass | Alzheimer's disease, wide (FinnGen) | rs6762578 | 3:128992047 | A/G | 0.778 | 0.0157 (0.0015) | 8.9E-26 | 0.0077 (0.0283) | 0.786 | 110.2 | 0.490 |
| Whole body fat-free mass | Alzheimer's disease, wide (FinnGen) | rs7141420 | 14:79899454 | T/C | 0.516 | 0.0131 (0.0013) | 1.2E-25 | -0.0070 (0.0215) | 0.744 | 109.5 | -0.534 |
| Whole body fat-free mass | Alzheimer's disease, wide (FinnGen) | rs3822742 | 5:139059017 | A/C | 0.371 | 0.0134 (0.0013) | 1.6E-25 | -0.0294 (0.0227) | 0.195 | 109.0 | -2.188 |
| Whole body fat-free mass | Alzheimer's disease, wide (FinnGen) | rs12041740 | 1:11236524 | A/G | 0.740 | -0.0147 (0.0014) | 2.1E-25 | 0.0180 (0.0237) | 0.448 | 108.5 | -1.227 |
| Whole body fat-free mass | Alzheimer's disease, wide (FinnGen) | rs12095997 | 1:51391845 | T/C | 0.089 | 0.0225 (0.0022) | 2.7E-25 | -0.0108 (0.0461) | 0.815 | 108.0 | -0.480 |
| Whole body fat-free mass | Alzheimer's disease, wide (FinnGen) | rs6570509 | 6:142716286 | T/G | 0.287 | -0.0142 (0.0014) | 3.9E-25 | -0.0084 (0.0243) | 0.730 | 107.3 | 0.593 |
| Whole body fat-free mass | Alzheimer's disease, wide (FinnGen) | rs7080472 | 10:96012950 | T/G | 0.425 | 0.0130 (0.0013) | 5.2E-25 | -0.0019 (0.0226) | 0.932 | 106.7 | -0.146 |
| Whole body fat-free mass | Alzheimer's disease, wide (FinnGen) | rs1924936 | 13:78443297 | A/T | 0.774 | 0.0153 (0.0015) | 7.8E-25 | -0.0028 (0.0275) | 0.919 | 105.9 | -0.183 |
| Whole body fat-free mass | Alzheimer's disease, wide (FinnGen) | rs10283100 | 8:120596023 | G/A | 0.945 | 0.0279 (0.0027) | 8.7E-25 | -0.0258 (0.0411) | 0.530 | 105.7 | -0.925 |
| Whole body fat-free mass | Alzheimer's disease, wide (FinnGen) | rs11240565 | 1:205722958 | T/C | 0.398 | 0.0130 (0.0013) | 8.8E-25 | -0.0360 (0.0220) | 0.102 | 105.6 | -2.775 |
| Whole body fat-free mass | Alzheimer's disease, wide (FinnGen) | rs365352 | 5:77401152 | A/G | 0.244 | -0.0148 (0.0014) | 9.3E-25 | 0.0165 (0.0273) | 0.546 | 105.5 | -1.115 |
| Whole body fat-free mass | Alzheimer's disease, wide (FinnGen) | rs9938120 | 16:19977733 | T/C | 0.144 | -0.0184 (0.0018) | 9.4E-25 | -0.0273 (0.0322) | 0.397 | 105.5 | 1.482 |
| Whole body fat-free mass | Alzheimer's disease, wide (FinnGen) | rs9350850 | 6:81050236 | C/T | 0.079 | 0.0234 (0.0023) | 1.5E-24 | 0.0163 (0.0336) | 0.628 | 104.6 | 0.697 |
| Whole body fat-free mass | Alzheimer's disease, wide (FinnGen) | rs946197 | 1:56602308 | C/G | 0.232 | 0.0150 (0.0015) | 1.6E-24 | -0.0179 (0.0251) | 0.476 | 104.5 | -1.193 |
| Whole body fat-free mass | Alzheimer's disease, wide (FinnGen) | rs11613704 | 12:980925 | A/C | 0.200 | 0.0158 (0.0015) | 2.1E-24 | -0.0004 (0.0265) | 0.989 | 103.9 | -0.025 |
| Whole body fat-free mass | Alzheimer's disease, wide (FinnGen) | rs12193797 | 6:41748352 | G/A | 0.133 | -0.0185 (0.0018) | 2.9E-24 | 0.0067 (0.0396) | 0.865 | 103.3 | -0.361 |
| Whole body fat-free mass | Alzheimer's disease, wide (FinnGen) | rs2252720 | 20:21223663 | T/C | 0.674 | -0.0135 (0.0013) | 4.6E-24 | 0.0314 (0.0236) | 0.184 | 102.4 | -2.320 |
| Whole body fat-free mass | Alzheimer's disease, wide (FinnGen) | rs10119967 | 9:99268421 | C/A | 0.204 | 0.0156 (0.0015) | 5.9E-24 | -0.0641 (0.0279) | 0.022 | 101.9 | -4.112 |
| Whole body fat-free mass | Alzheimer's disease, wide (FinnGen) | rs2163832 | 19:10745764 | C/T | 0.660 | -0.0133 (0.0013) | 6.4E-24 | 0.0178 (0.0235) | 0.450 | 101.7 | -1.341 |
| Whole body fat-free mass | Alzheimer's disease, wide (FinnGen) | rs676105 | 11:30443688 | C/T | 0.304 | 0.0136 (0.0014) | 7.8E-24 | 0.0191 (0.0231) | 0.409 | 101.3 | 1.401 |
| Whole body fat-free mass | Alzheimer's disease, wide (FinnGen) | rs9480933 | 6:109671391 | G/C | 0.350 | -0.0130 (0.0013) | 9.3E-24 | 0.0336 (0.0218) | 0.124 | 101.0 | -2.575 |
| Whole body fat-free mass | Alzheimer's disease, wide (FinnGen) | rs11648796 | 16:792190 | G/A | 0.232 | 0.0154 (0.0015) | 1.4E-23 | -0.0011 (0.0228) | 0.960 | 100.1 | -0.071 |
| Whole body fat-free mass | Alzheimer's disease, wide (FinnGen) | rs2265309 | 10:104487871 | C/T | 0.517 | -0.0124 (0.0012) | 1.5E-23 | 0.0058 (0.0213) | 0.785 | 100.0 | -0.466 |
| Whole body fat-free mass | Alzheimer's disease, wide (FinnGen) | rs13271368 | 8:126506140 | T/C | 0.237 | -0.0148 (0.0015) | 2.0E-23 | 0.0159 (0.0232) | 0.493 | 99.4 | -1.077 |
| Whole body fat-free mass | Alzheimer's disease, wide (FinnGen) | rs7815955 | 8:130719567 | T/A | 0.203 | -0.0152 (0.0015) | 7.8E-23 | 0.0055 (0.0279) | 0.842 | 96.8 | -0.362 |
| Whole body fat-free mass | Alzheimer's disease, wide (FinnGen) | rs6779752 | 3:85663849 | A/G | 0.638 | -0.0128 (0.0013) | 8.5E-23 | -0.0302 (0.0236) | 0.201 | 96.6 | 2.366 |
| Whole body fat-free mass | Alzheimer's disease, wide (FinnGen) | rs111365325 | 5:170865229 | T/C | 0.231 | -0.0145 (0.0015) | 9.6E-23 | 0.0233 (0.0264) | 0.377 | 96.3 | -1.612 |
| Whole body fat-free mass | Alzheimer's disease, wide (FinnGen) | rs33973388 | 18:46611842 | T/G | 0.435 | 0.0123 (0.0013) | 9.7E-23 | 0.0127 (0.0231) | 0.583 | 96.3 | 1.030 |
| Whole body fat-free mass | Alzheimer's disease, wide (FinnGen) | rs11578046 | 1:23425139 | A/G | 0.327 | -0.0129 (0.0013) | 1.1E-22 | -0.0096 (0.0226) | 0.672 | 96.1 | 0.742 |
| Whole body fat-free mass | Alzheimer's disease, wide (FinnGen) | rs67551338 | 12:3393100 | T/C | 0.061 | 0.0255 (0.0026) | 1.4E-22 | -0.0374 (0.0547) | 0.494 | 95.6 | -1.465 |
| Whole body fat-free mass | Alzheimer's disease, wide (FinnGen) | rs3809570 | 15:67000117 | A/C | 0.243 | 0.0142 (0.0015) | 2.1E-22 | -0.0133 (0.0255) | 0.601 | 94.8 | -0.940 |
| Whole body fat-free mass | Alzheimer's disease, wide (FinnGen) | rs16942324 | 15:89383854 | A/C | 0.027 | -0.0373 (0.0038) | 2.2E-22 | 0.0773 (0.0995) | 0.437 | 94.7 | -2.074 |
| Whole body fat-free mass | Alzheimer's disease, wide (FinnGen) | rs34848742 | 4:123828042 | G/T | 0.788 | -0.0148 (0.0015) | 2.3E-22 | 0.0085 (0.0275) | 0.758 | 94.7 | -0.576 |
| Whole body fat-free mass | Alzheimer's disease, wide (FinnGen) | rs13240065 | 7:73015369 | A/G | 0.128 | 0.0180 (0.0019) | 2.9E-22 | 0.0066 (0.0316) | 0.836 | 94.2 | 0.367 |
| Whole body fat-free mass | Alzheimer's disease, wide (FinnGen) | rs73052033 | 3:185828465 | C/T | 0.185 | -0.0154 (0.0016) | 5.2E-22 | 0.0246 (0.0288) | 0.394 | 93.0 | -1.596 |
| Whole body fat-free mass | Alzheimer's disease, wide (FinnGen) | rs7780752 | 7:93241640 | C/T | 0.360 | 0.0124 (0.0013) | 5.4E-22 | -0.0223 (0.0240) | 0.353 | 92.9 | -1.792 |
| Whole body fat-free mass | Alzheimer's disease, wide (FinnGen) | rs9291926 | 5:67599656 | G/T | 0.532 | -0.0120 (0.0012) | 6.1E-22 | 0.0192 (0.0213) | 0.365 | 92.7 | -1.604 |
| Whole body fat-free mass | Alzheimer's disease, wide (FinnGen) | rs2721938 | 8:116635611 | T/C | 0.600 | -0.0121 (0.0013) | 1.3E-21 | 0.0134 (0.0234) | 0.567 | 91.2 | -1.104 |
| Whole body fat-free mass | Alzheimer's disease, wide (FinnGen) | rs13430869 | 2:218146818 | T/G | 0.742 | 0.0135 (0.0014) | 1.4E-21 | -0.0061 (0.0221) | 0.784 | 91.1 | -0.452 |
| Whole body fat-free mass | Alzheimer's disease, wide (FinnGen) | rs62621197 | 19:8670147 | T/C | 0.037 | -0.0326 (0.0034) | 1.4E-21 | 0.1982 (0.0763) | 0.009 | 91.0 | -6.083 |
| Whole body fat-free mass | Alzheimer's disease, wide (FinnGen) | rs10938397 | 4:45182527 | G/A | 0.434 | 0.0119 (0.0013) | 1.7E-21 | -0.0146 (0.0212) | 0.492 | 90.6 | -1.225 |
| Whole body fat-free mass | Alzheimer's disease, wide (FinnGen) | rs10775348 | 16:88806348 | G/A | 0.704 | 0.0130 (0.0014) | 2.0E-21 | -0.0148 (0.0232) | 0.525 | 90.4 | -1.141 |
| Whole body fat-free mass | Alzheimer's disease, wide (FinnGen) | rs11042725 | 11:10325325 | A/C | 0.488 | -0.0117 (0.0012) | 3.1E-21 | -0.0219 (0.0212) | 0.302 | 89.5 | 1.865 |
| Whole body fat-free mass | Alzheimer's disease, wide (FinnGen) | rs10748128 | 12:69827658 | T/G | 0.345 | 0.0123 (0.0013) | 3.2E-21 | 0.0072 (0.0222) | 0.746 | 89.5 | 0.583 |
| Whole body fat-free mass | Alzheimer's disease, wide (FinnGen) | rs7619139 | 3:25110415 | A/T | 0.589 | 0.0119 (0.0013) | 3.4E-21 | -0.0334 (0.0216) | 0.122 | 89.3 | -2.800 |
| Whole body fat-free mass | Alzheimer's disease, wide (FinnGen) | rs247008 | 5:131447104 | G/A | 0.670 | 0.0125 (0.0013) | 3.6E-21 | -0.0199 (0.0218) | 0.362 | 89.2 | -1.588 |
| Whole body fat-free mass | Alzheimer's disease, wide (FinnGen) | rs62621812 | 7:127015083 | A/G | 0.020 | 0.0425 (0.0045) | 5.9E-21 | -0.0435 (0.0529) | 0.411 | 88.2 | -1.023 |
| Whole body fat-free mass | Alzheimer's disease, wide (FinnGen) | rs4968799 | 17:68158831 | T/A | 0.112 | -0.0185 (0.0020) | 7.3E-21 | 0.0005 (0.0362) | 0.988 | 87.8 | -0.027 |
| Whole body fat-free mass | Alzheimer's disease, wide (FinnGen) | rs2726036 | 16:28347140 | C/A | 0.398 | 0.0119 (0.0013) | 8.0E-21 | -0.0211 (0.0214) | 0.323 | 87.6 | -1.772 |
| Whole body fat-free mass | Alzheimer's disease, wide (FinnGen) | rs2952615 | 5:112138888 | C/G | 0.618 | -0.0119 (0.0013) | 8.9E-21 | 0.0286 (0.0216) | 0.185 | 87.4 | -2.396 |
| Whole body fat-free mass | Alzheimer's disease, wide (FinnGen) | rs17246129 | 2:227259964 | A/G | 0.305 | 0.0125 (0.0013) | 1.4E-20 | -0.0326 (0.0238) | 0.170 | 86.5 | -2.604 |
| Whole body fat-free mass | Alzheimer's disease, wide (FinnGen) | rs9461246 | 6:26381660 | A/G | 0.118 | -0.0178 (0.0019) | 1.5E-20 | 0.0023 (0.0299) | 0.939 | 86.3 | -0.129 |
| Whole body fat-free mass | Alzheimer's disease, wide (FinnGen) | rs1064213 | 2:198950240 | A/G | 0.478 | 0.0115 (0.0012) | 1.8E-20 | 0.0105 (0.0217) | 0.629 | 86.0 | 0.915 |
| Whole body fat-free mass | Alzheimer's disease, wide (FinnGen) | rs2015561 | 17:38593294 | G/A | 0.424 | -0.0117 (0.0013) | 1.9E-20 | 0.0116 (0.0214) | 0.586 | 85.9 | -0.996 |
| Whole body fat-free mass | Alzheimer's disease, wide (FinnGen) | rs1542224 | 2:223963874 | C/T | 0.719 | 0.0128 (0.0014) | 2.0E-20 | -0.0061 (0.0241) | 0.801 | 85.8 | -0.478 |
| Whole body fat-free mass | Alzheimer's disease, wide (FinnGen) | rs2678204 | 1:201800511 | G/T | 0.340 | 0.0121 (0.0013) | 2.4E-20 | -0.0117 (0.0233) | 0.616 | 85.4 | -0.969 |
| Whole body fat-free mass | Alzheimer's disease, wide (FinnGen) | rs3212260 | 14:24804088 | T/A | 0.258 | 0.0131 (0.0014) | 3.6E-20 | -0.0088 (0.0255) | 0.731 | 84.6 | -0.672 |
| Whole body fat-free mass | Alzheimer's disease, wide (FinnGen) | rs663344 | 9:98316331 | C/G | 0.181 | -0.0151 (0.0016) | 3.7E-20 | -0.0042 (0.0271) | 0.877 | 84.6 | 0.279 |
| Whole body fat-free mass | Alzheimer's disease, wide (FinnGen) | rs6585827 | 10:124165615 | A/G | 0.471 | 0.0114 (0.0012) | 3.8E-20 | -0.0863 (0.0220) | 8.6E-05 | 84.5 | -7.550 |
| Whole body fat-free mass | Alzheimer's disease, wide (FinnGen) | rs1910252 | 8:49407362 | T/C | 0.168 | 0.0153 (0.0017) | 3.9E-20 | -0.0110 (0.0308) | 0.721 | 84.5 | -0.719 |
| Whole body fat-free mass | Alzheimer's disease, wide (FinnGen) | rs703593 | 12:103064247 | G/A | 0.482 | -0.0114 (0.0012) | 4.1E-20 | 0.0268 (0.0214) | 0.210 | 84.4 | -2.351 |
| Whole body fat-free mass | Alzheimer's disease, wide (FinnGen) | rs55831773 | 17:7559037 | T/C | 0.199 | -0.0146 (0.0016) | 4.1E-20 | -0.0615 (0.0304) | 0.043 | 84.4 | 4.219 |
| Whole body fat-free mass | Alzheimer's disease, wide (FinnGen) | rs115179432 | 2:33348679 | G/A | 0.072 | -0.0221 (0.0024) | 4.5E-20 | -0.0832 (0.0454) | 0.067 | 84.2 | 3.767 |
| Whole body fat-free mass | Alzheimer's disease, wide (FinnGen) | rs55727637 | 16:15145252 | T/C | 0.305 | -0.0123 (0.0013) | 6.1E-20 | 0.0111 (0.0230) | 0.631 | 83.6 | -0.900 |
| Whole body fat-free mass | Alzheimer's disease, wide (FinnGen) | rs31211 | 5:134363145 | A/G | 0.251 | -0.0130 (0.0014) | 7.2E-20 | -0.0337 (0.0242) | 0.164 | 83.2 | 2.583 |
| Whole body fat-free mass | Alzheimer's disease, wide (FinnGen) | rs2140046 | 2:169706079 | C/T | 0.364 | -0.0117 (0.0013) | 8.2E-20 | -0.0034 (0.0215) | 0.875 | 83.0 | 0.290 |
| Whole body fat-free mass | Alzheimer's disease, wide (FinnGen) | rs11778491 | 8:120451362 | C/G | 0.252 | -0.0130 (0.0014) | 9.2E-20 | 0.0465 (0.0272) | 0.087 | 82.8 | -3.574 |
| Whole body fat-free mass | Alzheimer's disease, wide (FinnGen) | rs3740591 | 10:70287303 | T/C | 0.445 | 0.0114 (0.0013) | 1.7E-19 | -0.0238 (0.0225) | 0.290 | 81.5 | -2.088 |
| Whole body fat-free mass | Alzheimer's disease, wide (FinnGen) | rs4980067 | 10:81136129 | A/C | 0.505 | -0.0112 (0.0012) | 1.8E-19 | -0.0138 (0.0214) | 0.520 | 81.4 | 1.232 |
| Whole body fat-free mass | Alzheimer's disease, wide (FinnGen) | rs1841738 | 4:88557753 | G/A | 0.518 | -0.0112 (0.0012) | 3.2E-19 | 0.0320 (0.0219) | 0.143 | 80.3 | -2.861 |
| Whole body fat-free mass | Alzheimer's disease, wide (FinnGen) | rs10202845 | 2:42575820 | G/A | 0.113 | -0.0176 (0.0020) | 4.3E-19 | -0.0247 (0.0349) | 0.478 | 79.7 | 1.402 |
| Whole body fat-free mass | Alzheimer's disease, wide (FinnGen) | rs11001399 | 10:77221744 | C/A | 0.559 | -0.0112 (0.0013) | 5.2E-19 | -0.0132 (0.0214) | 0.537 | 79.4 | 1.180 |
| Whole body fat-free mass | Alzheimer's disease, wide (FinnGen) | rs843374 | 3:183997261 | T/A | 0.587 | -0.0112 (0.0013) | 5.4E-19 | 0.0183 (0.0217) | 0.398 | 79.3 | -1.632 |
| Whole body fat-free mass | Alzheimer's disease, wide (FinnGen) | rs7706886 | 5:32722319 | A/G | 0.271 | 0.0126 (0.0014) | 6.2E-19 | -0.0292 (0.0251) | 0.243 | 79.0 | -2.315 |
| Whole body fat-free mass | Alzheimer's disease, wide (FinnGen) | rs10803955 | 2:183228114 | G/A | 0.508 | -0.0110 (0.0012) | 6.4E-19 | -0.0202 (0.0212) | 0.340 | 79.0 | 1.840 |
| Whole body fat-free mass | Alzheimer's disease, wide (FinnGen) | rs111391498 | 4:1341553 | G/A | 0.047 | -0.0258 (0.0029) | 6.7E-19 | 0.0836 (0.0625) | 0.181 | 78.8 | -3.235 |
| Whole body fat-free mass | Alzheimer's disease, wide (FinnGen) | rs4865956 | 5:54882505 | A/T | 0.697 | -0.0120 (0.0014) | 7.3E-19 | 0.0130 (0.0236) | 0.582 | 78.7 | -1.083 |
| Whole body fat-free mass | Alzheimer's disease, wide (FinnGen) | rs28547519 | 7:121964618 | C/T | 0.366 | 0.0114 (0.0013) | 8.5E-19 | 0.0289 (0.0218) | 0.185 | 78.4 | 2.536 |
| Whole body fat-free mass | Alzheimer's disease, wide (FinnGen) | rs7647657 | 3:38573746 | G/A | 0.386 | -0.0113 (0.0013) | 9.1E-19 | 0.0089 (0.0216) | 0.681 | 78.2 | -0.788 |
| Whole body fat-free mass | Alzheimer's disease, wide (FinnGen) | rs2602713 | 19:4910021 | C/A | 0.440 | 0.0111 (0.0013) | 1.2E-18 | -0.0140 (0.0220) | 0.524 | 77.6 | -1.256 |
| Whole body fat-free mass | Alzheimer's disease, wide (FinnGen) | rs6874142 | 5:172753555 | G/T | 0.114 | 0.0182 (0.0021) | 1.4E-18 | -0.0689 (0.0368) | 0.061 | 77.5 | -3.787 |
| Whole body fat-free mass | Alzheimer's disease, wide (FinnGen) | rs76364830 | 8:13372120 | A/G | 0.063 | -0.0225 (0.0026) | 2.4E-18 | 0.0182 (0.0582) | 0.754 | 76.4 | -0.810 |
| Whole body fat-free mass | Alzheimer's disease, wide (FinnGen) | rs17556750 | 4:82155568 | A/C | 0.288 | 0.0119 (0.0014) | 3.7E-18 | 0.0141 (0.0220) | 0.524 | 75.5 | 1.185 |
| Whole body fat-free mass | Alzheimer's disease, wide (FinnGen) | rs11712872 | 3:52835514 | A/G | 0.115 | 0.0168 (0.0019) | 4.3E-18 | 0.0050 (0.0372) | 0.893 | 75.2 | 0.298 |
| Whole body fat-free mass | Alzheimer's disease, wide (FinnGen) | rs7728690 | 5:88411214 | T/C | 0.386 | -0.0110 (0.0013) | 5.0E-18 | -0.0047 (0.0228) | 0.838 | 74.9 | 0.427 |
| Whole body fat-free mass | Alzheimer's disease, wide (FinnGen) | rs76513770 | 16:72505534 | C/T | 0.128 | -0.0161 (0.0019) | 5.1E-18 | 0.0178 (0.0285) | 0.532 | 74.8 | -1.106 |
| Whole body fat-free mass | Alzheimer's disease, wide (FinnGen) | rs2281175 | 1:1665702 | C/T | 0.405 | 0.0114 (0.0013) | 6.3E-18 | 0.0093 (0.0216) | 0.666 | 74.4 | 0.813 |
| Whole body fat-free mass | Alzheimer's disease, wide (FinnGen) | rs3116201 | 2:233074205 | A/G | 0.098 | -0.0180 (0.0021) | 7.1E-18 | -0.0167 (0.0491) | 0.734 | 74.2 | 0.930 |
| Whole body fat-free mass | Alzheimer's disease, wide (FinnGen) | rs508347 | 7:28212824 | C/T | 0.703 | -0.0117 (0.0014) | 7.8E-18 | -0.0200 (0.0222) | 0.369 | 74.0 | 1.712 |
| Whole body fat-free mass | Alzheimer's disease, wide (FinnGen) | rs10777860 | 12:97792792 | A/G | 0.528 | -0.0107 (0.0012) | 7.8E-18 | 0.0017 (0.0214) | 0.938 | 74.0 | -0.159 |
| Whole body fat-free mass | Alzheimer's disease, wide (FinnGen) | rs744205 | 11:69929677 | A/G | 0.544 | 0.0107 (0.0012) | 1.0E-17 | 0.0038 (0.0213) | 0.859 | 73.5 | 0.355 |
| Whole body fat-free mass | Alzheimer's disease, wide (FinnGen) | rs7134283 | 12:24071748 | A/G | 0.283 | -0.0118 (0.0014) | 1.2E-17 | 0.0008 (0.0237) | 0.973 | 73.2 | -0.068 |
| Whole body fat-free mass | Alzheimer's disease, wide (FinnGen) | rs822549 | 7:148649180 | C/T | 0.267 | 0.0120 (0.0014) | 1.4E-17 | -0.0488 (0.0234) | 0.037 | 72.9 | -4.074 |
| Whole body fat-free mass | Alzheimer's disease, wide (FinnGen) | rs61729527 | 8:77761919 | T/C | 0.052 | -0.0238 (0.0028) | 1.9E-17 | -0.0182 (0.0430) | 0.672 | 72.3 | 0.764 |
| Whole body fat-free mass | Alzheimer's disease, wide (FinnGen) | rs968821 | 9:94187634 | C/G | 0.340 | -0.0111 (0.0013) | 2.1E-17 | -0.0021 (0.0240) | 0.930 | 72.1 | 0.189 |
| Whole body fat-free mass | Alzheimer's disease, wide (FinnGen) | rs9317002 | 13:59175727 | A/C | 0.515 | 0.0106 (0.0013) | 2.3E-17 | 0.0006 (0.0213) | 0.978 | 71.8 | 0.056 |
| Whole body fat-free mass | Alzheimer's disease, wide (FinnGen) | rs9951619 | 18:56882326 | G/T | 0.767 | 0.0125 (0.0015) | 4.0E-17 | 0.0039 (0.0227) | 0.864 | 70.8 | 0.313 |
| Whole body fat-free mass | Alzheimer's disease, wide (FinnGen) | rs3782232 | 12:57116249 | A/G | 0.071 | -0.0203 (0.0024) | 4.4E-17 | 0.0783 (0.0387) | 0.043 | 70.6 | -3.859 |
| Whole body fat-free mass | Alzheimer's disease, wide (FinnGen) | rs12188627 | 5:60720682 | G/A | 0.488 | -0.0104 (0.0012) | 6.3E-17 | 0.0062 (0.0212) | 0.771 | 69.9 | -0.598 |
| Whole body fat-free mass | Alzheimer's disease, wide (FinnGen) | rs11689727 | 2:25458100 | A/C | 0.331 | -0.0110 (0.0013) | 7.0E-17 | 0.0024 (0.0228) | 0.918 | 69.7 | -0.218 |
| Whole body fat-free mass | Alzheimer's disease, wide (FinnGen) | rs7312646 | 12:102388920 | C/A | 0.521 | -0.0105 (0.0013) | 9.2E-17 | 0.0188 (0.0213) | 0.379 | 69.1 | -1.787 |
| Whole body fat-free mass | Alzheimer's disease, wide (FinnGen) | rs6902789 | 6:105358192 | A/G | 0.368 | 0.0107 (0.0013) | 1.1E-16 | 0.0280 (0.0220) | 0.204 | 68.8 | 2.618 |
| Whole body fat-free mass | Alzheimer's disease, wide (FinnGen) | rs4858940 | 3:88254820 | C/T | 0.886 | 0.0161 (0.0019) | 1.2E-16 | 0.0034 (0.0352) | 0.922 | 68.6 | 0.211 |
| Whole body fat-free mass | Alzheimer's disease, wide (FinnGen) | rs2296316 | 14:65520246 | C/T | 0.465 | -0.0104 (0.0013) | 1.2E-16 | -0.0191 (0.0212) | 0.367 | 68.6 | 1.836 |
| Whole body fat-free mass | Alzheimer's disease, wide (FinnGen) | rs112560164 | 14:93112924 | A/G | 0.191 | 0.0131 (0.0016) | 1.3E-16 | 0.0498 (0.0306) | 0.103 | 68.5 | 3.793 |
| Whole body fat-free mass | Alzheimer's disease, wide (FinnGen) | rs9380508 | 6:35398689 | T/C | 0.775 | 0.0123 (0.0015) | 1.4E-16 | -0.0152 (0.0327) | 0.642 | 68.3 | -1.239 |
| Whole body fat-free mass | Alzheimer's disease, wide (FinnGen) | rs61992671 | 14:101531854 | G/A | 0.492 | -0.0107 (0.0013) | 1.4E-16 | -0.0085 (0.0218) | 0.696 | 68.2 | 0.791 |
| Whole body fat-free mass | Alzheimer's disease, wide (FinnGen) | rs1228024 | 11:47951353 | A/C | 0.661 | -0.0108 (0.0013) | 2.1E-16 | -0.0073 (0.0229) | 0.751 | 67.5 | 0.677 |
| Whole body fat-free mass | Alzheimer's disease, wide (FinnGen) | rs310796 | 12:77453226 | T/G | 0.681 | 0.0110 (0.0013) | 2.1E-16 | 0.0200 (0.0241) | 0.406 | 67.5 | 1.824 |
| Whole body fat-free mass | Alzheimer's disease, wide (FinnGen) | rs11794152 | 9:23345347 | G/A | 0.415 | 0.0104 (0.0013) | 2.4E-16 | 0.0461 (0.0214) | 0.031 | 67.3 | 4.451 |
| Whole body fat-free mass | Alzheimer's disease, wide (FinnGen) | rs1412234 | 9:28410683 | C/T | 0.327 | 0.0109 (0.0013) | 2.6E-16 | 0.0346 (0.0219) | 0.115 | 67.0 | 3.184 |
| Whole body fat-free mass | Alzheimer's disease, wide (FinnGen) | rs55674305 | 8:89435868 | A/G | 0.306 | -0.0110 (0.0014) | 3.1E-16 | -0.0025 (0.0231) | 0.913 | 66.8 | 0.226 |
| Whole body fat-free mass | Alzheimer's disease, wide (FinnGen) | rs578366 | 6:81591034 | G/A | 0.420 | -0.0102 (0.0013) | 3.5E-16 | 0.0052 (0.0214) | 0.807 | 66.5 | -0.509 |
| Whole body fat-free mass | Alzheimer's disease, wide (FinnGen) | rs2197563 | 2:233687080 | A/G | 0.595 | 0.0103 (0.0013) | 3.8E-16 | -0.0091 (0.0239) | 0.703 | 66.3 | -0.886 |
| Whole body fat-free mass | Alzheimer's disease, wide (FinnGen) | rs12820906 | 12:123493123 | G/A | 0.247 | 0.0117 (0.0014) | 3.9E-16 | -0.0280 (0.0263) | 0.288 | 66.3 | -2.386 |
| Whole body fat-free mass | Alzheimer's disease, wide (FinnGen) | rs10899736 | 7:50546925 | A/G | 0.440 | -0.0102 (0.0013) | 4.4E-16 | -0.0419 (0.0218) | 0.054 | 66.1 | 4.120 |
| Whole body fat-free mass | Alzheimer's disease, wide (FinnGen) | rs1049193 | 12:53873844 | C/G | 0.171 | -0.0134 (0.0016) | 4.6E-16 | -0.0670 (0.0338) | 0.048 | 66.0 | 5.001 |
| Whole body fat-free mass | Alzheimer's disease, wide (FinnGen) | rs141729694 | 5:87999371 | T/C | 0.074 | 0.0193 (0.0024) | 4.7E-16 | 0.0232 (0.0538) | 0.667 | 65.9 | 1.202 |
| Whole body fat-free mass | Alzheimer's disease, wide (FinnGen) | rs11855017 | 15:42096146 | A/C | 0.182 | 0.0131 (0.0016) | 4.8E-16 | 0.0154 (0.0317) | 0.628 | 65.9 | 1.175 |
| Whole body fat-free mass | Alzheimer's disease, wide (FinnGen) | rs667515 | 11:69449076 | C/G | 0.386 | -0.0104 (0.0013) | 5.9E-16 | -0.0127 (0.0224) | 0.571 | 65.5 | 1.226 |
| Whole body fat-free mass | Alzheimer's disease, wide (FinnGen) | rs357486 | 3:153885503 | C/T | 0.543 | 0.0100 (0.0012) | 6.7E-16 | 0.0493 (0.0214) | 0.021 | 65.2 | 4.911 |
| Whole body fat-free mass | Alzheimer's disease, wide (FinnGen) | rs11245450 | 10:126658075 | A/G | 0.422 | -0.0103 (0.0013) | 7.0E-16 | -0.0043 (0.0214) | 0.839 | 65.2 | 0.419 |
| Whole body fat-free mass | Alzheimer's disease, wide (FinnGen) | rs6984820 | 8:144993324 | T/C | 0.422 | -0.0101 (0.0013) | 8.5E-16 | 0.0479 (0.0217) | 0.027 | 64.7 | -4.723 |
| Whole body fat-free mass | Alzheimer's disease, wide (FinnGen) | rs9513510 | 13:99571922 | C/G | 0.699 | -0.0109 (0.0014) | 1.0E-15 | 0.0048 (0.0227) | 0.832 | 64.4 | -0.441 |
| Whole body fat-free mass | Alzheimer's disease, wide (FinnGen) | rs117543413 | 10:79543740 | T/C | 0.018 | -0.0382 (0.0048) | 1.2E-15 | 0.0439 (0.0881) | 0.618 | 64.0 | -1.148 |
| Whole body fat-free mass | Alzheimer's disease, wide (FinnGen) | rs10172196 | 2:36780549 | A/G | 0.306 | 0.0108 (0.0013) | 1.3E-15 | -0.0756 (0.0225) | 7.9E-04 | 63.9 | -7.025 |
| Whole body fat-free mass | Alzheimer's disease, wide (FinnGen) | rs603321 | 6:140256669 | G/A | 0.245 | -0.0115 (0.0014) | 1.6E-15 | 0.0029 (0.0277) | 0.918 | 63.5 | -0.251 |
| Whole body fat-free mass | Alzheimer's disease, wide (FinnGen) | rs12764498 | 10:5042294 | C/T | 0.118 | -0.0155 (0.0019) | 1.6E-15 | -0.0090 (0.0317) | 0.776 | 63.6 | 0.582 |
| Whole body fat-free mass | Alzheimer's disease, wide (FinnGen) | rs7229520 | 18:46516468 | A/G | 0.662 | -0.0105 (0.0013) | 2.0E-15 | -0.0047 (0.0222) | 0.832 | 63.0 | 0.449 |
| Whole body fat-free mass | Alzheimer's disease, wide (FinnGen) | rs1780673 | 20:35758359 | A/G | 0.811 | 0.0126 (0.0016) | 2.2E-15 | 0.0422 (0.0231) | 0.068 | 62.9 | 3.346 |
| Whole body fat-free mass | Alzheimer's disease, wide (FinnGen) | rs10434434 | 4:54271611 | C/A | 0.148 | -0.0139 (0.0018) | 2.4E-15 | 0.0042 (0.0333) | 0.901 | 62.7 | -0.303 |
| Whole body fat-free mass | Alzheimer's disease, wide (FinnGen) | rs1971955 | 12:122420437 | G/A | 0.854 | -0.0139 (0.0018) | 2.9E-15 | -0.0410 (0.0273) | 0.134 | 62.3 | 2.950 |
| Whole body fat-free mass | Alzheimer's disease, wide (FinnGen) | rs5752989 | 22:30365780 | A/G | 0.571 | -0.0099 (0.0013) | 2.9E-15 | -0.0076 (0.0212) | 0.722 | 62.3 | 0.764 |
| Whole body fat-free mass | Alzheimer's disease, wide (FinnGen) | rs34693680 | 3:98665549 | T/C | 0.132 | 0.0144 (0.0018) | 3.3E-15 | -0.0268 (0.0324) | 0.407 | 62.1 | -1.859 |
| Whole body fat-free mass | Alzheimer's disease, wide (FinnGen) | rs12140153 | 1:62579891 | T/G | 0.094 | -0.0171 (0.0022) | 3.8E-15 | -0.0344 (0.0395) | 0.384 | 61.8 | 2.017 |
| Whole body fat-free mass | Alzheimer's disease, wide (FinnGen) | rs35251247 | 11:43878459 | A/G | 0.292 | 0.0108 (0.0014) | 3.8E-15 | 0.0201 (0.0233) | 0.388 | 61.8 | 1.860 |
| Whole body fat-free mass | Alzheimer's disease, wide (FinnGen) | rs6081869 | 20:20060745 | G/T | 0.386 | -0.0102 (0.0013) | 4.4E-15 | 0.0236 (0.0214) | 0.271 | 61.5 | -2.323 |
| Whole body fat-free mass | Alzheimer's disease, wide (FinnGen) | rs74494415 | 18:74972138 | T/C | 0.040 | -0.0252 (0.0032) | 4.6E-15 | 0.0774 (0.0403) | 0.055 | 61.4 | -3.076 |
| Whole body fat-free mass | Alzheimer's disease, wide (FinnGen) | rs11049684 | 12:28665359 | T/C | 0.299 | -0.0106 (0.0014) | 4.8E-15 | 0.0634 (0.0242) | 0.009 | 61.4 | -5.975 |
| Whole body fat-free mass | Alzheimer's disease, wide (FinnGen) | rs10979612 | 9:111687379 | C/T | 0.073 | 0.0187 (0.0024) | 4.9E-15 | -0.0291 (0.0333) | 0.382 | 61.3 | -1.557 |
| Whole body fat-free mass | Alzheimer's disease, wide (FinnGen) | rs147110934 | 19:55993436 | T/G | 0.024 | -0.0316 (0.0040) | 5.6E-15 | -0.0181 (0.1386) | 0.896 | 61.0 | 0.574 |
| Whole body fat-free mass | Alzheimer's disease, wide (FinnGen) | rs798759 | 4:1730299 | G/A | 0.464 | -0.0097 (0.0012) | 6.7E-15 | -0.0065 (0.0222) | 0.770 | 60.7 | 0.673 |
| Whole body fat-free mass | Alzheimer's disease, wide (FinnGen) | rs10780906 | 9:90841143 | A/G | 0.588 | -0.0099 (0.0013) | 8.4E-15 | -0.0176 (0.0213) | 0.409 | 60.2 | 1.782 |
| Whole body fat-free mass | Alzheimer's disease, wide (FinnGen) | rs2287163 | 12:107349294 | T/C | 0.374 | -0.0099 (0.0013) | 8.4E-15 | -0.0056 (0.0223) | 0.802 | 60.2 | 0.564 |
| Whole body fat-free mass | Alzheimer's disease, wide (FinnGen) | rs7301341 | 12:94083105 | C/T | 0.327 | -0.0103 (0.0013) | 9.3E-15 | -0.0242 (0.0223) | 0.279 | 60.0 | 2.359 |
| Whole body fat-free mass | Alzheimer's disease, wide (FinnGen) | rs2104449 | 1:97082532 | T/G | 0.283 | 0.0107 (0.0014) | 9.4E-15 | 0.0120 (0.0276) | 0.665 | 60.0 | 1.125 |
| Whole body fat-free mass | Alzheimer's disease, wide (FinnGen) | rs4974223 | 3:56602088 | T/C | 0.904 | 0.0163 (0.0021) | 1.0E-14 | 0.0136 (0.0332) | 0.682 | 59.9 | 0.837 |
| Whole body fat-free mass | Alzheimer's disease, wide (FinnGen) | rs6142059 | 20:32544327 | C/T | 0.493 | 0.0096 (0.0012) | 1.1E-14 | -0.0169 (0.0212) | 0.427 | 59.8 | -1.753 |
| Whole body fat-free mass | Alzheimer's disease, wide (FinnGen) | rs2615075 | 1:225934295 | G/A | 0.376 | 0.0098 (0.0013) | 2.0E-14 | -0.0016 (0.0219) | 0.942 | 58.5 | -0.163 |
| Whole body fat-free mass | Alzheimer's disease, wide (FinnGen) | rs9379084 | 6:7231843 | A/G | 0.116 | -0.0152 (0.0020) | 2.7E-14 | -0.0078 (0.0341) | 0.820 | 57.9 | 0.514 |
| Whole body fat-free mass | Alzheimer's disease, wide (FinnGen) | rs11785562 | 8:23391493 | A/G | 0.201 | 0.0119 (0.0016) | 2.7E-14 | -0.0262 (0.0244) | 0.281 | 58.0 | -2.196 |
| Whole body fat-free mass | Alzheimer's disease, wide (FinnGen) | rs5742915 | 15:74336633 | C/T | 0.460 | 0.0095 (0.0012) | 3.2E-14 | -0.0296 (0.0216) | 0.171 | 57.6 | -3.123 |
| Whole body fat-free mass | Alzheimer's disease, wide (FinnGen) | rs1374370 | 2:85818273 | A/G | 0.305 | 0.0102 (0.0013) | 3.5E-14 | -0.0508 (0.0251) | 0.043 | 57.5 | -4.987 |
| Whole body fat-free mass | Alzheimer's disease, wide (FinnGen) | rs11611651 | 12:133380790 | A/G | 0.088 | 0.0166 (0.0022) | 4.3E-14 | 0.0737 (0.0504) | 0.144 | 57.0 | 4.452 |
| Whole body fat-free mass | Alzheimer's disease, wide (FinnGen) | rs981002 | 4:12881731 | A/T | 0.267 | -0.0106 (0.0014) | 5.3E-14 | -0.0133 (0.0248) | 0.591 | 56.6 | 1.261 |
| Whole body fat-free mass | Alzheimer's disease, wide (FinnGen) | rs2568958 | 1:72765116 | A/G | 0.604 | 0.0095 (0.0013) | 5.5E-14 | -0.0158 (0.0222) | 0.477 | 56.5 | -1.664 |
| Whole body fat-free mass | Alzheimer's disease, wide (FinnGen) | rs12375196 | 7:103416541 | A/C | 0.424 | 0.0095 (0.0013) | 6.5E-14 | 0.0139 (0.0213) | 0.514 | 56.2 | 1.468 |
| Whole body fat-free mass | Alzheimer's disease, wide (FinnGen) | rs6026578 | 20:57463472 | G/C | 0.625 | -0.0097 (0.0013) | 6.8E-14 | -0.0184 (0.0222) | 0.408 | 56.1 | 1.901 |
| Whole body fat-free mass | Alzheimer's disease, wide (FinnGen) | rs58857770 | 19:7208110 | G/C | 0.408 | -0.0095 (0.0013) | 7.6E-14 | 0.0389 (0.0212) | 0.066 | 55.9 | -4.084 |
| Whole body fat-free mass | Alzheimer's disease, wide (FinnGen) | rs9960619 | 18:33040095 | T/C | 0.344 | 0.0098 (0.0013) | 8.7E-14 | 0.0006 (0.0226) | 0.978 | 55.6 | 0.061 |
| Whole body fat-free mass | Alzheimer's disease, wide (FinnGen) | rs13272451 | 8:8886183 | T/G | 0.439 | 0.0093 (0.0013) | 8.8E-14 | 0.0127 (0.0360) | 0.724 | 55.6 | 1.359 |
| Whole body fat-free mass | Alzheimer's disease, wide (FinnGen) | rs11217863 | 11:120293138 | A/G | 0.116 | -0.0145 (0.0019) | 9.0E-14 | 0.0332 (0.0320) | 0.300 | 55.6 | -2.294 |
| Whole body fat-free mass | Alzheimer's disease, wide (FinnGen) | rs62621400 | 15:101718239 | G/C | 0.058 | -0.0199 (0.0027) | 9.8E-14 | -0.0208 (0.0446) | 0.642 | 55.4 | 1.045 |
| Whole body fat-free mass | Alzheimer's disease, wide (FinnGen) | rs3925 | 8:38281658 | A/G | 0.245 | -0.0107 (0.0014) | 1.2E-13 | 0.0083 (0.0275) | 0.764 | 55.0 | -0.774 |
| Whole body fat-free mass | Alzheimer's disease, wide (FinnGen) | rs12070699 | 1:190230408 | T/C | 0.563 | -0.0092 (0.0012) | 1.5E-13 | 0.0042 (0.0212) | 0.844 | 54.5 | -0.456 |
| Whole body fat-free mass | Alzheimer's disease, wide (FinnGen) | rs35756741 | 12:12868701 | T/C | 0.092 | -0.0159 (0.0022) | 1.6E-13 | 0.0106 (0.0349) | 0.762 | 54.4 | -0.667 |
| Whole body fat-free mass | Alzheimer's disease, wide (FinnGen) | rs35492502 | 1:217806224 | A/G | 0.296 | 0.0100 (0.0014) | 2.1E-13 | -0.0177 (0.0226) | 0.433 | 53.9 | -1.777 |
| Whole body fat-free mass | Alzheimer's disease, wide (FinnGen) | rs284662 | 19:41932275 | C/T | 0.620 | -0.0094 (0.0013) | 2.2E-13 | -0.0020 (0.0222) | 0.929 | 53.8 | 0.213 |
| Whole body fat-free mass | Alzheimer's disease, wide (FinnGen) | rs36695 | 5:127549187 | A/G | 0.504 | -0.0091 (0.0012) | 2.3E-13 | -0.0169 (0.0216) | 0.434 | 53.7 | 1.859 |
| Whole body fat-free mass | Alzheimer's disease, wide (FinnGen) | rs4926542 | 1:50263773 | T/C | 0.682 | -0.0098 (0.0013) | 2.4E-13 | 0.0032 (0.0220) | 0.885 | 53.7 | -0.327 |
| Whole body fat-free mass | Alzheimer's disease, wide (FinnGen) | rs1805165 | 2:88874891 | A/C | 0.721 | -0.0101 (0.0014) | 2.5E-13 | 0.0114 (0.0225) | 0.613 | 53.6 | -1.131 |
| Whole body fat-free mass | Alzheimer's disease, wide (FinnGen) | rs12209223 | 6:76164589 | A/C | 0.101 | 0.0151 (0.0021) | 2.6E-13 | -0.0131 (0.0310) | 0.674 | 53.5 | -0.869 |
| Whole body fat-free mass | Alzheimer's disease, wide (FinnGen) | rs9925273 | 16:375900 | G/A | 0.181 | -0.0118 (0.0016) | 2.7E-13 | 0.0150 (0.0289) | 0.604 | 53.4 | -1.270 |
| Whole body fat-free mass | Alzheimer's disease, wide (FinnGen) | rs55758152 | 5:171317318 | A/G | 0.326 | 0.0098 (0.0013) | 2.8E-13 | -0.0030 (0.0225) | 0.893 | 53.3 | -0.307 |
| Whole body fat-free mass | Alzheimer's disease, wide (FinnGen) | rs2578557 | 5:5473063 | T/C | 0.642 | -0.0095 (0.0013) | 2.9E-13 | -0.0171 (0.0215) | 0.425 | 53.3 | 1.809 |
| Whole body fat-free mass | Alzheimer's disease, wide (FinnGen) | rs2970592 | 1:212220847 | A/G | 0.588 | 0.0092 (0.0013) | 3.0E-13 | -0.0202 (0.0220) | 0.358 | 53.2 | -2.207 |
| Whole body fat-free mass | Alzheimer's disease, wide (FinnGen) | rs74048171 | 11:2093603 | A/C | 0.258 | -0.0104 (0.0014) | 3.2E-13 | 0.0155 (0.0255) | 0.544 | 53.1 | -1.497 |
| Whole body fat-free mass | Alzheimer's disease, wide (FinnGen) | rs57126421 | 18:2656989 | G/A | 0.238 | -0.0107 (0.0015) | 3.8E-13 | 0.0494 (0.0253) | 0.051 | 52.8 | -4.622 |
| Whole body fat-free mass | Alzheimer's disease, wide (FinnGen) | rs1521624 | 15:79412399 | A/C | 0.471 | -0.0091 (0.0013) | 4.0E-13 | -0.0140 (0.0212) | 0.510 | 52.7 | 1.540 |
| Whole body fat-free mass | Alzheimer's disease, wide (FinnGen) | rs11065015 | 12:120520863 | T/C | 0.027 | -0.0282 (0.0039) | 4.1E-13 | -0.0231 (0.0479) | 0.629 | 52.6 | 0.820 |
| Whole body fat-free mass | Alzheimer's disease, wide (FinnGen) | rs11545482 | 2:70315987 | T/C | 0.020 | -0.0316 (0.0044) | 4.4E-13 | -0.0769 (0.1493) | 0.607 | 52.5 | 2.432 |
| Whole body fat-free mass | Alzheimer's disease, wide (FinnGen) | rs73601548 | 10:18549889 | T/C | 0.115 | 0.0142 (0.0020) | 4.8E-13 | -0.0697 (0.0430) | 0.105 | 52.3 | -4.923 |
| Whole body fat-free mass | Alzheimer's disease, wide (FinnGen) | rs55800172 | 1:32414767 | A/G | 0.064 | 0.0182 (0.0025) | 5.7E-13 | 0.0832 (0.0558) | 0.136 | 52.0 | 4.566 |
| Whole body fat-free mass | Alzheimer's disease, wide (FinnGen) | rs11071182 | 15:55644676 | G/A | 0.870 | 0.0134 (0.0019) | 6.3E-13 | -0.0049 (0.0339) | 0.886 | 51.7 | -0.366 |
| Whole body fat-free mass | Alzheimer's disease, wide (FinnGen) | rs11160601 | 14:101186641 | T/C | 0.092 | 0.0156 (0.0022) | 6.5E-13 | 0.0008 (0.0294) | 0.978 | 51.7 | 0.051 |
| Whole body fat-free mass | Alzheimer's disease, wide (FinnGen) | rs3783256 | 13:30107388 | C/T | 0.648 | -0.0094 (0.0013) | 6.7E-13 | -0.0051 (0.0214) | 0.810 | 51.6 | 0.543 |
| Whole body fat-free mass | Alzheimer's disease, wide (FinnGen) | rs17828687 | 8:73454767 | A/C | 0.573 | -0.0091 (0.0013) | 6.8E-13 | 0.0024 (0.0216) | 0.910 | 51.6 | -0.265 |
| Whole body fat-free mass | Alzheimer's disease, wide (FinnGen) | rs71393968 | 16:67847341 | A/G | 0.046 | 0.0214 (0.0030) | 6.9E-13 | -0.0034 (0.0525) | 0.948 | 51.6 | -0.159 |
| Whole body fat-free mass | Alzheimer's disease, wide (FinnGen) | rs11709402 | 3:131551027 | G/A | 0.279 | 0.0100 (0.0014) | 7.1E-13 | 0.0230 (0.0240) | 0.339 | 51.5 | 2.310 |
| Whole body fat-free mass | Alzheimer's disease, wide (FinnGen) | rs4795318 | 17:36949053 | T/C | 0.520 | 0.0089 (0.0012) | 7.1E-13 | 0.0019 (0.0213) | 0.928 | 51.5 | 0.213 |
| Whole body fat-free mass | Alzheimer's disease, wide (FinnGen) | rs73013411 | 6:164126233 | A/C | 0.131 | 0.0131 (0.0018) | 8.9E-13 | 0.0239 (0.0391) | 0.540 | 51.1 | 1.820 |
| Whole body fat-free mass | Alzheimer's disease, wide (FinnGen) | rs16916881 | 8:95566270 | A/C | 0.235 | -0.0104 (0.0015) | 9.2E-13 | -0.0029 (0.0220) | 0.895 | 51.0 | 0.278 |
| Whole body fat-free mass | Alzheimer's disease, wide (FinnGen) | rs10870597 | 13:114999636 | G/A | 0.235 | -0.0105 (0.0015) | 1.0E-12 | 0.0015 (0.0249) | 0.952 | 50.8 | -0.143 |
| Whole body fat-free mass | Alzheimer's disease, wide (FinnGen) | rs6719296 | 2:103101108 | A/G | 0.495 | 0.0089 (0.0012) | 1.1E-12 | -0.0245 (0.0219) | 0.264 | 50.6 | -2.768 |
| Whole body fat-free mass | Alzheimer's disease, wide (FinnGen) | rs10945541 | 6:158740218 | G/A | 0.665 | -0.0093 (0.0013) | 1.1E-12 | 0.0166 (0.0218) | 0.448 | 50.7 | -1.776 |
| Whole body fat-free mass | Alzheimer's disease, wide (FinnGen) | rs60804050 | 1:118870373 | A/G | 0.256 | -0.0100 (0.0014) | 1.5E-12 | 0.0092 (0.0248) | 0.711 | 50.1 | -0.918 |
| Whole body fat-free mass | Alzheimer's disease, wide (FinnGen) | rs9540493 | 13:66205704 | G/A | 0.545 | -0.0089 (0.0013) | 1.5E-12 | 0.0065 (0.0214) | 0.759 | 50.1 | -0.731 |
| Whole body fat-free mass | Alzheimer's disease, wide (FinnGen) | rs7245985 | 19:30710410 | G/T | 0.208 | -0.0109 (0.0015) | 1.5E-12 | -0.0057 (0.0270) | 0.832 | 50.1 | 0.524 |
| Whole body fat-free mass | Alzheimer's disease, wide (FinnGen) | rs73619441 | 20:61564901 | G/T | 0.144 | -0.0126 (0.0018) | 1.5E-12 | -0.0016 (0.0336) | 0.962 | 50.1 | 0.127 |
| Whole body fat-free mass | Alzheimer's disease, wide (FinnGen) | rs2789366 | 1:235508270 | A/G | 0.346 | -0.0092 (0.0013) | 1.6E-12 | -0.0357 (0.0220) | 0.105 | 50.0 | 3.885 |
| Whole body fat-free mass | Alzheimer's disease, wide (FinnGen) | rs16844418 | 4:3471754 | C/T | 0.143 | -0.0125 (0.0018) | 1.9E-12 | -0.0190 (0.0323) | 0.555 | 49.6 | 1.522 |
| Whole body fat-free mass | Alzheimer's disease, wide (FinnGen) | rs10222924 | 4:49016761 | G/A | 0.699 | -0.0095 (0.0014) | 2.0E-12 | 0.0000 (0.0244) | 0.999 | 49.5 | 0.000 |
| Whole body fat-free mass | Alzheimer's disease, wide (FinnGen) | rs7192870 | 16:14397436 | C/T | 0.469 | -0.0089 (0.0013) | 2.0E-12 | -0.0076 (0.0218) | 0.726 | 49.4 | 0.858 |
| Whole body fat-free mass | Alzheimer's disease, wide (FinnGen) | rs11052457 | 12:33268956 | T/A | 0.038 | 0.0228 (0.0032) | 2.1E-12 | 0.0178 (0.0562) | 0.751 | 49.4 | 0.781 |
| Whole body fat-free mass | Alzheimer's disease, wide (FinnGen) | rs892511 | 1:110078255 | G/C | 0.028 | 0.0263 (0.0038) | 3.7E-12 | 0.0429 (0.0490) | 0.381 | 48.3 | 1.629 |
| Whole body fat-free mass | Alzheimer's disease, wide (FinnGen) | rs17400325 | 2:178565913 | C/T | 0.042 | 0.0215 (0.0031) | 3.8E-12 | -0.0909 (0.0746) | 0.223 | 48.2 | -4.225 |
| Whole body fat-free mass | Alzheimer's disease, wide (FinnGen) | rs9394166 | 6:33751122 | C/G | 0.380 | 0.0089 (0.0013) | 3.8E-12 | 0.0031 (0.0216) | 0.887 | 48.2 | 0.349 |
| Whole body fat-free mass | Alzheimer's disease, wide (FinnGen) | rs338361 | 15:68171116 | C/T | 0.706 | 0.0095 (0.0014) | 3.9E-12 | 0.0570 (0.0252) | 0.024 | 48.1 | 6.004 |
| Whole body fat-free mass | Alzheimer's disease, wide (FinnGen) | rs115461836 | 6:19078931 | C/T | 0.046 | 0.0208 (0.0030) | 4.2E-12 | -0.0062 (0.0642) | 0.923 | 48.0 | -0.297 |
| Whole body fat-free mass | Alzheimer's disease, wide (FinnGen) | rs1296328 | 4:137083193 | C/A | 0.559 | -0.0087 (0.0013) | 4.5E-12 | -0.0398 (0.0213) | 0.061 | 47.9 | 4.585 |
| Whole body fat-free mass | Alzheimer's disease, wide (FinnGen) | rs4132132 | 4:31001301 | C/T | 0.444 | 0.0087 (0.0013) | 4.7E-12 | -0.0275 (0.0217) | 0.205 | 47.8 | -3.157 |
| Whole body fat-free mass | Alzheimer's disease, wide (FinnGen) | rs9596810 | 13:54056553 | T/C | 0.435 | -0.0089 (0.0013) | 4.7E-12 | 0.0082 (0.0217) | 0.707 | 47.8 | -0.917 |
| Whole body fat-free mass | Alzheimer's disease, wide (FinnGen) | rs1927635 | 9:16461905 | C/T | 0.354 | 0.0091 (0.0013) | 5.0E-12 | 0.0409 (0.0213) | 0.055 | 47.7 | 4.508 |
| Whole body fat-free mass | Alzheimer's disease, wide (FinnGen) | rs1218826 | 13:27995760 | G/A | 0.666 | 0.0091 (0.0013) | 5.0E-12 | 0.0154 (0.0226) | 0.497 | 47.7 | 1.685 |
| Whole body fat-free mass | Alzheimer's disease, wide (FinnGen) | rs17363646 | 1:86823503 | G/A | 0.136 | 0.0124 (0.0018) | 5.5E-12 | -0.0027 (0.0244) | 0.912 | 47.5 | -0.217 |
| Whole body fat-free mass | Alzheimer's disease, wide (FinnGen) | rs17443541 | 2:200402624 | C/T | 0.189 | -0.0110 (0.0016) | 5.8E-12 | -0.0159 (0.0283) | 0.575 | 47.4 | 1.448 |
| Whole body fat-free mass | Alzheimer's disease, wide (FinnGen) | rs12889702 | 14:42894143 | C/A | 0.313 | 0.0092 (0.0013) | 6.4E-12 | 0.0119 (0.0233) | 0.609 | 47.2 | 1.289 |
| Whole body fat-free mass | Alzheimer's disease, wide (FinnGen) | rs254963 | 5:171224403 | G/A | 0.447 | -0.0086 (0.0013) | 6.6E-12 | 0.0226 (0.0213) | 0.288 | 47.1 | -2.620 |
| Whole body fat-free mass | Alzheimer's disease, wide (FinnGen) | rs1813212 | 11:89301382 | G/A | 0.446 | -0.0086 (0.0013) | 6.7E-12 | 0.0223 (0.0214) | 0.297 | 47.1 | -2.595 |
| Whole body fat-free mass | Alzheimer's disease, wide (FinnGen) | rs7188009 | 16:81660642 | A/G | 0.404 | 0.0087 (0.0013) | 7.0E-12 | 0.0219 (0.0241) | 0.364 | 47.0 | 2.519 |
| Whole body fat-free mass | Alzheimer's disease, wide (FinnGen) | rs774214 | 12:56918566 | C/T | 0.663 | -0.0090 (0.0013) | 7.2E-12 | -0.0084 (0.0221) | 0.706 | 47.0 | 0.934 |
| Whole body fat-free mass | Alzheimer's disease, wide (FinnGen) | rs6972291 | 7:55802063 | C/T | 0.176 | -0.0112 (0.0016) | 7.7E-12 | 0.0208 (0.0243) | 0.392 | 46.8 | -1.861 |
| Whole body fat-free mass | Alzheimer's disease, wide (FinnGen) | rs7731023 | 5:36181627 | G/A | 0.574 | 0.0086 (0.0013) | 7.8E-12 | -0.0142 (0.0222) | 0.522 | 46.8 | -1.657 |
| Whole body fat-free mass | Alzheimer's disease, wide (FinnGen) | rs12981554 | 19:3416668 | G/A | 0.542 | -0.0086 (0.0013) | 8.9E-12 | -0.0166 (0.0215) | 0.439 | 46.6 | 1.933 |
| Whole body fat-free mass | Alzheimer's disease, wide (FinnGen) | rs4648626 | 1:2200390 | A/C | 0.457 | 0.0085 (0.0012) | 9.1E-12 | 0.0088 (0.0214) | 0.681 | 46.5 | 1.037 |
| Whole body fat-free mass | Alzheimer's disease, wide (FinnGen) | rs35874463 | 15:67457698 | G/A | 0.058 | 0.0182 (0.0027) | 9.7E-12 | 0.0248 (0.0552) | 0.653 | 46.4 | 1.366 |
| Whole body fat-free mass | Alzheimer's disease, wide (FinnGen) | rs10995366 | 10:52772113 | A/G | 0.251 | -0.0098 (0.0014) | 1.1E-11 | -0.0031 (0.0241) | 0.899 | 46.1 | 0.317 |
| Whole body fat-free mass | Alzheimer's disease, wide (FinnGen) | rs77344209 | 3:55515277 | T/C | 0.252 | 0.0098 (0.0014) | 1.2E-11 | -0.0220 (0.0272) | 0.417 | 46.0 | -2.245 |
| Whole body fat-free mass | Alzheimer's disease, wide (FinnGen) | rs11593630 | 10:120491506 | T/C | 0.353 | -0.0088 (0.0013) | 1.2E-11 | 0.0039 (0.0220) | 0.859 | 46.0 | -0.443 |
| Whole body fat-free mass | Alzheimer's disease, wide (FinnGen) | rs1418433 | 6:44752568 | A/G | 0.588 | -0.0085 (0.0013) | 1.3E-11 | 0.0124 (0.0215) | 0.565 | 45.8 | -1.457 |
| Whole body fat-free mass | Alzheimer's disease, wide (FinnGen) | rs7912286 | 10:114693230 | G/A | 0.602 | -0.0087 (0.0013) | 1.3E-11 | 0.0455 (0.0217) | 0.036 | 45.8 | -5.248 |
| Whole body fat-free mass | Alzheimer's disease, wide (FinnGen) | rs7321045 | 13:97034410 | A/G | 0.449 | 0.0085 (0.0013) | 1.3E-11 | -0.0444 (0.0213) | 0.037 | 45.8 | -5.206 |
| Whole body fat-free mass | Alzheimer's disease, wide (FinnGen) | rs73004967 | 19:19717056 | G/A | 0.069 | -0.0167 (0.0025) | 1.3E-11 | 0.0486 (0.0447) | 0.277 | 45.8 | -2.904 |
| Whole body fat-free mass | Alzheimer's disease, wide (FinnGen) | rs71423263 | 2:144024781 | G/T | 0.141 | 0.0120 (0.0018) | 1.5E-11 | 0.0275 (0.0340) | 0.418 | 45.5 | 2.293 |
| Whole body fat-free mass | Alzheimer's disease, wide (FinnGen) | rs73213484 | 4:28489339 | T/A | 0.141 | -0.0120 (0.0018) | 1.5E-11 | -0.0093 (0.0301) | 0.756 | 45.5 | 0.774 |
| Whole body fat-free mass | Alzheimer's disease, wide (FinnGen) | rs6085658 | 20:6685377 | T/C | 0.403 | -0.0086 (0.0013) | 1.6E-11 | -0.0071 (0.0214) | 0.738 | 45.4 | 0.826 |
| Whole body fat-free mass | Alzheimer's disease, wide (FinnGen) | rs2122823 | 7:25939161 | T/C | 0.211 | 0.0103 (0.0015) | 2.0E-11 | 0.0124 (0.0247) | 0.615 | 45.0 | 1.201 |
| Whole body fat-free mass | Alzheimer's disease, wide (FinnGen) | rs12427047 | 12:90213070 | T/C | 0.243 | -0.0097 (0.0014) | 2.1E-11 | 0.0066 (0.0223) | 0.766 | 44.9 | -0.681 |
| Whole body fat-free mass | Alzheimer's disease, wide (FinnGen) | rs112957890 | 14:36220876 | G/A | 0.265 | 0.0095 (0.0014) | 2.1E-11 | -0.0258 (0.0254) | 0.309 | 44.8 | -2.706 |
| Whole body fat-free mass | Alzheimer's disease, wide (FinnGen) | rs12729817 | 1:218971896 | G/A | 0.474 | -0.0083 (0.0012) | 2.2E-11 | -0.0191 (0.0211) | 0.367 | 44.8 | 2.308 |
| Whole body fat-free mass | Alzheimer's disease, wide (FinnGen) | rs4268495 | 11:28645341 | C/G | 0.374 | -0.0086 (0.0013) | 2.2E-11 | -0.0137 (0.0220) | 0.531 | 44.8 | 1.597 |
| Whole body fat-free mass | Alzheimer's disease, wide (FinnGen) | rs114949263 | 7:150498245 | C/T | 0.112 | -0.0132 (0.0020) | 2.5E-11 | -0.0175 (0.0397) | 0.660 | 44.5 | 1.327 |
| Whole body fat-free mass | Alzheimer's disease, wide (FinnGen) | rs505575 | 5:111256812 | C/T | 0.673 | -0.0088 (0.0013) | 2.6E-11 | 0.0179 (0.0218) | 0.413 | 44.4 | -2.028 |
| Whole body fat-free mass | Alzheimer's disease, wide (FinnGen) | rs114177791 | 17:78555512 | T/G | 0.214 | 0.0101 (0.0015) | 2.7E-11 | -0.0388 (0.0268) | 0.148 | 44.4 | -3.837 |
| Whole body fat-free mass | Alzheimer's disease, wide (FinnGen) | rs61878760 | 11:12807189 | A/G | 0.083 | 0.0151 (0.0023) | 2.8E-11 | 0.0816 (0.0594) | 0.170 | 44.3 | 5.416 |
| Whole body fat-free mass | Alzheimer's disease, wide (FinnGen) | rs3845344 | 1:75001480 | T/C | 0.391 | 0.0084 (0.0013) | 3.1E-11 | 0.0283 (0.0214) | 0.185 | 44.1 | 3.363 |
| Whole body fat-free mass | Alzheimer's disease, wide (FinnGen) | rs9327336 | 5:123990270 | C/T | 0.343 | 0.0087 (0.0013) | 3.2E-11 | -0.0174 (0.0214) | 0.417 | 44.1 | -2.008 |
| Whole body fat-free mass | Alzheimer's disease, wide (FinnGen) | rs2663125 | 15:99563891 | C/T | 0.691 | -0.0089 (0.0013) | 3.2E-11 | -0.0107 (0.0251) | 0.671 | 44.0 | 1.198 |
| Whole body fat-free mass | Alzheimer's disease, wide (FinnGen) | rs67560975 | 17:47142930 | C/T | 0.116 | 0.0129 (0.0019) | 3.3E-11 | -0.0468 (0.0317) | 0.141 | 44.0 | -3.626 |
| Whole body fat-free mass | Alzheimer's disease, wide (FinnGen) | rs1056747 | 9:35690102 | G/A | 0.412 | -0.0084 (0.0013) | 3.4E-11 | -0.0100 (0.0215) | 0.643 | 43.9 | 1.196 |
| Whole body fat-free mass | Alzheimer's disease, wide (FinnGen) | rs17197114 | 14:21894526 | C/T | 0.177 | 0.0109 (0.0016) | 3.4E-11 | -0.0853 (0.0297) | 0.004 | 43.9 | -7.843 |
| Whole body fat-free mass | Alzheimer's disease, wide (FinnGen) | rs12694042 | 2:207029825 | T/C | 0.502 | -0.0082 (0.0012) | 3.8E-11 | 0.0037 (0.0217) | 0.864 | 43.7 | -0.450 |
| Whole body fat-free mass | Alzheimer's disease, wide (FinnGen) | rs4912905 | 5:142730376 | C/G | 0.249 | -0.0095 (0.0014) | 3.8E-11 | 0.0124 (0.0244) | 0.612 | 43.7 | -1.302 |
| Whole body fat-free mass | Alzheimer's disease, wide (FinnGen) | rs77848106 | 1:107971673 | A/C | 0.296 | -0.0090 (0.0014) | 3.9E-11 | 0.0185 (0.0214) | 0.387 | 43.7 | -2.050 |
| Whole body fat-free mass | Alzheimer's disease, wide (FinnGen) | rs4504126 | 3:33600582 | C/A | 0.027 | 0.0250 (0.0038) | 4.3E-11 | 0.0133 (0.0852) | 0.876 | 43.5 | 0.531 |
| Whole body fat-free mass | Alzheimer's disease, wide (FinnGen) | rs4439140 | 8:23113908 | A/G | 0.418 | -0.0085 (0.0013) | 4.7E-11 | 0.0036 (0.0217) | 0.867 | 43.3 | -0.424 |
| Whole body fat-free mass | Alzheimer's disease, wide (FinnGen) | rs10775406 | 17:46197755 | G/A | 0.760 | -0.0096 (0.0015) | 4.7E-11 | -0.0195 (0.0241) | 0.420 | 43.3 | 2.038 |
| Whole body fat-free mass | Alzheimer's disease, wide (FinnGen) | rs6665399 | 1:21553933 | C/T | 0.445 | 0.0082 (0.0012) | 4.9E-11 | 0.0194 (0.0213) | 0.363 | 43.2 | 2.377 |
| Whole body fat-free mass | Alzheimer's disease, wide (FinnGen) | rs3110496 | 17:27917771 | G/A | 0.686 | 0.0088 (0.0013) | 5.0E-11 | -0.0006 (0.0218) | 0.978 | 43.2 | -0.068 |
| Whole body fat-free mass | Alzheimer's disease, wide (FinnGen) | rs10950207 | 7:70105168 | T/C | 0.384 | 0.0084 (0.0013) | 5.8E-11 | 0.0195 (0.0222) | 0.381 | 42.9 | 2.330 |
| Whole body fat-free mass | Alzheimer's disease, wide (FinnGen) | rs9788443 | 14:23878279 | C/T | 0.048 | 0.0191 (0.0029) | 5.8E-11 | 0.1235 (0.0898) | 0.169 | 42.9 | 6.466 |
| Whole body fat-free mass | Alzheimer's disease, wide (FinnGen) | rs4525525 | 17:1866892 | T/G | 0.264 | -0.0094 (0.0014) | 5.9E-11 | -0.0076 (0.0245) | 0.756 | 42.9 | 0.812 |
| Whole body fat-free mass | Alzheimer's disease, wide (FinnGen) | rs1179905 | 6:90322922 | G/A | 0.195 | 0.0103 (0.0016) | 6.3E-11 | 0.0421 (0.0281) | 0.134 | 42.7 | 4.101 |
| Whole body fat-free mass | Alzheimer's disease, wide (FinnGen) | rs6416914 | 17:39262850 | G/A | 0.695 | -0.0088 (0.0014) | 6.5E-11 | 0.0153 (0.0245) | 0.533 | 42.7 | -1.733 |
| Whole body fat-free mass | Alzheimer's disease, wide (FinnGen) | rs8074074 | 17:17407816 | T/C | 0.755 | 0.0095 (0.0014) | 6.8E-11 | 0.0567 (0.0233) | 0.015 | 42.6 | 5.995 |
| Whole body fat-free mass | Alzheimer's disease, wide (FinnGen) | rs34017457 | 16:67166731 | A/G | 0.007 | 0.0488 (0.0075) | 6.9E-11 | -0.0597 (0.1407) | 0.671 | 42.5 | -1.224 |
| Whole body fat-free mass | Alzheimer's disease, wide (FinnGen) | rs10843397 | 12:29529523 | T/C | 0.242 | 0.0094 (0.0014) | 7.6E-11 | 0.0529 (0.0248) | 0.033 | 42.4 | 5.619 |
| Whole body fat-free mass | Alzheimer's disease, wide (FinnGen) | rs10511111 | 3:80599757 | C/T | 0.309 | 0.0087 (0.0013) | 7.9E-11 | 0.0092 (0.0229) | 0.689 | 42.3 | 1.055 |
| Whole body fat-free mass | Alzheimer's disease, wide (FinnGen) | rs113619763 | 22:46399175 | T/A | 0.062 | 0.0168 (0.0026) | 8.0E-11 | 0.0923 (0.0460) | 0.045 | 42.3 | 5.506 |
| Whole body fat-free mass | Alzheimer's disease, wide (FinnGen) | rs1268947 | 10:121132631 | C/G | 0.126 | 0.0122 (0.0019) | 8.9E-11 | 0.0042 (0.0334) | 0.899 | 42.1 | 0.345 |
| Whole body fat-free mass | Alzheimer's disease, wide (FinnGen) | rs60385590 | 3:53630199 | A/C | 0.258 | 0.0093 (0.0014) | 1.0E-10 | 0.0344 (0.0249) | 0.168 | 41.8 | 3.713 |
| Whole body fat-free mass | Alzheimer's disease, wide (FinnGen) | rs7755185 | 6:152339615 | G/A | 0.311 | 0.0087 (0.0013) | 1.0E-10 | 0.0042 (0.0219) | 0.849 | 41.8 | 0.485 |
| Whole body fat-free mass | Alzheimer's disease, wide (FinnGen) | rs56141370 | 9:127056213 | T/C | 0.115 | 0.0126 (0.0020) | 1.0E-10 | 0.0047 (0.0339) | 0.889 | 41.7 | 0.373 |
| Whole body fat-free mass | Alzheimer's disease, wide (FinnGen) | rs4635681 | 3:152310614 | G/A | 0.156 | 0.0110 (0.0017) | 1.1E-10 | -0.0197 (0.0267) | 0.460 | 41.6 | -1.791 |
| Whole body fat-free mass | Alzheimer's disease, wide (FinnGen) | rs1266876 | 6:51781580 | T/C | 0.350 | 0.0084 (0.0013) | 1.1E-10 | 0.0311 (0.0212) | 0.143 | 41.6 | 3.713 |
| Whole body fat-free mass | Alzheimer's disease, wide (FinnGen) | rs240113 | 6:101058886 | A/G | 0.531 | -0.0080 (0.0012) | 1.1E-10 | -0.0137 (0.0212) | 0.518 | 41.7 | 1.707 |
| Whole body fat-free mass | Alzheimer's disease, wide (FinnGen) | rs6900690 | 6:126223032 | A/G | 0.562 | 0.0080 (0.0012) | 1.1E-10 | 0.0412 (0.0215) | 0.056 | 41.6 | 5.130 |
| Whole body fat-free mass | Alzheimer's disease, wide (FinnGen) | rs12156265 | 8:144848756 | A/G | 0.590 | 0.0082 (0.0013) | 1.1E-10 | -0.0228 (0.0214) | 0.286 | 41.6 | -2.793 |
| Whole body fat-free mass | Alzheimer's disease, wide (FinnGen) | rs1008158 | 9:113828811 | G/A | 0.342 | 0.0085 (0.0013) | 1.1E-10 | 0.0037 (0.0219) | 0.866 | 41.6 | 0.436 |
| Whole body fat-free mass | Alzheimer's disease, wide (FinnGen) | rs1407031 | 20:6542634 | T/C | 0.397 | -0.0082 (0.0013) | 1.2E-10 | -0.0040 (0.0215) | 0.851 | 41.4 | 0.487 |
| Whole body fat-free mass | Alzheimer's disease, wide (FinnGen) | rs17115481 | 5:153358226 | A/G | 0.270 | -0.0090 (0.0014) | 1.3E-10 | -0.0031 (0.0231) | 0.894 | 41.4 | 0.344 |
| Whole body fat-free mass | Alzheimer's disease, wide (FinnGen) | rs62466118 | 7:92716556 | A/G | 0.028 | -0.0255 (0.0040) | 1.4E-10 | -0.0593 (0.1210) | 0.624 | 41.1 | 2.329 |
| Whole body fat-free mass | Alzheimer's disease, wide (FinnGen) | rs36100359 | 14:21578007 | A/G | 0.139 | -0.0115 (0.0018) | 1.4E-10 | 0.0100 (0.0300) | 0.739 | 41.2 | -0.868 |
| Whole body fat-free mass | Alzheimer's disease, wide (FinnGen) | rs10498672 | 6:7797840 | G/C | 0.176 | 0.0104 (0.0016) | 1.6E-10 | 0.0157 (0.0258) | 0.543 | 40.9 | 1.511 |
| Whole body fat-free mass | Alzheimer's disease, wide (FinnGen) | rs1657222 | 10:34837139 | A/G | 0.396 | 0.0081 (0.0013) | 1.7E-10 | 0.0303 (0.0212) | 0.153 | 40.8 | 3.725 |
| Whole body fat-free mass | Alzheimer's disease, wide (FinnGen) | rs67141907 | 13:42805360 | T/C | 0.149 | 0.0112 (0.0018) | 1.7E-10 | 0.0205 (0.0341) | 0.549 | 40.8 | 1.831 |
| Whole body fat-free mass | Alzheimer's disease, wide (FinnGen) | rs281385 | 19:49217261 | G/A | 0.879 | -0.0122 (0.0019) | 1.7E-10 | -0.0037 (0.0281) | 0.895 | 40.8 | 0.303 |
| Whole body fat-free mass | Alzheimer's disease, wide (FinnGen) | rs62439025 | 6:166592164 | C/G | 0.152 | 0.0110 (0.0017) | 1.8E-10 | 0.0427 (0.0339) | 0.207 | 40.6 | 3.872 |
| Whole body fat-free mass | Alzheimer's disease, wide (FinnGen) | rs6591 | 11:840363 | T/C | 0.544 | 0.0080 (0.0013) | 1.9E-10 | 0.0332 (0.0214) | 0.121 | 40.6 | 4.159 |
| Whole body fat-free mass | Alzheimer's disease, wide (FinnGen) | rs7843128 | 8:22473465 | C/T | 0.357 | -0.0083 (0.0013) | 2.0E-10 | -0.0149 (0.0226) | 0.511 | 40.5 | 1.803 |
| Whole body fat-free mass | Alzheimer's disease, wide (FinnGen) | rs62246311 | 3:9498143 | A/G | 0.102 | 0.0130 (0.0020) | 2.2E-10 | -0.0428 (0.0434) | 0.325 | 40.3 | -3.298 |
| Whole body fat-free mass | Alzheimer's disease, wide (FinnGen) | rs12146350 | 10:22843111 | G/C | 0.602 | -0.0081 (0.0013) | 2.3E-10 | -0.0040 (0.0220) | 0.854 | 40.2 | 0.496 |
| Whole body fat-free mass | Alzheimer's disease, wide (FinnGen) | rs4906203 | 14:102928991 | T/C | 0.234 | -0.0093 (0.0015) | 2.6E-10 | -0.0068 (0.0239) | 0.776 | 40.0 | 0.732 |
| Whole body fat-free mass | Alzheimer's disease, wide (FinnGen) | rs10188231 | 2:142297493 | G/C | 0.186 | -0.0100 (0.0016) | 2.7E-10 | -0.0096 (0.0259) | 0.713 | 39.9 | 0.956 |
| Whole body fat-free mass | Alzheimer's disease, wide (FinnGen) | rs10770705 | 12:20857467 | C/A | 0.663 | -0.0083 (0.0013) | 2.8E-10 | 0.0343 (0.0230) | 0.136 | 39.8 | -4.146 |
| Whole body fat-free mass | Alzheimer's disease, wide (FinnGen) | rs6772164 | 3:196078149 | A/C | 0.358 | 0.0081 (0.0013) | 3.1E-10 | -0.0165 (0.0232) | 0.478 | 39.6 | -2.026 |
| Whole body fat-free mass | Alzheimer's disease, wide (FinnGen) | rs76307059 | 6:169000888 | G/C | 0.046 | -0.0186 (0.0030) | 3.1E-10 | 0.0023 (0.0459) | 0.960 | 39.6 | -0.124 |
| Whole body fat-free mass | Alzheimer's disease, wide (FinnGen) | rs141866277 | 15:43950699 | T/A | 0.024 | -0.0257 (0.0041) | 3.1E-10 | -0.0836 (0.1549) | 0.590 | 39.6 | 3.258 |
| Whole body fat-free mass | Alzheimer's disease, wide (FinnGen) | rs3730071 | 12:49168798 | A/C | 0.030 | -0.0228 (0.0036) | 3.2E-10 | 0.2425 (0.1052) | 0.021 | 39.6 | -10.643 |
| Whole body fat-free mass | Alzheimer's disease, wide (FinnGen) | rs11142700 | 9:73757155 | C/T | 0.408 | -0.0080 (0.0013) | 3.3E-10 | -0.0059 (0.0225) | 0.792 | 39.5 | 0.741 |
| Whole body fat-free mass | Alzheimer's disease, wide (FinnGen) | rs10881583 | 9:137267439 | C/T | 0.241 | -0.0091 (0.0015) | 3.4E-10 | -0.0442 (0.0247) | 0.074 | 39.4 | 4.833 |
| Whole body fat-free mass | Alzheimer's disease, wide (FinnGen) | rs8007644 | 14:35218831 | A/G | 0.388 | 0.0081 (0.0013) | 3.4E-10 | 0.0165 (0.0213) | 0.439 | 39.4 | 2.042 |
| Whole body fat-free mass | Alzheimer's disease, wide (FinnGen) | rs10282707 | 7:17911038 | T/C | 0.397 | 0.0079 (0.0013) | 3.6E-10 | -0.0317 (0.0226) | 0.160 | 39.3 | -3.988 |
| Whole body fat-free mass | Alzheimer's disease, wide (FinnGen) | rs6575340 | 14:94023972 | A/G | 0.636 | 0.0081 (0.0013) | 3.6E-10 | 0.0002 (0.0218) | 0.994 | 39.3 | 0.025 |
| Whole body fat-free mass | Alzheimer's disease, wide (FinnGen) | rs11205354 | 1:150249101 | A/C | 0.444 | -0.0078 (0.0013) | 3.7E-10 | 0.0345 (0.0214) | 0.107 | 39.3 | -4.398 |
| Whole body fat-free mass | Alzheimer's disease, wide (FinnGen) | rs13085472 | 3:171129859 | C/T | 0.666 | -0.0083 (0.0013) | 4.1E-10 | -0.0270 (0.0223) | 0.226 | 39.1 | 3.272 |
| Whole body fat-free mass | Alzheimer's disease, wide (FinnGen) | rs12533548 | 7:148660579 | G/A | 0.651 | 0.0081 (0.0013) | 4.6E-10 | 0.0137 (0.0227) | 0.545 | 38.8 | 1.682 |
| Whole body fat-free mass | Alzheimer's disease, wide (FinnGen) | rs112544217 | 2:20222686 | T/C | 0.022 | -0.0268 (0.0043) | 4.8E-10 | -0.0113 (0.0891) | 0.899 | 38.8 | 0.421 |
| Whole body fat-free mass | Alzheimer's disease, wide (FinnGen) | rs284315 | 1:10734800 | G/A | 0.498 | -0.0077 (0.0012) | 4.9E-10 | 0.0077 (0.0219) | 0.724 | 38.7 | -1.002 |
| Whole body fat-free mass | Alzheimer's disease, wide (FinnGen) | rs117451679 | 12:17245591 | G/A | 0.106 | 0.0127 (0.0020) | 5.0E-10 | -0.0016 (0.0322) | 0.961 | 38.7 | -0.126 |
| Whole body fat-free mass | Alzheimer's disease, wide (FinnGen) | rs12047986 | 1:155043812 | G/A | 0.522 | -0.0077 (0.0012) | 5.1E-10 | -0.0263 (0.0220) | 0.233 | 38.6 | 3.416 |
| Whole body fat-free mass | Alzheimer's disease, wide (FinnGen) | rs11937249 | 4:110755321 | T/G | 0.636 | -0.0080 (0.0013) | 5.1E-10 | -0.0066 (0.0215) | 0.760 | 38.6 | 0.824 |
| Whole body fat-free mass | Alzheimer's disease, wide (FinnGen) | rs2422054 | 5:161382474 | A/T | 0.631 | 0.0080 (0.0013) | 5.1E-10 | -0.0364 (0.0213) | 0.088 | 38.6 | -4.555 |
| Whole body fat-free mass | Alzheimer's disease, wide (FinnGen) | rs4819021 | 21:46466927 | C/T | 0.477 | -0.0078 (0.0013) | 6.4E-10 | 0.0490 (0.0213) | 0.022 | 38.2 | -6.291 |
| Whole body fat-free mass | Alzheimer's disease, wide (FinnGen) | rs11684531 | 2:219835489 | G/A | 0.133 | -0.0113 (0.0018) | 6.6E-10 | -0.0428 (0.0421) | 0.309 | 38.1 | 3.802 |
| Whole body fat-free mass | Alzheimer's disease, wide (FinnGen) | rs11833839 | 12:132560957 | T/C | 0.057 | 0.0166 (0.0027) | 6.8E-10 | -0.0250 (0.0300) | 0.405 | 38.1 | -1.510 |
| Whole body fat-free mass | Alzheimer's disease, wide (FinnGen) | rs77093479 | 17:79429575 | G/C | 0.165 | -0.0104 (0.0017) | 6.9E-10 | -0.0008 (0.0282) | 0.978 | 38.0 | 0.077 |
| Whole body fat-free mass | Alzheimer's disease, wide (FinnGen) | rs34825238 | 12:103328352 | T/G | 0.255 | 0.0089 (0.0014) | 7.1E-10 | 0.0141 (0.0270) | 0.602 | 38.0 | 1.586 |
| Whole body fat-free mass | Alzheimer's disease, wide (FinnGen) | rs6946419 | 7:28768853 | C/T | 0.377 | -0.0079 (0.0013) | 7.2E-10 | 0.0343 (0.0218) | 0.115 | 38.0 | -4.334 |
| Whole body fat-free mass | Alzheimer's disease, wide (FinnGen) | rs752070 | 2:74824970 | G/A | 0.126 | 0.0114 (0.0019) | 8.2E-10 | -0.0144 (0.0307) | 0.638 | 37.7 | -1.258 |
| Whole body fat-free mass | Alzheimer's disease, wide (FinnGen) | rs2274116 | 9:139094805 | T/C | 0.341 | -0.0081 (0.0013) | 8.3E-10 | 0.0257 (0.0226) | 0.255 | 37.7 | -3.177 |
| Whole body fat-free mass | Alzheimer's disease, wide (FinnGen) | rs6693481 | 1:203766395 | C/T | 0.695 | -0.0083 (0.0013) | 8.6E-10 | -0.0272 (0.0222) | 0.220 | 37.6 | 3.292 |
| Whole body fat-free mass | Alzheimer's disease, wide (FinnGen) | rs2807742 | 10:28781367 | A/G | 0.771 | -0.0091 (0.0015) | 8.6E-10 | -0.0202 (0.0267) | 0.450 | 37.6 | 2.219 |
| Whole body fat-free mass | Alzheimer's disease, wide (FinnGen) | rs161799 | 1:8015560 | G/A | 0.344 | -0.0080 (0.0013) | 9.0E-10 | -0.0391 (0.0232) | 0.092 | 37.5 | 4.869 |
| Whole body fat-free mass | Alzheimer's disease, wide (FinnGen) | rs72754922 | 5:42547714 | A/G | 0.061 | -0.0160 (0.0026) | 9.1E-10 | 0.0186 (0.0492) | 0.706 | 37.5 | -1.166 |
| Whole body fat-free mass | Alzheimer's disease, wide (FinnGen) | rs35665085 | 22:17625915 | A/G | 0.056 | -0.0166 (0.0027) | 9.2E-10 | -0.0064 (0.0474) | 0.893 | 37.5 | 0.386 |
| Whole body fat-free mass | Alzheimer's disease, wide (FinnGen) | rs34227797 | 4:159982551 | C/G | 0.310 | 0.0082 (0.0013) | 9.3E-10 | -0.0017 (0.0230) | 0.942 | 37.5 | -0.207 |
| Whole body fat-free mass | Alzheimer's disease, wide (FinnGen) | rs2611732 | 5:95855576 | G/A | 0.602 | 0.0077 (0.0013) | 9.3E-10 | -0.0152 (0.0230) | 0.508 | 37.5 | -1.962 |
| Whole body fat-free mass | Alzheimer's disease, wide (FinnGen) | rs4980826 | 12:578349 | A/C | 0.396 | 0.0078 (0.0013) | 9.3E-10 | 0.0382 (0.0219) | 0.081 | 37.5 | 4.927 |
| Whole body fat-free mass | Alzheimer's disease, wide (FinnGen) | rs1910466 | 3:147086268 | C/T | 0.498 | -0.0076 (0.0012) | 9.4E-10 | -0.0134 (0.0213) | 0.530 | 37.5 | 1.756 |
| Whole body fat-free mass | Alzheimer's disease, wide (FinnGen) | rs56207600 | 11:126196537 | A/G | 0.111 | 0.0122 (0.0020) | 9.6E-10 | -0.0253 (0.0312) | 0.417 | 37.4 | -2.080 |
| Whole body fat-free mass | Alzheimer's disease, wide (FinnGen) | rs11925245 | 3:114183579 | G/A | 0.183 | -0.0099 (0.0016) | 1.0E-09 | -0.0059 (0.0271) | 0.828 | 37.3 | 0.596 |
| Whole body fat-free mass | Alzheimer's disease, wide (FinnGen) | rs1928850 | 9:16765520 | T/A | 0.922 | 0.0142 (0.0023) | 1.0E-09 | -0.0650 (0.0378) | 0.085 | 37.3 | -4.567 |
| Whole body fat-free mass | Alzheimer's disease, wide (FinnGen) | rs1941697 | 18:31251276 | A/G | 0.449 | 0.0076 (0.0013) | 1.0E-09 | 0.0263 (0.0213) | 0.217 | 37.3 | 3.444 |
| Whole body fat-free mass | Alzheimer's disease, wide (FinnGen) | rs4887925 | 16:73784416 | C/G | 0.413 | -0.0077 (0.0013) | 1.2E-09 | 0.0067 (0.0213) | 0.753 | 36.9 | -0.870 |
| Whole body fat-free mass | Alzheimer's disease, wide (FinnGen) | rs6781248 | 3:13726684 | A/G | 0.546 | 0.0076 (0.0012) | 1.3E-09 | 0.0285 (0.0217) | 0.190 | 36.8 | 3.771 |
| Whole body fat-free mass | Alzheimer's disease, wide (FinnGen) | rs3853474 | 5:141816555 | T/C | 0.377 | -0.0078 (0.0013) | 1.3E-09 | -0.0086 (0.0220) | 0.697 | 36.8 | 1.109 |
| Whole body fat-free mass | Alzheimer's disease, wide (FinnGen) | rs9654451 | 5:299543 | T/C | 0.129 | 0.0112 (0.0019) | 1.4E-09 | 0.0342 (0.0370) | 0.356 | 36.7 | 3.047 |
| Whole body fat-free mass | Alzheimer's disease, wide (FinnGen) | rs8002779 | 13:92015977 | A/G | 0.603 | -0.0077 (0.0013) | 1.5E-09 | -0.0011 (0.0212) | 0.959 | 36.5 | 0.144 |
| Whole body fat-free mass | Alzheimer's disease, wide (FinnGen) | rs2521349 | 17:67503501 | A/G | 0.385 | 0.0077 (0.0013) | 1.6E-09 | 0.0149 (0.0216) | 0.491 | 36.4 | 1.926 |
| Whole body fat-free mass | Alzheimer's disease, wide (FinnGen) | rs695922 | 5:53385529 | G/A | 0.837 | -0.0102 (0.0017) | 1.7E-09 | -0.0030 (0.0286) | 0.918 | 36.4 | 0.293 |
| Whole body fat-free mass | Alzheimer's disease, wide (FinnGen) | rs6563808 | 13:40766662 | C/T | 0.734 | -0.0085 (0.0014) | 1.7E-09 | -0.0248 (0.0227) | 0.273 | 36.3 | 2.908 |
| Whole body fat-free mass | Alzheimer's disease, wide (FinnGen) | rs5771118 | 22:50714289 | C/T | 0.742 | 0.0086 (0.0014) | 1.8E-09 | -0.0049 (0.0262) | 0.852 | 36.2 | -0.568 |
| Whole body fat-free mass | Alzheimer's disease, wide (FinnGen) | rs41458449 | 2:46929951 | C/G | 0.144 | -0.0106 (0.0018) | 1.9E-09 | 0.0290 (0.0271) | 0.286 | 36.1 | -2.731 |
| Whole body fat-free mass | Alzheimer's disease, wide (FinnGen) | rs6721191 | 2:10190115 | G/A | 0.578 | -0.0075 (0.0013) | 2.0E-09 | 0.0019 (0.0239) | 0.936 | 36.0 | -0.253 |
| Whole body fat-free mass | Alzheimer's disease, wide (FinnGen) | rs72703409 | 4:184225295 | C/G | 0.066 | -0.0150 (0.0025) | 2.0E-09 | 0.0116 (0.0562) | 0.836 | 35.9 | -0.772 |
| Whole body fat-free mass | Alzheimer's disease, wide (FinnGen) | rs572474 | 10:89791654 | G/A | 0.166 | 0.0100 (0.0017) | 2.0E-09 | 0.0107 (0.0284) | 0.708 | 36.0 | 1.067 |
| Whole body fat-free mass | Alzheimer's disease, wide (FinnGen) | rs9898189 | 17:80480516 | G/C | 0.644 | -0.0081 (0.0013) | 2.1E-09 | 0.0248 (0.0217) | 0.252 | 35.9 | -3.068 |
| Whole body fat-free mass | Alzheimer's disease, wide (FinnGen) | rs12731187 | 1:202013757 | T/C | 0.358 | -0.0078 (0.0013) | 2.5E-09 | 0.0021 (0.0218) | 0.924 | 35.5 | -0.270 |
| Whole body fat-free mass | Alzheimer's disease, wide (FinnGen) | rs9365939 | 6:166336825 | G/A | 0.548 | -0.0074 (0.0012) | 2.5E-09 | 0.0220 (0.0221) | 0.319 | 35.5 | -2.973 |
| Whole body fat-free mass | Alzheimer's disease, wide (FinnGen) | rs56130943 | 14:105906522 | C/A | 0.233 | 0.0090 (0.0015) | 2.9E-09 | -0.0152 (0.0269) | 0.573 | 35.3 | -1.694 |
| Whole body fat-free mass | Alzheimer's disease, wide (FinnGen) | rs148662000 | 1:228721530 | T/G | 0.064 | -0.0151 (0.0025) | 3.0E-09 | 0.0136 (0.0511) | 0.791 | 35.2 | -0.901 |
| Whole body fat-free mass | Alzheimer's disease, wide (FinnGen) | rs10939792 | 4:18483405 | G/C | 0.320 | 0.0079 (0.0013) | 3.2E-09 | 0.0054 (0.0243) | 0.823 | 35.1 | 0.686 |
| Whole body fat-free mass | Alzheimer's disease, wide (FinnGen) | rs876672 | 16:81571600 | G/A | 0.085 | -0.0132 (0.0022) | 3.2E-09 | 0.0074 (0.0367) | 0.840 | 35.0 | -0.559 |
| Whole body fat-free mass | Alzheimer's disease, wide (FinnGen) | rs7535501 | 1:176462695 | T/C | 0.944 | -0.0159 (0.0027) | 3.3E-09 | 0.0603 (0.0540) | 0.264 | 35.0 | -3.785 |
| Whole body fat-free mass | Alzheimer's disease, wide (FinnGen) | rs116337081 | 2:183954625 | T/C | 0.070 | 0.0143 (0.0024) | 3.6E-09 | 0.0828 (0.0501) | 0.098 | 34.8 | 5.775 |
| Whole body fat-free mass | Alzheimer's disease, wide (FinnGen) | rs2920665 | 8:6316097 | G/T | 0.174 | 0.0097 (0.0016) | 3.6E-09 | 0.0306 (0.0325) | 0.345 | 34.8 | 3.153 |
| Whole body fat-free mass | Alzheimer's disease, wide (FinnGen) | rs1176314 | 13:81221014 | G/T | 0.550 | -0.0074 (0.0013) | 3.6E-09 | -0.0360 (0.0218) | 0.099 | 34.9 | 4.850 |
| Whole body fat-free mass | Alzheimer's disease, wide (FinnGen) | rs13392079 | 2:169119178 | C/T | 0.756 | -0.0085 (0.0014) | 3.8E-09 | 0.0184 (0.0228) | 0.420 | 34.7 | -2.166 |
| Whole body fat-free mass | Alzheimer's disease, wide (FinnGen) | rs224048 | 10:64491549 | A/G | 0.550 | -0.0073 (0.0012) | 3.8E-09 | 0.0407 (0.0211) | 0.054 | 34.7 | -5.543 |
| Whole body fat-free mass | Alzheimer's disease, wide (FinnGen) | rs7280982 | 21:37468223 | A/G | 0.221 | -0.0088 (0.0015) | 4.2E-09 | 0.0075 (0.0251) | 0.765 | 34.5 | -0.848 |
| Whole body fat-free mass | Alzheimer's disease, wide (FinnGen) | rs116165844 | 20:62610556 | T/G | 0.137 | -0.0106 (0.0018) | 4.3E-09 | -0.0484 (0.0335) | 0.149 | 34.5 | 4.561 |
| Whole body fat-free mass | Alzheimer's disease, wide (FinnGen) | rs29946 | 19:34299865 | C/T | 0.557 | 0.0075 (0.0013) | 4.5E-09 | -0.0125 (0.0214) | 0.560 | 34.4 | -1.673 |
| Whole body fat-free mass | Alzheimer's disease, wide (FinnGen) | rs182224 | 5:67316442 | T/C | 0.090 | 0.0127 (0.0022) | 4.6E-09 | 0.0439 (0.0326) | 0.178 | 34.4 | 3.449 |
| Whole body fat-free mass | Alzheimer's disease, wide (FinnGen) | rs6747657 | 2:112003163 | A/G | 0.282 | 0.0081 (0.0014) | 4.7E-09 | 0.0302 (0.0238) | 0.204 | 34.3 | 3.751 |
| Whole body fat-free mass | Alzheimer's disease, wide (FinnGen) | rs11263853 | 1:36579648 | A/G | 0.810 | 0.0092 (0.0016) | 4.8E-09 | -0.0298 (0.0255) | 0.243 | 34.2 | -3.225 |
| Whole body fat-free mass | Alzheimer's disease, wide (FinnGen) | rs9985795 | 4:135213286 | C/T | 0.483 | -0.0073 (0.0012) | 5.1E-09 | -0.0089 (0.0214) | 0.676 | 34.2 | 1.224 |
| Whole body fat-free mass | Alzheimer's disease, wide (FinnGen) | rs34055910 | 17:64460104 | G/A | 0.379 | -0.0075 (0.0013) | 5.1E-09 | 0.0121 (0.0212) | 0.570 | 34.2 | -1.612 |
| Whole body fat-free mass | Alzheimer's disease, wide (FinnGen) | rs13185520 | 5:137807260 | A/G | 0.574 | -0.0073 (0.0013) | 5.4E-09 | 0.0111 (0.0221) | 0.614 | 34.1 | -1.519 |
| Whole body fat-free mass | Alzheimer's disease, wide (FinnGen) | rs116817990 | 1:178989260 | G/A | 0.034 | -0.0201 (0.0034) | 5.7E-09 | -0.0589 (0.0589) | 0.317 | 33.9 | 2.931 |
| Whole body fat-free mass | Alzheimer's disease, wide (FinnGen) | rs3772051 | 2:241672904 | A/G | 0.237 | -0.0086 (0.0015) | 5.9E-09 | -0.0185 (0.0333) | 0.578 | 33.9 | 2.149 |
| Whole body fat-free mass | Alzheimer's disease, wide (FinnGen) | rs236587 | 17:68203915 | C/T | 0.737 | -0.0083 (0.0014) | 6.2E-09 | -0.0334 (0.0232) | 0.151 | 33.8 | 4.028 |
| Whole body fat-free mass | Alzheimer's disease, wide (FinnGen) | rs118173451 | 22:28356600 | C/T | 0.016 | -0.0292 (0.0050) | 6.3E-09 | 0.0457 (0.0892) | 0.608 | 33.7 | -1.567 |
| Whole body fat-free mass | Alzheimer's disease, wide (FinnGen) | rs6500249 | 16:49778147 | G/A | 0.735 | -0.0082 (0.0014) | 6.4E-09 | 0.0195 (0.0267) | 0.464 | 33.7 | -2.373 |
| Whole body fat-free mass | Alzheimer's disease, wide (FinnGen) | rs5753630 | 22:31861950 | G/A | 0.439 | 0.0073 (0.0013) | 6.7E-09 | -0.0144 (0.0212) | 0.497 | 33.6 | -1.971 |
| Whole body fat-free mass | Alzheimer's disease, wide (FinnGen) | rs237738 | 20:47904920 | C/T | 0.823 | 0.0094 (0.0016) | 7.3E-09 | -0.0167 (0.0281) | 0.552 | 33.5 | -1.772 |
| Whole body fat-free mass | Alzheimer's disease, wide (FinnGen) | rs9800418 | 5:77641008 | C/T | 0.245 | 0.0084 (0.0014) | 7.9E-09 | 0.0053 (0.0243) | 0.828 | 33.3 | 0.634 |
| Whole body fat-free mass | Alzheimer's disease, wide (FinnGen) | rs1421035 | 10:131136401 | T/C | 0.348 | 0.0076 (0.0013) | 8.0E-09 | -0.0299 (0.0221) | 0.176 | 33.3 | -3.942 |
| Whole body fat-free mass | Alzheimer's disease, wide (FinnGen) | rs10020631 | 4:69353863 | A/G | 0.248 | -0.0083 (0.0014) | 8.2E-09 | -0.0136 (0.0248) | 0.585 | 33.2 | 1.636 |
| Whole body fat-free mass | Alzheimer's disease, wide (FinnGen) | rs4676442 | 2:241740907 | C/T | 0.356 | 0.0074 (0.0013) | 9.1E-09 | -0.0032 (0.0216) | 0.884 | 33.0 | -0.431 |
| Whole body fat-free mass | Alzheimer's disease, wide (FinnGen) | rs9491201 | 6:124934212 | T/C | 0.164 | 0.0096 (0.0017) | 9.5E-09 | 0.0125 (0.0415) | 0.763 | 32.9 | 1.302 |
| Whole body fat-free mass | Alzheimer's disease, wide (FinnGen) | rs1846221 | 10:4667302 | C/G | 0.175 | -0.0095 (0.0017) | 9.7E-09 | 0.0275 (0.0280) | 0.326 | 32.9 | -2.893 |
| Whole body fat-free mass | Alzheimer's disease, wide (FinnGen) | rs35651070 | 2:121613739 | A/G | 0.167 | -0.0095 (0.0017) | 9.9E-09 | 0.0076 (0.0296) | 0.798 | 32.9 | -0.800 |
| Whole body fat-free mass | Alzheimer's disease, wide (FinnGen) | rs2920974 | 2:55537126 | C/A | 0.560 | 0.0072 (0.0013) | 1.1E-08 | -0.0194 (0.0229) | 0.396 | 32.7 | -2.712 |
| Whole body fat-free mass | Alzheimer's disease, wide (FinnGen) | rs35612982 | 6:20682622 | C/T | 0.178 | -0.0092 (0.0016) | 1.1E-08 | -0.0142 (0.0276) | 0.606 | 32.7 | 1.536 |
| Whole body fat-free mass | Alzheimer's disease, wide (FinnGen) | rs8091374 | 18:71904385 | A/G | 0.161 | -0.0099 (0.0017) | 1.1E-08 | -0.0187 (0.0271) | 0.491 | 32.7 | 1.894 |
| Whole body fat-free mass | Alzheimer's disease, wide (FinnGen) | rs34345560 | 11:69081998 | A/G | 0.195 | 0.0090 (0.0016) | 1.2E-08 | 0.0139 (0.0245) | 0.569 | 32.4 | 1.545 |
| Whole body fat-free mass | Alzheimer's disease, wide (FinnGen) | rs6743107 | 2:100844781 | T/C | 0.275 | 0.0079 (0.0014) | 1.3E-08 | 0.0233 (0.0230) | 0.311 | 32.3 | 2.961 |
| Whole body fat-free mass | Alzheimer's disease, wide (FinnGen) | rs9783665 | 14:103271158 | A/T | 0.653 | -0.0074 (0.0013) | 1.3E-08 | 0.0196 (0.0223) | 0.379 | 32.3 | -2.642 |
| Whole body fat-free mass | Alzheimer's disease, wide (FinnGen) | rs8088739 | 18:60767088 | C/T | 0.244 | 0.0082 (0.0014) | 1.3E-08 | 0.0282 (0.0251) | 0.262 | 32.3 | 3.428 |
| Whole body fat-free mass | Alzheimer's disease, wide (FinnGen) | rs34831515 | 19:17275777 | T/C | 0.232 | -0.0084 (0.0015) | 1.3E-08 | 0.0355 (0.0229) | 0.120 | 32.3 | -4.225 |
| Whole body fat-free mass | Alzheimer's disease, wide (FinnGen) | rs35050648 | 19:46991243 | T/G | 0.236 | 0.0083 (0.0015) | 1.6E-08 | 0.0308 (0.0244) | 0.206 | 32.0 | 3.717 |
| Whole body fat-free mass | Alzheimer's disease, wide (FinnGen) | rs62460525 | 7:44902676 | A/C | 0.070 | 0.0137 (0.0024) | 1.7E-08 | 0.0052 (0.0619) | 0.932 | 31.8 | 0.379 |
| Whole body fat-free mass | Alzheimer's disease, wide (FinnGen) | rs2740761 | 7:55255986 | T/C | 0.208 | 0.0087 (0.0015) | 1.7E-08 | 0.0095 (0.0269) | 0.723 | 31.8 | 1.089 |
| Whole body fat-free mass | Alzheimer's disease, wide (FinnGen) | rs113978196 | 15:75916965 | A/G | 0.251 | -0.0081 (0.0014) | 1.8E-08 | 0.0567 (0.0238) | 0.017 | 31.8 | -7.019 |
| Whole body fat-free mass | Alzheimer's disease, wide (FinnGen) | rs1043801 | 21:47552527 | A/G | 0.058 | 0.0151 (0.0027) | 1.8E-08 | -0.0820 (0.0446) | 0.066 | 31.7 | -5.438 |
| Whole body fat-free mass | Alzheimer's disease, wide (FinnGen) | rs10848288 | 12:131600054 | T/C | 0.304 | -0.0076 (0.0014) | 1.9E-08 | -0.0098 (0.0239) | 0.681 | 31.6 | 1.288 |
| Whole body fat-free mass | Alzheimer's disease, wide (FinnGen) | rs3751866 | 16:84771304 | C/T | 0.247 | -0.0081 (0.0014) | 1.9E-08 | -0.0029 (0.0249) | 0.907 | 31.6 | 0.357 |
| Whole body fat-free mass | Alzheimer's disease, wide (FinnGen) | rs111598585 | 4:171635471 | T/C | 0.209 | -0.0086 (0.0015) | 2.0E-08 | -0.0344 (0.0350) | 0.326 | 31.5 | 4.007 |
| Whole body fat-free mass | Alzheimer's disease, wide (FinnGen) | rs2884345 | 14:53323211 | A/G | 0.710 | -0.0077 (0.0014) | 2.1E-08 | -0.0442 (0.0234) | 0.059 | 31.4 | 5.748 |
| Whole body fat-free mass | Alzheimer's disease, wide (FinnGen) | rs2252909 | 17:2278609 | T/C | 0.657 | -0.0075 (0.0013) | 2.1E-08 | -0.0249 (0.0215) | 0.247 | 31.4 | 3.336 |
| Whole body fat-free mass | Alzheimer's disease, wide (FinnGen) | rs12553221 | 9:37234008 | A/G | 0.362 | 0.0073 (0.0013) | 2.2E-08 | 0.0044 (0.0221) | 0.843 | 31.3 | 0.606 |
| Whole body fat-free mass | Alzheimer's disease, wide (FinnGen) | rs498685 | 18:13088673 | C/T | 0.594 | -0.0071 (0.0013) | 2.2E-08 | -0.0109 (0.0214) | 0.609 | 31.3 | 1.539 |
| Whole body fat-free mass | Alzheimer's disease, wide (FinnGen) | rs17011108 | 4:86800806 | C/T | 0.307 | 0.0076 (0.0014) | 2.3E-08 | 0.0359 (0.0237) | 0.130 | 31.2 | 4.742 |
| Whole body fat-free mass | Alzheimer's disease, wide (FinnGen) | rs12795042 | 11:133658168 | C/A | 0.622 | 0.0073 (0.0013) | 2.3E-08 | 0.0003 (0.0233) | 0.991 | 31.3 | 0.041 |
| Whole body fat-free mass | Alzheimer's disease, wide (FinnGen) | rs4800670 | 18:23415263 | C/G | 0.386 | -0.0072 (0.0013) | 2.3E-08 | -0.0404 (0.0218) | 0.064 | 31.3 | 5.635 |
| Whole body fat-free mass | Alzheimer's disease, wide (FinnGen) | rs73189390 | 21:17383170 | A/G | 0.184 | -0.0090 (0.0016) | 2.3E-08 | -0.0530 (0.0267) | 0.047 | 31.3 | 5.860 |
| Whole body fat-free mass | Alzheimer's disease, wide (FinnGen) | rs55854145 | 18:45928049 | C/A | 0.055 | -0.0153 (0.0027) | 2.4E-08 | -0.0437 (0.0545) | 0.422 | 31.1 | 2.853 |
| Whole body fat-free mass | Alzheimer's disease, wide (FinnGen) | rs7513326 | 1:159895536 | A/G | 0.508 | -0.0070 (0.0013) | 2.5E-08 | 0.0114 (0.0214) | 0.594 | 31.1 | -1.626 |
| Whole body fat-free mass | Alzheimer's disease, wide (FinnGen) | rs73158212 | 7:148383377 | G/A | 0.224 | -0.0084 (0.0015) | 2.5E-08 | -0.0635 (0.0284) | 0.025 | 31.1 | 7.562 |
| Whole body fat-free mass | Alzheimer's disease, wide (FinnGen) | rs6514066 | 20:44500794 | G/T | 0.624 | 0.0072 (0.0013) | 2.5E-08 | -0.0514 (0.0219) | 0.019 | 31.1 | -7.170 |
| Whole body fat-free mass | Alzheimer's disease, wide (FinnGen) | rs2427320 | 20:60976723 | G/A | 0.232 | -0.0082 (0.0015) | 2.5E-08 | -0.0005 (0.0285) | 0.987 | 31.1 | 0.061 |
| Whole body fat-free mass | Alzheimer's disease, wide (FinnGen) | rs4821940 | 22:40659573 | C/T | 0.550 | -0.0070 (0.0013) | 2.5E-08 | 0.0027 (0.0212) | 0.898 | 31.1 | -0.386 |
| Whole body fat-free mass | Alzheimer's disease, wide (FinnGen) | rs1168768 | 12:66509650 | T/C | 0.975 | 0.0221 (0.0040) | 2.6E-08 | 0.0649 (0.0713) | 0.362 | 31.0 | 2.933 |
| Whole body fat-free mass | Alzheimer's disease, wide (FinnGen) | rs7095768 | 10:53256505 | T/G | 0.263 | 0.0078 (0.0014) | 2.7E-08 | -0.0421 (0.0247) | 0.088 | 30.9 | -5.378 |
| Whole body fat-free mass | Alzheimer's disease, wide (FinnGen) | rs2293177 | 7:140244560 | T/C | 0.313 | 0.0074 (0.0013) | 2.9E-08 | 0.0406 (0.0224) | 0.070 | 30.8 | 5.470 |
| Whole body fat-free mass | Alzheimer's disease, wide (FinnGen) | rs13125082 | 4:57787000 | G/T | 0.278 | -0.0076 (0.0014) | 3.4E-08 | -0.0182 (0.0257) | 0.480 | 30.5 | 2.384 |
| Whole body fat-free mass | Alzheimer's disease, wide (FinnGen) | rs7683836 | 4:180167906 | A/G | 0.557 | -0.0069 (0.0013) | 3.4E-08 | 0.0215 (0.0212) | 0.311 | 30.5 | -3.112 |
| Whole body fat-free mass | Alzheimer's disease, wide (FinnGen) | rs926436 | 1:22563429 | A/G | 0.857 | 0.0098 (0.0018) | 3.5E-08 | -0.0360 (0.0297) | 0.226 | 30.4 | -3.681 |
| Whole body fat-free mass | Alzheimer's disease, wide (FinnGen) | rs34345690 | 3:192445575 | G/A | 0.227 | 0.0082 (0.0015) | 3.5E-08 | -0.0156 (0.0266) | 0.558 | 30.4 | -1.913 |
| Whole body fat-free mass | Alzheimer's disease, wide (FinnGen) | rs11021307 | 11:95501407 | T/C | 0.418 | 0.0069 (0.0013) | 3.6E-08 | 0.0131 (0.0223) | 0.556 | 30.4 | 1.890 |
| Whole body fat-free mass | Alzheimer's disease, wide (FinnGen) | rs294382 | 11:30607515 | C/A | 0.720 | -0.0076 (0.0014) | 4.0E-08 | 0.0296 (0.0255) | 0.246 | 30.1 | -3.894 |
| Whole body fat-free mass | Alzheimer's disease, wide (FinnGen) | rs434072 | 3:12916946 | G/T | 0.709 | -0.0075 (0.0014) | 4.1E-08 | -0.0151 (0.0225) | 0.502 | 30.1 | 2.006 |
| Whole body fat-free mass | Alzheimer's disease, wide (FinnGen) | rs1028197 | 4:124977659 | T/C | 0.225 | 0.0082 (0.0015) | 4.1E-08 | 0.0069 (0.0284) | 0.809 | 30.1 | 0.842 |
| Whole body fat-free mass | Alzheimer's disease, wide (FinnGen) | rs12213409 | 6:142610314 | G/T | 0.485 | -0.0068 (0.0012) | 4.7E-08 | -0.0030 (0.0212) | 0.888 | 29.8 | 0.440 |
| Walking pace | Alzheimer's disease (Bellenguez 2022) | rs13107325 | 4:103188709 | T/C | 0.075 | -0.0242 (0.0024) | 7.1E-24 | 0.0455 (0.0160) | 0.004 | 101.5 | -1.877 |
| Walking pace | Alzheimer's disease (Bellenguez 2022) | rs613872 | 18:53210302 | T/G | 0.826 | -0.0147 (0.0017) | 1.3E-18 | 0.0020 (0.0107) | 0.852 | 77.5 | -0.136 |
| Walking pace | Alzheimer's disease (Bellenguez 2022) | rs11039324 | 11:47665686 | A/G | 0.404 | -0.0102 (0.0013) | 2.2E-15 | -0.0243 (0.0083) | 0.003 | 62.9 | 2.380 |
| Walking pace | Alzheimer's disease (Bellenguez 2022) | rs2280406 | 3:49941436 | A/G | 0.506 | -0.0100 (0.0013) | 3.4E-15 | -0.0101 (0.0081) | 0.214 | 62.0 | 1.014 |
| Walking pace | Alzheimer's disease (Bellenguez 2022) | rs7896518 | 10:65104500 | G/A | 0.428 | 0.0100 (0.0013) | 1.1E-14 | -0.0297 (0.0082) | 2.9E-04 | 59.7 | -2.973 |
| Walking pace | Alzheimer's disease (Bellenguez 2022) | rs55872725 | 16:53809123 | T/C | 0.404 | -0.0099 (0.0013) | 1.9E-14 | -0.0153 (0.0082) | 0.063 | 58.6 | 1.549 |
| Walking pace | Alzheimer's disease (Bellenguez 2022) | rs273512 | 19:18224729 | T/C | 0.405 | -0.0097 (0.0013) | 6.9E-14 | 0.0135 (0.0088) | 0.126 | 56.1 | -1.397 |
| Walking pace | Alzheimer's disease (Bellenguez 2022) | rs9366651 | 6:26336696 | T/G | 0.507 | 0.0095 (0.0013) | 8.6E-14 | -0.0100 (0.0082) | 0.221 | 55.7 | -1.054 |
| Walking pace | Alzheimer's disease (Bellenguez 2022) | rs11150623 | 16:28881001 | T/G | 0.645 | -0.0097 (0.0013) | 2.4E-13 | -0.0130 (0.0085) | 0.126 | 53.7 | 1.342 |
| Walking pace | Alzheimer's disease (Bellenguez 2022) | rs7795394 | 7:113560607 | A/T | 0.625 | 0.0093 (0.0013) | 1.2E-12 | -0.0029 (0.0085) | 0.735 | 50.6 | -0.312 |
| Walking pace | Alzheimer's disease (Bellenguez 2022) | rs12042959 | 1:243533273 | G/A | 0.144 | 0.0126 (0.0018) | 4.1E-12 | 0.0021 (0.0114) | 0.857 | 48.1 | 0.167 |
| Walking pace | Alzheimer's disease (Bellenguez 2022) | rs2297600 | 1:32207581 | G/T | 0.172 | -0.0116 (0.0017) | 6.2E-12 | -0.0224 (0.0109) | 0.040 | 47.3 | 1.938 |
| Walking pace | Alzheimer's disease (Bellenguez 2022) | rs10828258 | 10:21929734 | G/A | 0.319 | -0.0093 (0.0014) | 6.7E-12 | 0.0030 (0.0086) | 0.730 | 47.1 | -0.322 |
| Walking pace | Alzheimer's disease (Bellenguez 2022) | rs57800857 | 4:140863365 | C/A | 0.365 | 0.0087 (0.0013) | 5.3E-11 | 0.0043 (0.0086) | 0.615 | 43.1 | 0.496 |
| Walking pace | Alzheimer's disease (Bellenguez 2022) | rs2645979 | 12:84017043 | A/G | 0.357 | 0.0086 (0.0013) | 6.7E-11 | 0.0010 (0.0085) | 0.910 | 42.6 | 0.116 |
| Walking pace | Alzheimer's disease (Bellenguez 2022) | rs4839898 | 6:97546759 | A/G | 0.110 | 0.0131 (0.0021) | 2.3E-10 | -0.0158 (0.0140) | 0.258 | 40.2 | -1.210 |
| Walking pace | Alzheimer's disease (Bellenguez 2022) | rs11548200 | 1:156290656 | C/T | 0.066 | -0.0160 (0.0026) | 3.3E-10 | -0.0308 (0.0177) | 0.083 | 39.5 | 1.920 |
| Walking pace | Alzheimer's disease (Bellenguez 2022) | rs8010773 | 14:46956863 | C/T | 0.382 | -0.0082 (0.0013) | 3.4E-10 | -0.0083 (0.0084) | 0.325 | 39.4 | 1.016 |
| Walking pace | Alzheimer's disease (Bellenguez 2022) | rs6763292 | 3:129044705 | G/A | 0.781 | 0.0096 (0.0015) | 3.8E-10 | -0.0118 (0.0100) | 0.236 | 39.2 | -1.231 |
| Walking pace | Alzheimer's disease (Bellenguez 2022) | rs10862220 | 12:81430599 | G/T | 0.675 | 0.0084 (0.0014) | 4.8E-10 | 0.0104 (0.0086) | 0.229 | 38.8 | 1.237 |
| Walking pace | Alzheimer's disease (Bellenguez 2022) | rs891387 | 18:21103909 | C/T | 0.495 | 0.0079 (0.0013) | 5.4E-10 | 0.0198 (0.0081) | 0.015 | 38.5 | 2.519 |
| Walking pace | Alzheimer's disease (Bellenguez 2022) | rs55680124 | 2:105984624 | T/C | 0.155 | -0.0107 (0.0018) | 8.3E-10 | -0.0165 (0.0116) | 0.155 | 37.7 | 1.536 |
| Walking pace | Alzheimer's disease (Bellenguez 2022) | rs10750025 | 11:113424042 | T/C | 0.682 | -0.0084 (0.0014) | 9.4E-10 | -0.0121 (0.0087) | 0.165 | 37.5 | 1.448 |
| Walking pace | Alzheimer's disease (Bellenguez 2022) | rs12883788 | 14:33303540 | T/C | 0.460 | -0.0078 (0.0013) | 9.4E-10 | -0.0015 (0.0082) | 0.850 | 37.5 | 0.192 |
| Walking pace | Alzheimer's disease (Bellenguez 2022) | rs4516268 | 17:1846831 | A/C | 0.194 | 0.0098 (0.0016) | 9.5E-10 | -0.0101 (0.0105) | 0.337 | 37.4 | -1.027 |
| Walking pace | Alzheimer's disease (Bellenguez 2022) | rs205262 | 6:34563164 | G/A | 0.269 | -0.0087 (0.0014) | 1.0E-09 | -0.0198 (0.0091) | 0.028 | 37.3 | 2.273 |
| Walking pace | Alzheimer's disease (Bellenguez 2022) | rs143384 | 20:34025756 | G/A | 0.404 | 0.0079 (0.0013) | 1.1E-09 | -0.0057 (0.0082) | 0.487 | 37.1 | -0.726 |
| Walking pace | Alzheimer's disease (Bellenguez 2022) | rs10883618 | 10:103117653 | A/G | 0.372 | 0.0078 (0.0013) | 2.4E-09 | -0.0068 (0.0083) | 0.412 | 35.6 | -0.871 |
| Walking pace | Alzheimer's disease (Bellenguez 2022) | rs11682482 | 2:226486479 | G/T | 0.681 | 0.0080 (0.0014) | 3.3E-09 | 0.0053 (0.0089) | 0.549 | 35.0 | 0.661 |
| Walking pace | Alzheimer's disease (Bellenguez 2022) | rs12461902 | 19:30265235 | A/G | 0.330 | -0.0080 (0.0014) | 3.6E-09 | -0.0074 (0.0094) | 0.435 | 34.8 | 0.924 |
| Walking pace | Alzheimer's disease (Bellenguez 2022) | rs11761141 | 7:69423362 | G/T | 0.326 | -0.0079 (0.0014) | 5.0E-09 | -0.0098 (0.0087) | 0.259 | 34.2 | 1.241 |
| Walking pace | Alzheimer's disease (Bellenguez 2022) | rs113825410 | 1:40057543 | G/A | 0.224 | -0.0088 (0.0015) | 6.4E-09 | 0.0176 (0.0097) | 0.069 | 33.7 | -1.997 |
| Walking pace | Alzheimer's disease (Bellenguez 2022) | rs4109292 | 10:126710654 | A/G | 0.494 | 0.0074 (0.0013) | 6.6E-09 | 0.0075 (0.0081) | 0.357 | 33.6 | 1.020 |
| Walking pace | Alzheimer's disease (Bellenguez 2022) | rs2037735 | 19:48012469 | T/C | 0.121 | -0.0113 (0.0019) | 6.7E-09 | -0.0206 (0.0136) | 0.131 | 33.6 | 1.828 |
| Walking pace | Alzheimer's disease (Bellenguez 2022) | rs1592 | 2:135722143 | C/A | 0.395 | 0.0075 (0.0013) | 7.3E-09 | 0.0391 (0.0084) | 3.5E-06 | 33.5 | 5.247 |
| Walking pace | Alzheimer's disease (Bellenguez 2022) | rs830627 | 3:71675270 | A/G | 0.417 | 0.0074 (0.0013) | 7.7E-09 | 0.0053 (0.0083) | 0.522 | 33.4 | 0.714 |
| Walking pace | Alzheimer's disease (Bellenguez 2022) | rs11848096 | 14:100969235 | C/T | 0.387 | -0.0075 (0.0013) | 9.2E-09 | -0.0010 (0.0084) | 0.903 | 33.0 | 0.133 |
| Walking pace | Alzheimer's disease (Bellenguez 2022) | rs11732213 | 4:1704244 | C/T | 0.196 | 0.0092 (0.0016) | 9.4E-09 | 0.0218 (0.0103) | 0.034 | 33.0 | 2.380 |
| Walking pace | Alzheimer's disease (Bellenguez 2022) | rs4715208 | 6:50829471 | G/A | 0.753 | -0.0084 (0.0015) | 1.1E-08 | -0.0043 (0.0092) | 0.639 | 32.7 | 0.513 |
| Walking pace | Alzheimer's disease (Bellenguez 2022) | rs2439823 | 10:99778226 | G/A | 0.546 | -0.0073 (0.0013) | 1.1E-08 | -0.0064 (0.0083) | 0.438 | 32.6 | 0.879 |
| Walking pace | Alzheimer's disease (Bellenguez 2022) | rs28519617 | 3:135874930 | G/T | 0.270 | -0.0081 (0.0014) | 1.4E-08 | 0.0163 (0.0093) | 0.078 | 32.2 | -2.005 |
| Walking pace | Alzheimer's disease (Bellenguez 2022) | rs2644135 | 1:201856256 | G/C | 0.656 | 0.0075 (0.0013) | 1.7E-08 | 0.0131 (0.0087) | 0.133 | 31.8 | 1.744 |
| Walking pace | Alzheimer's disease (Bellenguez 2022) | rs35711462 | 5:50847577 | G/A | 0.511 | -0.0072 (0.0013) | 1.7E-08 | 0.0025 (0.0081) | 0.759 | 31.9 | -0.349 |
| Walking pace | Alzheimer's disease (Bellenguez 2022) | rs2602731 | 19:4944771 | G/A | 0.681 | -0.0077 (0.0014) | 2.1E-08 | 0.0145 (0.0093) | 0.120 | 31.4 | -1.891 |
| Walking pace | Alzheimer's disease (Bellenguez 2022) | rs144333966 | 4:61109385 | G/A | 0.015 | 0.0305 (0.0054) | 2.2E-08 | -0.0346 (0.0345) | 0.317 | 31.3 | -1.136 |
| Walking pace | Alzheimer's disease (Bellenguez 2022) | rs68085814 | 17:47135875 | A/G | 0.300 | 0.0077 (0.0014) | 2.3E-08 | 0.0047 (0.0090) | 0.602 | 31.2 | 0.608 |
| Walking pace | Alzheimer's disease (Bellenguez 2022) | rs9783304 | 11:43660255 | T/G | 0.689 | 0.0076 (0.0014) | 2.4E-08 | 0.0033 (0.0088) | 0.704 | 31.1 | 0.432 |
| Walking pace | Alzheimer's disease (Bellenguez 2022) | rs8028757 | 15:75822794 | T/A | 0.130 | 0.0107 (0.0019) | 2.4E-08 | 0.0043 (0.0124) | 0.727 | 31.1 | 0.401 |
| Walking pace | Alzheimer's disease (Bellenguez 2022) | rs8011870 | 14:80173397 | A/G | 0.288 | -0.0078 (0.0014) | 2.6E-08 | 0.0051 (0.0094) | 0.588 | 31.0 | -0.652 |
| Walking pace | Alzheimer's disease (Bellenguez 2022) | rs7789719 | 7:66893916 | C/T | 0.782 | 0.0085 (0.0015) | 2.7E-08 | 0.0034 (0.0098) | 0.728 | 30.9 | 0.398 |
| Walking pace | Alzheimer's disease (Bellenguez 2022) | rs12747822 | 1:91201451 | A/T | 0.098 | 0.0118 (0.0021) | 3.8E-08 | -0.0177 (0.0138) | 0.199 | 30.2 | -1.504 |
| Walking pace | Alzheimer's disease (Bellenguez 2022) | rs11152989 | 6:96936061 | T/C | 0.312 | -0.0075 (0.0014) | 3.9E-08 | 0.0127 (0.0088) | 0.146 | 30.2 | -1.691 |
| Walking pace | Alzheimer's disease (Bellenguez 2022) | rs139398785 | 2:161132876 | G/C | 0.140 | -0.0100 (0.0018) | 4.2E-08 | 0.0146 (0.0121) | 0.228 | 30.1 | -1.461 |
| Walking pace | Alzheimer's disease (Bellenguez 2022) | rs45583845 | 14:57858194 | G/C | 0.034 | -0.0199 (0.0036) | 4.3E-08 | -0.0083 (0.0246) | 0.736 | 30.0 | 0.417 |
| Walking pace | Alzheimer's disease (Bellenguez 2022) | rs2170670 | 12:16944621 | A/G | 0.607 | -0.0071 (0.0013) | 4.7E-08 | -0.0015 (0.0083) | 0.860 | 29.9 | 0.212 |
| Walking pace | Alzheimer's disease (Bellenguez 2022) | rs11077815 | 17:74389890 | C/T | 0.624 | -0.0071 (0.0013) | 4.8E-08 | 0.0077 (0.0084) | 0.361 | 29.8 | -1.079 |
| Walking pace | All-cause dementia (FinnGen) | rs13107325 | 4:103188709 | T/C | 0.075 | -0.0242 (0.0024) | 7.1E-24 | 0.1247 (0.0827) | 0.131 | 101.5 | -5.144 |
| Walking pace | All-cause dementia (FinnGen) | rs613872 | 18:53210302 | T/G | 0.826 | -0.0147 (0.0017) | 1.3E-18 | -0.0030 (0.0261) | 0.909 | 77.5 | 0.204 |
| Walking pace | All-cause dementia (FinnGen) | rs11039324 | 11:47665686 | A/G | 0.404 | -0.0102 (0.0013) | 2.2E-15 | -0.0198 (0.0201) | 0.324 | 62.9 | 1.939 |
| Walking pace | All-cause dementia (FinnGen) | rs2280406 | 3:49941436 | A/G | 0.506 | -0.0100 (0.0013) | 3.4E-15 | 0.0553 (0.0196) | 0.005 | 62.0 | -5.551 |
| Walking pace | All-cause dementia (FinnGen) | rs55872725 | 16:53809123 | T/C | 0.404 | -0.0099 (0.0013) | 1.9E-14 | 0.0096 (0.0194) | 0.619 | 58.6 | -0.972 |
| Walking pace | All-cause dementia (FinnGen) | rs273512 | 19:18224729 | T/C | 0.405 | -0.0097 (0.0013) | 6.9E-14 | -0.0037 (0.0193) | 0.846 | 56.1 | 0.383 |
| Walking pace | All-cause dementia (FinnGen) | rs9366651 | 6:26336696 | T/G | 0.507 | 0.0095 (0.0013) | 8.6E-14 | -0.0101 (0.0202) | 0.618 | 55.7 | -1.065 |
| Walking pace | All-cause dementia (FinnGen) | rs11150623 | 16:28881001 | T/G | 0.645 | -0.0097 (0.0013) | 2.4E-13 | -0.0512 (0.0193) | 0.008 | 53.7 | 5.285 |
| Walking pace | All-cause dementia (FinnGen) | rs7795394 | 7:113560607 | A/T | 0.625 | 0.0093 (0.0013) | 1.2E-12 | 0.0116 (0.0210) | 0.582 | 50.6 | 1.247 |
| Walking pace | All-cause dementia (FinnGen) | rs12042959 | 1:243533273 | G/A | 0.144 | 0.0126 (0.0018) | 4.1E-12 | 0.0135 (0.0261) | 0.605 | 48.1 | 1.074 |
| Walking pace | All-cause dementia (FinnGen) | rs2297600 | 1:32207581 | G/T | 0.172 | -0.0116 (0.0017) | 6.2E-12 | -0.0004 (0.0232) | 0.985 | 47.3 | 0.035 |
| Walking pace | All-cause dementia (FinnGen) | rs10828258 | 10:21929734 | G/A | 0.319 | -0.0093 (0.0014) | 6.7E-12 | -0.0105 (0.0205) | 0.609 | 47.1 | 1.126 |
| Walking pace | All-cause dementia (FinnGen) | rs57800857 | 4:140863365 | C/A | 0.365 | 0.0087 (0.0013) | 5.3E-11 | 0.6433 (0.4432) | 0.147 | 43.1 | 74.146 |
| Walking pace | All-cause dementia (FinnGen) | rs2645979 | 12:84017043 | A/G | 0.357 | 0.0086 (0.0013) | 6.7E-11 | -0.0067 (0.0192) | 0.726 | 42.6 | -0.777 |
| Walking pace | All-cause dementia (FinnGen) | rs4839898 | 6:97546759 | A/G | 0.110 | 0.0131 (0.0021) | 2.3E-10 | -0.0417 (0.0308) | 0.176 | 40.2 | -3.194 |
| Walking pace | All-cause dementia (FinnGen) | rs11548200 | 1:156290656 | C/T | 0.066 | -0.0160 (0.0026) | 3.3E-10 | 0.0199 (0.0492) | 0.686 | 39.5 | -1.241 |
| Walking pace | All-cause dementia (FinnGen) | rs8010773 | 14:46956863 | C/T | 0.382 | -0.0082 (0.0013) | 3.4E-10 | 0.0335 (0.0211) | 0.113 | 39.4 | -4.102 |
| Walking pace | All-cause dementia (FinnGen) | rs6763292 | 3:129044705 | G/A | 0.781 | 0.0096 (0.0015) | 3.8E-10 | 0.0513 (0.0250) | 0.040 | 39.2 | 5.350 |
| Walking pace | All-cause dementia (FinnGen) | rs10862220 | 12:81430599 | G/T | 0.675 | 0.0084 (0.0014) | 4.8E-10 | -0.0269 (0.0192) | 0.161 | 38.8 | -3.199 |
| Walking pace | All-cause dementia (FinnGen) | rs891387 | 18:21103909 | C/T | 0.495 | 0.0079 (0.0013) | 5.4E-10 | 0.0205 (0.0192) | 0.287 | 38.5 | 2.608 |
| Walking pace | All-cause dementia (FinnGen) | rs55680124 | 2:105984624 | T/C | 0.155 | -0.0107 (0.0018) | 8.3E-10 | -0.0091 (0.0299) | 0.760 | 37.7 | 0.847 |
| Walking pace | All-cause dementia (FinnGen) | rs10750025 | 11:113424042 | T/C | 0.682 | -0.0084 (0.0014) | 9.4E-10 | 0.0131 (0.0261) | 0.616 | 37.5 | -1.568 |
| Walking pace | All-cause dementia (FinnGen) | rs12883788 | 14:33303540 | T/C | 0.460 | -0.0078 (0.0013) | 9.4E-10 | 0.0039 (0.0194) | 0.841 | 37.5 | -0.500 |
| Walking pace | All-cause dementia (FinnGen) | rs4516268 | 17:1846831 | A/C | 0.194 | 0.0098 (0.0016) | 9.5E-10 | 0.0056 (0.0219) | 0.798 | 37.4 | 0.570 |
| Walking pace | All-cause dementia (FinnGen) | rs205262 | 6:34563164 | G/A | 0.269 | -0.0087 (0.0014) | 1.0E-09 | -0.0112 (0.0215) | 0.603 | 37.3 | 1.286 |
| Walking pace | All-cause dementia (FinnGen) | rs143384 | 20:34025756 | G/A | 0.404 | 0.0079 (0.0013) | 1.1E-09 | -0.0099 (0.0193) | 0.610 | 37.1 | -1.261 |
| Walking pace | All-cause dementia (FinnGen) | rs10883618 | 10:103117653 | A/G | 0.372 | 0.0078 (0.0013) | 2.4E-09 | -0.0190 (0.0200) | 0.342 | 35.6 | -2.433 |
| Walking pace | All-cause dementia (FinnGen) | rs11682482 | 2:226486479 | G/T | 0.681 | 0.0080 (0.0014) | 3.3E-09 | 0.0119 (0.0201) | 0.553 | 35.0 | 1.484 |
| Walking pace | All-cause dementia (FinnGen) | rs12461902 | 19:30265235 | A/G | 0.330 | -0.0080 (0.0014) | 3.6E-09 | -0.0078 (0.0204) | 0.700 | 34.8 | 0.974 |
| Walking pace | All-cause dementia (FinnGen) | rs11761141 | 7:69423362 | G/T | 0.326 | -0.0079 (0.0014) | 5.0E-09 | -0.0189 (0.0204) | 0.354 | 34.2 | 2.393 |
| Walking pace | All-cause dementia (FinnGen) | rs113825410 | 1:40057543 | G/A | 0.224 | -0.0088 (0.0015) | 6.4E-09 | -0.0306 (0.0233) | 0.190 | 33.7 | 3.473 |
| Walking pace | All-cause dementia (FinnGen) | rs4109292 | 10:126710654 | A/G | 0.494 | 0.0074 (0.0013) | 6.6E-09 | 0.0081 (0.0193) | 0.673 | 33.6 | 1.102 |
| Walking pace | All-cause dementia (FinnGen) | rs2037735 | 19:48012469 | T/C | 0.121 | -0.0113 (0.0019) | 6.7E-09 | -0.0157 (0.0272) | 0.565 | 33.6 | 1.393 |
| Walking pace | All-cause dementia (FinnGen) | rs1592 | 2:135722143 | C/A | 0.395 | 0.0075 (0.0013) | 7.3E-09 | 0.0291 (0.0193) | 0.133 | 33.5 | 3.905 |
| Walking pace | All-cause dementia (FinnGen) | rs830627 | 3:71675270 | A/G | 0.417 | 0.0074 (0.0013) | 7.7E-09 | -0.0037 (0.0191) | 0.846 | 33.4 | -0.499 |
| Walking pace | All-cause dementia (FinnGen) | rs11848096 | 14:100969235 | C/T | 0.387 | -0.0075 (0.0013) | 9.2E-09 | 0.0158 (0.0195) | 0.418 | 33.0 | -2.104 |
| Walking pace | All-cause dementia (FinnGen) | rs11732213 | 4:1704244 | C/T | 0.196 | 0.0092 (0.0016) | 9.4E-09 | -0.0211 (0.0232) | 0.364 | 33.0 | -2.303 |
| Walking pace | All-cause dementia (FinnGen) | rs4715208 | 6:50829471 | G/A | 0.753 | -0.0084 (0.0015) | 1.1E-08 | 0.0067 (0.0222) | 0.764 | 32.7 | -0.799 |
| Walking pace | All-cause dementia (FinnGen) | rs2439823 | 10:99778226 | G/A | 0.546 | -0.0073 (0.0013) | 1.1E-08 | 0.0336 (0.0191) | 0.079 | 32.6 | -4.616 |
| Walking pace | All-cause dementia (FinnGen) | rs28519617 | 3:135874930 | G/T | 0.270 | -0.0081 (0.0014) | 1.4E-08 | 0.0263 (0.0235) | 0.262 | 32.2 | -3.235 |
| Walking pace | All-cause dementia (FinnGen) | rs2644135 | 1:201856256 | G/C | 0.656 | 0.0075 (0.0013) | 1.7E-08 | -0.0055 (0.0210) | 0.793 | 31.8 | -0.732 |
| Walking pace | All-cause dementia (FinnGen) | rs35711462 | 5:50847577 | G/A | 0.511 | -0.0072 (0.0013) | 1.7E-08 | -0.0119 (0.0192) | 0.536 | 31.9 | 1.661 |
| Walking pace | All-cause dementia (FinnGen) | rs2602731 | 19:4944771 | G/A | 0.681 | -0.0077 (0.0014) | 2.1E-08 | 0.0332 (0.0224) | 0.138 | 31.4 | -4.330 |
| Walking pace | All-cause dementia (FinnGen) | rs144333966 | 4:61109385 | G/A | 0.015 | 0.0305 (0.0054) | 2.2E-08 | 0.1335 (0.0551) | 0.015 | 31.3 | 4.383 |
| Walking pace | All-cause dementia (FinnGen) | rs68085814 | 17:47135875 | A/G | 0.300 | 0.0077 (0.0014) | 2.3E-08 | -0.0030 (0.0214) | 0.887 | 31.2 | -0.388 |
| Walking pace | All-cause dementia (FinnGen) | rs9783304 | 11:43660255 | T/G | 0.689 | 0.0076 (0.0014) | 2.4E-08 | -0.0029 (0.0207) | 0.888 | 31.1 | -0.380 |
| Walking pace | All-cause dementia (FinnGen) | rs8011870 | 14:80173397 | A/G | 0.288 | -0.0078 (0.0014) | 2.6E-08 | -0.0013 (0.0192) | 0.948 | 31.0 | 0.166 |
| Walking pace | All-cause dementia (FinnGen) | rs7789719 | 7:66893916 | C/T | 0.782 | 0.0085 (0.0015) | 2.7E-08 | -0.0163 (0.0223) | 0.467 | 30.9 | -1.908 |
| Walking pace | All-cause dementia (FinnGen) | rs12747822 | 1:91201451 | A/T | 0.098 | 0.0118 (0.0021) | 3.8E-08 | 0.0376 (0.0448) | 0.401 | 30.2 | 3.195 |
| Walking pace | All-cause dementia (FinnGen) | rs11152989 | 6:96936061 | T/C | 0.312 | -0.0075 (0.0014) | 3.9E-08 | 0.0109 (0.0207) | 0.599 | 30.2 | -1.451 |
| Walking pace | All-cause dementia (FinnGen) | rs45583845 | 14:57858194 | G/C | 0.034 | -0.0199 (0.0036) | 4.3E-08 | -0.0065 (0.0575) | 0.909 | 30.0 | 0.327 |
| Walking pace | All-cause dementia (FinnGen) | rs2170670 | 12:16944621 | A/G | 0.607 | -0.0071 (0.0013) | 4.7E-08 | 0.0067 (0.0200) | 0.737 | 29.9 | -0.945 |
| Walking pace | All-cause dementia (FinnGen) | rs11077815 | 17:74389890 | C/T | 0.624 | -0.0071 (0.0013) | 4.8E-08 | -0.0277 (0.0206) | 0.178 | 29.8 | 3.883 |
| Walking pace | Vascular dementia (FinnGen) | rs13107325 | 4:103188709 | T/C | 0.075 | -0.0242 (0.0024) | 7.1E-24 | 0.1048 (0.2098) | 0.617 | 101.5 | -4.323 |
| Walking pace | Vascular dementia (FinnGen) | rs613872 | 18:53210302 | T/G | 0.826 | -0.0147 (0.0017) | 1.3E-18 | -0.1516 (0.0671) | 0.024 | 77.5 | 10.308 |
| Walking pace | Vascular dementia (FinnGen) | rs11039324 | 11:47665686 | A/G | 0.404 | -0.0102 (0.0013) | 2.2E-15 | -0.0249 (0.0515) | 0.629 | 62.9 | 2.439 |
| Walking pace | Vascular dementia (FinnGen) | rs2280406 | 3:49941436 | A/G | 0.506 | -0.0100 (0.0013) | 3.4E-15 | 0.0763 (0.0504) | 0.130 | 62.0 | -7.660 |
| Walking pace | Vascular dementia (FinnGen) | rs55872725 | 16:53809123 | T/C | 0.404 | -0.0099 (0.0013) | 1.9E-14 | 0.0166 (0.0498) | 0.739 | 58.6 | -1.681 |
| Walking pace | Vascular dementia (FinnGen) | rs273512 | 19:18224729 | T/C | 0.405 | -0.0097 (0.0013) | 6.9E-14 | -0.0452 (0.0496) | 0.363 | 56.1 | 4.677 |
| Walking pace | Vascular dementia (FinnGen) | rs9366651 | 6:26336696 | T/G | 0.507 | 0.0095 (0.0013) | 8.6E-14 | 0.0474 (0.0520) | 0.362 | 55.7 | 4.997 |
| Walking pace | Vascular dementia (FinnGen) | rs11150623 | 16:28881001 | T/G | 0.645 | -0.0097 (0.0013) | 2.4E-13 | -0.0572 (0.0496) | 0.249 | 53.7 | 5.904 |
| Walking pace | Vascular dementia (FinnGen) | rs7795394 | 7:113560607 | A/T | 0.625 | 0.0093 (0.0013) | 1.2E-12 | -0.0415 (0.0538) | 0.441 | 50.6 | -4.460 |
| Walking pace | Vascular dementia (FinnGen) | rs12042959 | 1:243533273 | G/A | 0.144 | 0.0126 (0.0018) | 4.1E-12 | 0.0129 (0.0672) | 0.848 | 48.1 | 1.027 |
| Walking pace | Vascular dementia (FinnGen) | rs2297600 | 1:32207581 | G/T | 0.172 | -0.0116 (0.0017) | 6.2E-12 | -0.0716 (0.0597) | 0.231 | 47.3 | 6.193 |
| Walking pace | Vascular dementia (FinnGen) | rs10828258 | 10:21929734 | G/A | 0.319 | -0.0093 (0.0014) | 6.7E-12 | -0.0857 (0.0529) | 0.105 | 47.1 | 9.189 |
| Walking pace | Vascular dementia (FinnGen) | rs57800857 | 4:140863365 | C/A | 0.365 | 0.0087 (0.0013) | 5.3E-11 | -0.6580 (1.1243) | 0.558 | 43.1 | -75.841 |
| Walking pace | Vascular dementia (FinnGen) | rs2645979 | 12:84017043 | A/G | 0.357 | 0.0086 (0.0013) | 6.7E-11 | 0.0302 (0.0494) | 0.541 | 42.6 | 3.504 |
| Walking pace | Vascular dementia (FinnGen) | rs4839898 | 6:97546759 | A/G | 0.110 | 0.0131 (0.0021) | 2.3E-10 | -0.0620 (0.0788) | 0.432 | 40.2 | -4.749 |
| Walking pace | Vascular dementia (FinnGen) | rs11548200 | 1:156290656 | C/T | 0.066 | -0.0160 (0.0026) | 3.3E-10 | 0.1782 (0.1247) | 0.153 | 39.5 | -11.109 |
| Walking pace | Vascular dementia (FinnGen) | rs8010773 | 14:46956863 | C/T | 0.382 | -0.0082 (0.0013) | 3.4E-10 | 0.0582 (0.0543) | 0.284 | 39.4 | -7.126 |
| Walking pace | Vascular dementia (FinnGen) | rs6763292 | 3:129044705 | G/A | 0.781 | 0.0096 (0.0015) | 3.8E-10 | 0.0426 (0.0642) | 0.507 | 39.2 | 4.443 |
| Walking pace | Vascular dementia (FinnGen) | rs10862220 | 12:81430599 | G/T | 0.675 | 0.0084 (0.0014) | 4.8E-10 | -0.0350 (0.0494) | 0.478 | 38.8 | -4.163 |
| Walking pace | Vascular dementia (FinnGen) | rs891387 | 18:21103909 | C/T | 0.495 | 0.0079 (0.0013) | 5.4E-10 | -0.0211 (0.0494) | 0.670 | 38.5 | -2.684 |
| Walking pace | Vascular dementia (FinnGen) | rs55680124 | 2:105984624 | T/C | 0.155 | -0.0107 (0.0018) | 8.3E-10 | -0.0535 (0.0768) | 0.486 | 37.7 | 4.980 |
| Walking pace | Vascular dementia (FinnGen) | rs10750025 | 11:113424042 | T/C | 0.682 | -0.0084 (0.0014) | 9.4E-10 | 0.0029 (0.0664) | 0.965 | 37.5 | -0.347 |
| Walking pace | Vascular dementia (FinnGen) | rs12883788 | 14:33303540 | T/C | 0.460 | -0.0078 (0.0013) | 9.4E-10 | -0.0199 (0.0499) | 0.689 | 37.5 | 2.551 |
| Walking pace | Vascular dementia (FinnGen) | rs4516268 | 17:1846831 | A/C | 0.194 | 0.0098 (0.0016) | 9.5E-10 | -0.0410 (0.0562) | 0.465 | 37.4 | -4.171 |
| Walking pace | Vascular dementia (FinnGen) | rs205262 | 6:34563164 | G/A | 0.269 | -0.0087 (0.0014) | 1.0E-09 | 0.0093 (0.0558) | 0.867 | 37.3 | -1.068 |
| Walking pace | Vascular dementia (FinnGen) | rs143384 | 20:34025756 | G/A | 0.404 | 0.0079 (0.0013) | 1.1E-09 | 0.0484 (0.0498) | 0.331 | 37.1 | 6.163 |
| Walking pace | Vascular dementia (FinnGen) | rs10883618 | 10:103117653 | A/G | 0.372 | 0.0078 (0.0013) | 2.4E-09 | -0.0937 (0.0515) | 0.069 | 35.6 | -11.997 |
| Walking pace | Vascular dementia (FinnGen) | rs11682482 | 2:226486479 | G/T | 0.681 | 0.0080 (0.0014) | 3.3E-09 | 0.0394 (0.0518) | 0.447 | 35.0 | 4.913 |
| Walking pace | Vascular dementia (FinnGen) | rs12461902 | 19:30265235 | A/G | 0.330 | -0.0080 (0.0014) | 3.6E-09 | 0.0433 (0.0522) | 0.406 | 34.8 | -5.405 |
| Walking pace | Vascular dementia (FinnGen) | rs11761141 | 7:69423362 | G/T | 0.326 | -0.0079 (0.0014) | 5.0E-09 | -0.0163 (0.0523) | 0.755 | 34.2 | 2.063 |
| Walking pace | Vascular dementia (FinnGen) | rs113825410 | 1:40057543 | G/A | 0.224 | -0.0088 (0.0015) | 6.4E-09 | 0.0278 (0.0599) | 0.642 | 33.7 | -3.155 |
| Walking pace | Vascular dementia (FinnGen) | rs4109292 | 10:126710654 | A/G | 0.494 | 0.0074 (0.0013) | 6.6E-09 | -0.0717 (0.0494) | 0.147 | 33.6 | -9.751 |
| Walking pace | Vascular dementia (FinnGen) | rs2037735 | 19:48012469 | T/C | 0.121 | -0.0113 (0.0019) | 6.7E-09 | -0.1530 (0.0699) | 0.029 | 33.6 | 13.579 |
| Walking pace | Vascular dementia (FinnGen) | rs1592 | 2:135722143 | C/A | 0.395 | 0.0075 (0.0013) | 7.3E-09 | 0.0115 (0.0497) | 0.817 | 33.5 | 1.543 |
| Walking pace | Vascular dementia (FinnGen) | rs830627 | 3:71675270 | A/G | 0.417 | 0.0074 (0.0013) | 7.7E-09 | -0.0550 (0.0491) | 0.263 | 33.4 | -7.412 |
| Walking pace | Vascular dementia (FinnGen) | rs11848096 | 14:100969235 | C/T | 0.387 | -0.0075 (0.0013) | 9.2E-09 | 0.0420 (0.0502) | 0.403 | 33.0 | -5.594 |
| Walking pace | Vascular dementia (FinnGen) | rs11732213 | 4:1704244 | C/T | 0.196 | 0.0092 (0.0016) | 9.4E-09 | -0.0995 (0.0600) | 0.097 | 33.0 | -10.861 |
| Walking pace | Vascular dementia (FinnGen) | rs4715208 | 6:50829471 | G/A | 0.753 | -0.0084 (0.0015) | 1.1E-08 | 0.0570 (0.0573) | 0.320 | 32.7 | -6.798 |
| Walking pace | Vascular dementia (FinnGen) | rs2439823 | 10:99778226 | G/A | 0.546 | -0.0073 (0.0013) | 1.1E-08 | 0.0803 (0.0492) | 0.103 | 32.6 | -11.032 |
| Walking pace | Vascular dementia (FinnGen) | rs28519617 | 3:135874930 | G/T | 0.270 | -0.0081 (0.0014) | 1.4E-08 | -0.0163 (0.0599) | 0.786 | 32.2 | 2.005 |
| Walking pace | Vascular dementia (FinnGen) | rs2644135 | 1:201856256 | G/C | 0.656 | 0.0075 (0.0013) | 1.7E-08 | 0.0729 (0.0538) | 0.175 | 31.8 | 9.705 |
| Walking pace | Vascular dementia (FinnGen) | rs35711462 | 5:50847577 | G/A | 0.511 | -0.0072 (0.0013) | 1.7E-08 | -0.0488 (0.0492) | 0.322 | 31.9 | 6.810 |
| Walking pace | Vascular dementia (FinnGen) | rs2602731 | 19:4944771 | G/A | 0.681 | -0.0077 (0.0014) | 2.1E-08 | 0.0214 (0.0577) | 0.710 | 31.4 | -2.791 |
| Walking pace | Vascular dementia (FinnGen) | rs144333966 | 4:61109385 | G/A | 0.015 | 0.0305 (0.0054) | 2.2E-08 | 0.0479 (0.1432) | 0.738 | 31.3 | 1.572 |
| Walking pace | Vascular dementia (FinnGen) | rs68085814 | 17:47135875 | A/G | 0.300 | 0.0077 (0.0014) | 2.3E-08 | 0.0580 (0.0546) | 0.288 | 31.2 | 7.500 |
| Walking pace | Vascular dementia (FinnGen) | rs9783304 | 11:43660255 | T/G | 0.689 | 0.0076 (0.0014) | 2.4E-08 | 0.0088 (0.0533) | 0.869 | 31.1 | 1.152 |
| Walking pace | Vascular dementia (FinnGen) | rs8011870 | 14:80173397 | A/G | 0.288 | -0.0078 (0.0014) | 2.6E-08 | 0.0348 (0.0496) | 0.483 | 31.0 | -4.446 |
| Walking pace | Vascular dementia (FinnGen) | rs7789719 | 7:66893916 | C/T | 0.782 | 0.0085 (0.0015) | 2.7E-08 | 0.0032 (0.0571) | 0.955 | 30.9 | 0.375 |
| Walking pace | Vascular dementia (FinnGen) | rs12747822 | 1:91201451 | A/T | 0.098 | 0.0118 (0.0021) | 3.8E-08 | 0.0462 (0.1153) | 0.689 | 30.2 | 3.926 |
| Walking pace | Vascular dementia (FinnGen) | rs11152989 | 6:96936061 | T/C | 0.312 | -0.0075 (0.0014) | 3.9E-08 | -0.0244 (0.0534) | 0.648 | 30.2 | 3.248 |
| Walking pace | Vascular dementia (FinnGen) | rs45583845 | 14:57858194 | G/C | 0.034 | -0.0199 (0.0036) | 4.3E-08 | -0.0110 (0.1500) | 0.942 | 30.0 | 0.553 |
| Walking pace | Vascular dementia (FinnGen) | rs2170670 | 12:16944621 | A/G | 0.607 | -0.0071 (0.0013) | 4.7E-08 | 0.0085 (0.0514) | 0.868 | 29.9 | -1.199 |
| Walking pace | Vascular dementia (FinnGen) | rs11077815 | 17:74389890 | C/T | 0.624 | -0.0071 (0.0013) | 4.8E-08 | 0.0338 (0.0529) | 0.523 | 29.8 | -4.738 |
| Walking pace | Alzheimer's disease, wide (FinnGen) | rs13107325 | 4:103188709 | T/C | 0.075 | -0.0242 (0.0024) | 7.1E-24 | 0.1306 (0.0917) | 0.154 | 101.5 | -5.387 |
| Walking pace | Alzheimer's disease, wide (FinnGen) | rs613872 | 18:53210302 | T/G | 0.826 | -0.0147 (0.0017) | 1.3E-18 | 0.0293 (0.0290) | 0.313 | 77.5 | -1.992 |
| Walking pace | Alzheimer's disease, wide (FinnGen) | rs11039324 | 11:47665686 | A/G | 0.404 | -0.0102 (0.0013) | 2.2E-15 | -0.0254 (0.0223) | 0.254 | 62.9 | 2.488 |
| Walking pace | Alzheimer's disease, wide (FinnGen) | rs2280406 | 3:49941436 | A/G | 0.506 | -0.0100 (0.0013) | 3.4E-15 | 0.0734 (0.0217) | 7.3E-04 | 62.0 | -7.368 |
| Walking pace | Alzheimer's disease, wide (FinnGen) | rs55872725 | 16:53809123 | T/C | 0.404 | -0.0099 (0.0013) | 1.9E-14 | -0.0028 (0.0215) | 0.896 | 58.6 | 0.283 |
| Walking pace | Alzheimer's disease, wide (FinnGen) | rs273512 | 19:18224729 | T/C | 0.405 | -0.0097 (0.0013) | 6.9E-14 | 0.0002 (0.0214) | 0.991 | 56.1 | -0.021 |
| Walking pace | Alzheimer's disease, wide (FinnGen) | rs9366651 | 6:26336696 | T/G | 0.507 | 0.0095 (0.0013) | 8.6E-14 | -0.0237 (0.0224) | 0.288 | 55.7 | -2.499 |
| Walking pace | Alzheimer's disease, wide (FinnGen) | rs11150623 | 16:28881001 | T/G | 0.645 | -0.0097 (0.0013) | 2.4E-13 | -0.0417 (0.0214) | 0.051 | 53.7 | 4.304 |
| Walking pace | Alzheimer's disease, wide (FinnGen) | rs7795394 | 7:113560607 | A/T | 0.625 | 0.0093 (0.0013) | 1.2E-12 | 0.0095 (0.0233) | 0.685 | 50.6 | 1.021 |
| Walking pace | Alzheimer's disease, wide (FinnGen) | rs12042959 | 1:243533273 | G/A | 0.144 | 0.0126 (0.0018) | 4.1E-12 | 0.0131 (0.0290) | 0.650 | 48.1 | 1.042 |
| Walking pace | Alzheimer's disease, wide (FinnGen) | rs2297600 | 1:32207581 | G/T | 0.172 | -0.0116 (0.0017) | 6.2E-12 | 0.0061 (0.0258) | 0.813 | 47.3 | -0.528 |
| Walking pace | Alzheimer's disease, wide (FinnGen) | rs10828258 | 10:21929734 | G/A | 0.319 | -0.0093 (0.0014) | 6.7E-12 | 0.0146 (0.0228) | 0.520 | 47.1 | -1.565 |
| Walking pace | Alzheimer's disease, wide (FinnGen) | rs57800857 | 4:140863365 | C/A | 0.365 | 0.0087 (0.0013) | 5.3E-11 | 0.1970 (0.5031) | 0.695 | 43.1 | 22.706 |
| Walking pace | Alzheimer's disease, wide (FinnGen) | rs2645979 | 12:84017043 | A/G | 0.357 | 0.0086 (0.0013) | 6.7E-11 | -0.0077 (0.0213) | 0.719 | 42.6 | -0.893 |
| Walking pace | Alzheimer's disease, wide (FinnGen) | rs4839898 | 6:97546759 | A/G | 0.110 | 0.0131 (0.0021) | 2.3E-10 | -0.0370 (0.0342) | 0.280 | 40.2 | -2.834 |
| Walking pace | Alzheimer's disease, wide (FinnGen) | rs11548200 | 1:156290656 | C/T | 0.066 | -0.0160 (0.0026) | 3.3E-10 | -0.0083 (0.0545) | 0.879 | 39.5 | 0.517 |
| Walking pace | Alzheimer's disease, wide (FinnGen) | rs8010773 | 14:46956863 | C/T | 0.382 | -0.0082 (0.0013) | 3.4E-10 | 0.0318 (0.0234) | 0.175 | 39.4 | -3.893 |
| Walking pace | Alzheimer's disease, wide (FinnGen) | rs6763292 | 3:129044705 | G/A | 0.781 | 0.0096 (0.0015) | 3.8E-10 | 0.0335 (0.0278) | 0.228 | 39.2 | 3.494 |
| Walking pace | Alzheimer's disease, wide (FinnGen) | rs10862220 | 12:81430599 | G/T | 0.675 | 0.0084 (0.0014) | 4.8E-10 | -0.0117 (0.0213) | 0.583 | 38.8 | -1.392 |
| Walking pace | Alzheimer's disease, wide (FinnGen) | rs891387 | 18:21103909 | C/T | 0.495 | 0.0079 (0.0013) | 5.4E-10 | 0.0066 (0.0213) | 0.757 | 38.5 | 0.840 |
| Walking pace | Alzheimer's disease, wide (FinnGen) | rs55680124 | 2:105984624 | T/C | 0.155 | -0.0107 (0.0018) | 8.3E-10 | -0.0395 (0.0332) | 0.234 | 37.7 | 3.677 |
| Walking pace | Alzheimer's disease, wide (FinnGen) | rs10750025 | 11:113424042 | T/C | 0.682 | -0.0084 (0.0014) | 9.4E-10 | 0.0092 (0.0290) | 0.751 | 37.5 | -1.101 |
| Walking pace | Alzheimer's disease, wide (FinnGen) | rs12883788 | 14:33303540 | T/C | 0.460 | -0.0078 (0.0013) | 9.4E-10 | 0.0066 (0.0215) | 0.758 | 37.5 | -0.846 |
| Walking pace | Alzheimer's disease, wide (FinnGen) | rs4516268 | 17:1846831 | A/C | 0.194 | 0.0098 (0.0016) | 9.5E-10 | 0.0011 (0.0243) | 0.964 | 37.4 | 0.112 |
| Walking pace | Alzheimer's disease, wide (FinnGen) | rs205262 | 6:34563164 | G/A | 0.269 | -0.0087 (0.0014) | 1.0E-09 | -0.0104 (0.0239) | 0.665 | 37.3 | 1.194 |
| Walking pace | Alzheimer's disease, wide (FinnGen) | rs143384 | 20:34025756 | G/A | 0.404 | 0.0079 (0.0013) | 1.1E-09 | -0.0114 (0.0214) | 0.592 | 37.1 | -1.452 |
| Walking pace | Alzheimer's disease, wide (FinnGen) | rs10883618 | 10:103117653 | A/G | 0.372 | 0.0078 (0.0013) | 2.4E-09 | -0.0103 (0.0222) | 0.642 | 35.6 | -1.319 |
| Walking pace | Alzheimer's disease, wide (FinnGen) | rs11682482 | 2:226486479 | G/T | 0.681 | 0.0080 (0.0014) | 3.3E-09 | 0.0107 (0.0223) | 0.630 | 35.0 | 1.334 |
| Walking pace | Alzheimer's disease, wide (FinnGen) | rs12461902 | 19:30265235 | A/G | 0.330 | -0.0080 (0.0014) | 3.6E-09 | -0.0040 (0.0226) | 0.858 | 34.8 | 0.499 |
| Walking pace | Alzheimer's disease, wide (FinnGen) | rs11761141 | 7:69423362 | G/T | 0.326 | -0.0079 (0.0014) | 5.0E-09 | -0.0073 (0.0227) | 0.746 | 34.2 | 0.924 |
| Walking pace | Alzheimer's disease, wide (FinnGen) | rs113825410 | 1:40057543 | G/A | 0.224 | -0.0088 (0.0015) | 6.4E-09 | -0.0171 (0.0259) | 0.509 | 33.7 | 1.941 |
| Walking pace | Alzheimer's disease, wide (FinnGen) | rs4109292 | 10:126710654 | A/G | 0.494 | 0.0074 (0.0013) | 6.6E-09 | 0.0115 (0.0213) | 0.591 | 33.6 | 1.564 |
| Walking pace | Alzheimer's disease, wide (FinnGen) | rs2037735 | 19:48012469 | T/C | 0.121 | -0.0113 (0.0019) | 6.7E-09 | 0.0018 (0.0302) | 0.952 | 33.6 | -0.160 |
| Walking pace | Alzheimer's disease, wide (FinnGen) | rs1592 | 2:135722143 | C/A | 0.395 | 0.0075 (0.0013) | 7.3E-09 | 0.0095 (0.0215) | 0.659 | 33.5 | 1.275 |
| Walking pace | Alzheimer's disease, wide (FinnGen) | rs830627 | 3:71675270 | A/G | 0.417 | 0.0074 (0.0013) | 7.7E-09 | 0.0039 (0.0212) | 0.854 | 33.4 | 0.526 |
| Walking pace | Alzheimer's disease, wide (FinnGen) | rs11848096 | 14:100969235 | C/T | 0.387 | -0.0075 (0.0013) | 9.2E-09 | -0.0027 (0.0216) | 0.899 | 33.0 | 0.360 |
| Walking pace | Alzheimer's disease, wide (FinnGen) | rs11732213 | 4:1704244 | C/T | 0.196 | 0.0092 (0.0016) | 9.4E-09 | -0.0032 (0.0258) | 0.902 | 33.0 | -0.349 |
| Walking pace | Alzheimer's disease, wide (FinnGen) | rs4715208 | 6:50829471 | G/A | 0.753 | -0.0084 (0.0015) | 1.1E-08 | 0.0149 (0.0246) | 0.545 | 32.7 | -1.777 |
| Walking pace | Alzheimer's disease, wide (FinnGen) | rs2439823 | 10:99778226 | G/A | 0.546 | -0.0073 (0.0013) | 1.1E-08 | 0.0417 (0.0212) | 0.049 | 32.6 | -5.729 |
| Walking pace | Alzheimer's disease, wide (FinnGen) | rs28519617 | 3:135874930 | G/T | 0.270 | -0.0081 (0.0014) | 1.4E-08 | 0.0184 (0.0260) | 0.480 | 32.2 | -2.263 |
| Walking pace | Alzheimer's disease, wide (FinnGen) | rs2644135 | 1:201856256 | G/C | 0.656 | 0.0075 (0.0013) | 1.7E-08 | 0.0026 (0.0233) | 0.911 | 31.8 | 0.346 |
| Walking pace | Alzheimer's disease, wide (FinnGen) | rs35711462 | 5:50847577 | G/A | 0.511 | -0.0072 (0.0013) | 1.7E-08 | 0.0040 (0.0213) | 0.851 | 31.9 | -0.558 |
| Walking pace | Alzheimer's disease, wide (FinnGen) | rs2602731 | 19:4944771 | G/A | 0.681 | -0.0077 (0.0014) | 2.1E-08 | 0.0274 (0.0248) | 0.271 | 31.4 | -3.574 |
| Walking pace | Alzheimer's disease, wide (FinnGen) | rs144333966 | 4:61109385 | G/A | 0.015 | 0.0305 (0.0054) | 2.2E-08 | 0.0804 (0.0611) | 0.189 | 31.3 | 2.639 |
| Walking pace | Alzheimer's disease, wide (FinnGen) | rs68085814 | 17:47135875 | A/G | 0.300 | 0.0077 (0.0014) | 2.3E-08 | -0.0073 (0.0237) | 0.758 | 31.2 | -0.944 |
| Walking pace | Alzheimer's disease, wide (FinnGen) | rs9783304 | 11:43660255 | T/G | 0.689 | 0.0076 (0.0014) | 2.4E-08 | -0.0193 (0.0230) | 0.401 | 31.1 | -2.528 |
| Walking pace | Alzheimer's disease, wide (FinnGen) | rs8011870 | 14:80173397 | A/G | 0.288 | -0.0078 (0.0014) | 2.6E-08 | -0.0065 (0.0213) | 0.762 | 31.0 | 0.830 |
| Walking pace | Alzheimer's disease, wide (FinnGen) | rs7789719 | 7:66893916 | C/T | 0.782 | 0.0085 (0.0015) | 2.7E-08 | -0.0214 (0.0248) | 0.387 | 30.9 | -2.505 |
| Walking pace | Alzheimer's disease, wide (FinnGen) | rs12747822 | 1:91201451 | A/T | 0.098 | 0.0118 (0.0021) | 3.8E-08 | 0.0773 (0.0497) | 0.120 | 30.2 | 6.568 |
| Walking pace | Alzheimer's disease, wide (FinnGen) | rs11152989 | 6:96936061 | T/C | 0.312 | -0.0075 (0.0014) | 3.9E-08 | 0.0079 (0.0230) | 0.732 | 30.2 | -1.052 |
| Walking pace | Alzheimer's disease, wide (FinnGen) | rs45583845 | 14:57858194 | G/C | 0.034 | -0.0199 (0.0036) | 4.3E-08 | -0.0274 (0.0638) | 0.668 | 30.0 | 1.377 |
| Walking pace | Alzheimer's disease, wide (FinnGen) | rs2170670 | 12:16944621 | A/G | 0.607 | -0.0071 (0.0013) | 4.7E-08 | -0.0070 (0.0221) | 0.753 | 29.9 | 0.988 |
| Walking pace | Alzheimer's disease, wide (FinnGen) | rs11077815 | 17:74389890 | C/T | 0.624 | -0.0071 (0.0013) | 4.8E-08 | -0.0066 (0.0228) | 0.771 | 29.8 | 0.925 |

EA, effect allele; OA, other allele; EAF, effect allele frequency; beta_x (SE), SNP-exposure association; beta_y (SE), SNP-outcome association; F, SNP F statistic.

**Table S2. SNP instruments for reverse Mendelian randomization**

| **Exposure** | **Outcome** | **SNP** | **chr:pos** | **EA/OA** | **EAF** | **beta_x (SE)** | **beta_y (SE)** | **F** | **Wald ratio** |
| --- | --- | --- | --- | --- | --- | --- | --- | --- | --- |
| Alzheimer's disease (Bellenguez 2022) | Appendicular lean mass | rs117310449 | 19:45393516 | T/C | 0.017 | 0.7701 (0.0338) | 0.0228 (0.0088) | 519.1 | 0.030 |
| Alzheimer's disease (Bellenguez 2022) | Appendicular lean mass | rs6733839 | 2:127892810 | T/C | 0.389 | 0.1686 (0.0084) | 0.0025 (0.0020) | 402.9 | 0.015 |
| Alzheimer's disease (Bellenguez 2022) | Appendicular lean mass | rs61679753 | 19:45400747 | A/T | 0.030 | -0.5412 (0.0280) | 0.0054 (0.0055) | 373.6 | -0.010 |
| Alzheimer's disease (Bellenguez 2022) | Appendicular lean mass | rs148601586 | 19:45346666 | G/C | 0.015 | 0.6483 (0.0366) | 0.0082 (0.0085) | 313.8 | 0.013 |
| Alzheimer's disease (Bellenguez 2022) | Appendicular lean mass | rs365653 | 19:45361646 | G/A | 0.108 | -0.2560 (0.0146) | -0.0023 (0.0030) | 307.4 | 0.009 |
| Alzheimer's disease (Bellenguez 2022) | Appendicular lean mass | rs10792832 | 11:85867875 | G/A | 0.642 | 0.1056 (0.0084) | -0.0046 (0.0020) | 158.0 | -0.044 |
| Alzheimer's disease (Bellenguez 2022) | Appendicular lean mass | rs1532276 | 8:27466157 | C/T | 0.608 | 0.1003 (0.0083) | 0.0011 (0.0019) | 146.0 | 0.011 |
| Alzheimer's disease (Bellenguez 2022) | Appendicular lean mass | rs6656401 | 1:207692049 | G/A | 0.812 | -0.1253 (0.0104) | -0.0012 (0.0024) | 145.2 | 0.010 |
| Alzheimer's disease (Bellenguez 2022) | Appendicular lean mass | rs12151021 | 19:1050874 | G/A | 0.664 | -0.1055 (0.0093) | -0.0020 (0.0020) | 128.7 | 0.019 |
| Alzheimer's disease (Bellenguez 2022) | Appendicular lean mass | rs112481437 | 19:45549135 | A/G | 0.028 | 0.2935 (0.0278) | 0.0078 (0.0060) | 111.5 | 0.027 |
| Alzheimer's disease (Bellenguez 2022) | Appendicular lean mass | rs1582763 | 11:60021948 | A/G | 0.371 | -0.0860 (0.0084) | 0.0057 (0.0020) | 104.8 | -0.066 |
| Alzheimer's disease (Bellenguez 2022) | Appendicular lean mass | rs117618017 | 15:63569902 | T/C | 0.144 | 0.1130 (0.0119) | 0.0002 (0.0027) | 90.2 | 0.002 |
| Alzheimer's disease (Bellenguez 2022) | Appendicular lean mass | rs143332484 | 6:41129207 | T/C | 0.013 | 0.3346 (0.0376) | 0.0054 (0.0095) | 79.2 | 0.016 |
| Alzheimer's disease (Bellenguez 2022) | Appendicular lean mass | rs7384878 | 7:99932049 | T/C | 0.690 | 0.0775 (0.0089) | 0.0033 (0.0020) | 75.8 | 0.043 |
| Alzheimer's disease (Bellenguez 2022) | Appendicular lean mass | rs10933431 | 2:233981912 | C/G | 0.766 | 0.0852 (0.0099) | -0.0031 (0.0023) | 74.1 | -0.036 |
| Alzheimer's disease (Bellenguez 2022) | Appendicular lean mass | rs35472547 | 6:32560370 | T/G | 0.163 | -0.0954 (0.0113) | -0.0207 (0.0024) | 71.3 | 0.217 |
| Alzheimer's disease (Bellenguez 2022) | Appendicular lean mass | rs4292 | 17:61554341 | T/C | 0.617 | 0.0688 (0.0084) | 0.0096 (0.0020) | 67.1 | 0.140 |
| Alzheimer's disease (Bellenguez 2022) | Appendicular lean mass | rs11769559 | 7:143111296 | C/T | 0.406 | -0.0695 (0.0085) | -0.0040 (0.0019) | 66.8 | 0.058 |
| Alzheimer's disease (Bellenguez 2022) | Appendicular lean mass | rs6014724 | 20:54998544 | G/A | 0.090 | -0.1176 (0.0145) | 0.0086 (0.0033) | 65.8 | -0.073 |
| Alzheimer's disease (Bellenguez 2022) | Appendicular lean mass | rs12590654 | 14:92938855 | A/G | 0.328 | -0.0693 (0.0087) | -0.0050 (0.0020) | 63.5 | 0.072 |
| Alzheimer's disease (Bellenguez 2022) | Appendicular lean mass | rs11218343 | 11:121435587 | C/T | 0.039 | -0.1654 (0.0214) | 0.0055 (0.0050) | 59.7 | -0.033 |
| Alzheimer's disease (Bellenguez 2022) | Appendicular lean mass | rs6586028 | 10:82253984 | T/C | 0.804 | 0.0791 (0.0103) | 0.0008 (0.0023) | 59.0 | 0.010 |
| Alzheimer's disease (Bellenguez 2022) | Appendicular lean mass | rs755951 | 8:27226790 | C/A | 0.412 | 0.0618 (0.0083) | 0.0064 (0.0019) | 55.4 | 0.104 |
| Alzheimer's disease (Bellenguez 2022) | Appendicular lean mass | rs62375397 | 5:86298531 | T/C | 0.210 | 0.0746 (0.0100) | 0.0073 (0.0023) | 55.6 | 0.098 |
| Alzheimer's disease (Bellenguez 2022) | Appendicular lean mass | rs9676738 | 19:45704771 | A/G | 0.042 | 0.1689 (0.0228) | 0.0060 (0.0051) | 54.9 | 0.036 |
| Alzheimer's disease (Bellenguez 2022) | Appendicular lean mass | rs6846529 | 4:11025131 | T/C | 0.717 | -0.0673 (0.0091) | -0.0025 (0.0021) | 54.7 | 0.037 |
| Alzheimer's disease (Bellenguez 2022) | Appendicular lean mass | rs1385742 | 6:47595155 | T/A | 0.643 | -0.0624 (0.0086) | -0.0056 (0.0020) | 52.6 | 0.090 |
| Alzheimer's disease (Bellenguez 2022) | Appendicular lean mass | rs57402520 | 17:5139807 | A/G | 0.122 | 0.0905 (0.0126) | 0.0035 (0.0030) | 51.6 | 0.039 |
| Alzheimer's disease (Bellenguez 2022) | Appendicular lean mass | rs5848 | 17:42430244 | T/C | 0.289 | 0.0646 (0.0092) | 0.0086 (0.0021) | 49.3 | 0.133 |
| Alzheimer's disease (Bellenguez 2022) | Appendicular lean mass | rs12444183 | 16:81773209 | G/A | 0.613 | 0.0591 (0.0084) | 0.0002 (0.0020) | 49.5 | 0.003 |
| Alzheimer's disease (Bellenguez 2022) | Appendicular lean mass | rs7912495 | 10:11718713 | G/A | 0.462 | 0.0572 (0.0082) | 0.0018 (0.0019) | 48.7 | 0.031 |
| Alzheimer's disease (Bellenguez 2022) | Appendicular lean mass | rs34173062 | 8:145158607 | A/G | 0.081 | 0.1139 (0.0163) | 0.0047 (0.0039) | 48.8 | 0.041 |
| Alzheimer's disease (Bellenguez 2022) | Appendicular lean mass | rs11500477 | 11:47409051 | A/T | 0.375 | 0.0585 (0.0084) | -0.0047 (0.0020) | 48.5 | -0.080 |
| Alzheimer's disease (Bellenguez 2022) | Appendicular lean mass | rs113706587 | 5:179628150 | A/G | 0.110 | 0.0925 (0.0133) | -0.0064 (0.0031) | 48.4 | -0.069 |
| Alzheimer's disease (Bellenguez 2022) | Appendicular lean mass | rs1140239 | 16:30021402 | T/C | 0.379 | -0.0592 (0.0086) | 0.0273 (0.0019) | 47.4 | -0.461 |
| Alzheimer's disease (Bellenguez 2022) | Appendicular lean mass | rs593742 | 15:59045774 | G/A | 0.295 | -0.0610 (0.0090) | -0.0003 (0.0021) | 45.9 | 0.005 |
| Alzheimer's disease (Bellenguez 2022) | Appendicular lean mass | rs4714447 | 6:41239822 | T/C | 0.345 | 0.0578 (0.0086) | 0.0002 (0.0020) | 45.2 | 0.003 |
| Alzheimer's disease (Bellenguez 2022) | Appendicular lean mass | rs74685827 | 11:121353077 | G/T | 0.019 | 0.1964 (0.0303) | -0.0078 (0.0068) | 42.0 | -0.040 |
| Alzheimer's disease (Bellenguez 2022) | Appendicular lean mass | rs28577986 | 14:106200232 | A/G | 0.263 | 0.0698 (0.0111) | 0.0005 (0.0021) | 39.5 | 0.007 |
| Alzheimer's disease (Bellenguez 2022) | Appendicular lean mass | rs3822030 | 4:987343 | T/G | 0.571 | 0.0514 (0.0083) | 0.0085 (0.0019) | 38.4 | 0.165 |
| Alzheimer's disease (Bellenguez 2022) | Appendicular lean mass | rs17125924 | 14:53391680 | G/A | 0.089 | 0.0881 (0.0142) | 0.0127 (0.0033) | 38.5 | 0.144 |
| Alzheimer's disease (Bellenguez 2022) | Appendicular lean mass | rs190651665 | 19:45456103 | G/T | 0.030 | -0.1598 (0.0260) | -0.0070 (0.0053) | 37.8 | 0.044 |
| Alzheimer's disease (Bellenguez 2022) | Appendicular lean mass | rs2154481 | 21:27473875 | T/C | 0.524 | 0.0500 (0.0082) | 0.0033 (0.0019) | 37.2 | 0.066 |
| Alzheimer's disease (Bellenguez 2022) | Appendicular lean mass | rs2830489 | 21:28148191 | T/C | 0.281 | -0.0547 (0.0091) | -0.0005 (0.0021) | 36.1 | 0.009 |
| Alzheimer's disease (Bellenguez 2022) | Appendicular lean mass | rs4734295 | 8:96000919 | G/A | 0.461 | 0.0487 (0.0081) | 0.0006 (0.0019) | 36.1 | 0.012 |
| Alzheimer's disease (Bellenguez 2022) | Appendicular lean mass | rs74504435 | 7:54949256 | G/A | 0.093 | -0.0842 (0.0141) | -0.0018 (0.0033) | 35.7 | 0.021 |
| Alzheimer's disease (Bellenguez 2022) | Appendicular lean mass | rs199451 | 17:44801784 | A/G | 0.212 | -0.0594 (0.0100) | 0.0040 (0.0023) | 35.3 | -0.067 |
| Alzheimer's disease (Bellenguez 2022) | Appendicular lean mass | rs1065712 | 8:11702122 | C/G | 0.053 | 0.1081 (0.0185) | 0.0034 (0.0045) | 34.1 | 0.031 |
| Alzheimer's disease (Bellenguez 2022) | Appendicular lean mass | rs149080927 | 19:1854253 | GC/G | 0.520 | -0.0535 (0.0092) | 0.0055 (0.0019) | 33.8 | -0.103 |
| Alzheimer's disease (Bellenguez 2022) | Appendicular lean mass | rs7068231 | 10:61784928 | G/T | 0.597 | 0.0487 (0.0084) | 0.0055 (0.0020) | 33.6 | 0.113 |
| Alzheimer's disease (Bellenguez 2022) | Appendicular lean mass | rs1799777 | 9:107666035 | GC/G | 0.129 | 0.0708 (0.0123) | -0.0016 (0.0029) | 33.1 | -0.023 |
| Alzheimer's disease (Bellenguez 2022) | Appendicular lean mass | rs2070901 | 1:161185058 | T/G | 0.265 | -0.0527 (0.0092) | 0.0013 (0.0021) | 32.8 | -0.025 |
| Alzheimer's disease (Bellenguez 2022) | Appendicular lean mass | rs74745468 | 14:39887676 | A/G | 0.087 | 0.0822 (0.0145) | -0.0002 (0.0034) | 32.1 | -0.002 |
| Alzheimer's disease (Bellenguez 2022) | Appendicular lean mass | rs1693551 | 8:101675584 | C/T | 0.467 | 0.0459 (0.0081) | 0.0086 (0.0019) | 32.1 | 0.187 |
| Alzheimer's disease (Bellenguez 2022) | Appendicular lean mass | rs4485362 | 16:81975831 | G/T | 0.460 | -0.0463 (0.0083) | -0.0015 (0.0019) | 31.1 | 0.032 |
| Alzheimer's disease (Bellenguez 2022) | Appendicular lean mass | rs11495981 | 7:28177301 | T/C | 0.209 | -0.0561 (0.0100) | -0.0129 (0.0024) | 31.5 | 0.230 |
| Alzheimer's disease (Bellenguez 2022) | Appendicular lean mass | rs61762319 | 3:154801978 | G/A | 0.026 | 0.1430 (0.0255) | 0.0102 (0.0056) | 31.4 | 0.071 |
| Alzheimer's disease (Bellenguez 2022) | Appendicular lean mass | rs976271 | 6:114682727 | A/G | 0.361 | 0.0467 (0.0084) | 0.0004 (0.0020) | 30.9 | 0.009 |
| Alzheimer's disease (Bellenguez 2022) | Appendicular lean mass | rs2526377 | 17:56410041 | G/A | 0.445 | -0.0452 (0.0082) | 0.0006 (0.0019) | 30.4 | -0.013 |
| Alzheimer's disease (Bellenguez 2022) | Low hand grip strength (FNIH, 60+) | rs117310449 | 19:45393516 | T/C | 0.017 | 0.7701 (0.0338) | 0.0191 (0.0501) | 519.1 | 0.025 |
| Alzheimer's disease (Bellenguez 2022) | Low hand grip strength (FNIH, 60+) | rs6733839 | 2:127892810 | T/C | 0.389 | 0.1686 (0.0084) | 0.0009 (0.0109) | 402.9 | 0.005 |
| Alzheimer's disease (Bellenguez 2022) | Low hand grip strength (FNIH, 60+) | rs61679753 | 19:45400747 | A/T | 0.030 | -0.5412 (0.0280) | -0.0146 (0.0303) | 373.6 | 0.027 |
| Alzheimer's disease (Bellenguez 2022) | Low hand grip strength (FNIH, 60+) | rs148601586 | 19:45346666 | G/C | 0.015 | 0.6483 (0.0366) | 0.0125 (0.0499) | 313.8 | 0.019 |
| Alzheimer's disease (Bellenguez 2022) | Low hand grip strength (FNIH, 60+) | rs365653 | 19:45361646 | G/A | 0.108 | -0.2560 (0.0146) | 0.0342 (0.0164) | 307.4 | -0.134 |
| Alzheimer's disease (Bellenguez 2022) | Low hand grip strength (FNIH, 60+) | rs10792832 | 11:85867875 | G/A | 0.642 | 0.1056 (0.0084) | -0.0218 (0.0109) | 158.0 | -0.206 |
| Alzheimer's disease (Bellenguez 2022) | Low hand grip strength (FNIH, 60+) | rs1532276 | 8:27466157 | C/T | 0.608 | 0.1003 (0.0083) | -0.0009 (0.0108) | 146.0 | -0.009 |
| Alzheimer's disease (Bellenguez 2022) | Low hand grip strength (FNIH, 60+) | rs6656401 | 1:207692049 | G/A | 0.812 | -0.1253 (0.0104) | -0.0047 (0.0138) | 145.2 | 0.038 |
| Alzheimer's disease (Bellenguez 2022) | Low hand grip strength (FNIH, 60+) | rs12151021 | 19:1050874 | G/A | 0.664 | -0.1055 (0.0093) | -0.0025 (0.0115) | 128.7 | 0.024 |
| Alzheimer's disease (Bellenguez 2022) | Low hand grip strength (FNIH, 60+) | rs112481437 | 19:45549135 | A/G | 0.028 | 0.2935 (0.0278) | -0.0174 (0.0355) | 111.5 | -0.059 |
| Alzheimer's disease (Bellenguez 2022) | Low hand grip strength (FNIH, 60+) | rs1582763 | 11:60021948 | A/G | 0.371 | -0.0860 (0.0084) | -0.0034 (0.0108) | 104.8 | 0.040 |
| Alzheimer's disease (Bellenguez 2022) | Low hand grip strength (FNIH, 60+) | rs117618017 | 15:63569902 | T/C | 0.144 | 0.1130 (0.0119) | -0.0058 (0.0156) | 90.2 | -0.051 |
| Alzheimer's disease (Bellenguez 2022) | Low hand grip strength (FNIH, 60+) | rs143332484 | 6:41129207 | T/C | 0.013 | 0.3346 (0.0376) | 0.0399 (0.0558) | 79.2 | 0.119 |
| Alzheimer's disease (Bellenguez 2022) | Low hand grip strength (FNIH, 60+) | rs7384878 | 7:99932049 | T/C | 0.690 | 0.0775 (0.0089) | 0.0072 (0.0115) | 75.8 | 0.093 |
| Alzheimer's disease (Bellenguez 2022) | Low hand grip strength (FNIH, 60+) | rs10933431 | 2:233981912 | C/G | 0.766 | 0.0852 (0.0099) | -0.0045 (0.0129) | 74.1 | -0.053 |
| Alzheimer's disease (Bellenguez 2022) | Low hand grip strength (FNIH, 60+) | rs35472547 | 6:32560370 | T/G | 0.163 | -0.0954 (0.0113) | 0.1075 (0.0151) | 71.3 | -1.127 |
| Alzheimer's disease (Bellenguez 2022) | Low hand grip strength (FNIH, 60+) | rs4292 | 17:61554341 | T/C | 0.617 | 0.0688 (0.0084) | -0.0169 (0.0110) | 67.1 | -0.246 |
| Alzheimer's disease (Bellenguez 2022) | Low hand grip strength (FNIH, 60+) | rs11769559 | 7:143111296 | C/T | 0.406 | -0.0695 (0.0085) | -0.0087 (0.0132) | 66.8 | 0.125 |
| Alzheimer's disease (Bellenguez 2022) | Low hand grip strength (FNIH, 60+) | rs6014724 | 20:54998544 | G/A | 0.090 | -0.1176 (0.0145) | 0.0008 (0.0184) | 65.8 | -0.007 |
| Alzheimer's disease (Bellenguez 2022) | Low hand grip strength (FNIH, 60+) | rs12590654 | 14:92938855 | A/G | 0.328 | -0.0693 (0.0087) | 0.0236 (0.0111) | 63.5 | -0.341 |
| Alzheimer's disease (Bellenguez 2022) | Low hand grip strength (FNIH, 60+) | rs11218343 | 11:121435587 | C/T | 0.039 | -0.1654 (0.0214) | 0.0062 (0.0272) | 59.7 | -0.037 |
| Alzheimer's disease (Bellenguez 2022) | Low hand grip strength (FNIH, 60+) | rs6586028 | 10:82253984 | T/C | 0.804 | 0.0791 (0.0103) | 0.0202 (0.0132) | 59.0 | 0.255 |
| Alzheimer's disease (Bellenguez 2022) | Low hand grip strength (FNIH, 60+) | rs755951 | 8:27226790 | C/A | 0.412 | 0.0618 (0.0083) | -0.0129 (0.0107) | 55.4 | -0.209 |
| Alzheimer's disease (Bellenguez 2022) | Low hand grip strength (FNIH, 60+) | rs62375397 | 5:86298531 | T/C | 0.210 | 0.0746 (0.0100) | 0.0330 (0.0128) | 55.6 | 0.442 |
| Alzheimer's disease (Bellenguez 2022) | Low hand grip strength (FNIH, 60+) | rs9676738 | 19:45704771 | A/G | 0.042 | 0.1689 (0.0228) | 0.0017 (0.0287) | 54.9 | 0.010 |
| Alzheimer's disease (Bellenguez 2022) | Low hand grip strength (FNIH, 60+) | rs6846529 | 4:11025131 | T/C | 0.717 | -0.0673 (0.0091) | 0.0196 (0.0119) | 54.7 | -0.291 |
| Alzheimer's disease (Bellenguez 2022) | Low hand grip strength (FNIH, 60+) | rs1385742 | 6:47595155 | T/A | 0.643 | -0.0624 (0.0086) | 0.0026 (0.0112) | 52.6 | -0.042 |
| Alzheimer's disease (Bellenguez 2022) | Low hand grip strength (FNIH, 60+) | rs57402520 | 17:5139807 | A/G | 0.122 | 0.0905 (0.0126) | -0.0090 (0.0191) | 51.6 | -0.099 |
| Alzheimer's disease (Bellenguez 2022) | Low hand grip strength (FNIH, 60+) | rs5848 | 17:42430244 | T/C | 0.289 | 0.0646 (0.0092) | -0.0161 (0.0119) | 49.3 | -0.249 |
| Alzheimer's disease (Bellenguez 2022) | Low hand grip strength (FNIH, 60+) | rs12444183 | 16:81773209 | G/A | 0.613 | 0.0591 (0.0084) | -0.0018 (0.0109) | 49.5 | -0.030 |
| Alzheimer's disease (Bellenguez 2022) | Low hand grip strength (FNIH, 60+) | rs7912495 | 10:11718713 | G/A | 0.462 | 0.0572 (0.0082) | -0.0076 (0.0106) | 48.7 | -0.133 |
| Alzheimer's disease (Bellenguez 2022) | Low hand grip strength (FNIH, 60+) | rs34173062 | 8:145158607 | A/G | 0.081 | 0.1139 (0.0163) | -0.0054 (0.0222) | 48.8 | -0.047 |
| Alzheimer's disease (Bellenguez 2022) | Low hand grip strength (FNIH, 60+) | rs11500477 | 11:47409051 | A/T | 0.375 | 0.0585 (0.0084) | -0.0339 (0.0110) | 48.5 | -0.579 |
| Alzheimer's disease (Bellenguez 2022) | Low hand grip strength (FNIH, 60+) | rs113706587 | 5:179628150 | A/G | 0.110 | 0.0925 (0.0133) | 0.0142 (0.0174) | 48.4 | 0.154 |
| Alzheimer's disease (Bellenguez 2022) | Low hand grip strength (FNIH, 60+) | rs1140239 | 16:30021402 | T/C | 0.379 | -0.0592 (0.0086) | 0.0053 (0.0124) | 47.4 | -0.090 |
| Alzheimer's disease (Bellenguez 2022) | Low hand grip strength (FNIH, 60+) | rs593742 | 15:59045774 | G/A | 0.295 | -0.0610 (0.0090) | -0.0061 (0.0114) | 45.9 | 0.100 |
| Alzheimer's disease (Bellenguez 2022) | Low hand grip strength (FNIH, 60+) | rs4714447 | 6:41239822 | T/C | 0.345 | 0.0578 (0.0086) | 0.0064 (0.0111) | 45.2 | 0.111 |
| Alzheimer's disease (Bellenguez 2022) | Low hand grip strength (FNIH, 60+) | rs74685827 | 11:121353077 | G/T | 0.019 | 0.1964 (0.0303) | -0.0079 (0.0388) | 42.0 | -0.040 |
| Alzheimer's disease (Bellenguez 2022) | Low hand grip strength (FNIH, 60+) | rs28577986 | 14:106200232 | A/G | 0.263 | 0.0698 (0.0111) | -0.0224 (0.0129) | 39.5 | -0.321 |
| Alzheimer's disease (Bellenguez 2022) | Low hand grip strength (FNIH, 60+) | rs3822030 | 4:987343 | T/G | 0.571 | 0.0514 (0.0083) | -0.0118 (0.0108) | 38.4 | -0.230 |
| Alzheimer's disease (Bellenguez 2022) | Low hand grip strength (FNIH, 60+) | rs17125924 | 14:53391680 | G/A | 0.089 | 0.0881 (0.0142) | -0.0132 (0.0182) | 38.5 | -0.150 |
| Alzheimer's disease (Bellenguez 2022) | Low hand grip strength (FNIH, 60+) | rs190651665 | 19:45456103 | G/T | 0.030 | -0.1598 (0.0260) | 0.0741 (0.0292) | 37.8 | -0.464 |
| Alzheimer's disease (Bellenguez 2022) | Low hand grip strength (FNIH, 60+) | rs2154481 | 21:27473875 | T/C | 0.524 | 0.0500 (0.0082) | 0.0039 (0.0106) | 37.2 | 0.078 |
| Alzheimer's disease (Bellenguez 2022) | Low hand grip strength (FNIH, 60+) | rs2830489 | 21:28148191 | T/C | 0.281 | -0.0547 (0.0091) | 0.0024 (0.0117) | 36.1 | -0.044 |
| Alzheimer's disease (Bellenguez 2022) | Low hand grip strength (FNIH, 60+) | rs4734295 | 8:96000919 | G/A | 0.461 | 0.0487 (0.0081) | 0.0117 (0.0106) | 36.1 | 0.240 |
| Alzheimer's disease (Bellenguez 2022) | Low hand grip strength (FNIH, 60+) | rs74504435 | 7:54949256 | G/A | 0.093 | -0.0842 (0.0141) | 0.0102 (0.0181) | 35.7 | -0.121 |
| Alzheimer's disease (Bellenguez 2022) | Low hand grip strength (FNIH, 60+) | rs199451 | 17:44801784 | A/G | 0.212 | -0.0594 (0.0100) | 0.0373 (0.0127) | 35.3 | -0.628 |
| Alzheimer's disease (Bellenguez 2022) | Low hand grip strength (FNIH, 60+) | rs1065712 | 8:11702122 | C/G | 0.053 | 0.1081 (0.0185) | 0.0160 (0.0242) | 34.1 | 0.148 |
| Alzheimer's disease (Bellenguez 2022) | Low hand grip strength (FNIH, 60+) | rs149080927 | 19:1854253 | GC/G | 0.520 | -0.0535 (0.0092) | -0.0232 (0.0123) | 33.8 | 0.434 |
| Alzheimer's disease (Bellenguez 2022) | Low hand grip strength (FNIH, 60+) | rs7068231 | 10:61784928 | G/T | 0.597 | 0.0487 (0.0084) | -0.0235 (0.0108) | 33.6 | -0.483 |
| Alzheimer's disease (Bellenguez 2022) | Low hand grip strength (FNIH, 60+) | rs1799777 | 9:107666035 | GC/G | 0.129 | 0.0708 (0.0123) | 0.0180 (0.0184) | 33.1 | 0.254 |
| Alzheimer's disease (Bellenguez 2022) | Low hand grip strength (FNIH, 60+) | rs2070901 | 1:161185058 | T/G | 0.265 | -0.0527 (0.0092) | -0.0099 (0.0119) | 32.8 | 0.188 |
| Alzheimer's disease (Bellenguez 2022) | Low hand grip strength (FNIH, 60+) | rs74745468 | 14:39887676 | A/G | 0.087 | 0.0822 (0.0145) | -0.0002 (0.0191) | 32.1 | -0.002 |
| Alzheimer's disease (Bellenguez 2022) | Low hand grip strength (FNIH, 60+) | rs1693551 | 8:101675584 | C/T | 0.467 | 0.0459 (0.0081) | 0.0138 (0.0105) | 32.1 | 0.301 |
| Alzheimer's disease (Bellenguez 2022) | Low hand grip strength (FNIH, 60+) | rs4485362 | 16:81975831 | G/T | 0.460 | -0.0463 (0.0083) | 0.0061 (0.0106) | 31.1 | -0.132 |
| Alzheimer's disease (Bellenguez 2022) | Low hand grip strength (FNIH, 60+) | rs11495981 | 7:28177301 | T/C | 0.209 | -0.0561 (0.0100) | 0.0260 (0.0128) | 31.5 | -0.463 |
| Alzheimer's disease (Bellenguez 2022) | Low hand grip strength (FNIH, 60+) | rs61762319 | 3:154801978 | G/A | 0.026 | 0.1430 (0.0255) | 0.0456 (0.0324) | 31.4 | 0.319 |
| Alzheimer's disease (Bellenguez 2022) | Low hand grip strength (FNIH, 60+) | rs976271 | 6:114682727 | A/G | 0.361 | 0.0467 (0.0084) | 0.0027 (0.0109) | 30.9 | 0.058 |
| Alzheimer's disease (Bellenguez 2022) | Low hand grip strength (FNIH, 60+) | rs2526377 | 17:56410041 | G/A | 0.445 | -0.0452 (0.0082) | -0.0048 (0.0107) | 30.4 | 0.106 |
| Alzheimer's disease (Bellenguez 2022) | Hand grip strength (right) | rs117310449 | 19:45393516 | T/C | 0.017 | 0.7701 (0.0338) | 0.0010 (0.0069) | 519.1 | 0.001 |
| Alzheimer's disease (Bellenguez 2022) | Hand grip strength (right) | rs6733839 | 2:127892810 | T/C | 0.389 | 0.1686 (0.0084) | 0.0035 (0.0015) | 402.9 | 0.021 |
| Alzheimer's disease (Bellenguez 2022) | Hand grip strength (right) | rs61679753 | 19:45400747 | A/T | 0.030 | -0.5412 (0.0280) | -0.0011 (0.0043) | 373.6 | 0.002 |
| Alzheimer's disease (Bellenguez 2022) | Hand grip strength (right) | rs148601586 | 19:45346666 | G/C | 0.015 | 0.6483 (0.0366) | 0.0015 (0.0066) | 313.8 | 0.002 |
| Alzheimer's disease (Bellenguez 2022) | Hand grip strength (right) | rs365653 | 19:45361646 | G/A | 0.108 | -0.2560 (0.0146) | -0.0065 (0.0023) | 307.4 | 0.025 |
| Alzheimer's disease (Bellenguez 2022) | Hand grip strength (right) | rs10792832 | 11:85867875 | G/A | 0.642 | 0.1056 (0.0084) | -0.0035 (0.0015) | 158.0 | -0.033 |
| Alzheimer's disease (Bellenguez 2022) | Hand grip strength (right) | rs1532276 | 8:27466157 | C/T | 0.608 | 0.1003 (0.0083) | -0.0013 (0.0015) | 146.0 | -0.013 |
| Alzheimer's disease (Bellenguez 2022) | Hand grip strength (right) | rs6656401 | 1:207692049 | G/A | 0.812 | -0.1253 (0.0104) | -0.0010 (0.0019) | 145.2 | 0.008 |
| Alzheimer's disease (Bellenguez 2022) | Hand grip strength (right) | rs12151021 | 19:1050874 | G/A | 0.664 | -0.1055 (0.0093) | 0.0011 (0.0016) | 128.7 | -0.010 |
| Alzheimer's disease (Bellenguez 2022) | Hand grip strength (right) | rs112481437 | 19:45549135 | A/G | 0.028 | 0.2935 (0.0278) | 0.0009 (0.0047) | 111.5 | 0.003 |
| Alzheimer's disease (Bellenguez 2022) | Hand grip strength (right) | rs1582763 | 11:60021948 | A/G | 0.371 | -0.0860 (0.0084) | -0.0010 (0.0015) | 104.8 | 0.012 |
| Alzheimer's disease (Bellenguez 2022) | Hand grip strength (right) | rs117618017 | 15:63569902 | T/C | 0.144 | 0.1130 (0.0119) | 0.0023 (0.0022) | 90.2 | 0.021 |
| Alzheimer's disease (Bellenguez 2022) | Hand grip strength (right) | rs143332484 | 6:41129207 | T/C | 0.013 | 0.3346 (0.0376) | -0.0051 (0.0075) | 79.2 | -0.015 |
| Alzheimer's disease (Bellenguez 2022) | Hand grip strength (right) | rs7384878 | 7:99932049 | T/C | 0.690 | 0.0775 (0.0089) | -0.0050 (0.0016) | 75.8 | -0.064 |
| Alzheimer's disease (Bellenguez 2022) | Hand grip strength (right) | rs10933431 | 2:233981912 | C/G | 0.766 | 0.0852 (0.0099) | -0.0041 (0.0018) | 74.1 | -0.048 |
| Alzheimer's disease (Bellenguez 2022) | Hand grip strength (right) | rs4292 | 17:61554341 | T/C | 0.617 | 0.0688 (0.0084) | 0.0000 (0.0015) | 67.1 | 0.000 |
| Alzheimer's disease (Bellenguez 2022) | Hand grip strength (right) | rs6014724 | 20:54998544 | G/A | 0.090 | -0.1176 (0.0145) | -0.0056 (0.0026) | 65.8 | 0.048 |
| Alzheimer's disease (Bellenguez 2022) | Hand grip strength (right) | rs12590654 | 14:92938855 | A/G | 0.328 | -0.0693 (0.0087) | -0.0018 (0.0016) | 63.5 | 0.026 |
| Alzheimer's disease (Bellenguez 2022) | Hand grip strength (right) | rs11218343 | 11:121435587 | C/T | 0.039 | -0.1654 (0.0214) | 0.0006 (0.0039) | 59.7 | -0.004 |
| Alzheimer's disease (Bellenguez 2022) | Hand grip strength (right) | rs6586028 | 10:82253984 | T/C | 0.804 | 0.0791 (0.0103) | -0.0053 (0.0018) | 59.0 | -0.067 |
| Alzheimer's disease (Bellenguez 2022) | Hand grip strength (right) | rs755951 | 8:27226790 | C/A | 0.412 | 0.0618 (0.0083) | 0.0036 (0.0015) | 55.4 | 0.058 |
| Alzheimer's disease (Bellenguez 2022) | Hand grip strength (right) | rs62375397 | 5:86298531 | T/C | 0.210 | 0.0746 (0.0100) | -0.0020 (0.0018) | 55.6 | -0.027 |
| Alzheimer's disease (Bellenguez 2022) | Hand grip strength (right) | rs9676738 | 19:45704771 | A/G | 0.042 | 0.1689 (0.0228) | 0.0008 (0.0040) | 54.9 | 0.005 |
| Alzheimer's disease (Bellenguez 2022) | Hand grip strength (right) | rs6846529 | 4:11025131 | T/C | 0.717 | -0.0673 (0.0091) | -0.0040 (0.0017) | 54.7 | 0.059 |
| Alzheimer's disease (Bellenguez 2022) | Hand grip strength (right) | rs1385742 | 6:47595155 | T/A | 0.643 | -0.0624 (0.0086) | -0.0038 (0.0016) | 52.6 | 0.060 |
| Alzheimer's disease (Bellenguez 2022) | Hand grip strength (right) | rs5848 | 17:42430244 | T/C | 0.289 | 0.0646 (0.0092) | 0.0029 (0.0017) | 49.3 | 0.044 |
| Alzheimer's disease (Bellenguez 2022) | Hand grip strength (right) | rs12444183 | 16:81773209 | G/A | 0.613 | 0.0591 (0.0084) | 0.0002 (0.0015) | 49.5 | 0.003 |
| Alzheimer's disease (Bellenguez 2022) | Hand grip strength (right) | rs7912495 | 10:11718713 | G/A | 0.462 | 0.0572 (0.0082) | 0.0010 (0.0015) | 48.7 | 0.017 |
| Alzheimer's disease (Bellenguez 2022) | Hand grip strength (right) | rs34173062 | 8:145158607 | A/G | 0.081 | 0.1139 (0.0163) | 0.0063 (0.0030) | 48.8 | 0.055 |
| Alzheimer's disease (Bellenguez 2022) | Hand grip strength (right) | rs11500477 | 11:47409051 | A/T | 0.375 | 0.0585 (0.0084) | 0.0062 (0.0016) | 48.5 | 0.106 |
| Alzheimer's disease (Bellenguez 2022) | Hand grip strength (right) | rs113706587 | 5:179628150 | A/G | 0.110 | 0.0925 (0.0133) | -0.0047 (0.0025) | 48.4 | -0.051 |
| Alzheimer's disease (Bellenguez 2022) | Hand grip strength (right) | rs593742 | 15:59045774 | G/A | 0.295 | -0.0610 (0.0090) | -0.0017 (0.0016) | 45.9 | 0.028 |
| Alzheimer's disease (Bellenguez 2022) | Hand grip strength (right) | rs4714447 | 6:41239822 | T/C | 0.345 | 0.0578 (0.0086) | -0.0011 (0.0016) | 45.2 | -0.019 |
| Alzheimer's disease (Bellenguez 2022) | Hand grip strength (right) | rs74685827 | 11:121353077 | G/T | 0.019 | 0.1964 (0.0303) | 0.0036 (0.0054) | 42.0 | 0.018 |
| Alzheimer's disease (Bellenguez 2022) | Hand grip strength (right) | rs28577986 | 14:106200232 | A/G | 0.263 | 0.0698 (0.0111) | 0.0011 (0.0017) | 39.5 | 0.015 |
| Alzheimer's disease (Bellenguez 2022) | Hand grip strength (right) | rs3822030 | 4:987343 | T/G | 0.571 | 0.0514 (0.0083) | -0.0027 (0.0015) | 38.4 | -0.052 |
| Alzheimer's disease (Bellenguez 2022) | Hand grip strength (right) | rs17125924 | 14:53391680 | G/A | 0.089 | 0.0881 (0.0142) | 0.0072 (0.0026) | 38.5 | 0.082 |
| Alzheimer's disease (Bellenguez 2022) | Hand grip strength (right) | rs190651665 | 19:45456103 | G/T | 0.030 | -0.1598 (0.0260) | -0.0085 (0.0042) | 37.8 | 0.053 |
| Alzheimer's disease (Bellenguez 2022) | Hand grip strength (right) | rs2154481 | 21:27473875 | T/C | 0.524 | 0.0500 (0.0082) | -0.0003 (0.0015) | 37.2 | -0.007 |
| Alzheimer's disease (Bellenguez 2022) | Hand grip strength (right) | rs2830489 | 21:28148191 | T/C | 0.281 | -0.0547 (0.0091) | 0.0024 (0.0017) | 36.1 | -0.044 |
| Alzheimer's disease (Bellenguez 2022) | Hand grip strength (right) | rs4734295 | 8:96000919 | G/A | 0.461 | 0.0487 (0.0081) | -0.0023 (0.0015) | 36.1 | -0.046 |
| Alzheimer's disease (Bellenguez 2022) | Hand grip strength (right) | rs74504435 | 7:54949256 | G/A | 0.093 | -0.0842 (0.0141) | -0.0013 (0.0026) | 35.7 | 0.015 |
| Alzheimer's disease (Bellenguez 2022) | Hand grip strength (right) | rs199451 | 17:44801784 | A/G | 0.212 | -0.0594 (0.0100) | -0.0132 (0.0018) | 35.3 | 0.223 |
| Alzheimer's disease (Bellenguez 2022) | Hand grip strength (right) | rs1065712 | 8:11702122 | C/G | 0.053 | 0.1081 (0.0185) | 0.0014 (0.0035) | 34.1 | 0.013 |
| Alzheimer's disease (Bellenguez 2022) | Hand grip strength (right) | rs7068231 | 10:61784928 | G/T | 0.597 | 0.0487 (0.0084) | 0.0020 (0.0015) | 33.6 | 0.041 |
| Alzheimer's disease (Bellenguez 2022) | Hand grip strength (right) | rs2070901 | 1:161185058 | T/G | 0.265 | -0.0527 (0.0092) | 0.0001 (0.0017) | 32.8 | -0.002 |
| Alzheimer's disease (Bellenguez 2022) | Hand grip strength (right) | rs74745468 | 14:39887676 | A/G | 0.087 | 0.0822 (0.0145) | -0.0039 (0.0027) | 32.1 | -0.047 |
| Alzheimer's disease (Bellenguez 2022) | Hand grip strength (right) | rs1693551 | 8:101675584 | C/T | 0.467 | 0.0459 (0.0081) | 0.0022 (0.0015) | 32.1 | 0.048 |
| Alzheimer's disease (Bellenguez 2022) | Hand grip strength (right) | rs4485362 | 16:81975831 | G/T | 0.460 | -0.0463 (0.0083) | 0.0017 (0.0015) | 31.1 | -0.036 |
| Alzheimer's disease (Bellenguez 2022) | Hand grip strength (right) | rs11495981 | 7:28177301 | T/C | 0.209 | -0.0561 (0.0100) | -0.0003 (0.0019) | 31.5 | 0.006 |
| Alzheimer's disease (Bellenguez 2022) | Hand grip strength (right) | rs61762319 | 3:154801978 | G/A | 0.026 | 0.1430 (0.0255) | 0.0038 (0.0045) | 31.4 | 0.026 |
| Alzheimer's disease (Bellenguez 2022) | Hand grip strength (right) | rs976271 | 6:114682727 | A/G | 0.361 | 0.0467 (0.0084) | -0.0009 (0.0015) | 30.9 | -0.020 |
| Alzheimer's disease (Bellenguez 2022) | Hand grip strength (right) | rs2526377 | 17:56410041 | G/A | 0.445 | -0.0452 (0.0082) | 0.0009 (0.0015) | 30.4 | -0.020 |
| Alzheimer's disease (Bellenguez 2022) | Whole body fat-free mass | rs117310449 | 19:45393516 | T/C | 0.017 | 0.7701 (0.0338) | -0.0085 (0.0058) | 519.1 | -0.011 |
| Alzheimer's disease (Bellenguez 2022) | Whole body fat-free mass | rs6733839 | 2:127892810 | T/C | 0.389 | 0.1686 (0.0084) | 0.0013 (0.0013) | 402.9 | 0.008 |
| Alzheimer's disease (Bellenguez 2022) | Whole body fat-free mass | rs61679753 | 19:45400747 | A/T | 0.030 | -0.5412 (0.0280) | 0.0194 (0.0036) | 373.6 | -0.036 |
| Alzheimer's disease (Bellenguez 2022) | Whole body fat-free mass | rs148601586 | 19:45346666 | G/C | 0.015 | 0.6483 (0.0366) | -0.0059 (0.0056) | 313.8 | -0.009 |
| Alzheimer's disease (Bellenguez 2022) | Whole body fat-free mass | rs365653 | 19:45361646 | G/A | 0.108 | -0.2560 (0.0146) | 0.0015 (0.0020) | 307.4 | -0.006 |
| Alzheimer's disease (Bellenguez 2022) | Whole body fat-free mass | rs10792832 | 11:85867875 | G/A | 0.642 | 0.1056 (0.0084) | -0.0027 (0.0013) | 158.0 | -0.026 |
| Alzheimer's disease (Bellenguez 2022) | Whole body fat-free mass | rs1532276 | 8:27466157 | C/T | 0.608 | 0.1003 (0.0083) | -0.0000 (0.0013) | 146.0 | -0.000 |
| Alzheimer's disease (Bellenguez 2022) | Whole body fat-free mass | rs6656401 | 1:207692049 | G/A | 0.812 | -0.1253 (0.0104) | 0.0002 (0.0016) | 145.2 | -0.001 |
| Alzheimer's disease (Bellenguez 2022) | Whole body fat-free mass | rs12151021 | 19:1050874 | G/A | 0.664 | -0.1055 (0.0093) | -0.0002 (0.0013) | 128.7 | 0.002 |
| Alzheimer's disease (Bellenguez 2022) | Whole body fat-free mass | rs112481437 | 19:45549135 | A/G | 0.028 | 0.2935 (0.0278) | -0.0010 (0.0039) | 111.5 | -0.003 |
| Alzheimer's disease (Bellenguez 2022) | Whole body fat-free mass | rs1582763 | 11:60021948 | A/G | 0.371 | -0.0860 (0.0084) | 0.0007 (0.0013) | 104.8 | -0.008 |
| Alzheimer's disease (Bellenguez 2022) | Whole body fat-free mass | rs117618017 | 15:63569902 | T/C | 0.144 | 0.1130 (0.0119) | -0.0020 (0.0018) | 90.2 | -0.018 |
| Alzheimer's disease (Bellenguez 2022) | Whole body fat-free mass | rs143332484 | 6:41129207 | T/C | 0.013 | 0.3346 (0.0376) | -0.0067 (0.0062) | 79.2 | -0.020 |
| Alzheimer's disease (Bellenguez 2022) | Whole body fat-free mass | rs7384878 | 7:99932049 | T/C | 0.690 | 0.0775 (0.0089) | 0.0010 (0.0013) | 75.8 | 0.012 |
| Alzheimer's disease (Bellenguez 2022) | Whole body fat-free mass | rs10933431 | 2:233981912 | C/G | 0.766 | 0.0852 (0.0099) | -0.0028 (0.0015) | 74.1 | -0.032 |
| Alzheimer's disease (Bellenguez 2022) | Whole body fat-free mass | rs4292 | 17:61554341 | T/C | 0.617 | 0.0688 (0.0084) | 0.0014 (0.0013) | 67.1 | 0.021 |
| Alzheimer's disease (Bellenguez 2022) | Whole body fat-free mass | rs6014724 | 20:54998544 | G/A | 0.090 | -0.1176 (0.0145) | 0.0034 (0.0022) | 65.8 | -0.029 |
| Alzheimer's disease (Bellenguez 2022) | Whole body fat-free mass | rs12590654 | 14:92938855 | A/G | 0.328 | -0.0693 (0.0087) | -0.0032 (0.0013) | 63.5 | 0.047 |
| Alzheimer's disease (Bellenguez 2022) | Whole body fat-free mass | rs11218343 | 11:121435587 | C/T | 0.039 | -0.1654 (0.0214) | 0.0042 (0.0033) | 59.7 | -0.025 |
| Alzheimer's disease (Bellenguez 2022) | Whole body fat-free mass | rs6586028 | 10:82253984 | T/C | 0.804 | 0.0791 (0.0103) | -0.0030 (0.0015) | 59.0 | -0.037 |
| Alzheimer's disease (Bellenguez 2022) | Whole body fat-free mass | rs755951 | 8:27226790 | C/A | 0.412 | 0.0618 (0.0083) | -0.0007 (0.0013) | 55.4 | -0.011 |
| Alzheimer's disease (Bellenguez 2022) | Whole body fat-free mass | rs62375397 | 5:86298531 | T/C | 0.210 | 0.0746 (0.0100) | 0.0069 (0.0015) | 55.6 | 0.092 |
| Alzheimer's disease (Bellenguez 2022) | Whole body fat-free mass | rs9676738 | 19:45704771 | A/G | 0.042 | 0.1689 (0.0228) | 0.0070 (0.0033) | 54.9 | 0.041 |
| Alzheimer's disease (Bellenguez 2022) | Whole body fat-free mass | rs6846529 | 4:11025131 | T/C | 0.717 | -0.0673 (0.0091) | -0.0002 (0.0014) | 54.7 | 0.003 |
| Alzheimer's disease (Bellenguez 2022) | Whole body fat-free mass | rs1385742 | 6:47595155 | T/A | 0.643 | -0.0624 (0.0086) | -0.0025 (0.0013) | 52.6 | 0.040 |
| Alzheimer's disease (Bellenguez 2022) | Whole body fat-free mass | rs5848 | 17:42430244 | T/C | 0.289 | 0.0646 (0.0092) | 0.0023 (0.0014) | 49.3 | 0.036 |
| Alzheimer's disease (Bellenguez 2022) | Whole body fat-free mass | rs12444183 | 16:81773209 | G/A | 0.613 | 0.0591 (0.0084) | -0.0028 (0.0013) | 49.5 | -0.048 |
| Alzheimer's disease (Bellenguez 2022) | Whole body fat-free mass | rs7912495 | 10:11718713 | G/A | 0.462 | 0.0572 (0.0082) | 0.0001 (0.0013) | 48.7 | 0.001 |
| Alzheimer's disease (Bellenguez 2022) | Whole body fat-free mass | rs34173062 | 8:145158607 | A/G | 0.081 | 0.1139 (0.0163) | -0.0049 (0.0025) | 48.8 | -0.043 |
| Alzheimer's disease (Bellenguez 2022) | Whole body fat-free mass | rs11500477 | 11:47409051 | A/T | 0.375 | 0.0585 (0.0084) | -0.0055 (0.0013) | 48.5 | -0.094 |
| Alzheimer's disease (Bellenguez 2022) | Whole body fat-free mass | rs113706587 | 5:179628150 | A/G | 0.110 | 0.0925 (0.0133) | -0.0022 (0.0021) | 48.4 | -0.024 |
| Alzheimer's disease (Bellenguez 2022) | Whole body fat-free mass | rs593742 | 15:59045774 | G/A | 0.295 | -0.0610 (0.0090) | 0.0008 (0.0014) | 45.9 | -0.013 |
| Alzheimer's disease (Bellenguez 2022) | Whole body fat-free mass | rs4714447 | 6:41239822 | T/C | 0.345 | 0.0578 (0.0086) | -0.0008 (0.0013) | 45.2 | -0.015 |
| Alzheimer's disease (Bellenguez 2022) | Whole body fat-free mass | rs74685827 | 11:121353077 | G/T | 0.019 | 0.1964 (0.0303) | -0.0007 (0.0045) | 42.0 | -0.004 |
| Alzheimer's disease (Bellenguez 2022) | Whole body fat-free mass | rs28577986 | 14:106200232 | A/G | 0.263 | 0.0698 (0.0111) | -0.0011 (0.0014) | 39.5 | -0.015 |
| Alzheimer's disease (Bellenguez 2022) | Whole body fat-free mass | rs3822030 | 4:987343 | T/G | 0.571 | 0.0514 (0.0083) | 0.0000 (0.0013) | 38.4 | 0.001 |
| Alzheimer's disease (Bellenguez 2022) | Whole body fat-free mass | rs17125924 | 14:53391680 | G/A | 0.089 | 0.0881 (0.0142) | 0.0115 (0.0022) | 38.5 | 0.131 |
| Alzheimer's disease (Bellenguez 2022) | Whole body fat-free mass | rs190651665 | 19:45456103 | G/T | 0.030 | -0.1598 (0.0260) | -0.0076 (0.0035) | 37.8 | 0.047 |
| Alzheimer's disease (Bellenguez 2022) | Whole body fat-free mass | rs2154481 | 21:27473875 | T/C | 0.524 | 0.0500 (0.0082) | 0.0015 (0.0013) | 37.2 | 0.031 |
| Alzheimer's disease (Bellenguez 2022) | Whole body fat-free mass | rs2830489 | 21:28148191 | T/C | 0.281 | -0.0547 (0.0091) | 0.0009 (0.0014) | 36.1 | -0.017 |
| Alzheimer's disease (Bellenguez 2022) | Whole body fat-free mass | rs4734295 | 8:96000919 | G/A | 0.461 | 0.0487 (0.0081) | -0.0023 (0.0013) | 36.1 | -0.048 |
| Alzheimer's disease (Bellenguez 2022) | Whole body fat-free mass | rs74504435 | 7:54949256 | G/A | 0.093 | -0.0842 (0.0141) | 0.0007 (0.0021) | 35.7 | -0.008 |
| Alzheimer's disease (Bellenguez 2022) | Whole body fat-free mass | rs199451 | 17:44801784 | A/G | 0.212 | -0.0594 (0.0100) | 0.0013 (0.0015) | 35.3 | -0.023 |
| Alzheimer's disease (Bellenguez 2022) | Whole body fat-free mass | rs1065712 | 8:11702122 | C/G | 0.053 | 0.1081 (0.0185) | -0.0005 (0.0029) | 34.1 | -0.004 |
| Alzheimer's disease (Bellenguez 2022) | Whole body fat-free mass | rs7068231 | 10:61784928 | G/T | 0.597 | 0.0487 (0.0084) | 0.0032 (0.0013) | 33.6 | 0.066 |
| Alzheimer's disease (Bellenguez 2022) | Whole body fat-free mass | rs2070901 | 1:161185058 | T/G | 0.265 | -0.0527 (0.0092) | -0.0028 (0.0014) | 32.8 | 0.054 |
| Alzheimer's disease (Bellenguez 2022) | Whole body fat-free mass | rs74745468 | 14:39887676 | A/G | 0.087 | 0.0822 (0.0145) | -0.0040 (0.0023) | 32.1 | -0.049 |
| Alzheimer's disease (Bellenguez 2022) | Whole body fat-free mass | rs1693551 | 8:101675584 | C/T | 0.467 | 0.0459 (0.0081) | 0.0018 (0.0012) | 32.1 | 0.039 |
| Alzheimer's disease (Bellenguez 2022) | Whole body fat-free mass | rs4485362 | 16:81975831 | G/T | 0.460 | -0.0463 (0.0083) | 0.0031 (0.0013) | 31.1 | -0.066 |
| Alzheimer's disease (Bellenguez 2022) | Whole body fat-free mass | rs11495981 | 7:28177301 | T/C | 0.209 | -0.0561 (0.0100) | -0.0067 (0.0015) | 31.5 | 0.120 |
| Alzheimer's disease (Bellenguez 2022) | Whole body fat-free mass | rs61762319 | 3:154801978 | G/A | 0.026 | 0.1430 (0.0255) | 0.0064 (0.0037) | 31.4 | 0.045 |
| Alzheimer's disease (Bellenguez 2022) | Whole body fat-free mass | rs976271 | 6:114682727 | A/G | 0.361 | 0.0467 (0.0084) | 0.0036 (0.0013) | 30.9 | 0.076 |
| Alzheimer's disease (Bellenguez 2022) | Whole body fat-free mass | rs2526377 | 17:56410041 | G/A | 0.445 | -0.0452 (0.0082) | 0.0011 (0.0013) | 30.4 | -0.024 |
| Alzheimer's disease (Bellenguez 2022) | Walking pace | rs117310449 | 19:45393516 | T/C | 0.017 | 0.7701 (0.0338) | 0.0158 (0.0059) | 519.1 | 0.021 |
| Alzheimer's disease (Bellenguez 2022) | Walking pace | rs6733839 | 2:127892810 | T/C | 0.389 | 0.1686 (0.0084) | 0.0013 (0.0013) | 402.9 | 0.008 |
| Alzheimer's disease (Bellenguez 2022) | Walking pace | rs61679753 | 19:45400747 | A/T | 0.030 | -0.5412 (0.0280) | -0.0058 (0.0037) | 373.6 | 0.011 |
| Alzheimer's disease (Bellenguez 2022) | Walking pace | rs148601586 | 19:45346666 | G/C | 0.015 | 0.6483 (0.0366) | 0.0020 (0.0057) | 313.8 | 0.003 |
| Alzheimer's disease (Bellenguez 2022) | Walking pace | rs365653 | 19:45361646 | G/A | 0.108 | -0.2560 (0.0146) | -0.0043 (0.0020) | 307.4 | 0.017 |
| Alzheimer's disease (Bellenguez 2022) | Walking pace | rs10792832 | 11:85867875 | G/A | 0.642 | 0.1056 (0.0084) | 0.0002 (0.0013) | 158.0 | 0.002 |
| Alzheimer's disease (Bellenguez 2022) | Walking pace | rs1532276 | 8:27466157 | C/T | 0.608 | 0.1003 (0.0083) | -0.0010 (0.0013) | 146.0 | -0.010 |
| Alzheimer's disease (Bellenguez 2022) | Walking pace | rs6656401 | 1:207692049 | G/A | 0.812 | -0.1253 (0.0104) | -0.0007 (0.0017) | 145.2 | 0.005 |
| Alzheimer's disease (Bellenguez 2022) | Walking pace | rs12151021 | 19:1050874 | G/A | 0.664 | -0.1055 (0.0093) | -0.0006 (0.0014) | 128.7 | 0.006 |
| Alzheimer's disease (Bellenguez 2022) | Walking pace | rs112481437 | 19:45549135 | A/G | 0.028 | 0.2935 (0.0278) | 0.0051 (0.0040) | 111.5 | 0.017 |
| Alzheimer's disease (Bellenguez 2022) | Walking pace | rs1582763 | 11:60021948 | A/G | 0.371 | -0.0860 (0.0084) | 0.0007 (0.0013) | 104.8 | -0.008 |
| Alzheimer's disease (Bellenguez 2022) | Walking pace | rs117618017 | 15:63569902 | T/C | 0.144 | 0.1130 (0.0119) | 0.0038 (0.0018) | 90.2 | 0.034 |
| Alzheimer's disease (Bellenguez 2022) | Walking pace | rs143332484 | 6:41129207 | T/C | 0.013 | 0.3346 (0.0376) | 0.0128 (0.0064) | 79.2 | 0.038 |
| Alzheimer's disease (Bellenguez 2022) | Walking pace | rs7384878 | 7:99932049 | T/C | 0.690 | 0.0775 (0.0089) | -0.0002 (0.0014) | 75.8 | -0.003 |
| Alzheimer's disease (Bellenguez 2022) | Walking pace | rs10933431 | 2:233981912 | C/G | 0.766 | 0.0852 (0.0099) | 0.0004 (0.0015) | 74.1 | 0.004 |
| Alzheimer's disease (Bellenguez 2022) | Walking pace | rs4292 | 17:61554341 | T/C | 0.617 | 0.0688 (0.0084) | 0.0038 (0.0013) | 67.1 | 0.056 |
| Alzheimer's disease (Bellenguez 2022) | Walking pace | rs6014724 | 20:54998544 | G/A | 0.090 | -0.1176 (0.0145) | -0.0031 (0.0022) | 65.8 | 0.027 |
| Alzheimer's disease (Bellenguez 2022) | Walking pace | rs12590654 | 14:92938855 | A/G | 0.328 | -0.0693 (0.0087) | -0.0013 (0.0013) | 63.5 | 0.018 |
| Alzheimer's disease (Bellenguez 2022) | Walking pace | rs11218343 | 11:121435587 | C/T | 0.039 | -0.1654 (0.0214) | 0.0003 (0.0033) | 59.7 | -0.002 |
| Alzheimer's disease (Bellenguez 2022) | Walking pace | rs6586028 | 10:82253984 | T/C | 0.804 | 0.0791 (0.0103) | 0.0018 (0.0016) | 59.0 | 0.023 |
| Alzheimer's disease (Bellenguez 2022) | Walking pace | rs755951 | 8:27226790 | C/A | 0.412 | 0.0618 (0.0083) | 0.0060 (0.0013) | 55.4 | 0.098 |
| Alzheimer's disease (Bellenguez 2022) | Walking pace | rs62375397 | 5:86298531 | T/C | 0.210 | 0.0746 (0.0100) | -0.0018 (0.0015) | 55.6 | -0.025 |
| Alzheimer's disease (Bellenguez 2022) | Walking pace | rs9676738 | 19:45704771 | A/G | 0.042 | 0.1689 (0.0228) | -0.0005 (0.0034) | 54.9 | -0.003 |
| Alzheimer's disease (Bellenguez 2022) | Walking pace | rs6846529 | 4:11025131 | T/C | 0.717 | -0.0673 (0.0091) | -0.0013 (0.0014) | 54.7 | 0.019 |
| Alzheimer's disease (Bellenguez 2022) | Walking pace | rs1385742 | 6:47595155 | T/A | 0.643 | -0.0624 (0.0086) | -0.0017 (0.0013) | 52.6 | 0.027 |
| Alzheimer's disease (Bellenguez 2022) | Walking pace | rs5848 | 17:42430244 | T/C | 0.289 | 0.0646 (0.0092) | 0.0004 (0.0014) | 49.3 | 0.007 |
| Alzheimer's disease (Bellenguez 2022) | Walking pace | rs12444183 | 16:81773209 | G/A | 0.613 | 0.0591 (0.0084) | 0.0029 (0.0013) | 49.5 | 0.049 |
| Alzheimer's disease (Bellenguez 2022) | Walking pace | rs7912495 | 10:11718713 | G/A | 0.462 | 0.0572 (0.0082) | 0.0038 (0.0013) | 48.7 | 0.066 |
| Alzheimer's disease (Bellenguez 2022) | Walking pace | rs34173062 | 8:145158607 | A/G | 0.081 | 0.1139 (0.0163) | 0.0013 (0.0026) | 48.8 | 0.011 |
| Alzheimer's disease (Bellenguez 2022) | Walking pace | rs11500477 | 11:47409051 | A/T | 0.375 | 0.0585 (0.0084) | 0.0067 (0.0013) | 48.5 | 0.114 |
| Alzheimer's disease (Bellenguez 2022) | Walking pace | rs113706587 | 5:179628150 | A/G | 0.110 | 0.0925 (0.0133) | 0.0002 (0.0021) | 48.4 | 0.002 |
| Alzheimer's disease (Bellenguez 2022) | Walking pace | rs593742 | 15:59045774 | G/A | 0.295 | -0.0610 (0.0090) | -0.0031 (0.0014) | 45.9 | 0.051 |
| Alzheimer's disease (Bellenguez 2022) | Walking pace | rs4714447 | 6:41239822 | T/C | 0.345 | 0.0578 (0.0086) | 0.0045 (0.0013) | 45.2 | 0.078 |
| Alzheimer's disease (Bellenguez 2022) | Walking pace | rs74685827 | 11:121353077 | G/T | 0.019 | 0.1964 (0.0303) | 0.0016 (0.0046) | 42.0 | 0.008 |
| Alzheimer's disease (Bellenguez 2022) | Walking pace | rs28577986 | 14:106200232 | A/G | 0.263 | 0.0698 (0.0111) | 0.0011 (0.0014) | 39.5 | 0.015 |
| Alzheimer's disease (Bellenguez 2022) | Walking pace | rs3822030 | 4:987343 | T/G | 0.571 | 0.0514 (0.0083) | -0.0033 (0.0013) | 38.4 | -0.063 |
| Alzheimer's disease (Bellenguez 2022) | Walking pace | rs17125924 | 14:53391680 | G/A | 0.089 | 0.0881 (0.0142) | 0.0016 (0.0022) | 38.5 | 0.018 |
| Alzheimer's disease (Bellenguez 2022) | Walking pace | rs190651665 | 19:45456103 | G/T | 0.030 | -0.1598 (0.0260) | -0.0023 (0.0036) | 37.8 | 0.015 |
| Alzheimer's disease (Bellenguez 2022) | Walking pace | rs2154481 | 21:27473875 | T/C | 0.524 | 0.0500 (0.0082) | 0.0016 (0.0013) | 37.2 | 0.033 |
| Alzheimer's disease (Bellenguez 2022) | Walking pace | rs2830489 | 21:28148191 | T/C | 0.281 | -0.0547 (0.0091) | -0.0019 (0.0014) | 36.1 | 0.034 |
| Alzheimer's disease (Bellenguez 2022) | Walking pace | rs4734295 | 8:96000919 | G/A | 0.461 | 0.0487 (0.0081) | 0.0021 (0.0013) | 36.1 | 0.044 |
| Alzheimer's disease (Bellenguez 2022) | Walking pace | rs74504435 | 7:54949256 | G/A | 0.093 | -0.0842 (0.0141) | 0.0034 (0.0022) | 35.7 | -0.041 |
| Alzheimer's disease (Bellenguez 2022) | Walking pace | rs199451 | 17:44801784 | A/G | 0.212 | -0.0594 (0.0100) | 0.0047 (0.0015) | 35.3 | -0.079 |
| Alzheimer's disease (Bellenguez 2022) | Walking pace | rs1065712 | 8:11702122 | C/G | 0.053 | 0.1081 (0.0185) | -0.0002 (0.0030) | 34.1 | -0.002 |
| Alzheimer's disease (Bellenguez 2022) | Walking pace | rs7068231 | 10:61784928 | G/T | 0.597 | 0.0487 (0.0084) | 0.0006 (0.0013) | 33.6 | 0.013 |
| Alzheimer's disease (Bellenguez 2022) | Walking pace | rs2070901 | 1:161185058 | T/G | 0.265 | -0.0527 (0.0092) | 0.0016 (0.0014) | 32.8 | -0.030 |
| Alzheimer's disease (Bellenguez 2022) | Walking pace | rs74745468 | 14:39887676 | A/G | 0.087 | 0.0822 (0.0145) | -0.0017 (0.0023) | 32.1 | -0.021 |
| Alzheimer's disease (Bellenguez 2022) | Walking pace | rs1693551 | 8:101675584 | C/T | 0.467 | 0.0459 (0.0081) | -0.0024 (0.0013) | 32.1 | -0.053 |
| Alzheimer's disease (Bellenguez 2022) | Walking pace | rs4485362 | 16:81975831 | G/T | 0.460 | -0.0463 (0.0083) | -0.0003 (0.0013) | 31.1 | 0.007 |
| Alzheimer's disease (Bellenguez 2022) | Walking pace | rs11495981 | 7:28177301 | T/C | 0.209 | -0.0561 (0.0100) | 0.0019 (0.0016) | 31.5 | -0.033 |
| Alzheimer's disease (Bellenguez 2022) | Walking pace | rs61762319 | 3:154801978 | G/A | 0.026 | 0.1430 (0.0255) | -0.0042 (0.0038) | 31.4 | -0.029 |
| Alzheimer's disease (Bellenguez 2022) | Walking pace | rs976271 | 6:114682727 | A/G | 0.361 | 0.0467 (0.0084) | -0.0004 (0.0013) | 30.9 | -0.009 |
| Alzheimer's disease (Bellenguez 2022) | Walking pace | rs2526377 | 17:56410041 | G/A | 0.445 | -0.0452 (0.0082) | -0.0021 (0.0013) | 30.4 | 0.047 |
| Alzheimer's disease (Bellenguez 2022) | Hand grip strength (left) | rs117310449 | 19:45393516 | T/C | 0.017 | 0.7701 (0.0338) | -0.0011 (0.0069) | 519.1 | -0.001 |
| Alzheimer's disease (Bellenguez 2022) | Hand grip strength (left) | rs6733839 | 2:127892810 | T/C | 0.389 | 0.1686 (0.0084) | 0.0035 (0.0015) | 402.9 | 0.021 |
| Alzheimer's disease (Bellenguez 2022) | Hand grip strength (left) | rs61679753 | 19:45400747 | A/T | 0.030 | -0.5412 (0.0280) | -0.0045 (0.0043) | 373.6 | 0.008 |
| Alzheimer's disease (Bellenguez 2022) | Hand grip strength (left) | rs148601586 | 19:45346666 | G/C | 0.015 | 0.6483 (0.0366) | 0.0034 (0.0066) | 313.8 | 0.005 |
| Alzheimer's disease (Bellenguez 2022) | Hand grip strength (left) | rs365653 | 19:45361646 | G/A | 0.108 | -0.2560 (0.0146) | -0.0037 (0.0023) | 307.4 | 0.014 |
| Alzheimer's disease (Bellenguez 2022) | Hand grip strength (left) | rs10792832 | 11:85867875 | G/A | 0.642 | 0.1056 (0.0084) | -0.0033 (0.0015) | 158.0 | -0.031 |
| Alzheimer's disease (Bellenguez 2022) | Hand grip strength (left) | rs1532276 | 8:27466157 | C/T | 0.608 | 0.1003 (0.0083) | -0.0009 (0.0015) | 146.0 | -0.009 |
| Alzheimer's disease (Bellenguez 2022) | Hand grip strength (left) | rs6656401 | 1:207692049 | G/A | 0.812 | -0.1253 (0.0104) | -0.0016 (0.0019) | 145.2 | 0.013 |
| Alzheimer's disease (Bellenguez 2022) | Hand grip strength (left) | rs12151021 | 19:1050874 | G/A | 0.664 | -0.1055 (0.0093) | 0.0025 (0.0016) | 128.7 | -0.023 |
| Alzheimer's disease (Bellenguez 2022) | Hand grip strength (left) | rs112481437 | 19:45549135 | A/G | 0.028 | 0.2935 (0.0278) | -0.0003 (0.0047) | 111.5 | -0.001 |
| Alzheimer's disease (Bellenguez 2022) | Hand grip strength (left) | rs1582763 | 11:60021948 | A/G | 0.371 | -0.0860 (0.0084) | -0.0009 (0.0015) | 104.8 | 0.010 |
| Alzheimer's disease (Bellenguez 2022) | Hand grip strength (left) | rs117618017 | 15:63569902 | T/C | 0.144 | 0.1130 (0.0119) | 0.0019 (0.0022) | 90.2 | 0.017 |
| Alzheimer's disease (Bellenguez 2022) | Hand grip strength (left) | rs143332484 | 6:41129207 | T/C | 0.013 | 0.3346 (0.0376) | -0.0053 (0.0075) | 79.2 | -0.016 |
| Alzheimer's disease (Bellenguez 2022) | Hand grip strength (left) | rs7384878 | 7:99932049 | T/C | 0.690 | 0.0775 (0.0089) | -0.0045 (0.0016) | 75.8 | -0.058 |
| Alzheimer's disease (Bellenguez 2022) | Hand grip strength (left) | rs10933431 | 2:233981912 | C/G | 0.766 | 0.0852 (0.0099) | -0.0040 (0.0018) | 74.1 | -0.047 |
| Alzheimer's disease (Bellenguez 2022) | Hand grip strength (left) | rs4292 | 17:61554341 | T/C | 0.617 | 0.0688 (0.0084) | 0.0001 (0.0015) | 67.1 | 0.001 |
| Alzheimer's disease (Bellenguez 2022) | Hand grip strength (left) | rs6014724 | 20:54998544 | G/A | 0.090 | -0.1176 (0.0145) | -0.0036 (0.0026) | 65.8 | 0.031 |
| Alzheimer's disease (Bellenguez 2022) | Hand grip strength (left) | rs12590654 | 14:92938855 | A/G | 0.328 | -0.0693 (0.0087) | -0.0006 (0.0016) | 63.5 | 0.009 |
| Alzheimer's disease (Bellenguez 2022) | Hand grip strength (left) | rs11218343 | 11:121435587 | C/T | 0.039 | -0.1654 (0.0214) | 0.0000 (0.0039) | 59.7 | -0.000 |
| Alzheimer's disease (Bellenguez 2022) | Hand grip strength (left) | rs6586028 | 10:82253984 | T/C | 0.804 | 0.0791 (0.0103) | -0.0040 (0.0018) | 59.0 | -0.051 |
| Alzheimer's disease (Bellenguez 2022) | Hand grip strength (left) | rs755951 | 8:27226790 | C/A | 0.412 | 0.0618 (0.0083) | 0.0019 (0.0015) | 55.4 | 0.030 |
| Alzheimer's disease (Bellenguez 2022) | Hand grip strength (left) | rs62375397 | 5:86298531 | T/C | 0.210 | 0.0746 (0.0100) | -0.0023 (0.0018) | 55.6 | -0.031 |
| Alzheimer's disease (Bellenguez 2022) | Hand grip strength (left) | rs9676738 | 19:45704771 | A/G | 0.042 | 0.1689 (0.0228) | 0.0017 (0.0040) | 54.9 | 0.010 |
| Alzheimer's disease (Bellenguez 2022) | Hand grip strength (left) | rs6846529 | 4:11025131 | T/C | 0.717 | -0.0673 (0.0091) | -0.0034 (0.0017) | 54.7 | 0.051 |
| Alzheimer's disease (Bellenguez 2022) | Hand grip strength (left) | rs1385742 | 6:47595155 | T/A | 0.643 | -0.0624 (0.0086) | -0.0027 (0.0016) | 52.6 | 0.043 |
| Alzheimer's disease (Bellenguez 2022) | Hand grip strength (left) | rs5848 | 17:42430244 | T/C | 0.289 | 0.0646 (0.0092) | 0.0029 (0.0017) | 49.3 | 0.045 |
| Alzheimer's disease (Bellenguez 2022) | Hand grip strength (left) | rs12444183 | 16:81773209 | G/A | 0.613 | 0.0591 (0.0084) | 0.0007 (0.0015) | 49.5 | 0.012 |
| Alzheimer's disease (Bellenguez 2022) | Hand grip strength (left) | rs7912495 | 10:11718713 | G/A | 0.462 | 0.0572 (0.0082) | 0.0011 (0.0015) | 48.7 | 0.019 |
| Alzheimer's disease (Bellenguez 2022) | Hand grip strength (left) | rs34173062 | 8:145158607 | A/G | 0.081 | 0.1139 (0.0163) | 0.0060 (0.0030) | 48.8 | 0.053 |
| Alzheimer's disease (Bellenguez 2022) | Hand grip strength (left) | rs11500477 | 11:47409051 | A/T | 0.375 | 0.0585 (0.0084) | 0.0072 (0.0016) | 48.5 | 0.123 |
| Alzheimer's disease (Bellenguez 2022) | Hand grip strength (left) | rs113706587 | 5:179628150 | A/G | 0.110 | 0.0925 (0.0133) | -0.0015 (0.0025) | 48.4 | -0.017 |
| Alzheimer's disease (Bellenguez 2022) | Hand grip strength (left) | rs593742 | 15:59045774 | G/A | 0.295 | -0.0610 (0.0090) | -0.0014 (0.0016) | 45.9 | 0.023 |
| Alzheimer's disease (Bellenguez 2022) | Hand grip strength (left) | rs4714447 | 6:41239822 | T/C | 0.345 | 0.0578 (0.0086) | -0.0018 (0.0016) | 45.2 | -0.032 |
| Alzheimer's disease (Bellenguez 2022) | Hand grip strength (left) | rs74685827 | 11:121353077 | G/T | 0.019 | 0.1964 (0.0303) | -0.0001 (0.0054) | 42.0 | -0.000 |
| Alzheimer's disease (Bellenguez 2022) | Hand grip strength (left) | rs28577986 | 14:106200232 | A/G | 0.263 | 0.0698 (0.0111) | 0.0014 (0.0017) | 39.5 | 0.020 |
| Alzheimer's disease (Bellenguez 2022) | Hand grip strength (left) | rs3822030 | 4:987343 | T/G | 0.571 | 0.0514 (0.0083) | -0.0007 (0.0015) | 38.4 | -0.013 |
| Alzheimer's disease (Bellenguez 2022) | Hand grip strength (left) | rs17125924 | 14:53391680 | G/A | 0.089 | 0.0881 (0.0142) | 0.0088 (0.0026) | 38.5 | 0.100 |
| Alzheimer's disease (Bellenguez 2022) | Hand grip strength (left) | rs190651665 | 19:45456103 | G/T | 0.030 | -0.1598 (0.0260) | -0.0114 (0.0042) | 37.8 | 0.071 |
| Alzheimer's disease (Bellenguez 2022) | Hand grip strength (left) | rs2154481 | 21:27473875 | T/C | 0.524 | 0.0500 (0.0082) | -0.0005 (0.0015) | 37.2 | -0.010 |
| Alzheimer's disease (Bellenguez 2022) | Hand grip strength (left) | rs2830489 | 21:28148191 | T/C | 0.281 | -0.0547 (0.0091) | 0.0007 (0.0017) | 36.1 | -0.014 |
| Alzheimer's disease (Bellenguez 2022) | Hand grip strength (left) | rs4734295 | 8:96000919 | G/A | 0.461 | 0.0487 (0.0081) | -0.0021 (0.0015) | 36.1 | -0.043 |
| Alzheimer's disease (Bellenguez 2022) | Hand grip strength (left) | rs74504435 | 7:54949256 | G/A | 0.093 | -0.0842 (0.0141) | 0.0020 (0.0026) | 35.7 | -0.024 |
| Alzheimer's disease (Bellenguez 2022) | Hand grip strength (left) | rs199451 | 17:44801784 | A/G | 0.212 | -0.0594 (0.0100) | -0.0139 (0.0018) | 35.3 | 0.234 |
| Alzheimer's disease (Bellenguez 2022) | Hand grip strength (left) | rs1065712 | 8:11702122 | C/G | 0.053 | 0.1081 (0.0185) | 0.0043 (0.0035) | 34.1 | 0.040 |
| Alzheimer's disease (Bellenguez 2022) | Hand grip strength (left) | rs7068231 | 10:61784928 | G/T | 0.597 | 0.0487 (0.0084) | -0.0001 (0.0015) | 33.6 | -0.001 |
| Alzheimer's disease (Bellenguez 2022) | Hand grip strength (left) | rs2070901 | 1:161185058 | T/G | 0.265 | -0.0527 (0.0092) | 0.0009 (0.0017) | 32.8 | -0.018 |
| Alzheimer's disease (Bellenguez 2022) | Hand grip strength (left) | rs74745468 | 14:39887676 | A/G | 0.087 | 0.0822 (0.0145) | -0.0076 (0.0027) | 32.1 | -0.093 |
| Alzheimer's disease (Bellenguez 2022) | Hand grip strength (left) | rs1693551 | 8:101675584 | C/T | 0.467 | 0.0459 (0.0081) | 0.0003 (0.0015) | 32.1 | 0.007 |
| Alzheimer's disease (Bellenguez 2022) | Hand grip strength (left) | rs4485362 | 16:81975831 | G/T | 0.460 | -0.0463 (0.0083) | 0.0017 (0.0015) | 31.1 | -0.036 |
| Alzheimer's disease (Bellenguez 2022) | Hand grip strength (left) | rs11495981 | 7:28177301 | T/C | 0.209 | -0.0561 (0.0100) | -0.0015 (0.0018) | 31.5 | 0.027 |
| Alzheimer's disease (Bellenguez 2022) | Hand grip strength (left) | rs61762319 | 3:154801978 | G/A | 0.026 | 0.1430 (0.0255) | 0.0032 (0.0044) | 31.4 | 0.022 |
| Alzheimer's disease (Bellenguez 2022) | Hand grip strength (left) | rs976271 | 6:114682727 | A/G | 0.361 | 0.0467 (0.0084) | -0.0001 (0.0015) | 30.9 | -0.003 |
| Alzheimer's disease (Bellenguez 2022) | Hand grip strength (left) | rs2526377 | 17:56410041 | G/A | 0.445 | -0.0452 (0.0082) | -0.0006 (0.0015) | 30.4 | 0.014 |
| All-cause dementia (FinnGen) | Appendicular lean mass | rs769449 | 19:45410002 | A/G | 0.161 | 1.0557 (0.0287) | 0.0039 (0.0029) | 1353.1 | 0.004 |
| All-cause dementia (FinnGen) | Appendicular lean mass | rs34041051 | 19:45442349 | C/T | 0.375 | 0.1769 (0.0201) | -0.0051 (0.0021) | 77.5 | -0.029 |
| All-cause dementia (FinnGen) | Appendicular lean mass | rs111371860 | 19:45345787 | T/A | 0.098 | 0.2879 (0.0332) | 0.0004 (0.0039) | 75.2 | 0.001 |
| All-cause dementia (FinnGen) | Appendicular lean mass | rs41289514 | 19:45359667 | G/A | 0.011 | 0.7695 (0.1009) | 0.0216 (0.0093) | 58.2 | 0.028 |
| All-cause dementia (FinnGen) | Appendicular lean mass | rs187876027 | 19:46779644 | A/G | 0.039 | 0.2814 (0.0500) | -0.0044 (0.0050) | 31.7 | -0.016 |
| All-cause dementia (FinnGen) | Appendicular lean mass | rs11902339 | 2:433940 | T/C | 0.213 | 0.1306 (0.0234) | 0.0080 (0.0021) | 31.1 | 0.061 |
| All-cause dementia (FinnGen) | Low hand grip strength (FNIH, 60+) | rs769449 | 19:45410002 | A/G | 0.161 | 1.0557 (0.0287) | 0.0216 (0.0163) | 1353.1 | 0.020 |
| All-cause dementia (FinnGen) | Low hand grip strength (FNIH, 60+) | rs34041051 | 19:45442349 | C/T | 0.375 | 0.1769 (0.0201) | 0.0267 (0.0118) | 77.5 | 0.151 |
| All-cause dementia (FinnGen) | Low hand grip strength (FNIH, 60+) | rs111371860 | 19:45345787 | T/A | 0.098 | 0.2879 (0.0332) | 0.0288 (0.0216) | 75.2 | 0.100 |
| All-cause dementia (FinnGen) | Low hand grip strength (FNIH, 60+) | rs41289514 | 19:45359667 | G/A | 0.011 | 0.7695 (0.1009) | 0.0835 (0.0524) | 58.2 | 0.109 |
| All-cause dementia (FinnGen) | Low hand grip strength (FNIH, 60+) | rs187876027 | 19:46779644 | A/G | 0.039 | 0.2814 (0.0500) | 0.0154 (0.0276) | 31.7 | 0.055 |
| All-cause dementia (FinnGen) | Low hand grip strength (FNIH, 60+) | rs11902339 | 2:433940 | T/C | 0.213 | 0.1306 (0.0234) | 0.0042 (0.0121) | 31.1 | 0.032 |
| All-cause dementia (FinnGen) | Hand grip strength (right) | rs769449 | 19:45410002 | A/G | 0.161 | 1.0557 (0.0287) | 0.0014 (0.0022) | 1353.1 | 0.001 |
| All-cause dementia (FinnGen) | Hand grip strength (right) | rs34041051 | 19:45442349 | C/T | 0.375 | 0.1769 (0.0201) | -0.0012 (0.0017) | 77.5 | -0.007 |
| All-cause dementia (FinnGen) | Hand grip strength (right) | rs111371860 | 19:45345787 | T/A | 0.098 | 0.2879 (0.0332) | -0.0049 (0.0031) | 75.2 | -0.017 |
| All-cause dementia (FinnGen) | Hand grip strength (right) | rs41289514 | 19:45359667 | G/A | 0.011 | 0.7695 (0.1009) | -0.0016 (0.0073) | 58.2 | -0.002 |
| All-cause dementia (FinnGen) | Hand grip strength (right) | rs187876027 | 19:46779644 | A/G | 0.039 | 0.2814 (0.0500) | -0.0033 (0.0039) | 31.7 | -0.012 |
| All-cause dementia (FinnGen) | Hand grip strength (right) | rs11902339 | 2:433940 | T/C | 0.213 | 0.1306 (0.0234) | -0.0014 (0.0017) | 31.1 | -0.011 |
| All-cause dementia (FinnGen) | Whole body fat-free mass | rs769449 | 19:45410002 | A/G | 0.161 | 1.0557 (0.0287) | -0.0066 (0.0019) | 1353.1 | -0.006 |
| All-cause dementia (FinnGen) | Whole body fat-free mass | rs34041051 | 19:45442349 | C/T | 0.375 | 0.1769 (0.0201) | -0.0051 (0.0014) | 77.5 | -0.029 |
| All-cause dementia (FinnGen) | Whole body fat-free mass | rs111371860 | 19:45345787 | T/A | 0.098 | 0.2879 (0.0332) | 0.0078 (0.0026) | 75.2 | 0.027 |
| All-cause dementia (FinnGen) | Whole body fat-free mass | rs41289514 | 19:45359667 | G/A | 0.011 | 0.7695 (0.1009) | -0.0082 (0.0061) | 58.2 | -0.011 |
| All-cause dementia (FinnGen) | Whole body fat-free mass | rs187876027 | 19:46779644 | A/G | 0.039 | 0.2814 (0.0500) | -0.0026 (0.0033) | 31.7 | -0.009 |
| All-cause dementia (FinnGen) | Whole body fat-free mass | rs11902339 | 2:433940 | T/C | 0.213 | 0.1306 (0.0234) | 0.0087 (0.0014) | 31.1 | 0.067 |
| All-cause dementia (FinnGen) | Walking pace | rs769449 | 19:45410002 | A/G | 0.161 | 1.0557 (0.0287) | 0.0056 (0.0019) | 1353.1 | 0.005 |
| All-cause dementia (FinnGen) | Walking pace | rs34041051 | 19:45442349 | C/T | 0.375 | 0.1769 (0.0201) | -0.0012 (0.0014) | 77.5 | -0.007 |
| All-cause dementia (FinnGen) | Walking pace | rs111371860 | 19:45345787 | T/A | 0.098 | 0.2879 (0.0332) | -0.0050 (0.0026) | 75.2 | -0.017 |
| All-cause dementia (FinnGen) | Walking pace | rs41289514 | 19:45359667 | G/A | 0.011 | 0.7695 (0.1009) | 0.0132 (0.0062) | 58.2 | 0.017 |
| All-cause dementia (FinnGen) | Walking pace | rs187876027 | 19:46779644 | A/G | 0.039 | 0.2814 (0.0500) | -0.0026 (0.0033) | 31.7 | -0.009 |
| All-cause dementia (FinnGen) | Walking pace | rs11902339 | 2:433940 | T/C | 0.213 | 0.1306 (0.0234) | -0.0021 (0.0014) | 31.1 | -0.016 |
| All-cause dementia (FinnGen) | Hand grip strength (left) | rs769449 | 19:45410002 | A/G | 0.161 | 1.0557 (0.0287) | -0.0008 (0.0022) | 1353.1 | -0.001 |
| All-cause dementia (FinnGen) | Hand grip strength (left) | rs34041051 | 19:45442349 | C/T | 0.375 | 0.1769 (0.0201) | -0.0017 (0.0017) | 77.5 | -0.010 |
| All-cause dementia (FinnGen) | Hand grip strength (left) | rs111371860 | 19:45345787 | T/A | 0.098 | 0.2879 (0.0332) | -0.0056 (0.0031) | 75.2 | -0.019 |
| All-cause dementia (FinnGen) | Hand grip strength (left) | rs41289514 | 19:45359667 | G/A | 0.011 | 0.7695 (0.1009) | -0.0065 (0.0073) | 58.2 | -0.008 |
| All-cause dementia (FinnGen) | Hand grip strength (left) | rs187876027 | 19:46779644 | A/G | 0.039 | 0.2814 (0.0500) | -0.0047 (0.0039) | 31.7 | -0.017 |
| All-cause dementia (FinnGen) | Hand grip strength (left) | rs11902339 | 2:433940 | T/C | 0.213 | 0.1306 (0.0234) | -0.0018 (0.0017) | 31.1 | -0.014 |
| Alzheimer's disease, wide (FinnGen) | Appendicular lean mass | rs59007384 | 19:45396665 | T/G | 0.221 | 0.9936 (0.0278) | 0.0045 (0.0024) | 1277.4 | 0.005 |
| Alzheimer's disease, wide (FinnGen) | Appendicular lean mass | rs2965109 | 19:45225345 | T/C | 0.322 | -0.1959 (0.0230) | -0.0009 (0.0020) | 72.5 | 0.005 |
| Alzheimer's disease, wide (FinnGen) | Appendicular lean mass | rs11666698 | 19:46157748 | G/A | 0.153 | 0.2080 (0.0299) | 0.0038 (0.0032) | 48.4 | 0.018 |
| Alzheimer's disease, wide (FinnGen) | Appendicular lean mass | rs1057335 | 17:1657653 | A/G | 0.182 | 0.1613 (0.0280) | -0.0071 (0.0024) | 33.2 | -0.044 |
| Alzheimer's disease, wide (FinnGen) | Appendicular lean mass | rs5848 | 17:42430244 | T/C | 0.344 | 0.1230 (0.0224) | 0.0086 (0.0021) | 30.1 | 0.070 |
| Alzheimer's disease, wide (FinnGen) | Low hand grip strength (FNIH, 60+) | rs59007384 | 19:45396665 | T/G | 0.221 | 0.9936 (0.0278) | 0.0190 (0.0131) | 1277.4 | 0.019 |
| Alzheimer's disease, wide (FinnGen) | Low hand grip strength (FNIH, 60+) | rs2965109 | 19:45225345 | T/C | 0.322 | -0.1959 (0.0230) | -0.0153 (0.0109) | 72.5 | 0.078 |
| Alzheimer's disease, wide (FinnGen) | Low hand grip strength (FNIH, 60+) | rs11666698 | 19:46157748 | G/A | 0.153 | 0.2080 (0.0299) | 0.0055 (0.0175) | 48.4 | 0.026 |
| Alzheimer's disease, wide (FinnGen) | Low hand grip strength (FNIH, 60+) | rs1057335 | 17:1657653 | A/G | 0.182 | 0.1613 (0.0280) | 0.0003 (0.0136) | 33.2 | 0.002 |
| Alzheimer's disease, wide (FinnGen) | Low hand grip strength (FNIH, 60+) | rs5848 | 17:42430244 | T/C | 0.344 | 0.1230 (0.0224) | -0.0161 (0.0119) | 30.1 | -0.131 |
| Alzheimer's disease, wide (FinnGen) | Hand grip strength (right) | rs59007384 | 19:45396665 | T/G | 0.221 | 0.9936 (0.0278) | 0.0011 (0.0018) | 1277.4 | 0.001 |
| Alzheimer's disease, wide (FinnGen) | Hand grip strength (right) | rs2965109 | 19:45225345 | T/C | 0.322 | -0.1959 (0.0230) | -0.0007 (0.0015) | 72.5 | 0.004 |
| Alzheimer's disease, wide (FinnGen) | Hand grip strength (right) | rs11666698 | 19:46157748 | G/A | 0.153 | 0.2080 (0.0299) | -0.0002 (0.0025) | 48.4 | -0.001 |
| Alzheimer's disease, wide (FinnGen) | Hand grip strength (right) | rs1057335 | 17:1657653 | A/G | 0.182 | 0.1613 (0.0280) | -0.0023 (0.0019) | 33.2 | -0.014 |
| Alzheimer's disease, wide (FinnGen) | Hand grip strength (right) | rs5848 | 17:42430244 | T/C | 0.344 | 0.1230 (0.0224) | 0.0029 (0.0017) | 30.1 | 0.023 |
| Alzheimer's disease, wide (FinnGen) | Whole body fat-free mass | rs59007384 | 19:45396665 | T/G | 0.221 | 0.9936 (0.0278) | -0.0028 (0.0015) | 1277.4 | -0.003 |
| Alzheimer's disease, wide (FinnGen) | Whole body fat-free mass | rs2965109 | 19:45225345 | T/C | 0.322 | -0.1959 (0.0230) | 0.0037 (0.0013) | 72.5 | -0.019 |
| Alzheimer's disease, wide (FinnGen) | Whole body fat-free mass | rs11666698 | 19:46157748 | G/A | 0.153 | 0.2080 (0.0299) | -0.0043 (0.0021) | 48.4 | -0.020 |
| Alzheimer's disease, wide (FinnGen) | Whole body fat-free mass | rs1057335 | 17:1657653 | A/G | 0.182 | 0.1613 (0.0280) | -0.0025 (0.0016) | 33.2 | -0.016 |
| Alzheimer's disease, wide (FinnGen) | Whole body fat-free mass | rs5848 | 17:42430244 | T/C | 0.344 | 0.1230 (0.0224) | 0.0023 (0.0014) | 30.1 | 0.019 |
| Alzheimer's disease, wide (FinnGen) | Walking pace | rs59007384 | 19:45396665 | T/G | 0.221 | 0.9936 (0.0278) | 0.0039 (0.0016) | 1277.4 | 0.004 |
| Alzheimer's disease, wide (FinnGen) | Walking pace | rs2965109 | 19:45225345 | T/C | 0.322 | -0.1959 (0.0230) | -0.0030 (0.0013) | 72.5 | 0.015 |
| Alzheimer's disease, wide (FinnGen) | Walking pace | rs11666698 | 19:46157748 | G/A | 0.153 | 0.2080 (0.0299) | 0.0032 (0.0021) | 48.4 | 0.015 |
| Alzheimer's disease, wide (FinnGen) | Walking pace | rs1057335 | 17:1657653 | A/G | 0.182 | 0.1613 (0.0280) | 0.0017 (0.0016) | 33.2 | 0.011 |
| Alzheimer's disease, wide (FinnGen) | Walking pace | rs5848 | 17:42430244 | T/C | 0.344 | 0.1230 (0.0224) | 0.0004 (0.0014) | 30.1 | 0.004 |
| Alzheimer's disease, wide (FinnGen) | Hand grip strength (left) | rs59007384 | 19:45396665 | T/G | 0.221 | 0.9936 (0.0278) | -0.0009 (0.0018) | 1277.4 | -0.001 |
| Alzheimer's disease, wide (FinnGen) | Hand grip strength (left) | rs2965109 | 19:45225345 | T/C | 0.322 | -0.1959 (0.0230) | -0.0004 (0.0015) | 72.5 | 0.002 |
| Alzheimer's disease, wide (FinnGen) | Hand grip strength (left) | rs11666698 | 19:46157748 | G/A | 0.153 | 0.2080 (0.0299) | -0.0023 (0.0025) | 48.4 | -0.011 |
| Alzheimer's disease, wide (FinnGen) | Hand grip strength (left) | rs1057335 | 17:1657653 | A/G | 0.182 | 0.1613 (0.0280) | -0.0020 (0.0019) | 33.2 | -0.013 |
| Alzheimer's disease, wide (FinnGen) | Hand grip strength (left) | rs5848 | 17:42430244 | T/C | 0.344 | 0.1230 (0.0224) | 0.0029 (0.0017) | 30.1 | 0.024 |

**Reverse SNP files did not contain outcome P value columns; beta (SE) values are reported.**
