# Supplementary material for: Muscle quality index combined with plasma metabolomics: enhancing risk stratification for post-stroke dementia
Source: Front Aging Neurosci. 2026 Sep 29;18:1837030. doi: 10.3389/fnagi.2026.1837030 (PMC13658159; doi:10.3389/fnagi.2026.1837030)
Supplement: Supplementary file 3 [file Data_Sheet_3.DOCX]

| **Table S17. Forward Mendelian randomization results** | | | | | | | | | |
| --- | --- | --- | --- | --- | --- | --- | --- | --- | --- |
| **Exposure** | **Outcome** | **Method** | **SNPs** | **F mean/min** | **OR (95% CI)** | ***P*** | ***Q P*** | ***Egger P*** | ***PRESSO P*** |
| Hand grip strength (left) | Alzheimer's disease (Bellenguez 2022) | IVW-FE | 146 | 47.8/29.8 | 0.91 (0.80–1.04) | 0.161 | <0.001 | — | <0.001 |
| Hand grip strength (left) | Alzheimer's disease (Bellenguez 2022) | IVW-MRE | 146 | 47.8/29.8 | 0.91 (0.75–1.11) | 0.347 | <0.001 | — | <0.001 |
| Hand grip strength (left) | Alzheimer's disease (Bellenguez 2022) | MR-Egger | 146 | 47.8/29.8 | 0.92 (0.76–1.12) | 0.400 | <0.001 | 0.303 | <0.001 |
| Hand grip strength (left) | Alzheimer's disease (Bellenguez 2022) | Weighted median | 146 | 47.8/29.8 | 0.87 (0.71–1.07) | 0.182 | — | — | <0.001 |
| Hand grip strength (left) | Alzheimer's disease (Bellenguez 2022) | Simple mode | 146 | 47.8/29.8 | 0.80 (0.55–1.17) | 0.254 | — | — | <0.001 |
| Hand grip strength (left) | Alzheimer's disease (Bellenguez 2022) | Weighted mode | 146 | 47.8/29.8 | 0.86 (0.60–1.23) | 0.395 | — | — | <0.001 |
| Hand grip strength (left) | Alzheimer's disease (Bellenguez 2022) | MR-PRESSO | 146 | 47.8/29.8 | 0.89 (0.78–1.03) | 0.125 | — | — | <0.001 |
| Hand grip strength (left) | All-cause dementia (FinnGen) | IVW-FE | 142 | 47.3/29.8 | 0.81 (0.59–1.12) | 0.201 | 0.216 | — | 0.220 |
| Hand grip strength (left) | All-cause dementia (FinnGen) | IVW-MRE | 142 | 47.3/29.8 | 0.81 (0.59–1.13) | 0.221 | 0.216 | — | 0.220 |
| Hand grip strength (left) | All-cause dementia (FinnGen) | MR-Egger | 142 | 47.3/29.8 | 0.82 (0.59–1.14) | 0.234 | 0.210 | 0.446 | 0.220 |
| Hand grip strength (left) | All-cause dementia (FinnGen) | Weighted median | 142 | 47.3/29.8 | 0.77 (0.48–1.25) | 0.295 | — | — | 0.220 |
| Hand grip strength (left) | All-cause dementia (FinnGen) | Simple mode | 142 | 47.3/29.8 | 0.48 (0.18–1.28) | 0.141 | — | — | 0.220 |
| Hand grip strength (left) | All-cause dementia (FinnGen) | Weighted mode | 142 | 47.3/29.8 | 0.59 (0.23–1.48) | 0.257 | — | — | 0.220 |
| Hand grip strength (left) | All-cause dementia (FinnGen) | MR-PRESSO | 142 | 47.3/29.8 | 0.77 (0.56–1.06) | 0.105 | — | — | 0.220 |
| Hand grip strength (left) | Vascular dementia (FinnGen) | IVW-FE | 142 | 47.3/29.8 | 0.45 (0.20–1.01) | 0.052 | 0.396 | — | 0.397 |
| Hand grip strength (left) | Vascular dementia (FinnGen) | IVW-MRE | 142 | 47.3/29.8 | 0.45 (0.20–1.02) | 0.056 | 0.396 | — | 0.397 |
| Hand grip strength (left) | Vascular dementia (FinnGen) | MR-Egger | 142 | 47.3/29.8 | 0.45 (0.20–1.02) | 0.057 | 0.375 | 0.758 | 0.397 |
| Hand grip strength (left) | Vascular dementia (FinnGen) | Weighted median | 142 | 47.3/29.8 | 0.54 (0.16–1.77) | 0.306 | — | — | 0.397 |
| Hand grip strength (left) | Vascular dementia (FinnGen) | Simple mode | 142 | 47.3/29.8 | 2.52 (0.16–38.45) | 0.507 | — | — | 0.397 |
| Hand grip strength (left) | Vascular dementia (FinnGen) | Weighted mode | 142 | 47.3/29.8 | 2.30 (0.28–18.81) | 0.437 | — | — | 0.397 |
| Hand grip strength (left) | Vascular dementia (FinnGen) | MR-PRESSO | 142 | 47.3/29.8 | 0.48 (0.21–1.11) | 0.087 | — | — | 0.397 |
| Hand grip strength (left) | Alzheimer's disease, wide (FinnGen) | IVW-FE | 142 | 47.3/29.8 | 0.89 (0.63–1.26) | 0.508 | 0.304 | — | 0.313 |
| Hand grip strength (left) | Alzheimer's disease, wide (FinnGen) | IVW-MRE | 142 | 47.3/29.8 | 0.89 (0.62–1.27) | 0.520 | 0.304 | — | 0.313 |
| Hand grip strength (left) | Alzheimer's disease, wide (FinnGen) | MR-Egger | 142 | 47.3/29.8 | 0.89 (0.62–1.28) | 0.541 | 0.299 | 0.395 | 0.313 |
| Hand grip strength (left) | Alzheimer's disease, wide (FinnGen) | Weighted median | 142 | 47.3/29.8 | 0.86 (0.50–1.50) | 0.603 | — | — | 0.313 |
| Hand grip strength (left) | Alzheimer's disease, wide (FinnGen) | Simple mode | 142 | 47.3/29.8 | 0.95 (0.26–3.48) | 0.937 | — | — | 0.313 |
| Hand grip strength (left) | Alzheimer's disease, wide (FinnGen) | Weighted mode | 142 | 47.3/29.8 | 0.99 (0.36–2.67) | 0.977 | — | — | 0.313 |
| Hand grip strength (left) | Alzheimer's disease, wide (FinnGen) | MR-PRESSO | 142 | 47.3/29.8 | 0.90 (0.63–1.28) | 0.552 | — | — | 0.313 |
| Hand grip strength (right) | Alzheimer's disease (Bellenguez 2022) | IVW-FE | 162 | 47.4/29.9 | 0.93 (0.82–1.05) | 0.260 | <0.001 | — | <0.001 |
| Hand grip strength (right) | Alzheimer's disease (Bellenguez 2022) | IVW-MRE | 162 | 47.4/29.9 | 0.93 (0.78–1.11) | 0.420 | <0.001 | — | <0.001 |
| Hand grip strength (right) | Alzheimer's disease (Bellenguez 2022) | MR-Egger | 162 | 47.4/29.9 | 0.93 (0.78–1.11) | 0.415 | <0.001 | 0.716 | <0.001 |
| Hand grip strength (right) | Alzheimer's disease (Bellenguez 2022) | Weighted median | 162 | 47.4/29.9 | 0.85 (0.70–1.04) | 0.114 | — | — | <0.001 |
| Hand grip strength (right) | Alzheimer's disease (Bellenguez 2022) | Simple mode | 162 | 47.4/29.9 | 0.89 (0.59–1.32) | 0.551 | — | — | <0.001 |
| Hand grip strength (right) | Alzheimer's disease (Bellenguez 2022) | Weighted mode | 162 | 47.4/29.9 | 0.92 (0.67–1.26) | 0.607 | — | — | <0.001 |
| Hand grip strength (right) | Alzheimer's disease (Bellenguez 2022) | MR-PRESSO | 162 | 47.4/29.9 | 0.89 (0.78–1.01) | 0.073 | — | — | <0.001 |
| Hand grip strength (right) | All-cause dementia (FinnGen) | IVW-FE | 161 | 46.9/29.9 | 0.87 (0.65–1.17) | 0.361 | 0.018 | — | 0.020 |
| Hand grip strength (right) | All-cause dementia (FinnGen) | IVW-MRE | 161 | 46.9/29.9 | 0.87 (0.63–1.21) | 0.414 | 0.018 | — | 0.020 |
| Hand grip strength (right) | All-cause dementia (FinnGen) | MR-Egger | 161 | 46.9/29.9 | 0.87 (0.63–1.21) | 0.417 | 0.018 | 0.333 | 0.020 |
| Hand grip strength (right) | All-cause dementia (FinnGen) | Weighted median | 161 | 46.9/29.9 | 0.85 (0.55–1.34) | 0.492 | — | — | 0.020 |
| Hand grip strength (right) | All-cause dementia (FinnGen) | Simple mode | 161 | 46.9/29.9 | 1.64 (0.56–4.81) | 0.368 | — | — | 0.020 |
| Hand grip strength (right) | All-cause dementia (FinnGen) | Weighted mode | 161 | 46.9/29.9 | 1.42 (0.55–3.62) | 0.467 | — | — | 0.020 |
| Hand grip strength (right) | All-cause dementia (FinnGen) | MR-PRESSO | 161 | 46.9/29.9 | 0.93 (0.68–1.26) | 0.640 | — | — | 0.020 |
| Hand grip strength (right) | Vascular dementia (FinnGen) | IVW-FE | 161 | 46.9/29.9 | 0.51 (0.24–1.10) | 0.085 | 0.035 | — | 0.037 |
| Hand grip strength (right) | Vascular dementia (FinnGen) | IVW-MRE | 161 | 46.9/29.9 | 0.51 (0.22–1.18) | 0.117 | 0.035 | — | 0.037 |
| Hand grip strength (right) | Vascular dementia (FinnGen) | MR-Egger | 161 | 46.9/29.9 | 0.51 (0.22–1.18) | 0.120 | 0.032 | 0.706 | 0.037 |
| Hand grip strength (right) | Vascular dementia (FinnGen) | Weighted median | 161 | 46.9/29.9 | 0.92 (0.29–2.90) | 0.893 | — | — | 0.037 |
| Hand grip strength (right) | Vascular dementia (FinnGen) | Simple mode | 161 | 46.9/29.9 | 4.15 (0.39–44.66) | 0.240 | — | — | 0.037 |
| Hand grip strength (right) | Vascular dementia (FinnGen) | Weighted mode | 161 | 46.9/29.9 | 2.27 (0.31–16.73) | 0.419 | — | — | 0.037 |
| Hand grip strength (right) | Vascular dementia (FinnGen) | MR-PRESSO | 161 | 46.9/29.9 | 0.77 (0.35–1.68) | 0.508 | — | — | 0.037 |
| Hand grip strength (right) | Alzheimer's disease, wide (FinnGen) | IVW-FE | 161 | 46.9/29.9 | 0.85 (0.61–1.18) | 0.329 | 0.090 | — | 0.100 |
| Hand grip strength (right) | Alzheimer's disease, wide (FinnGen) | IVW-MRE | 161 | 46.9/29.9 | 0.85 (0.60–1.21) | 0.363 | 0.090 | — | 0.100 |
| Hand grip strength (right) | Alzheimer's disease, wide (FinnGen) | MR-Egger | 161 | 46.9/29.9 | 0.85 (0.60–1.21) | 0.366 | 0.083 | 0.752 | 0.100 |
| Hand grip strength (right) | Alzheimer's disease, wide (FinnGen) | Weighted median | 161 | 46.9/29.9 | 0.87 (0.52–1.44) | 0.577 | — | — | 0.100 |
| Hand grip strength (right) | Alzheimer's disease, wide (FinnGen) | Simple mode | 161 | 46.9/29.9 | 0.60 (0.19–1.96) | 0.400 | — | — | 0.100 |
| Hand grip strength (right) | Alzheimer's disease, wide (FinnGen) | Weighted mode | 161 | 46.9/29.9 | 1.01 (0.39–2.60) | 0.979 | — | — | 0.100 |
| Hand grip strength (right) | Alzheimer's disease, wide (FinnGen) | MR-PRESSO | 161 | 46.9/29.9 | 0.92 (0.66–1.28) | 0.626 | — | — | 0.100 |
| Low hand grip strength (FNIH, 60+) | Alzheimer's disease (Bellenguez 2022) | IVW-FE | 3 | 35.2/30.6 | 1.03 (0.89–1.20) | 0.664 | 0.461 | — | — |
| Low hand grip strength (FNIH, 60+) | Alzheimer's disease (Bellenguez 2022) | IVW-MRE | 3 | 35.2/30.6 | 1.03 (0.89–1.20) | 0.664 | 0.461 | — | — |
| Low hand grip strength (FNIH, 60+) | Alzheimer's disease (Bellenguez 2022) | MR-Egger | 3 | 35.2/30.6 | 1.13 (0.91–1.41) | 0.476 | 0.525 | 0.479 | — |
| Low hand grip strength (FNIH, 60+) | Alzheimer's disease (Bellenguez 2022) | Weighted median | 3 | 35.2/30.6 | 1.03 (0.86–1.24) | 0.725 | — | — | — |
| Low hand grip strength (FNIH, 60+) | Alzheimer's disease (Bellenguez 2022) | Simple mode | 3 | 35.2/30.6 | 1.04 (0.86–1.27) | 0.671 | — | — | — |
| Low hand grip strength (FNIH, 60+) | Alzheimer's disease (Bellenguez 2022) | Weighted mode | 3 | 35.2/30.6 | 1.03 (0.86–1.24) | 0.725 | — | — | — |
| Low hand grip strength (FNIH, 60+) | All-cause dementia (FinnGen) | IVW-FE | 4 | 43.0/30.6 | 0.97 (0.73–1.29) | 0.826 | 0.549 | — | 0.547 |
| Low hand grip strength (FNIH, 60+) | All-cause dementia (FinnGen) | IVW-MRE | 4 | 43.0/30.6 | 0.97 (0.73–1.29) | 0.826 | 0.549 | — | 0.547 |
| Low hand grip strength (FNIH, 60+) | All-cause dementia (FinnGen) | MR-Egger | 4 | 43.0/30.6 | 0.92 (0.68–1.23) | 0.627 | 0.765 | 0.336 | 0.547 |
| Low hand grip strength (FNIH, 60+) | All-cause dementia (FinnGen) | Weighted median | 4 | 43.0/30.6 | 1.10 (0.78–1.55) | 0.591 | — | — | 0.547 |
| Low hand grip strength (FNIH, 60+) | All-cause dementia (FinnGen) | Simple mode | 4 | 43.0/30.6 | 1.16 (0.73–1.86) | 0.529 | — | — | 0.547 |
| Low hand grip strength (FNIH, 60+) | All-cause dementia (FinnGen) | Weighted mode | 4 | 43.0/30.6 | 1.14 (0.74–1.76) | 0.553 | — | — | 0.547 |
| Low hand grip strength (FNIH, 60+) | Vascular dementia (FinnGen) | IVW-FE | 4 | 43.0/30.6 | 1.11 (0.54–2.32) | 0.772 | 0.401 | — | 0.403 |
| Low hand grip strength (FNIH, 60+) | Vascular dementia (FinnGen) | IVW-MRE | 4 | 43.0/30.6 | 1.11 (0.54–2.32) | 0.772 | 0.401 | — | 0.403 |
| Low hand grip strength (FNIH, 60+) | Vascular dementia (FinnGen) | MR-Egger | 4 | 43.0/30.6 | 1.03 (0.44–2.42) | 0.945 | 0.289 | 0.605 | 0.403 |
| Low hand grip strength (FNIH, 60+) | Vascular dementia (FinnGen) | Weighted median | 4 | 43.0/30.6 | 1.00 (0.41–2.43) | 0.998 | — | — | 0.403 |
| Low hand grip strength (FNIH, 60+) | Vascular dementia (FinnGen) | Simple mode | 4 | 43.0/30.6 | 0.87 (0.32–2.38) | 0.786 | — | — | 0.403 |
| Low hand grip strength (FNIH, 60+) | Vascular dementia (FinnGen) | Weighted mode | 4 | 43.0/30.6 | 1.03 (0.39–2.75) | 0.954 | — | — | 0.403 |
| Low hand grip strength (FNIH, 60+) | Alzheimer's disease, wide (FinnGen) | IVW-FE | 4 | 43.0/30.6 | 1.08 (0.79–1.47) | 0.648 | 0.156 | — | 0.163 |
| Low hand grip strength (FNIH, 60+) | Alzheimer's disease, wide (FinnGen) | IVW-MRE | 4 | 43.0/30.6 | 1.08 (0.71–1.63) | 0.730 | 0.156 | — | 0.163 |
| Low hand grip strength (FNIH, 60+) | Alzheimer's disease, wide (FinnGen) | MR-Egger | 4 | 43.0/30.6 | 1.00 (0.67–1.50) | 0.989 | 0.223 | 0.348 | 0.163 |
| Low hand grip strength (FNIH, 60+) | Alzheimer's disease, wide (FinnGen) | Weighted median | 4 | 43.0/30.6 | 1.08 (0.71–1.65) | 0.721 | — | — | 0.163 |
| Low hand grip strength (FNIH, 60+) | Alzheimer's disease, wide (FinnGen) | Simple mode | 4 | 43.0/30.6 | 1.44 (0.84–2.46) | 0.188 | — | — | 0.163 |
| Low hand grip strength (FNIH, 60+) | Alzheimer's disease, wide (FinnGen) | Weighted mode | 4 | 43.0/30.6 | 0.98 (0.58–1.63) | 0.927 | — | — | 0.163 |
| Low hand grip strength (FNIH, 60+) | Alzheimer's disease, wide (FinnGen) | MR-PRESSO | 4 | 43.0/30.6 | 1.44 (0.95–2.19) | 0.086 | — | — | 0.163 |
| Appendicular lean mass | Alzheimer's disease (Bellenguez 2022) | IVW-FE | 578 | 98.2/28.8 | 0.90 (0.87–0.94) | <0.001 | <0.001 | — | <0.001 |
| Appendicular lean mass | Alzheimer's disease (Bellenguez 2022) | IVW-MRE | 578 | 98.2/28.8 | 0.90 (0.86–0.94) | <0.001 | <0.001 | — | <0.001 |
| Appendicular lean mass | Alzheimer's disease (Bellenguez 2022) | MR-Egger | 578 | 98.2/28.8 | 0.90 (0.86–0.94) | <0.001 | <0.001 | 0.301 | <0.001 |
| Appendicular lean mass | Alzheimer's disease (Bellenguez 2022) | Weighted median | 578 | 98.2/28.8 | 0.92 (0.87–0.98) | 0.008 | — | — | <0.001 |
| Appendicular lean mass | Alzheimer's disease (Bellenguez 2022) | Simple mode | 578 | 98.2/28.8 | 0.90 (0.79–1.03) | 0.127 | — | — | <0.001 |
| Appendicular lean mass | Alzheimer's disease (Bellenguez 2022) | Weighted mode | 578 | 98.2/28.8 | 0.92 (0.84–1.01) | 0.087 | — | — | <0.001 |
| Appendicular lean mass | Alzheimer's disease (Bellenguez 2022) | MR-PRESSO | 578 | 98.2/28.8 | 0.91 (0.87–0.94) | <0.001 | — | — | <0.001 |
| Appendicular lean mass | All-cause dementia (FinnGen) | IVW-FE | 571 | 99.1/28.8 | 0.93 (0.86–1.01) | 0.099 | 0.735 | — | 0.735 |
| Appendicular lean mass | All-cause dementia (FinnGen) | IVW-MRE | 571 | 99.1/28.8 | 0.93 (0.86–1.01) | 0.099 | 0.735 | — | 0.735 |
| Appendicular lean mass | All-cause dementia (FinnGen) | MR-Egger | 571 | 99.1/28.8 | 0.93 (0.86–1.01) | 0.100 | 0.725 | 0.979 | 0.735 |
| Appendicular lean mass | All-cause dementia (FinnGen) | Weighted median | 571 | 99.1/28.8 | 0.95 (0.82–1.09) | 0.455 | — | — | 0.735 |
| Appendicular lean mass | All-cause dementia (FinnGen) | Simple mode | 571 | 99.1/28.8 | 1.15 (0.86–1.53) | 0.355 | — | — | 0.735 |
| Appendicular lean mass | All-cause dementia (FinnGen) | Weighted mode | 571 | 99.1/28.8 | 1.06 (0.85–1.32) | 0.630 | — | — | 0.735 |
| Appendicular lean mass | All-cause dementia (FinnGen) | MR-PRESSO | 571 | 99.1/28.8 | 0.92 (0.85–1.01) | 0.078 | — | — | 0.735 |
| Appendicular lean mass | Vascular dementia (FinnGen) | IVW-FE | 571 | 99.1/28.8 | 0.95 (0.76–1.18) | 0.629 | 0.287 | — | 0.291 |
| Appendicular lean mass | Vascular dementia (FinnGen) | IVW-MRE | 571 | 99.1/28.8 | 0.95 (0.76–1.18) | 0.634 | 0.287 | — | 0.291 |
| Appendicular lean mass | Vascular dementia (FinnGen) | MR-Egger | 571 | 99.1/28.8 | 0.95 (0.76–1.19) | 0.653 | 0.297 | 0.157 | 0.291 |
| Appendicular lean mass | Vascular dementia (FinnGen) | Weighted median | 571 | 99.1/28.8 | 1.01 (0.70–1.46) | 0.951 | — | — | 0.291 |
| Appendicular lean mass | Vascular dementia (FinnGen) | Simple mode | 571 | 99.1/28.8 | 1.07 (0.55–2.09) | 0.844 | — | — | 0.291 |
| Appendicular lean mass | Vascular dementia (FinnGen) | Weighted mode | 571 | 99.1/28.8 | 1.07 (0.64–1.80) | 0.800 | — | — | 0.291 |
| Appendicular lean mass | Vascular dementia (FinnGen) | MR-PRESSO | 571 | 99.1/28.8 | 1.12 (0.90–1.41) | 0.303 | — | — | 0.291 |
| Appendicular lean mass | Alzheimer's disease, wide (FinnGen) | IVW-FE | 571 | 99.1/28.8 | 0.93 (0.84–1.02) | 0.106 | 0.798 | — | 0.795 |
| Appendicular lean mass | Alzheimer's disease, wide (FinnGen) | IVW-MRE | 571 | 99.1/28.8 | 0.93 (0.84–1.02) | 0.106 | 0.798 | — | 0.795 |
| Appendicular lean mass | Alzheimer's disease, wide (FinnGen) | MR-Egger | 571 | 99.1/28.8 | 0.93 (0.84–1.02) | 0.106 | 0.789 | 0.854 | 0.795 |
| Appendicular lean mass | Alzheimer's disease, wide (FinnGen) | Weighted median | 571 | 99.1/28.8 | 0.91 (0.78–1.07) | 0.246 | — | — | 0.795 |
| Appendicular lean mass | Alzheimer's disease, wide (FinnGen) | Simple mode | 571 | 99.1/28.8 | 1.05 (0.79–1.41) | 0.718 | — | — | 0.795 |
| Appendicular lean mass | Alzheimer's disease, wide (FinnGen) | Weighted mode | 571 | 99.1/28.8 | 1.05 (0.82–1.36) | 0.676 | — | — | 0.795 |
| Appendicular lean mass | Alzheimer's disease, wide (FinnGen) | MR-PRESSO | 571 | 99.1/28.8 | 0.94 (0.85–1.03) | 0.206 | — | — | 0.795 |
| Whole body fat-free mass | Alzheimer's disease (Bellenguez 2022) | IVW-FE | 517 | 86.1/29.8 | 0.77 (0.73–0.82) | <0.001 | <0.001 | — | <0.001 |
| Whole body fat-free mass | Alzheimer's disease (Bellenguez 2022) | IVW-MRE | 517 | 86.1/29.8 | 0.77 (0.72–0.83) | <0.001 | <0.001 | — | <0.001 |
| Whole body fat-free mass | Alzheimer's disease (Bellenguez 2022) | MR-Egger | 517 | 86.1/29.8 | 0.77 (0.72–0.83) | <0.001 | <0.001 | 0.766 | <0.001 |
| Whole body fat-free mass | Alzheimer's disease (Bellenguez 2022) | Weighted median | 517 | 86.1/29.8 | 0.79 (0.71–0.87) | <0.001 | — | — | <0.001 |
| Whole body fat-free mass | Alzheimer's disease (Bellenguez 2022) | Simple mode | 517 | 86.1/29.8 | 0.75 (0.58–0.98) | 0.032 | — | — | <0.001 |
| Whole body fat-free mass | Alzheimer's disease (Bellenguez 2022) | Weighted mode | 517 | 86.1/29.8 | 0.80 (0.69–0.94) | 0.005 | — | — | <0.001 |
| Whole body fat-free mass | Alzheimer's disease (Bellenguez 2022) | MR-PRESSO | 517 | 86.1/29.8 | 0.79 (0.74–0.84) | <0.001 | — | — | <0.001 |
| Whole body fat-free mass | All-cause dementia (FinnGen) | IVW-FE | 513 | 85.9/29.8 | 0.91 (0.78–1.05) | 0.185 | 0.215 | — | 0.199 |
| Whole body fat-free mass | All-cause dementia (FinnGen) | IVW-MRE | 513 | 85.9/29.8 | 0.91 (0.78–1.05) | 0.196 | 0.215 | — | 0.199 |
| Whole body fat-free mass | All-cause dementia (FinnGen) | MR-Egger | 513 | 85.9/29.8 | 0.91 (0.78–1.05) | 0.196 | 0.208 | 0.622 | 0.199 |
| Whole body fat-free mass | All-cause dementia (FinnGen) | Weighted median | 513 | 85.9/29.8 | 0.88 (0.70–1.11) | 0.278 | — | — | 0.199 |
| Whole body fat-free mass | All-cause dementia (FinnGen) | Simple mode | 513 | 85.9/29.8 | 1.13 (0.68–1.87) | 0.643 | — | — | 0.199 |
| Whole body fat-free mass | All-cause dementia (FinnGen) | Weighted mode | 513 | 85.9/29.8 | 0.78 (0.53–1.13) | 0.191 | — | — | 0.199 |
| Whole body fat-free mass | All-cause dementia (FinnGen) | MR-PRESSO | 513 | 85.9/29.8 | 0.92 (0.80–1.08) | 0.310 | — | — | 0.199 |
| Whole body fat-free mass | Vascular dementia (FinnGen) | IVW-FE | 513 | 85.9/29.8 | 0.83 (0.57–1.21) | 0.340 | 0.197 | — | 0.190 |
| Whole body fat-free mass | Vascular dementia (FinnGen) | IVW-MRE | 513 | 85.9/29.8 | 0.83 (0.57–1.22) | 0.352 | 0.197 | — | 0.190 |
| Whole body fat-free mass | Vascular dementia (FinnGen) | MR-Egger | 513 | 85.9/29.8 | 0.83 (0.57–1.22) | 0.353 | 0.192 | 0.525 | 0.190 |
| Whole body fat-free mass | Vascular dementia (FinnGen) | Weighted median | 513 | 85.9/29.8 | 1.35 (0.76–2.40) | 0.306 | — | — | 0.190 |
| Whole body fat-free mass | Vascular dementia (FinnGen) | Simple mode | 513 | 85.9/29.8 | 1.88 (0.50–7.15) | 0.353 | — | — | 0.190 |
| Whole body fat-free mass | Vascular dementia (FinnGen) | Weighted mode | 513 | 85.9/29.8 | 1.69 (0.74–3.88) | 0.215 | — | — | 0.190 |
| Whole body fat-free mass | Vascular dementia (FinnGen) | MR-PRESSO | 513 | 85.9/29.8 | 1.03 (0.70–1.51) | 0.894 | — | — | 0.190 |
| Whole body fat-free mass | Alzheimer's disease, wide (FinnGen) | IVW-FE | 513 | 85.9/29.8 | 0.88 (0.75–1.04) | 0.131 | 0.135 | — | 0.140 |
| Whole body fat-free mass | Alzheimer's disease, wide (FinnGen) | IVW-MRE | 513 | 85.9/29.8 | 0.88 (0.75–1.04) | 0.144 | 0.135 | — | 0.140 |
| Whole body fat-free mass | Alzheimer's disease, wide (FinnGen) | MR-Egger | 513 | 85.9/29.8 | 0.88 (0.75–1.04) | 0.145 | 0.128 | 0.986 | 0.140 |
| Whole body fat-free mass | Alzheimer's disease, wide (FinnGen) | Weighted median | 513 | 85.9/29.8 | 0.80 (0.61–1.05) | 0.115 | — | — | 0.140 |
| Whole body fat-free mass | Alzheimer's disease, wide (FinnGen) | Simple mode | 513 | 85.9/29.8 | 1.09 (0.64–1.85) | 0.758 | — | — | 0.140 |
| Whole body fat-free mass | Alzheimer's disease, wide (FinnGen) | Weighted mode | 513 | 85.9/29.8 | 0.80 (0.52–1.21) | 0.287 | — | — | 0.140 |
| Whole body fat-free mass | Alzheimer's disease, wide (FinnGen) | MR-PRESSO | 513 | 85.9/29.8 | 0.92 (0.77–1.08) | 0.300 | — | — | 0.140 |
| Walking pace | Alzheimer's disease (Bellenguez 2022) | IVW-FE | 56 | 40.3/29.8 | 1.22 (0.94–1.60) | 0.139 | <0.001 | — | <0.001 |
| Walking pace | Alzheimer's disease (Bellenguez 2022) | IVW-MRE | 56 | 40.3/29.8 | 1.22 (0.82–1.82) | 0.318 | <0.001 | — | <0.001 |
| Walking pace | Alzheimer's disease (Bellenguez 2022) | MR-Egger | 56 | 40.3/29.8 | 1.22 (0.82–1.82) | 0.338 | <0.001 | 0.888 | <0.001 |
| Walking pace | Alzheimer's disease (Bellenguez 2022) | Weighted median | 56 | 40.3/29.8 | 1.26 (0.80–1.98) | 0.321 | — | — | <0.001 |
| Walking pace | Alzheimer's disease (Bellenguez 2022) | Simple mode | 56 | 40.3/29.8 | 1.80 (0.74–4.39) | 0.196 | — | — | <0.001 |
| Walking pace | Alzheimer's disease (Bellenguez 2022) | Weighted mode | 56 | 40.3/29.8 | 1.76 (0.69–4.45) | 0.234 | — | — | <0.001 |
| Walking pace | Alzheimer's disease (Bellenguez 2022) | MR-PRESSO | 56 | 40.3/29.8 | 1.27 (0.95–1.71) | 0.112 | — | — | <0.001 |
| Walking pace | All-cause dementia (FinnGen) | IVW-FE | 53 | 40.3/29.8 | 1.06 (0.54–2.05) | 0.872 | 0.225 | — | 0.240 |
| Walking pace | All-cause dementia (FinnGen) | IVW-MRE | 53 | 40.3/29.8 | 1.06 (0.52–2.14) | 0.880 | 0.225 | — | 0.240 |
| Walking pace | All-cause dementia (FinnGen) | MR-Egger | 53 | 40.3/29.8 | 1.08 (0.53–2.22) | 0.833 | 0.203 | 0.660 | 0.240 |
| Walking pace | All-cause dementia (FinnGen) | Weighted median | 53 | 40.3/29.8 | 1.19 (0.45–3.11) | 0.724 | — | — | 0.240 |
| Walking pace | All-cause dementia (FinnGen) | Simple mode | 53 | 40.3/29.8 | 0.99 (0.36–2.74) | 0.985 | — | — | 0.240 |
| Walking pace | All-cause dementia (FinnGen) | Weighted mode | 53 | 40.3/29.8 | 0.99 (0.40–2.43) | 0.983 | — | — | 0.240 |
| Walking pace | All-cause dementia (FinnGen) | MR-PRESSO | 53 | 40.3/29.8 | 0.82 (0.41–1.65) | 0.580 | — | — | 0.240 |
| Walking pace | Vascular dementia (FinnGen) | IVW-FE | 53 | 40.3/29.8 | 1.21 (0.22–6.61) | 0.829 | 0.583 | — | 0.592 |
| Walking pace | Vascular dementia (FinnGen) | IVW-MRE | 53 | 40.3/29.8 | 1.21 (0.22–6.61) | 0.829 | 0.583 | — | 0.592 |
| Walking pace | Vascular dementia (FinnGen) | MR-Egger | 53 | 40.3/29.8 | 1.14 (0.20–6.35) | 0.884 | 0.553 | 0.646 | 0.592 |
| Walking pace | Vascular dementia (FinnGen) | Weighted median | 53 | 40.3/29.8 | 2.93 (0.25–35.09) | 0.395 | — | — | 0.592 |
| Walking pace | Vascular dementia (FinnGen) | Simple mode | 53 | 40.3/29.8 | 1.04 (0.04–26.43) | 0.980 | — | — | 0.592 |
| Walking pace | Vascular dementia (FinnGen) | Weighted mode | 53 | 40.3/29.8 | 2.16 (0.10–45.89) | 0.622 | — | — | 0.592 |
| Walking pace | Vascular dementia (FinnGen) | MR-PRESSO | 53 | 40.3/29.8 | 0.62 (0.11–3.57) | 0.593 | — | — | 0.592 |
| Walking pace | Alzheimer's disease, wide (FinnGen) | IVW-FE | 53 | 40.3/29.8 | 0.76 (0.36–1.58) | 0.465 | 0.834 | — | 0.832 |
| Walking pace | Alzheimer's disease, wide (FinnGen) | IVW-MRE | 53 | 40.3/29.8 | 0.76 (0.36–1.58) | 0.465 | 0.834 | — | 0.832 |
| Walking pace | Alzheimer's disease, wide (FinnGen) | MR-Egger | 53 | 40.3/29.8 | 0.79 (0.37–1.65) | 0.527 | 0.818 | 0.551 | 0.832 |
| Walking pace | Alzheimer's disease, wide (FinnGen) | Weighted median | 53 | 40.3/29.8 | 1.03 (0.37–2.88) | 0.955 | — | — | 0.832 |
| Walking pace | Alzheimer's disease, wide (FinnGen) | Simple mode | 53 | 40.3/29.8 | 0.98 (0.33–2.90) | 0.973 | — | — | 0.832 |
| Walking pace | Alzheimer's disease, wide (FinnGen) | Weighted mode | 53 | 40.3/29.8 | 0.98 (0.30–3.17) | 0.975 | — | — | 0.832 |
| Walking pace | Alzheimer's disease, wide (FinnGen) | MR-PRESSO | 53 | 40.3/29.8 | 0.83 (0.39–1.76) | 0.621 | — | — | 0.832 |
| OR: Odds ratio; CI: Confidence interval; IVW-FE: Ixed-effect inverse-variance weighted; IVW-MRE: Multiplicative random-effects inverse-variance weighted; *Q P*: Cochran's Q heterogeneity *P* value; *Egger P*: MR-Egger intercept *P* value; *PRESSO P:* MR-PRESSO global test *P* value. F mean/min are shown as mean/minimum SNP F statistics. | | | | | | | | | |
