# Supplementary material for: Muscle quality index combined with plasma metabolomics: enhancing risk stratification for post-stroke dementia
Source: Front Aging Neurosci. 2026 Sep 29;18:1837030. doi: 10.3389/fnagi.2026.1837030 (PMC13658159; doi:10.3389/fnagi.2026.1837030)
Supplement: Supplementary file 4 [file Data_Sheet_4.DOCX]

| **Table S18. Multivariable Mendelian randomization results** | | | | | |
| --- | --- | --- | --- | --- | --- |
| **Outcome** | **Exposure** | **SNPs** | **Beta (SE)** | **OR (95% CI)** | ***P value*** |
| Alzheimer's disease (Bellenguez 2022) | Hand grip strength (left) | 532 | -0.117 (0.138) | 0.89 (0.68–1.17) | 0.396 |
| Alzheimer's disease (Bellenguez 2022) | Appendicular lean mass | 532 | -0.080 (0.036) | 0.92 (0.86–0.99) | 0.027 |
| All-cause dementia (FinnGen) | Hand grip strength (left) | 522 | -0.102 (0.269) | 0.90 (0.53–1.53) | 0.705 |
| All-cause dementia (FinnGen) | Appendicular lean mass | 522 | -0.068 (0.070) | 0.93 (0.81–1.07) | 0.327 |
| Vascular dementia (FinnGen) | Hand grip strength (left) | 522 | -1.811 (0.696) | 0.16 (0.04–0.64) | 0.009 |
| Vascular dementia (FinnGen) | Appendicular lean mass | 522 | 0.299 (0.180) | 1.35 (0.95–1.92) | 0.097 |
| Alzheimer's disease, wide (FinnGen) | Hand grip strength (left) | 522 | 0.100 (0.299) | 1.11 (0.62–1.98) | 0.737 |
| Alzheimer's disease, wide (FinnGen) | Appendicular lean mass | 522 | -0.109 (0.077) | 0.90 (0.77–1.04) | 0.158 |
| MVMR estimates are shown as beta (SE) and OR (95% CI). MVMR: Multivariable Mendelian randomization; SNPs: single-nucleotide polymorphisms; OR: Odds ratio; CI: Confidence interval. | | | | | |
