# Supplementary material for: Muscle quality index combined with plasma metabolomics: enhancing risk stratification for post-stroke dementia
Source: Front Aging Neurosci. 2026 Sep 29;18:1837030. doi: 10.3389/fnagi.2026.1837030 (PMC13658159; doi:10.3389/fnagi.2026.1837030)
Supplement: Supplementary file 5 [file Data_Sheet_5.DOCX]

| **Table S19. Reverse Mendelian randomization results** | | | | | | | | | |
| --- | --- | --- | --- | --- | --- | --- | --- | --- | --- |
| **Exposure** | **Outcome** | **Method** | **SNPs** | **F mean/min** | **OR (95% CI)** | ***P*** | ***Q P*** | ***Egger P*** | ***PRESSO P*** |
| Alzheimer's disease (Bellenguez 2022) | Hand grip strength (left) | IVW-FE | 53 | 88.7/30.4 | 1.01 (1.00–1.01) | 0.010 | <0.001 | — | <0.001 |
| Alzheimer's disease (Bellenguez 2022) | Hand grip strength (left) | IVW-MRE | 53 | 88.7/30.4 | 1.01 (1.00–1.02) | 0.153 | <0.001 | — | <0.001 |
| Alzheimer's disease (Bellenguez 2022) | Hand grip strength (left) | MR-Egger | 53 | 88.7/30.4 | 1.01 (1.00–1.02) | 0.093 | <0.001 | 0.149 | <0.001 |
| Alzheimer's disease (Bellenguez 2022) | Hand grip strength (left) | Weighted median | 53 | 88.7/30.4 | 1.01 (1.00–1.02) | 0.071 | — | — | <0.001 |
| Alzheimer's disease (Bellenguez 2022) | Hand grip strength (left) | MR-PRESSO | 53 | 88.7/30.4 | 1.01 (1.00–1.01) | 0.004 | — | — | <0.001 |
| Alzheimer's disease (Bellenguez 2022) | Hand grip strength (right) | IVW-FE | 53 | 88.7/30.4 | 1.01 (1.00–1.01) | 0.005 | <0.001 | — | <0.001 |
| Alzheimer's disease (Bellenguez 2022) | Hand grip strength (right) | IVW-MRE | 53 | 88.7/30.4 | 1.01 (1.00–1.02) | 0.125 | <0.001 | — | <0.001 |
| Alzheimer's disease (Bellenguez 2022) | Hand grip strength (right) | MR-Egger | 53 | 88.7/30.4 | 1.01 (1.00–1.02) | 0.067 | <0.001 | 0.100 | <0.001 |
| Alzheimer's disease (Bellenguez 2022) | Hand grip strength (right) | Weighted median | 53 | 88.7/30.4 | 1.00 (0.99–1.01) | 0.578 | — | — | <0.001 |
| Alzheimer's disease (Bellenguez 2022) | Hand grip strength (right) | MR-PRESSO | 53 | 88.7/30.4 | 1.01 (1.00–1.01) | 0.024 | — | — | <0.001 |
| Alzheimer's disease (Bellenguez 2022) | Low hand grip strength (FNIH, 60+) | IVW-FE | 59 | 84.8/30.4 | 0.95 (0.92–0.99) | 0.010 | <0.001 | — | <0.001 |
| Alzheimer's disease (Bellenguez 2022) | Low hand grip strength (FNIH, 60+) | IVW-MRE | 59 | 84.8/30.4 | 0.95 (0.91–1.01) | 0.086 | <0.001 | — | <0.001 |
| Alzheimer's disease (Bellenguez 2022) | Low hand grip strength (FNIH, 60+) | MR-Egger | 59 | 84.8/30.4 | 0.95 (0.90–1.00) | 0.073 | <0.001 | 0.379 | <0.001 |
| Alzheimer's disease (Bellenguez 2022) | Low hand grip strength (FNIH, 60+) | Weighted median | 59 | 84.8/30.4 | 1.01 (0.95–1.07) | 0.807 | — | — | <0.001 |
| Alzheimer's disease (Bellenguez 2022) | Low hand grip strength (FNIH, 60+) | MR-PRESSO | 59 | 84.8/30.4 | 0.97 (0.94–1.01) | 0.160 | — | — | <0.001 |
| Alzheimer's disease (Bellenguez 2022) | Appendicular lean mass | IVW-FE | 59 | 84.8/30.4 | 1.02 (1.01–1.02) | <0.001 | <0.001 | — | <0.001 |
| Alzheimer's disease (Bellenguez 2022) | Appendicular lean mass | IVW-MRE | 59 | 84.8/30.4 | 1.02 (1.00–1.03) | 0.111 | <0.001 | — | <0.001 |
| Alzheimer's disease (Bellenguez 2022) | Appendicular lean mass | MR-Egger | 59 | 84.8/30.4 | 1.01 (0.99–1.03) | 0.173 | <0.001 | 0.105 | <0.001 |
| Alzheimer's disease (Bellenguez 2022) | Appendicular lean mass | Weighted median | 59 | 84.8/30.4 | 1.01 (1.00–1.02) | 0.029 | — | — | <0.001 |
| Alzheimer's disease (Bellenguez 2022) | Appendicular lean mass | MR-PRESSO | 59 | 84.8/30.4 | 1.02 (1.01–1.02) | <0.001 | — | — | <0.001 |
| Alzheimer's disease (Bellenguez 2022) | Whole body fat-free mass | IVW-FE | 53 | 88.7/30.4 | 1.00 (0.99–1.00) | 0.024 | <0.001 | — | <0.001 |
| Alzheimer's disease (Bellenguez 2022) | Whole body fat-free mass | IVW-MRE | 53 | 88.7/30.4 | 1.00 (0.99–1.00) | 0.258 | <0.001 | — | <0.001 |
| Alzheimer's disease (Bellenguez 2022) | Whole body fat-free mass | MR-Egger | 53 | 88.7/30.4 | 0.99 (0.99–1.00) | 0.257 | <0.001 | 0.811 | <0.001 |
| Alzheimer's disease (Bellenguez 2022) | Whole body fat-free mass | Weighted median | 53 | 88.7/30.4 | 0.99 (0.98–1.00) | 0.020 | — | — | <0.001 |
| Alzheimer's disease (Bellenguez 2022) | Whole body fat-free mass | MR-PRESSO | 53 | 88.7/30.4 | 0.99 (0.99–1.00) | 0.003 | — | — | <0.001 |
| Alzheimer's disease (Bellenguez 2022) | Walking pace | IVW-FE | 53 | 88.7/30.4 | 1.01 (1.01–1.02) | <0.001 | <0.001 | — | <0.001 |
| Alzheimer's disease (Bellenguez 2022) | Walking pace | IVW-MRE | 53 | 88.7/30.4 | 1.01 (1.01–1.02) | <0.001 | <0.001 | — | <0.001 |
| Alzheimer's disease (Bellenguez 2022) | Walking pace | MR-Egger | 53 | 88.7/30.4 | 1.01 (1.00–1.02) | 0.002 | <0.001 | 0.216 | <0.001 |
| Alzheimer's disease (Bellenguez 2022) | Walking pace | Weighted median | 53 | 88.7/30.4 | 1.01 (1.00–1.02) | 0.004 | — | — | <0.001 |
| Alzheimer's disease (Bellenguez 2022) | Walking pace | MR-PRESSO | 53 | 88.7/30.4 | 1.01 (1.01–1.02) | <0.001 | — | — | <0.001 |
| All-cause dementia (FinnGen) | Hand grip strength (left) | IVW-FE | 6 | 271.1/31.1 | 1.00 (0.99–1.00) | 0.164 | 0.315 | — | 0.337 |
| All-cause dementia (FinnGen) | Hand grip strength (left) | IVW-MRE | 6 | 271.1/31.1 | 1.00 (0.99–1.00) | 0.200 | 0.315 | — | 0.337 |
| All-cause dementia (FinnGen) | Hand grip strength (left) | MR-Egger | 6 | 271.1/31.1 | 1.00 (1.00–1.01) | 0.735 | 0.673 | 0.132 | 0.337 |
| All-cause dementia (FinnGen) | Hand grip strength (left) | Weighted median | 6 | 271.1/31.1 | 1.00 (0.99–1.00) | 0.279 | — | — | 0.337 |
| All-cause dementia (FinnGen) | Hand grip strength (left) | MR-PRESSO | 6 | 271.1/31.1 | 0.99 (0.98–1.00) | 0.009 | — | — | 0.337 |
| All-cause dementia (FinnGen) | Hand grip strength (right) | IVW-FE | 6 | 271.1/31.1 | 1.00 (1.00–1.00) | 0.876 | 0.428 | — | 0.438 |
| All-cause dementia (FinnGen) | Hand grip strength (right) | IVW-MRE | 6 | 271.1/31.1 | 1.00 (1.00–1.00) | 0.876 | 0.428 | — | 0.438 |
| All-cause dementia (FinnGen) | Hand grip strength (right) | MR-Egger | 6 | 271.1/31.1 | 1.00 (1.00–1.01) | 0.317 | 0.771 | 0.153 | 0.438 |
| All-cause dementia (FinnGen) | Hand grip strength (right) | Weighted median | 6 | 271.1/31.1 | 1.00 (1.00–1.00) | 0.734 | — | — | 0.438 |
| All-cause dementia (FinnGen) | Low hand grip strength (FNIH, 60+) | IVW-FE | 6 | 271.1/31.1 | 1.03 (1.01–1.06) | 0.016 | 0.320 | — | 0.314 |
| All-cause dementia (FinnGen) | Low hand grip strength (FNIH, 60+) | IVW-MRE | 6 | 271.1/31.1 | 1.03 (1.00–1.07) | 0.027 | 0.320 | — | 0.314 |
| All-cause dementia (FinnGen) | Low hand grip strength (FNIH, 60+) | MR-Egger | 6 | 271.1/31.1 | 1.01 (0.97–1.05) | 0.600 | 0.499 | 0.189 | 0.314 |
| All-cause dementia (FinnGen) | Low hand grip strength (FNIH, 60+) | Weighted median | 6 | 271.1/31.1 | 1.02 (0.99–1.05) | 0.123 | — | — | 0.314 |
| All-cause dementia (FinnGen) | Low hand grip strength (FNIH, 60+) | MR-PRESSO | 6 | 271.1/31.1 | 1.11 (1.03–1.18) | 0.004 | — | — | 0.314 |
| All-cause dementia (FinnGen) | Appendicular lean mass | IVW-FE | 6 | 271.1/31.1 | 1.00 (1.00–1.01) | 0.092 | <0.001 | — | <0.001 |
| All-cause dementia (FinnGen) | Appendicular lean mass | IVW-MRE | 6 | 271.1/31.1 | 1.00 (0.99–1.02) | 0.456 | <0.001 | — | <0.001 |
| All-cause dementia (FinnGen) | Appendicular lean mass | MR-Egger | 6 | 271.1/31.1 | 1.00 (0.99–1.02) | 0.736 | <0.001 | 0.884 | <0.001 |
| All-cause dementia (FinnGen) | Appendicular lean mass | Weighted median | 6 | 271.1/31.1 | 1.00 (1.00–1.01) | 0.171 | — | — | <0.001 |
| All-cause dementia (FinnGen) | Appendicular lean mass | MR-PRESSO | 6 | 271.1/31.1 | 1.00 (1.00–1.01) | 0.234 | — | — | <0.001 |
| All-cause dementia (FinnGen) | Whole body fat-free mass | IVW-FE | 6 | 271.1/31.1 | 1.00 (0.99–1.00) | 0.004 | <0.001 | — | <0.001 |
| All-cause dementia (FinnGen) | Whole body fat-free mass | IVW-MRE | 6 | 271.1/31.1 | 1.00 (0.98–1.01) | 0.429 | <0.001 | — | <0.001 |
| All-cause dementia (FinnGen) | Whole body fat-free mass | MR-Egger | 6 | 271.1/31.1 | 0.99 (0.97–1.01) | 0.279 | <0.001 | 0.382 | <0.001 |
| All-cause dementia (FinnGen) | Whole body fat-free mass | Weighted median | 6 | 271.1/31.1 | 0.99 (0.99–1.00) | <0.001 | — | — | <0.001 |
| All-cause dementia (FinnGen) | Walking pace | IVW-FE | 6 | 271.1/31.1 | 1.00 (1.00–1.01) | 0.022 | 0.011 | — | 0.012 |
| All-cause dementia (FinnGen) | Walking pace | IVW-MRE | 6 | 271.1/31.1 | 1.00 (1.00–1.01) | 0.183 | 0.011 | — | 0.012 |
| All-cause dementia (FinnGen) | Walking pace | MR-Egger | 6 | 271.1/31.1 | 1.01 (1.00–1.01) | 0.031 | 0.247 | 0.058 | 0.012 |
| All-cause dementia (FinnGen) | Walking pace | Weighted median | 6 | 271.1/31.1 | 1.00 (1.00–1.01) | 0.015 | — | — | 0.012 |
| All-cause dementia (FinnGen) | Walking pace | MR-PRESSO | 6 | 271.1/31.1 | 1.00 (0.99–1.01) | 0.861 | — | — | 0.012 |
| Alzheimer's disease, wide (FinnGen) | Hand grip strength (left) | IVW-FE | 5 | 292.3/30.2 | 1.00 (1.00–1.00) | 0.629 | 0.278 | — | 0.285 |
| Alzheimer's disease, wide (FinnGen) | Hand grip strength (left) | IVW-MRE | 5 | 292.3/30.2 | 1.00 (1.00–1.00) | 0.668 | 0.278 | — | 0.285 |
| Alzheimer's disease, wide (FinnGen) | Hand grip strength (left) | MR-Egger | 5 | 292.3/30.2 | 1.00 (0.99–1.00) | 0.771 | 0.165 | 0.987 | 0.285 |
| Alzheimer's disease, wide (FinnGen) | Hand grip strength (left) | Weighted median | 5 | 292.3/30.2 | 1.00 (1.00–1.00) | 0.627 | — | — | 0.285 |
| Alzheimer's disease, wide (FinnGen) | Hand grip strength (right) | IVW-FE | 5 | 292.3/30.2 | 1.00 (1.00–1.00) | 0.498 | 0.346 | — | 0.359 |
| Alzheimer's disease, wide (FinnGen) | Hand grip strength (right) | IVW-MRE | 5 | 292.3/30.2 | 1.00 (1.00–1.00) | 0.521 | 0.346 | — | 0.359 |
| Alzheimer's disease, wide (FinnGen) | Hand grip strength (right) | MR-Egger | 5 | 292.3/30.2 | 1.00 (1.00–1.01) | 0.652 | 0.215 | 0.986 | 0.359 |
| Alzheimer's disease, wide (FinnGen) | Hand grip strength (right) | Weighted median | 5 | 292.3/30.2 | 1.00 (1.00–1.00) | 0.513 | — | — | 0.359 |
| Alzheimer's disease, wide (FinnGen) | Low hand grip strength (FNIH, 60+) | IVW-FE | 5 | 292.3/30.2 | 1.02 (0.99–1.04) | 0.119 | 0.466 | — | 0.474 |
| Alzheimer's disease, wide (FinnGen) | Low hand grip strength (FNIH, 60+) | IVW-MRE | 5 | 292.3/30.2 | 1.02 (0.99–1.04) | 0.119 | 0.466 | — | 0.474 |
| Alzheimer's disease, wide (FinnGen) | Low hand grip strength (FNIH, 60+) | MR-Egger | 5 | 292.3/30.2 | 1.03 (1.00–1.06) | 0.125 | 0.772 | 0.215 | 0.474 |
| Alzheimer's disease, wide (FinnGen) | Low hand grip strength (FNIH, 60+) | Weighted median | 5 | 292.3/30.2 | 1.02 (0.99–1.05) | 0.136 | — | — | 0.474 |
| Alzheimer's disease, wide (FinnGen) | Appendicular lean mass | IVW-FE | 5 | 292.3/30.2 | 1.00 (1.00–1.01) | 0.033 | <0.001 | — | <0.001 |
| Alzheimer's disease, wide (FinnGen) | Appendicular lean mass | IVW-MRE | 5 | 292.3/30.2 | 1.00 (0.99–1.02) | 0.403 | <0.001 | — | <0.001 |
| Alzheimer's disease, wide (FinnGen) | Appendicular lean mass | MR-Egger | 5 | 292.3/30.2 | 1.00 (0.99–1.02) | 0.641 | <0.001 | 0.802 | <0.001 |
| Alzheimer's disease, wide (FinnGen) | Appendicular lean mass | Weighted median | 5 | 292.3/30.2 | 1.00 (1.00–1.01) | 0.056 | — | — | <0.001 |
| Alzheimer's disease, wide (FinnGen) | Appendicular lean mass | MR-PRESSO | 5 | 292.3/30.2 | 1.00 (1.00–1.01) | 0.037 | — | — | <0.001 |
| Alzheimer's disease, wide (FinnGen) | Whole body fat-free mass | IVW-FE | 5 | 292.3/30.2 | 1.00 (0.99–1.00) | 0.008 | 0.008 | — | 0.007 |
| Alzheimer's disease, wide (FinnGen) | Whole body fat-free mass | IVW-MRE | 5 | 292.3/30.2 | 1.00 (0.99–1.00) | 0.150 | 0.008 | — | 0.007 |
| Alzheimer's disease, wide (FinnGen) | Whole body fat-free mass | MR-Egger | 5 | 292.3/30.2 | 0.99 (0.99–1.00) | 0.204 | 0.011 | 0.464 | 0.007 |
| Alzheimer's disease, wide (FinnGen) | Whole body fat-free mass | Weighted median | 5 | 292.3/30.2 | 1.00 (0.99–1.00) | 0.013 | — | — | 0.007 |
| Alzheimer's disease, wide (FinnGen) | Walking pace | IVW-FE | 5 | 292.3/30.2 | 1.00 (1.00–1.01) | 0.001 | 0.385 | — | 0.397 |
| Alzheimer's disease, wide (FinnGen) | Walking pace | IVW-MRE | 5 | 292.3/30.2 | 1.00 (1.00–1.01) | 0.001 | 0.385 | — | 0.397 |
| Alzheimer's disease, wide (FinnGen) | Walking pace | MR-Egger | 5 | 292.3/30.2 | 1.01 (1.00–1.01) | 0.066 | 0.287 | 0.620 | 0.397 |
| Alzheimer's disease, wide (FinnGen) | Walking pace | Weighted median | 5 | 292.3/30.2 | 1.00 (1.00–1.01) | 0.004 | — | — | 0.397 |
| OR: Odds ratio; CI: Confidence interval; IVW-FE: Ixed-effect inverse-variance weighted; IVW-MRE: Multiplicative random-effects inverse-variance weighted; *Q P*: Cochran's Q heterogeneity *P* value; *Egger P*: MR-Egger intercept *P* value; *PRESSO P:* MR-PRESSO global test *P* value. F mean/min are shown as mean/minimum SNP F statistics. | | | | | | | | | |
